# Ultraconserved elements (UCEs) resolve the phylogeny of Australasian smurf-weevils

**DOI:** 10.1371/journal.pone.0188044

**Published:** 2017-11-22

**Authors:** Matthew H. Van Dam, Athena W. Lam, Katayo Sagata, Bradley Gewa, Raymond Laufa, Michael Balke, Brant C. Faircloth, Alexander Riedel

**Affiliations:** 1 SNSB-Zoological State Collection, Münchhausenstraße 21, München, Germany; 2 School of Natural & Physical Sciences, The University of Papua New Guinea, UNIVERSITY 134, National Capital District, Papua New Guinea; 3 The New Guinea Binatang Research Center, Madang, Papua New Guinea; 4 GeoBioCenter, Ludwig-Maximilians-Universität, München, Germany; 5 Department of Biological Sciences and Museum of Natural Science, Louisiana State University, Baton Rouge, LA, United States of America; 6 State Museum of Natural History Karlsruhe, Karlsruhe, Germany; Vanderbilt University, UNITED STATES

## Abstract

Weevils (Curculionoidea) comprise one of the most diverse groups of organisms on earth. There is hardly a vascular plant or plant part without its own species of weevil feeding on it and weevil species diversity is greater than the number of fishes, birds, reptiles, amphibians and mammals combined. Here, we employ ultraconserved elements (UCEs) designed for beetles and a novel partitioning strategy of loci to help resolve phylogenetic relationships within the radiation of Australasian smurf-weevils (Eupholini). Despite being emblematic of the New Guinea fauna, no previous phylogenetic studies have been conducted on the Eupholini. In addition to a comprehensive collection of fresh specimens, we supplement our taxon sampling with museum specimens, and this study is the first target enrichment phylogenomic dataset incorporating beetle specimens from museum collections. We use both concatenated and species tree analyses to examine the relationships and taxonomy of this group. For species tree analyses we present a novel partitioning strategy to better model the molecular evolutionary process in UCEs. We found that the current taxonomy is problematic, largely grouping species on the basis of similar color patterns. Finally, our results show that most loci required multiple partitions for nucleotide rate substitution, suggesting that single partitions may not be the optimal partitioning strategy to accommodate rate heterogeneity for UCE loci.

## Introduction

The Curculionoidea represents a diverse group of phytophagous insects with ~62,000 described species and an estimated 200,000 undescribed species [1]. They are not only highly diverse, but also of major economic importance because they cause destruction to many crop and ornamental plant species worldwide. Curculionoidea are a relatively old group dating to the Upper Jurassic 161–151 Ma [1–3]. Despite both their diversity and economic importance, there are still few resources for conducting phylogenomic research on this group. Here, we test a set of target enrichment baits designed to enrich ultraconserved elements (UCEs) across Coleoptera [4], and we use the resulting sequence data to examine the phylogeny of Australasian smurf-weevils in the tribe Eupholini. We had two main goals: 1) to examine the utility of these newly designed universal Coleoptera UCE baits for phylogenetic studies of weevils, using freshly collected individuals as well as older museum specimens (up to 65 years); and 2) to test the monophyly of the three main genera within the Eupholini, examining the validity of previous ecomorphology-driven classification efforts.

Ultraconserved genomic elements (UCEs, *sensu* Faircloth *et al*. 2012 [5]), provide a powerful approach to sequence many independent regions of the genome. UCEs have proven useful in resolving phylogenies at multiple phylogenetic scales both shallow and deep [6–9]. UCEs, like many other reduced representation techniques that rely on oligonucleotide “bait” capture procedures, also have the potential to perform well when collecting data from museum samples [10,11]. Given the challenges with obtaining samples for many rare taxa and/or from logistically challenging regions of the world, such approaches can be highly beneficial [12,10]. The challenge of obtaining weevil samples is even more complex because many species can be found only on specific host plants at very particular times of the year, and many weevil hosts are unknown. In addition to the challenges of sampling, many weevil groups have undergone rapid radiations [2,13] necessitating the development of large genome-wide data sets to take on such challenges.

**Fig 1:**
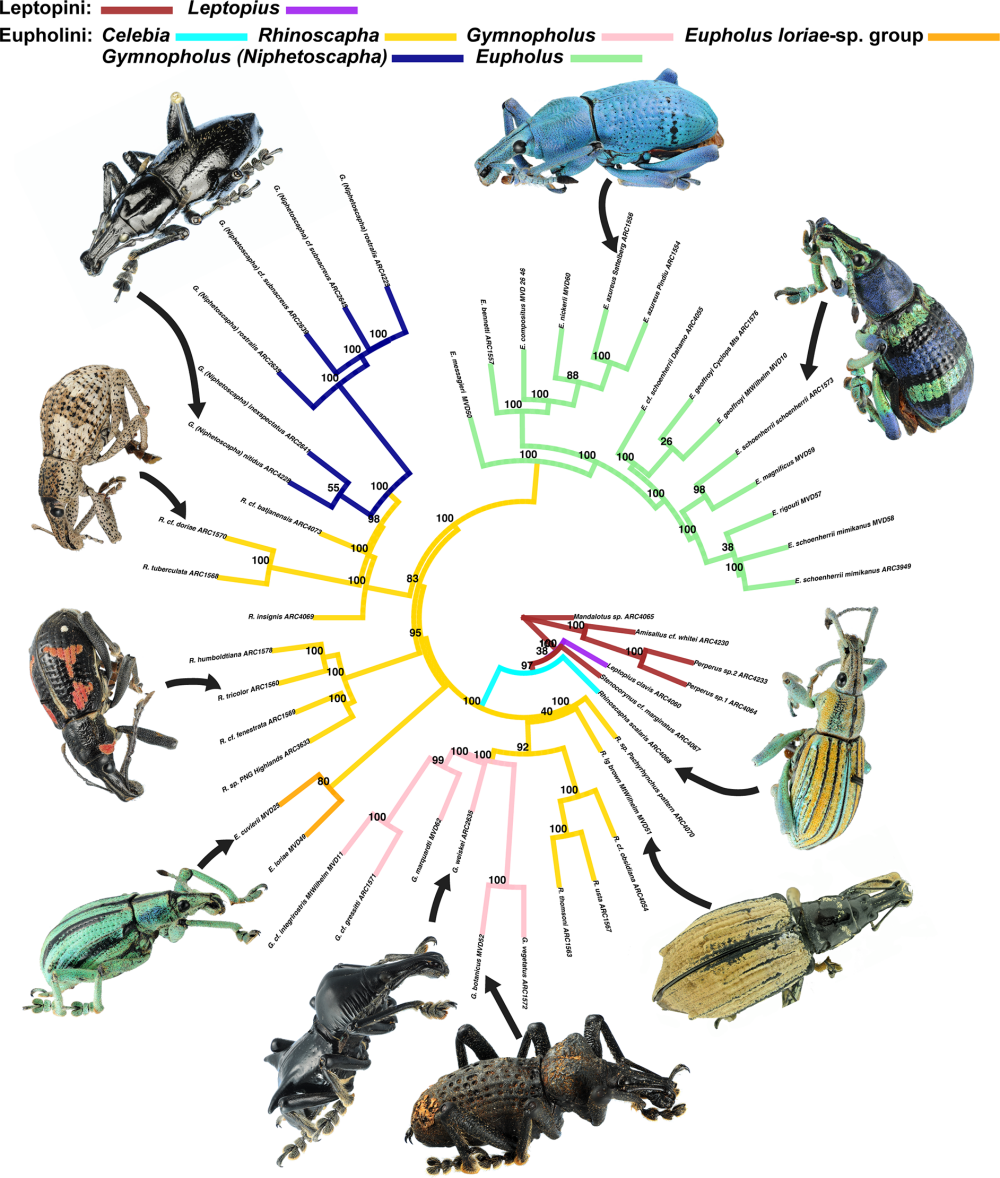
ASTRAL species tree, input trees derived from multi-partitioned MrBayes analyses of individual gene trees (i.e. using the 6 character in sets in Fig 2 to inform the partitioning strategy). Node values indicate support values of MrBayes posterior used as ASTRAL bootstrap replicates, branch colors correspond to species clades. Weevils from top clockwise: *Eupholus azureus* Macleay, *Eupholus schoenherrii schoenherrii* (Guérin-Méneville), *Celebia arrogans* (Boisduval), *Rhinoscapha sp*. “Large-brown Mt.Wilhelm 2700m”, *Gymnopholus (Symbiopholus) acarifer* Gressitt, *Gymnopholus regalis* Gressitt, *Eupholus cuvierii* (Guérin-Méneville), *Rhinoscapha tricolor* Faust, *Rhinoscapha doriae* Pascoe, *Gymnopholus nitidus* Gressitt & Sedlacek.

The Eupholini represent a tribe of weevils with approximately 300 known species. New Guinea is the Eupholini’s center of diversity both in terms of species and genera. Currently, there are three main genera recognized within the Eupholini, although the monophyly of each is somewhat questionable: *Eupholus* Boisduval (63 species), *Gymnopholus* Heller (76 species) and *Rhinoscapha* Montrouzier (137 species) [14,15]. *Eupholus* species are brilliantly colored and can be divided in two morphologically distinct groups, i.e. the *E*. *schoenherri*-group and the *E*. *loriae*-group [16]. Members of the *E*. *schoenherri*-group appear to feed exclusively on toxic wild yams (*Dioscorea*) (Fig 1). Most *Eupholus* species (~58) occur in habitats below 1,000m elevation with few (~5) above 1,500m. Members of *Gymnopholus* are of dark or cryptic coloration and usually occur above 1,500m. *Gymnopholus* species in the subgenus *Symbiopholus* (33 species) occur above 2,000m and exhibit epizoic symbiosis, i.e. lichens, algae and moss growing on their integument that enhance camouflage (Fig 1) [17,18]. *Gymnopholus* species are polyphagous, browsing on a variety of plants. Species in the third genus, *Rhinoscapha*, are sometimes brilliantly colored and other times more cryptic. Although their hosts are less well known, *Rhinoscapha* species can be found at all elevational zones, with certain clades showing some elevational zonation.

## Materials and methods

### UCE probe design

The 1,172 UCE loci that we tested with Eupholini were identified in Faircloth 2017. The 13,674 baits targeting these loci were designed from six different Coleoptera genomes and one genome from the sister Order Strepsiptera. Of these taxa, *Dendroctonus ponderosa*e (Curculionidae: Scolytinae) is most closely related to the Eupholini (Curculionidae: Entiminae): they shared a most recent common ancestor ~105 Ma [2]. We had the bait set synthesized by MYcroarray (MYcroarray, Ann Arbor MI, USA) as described, with no modifications.

### Laboratory protocols

We extracted DNA non-destructively from 48 samples, using DNeasy Tissue kits (Qiagen; Macherey-Nagel). Nine of these 48 samples were pinned museum specimens that ranged in age from 1952 to 2006. Tissue was extracted from the pronotum and/or leg of each specimen for specimens preserved in ethanol. For pinned specimens, tissue was taken out of the pronotum, mesothorax and abdomen using sterilized forceps (flame treated then soaked in 10% bleach between use) and placed in a 1.5–2.0 ml centrifuge tube. In the case of large pinned specimens, the tissue from the pronotum, mesothorax and abdomen were placed in separate centrifuge tubes, the resulting eluates from the specimen parts were later combined. We added 4μl of RNase-A to remove RNA from the samples during the extraction process according to manufacturer's protocol (http://www.bea.ki.se/documents/EN-DNeasy%20handbook.pdf). In the case of extractions obtained without the use of RNase-A during the extraction process, 1μl of 10 μg/mL RNase-A was added to each genomic DNA (gDNA) sample, the mixture was then incubated at 37°C for 30 minutes, followed by purification via ethanol precipitation. Extracted DNA quantity ranged from 253–1600 ng (mean 556 ng) measured by Qubit fluorometer (Life Technologies, Inc.). Subsequent library preparation and sequence capture steps were performed by RAPiD Genomics (Gainesville, FL, USA). Briefly, to confirm that ethanol preserved samples’ DNA was not degraded we visualized their DNA on a polyacrylamide gel for a few exemplar specimens, while all museum samples’ DNA was visualized on a polyacrylamide gel to assess the amount of degradation. Following visual examination, samples that were not highly degraded (>500 bp) were sonicated to a target size of 450 bp. Capture was performed with two pools of 24 samples each. Libraries were constructed by repairing the ends of the sheared fragments followed by the ligation of an adenine residue to the 3’-end of the blunt-end fragments. Next, custom indexed adapters suited for Illumina Sequencing platform were ligated to the libraries (see S1 Supporting Information Links). Finally, ligated fragments were PCR-amplified using standard cycling protocols (e.g. Mamanova et al. 2010 [19]), with Pre-capture PCR having 8–12 cycles, and Post-capture PCR with 13 cycles. Enrichment procedures followed the MYcroarray MYBaits kit Version-3 protocol [20], except that RAPiD Genomics used chicken C_0_t-1 DNA as blocking reagent. In Hymenoptera, chicken C_0_t-1 was shown to work better than “homebrew”, taxon specific C_0_t-1 DNA [21]. The 48 indexed libraries were pooled in equimolar ratios to a total of 250 ng/μl. The 48 MYBaits reactions were used at the full concentration. We sequenced two pools of these samples each using a half lane of paired-end, 100 base pair reads on an illumina HiSeq 3000. We performed these two sequencing runs primarily to see if this substantially improved the number of loci captured. Voucher specimens are stored in museum collections (SNSB-Zoological State Collection, München (ZSM), State Museum of Natural History Karlsruhe (SMNK), California Academy of Sciences (CAS)), see S3 Table for details.

### Analysis of captured UCE data

We used ILLUMIPROCESSOR [22], which is a parallel wrapper of TRIMMOMATIC [23–25], to clean and trim reads. We performed this step for each of the two sequencing runs, we then concatenated the two different sequencing runs together. After trimming, we generated summary stats on the trimmed reads using the PHYLUCE v1.6 package [26], script “*phyluce_assembly_get_fastq_lengths*”. All programs hereafter beginning with “*phyluce*” are PYTHON programs part of the PHYLUCE package. We assembled the cleaned/trimmed reads using “*phyluce_assembly_assemblo_trinity*” with TRINITY v2013-02-25 [27]. Next we generated summary statistics (counts/lengths) of the assembled contigs using “*phyluce_assembly_get_fasta_lengths*”. To identify UCE loci from our contigs we needed to match these to the probes, we used “*phyluce_assembly_match_contigs_to_probes*”, this was set to a minimum coverage of 80% and minimum identity of 80%. We used “*phyluce_assembly_get_match_counts*” to create an initial database of loci counts per taxon. To obtain average read depth of our contigs by taxon we used “*phyluce_assembly_get_trinity_coverage*”, the results of which were then used to calculate average read depth among our UCE loci per taxon with “*phyluce_assembly_get_trinity_coverage_for_uce_loci*” using the sqlite database created in the “*phyluce_assembly_match_contigs_to_probes*” step. Next, we used “*phyluce_assembly_get_fastas_from_match_counts*” to get a count of the number of UCE loci captured for each taxa. To examine the effect of preservation type on number of loci captured, we performed a Welch Two Sample t-test between museum-pinned and ethanol preserved samples. We then divided each UCE loci into a separate fasta file using “*phyluce_assembly_explode_get_fastas_file*”, as we later wanted to construct individual gene tree phylogenies, and then aligned the sequences and trimmed the edges using “*phyluce_align_seqcap_align*” which implements MAFFT v7.130b [28]. We did not use internal alignment trimming because our taxa are relatively closely related. However we eliminated columns that were entirely composed of “-”,”n” and or “?” with a custom *R* script using "deleteEmptyCells" function in the "*ips*" library [29]. Lastly we used “*phyluce_align_get_only_loci_with_min_taxa*” to select the minimum percentage of missing taxa in our UCE loci alignments.

### UCE phylogenomics

We performed two different types of phylogenomic analyses: (1) a concatenated analyses using RAxML v8.0.19 [30], and (2) several species tree analyses using ASTRAL v5.1.0 with TRee ALgorithm III (https://github.com/chaoszhang/ASTRAL/tree/multiind) [31–33] and SVDQuartets [34,35] which is implemented in PAUP* v4.0a152 [36].

#### Concatenated analyses

We set each UCE locus as its own character set and then used PartitionFinder v2.1.1 [37] implementing the “greedy” search algorithm [38] to select for the best partitioning strategy for the data under the General Time Reversible + gamma (GTRGAMMA) site rate substitution model using the AICc metric. We then conducted 20 maximum-likelihood (ML) searches in RAxML. We also performed non-parametric bootstrap replicates under GTRGAMMA using the autoMRE option to optimize the number of bootstrap replicates for this large dataset. We reconciled the bootstrap replicates with the best fitting ML tree.

#### Species tree analyses

**Fig 2:**
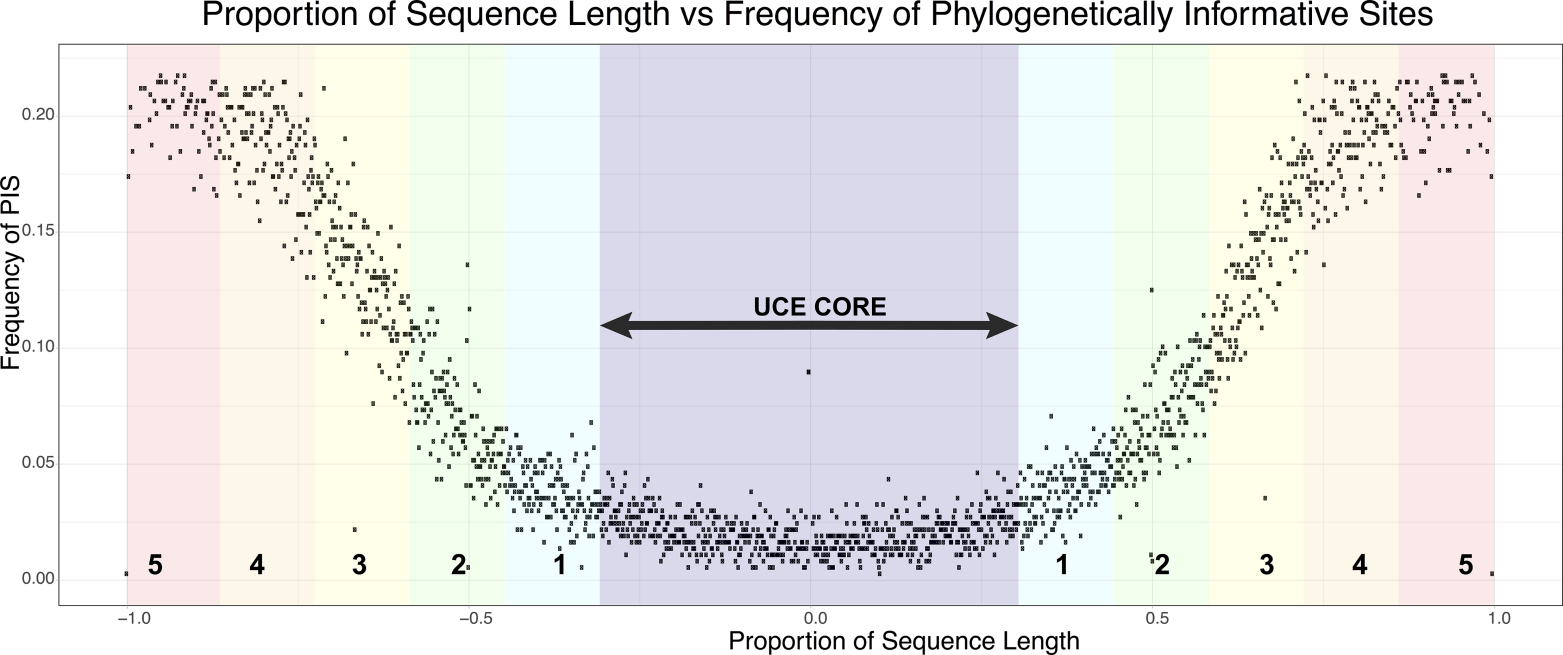
Shows approximate position of character sets used for each locus in PartitionFinder 2 for ASTRAL analyses, overlaid on the frequency of PIS in the final UCE data set. UCE Core refers to the section of the locus that corresponds to the length of UCE probe. Numbers 1–5 on left of UCE Core correspond to matching characters sets on the right, such that e.g. both sections 5 are the same character set. Character sets 1–5 correspond to one fifth of the length of the locus (left and right of UCE Core) minus the sites from the UCE Core.

Here, we performed several different analyses. First we reconstructed the ML gene tree estimated for each of the UCE loci in RAxML, below we list the specific process and settings in RAxML for our partitioned gene trees. Here we used an initial character set of the UCE core central (160bp) and divided up the remaining length of the locus into fifths. This character set was chosen based on a visual inspection of the number of phylogenetically informative sites over the proportion of loci length (Fig 2). This resulted in 5 character sets (6 including the UCE core), that were grouped together based on their proportional length from the conserved UCE core. We then used PartitionFinder v2.1.1 [37] implementing the “greedy” search algorithm [38] to select the best partitioning strategy for each locus, under the GTRGAMMA site rate substitution model according to the AICc metric. Next, we conducted 20 maximum-likelihood (ML), best tree searches for each partitioned locus in RAxML, as well as 200 non-parametric bootstrap replicates. We reconciled the bootstrap replicates with the best fitting ML tree of each locus. We also wanted to examine the effect of removing poorly supported/oversaturated gene trees on the topology and or support of the species tree. Using modified R code from Borowiec et al. 2015 [39], we first calculated the average of the bootstrap support values for each gene tree and then eliminated gene trees that were in the lowest 10% quantile of average bootstrap support. We then identified potentially over-saturated loci, based on departure from a linear regression between uncorrected p-distances and inferred distances of the tips for each of the UCE loci [40]. We then eliminated the outlier trees that were potentially over-saturated. Next we estimated a species tree using ASTRAL and assessed support with 200 bootstrap replicates for the complete set of genes, the set without the lowest 10% average bootstrap support, and the remaining trees without potentially oversaturated loci. Lastly, for comparative purposes, we also reconstructed a species tree in ASTRAL based on single character sets / partition and performed the same RAxML analyses as described above for the complete dataset.

Based on the results of the species tree topology and support values described above, we proceeded with gene tree reconstruction in MrBayes v3.2.5 [41]. We also used the same partitioning strategy described above, estimated the most appropriate site rate substitution model for MrBayes using PartitionFinder v2.1.1. We conducted 2 independent runs of 1 cold chain and 3 heated chains (default settings) for 1x10^7^ Markov chain Monte Carlo (MCMC) generations sampling every 1000 generations in MrBayes. We removed the first 25% as the burnin. We combined the two independent runs and created a maximum clade credibility topology (MCCT) for each locus with “sumtrees.py” part of the *DendroPy* package [42] as well as with custom scripts, see S1 Supporting Information Links. These gene trees were then input to ASTRAL to create a species tree. In order to assess support of the species tree we used each of the MCMC generations as a bootstrap replicate in ASTRAL (combined 15000 samples of the run after the burnin for each of the gene trees), thus creating support values akin to posterior probabilities of nodes. Lastly, for comparative purposes, we also reconstructed a species tree in ASTRAL based on single character sets/partitions per-locus and performed the same model selection and gene tree reconstruction as described above in MrBayes. We will refer to the analyses based on single character sets / partitions per-locus as ASTRAL-(RAxML or MrBayes)-single-partition analyses and those based on multiple partitions as ASTRAL-(RAxML or MrBayes)-multi-partition analyses.

Finally, we used another species tree method to look for congruence between methods. We created a species tree using SVDQuartets [34,35] where we evaluated all of the quartets with the ‘evalQuartets = all’ command using the *Quartet FM* (QFM) algorithm [43]. We assessed support with 200 bootstrap replicates.

We also wanted to more objectively examine the differences that resulted from these methods rather than just a visual comparison. Therefore, we calculated three tree distance metrics that rely on the tree topology between two trees in the R package *Phangorn* [44]; the Robinson-Foulds distance (RF-dist) [45], the path distance metric between pairs of taxa (Path-dist) [46] and the approximate subtree prune and regraft distance (SPR-dist) [47,48]. Visualizing support values and color coding on trees was implemented in the R package *phytools* [49].

### UCE capture and preservation

We investigated if specimen age or preservation type (museum pinned or ethanol preservation) had a greater effect on the number of UCEs captured using generalized linear models (GLM). Next, we compared our dataset to those of two other studies which utilized UCEs and museum samples, specifically *Xylocopa* (carpenter bees) [11] and *Aphelocoma* (scrub-jays) [10]. These studies were chosen because of their data availability and their systematic sampling by specimen age. We wanted to see if the number of UCE loci captured, given specimen age, was similar between studies. We first transformed the fraction of UCE loci captured within each taxon (number of UCE loci captured per-specimen divided by the maximum number of loci captured, within a clade e.g. Eupholini weevils or scrub-jays), and then logit transformed the data and performed a likelihood-ratio test, to see if a model with independent slopes vs one with a single slope fit the data better.

## Results

**Fig 3:**
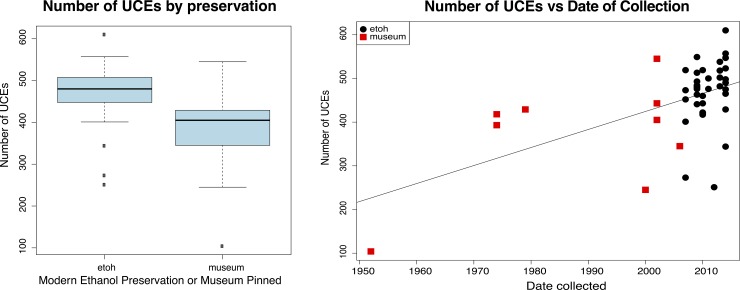
Left: Boxplot of number of UCEs by preservation type. Average number of UCEs for modern ethanol preserved specimens was higher than for museum pinned specimens. Right: Plot of number of UCEs vs their date of collection. Plot shows general trend of fewer UCEs captured for the older the specimens, however the exact rate of decrease would require more specimens systematically sampled by precise preservation type.

**Fig 4:**
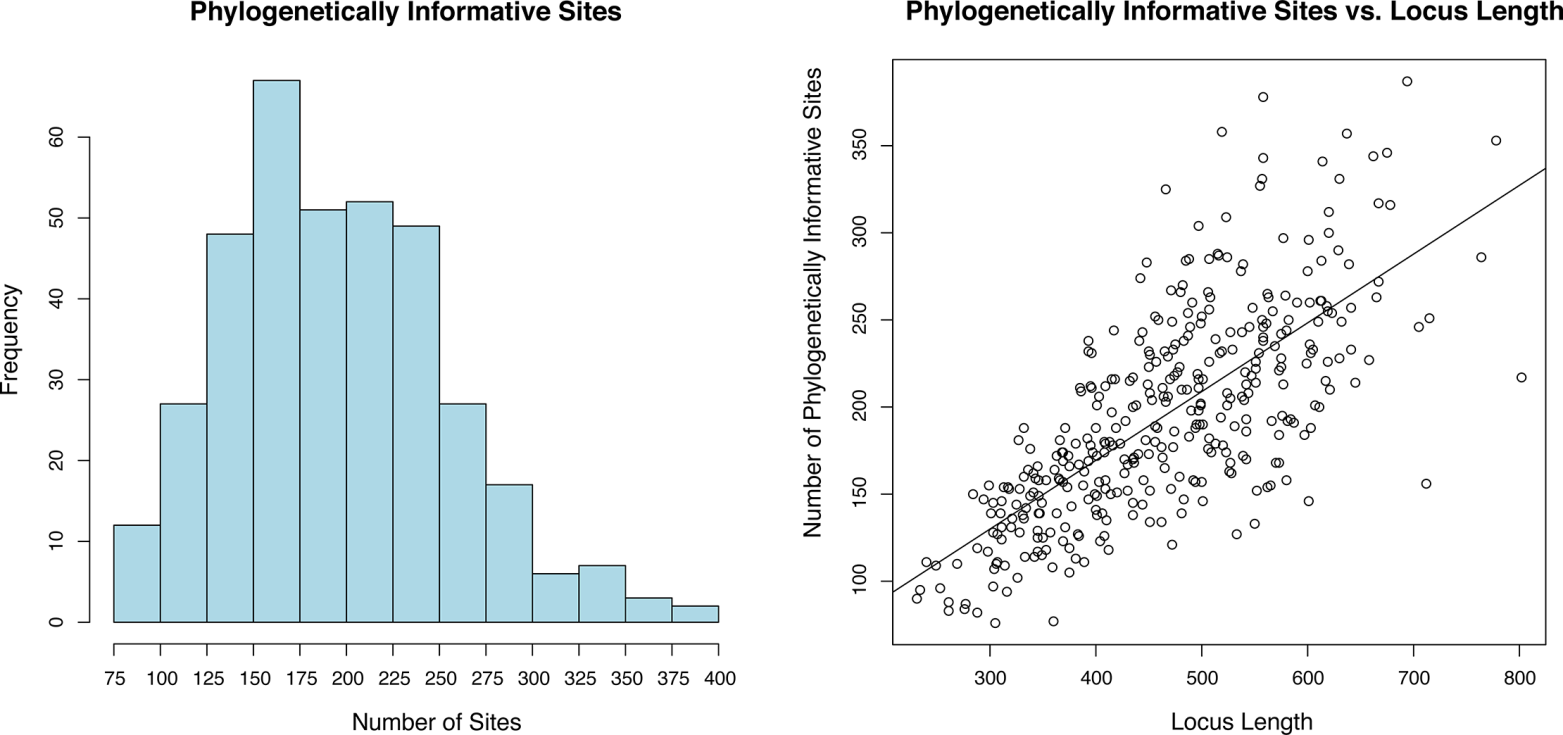
Left: Histogram of the number of potentially informative sites per locus from final data matrices, calculated with *pis* function it the R library *ips*. Right shows linear regression between the number of phylogenetically informative sites and loci length. Loci of shorter length tend to have a narrower range of informative sites whereas longer loci tend to have a wider range, giving the distribution a clubbed appearance.

We sequenced on average ~1.2x10^7^ reads per sample with an average length of ~100 bp, see S1 Table. The UCE loci alignment results from the first round of sequencing and UCE loci alignment results from concatenation of reads (1^st^ and 2^nd^ rounds together), showed only a modest increase in the number of loci captured, for example at the 70% complete level the difference was only 36 loci (359 1^st^ vs 395 2^nd^ round of sequencing), see S2 Table. The following results refers to only those of the final concatenated dataset. The average per-sample read depth of all contigs was 11.8X, while the average per-sample read depth of UCE loci was 28.9X. Across all samples, we captured a total of 940 out of 1,172 UCE loci. After removing duplicate loci, we recovered an average of 451 UCE loci from each sample, with a range of 104–610 loci; SE ±13.0 loci. We recovered an average of 470 loci with a range of 251–610; SE ±11.0 loci from ethanol preserved specimens, and we recovered fewer loci from the 9 museum samples (mean of 370 loci with a range of 104–545; SE ±42.6 loci; ethanol vs museum pinned, Welch Two Sample t-test *p*-value of 0.04812) (Fig 3). Of the 806 UCE alignments containing >2 taxa, we selected 368 alignments to create a ≥70% complete matrix (n = 33 taxa) with a total of 171,290 bp for all further phylogenetic analyses. The mean number of phylogenetically informative sites (PIC) per locus was 195.2 (range 76–387 PIC; SE ±3.1) (Fig 4), the loci had a mean length of 465.4 sites (range of 231–802 sites; SE ±5.6), and locus length was correlated with the number of PIC (R-squared 0.5139, *p*-value 2.2e-16) (Fig 4).

### Phylogenetic analyses

#### Concatenated analyses

**Fig 5:**
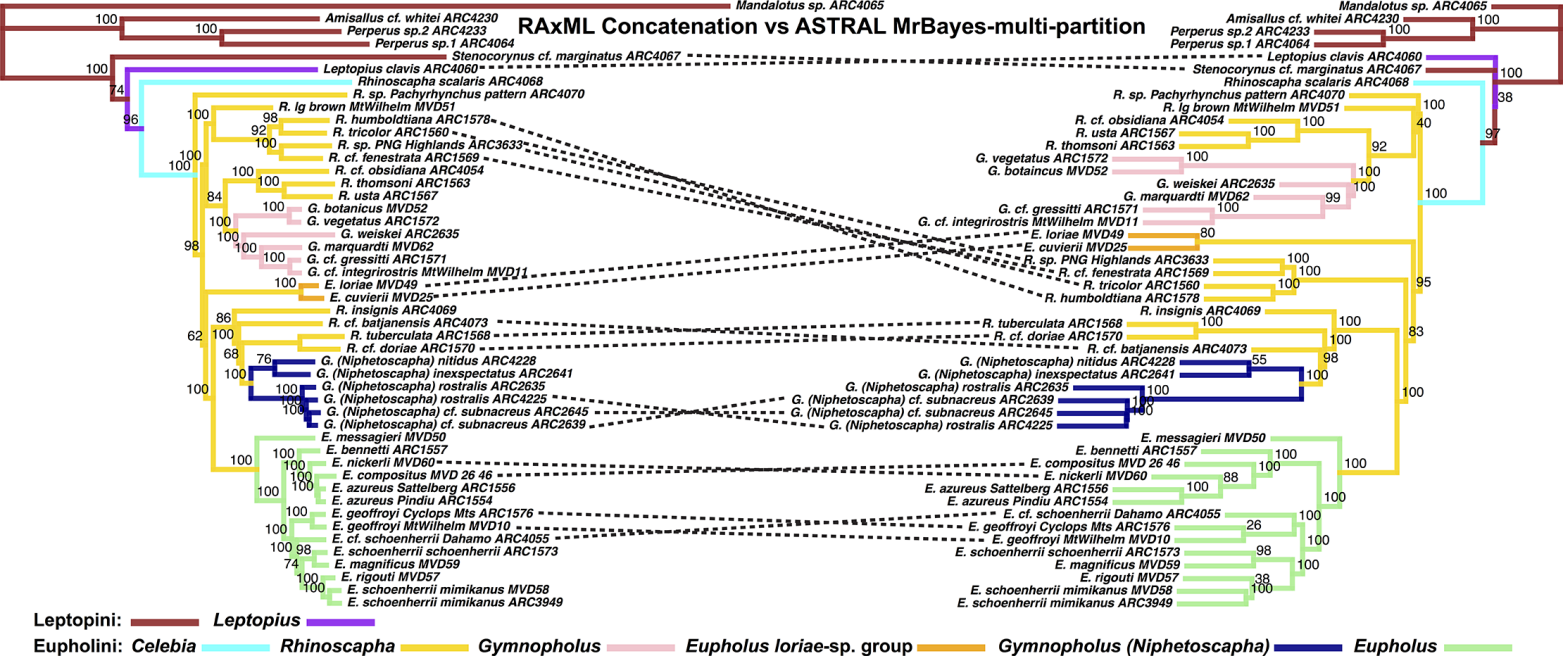
Phylogenetic tree results of the Eupholini weevils, branch colors correspond to species clades: LEFT: RAxML tree from concatenation of loci. Dashed lines denote nodes that differ between trees. Node values indicate bootstrap support values. RIGHT: ASTRAL species tree, input trees derived from multi-partitioned MrBayes analyses of individual gene trees. Node values are derived from posterior distribution of MrBayes trees (minus burn-in) where each sample of the MCMC generation represents a bootstrap sample to ASTRAL.

PartitionFinder suggested that the optimal scheme for the concatenated alignment contained 126 partitions, and ML analysis inferred a tree where most of the nodes were highly supported (≥95% bootstrap support (bs)) (Fig 5). We recovered the Leptopini as a paraphyletic grade containing the Eupholini. The genus *Celebia* was sister to all of the other Eupholini in all analyses. The three main genera of the Eupholini (*Rhinoscapha*, *Eupholus* and *Gymnopholus)* were polyphyletic. *Rhinoscapha* appeared in three separate places, the *R*. *tricolor*-clade sister to *R*. *usta*-clade which is sister to the *Gymnopholus* clade containing the two subgenera *Gymnopholus* s. str. and *Symbiopholus*. The *Eupholus loriae*-group was sister to the remaining taxa. These include the third subgenus of *Gymnopholus*, *Niphetoscapha* which was sister to the *R*. *tuberculata*-clade. The clade of *Niphetoscapha*+*R*. *tuberculata*-clade was sister to the *R*. *doriae-*clade. The clade of *Niphetoscapha*+*R*. *tuberculata*-clade+*R*. *doriae-*clade is sister to the remaining members of *Eupholus*.

#### Species tree analyses

**Fig 6:**
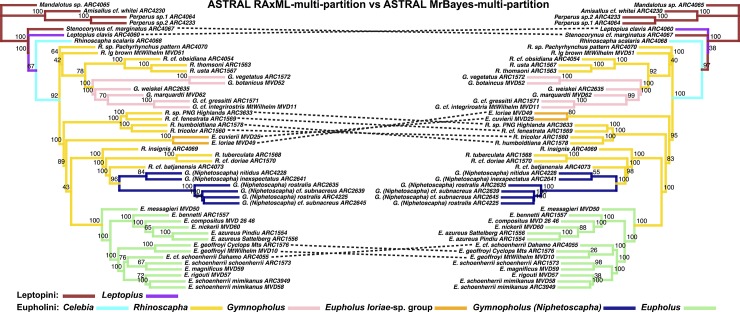
LEFT: ASTRAL species tree derived from multi-partitioned RAxML trees. Node values indicate bootstrap support values. RIGHT: ASTRAL species tree, input trees derived from multi-partitioned MrBayes analyses of individual gene trees. Node values are derived from posterior distribution of MrBayes trees (minus burn-in) where each sample of the MCMC generation represents a bootstrap sample to ASTRAL.

**Fig 7:**
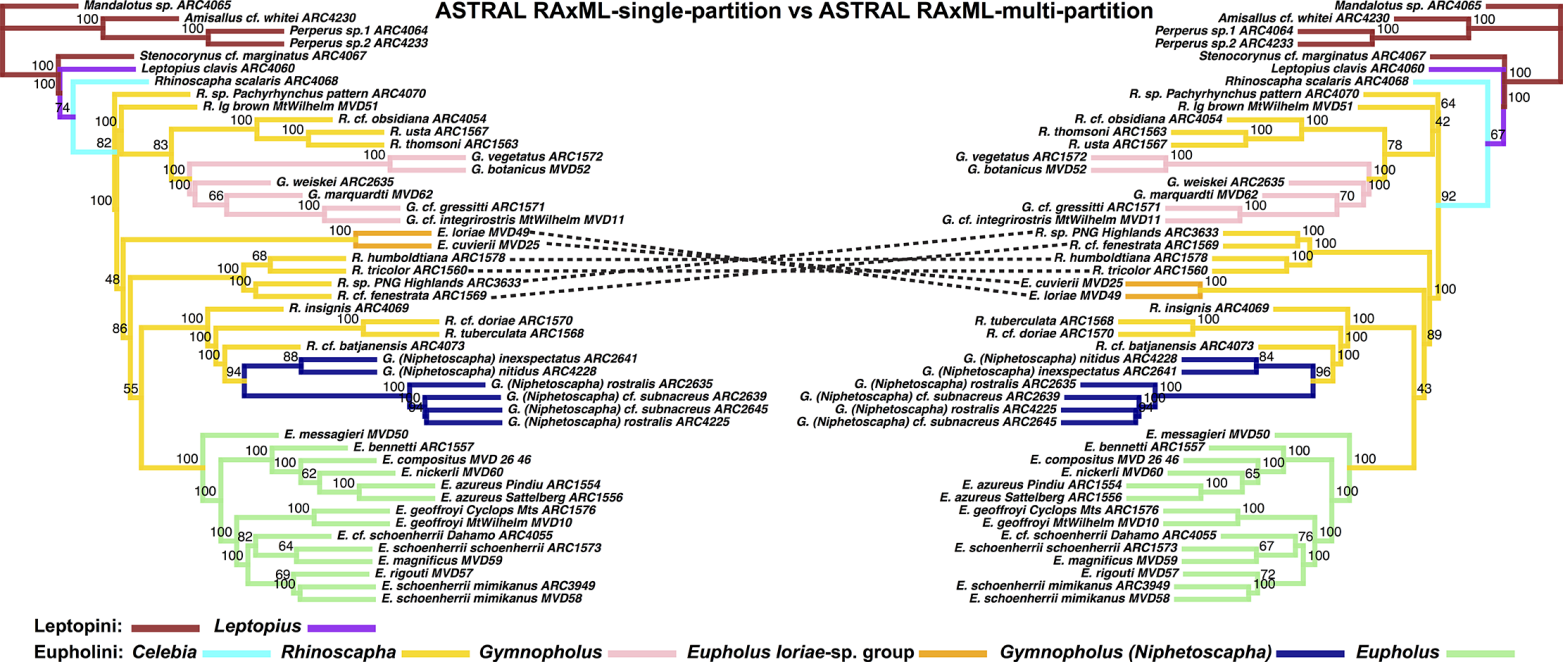
Phylogenetic tree results of the Eupholini weevils, branch colors correspond to species clades: LEFT: SVDQuartets species tree. Dashed lines denote nodes that differ between trees. Node values indicate bootstrap support values. RIGHT: ASTRAL species tree, input trees derived from multi-partitioned MrBayes analyses of individual gene trees. Node values indicate support values of MrBayes posterior (minus burn-in) used as ASTRAL bootstrap replicates.

**Fig 8:**
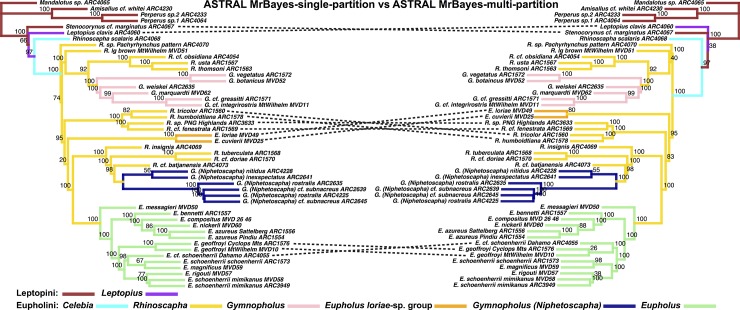
Phylogenetic tree results of the Eupholini weevils, branch colors correspond to species clades: LEFT: ASTRAL species tree, input trees derived from single-partitioned MrBayes analyses (each gene tree reconstructed using a single partition), of individual gene trees. RIGHT: ASTRAL species tree, input trees derived from multi-partitioned MrBayes analyses of individual gene trees. Node values indicate support values of MrBayes posterior used as ASTRAL bootstrap replicates.

**Fig 9:**
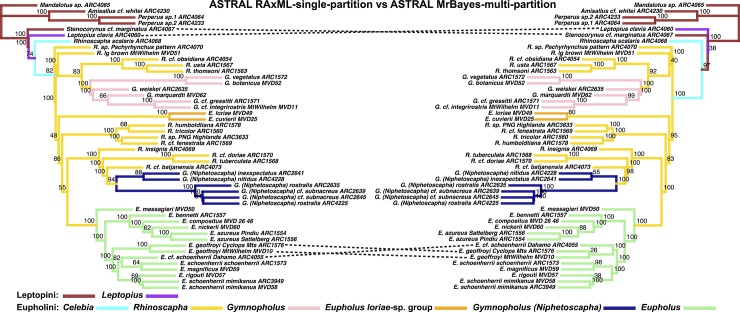
Phylogenetic tree results of the Eupholini weevils, branch colors correspond to species clades: LEFT: ASTRAL species tree, input trees derived from single-partitioned RAxML analyses (each gene tree reconstructed using a single partition), of individual gene trees. RIGHT: ASTRAL species tree, input trees derived from multi-partitioned RAxML analyses of individual gene trees. Node values indicate bootstrap support values.

The PartitionFinder results, using the initial 6 character sets under the GTRGAMMA site rate substitution model (for use in RAxML), had the majority of loci with between 1–2 character partitioning schemes: 53 loci with a single partition, 204 loci with 2 partitions, 102 loci with 3 partitions and 9 loci with 4 partitions. There was no difference in topology between the ASTRAL species tree for the full analyses, and the ASTRAL species tree where we removed loci producing gene trees having the lowest 10% quantile of average bootstrap support removed, and the ASTRAL species tree where we removed both these low support species trees and loci we identified as being potentially over-saturated (see S1 and S2 Figs). There was also essentially no difference in support between these tree subsets. Although some nodes varied in their support across analyses, poorly supported nodes did not show increased support between the reduced subsets of trees and the complete set (S1 and S2 Figs). The relationships between the Leptopini and Eupholini remained the same between species tree topologies and concatenated gene tree. The species trees recovered the same species groups as in the concatenated analyses, but the backbone of the tree was substantially different. For the particular differences between species groups see Figs 5–9. The PartitionFinder results, using the initial 6 character sets in Bayesian phylogenetic analyses (MrBayes MCCT trees) selected the majority of loci with more than 2 partitions according to the AICc metric: 16 loci with 1 partition, 109 loci with 2 partitions, 148 loci with 3 partitions, 81 loci with 4 partitions and 14 loci with 5 partitions, 0 loci had 6 partitions. We also compared locus length to the number of partitions in the MrBayes and RaxML analyses, and found that in both analyses locus length was positively correlated with the number of partitions ([MrBayes: *p-*value = 5.251e-08, R-squared = 0.078], [RaxML: *p-*value = 2.2e-16, R-squared = 0.2237]), (see S1 File, partitioning data). We assessed convergence between the two independent MrBayes runs using the average standard deviation of split frequencies using a custom unix script (see S1 Supporting Information Links). All but two analyses were at or below 0.01 in average standard deviation of split frequencies, the exceptions being 0.02 and 0.03, we subsequently produced the MrBayes MCCT trees.

The most substantial topological difference is between the RAxML-concatenated gene tree and the species tree analyses (see Fig 5, Table 1). Although the partitioning results differed in the number of partitions, the ASTRAL species trees derived from MrBayes-multi-partition gene trees were only slightly different from the RAxML-multi-partition derived gene trees (Fig 6). The *Eupholus loriae*-group and the *Rhinoscapha tricolor*-clade switched as being the sister clade to the remaining Eupholini. This node was not well supported in both analyses. These clades showed similar differences between both ASTRAL-RAxML-multi-partition and ASTRAL-RAxML-single-partition (Fig 7). The same difference also appears between the *Eupholus loriae*-group and the *Rhinoscapha tricolor*-clade in the ASTRAL MrBayes-single-partition and ASTRAL-MrBayes-multi-partition (Fig 8). The two MrBayes (single and multi-partitioned) derived ASTRAL species trees had the additional difference between the placement of the *E*. *geoffroyi* clade. The ASTRAL-RAxML-single-partition and MrBayes-multi-partition species trees only differed between the placement of the *E*. *geoffroyi* clade and the placement of *Letopius clavis* (Fig 9).

**Table 1:** Pairwise comparisons of topological distances between different tree reconstruction methods used. Tree to tree topological distance metrics used the subtree prune and regraft distance (SPR_DIST), Robinson-Foulds distance (RF_DIST), path distance metric between pairs of taxa (PATH.DIFF_DIST). The ASTRAL trees based on multiple partitions are the ASTRAL RAxML tree using six character-sets (astral_raxmltree), ASTRAL RAxML tree based on single character-sets/partitions (astral_singlepart_raxmltree), ASTRAL MrBayes tree using six character-sets (astral_mrbtree) and ASTRAL MrBayes tree based on single character-sets/partitions (astral_singlepart_mrbtree). Results of lower triangle shown.

SPR_DIST	raxml_concat	astral_raxmltree	astral_singlepart_raxmltree	astral_mrbtree	astral_singlepart_mrbtree	svdq_tree
raxml_concat	**-**					
astral_raxmltree	5	**-**				
astral_singlepart_raxmltree	5	2	**-**			
astral_mrbtree	7	3	3	**-**		
astral_singlepart_mrbtree	5	1	1	4	**-**	
svdq_tree	3	5	3	5	4	**-**
**RF_DIST**	raxml_concat	astral_raxmltree	astral_singlepart_raxmltree	astral_mrbtree	astral_singlepart_mrbtree	svdq_tree
raxml_concat	**-**					
astral_raxmltree	14	**-**				
astral_singlepart_raxmltree	14	4	**-**			
astral_mrbtree	18	8	8	**-**		
astral_singlepart_mrbtree	12	2	2	10	**-**	
svdq_tree	10	10	6	10	8	**-**
**PATH.DIFF_DIST**	raxml_concat	astral_raxmltree	astral_singlepart_raxmltree	astral_mrbtree	astral_singlepart_mrbtree	svdq_tree
raxml_concat	**-**					
astral_raxmltree	46.5	**-**				
astral_singlepart_raxmltree	44.8	27.7	**-**			
astral_mrbtree	54.4	24.1	29.1	**-**		
astral_singlepart_mrbtree	37.5	23.0	15.9	33.3	**-**	
svdq_tree	42.0	33.3	19.0	32.7	24.6	**-**

**Fig 10:**
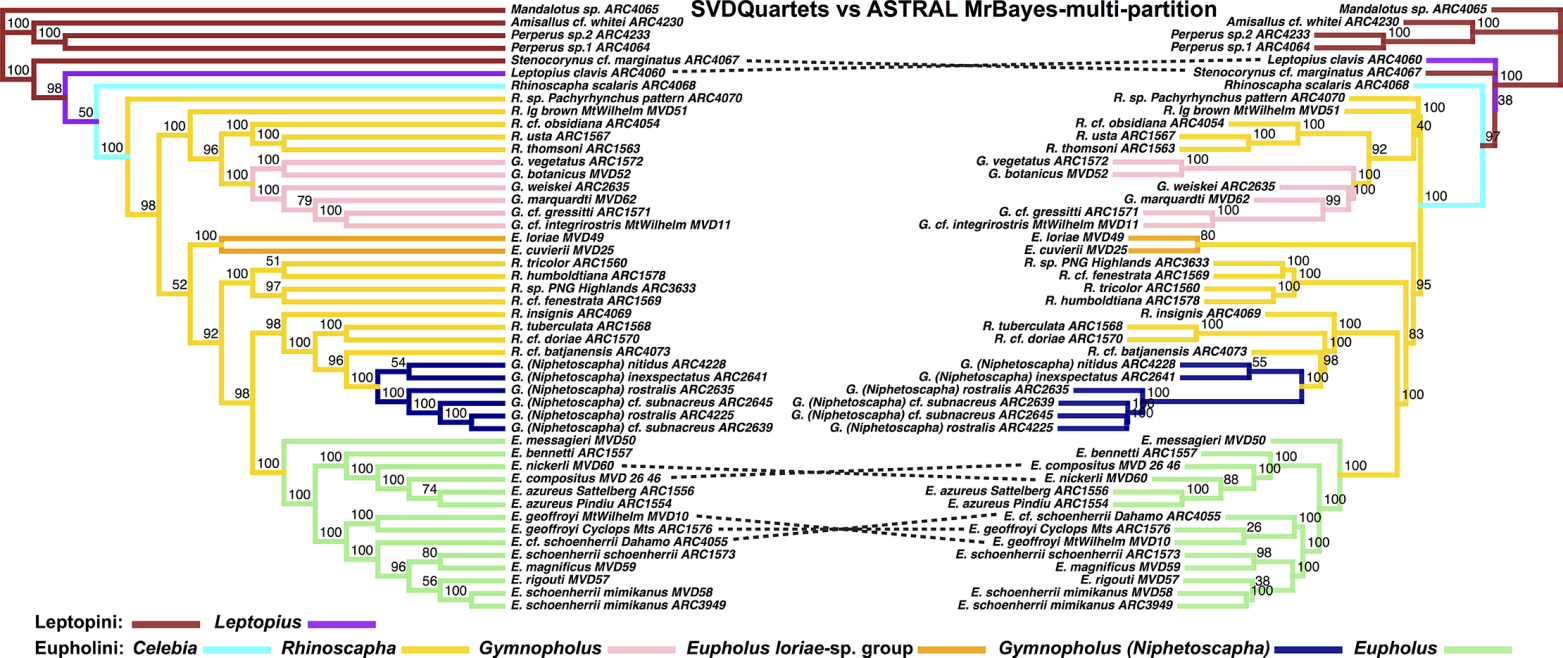
Linear regression of logit proportion of UCE loci captured versus specimen age. Number of UCE loci and specimen age for; *Xylocopa* (carpenter bees) from Blaimer et al. 2016, *Aphelocoma* (scrub-jays) from McCormack et al. 2016, Eupholini (smurf weevils) from this study. Specimen age is in years from when individual was first collected and preserved. Regressions show a decline in the number of UCE loci captured as specimen age increases, the rate of decline is similar between studies.

The results from the SVDQuartets species tree were much the same as those from the other two species tree analyses. In particular, the backbone topology was most similar to the results of the ASTRAL species tree derived from the MrBayes-multi-partition analyses. One difference is that *E*. *compositus* is sister to *E*. *azureus* in the SVDQuartets species tree (Fig 10). Also, *E*. cf. *schoenherrii* is sister to the *E*. *geoffroyi*-clade in the SVDQuartets and ASTRAL-MrBayes-multi-partition trees, whereas the ASTRAL-RAxML-multi-partition tree have the *E*. *geoffroyi*-clade sister to the other *E*. *schoenherrii* species (Figs 6 and 7). Only the placement of *E*. *cf*. *schoenherii* was strongly supported in both the SVDQuartets and ASTRAL-MrBayes-multi-partition trees, but no other conflicting nodes were strongly supported.

The topological tree-to-tree metrics derived from the RF-dist, Path-dist and SPR-dist all give relatively the same result (Table 1). These values show that the ASTRAL-RAxML-single-partition and ASTRAL-MrBayes-single-partition trees have the most similar topology. They are then most similar to the ASTRAL-RAxML species tree based on multiple partitions. The ASTRAL-RAxML-single-partition species tree is the most similar of the species tree methods to the RAxML-concatenated gene tree. The Path-dist metric shows that the ASTRAL-MrBayes species tree based on multiple partitions and SVDQuartets species trees are slightly more similar to each other than is the ASTRAL-RAxML-multi-partition species tree to the SVDQuartets species trees, while the other metrics give equal difference between the ASTRAL species trees and the SVDQuartets species trees. Most importantly the species tree methods are more similar to each other, except for the SVDQuartets species tree as mentioned above, than they are to the concatenated RAxML topology.

### UCE capture and preservation

**Fig 11:**
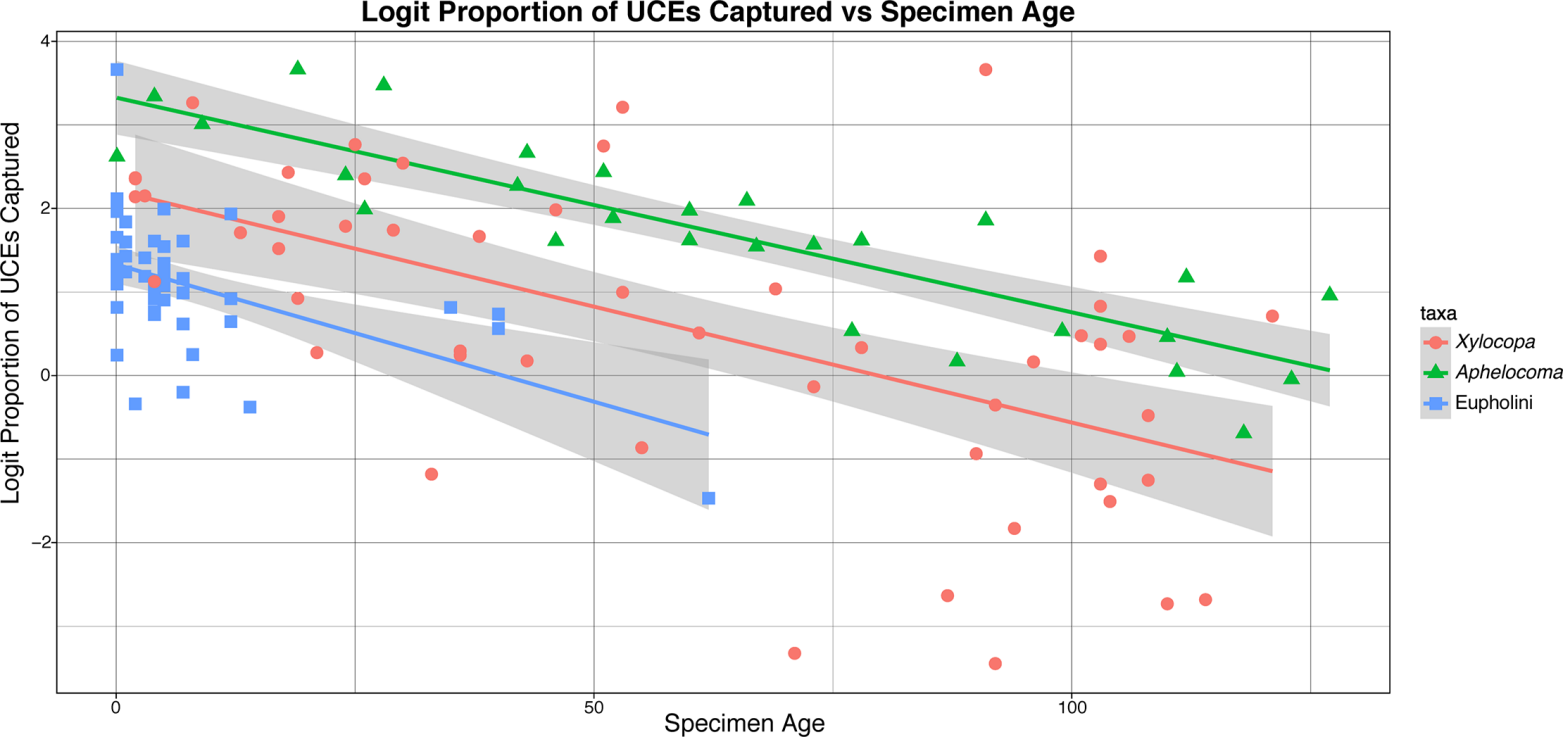
Linear regression of logit proportion of UCE loci captured verses specimen age. Number of UCE loci and specimen age for; *Xylocopa* (carpenter bees) from Blaimer et al. 2016, *Aphelocoma* (scrub-jays) from McCormack et al. 2016, Eupholini (smurf weevils) from this study. Specimen age is in years from when individual was first collected and preserved. Regressions show a decline in the number of UCE loci captured as specimen age increases, the rate of decline is similar between studies.

Within the Eupholini dataset, we used a GLM to test which variable (specimen age and or preservation type) has the most influence on the UCE number, we found that specimen age was the only significant contributor to the number of UCEs captured (*p*-value of 0.00886 for the coefficient of specimen age vs *p*-value of 0.75418 for collection type). Next, we compared our dataset to those of two other studies which utilized UCEs and museum samples, specifically *Xylocopa* (carpenter bees) [11] and *Aphelocoma* (scrub-jays) [10]. From the likelihood-ratio test results we find that the preferred model is one with a single slope (Chi-square test *p*-value 0.8598), indicating that UCE capture rate through time is approximately the same between datasets (Fig 11).

## Discussion

UCEs are now widely used for different vertebrate taxa [5,7,50–56]. In invertebrates, UCEs have been used in Hymenoptera (primarily ants [8,21,11,57–60]), but also Coleoptera [61] and arachnids [62]. Transcriptome-based analyses constitute another major source of phylogenomic data in arthropods [63–69]. For hyperdiverse beetles, there is great potential for both UCE- and exon-based markers to help resolve relationships within this diverse group. Here, we tested the utility of UCEs designed across Coleoptera on a radiation of weevils from Australasia. We captured significantly more loci than Baca et al. (2017) [61] which focused on Adephaga beetles (305 at 50% vs [368 at 70%, 537 at 50% this study]). This is probably due to the phylogenetic distance between the species from which the probes were designed and the target taxa. The Coleoptera UCE markers capture a similar number of loci when compared to the 1st round of Hymenoptera UCEs. This suggests that with more forthcoming weevil genomes (personal comm. D. McKenna), capturing thousands of UCE loci could be possible. Why UCEs are not more universal within Coleoptera as compared to vertebrate lineages is still an open question. One possible explanation is that beetles are extremely diverse both in terms of life history and genomics. Recent studies of beetle genomes show a large diversity in their size and complexity, potentially even incorporating fungal genomic elements [70]. Additionally, Coleoptera originated ~300 Ma [71,72], compounding the problem, for comparison the Mammalia–Root ~223 Ma [73], and the Palaeognathae (ratite) and Neognathae (birds) divergence of ~100 Ma [74].

Here, we also find that we can enrich UCE loci from Coleoptera museum specimens, which can significantly aid sampling efforts for future studies, similar to other studies [11,75,76]. Our results are consistent with the findings of McCormack et al. 2016 [10] and Blaimer et al. 2016 [11], who showed a gradual decline in UCE capture rate with specimen age (Fig 11). Generally speaking, the capture rate we observed correlates similarly to the aforementioned studies, according to a likelihood ratio test between models’ slopes, which is re-assuring because Coleoptera, due to their thicker exoskeletons compared to other insects (e.g. *Drosophila*), may undergo a different decomposition process following collection. We hypothesize that in large insects and or those with thick cuticle, their initial drying time (period spent air drying for preservation after dispatching the insect) can be longer than other insects, and length of initial drying time is key to preventing decay and preserving the specimens for later use [10,11,77,78]. We suspect that in the case of tropical Coleoptera with thick exoskeletons, more decay tends to occur because of their thickened cuticle and the humid conditions under which they are collected, however this needs to be formally quantified. Additionally, there is substantial variance in the procedures through which beetle specimens are preserved. For example, when collecting weevils on the Solomon Islands in 1959, weevil researcher Dr. Charles O’Brien, would actively dry his weevils at the end of the day on top of a kerosene powered lantern before packing them into a tin with cotton (personal communication). With such information one can start to make better sense as to why some historic samples fail and others work relatively well. For example potentially overheating samples (as described above) could shear DNA as well as specimens that were not dried quickly could have DNA degradation due to enzymatic decomposition. Having such information will be invaluable to identify particular collections that may harbor particularly valuable specimens for genomic work. More systematic studies need to be undertaken to fully explore this area of museum phylogenomics in beetles and preservation methods, but our results at least show the feasibility of using tropical beetle specimens to bolster sampling.

All of our phylogenetic reconstructions show the same result concerning the classification of the Eupholini: the main genera *Eupholus*, *Gymnopholus* and *Rhinoscapha* are polyphyletic. This should not be too surprising because they were described and delineated in the late 19^th^ and early 20^th^ centuries when there were fewer known species and inadequate examination of internal structures (such as genitalia) during classification. For instance, in *Eupholus*, species were apparently assigned to that genus mostly based on coloration, i.e. all bright blue or green species were placed in *Eupholus* [16].

Lastly, for the majority of loci we found that by using multiple character sets which resulted in multiple partition schemes was a better fit for the data, than a single partitioning scheme according to AICc model selection. This indicates that using each UCE locus as a single partition as commonly performed may not be the best fit for the data. With these data, differences appear between species tree topologies based on both the partitioning strategy and initial method used to reconstruct the gene trees. This indicates that using only single partitions may not be adequate for many UCE loci. Additionally, the method used to reconstruct the gene trees also has an effect on the resulting species tree topology. What general effect gene tree partitioning has on species tree reconstruction and resulting branch lengths remains an open question.

We find great potential for UCE loci to tackle some of the more challenging issues in beetle systematics such as in the Eupholini radiation. We suggest that the use of UCEs and similar phylogenomic markers may also provide insights into the deeper relationships of diverse, higher-order taxonomic groups such as the Curculionoidea. Museum collections should also be recognized as a more prominent genomic resource, especially for tropical arthropods. Here, we demonstrate with a logistically challenging group how collection efforts of the past can aid current and future studies by increasing sampling, an undertaking that would otherwise require many years of effort to recollect the same samples. What steps museums should take to help preserve this genomic resource should be studied in a systematic fashion across institutions, as many existing museum specimens may be irreplaceable.

## Supporting information

S1 Table Data results. (XLS)

S2 Table Results of UCE loci alignments percent completeness (number of taxa missing from total number of taxa). Matrix % complete indicate the percent completeness of the UCE loci alignments. 1st round of sequencing indicates the number of UCE loci alignments from the first round of sequencing of the 48 samples on an illumina HiSeq 3000 half lane. 2nd round of sequencing indicates the number UCE loci alignments from the concatenation of reads from the second round of sequencing (same 48 samples on an illumina HiSeq 3000 half lane) and the first round of sequencing.
(XLS)

S3 Table Table of specimens used in this stud with museum where they are deposited the year the specimen was dispatched and the subsequent preservation type. Museum abbreviations as follows: SNSB-Zoological State Collection, München (ZSM), State Museum of Natural History Karlsruhe (SMNK), California Academy of Sciences (CAS).
(XLS)

S1 Fig ASTRAL-RAxML partitioned Eupholini tree, minus 10% lowest quantile of average bootstrap values loci. (PDF)

S2 Fig ASTRAL-RAxML partitioned Eupholini species tree, minus 10% lowest quantile of average bootstrap values and potentially oversaturated loci. (PDF)

S1 Supporting Information Links Links to supporting R/unix code, alignments and raw sequence reads. (DOCX)

S1 File Partitioning strategy by loci. (ZIP)

## References

[1] Oberprieler RG, Marvaldi AE, Anderson RS. Zootaxa,Weevils, weevils, weevils everywhere. Zootaxa. 2007;1668: 491–520.

[2] McKenna DD, Sequeira AS, Marvaldi AE, Farrell BD. Temporal lags and overlap in the diversification of weevils and flowering plants. Proc Natl Acad Sci. 2009;106: 7083–7088. doi: 10.1073/pnas.0810618106 19365072 10.1073/pnas.0810618106 PMC2678426

[3] Arnoldi L. Family Eobelidae L. Arnoldi fam. nov. In: Arnoldi LV, Zherikhin V., Nikitrin LM, Ponomarenko A., editors. Mesozoic Coleoptera. English tr. Moscow, Russia: Nauka Publishers; 1977 pp. 195–241.

[4] Faircloth BC. Identifying conserved genomic elements and designing universal bait sets to enrich them. Methods Ecol Evol. 2017; doi: 10.1111/2041-210X.12754

[5] Faircloth BC, McCormack JE, Crawford NG, Harvey MG, Brumfield RT, Glenn TC. Ultraconserved Elements Anchor Thousands of Genetic Markers Spanning Multiple Evolutionary Timescales. Syst Biol. 2012;61: 717–726. doi: 10.1093/sysbio/sys004 22232343 10.1093/sysbio/sys004

[6] Jesovnik A, Sosa-Calvo J, Lloyd MW, Branstetter MG, Fernandez F, Schultz TR. Phylogenomic species delimitation and host-symbiont coevolution in the fungus-farming ant genus *Sericomyrmex* Mayr (Hymenoptera: Formicidae): ultraconserved elements (UCEs) resolve a recent radiation. Syst Entomol. 2017;42: 523–542. doi: 10.1111/syen.12228

[7] Moyle RG, Oliveros CH, Andersen MJ, Hosner PA, Benz BW, Manthey JD, et al Tectonic collision and uplift of Wallacea triggered the global songbird radiation. Nat Commun. 2016;7: 12709 doi: 10.1038/ncomms12709 27575437 10.1038/ncomms12709 PMC5013600

[8] Blaimer BB, Brady SG, Schultz TR, Lloyd MW, Fisher BL, Ward PS. Phylogenomic methods outperform traditional multi-locus approaches in resolving deep evolutionary history: a case study of formicine ants. BMC Evol Biol. 2015;15: 271 doi: 10.1186/s12862-015-0552-5 26637372 10.1186/s12862-015-0552-5 PMC4670518

[9] Faircloth BC, Sorenson L, Santini F, Alfaro ME. A Phylogenomic Perspective on the Radiation of Ray-Finned Fishes Based upon Targeted Sequencing of Ultraconserved Elements (UCEs). PLoS One. 2013;8: e65923 doi: 10.1371/journal.pone.0065923 23824177 10.1371/journal.pone.0065923 PMC3688804

[10] McCormack JE, Tsai WLE, Faircloth BC. Sequence capture of ultraconserved elements from bird museum specimens. Mol Ecol Resour. 2016;16: 1189–1203. doi: 10.1111/1755-0998.12466 26391430 10.1111/1755-0998.12466

[11] Blaimer BB, Lloyd MW, Guillory WX, Brady SG. Sequence Capture and Phylogenetic Utility of Genomic Ultraconserved Elements Obtained from Pinned Insect Specimens. PLoS One. 2016;11: e0161531 doi: 10.1371/journal.pone.0161531 27556533 10.1371/journal.pone.0161531 PMC4996520

[12] Bi K, Linderoth T, Vanderpool D, Good JM, Nielsen R, Moritz C. Unlocking the vault: next-generation museum population genomics. Mol Ecol. 2013;22: 6018–6032. doi: 10.1111/mec.12516 24118668 10.1111/mec.12516 PMC4134471

[13] Tänzler R, Van Dam MH, Toussaint EFA, Suhardjono YR, Balke M, Riedel A. Macroevolution of hyperdiverse flightless beetles reflects the complex geological history of the Sunda Arc. Sci Rep. 2016;6: 18793 doi: 10.1038/srep18793 26742575 10.1038/srep18793 PMC4732383

[14] Riedel A. Revision of the genus Penthoscapha Heller (Coleoptera, Curculionoidea, Entiminae, Eupholini) with notes on the genera of Eupholini from New Guinea. Zootaxa. 2009;2224: 1–29.

[15] Setliff GP. Annotated checklist of weevils from the Papuan region (Coleoptera, Curculionoidea) [Internet]. Zootaxa. 2007 Available: http://www.coleoptera-atlas.com/IMG/pdf/curculionidae_from_png_by_gregory_p._setliff.pdf

[16] Riedel A. Two New Species Of Eupholus Boisduval (Coleoptera, Curculionidae, Entiminae) From West New Guinea, A Discussion Of Their Taxonomic Characters, And Notes On Nomenclature. Zootaxa. 2002;90: 1–16.

[17] Gressitt JL, Sedlacek J, Szent-Ivany JJH. Flora and Fauna on Backs of Large Papuan Moss-Forest Weevils. Science. 1965;150 Available: http://science.sciencemag.org/content/150/3705/1833/tab-pdf 10.1126/science.150.3705.1833 17841980

[18] Gressitt JL, Sedlacek J. Papuan weevil genus Gymnopholus: supplement and further studies in epizoic symbiosis. Pacific Insects. 1967;9: 481–500. Available: http://hbs.bishopmuseum.org/pi/pdf/9(3)-481.pdf

[19] Mamanova L, Coffey AJ, Scott CE, Kozarewa I, Turner EH, Kumar A, et al Target-enrichment strategies for next-generation sequencing. Nat Methods. 2010;7: 111–118. doi: 10.1038/nmeth.1419 20111037 10.1038/nmeth.1419

[20] Blumenstiel B, Cibulskis K, Fisher S, DeFelice M, Barry A, Fennell T, et al Targeted Exon Sequencing by In-Solution Hybrid Selection. Current Protocols in Human Genetics. Hoboken, NJ, USA: John Wiley & Sons, Inc.; 2010 p. Unit 18.4. doi: 10.1002/0471142905.hg1804s66 10.1002/0471142905.hg1804s66 20582916

[21] Faircloth BC, Branstetter MG, White ND, Brady SG. Target enrichment of ultraconserved elements from arthropods provides a genomic perspective on relationships among Hymenoptera. Mol Ecol Resour. 2015;15: 489–501. doi: 10.1111/1755-0998.12328 25207863 10.1111/1755-0998.12328 PMC4407909

[22] Faircloth BC. Illumiprocessor—software for Illumina read quality filtering. 2011; doi: 10.6079/J9ILL

[23] Lohse M, Bolger AM, Nagel A, Fernie AR, Lunn JE, Stitt M, et al RobiNA: a user-friendly, integrated software solution for RNA-Seq-based transcriptomics. Nucleic Acids Res. 2012;40: W622–W627. doi: 10.1093/nar/gks540 22684630 10.1093/nar/gks540 PMC3394330

[24] Bolger AM, Lohse M, Usadel B. Trimmomatic: a flexible trimmer for Illumina sequence data. Bioinformatics. 2014;30: 2114–2120. doi: 10.1093/bioinformatics/btu170 24695404 10.1093/bioinformatics/btu170 PMC4103590

[25] Del Fabbro C, Scalabrin S, Morgante M, Giorgi FM, Binkley G. An Extensive Evaluation of Read Trimming Effects on Illumina NGS Data Analysis. PLoS One. 2013;8: e85024 doi: 10.1371/journal.pone.0085024 24376861 10.1371/journal.pone.0085024 PMC3871669

[26] Faircloth BC. PHYLUCE is a software package for the analysis of conserved genomic loci. Bioinformatics. 2016;32: 786–788. doi: 10.1093/bioinformatics/btv646 26530724 10.1093/bioinformatics/btv646

[27] Grabherr MG, Haas BJ, Yassour M, Levin JZ, Thompson DA, Amit I, et al Full-length transcriptome assembly from RNA-Seq data without a reference genome. Nat Biotechnol. 2011;29: 644–52. doi: 10.1038/nbt.1883 21572440 10.1038/nbt.1883 PMC3571712

[28] Katoh K, Misawa K, Kuma K, Miyata T. MAFFT: a novel method for rapid multiple sequence alignment based on fast Fourier transform. Nucleic Acids Res. 2002;30: 3059–66. Available: http://www.ncbi.nlm.nih.gov/pubmed/12136088 12136088 10.1093/nar/gkf436 PMC135756

[29] Heibl C. PHYLOCH: R language tree plotting tools and interfaces to diverse phylogenetic software packages. [Internet]. [cited 30 Jun 2017]. Available: http://www.christophheibl.de/Rpackages.html

[30] Stamatakis A. RAxML version 8: a tool for phylogenetic analysis and post-analysis of large phylogenies. Bioinformatics. 2014;30: 1312–1313. doi: 10.1093/bioinformatics/btu033 24451623 10.1093/bioinformatics/btu033 PMC3998144

[31] Mirarab S, Reaz R, Bayzid MS, Zimmermann T, Swenson MS, Warnow T. ASTRAL: genome-scale coalescent-based species tree estimation. Bioinformatics. 2014;30: i541–i548. doi: 10.1093/bioinformatics/btu462 25161245 10.1093/bioinformatics/btu462 PMC4147915

[32] Mirarab S, Warnow T. ASTRAL-II: coalescent-based species tree estimation with many hundreds of taxa and thousands of genes. Bioinformatics. 2015;31: i44–i52. doi: 10.1093/bioinformatics/btv234 26072508 10.1093/bioinformatics/btv234 PMC4765870

[33] Sayyari E, Mirarab S, H S, H. M, EJ C, N M, et al Fast Coalescent-Based Computation of Local Branch Support from Quartet Frequencies. Mol Biol Evol. 2016;33: 1654–1668. doi: 10.1093/molbev/msw079 27189547 10.1093/molbev/msw079 PMC4915361

[34] Chifman J, Kubatko L. Quartet Inference from SNP Data Under the Coalescent Model. Bioinformatics. 2014;30: 3317–3324. doi: 10.1093/bioinformatics/btu530 25104814 10.1093/bioinformatics/btu530 PMC4296144

[35] Chifman J, Kubatko L. Identifiability of the unrooted species tree topology under the coalescent model with time-reversible substitution processes, site-specific rate variation, and invariable sites. J Theor Biol. 2015;374: 35–47. doi: 10.1016/j.jtbi.2015.03.006 25791286 10.1016/j.jtbi.2015.03.006

[36] Swofford DL. Phylogenetic Analysis Using Parsimony PAUP* 4.0 beta version [Internet]. 2001. Available: http://benedick.rutgers.edu/software-manuals/PAUP4-manual.pdf

[37] Lanfear R, Frandsen PB, Wright AM, Senfeld T, Calcott B. PartitionFinder 2: New Methods for Selecting Partitioned Models of Evolution for Molecular and Morphological Phylogenetic Analyses. Mol Biol Evol. 2016;11: msw260 doi: 10.1093/molbev/msw260 10.1093/molbev/msw260 28013191

[38] Lanfear R, Calcott B, Ho SYW, Guindon S. PartitionFinder: Combined Selection of Partitioning Schemes and Substitution Models for Phylogenetic Analyses. Mol Biol Evol. 2012;29: 1695–1701. doi: 10.1093/molbev/mss020 22319168 10.1093/molbev/mss020

[39] Borowiec ML, Lee EK, Chiu JC, Plachetzki DC. Extracting phylogenetic signal and accounting for bias in whole-genome data sets supports the Ctenophora as sister to remaining Metazoa. BMC Genomics. 2015;16: 987 doi: 10.1186/s12864-015-2146-4 26596625 10.1186/s12864-015-2146-4 PMC4657218

[40] Philippe H, Forterre P. The rooting of the universal tree of life is not reliable. J Mol Evol. 1999;49: 509–23. Available: http://www.ncbi.nlm.nih.gov/pubmed/10486008 10486008 10.1007/pl00006573

[41] Ronquist F, Teslenko M, van der Mark P, Ayres DL, Darling A, Höhna S, et al MrBayes 3.2: Efficient Bayesian Phylogenetic Inference and Model Choice Across a Large Model Space. Syst Biol. 2012;61: 539–542. doi: 10.1093/sysbio/sys029 22357727 10.1093/sysbio/sys029 PMC3329765

[42] Sukumaran J, Holder MT. DendroPy: a Python library for phylogenetic computing. Bioinformatics. 2010;26: 1569–1571. doi: 10.1093/bioinformatics/btq228 20421198 10.1093/bioinformatics/btq228

[43] Reaz R, Bayzid MS, Rahman MS. Accurate phylogenetic tree reconstruction from quartets: a heuristic approach. PLoS One. 2014;9: e104008 doi: 10.1371/journal.pone.0104008 25117474 10.1371/journal.pone.0104008 PMC4130513

[44] Schliep KP. phangorn: phylogenetic analysis in R. Bioinformatics. 2011;27: 592–593. doi: 10.1093/bioinformatics/btq706 21169378 10.1093/bioinformatics/btq706 PMC3035803

[45] Robinson DF, Foulds LR. Comparison of phylogenetic trees. Math Biosci. 1981;53: 131–147. doi: 10.1016/0025-5564(81)90043-2

[46] Steel MA, Penny D. Distributions of Tree Comparison Metrics-Some New Results. Syst Biol. 1993;42: 126 doi: 10.2307/2992536

[47] Hickey G, Dehne F, Rau-Chaplin A, Blouin C. SPR distance computation for unrooted trees. Evol Bioinform Online. 2008;4: 17–27. Available: http://www.ncbi.nlm.nih.gov/pubmed/19204804 19204804 10.4137/ebo.s419 PMC2614206

[48] De Oliveira Martins L, Mallo D, Posada D. A Bayesian Supertree Model for Genome-Wide Species Tree Reconstruction. Syst Biol. 2016;65: 397–416. doi: 10.1093/sysbio/syu082 25281847 10.1093/sysbio/syu082 PMC4851173

[49] Revell LJ. phytools: an R package for phylogenetic comparative biology (and other things). Methods Ecol Evol. 2012;3: 217–223. doi: 10.1111/j.2041-210X.2011.00169.x

[50] Crawford NG, Faircloth BC, McCormack JE, Brumfield RT, Winker K, Glenn TC. More than 1000 ultraconserved elements provide evidence that turtles are the sister group of archosaurs. Biol Lett. 2102;8: 783–786. doi: 10.1098/rsbl.2012.0331 22593086 10.1098/rsbl.2012.0331 PMC3440978

[51] Crawford NG, Parham JF, Sellas AB, Faircloth BC, Glenn TC, Papenfuss TJ, et al A phylogenomic analysis of turtles. Mol Phylogenet Evol. 2015;83: 250–257. doi: 10.1016/j.ympev.2014.10.021 25450099 10.1016/j.ympev.2014.10.021

[52] McCormack JE, Harvey MG, Faircloth BC, Crawford NG, Glenn TC, Brumfield RT. A Phylogeny of Birds Based on Over 1,500 Loci Collected by Target Enrichment and High-Throughput Sequencing. Alvarez N, editor. PLoS One. 2013;8: e54848 doi: 10.1371/journal.pone.0054848 23382987 10.1371/journal.pone.0054848 PMC3558522

[53] Smith BT, Harvey MG, Faircloth BC, Glenn TC, Brumfield RT. Target Capture and Massively Parallel Sequencing of Ultraconserved Elements for Comparative Studies at Shallow Evolutionary Time Scales. Syst Biol. 2014;63: 83–95. doi: 10.1093/sysbio/syt061 24021724 10.1093/sysbio/syt061

[54] Gilbert PS, Chang J, Pan C, Sobel EM, Sinsheimer JS, Faircloth BC, et al Genome-wide ultraconserved elements exhibit higher phylogenetic informativeness than traditional gene markers in percomorph fishes. Mol Phylogenet Evol. 2015;92: 140–146. doi: 10.1016/j.ympev.2015.05.027 26079130 10.1016/j.ympev.2015.05.027 PMC4583375

[55] Oswald JA, Harvey MG, Remsen RC, Foxworth DU, Cardiff SW, Dittmann DL, et al Willet be one species or two? A genomic view of the evolutionary history of *Tringa semipalmata*. Auk. 2016;133: 593–614. doi: 10.1642/AUK-15-232.1

[56] Alexander AM, Su Y-C, Oliveros CH, Olson K V., Travers SL, Brown RM. Genomic data reveals potential for hybridization, introgression, and incomplete lineage sorting to confound phylogenetic relationships in an adaptive radiation of narrow-mouth frogs. Evolution. 2017;71: 475–488. doi: 10.1111/evo.13133 27886369 10.1111/evo.13133

[57] Branstetter MG, Danforth BN, Pitts JP, Faircloth BC, Ward PS, Buffington ML, et al Phylogenomic analysis of ants, bees and stinging wasps: Improved taxon sampling enhances understanding of hymenopteran evolution. Nature Ecology and Evolution. 2017; 27:1019–1025 10.1016/j.cub.2017.03.027 28376325

[58] Branstetter MG, Longino JT, Reyes-López J, Schultz TR, Brady SG. Into the tropics: phylogenomics and evolutionary dynamics of a contrarian clade of ants Cold Spring Harbor Laboratory Press; bioRxiv. 2016; Available: http://www.biorxiv.org/content/early/2016/02/18/039966

[59] Branstetter MG, Longino JT, Ward PS, Faircloth BC. Enriching the ant tree of life: enhanced UCE bait set for genome-scale phylogenetics of ants and other Hymenoptera. Methods Ecol Evol. 2017;8: 768–776. doi: 10.1111/2041-210X.12742

[60] Bossert S, Murray EA, Blaimer BB, Danforth BN. The impact of GC bias on phylogenetic accuracy using targeted enrichment phylogenomic data. Mol Phylogenet Evol. 2017;111: 149–157. doi: 10.1016/j.ympev.2017.03.022 28390323 10.1016/j.ympev.2017.03.022

[61] Baca SM, Alexander A, Gustafson GT, Short AEZ. Ultraconserved elements show utility in phylogenetic inference of Adephaga (Coleoptera) and suggest paraphyly of “Hydradephega.” Syst Entomol. 2017; doi: 10.1111/syen.12244

[62] Starrett J, Derkarabetian S, Hedin M, Bryson RW, McCormack JE, Faircloth BC. High phylogenetic utility of an ultraconserved element probe set designed for Arachnida. Mol Ecol Resour. 2017;17: 812–823. doi: 10.1111/1755-0998.12621 27768256 10.1111/1755-0998.12621

[63] Brewer MS, Bond JE. Ordinal-Level Phylogenomics of the Arthropod Class Diplopoda (Millipedes) Based on an Analysis of 221 Nuclear Protein-Coding Loci Generated Using Next-Generation Sequence Analyses. PLoS One. 2013;8: e79935 doi: 10.1371/journal.pone.0079935 24236165 10.1371/journal.pone.0079935 PMC3827447

[64] Hedin M, Starrett J, Akhter S, Sch?nhofer AL, Shultz JW. Phylogenomic Resolution of Paleozoic Divergences in Harvestmen (Arachnida, Opiliones) via Analysis of Next-Generation Transcriptome Data. 2012;7: e42888 doi: 10.1371/journal.pone.0042888 22936998 10.1371/journal.pone.0042888 PMC3427324

[65] Misof B, Liu S, Meusemann K, Peters RS, Donath A, Mayer C, et al Phylogenomics resolves the timing and pattern of insect evolution. Science 2014;346 Available: http://science.sciencemag.org/content/346/6210/763 10.1126/science.1257570 25378627

[66] Bazinet AL, Cummings MP, Mitter KT, Mitter CW. Can RNA-Seq Resolve the Rapid Radiation of Advanced Moths and Butterflies (Hexapoda: Lepidoptera: Apoditrysia)? An Exploratory Study. PLoS One. 2013;8: e82615 doi: 10.1371/journal.pone.0082615 24324810 10.1371/journal.pone.0082615 PMC3853519

[67] Bazinet AL, Mitter KT, Davis DR, Van Nieukerken EJ, Cummings MP, Mitter C. Phylotranscriptomics resolves ancient divergences in the Lepidoptera. Syst Entomol. 2017;42: 305–316. doi: 10.1111/syen.12217

[68] Garrison NL, Rodriguez J, Agnarsson I, Coddington JA, Griswold CE, Hamilton CA, et al Spider phylogenomics: untangling the Spider Tree of Life. PeerJ. 2016;4: e1719 doi: 10.7717/peerj.1719 26925338 10.7717/peerj.1719 PMC4768681

[69] Carlson DE, Hedin M. Comparative transcriptomics of Entelegyne spiders (Araneae, Entelegynae), with emphasis on molecular evolution of orphan genes. PLoS One. 2017;12: e0174102 doi: 10.1371/journal.pone.0174102 28379977 10.1371/journal.pone.0174102 PMC5381867

[70] McKenna DD, Scully ED, Pauchet Y, Hoover K, Kirsch R, Geib SM, et al Genome of the Asian longhorned beetle (Anoplophora glabripennis), a globally significant invasive species, reveals key functional and evolutionary innovations at the beetle–plant interface. Genome Biol. 2016;17 doi: 10.1186/s13059-016-1088-8 27832824 10.1186/s13059-016-1088-8 PMC5105290

[71] Bethoux O. The earliest beetle identified. J Paleontol. 2009;83: 931–937. doi: 10.1666/08-158.1

[72] Smith DM, Marcot JD. The fossil record and macroevolutionary history of the beetles. Proc R Soc London B Biol Sci. 2015;282 Available: http://rspb.royalsocietypublishing.org/content/282/1805/20150060 10.1098/rspb.2015.0060 PMC4389621 25788597

[73] Tarver JE, dos Reis M, Mirarab S, Moran RJ, Parker S, O’Reilly JE, et al The Interrelationships of Placental Mammals and the Limits of Phylogenetic Inference. Genome Biol Evol. 2016;8: 330–44. doi: 10.1093/gbe/evv261 26733575 10.1093/gbe/evv261 PMC4779606

[74] Jarvis ED, Mirarab S, Aberer AJ, Li B, Houde P, Li C, et al Whole-genome analyses resolve early branches in the tree of life of modern birds. Science. 2014;346 Available: http://science.sciencemag.org/content/346/6215/1320/tab-pdf 10.1126/science.1253451 PMC4405904 25504713

[75] Tin MM-Y, Economo EP, Mikheyev AS. Sequencing Degraded DNA from Non-Destructively Sampled Museum Specimens for RAD-Tagging and Low-Coverage Shotgun Phylogenetics. Caramelli D, editor. PLoS One. 2014;9: e96793 doi: 10.1371/journal.pone.0096793 24828244 10.1371/journal.pone.0096793 PMC4020769

[76] Kanda K, Pflug JM, Sproul JS, Dasenko MA, Maddison DR. Successful Recovery of Nuclear Protein-Coding Genes from Small Insects in Museums Using Illumina Sequencing. O’Grady P, editor. PLoS One. 2015;10: e0143929 doi: 10.1371/journal.pone.0143929 26716693 10.1371/journal.pone.0143929 PMC4696846

[77] Lindahl T. Instability and decay of the primary structure of DNA. Nature. 1993;362: 709–15. doi: 10.1038/362709a0 8469282 10.1038/362709a0

[78] Gilbert MTP, Moore W, Melchior L, Worobey M. DNA Extraction from Dry Museum Beetles without Conferring External Morphological Damage. Hofreiter M, editor. PLoS One. 2007;2: e272 doi: 10.1371/journal.pone.0000272 17342206 10.1371/journal.pone.0000272 PMC1803022

