# Correction: Ultraconserved elements (UCEs) resolve the phylogeny of Australasian smurf-weevils

**DOI:** 10.1371/journal.pone.0205049

**Published:** 2018-09-27

**Authors:** Matthew H. Van Dam, Athena W. Lam, Katayo Sagata, Bradley Gewa, Raymond Laufa, Michael Balke, Brant C. Faircloth, Alexander Riedel

The figure captions for Figs 7, 9 and 10 are incorrect. The figures appear in the correct order. Please view the corrected figure captions for Figs 7, 9 and 10 here.

**Fig 7:**
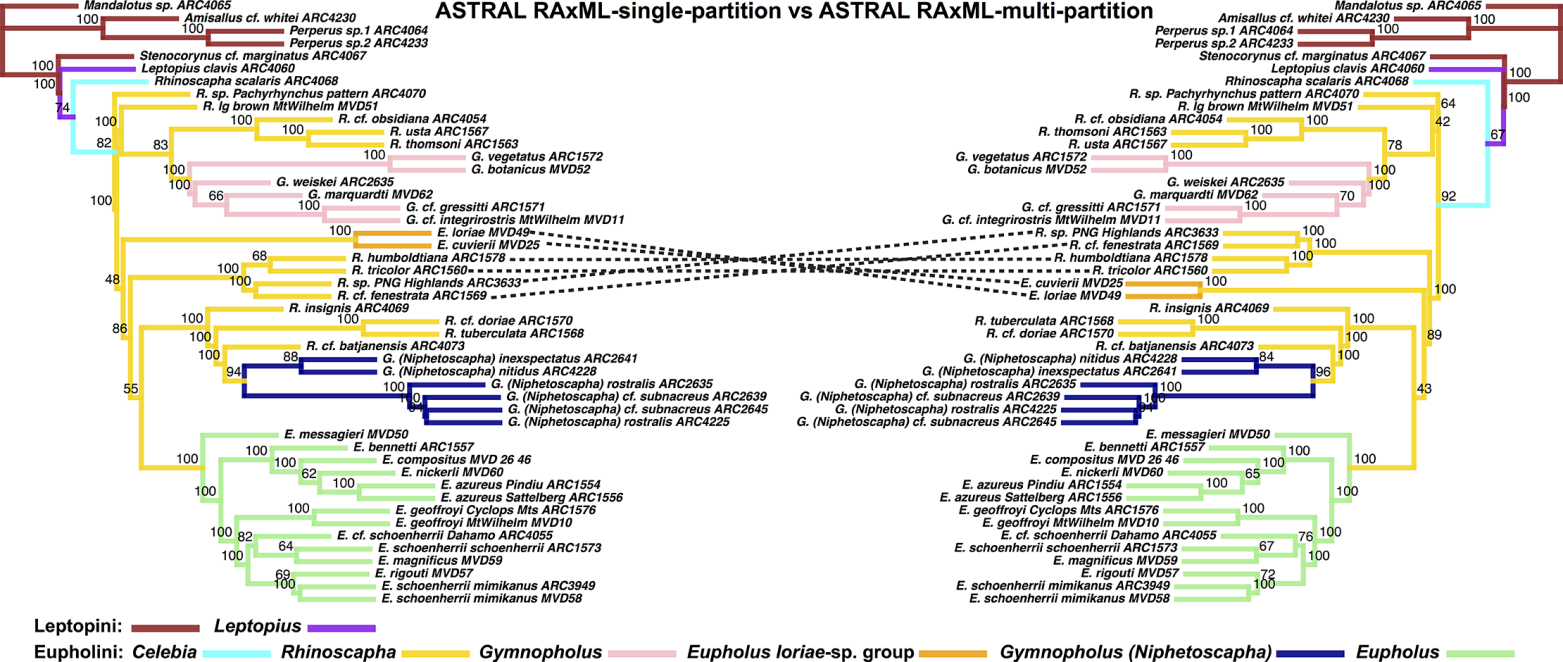
ASTRAL species tree derived from RAxML trees. Node values indicate bootstrap support values. LEFT: ASTRAL species tree, input trees derived from single-partitioned RAxML analyses of individual gene trees. RIGHT: ASTRAL species tree, input trees derived from multi-partitioned RAxML analyses of individual gene trees.

**Fig 9:**
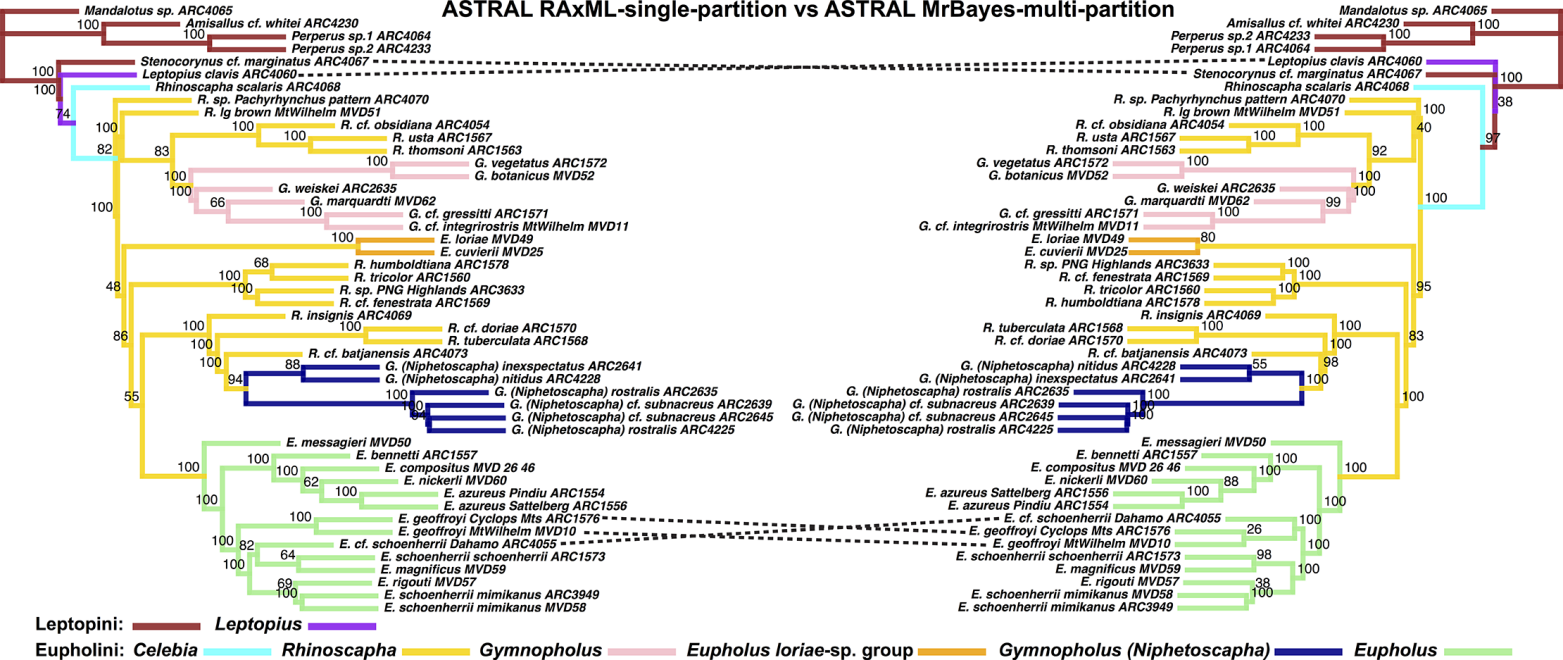
ASTRAL species tree derived from RAxML single-partition versus MrBayes multi-partition. LEFT: ASTRAL species tree, input trees derived from single-partitioned RAxML analyses (each gene tree reconstructed using a single partition), of individual gene trees. RIGHT: ASTRAL species tree, input trees derived from multi-partitioned MrBayes analyses of individual gene trees. Node values indicate support values of MrBayes posterior (minus burn-in) used as ASTRAL bootstrap replicates.

**Fig 10:**
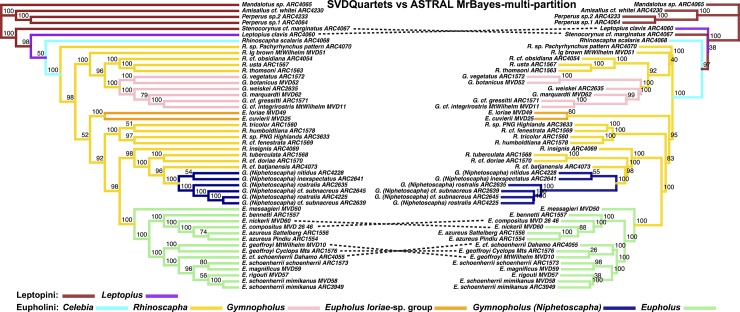
Phylogenetic tree results of the Eupholini weevils, branch colors correspond to species clades. LEFT: SVDQuartets species tree. Dashed lines denote nodes that differ between trees. Node values indicate bootstrap support values. RIGHT: ASTRAL species tree, input trees derived from multi-partitioned MrBayes analyses of individual gene trees. Node values indicate support values of MrBayes posterior (minus burn-in) used as ASTRAL bootstrap replicates.
