# Supplementary figures and images for: Ultraconserved elements (UCEs) resolve the phylogeny of Australasian smurf-weevils
Source: PLoS One. 2017 Nov 22;12(11):e0188044. doi: 10.1371/journal.pone.0188044 (PMC5699822; doi:10.1371/journal.pone.0188044)

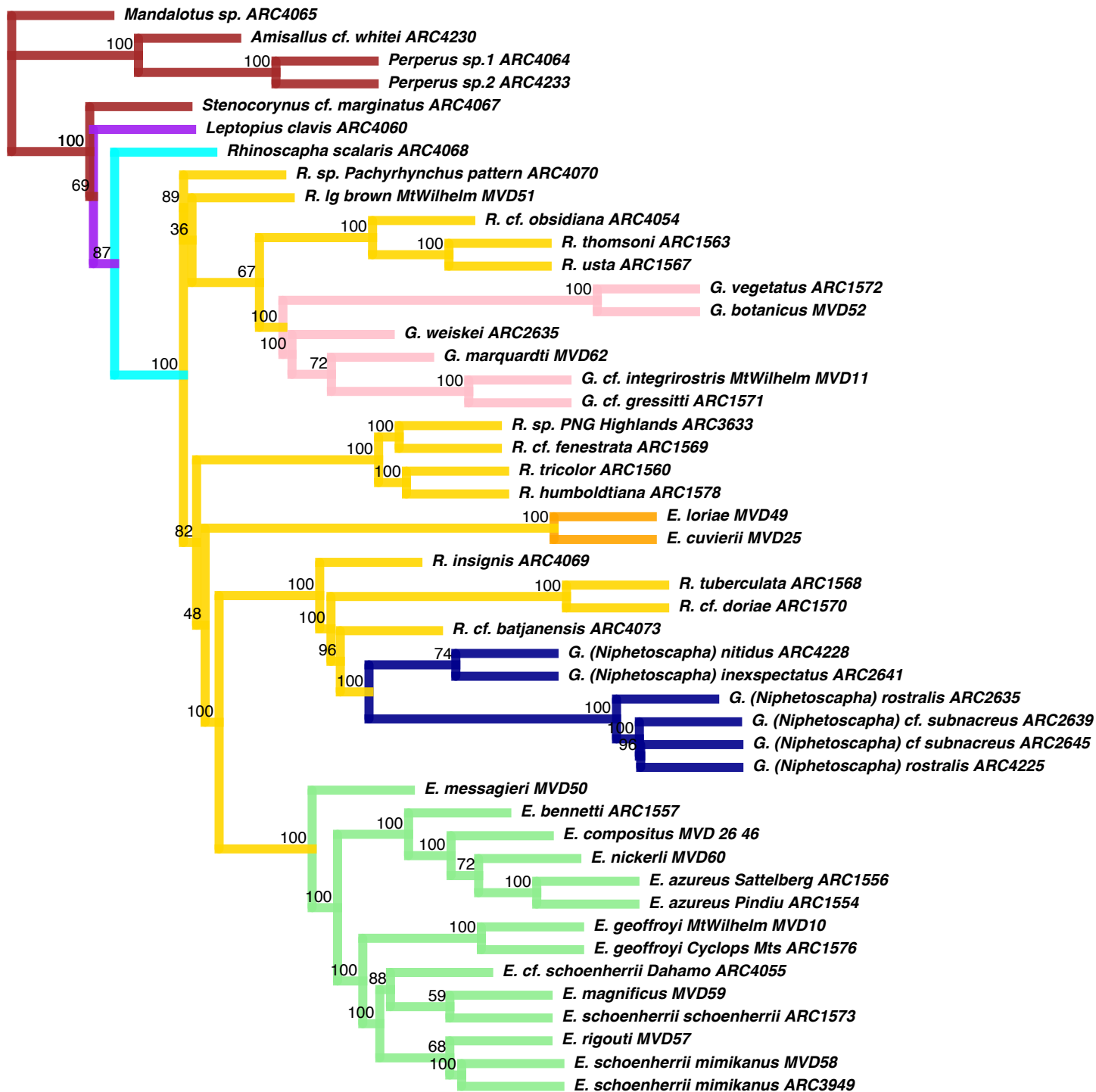

Supplement: S1 Fig — (PDF) [file pone.0188044.s004.pdf]

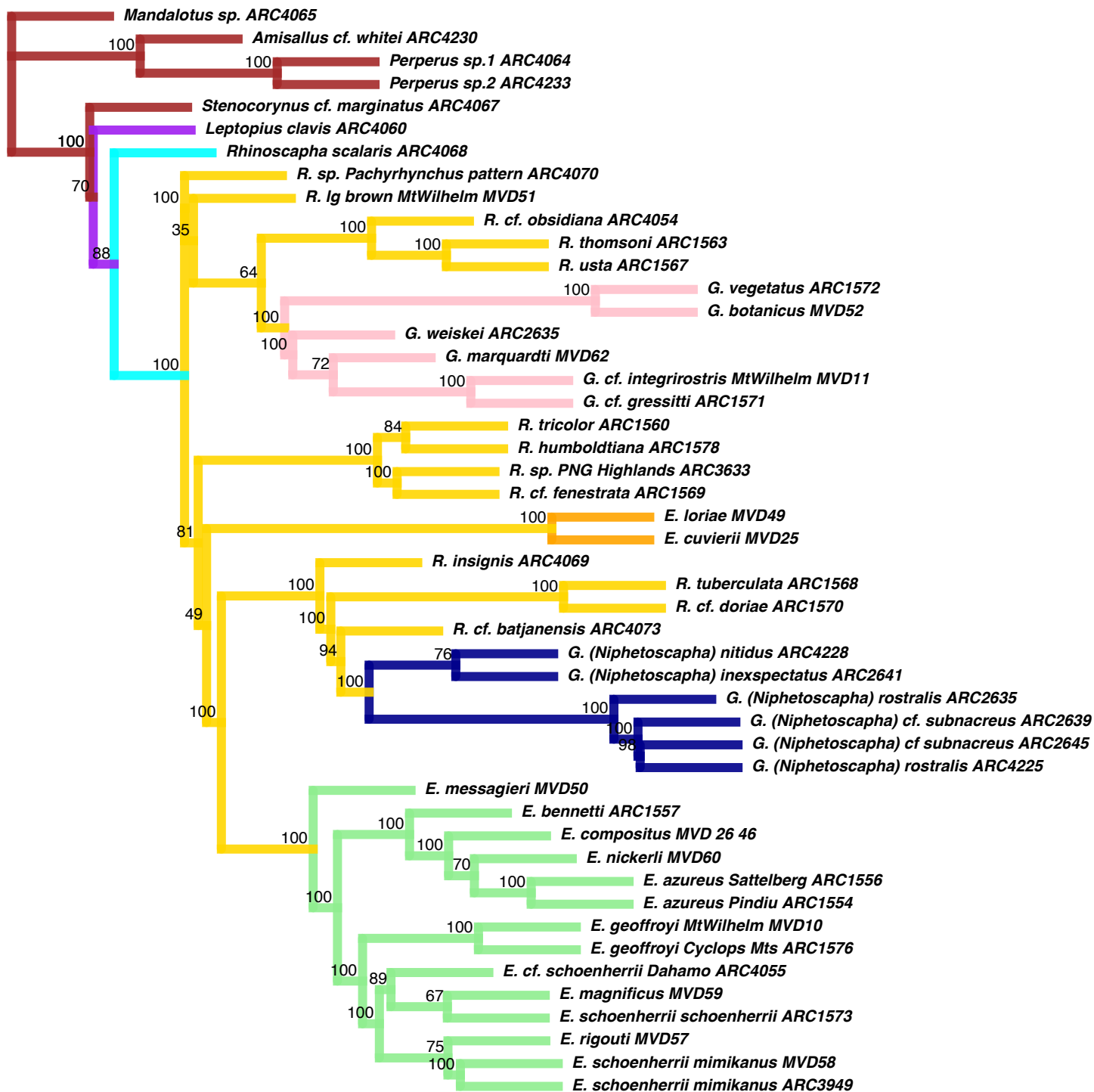

Supplement: S2 Fig — (PDF) [file pone.0188044.s005.pdf]

**Locus Length vs. Number of Partitions for MrBayes**

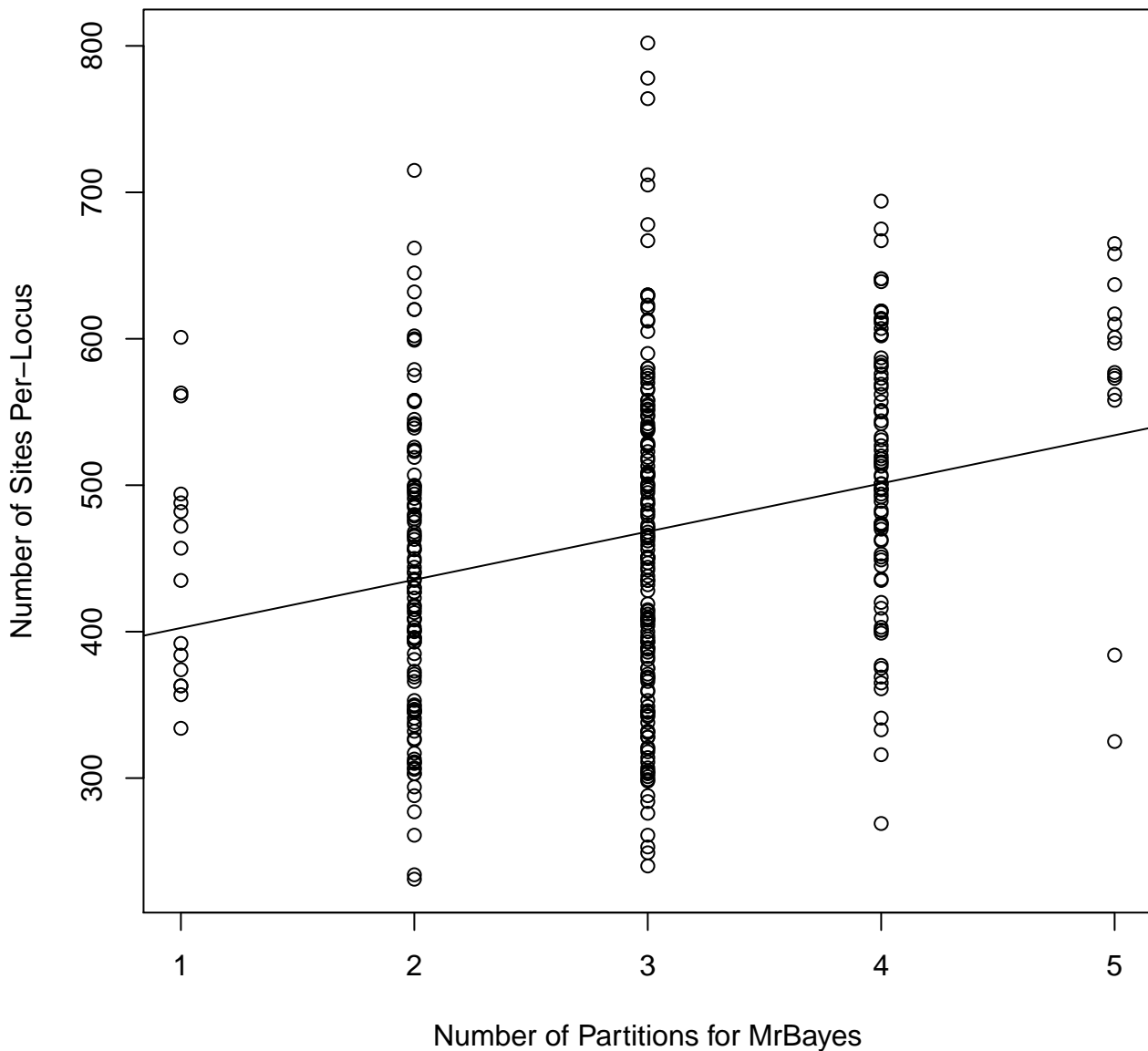

Supplement: S1 File — (ZIP) [file pone.0188044.s007.zip › Supplemental_Partition_Number_of_partitions_PIS_Charsets/Number of Sites vs. Number of Partitions-mrbayes.pdf]

# Locus Length vs. Number of Partitions for RAxML

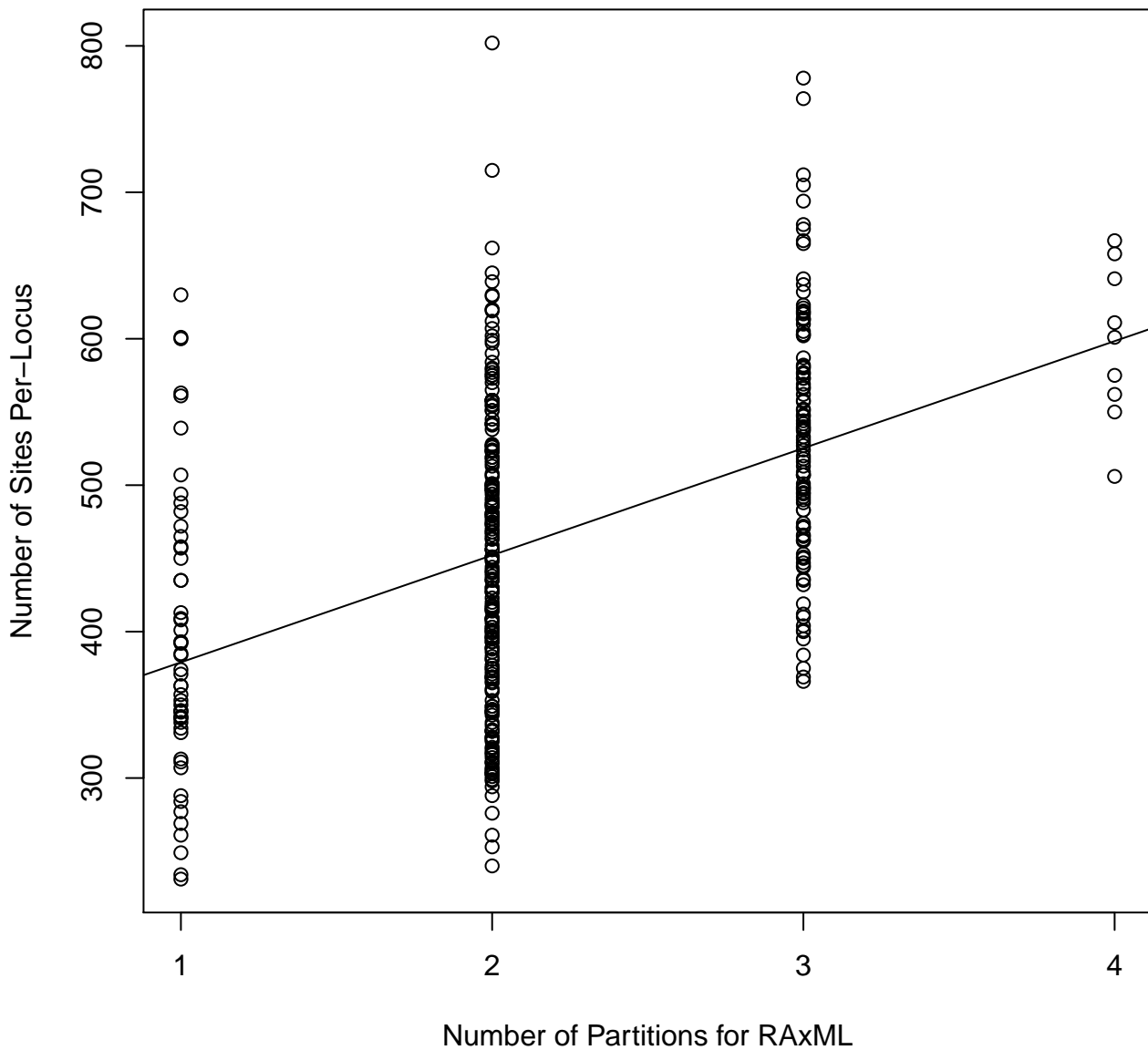

Supplement: S1 File — (ZIP) [file pone.0188044.s007.zip › Supplemental_Partition_Number_of_partitions_PIS_Charsets/Number of Sites vs. Number of Partitions-raxml.pdf]
