# Supplementary material for: Ultraconserved elements (UCEs) resolve the phylogeny of Australasian smurf-weevils
Source: PLoS One. 2017 Nov 22;12(11):e0188044. doi: 10.1371/journal.pone.0188044 (PMC5699822; doi:10.1371/journal.pone.0188044)
Supplement: S1 Supporting Information Links — (DOCX) [file pone.0188044.s006.docx]

Links to UCE loci alignments: 10.6084/m9.figshare.5172478 (<https://figshare.com/articles/Ultraconserved_elements_UCEs_resolve_the_phylogeny_of_Australasian_smurf-weevils/5172478)>.

R/UNIX CODE at MHVD’s github page: <https://github.com/matthewhvandam/weevil-UCE/tree/master>.

Raw Sequence reads: NCBI BioProject ID: PRJNA394929
