# Supplementary material for: Ultraconserved elements (UCEs) resolve the phylogeny of Australasian smurf-weevils
Source: PLoS One. 2017 Nov 22;12(11):e0188044. doi: 10.1371/journal.pone.0188044 (PMC5699822; doi:10.1371/journal.pone.0188044)

uce–93
MrBayes

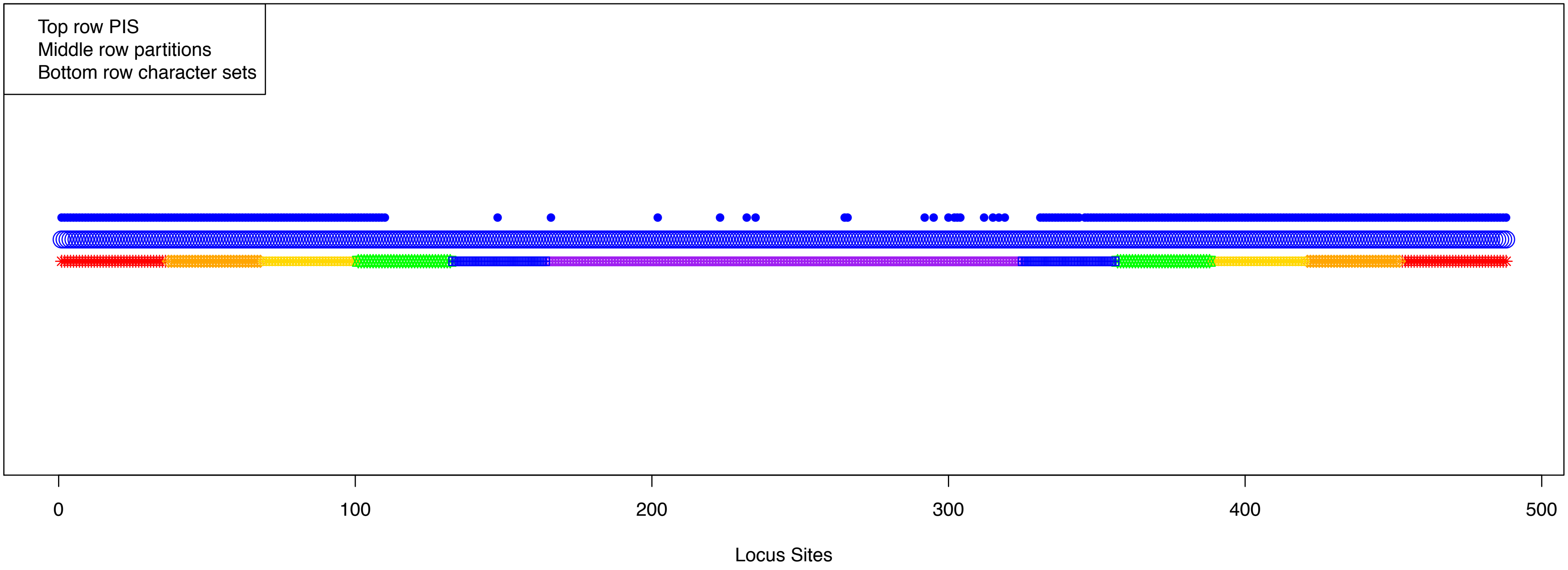

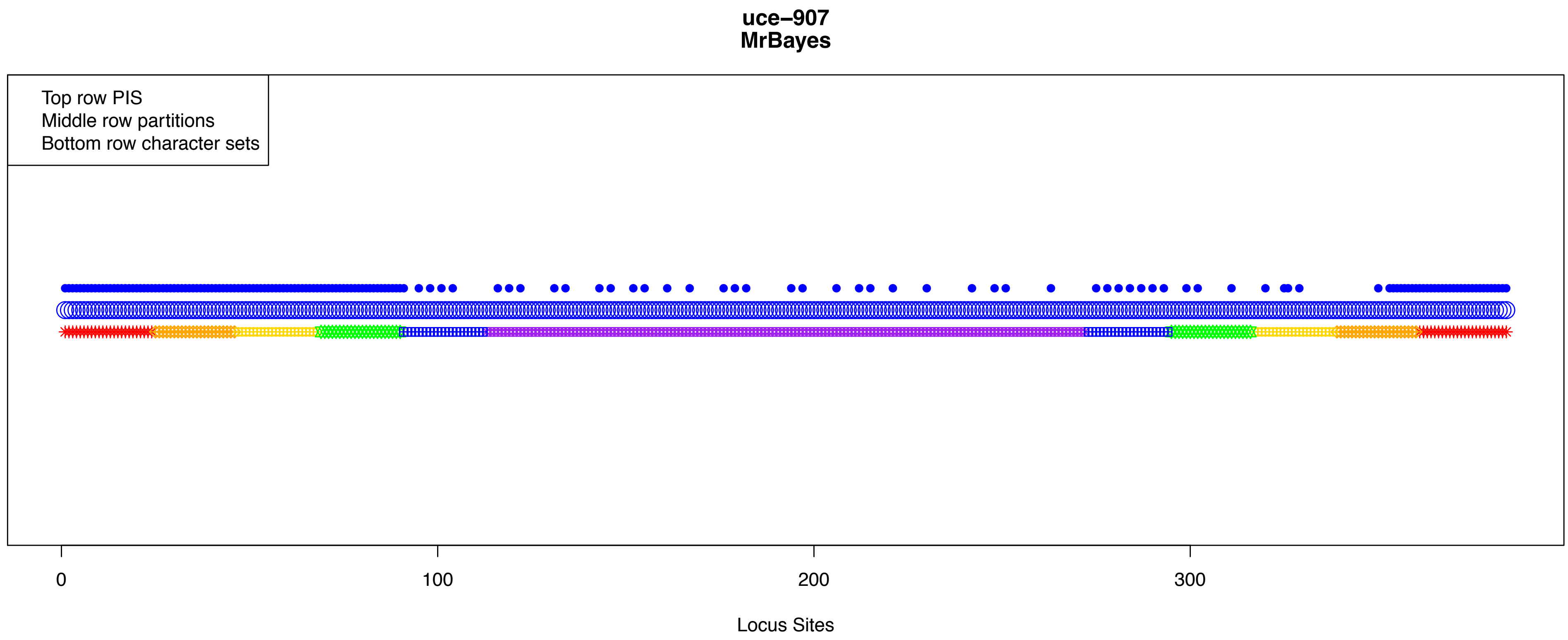
uce–907
MrBayes
Top row PIS
Middle row partitions
Bottom row character sets
0
100
200
300
Locus Sites

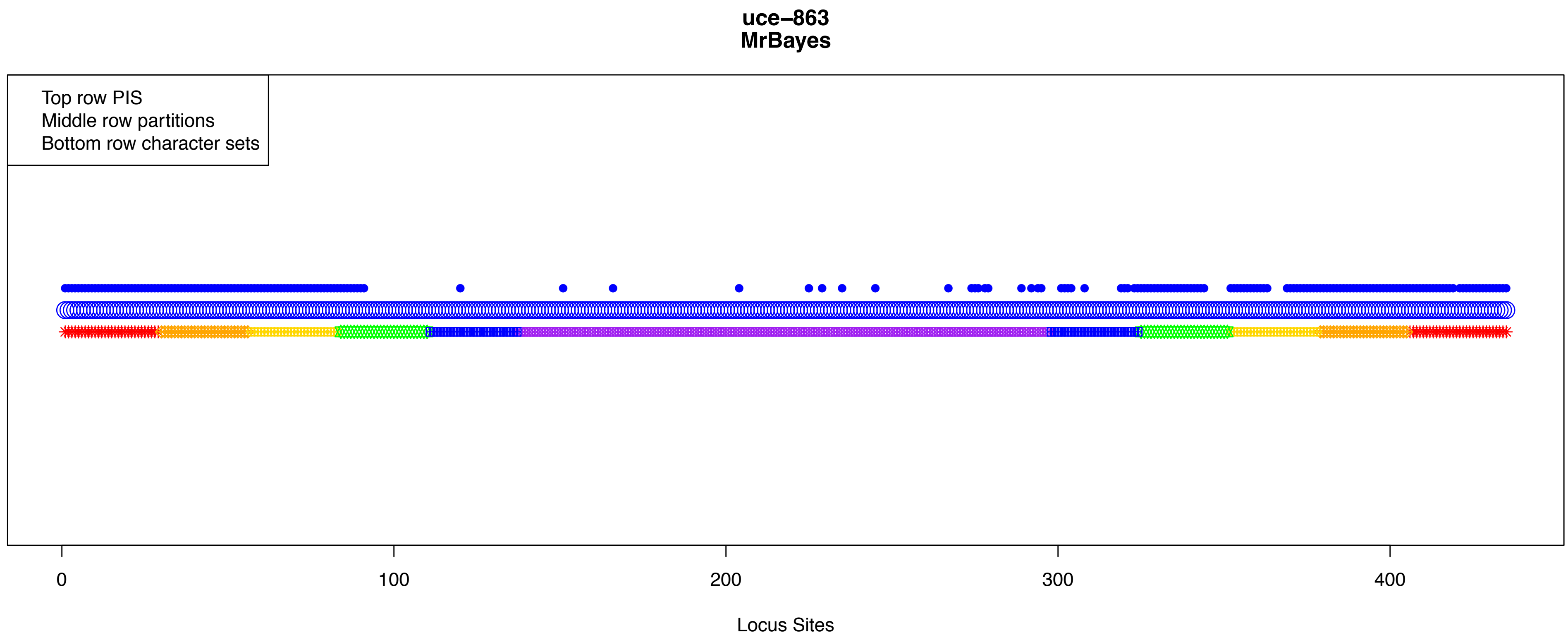
uce–863
MrBayes
Top row PIS
Middle row partitions
Bottom row character sets
0
100
200
300
400
Locus Sites

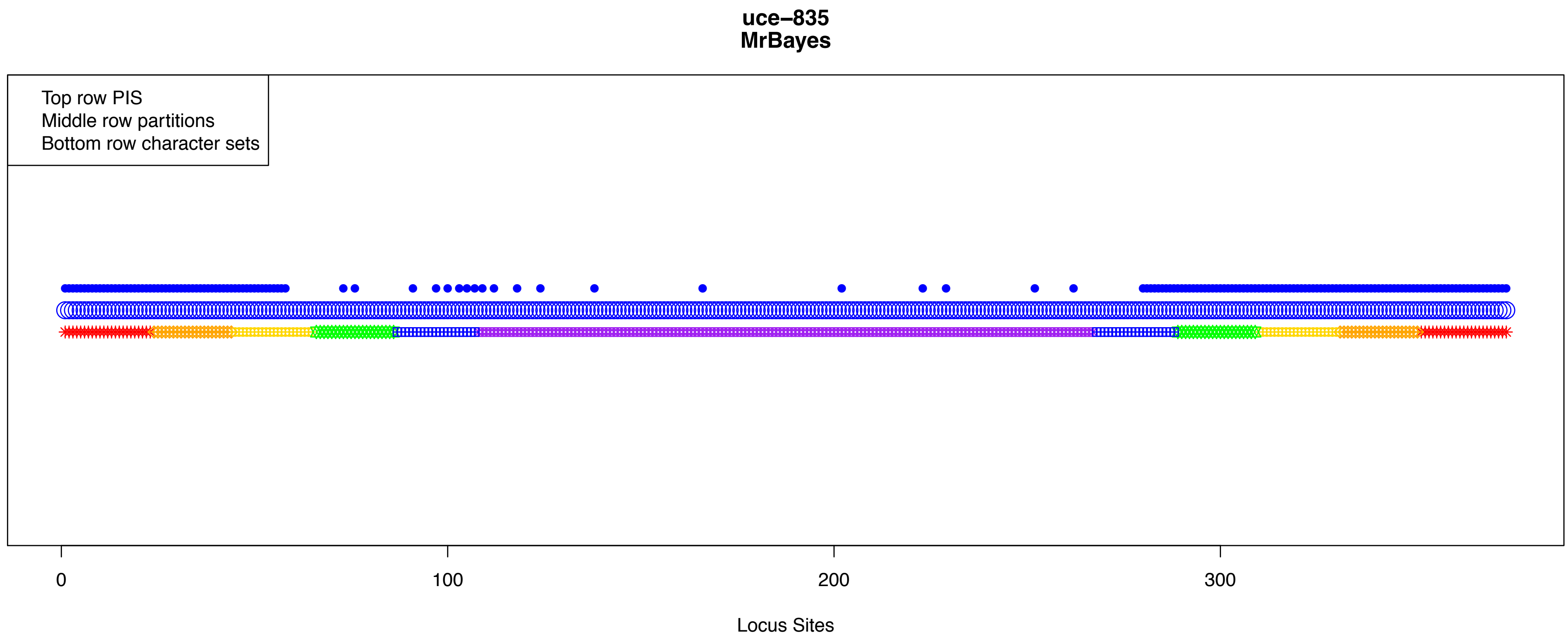
uce–835
MrBayes
Top row PIS
Middle row partitions
Bottom row character sets
0
100
200
300
Locus Sites

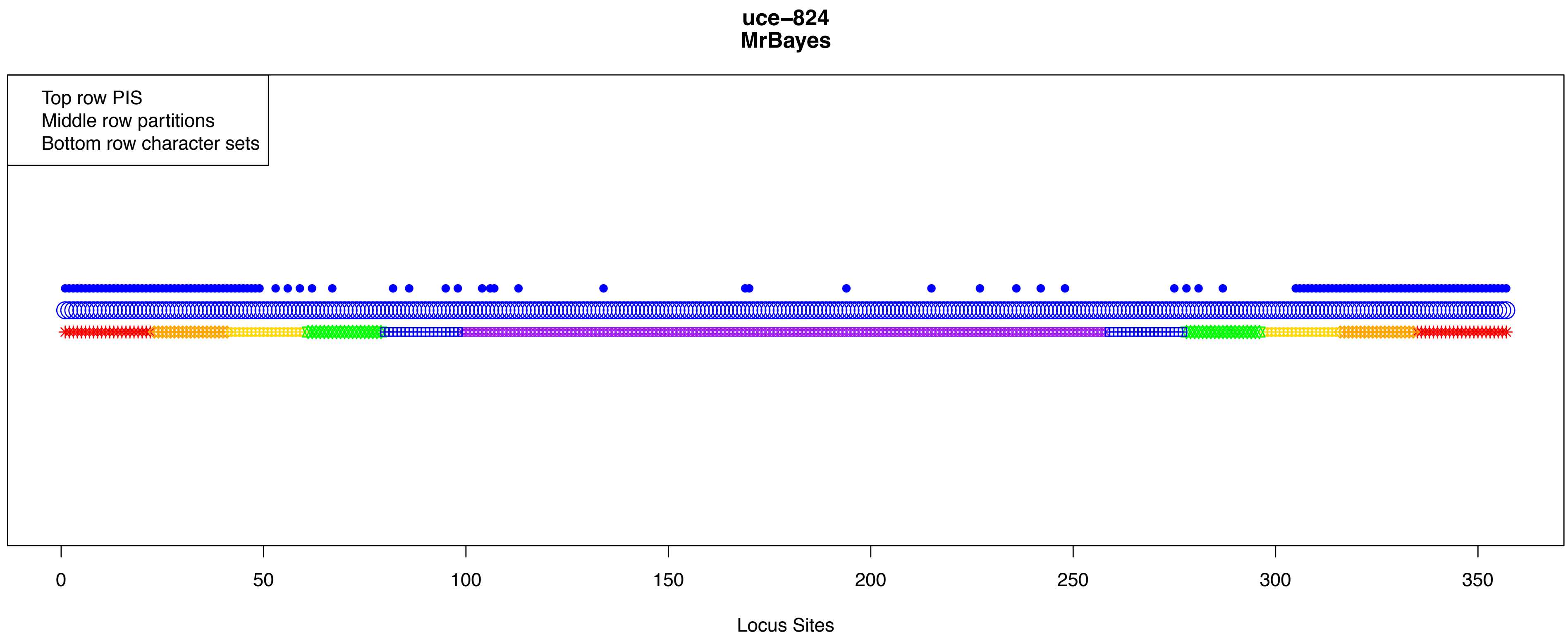

uce–824
MrBayes
Top row PIS
Middle row partitions
Bottom row character sets
0
50
100
150
200
250
300
350
Locus Sites

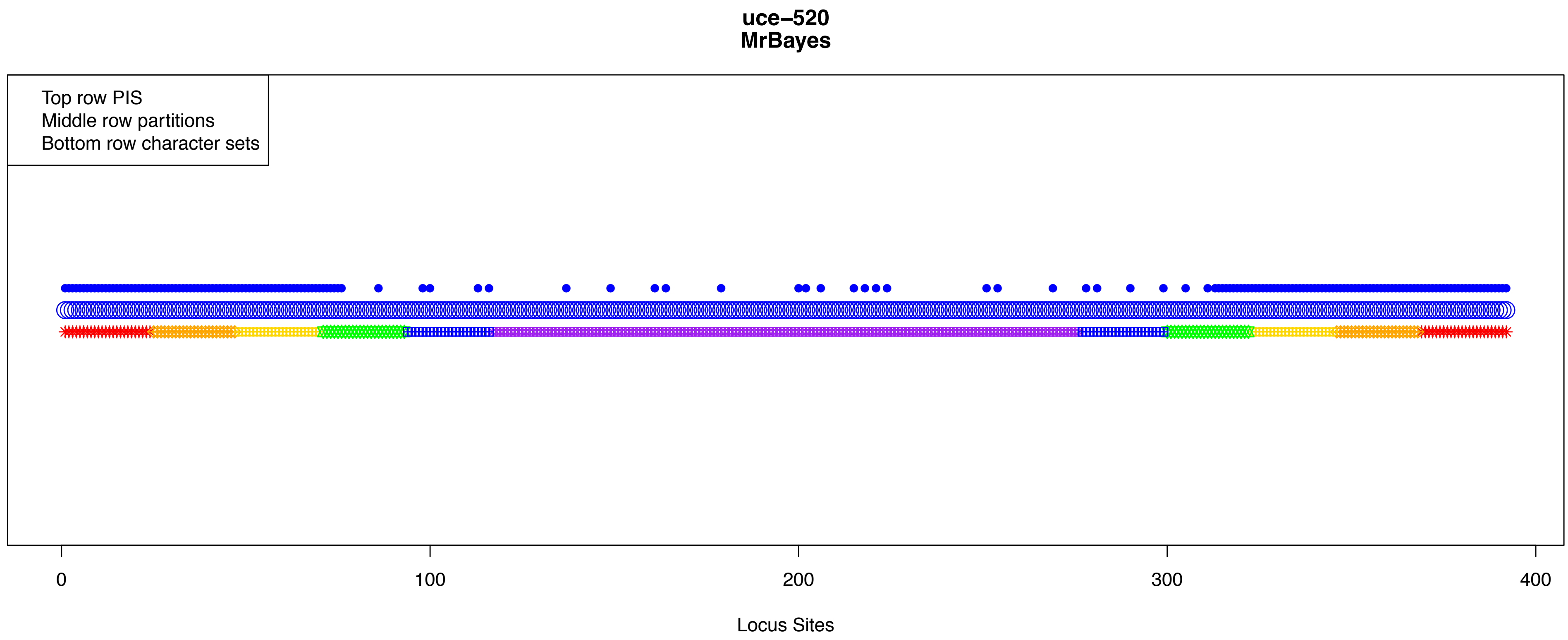

uce–520
MrBayes
Top row PIS
Middle row partitions
Bottom row character sets
0
100
200
300
400
Locus Sites

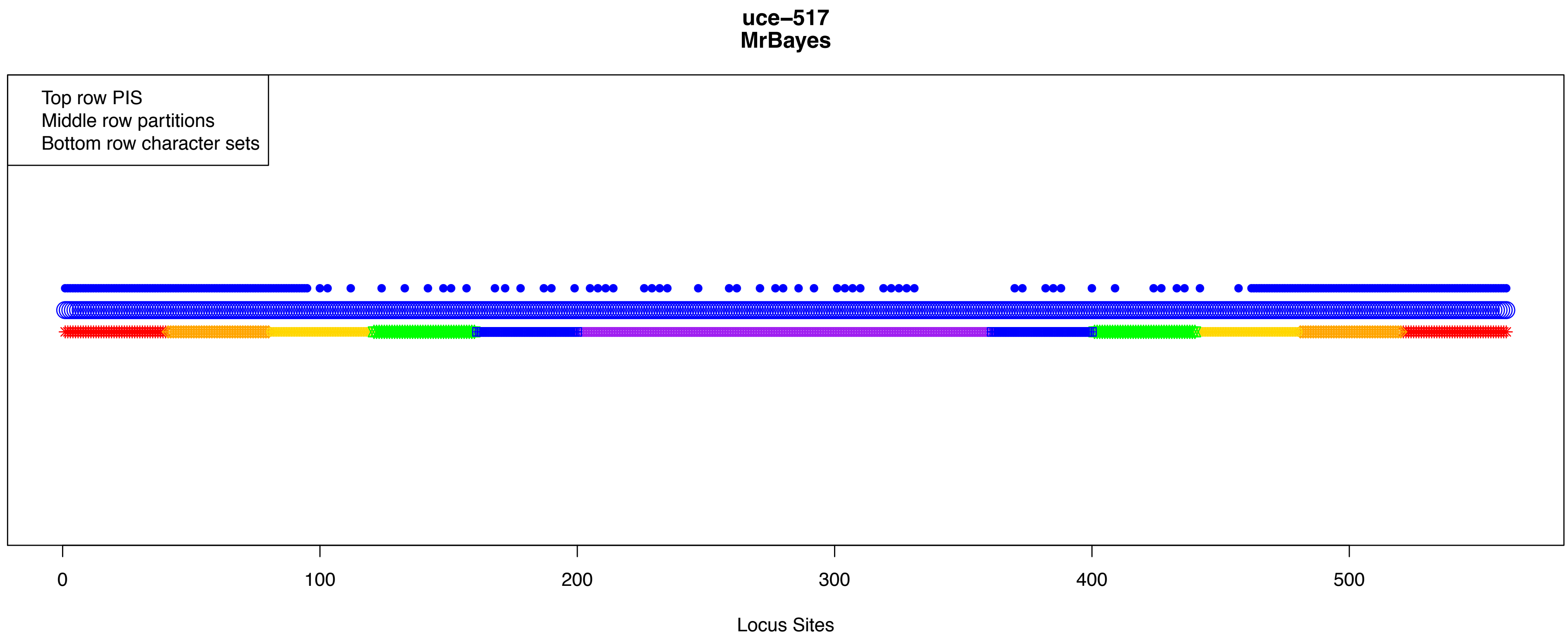
uce–517
MrBayes
Top row PIS
Middle row partitions
Bottom row character sets
0
100
200
300
400
500
Locus Sites

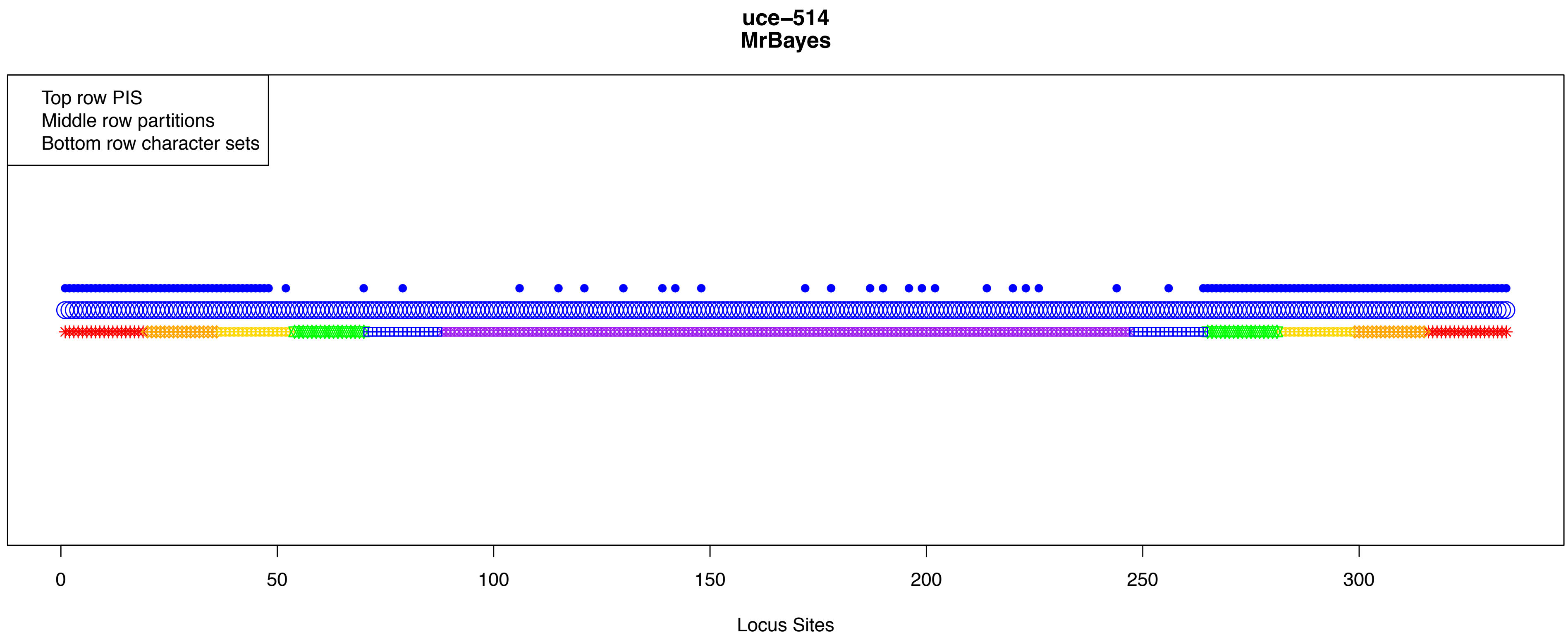
uce–514
MrBayes
Top row PIS
Middle row partitions
Bottom row character sets
0
50
100
150
200
250
300
Locus Sites

uce–507
MrBayes

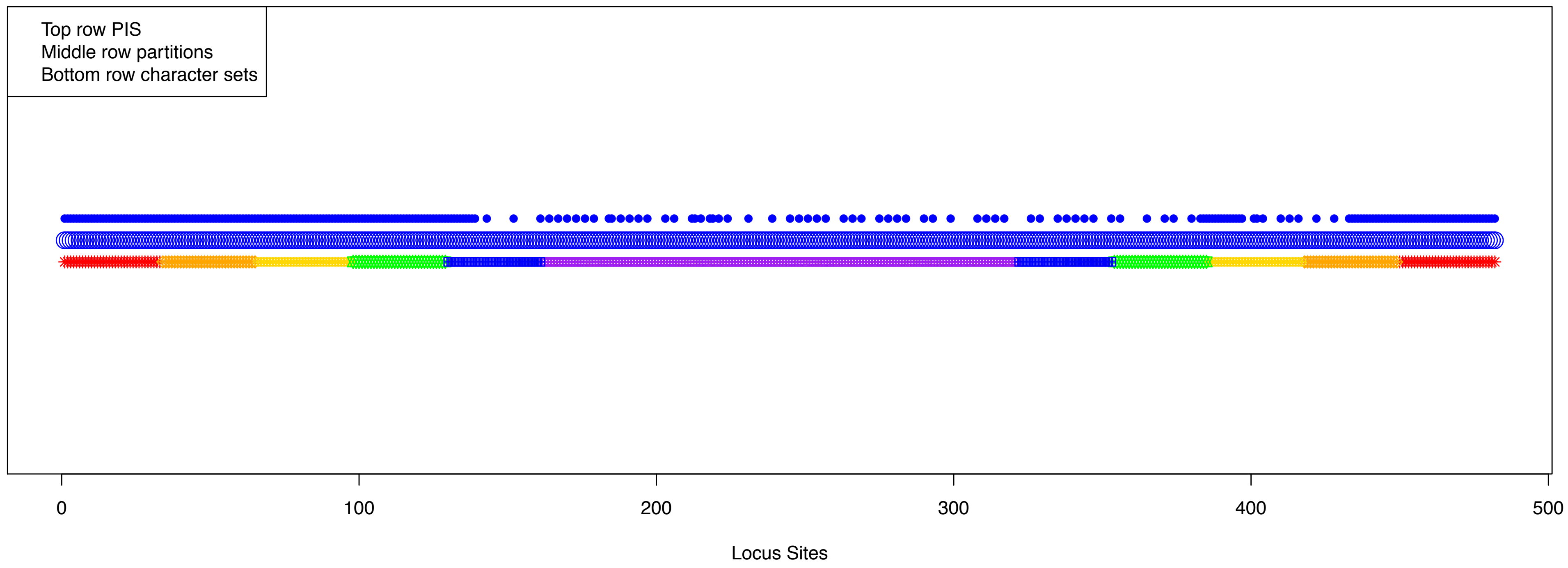

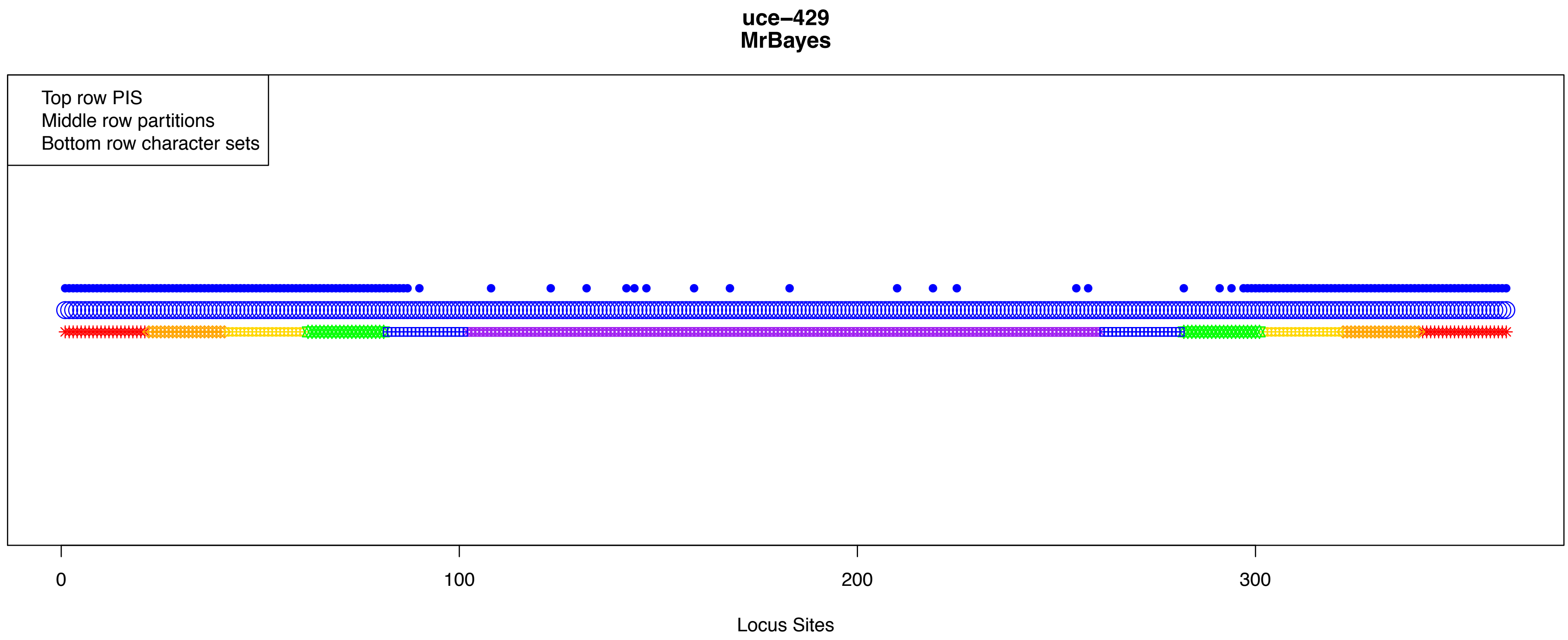
uce–429
MrBayes
Top row PIS
Middle row partitions
Bottom row character sets
0
100
200
300
Locus Sites

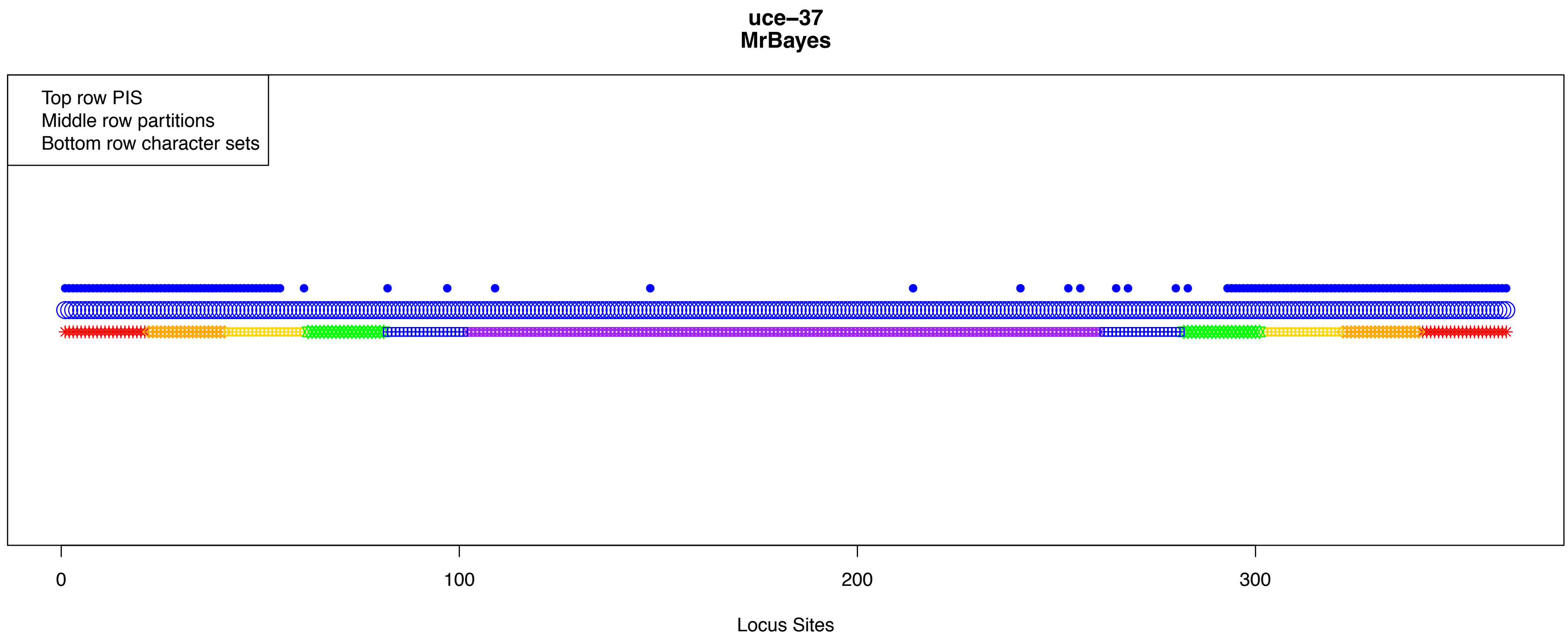
uce–37
MrBayes
Top row PIS
Middle row partitions
Bottom row character sets
0
100
200
300
Locus Sites

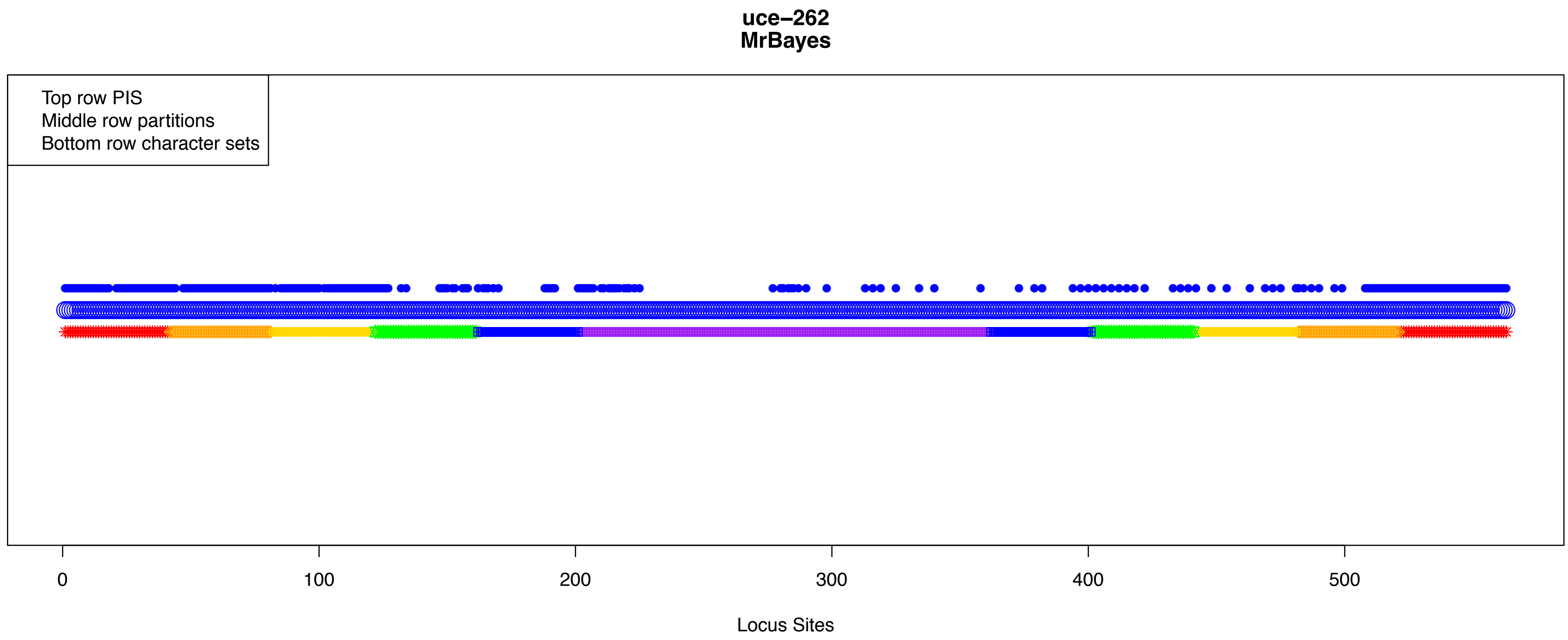
uce–262
MrBayes
Top row PIS
Middle row partitions
Bottom row character sets
0
100
200
300
400
500
Locus Sites

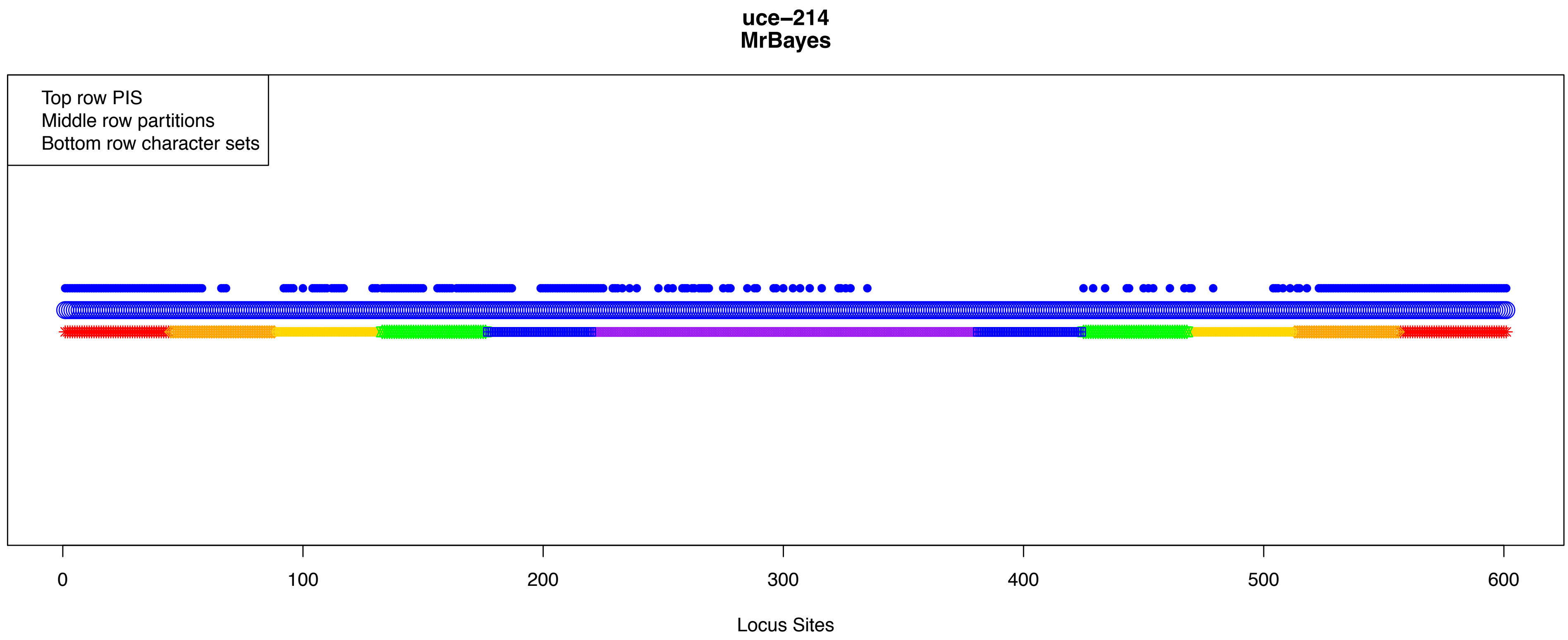
uce–214
MrBayes
Top row PIS
Middle row partitions
Bottom row character sets
0
100
200
300
400
500
600
Locus Sites

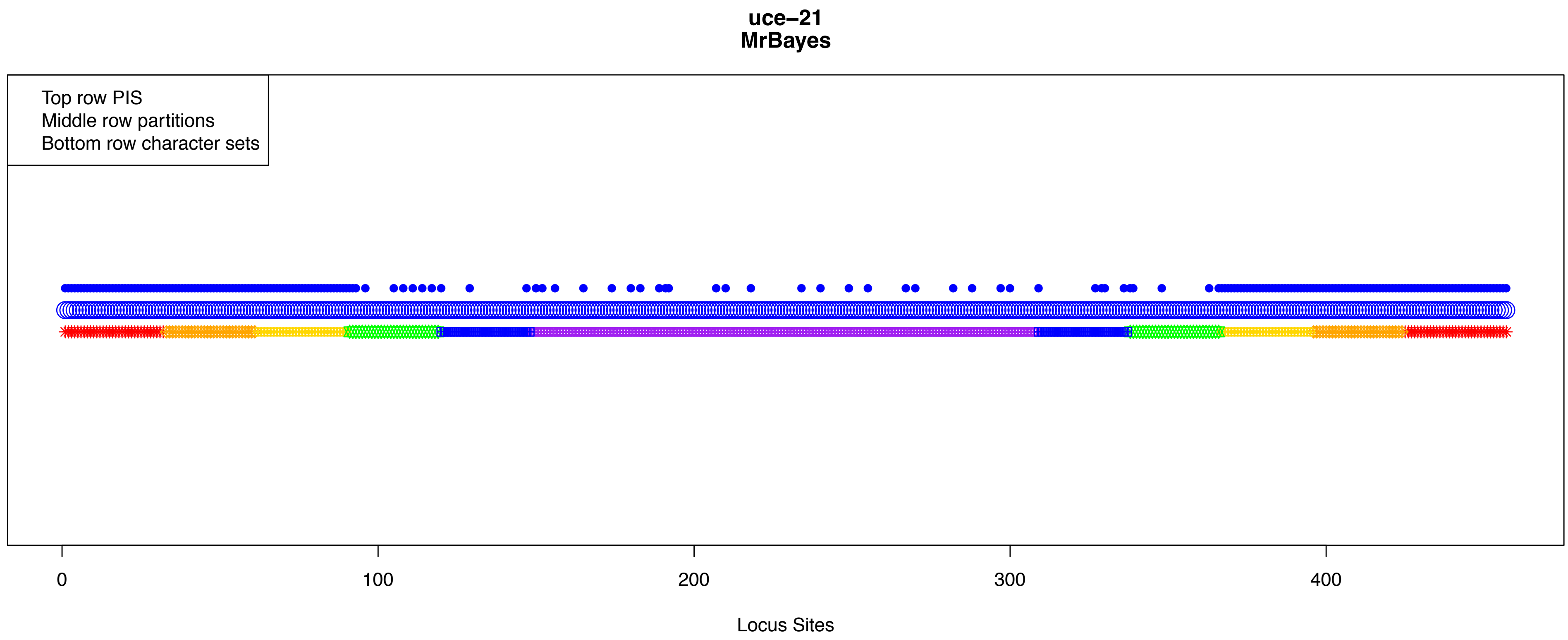
uce–21
MrBayes
Top row PIS
Middle row partitions
Bottom row character sets
0
100
200
300
400
Locus Sites

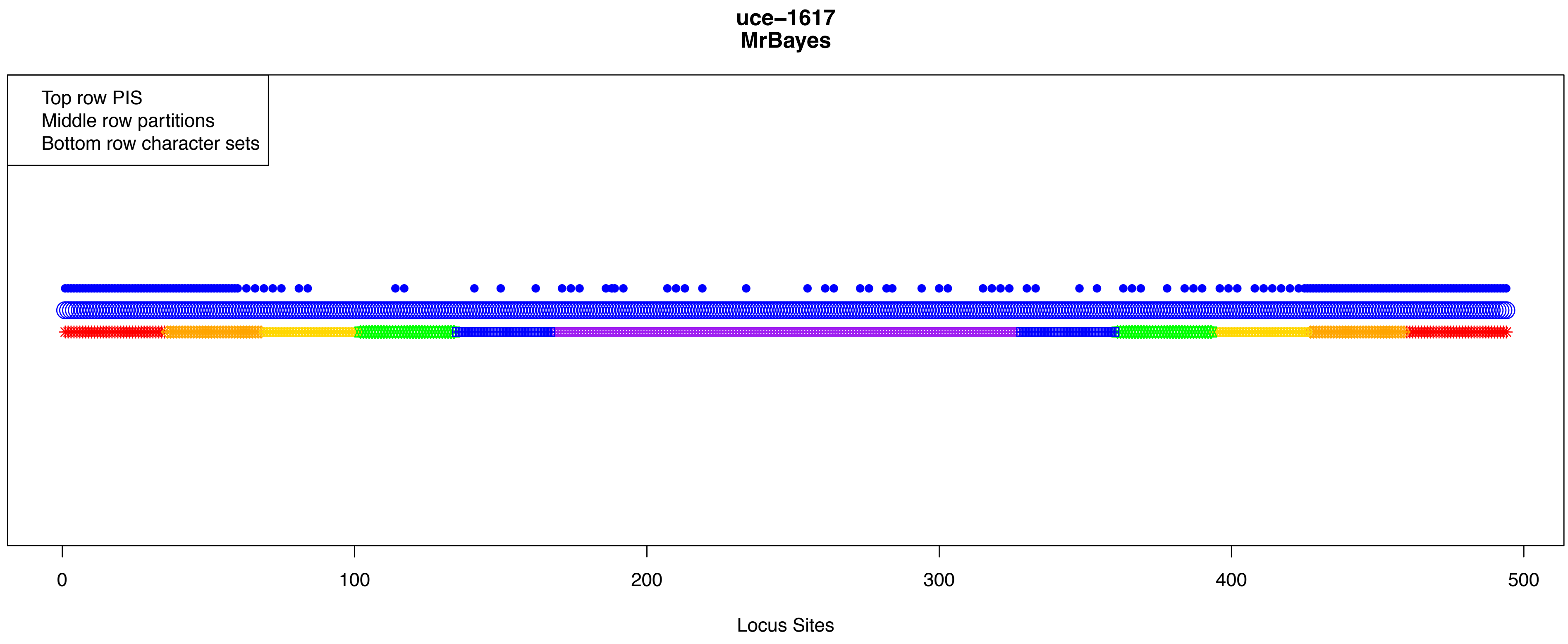
uce–1617
MrBayes
Top row PIS
Middle row partitions
Bottom row character sets
0
100
200
300
400
500
Locus Sites

uce–1348
MrBayes

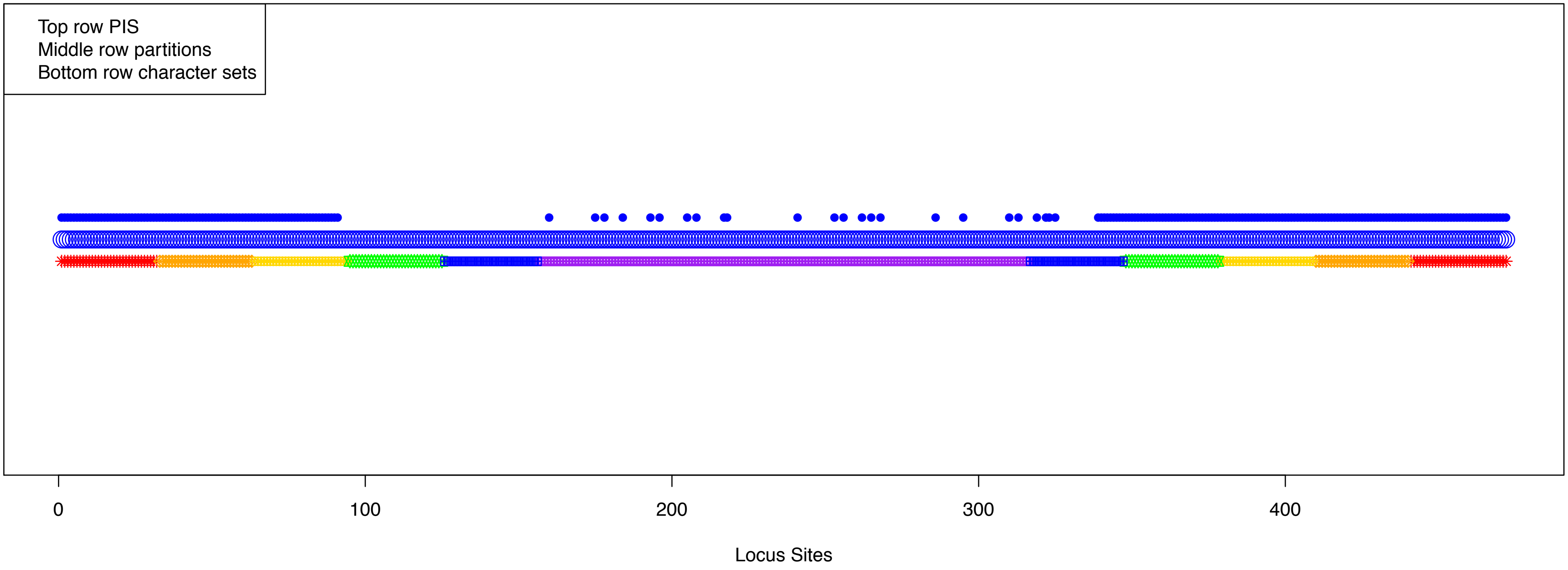

Supplement: S1 File — (ZIP) [file pone.0188044.s007.zip › Supplemental_Partition_Number_of_partitions_PIS_Charsets/partitions1-MrBayes.pdf]
