# Supplementary material for: Ultraconserved elements (UCEs) resolve the phylogeny of Australasian smurf-weevils
Source: PLoS One. 2017 Nov 22;12(11):e0188044. doi: 10.1371/journal.pone.0188044 (PMC5699822; doi:10.1371/journal.pone.0188044)
Supplement: S1 File — (ZIP) [file pone.0188044.s007.zip › Supplemental_Partition_Number_of_partitions_PIS_Charsets/partitions1-RAxML.pdf]

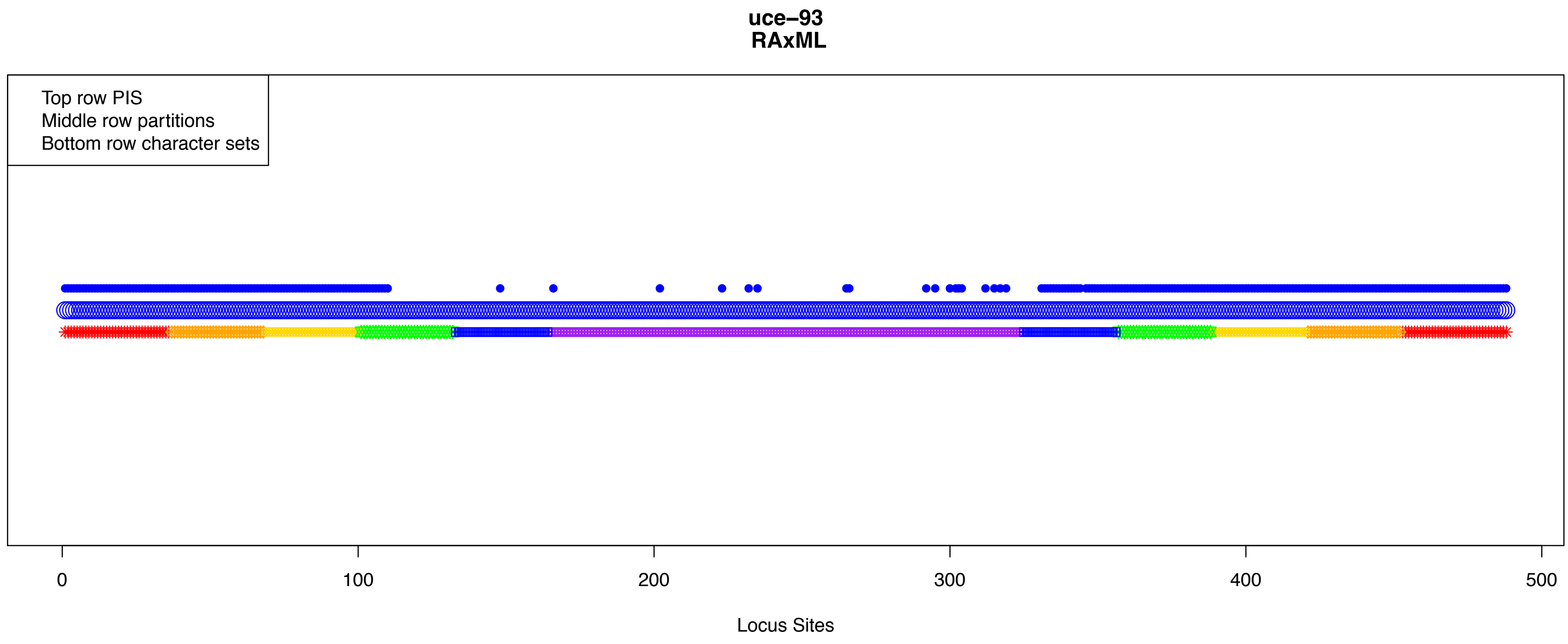

uce–93
RAxML
Top row PIS
Middle row partitions
Bottom row character sets
0
100
200
300
400
500
Locus Sites

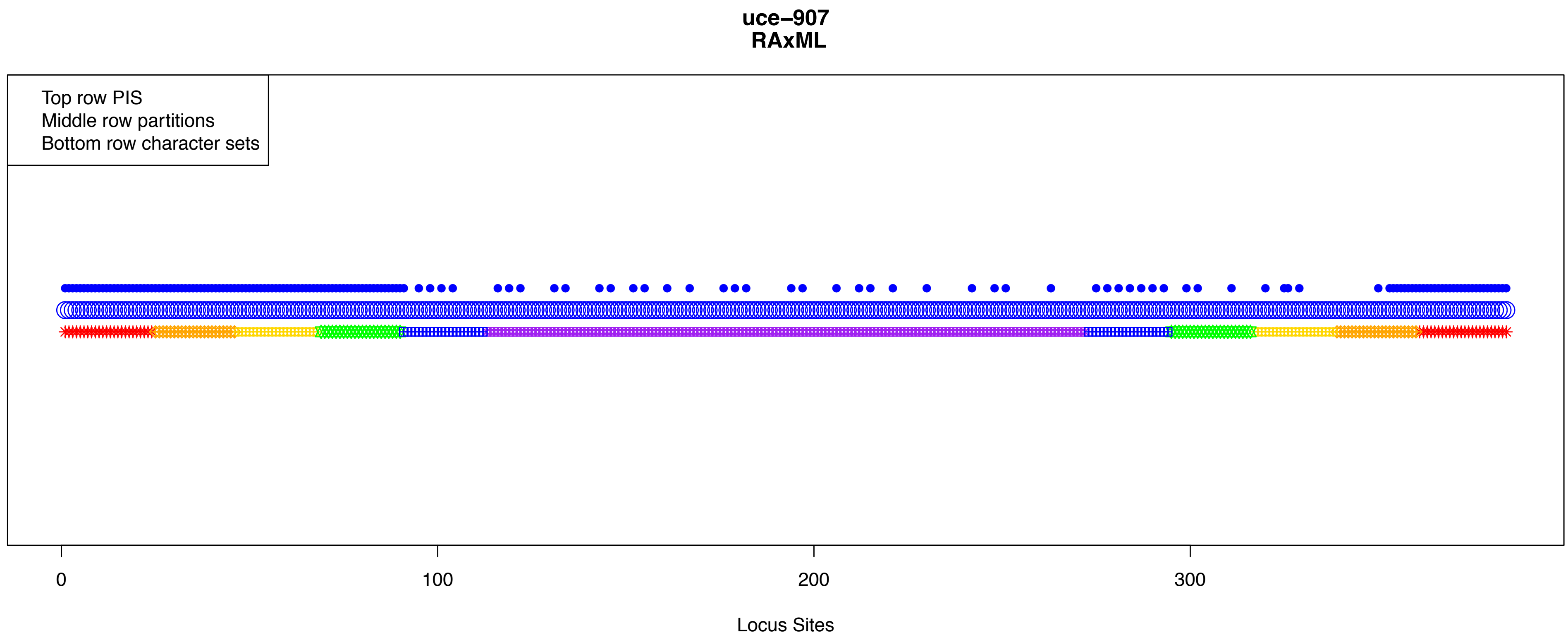
uce–907
RAxML
Top row PIS
Middle row partitions
Bottom row character sets
0
100
200
300
Locus Sites

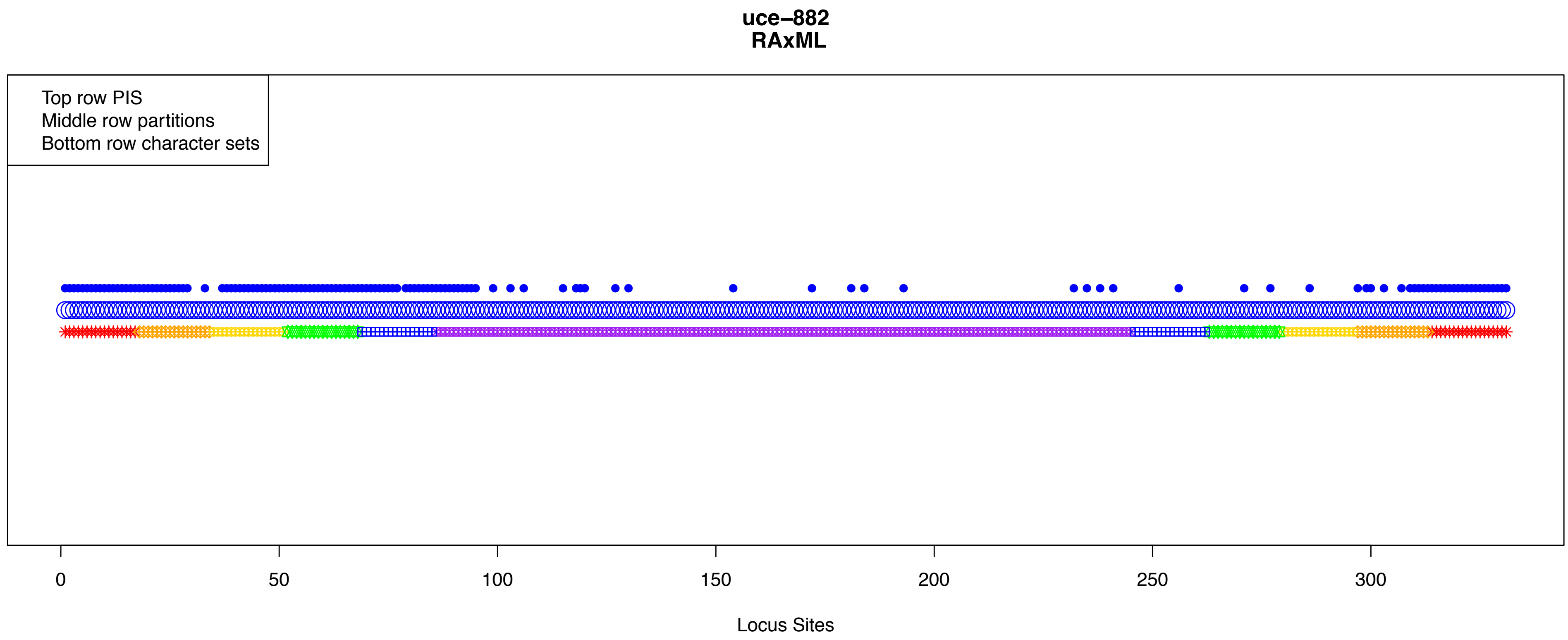

uce–882
RAxML
Top row PIS
Middle row partitions
Bottom row character sets
0
50
100
150
200
250
300
Locus Sites

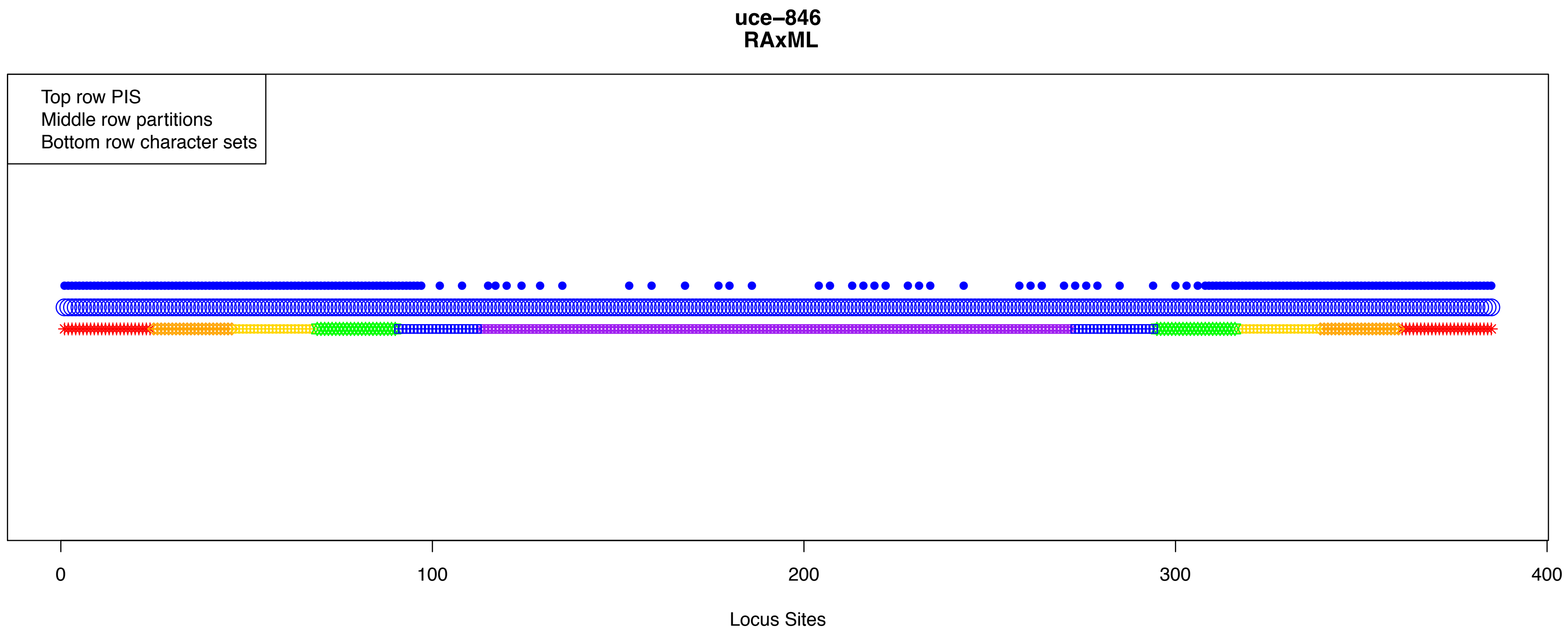
uce–846
RAxML
Top row PIS
Middle row partitions
Bottom row character sets
0
100
200
300
400
Locus Sites

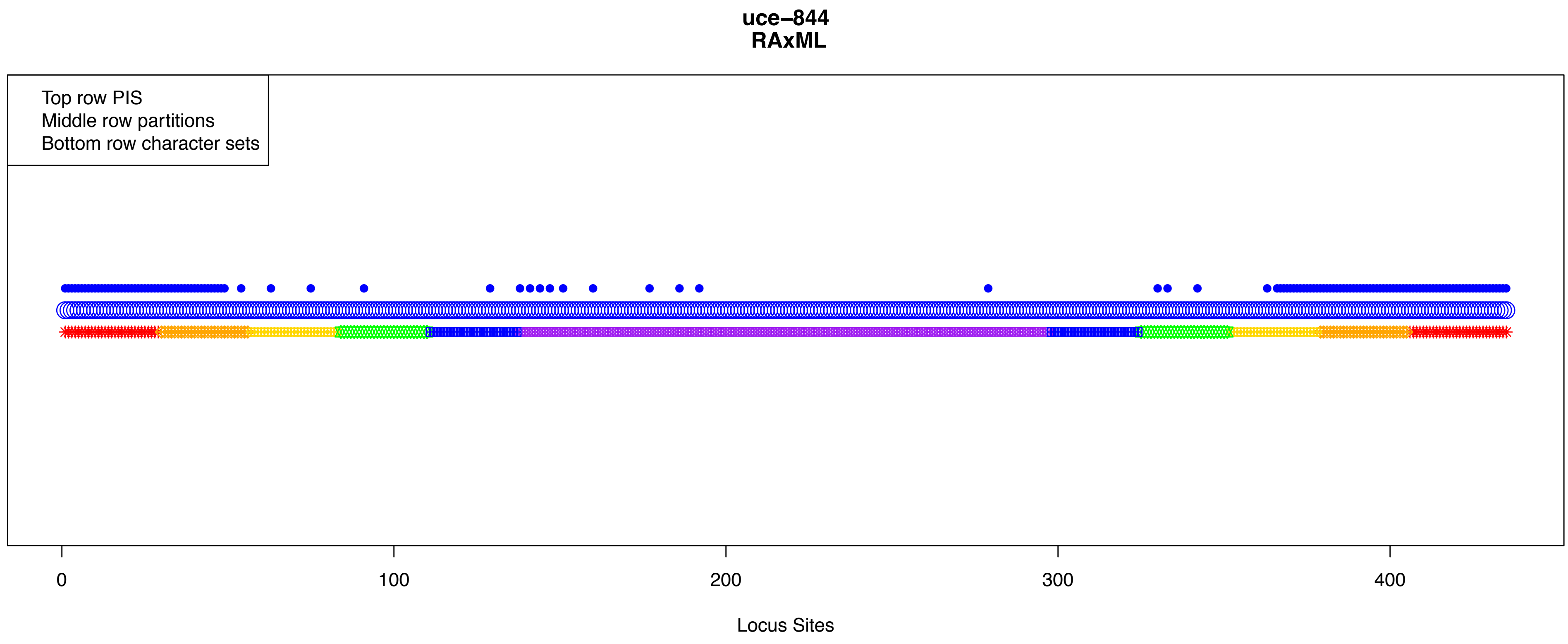

uce–844
RAxML
Top row PIS
Middle row partitions
Bottom row character sets
0
100
200
300
400
Locus Sites

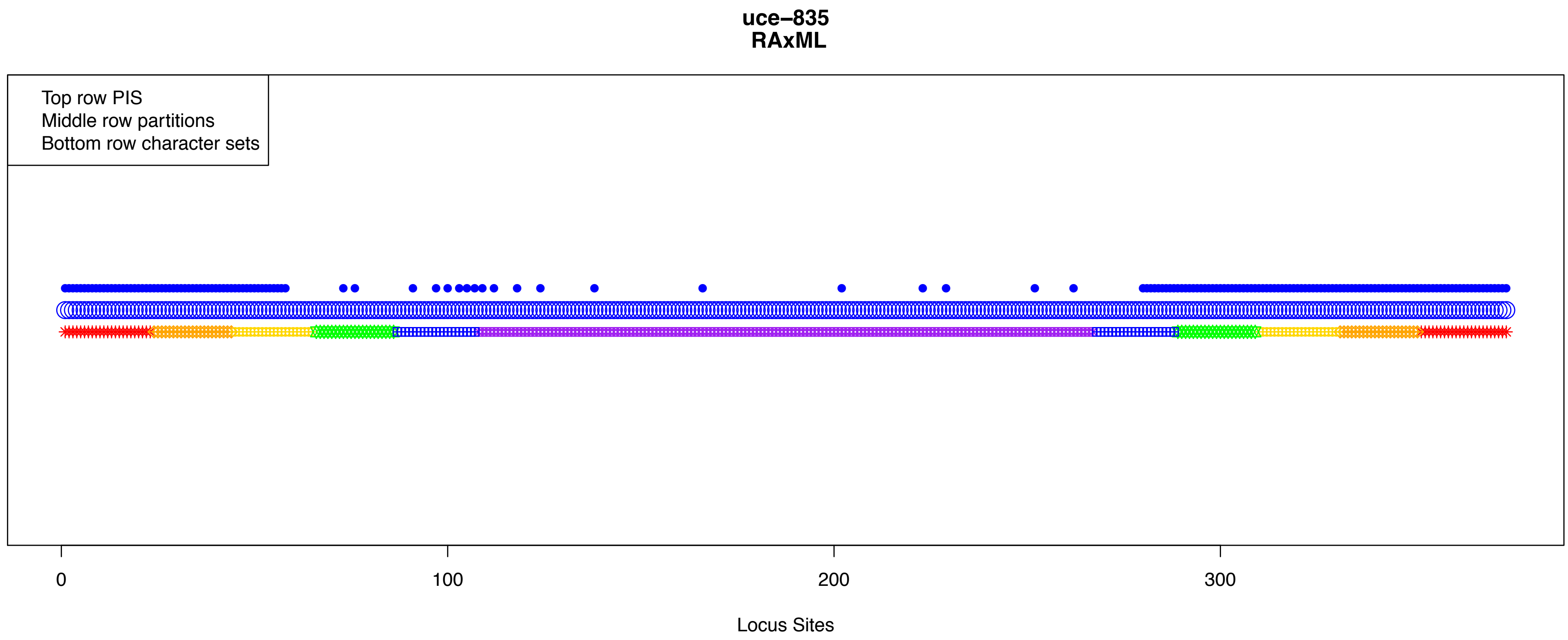
uce–835
RAxML
Top row PIS
Middle row partitions
Bottom row character sets
0
100
200
300
Locus Sites

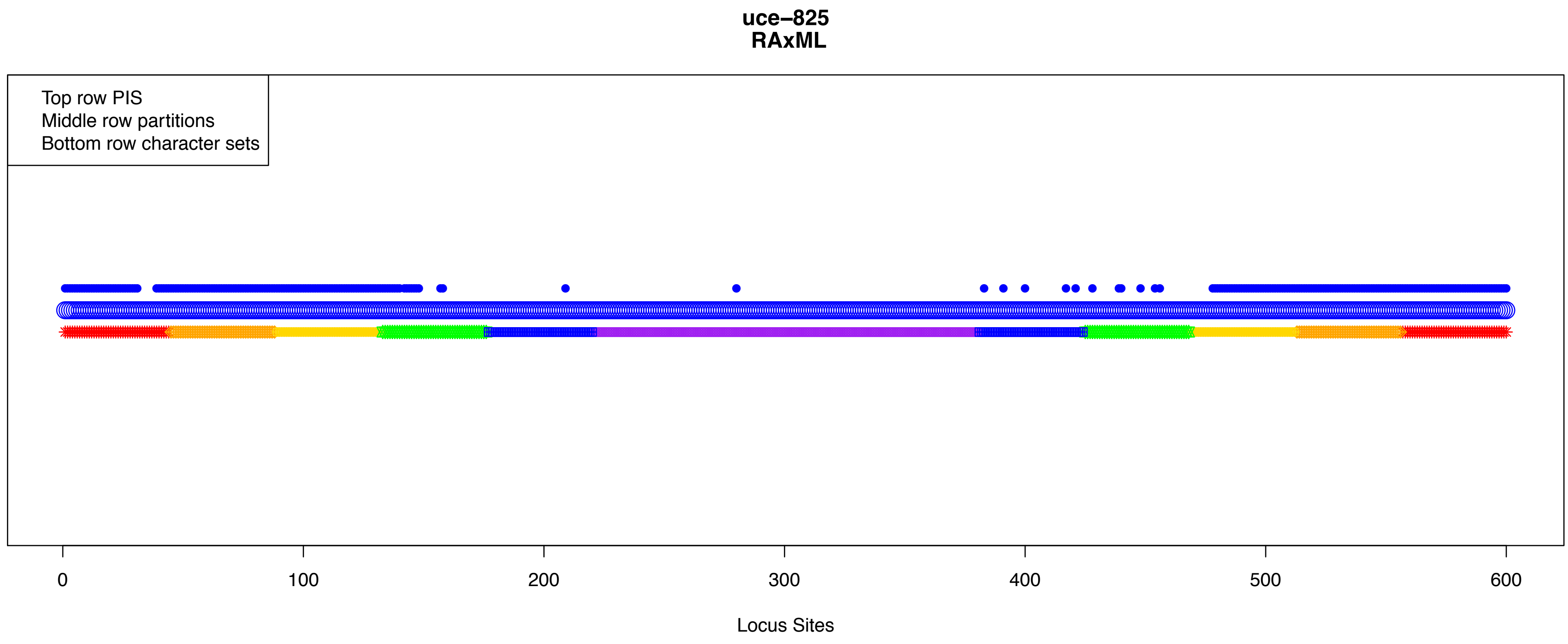

uce–825
RAxML
Top row PIS
Middle row partitions
Bottom row character sets
0
100
200
300
400
500
600
Locus Sites

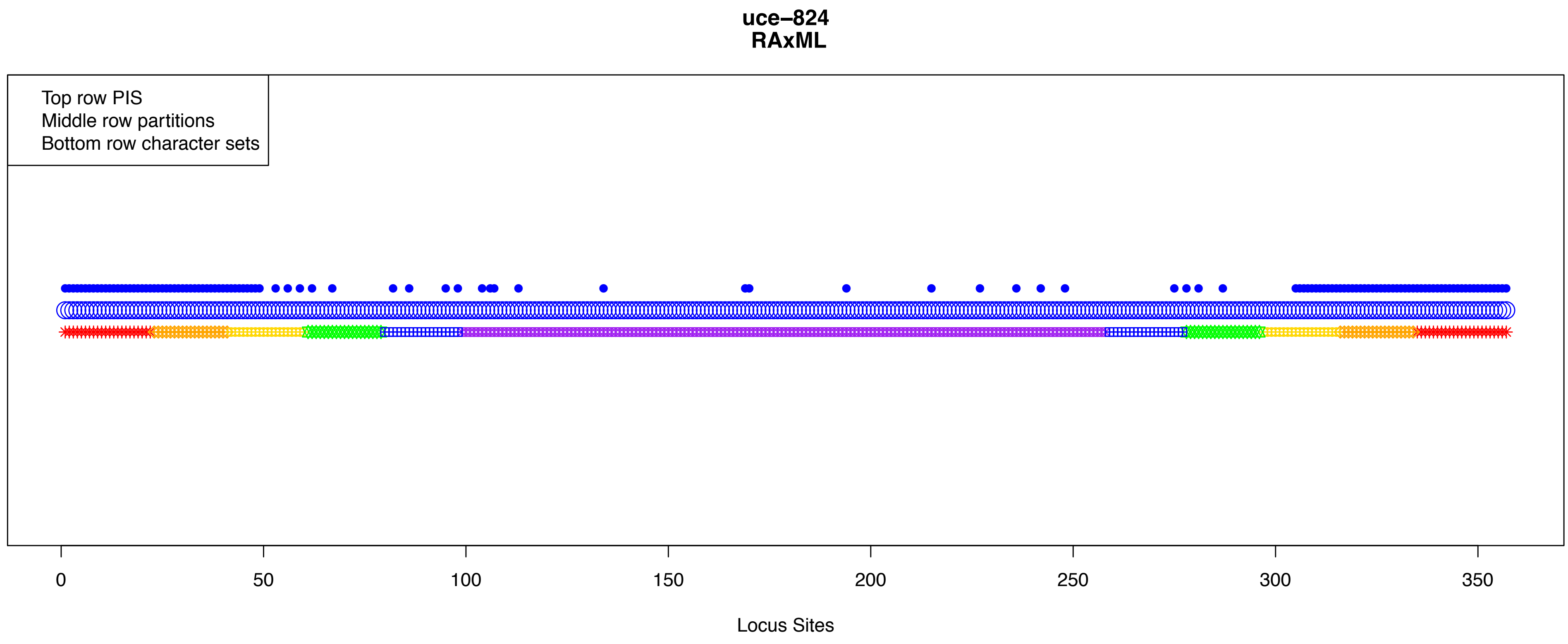
uce–824
RAxML
Top row PIS
Middle row partitions
Bottom row character sets
0
50
100
150
200
250
300
350
Locus Sites

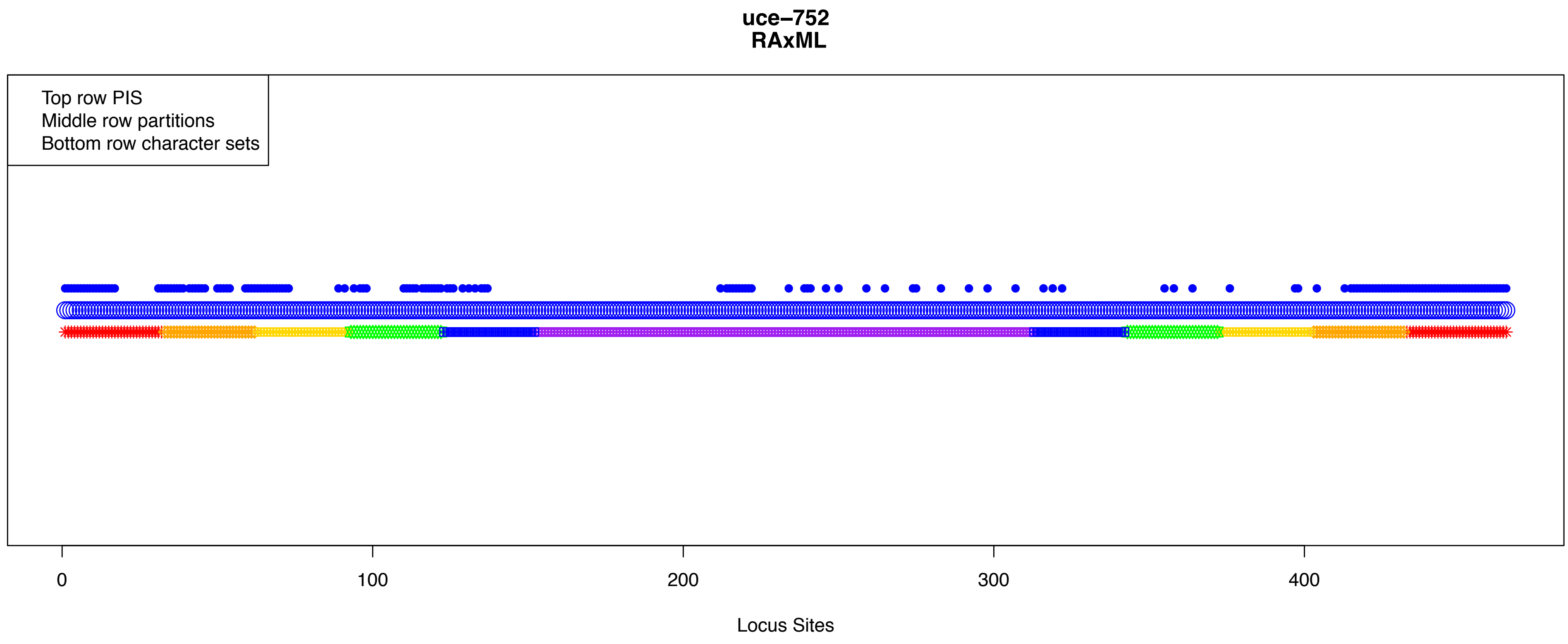
uce–752
RAxML
Top row PIS
Middle row partitions
Bottom row character sets
0
100
200
300
400
Locus Sites

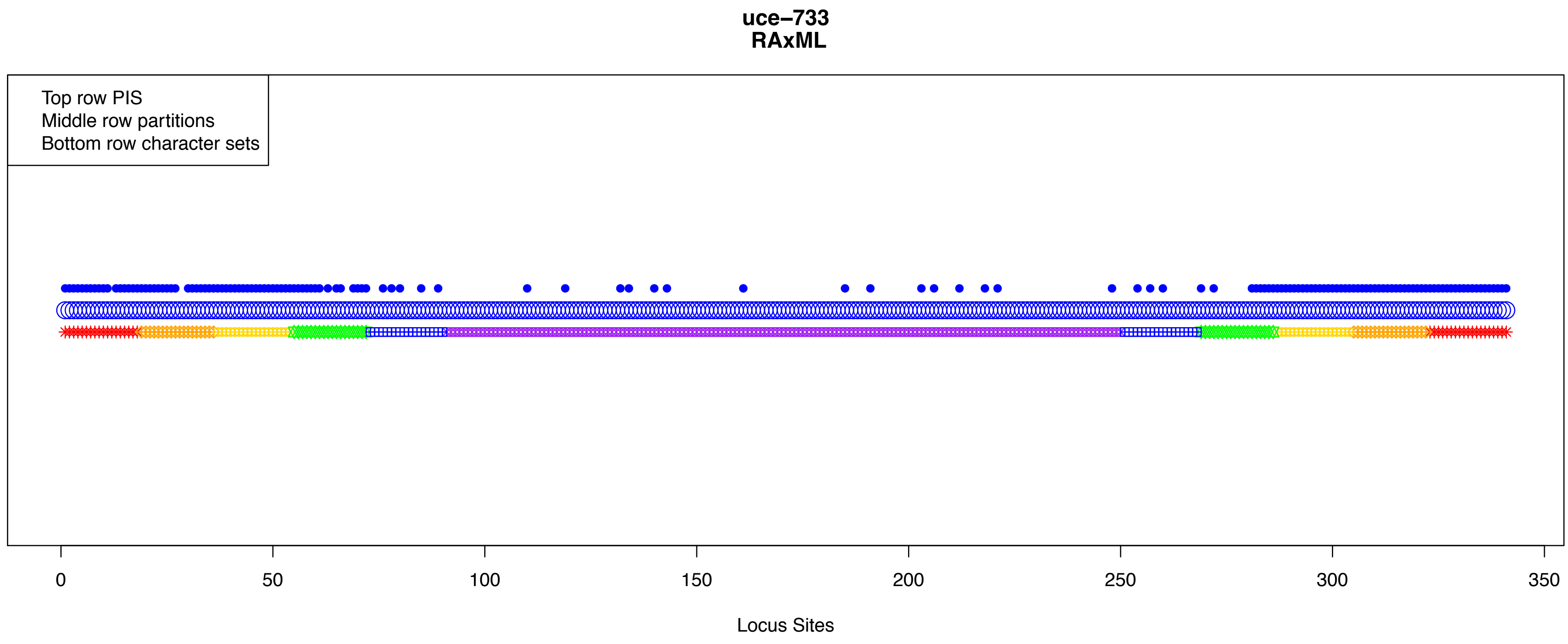
uce–733
RAxML
Top row PIS
Middle row partitions
Bottom row character sets
0
50
100
150
200
250
300
350
Locus Sites

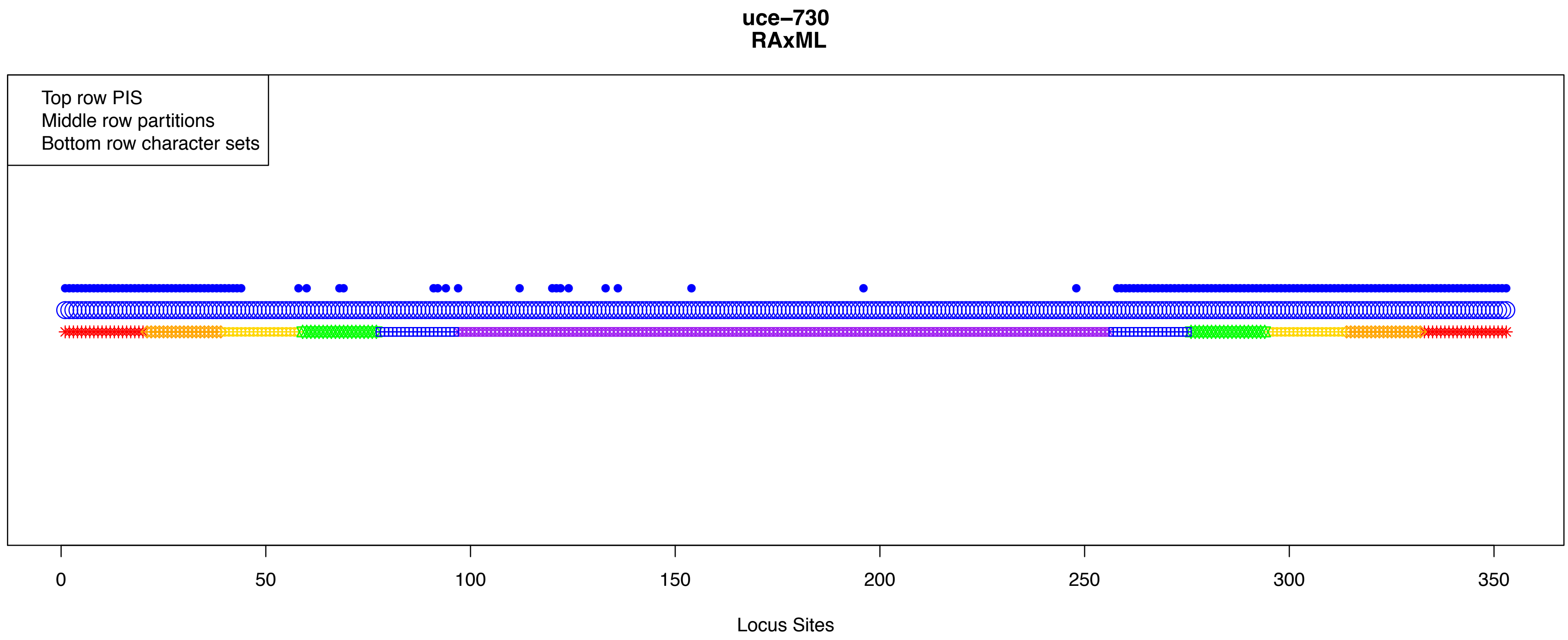

uce–730
RAxML
Top row PIS
Middle row partitions
Bottom row character sets
0
50
100
150
200
250
300
350
Locus Sites

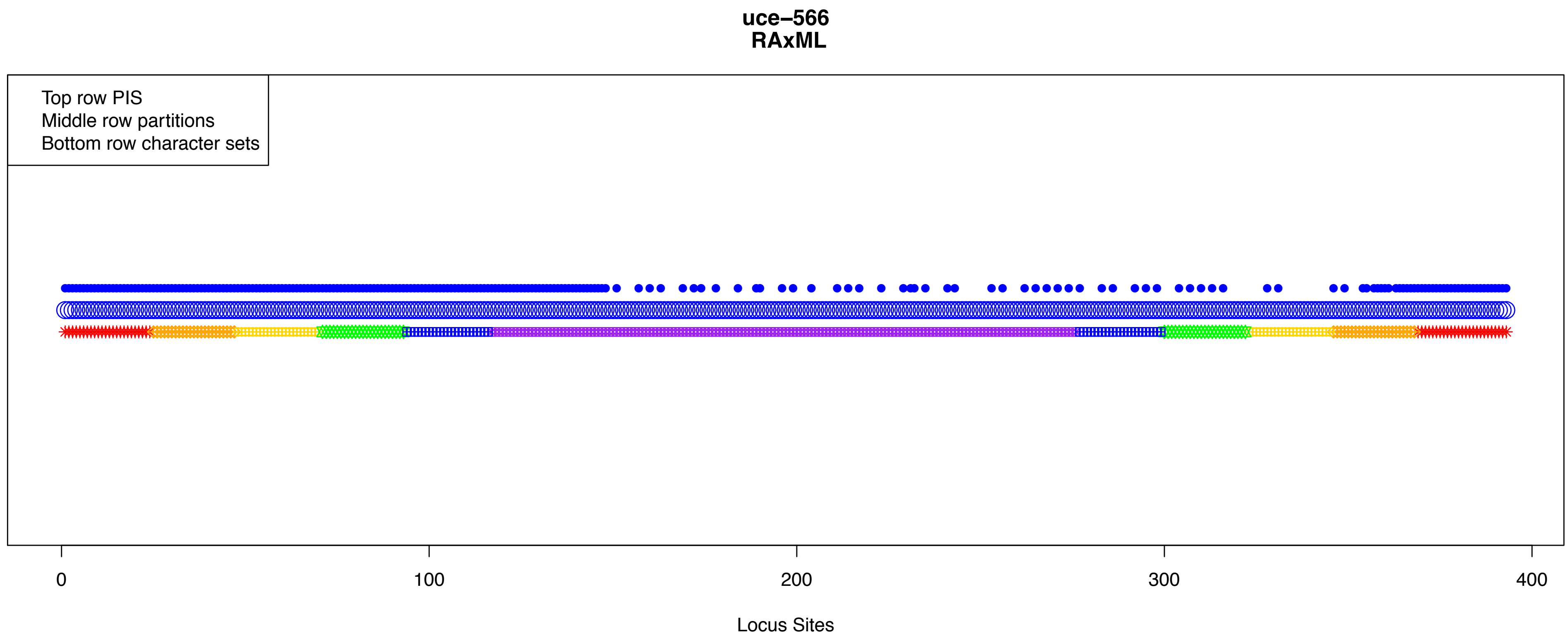
uce–566
RAxML
Top row PIS
Middle row partitions
Bottom row character sets
0
100
200
300
400
Locus Sites

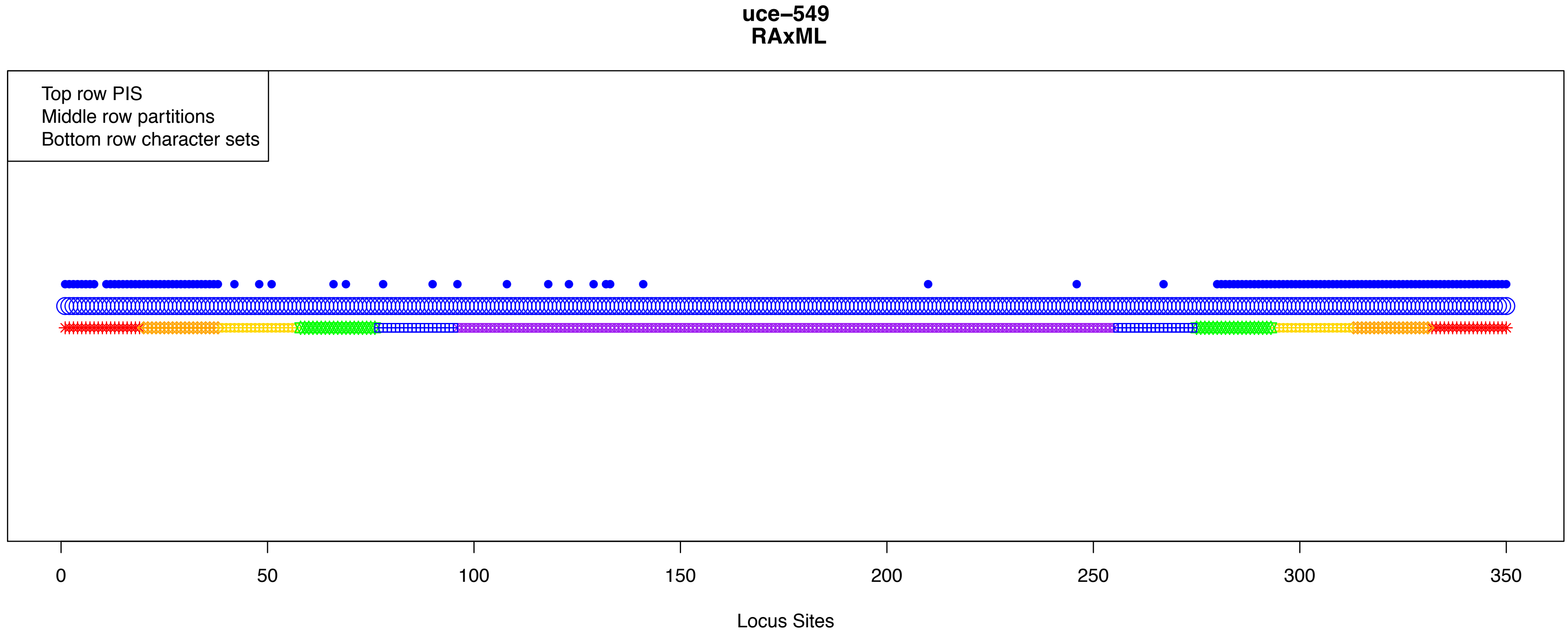
uce–549
RAxML
Top row PIS
Middle row partitions
Bottom row character sets
0
50
100
150
200
250
300
350
Locus Sites

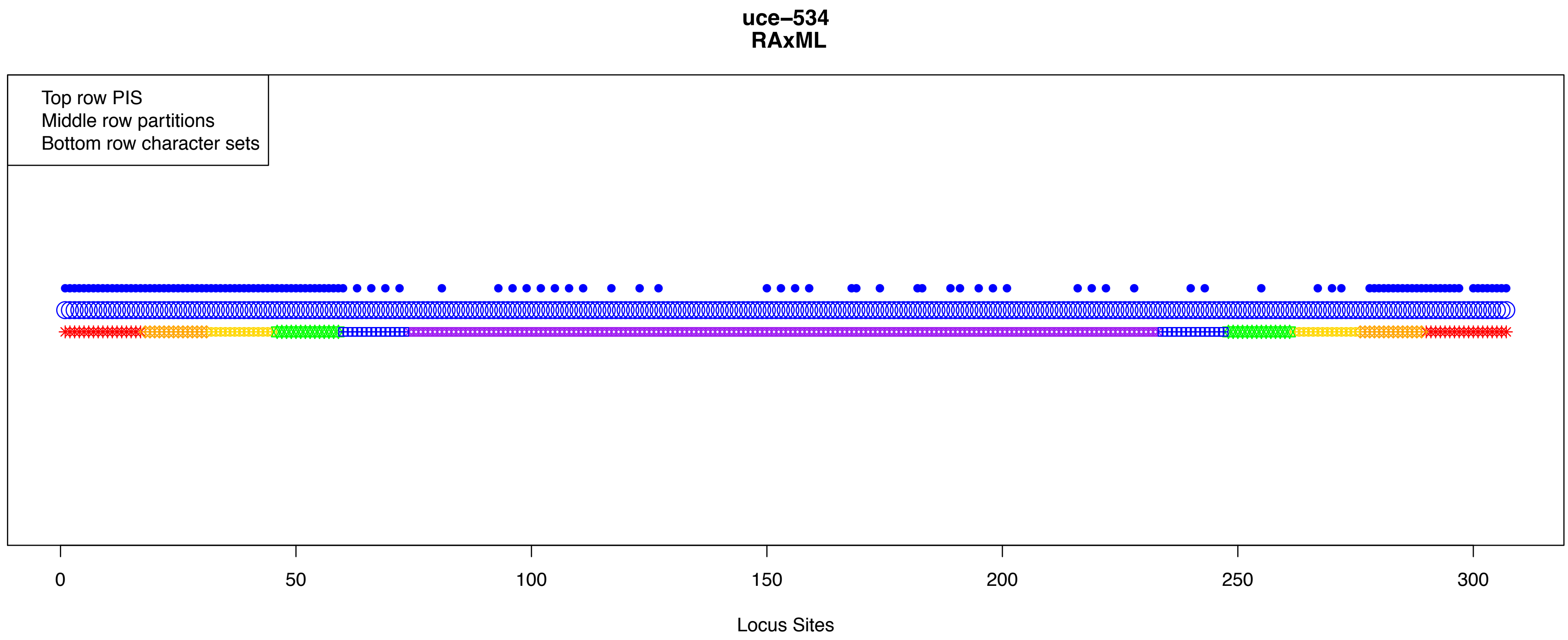
uce–534
RAxML
Top row PIS
Middle row partitions
Bottom row character sets
0
50
100
150
200
250
300
Locus Sites

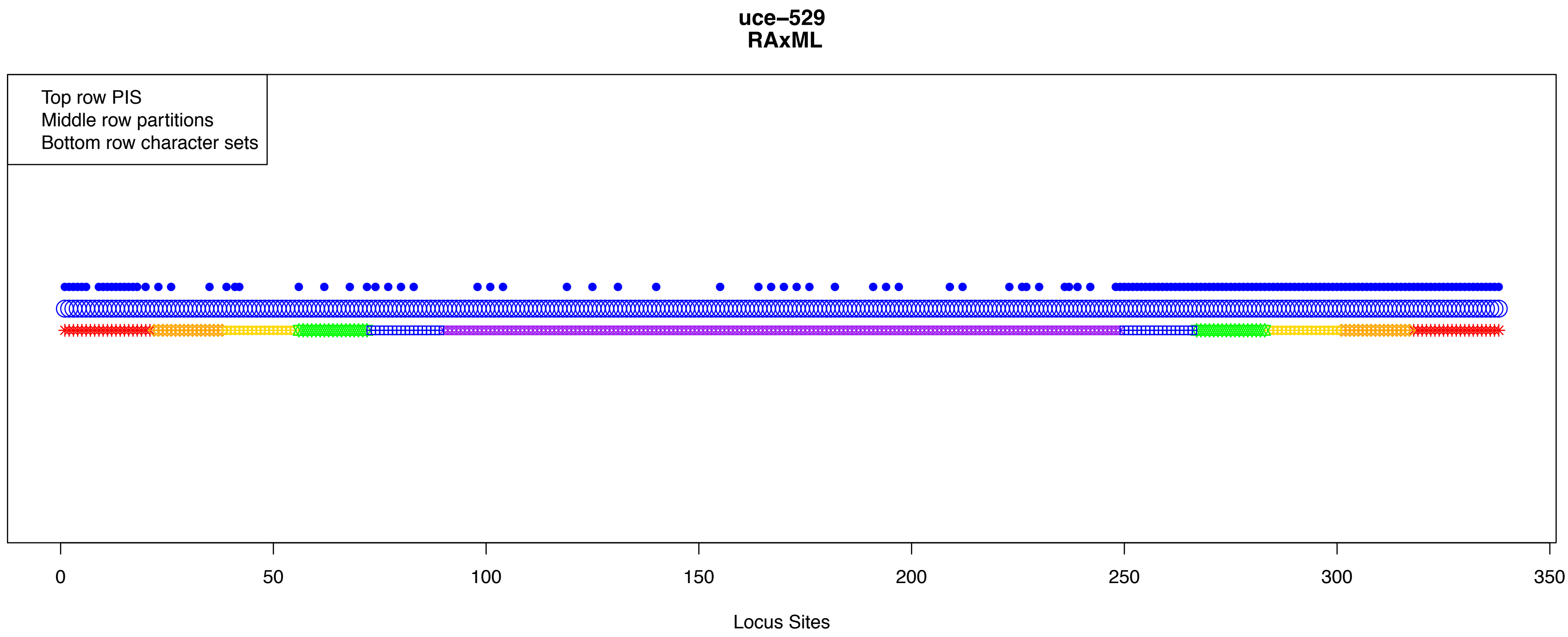

uce–529
RAxML
Top row PIS
Middle row partitions
Bottom row character sets
0
50
100
150
200
250
300
350
Locus Sites

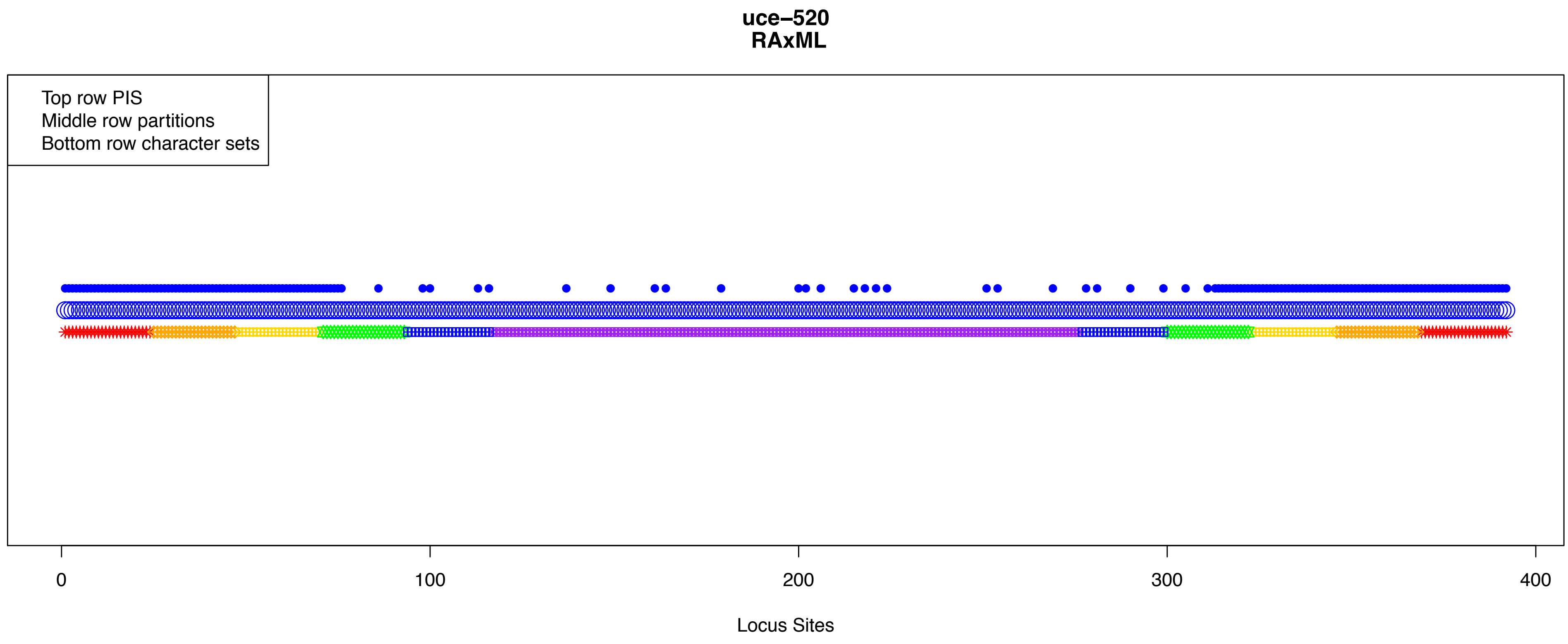

uce–520
RAxML
Top row PIS
Middle row partitions
Bottom row character sets
0
100
200
300
400
Locus Sites

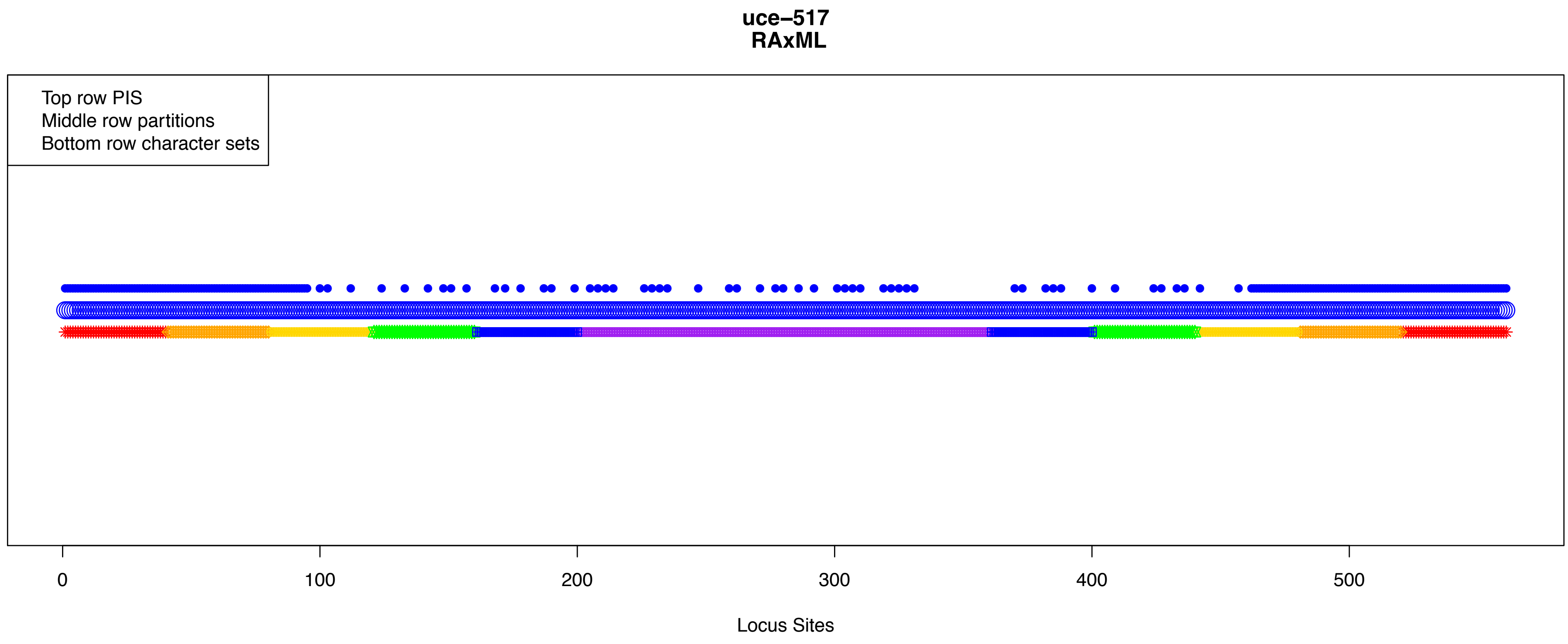

uce–517
RAxML
Top row PIS
Middle row partitions
Bottom row character sets
0
100
200
300
400
500
Locus Sites

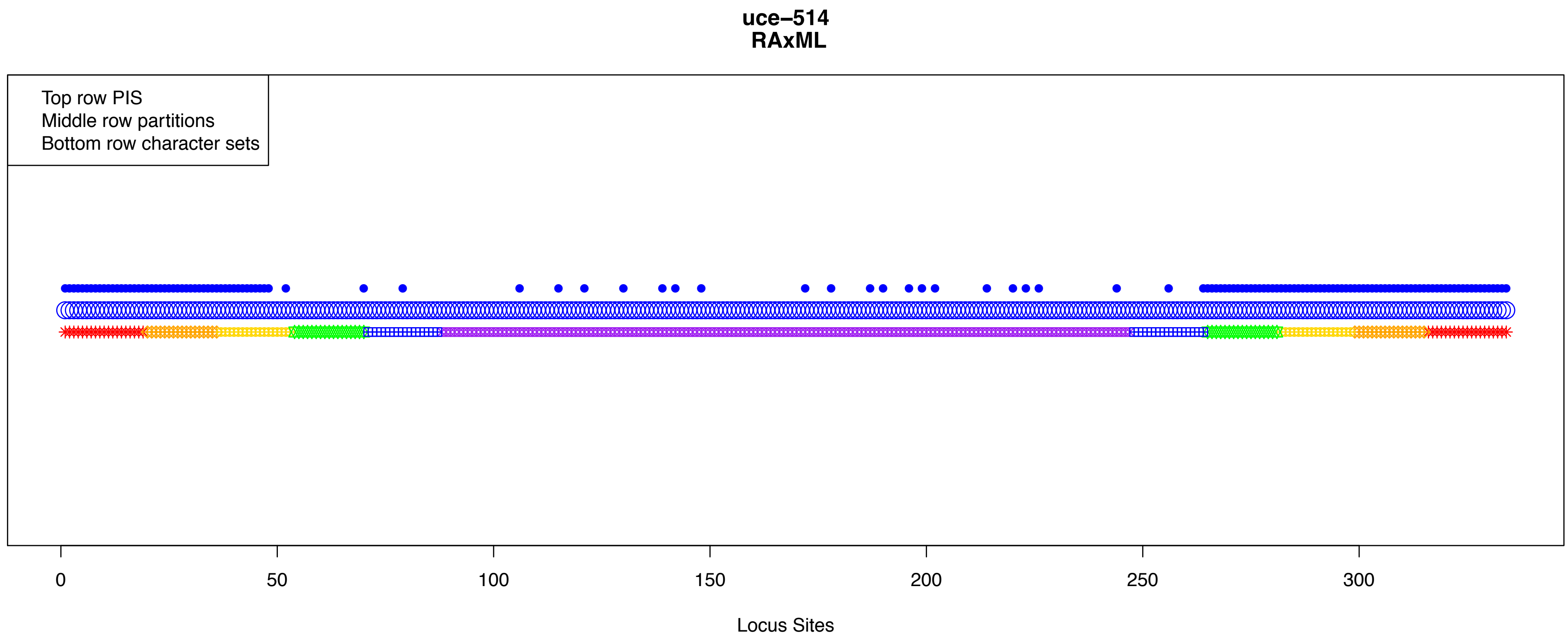

uce–514
RAxML
Top row PIS
Middle row partitions
Bottom row character sets
0
50
100
150
200
250
300
Locus Sites

uce–507
RAxML

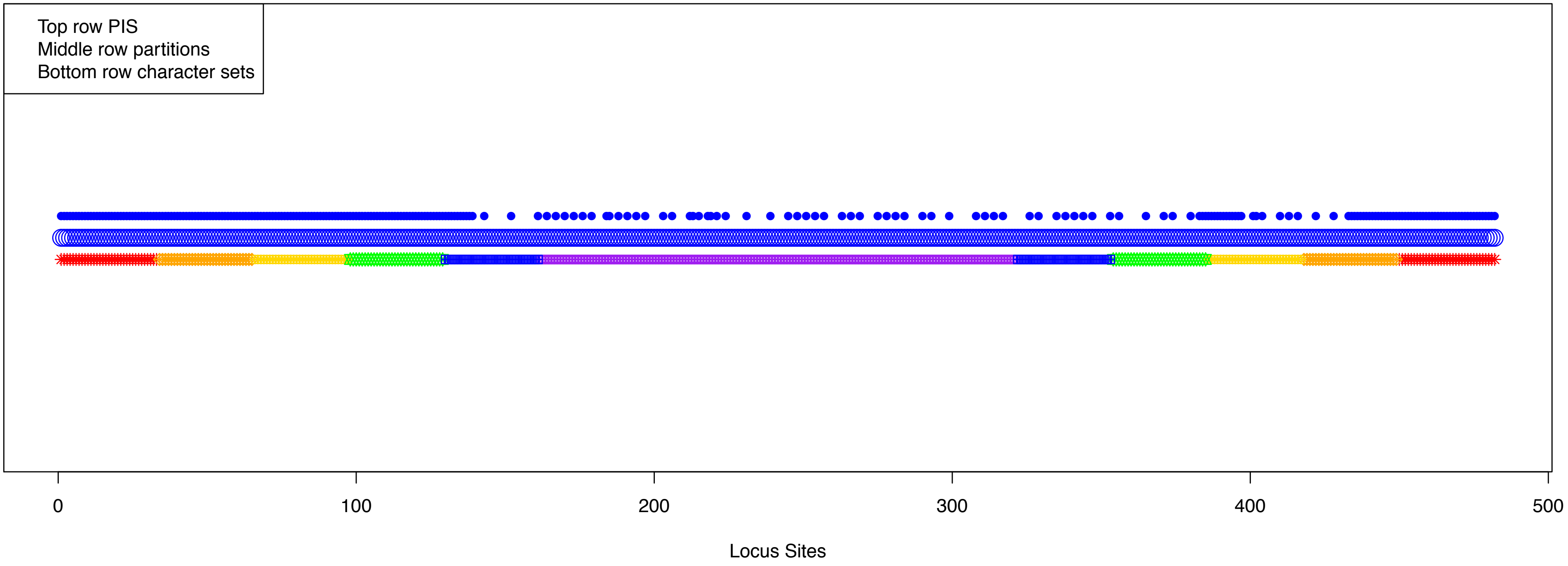

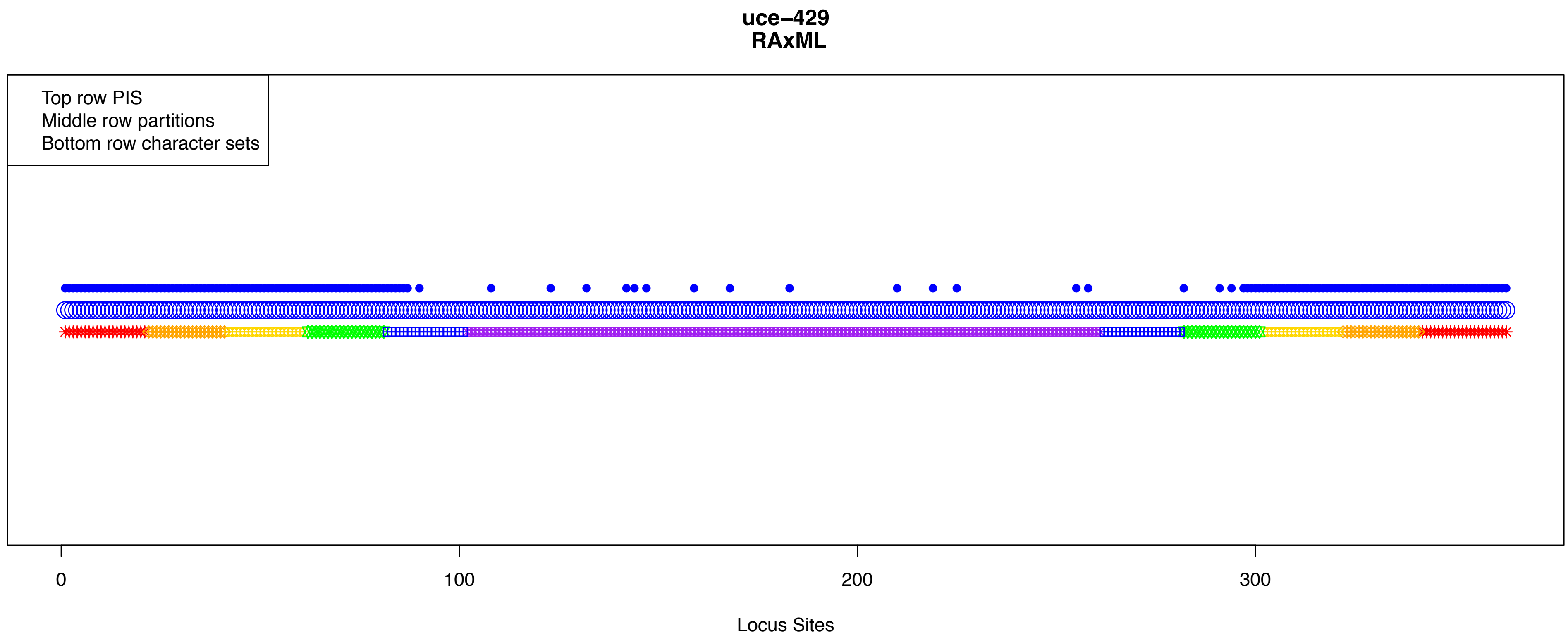
uce–429
RAxML
Top row PIS
Middle row partitions
Bottom row character sets
0
100
200
300
Locus Sites

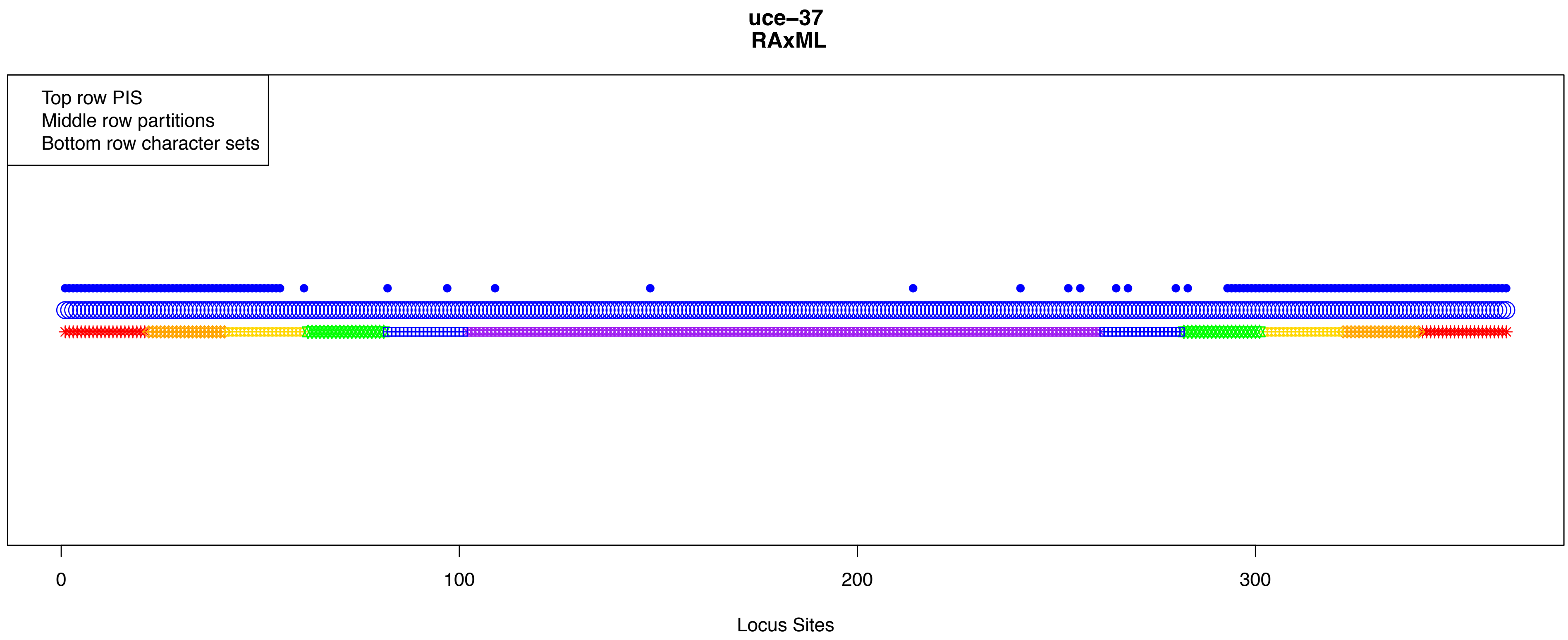
uce–37
RAxML
Top row PIS
Middle row partitions
Bottom row character sets
0
100
200
300
Locus Sites

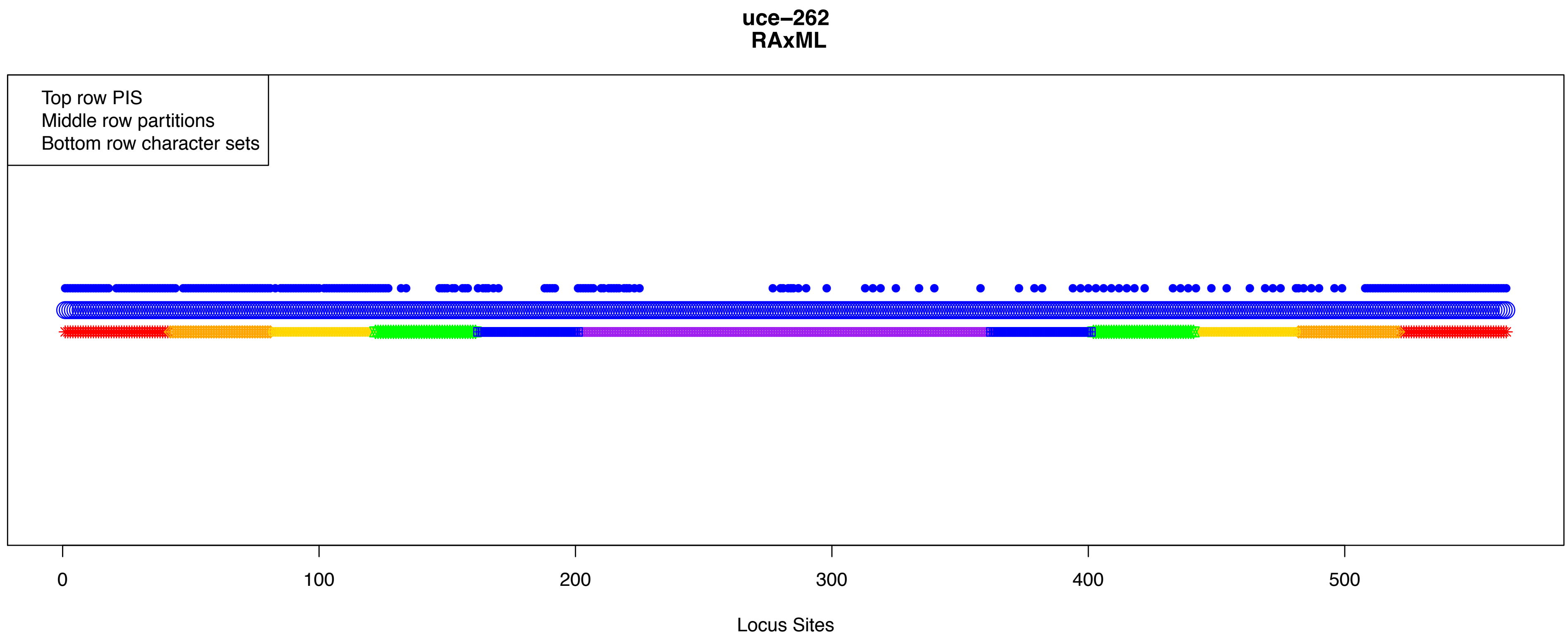

uce–262
RAxML
Top row PIS
Middle row partitions
Bottom row character sets
0
100
200
300
400
500
Locus Sites

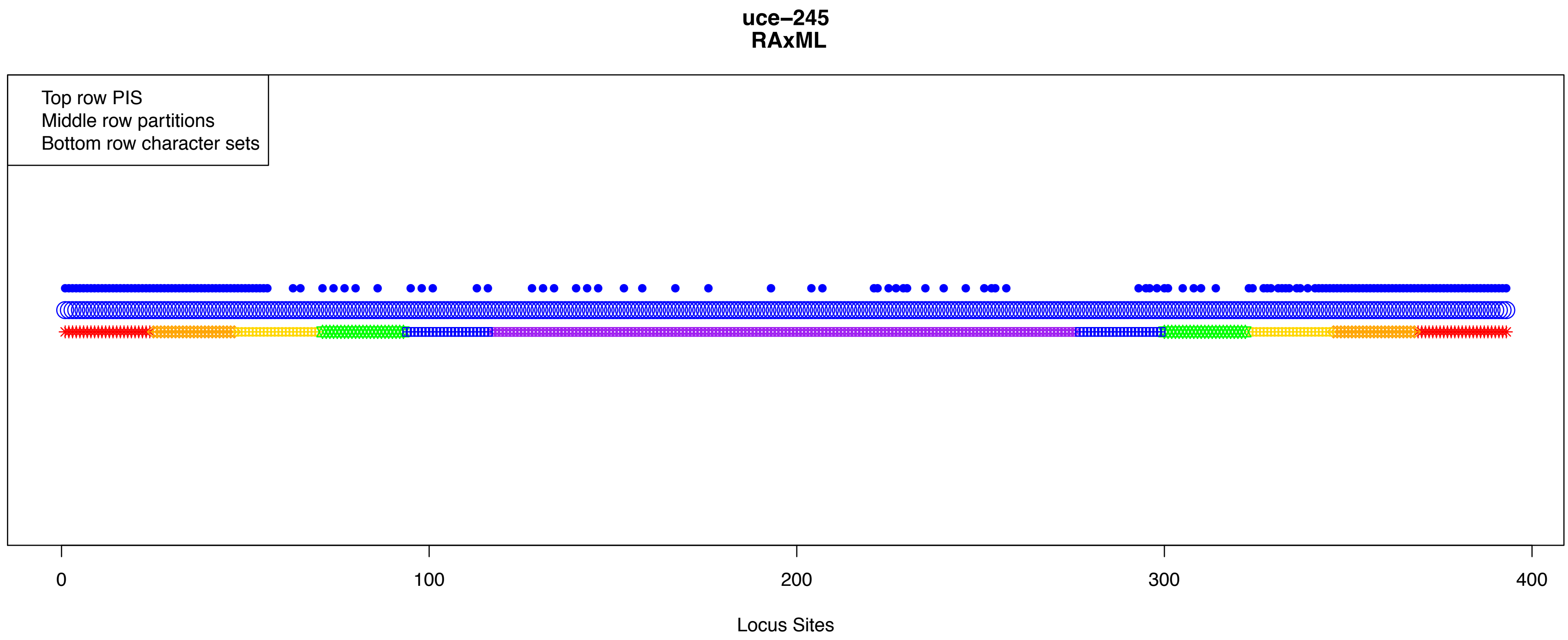

uce–245
RAxML
Top row PIS
Middle row partitions
Bottom row character sets
0
100
200
300
400
Locus Sites

uce–214
RAxML

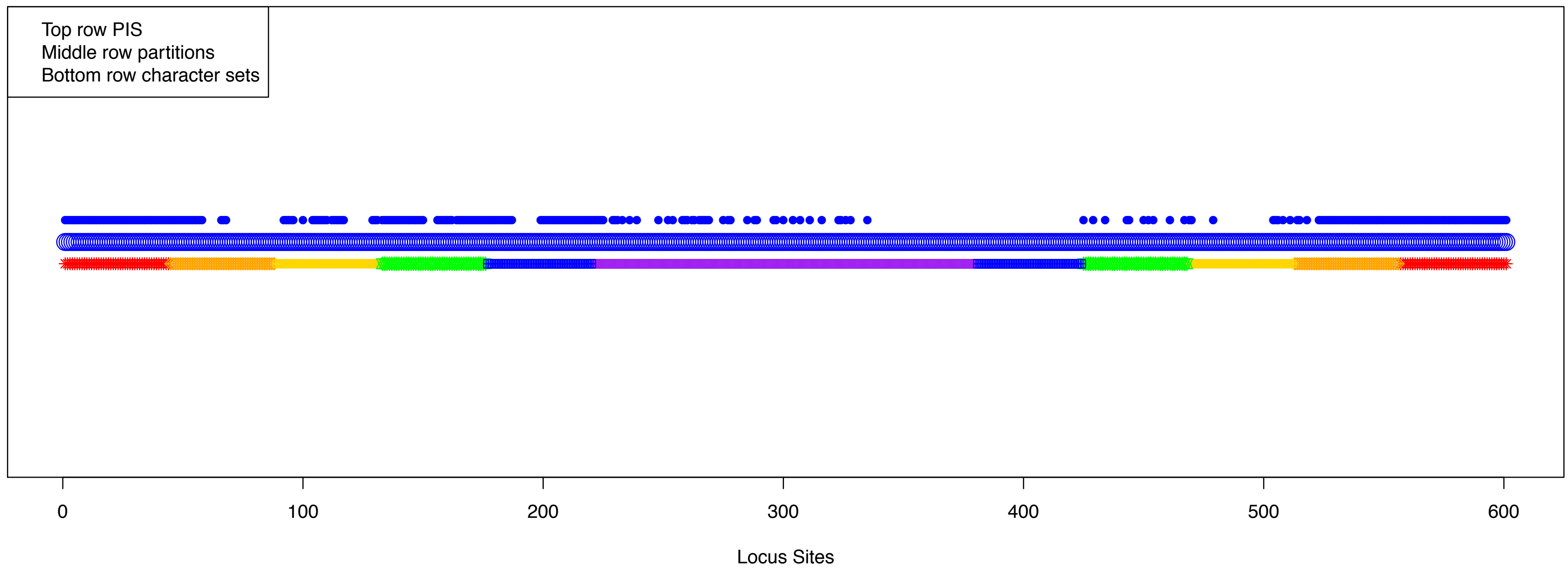

uce–21
RAxML

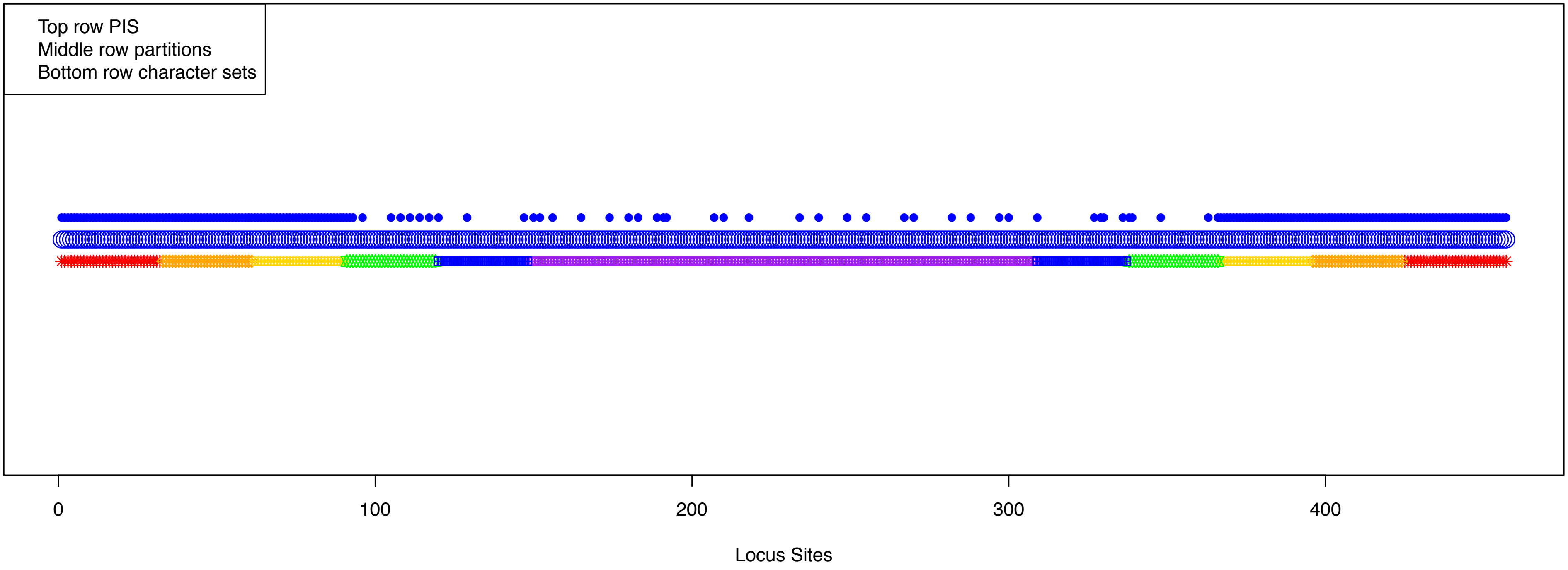

Top row PIS
Middle row partitions
Bottom row character sets
0
100
200
300
400
Locus Sites

uce–1812
RAxML

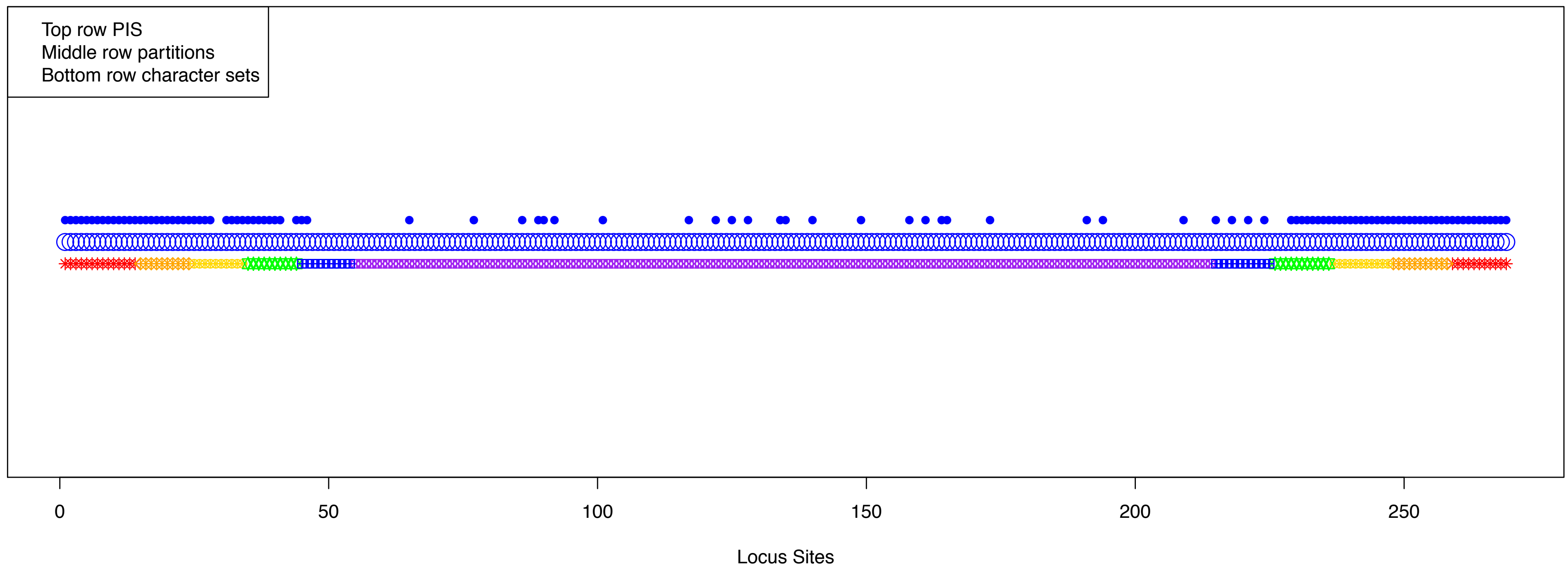

uce–1748
RAxML

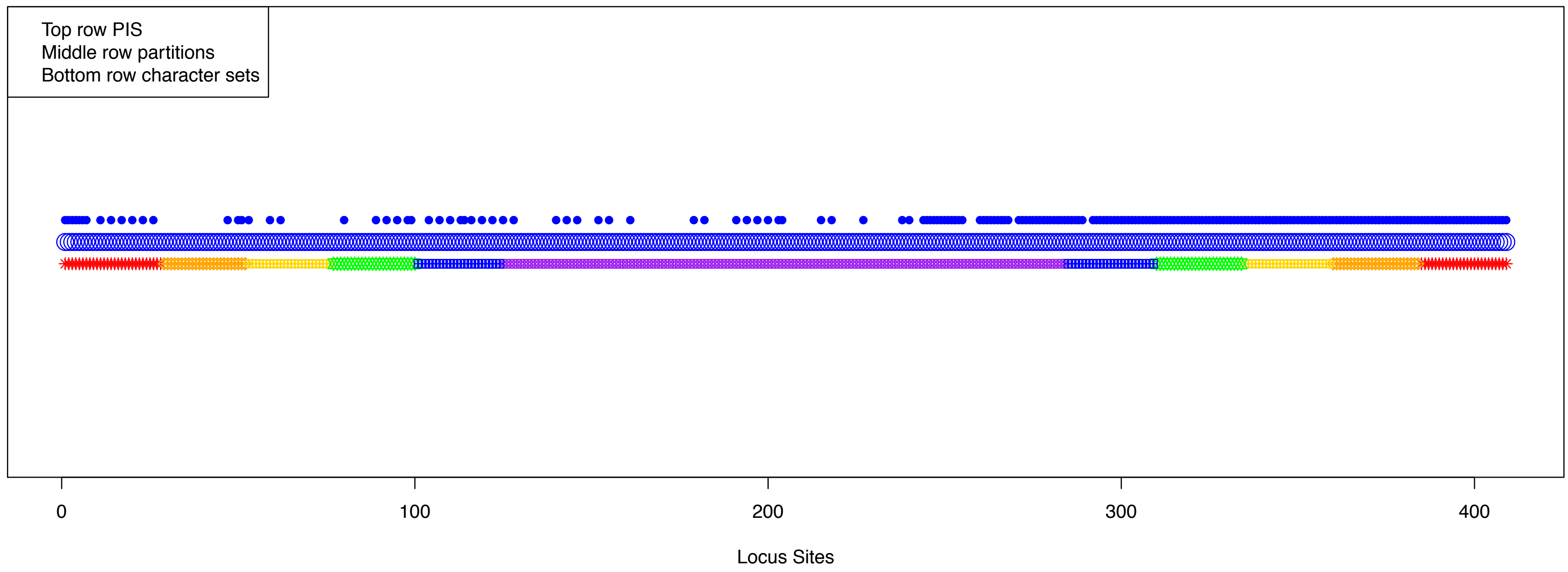

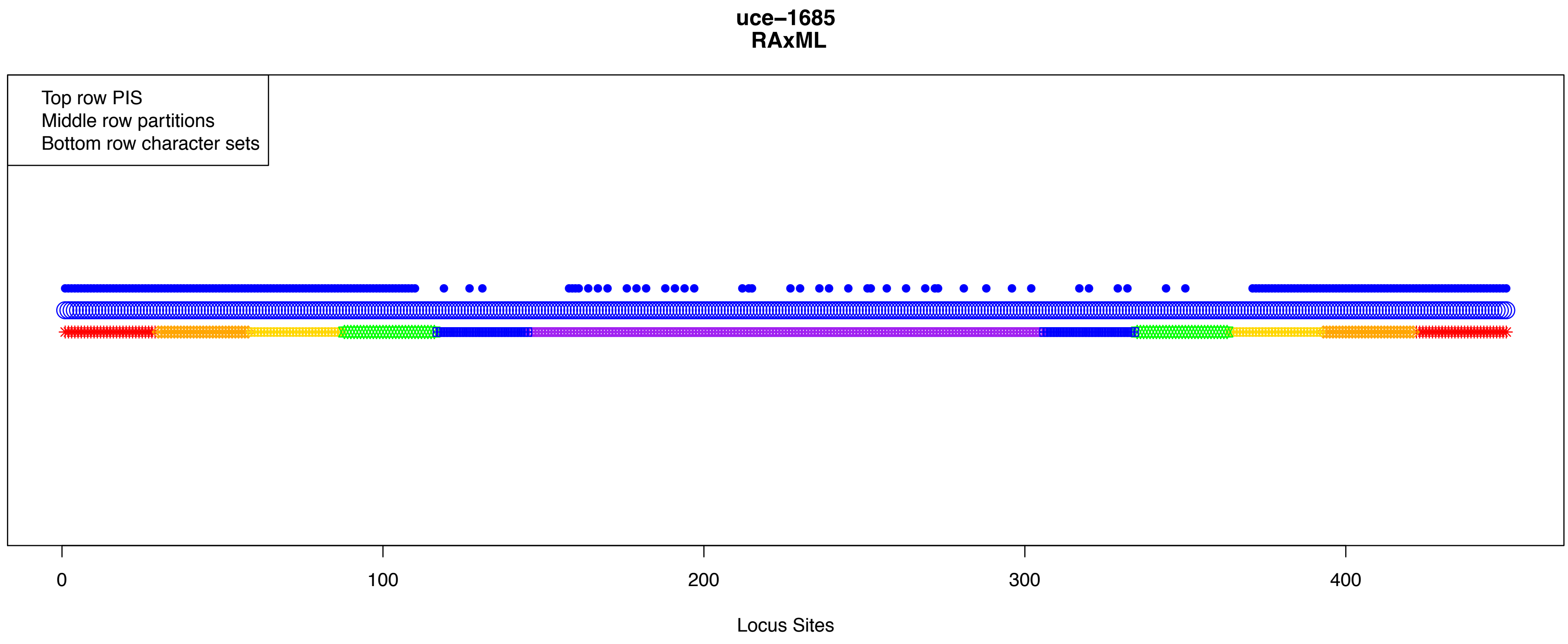

uce–1685
RAxML
Top row PIS
Middle row partitions
Bottom row character sets
0
100
200
300
400
Locus Sites

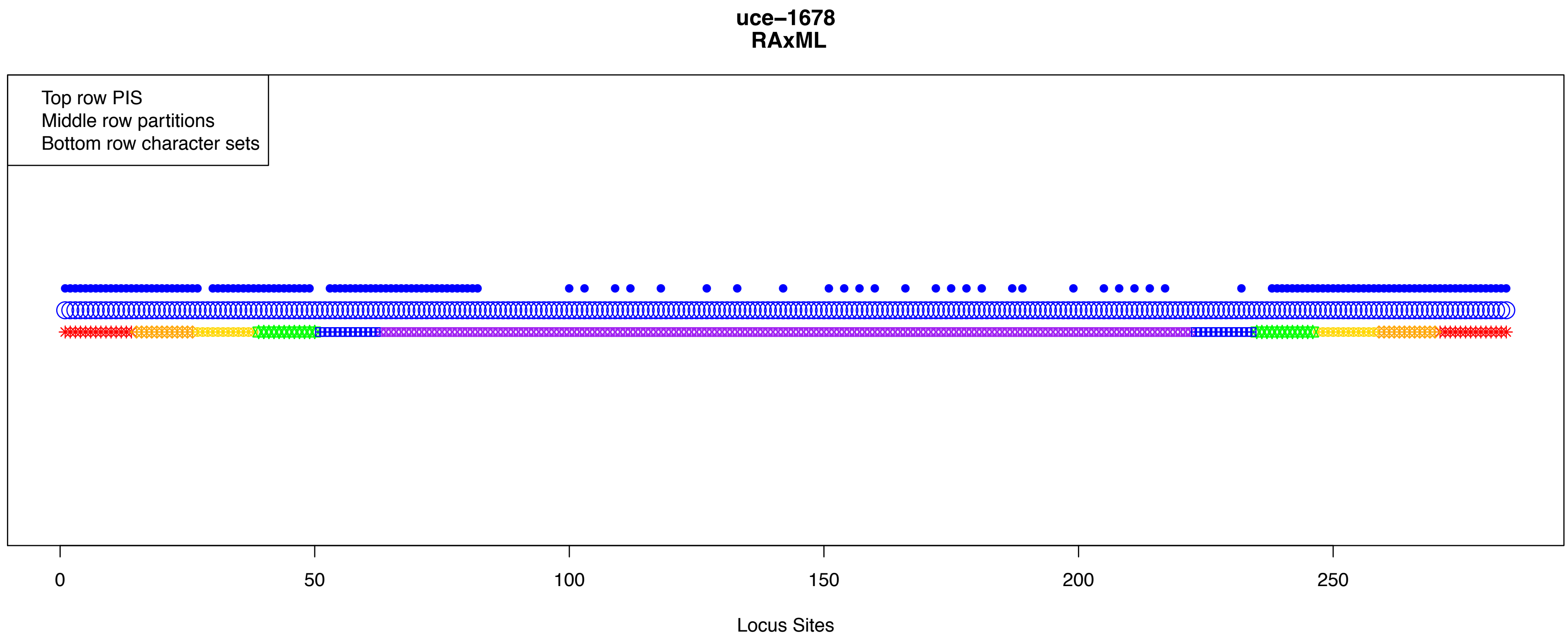
uce–1678
RAxML
Top row PIS
Middle row partitions
Bottom row character sets
0
50
100
150
200
250
Locus Sites

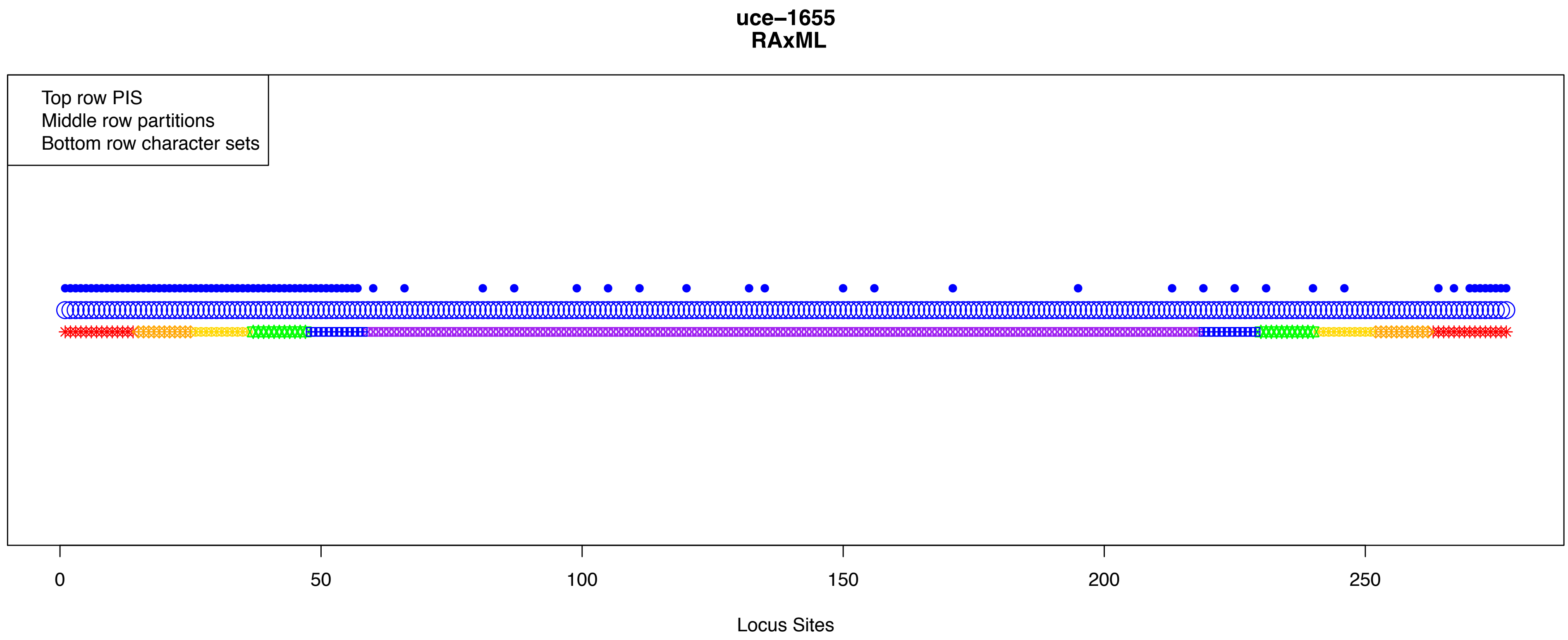
uce–1655
RAxML
Top row PIS
Middle row partitions
Bottom row character sets
0
50
100
150
200
250
Locus Sites

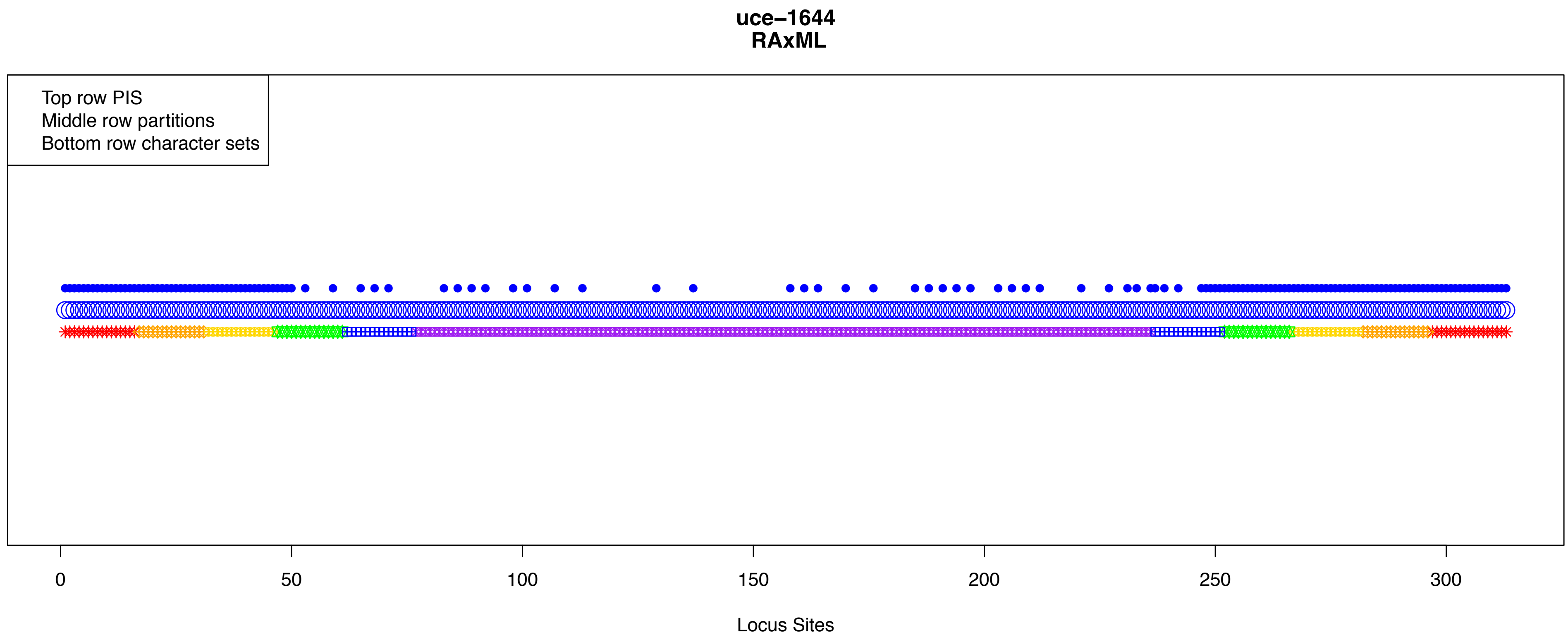

uce–1644
RAxML
Top row PIS
Middle row partitions
Bottom row character sets
0
50
100
150
200
250
300
Locus Sites

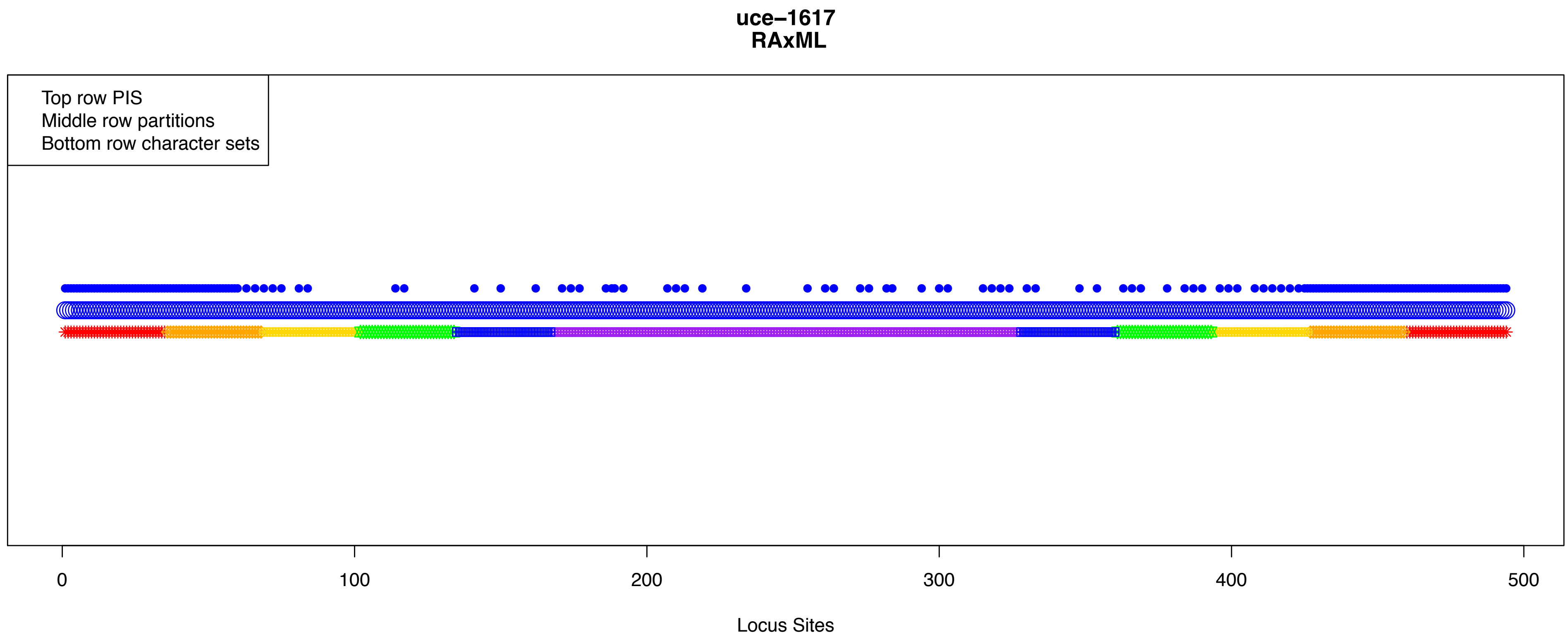
uce–1617
RAxML
Top row PIS
Middle row partitions
Bottom row character sets
0
100
200
300
400
500
Locus Sites

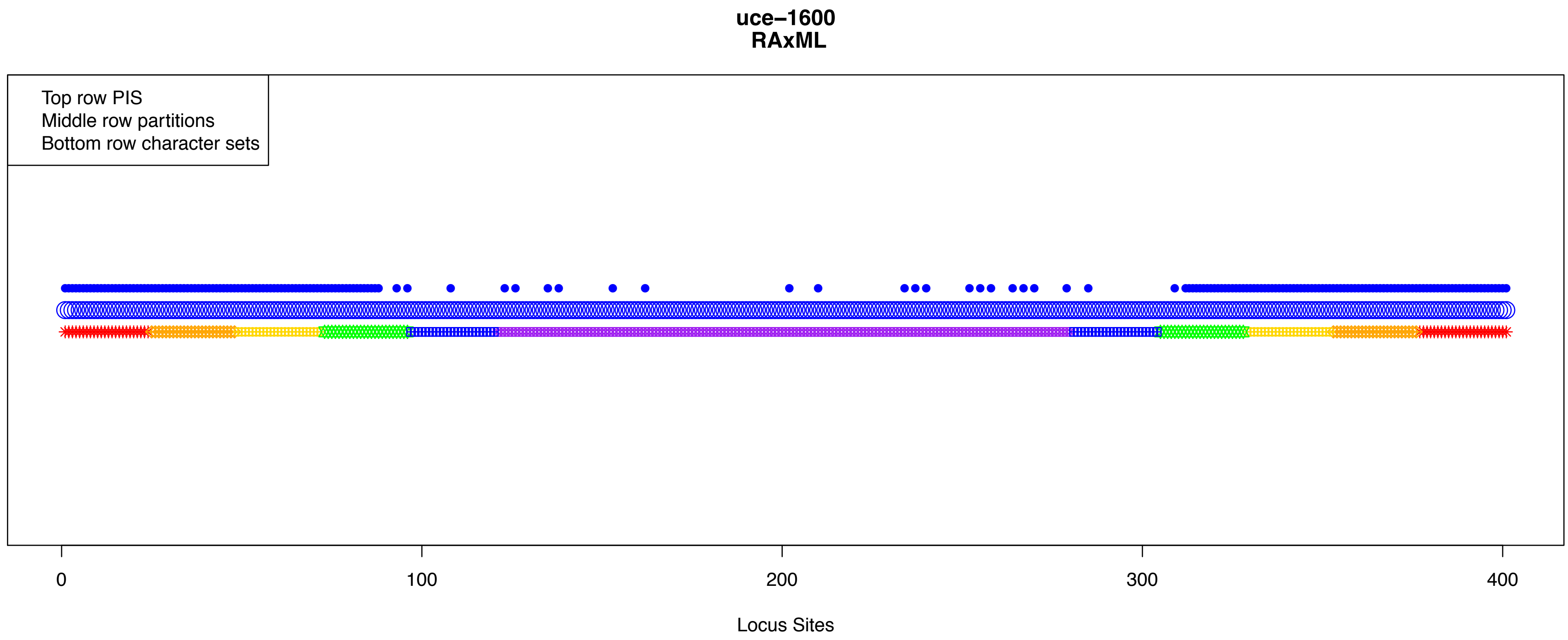

uce–1600
RAxML
Top row PIS
Middle row partitions
Bottom row character sets
0
100
200
300
400
Locus Sites

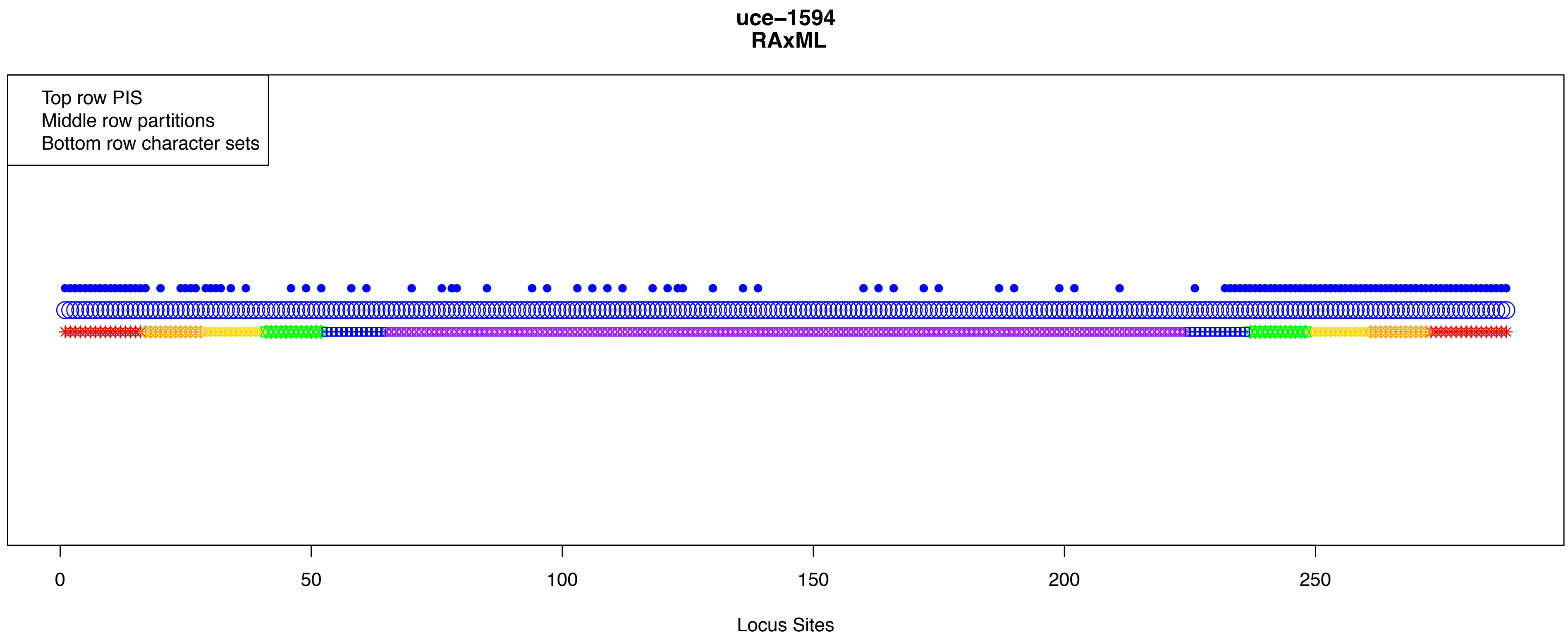
uce–1594
RAxML
Top row PIS
Middle row partitions
Bottom row character sets
0
50
100
150
200
250
Locus Sites

uce–1591
RAxML

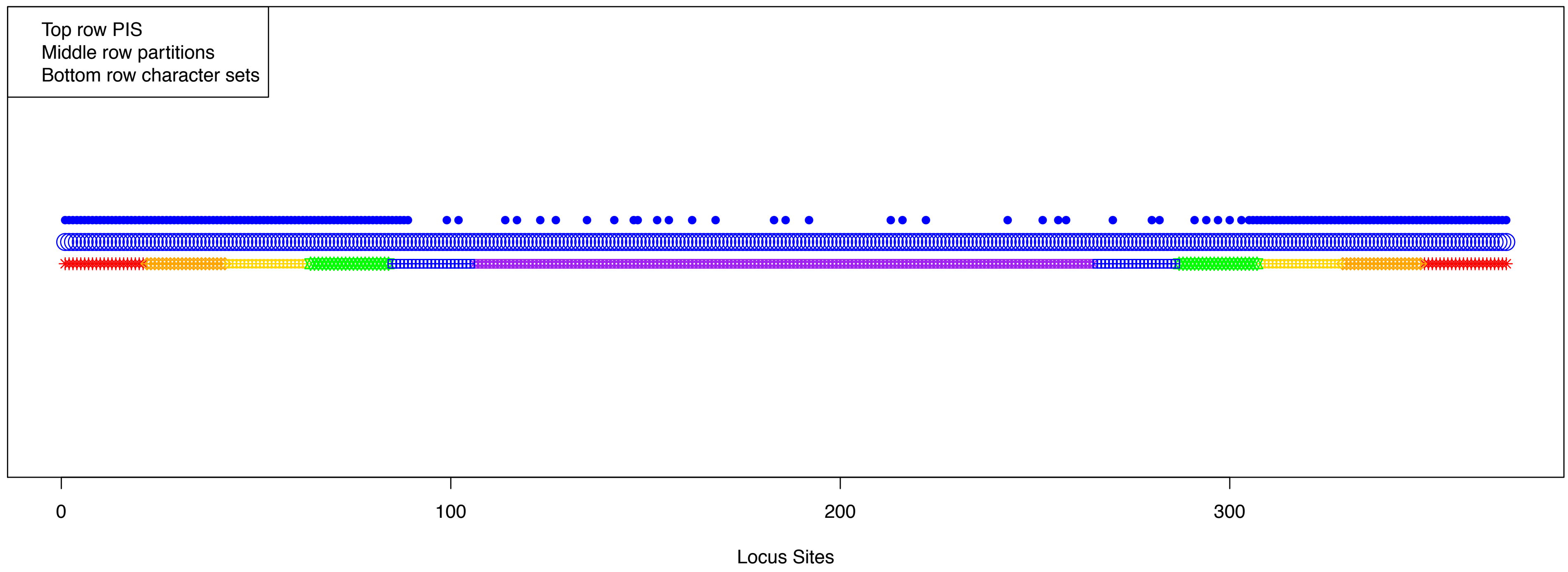

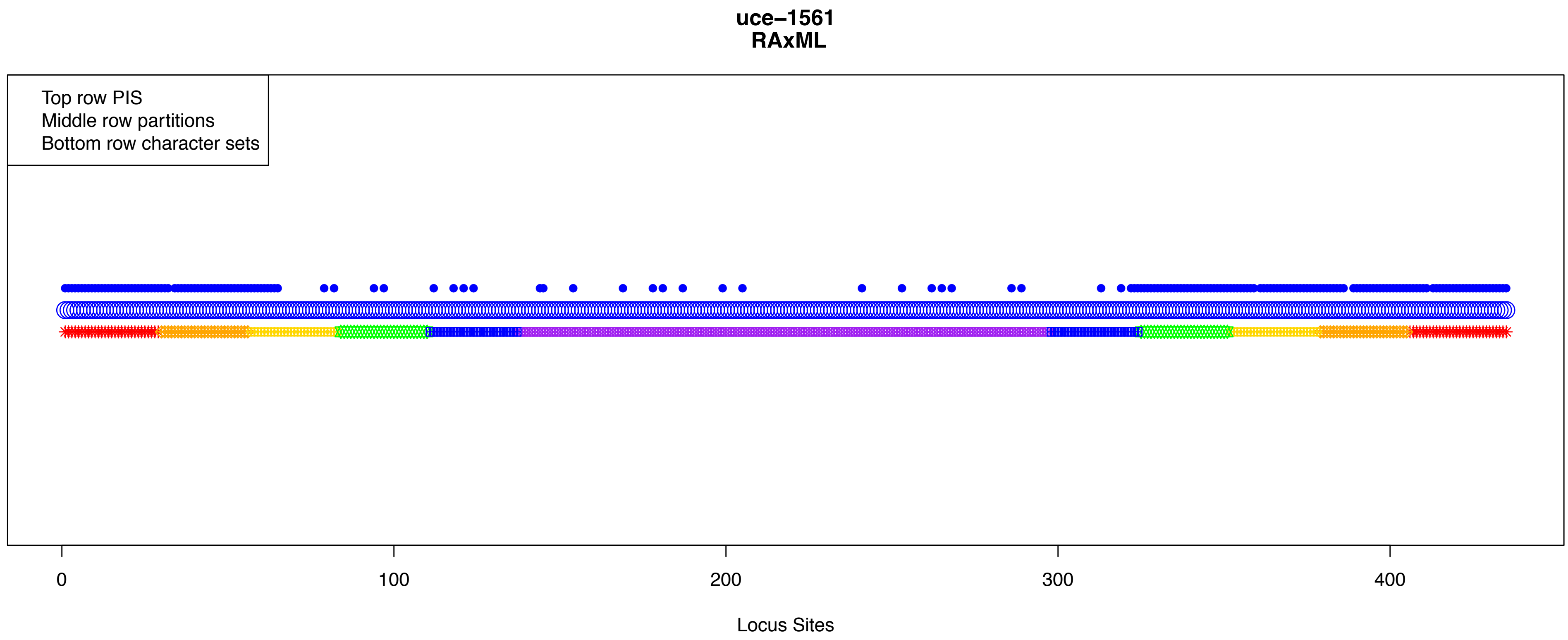

uce–1561
RAxML
Top row PIS
Middle row partitions
Bottom row character sets
0
100
200
300
400
Locus Sites

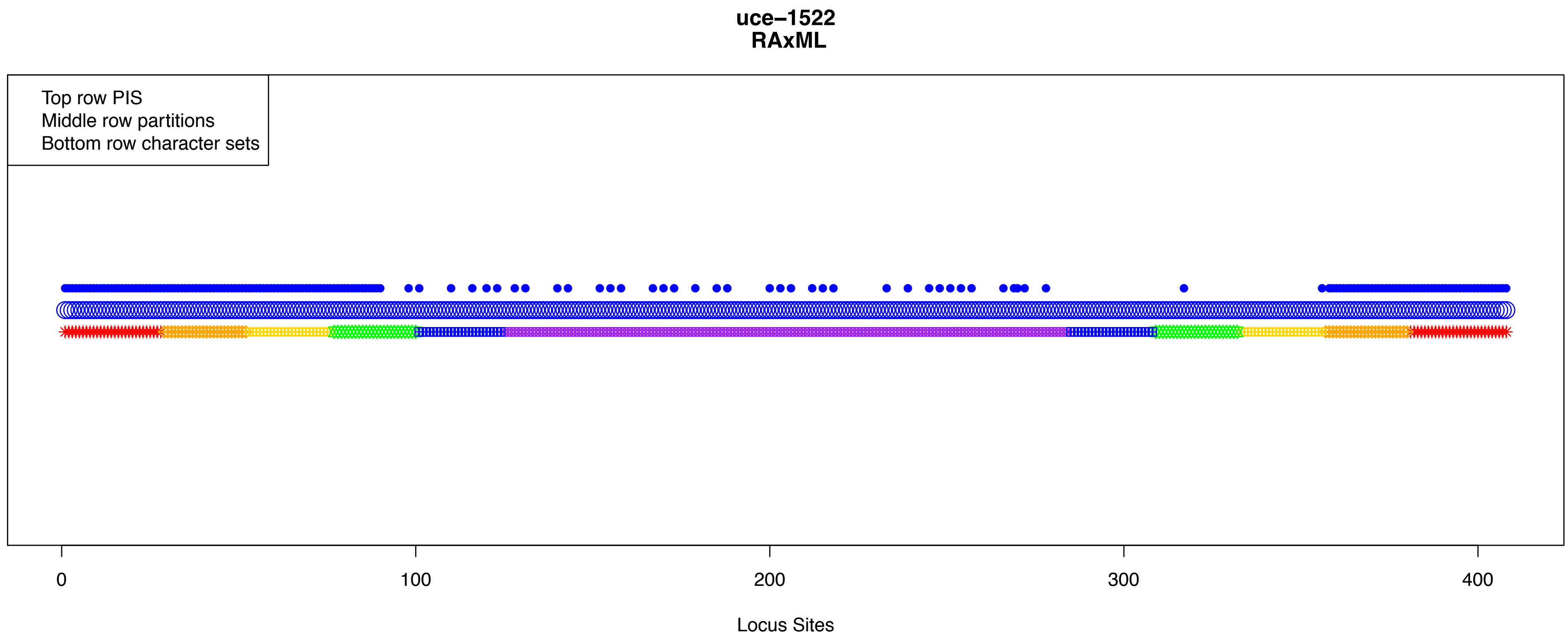
uce–1522
RAxML
Top row PIS
Middle row partitions
Bottom row character sets
0
100
200
300
400
Locus Sites

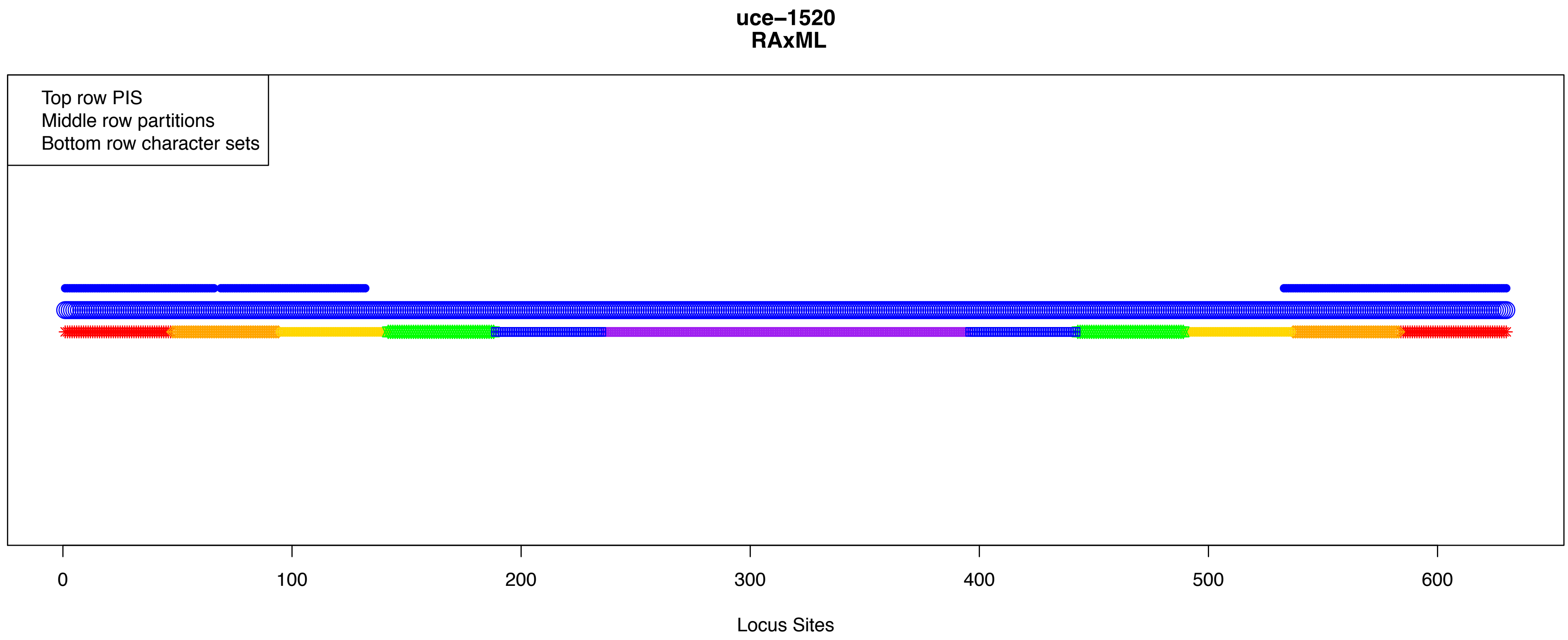

uce–1520
RAxML
Top row PIS
Middle row partitions
Bottom row character sets
0
100
200
300
400
500
600
Locus Sites

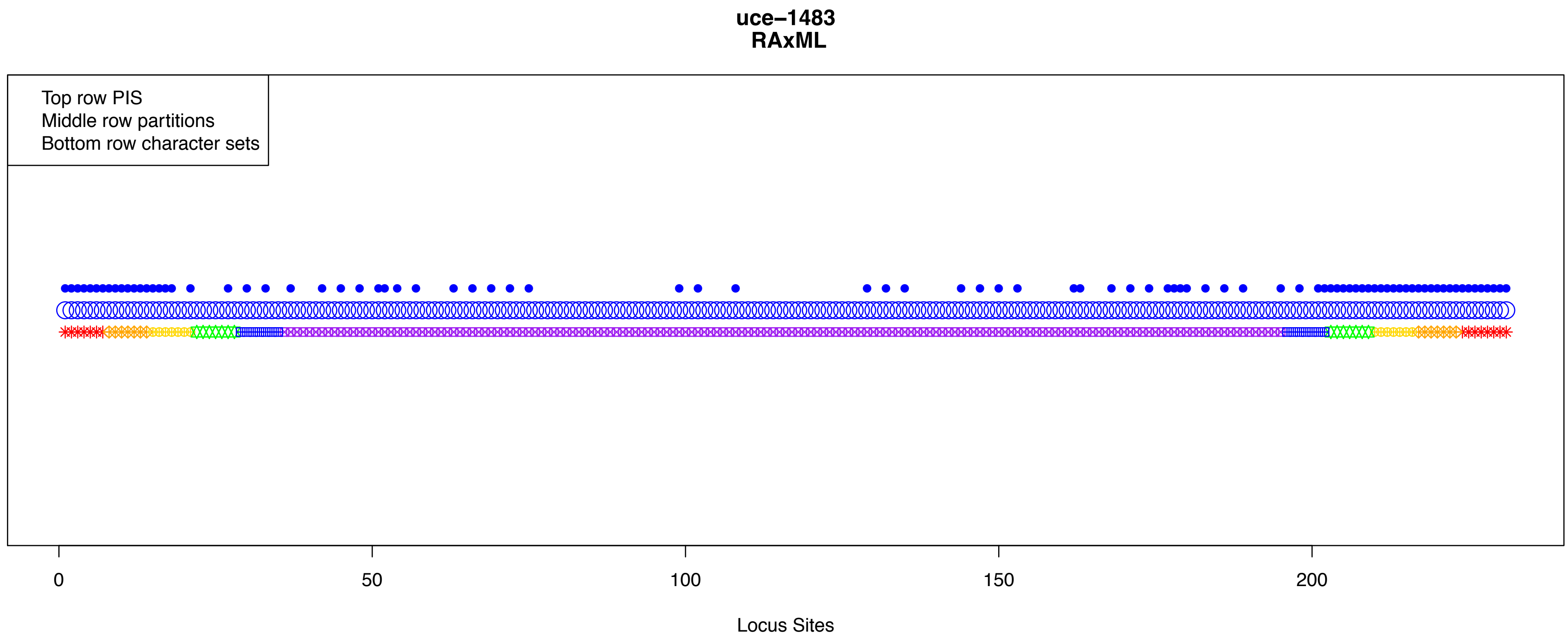
uce–1483
RAxML
Top row PIS
Middle row partitions
Bottom row character sets
0
50
100
150
200
Locus Sites

uce–145
RAxML

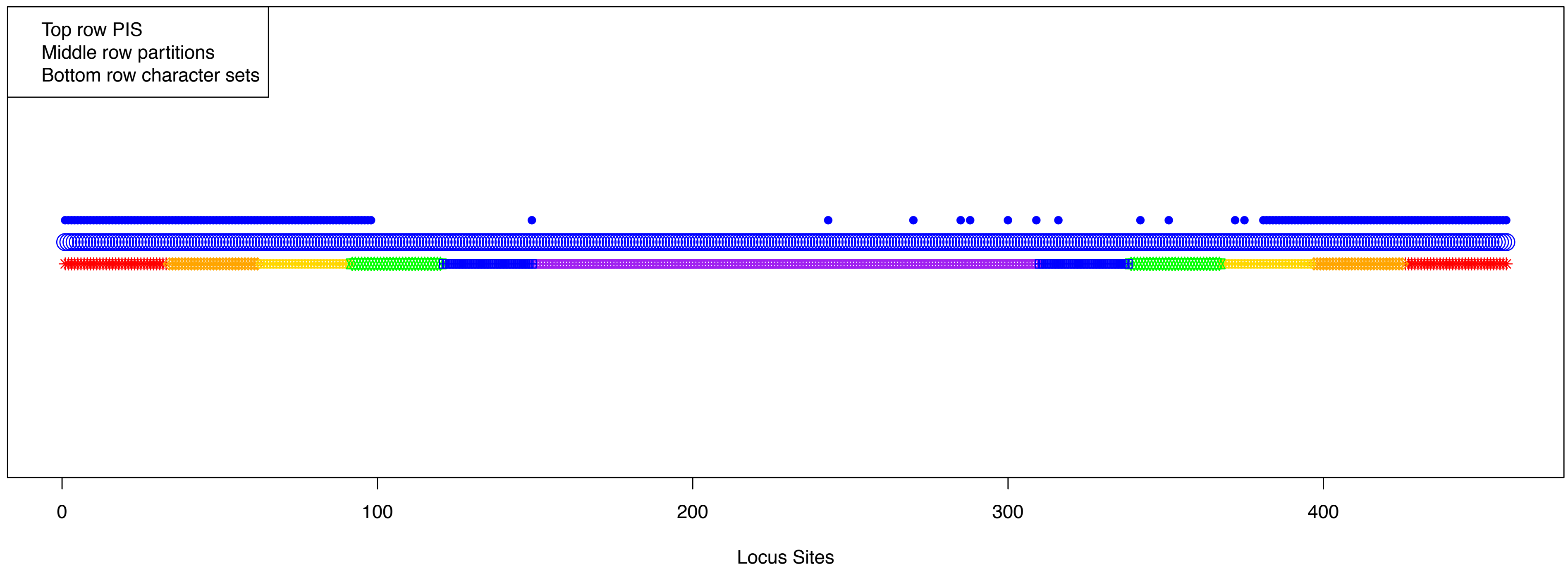

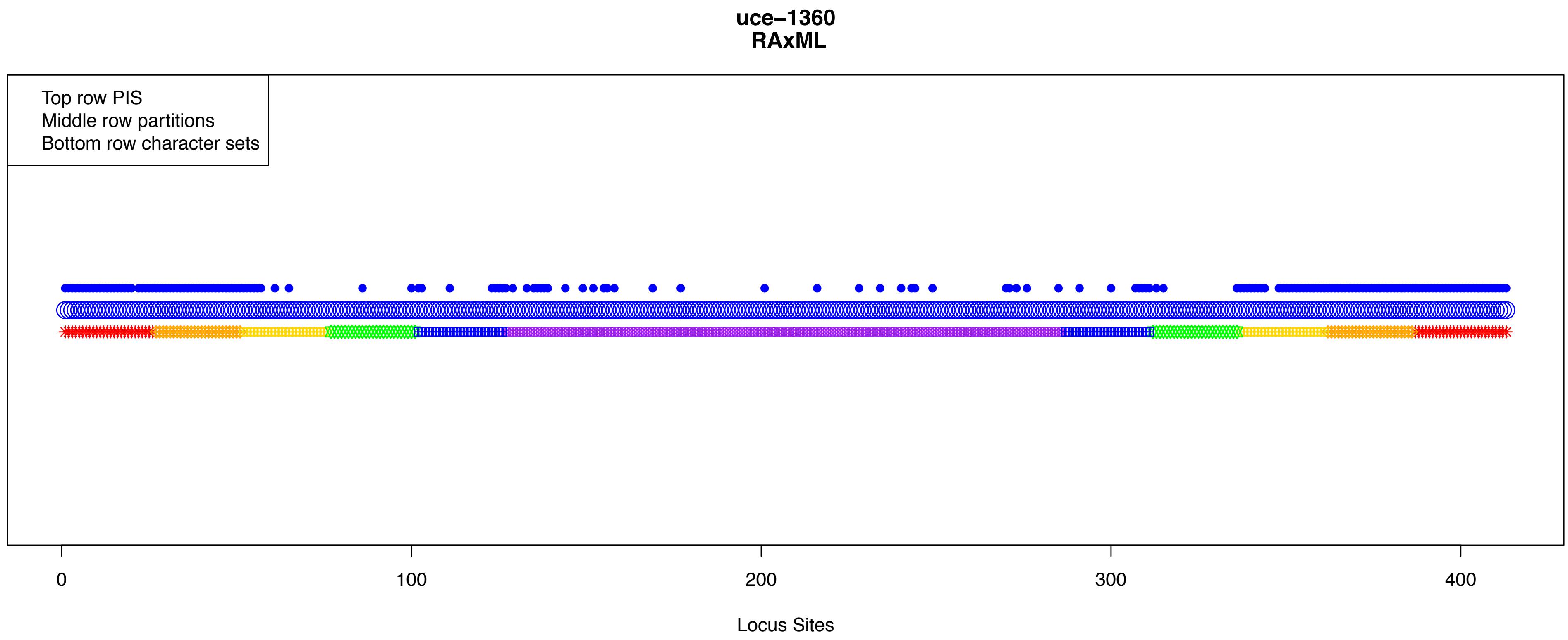
uce–1360
RAxML
Top row PIS
Middle row partitions
Bottom row character sets
0
100
200
300
400
Locus Sites

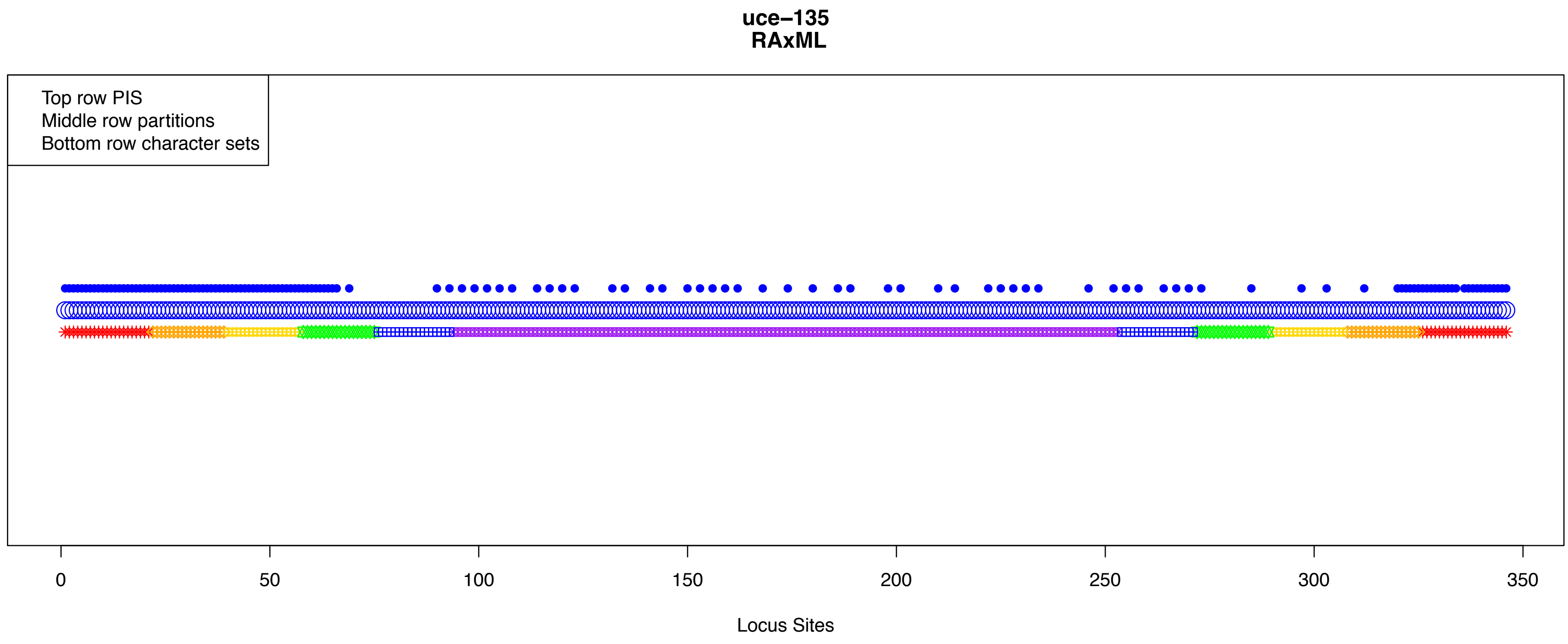

uce–135
RAxML
Top row PIS
Middle row partitions
Bottom row character sets
0
50
100
150
200
250
300
350
Locus Sites

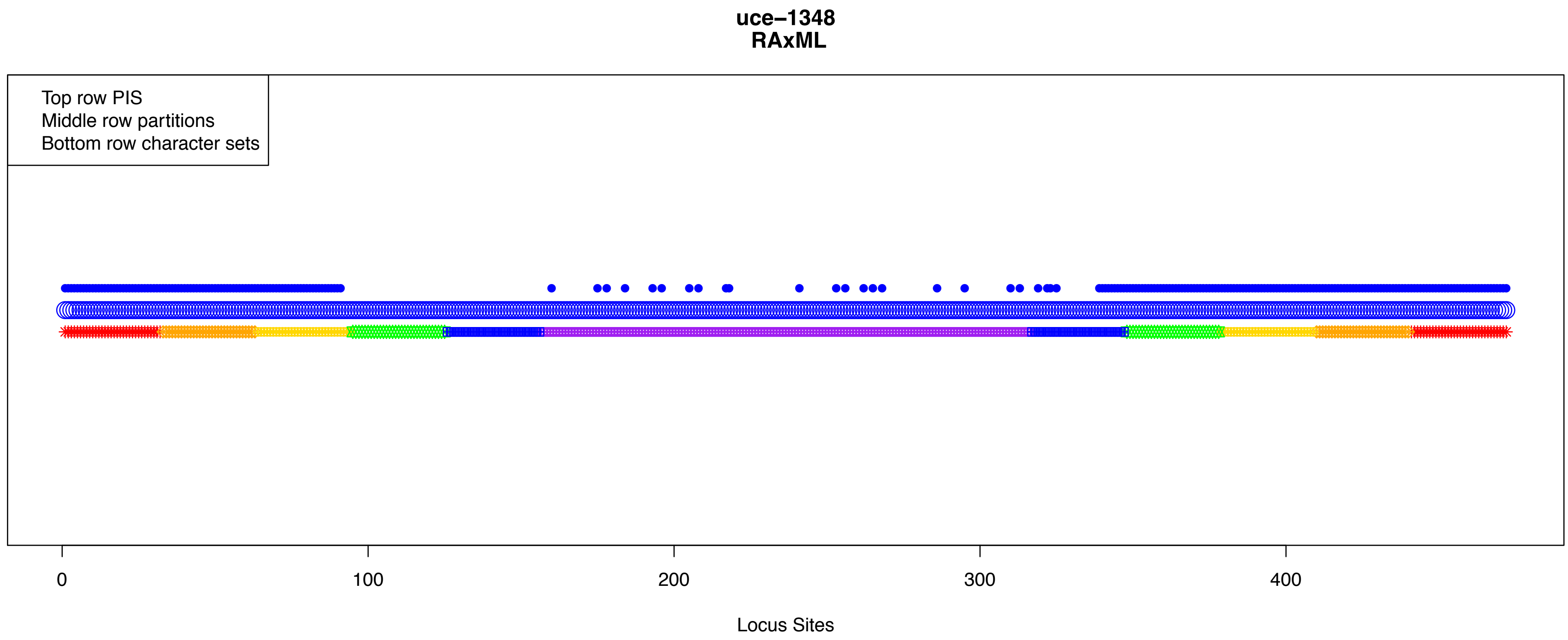

uce–1348
RAxML
Top row PIS
Middle row partitions
Bottom row character sets
0
100
200
300
400
Locus Sites

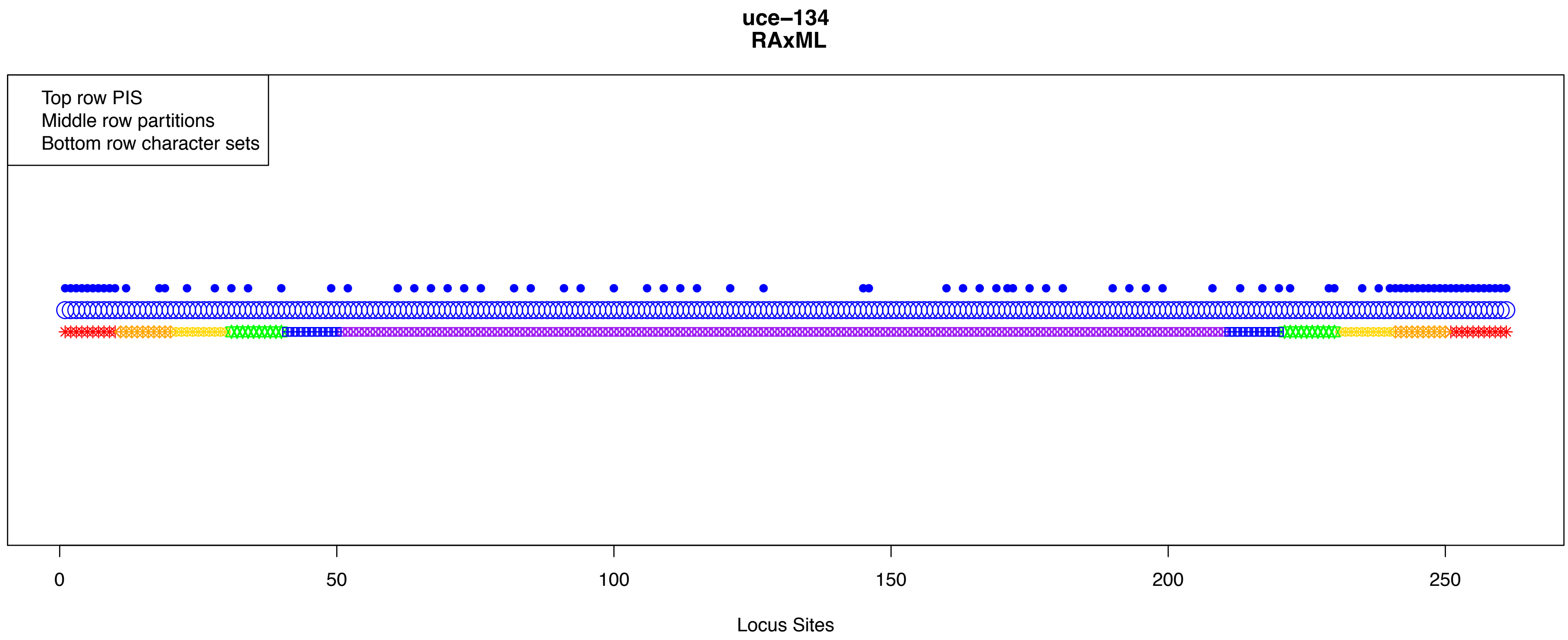

uce–134
RAxML
Top row PIS
Middle row partitions
Bottom row character sets
0
50
100
150
200
250
Locus Sites

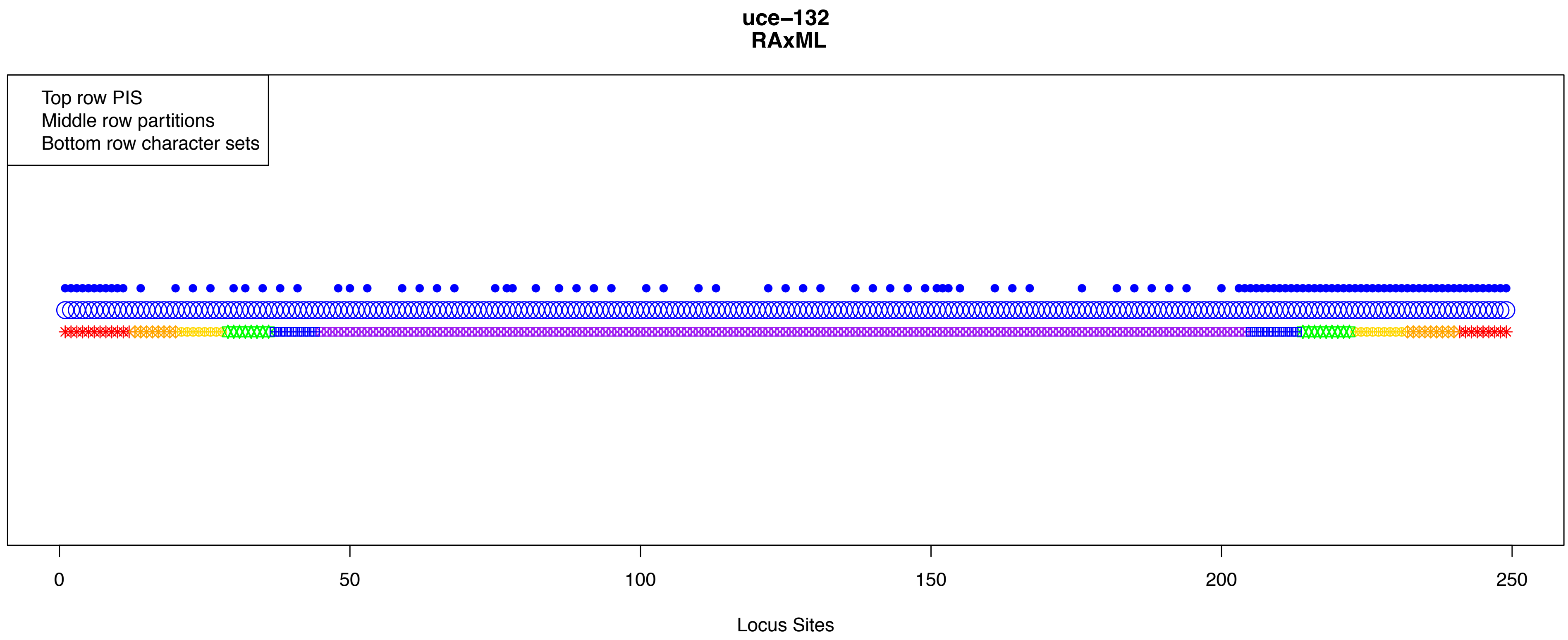
uce–132
RAxML
Top row PIS
Middle row partitions
Bottom row character sets
0
50
100
150
200
250
Locus Sites

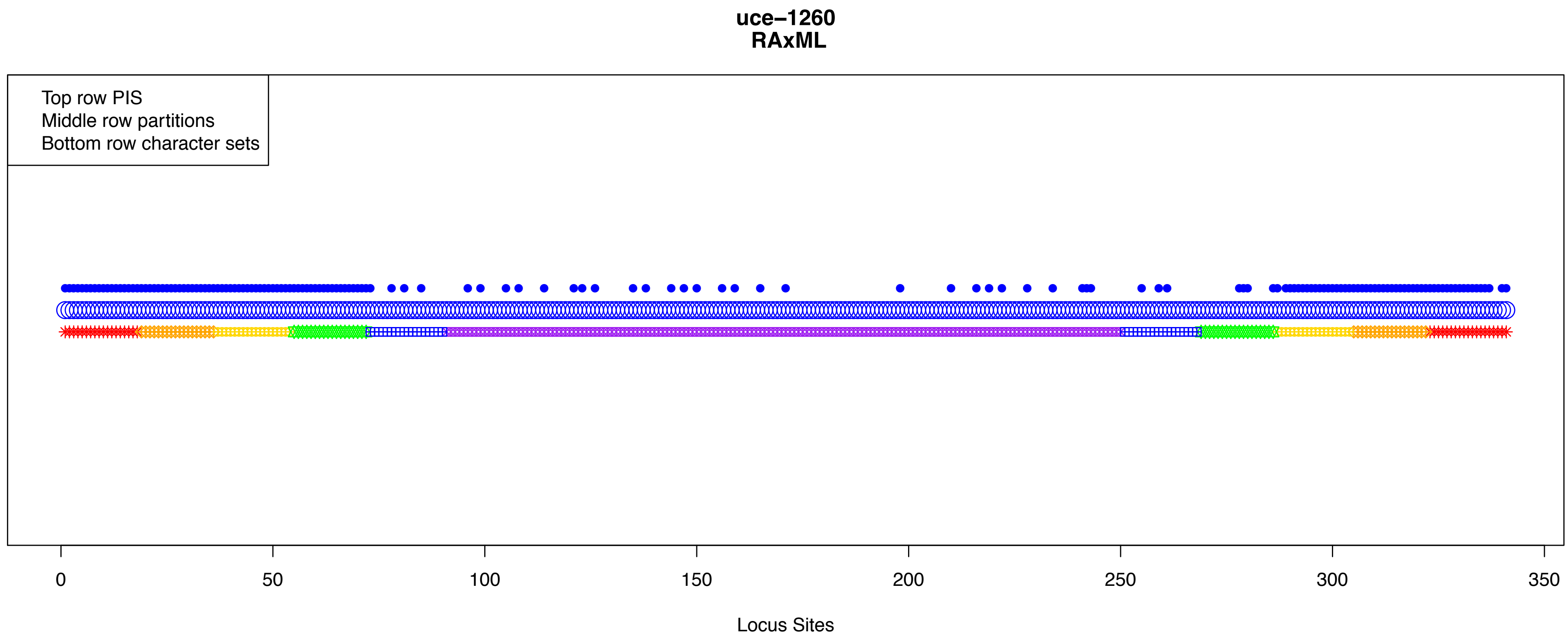
uce–1260
RAxML
Top row PIS
Middle row partitions
Bottom row character sets
0
50
100
150
200
250
300
350
Locus Sites

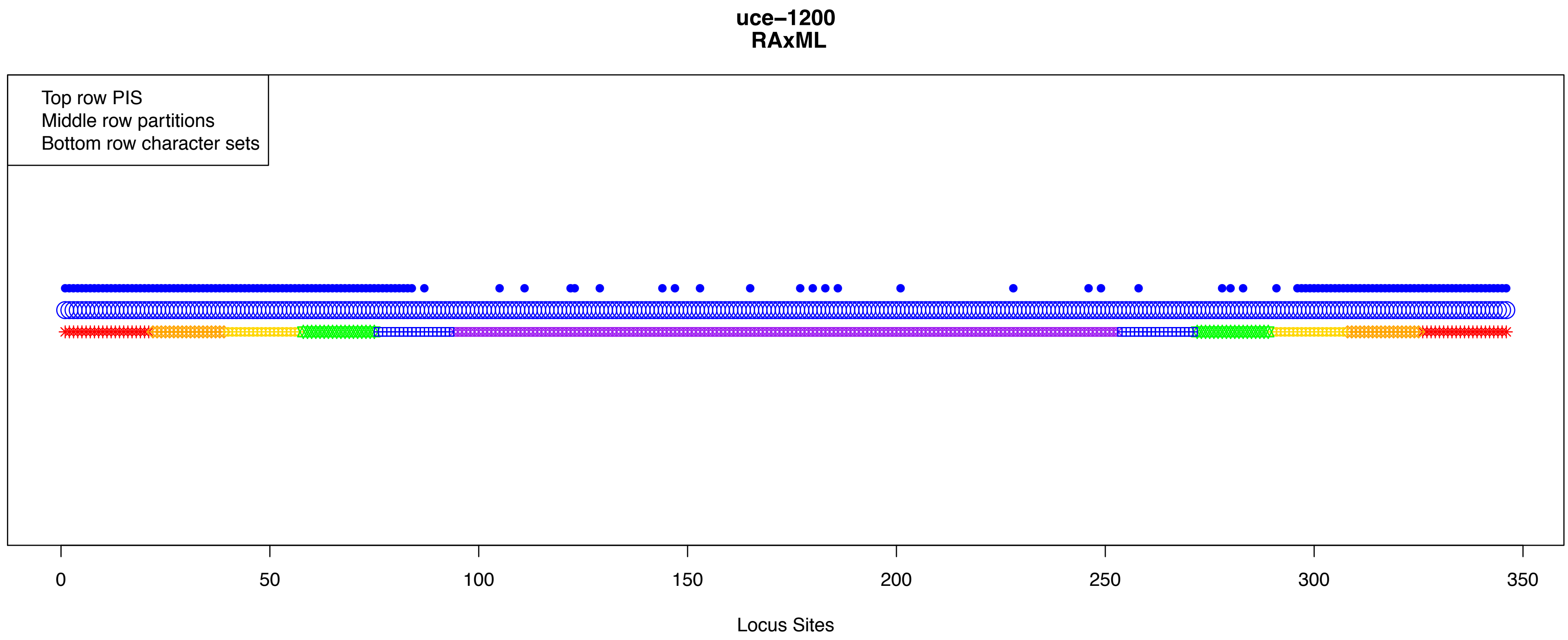
uce–1200
RAxML
Top row PIS
Middle row partitions
Bottom row character sets
0
50
100
150
200
250
300
350
Locus Sites

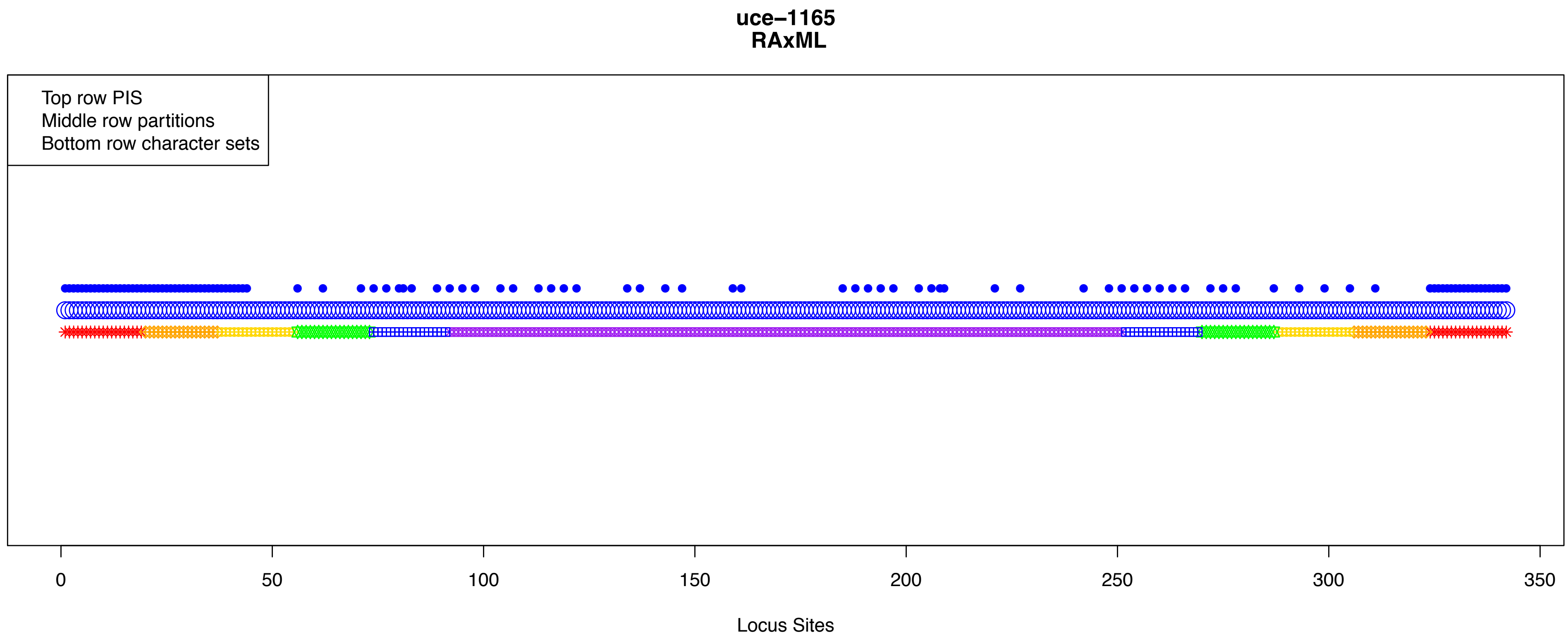

uce–1165
RAxML
Top row PIS
Middle row partitions
Bottom row character sets
0
50
100
150
200
250
300
350
Locus Sites

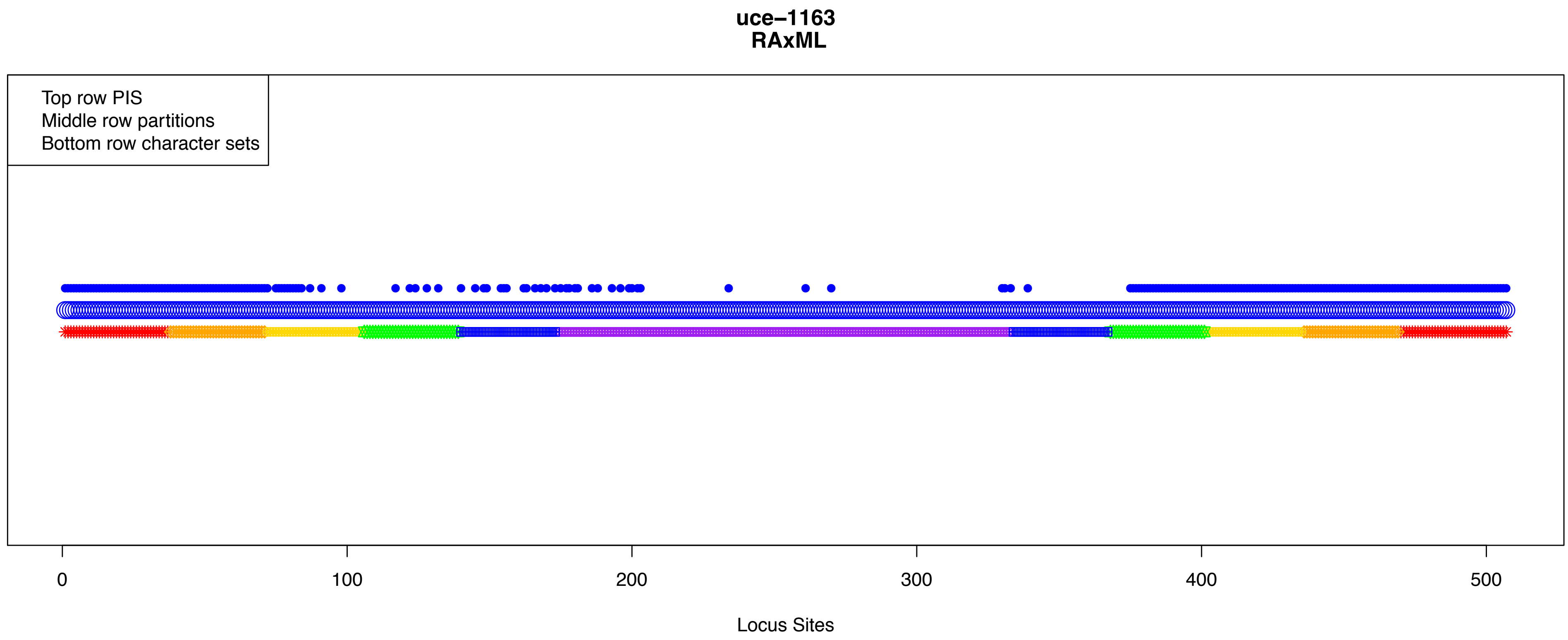

uce–1163
RAxML
Top row PIS
Middle row partitions
Bottom row character sets
0
100
200
300
400
500
Locus Sites

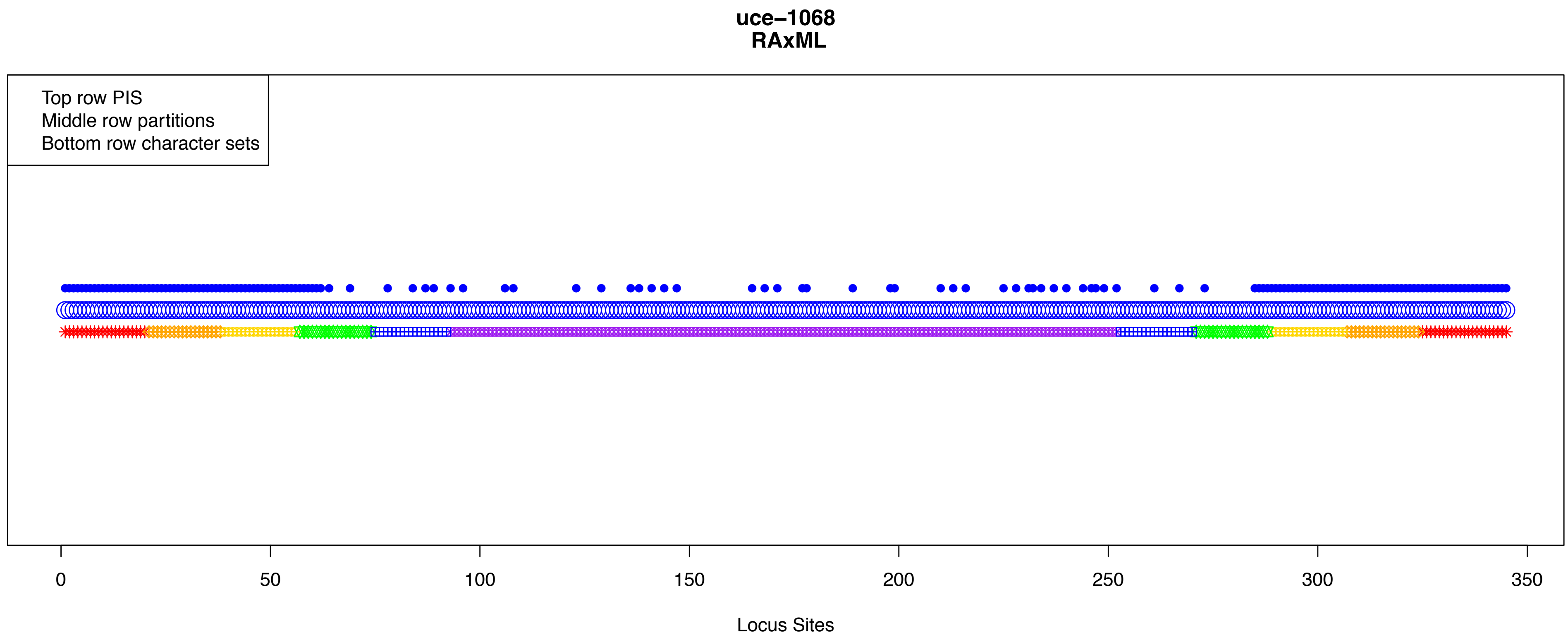
uce−1068
RAxML
Top row PIS
Middle row partitions
Bottom row character sets
0
50
100
150
200
250
300
350
Locus Sites

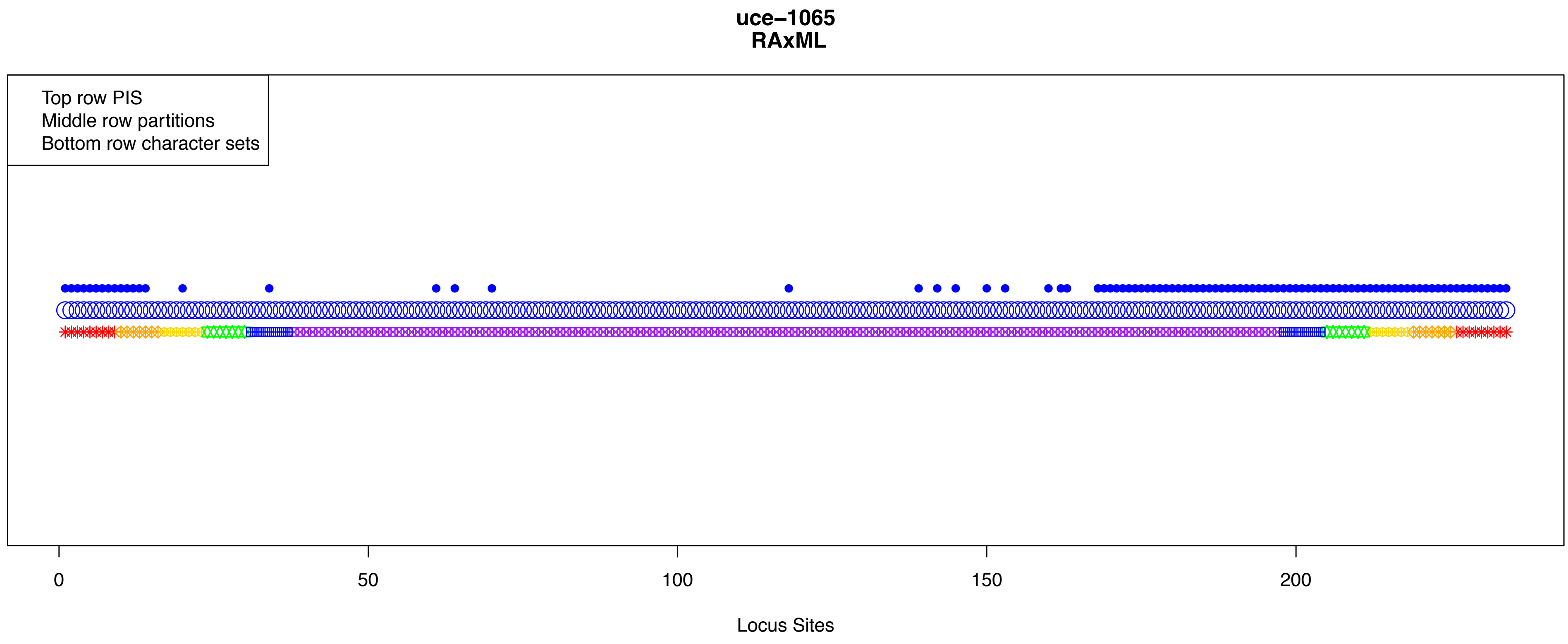
uce–1065
RAxML
Top row PIS
Middle row partitions
Bottom row character sets
0
50
100
150
200
Locus Sites

uce–1027
RAxML

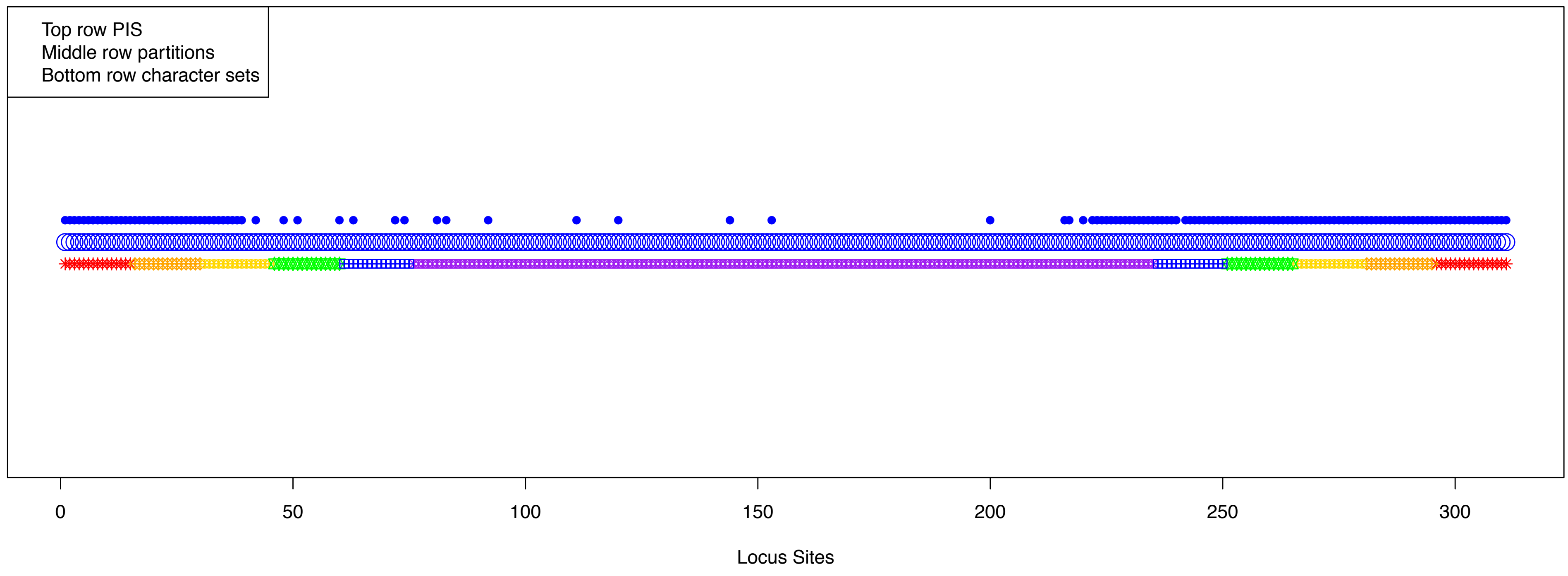

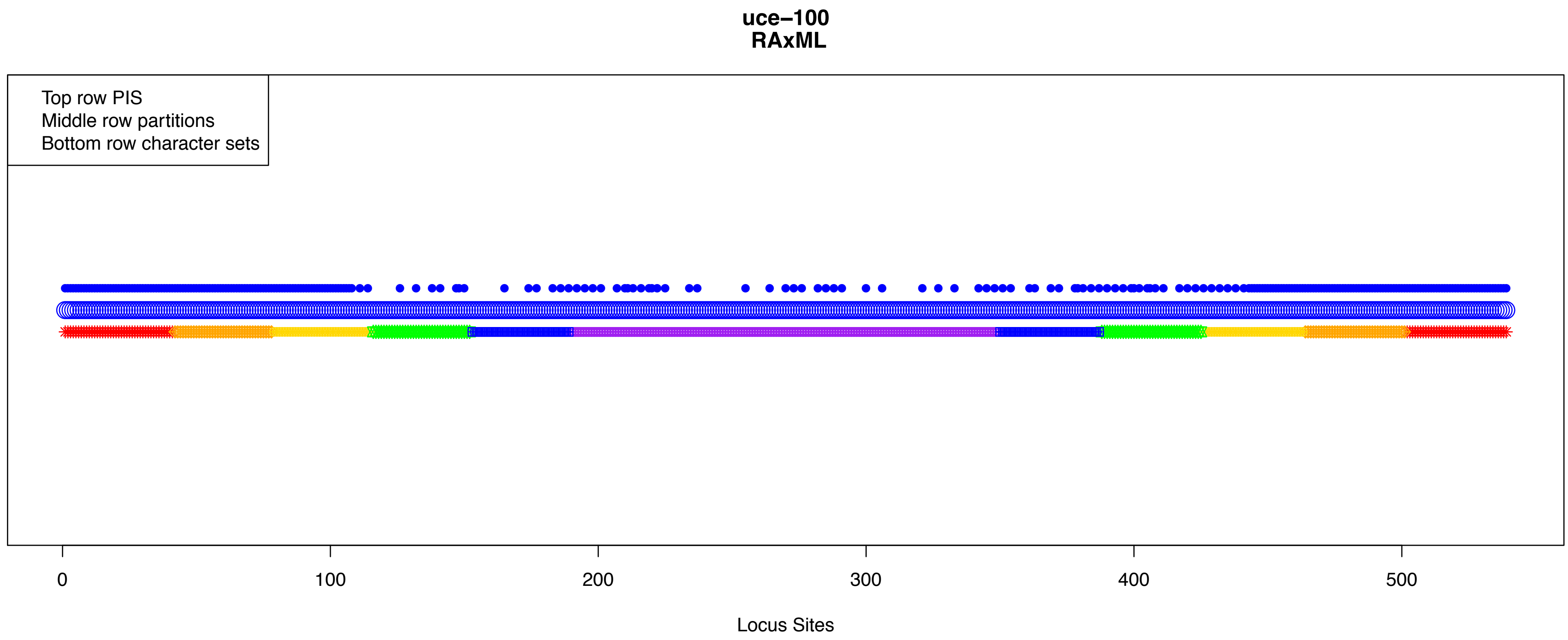
uce–100
RAxML
Top row PIS
Middle row partitions
Bottom row character sets
0
100
200
300
400
500
Locus Sites
