# Supplementary material for: Ultraconserved elements (UCEs) resolve the phylogeny of Australasian smurf-weevils
Source: PLoS One. 2017 Nov 22;12(11):e0188044. doi: 10.1371/journal.pone.0188044 (PMC5699822; doi:10.1371/journal.pone.0188044)
Supplement: S1 File — (ZIP) [file pone.0188044.s007.zip › Supplemental_Partition_Number_of_partitions_PIS_Charsets/partitions2-MrBayes.pdf]

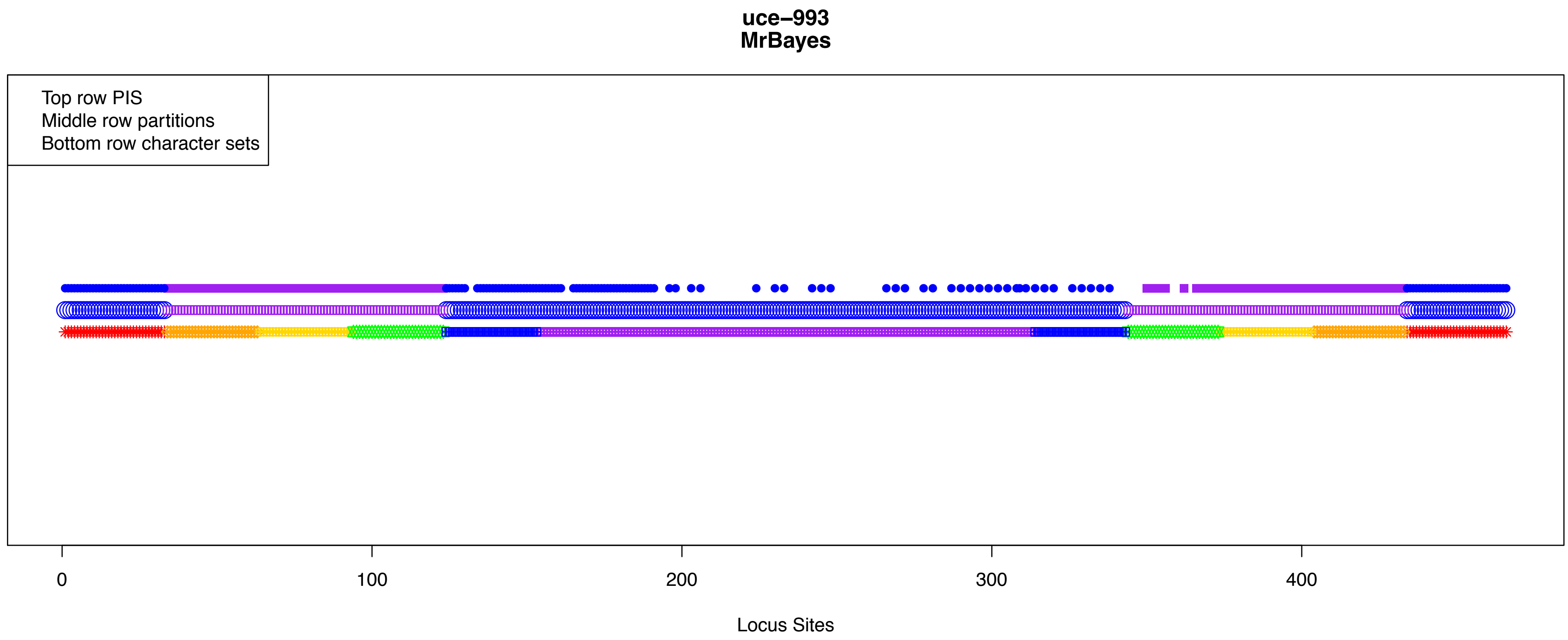
uce–993
MrBayes
Top row PIS
Middle row partitions
Bottom row character sets
0
100
200
300
400
Locus Sites

uce–976
MrBayes

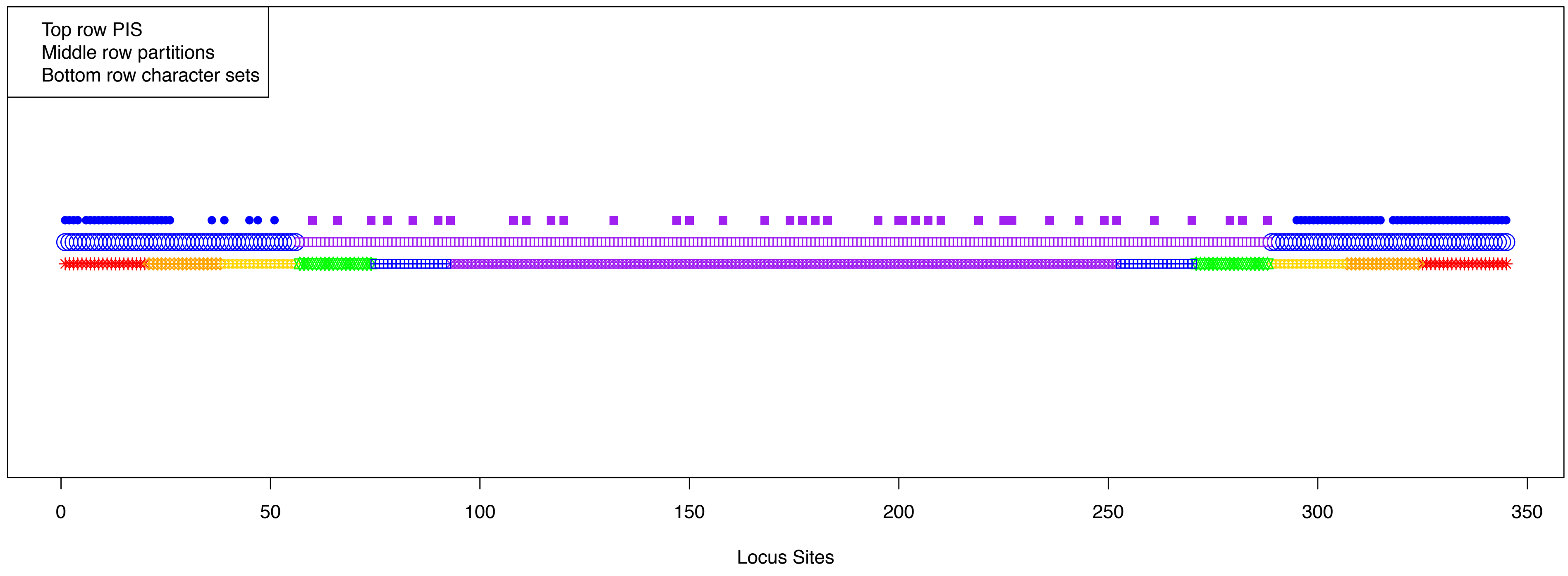

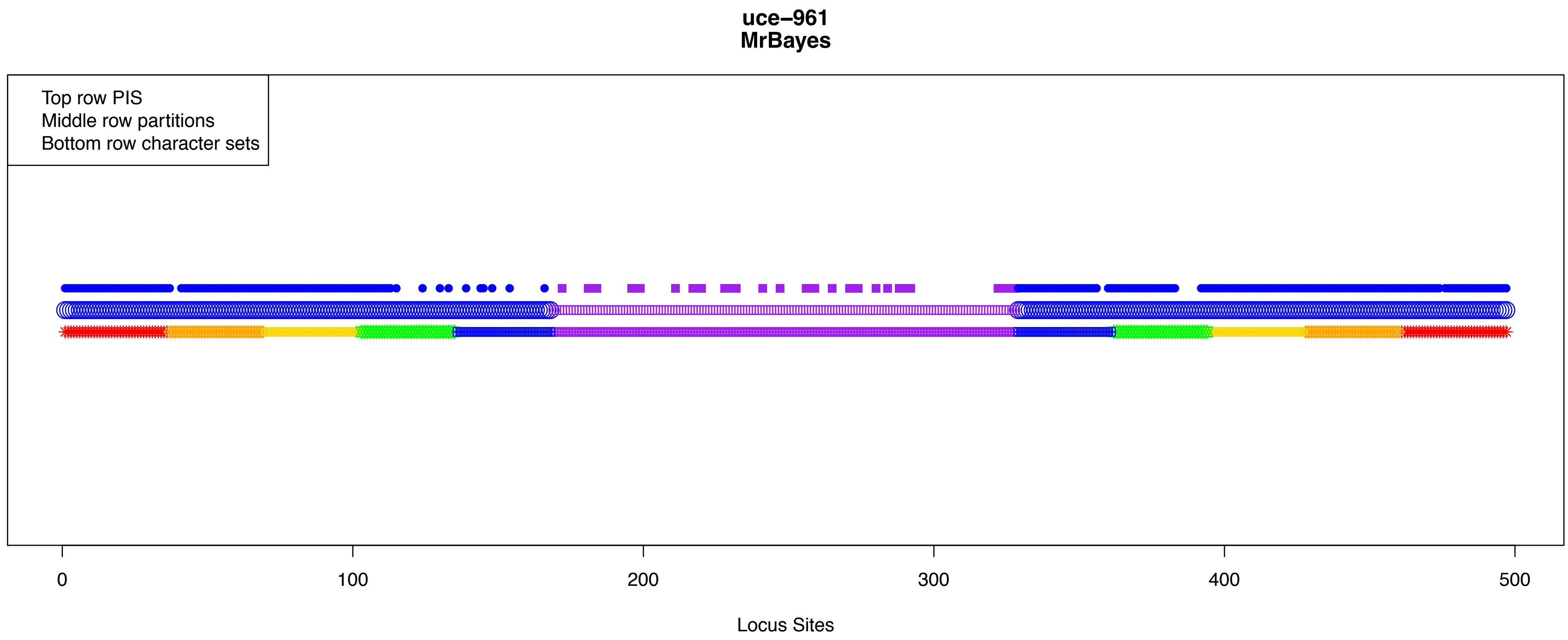

uce–961
MrBayes
Top row PIS
Middle row partitions
Bottom row character sets
0
100
200
300
400
500
Locus Sites

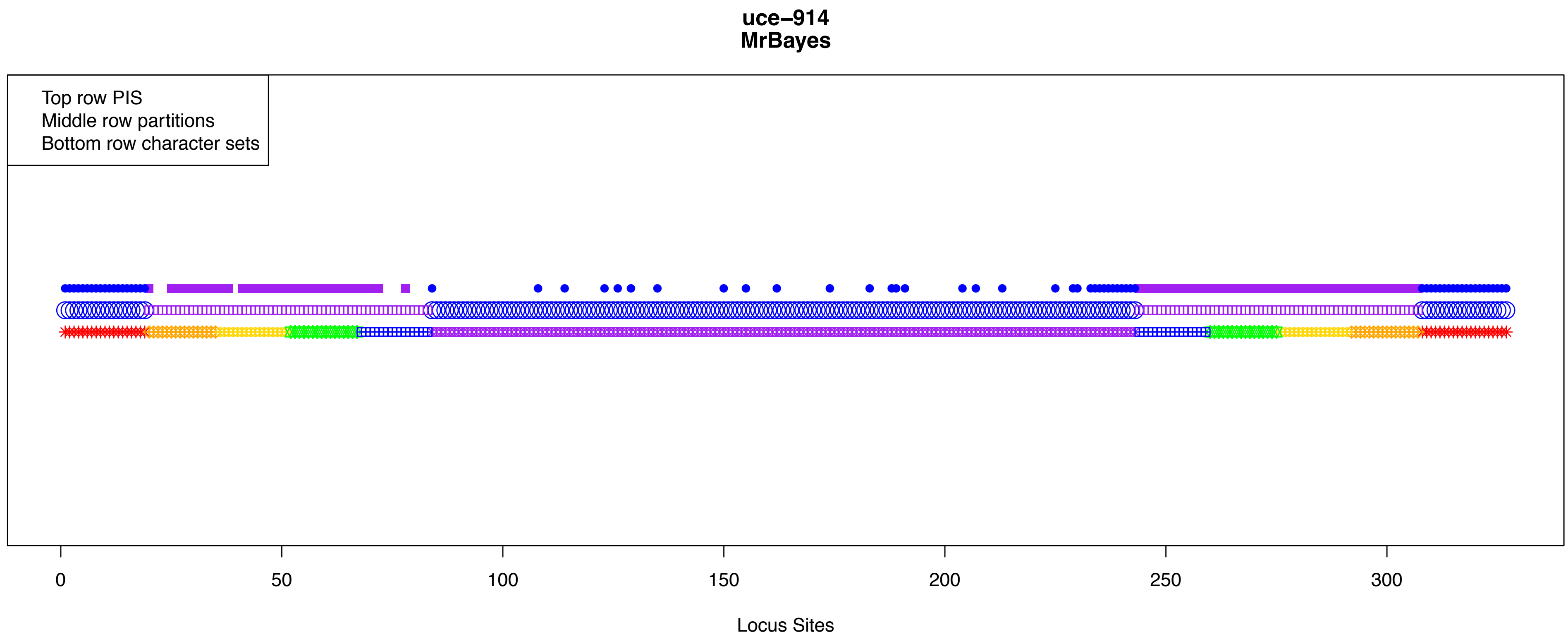

uce–914
MrBayes
Top row PIS
Middle row partitions
Bottom row character sets
0
50
100
150
200
250
300
Locus Sites

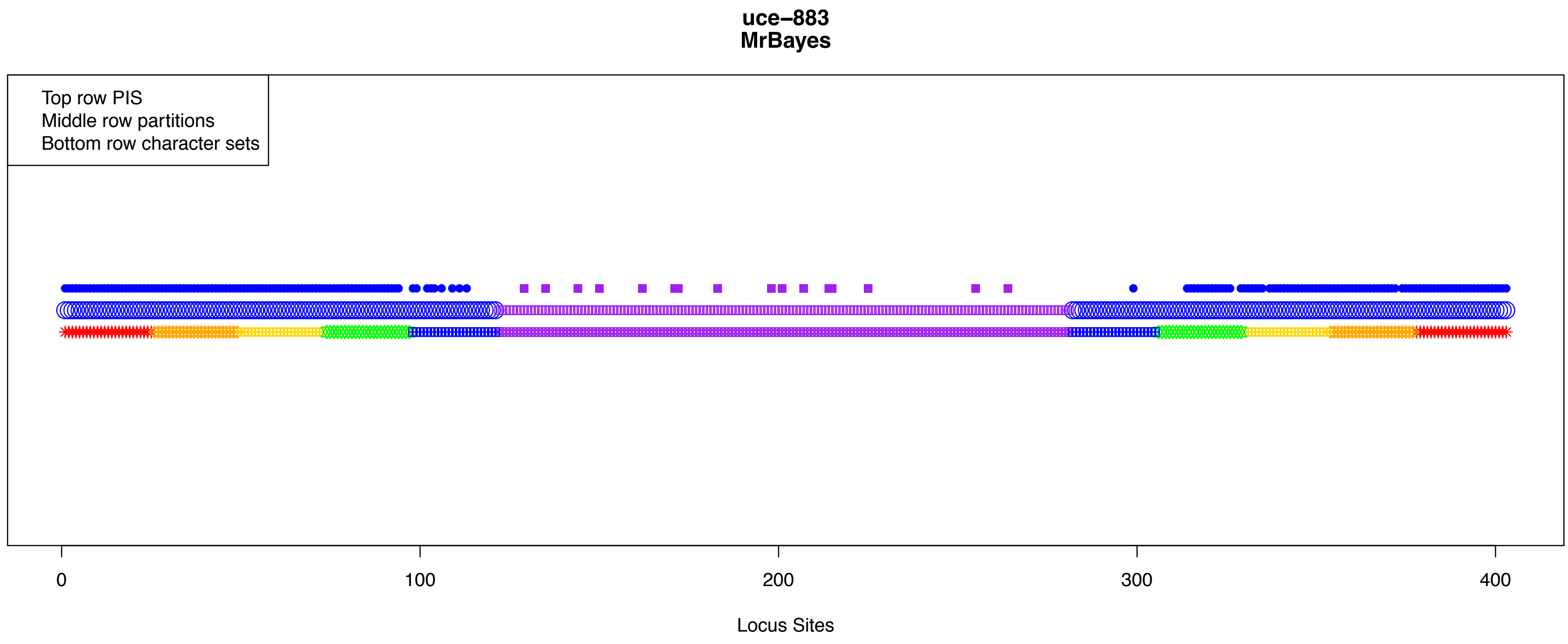
uce–883
MrBayes
Top row PIS
Middle row partitions
Bottom row character sets
0
100
200
300
400
Locus Sites

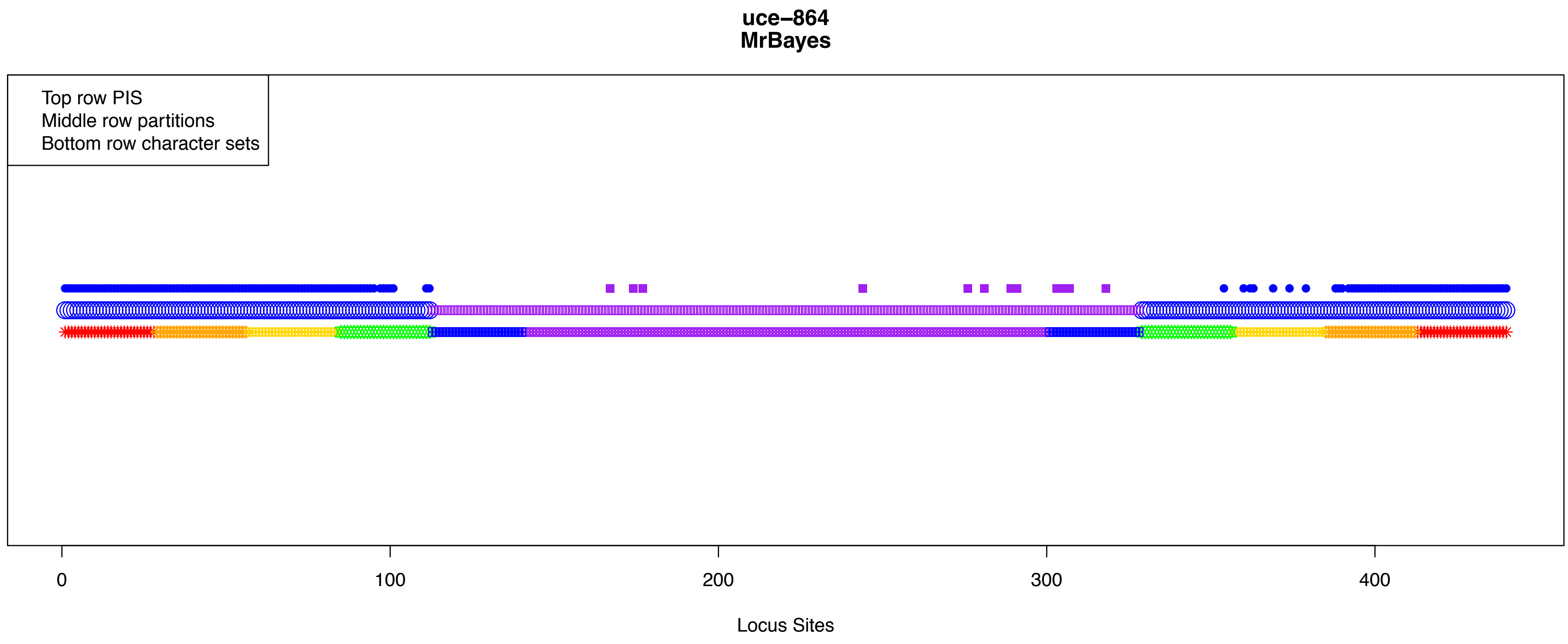
uce–864
MrBayes
Top row PIS
Middle row partitions
Bottom row character sets
0
100
200
300
400
Locus Sites

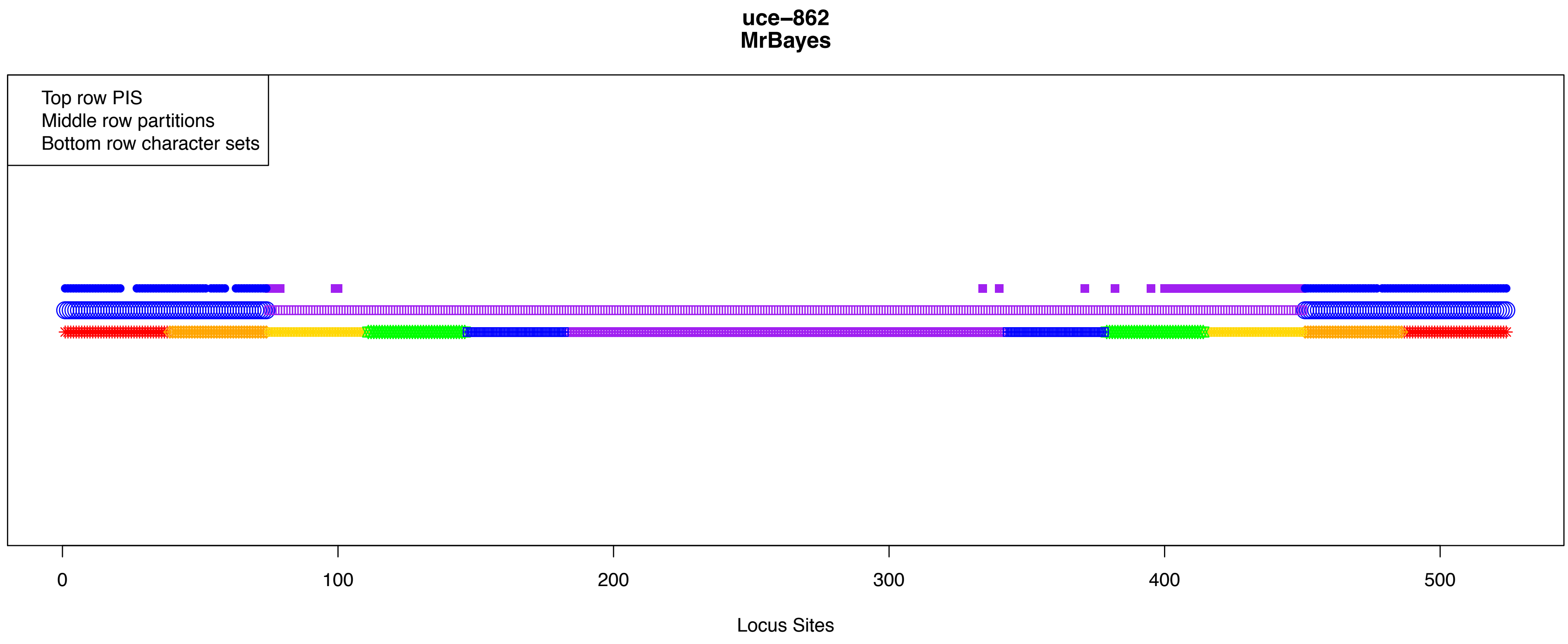

uce–862
MrBayes
Top row PIS
Middle row partitions
Bottom row character sets
0
100
200
300
400
500
Locus Sites

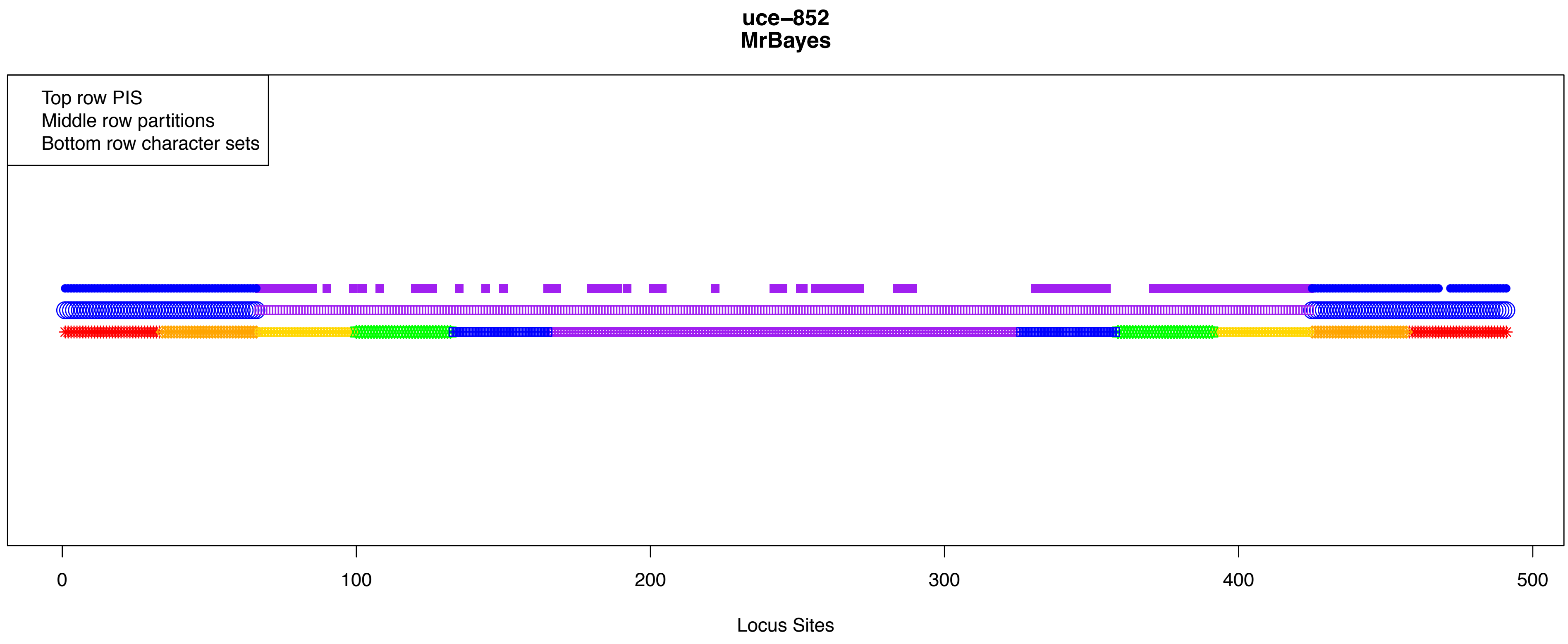
uce–852
MrBayes
Top row PIS
Middle row partitions
Bottom row character sets
0
100
200
300
400
500
Locus Sites

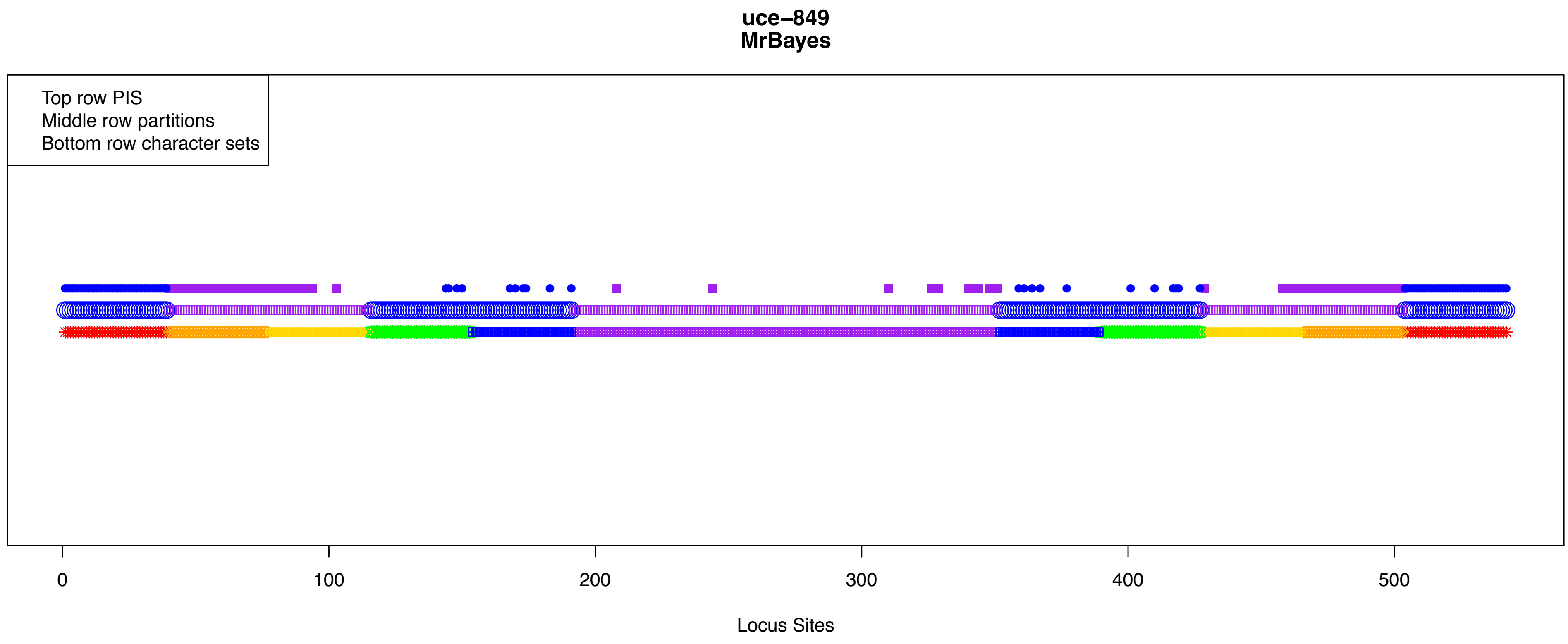

uce–849
MrBayes
Top row PIS
Middle row partitions
Bottom row character sets
0
100
200
300
400
500
Locus Sites

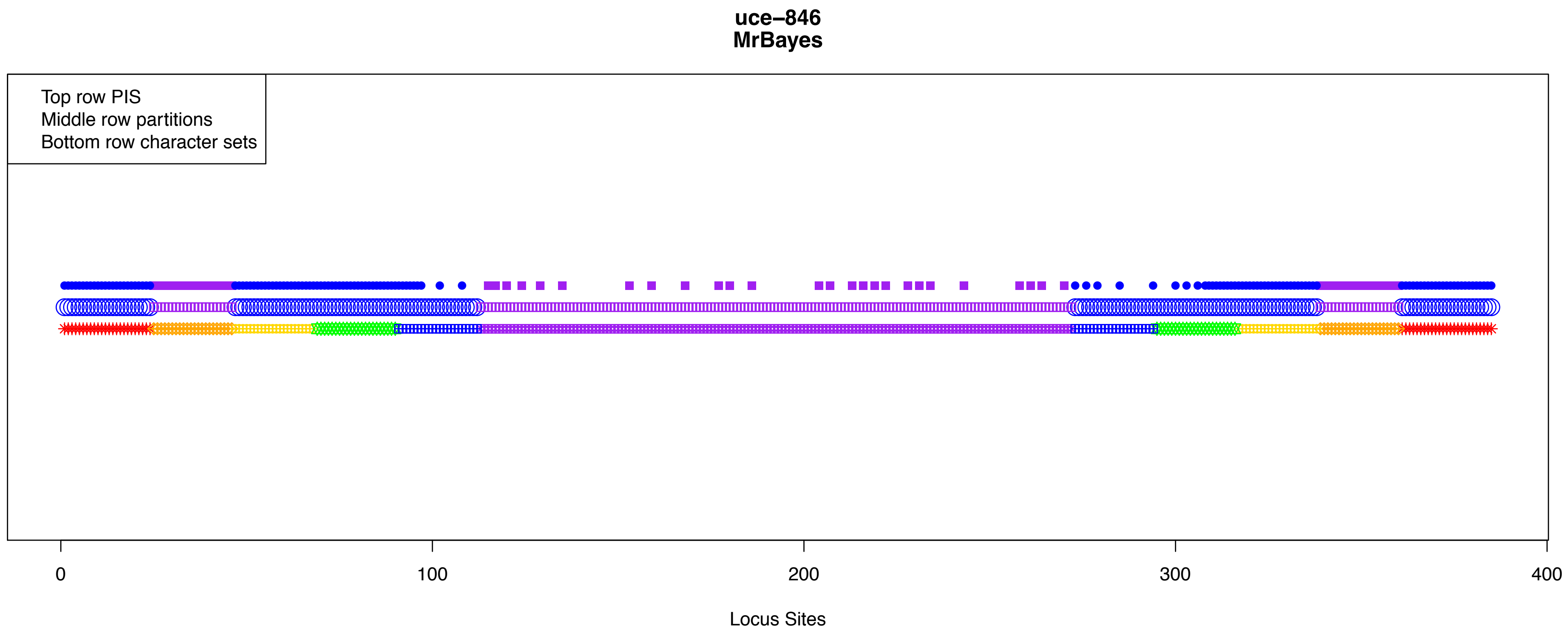

uce–846
MrBayes
Top row PIS
Middle row partitions
Bottom row character sets
0
100
200
300
400
Locus Sites

uce–843
MrBayes

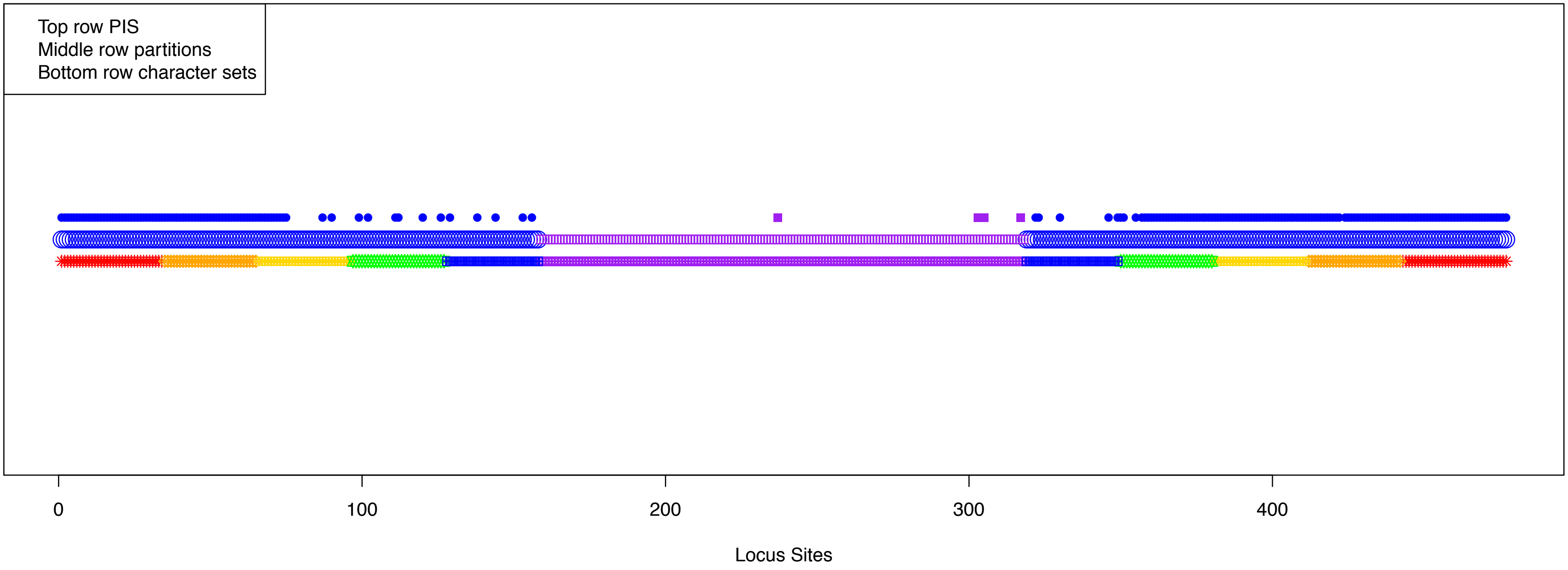

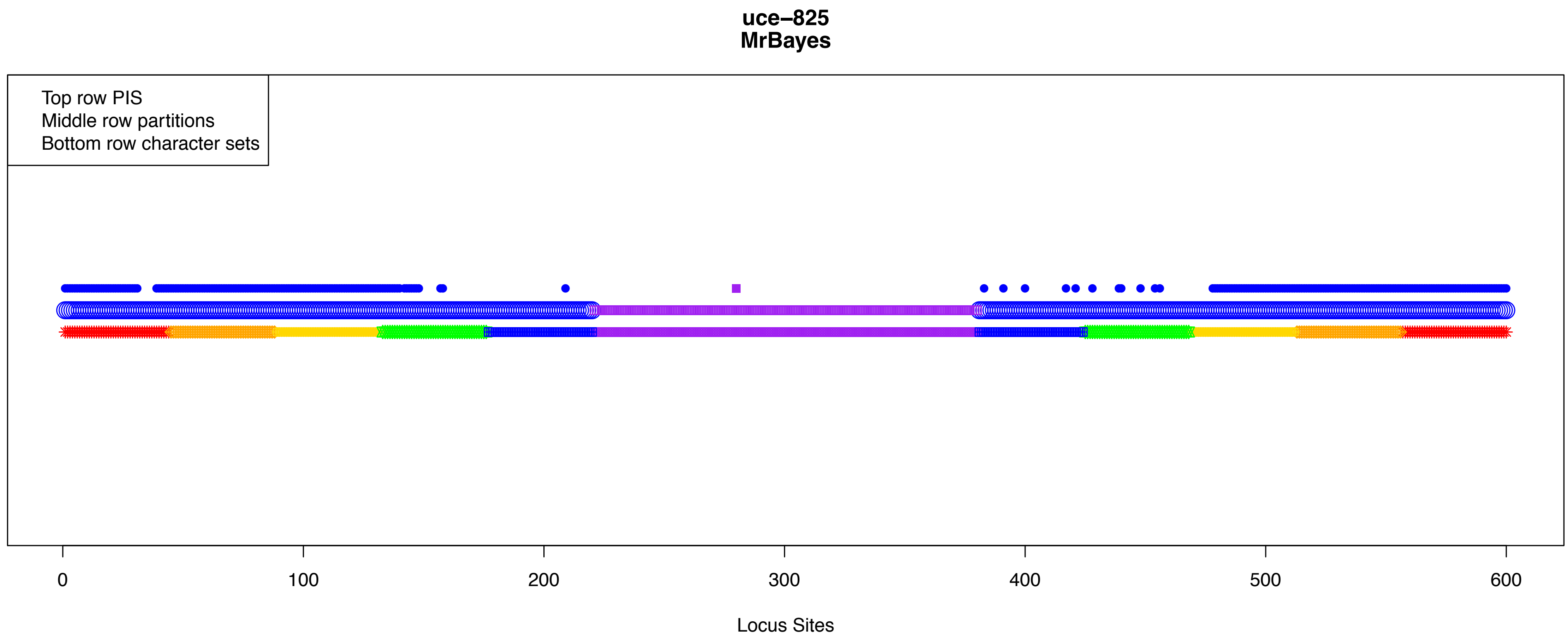
uce–825
MrBayes
Top row PIS
Middle row partitions
Bottom row character sets
0
100
200
300
400
500
600
Locus Sites

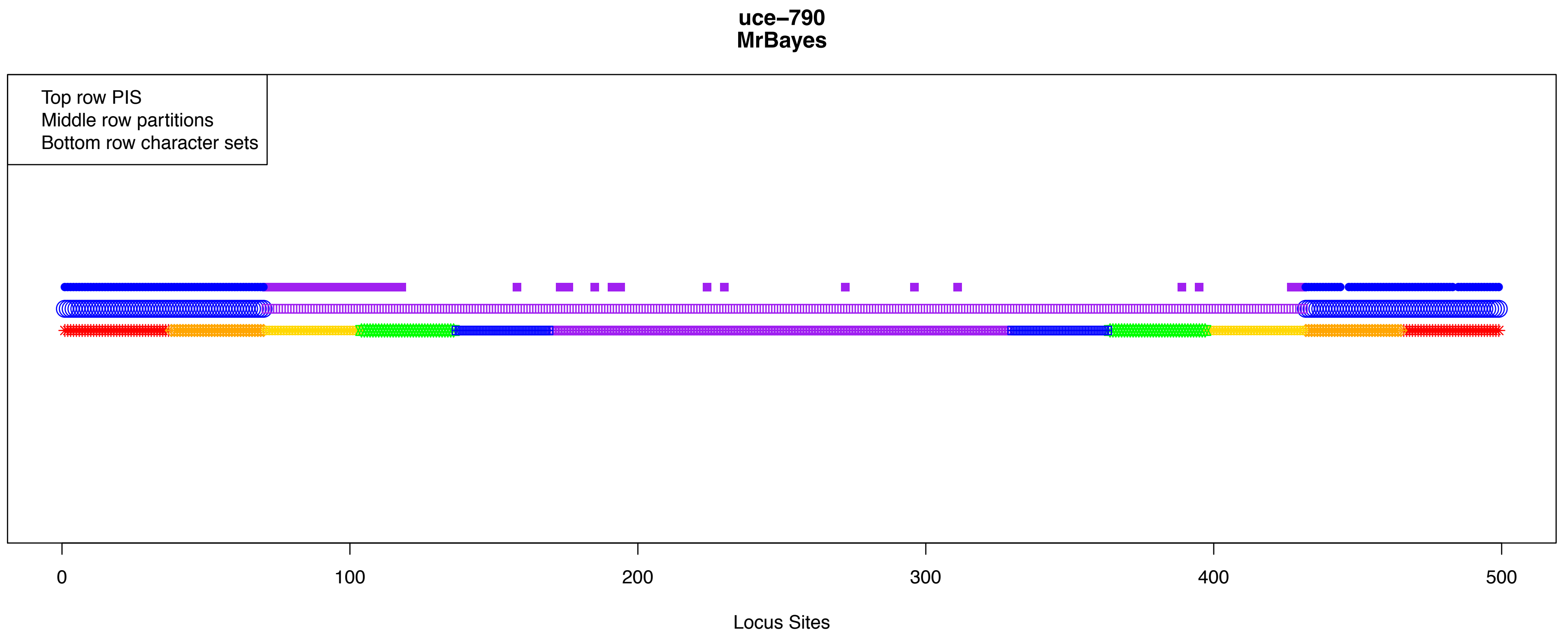
uce–790
MrBayes
Top row PIS
Middle row partitions
Bottom row character sets
0
100
200
300
400
500
Locus Sites

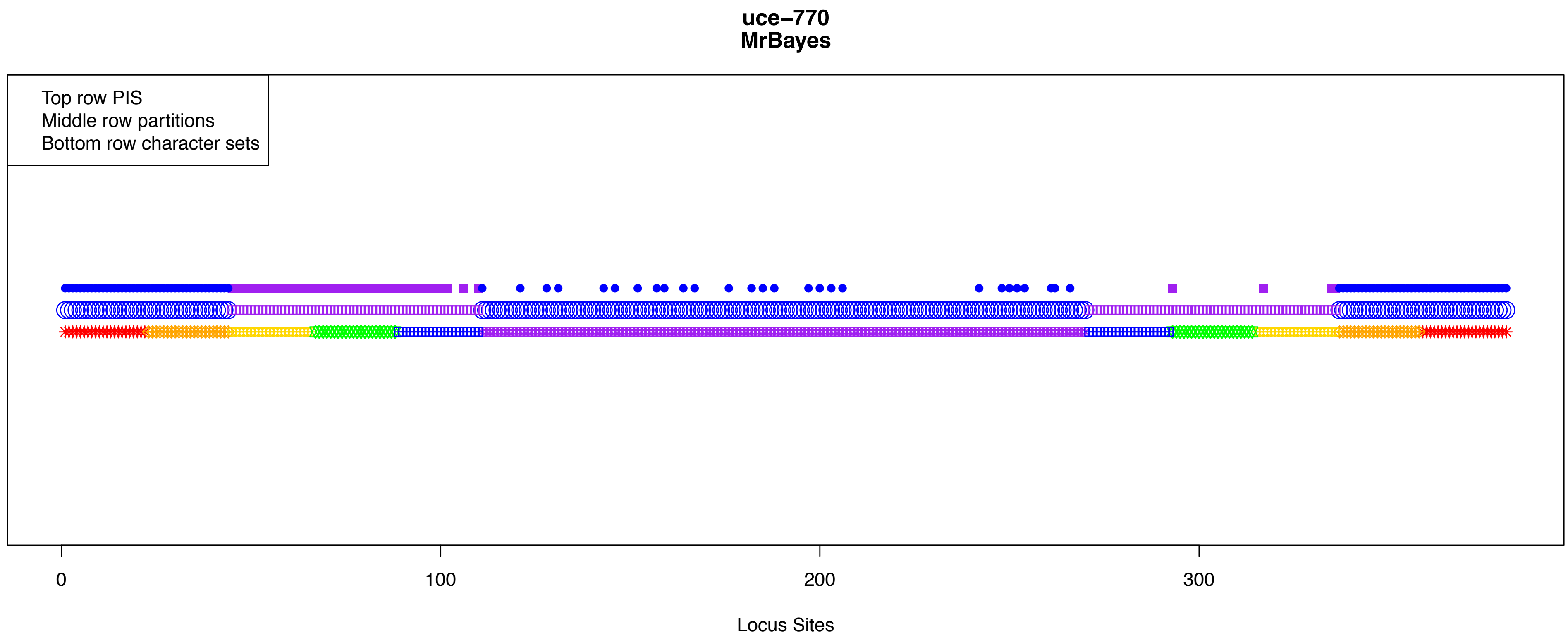
uce–770
MrBayes
Top row PIS
Middle row partitions
Bottom row character sets
0
100
200
300
Locus Sites

uce–760
MrBayes

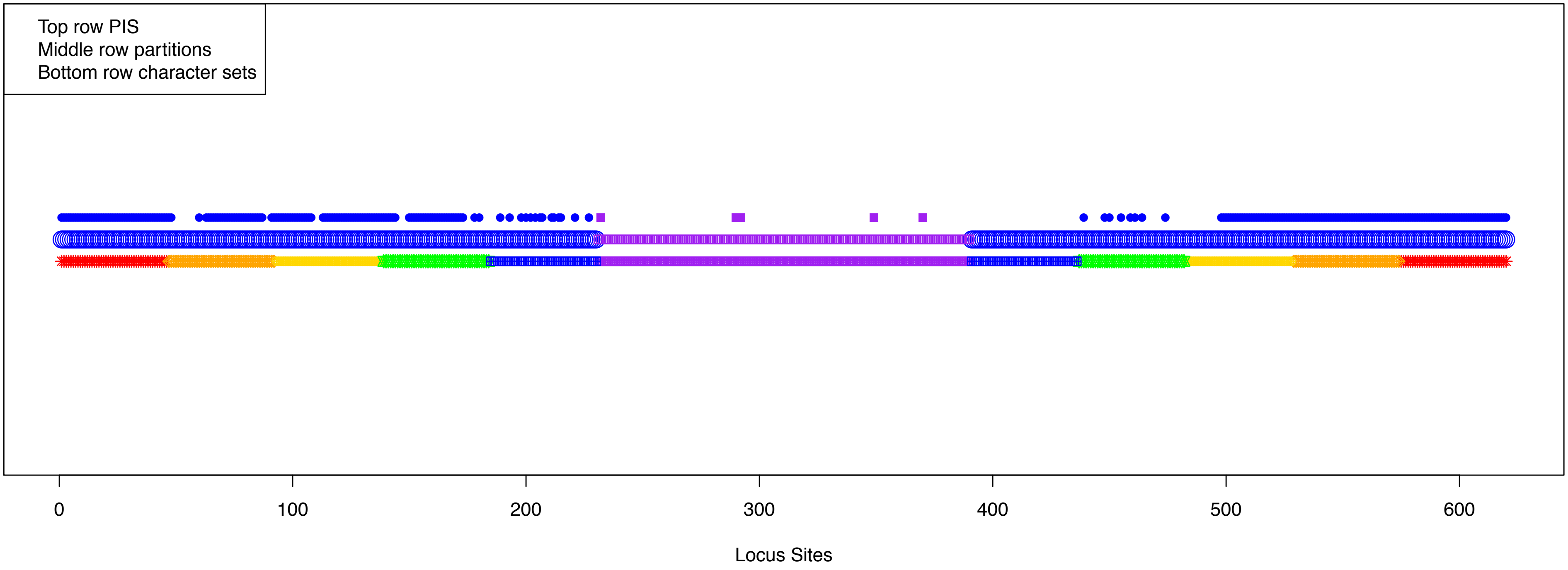

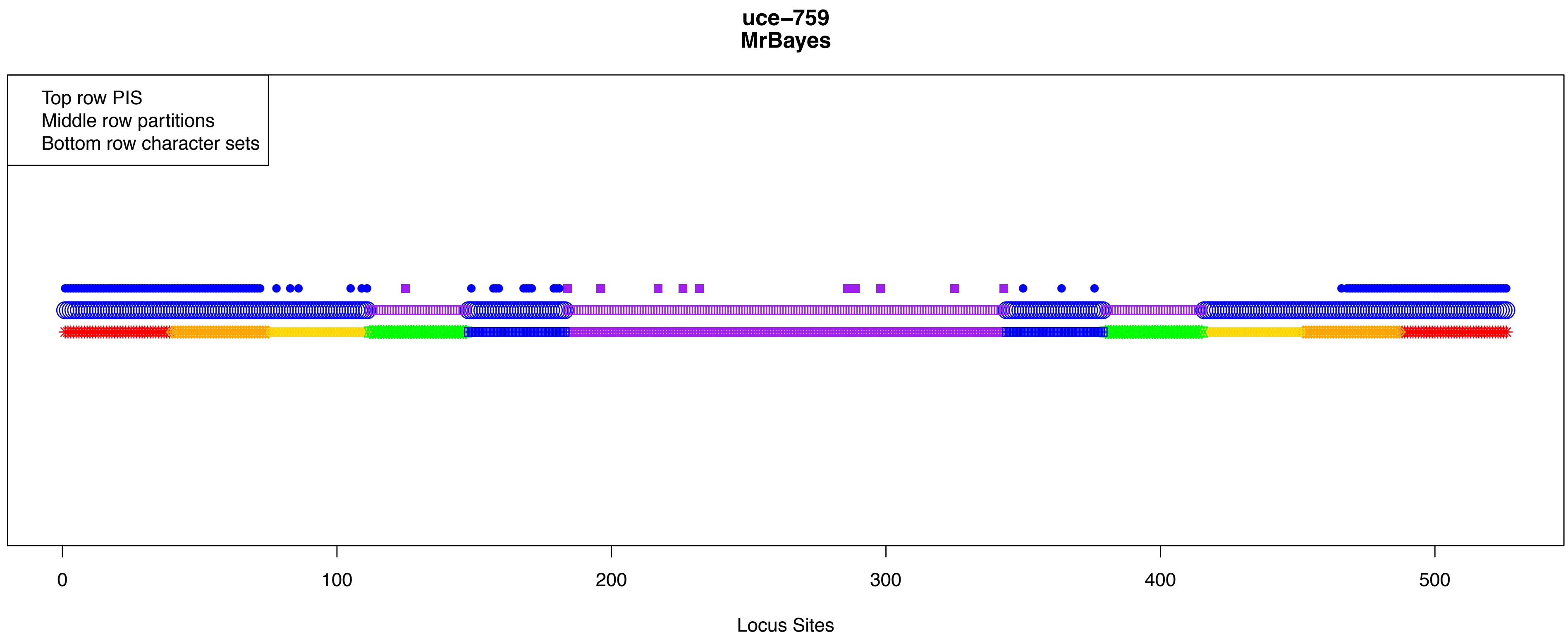
uce–759
MrBayes
Top row PIS
Middle row partitions
Bottom row character sets
0
100
200
300
400
500
Locus Sites

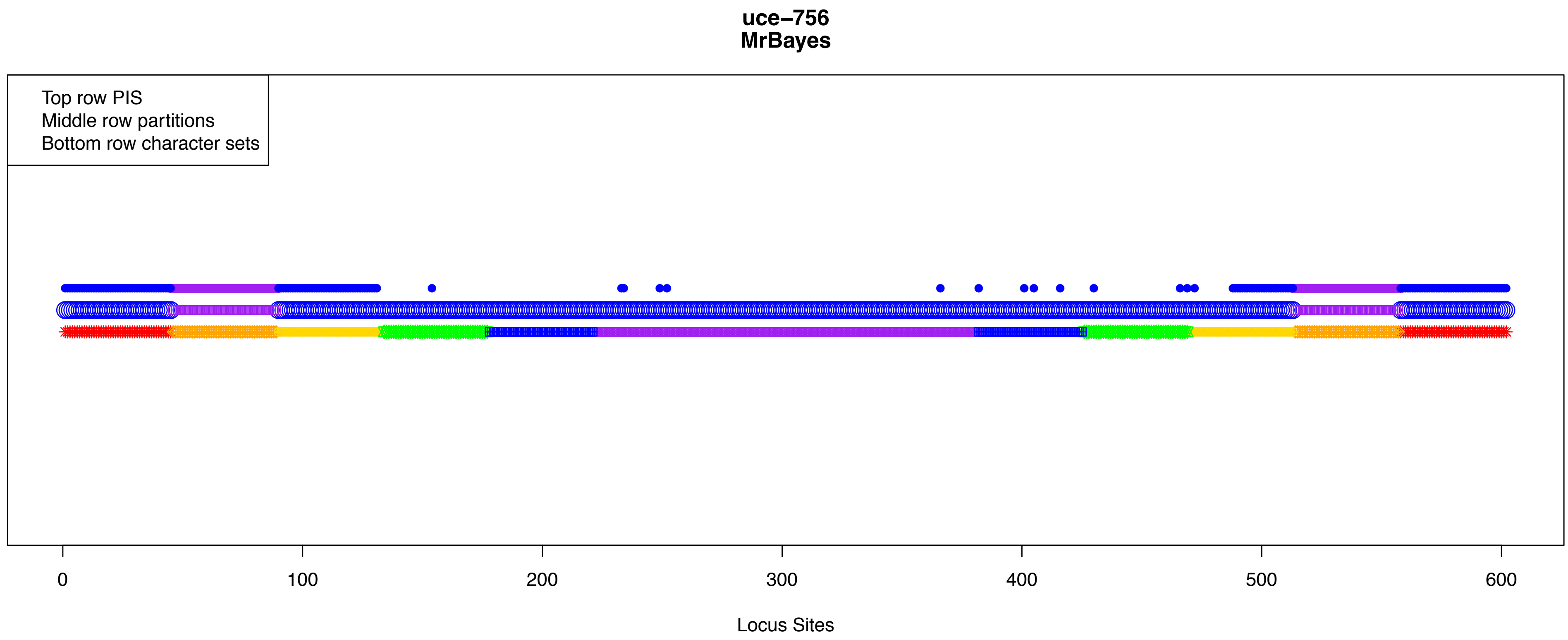

uce–756
MrBayes
Top row PIS
Middle row partitions
Bottom row character sets
0
100
200
300
400
500
600
Locus Sites

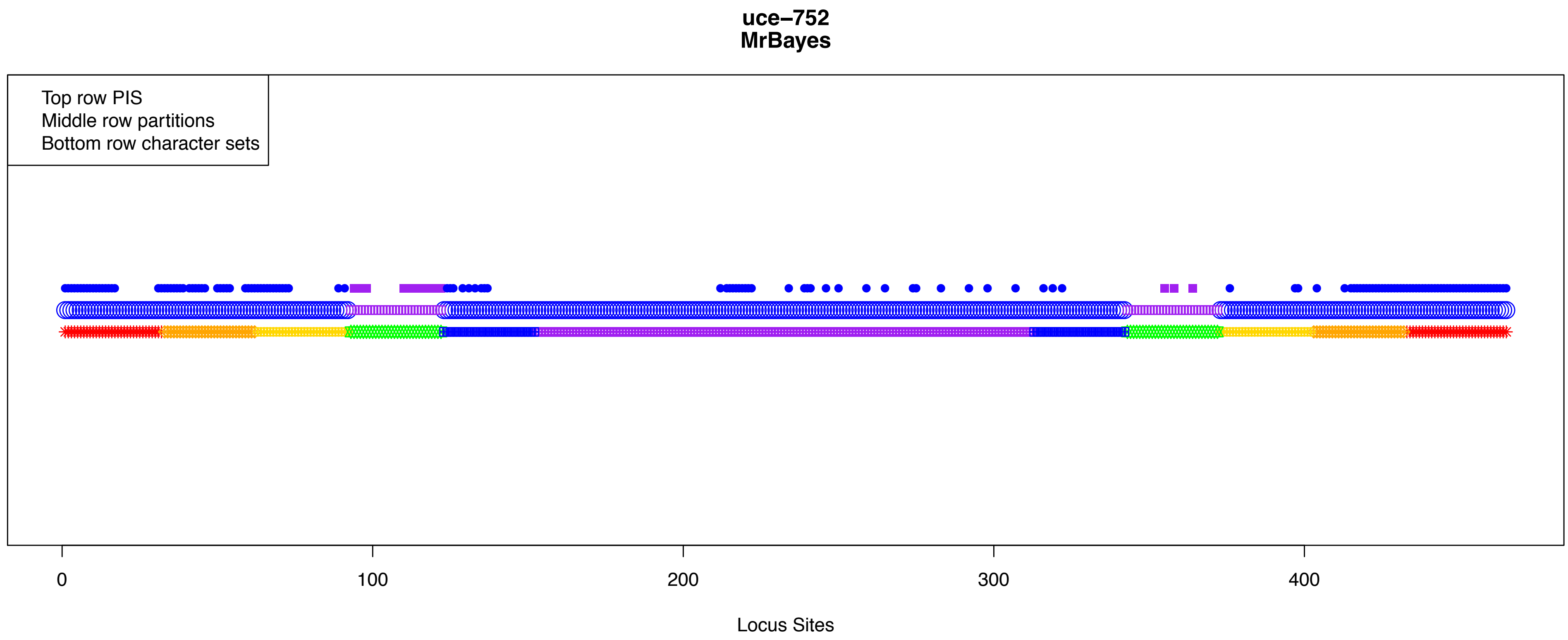
uce–752
MrBayes
Top row PIS
Middle row partitions
Bottom row character sets
0
100
200
300
400
Locus Sites

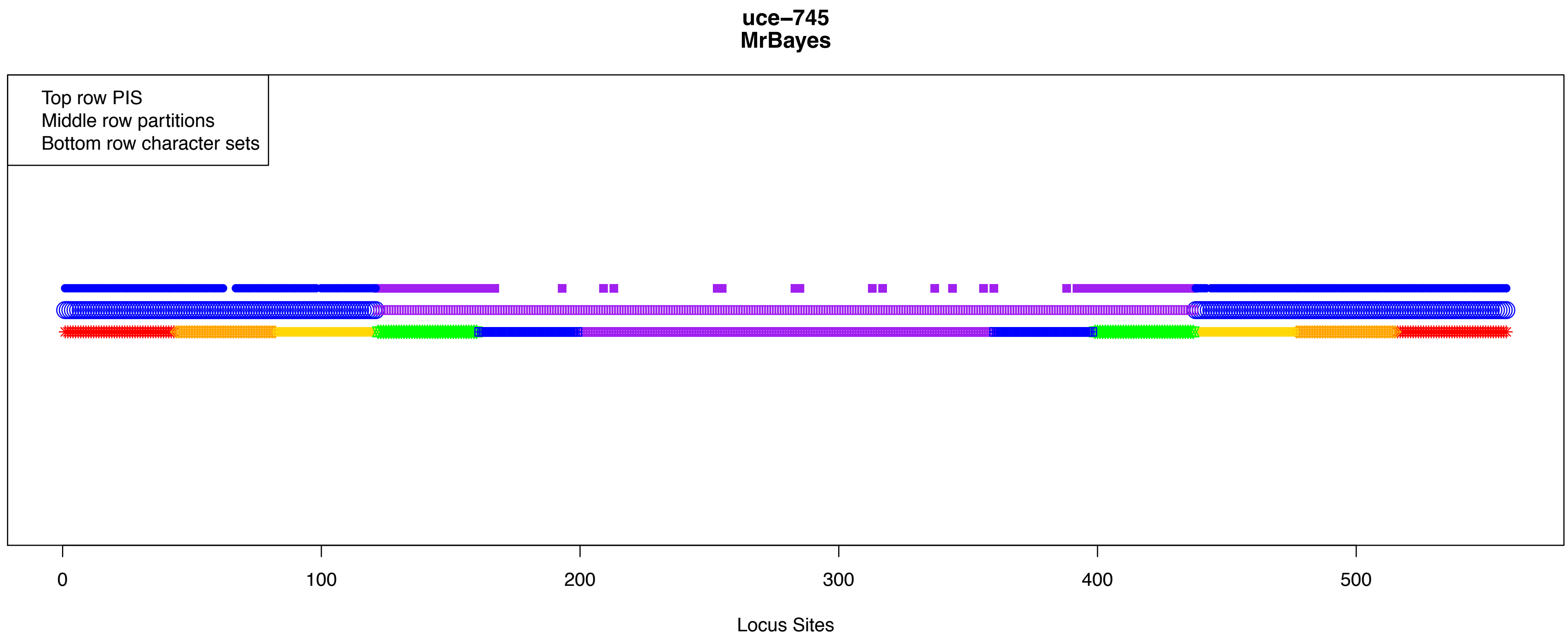
uce–745
MrBayes
Top row PIS
Middle row partitions
Bottom row character sets
0
100
200
300
400
500
Locus Sites

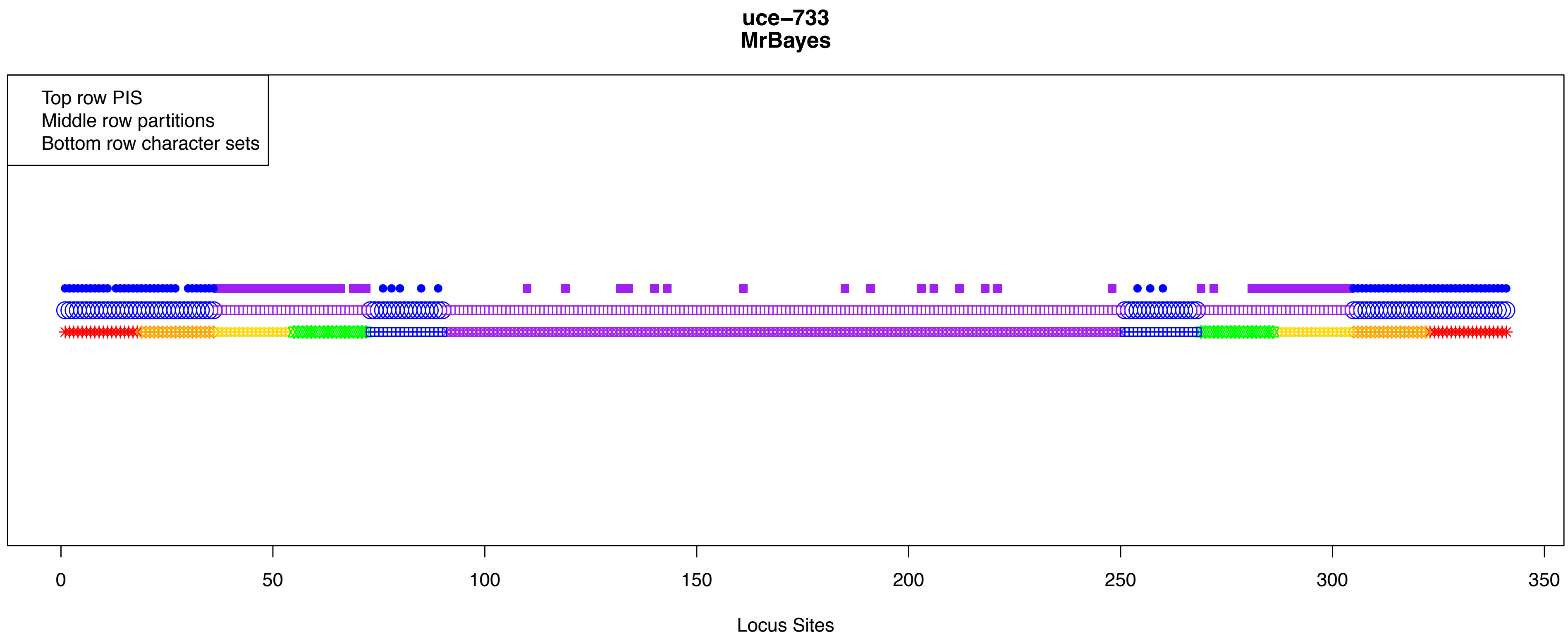

uce–733
MrBayes
Top row PIS
Middle row partitions
Bottom row character sets
0
50
100
150
200
250
300
350
Locus Sites

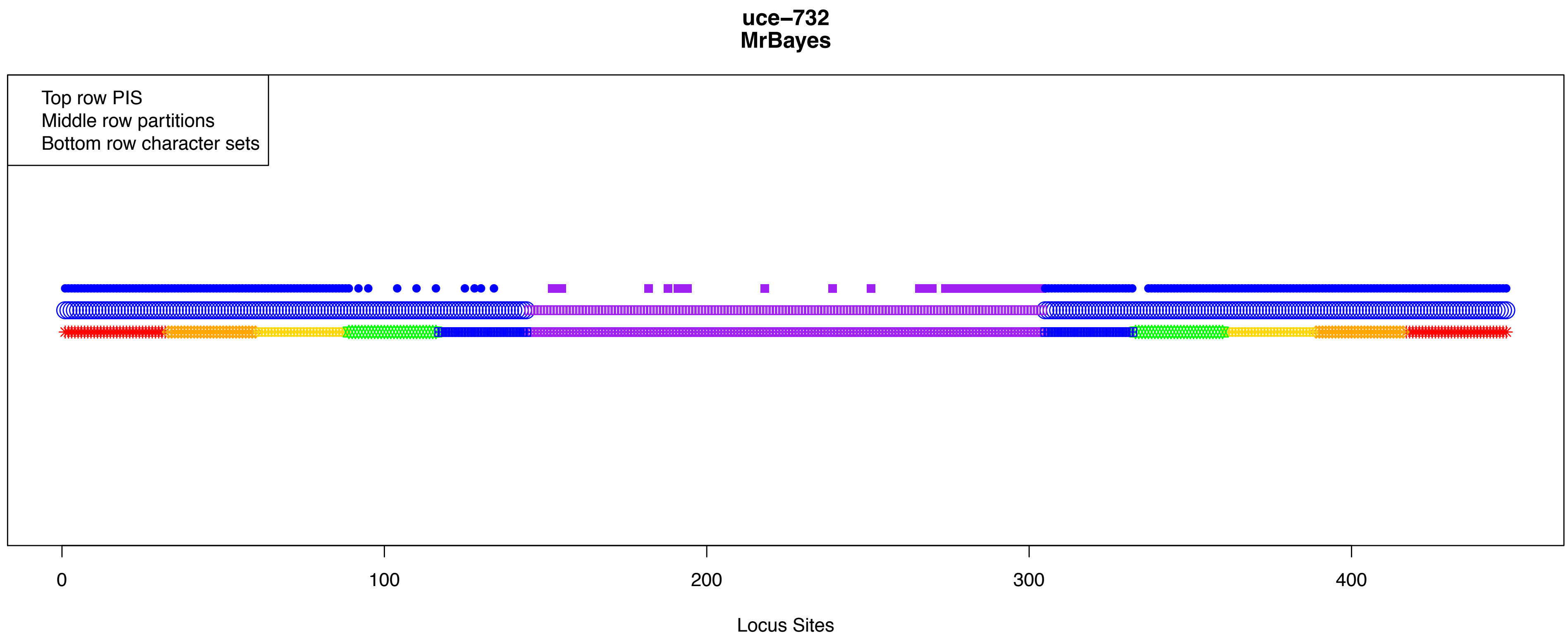
uce–732
MrBayes
Top row PIS
Middle row partitions
Bottom row character sets
0
100
200
300
400
Locus Sites

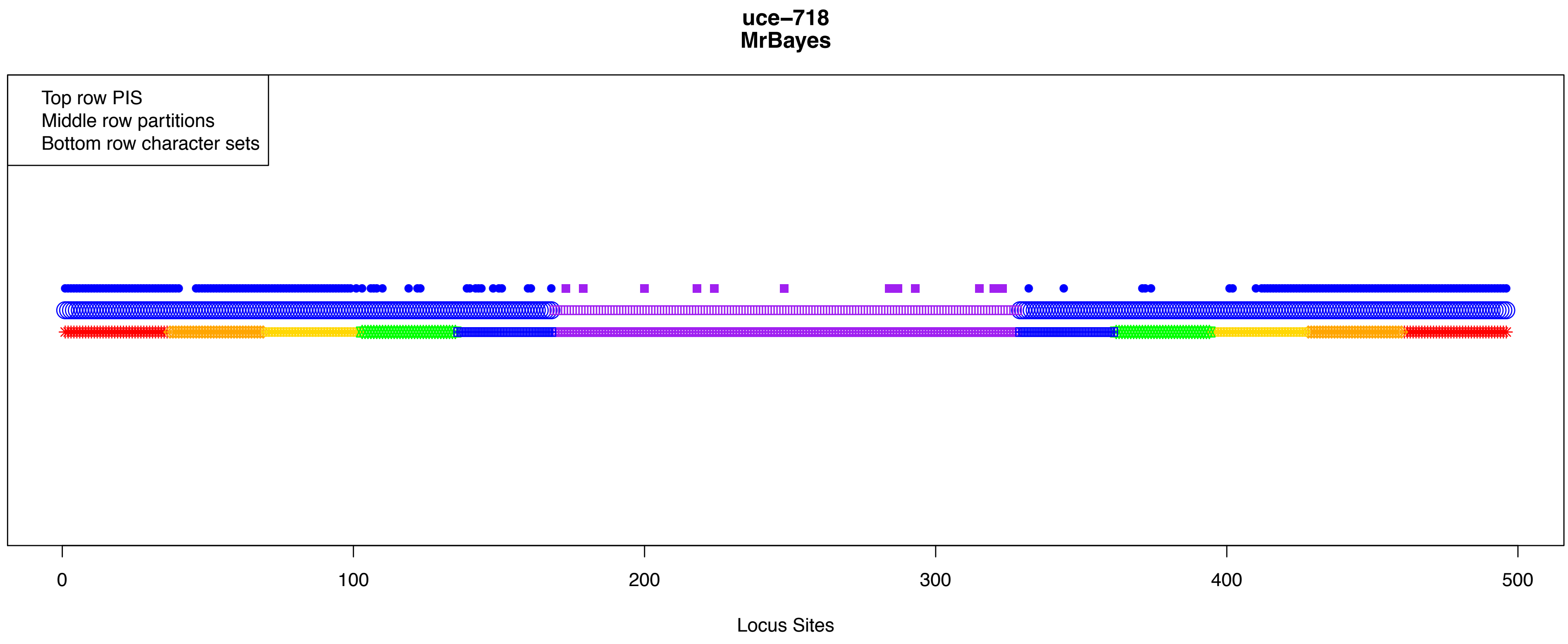
uce–718
MrBayes
Top row PIS
Middle row partitions
Bottom row character sets
0
100
200
300
400
500
Locus Sites

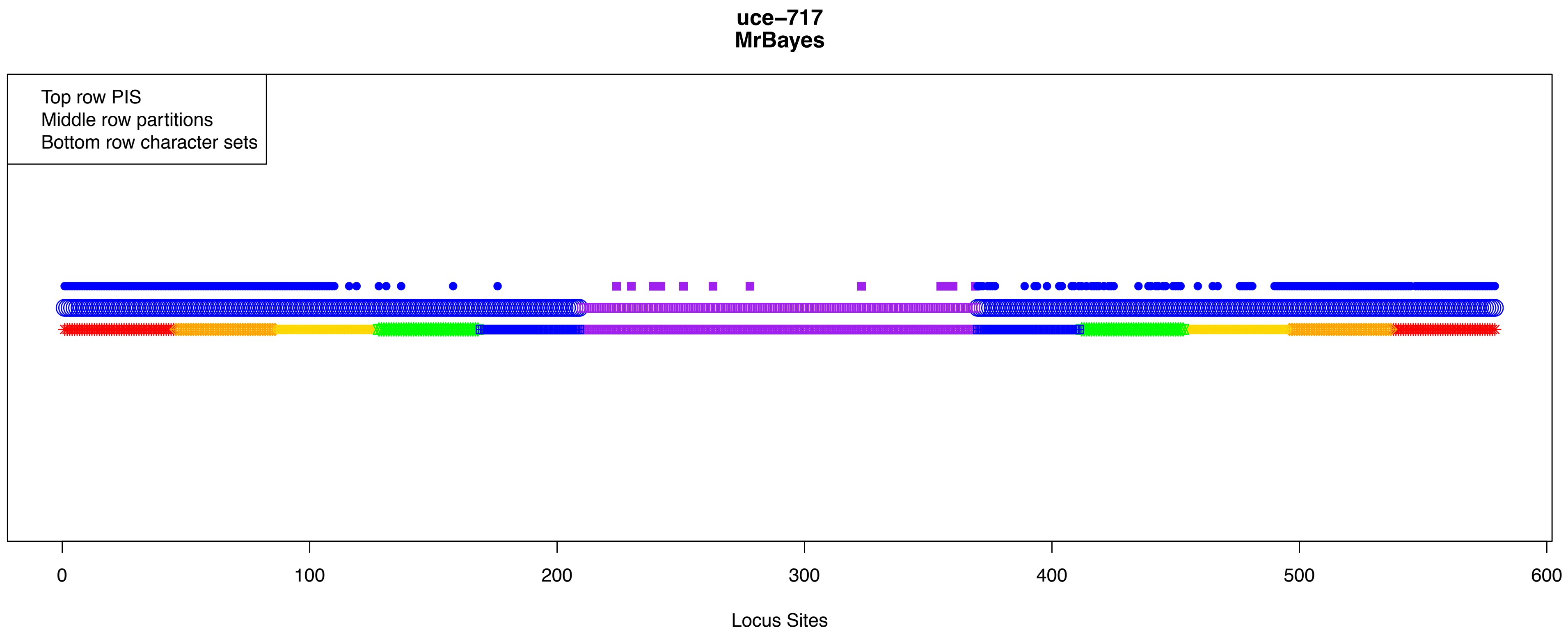
uce–717
MrBayes
Top row PIS
Middle row partitions
Bottom row character sets
0
100
200
300
400
500
600
Locus Sites

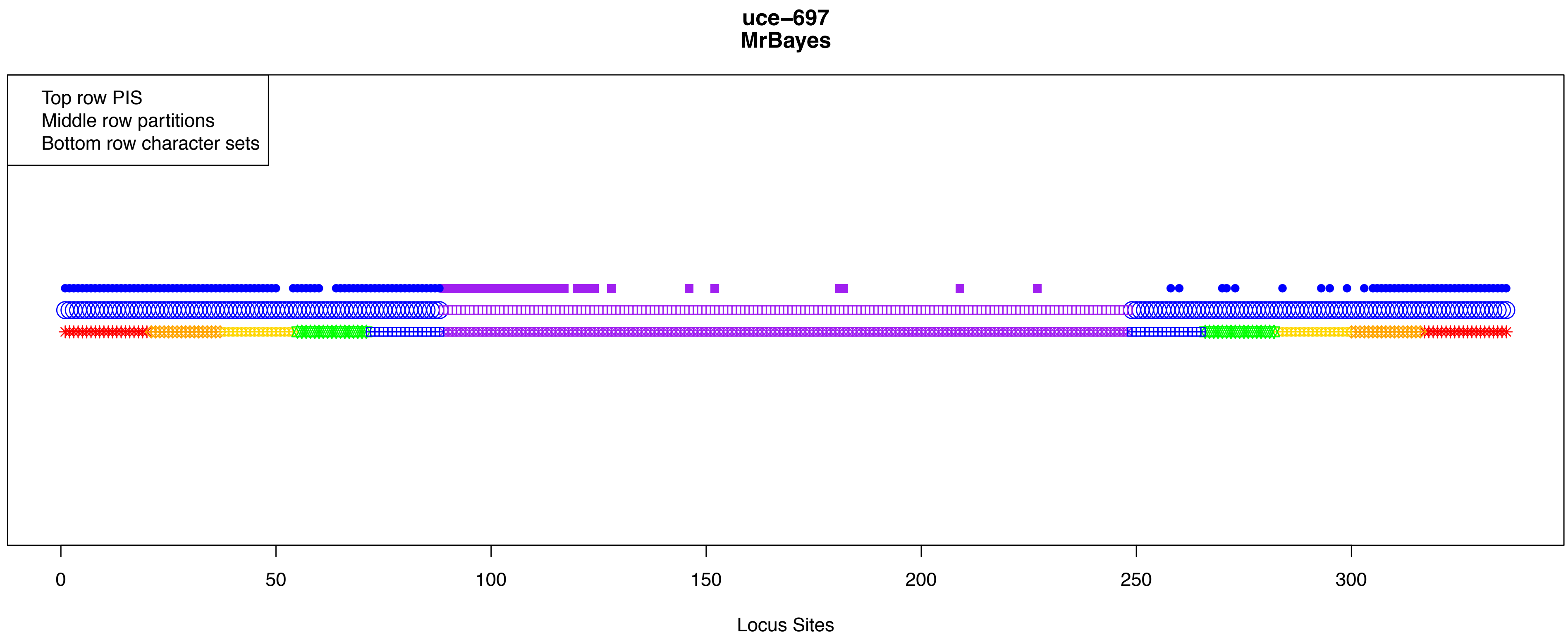

uce–697
MrBayes
Top row PIS
Middle row partitions
Bottom row character sets
0
50
100
150
200
250
300
Locus Sites

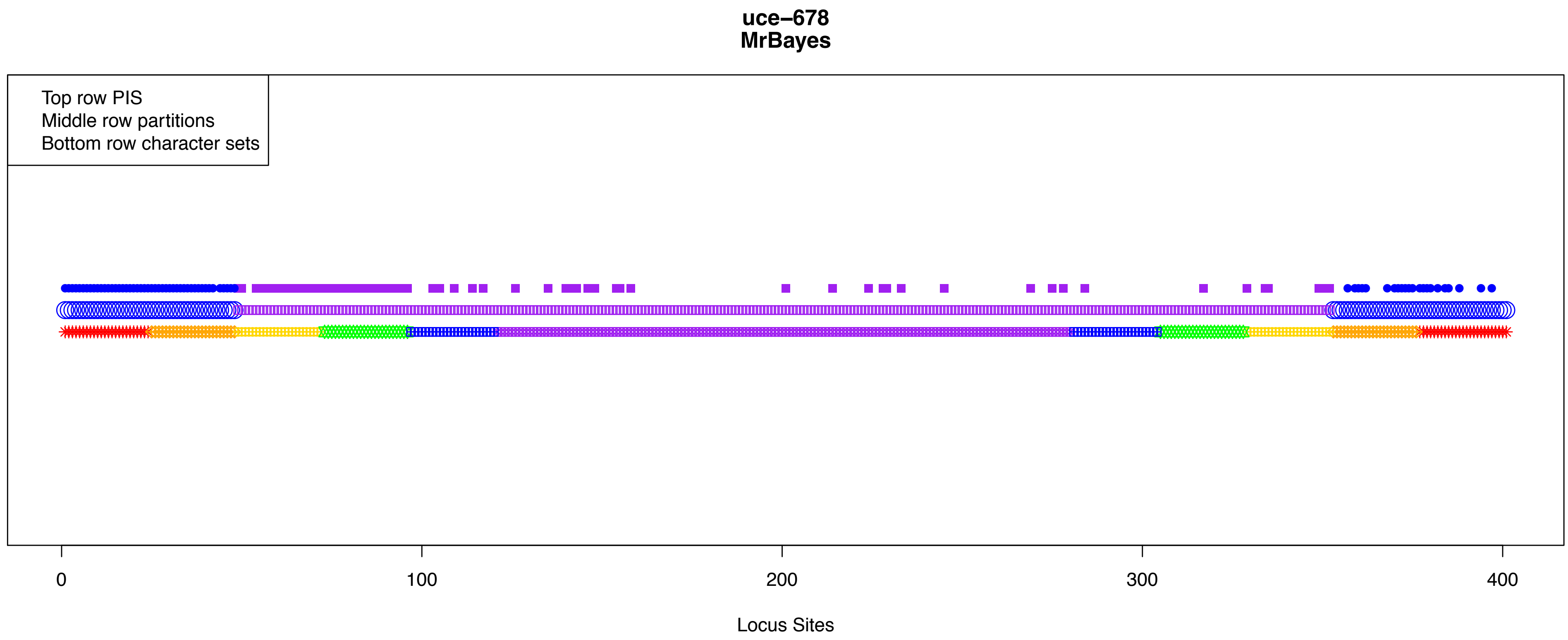

uce–678
MrBayes
Top row PIS
Middle row partitions
Bottom row character sets
0
100
200
300
400
Locus Sites

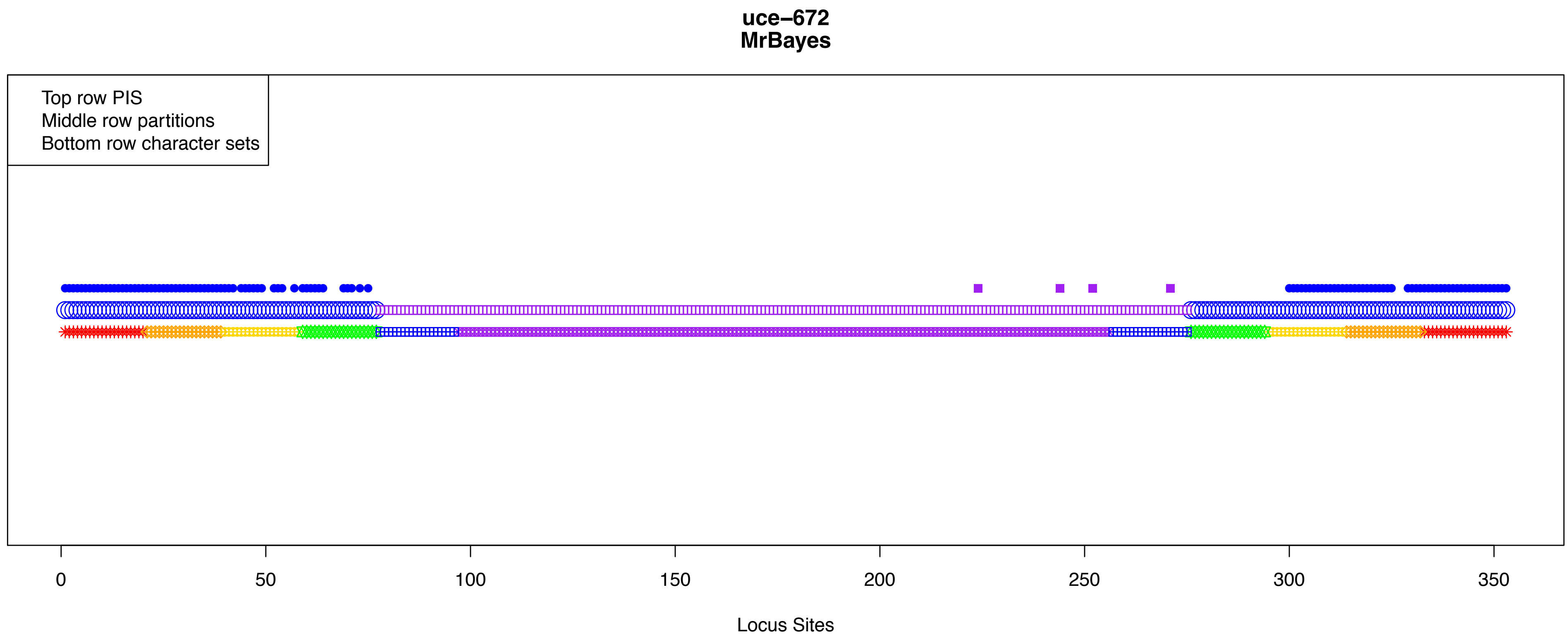
uce–672
MrBayes
Top row PIS
Middle row partitions
Bottom row character sets
0
50
100
150
200
250
300
350
Locus Sites

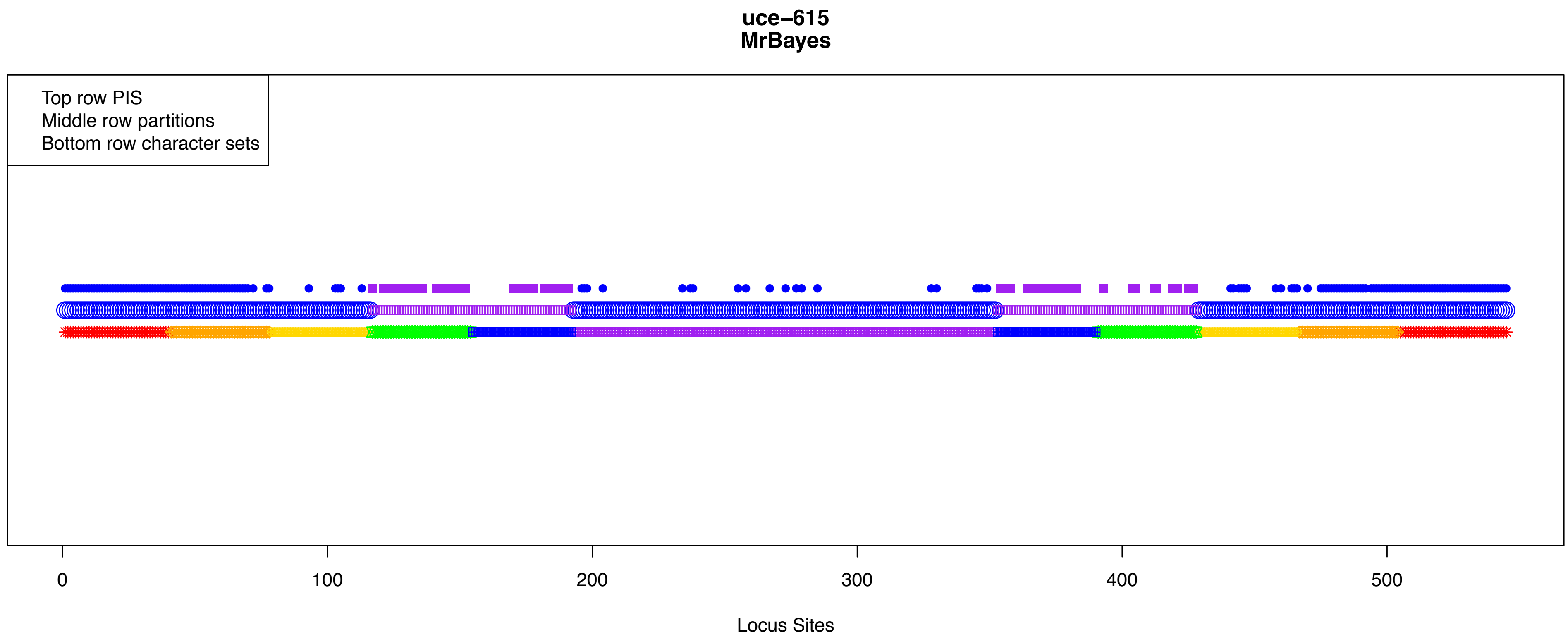
uce–615
MrBayes
Top row PIS
Middle row partitions
Bottom row character sets
0
100
200
300
400
500
Locus Sites

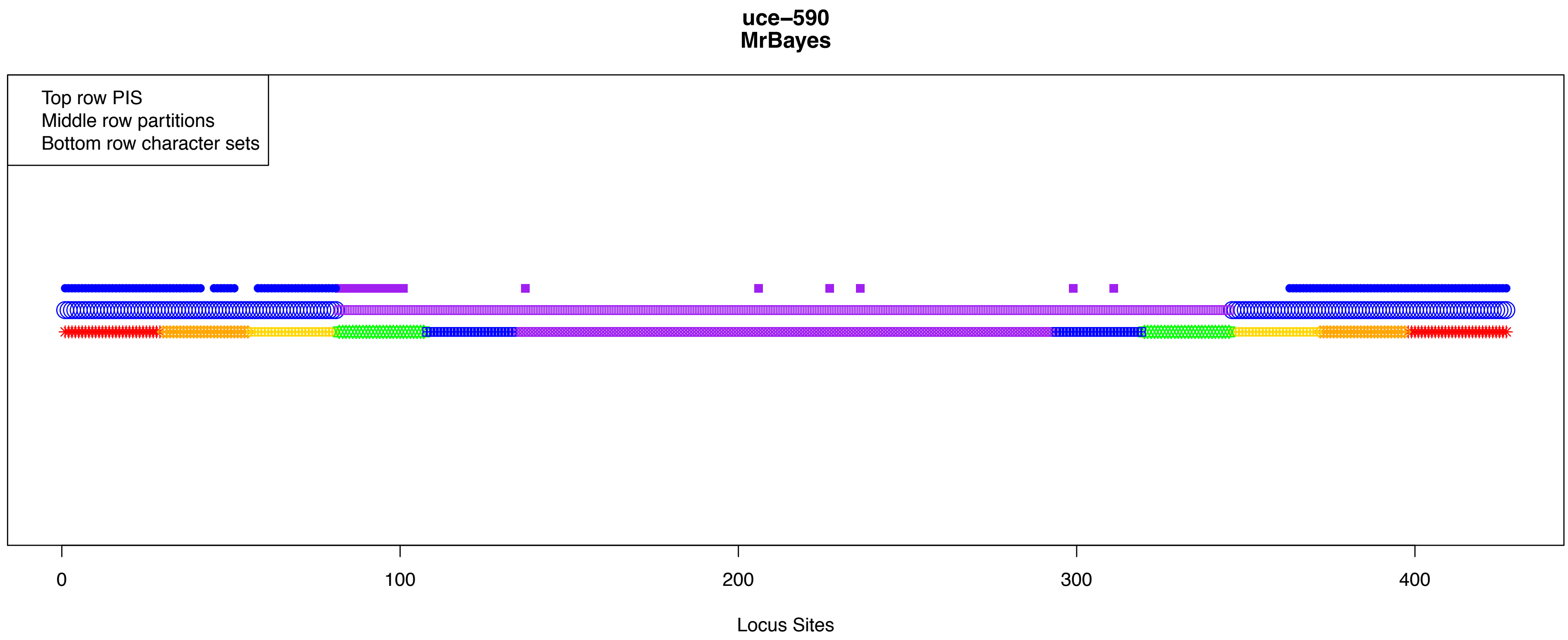
uce–590
MrBayes
Top row PIS
Middle row partitions
Bottom row character sets
0
100
200
300
400
Locus Sites

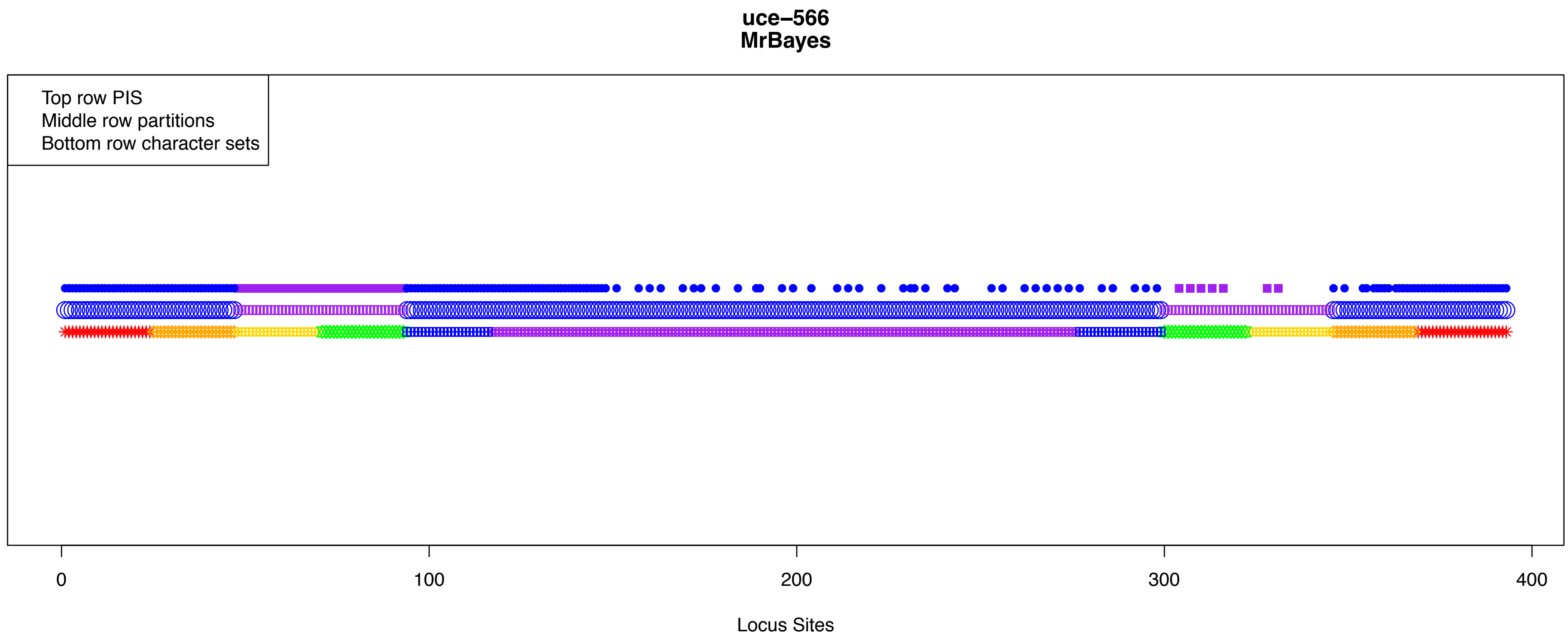

uce–566
MrBayes
Top row PIS
Middle row partitions
Bottom row character sets
0
100
200
300
400
Locus Sites

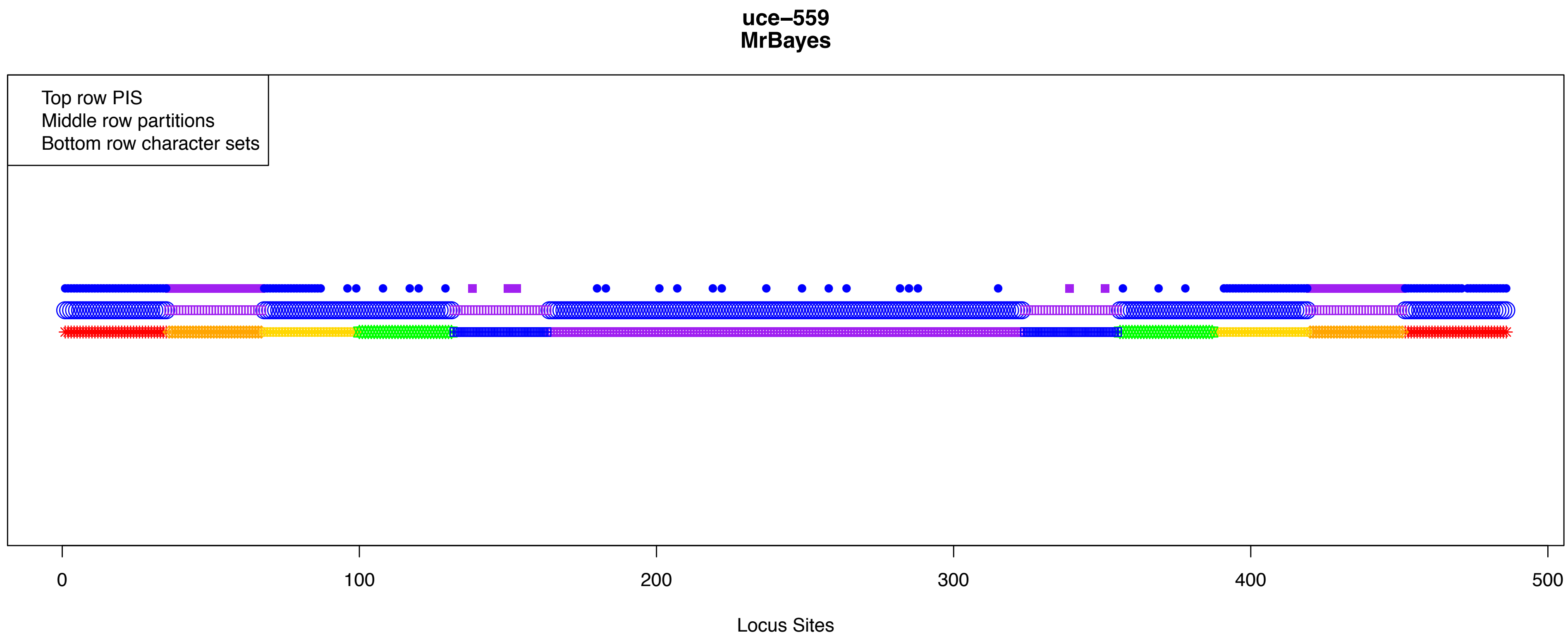
uce–559
MrBayes
Top row PIS
Middle row partitions
Bottom row character sets
0
100
200
300
400
500
Locus Sites

uce–549
MrBayes

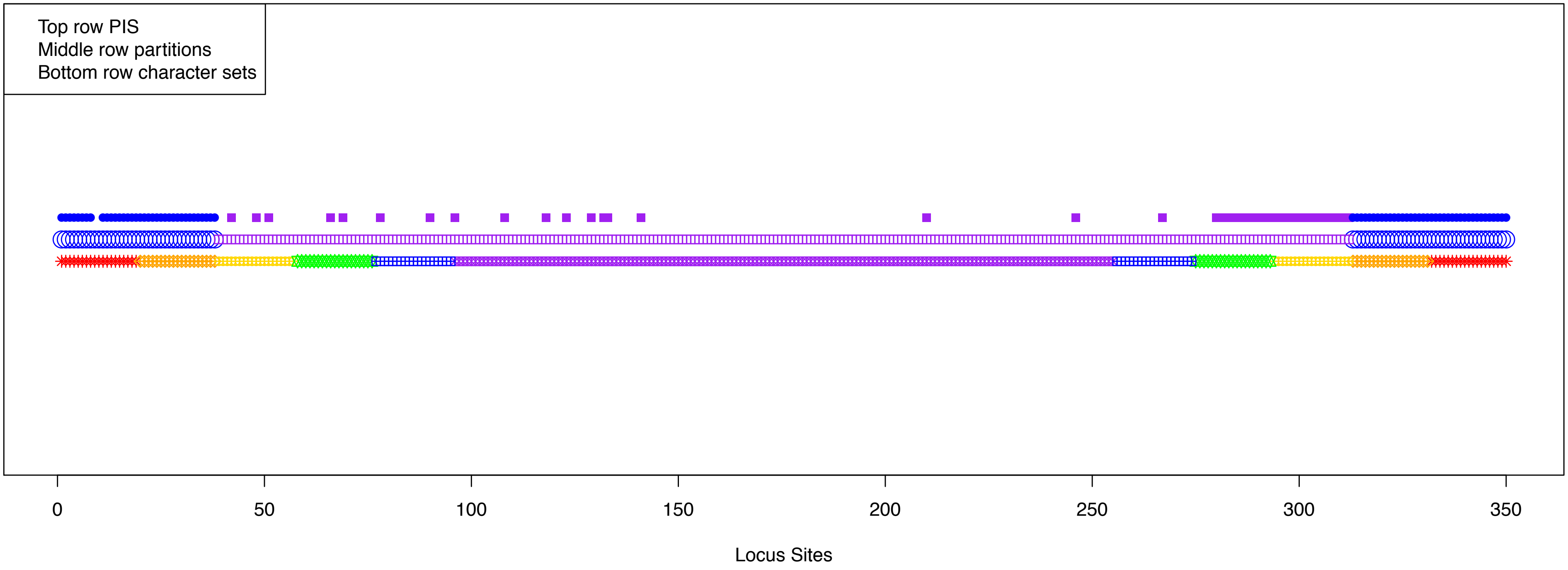

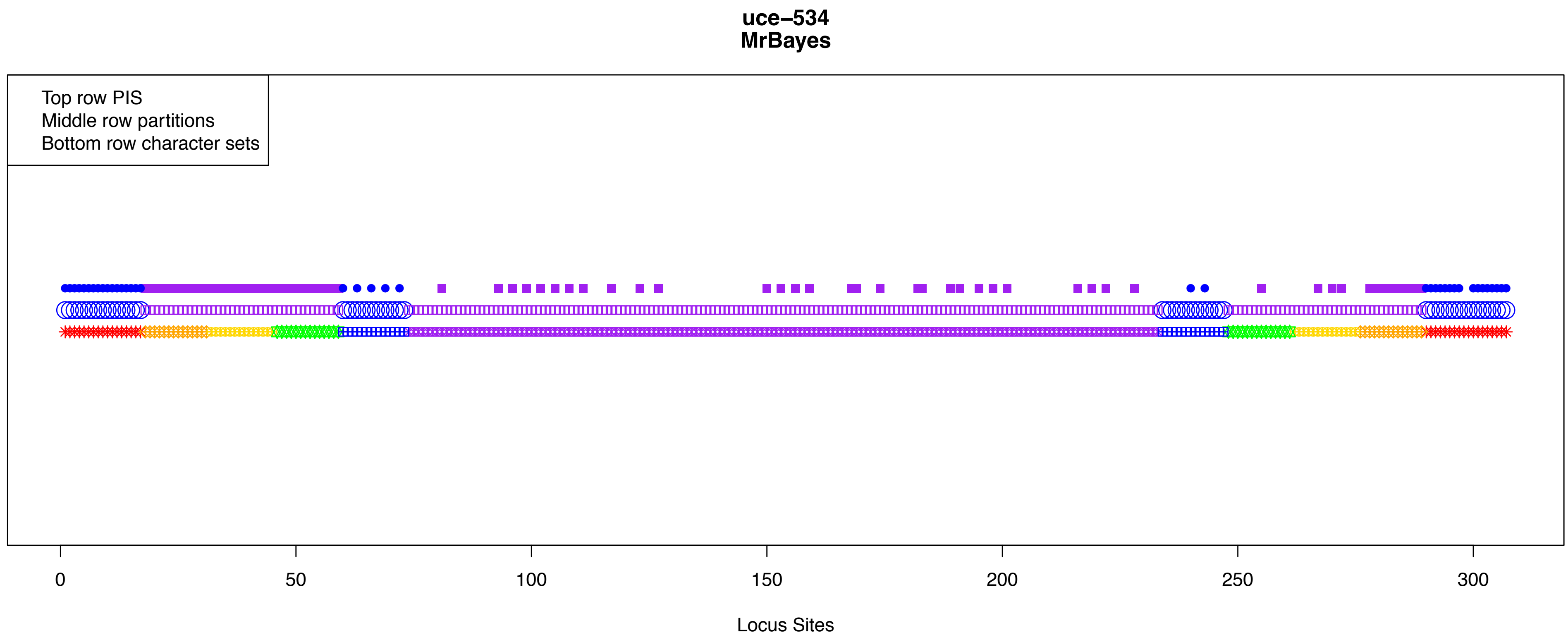
uce–534
MrBayes
Top row PIS
Middle row partitions
Bottom row character sets
0
50
100
150
200
250
300
Locus Sites

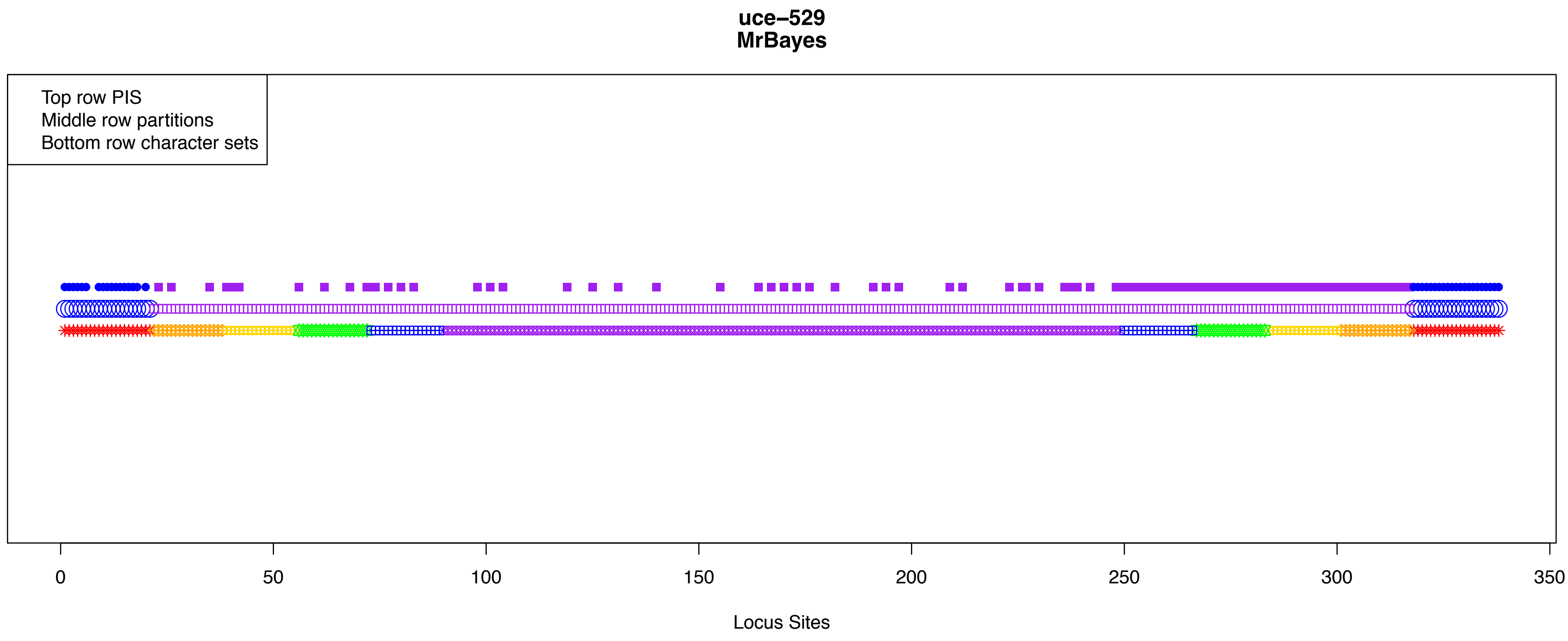
uce–529
MrBayes
Top row PIS
Middle row partitions
Bottom row character sets
0
50
100
150
200
250
300
350
Locus Sites

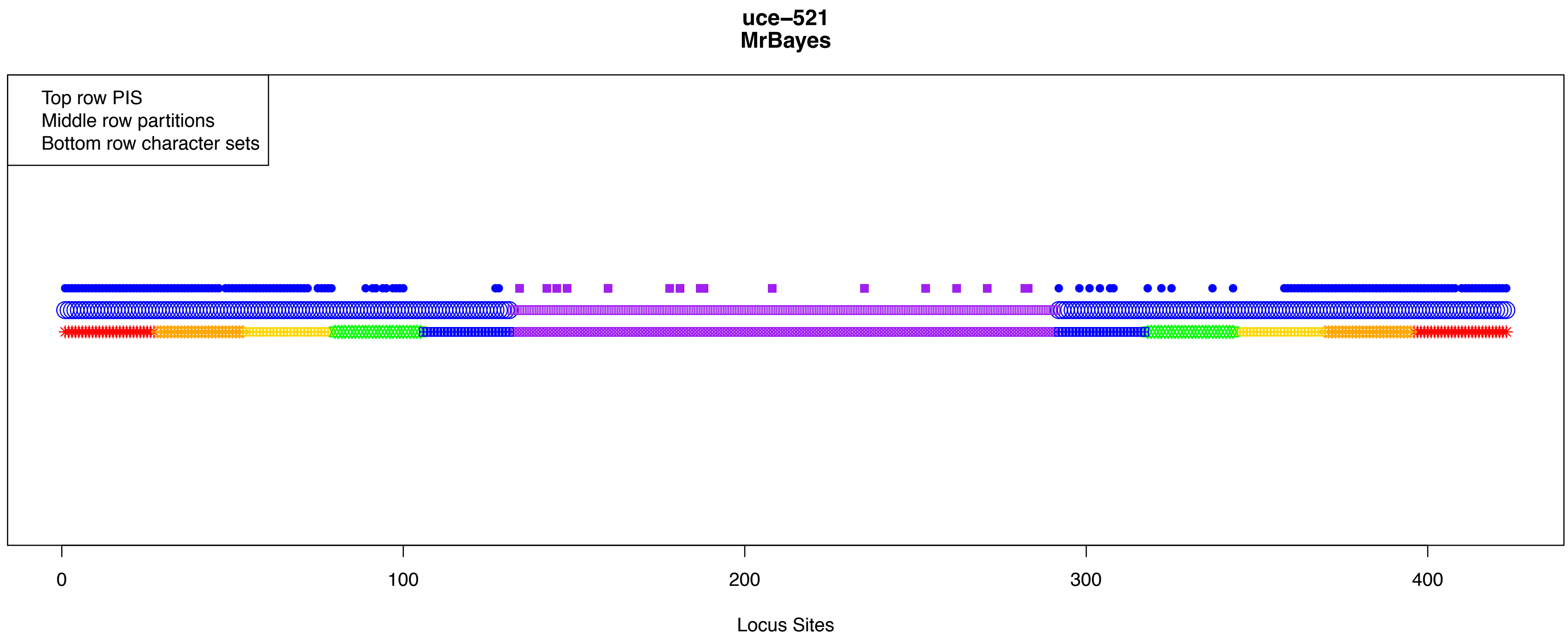
uce–521
MrBayes
Top row PIS
Middle row partitions
Bottom row character sets
0
100
200
300
400
Locus Sites

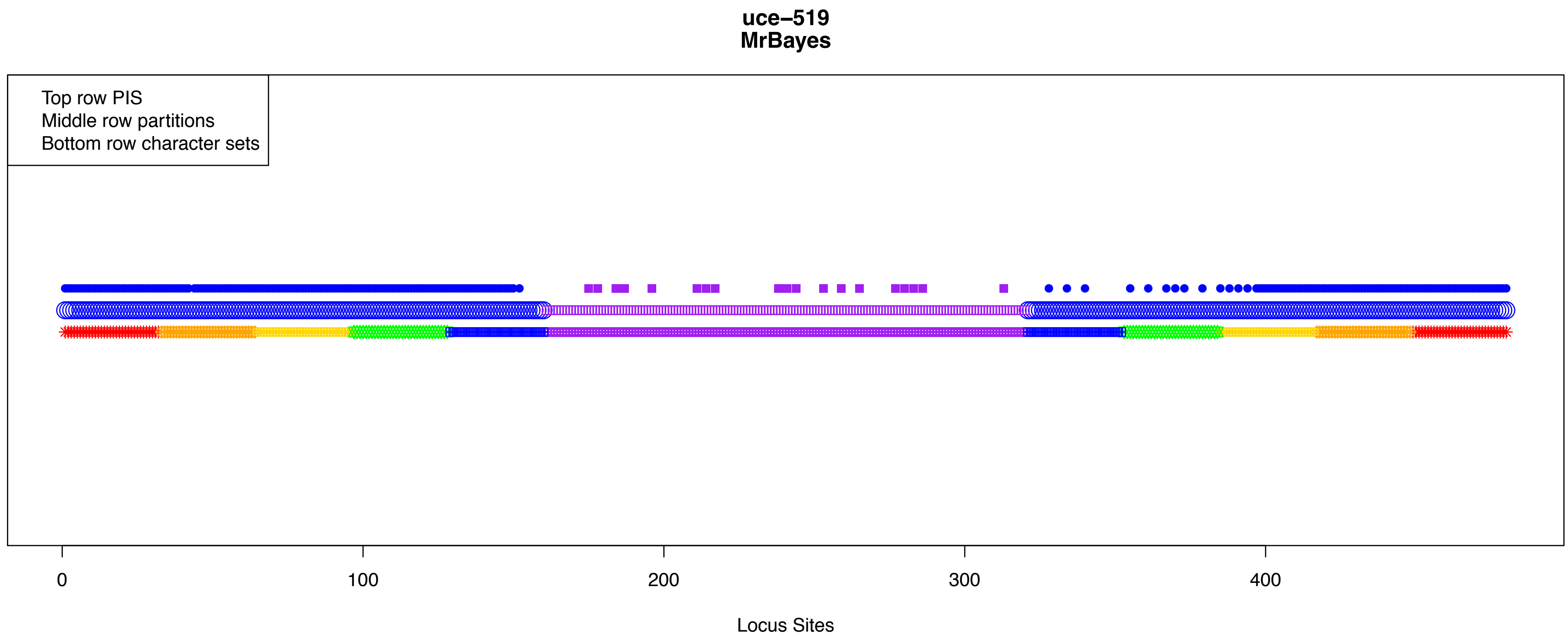
uce–519
MrBayes
Top row PIS
Middle row partitions
Bottom row character sets
0
100
200
300
400
Locus Sites

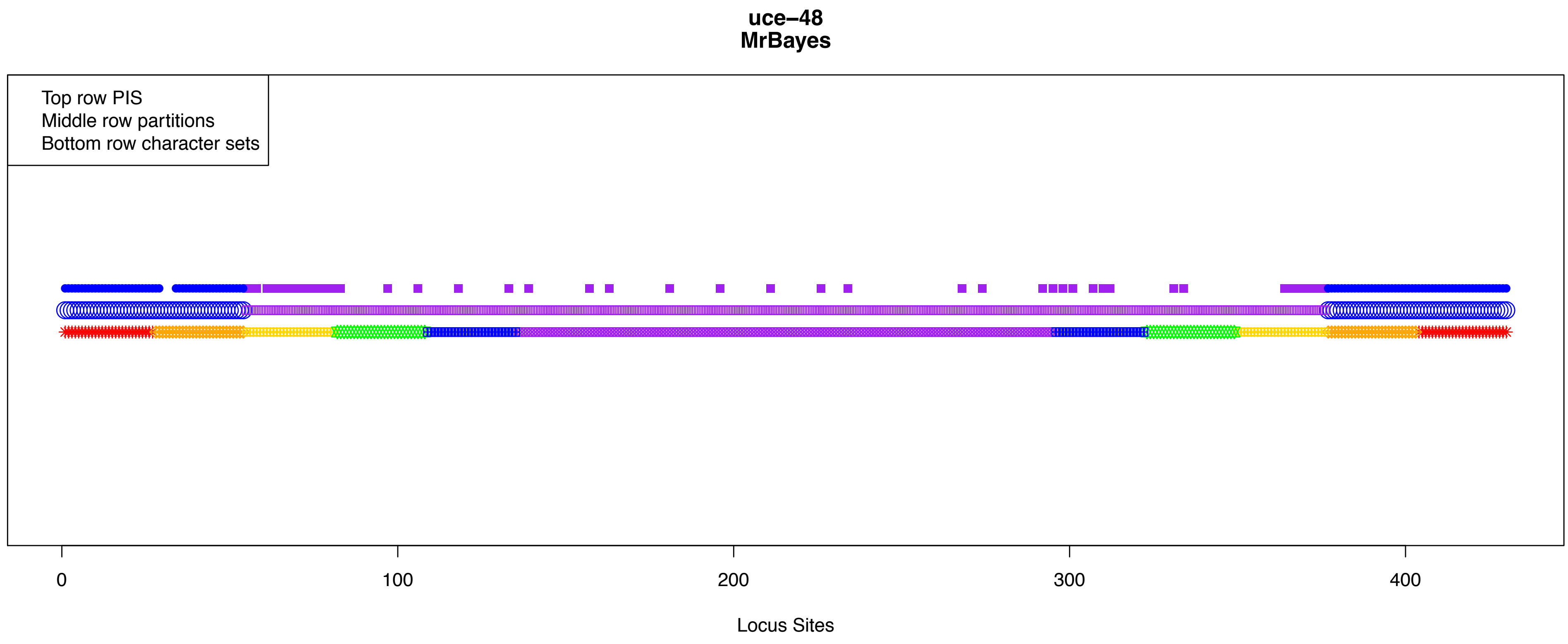
uce–48
MrBayes
Top row PIS
Middle row partitions
Bottom row character sets
0
100
200
300
400
Locus Sites

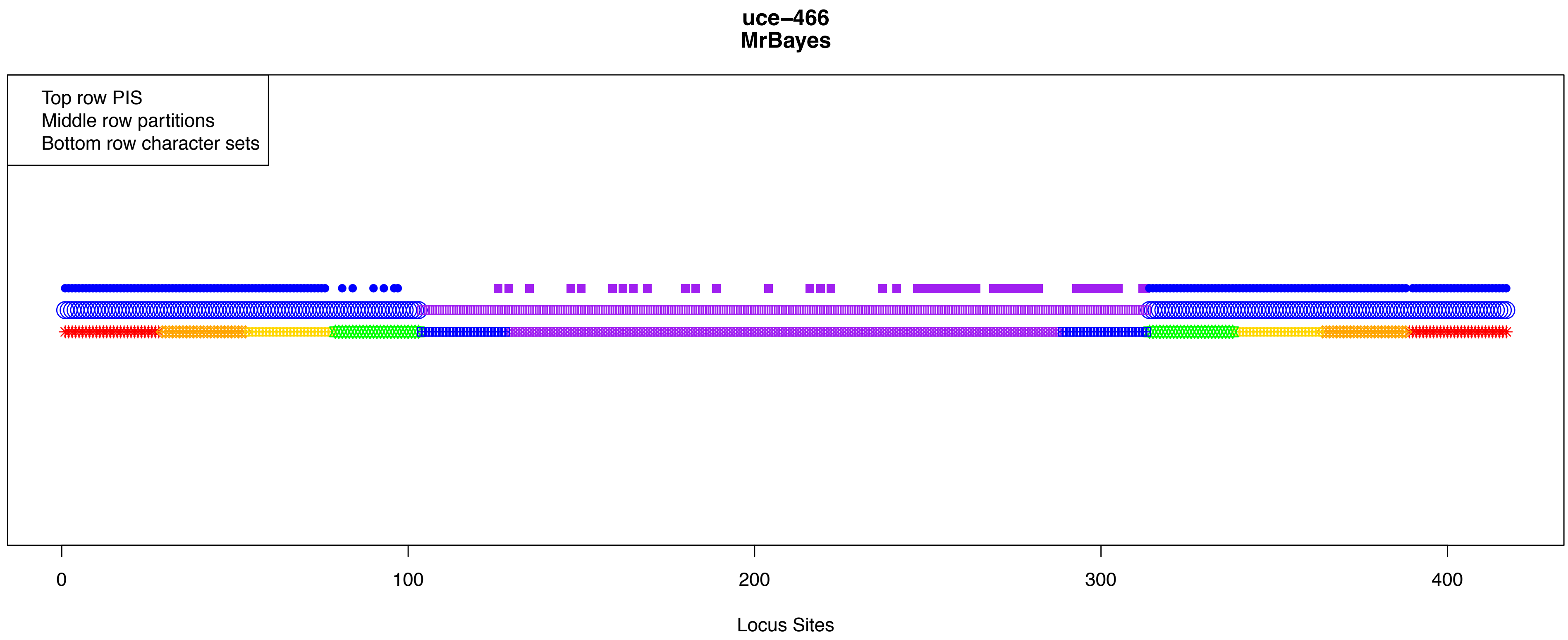
uce–466
MrBayes
Top row PIS
Middle row partitions
Bottom row character sets
0
100
200
300
400
Locus Sites

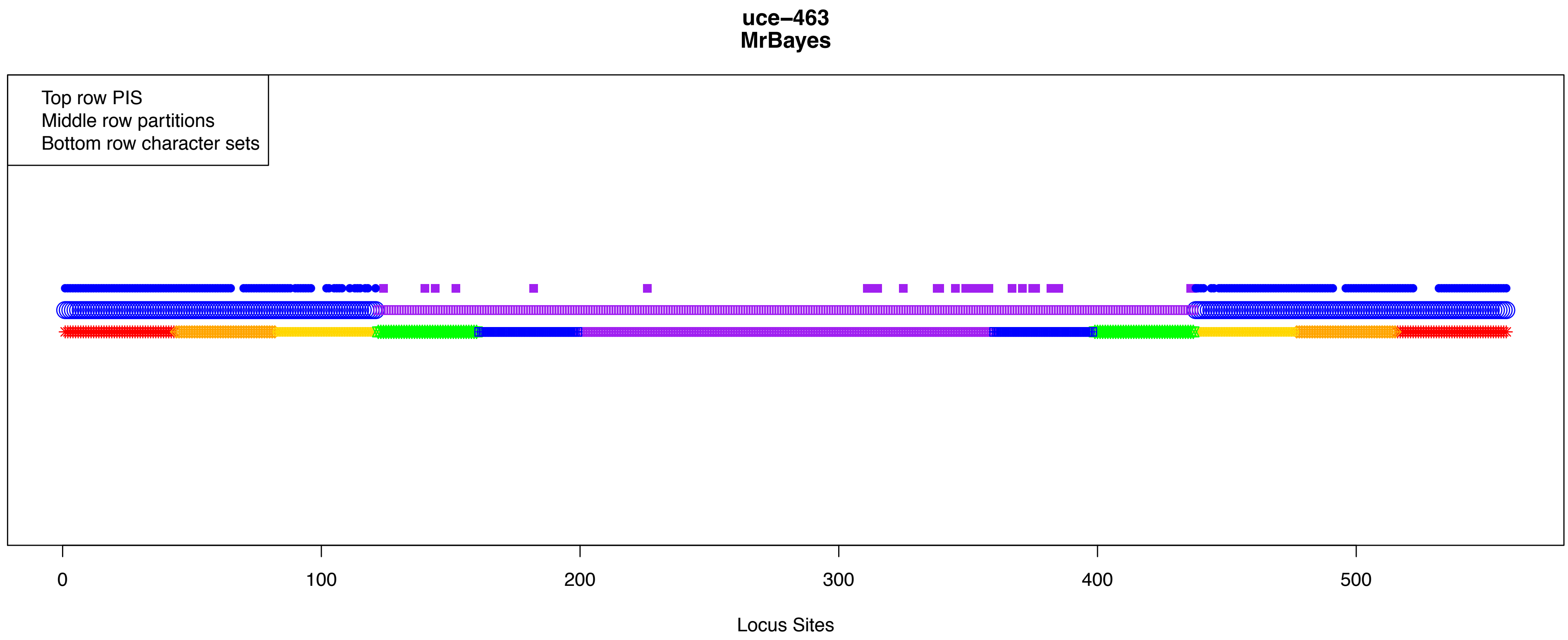

uce–463
MrBayes
Top row PIS
Middle row partitions
Bottom row character sets
0
100
200
300
400
500
Locus Sites

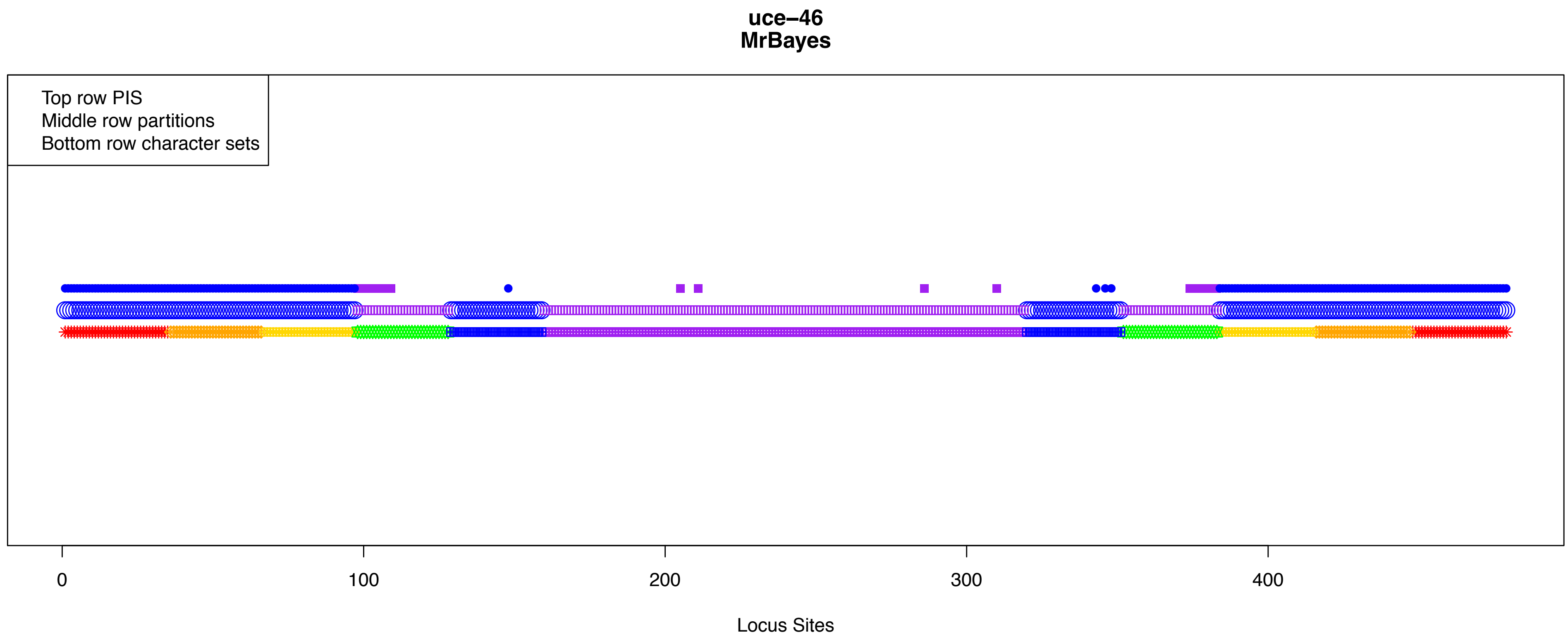

uce–46
MrBayes
Top row PIS
Middle row partitions
Bottom row character sets
0
100
200
300
400
Locus Sites

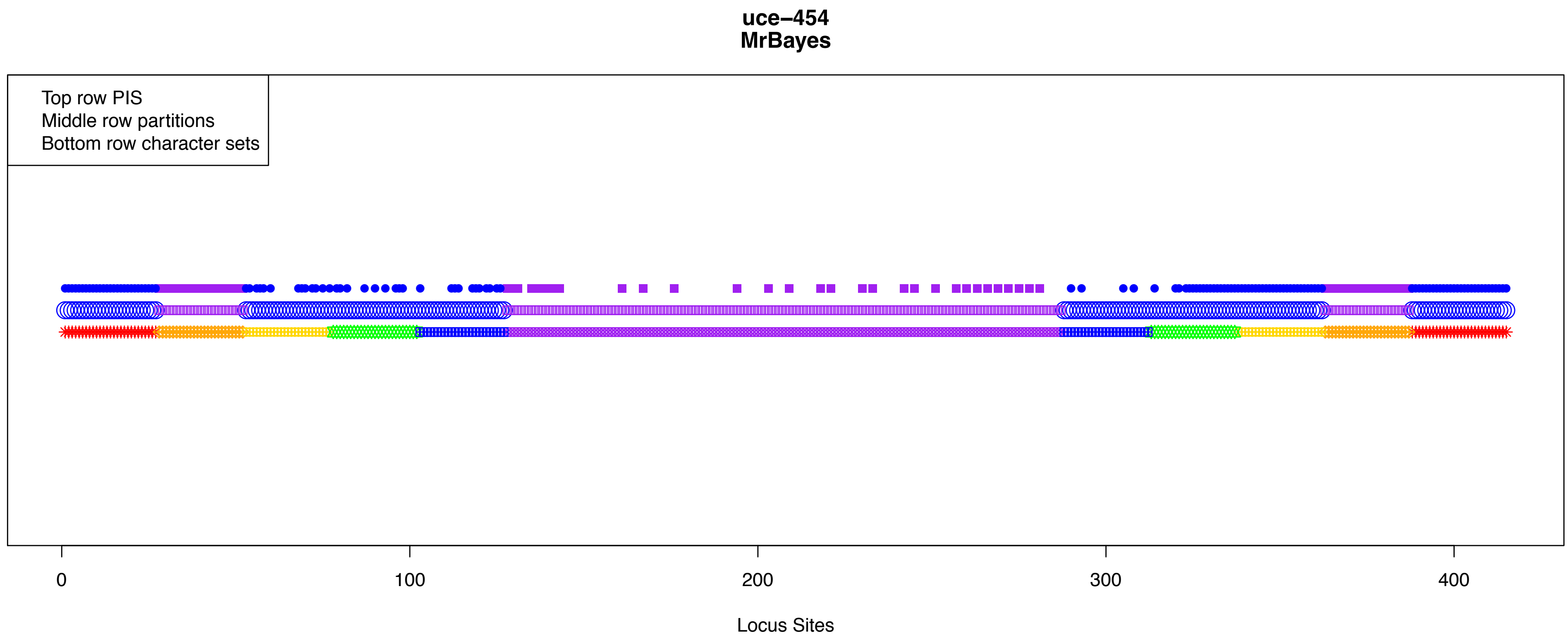
uce–454
MrBayes
Top row PIS
Middle row partitions
Bottom row character sets
0
100
200
300
400
Locus Sites

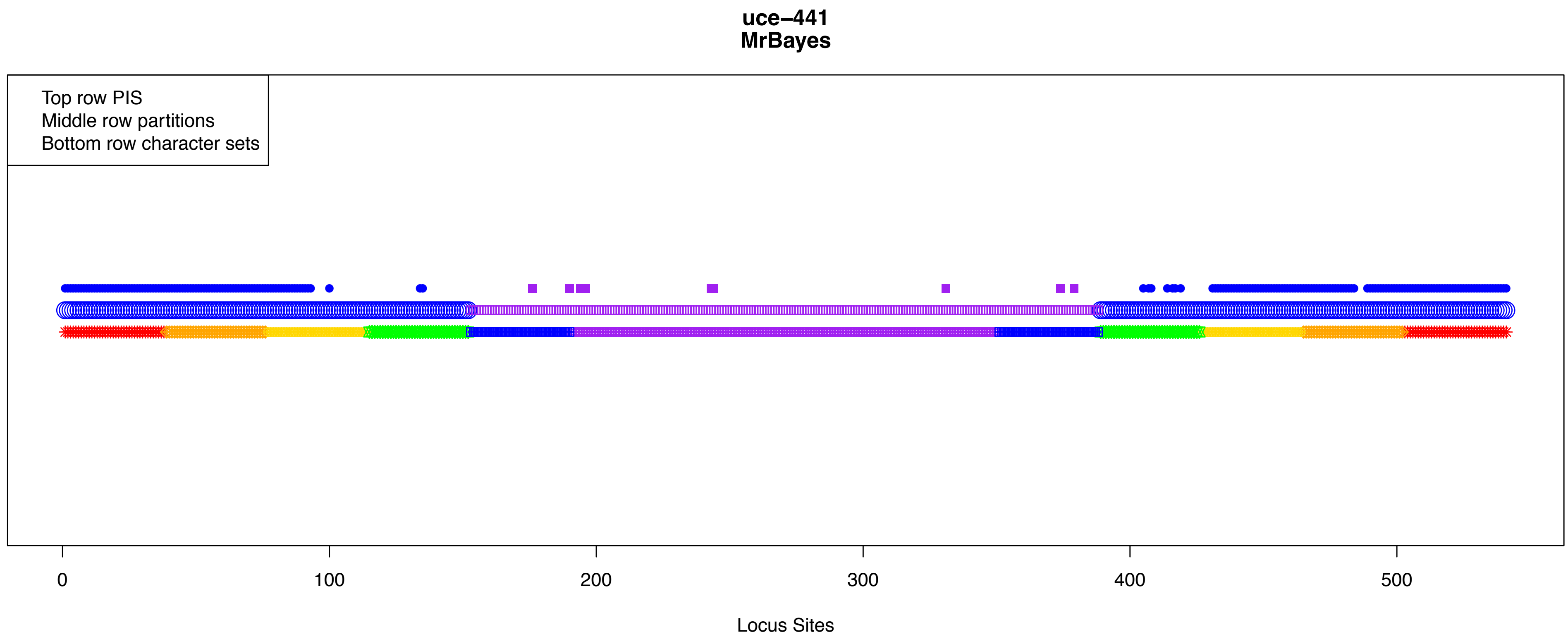
uce–441
MrBayes
Top row PIS
Middle row partitions
Bottom row character sets
0
100
200
300
400
500
Locus Sites

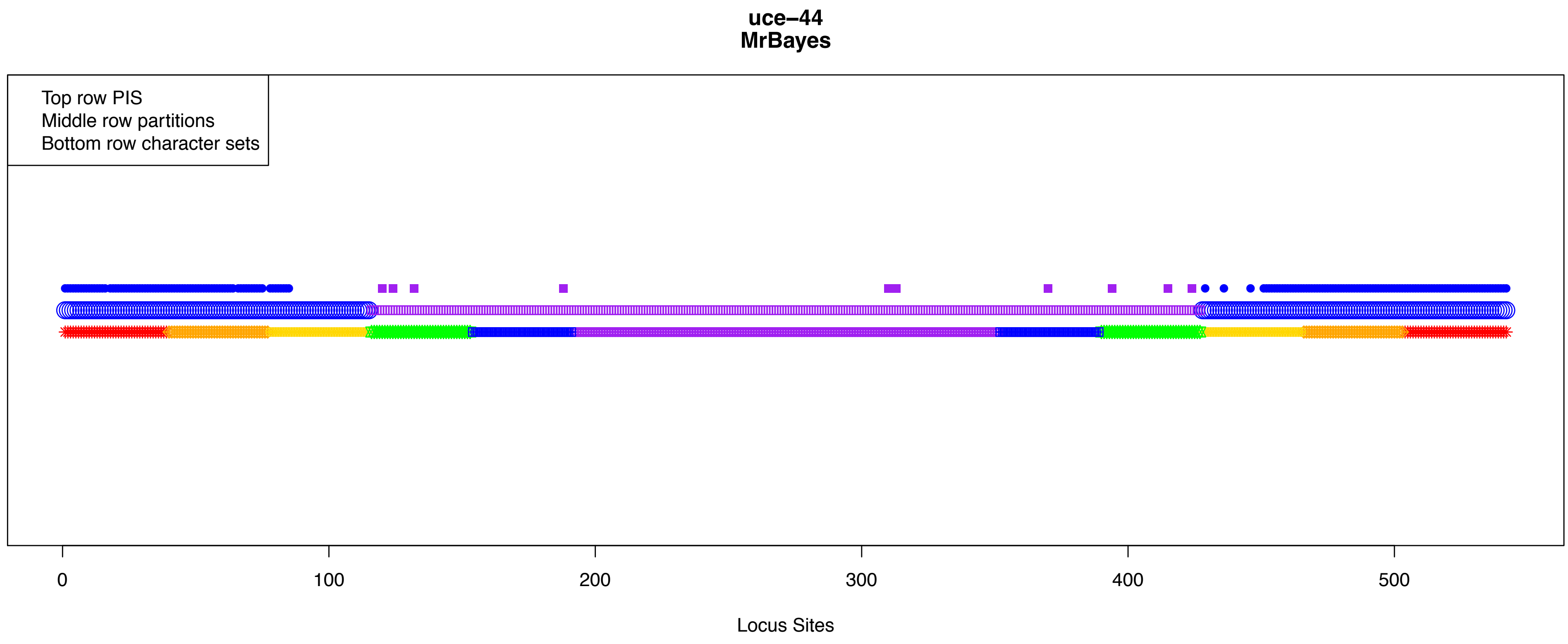
uce–44
MrBayes
Top row PIS
Middle row partitions
Bottom row character sets
0
100
200
300
400
500
Locus Sites

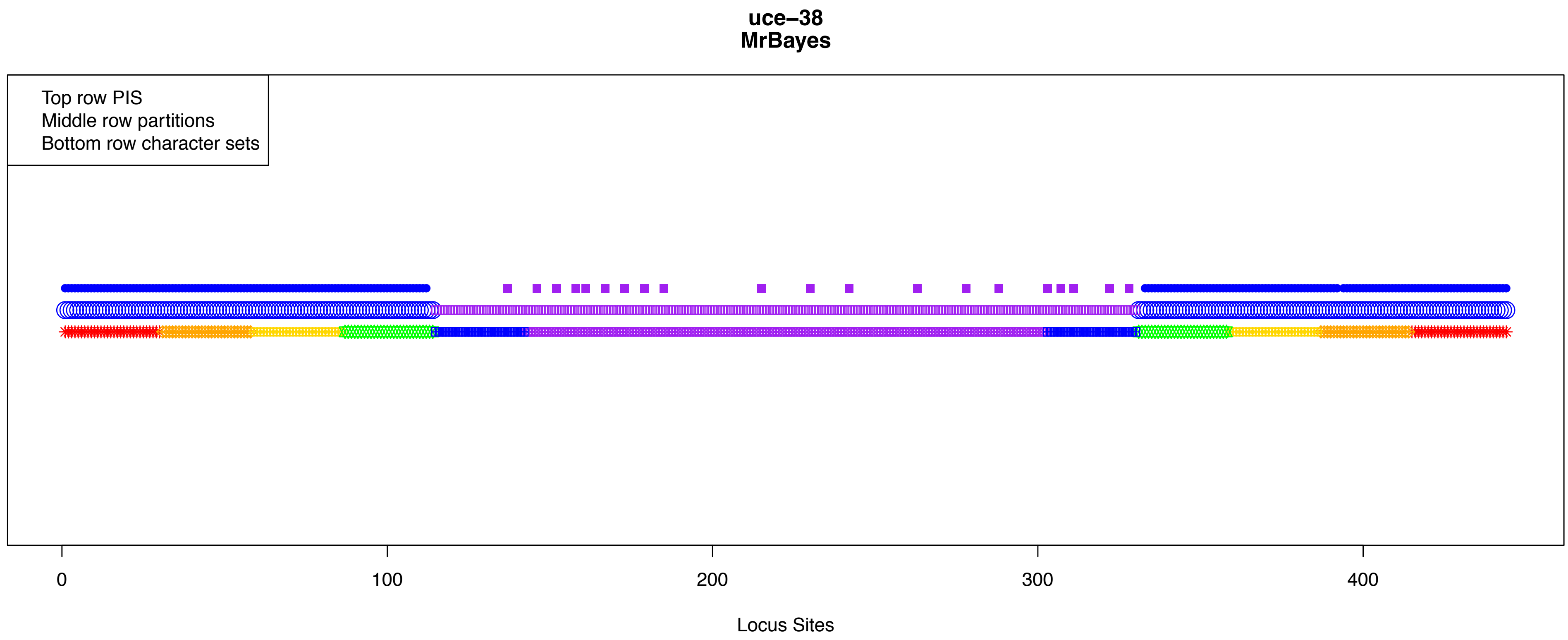
uce–38
MrBayes
Top row PIS
Middle row partitions
Bottom row character sets
0
100
200
300
400
Locus Sites

uce–365
MrBayes

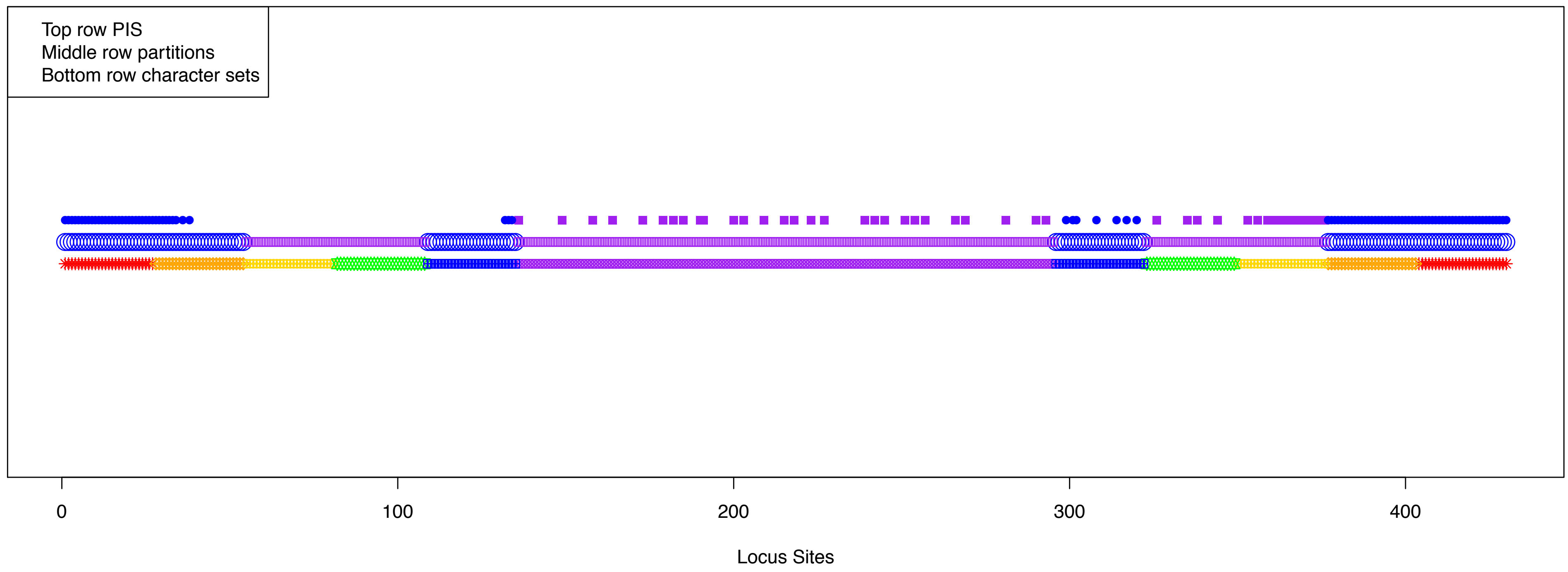

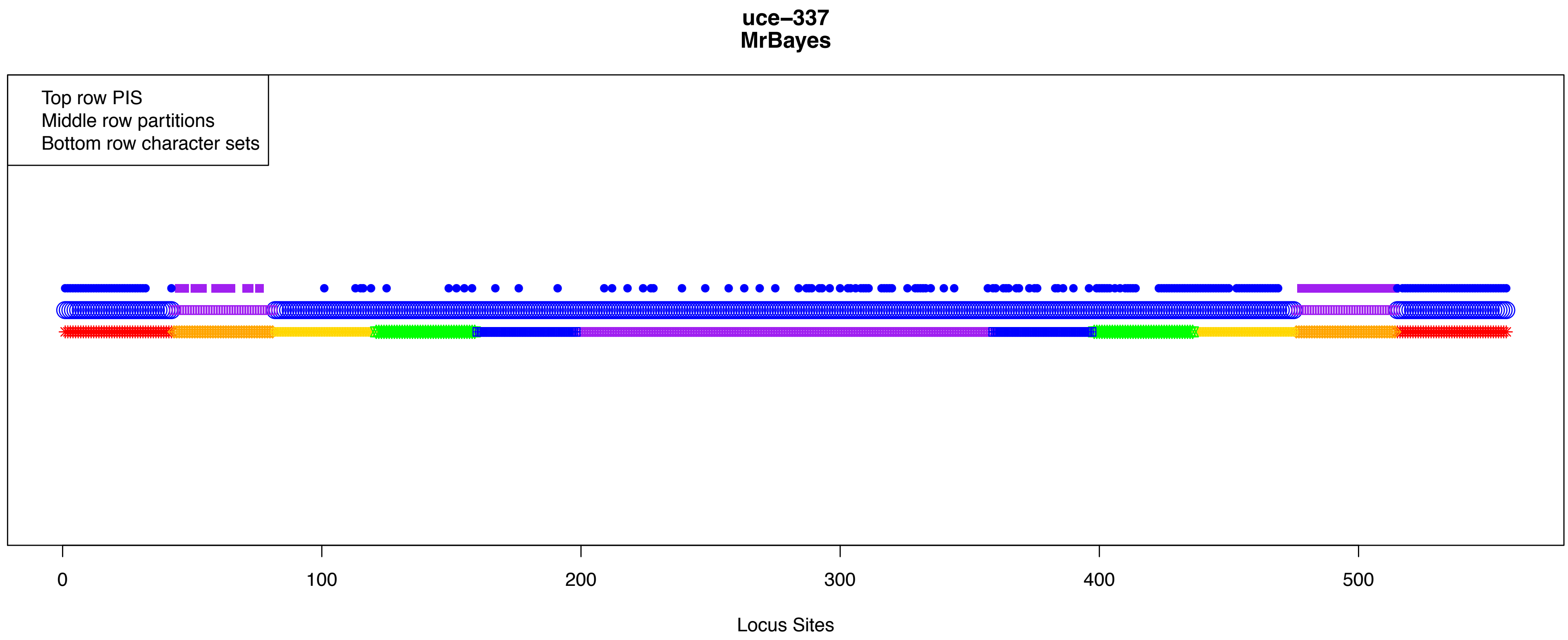

uce–337
MrBayes
Top row PIS
Middle row partitions
Bottom row character sets
0
100
200
300
400
500
Locus Sites

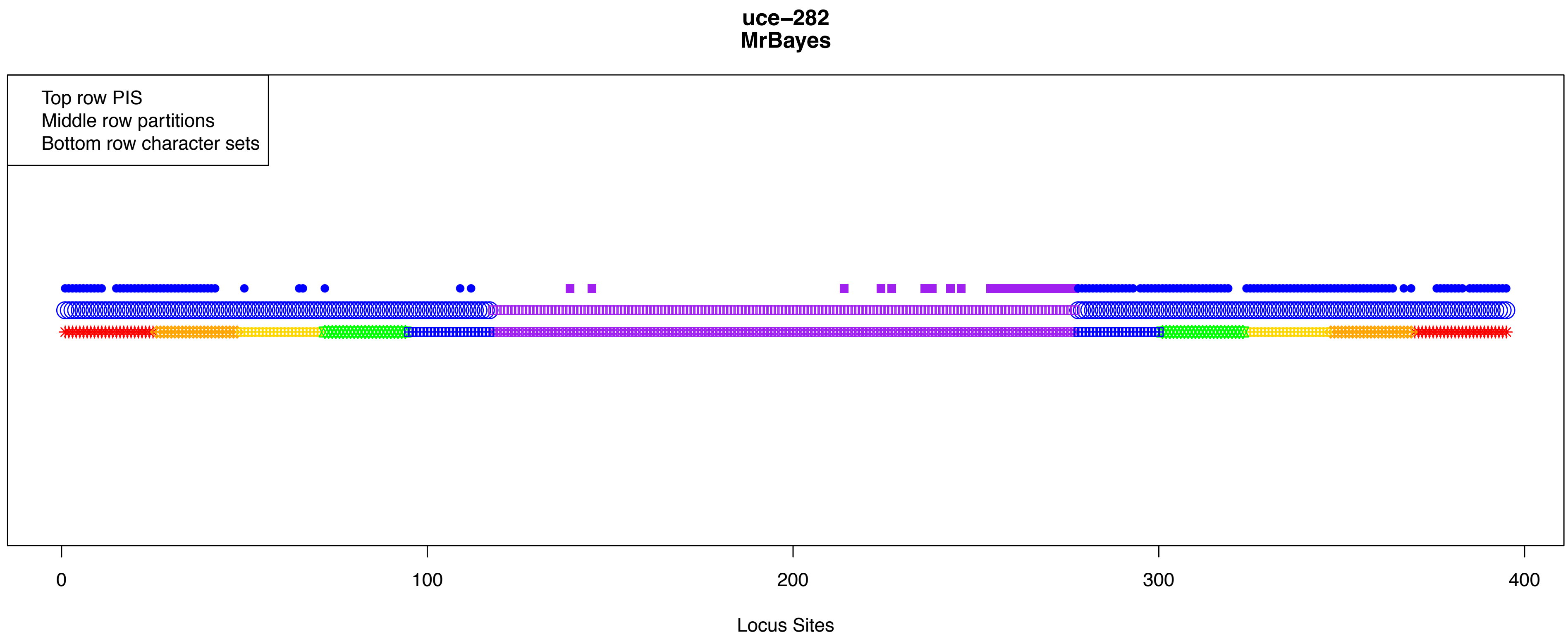
uce–282
MrBayes
Top row PIS
Middle row partitions
Bottom row character sets
0
100
200
300
400
Locus Sites

uce–28
MrBayes

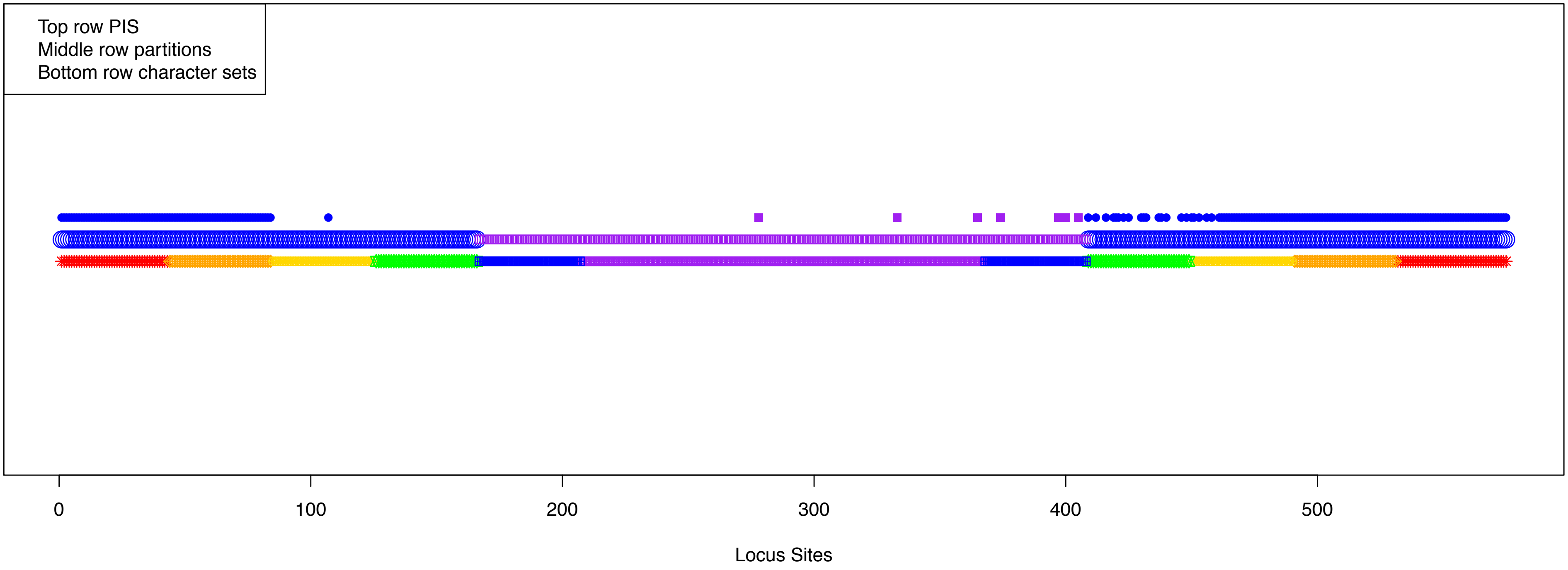

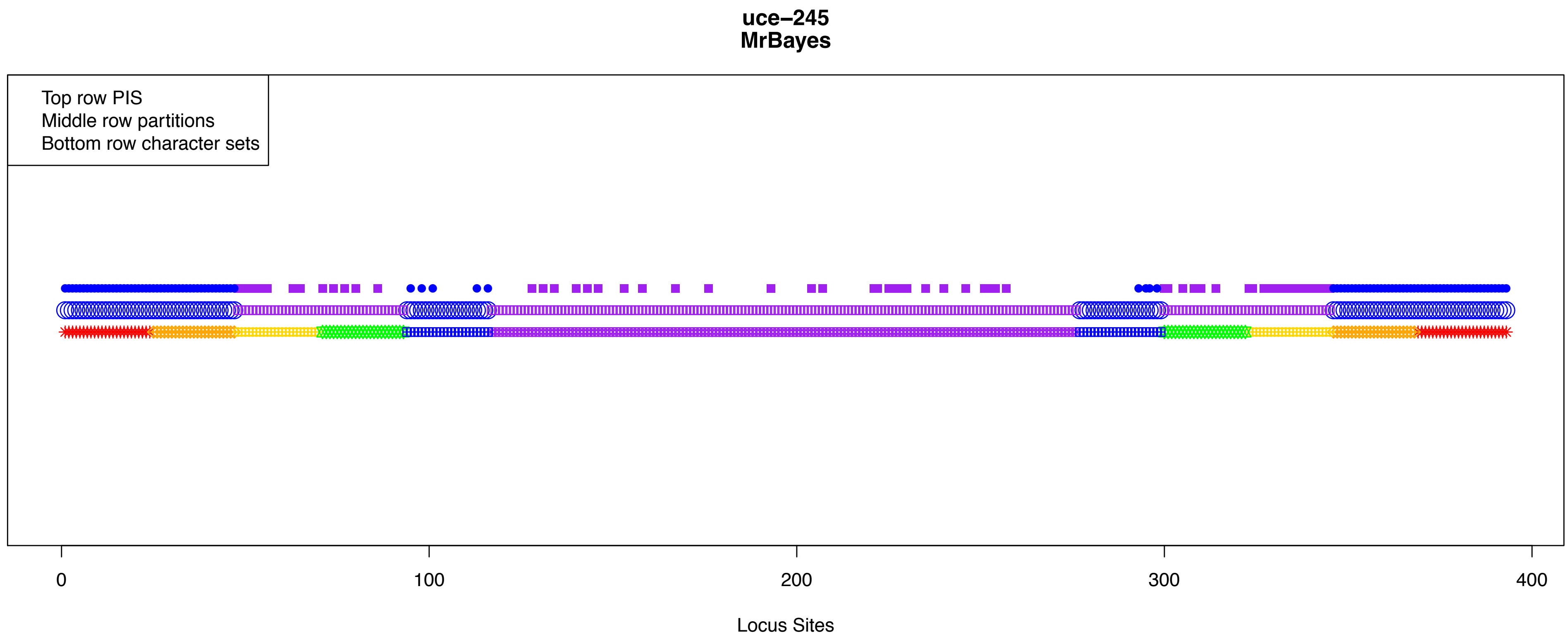
uce–245
MrBayes
Top row PIS
Middle row partitions
Bottom row character sets
0
100
200
300
400
Locus Sites

uce–233
MrBayes

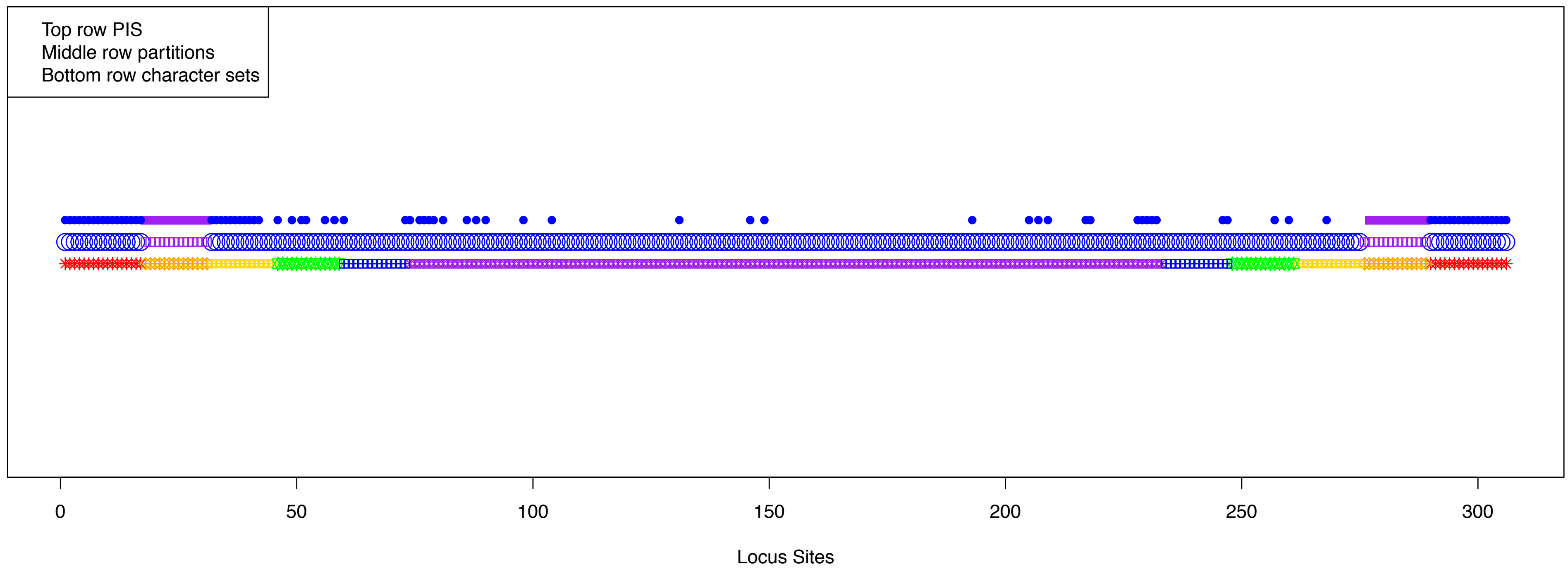

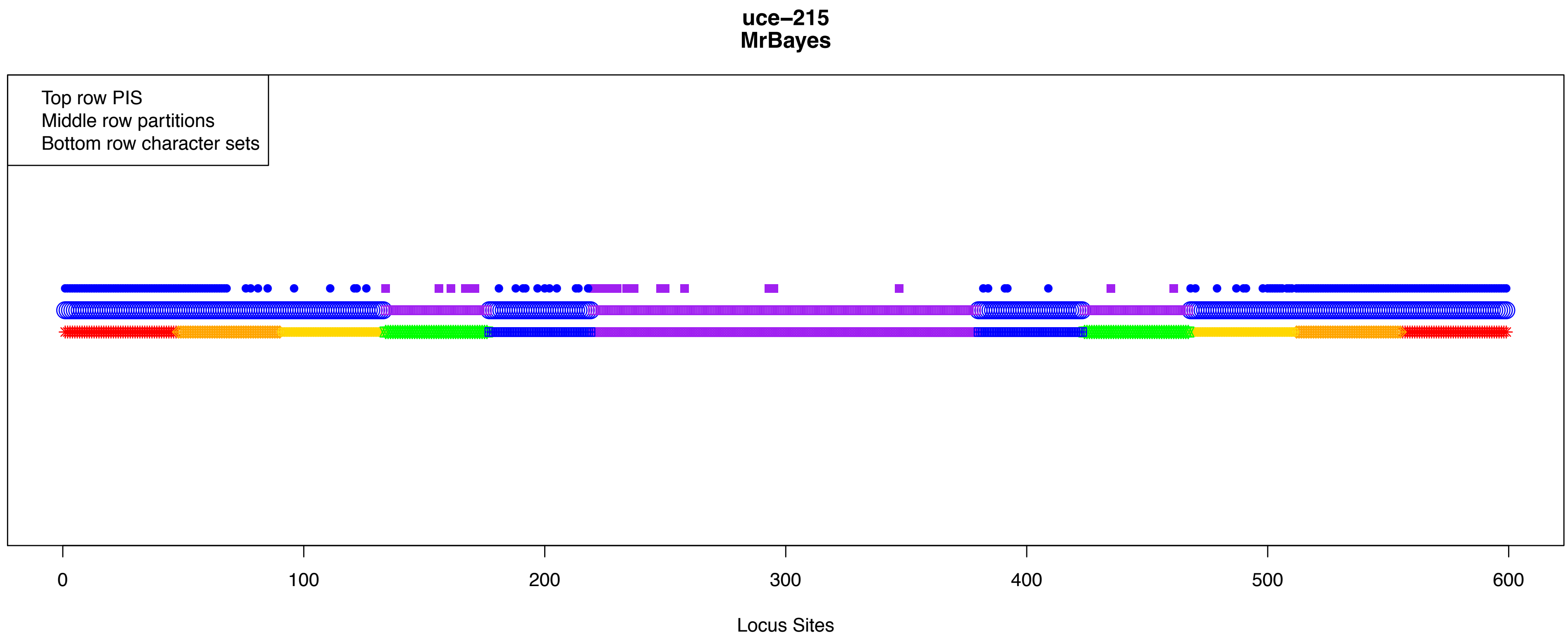
uce–215
MrBayes
Top row PIS
Middle row partitions
Bottom row character sets
0
100
200
300
400
500
600
Locus Sites

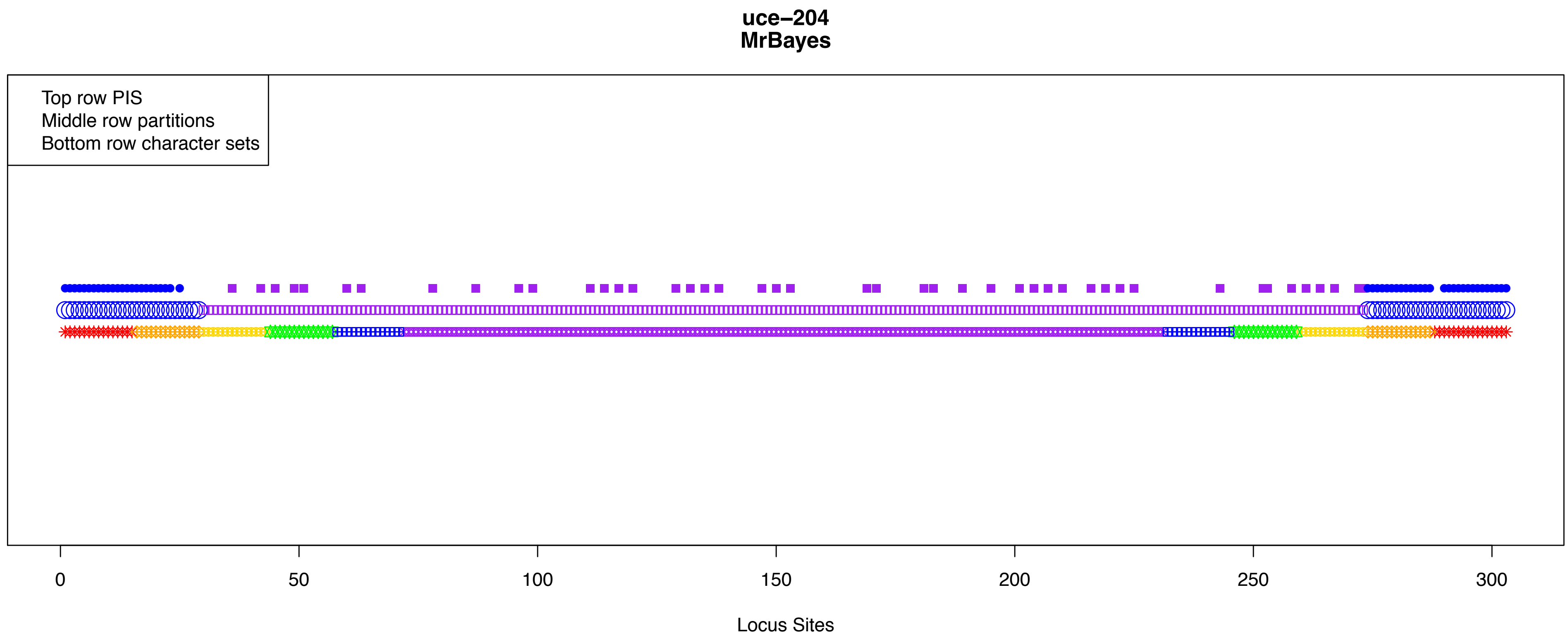
uce–204
MrBayes
Top row PIS
Middle row partitions
Bottom row character sets
0
50
100
150
200
250
300
Locus Sites

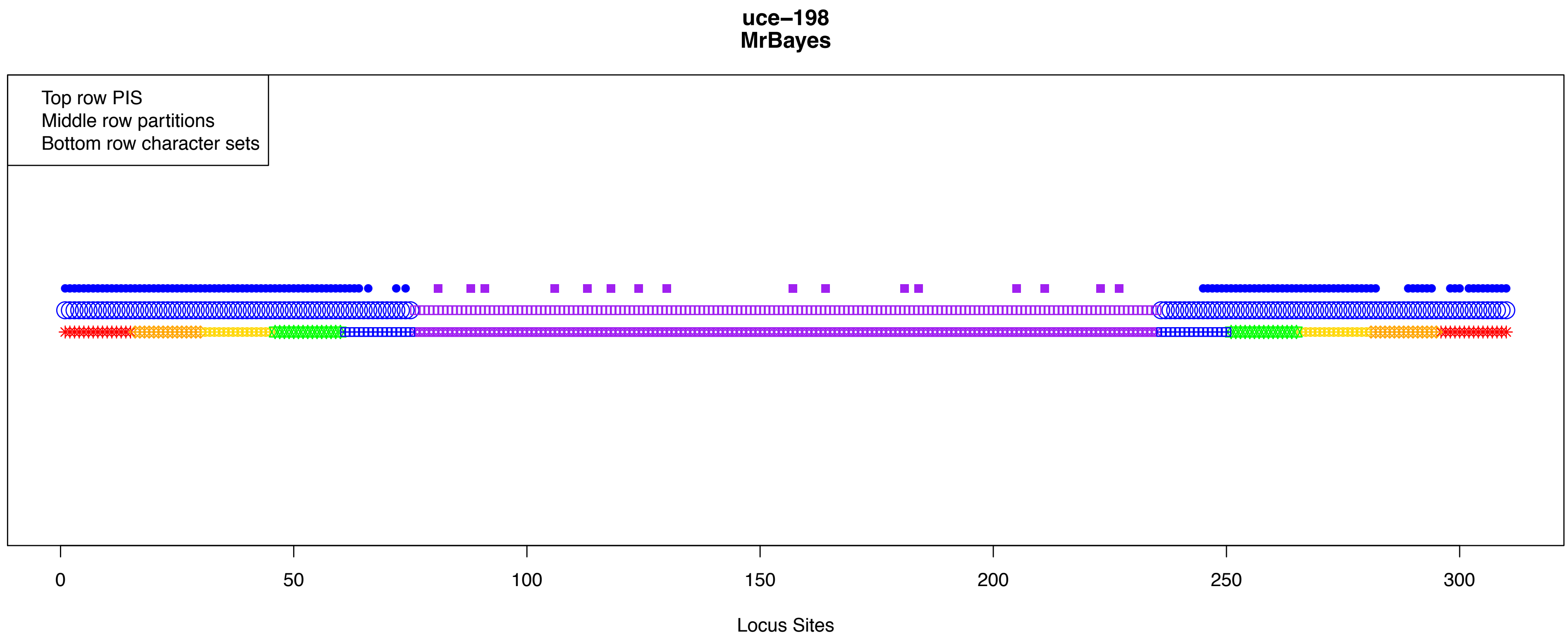

uce–198
MrBayes
Top row PIS
Middle row partitions
Bottom row character sets
0
50
100
150
200
250
300
Locus Sites

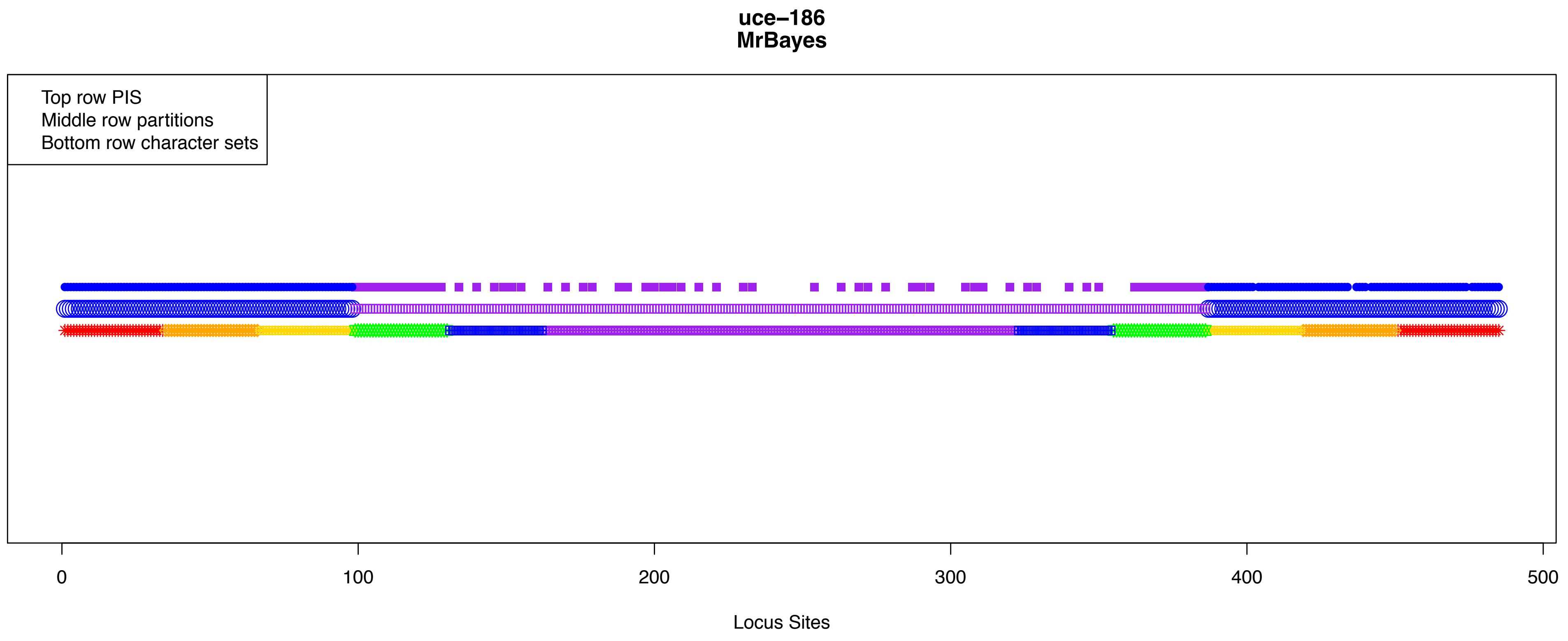

uce–186
MrBayes
Top row PIS
Middle row partitions
Bottom row character sets
0
100
200
300
400
500
Locus Sites

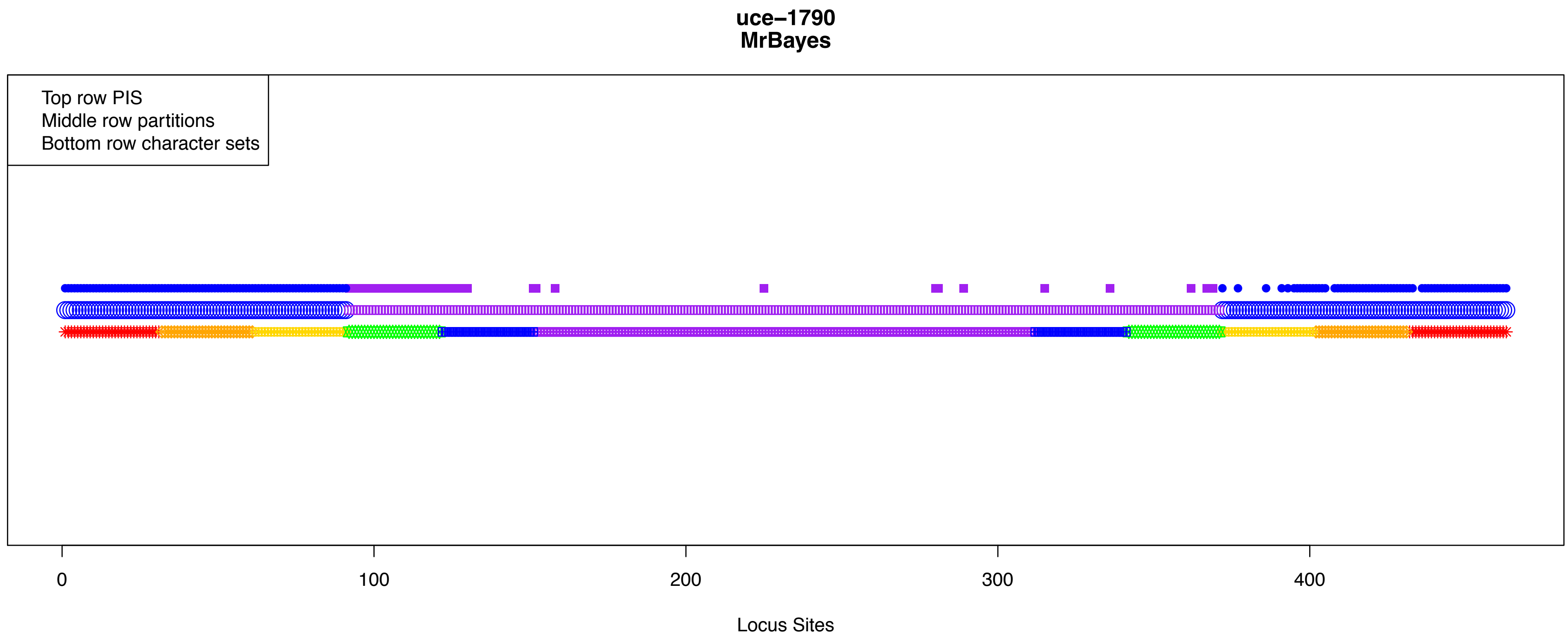
uce–1790
MrBayes
Top row PIS
Middle row partitions
Bottom row character sets
0
100
200
300
400
Locus Sites

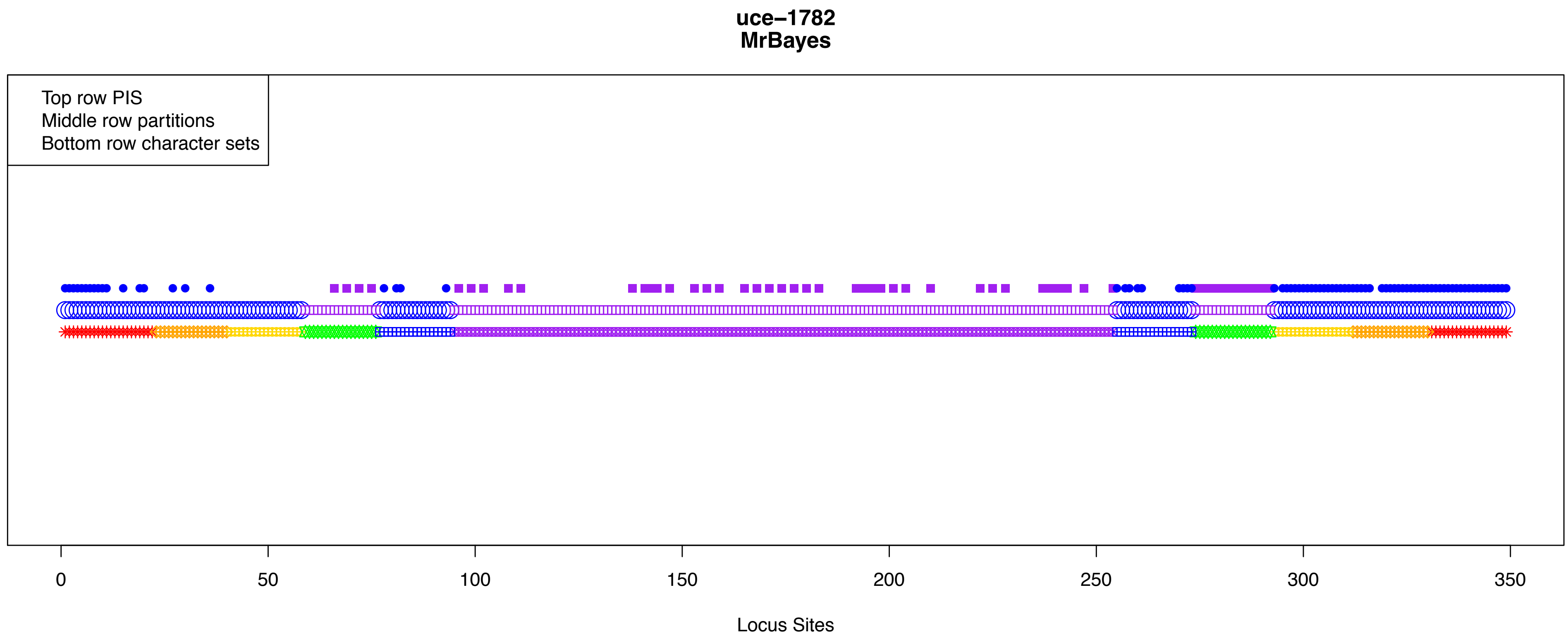

uce–1782
MrBayes
Top row PIS
Middle row partitions
Bottom row character sets
0
50
100
150
200
250
300
350
Locus Sites

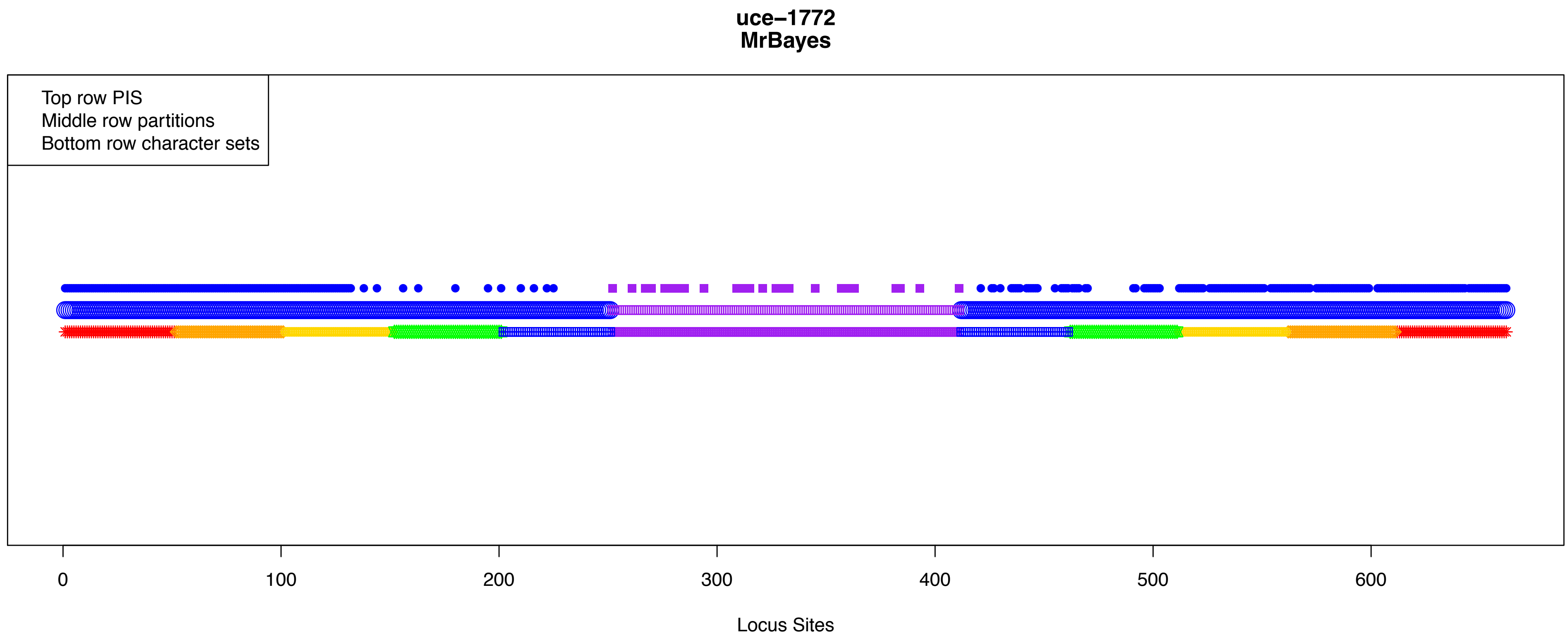

uce–1772
MrBayes
Top row PIS
Middle row partitions
Bottom row character sets
0
100
200
300
400
500
600
Locus Sites

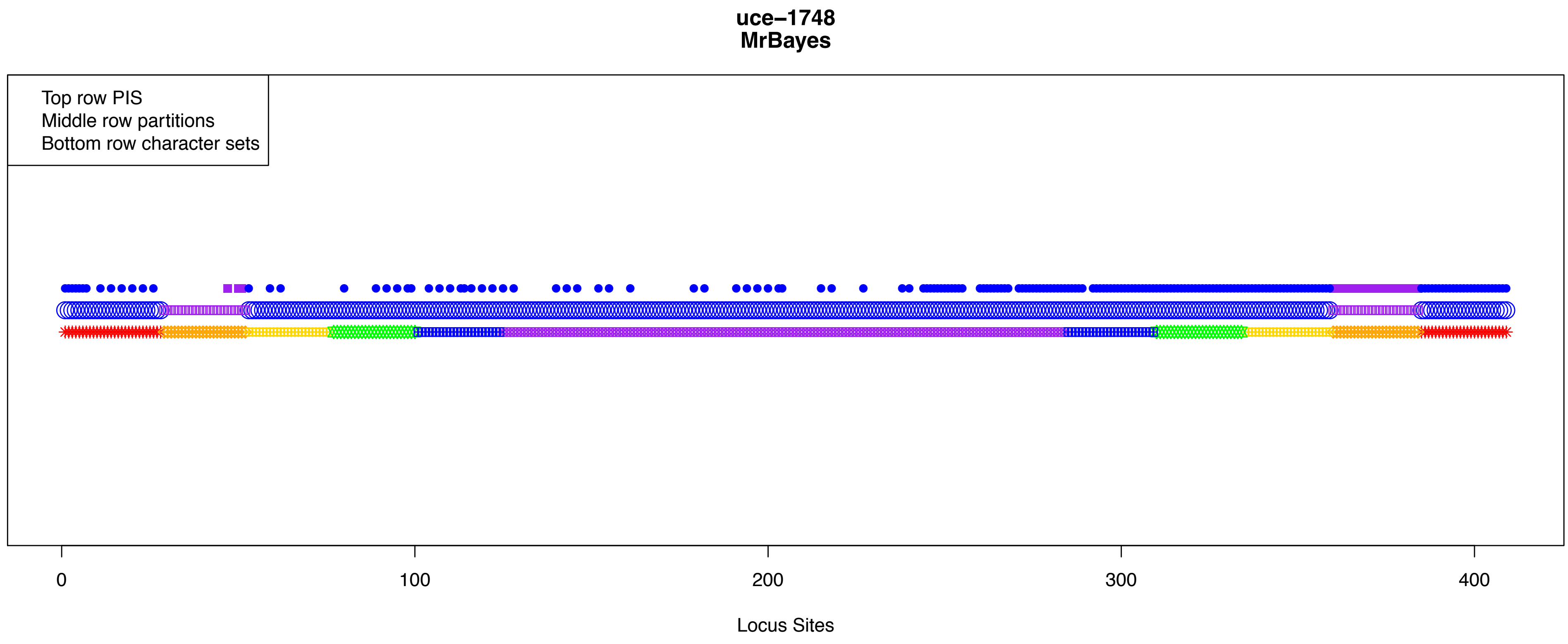
uce–1748
MrBayes
Top row PIS
Middle row partitions
Bottom row character sets
0
100
200
300
400
Locus Sites

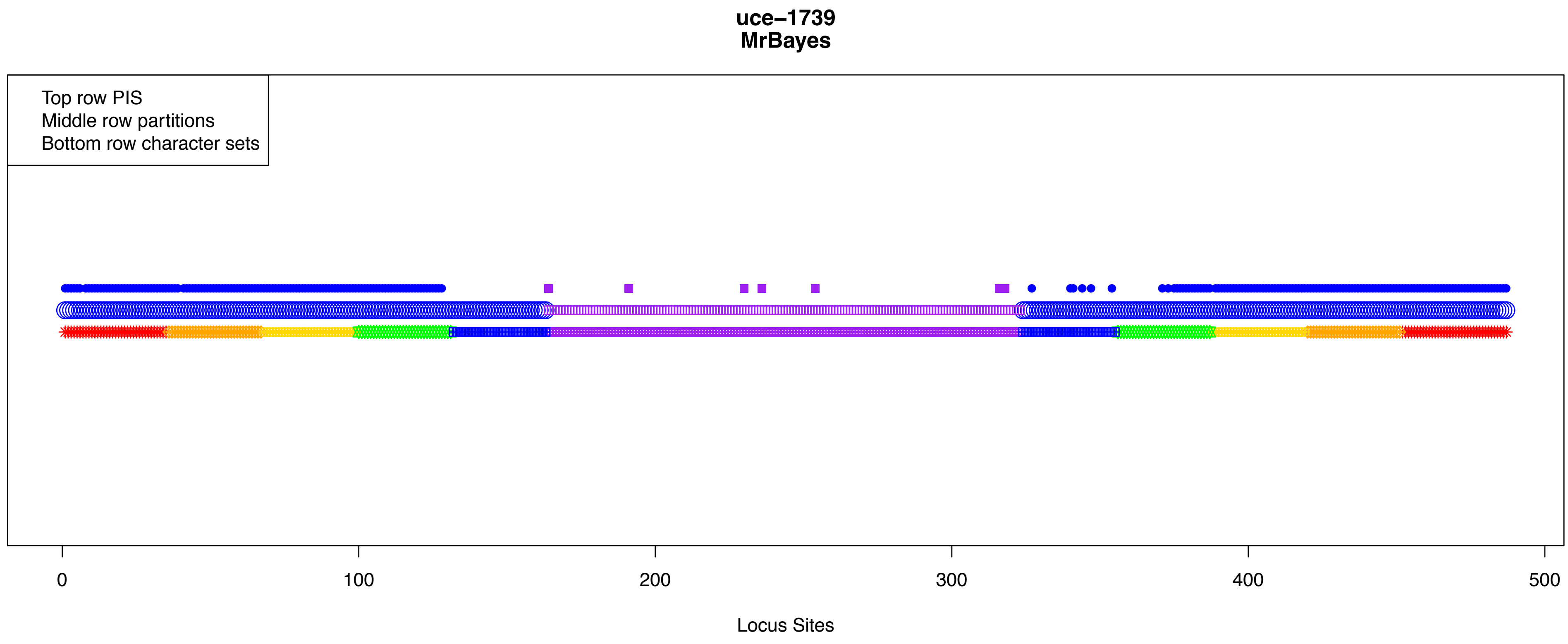
uce–1739
MrBayes
Top row PIS
Middle row partitions
Bottom row character sets
0
100
200
300
400
500
Locus Sites

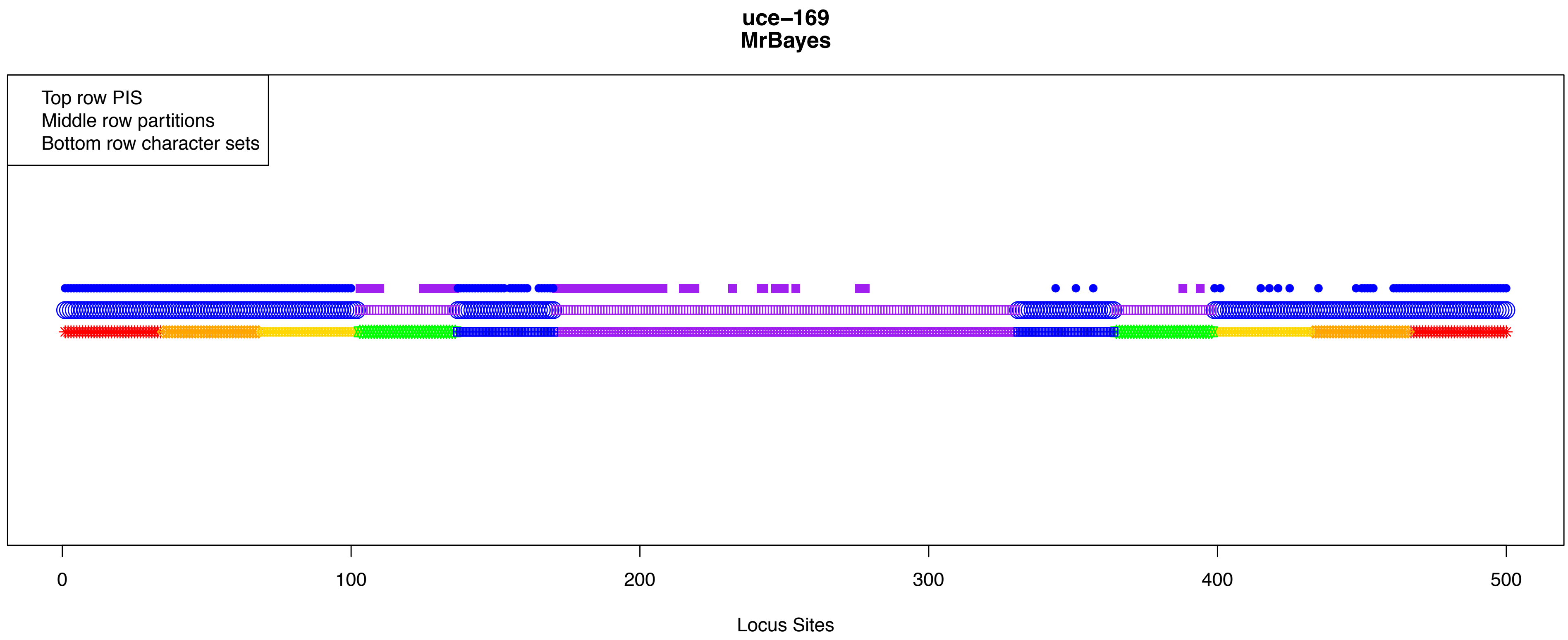

uce–169
MrBayes
Top row PIS
Middle row partitions
Bottom row character sets
0
100
200
300
400
500
Locus Sites

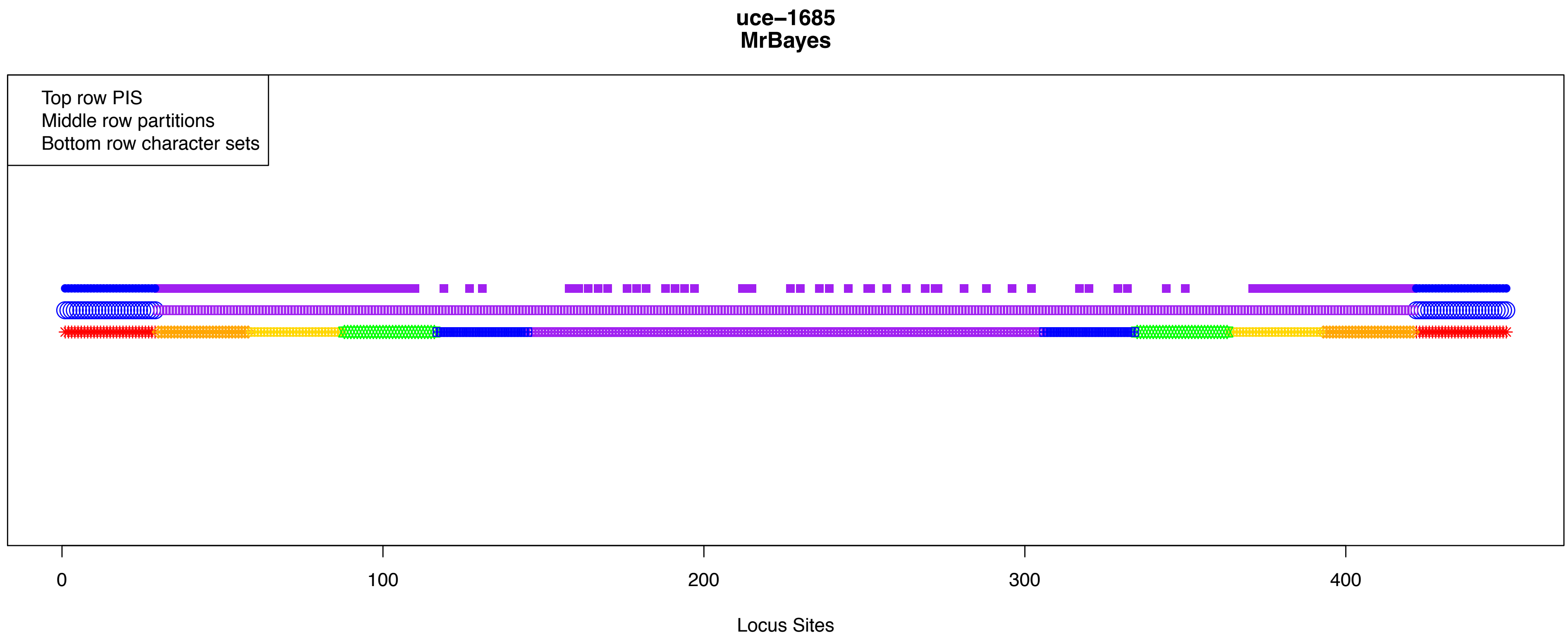
uce–1685
MrBayes
Top row PIS
Middle row partitions
Bottom row character sets
0
100
200
300
400
Locus Sites

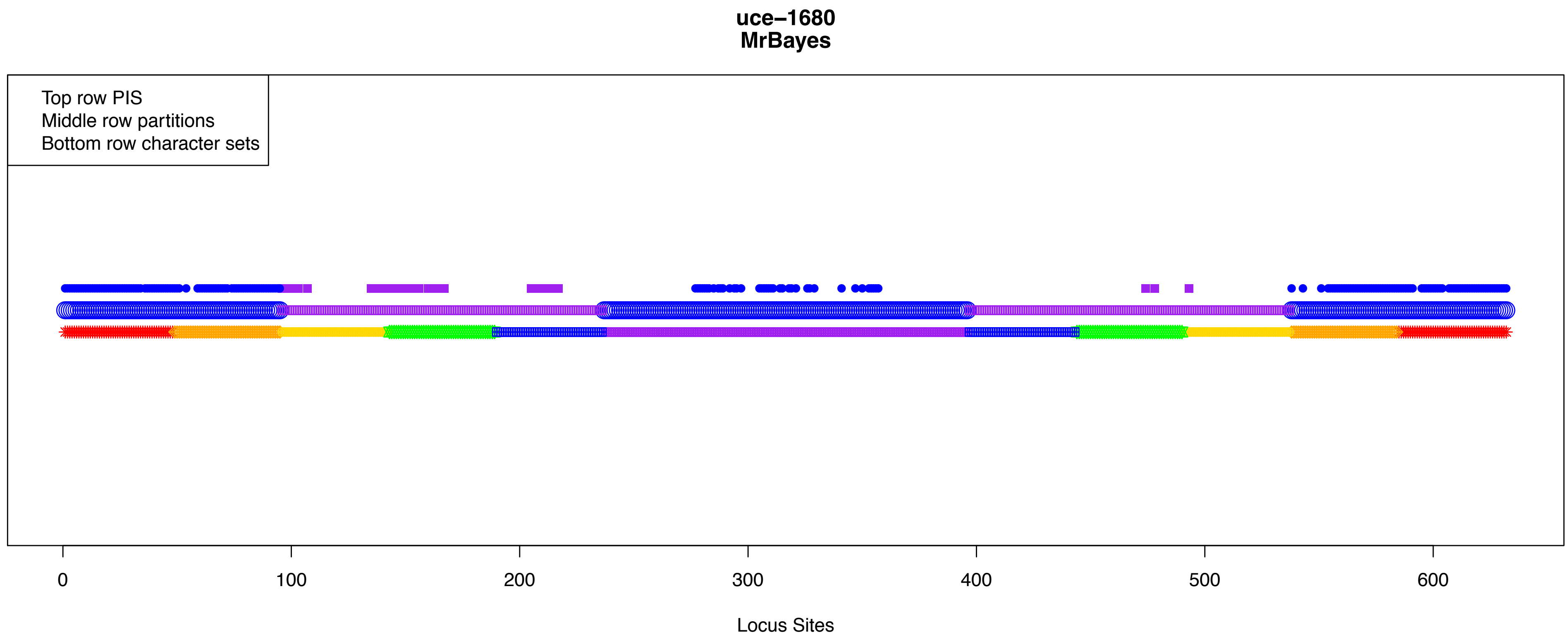

uce–1680
MrBayes
Top row PIS
Middle row partitions
Bottom row character sets
0
100
200
300
400
500
600
Locus Sites

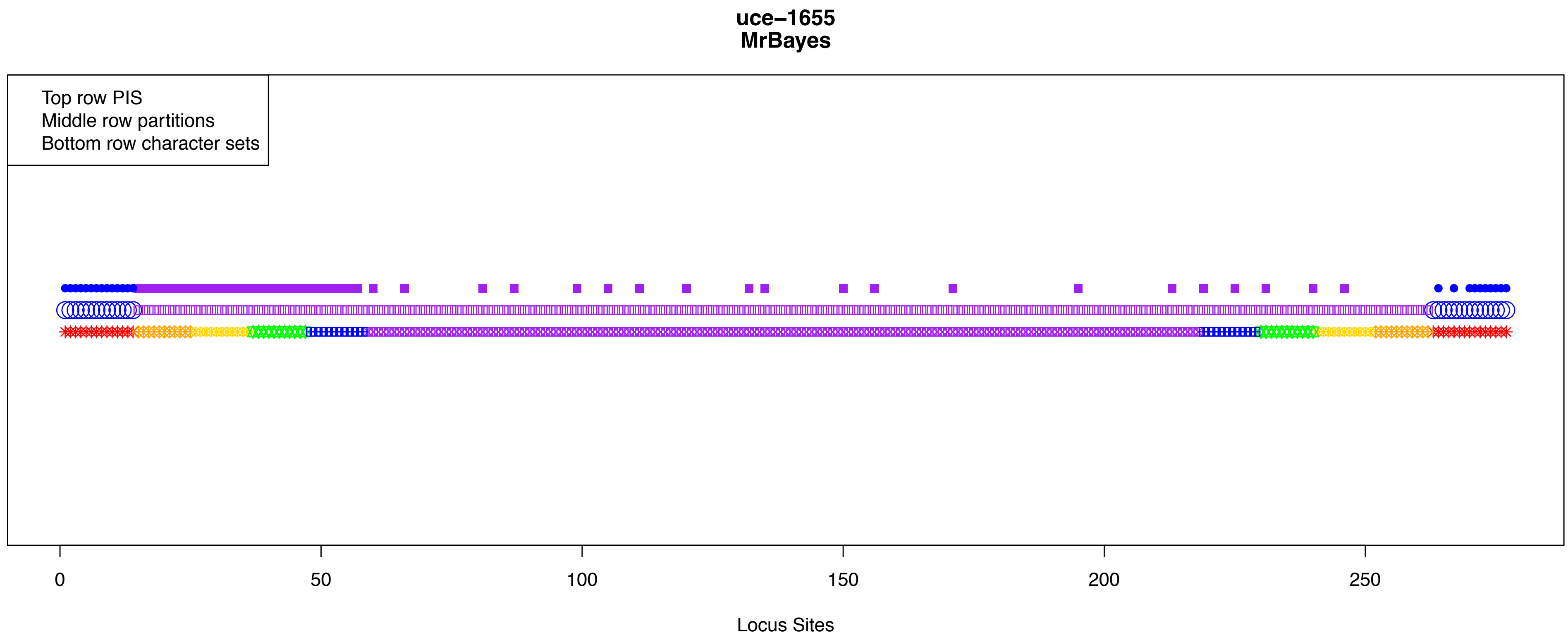
uce–1655
MrBayes
Top row PIS
Middle row partitions
Bottom row character sets
0
50
100
150
200
250
Locus Sites

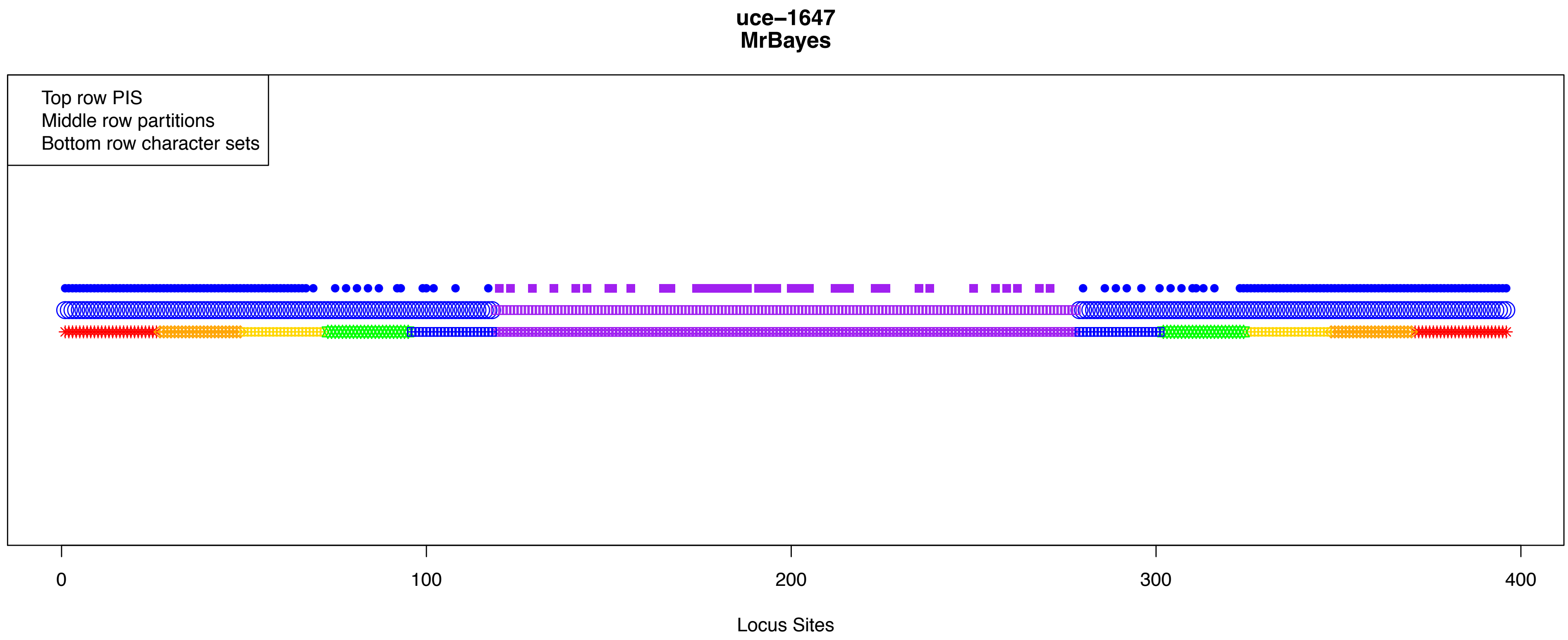
uce–1647
MrBayes
Top row PIS
Middle row partitions
Bottom row character sets
0
100
200
300
400
Locus Sites

uce–1645
MrBayes

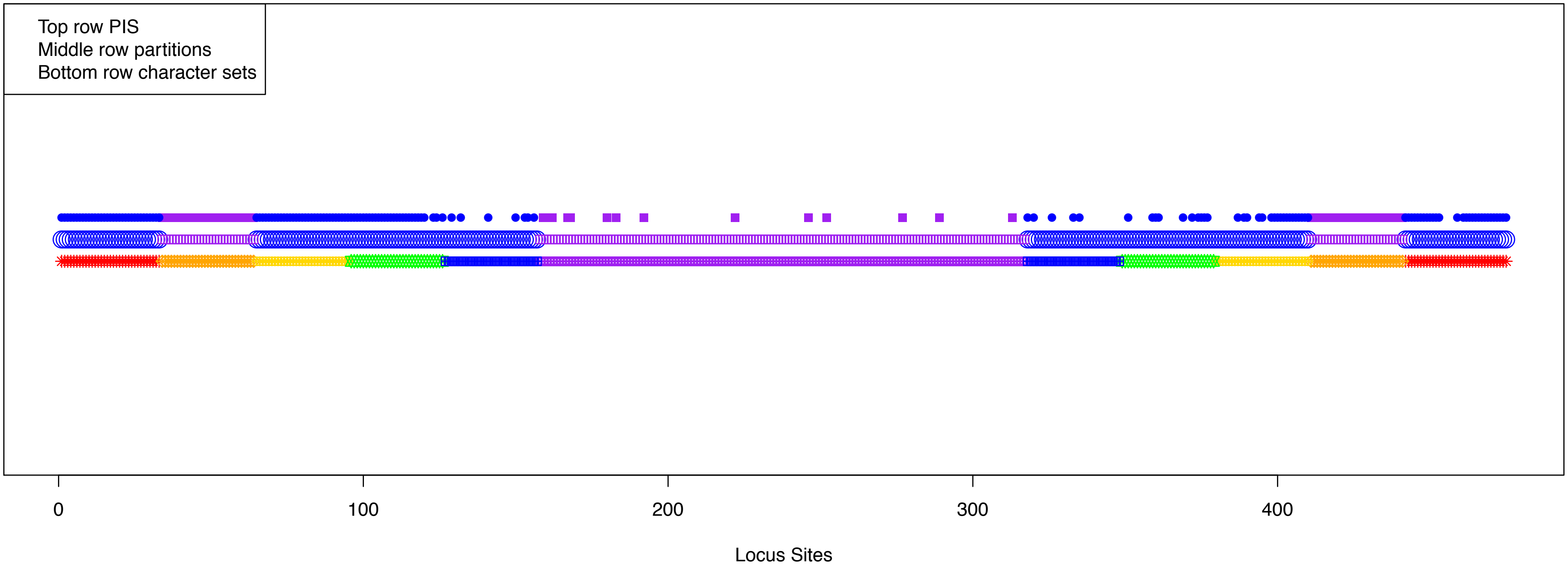

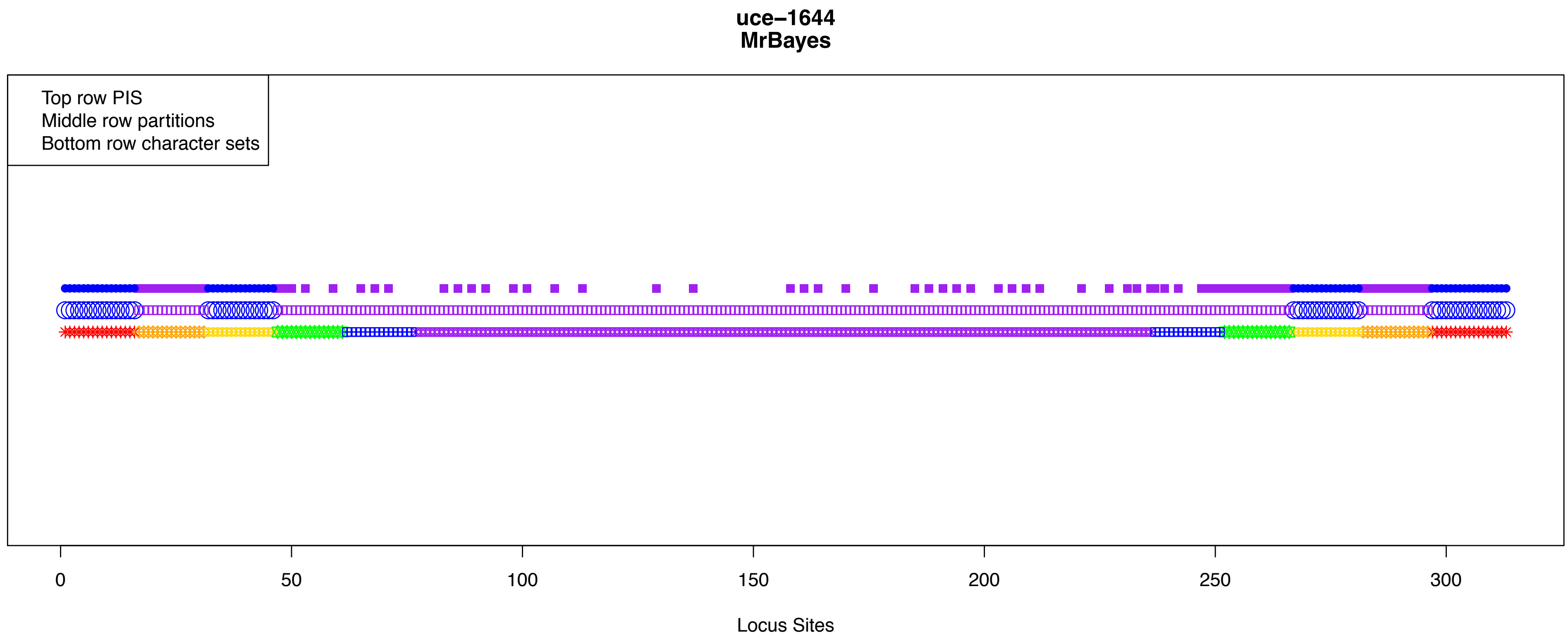
uce–1644
MrBayes
Top row PIS
Middle row partitions
Bottom row character sets
0
50
100
150
200
250
300
Locus Sites

uce–1608
MrBayes

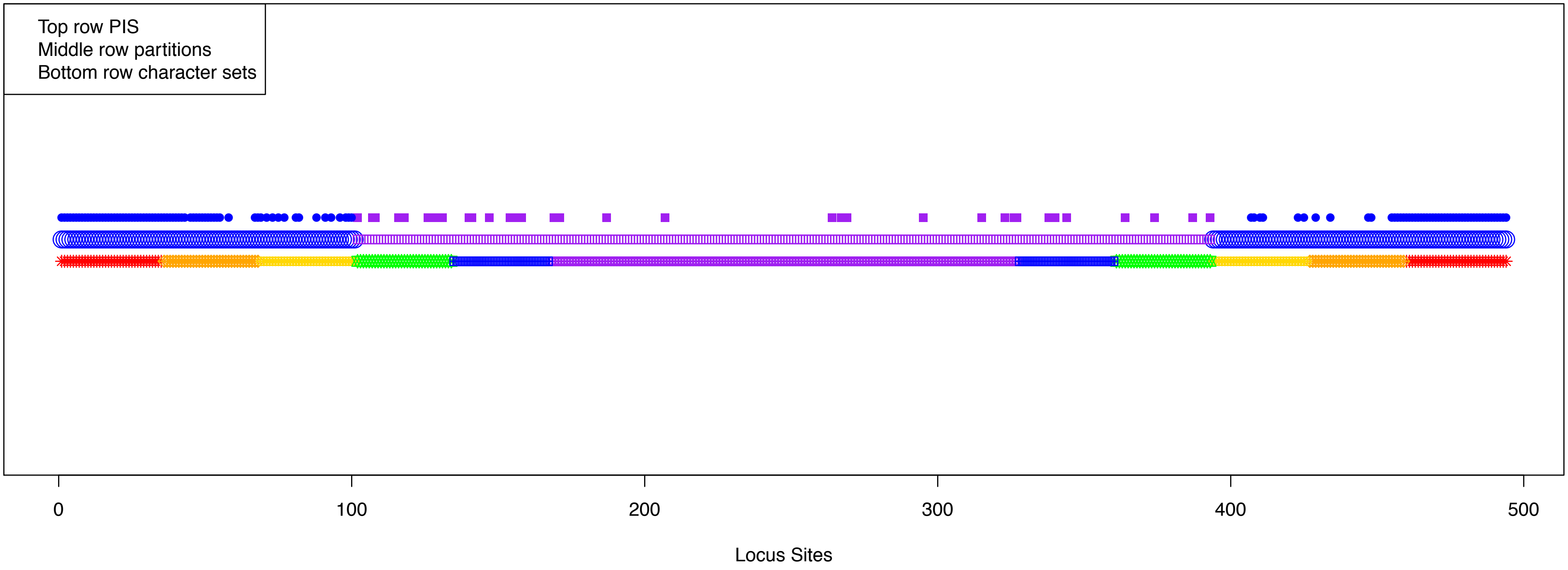

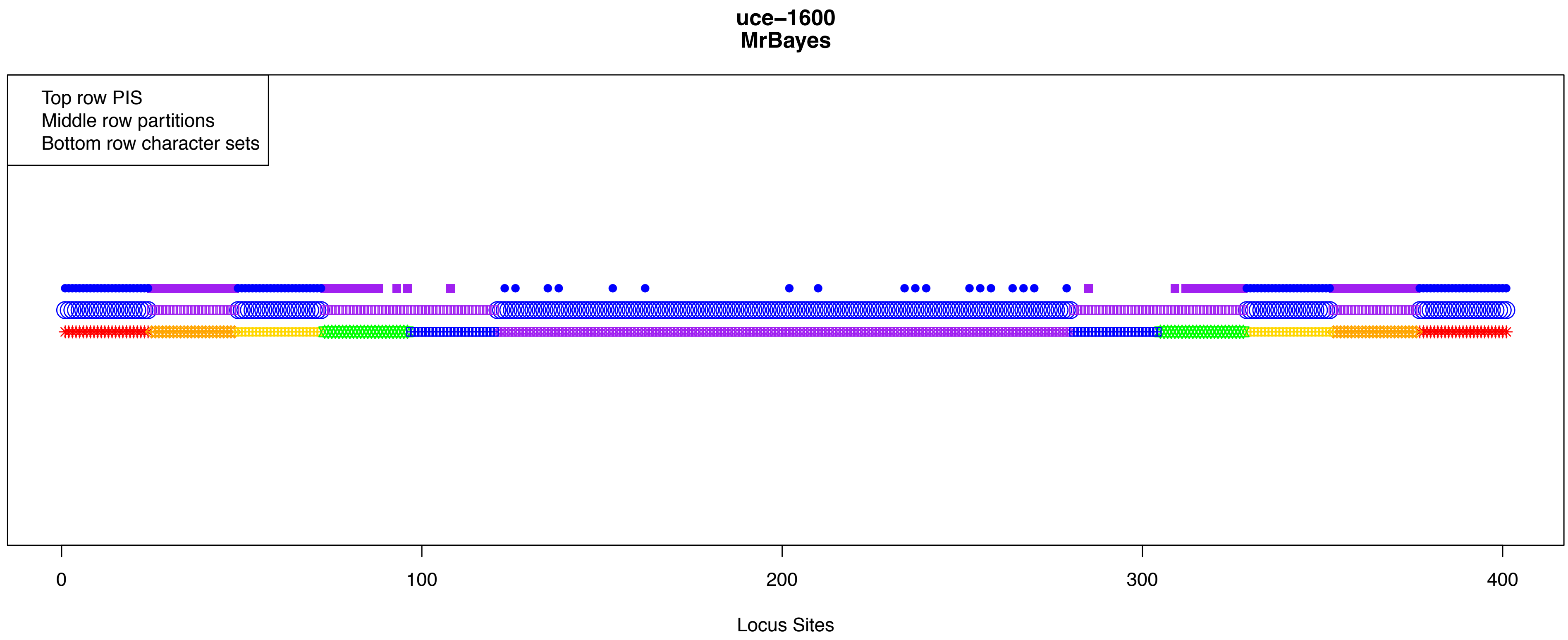
uce–1600
MrBayes
Top row PIS
Middle row partitions
Bottom row character sets
0
100
200
300
400
Locus Sites

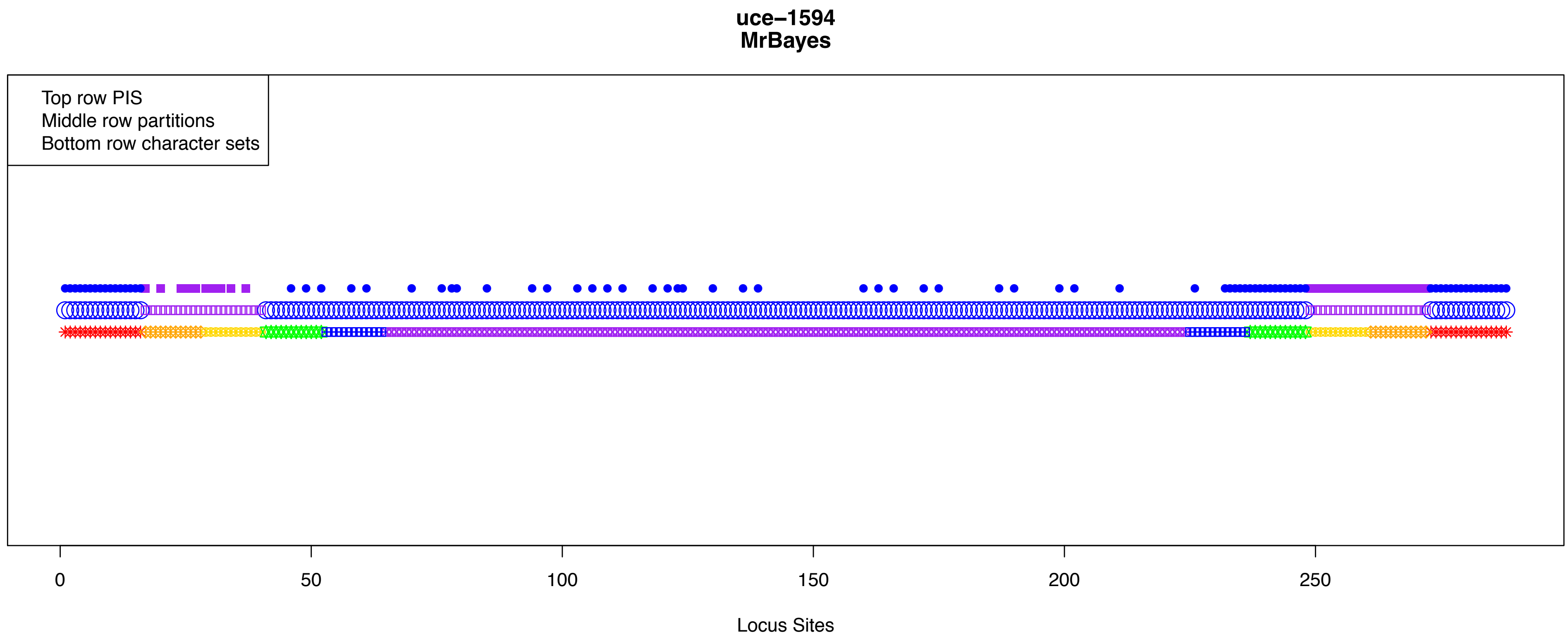

uce–1594
MrBayes
Top row PIS
Middle row partitions
Bottom row character sets
0
50
100
150
200
250
Locus Sites

uce–1591
MrBayes

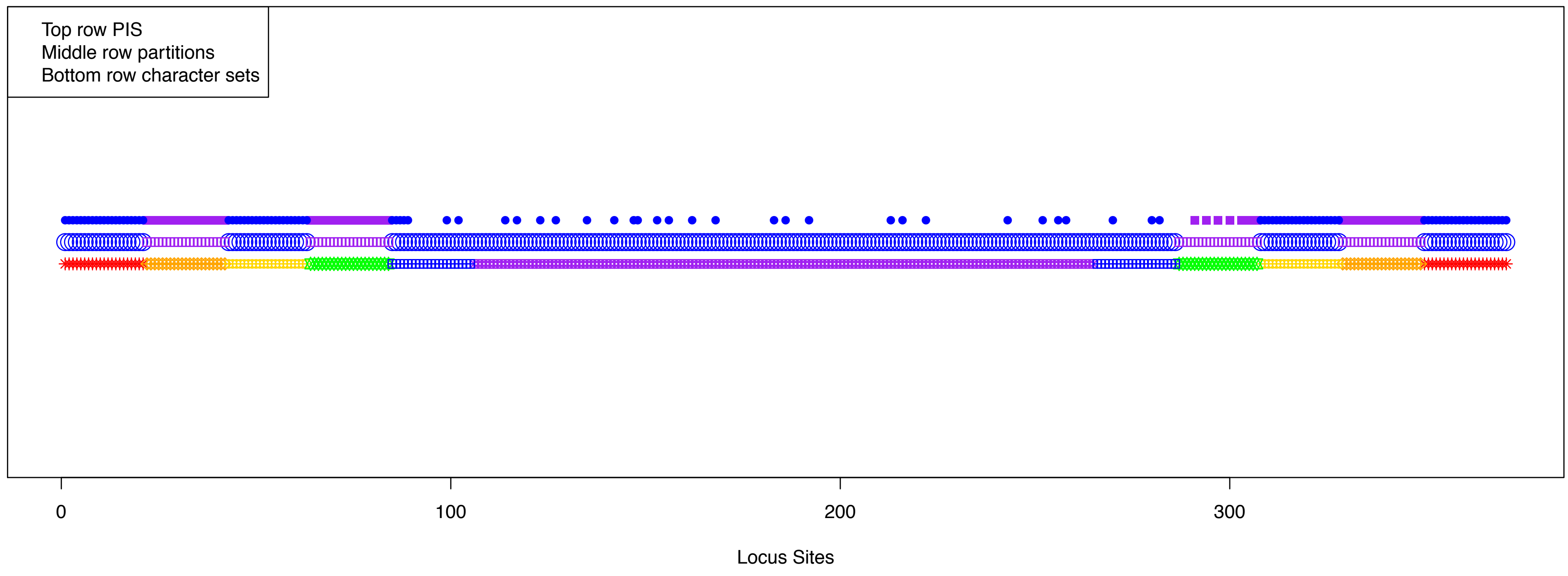

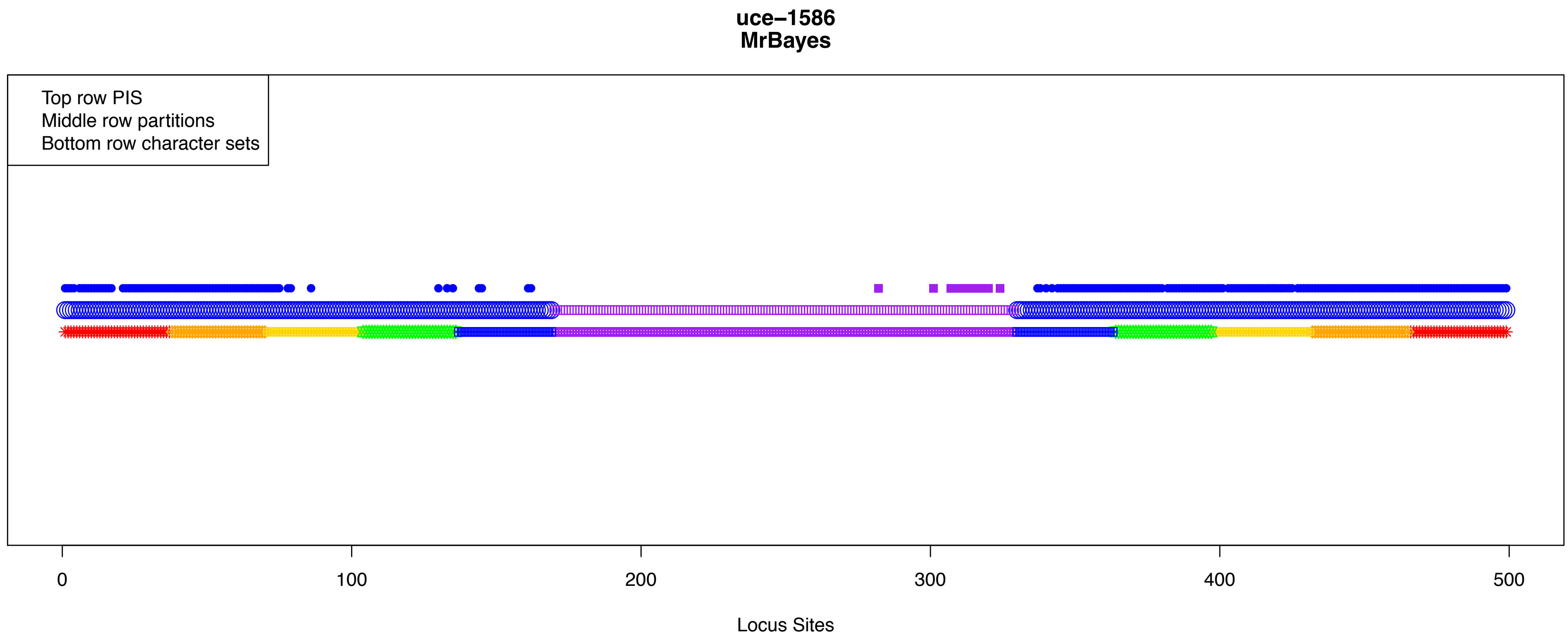
uce–1586
MrBayes
Top row PIS
Middle row partitions
Bottom row character sets
0
100
200
300
400
500
Locus Sites

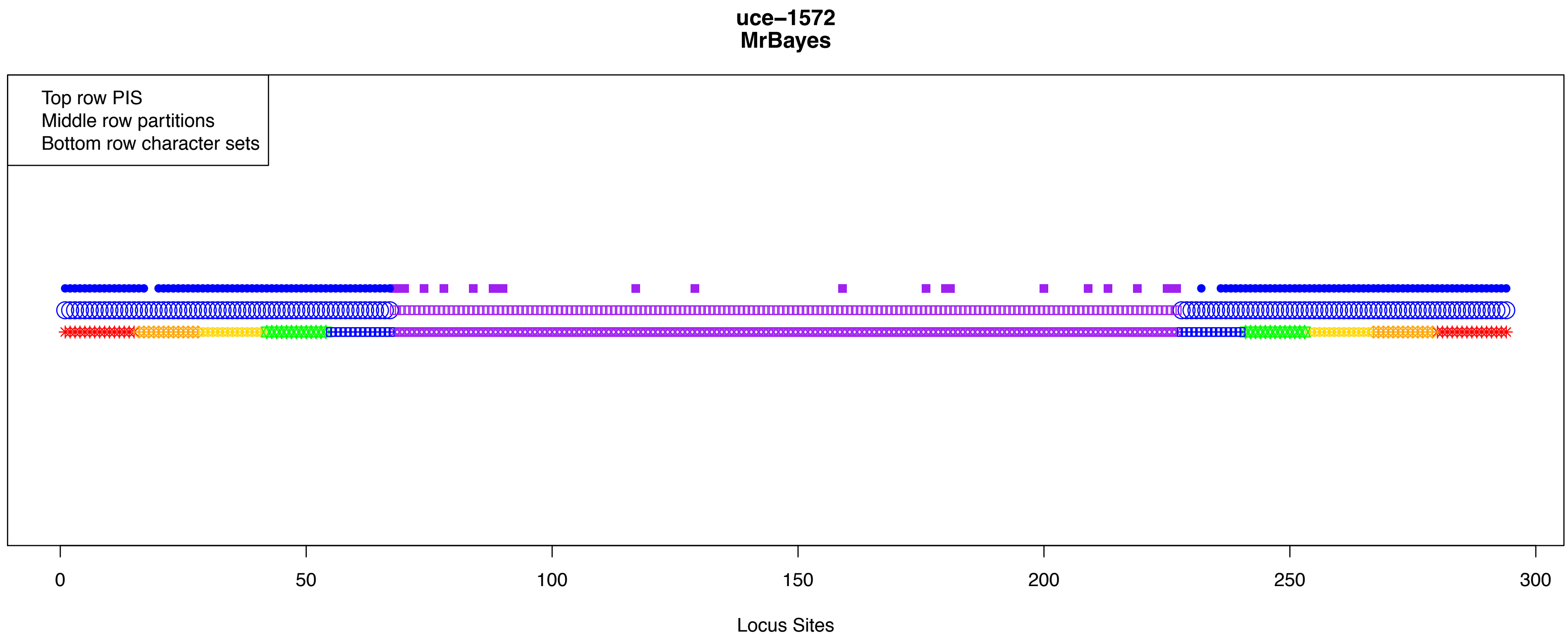

uce–1572
MrBayes
Top row PIS
Middle row partitions
Bottom row character sets
0
50
100
150
200
250
300
Locus Sites

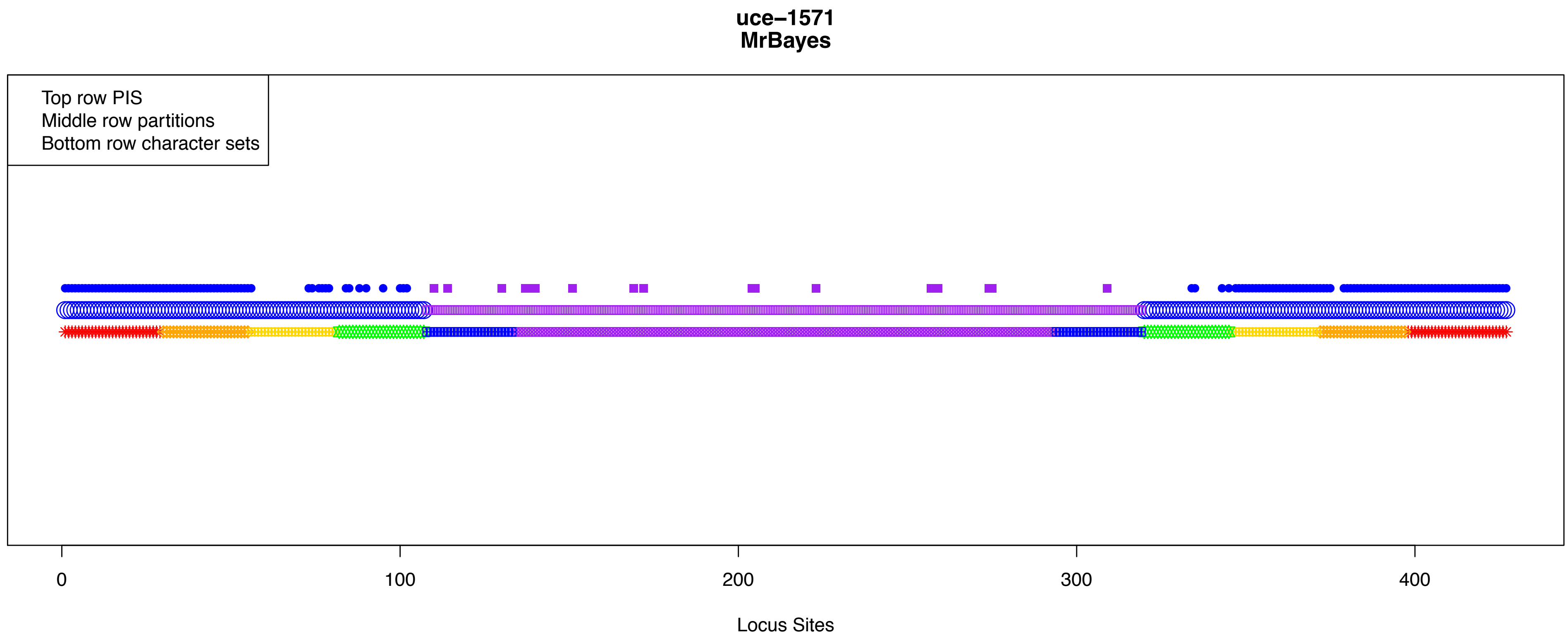
uce–1571
MrBayes
Top row PIS
Middle row partitions
Bottom row character sets
0
100
200
300
400
Locus Sites

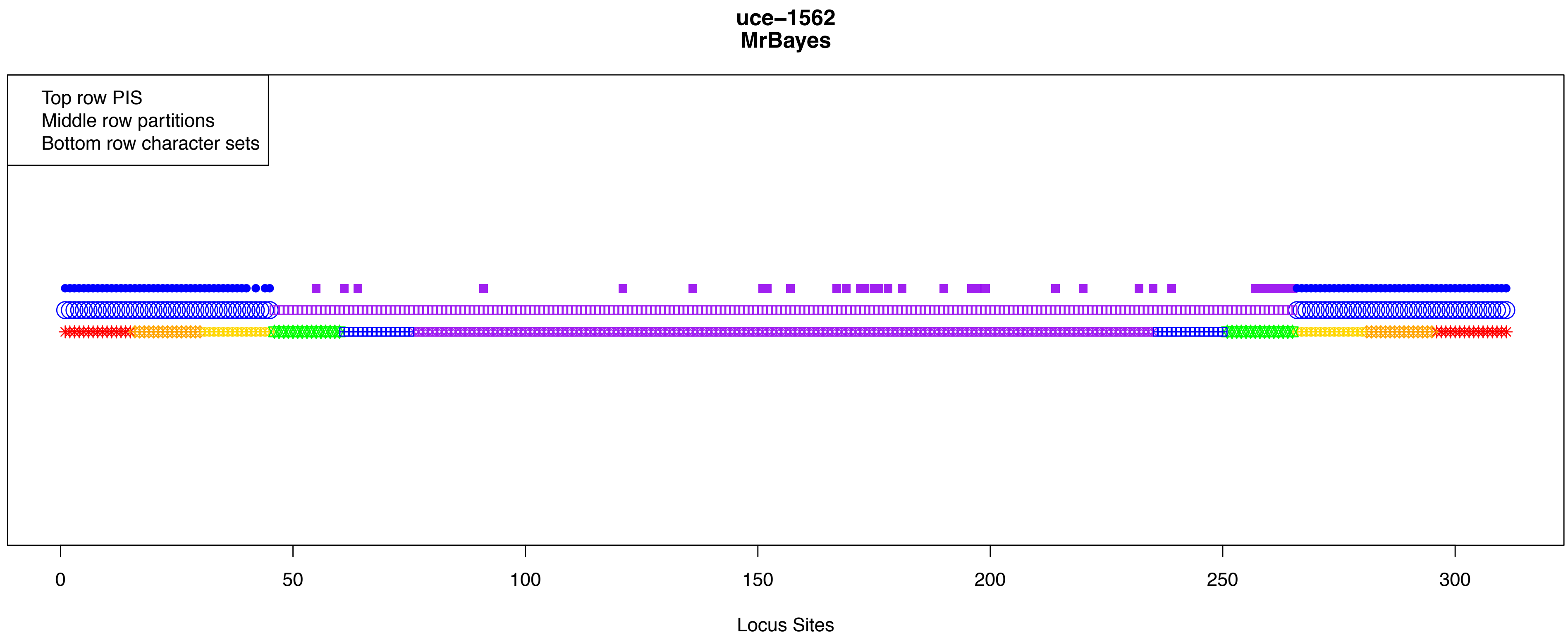

uce–1562
MrBayes
Top row PIS
Middle row partitions
Bottom row character sets
0
50
100
150
200
250
300
Locus Sites

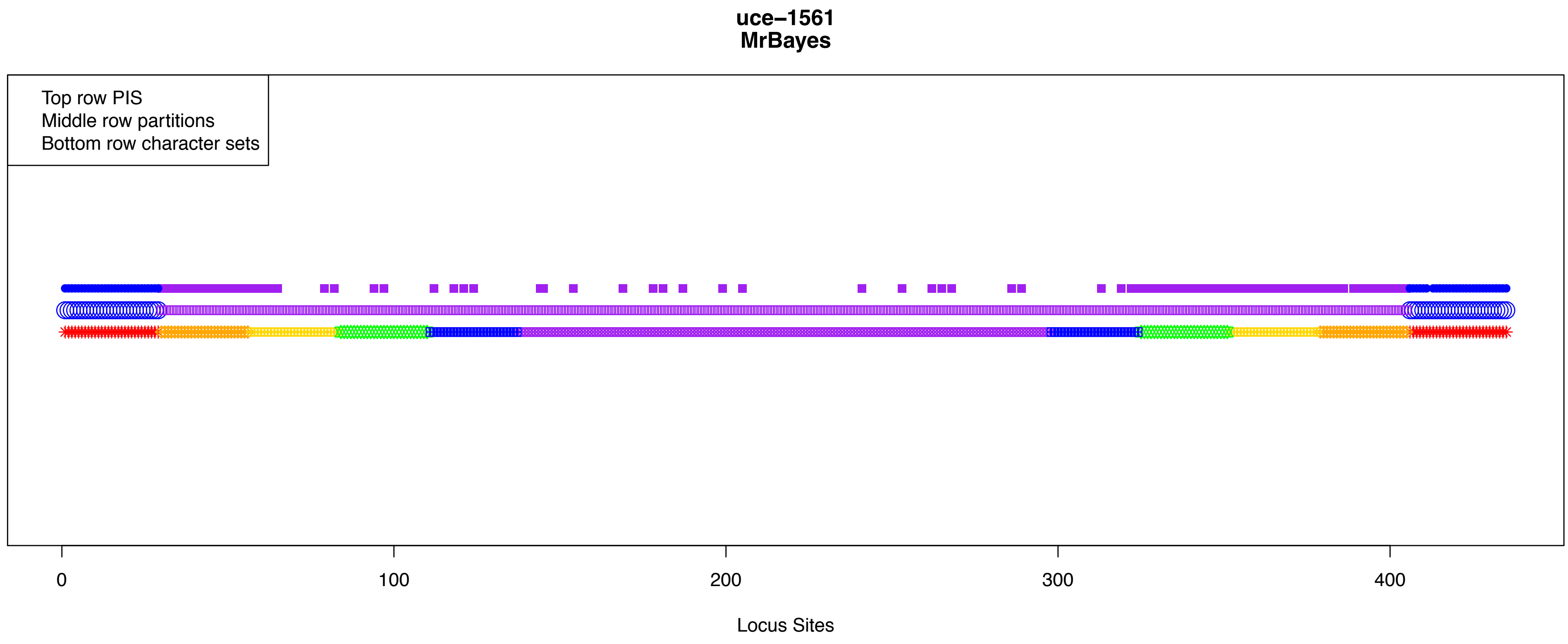

uce–1561
MrBayes
Top row PIS
Middle row partitions
Bottom row character sets
0
100
200
300
400
Locus Sites

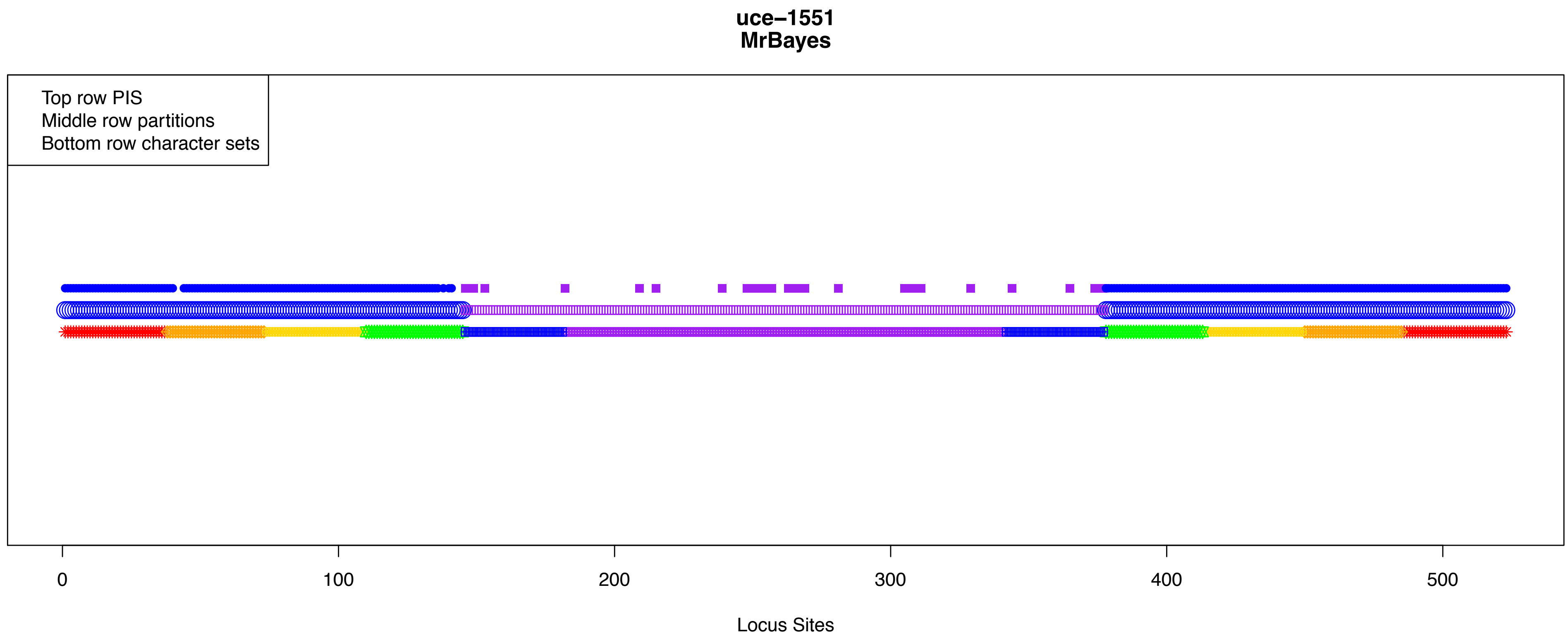

uce–1551
MrBayes
Top row PIS
Middle row partitions
Bottom row character sets
0
100
200
300
400
500
Locus Sites

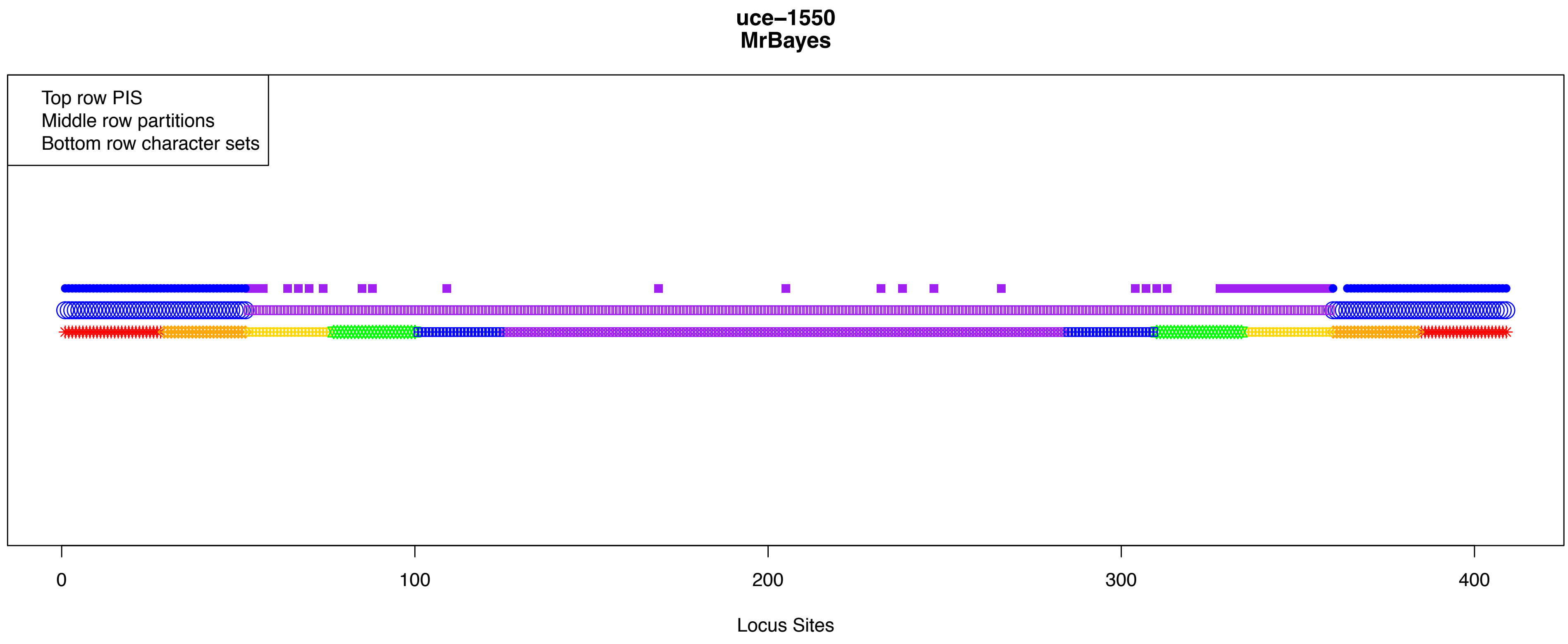
uce–1550
MrBayes
Top row PIS
Middle row partitions
Bottom row character sets
0
100
200
300
400
Locus Sites

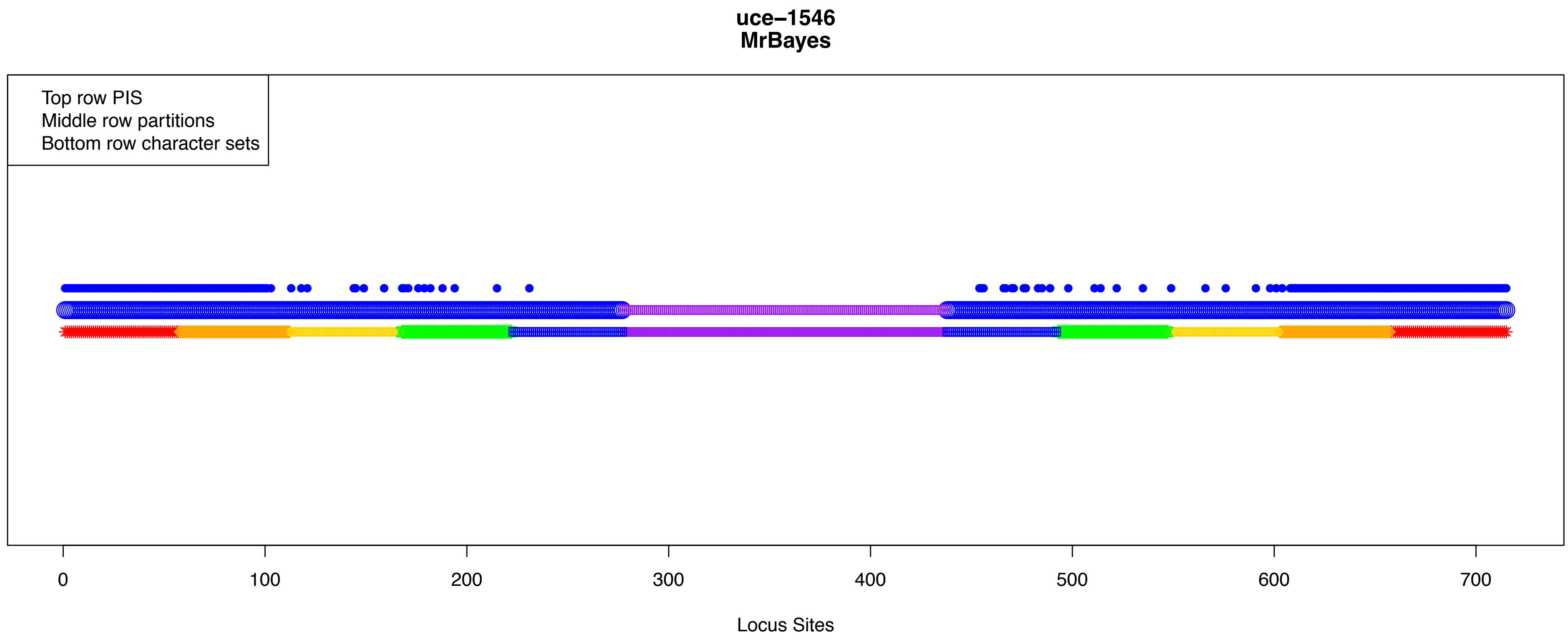

uce–1546
MrBayes
Top row PIS
Middle row partitions
Bottom row character sets
0
100
200
300
400
500
600
700
Locus Sites

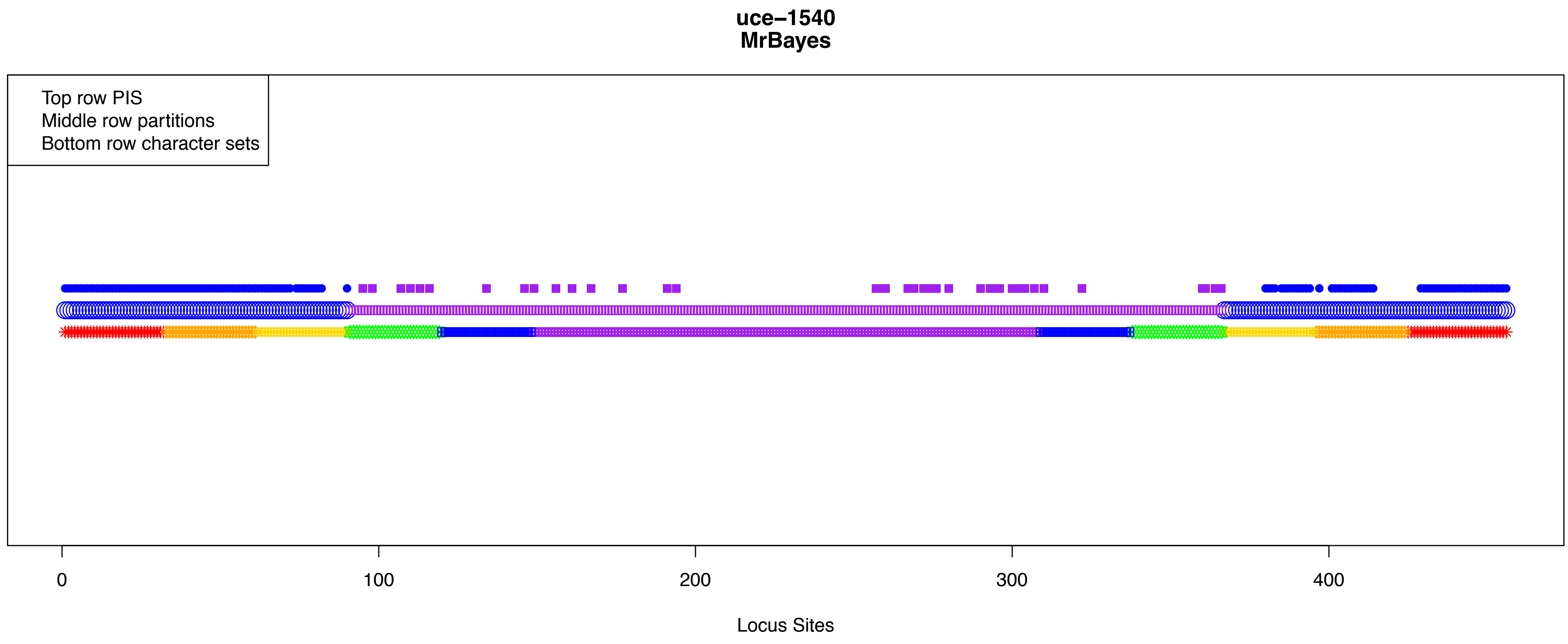
uce–1540
MrBayes
Top row PIS
Middle row partitions
Bottom row character sets
0
100
200
300
400
Locus Sites

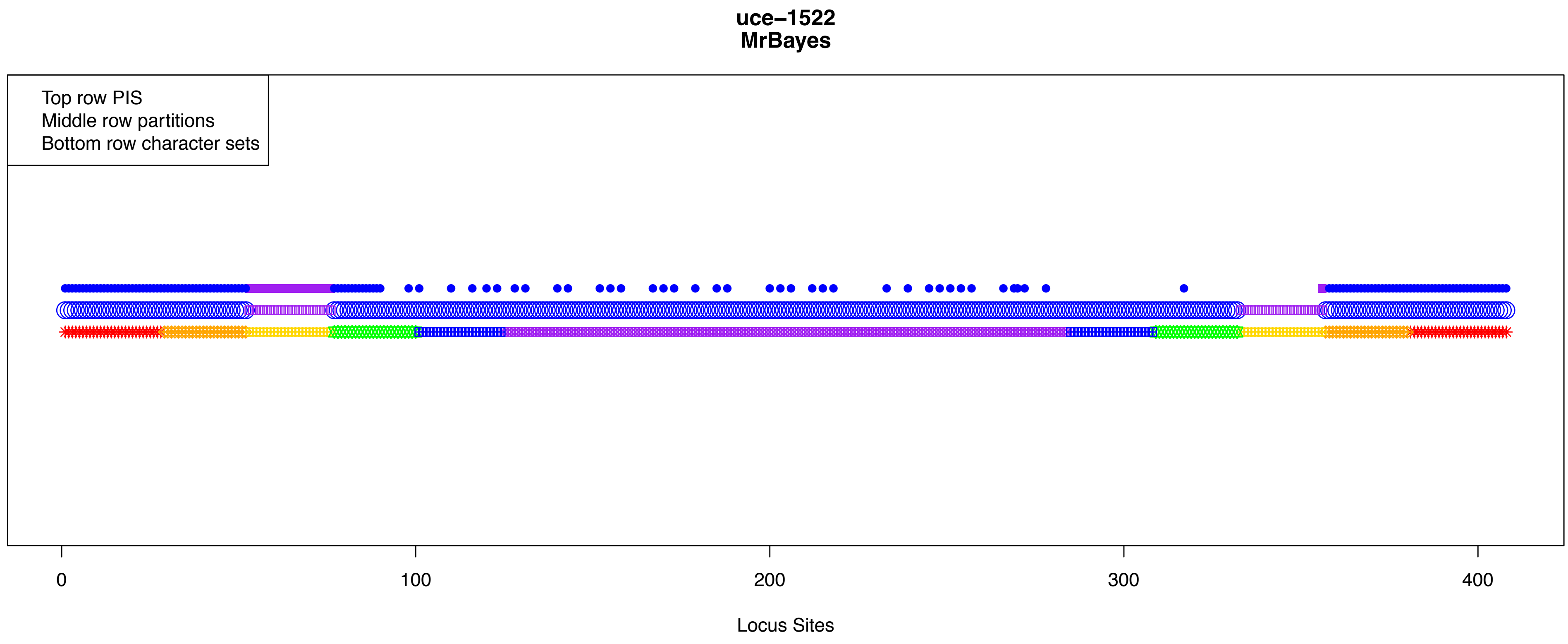
uce–1522
MrBayes
Top row PIS
Middle row partitions
Bottom row character sets
0
100
200
300
400
Locus Sites

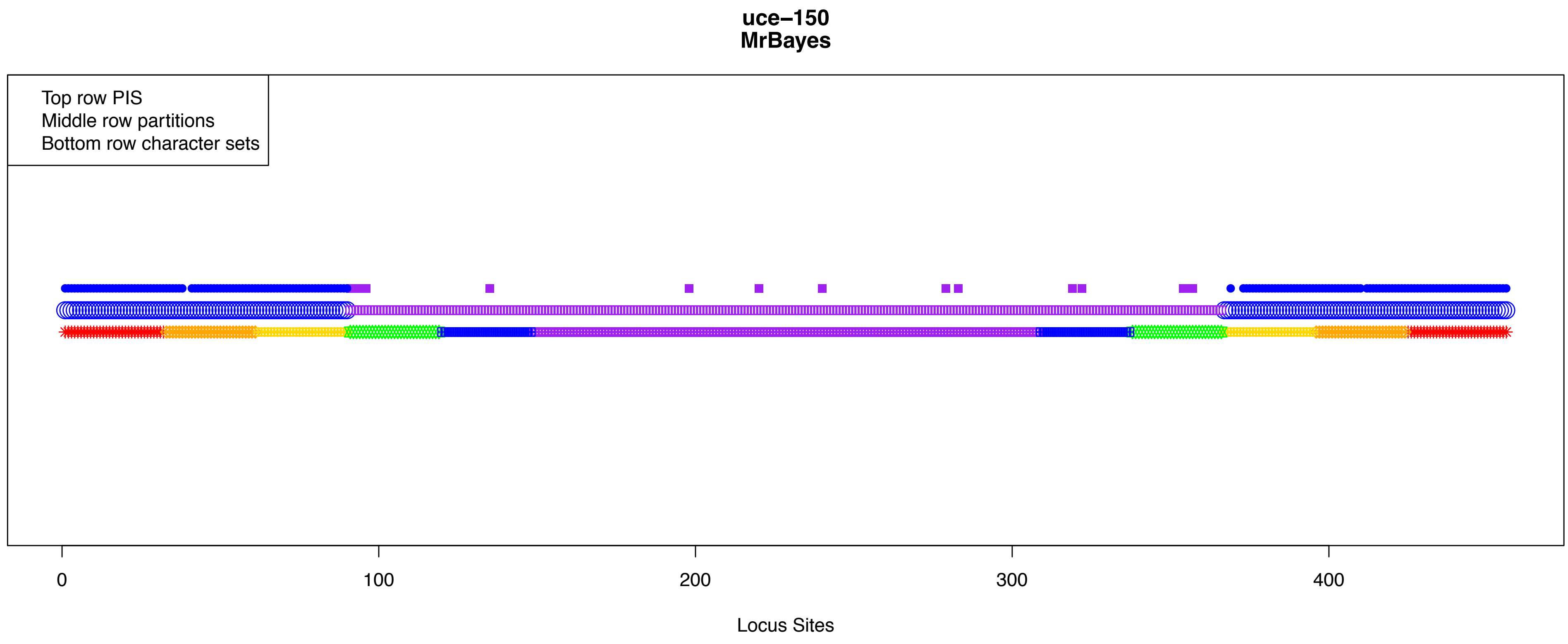
uce–150
MrBayes
Top row PIS
Middle row partitions
Bottom row character sets
0
100
200
300
400
Locus Sites

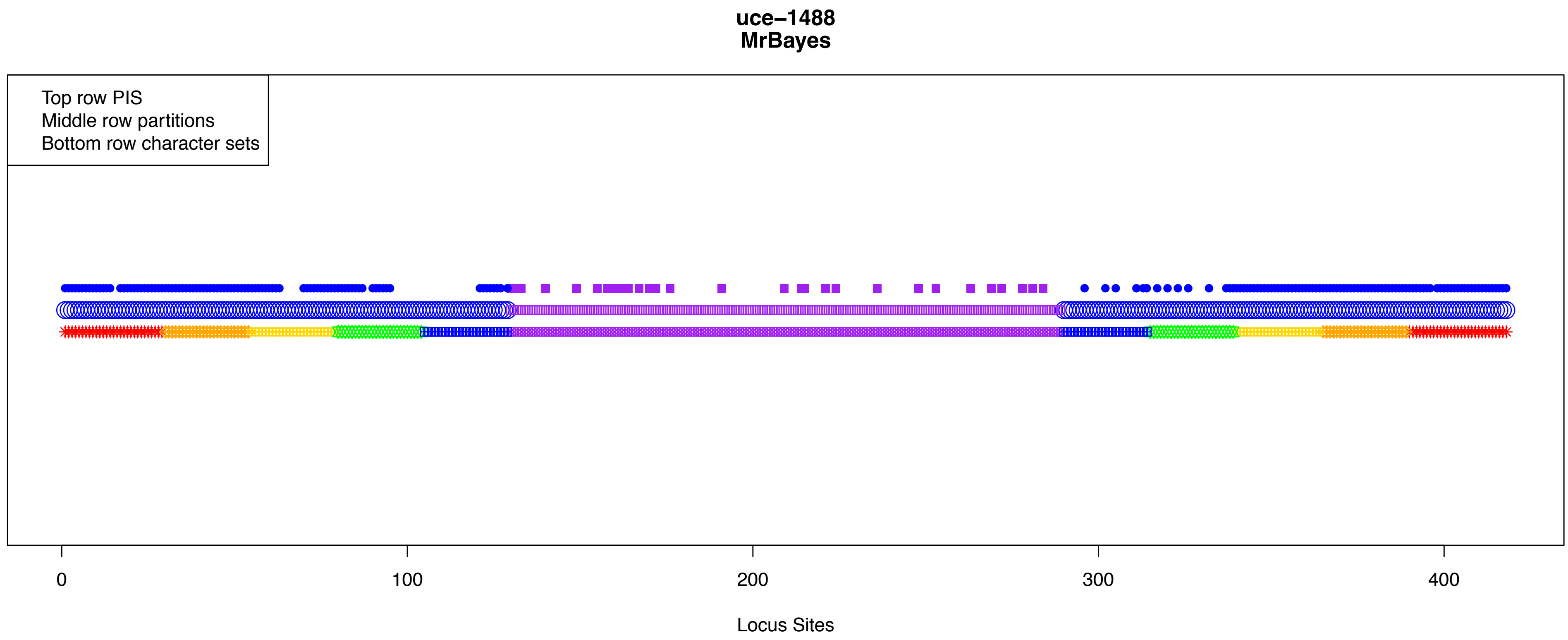

uce–1488
MrBayes
Top row PIS
Middle row partitions
Bottom row character sets
0
100
200
300
400
Locus Sites

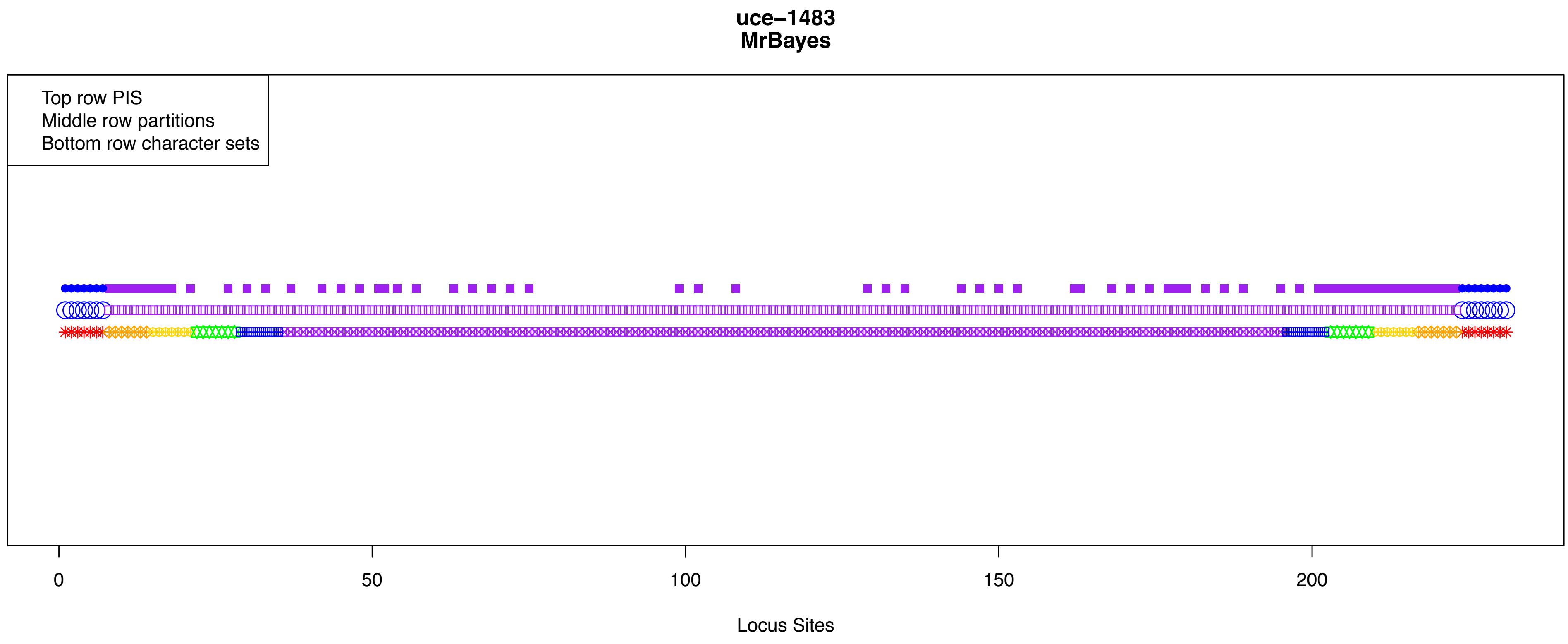
uce–1483
MrBayes
Top row PIS
Middle row partitions
Bottom row character sets
0
50
100
150
200
Locus Sites

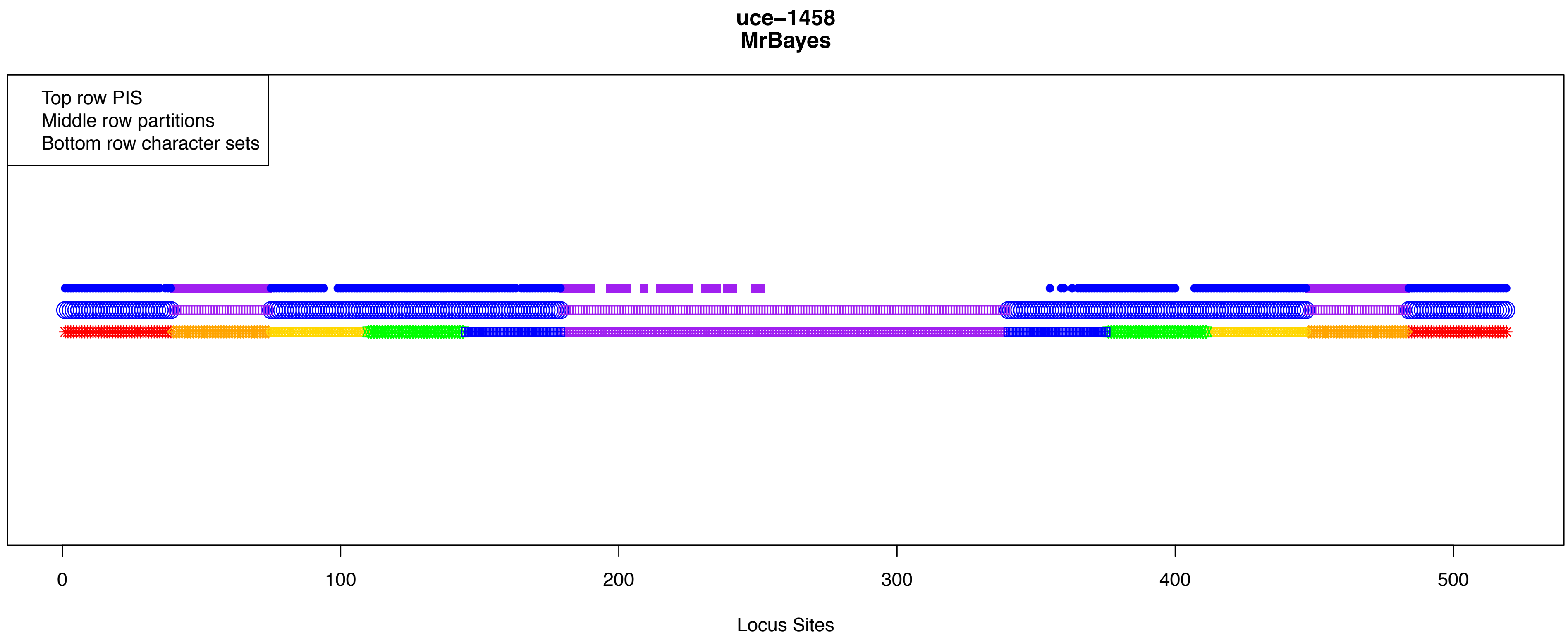
uce–1458
MrBayes
Top row PIS
Middle row partitions
Bottom row character sets
0
100
200
300
400
500
Locus Sites

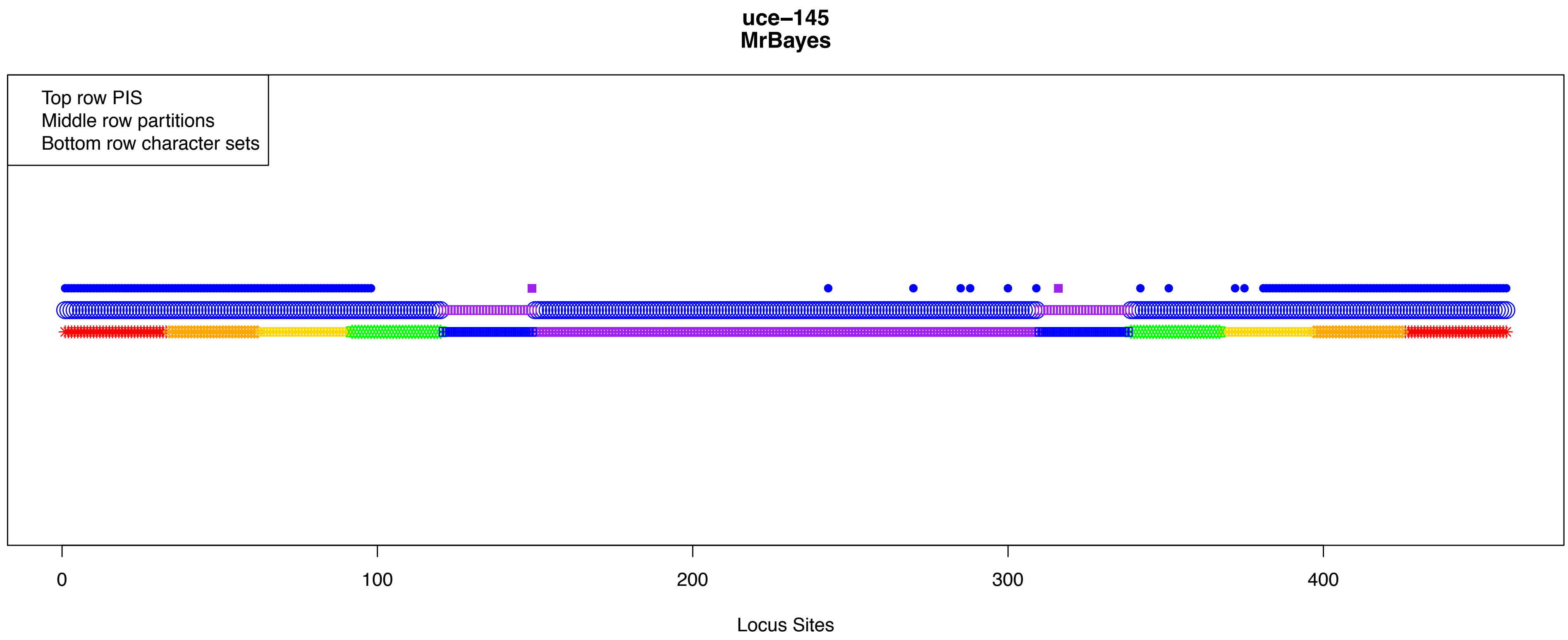

uce–145
MrBayes
Top row PIS
Middle row partitions
Bottom row character sets
0
100
200
300
400
Locus Sites

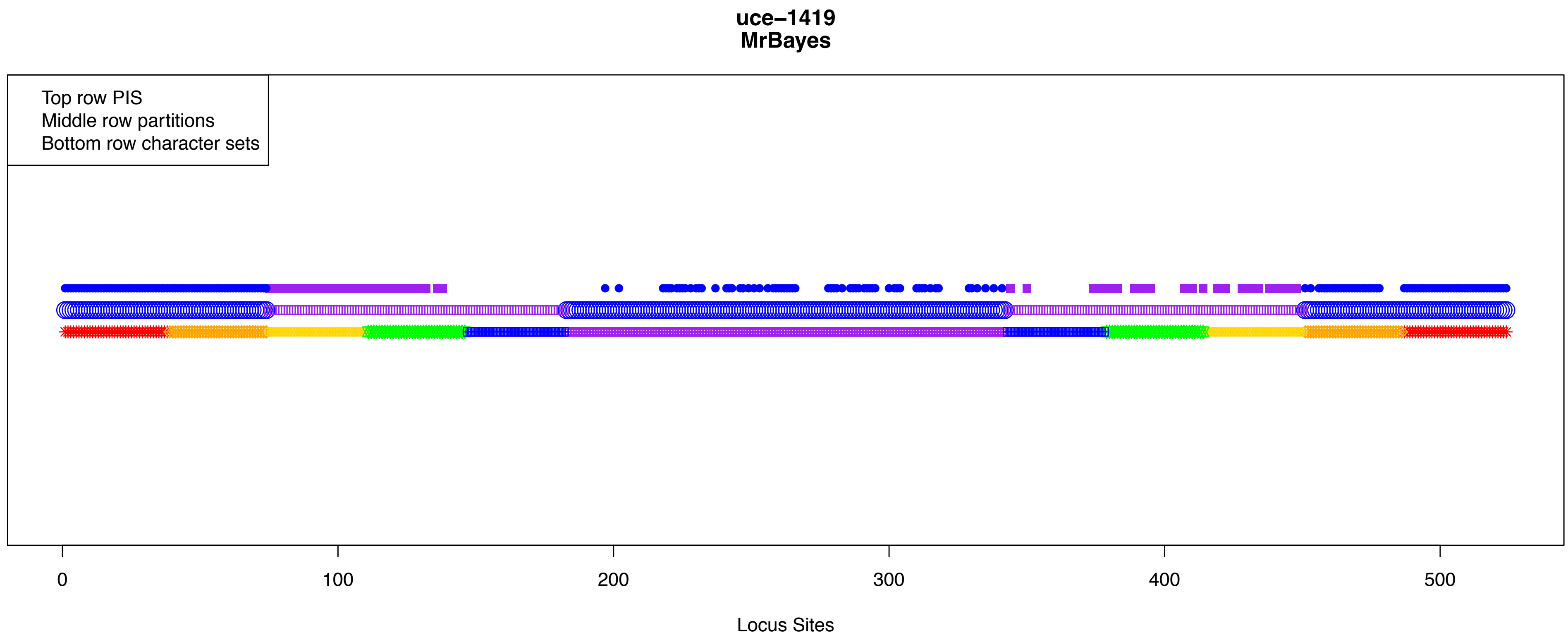

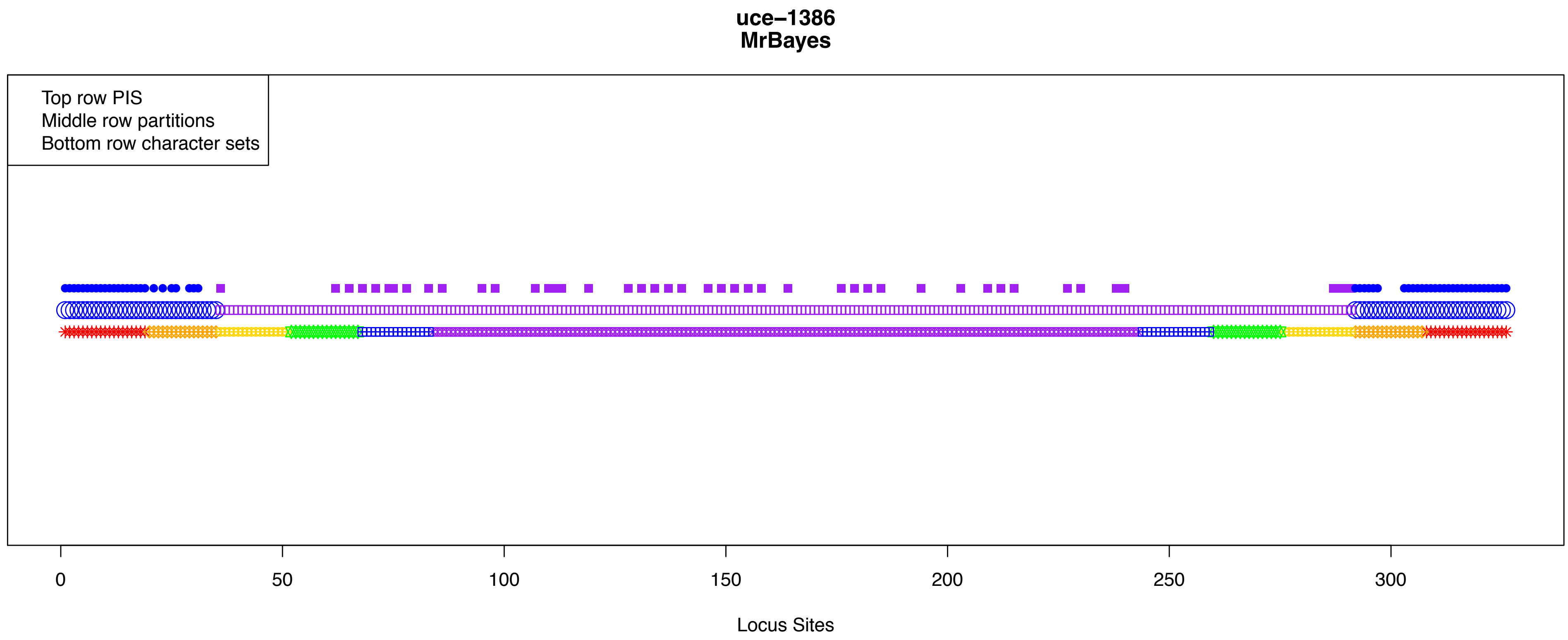
uce–1386
MrBayes
Top row PIS
Middle row partitions
Bottom row character sets
0
50
100
150
200
250
300
Locus Sites

uce–1382
MrBayes

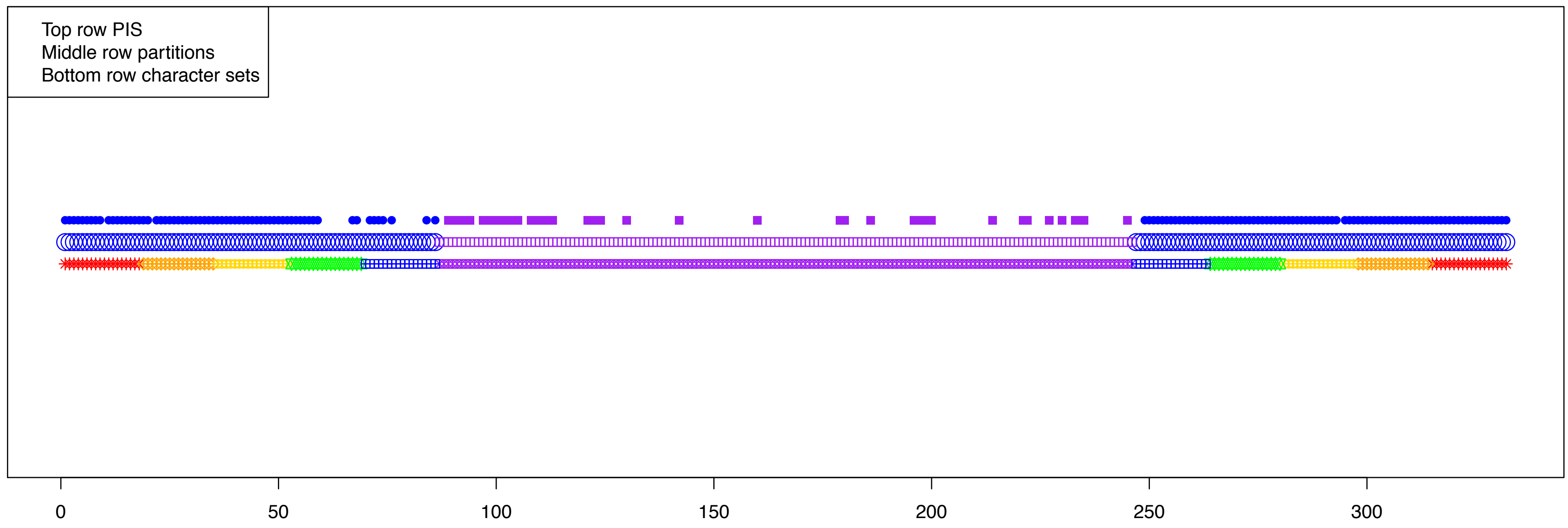

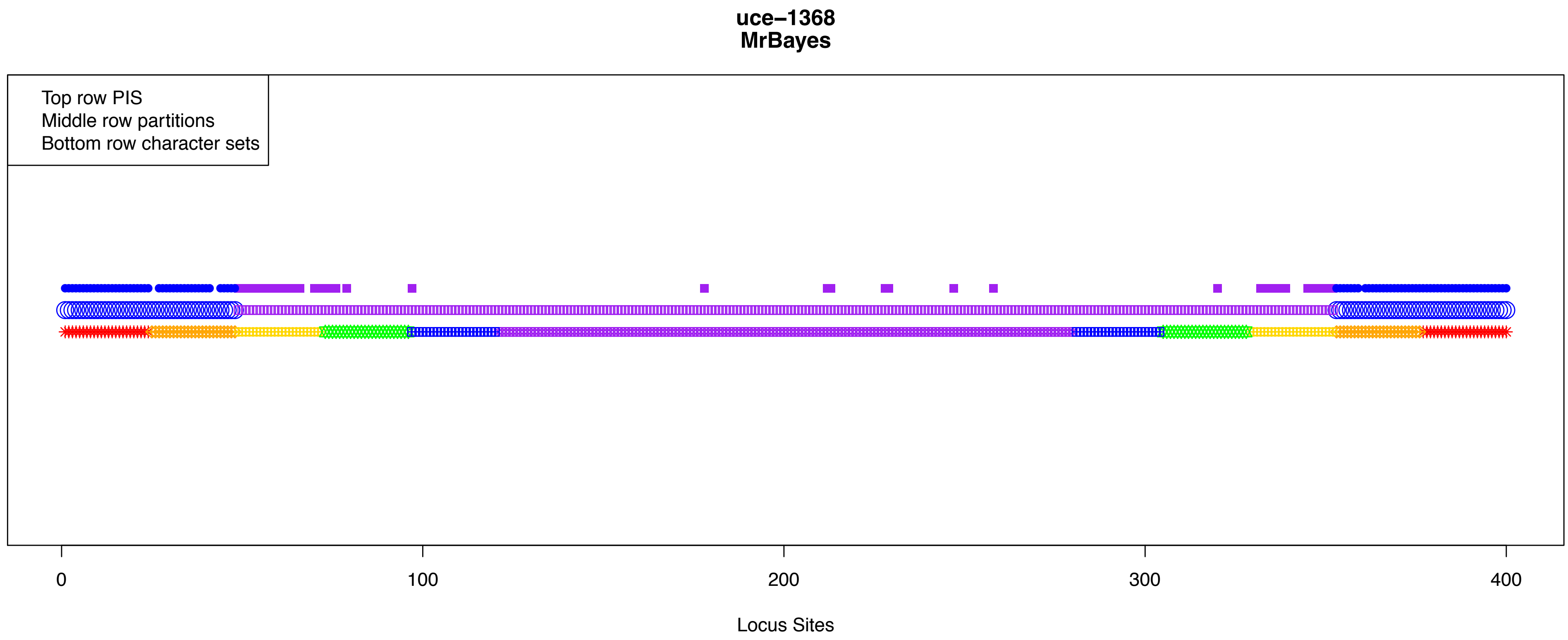
uce–1368
MrBayes
Top row PIS
Middle row partitions
Bottom row character sets
0
100
200
300
400
Locus Sites

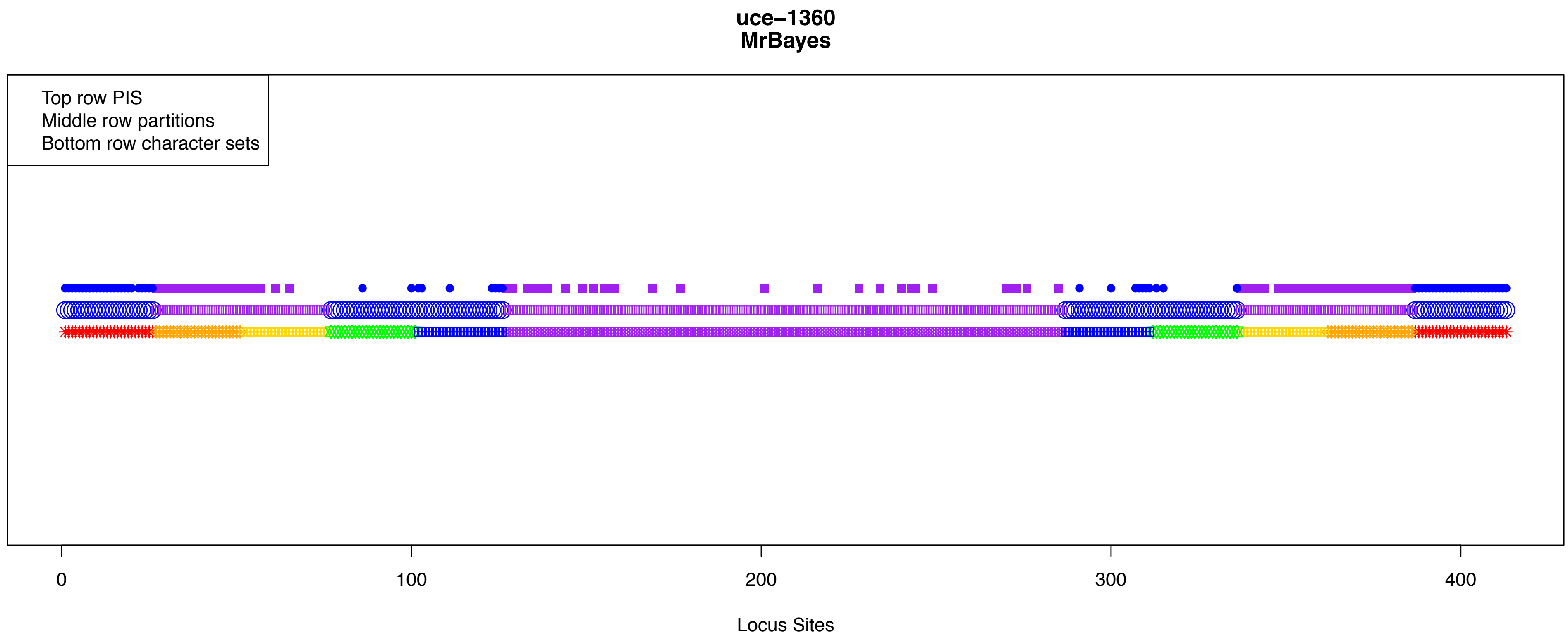
uce–1360
MrBayes
Top row PIS
Middle row partitions
Bottom row character sets
0
100
200
300
400
Locus Sites

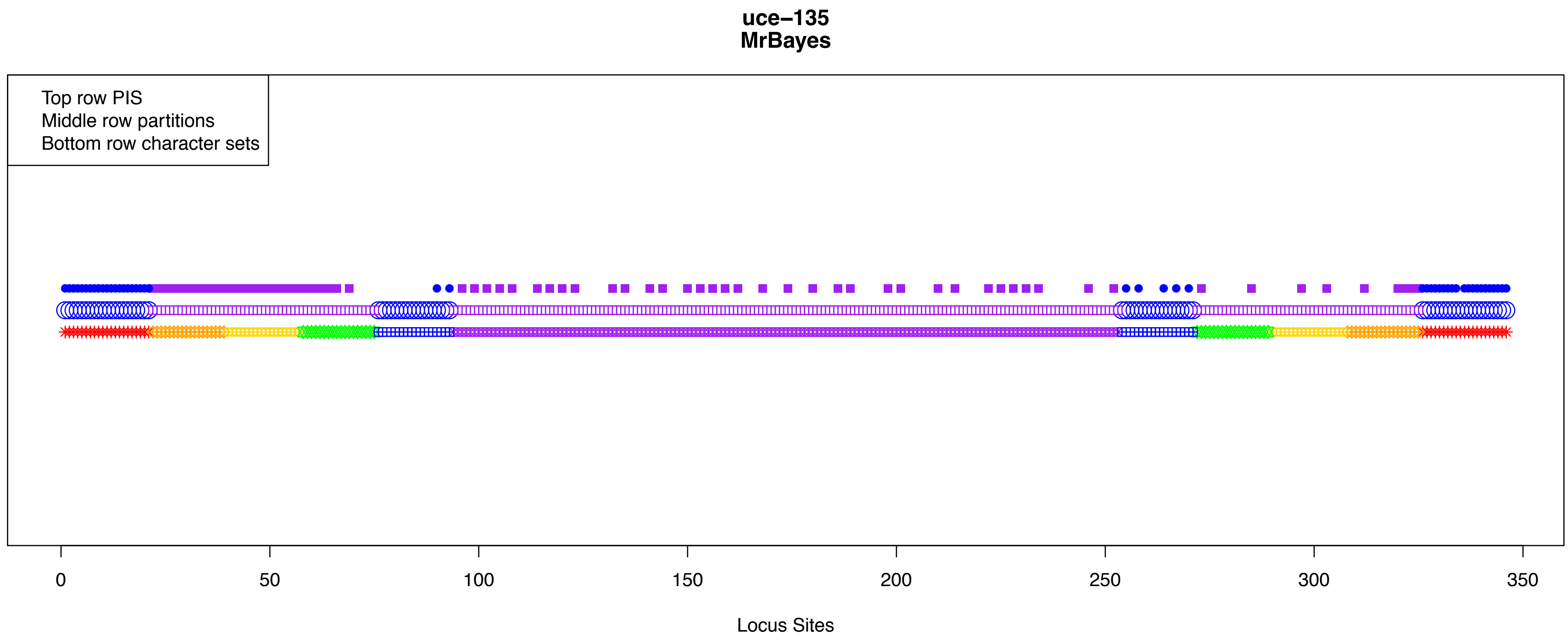
uce–135
MrBayes
Top row PIS
Middle row partitions
Bottom row character sets
0
50
100
150
200
250
300
350
Locus Sites

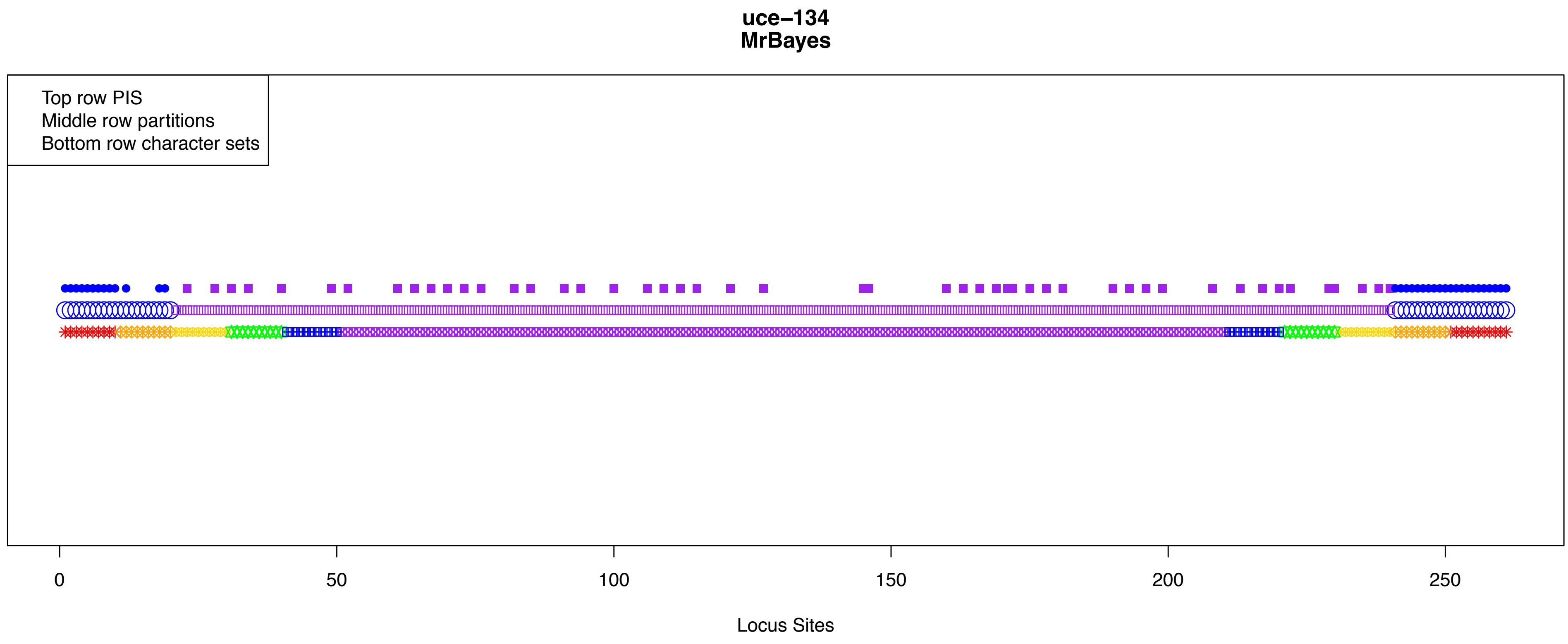
uce–134
MrBayes
Top row PIS
Middle row partitions
Bottom row character sets
0
50
100
150
200
250
Locus Sites

uce–1318
MrBayes

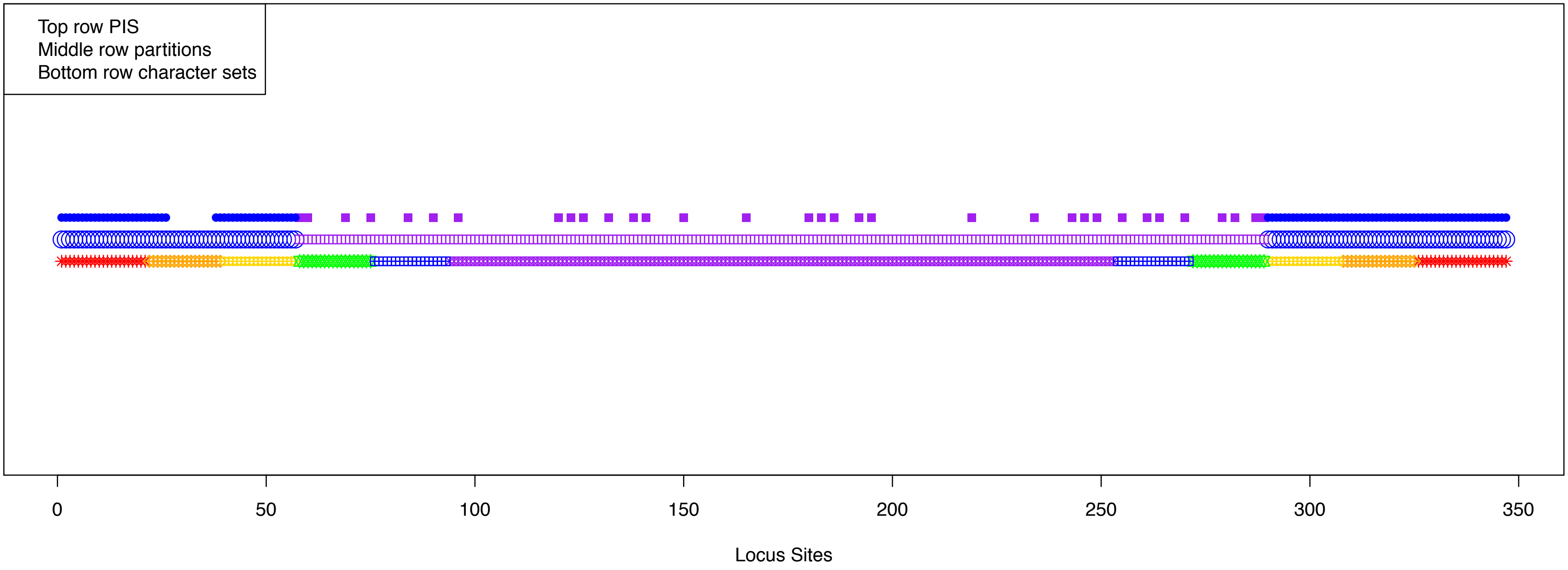

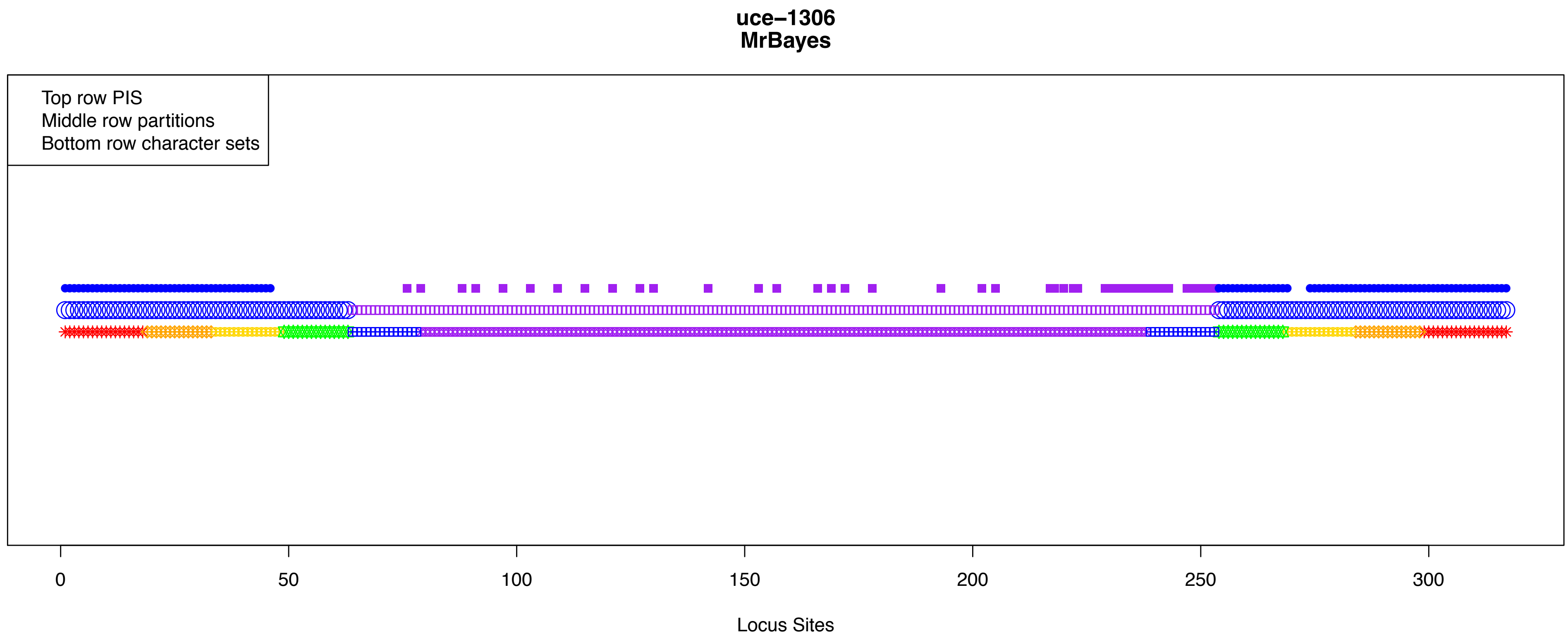
uce–1306
MrBayes
Top row PIS
Middle row partitions
Bottom row character sets
0
50
100
150
200
250
300
Locus Sites

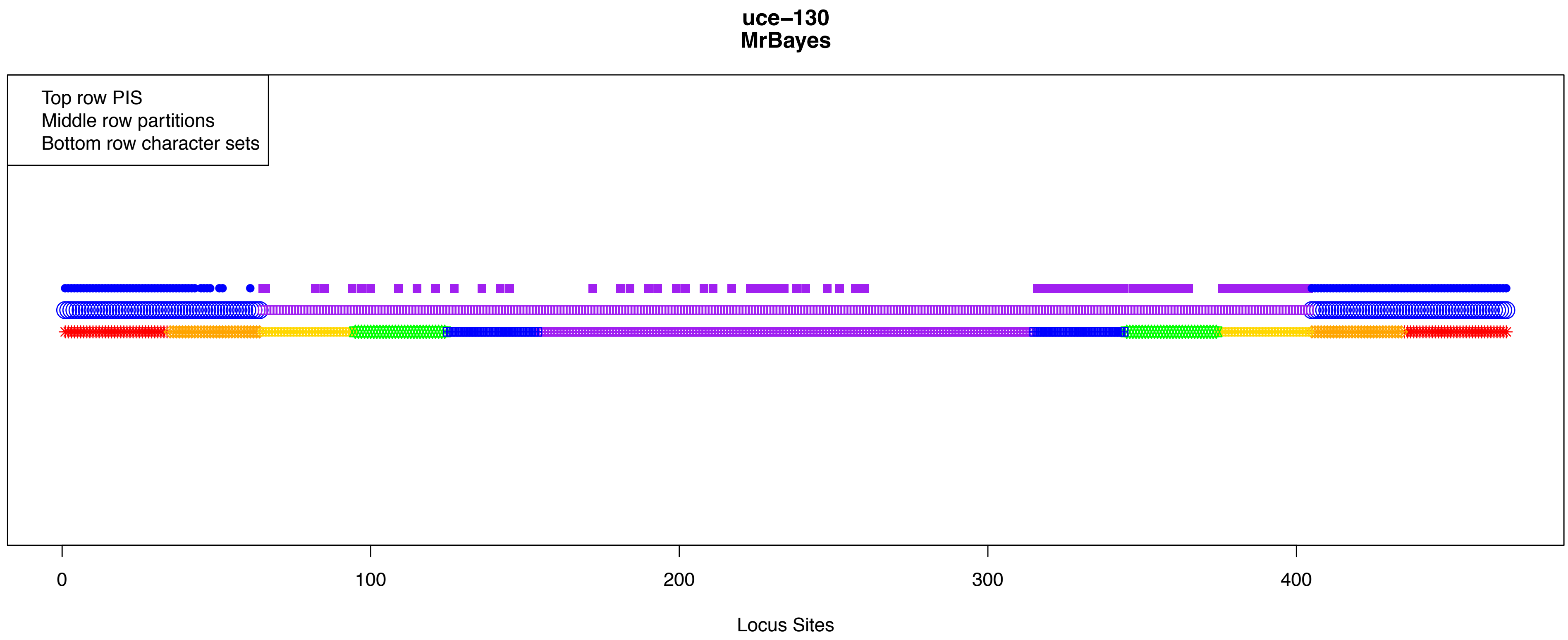

uce–130
MrBayes
Top row PIS
Middle row partitions
Bottom row character sets
0
100
200
300
400
Locus Sites

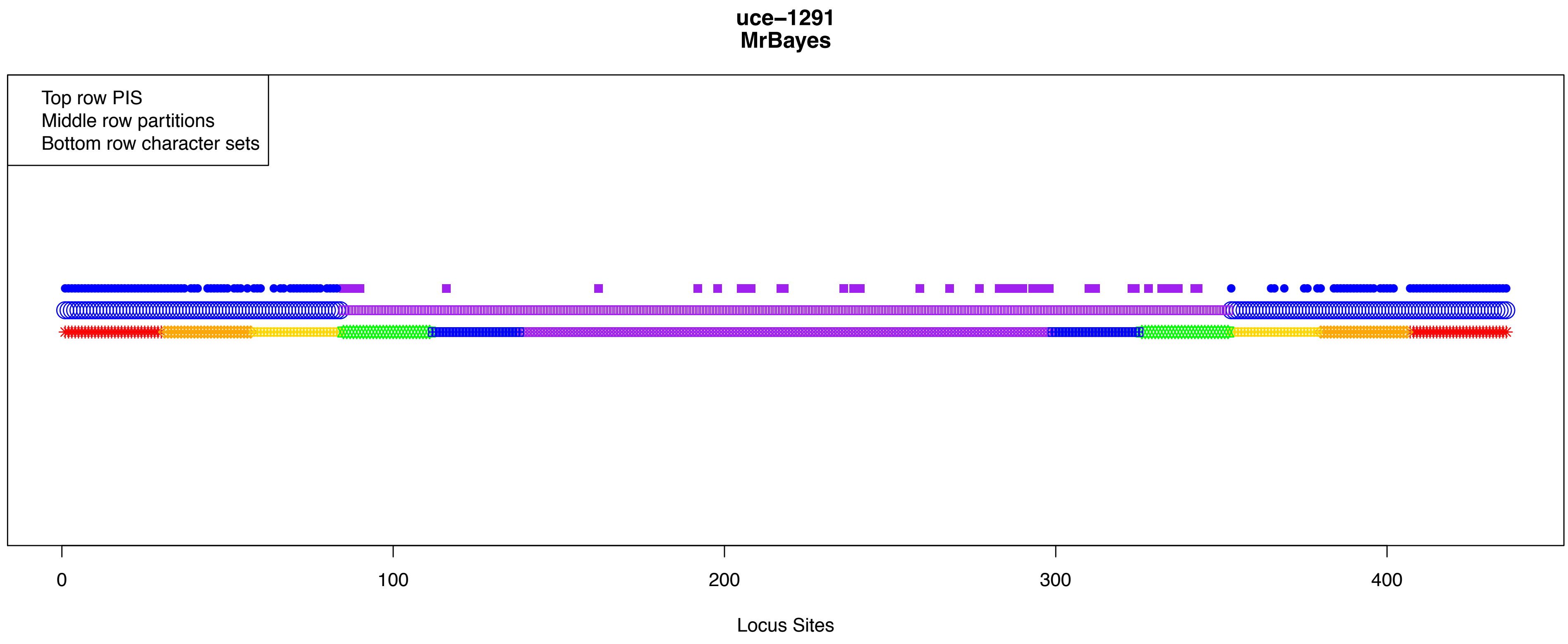
uce–1291
MrBayes
Top row PIS
Middle row partitions
Bottom row character sets
0
100
200
300
400
Locus Sites

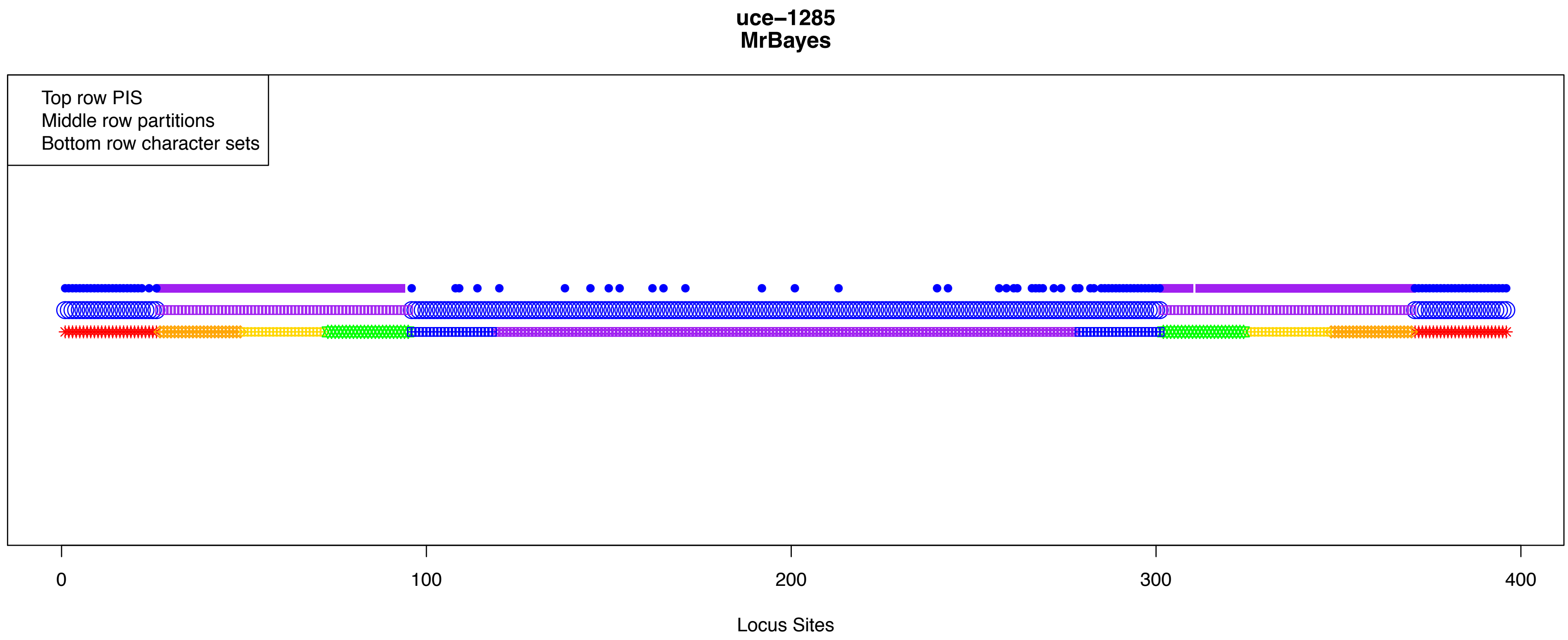
uce–1285
MrBayes
Top row PIS
Middle row partitions
Bottom row character sets
0
100
200
300
400
Locus Sites

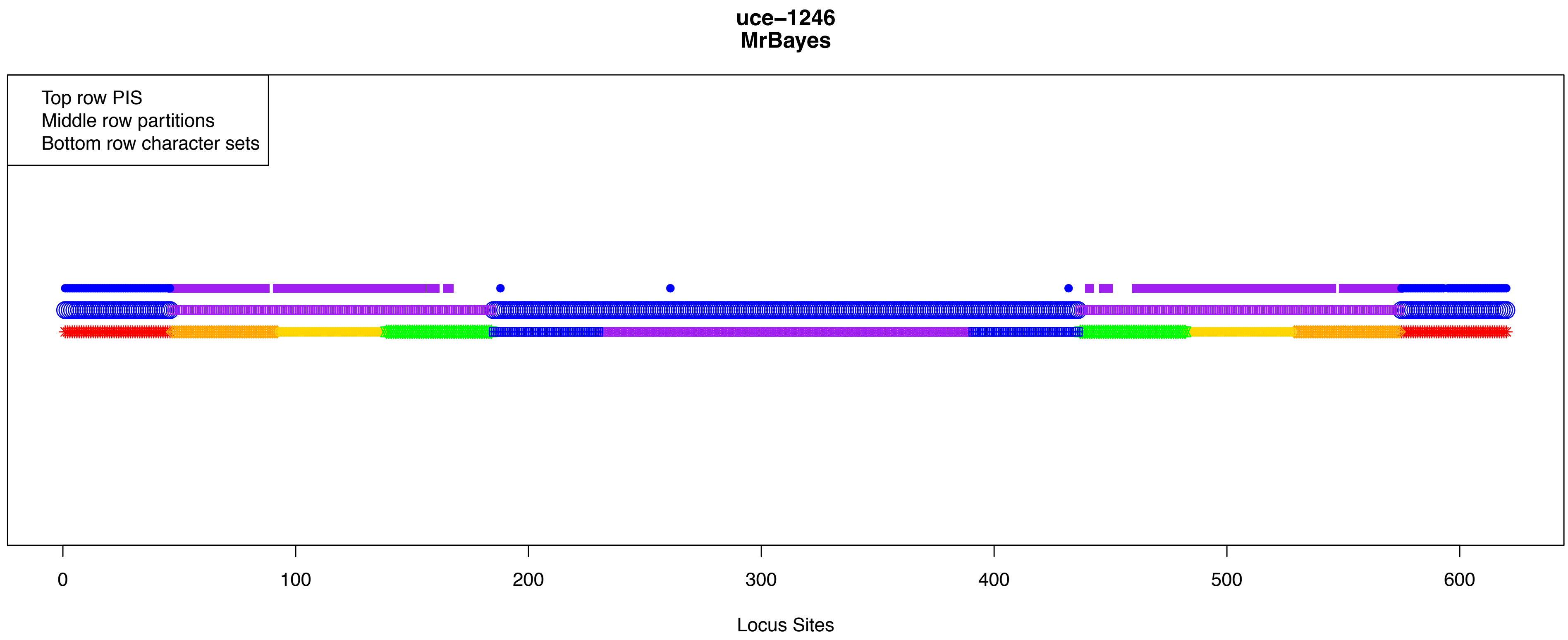
uce–1246
MrBayes
Top row PIS
Middle row partitions
Bottom row character sets
0
100
200
300
400
500
600
Locus Sites

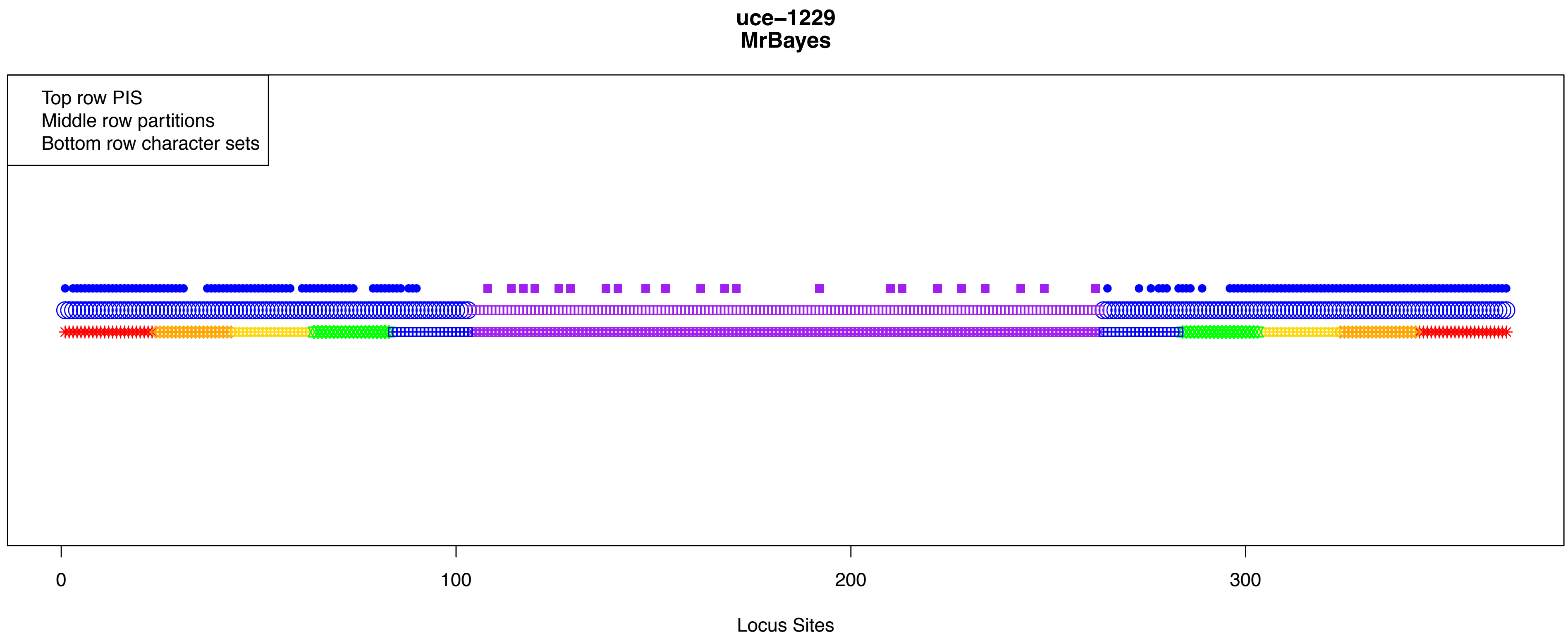

uce–1229
MrBayes
Top row PIS
Middle row partitions
Bottom row character sets
0
100
200
300
Locus Sites

uce–1204
MrBayes

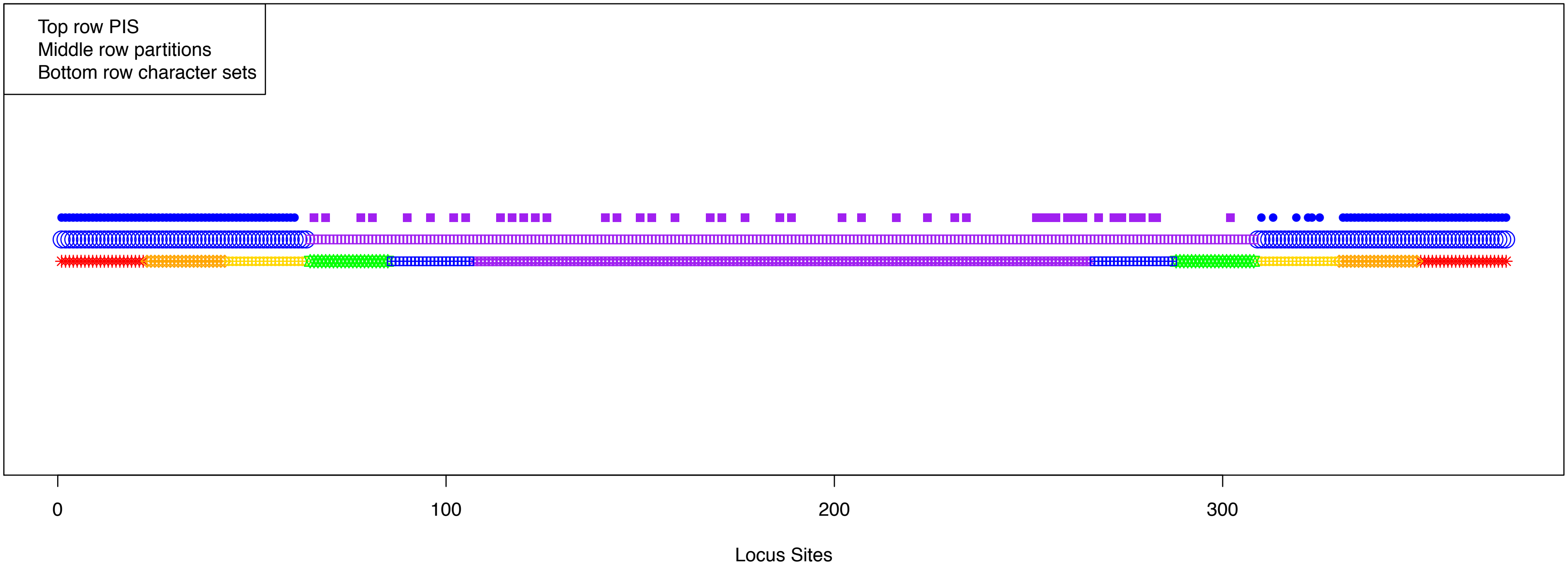

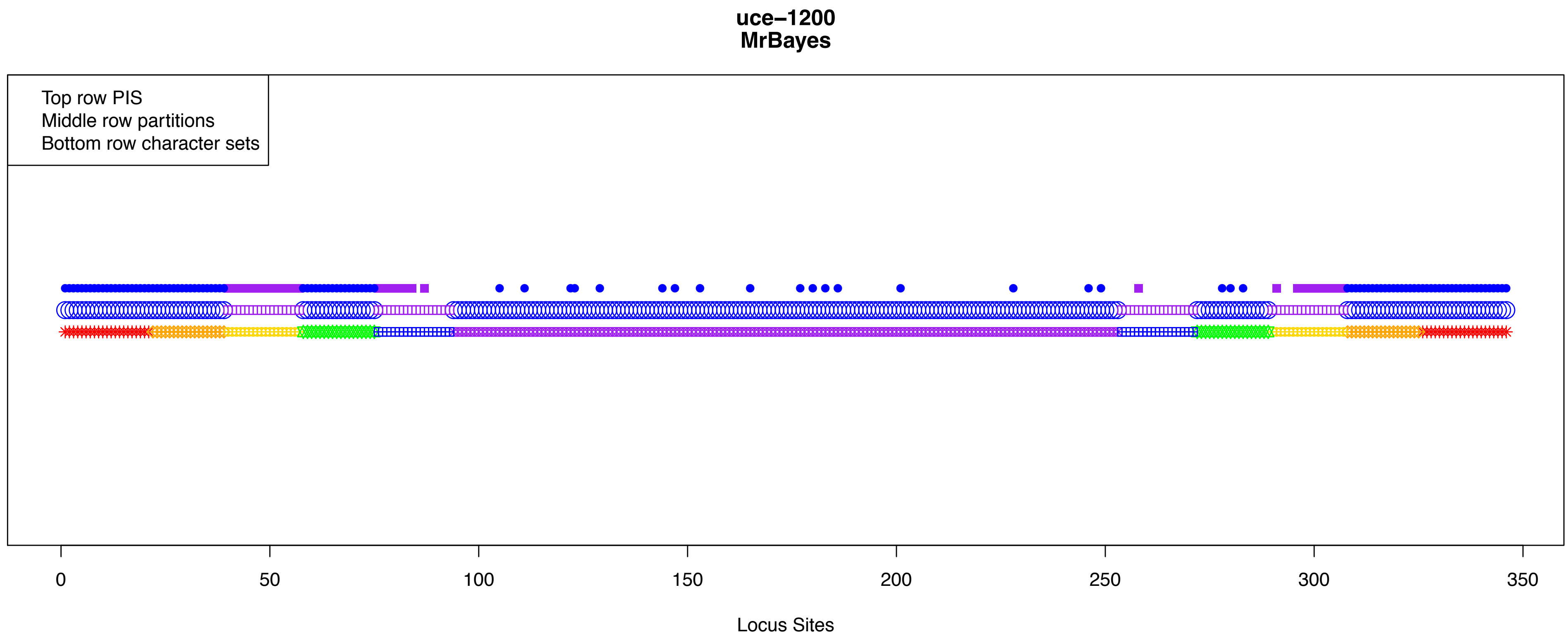
uce–1200
MrBayes
Top row PIS
Middle row partitions
Bottom row character sets
0
50
100
150
200
250
300
350
Locus Sites

uce–1173
MrBayes

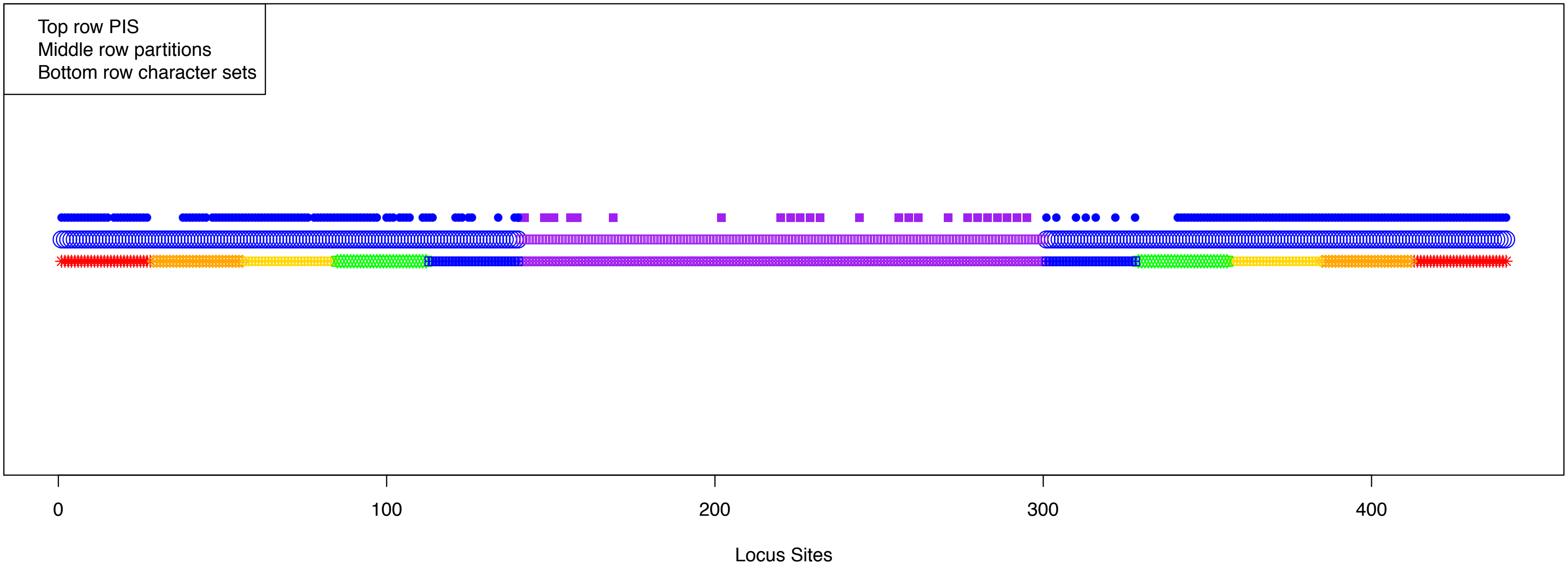

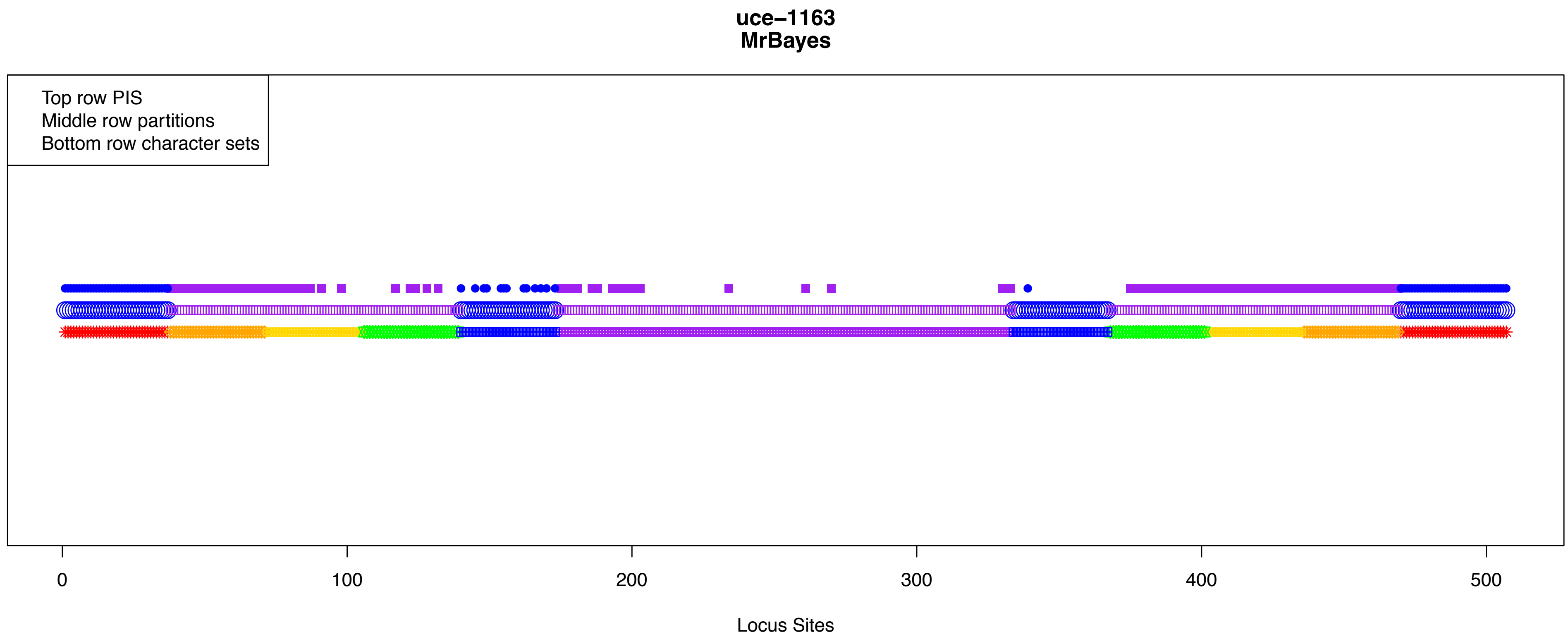
uce–1163
MrBayes
Top row PIS
Middle row partitions
Bottom row character sets
0
100
200
300
400
500
Locus Sites

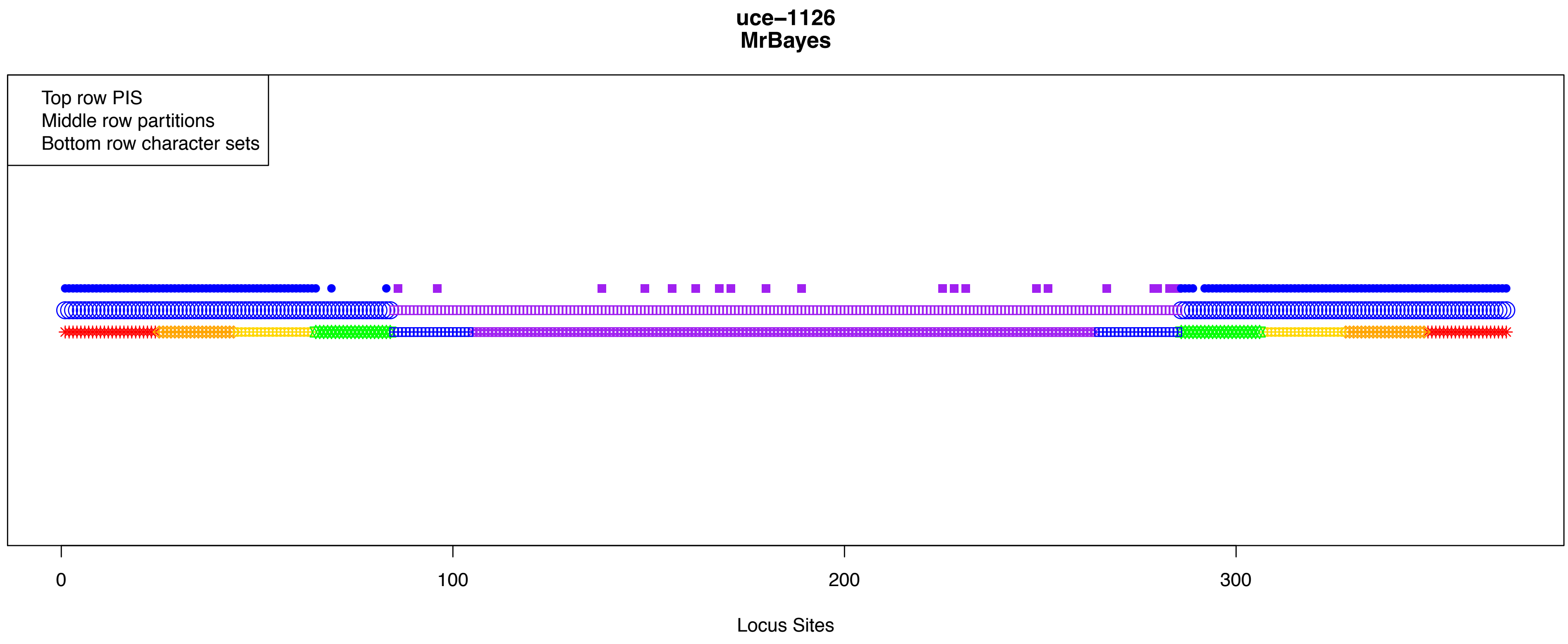

uce–1126
MrBayes
Top row PIS
Middle row partitions
Bottom row character sets
0
100
200
300
Locus Sites

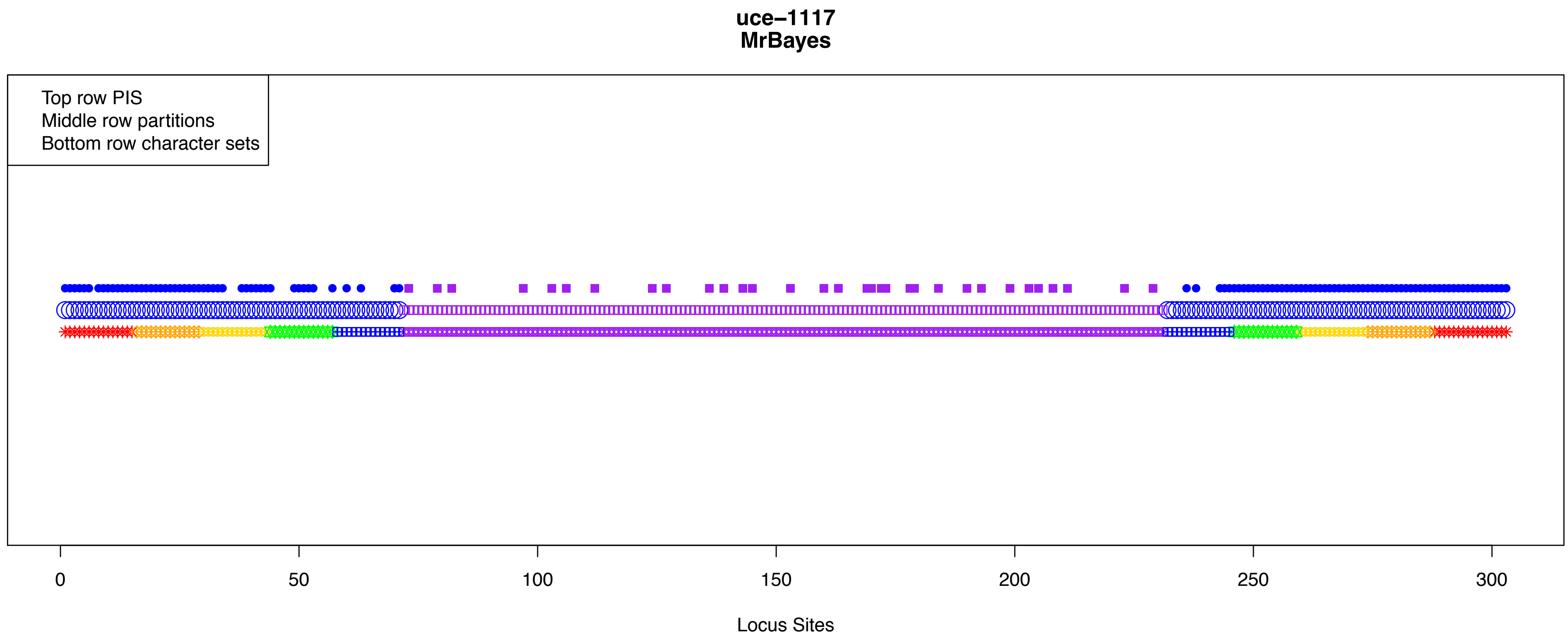
uce–1117
MrBayes
Top row PIS
Middle row partitions
Bottom row character sets
0
50
100
150
200
250
300
Locus Sites

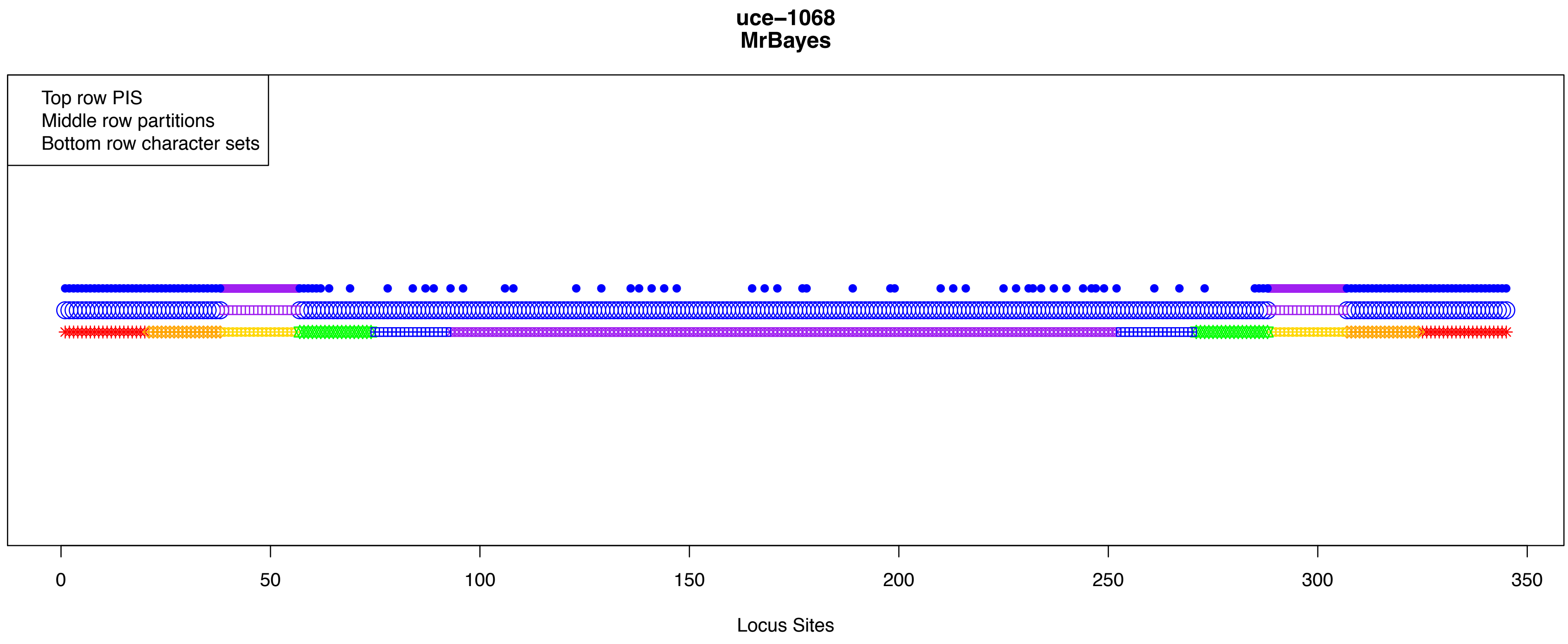

uce–1068
MrBayes
Top row PIS
Middle row partitions
Bottom row character sets
0
50
100
150
200
250
300
350
Locus Sites

uce–1065
MrBayes

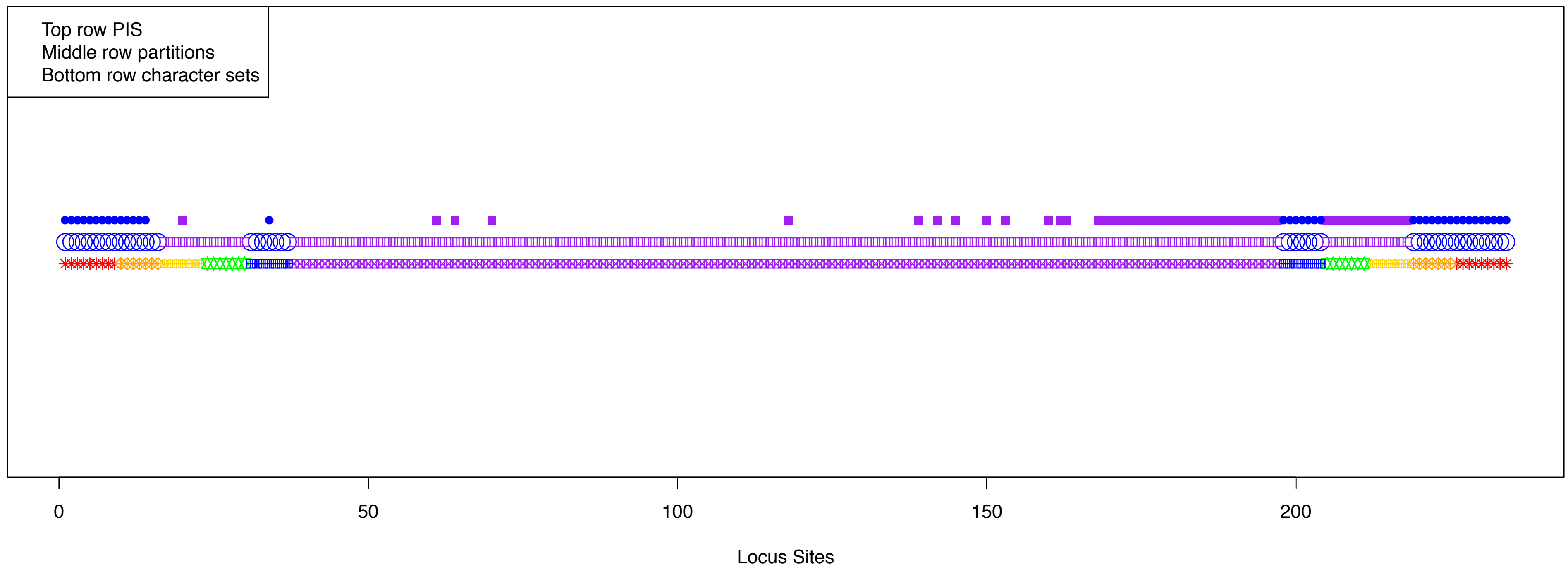

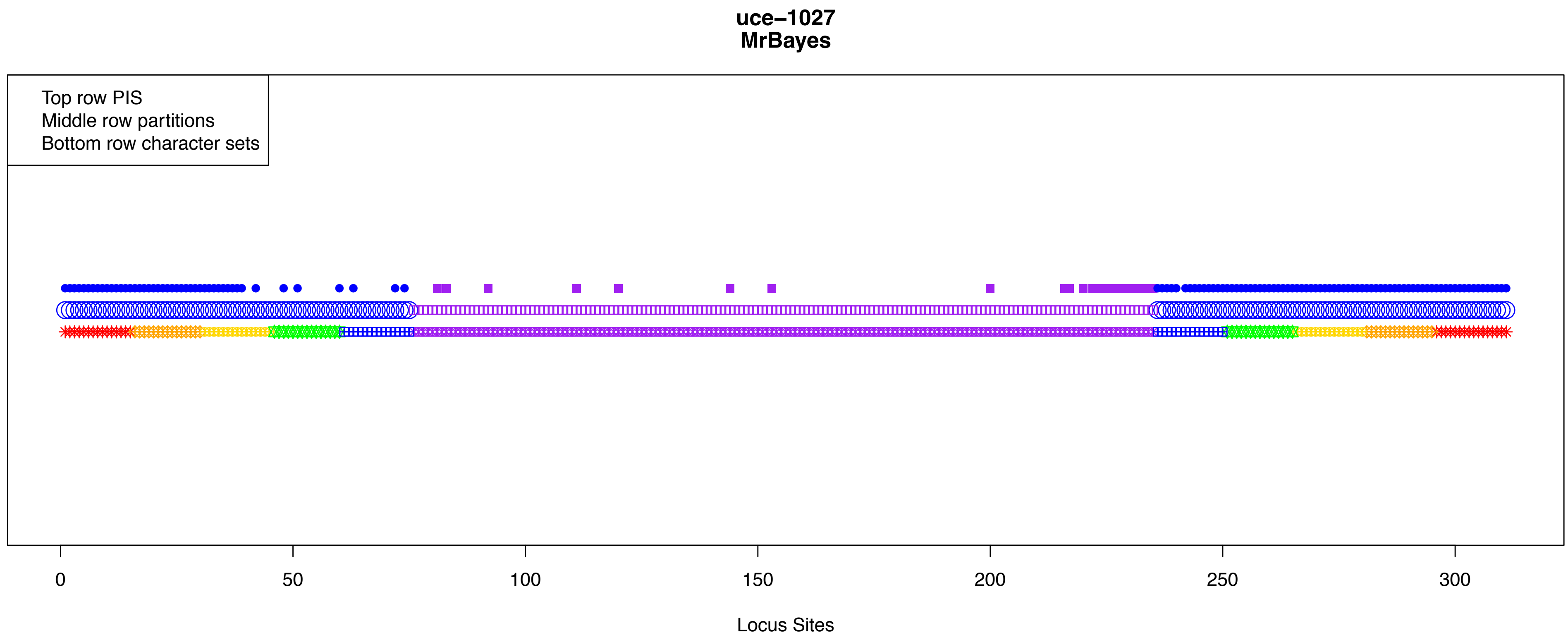
uce–1027
MrBayes
Top row PIS
Middle row partitions
Bottom row character sets
0
50
100
150
200
250
300
Locus Sites

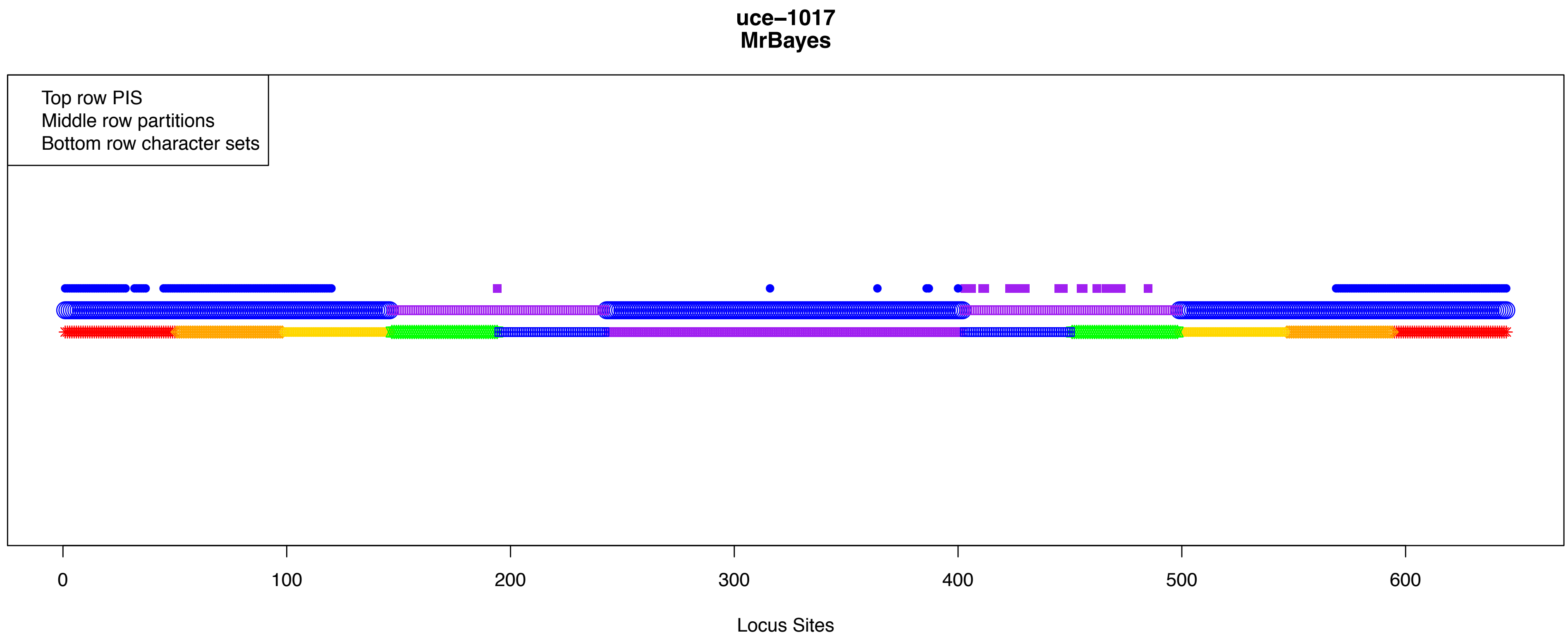

uce–1017
MrBayes
Top row PIS
Middle row partitions
Bottom row character sets
0
100
200
300
400
500
600
Locus Sites

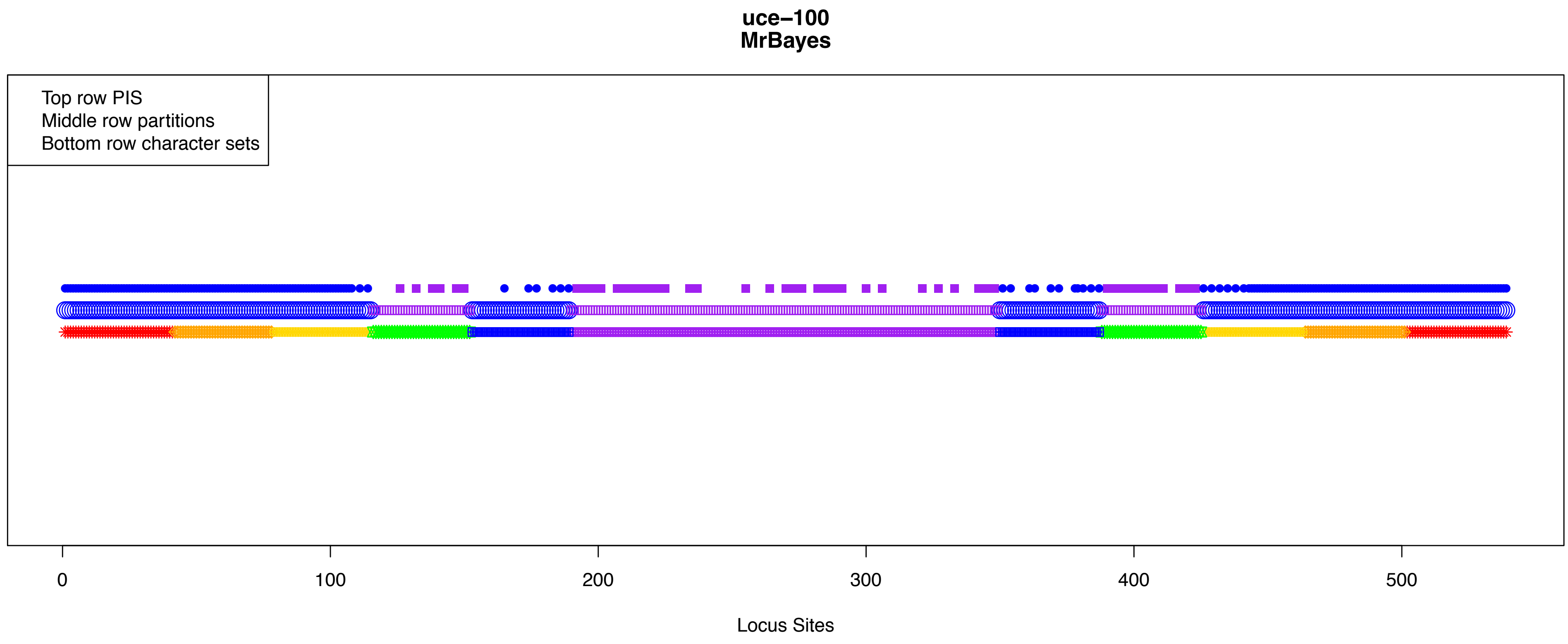

uce–100
MrBayes
Top row PIS
Middle row partitions
Bottom row character sets
0
100
200
300
400
500
Locus Sites
