# Supplementary material for: Ultraconserved elements (UCEs) resolve the phylogeny of Australasian smurf-weevils
Source: PLoS One. 2017 Nov 22;12(11):e0188044. doi: 10.1371/journal.pone.0188044 (PMC5699822; doi:10.1371/journal.pone.0188044)
Supplement: S1 File — (ZIP) [file pone.0188044.s007.zip › Supplemental_Partition_Number_of_partitions_PIS_Charsets/partitions2-RAxML.pdf]

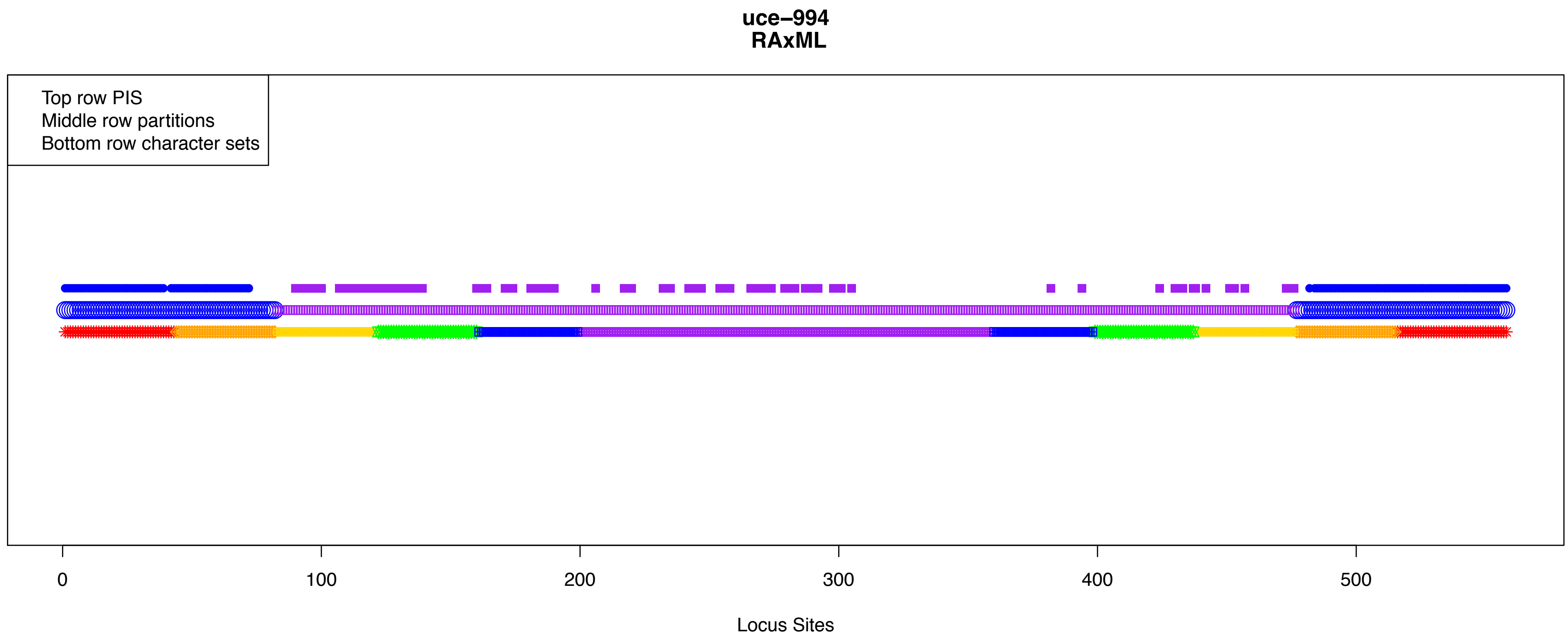
uce–994
RAxML
Top row PIS
Middle row partitions
Bottom row character sets
0
100
200
300
400
500
Locus Sites

uce–993
RAxML

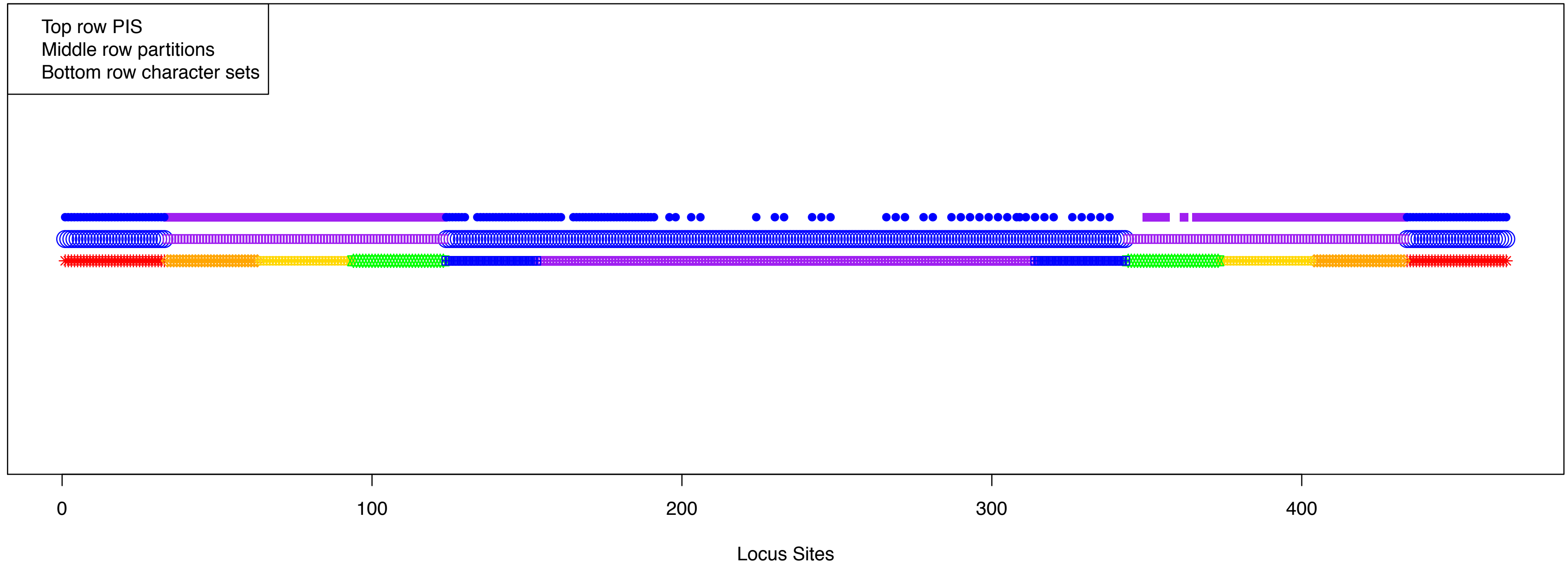

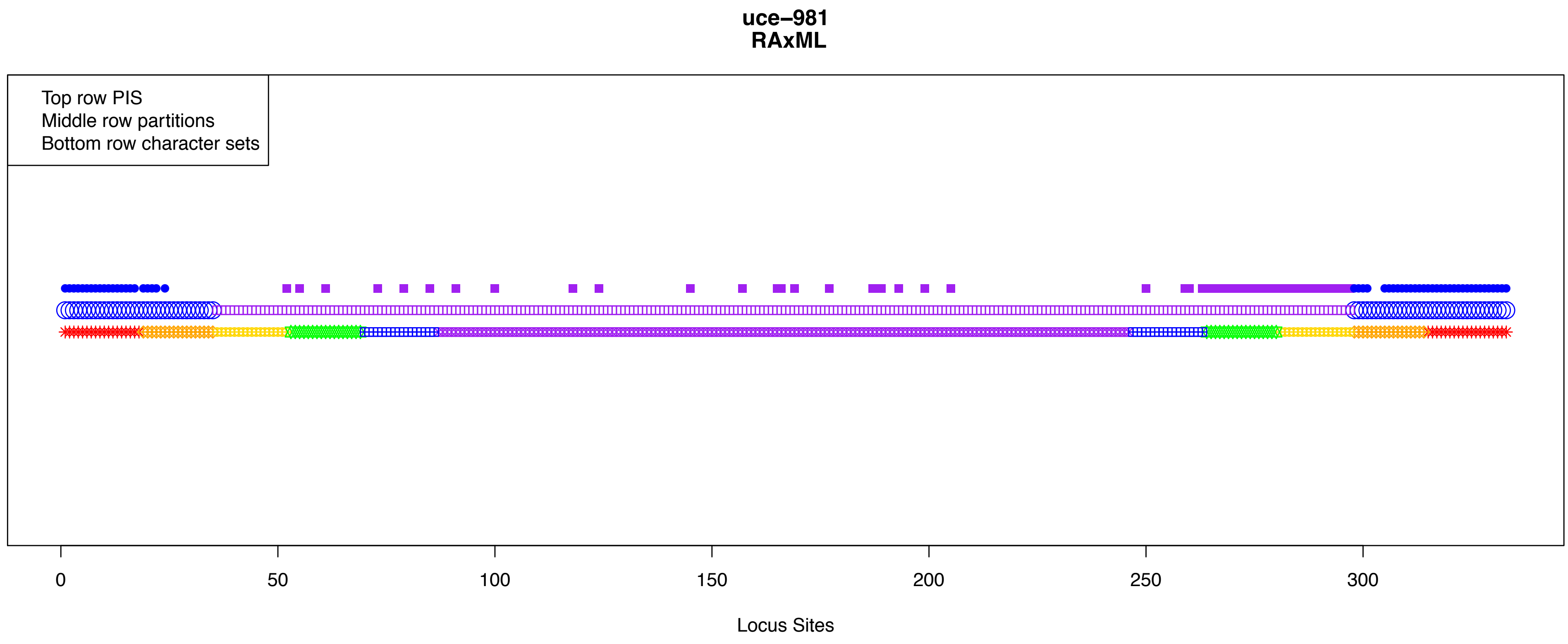

uce–981
RAxML
Top row PIS
Middle row partitions
Bottom row character sets
0
50
100
150
200
250
300
Locus Sites

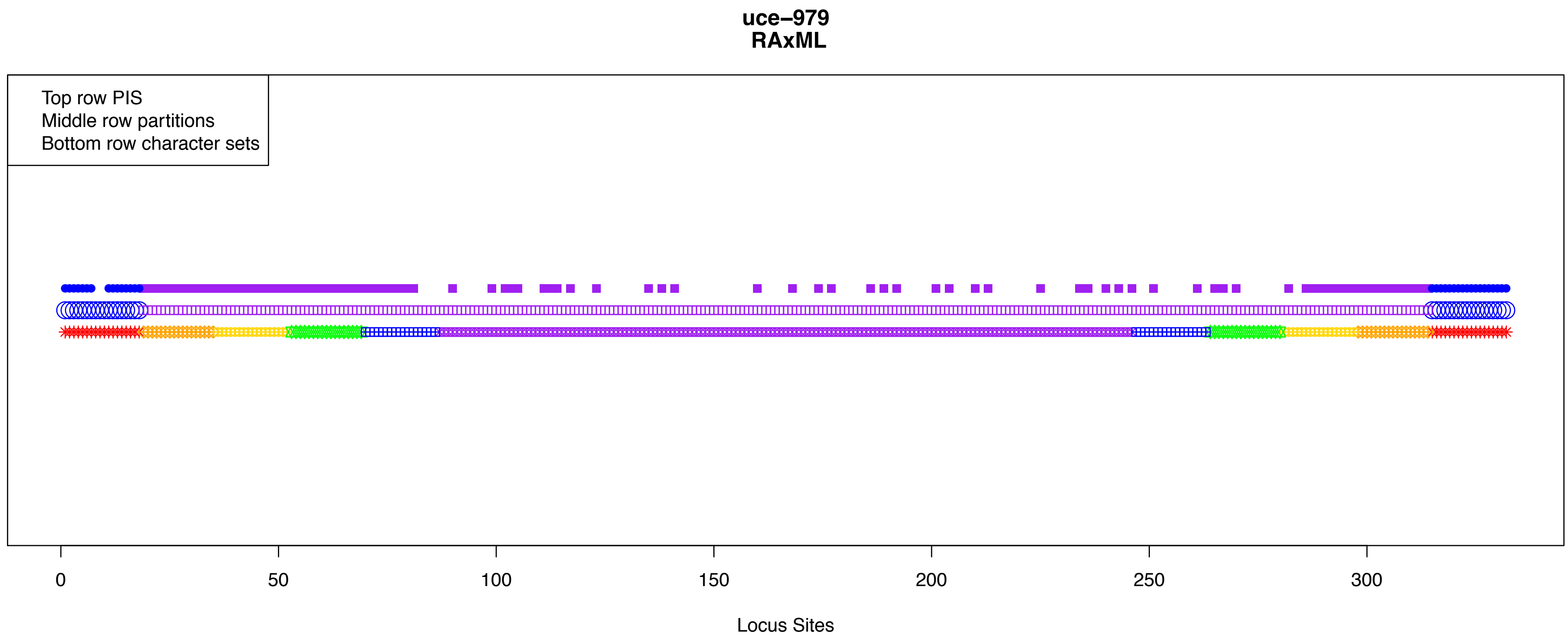

uce–979
RAxML
Top row PIS
Middle row partitions
Bottom row character sets
0
50
100
150
200
250
300
Locus Sites

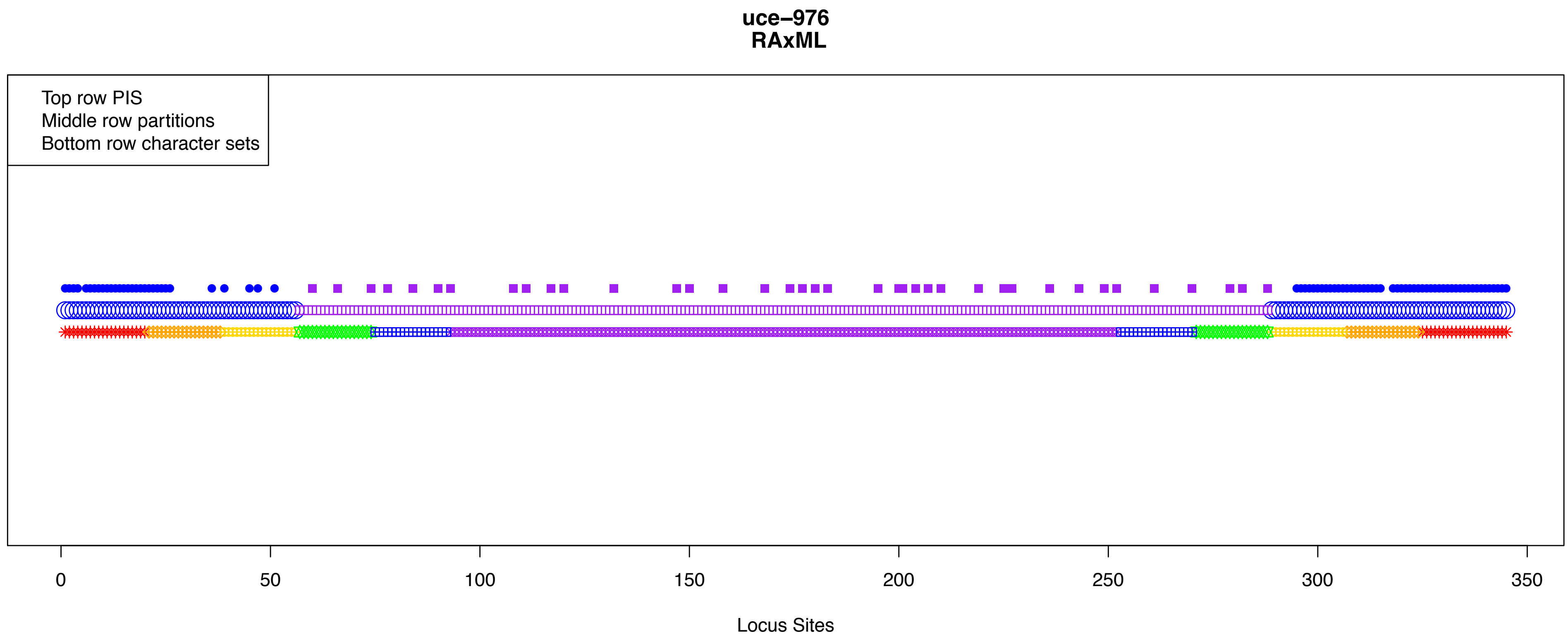
uce–976
RAxML
Top row PIS
Middle row partitions
Bottom row character sets
0
50
100
150
200
250
300
350
Locus Sites

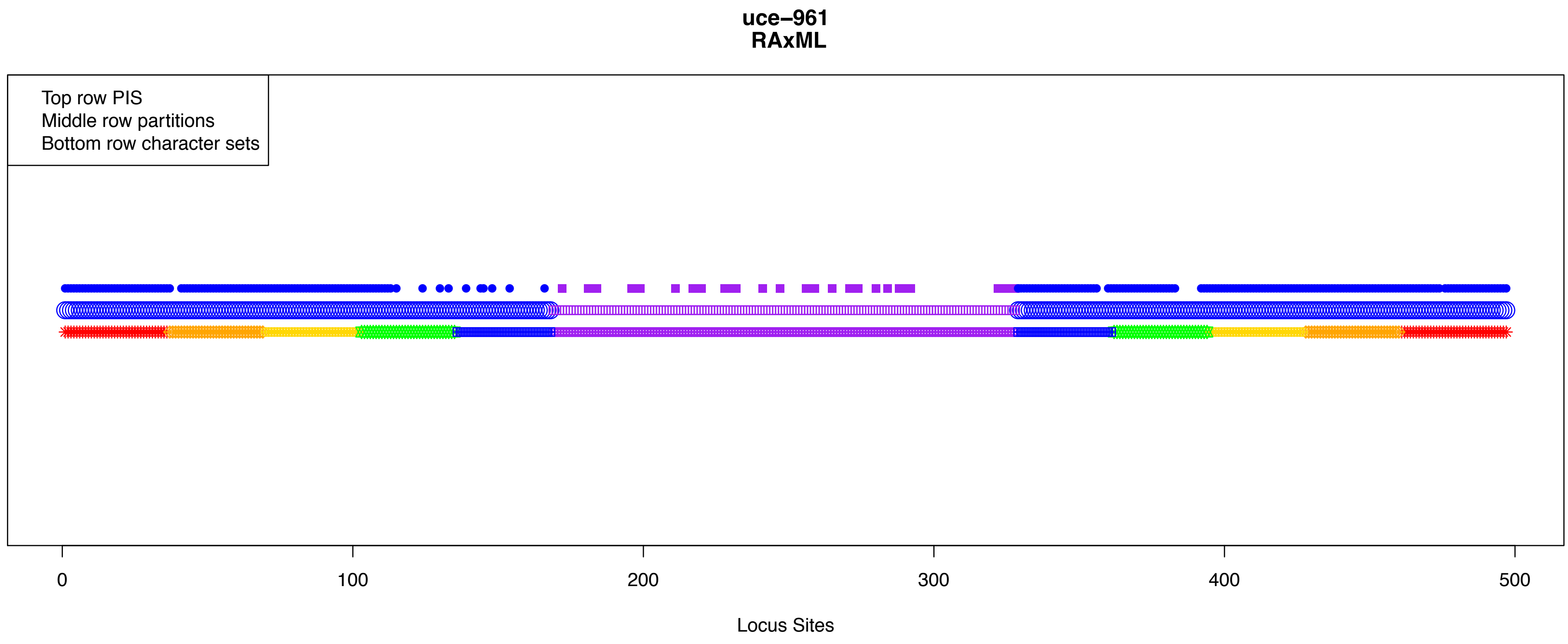
uce–961
RAxML
Top row PIS
Middle row partitions
Bottom row character sets
0
100
200
300
400
500
Locus Sites

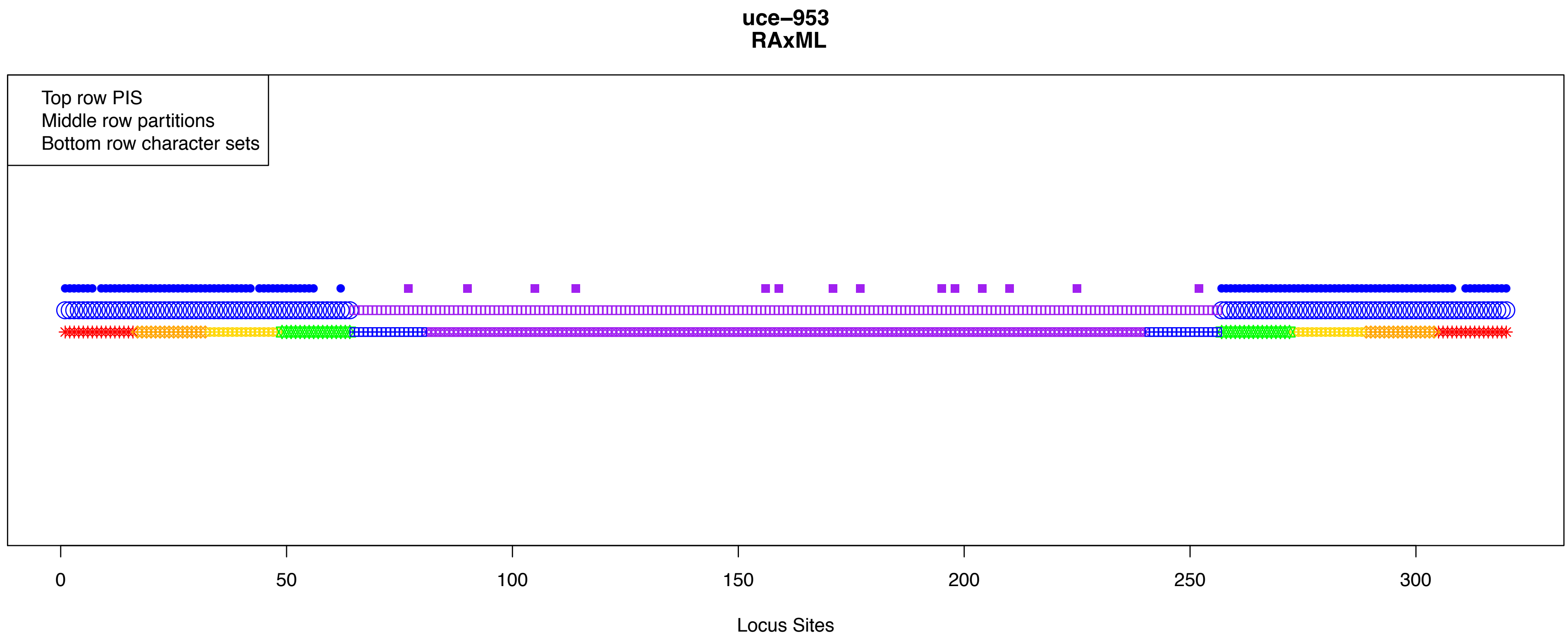
uce–953
RAxML
Top row PIS
Middle row partitions
Bottom row character sets
0
50
100
150
200
250
300
Locus Sites

uce–935
RAxML

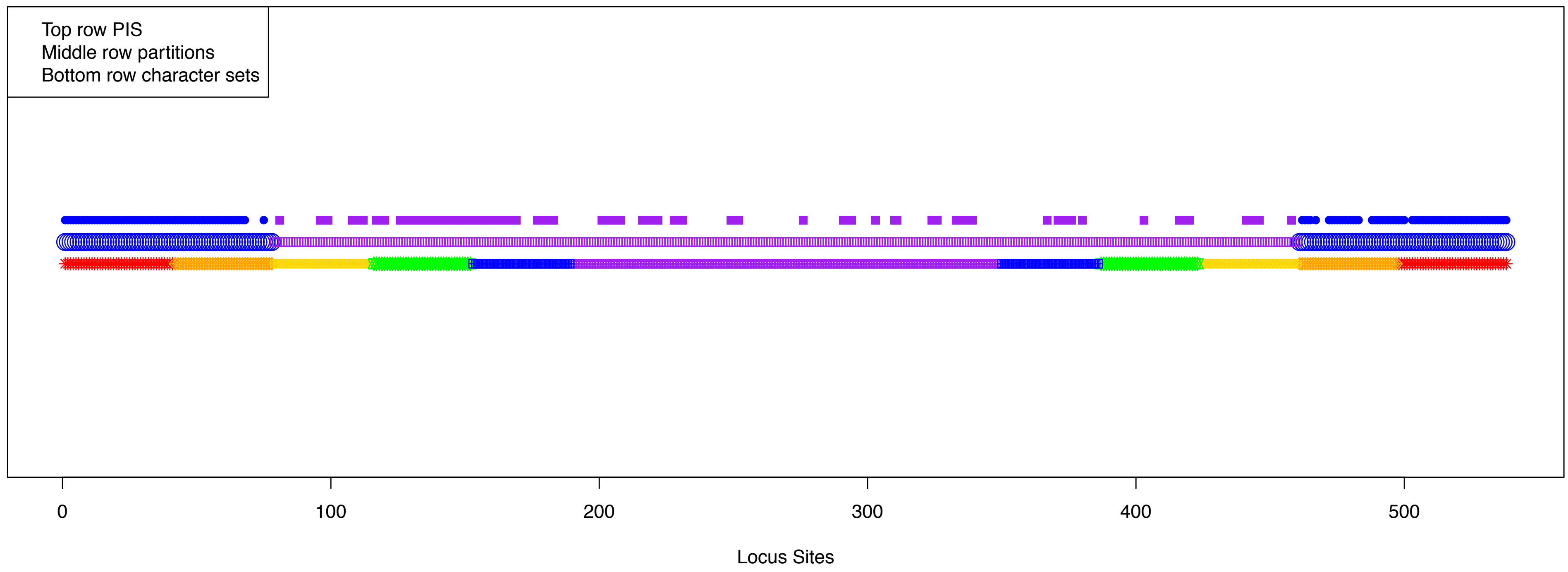

uce–914
RAxML

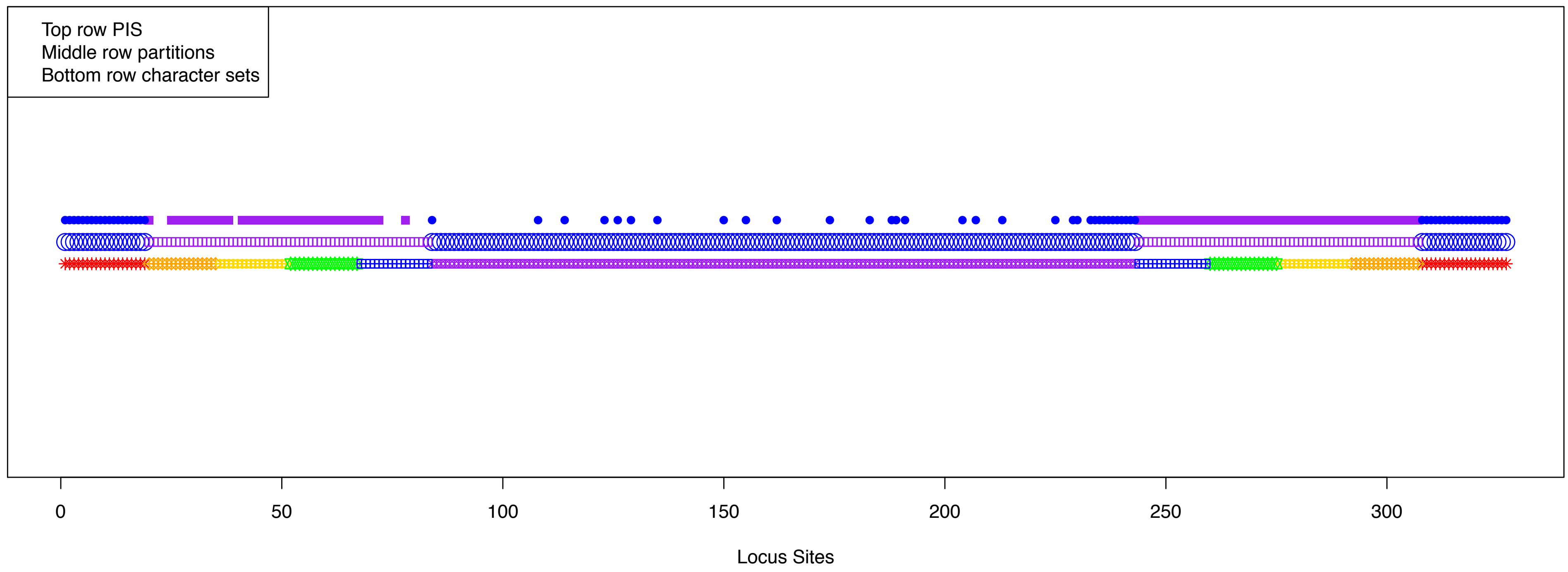

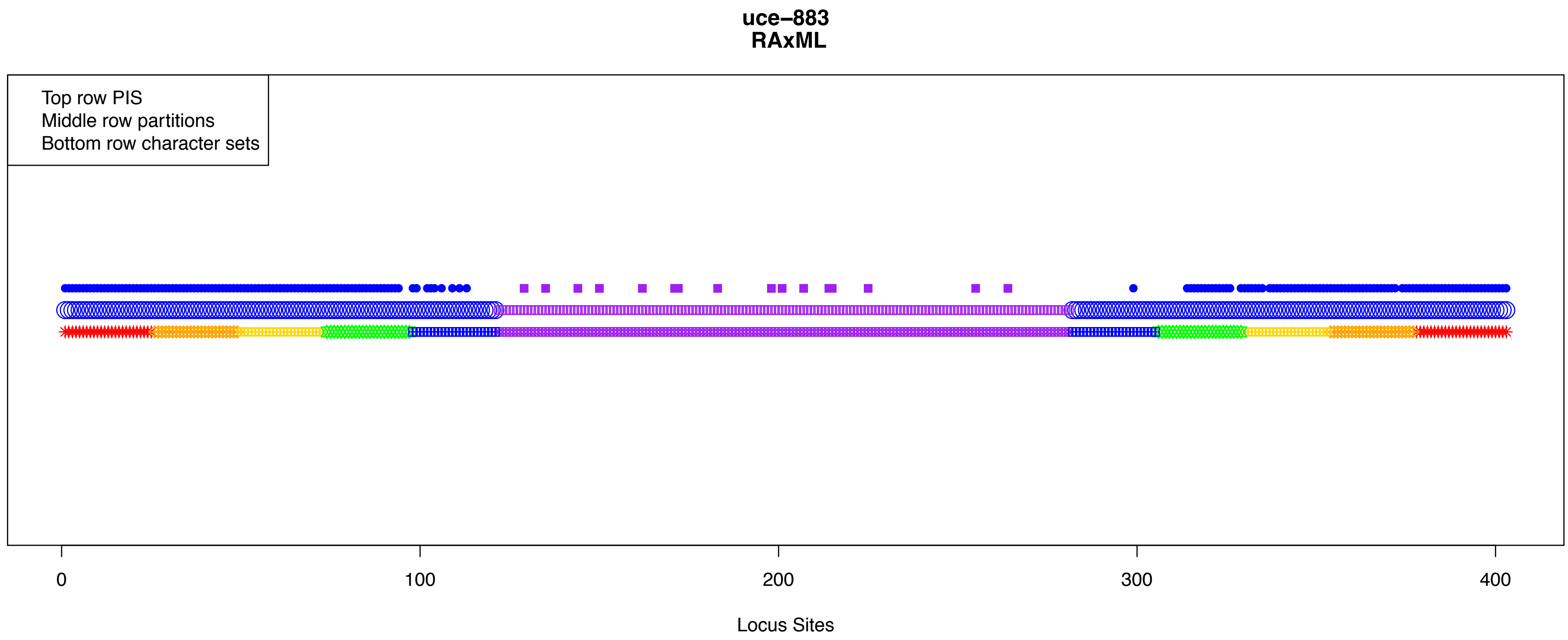
uce–883
RAxML
Top row PIS
Middle row partitions
Bottom row character sets
0
100
200
300
400
Locus Sites

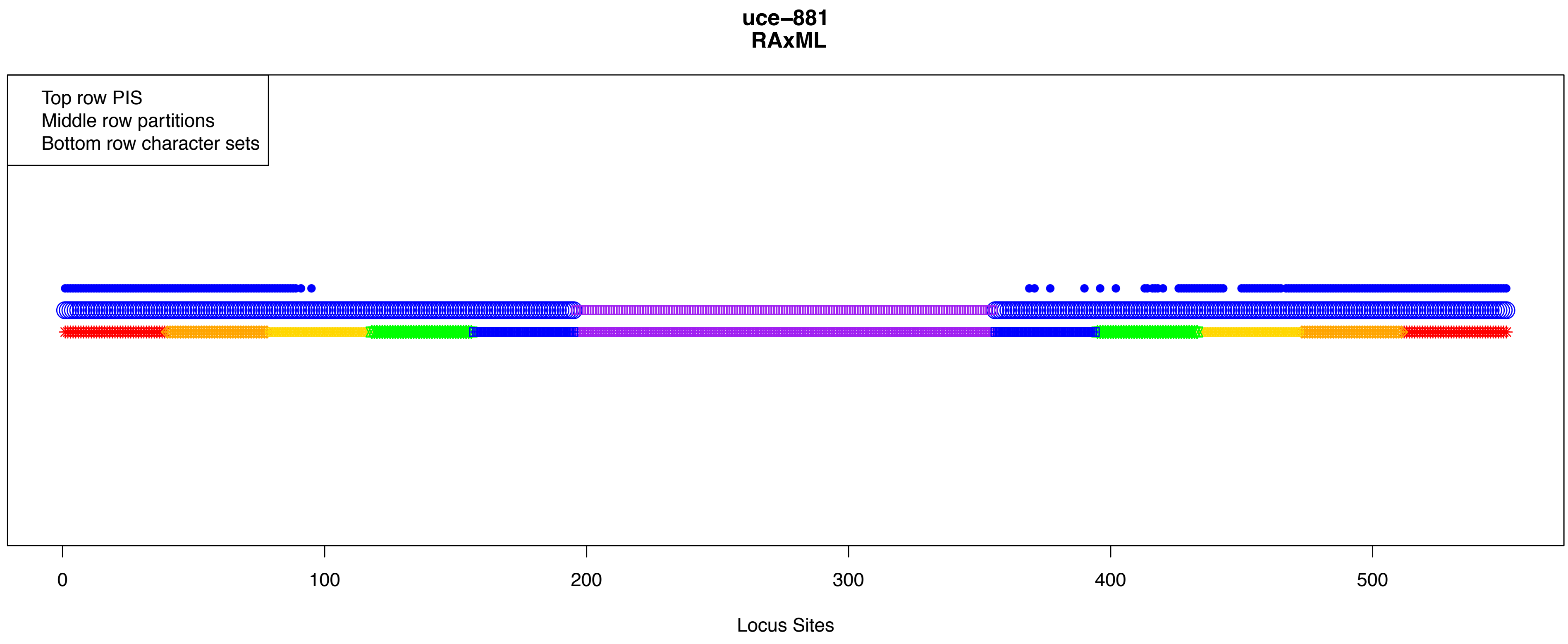
uce–881
RAxML
Top row PIS
Middle row partitions
Bottom row character sets
0
100
200
300
400
500
Locus Sites

uce–877
RAxML

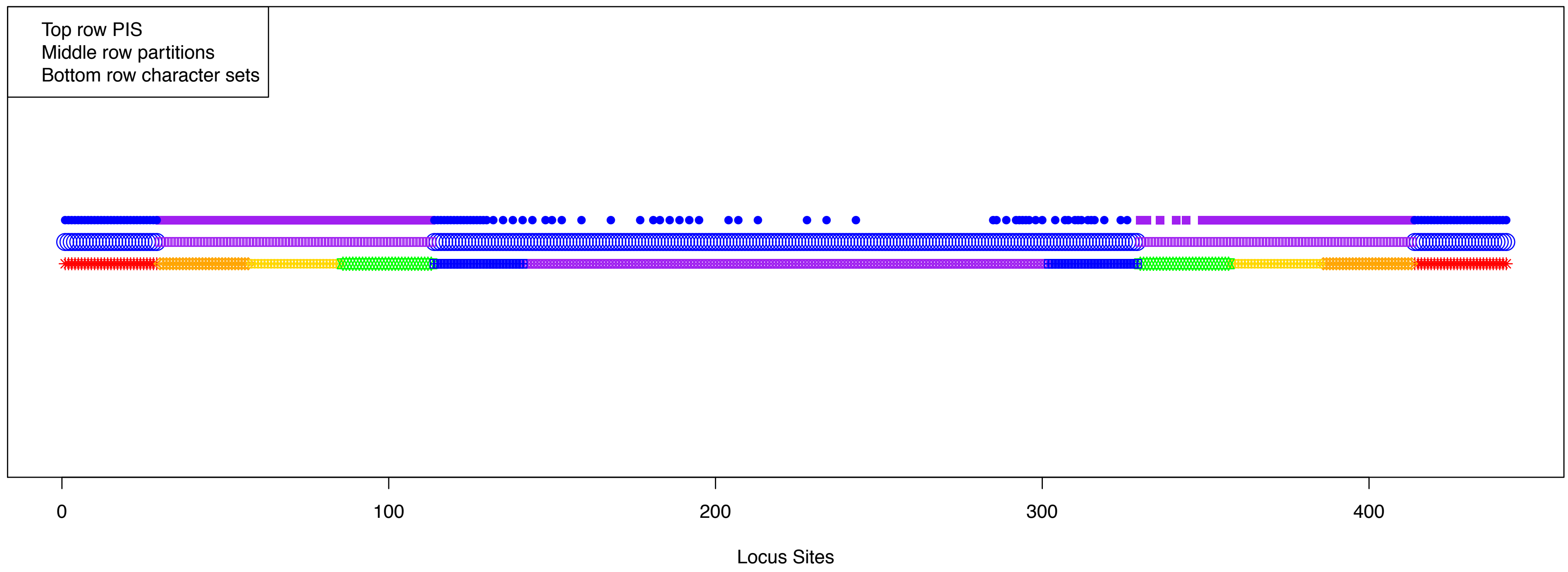

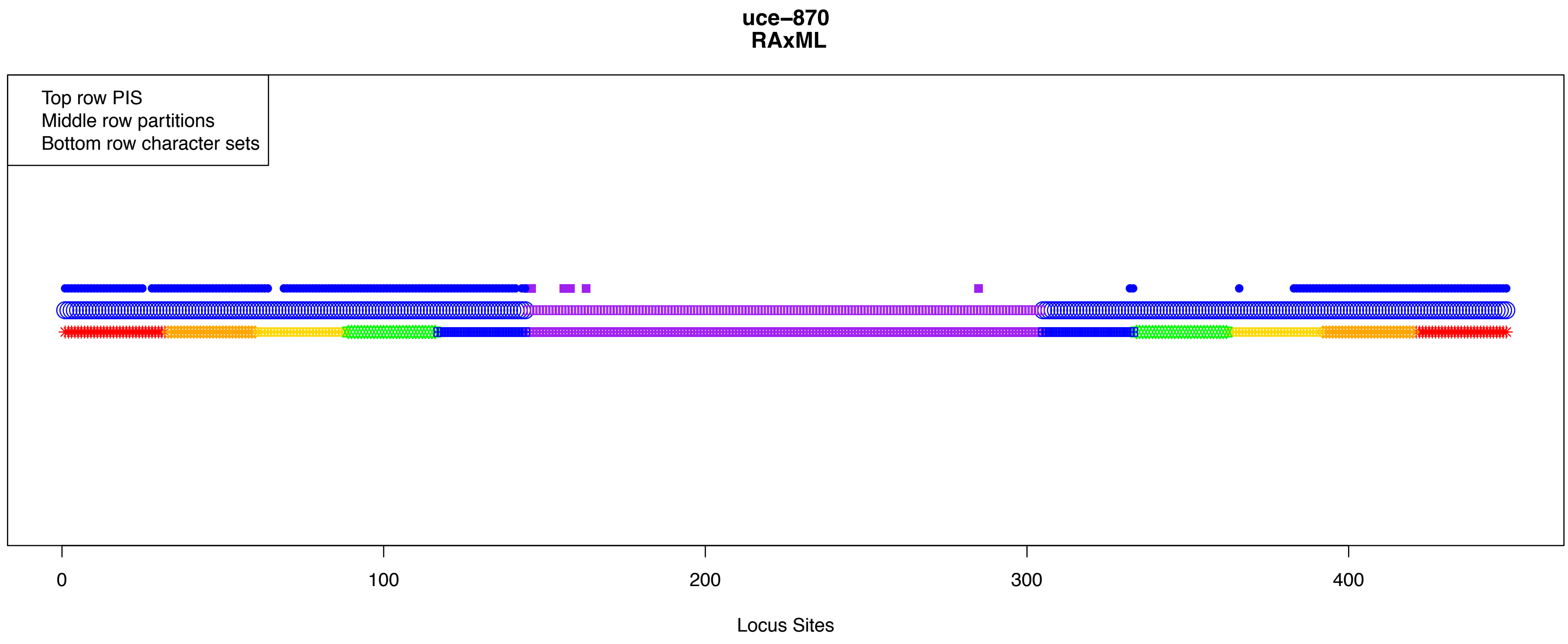

uce–870
RAxML
Top row PIS
Middle row partitions
Bottom row character sets
0
100
200
300
400
Locus Sites

uce–866
RAxML

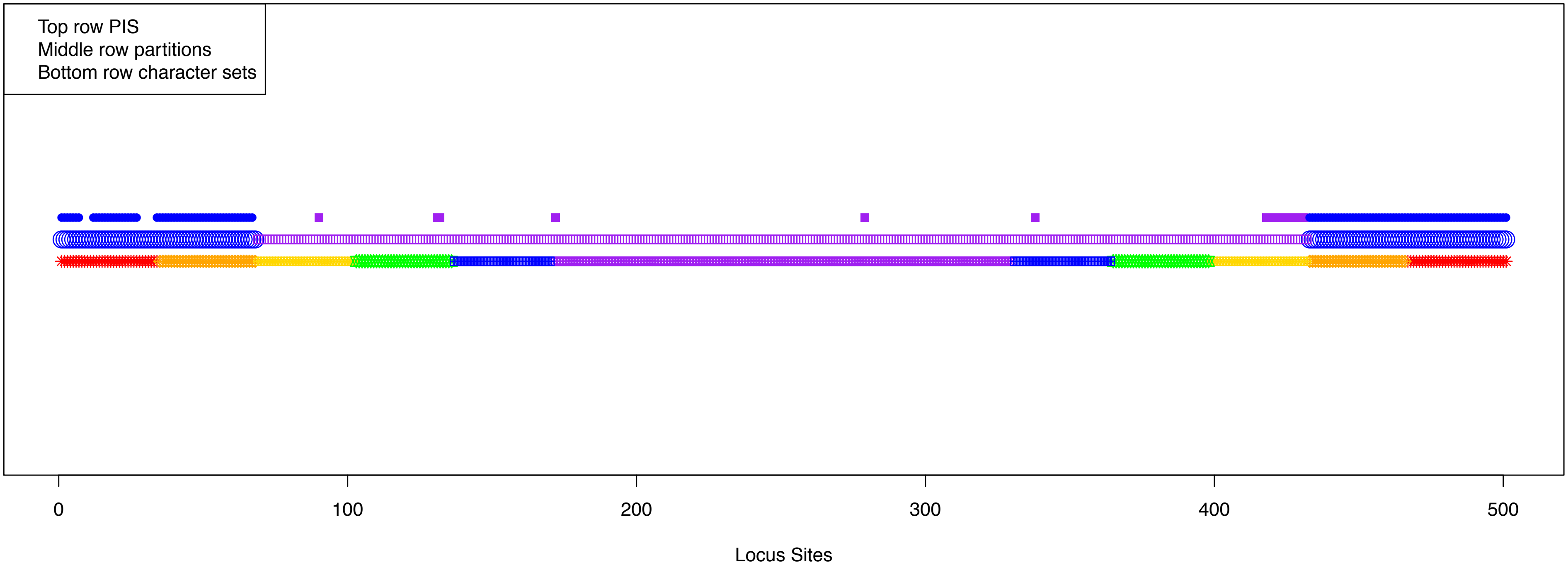

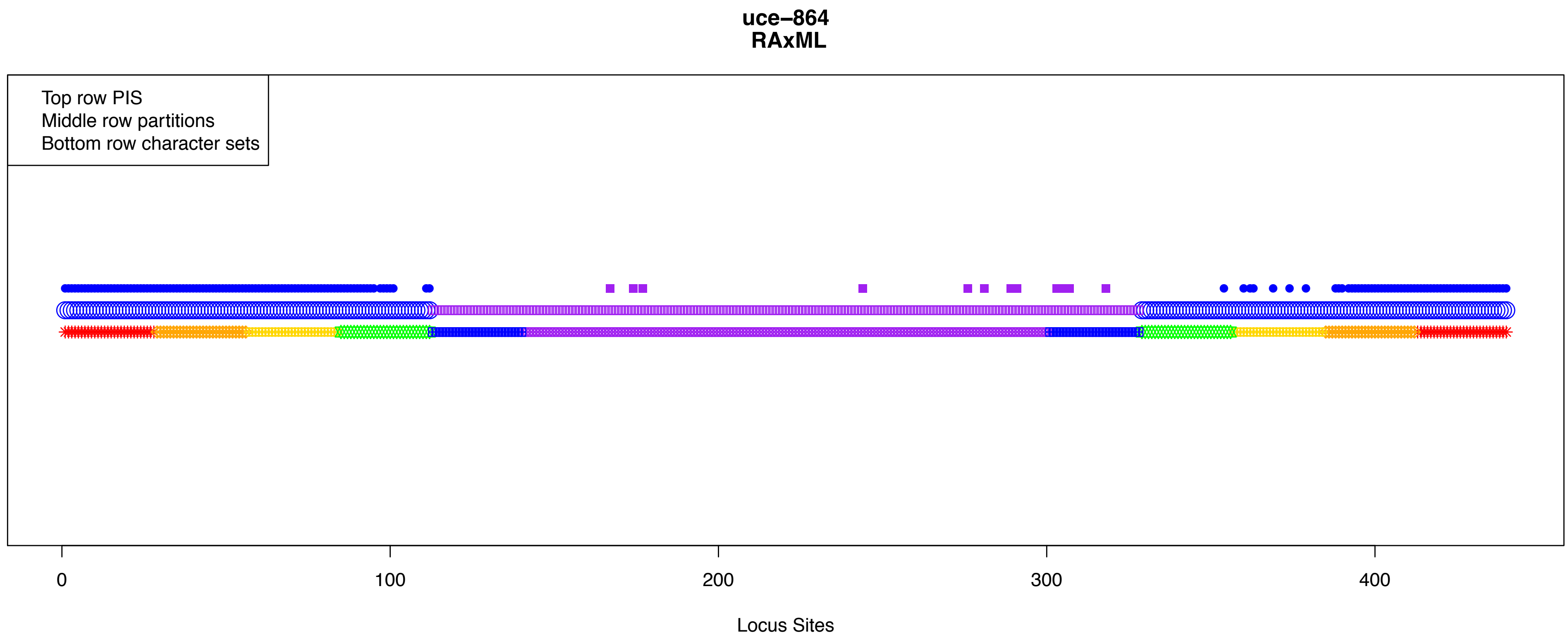
uce–864
RAxML
Top row PIS
Middle row partitions
Bottom row character sets
0
100
200
300
400
Locus Sites

uce–863
RAxML

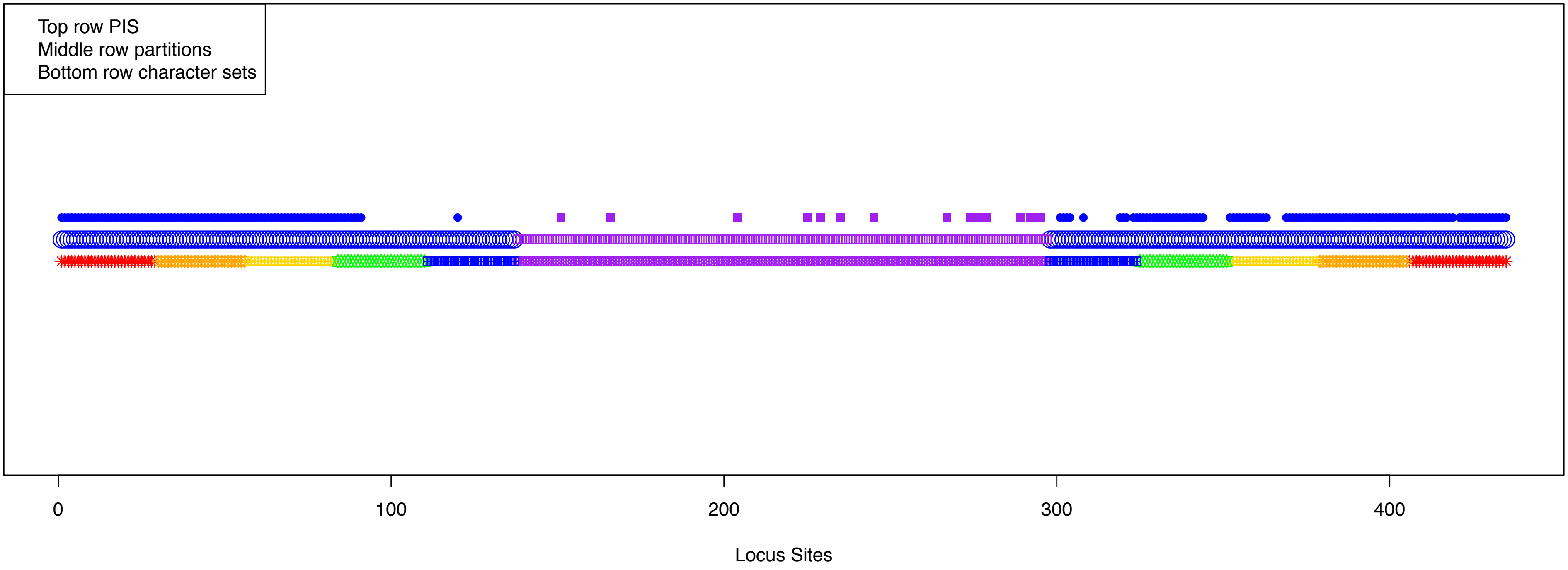

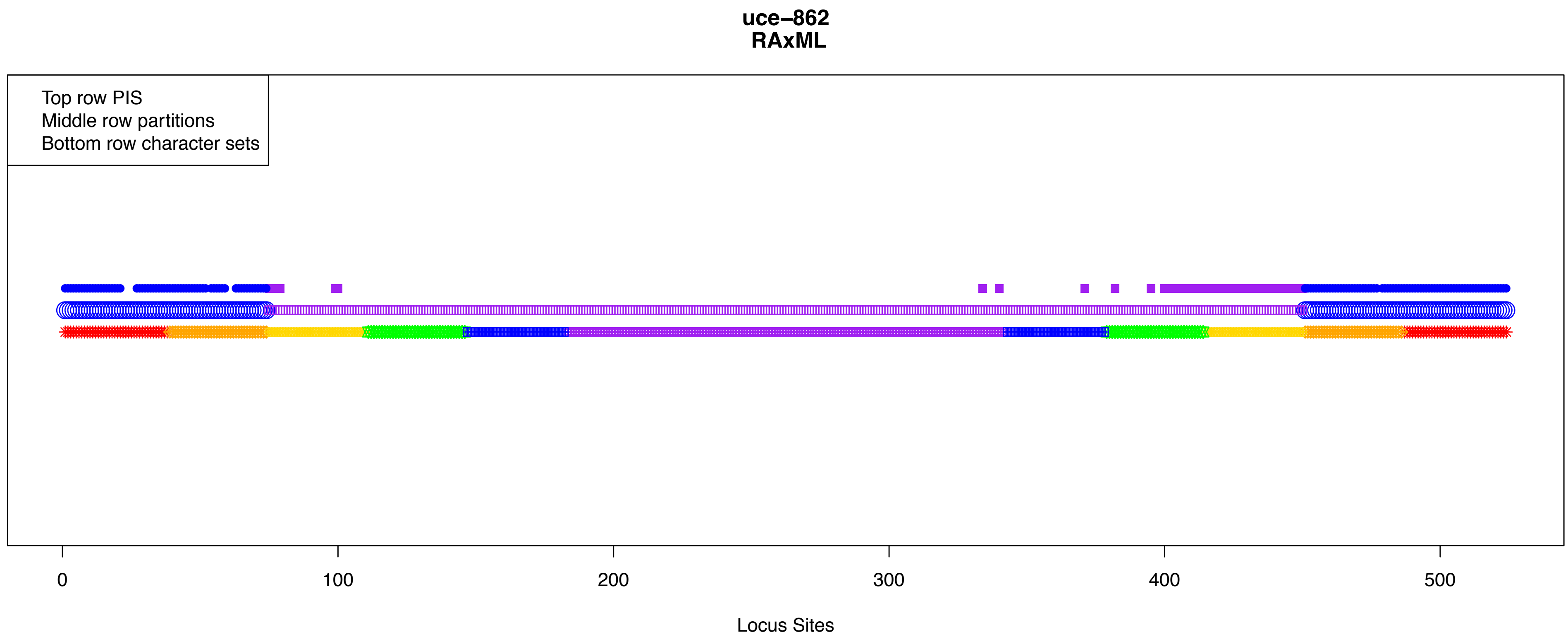
uce–862
RAxML
Top row PIS
Middle row partitions
Bottom row character sets
0
100
200
300
400
500
Locus Sites

uce–861
RAxML

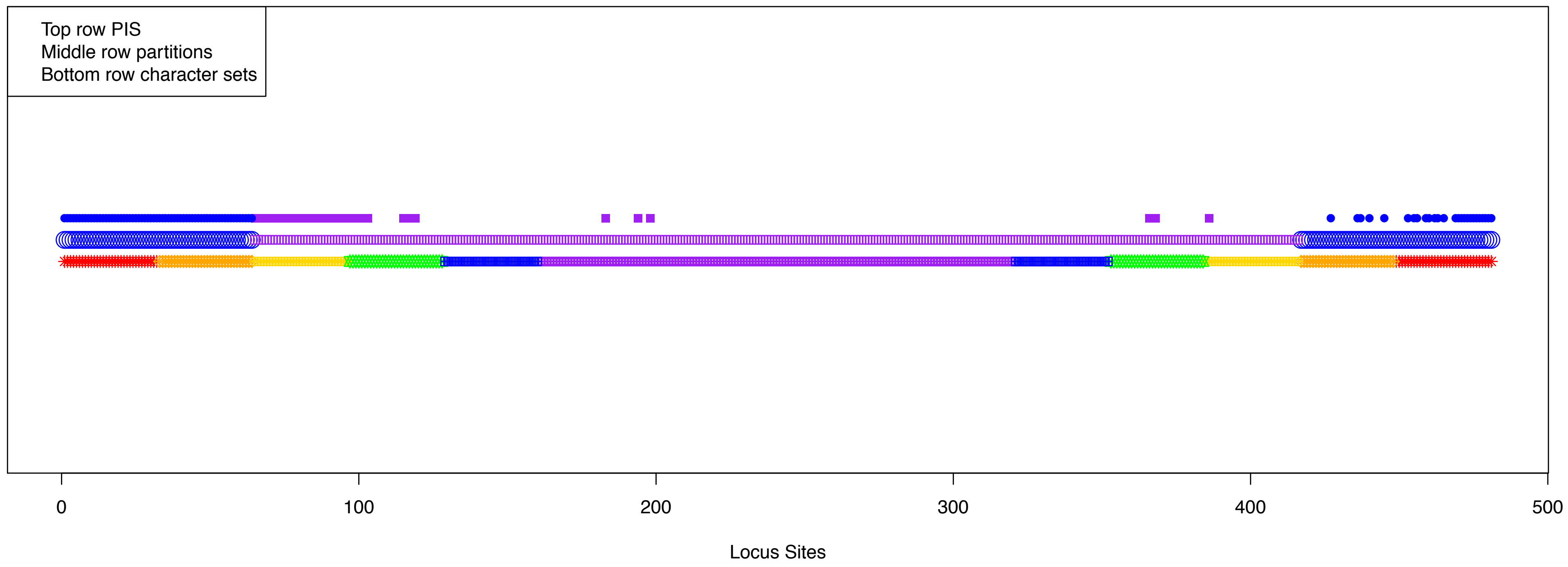

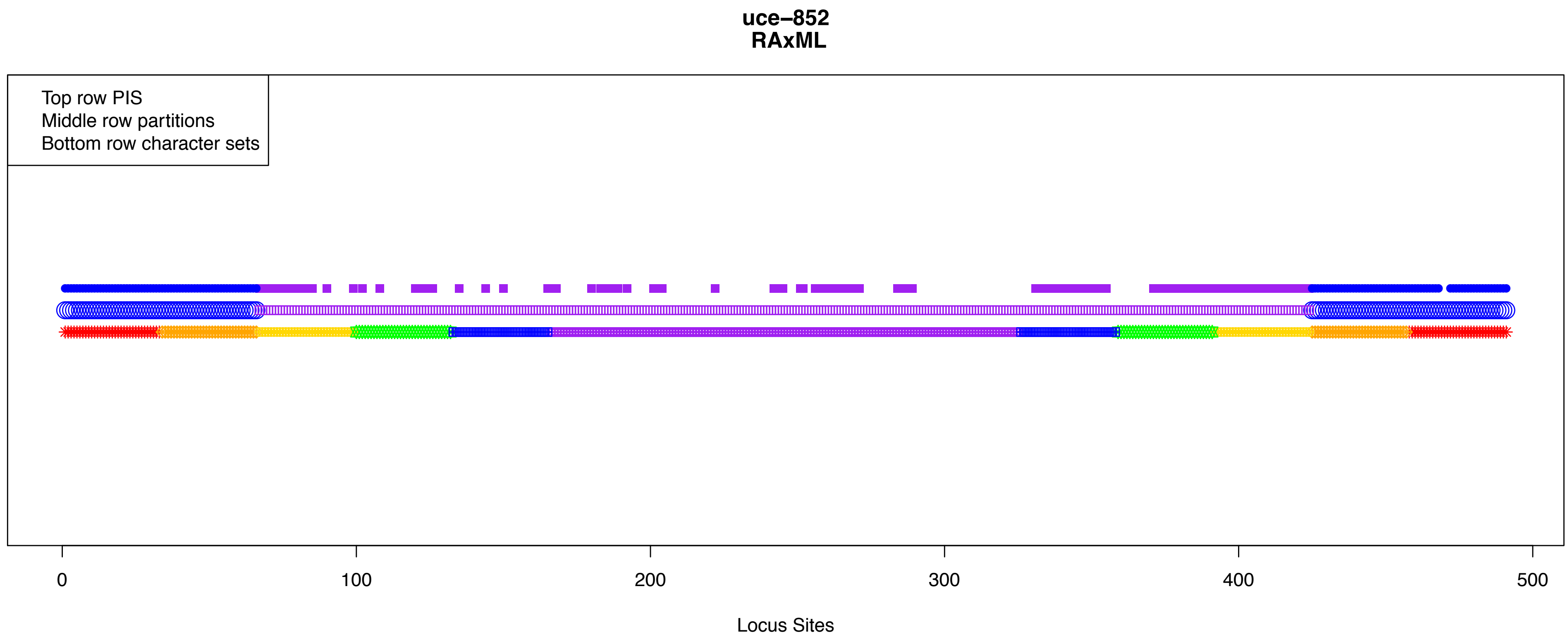
uce–852
RAxML
Top row PIS
Middle row partitions
Bottom row character sets
0
100
200
300
400
500
Locus Sites

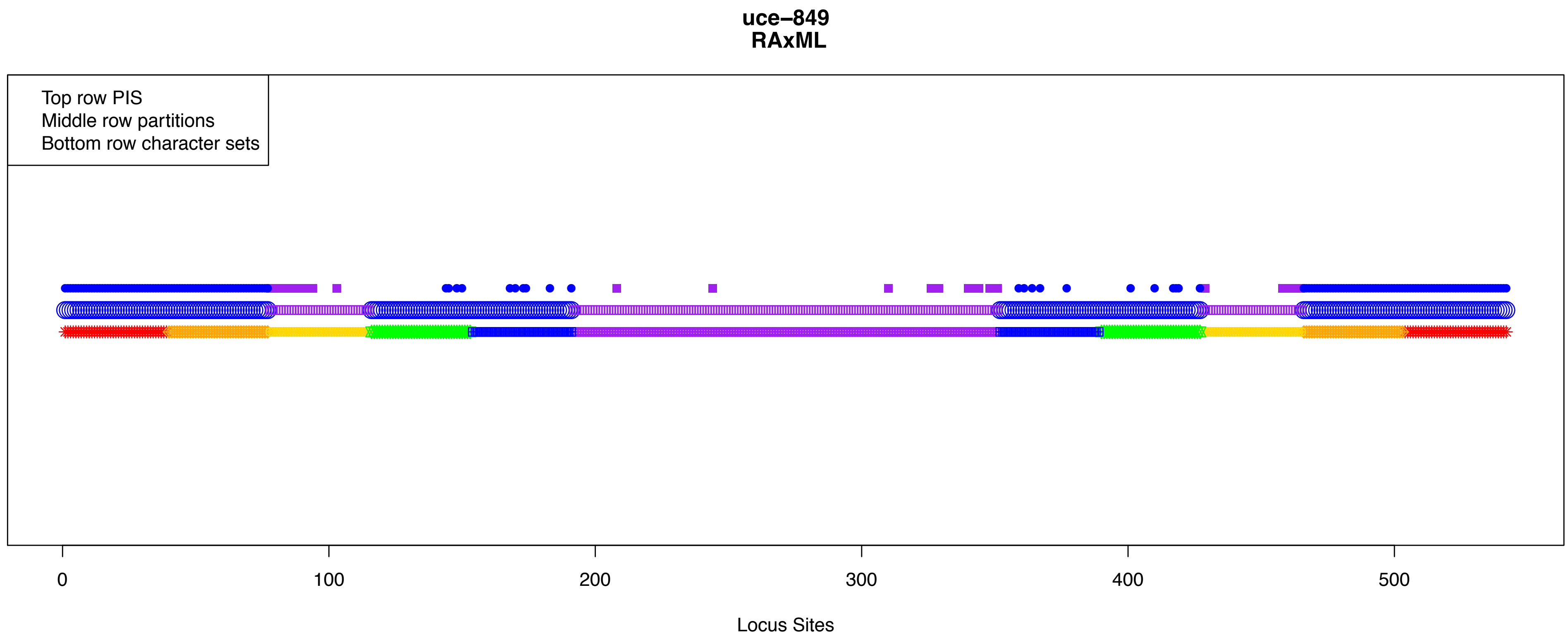

uce–849
RAxML
Top row PIS
Middle row partitions
Bottom row character sets
0
100
200
300
400
500
Locus Sites

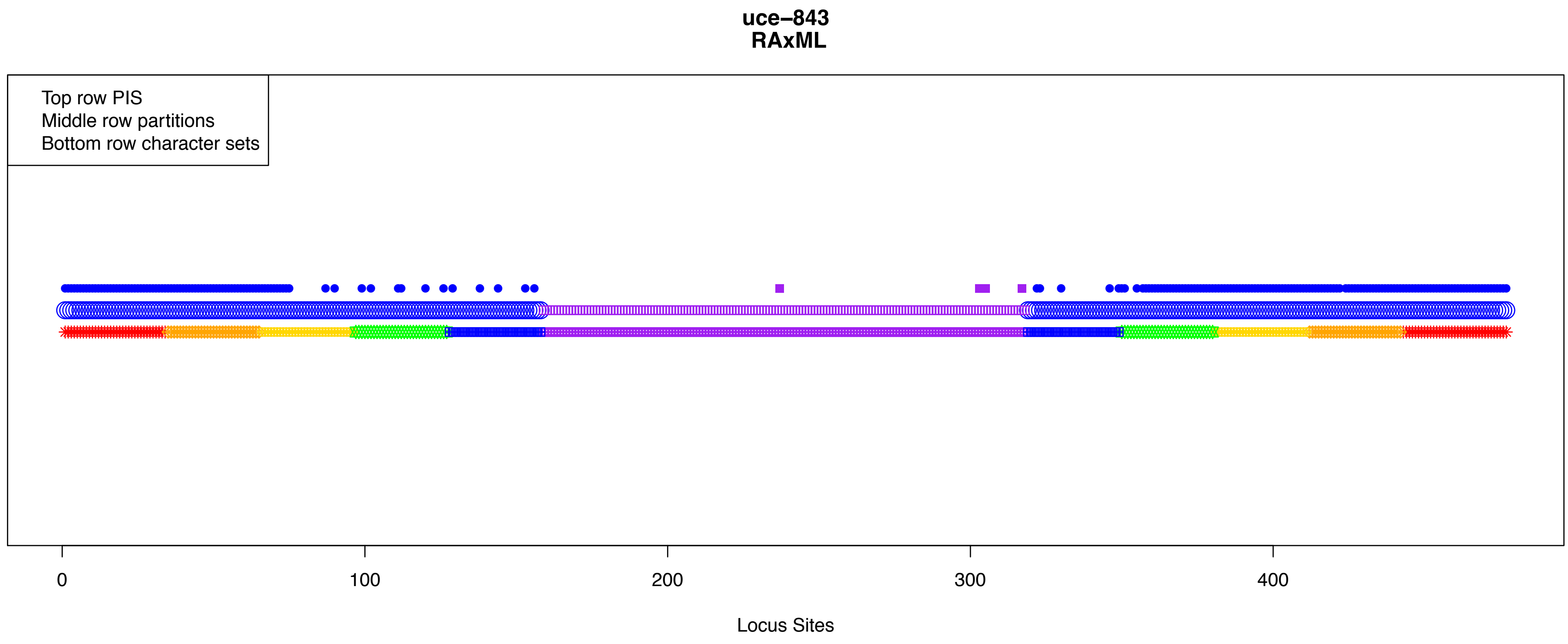

uce–843
RAxML
Top row PIS
Middle row partitions
Bottom row character sets
0
100
200
300
400
Locus Sites

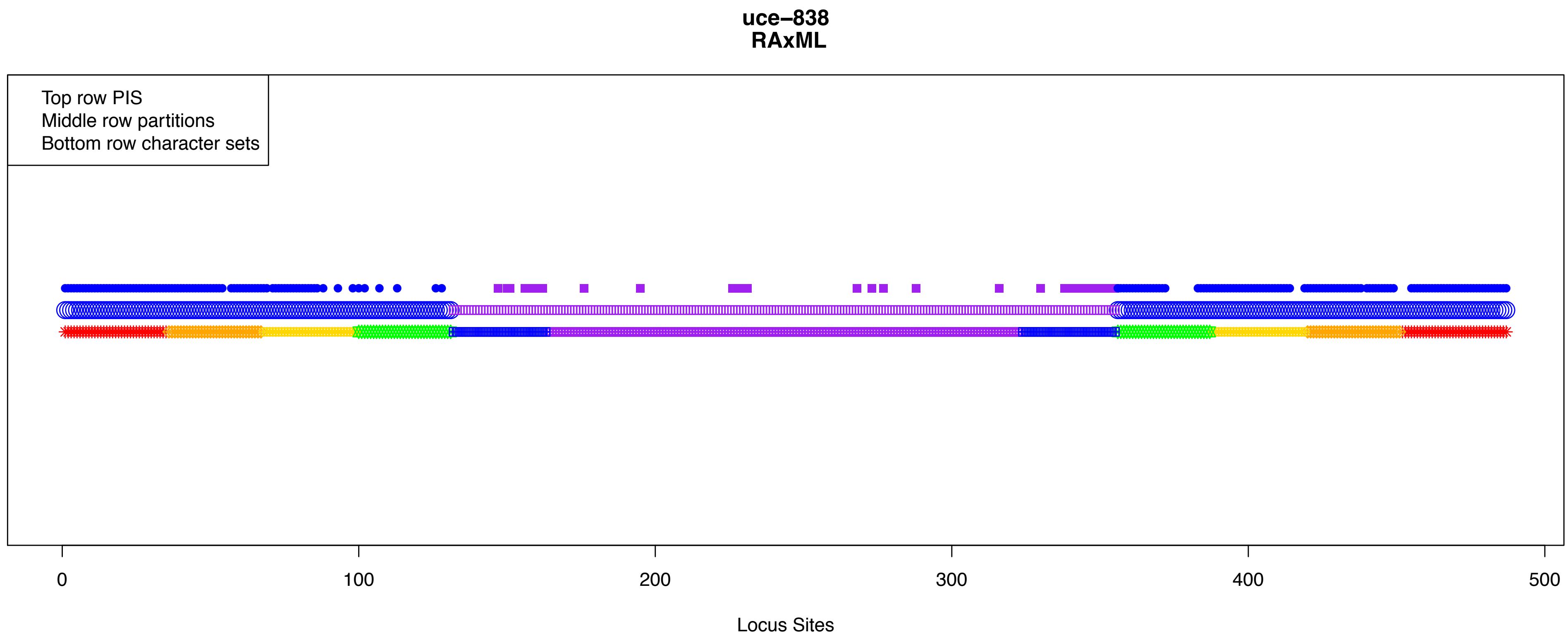

uce–838
RAxML
Top row PIS
Middle row partitions
Bottom row character sets
0
100
200
300
400
500
Locus Sites

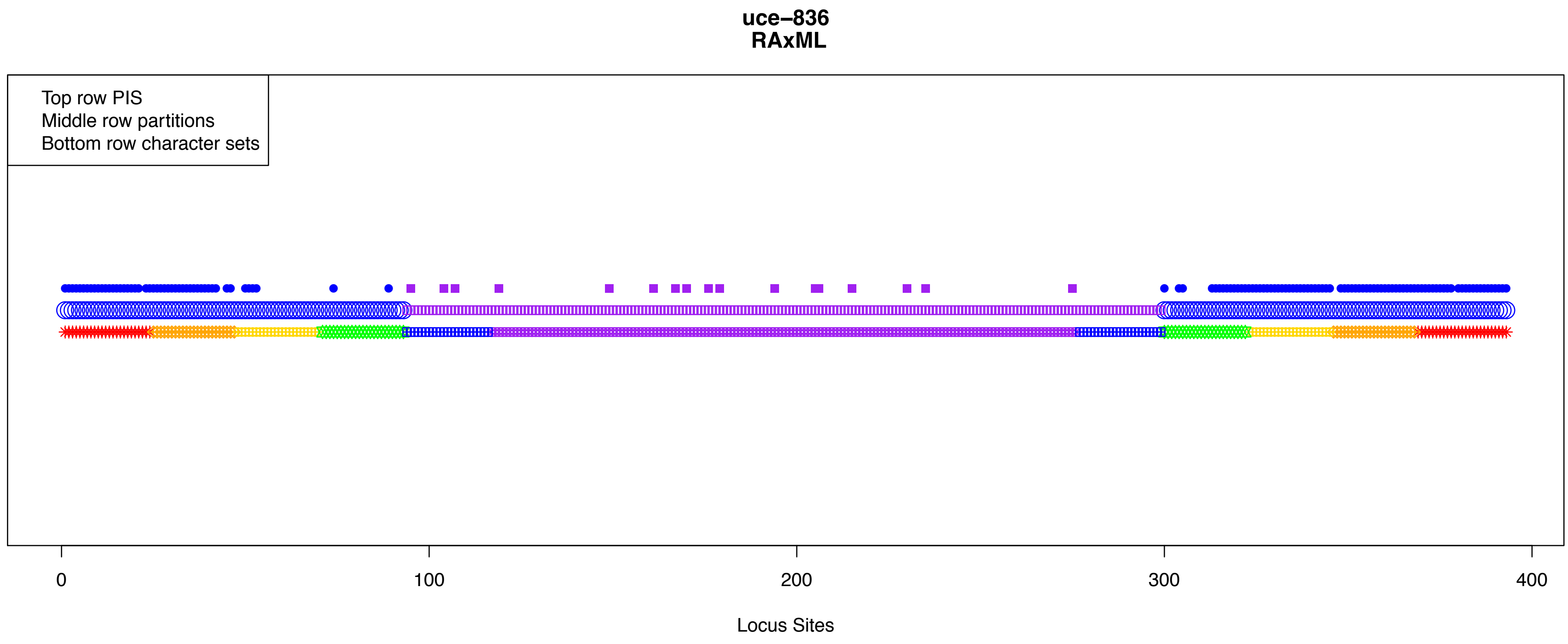
uce–836
RAxML
Top row PIS
Middle row partitions
Bottom row character sets
0
100
200
300
400
Locus Sites

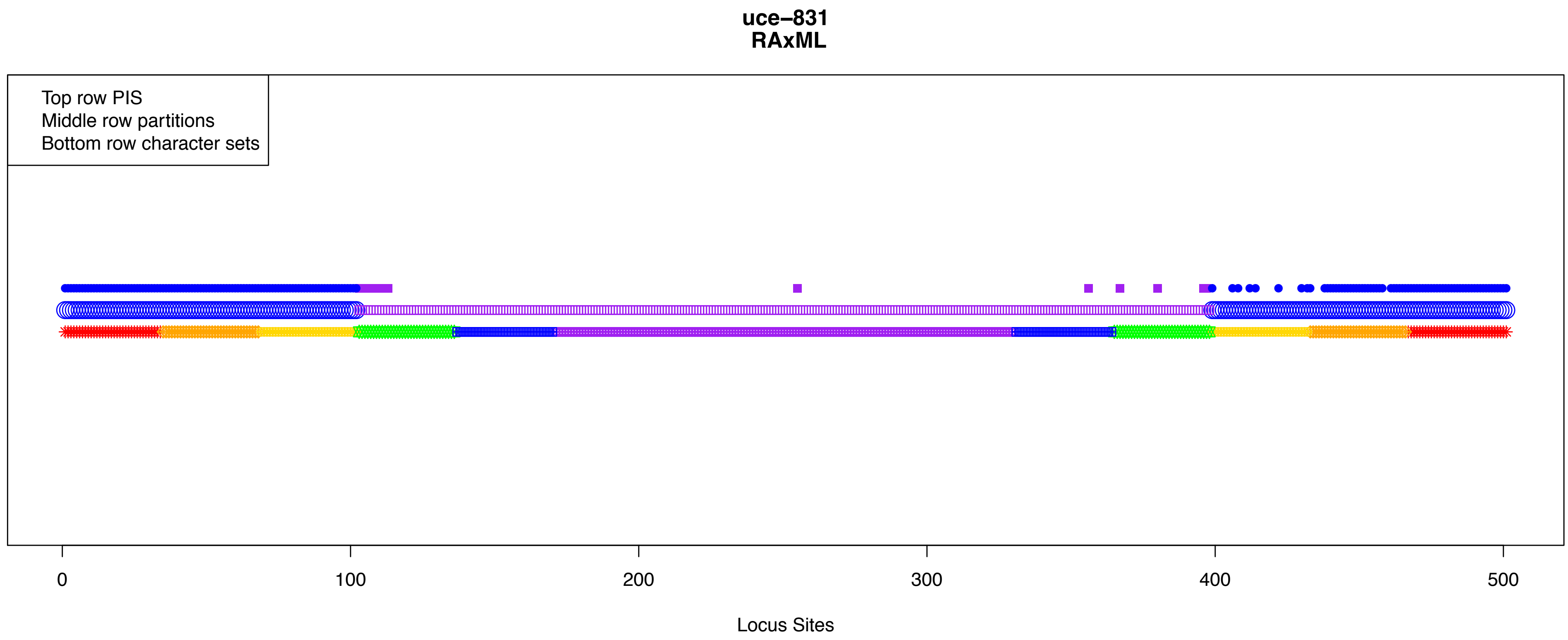
uce–831
RAxML
Top row PIS
Middle row partitions
Bottom row character sets
0
100
200
300
400
500
Locus Sites

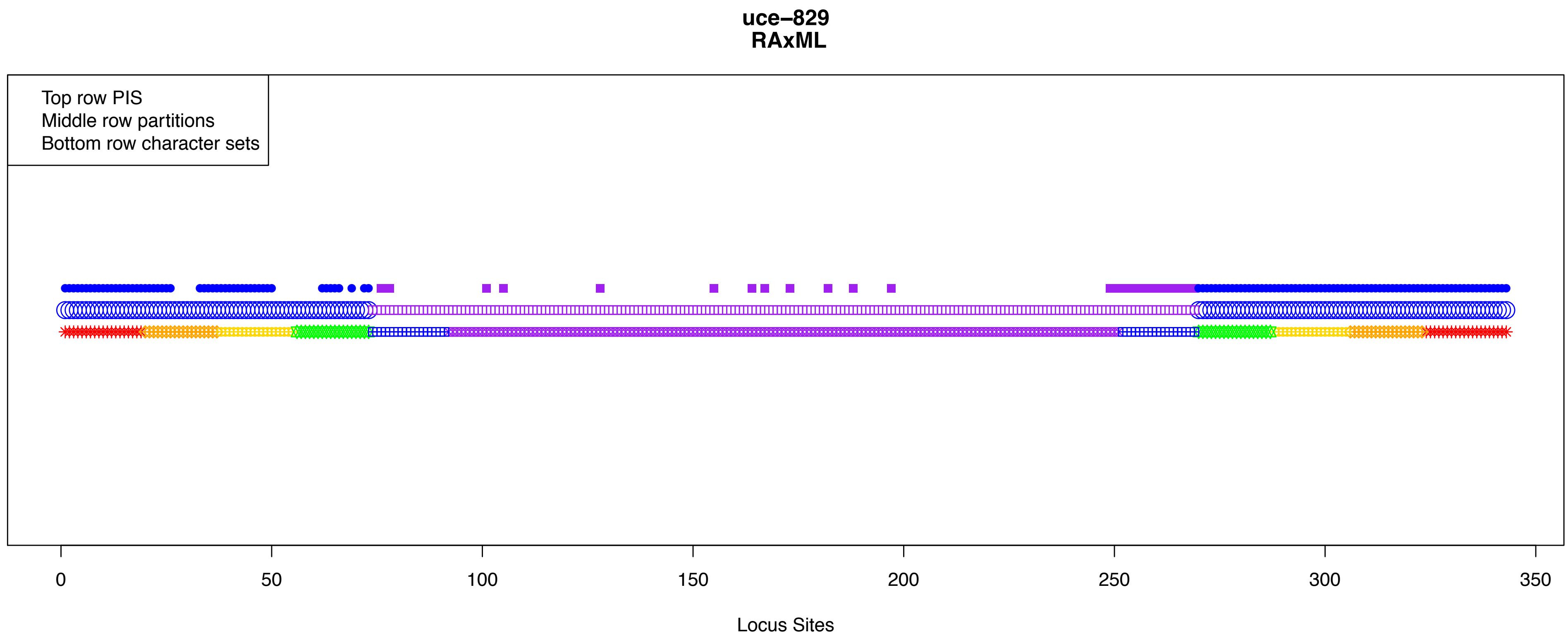
uce–829
RAxML
Top row PIS
Middle row partitions
Bottom row character sets
0
50
100
150
200
250
300
350
Locus Sites

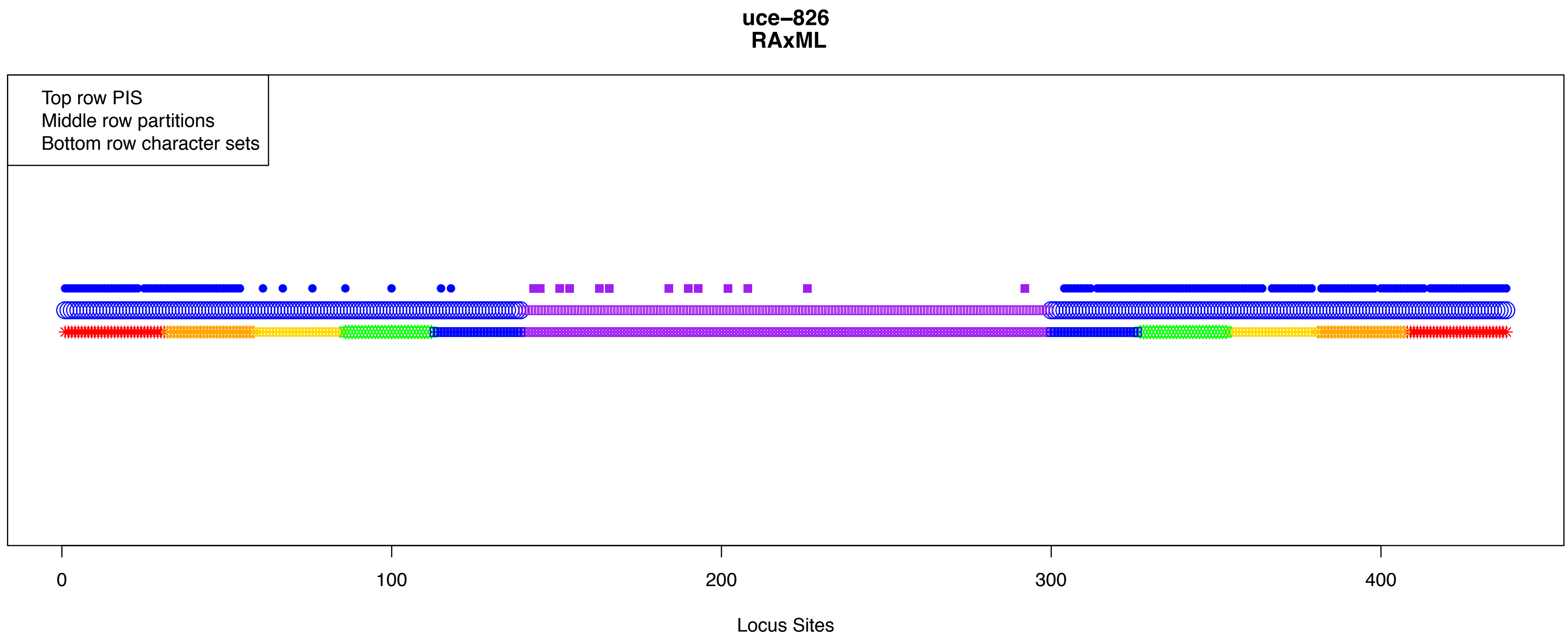
uce–826
RAxML
Top row PIS
Middle row partitions
Bottom row character sets
0
100
200
300
400
Locus Sites

uce–804
RAxML

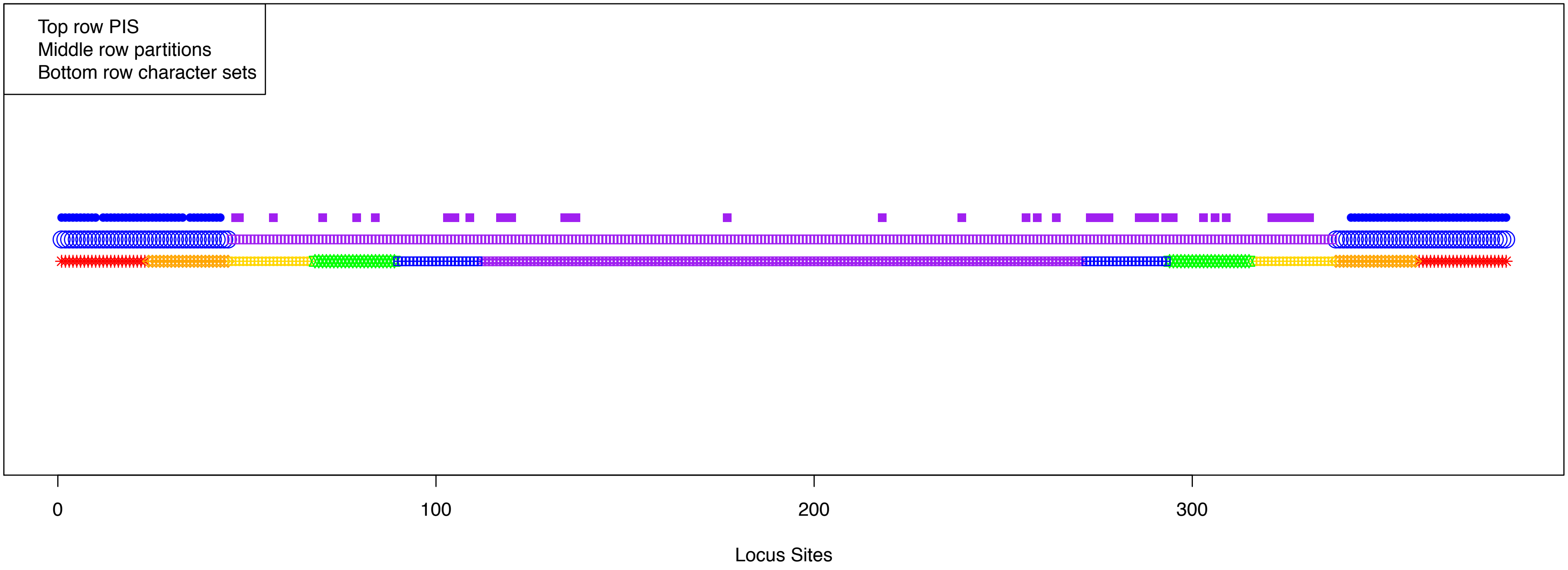

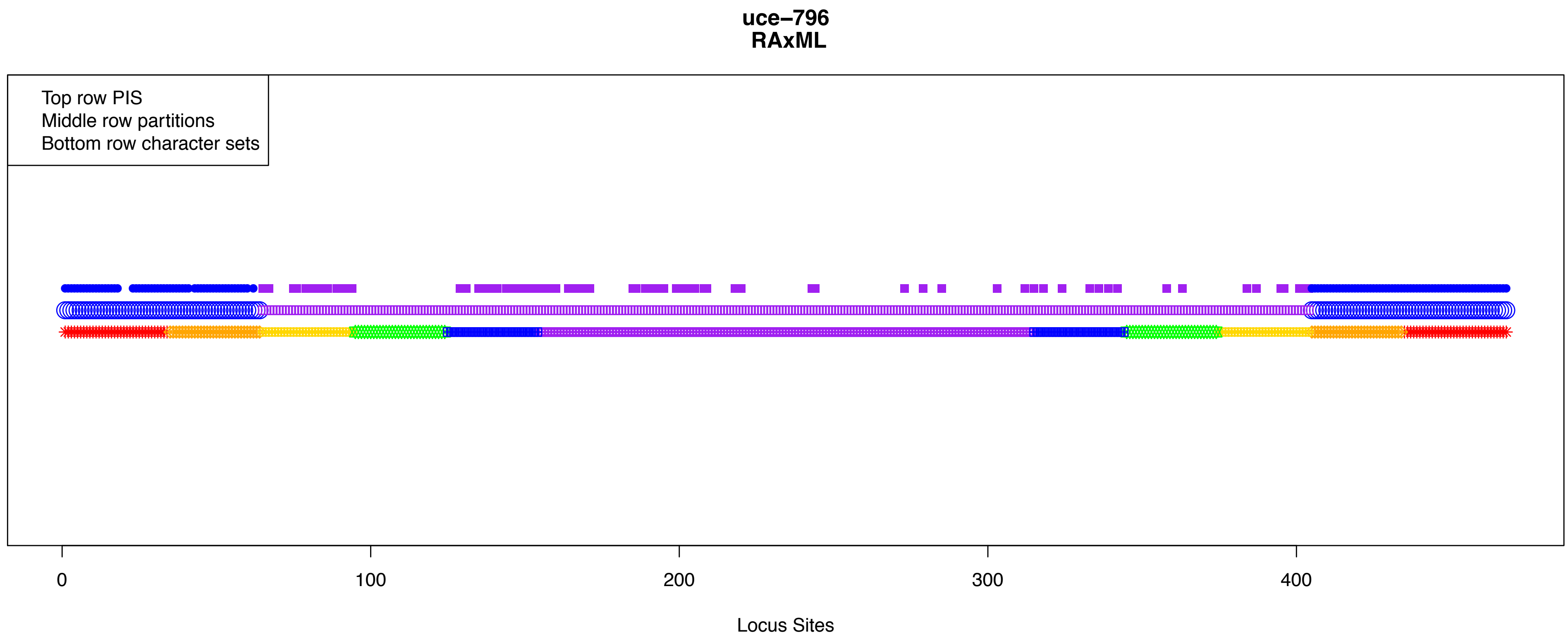

uce–796
RAxML
Top row PIS
Middle row partitions
Bottom row character sets
0
100
200
300
400
Locus Sites

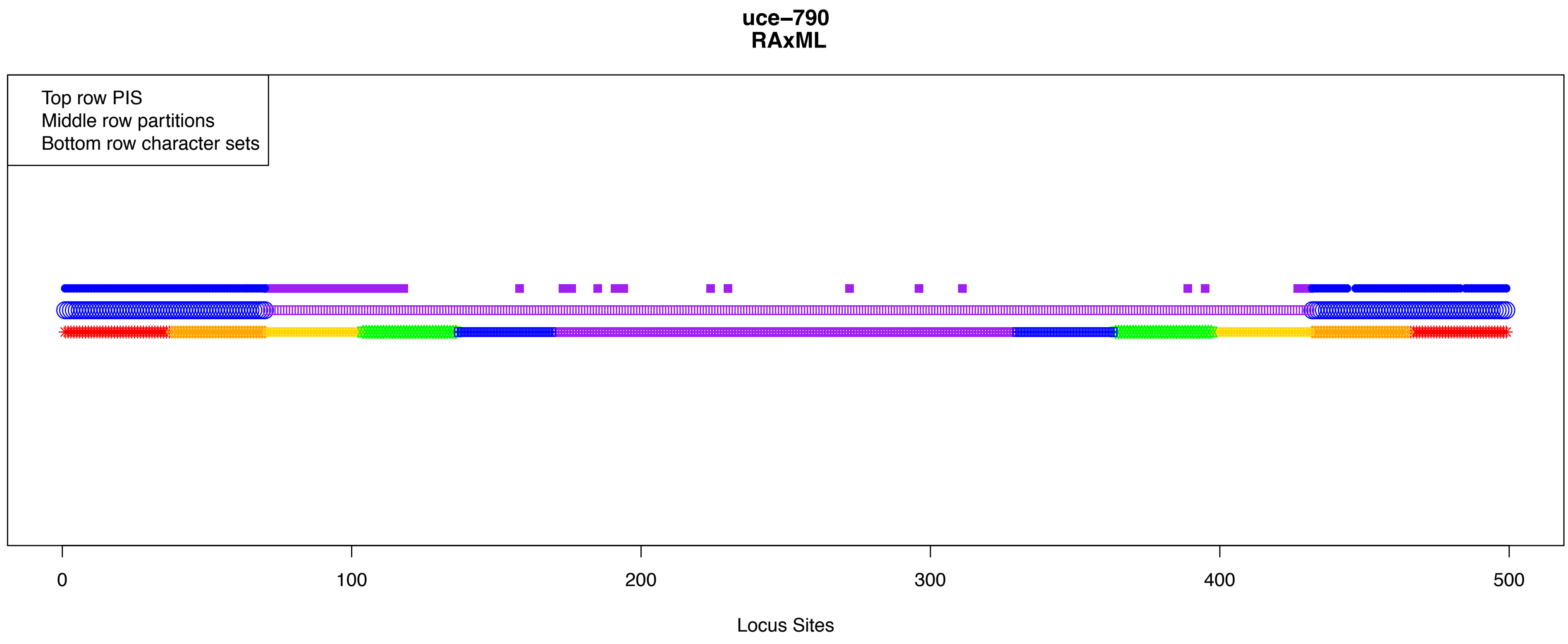
uce–790
RAxML
Top row PIS
Middle row partitions
Bottom row character sets
0
100
200
300
400
500
Locus Sites

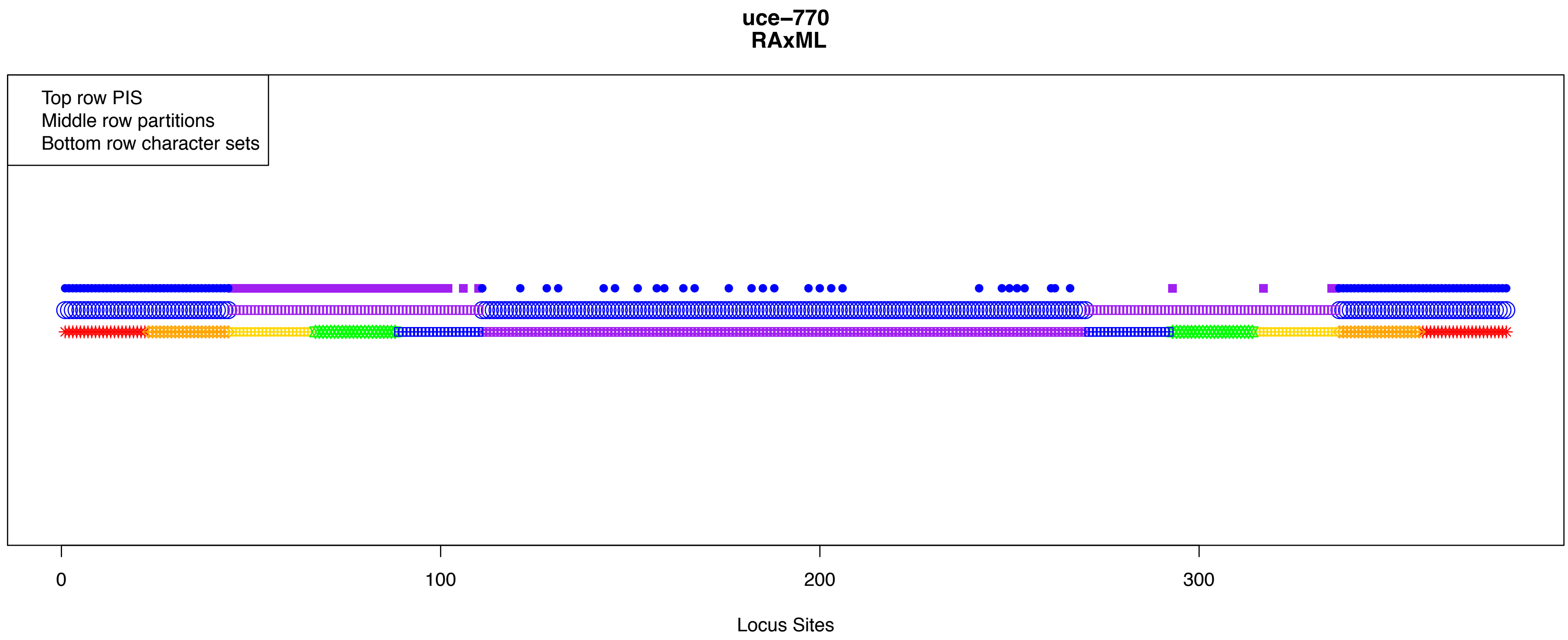
uce–770
RAxML
Top row PIS
Middle row partitions
Bottom row character sets
0
100
200
300
Locus Sites

uce–761
RAxML

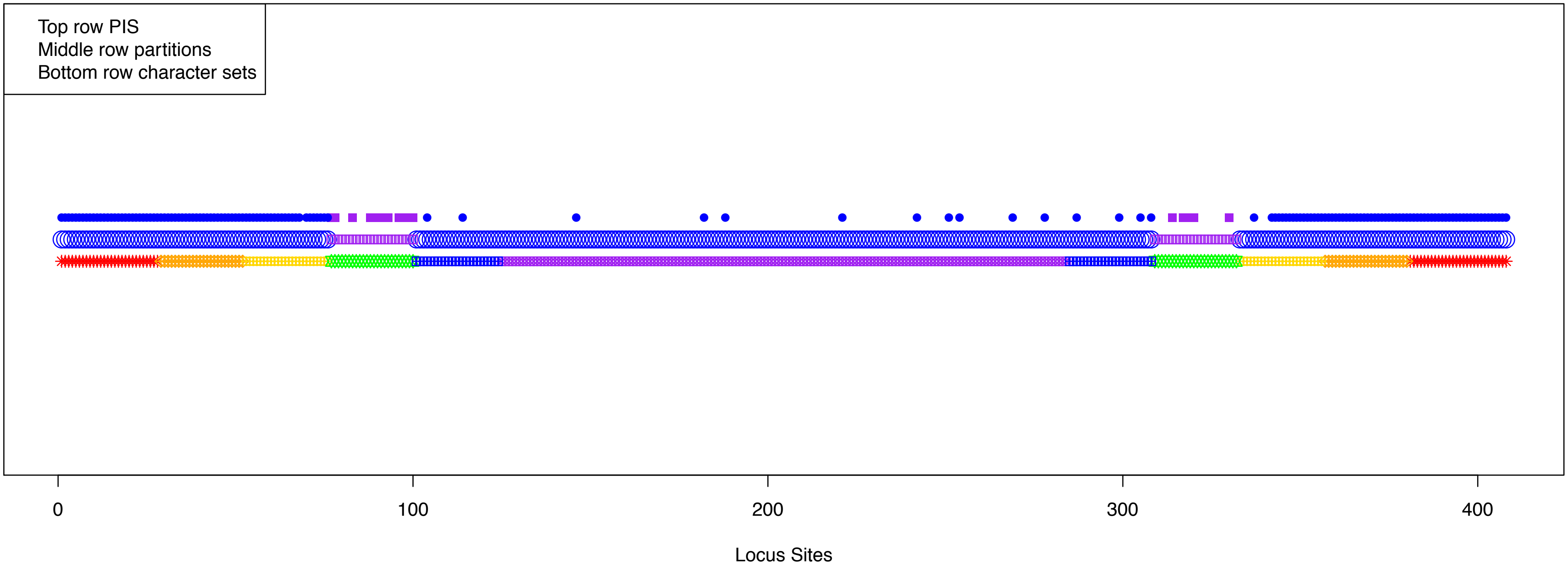

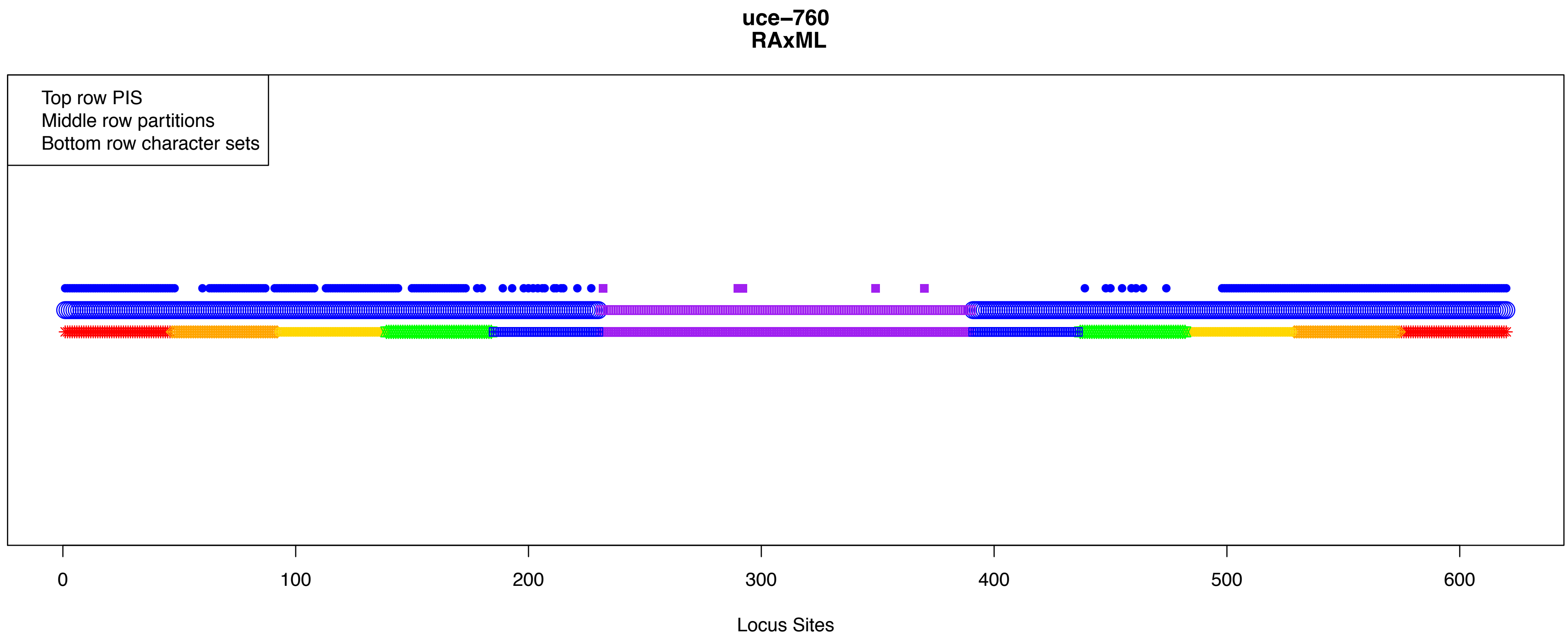

uce–760
RAxML
Top row PIS
Middle row partitions
Bottom row character sets
0
100
200
300
400
500
600
Locus Sites

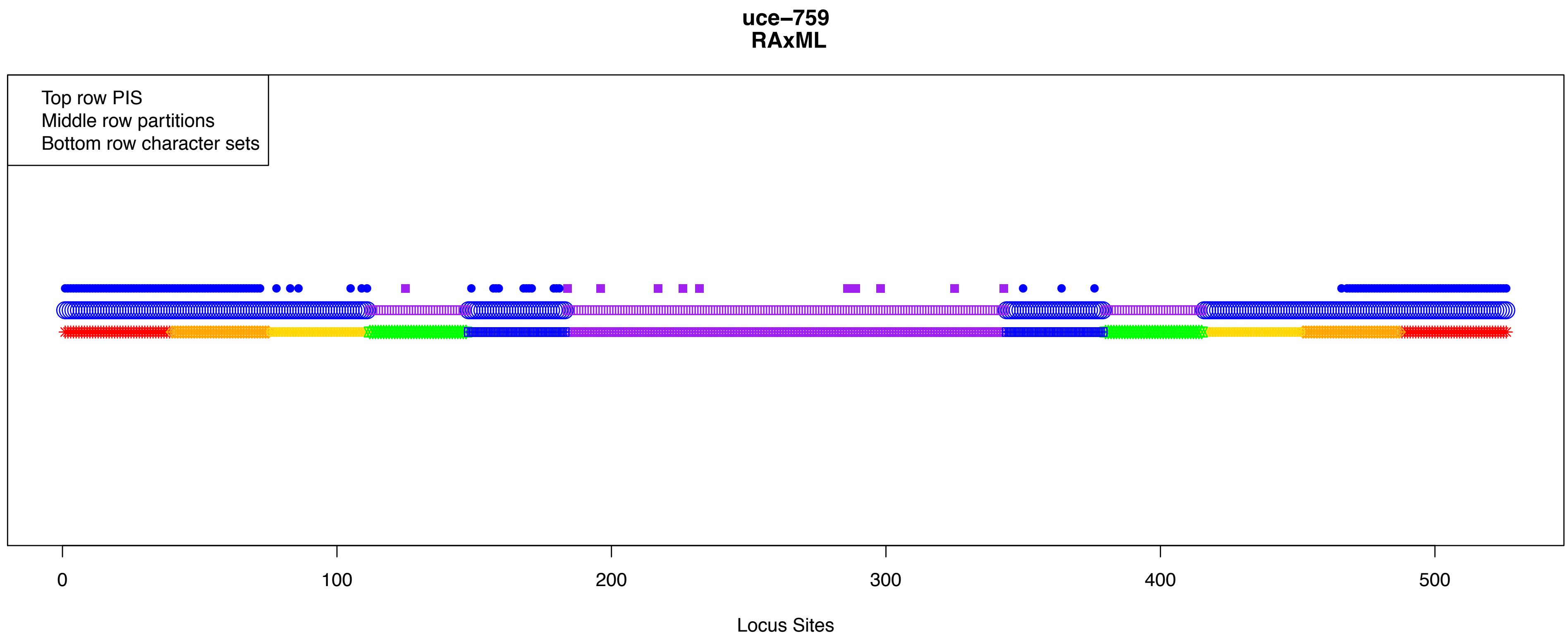

uce–759
RAxML
Top row PIS
Middle row partitions
Bottom row character sets
0
100
200
300
400
500
Locus Sites

uce–756
RAxML

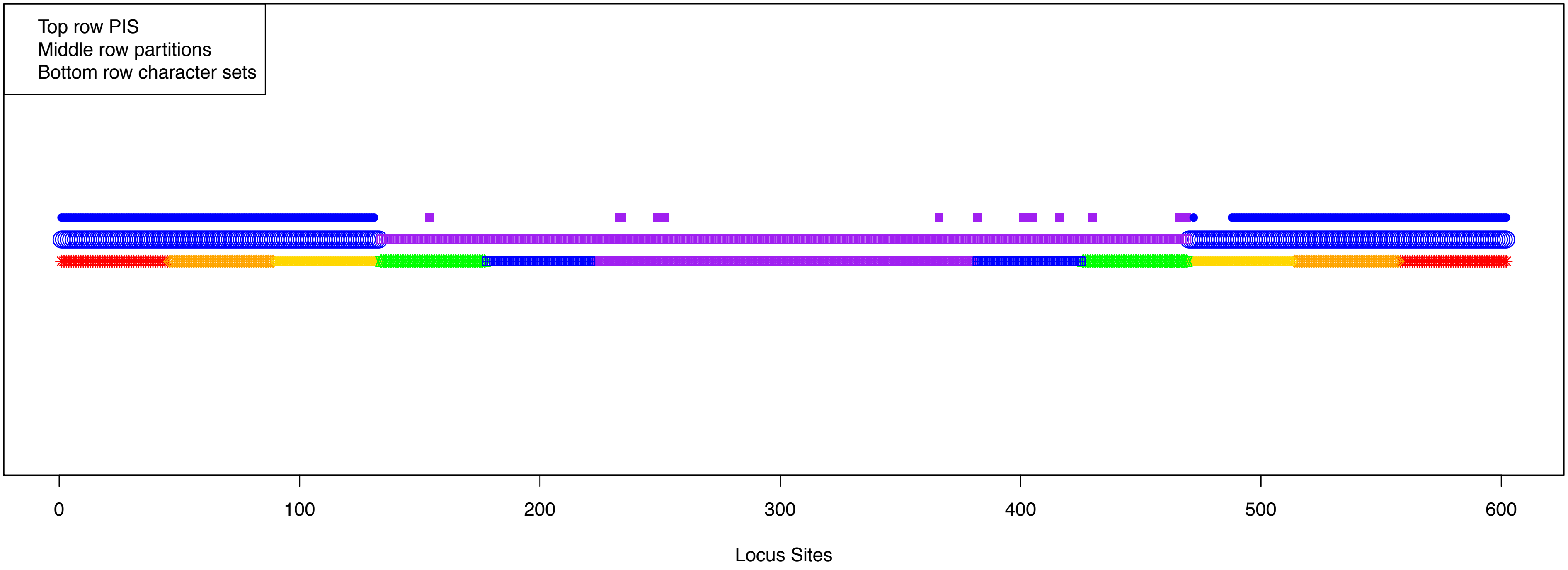

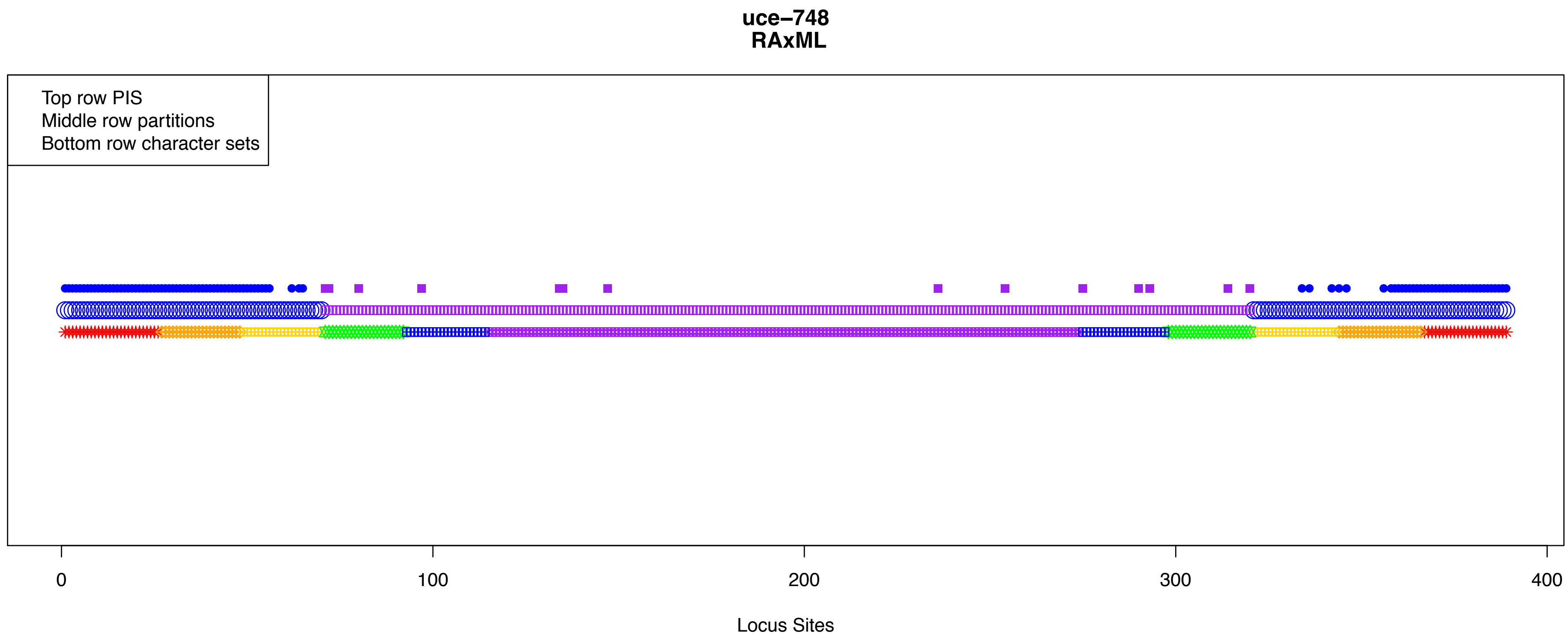
uce–748
RAxML
Top row PIS
Middle row partitions
Bottom row character sets
0
100
200
300
400
Locus Sites

uce–745
RAxML

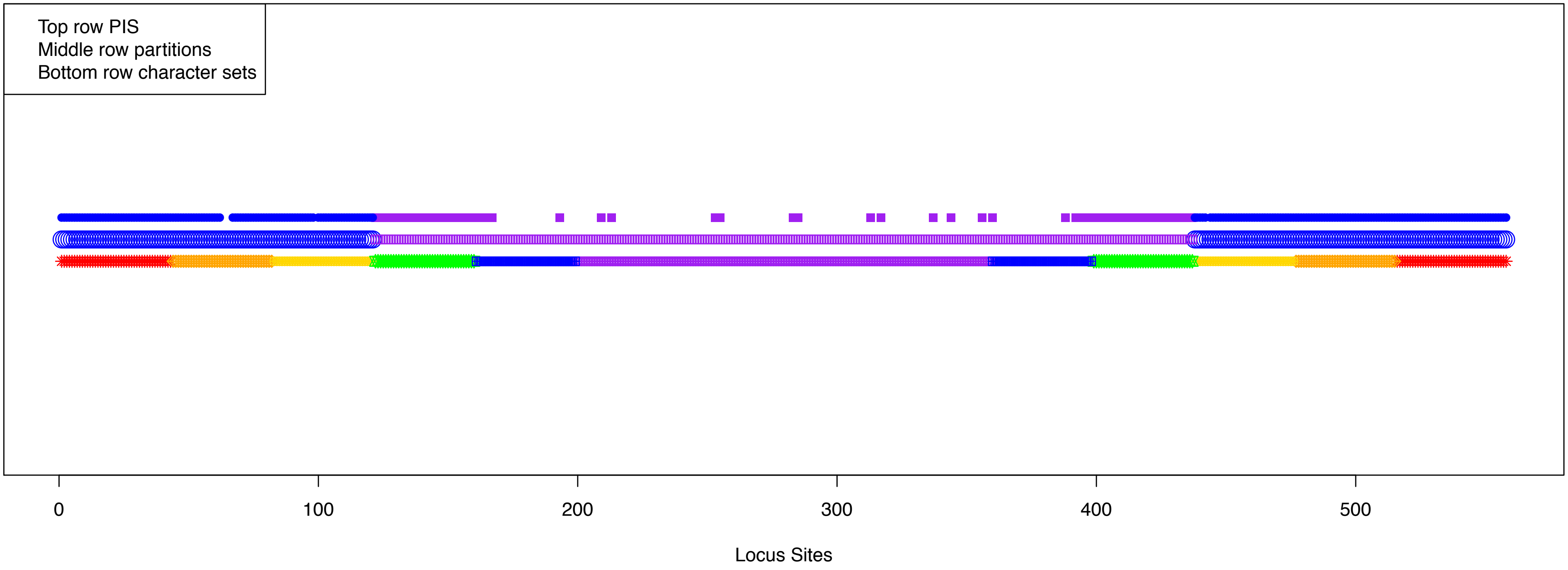

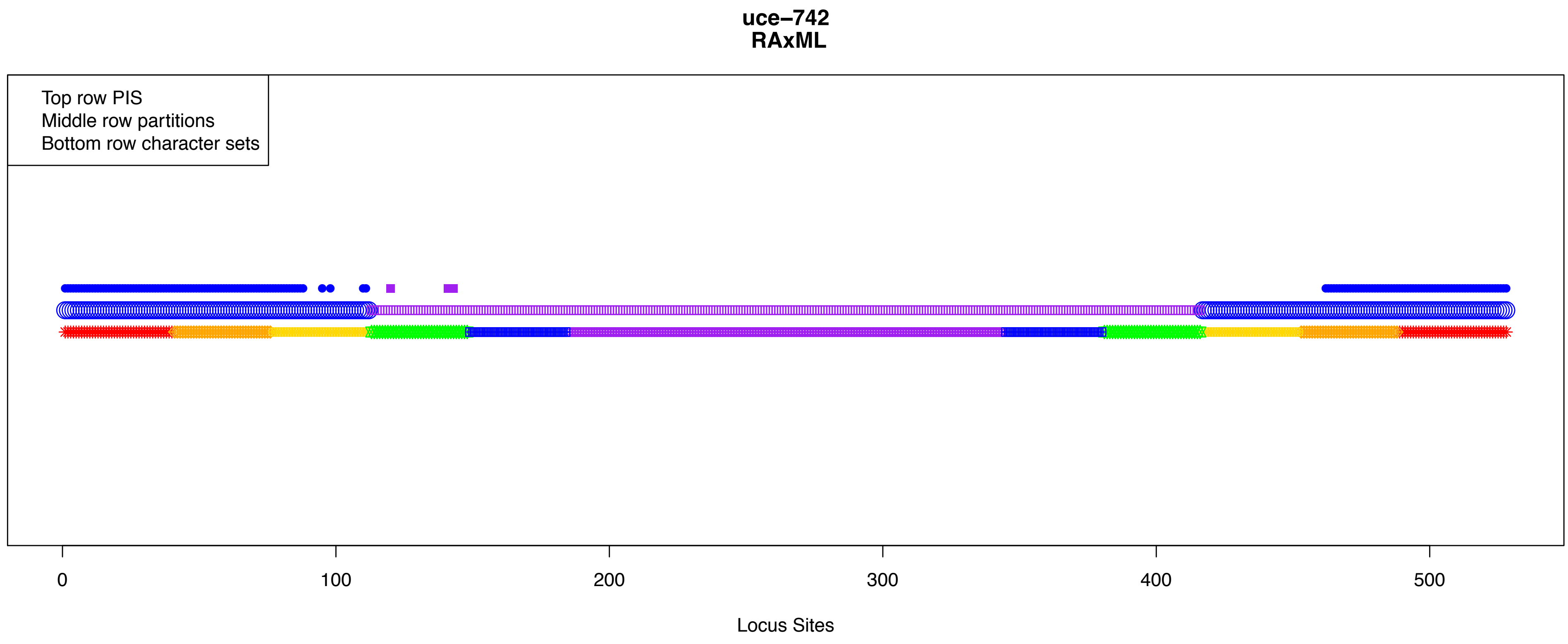
uce–742
RAxML
Top row PIS
Middle row partitions
Bottom row character sets
0
100
200
300
400
500
Locus Sites

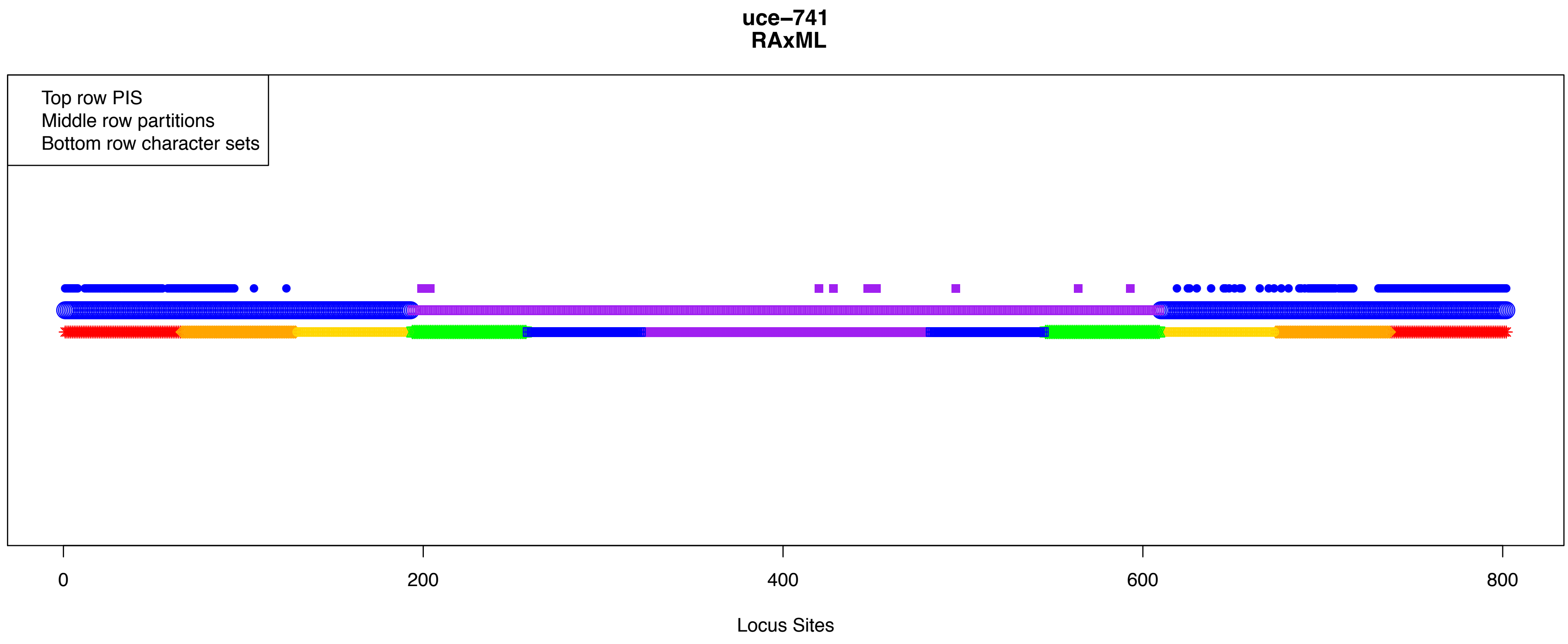

uce–741
RAxML
Top row PIS
Middle row partitions
Bottom row character sets
0
200
400
600
800
Locus Sites

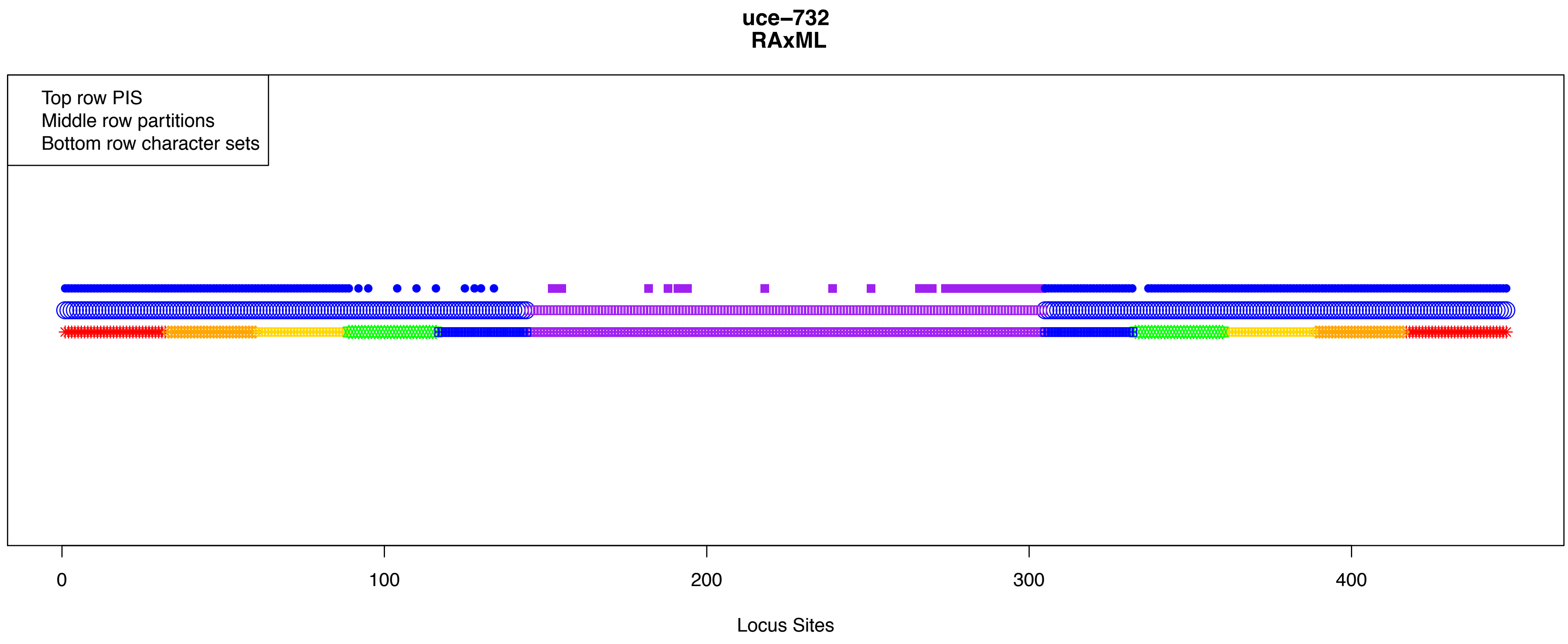
uce–732
RAxML
Top row PIS
Middle row partitions
Bottom row character sets
0
100
200
300
400
Locus Sites

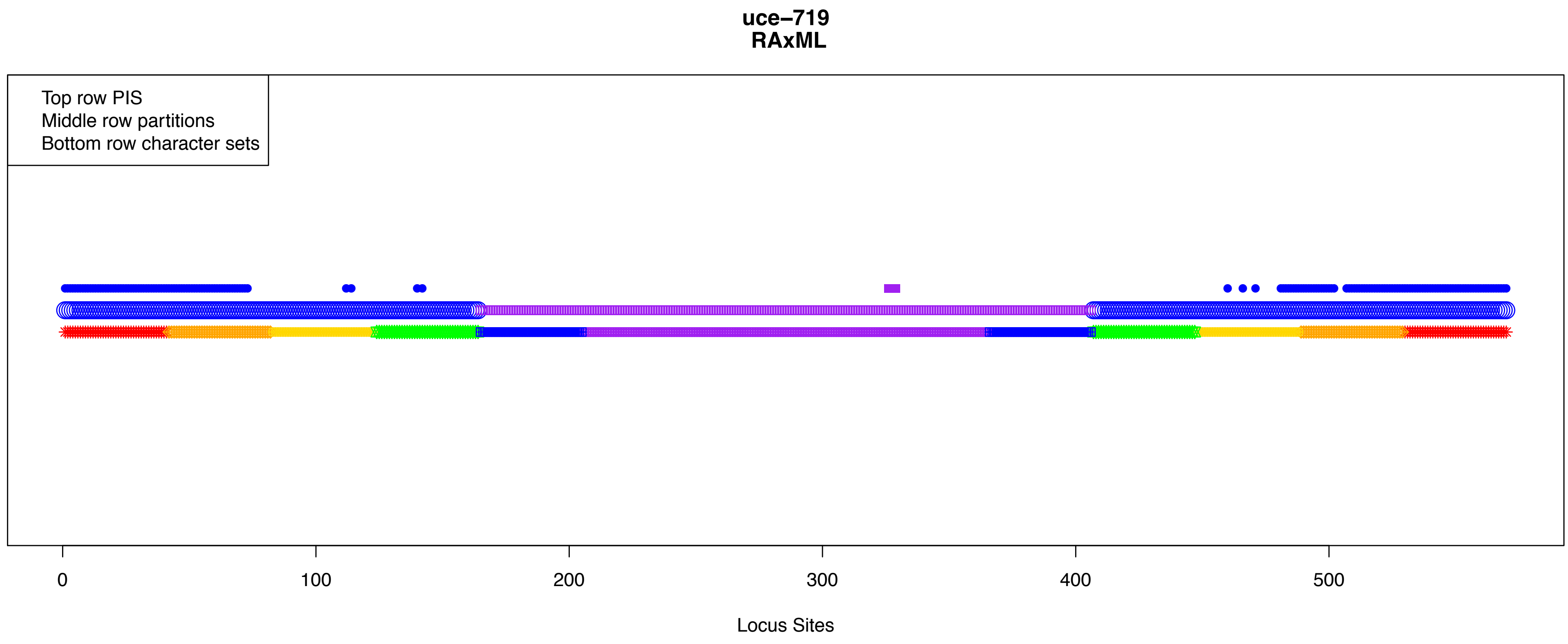
uce–719
RAxML
Top row PIS
Middle row partitions
Bottom row character sets
0
100
200
300
400
500
Locus Sites

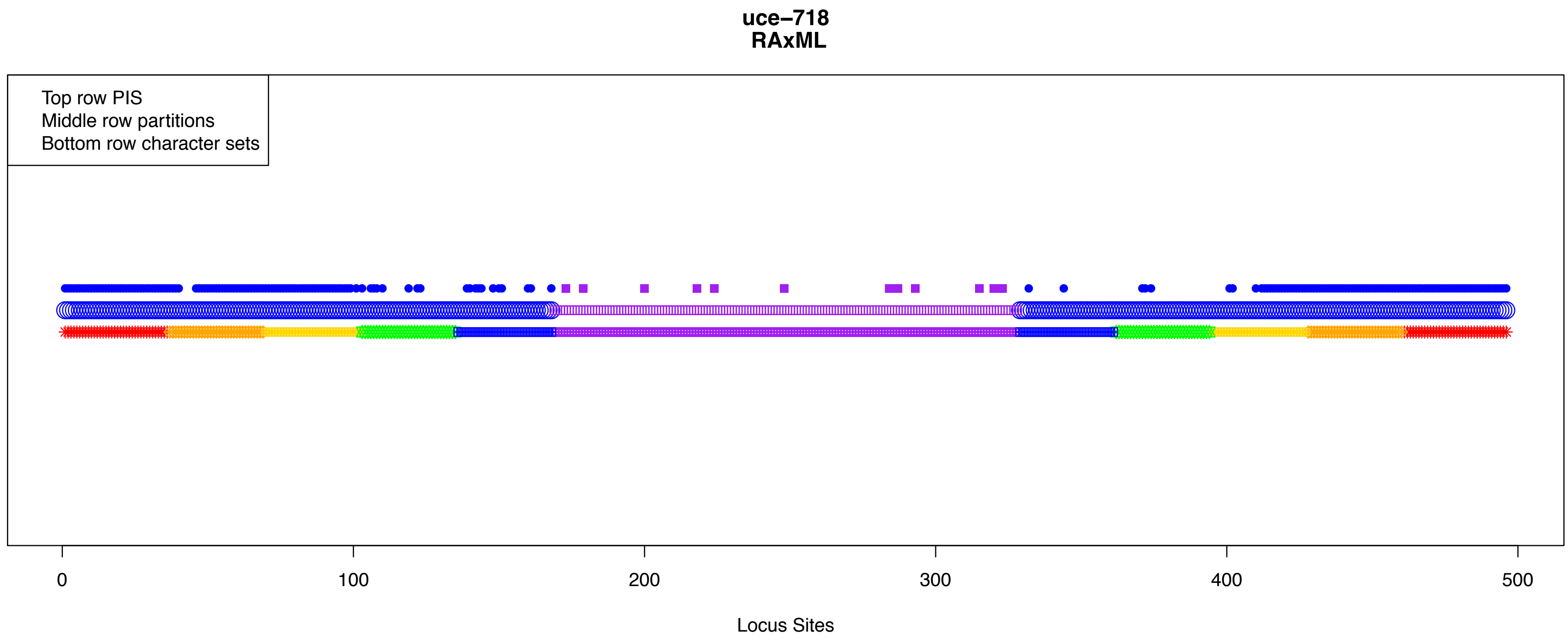

uce–718
RAxML
Top row PIS
Middle row partitions
Bottom row character sets
0
100
200
300
400
500
Locus Sites

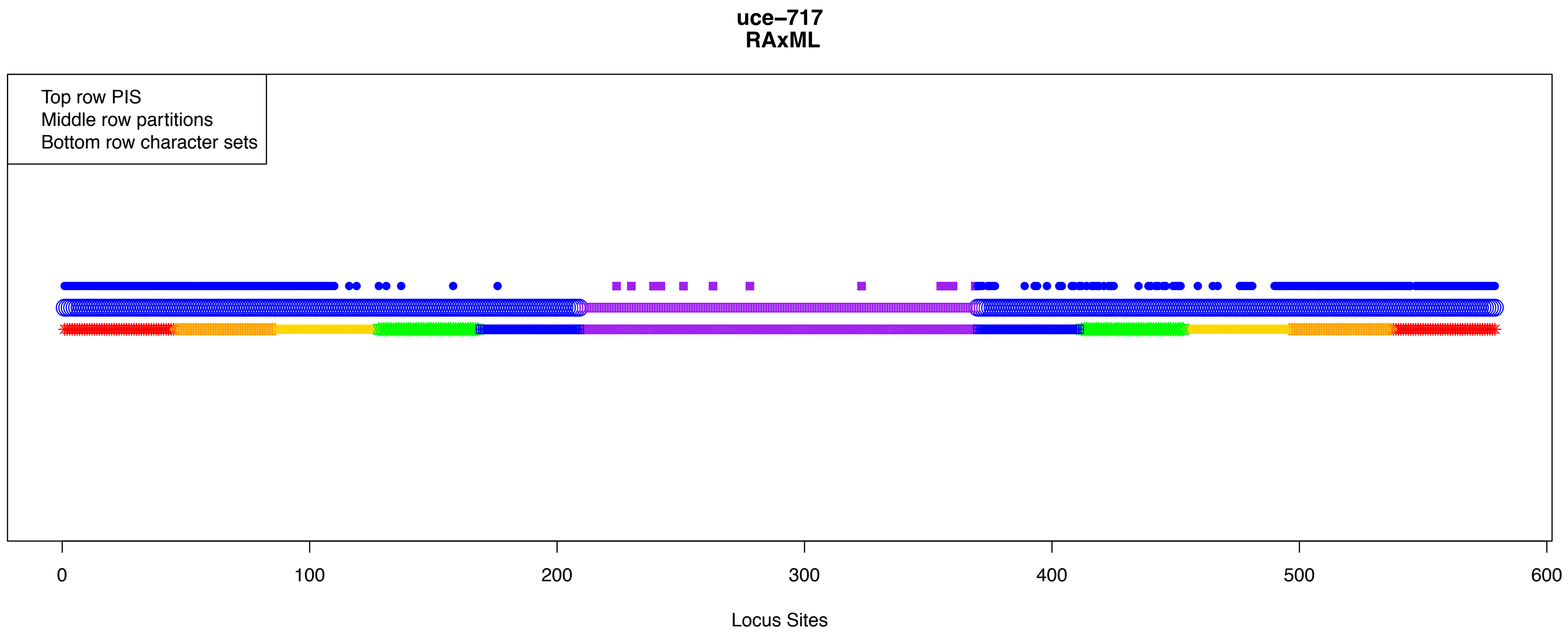

uce–717
RAxML
Top row PIS
Middle row partitions
Bottom row character sets
0
100
200
300
400
500
600
Locus Sites

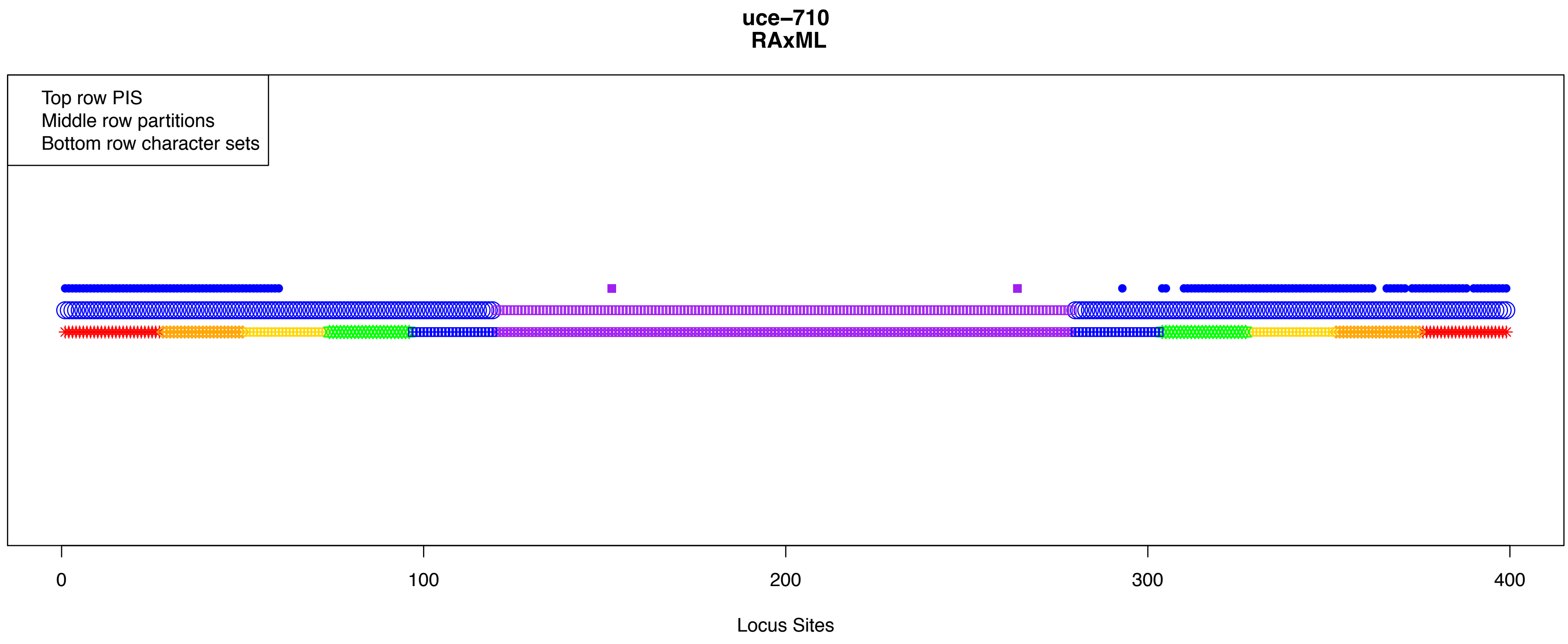
uce–710
RAxML
Top row PIS
Middle row partitions
Bottom row character sets
0
100
200
300
400
Locus Sites

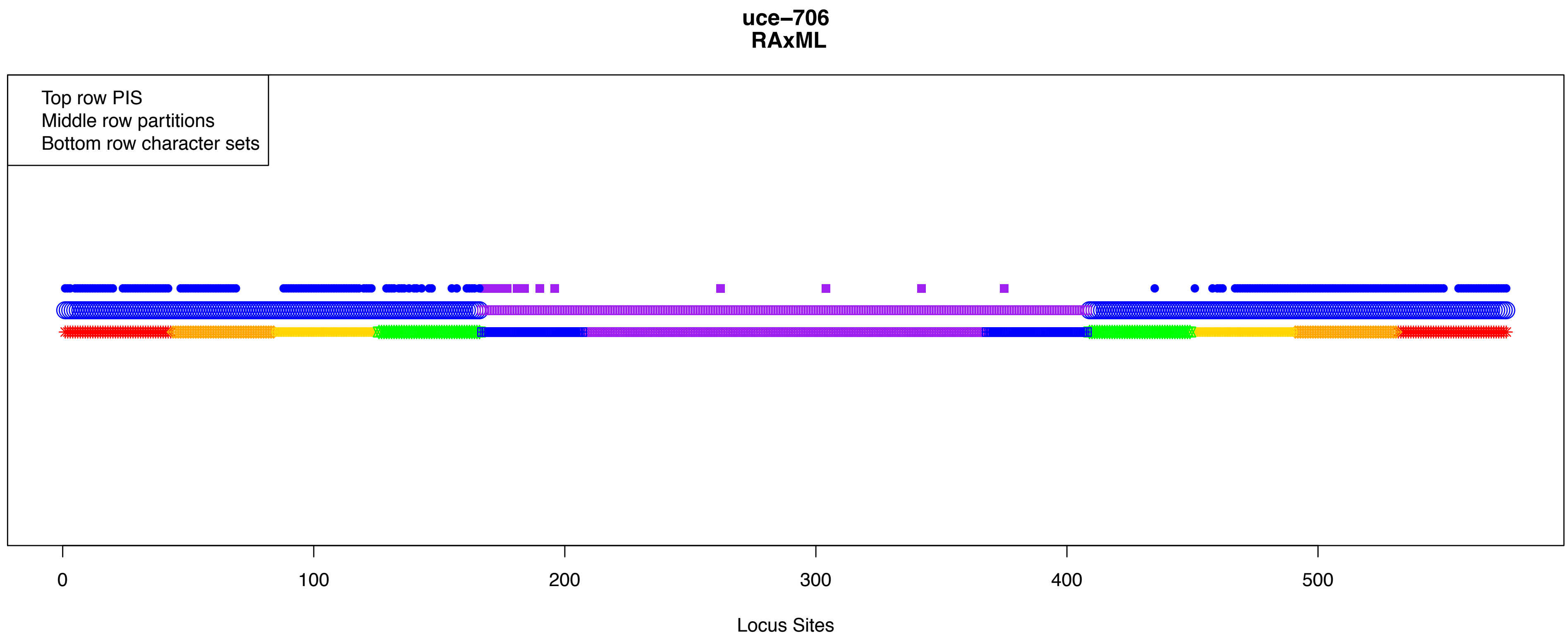
uce–706
RAxML
Top row PIS
Middle row partitions
Bottom row character sets
0
100
200
300
400
500
Locus Sites

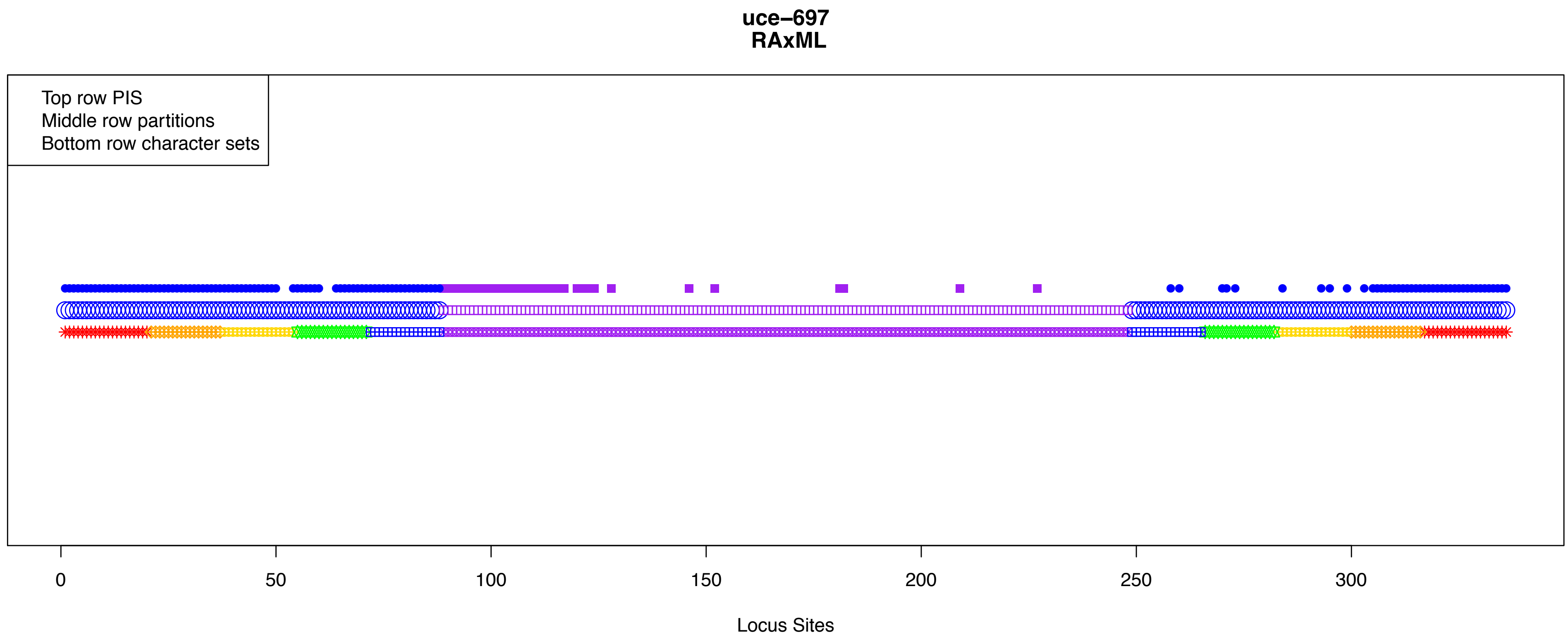
uce–697
RAxML
Top row PIS
Middle row partitions
Bottom row character sets
0
50
100
150
200
250
300
Locus Sites

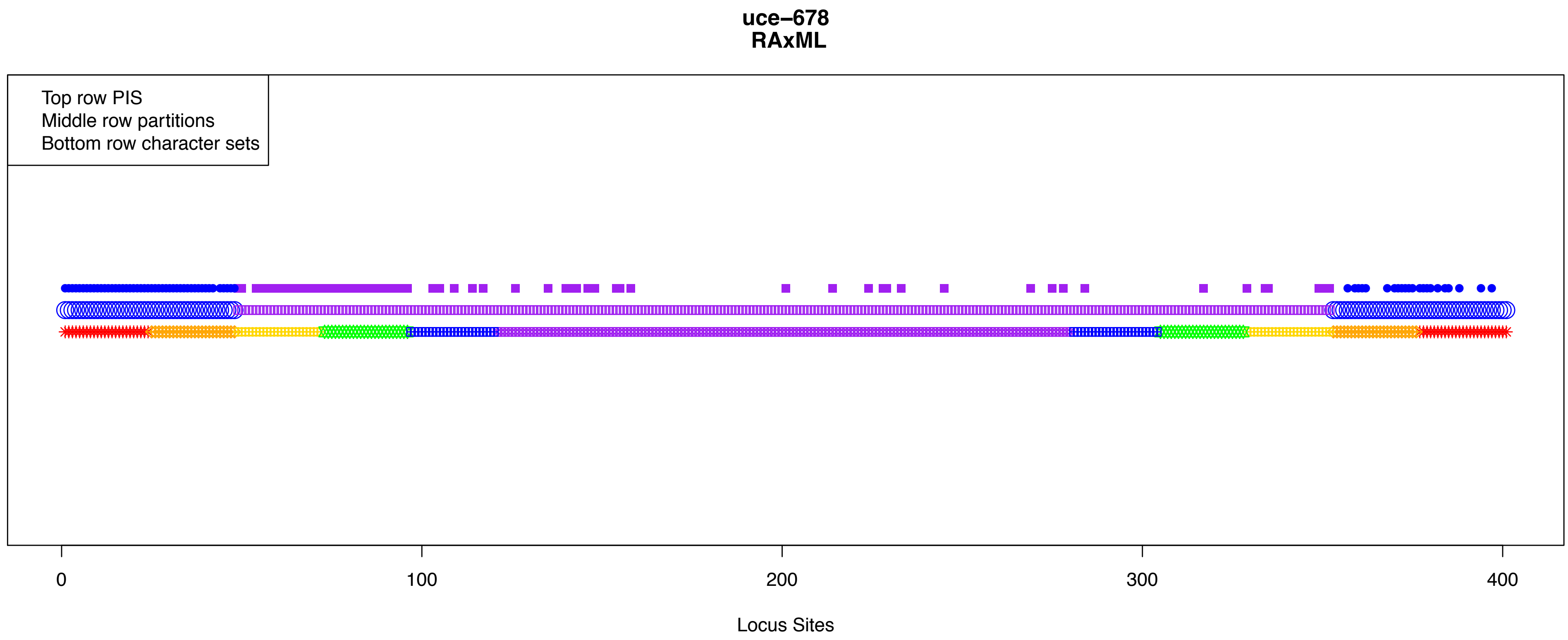
uce–678
RAxML
Top row PIS
Middle row partitions
Bottom row character sets
0
100
200
300
400
Locus Sites

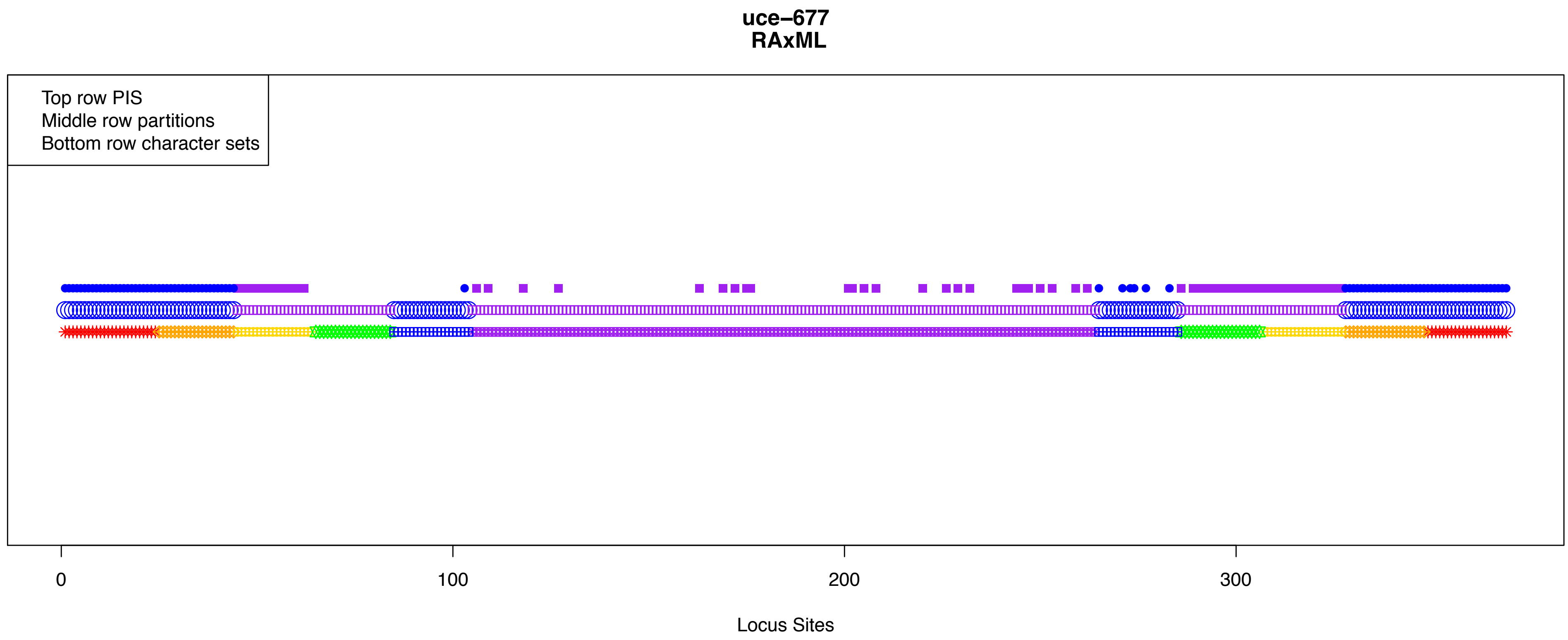
uce–677
RAxML
Top row PIS
Middle row partitions
Bottom row character sets
0
100
200
300
Locus Sites

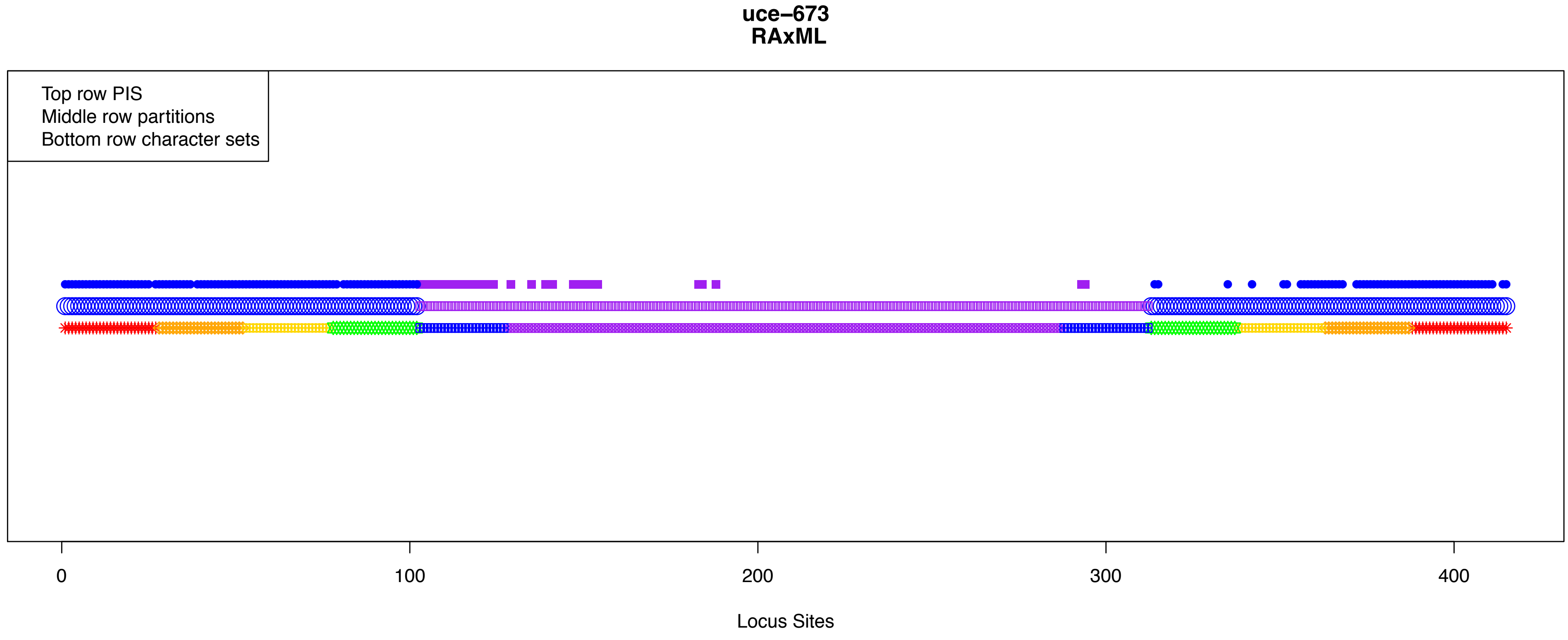
uce–673
RAxML
Top row PIS
Middle row partitions
Bottom row character sets
0
100
200
300
400
Locus Sites

uce–672
RAxML

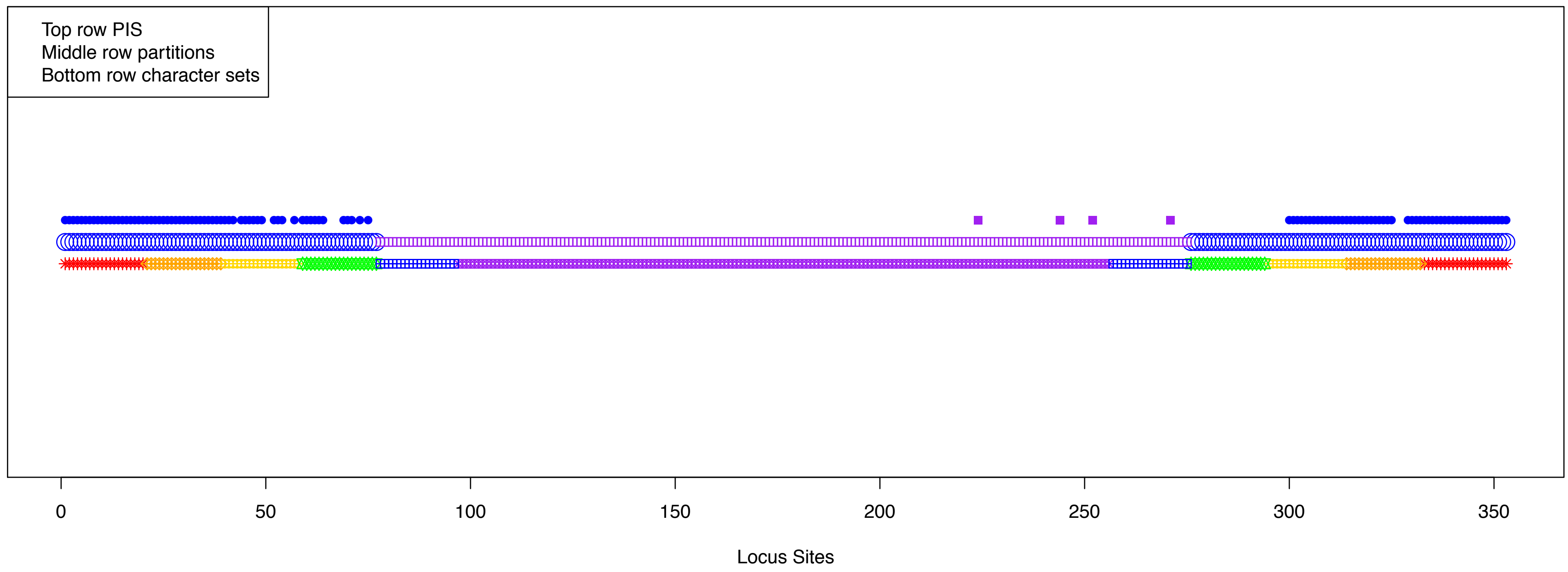

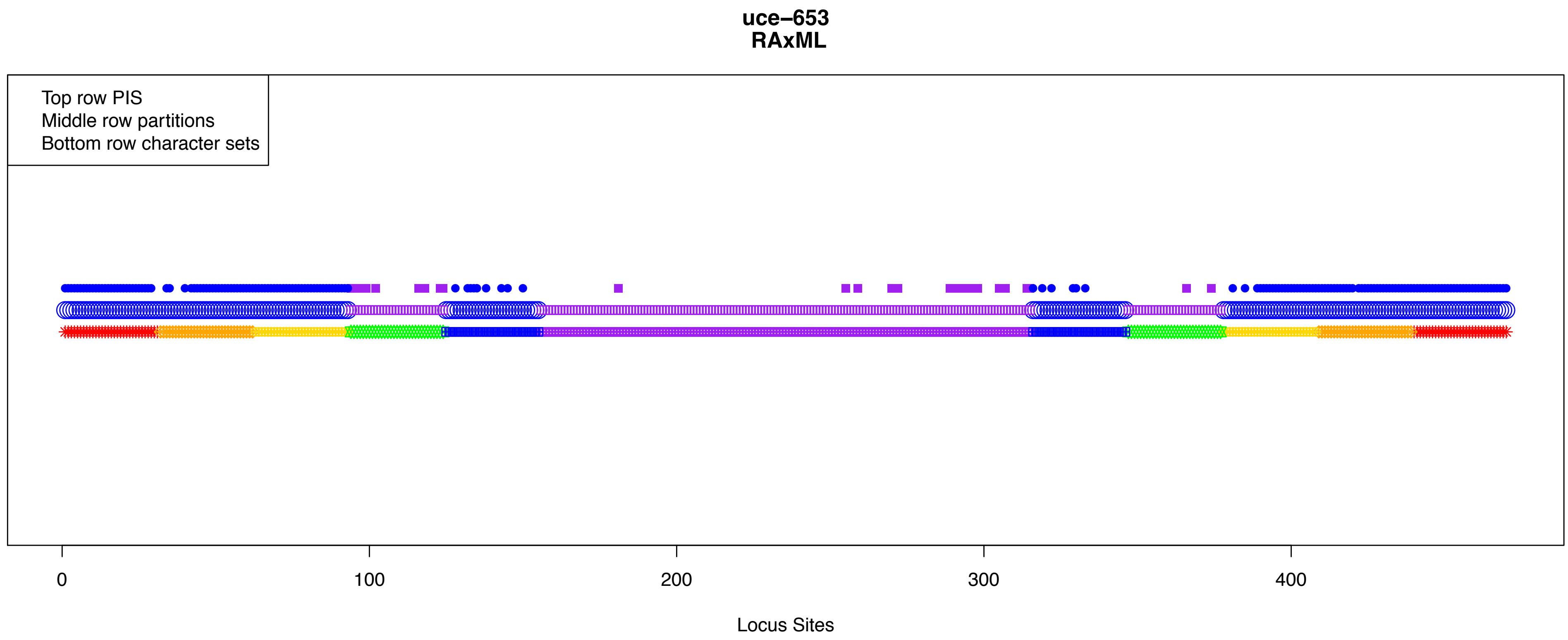
uce–653
RAxML
Top row PIS
Middle row partitions
Bottom row character sets
0
100
200
300
400
Locus Sites

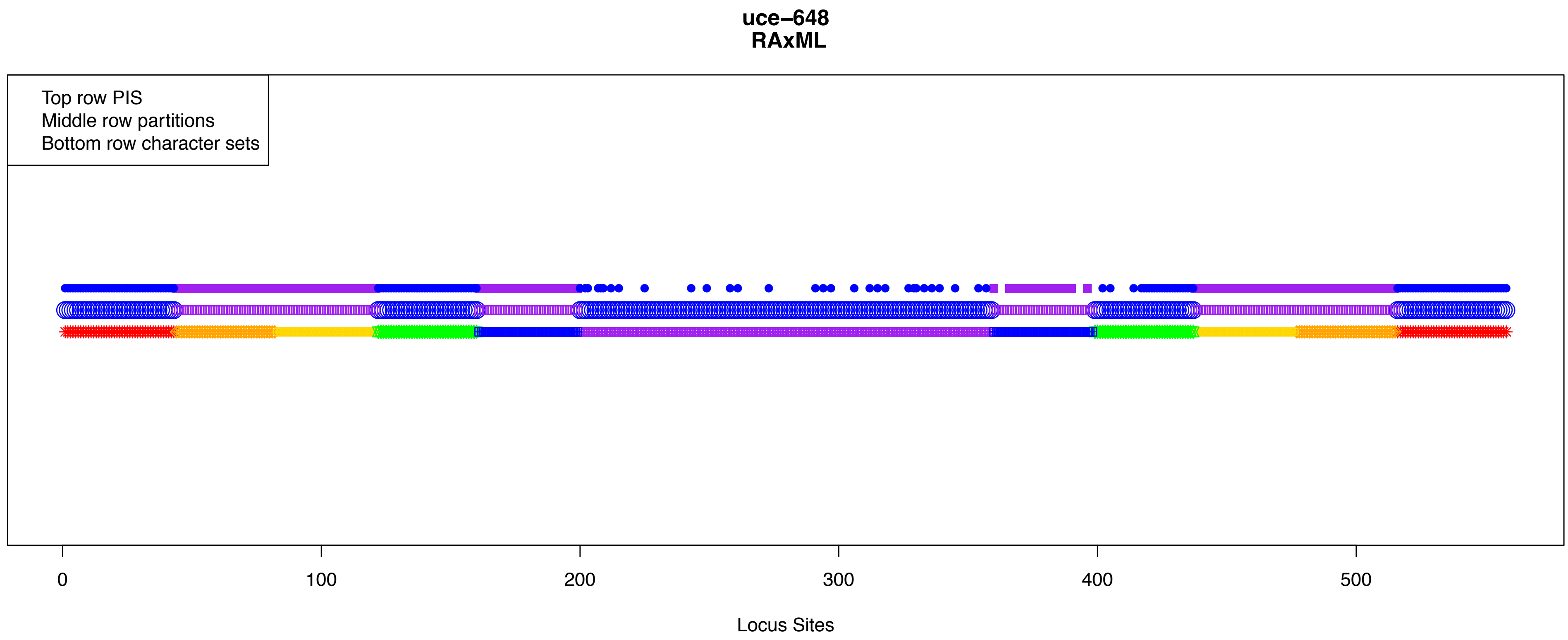
uce–648
RAxML
Top row PIS
Middle row partitions
Bottom row character sets
0
100
200
300
400
500
Locus Sites

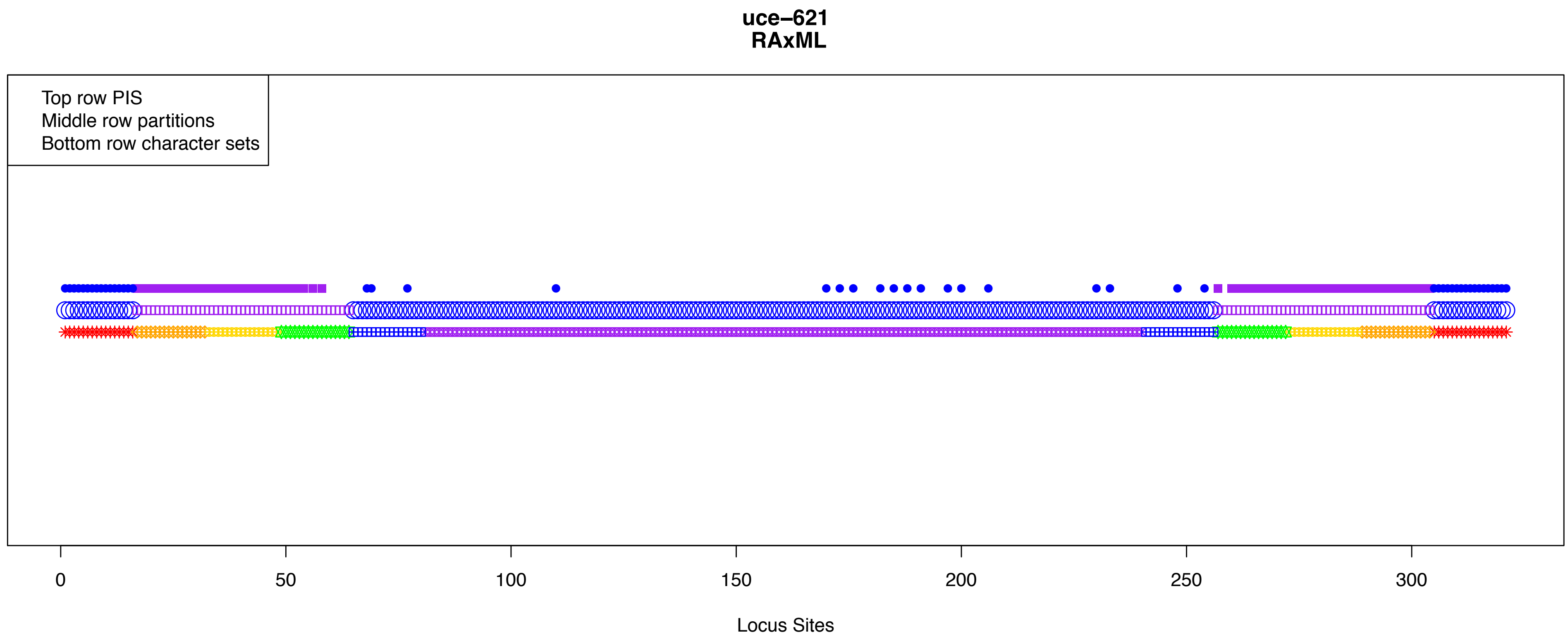
uce–621
RAxML
Top row PIS
Middle row partitions
Bottom row character sets
0
50
100
150
200
250
300
Locus Sites

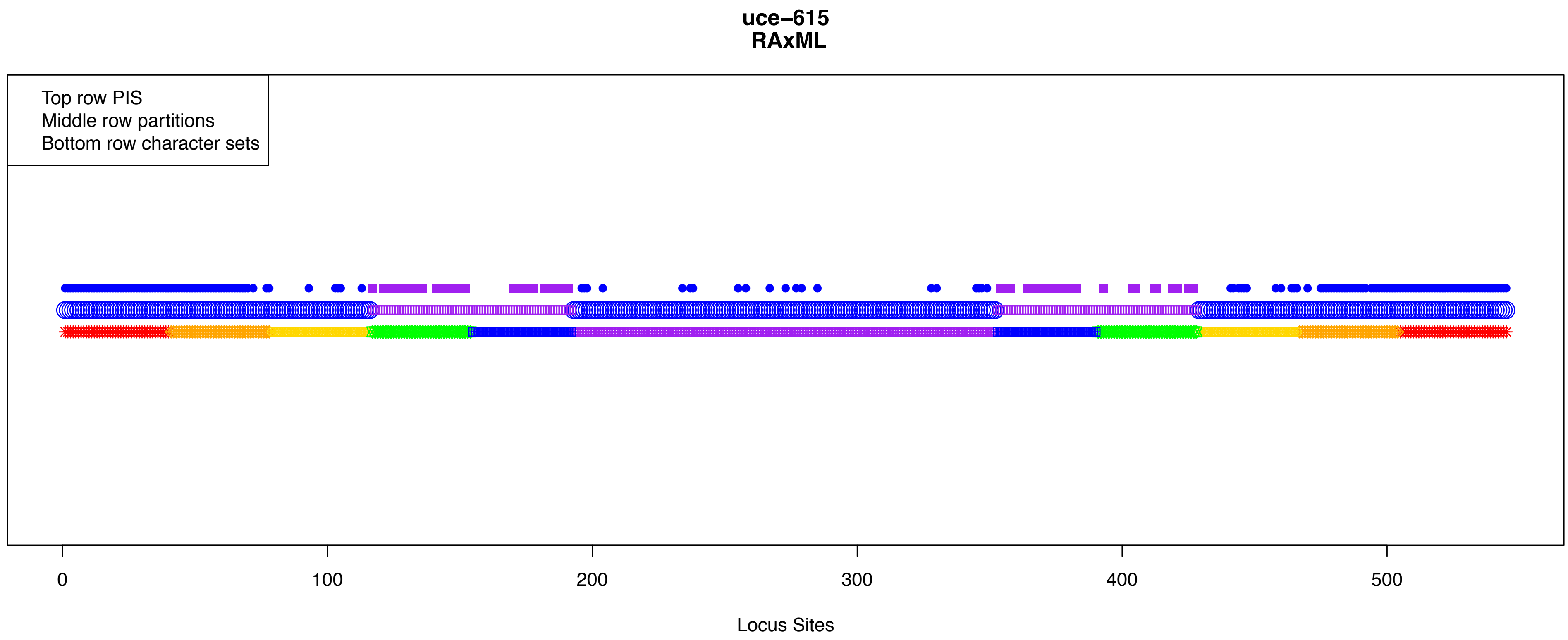
uce–615
RAxML
Top row PIS
Middle row partitions
Bottom row character sets
0
100
200
300
400
500
Locus Sites

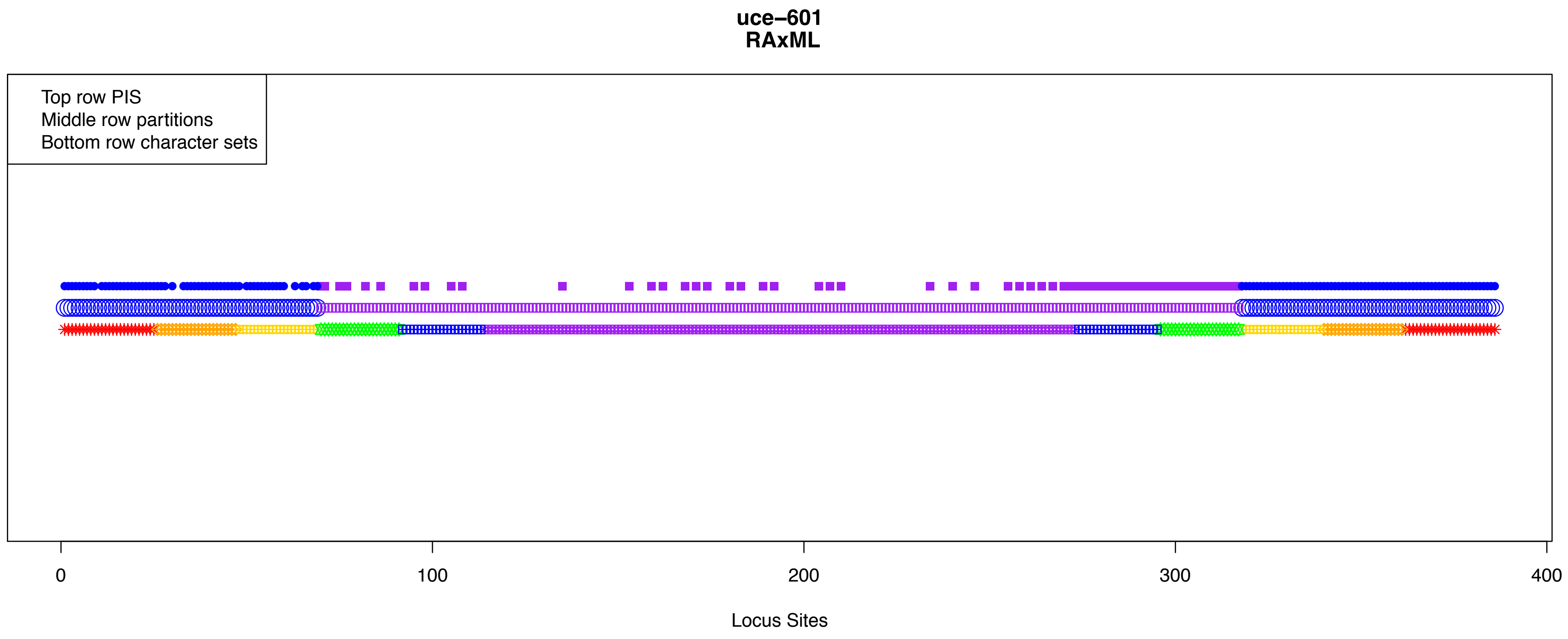
uce–601
RAxML
Top row PIS
Middle row partitions
Bottom row character sets
0
100
200
300
400
Locus Sites

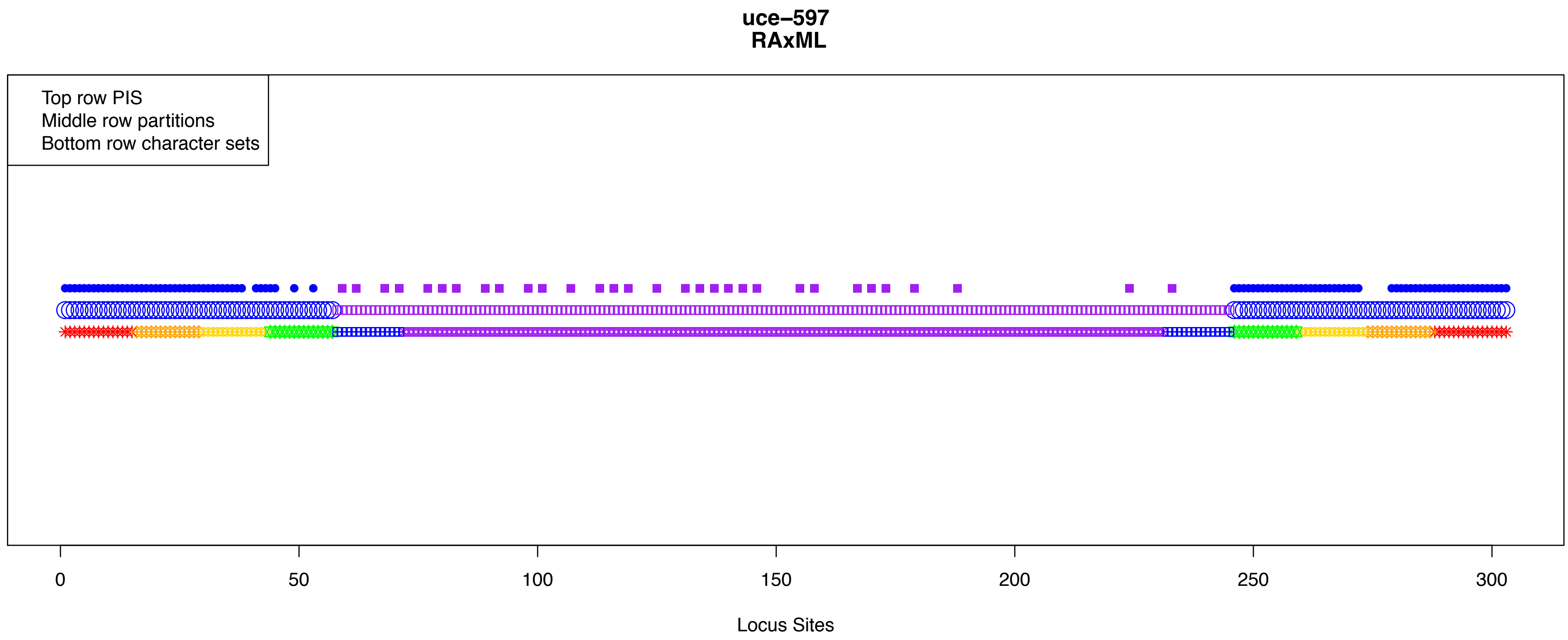
uce–597
RAxML
Top row PIS
Middle row partitions
Bottom row character sets
0
50
100
150
200
250
300
Locus Sites

uce–590
RAxML

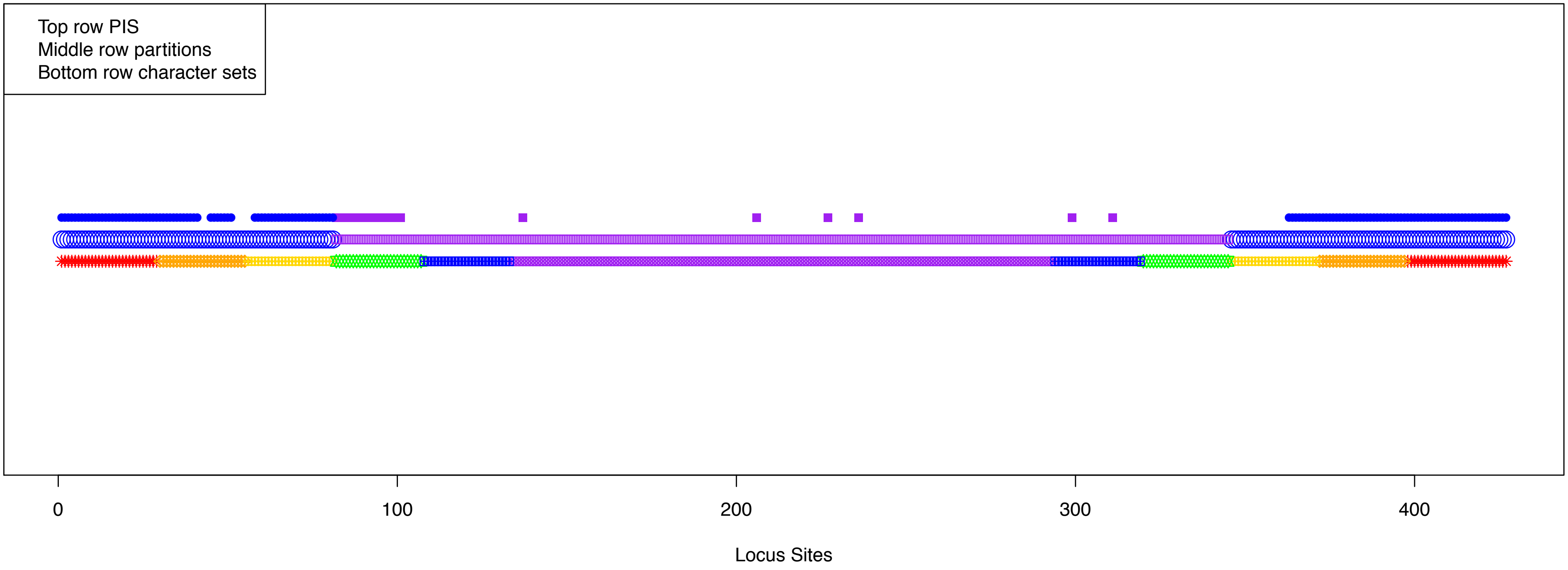

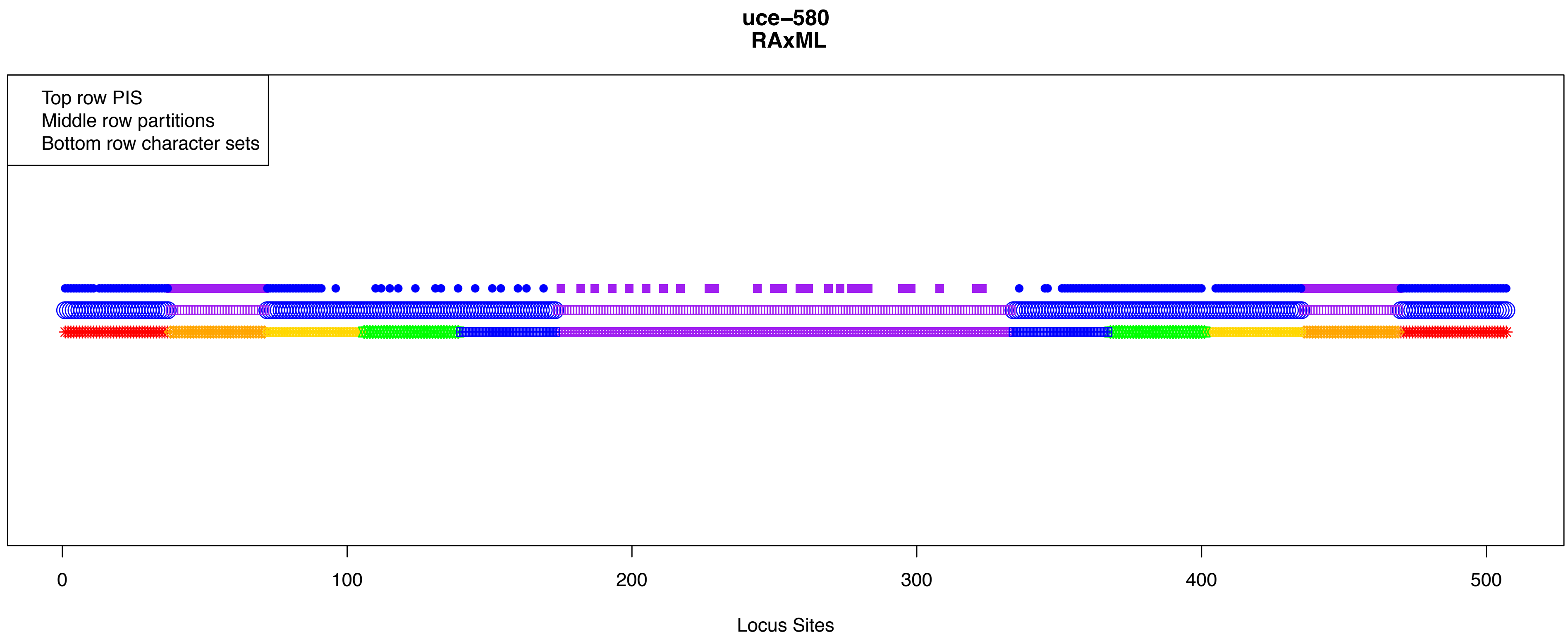
uce–580
RAxML
Top row PIS
Middle row partitions
Bottom row character sets
0
100
200
300
400
500
Locus Sites

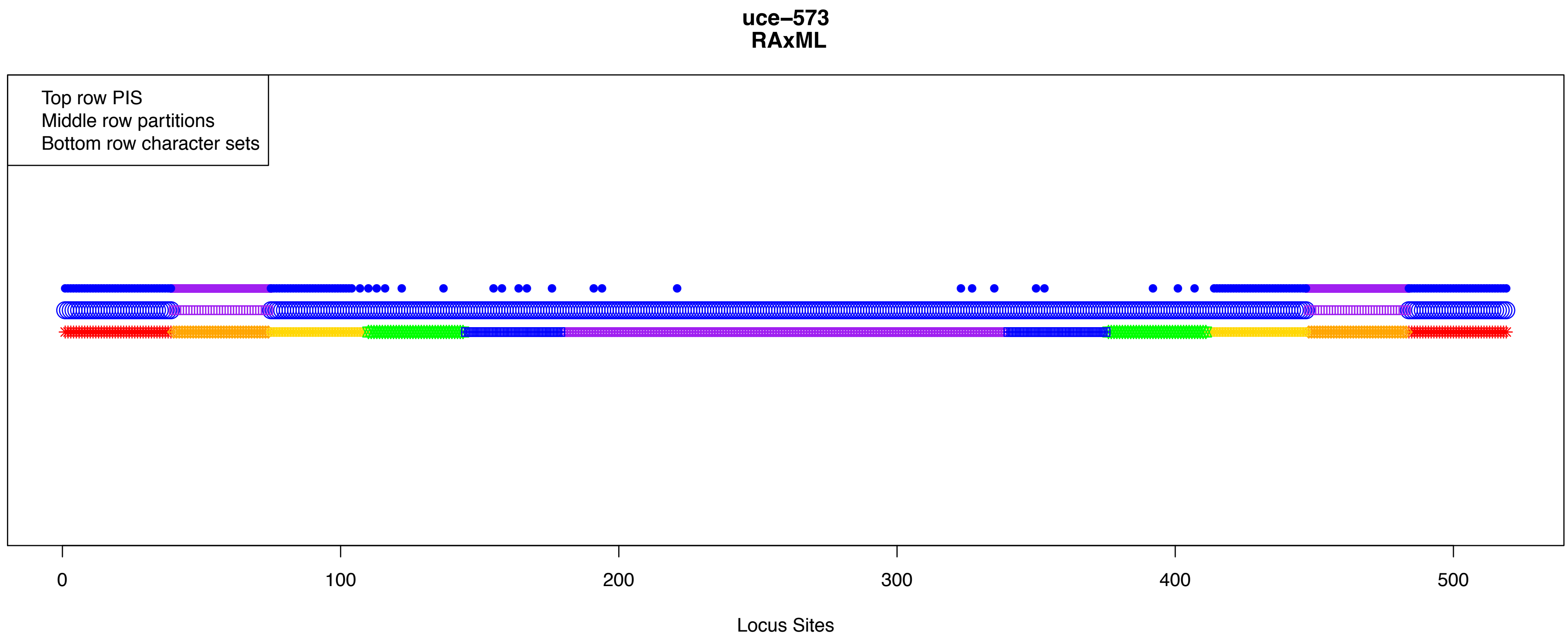
uce–573
RAxML
Top row PIS
Middle row partitions
Bottom row character sets
0
100
200
300
400
500
Locus Sites

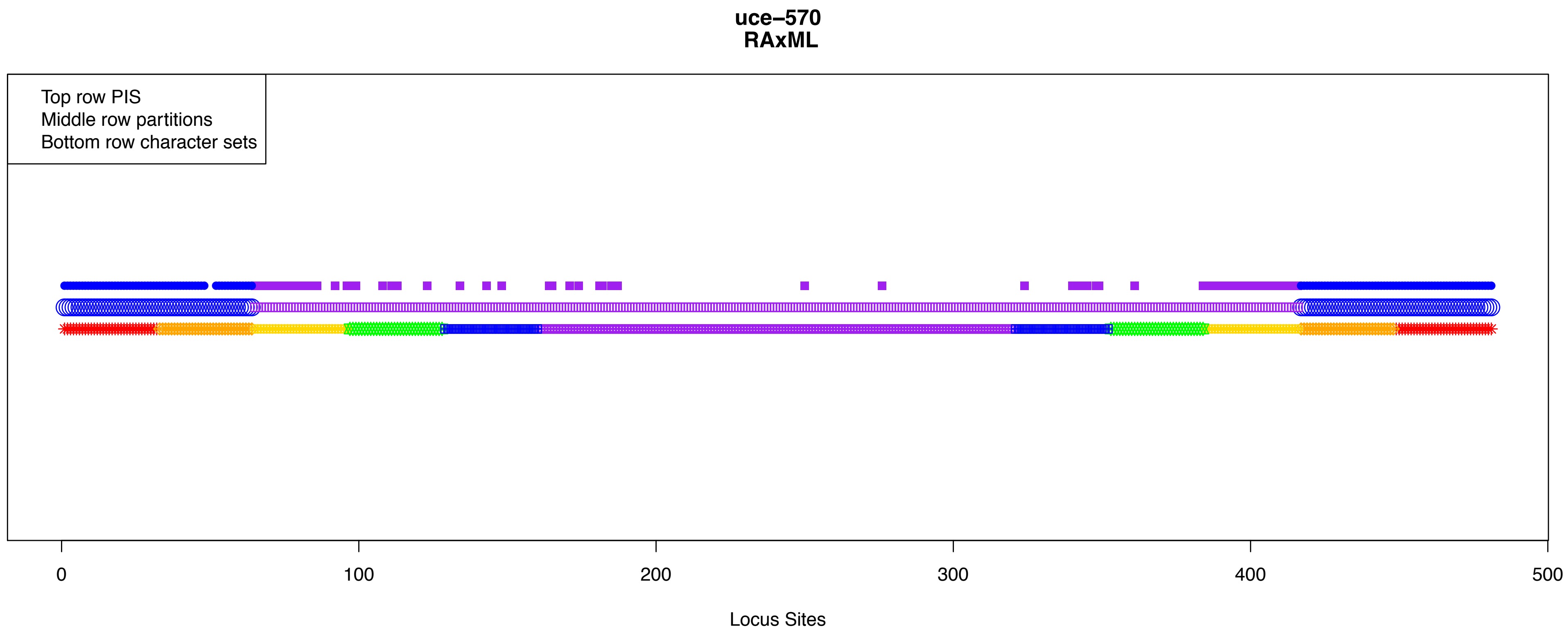
uce–570
RAxML
Top row PIS
Middle row partitions
Bottom row character sets
0
100
200
300
400
500
Locus Sites

uce–569
RAxML

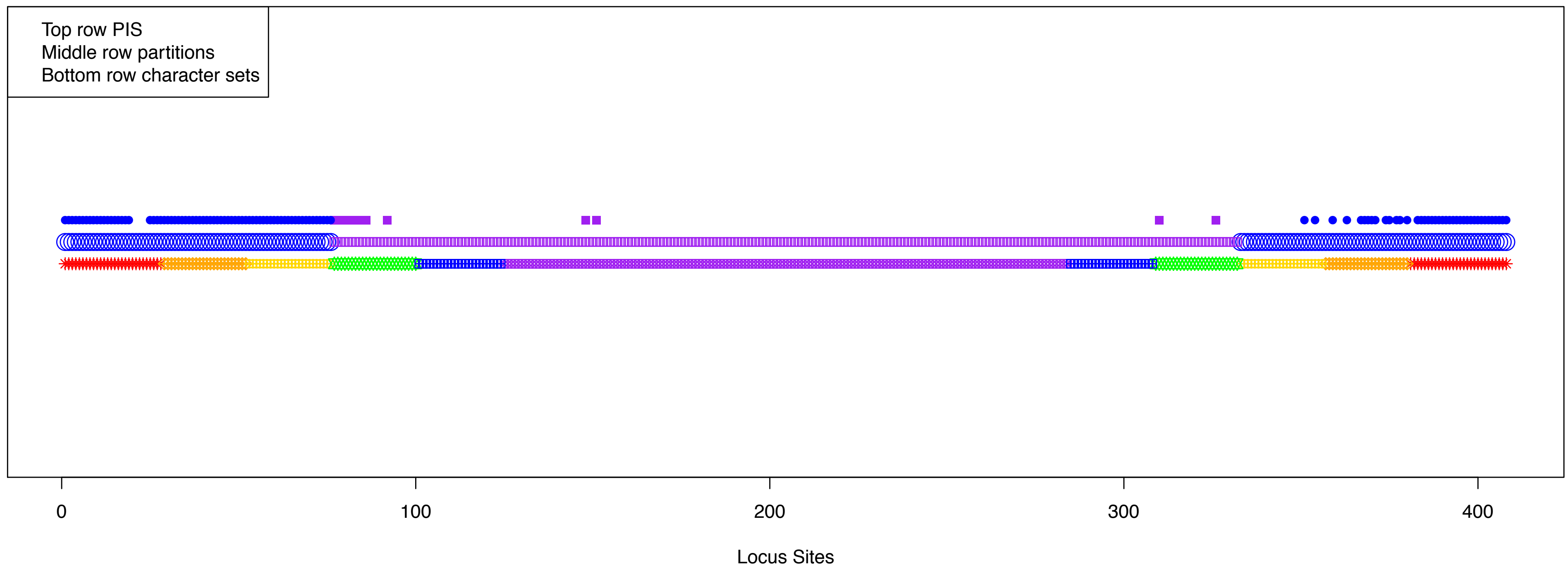

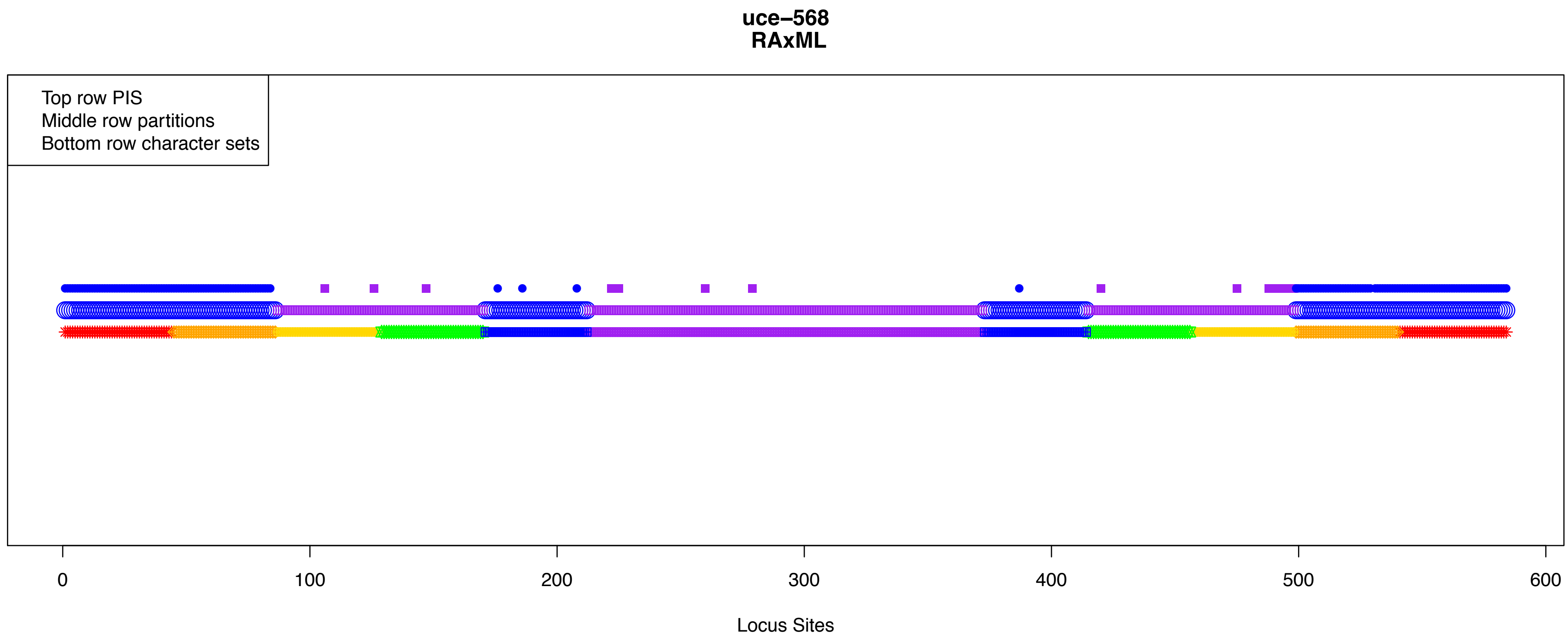
uce–568
RAxML
Top row PIS
Middle row partitions
Bottom row character sets
0
100
200
300
400
500
600
Locus Sites

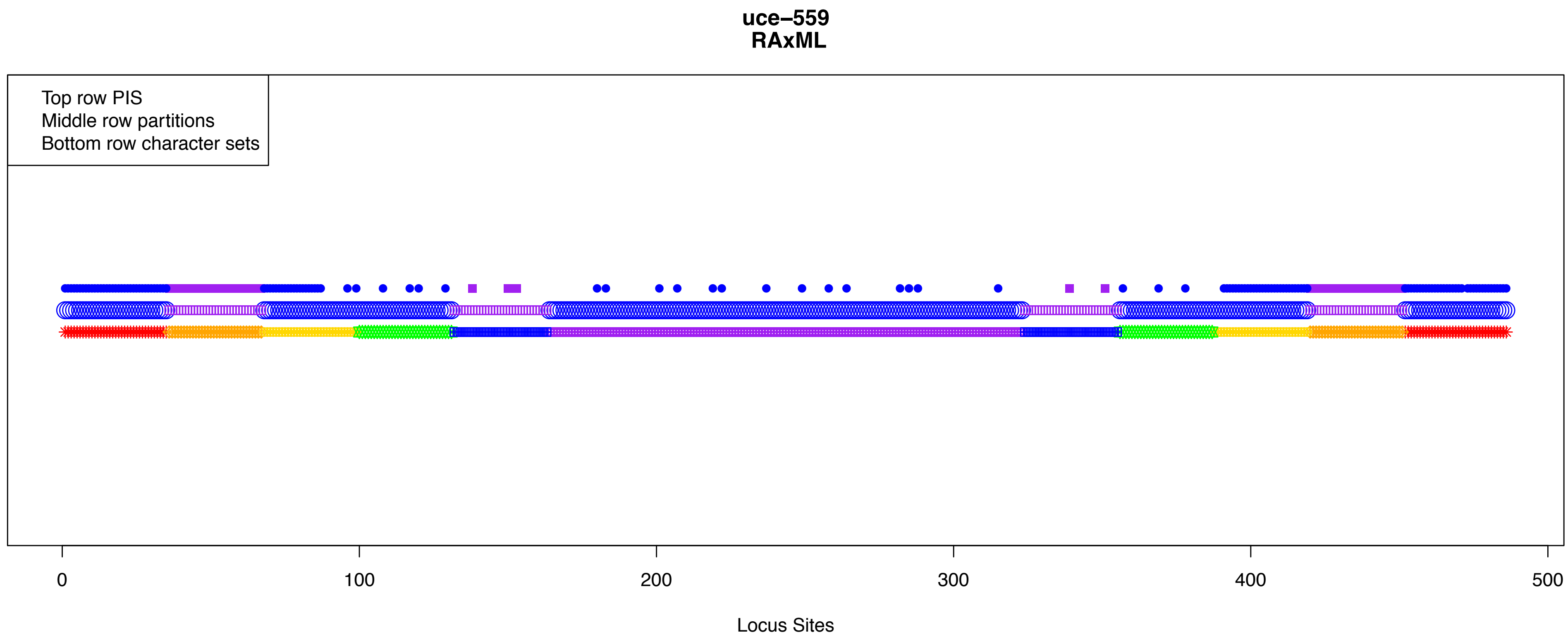

uce–559
RAxML
Top row PIS
Middle row partitions
Bottom row character sets
0
100
200
300
400
500
Locus Sites

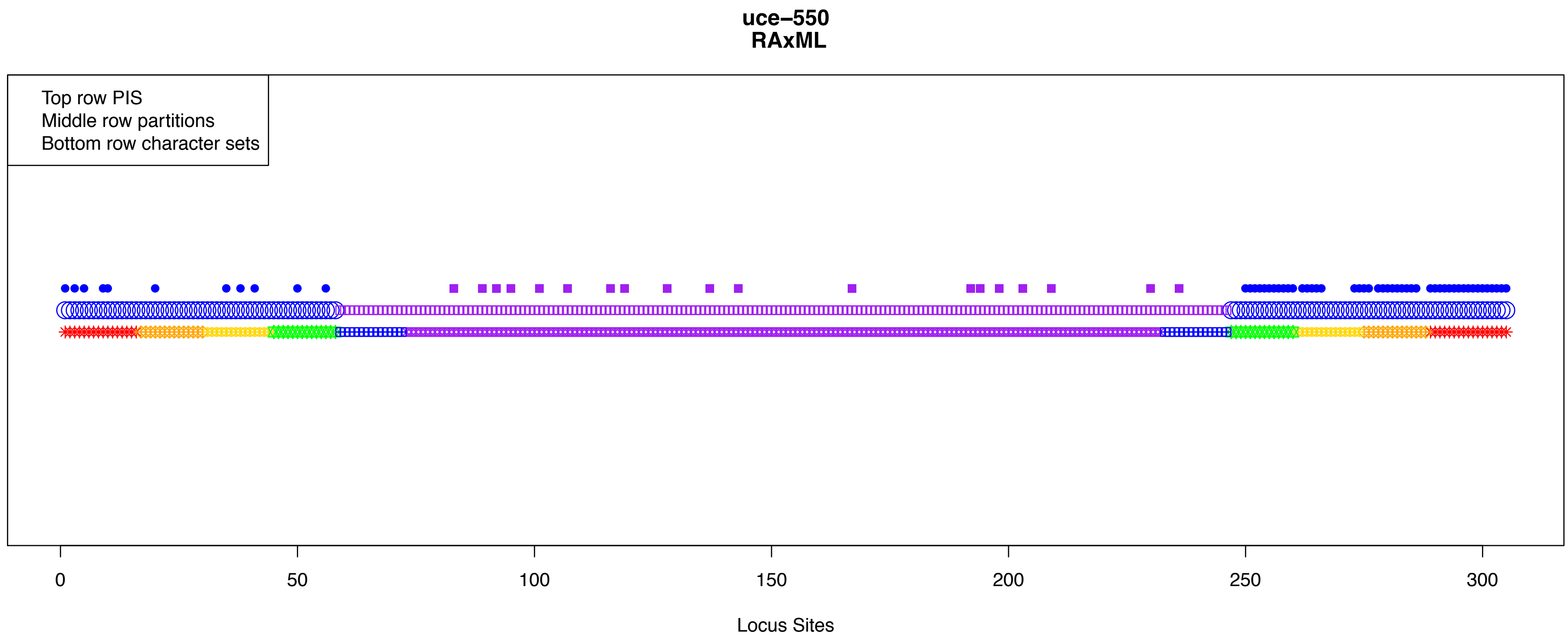
uce–550
RAxML
Top row PIS
Middle row partitions
Bottom row character sets
0
50
100
150
200
250
300
Locus Sites

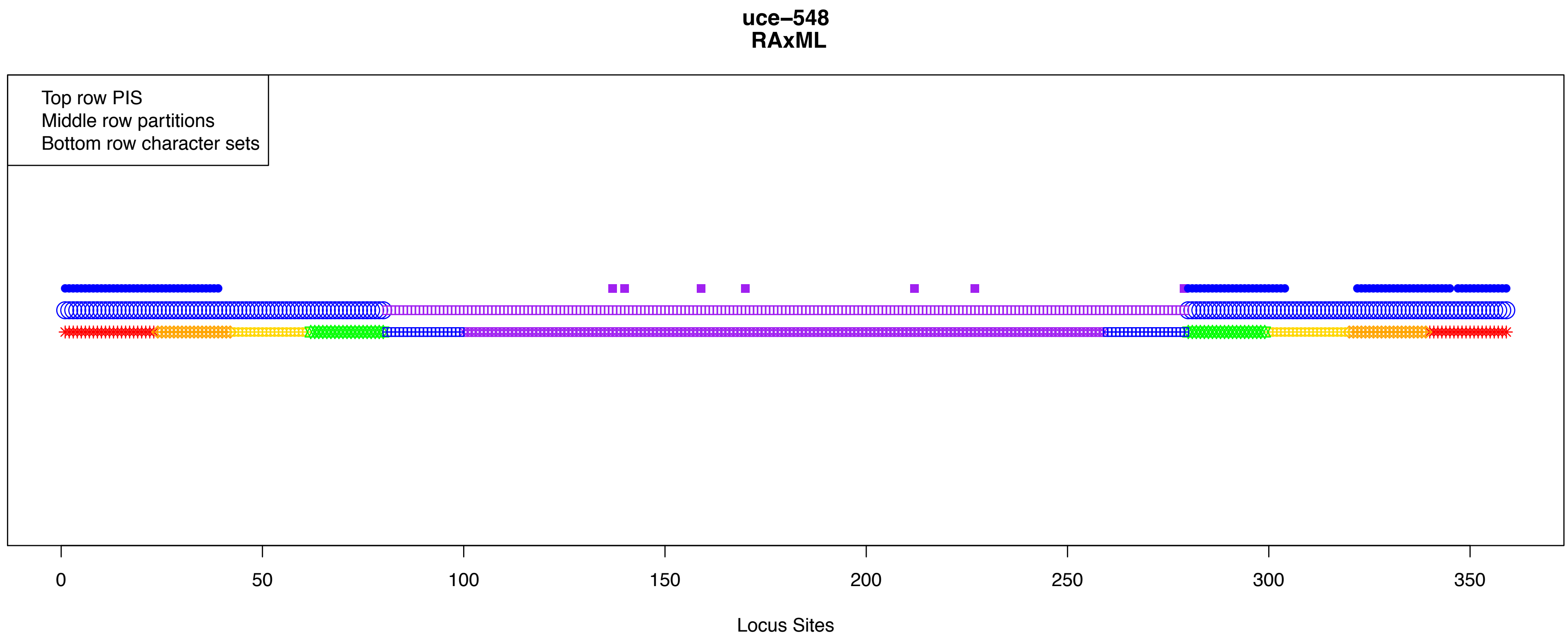
uce–548
RAxML
Top row PIS
Middle row partitions
Bottom row character sets
0
50
100
150
200
250
300
350
Locus Sites

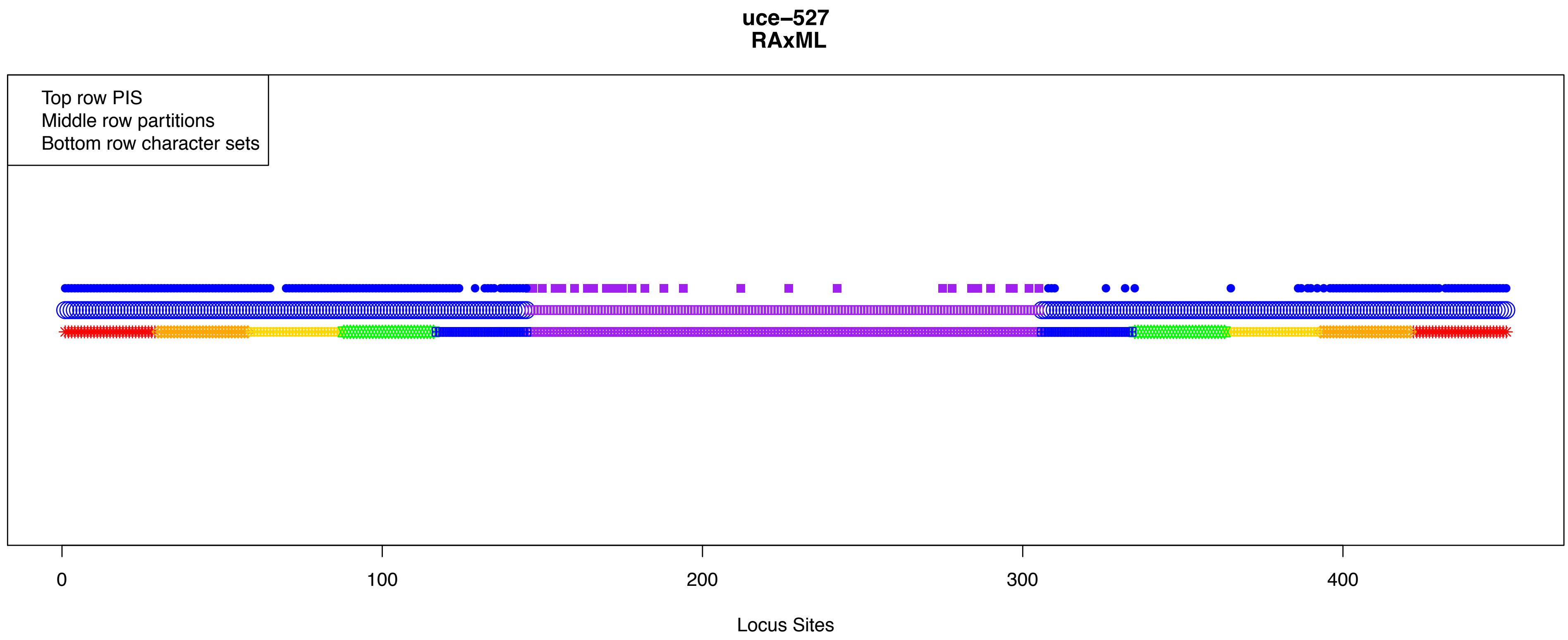

uce–527
RAxML
Top row PIS
Middle row partitions
Bottom row character sets
0
100
200
300
400
Locus Sites

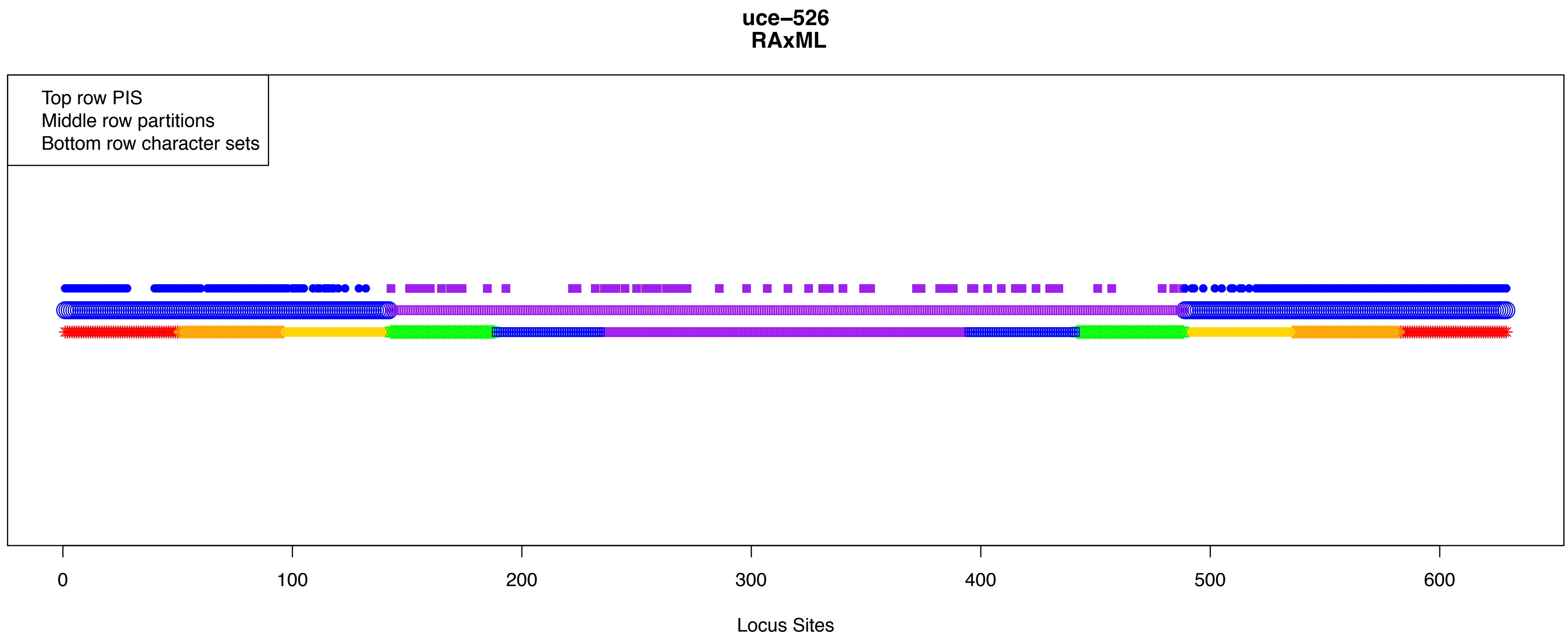

uce–526
RAxML
Top row PIS
Middle row partitions
Bottom row character sets
0
100
200
300
400
500
600
Locus Sites

uce–521
RAxML

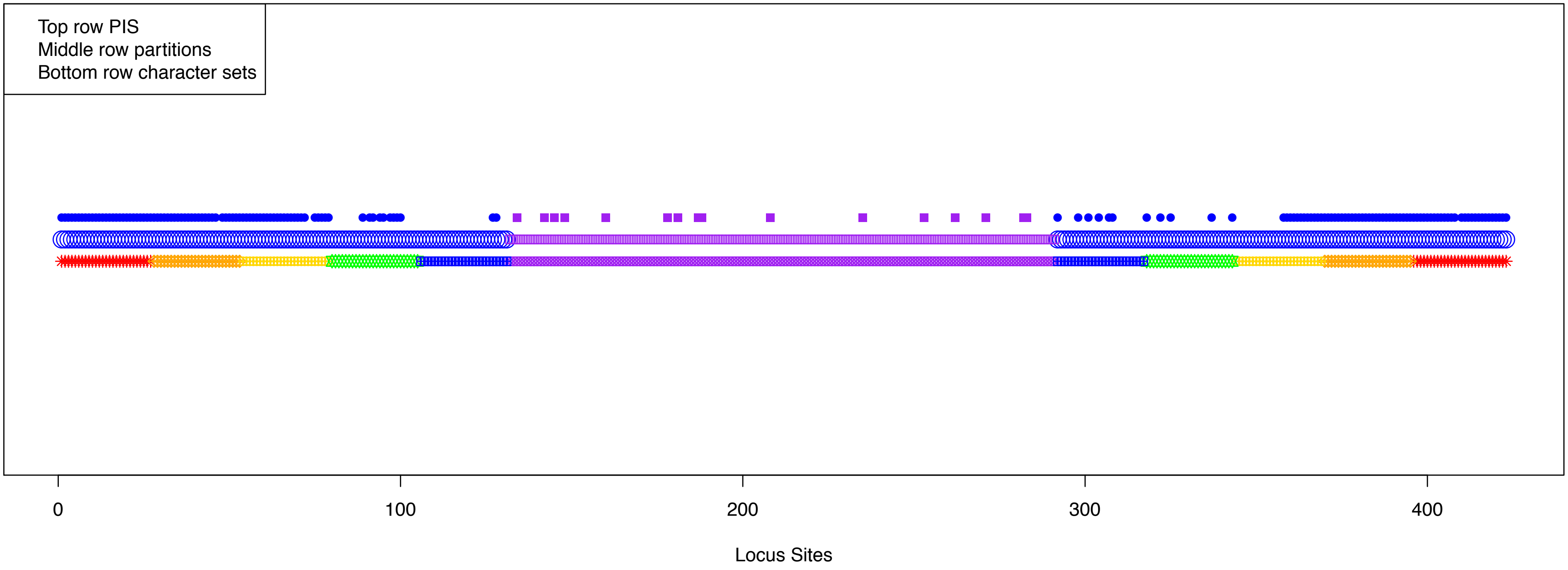

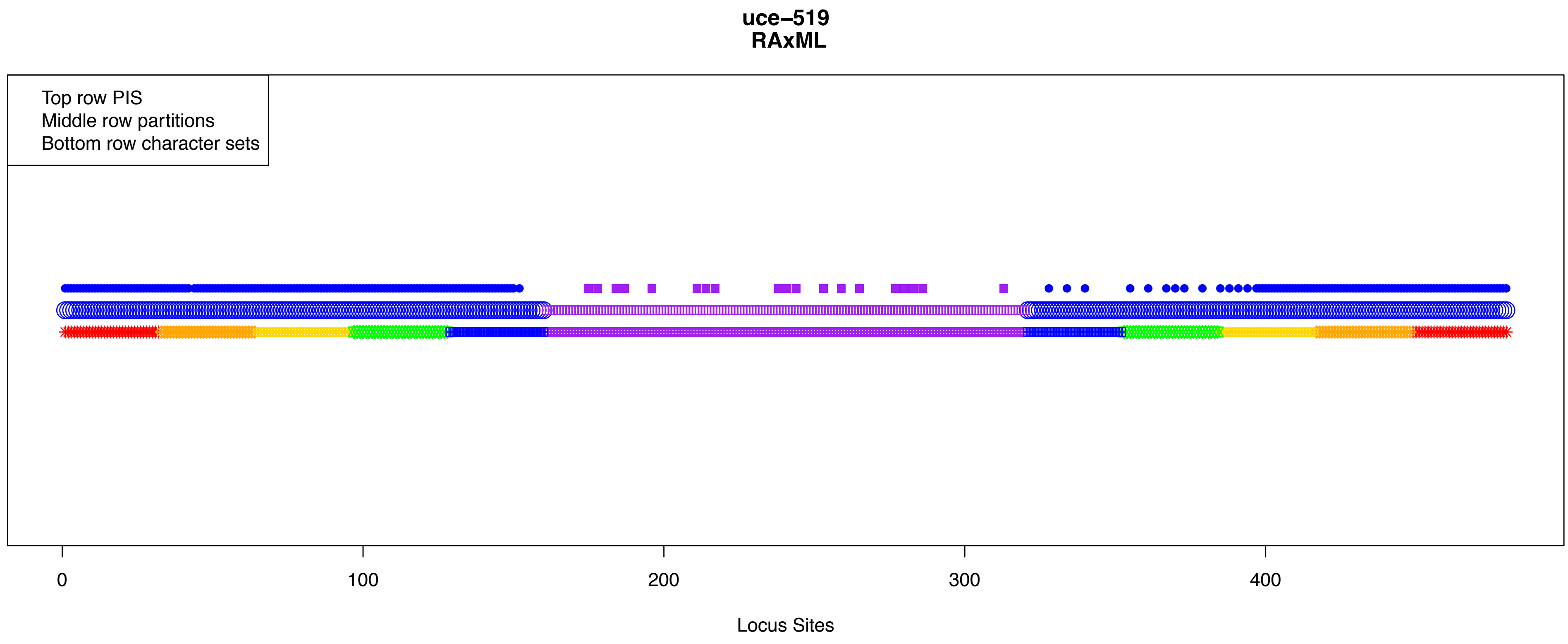
uce–519
RAxML
Top row PIS
Middle row partitions
Bottom row character sets
0
100
200
300
400
Locus Sites

uce–51
RAxML

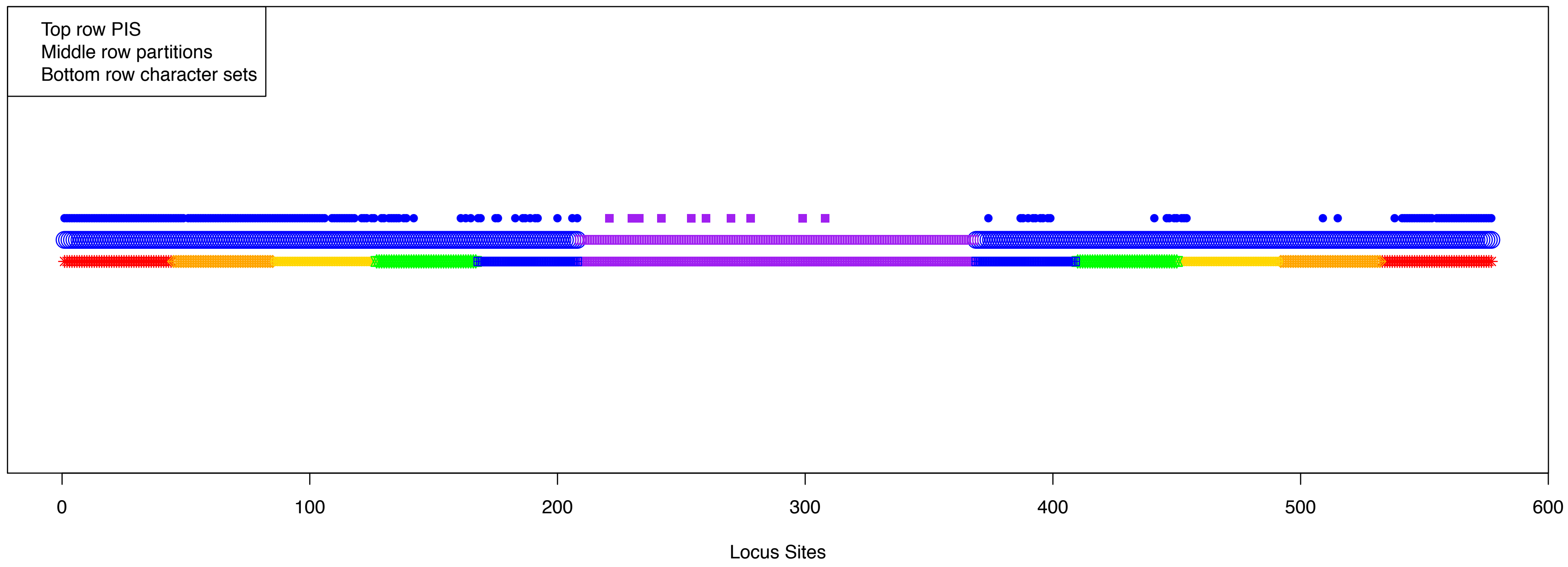

Top row PIS
Middle row partitions
Bottom row character sets
0
100
200
300
400
500
600
Locus Sites

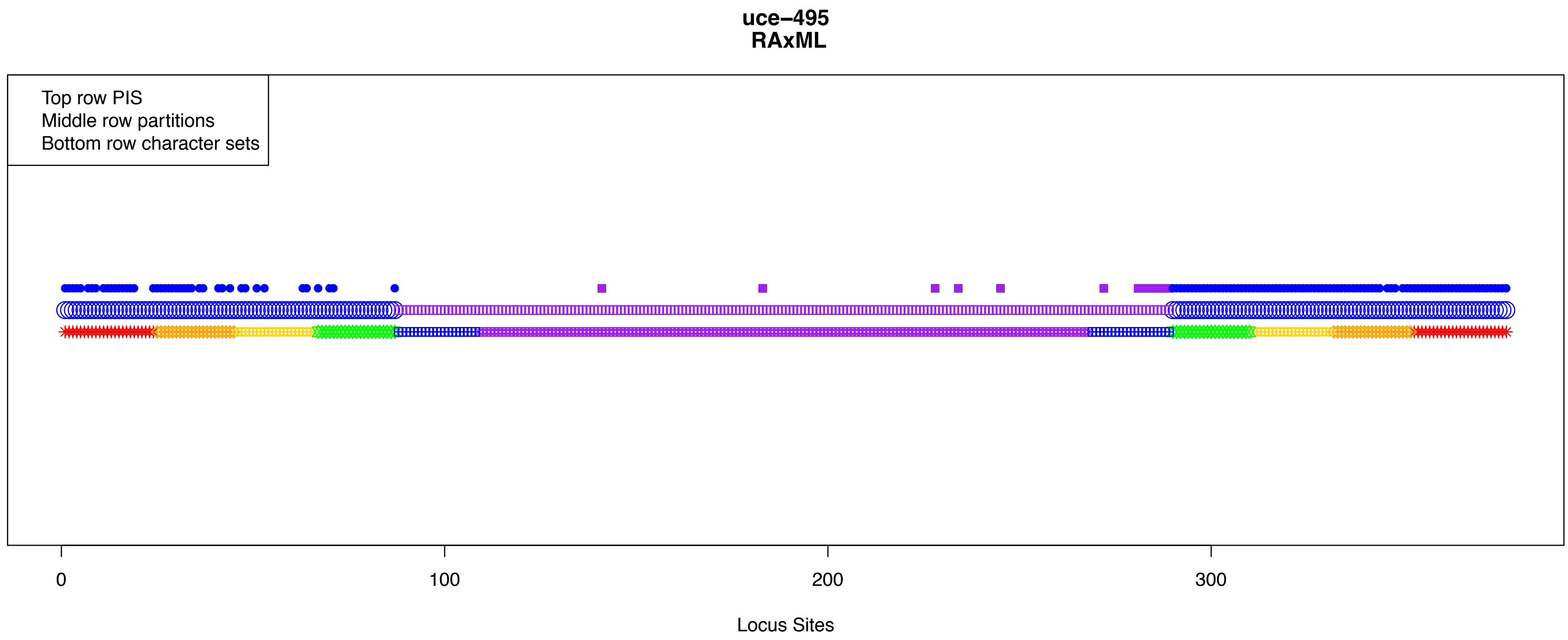
uce–495
RAxML
Top row PIS
Middle row partitions
Bottom row character sets
0
100
200
300
Locus Sites

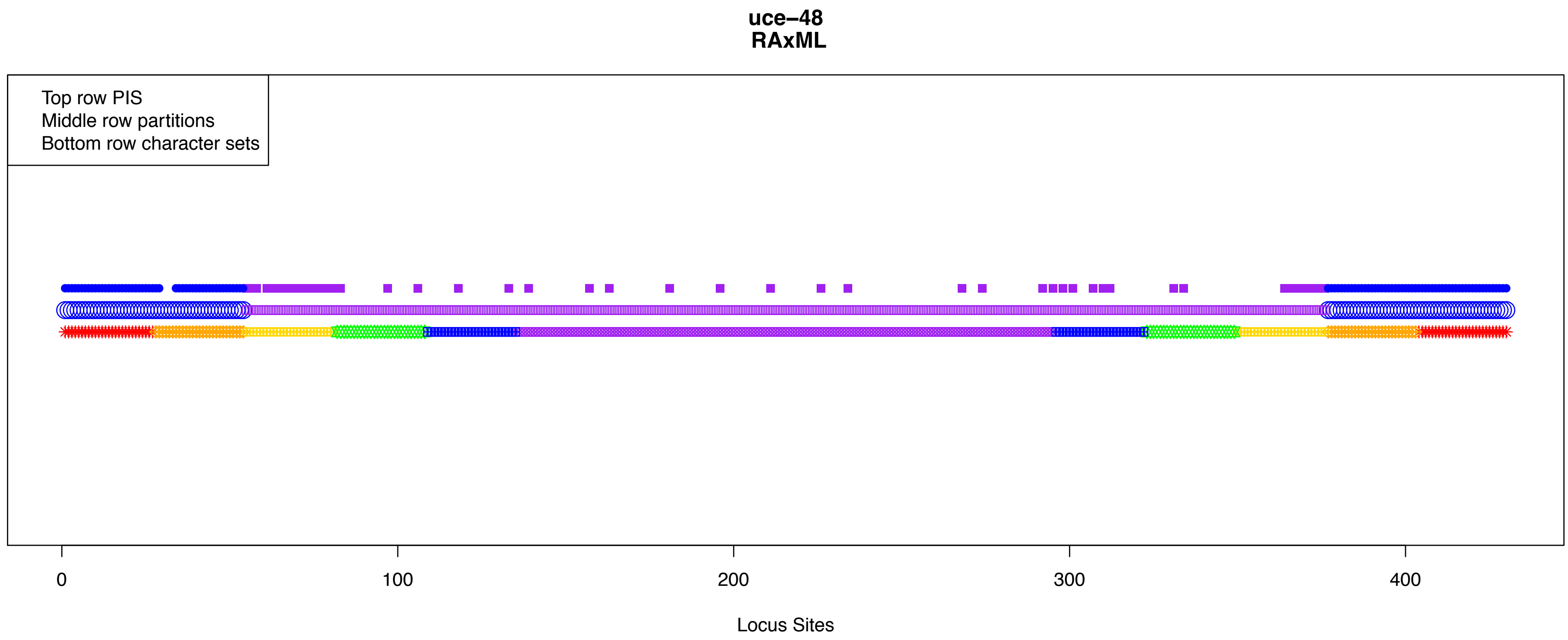
uce–48
RAxML
Top row PIS
Middle row partitions
Bottom row character sets
0
100
200
300
400
Locus Sites

uce–475
RAxML

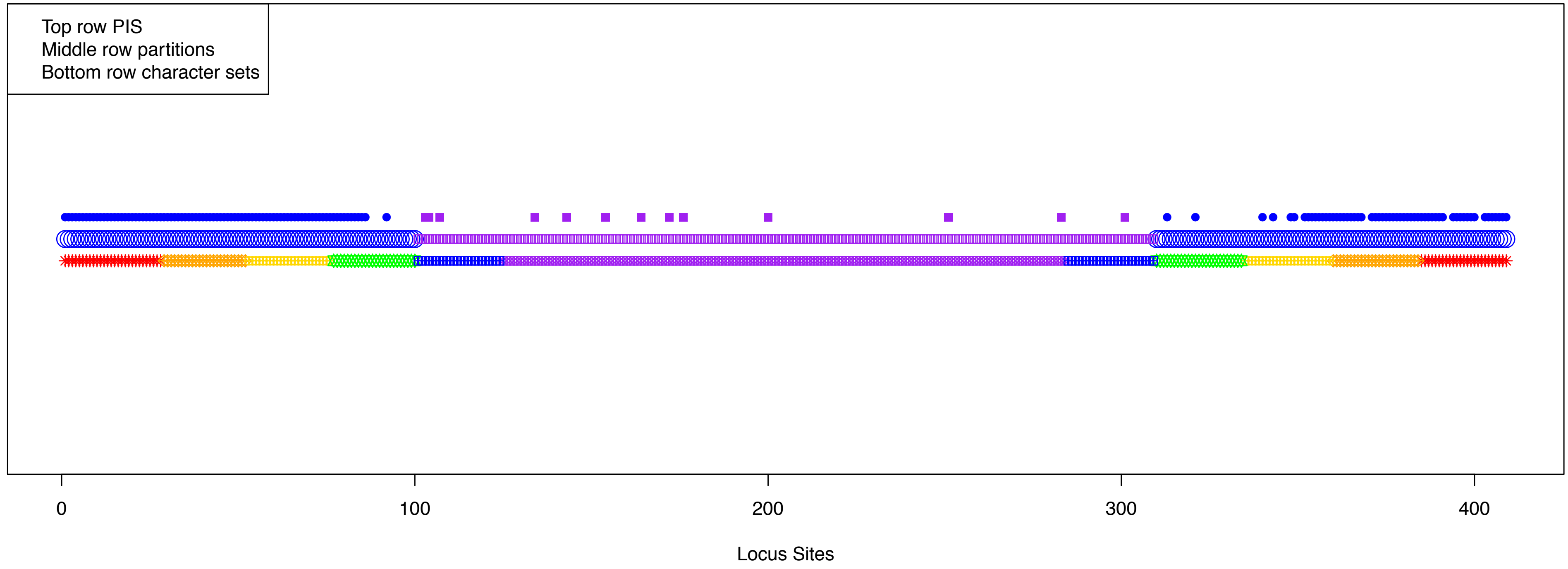

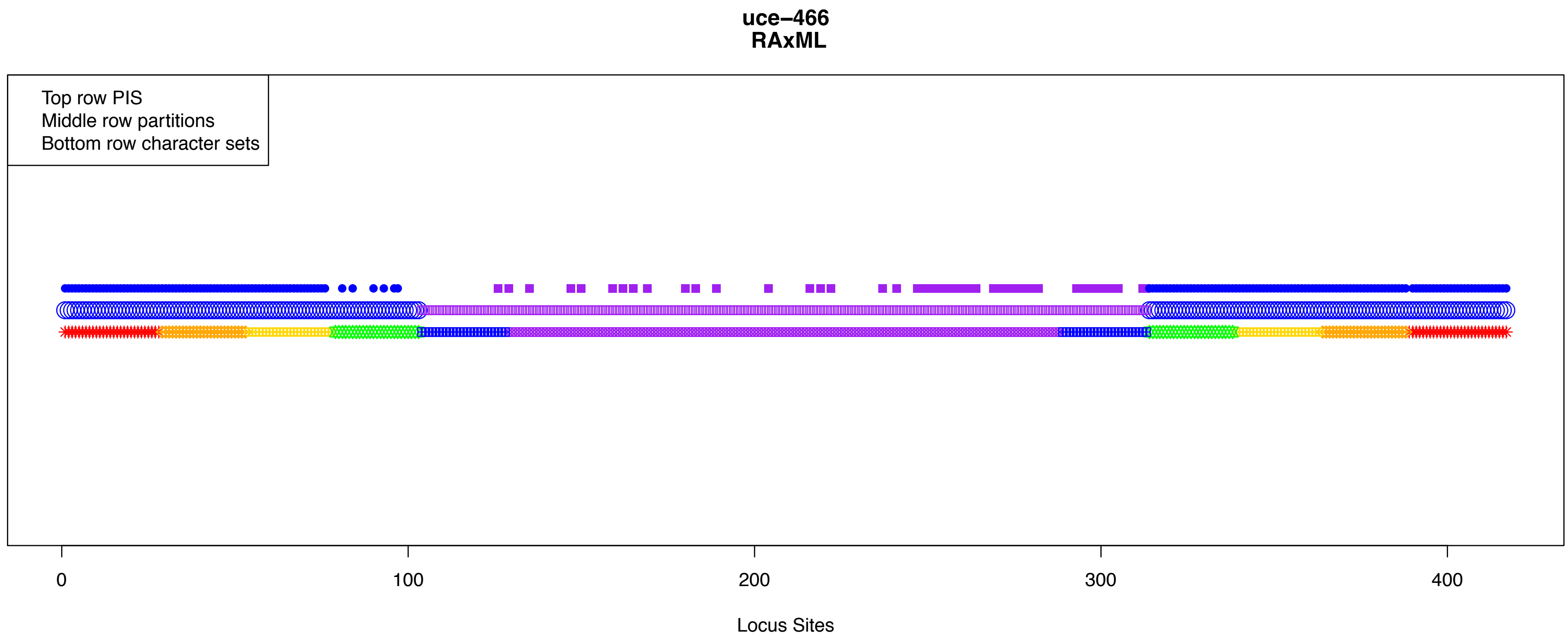
uce–466
RAxML
Top row PIS
Middle row partitions
Bottom row character sets
0
100
200
300
400
Locus Sites

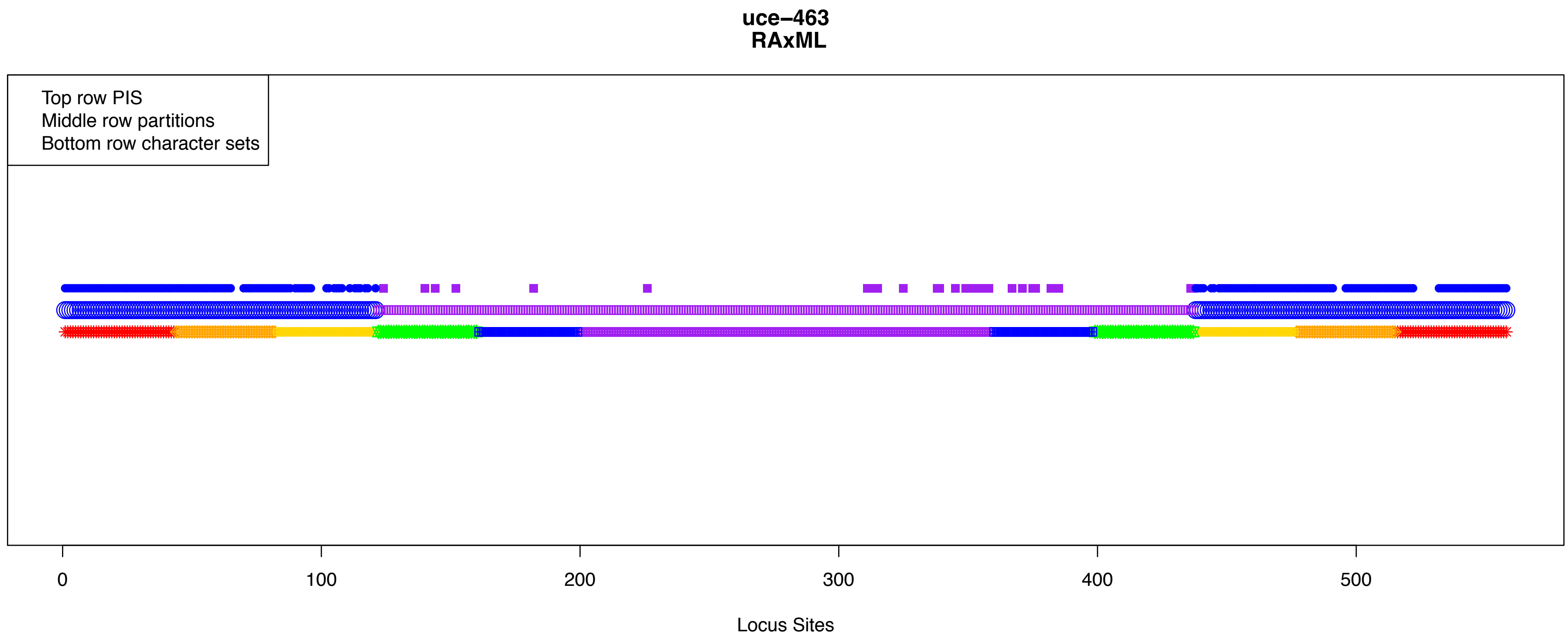
uce–463
RAxML
Top row PIS
Middle row partitions
Bottom row character sets
0
100
200
300
400
500
Locus Sites

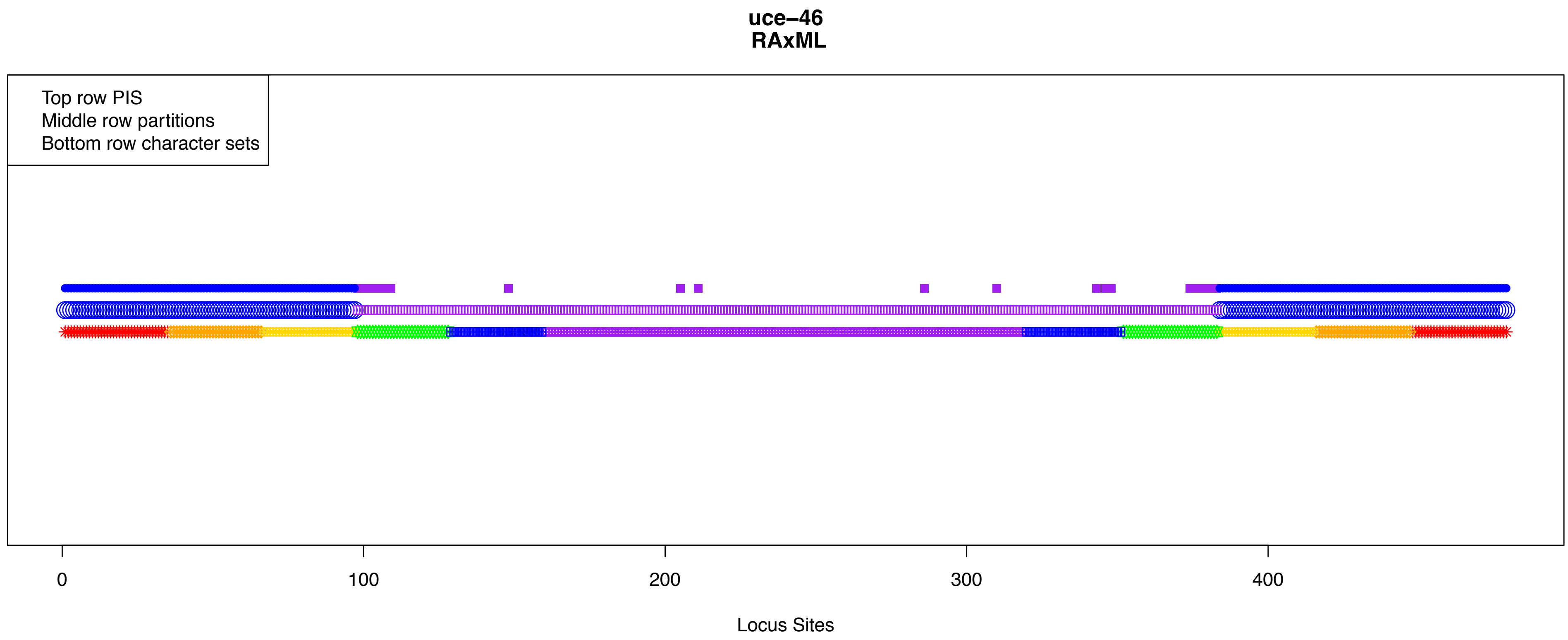

uce–46
RAxML
Top row PIS
Middle row partitions
Bottom row character sets
0
100
200
300
400
Locus Sites

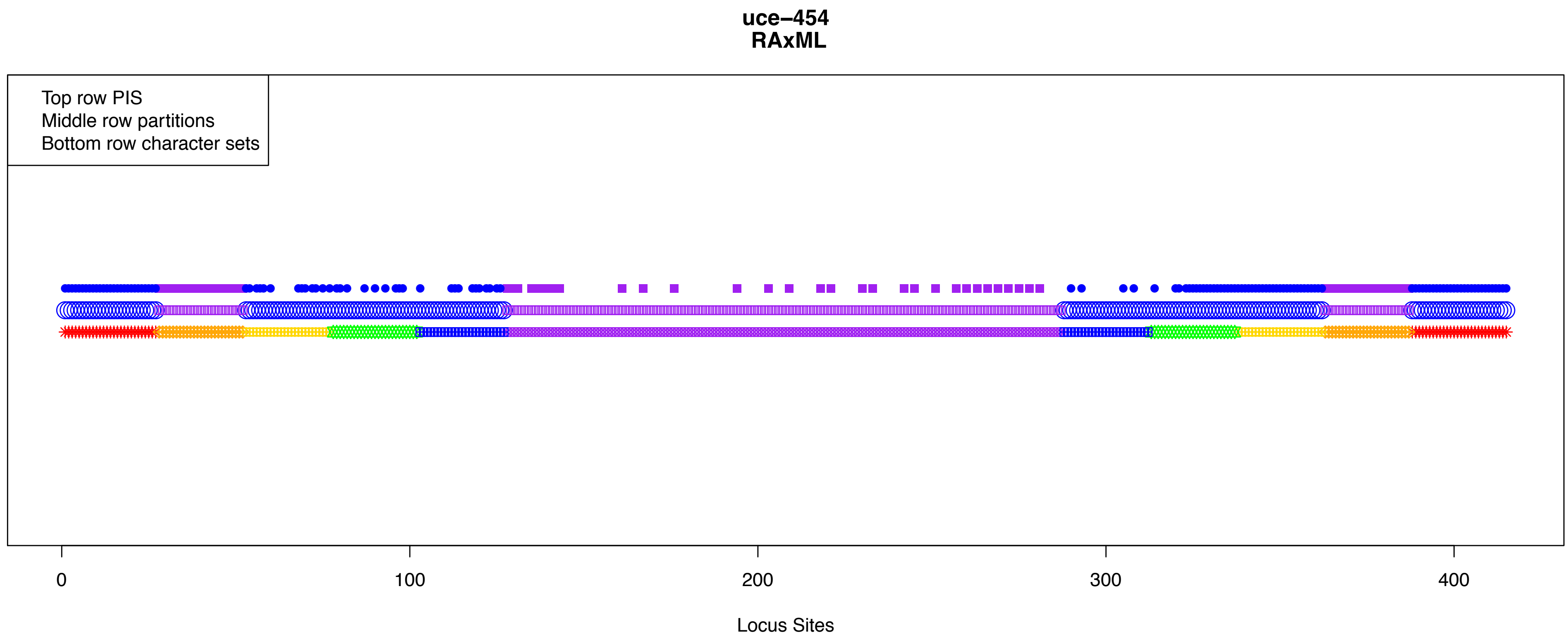

uce–454
RAxML
Top row PIS
Middle row partitions
Bottom row character sets
0
100
200
300
400
Locus Sites

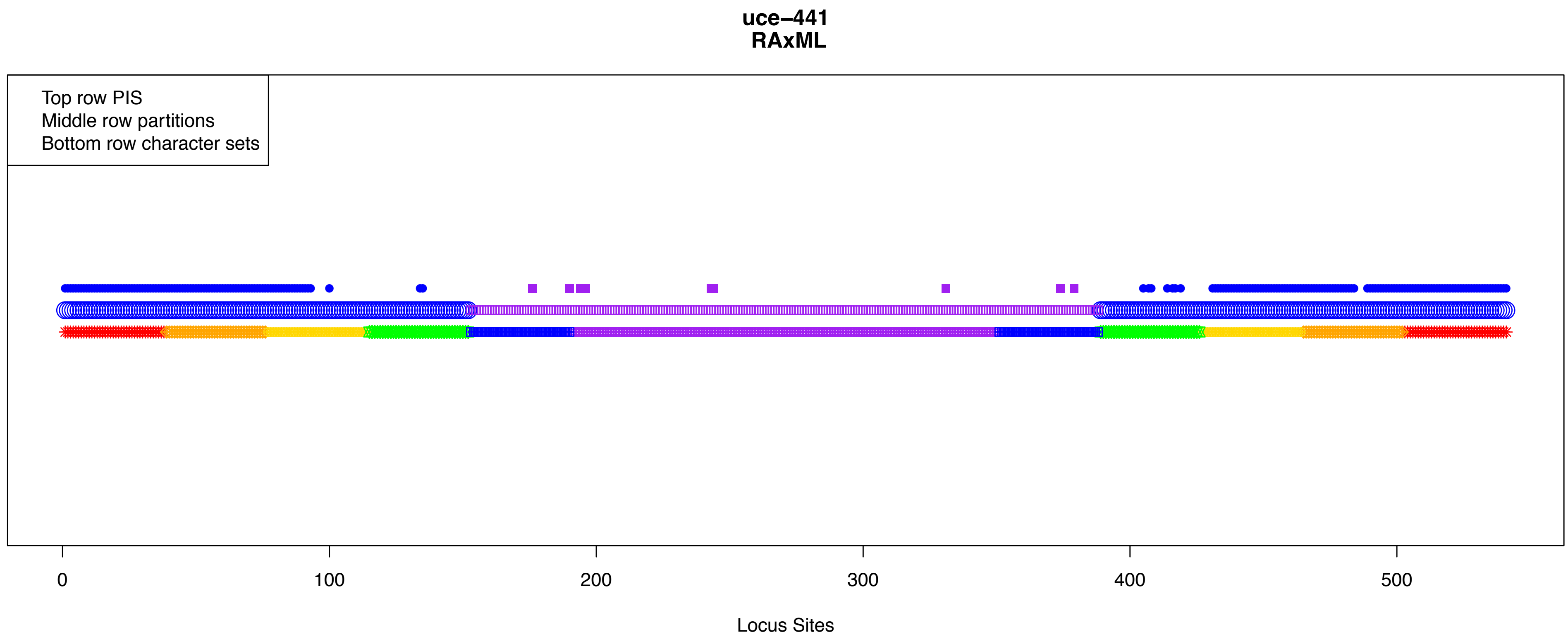

uce–441
RAxML
Top row PIS
Middle row partitions
Bottom row character sets
0
100
200
300
400
500
Locus Sites

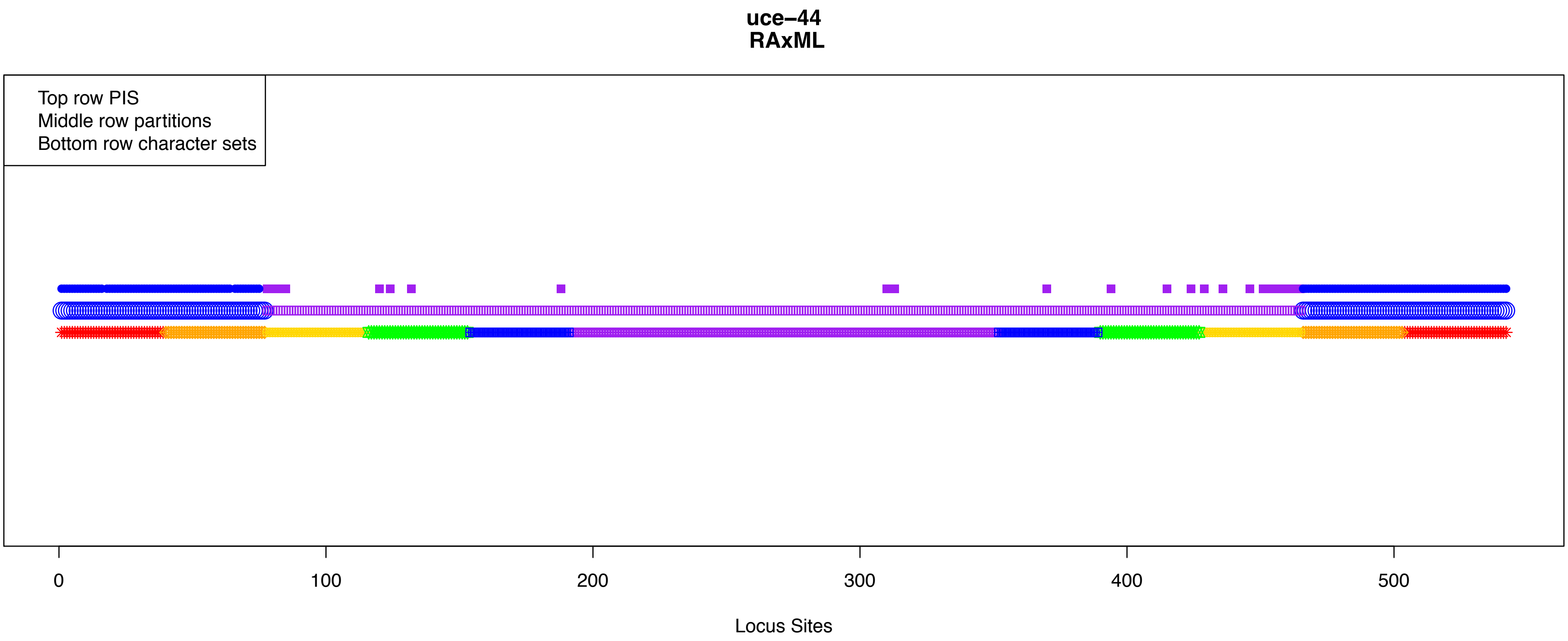
uce–44
RAxML
Top row PIS
Middle row partitions
Bottom row character sets
0
100
200
300
400
500
Locus Sites

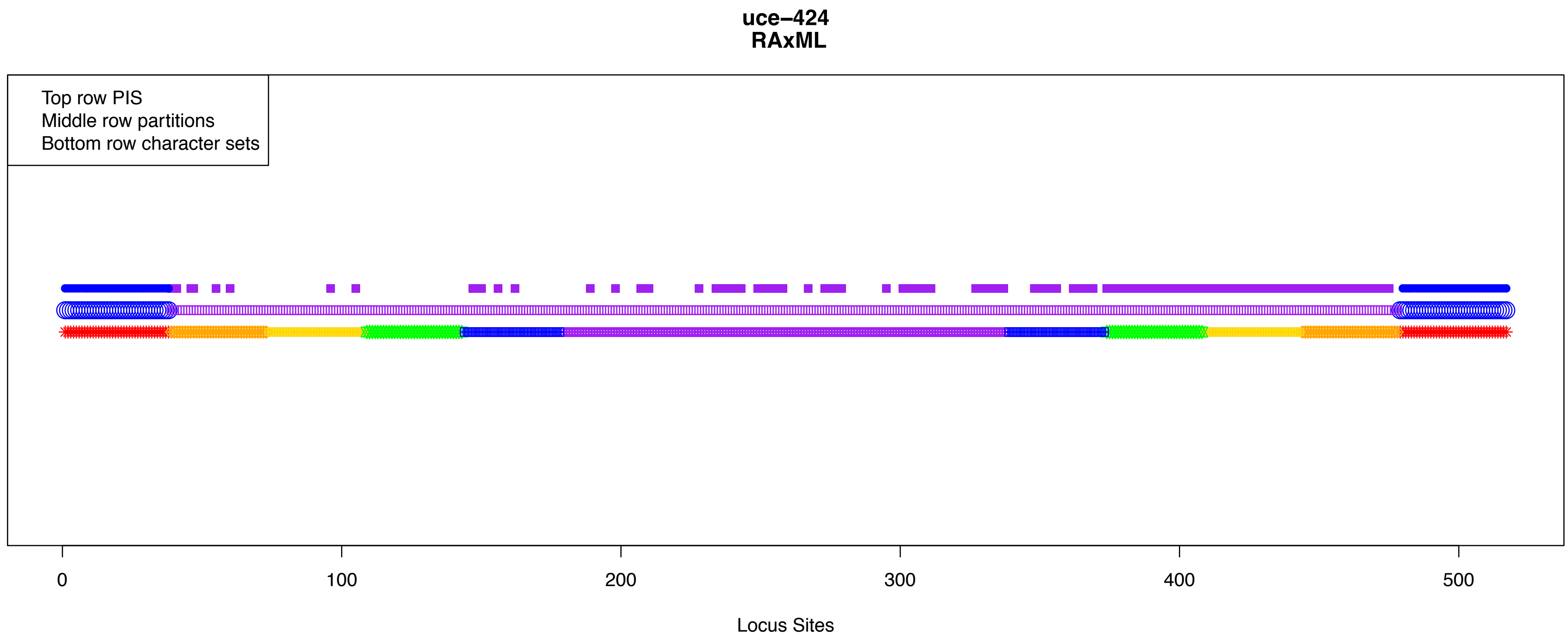
uce–424
RAxML
Top row PIS
Middle row partitions
Bottom row character sets
0
100
200
300
400
500
Locus Sites

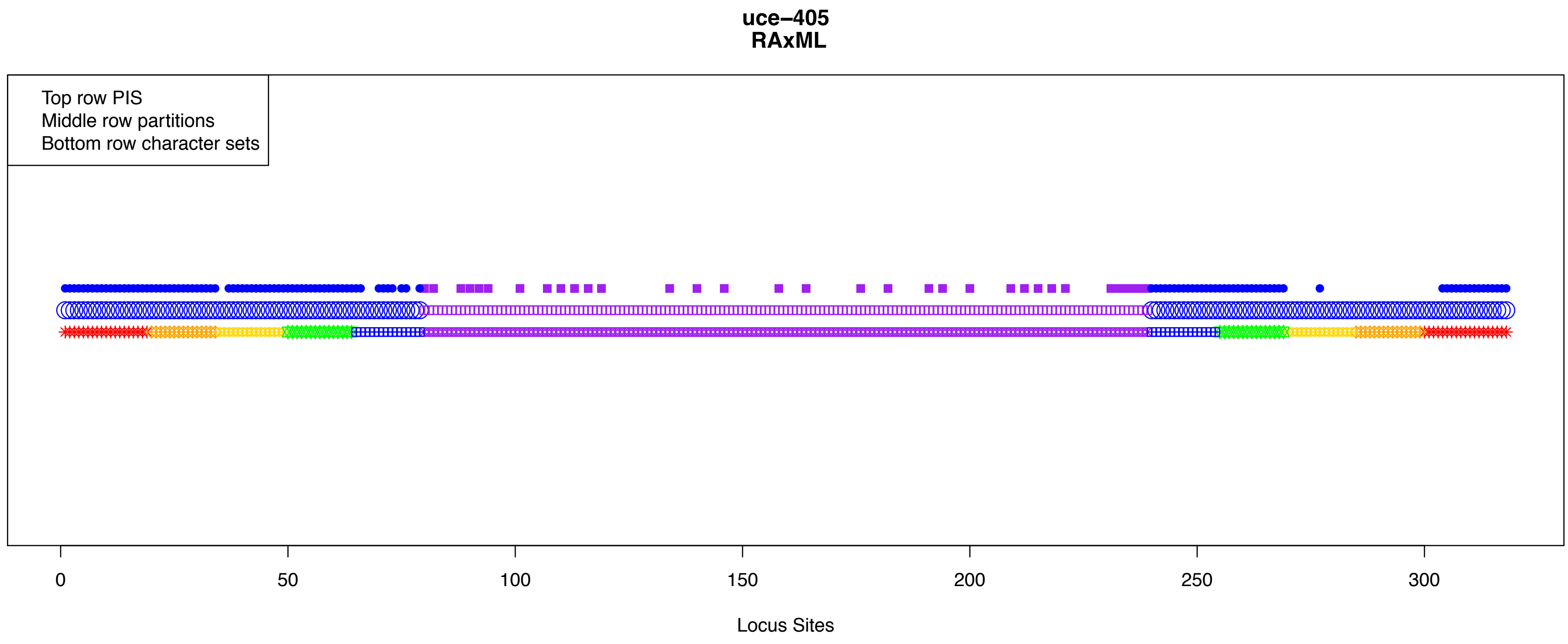
uce–405
RAxML
Top row PIS
Middle row partitions
Bottom row character sets
0
50
100
150
200
250
300
Locus Sites

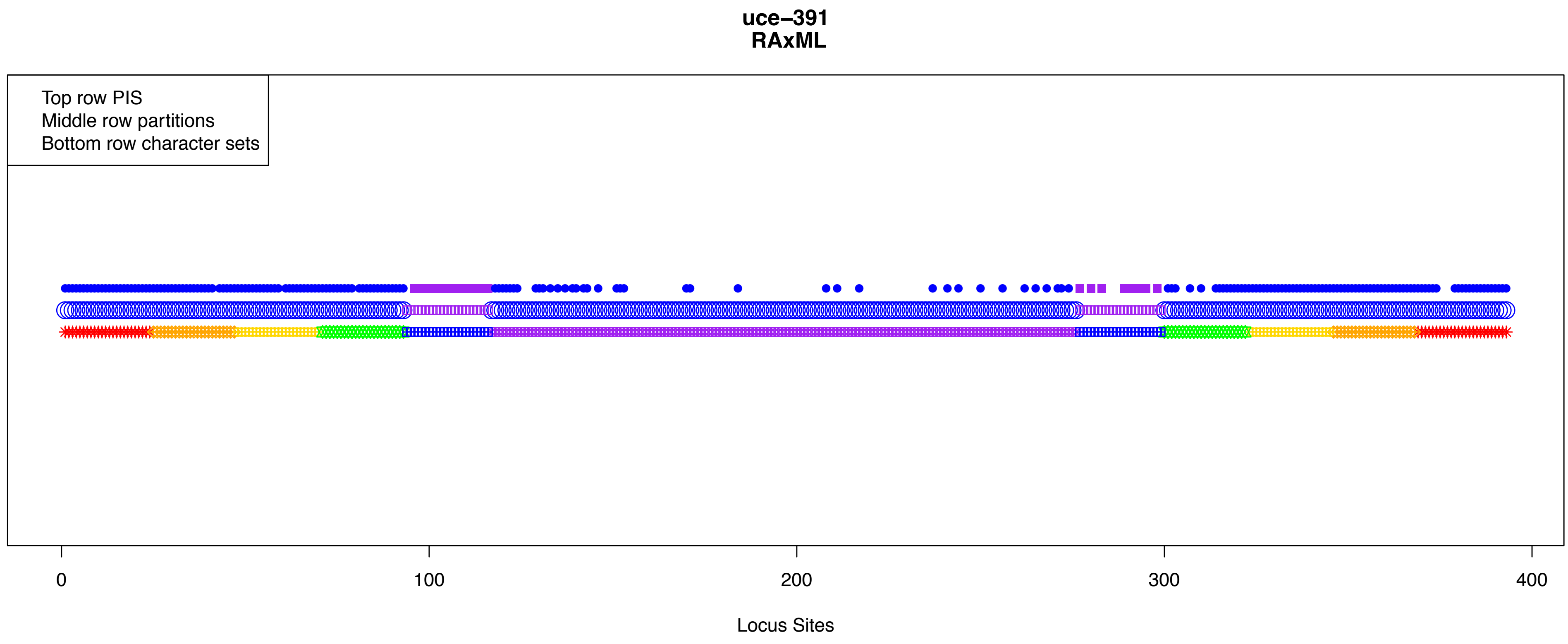

uce–391
RAxML
Top row PIS
Middle row partitions
Bottom row character sets
0
100
200
300
400
Locus Sites

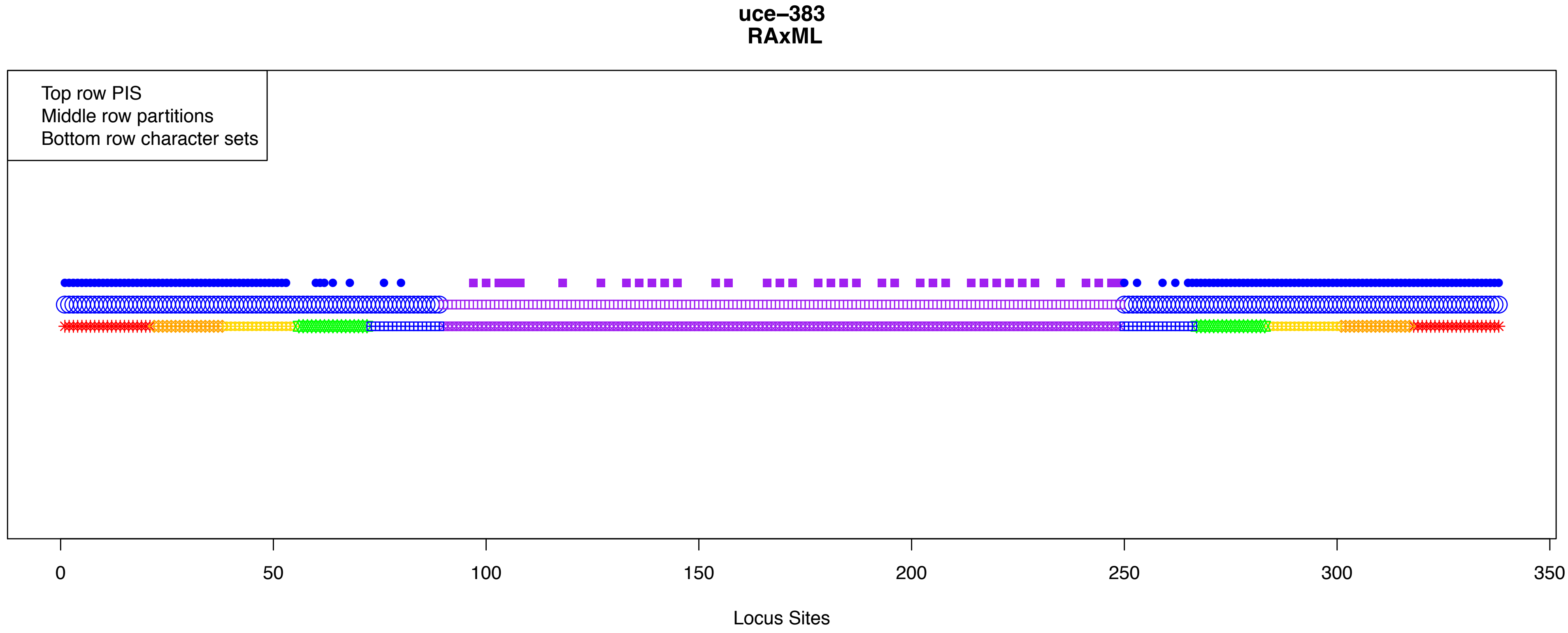
uce–383
RAxML
Top row PIS
Middle row partitions
Bottom row character sets
0
50
100
150
200
250
300
350
Locus Sites

uce–38
RAxML

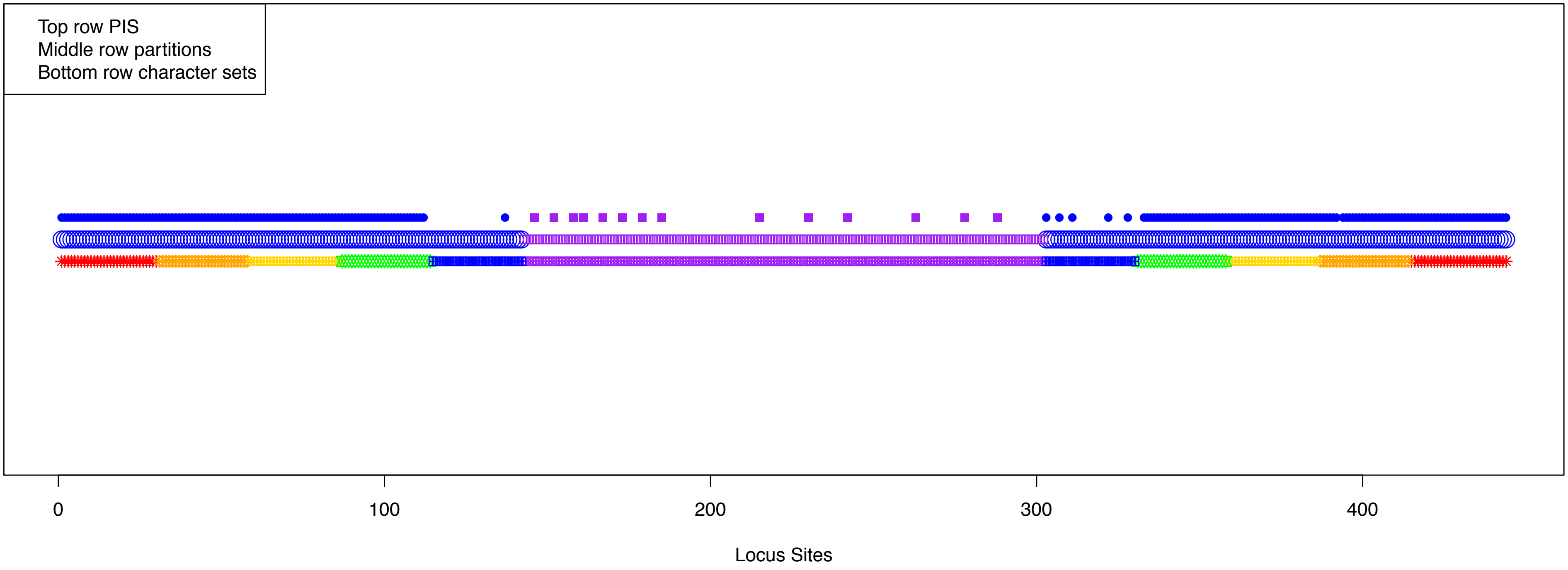
Top row PIS
Middle row partitions
Bottom row character sets
0
100
200
300
400
Locus Sites

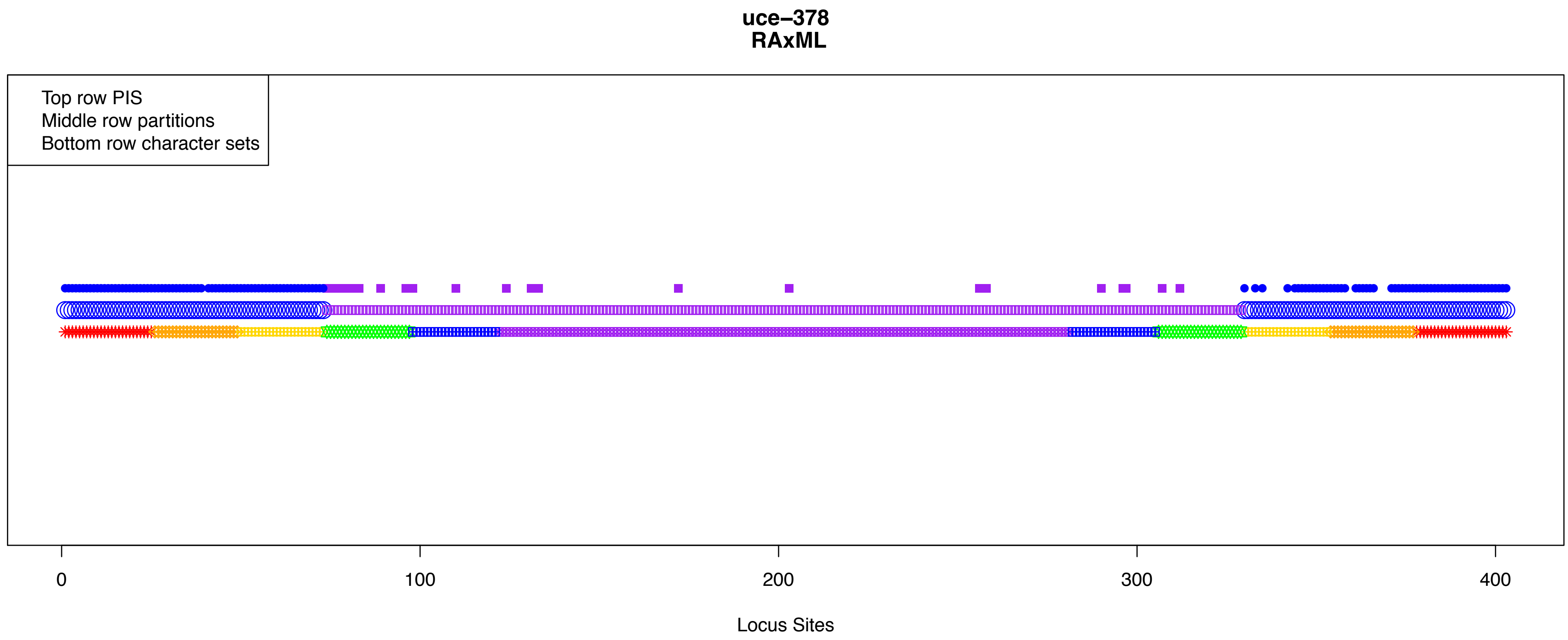

uce–378
RAxML
Top row PIS
Middle row partitions
Bottom row character sets
0
100
200
300
400
Locus Sites

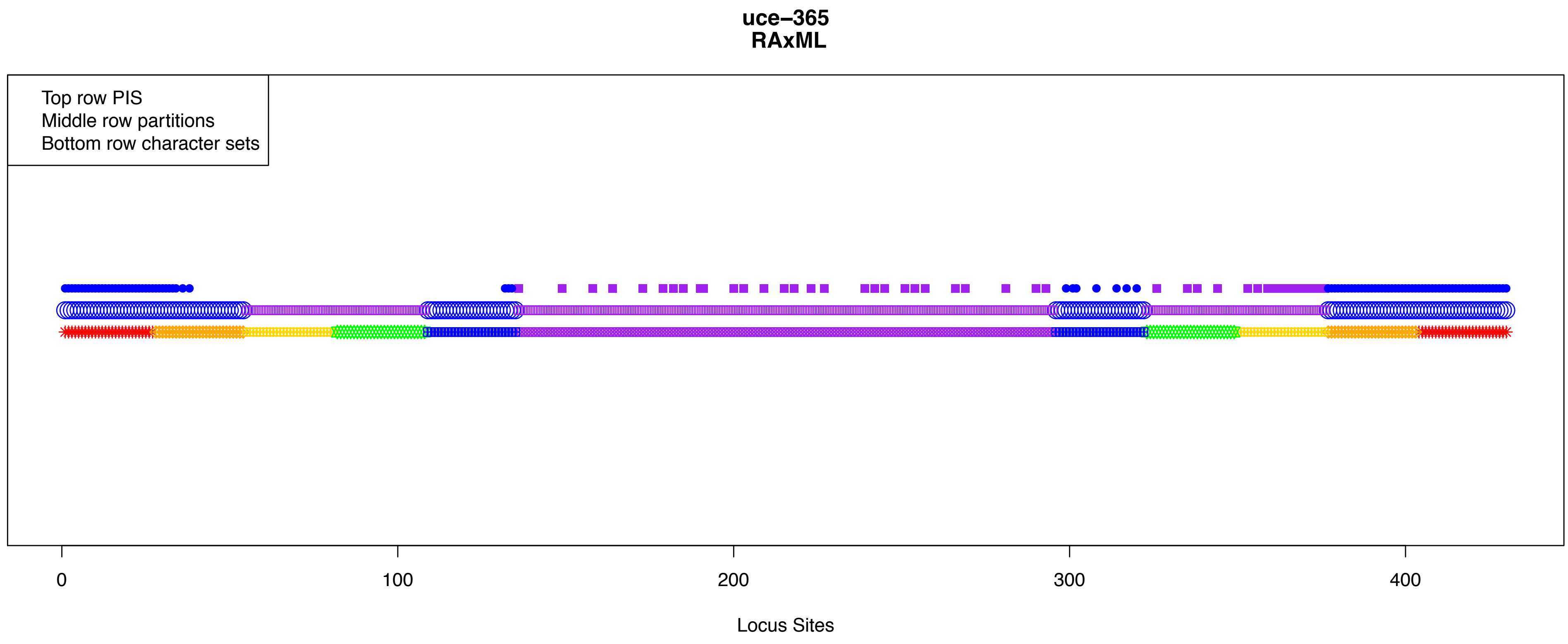

uce–365
RAxML
Top row PIS
Middle row partitions
Bottom row character sets
0
100
200
300
400
Locus Sites

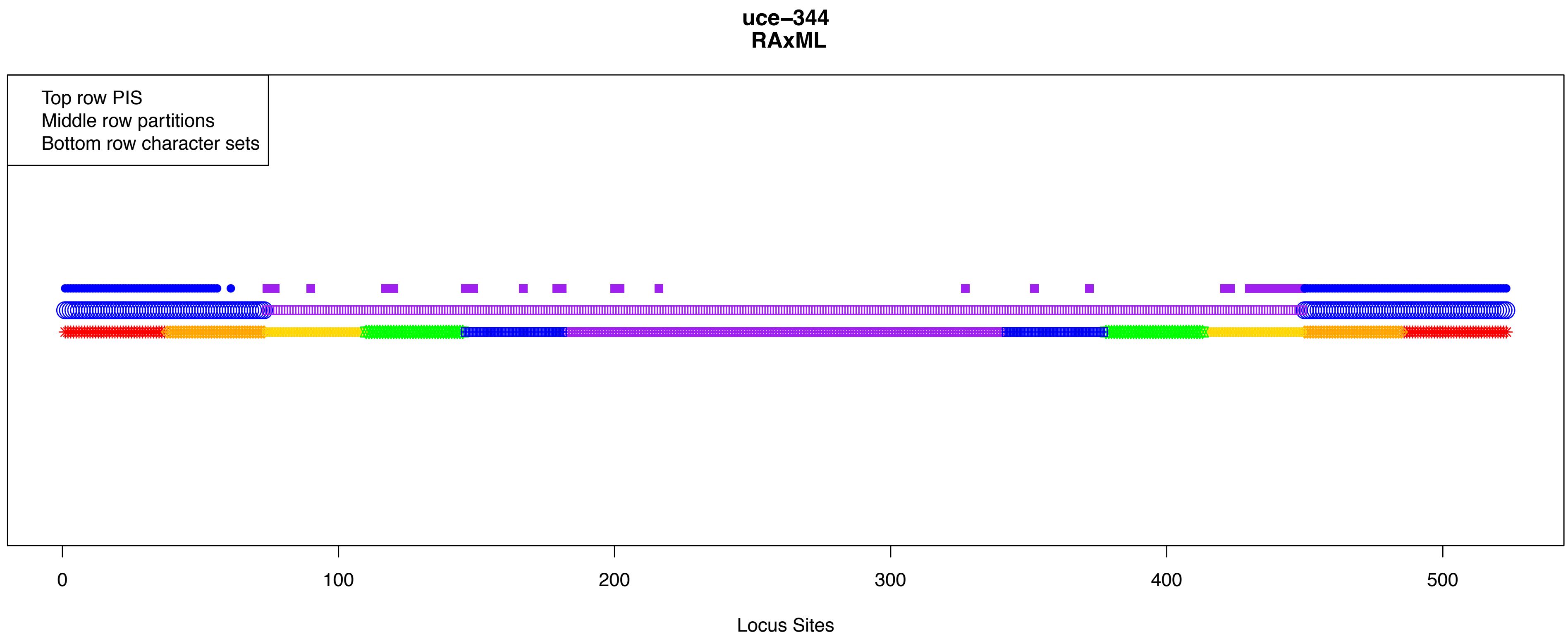

uce–344
RAxML
Top row PIS
Middle row partitions
Bottom row character sets
0
100
200
300
400
500
Locus Sites

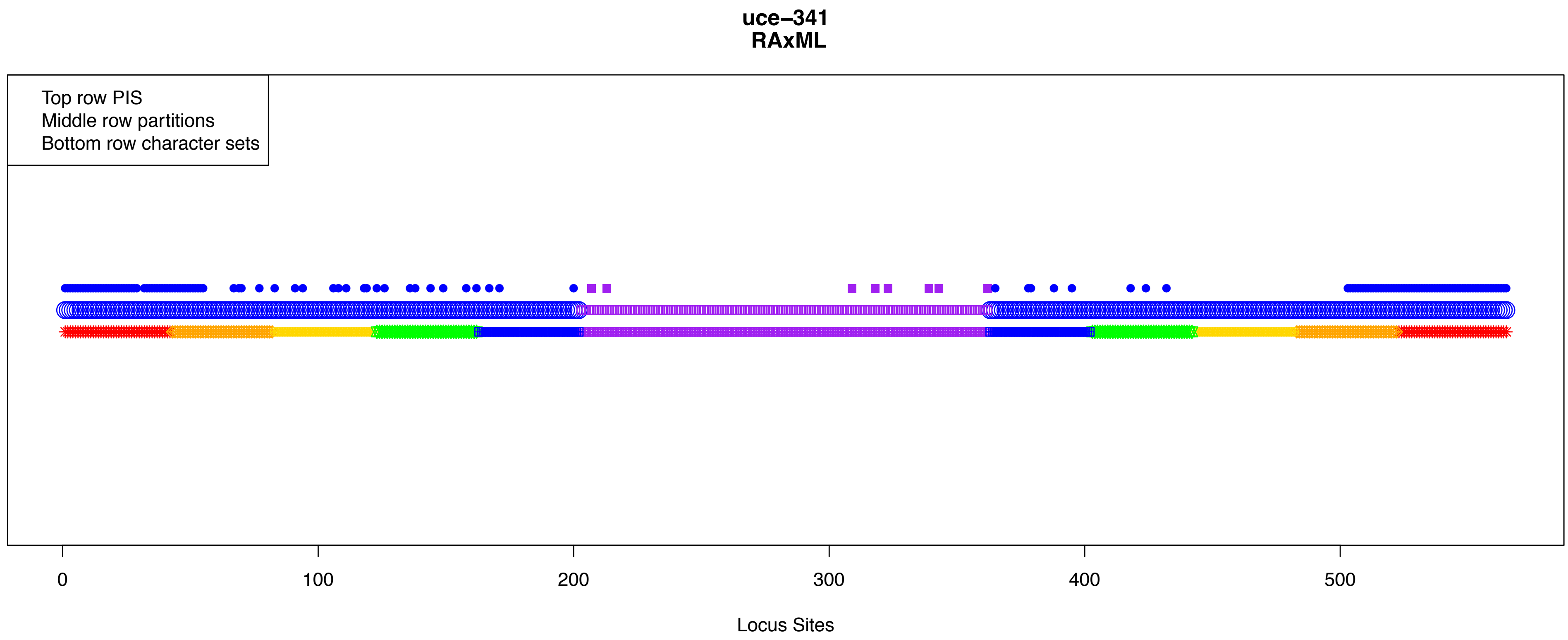
uce–341
RAxML
Top row PIS
Middle row partitions
Bottom row character sets
0
100
200
300
400
500
Locus Sites

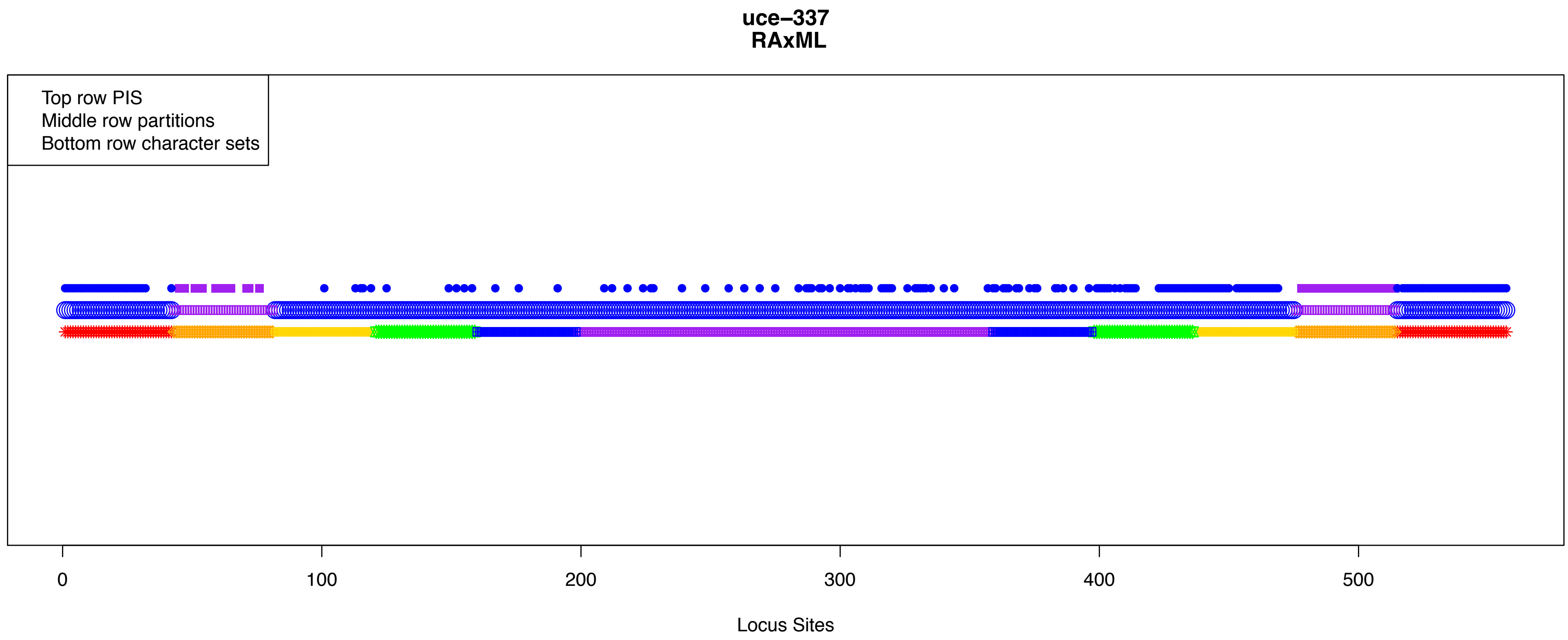
uce–337
RAxML
Top row PIS
Middle row partitions
Bottom row character sets
0
100
200
300
400
500
Locus Sites

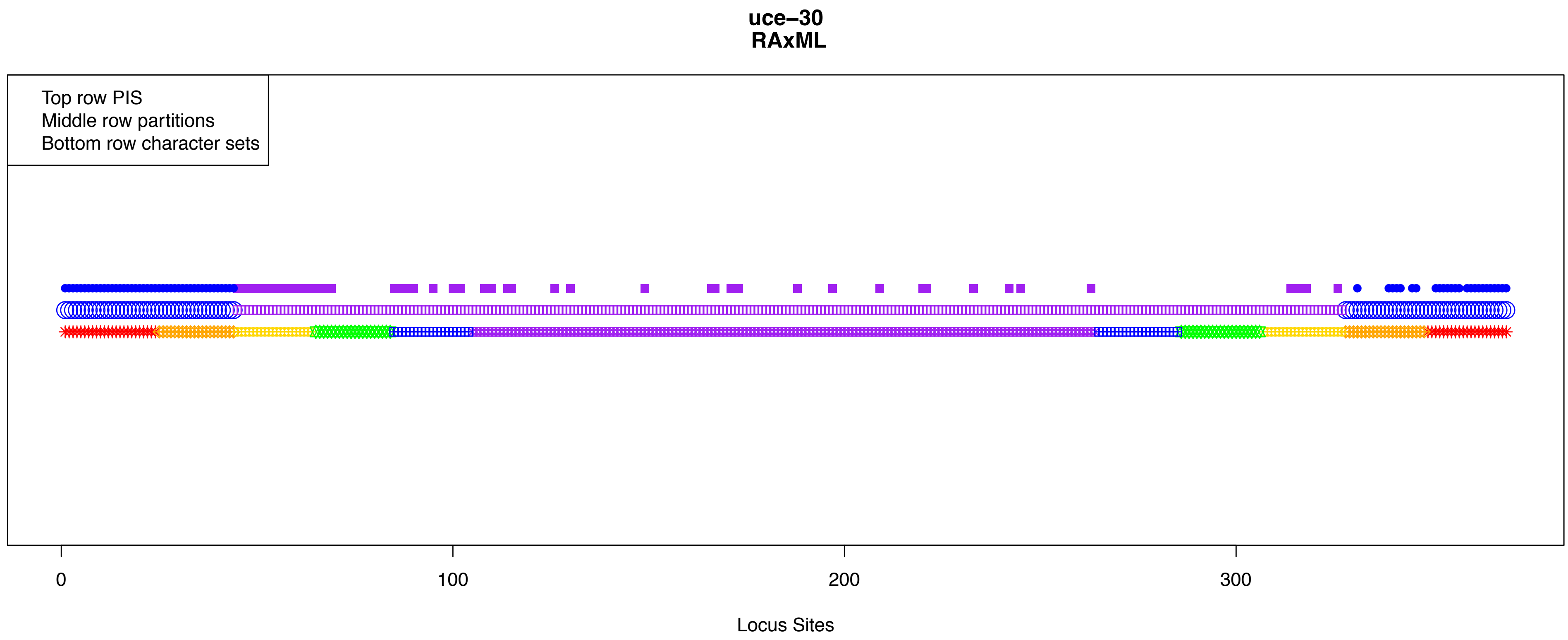
uce–30
RAxML
Top row PIS
Middle row partitions
Bottom row character sets
0
100
200
300
Locus Sites

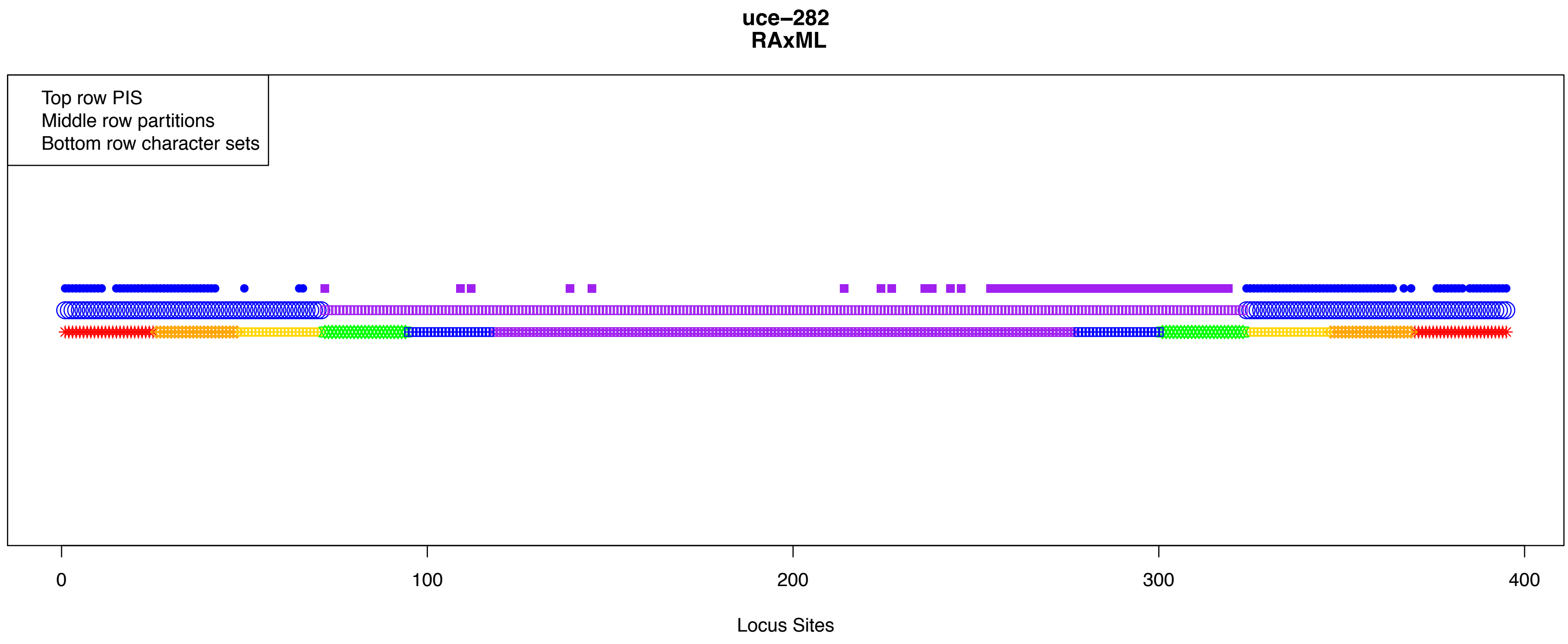
uce–282
RAxML
Top row PIS
Middle row partitions
Bottom row character sets
0
100
200
300
400
Locus Sites

uce–28
RAxML

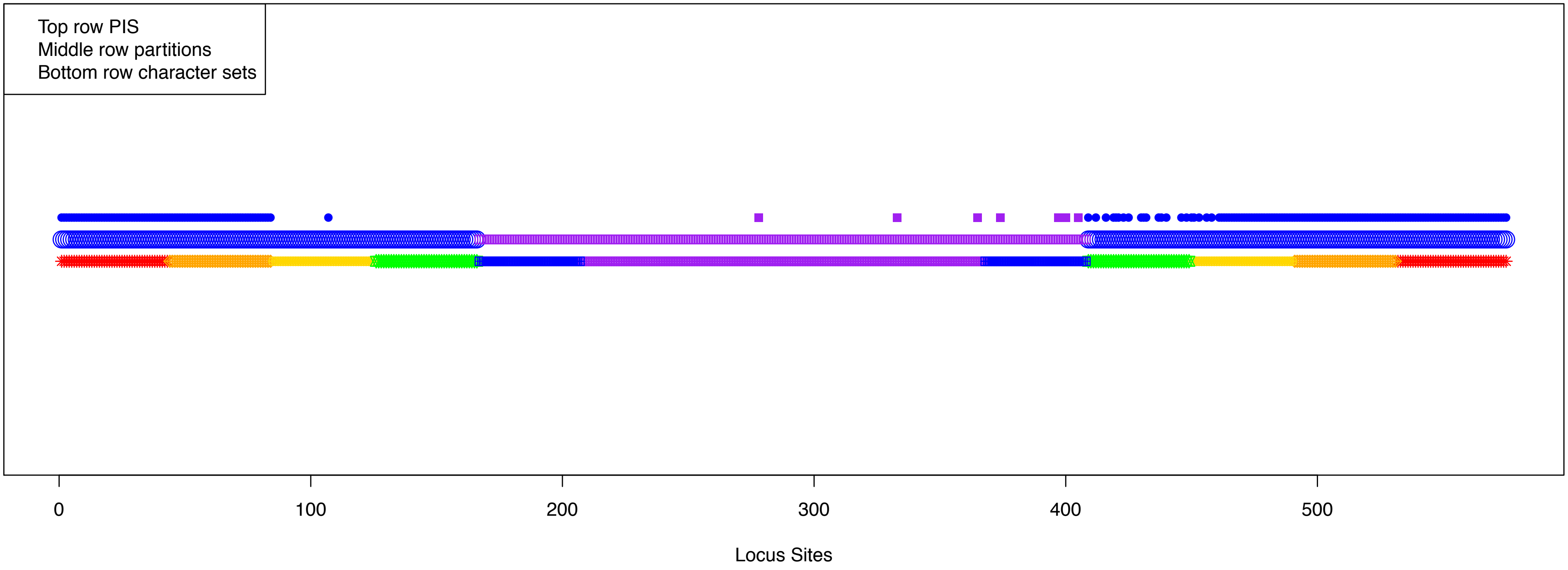

Top row PIS
Middle row partitions
Bottom row character sets
0
100
200
300
400
500
Locus Sites

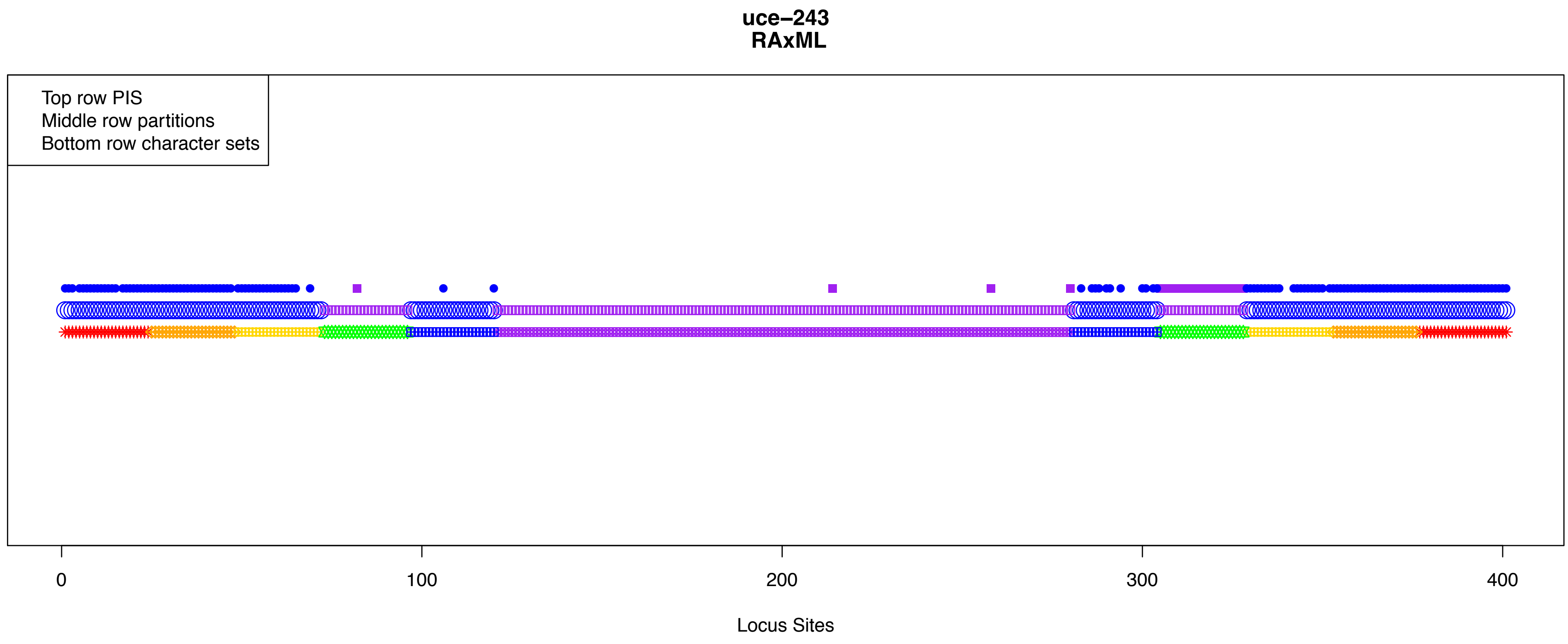
uce–243
RAxML
Top row PIS
Middle row partitions
Bottom row character sets
0
100
200
300
400
Locus Sites

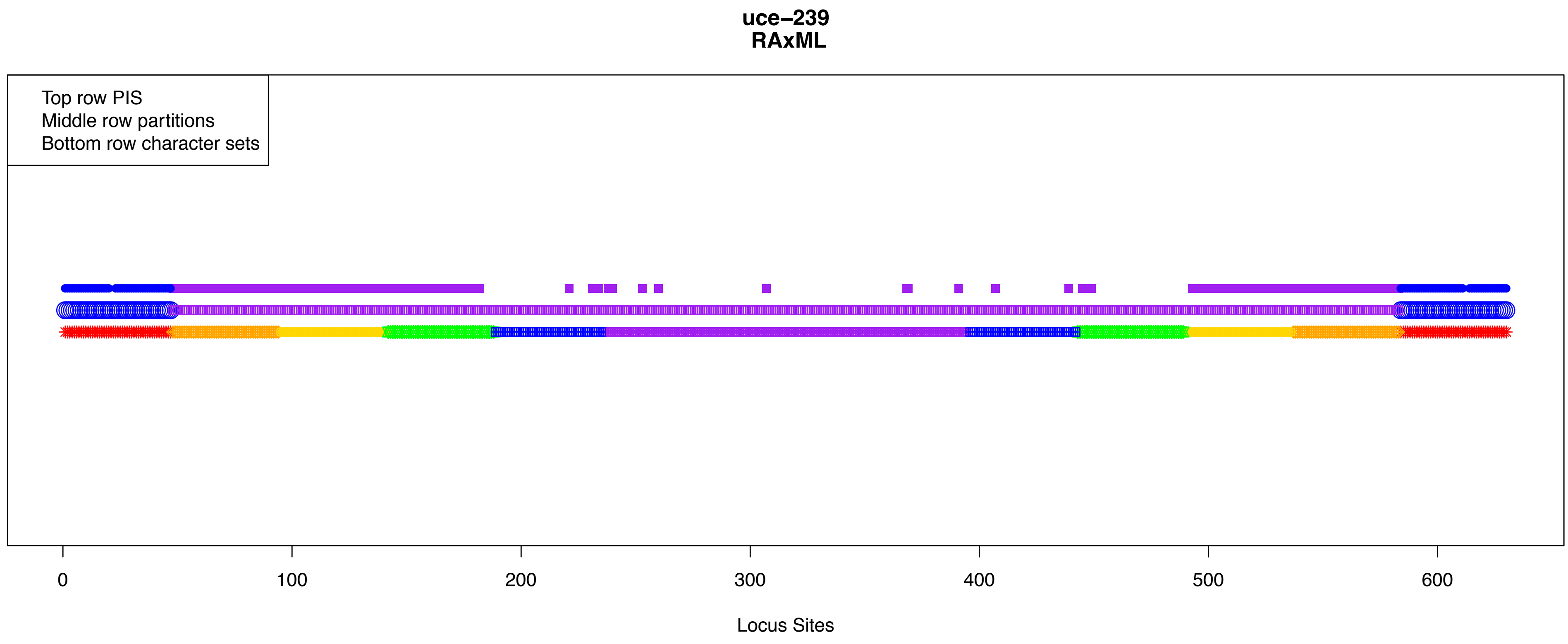
uce–239
RAxML
Top row PIS
Middle row partitions
Bottom row character sets
0
100
200
300
400
500
600
Locus Sites

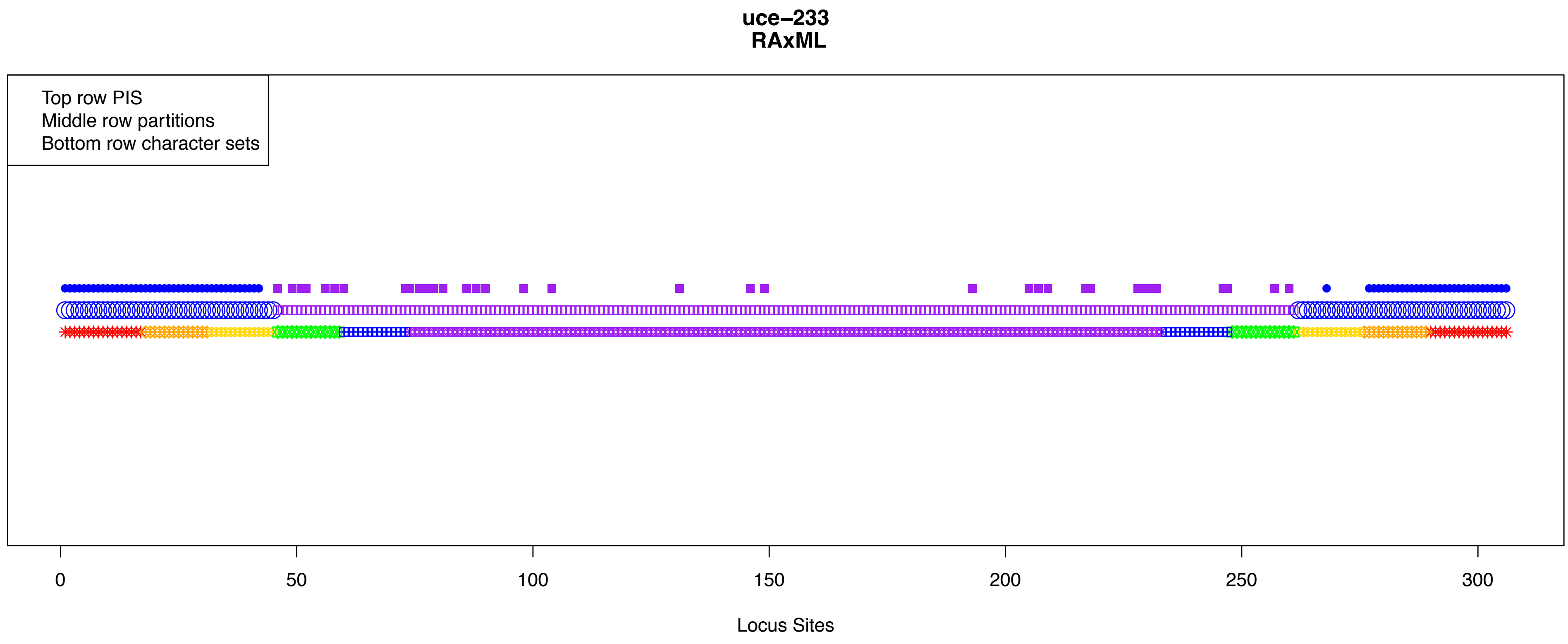
uce–233
RAxML
Top row PIS
Middle row partitions
Bottom row character sets
0
50
100
150
200
250
300
Locus Sites

uce–232
RAxML

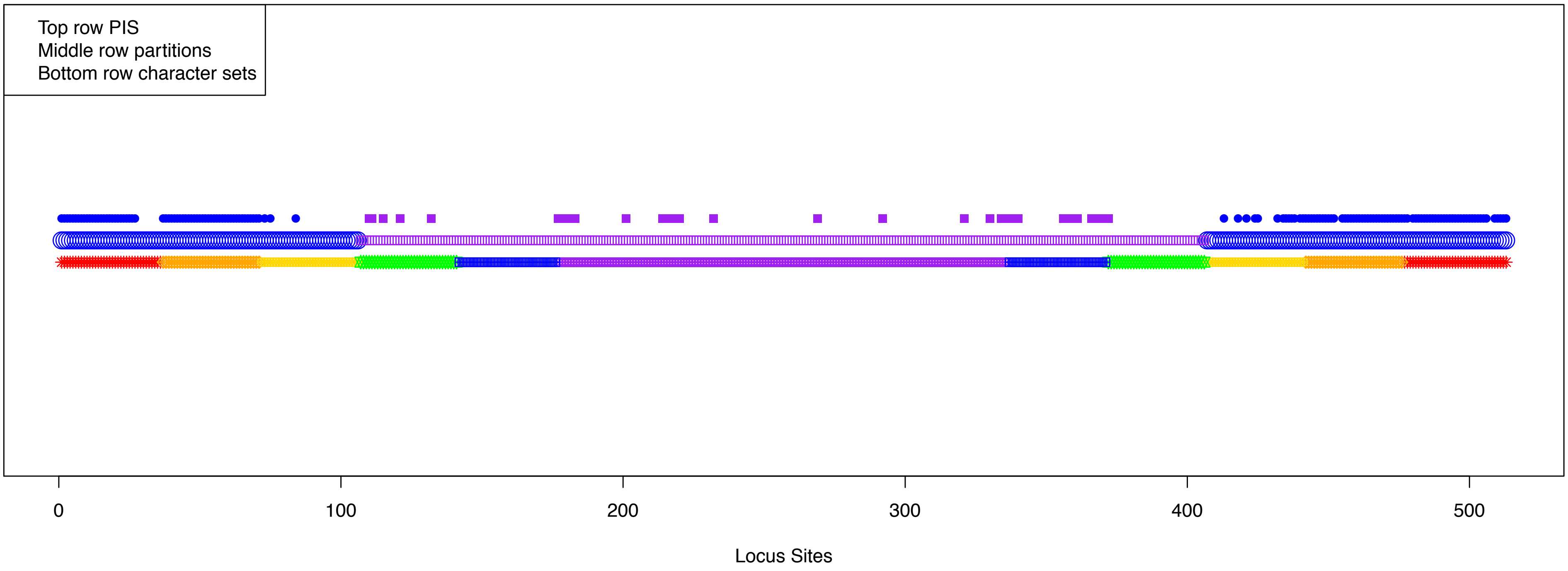

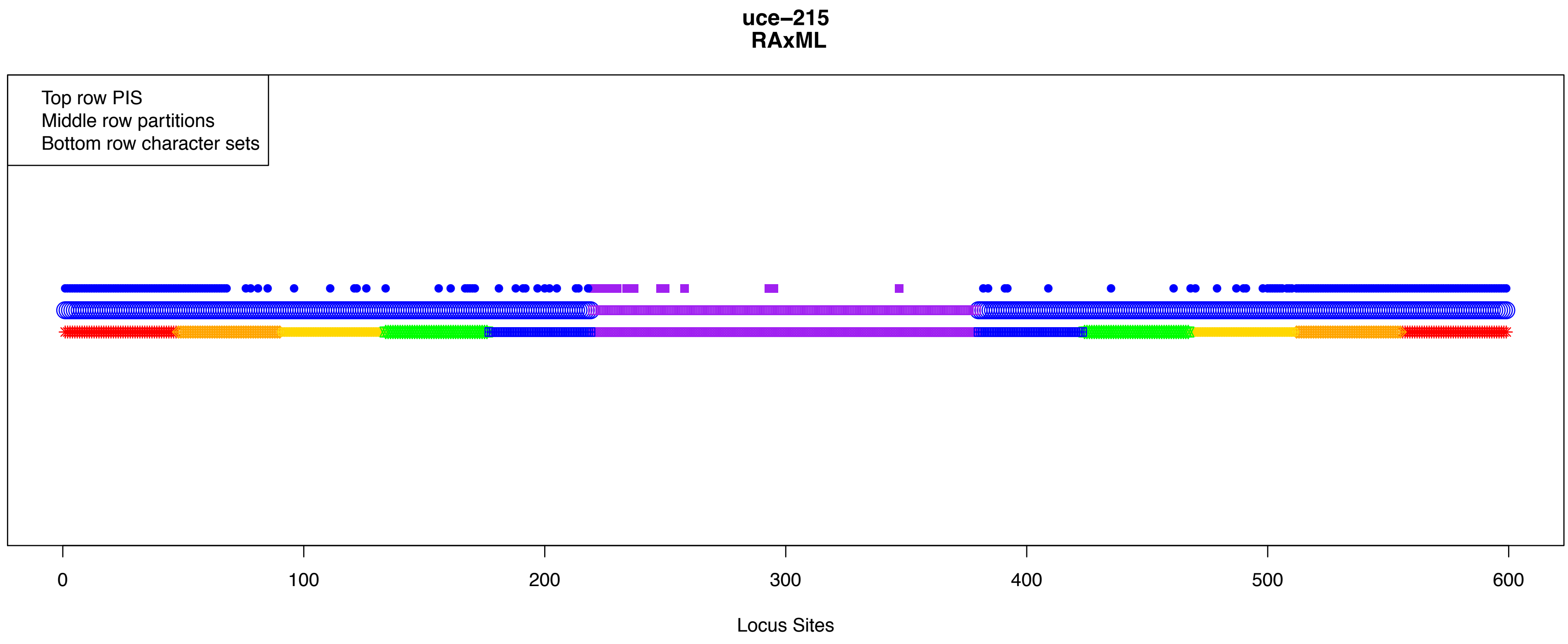

uce–215
RAxML
Top row PIS
Middle row partitions
Bottom row character sets
0
100
200
300
400
500
600
Locus Sites

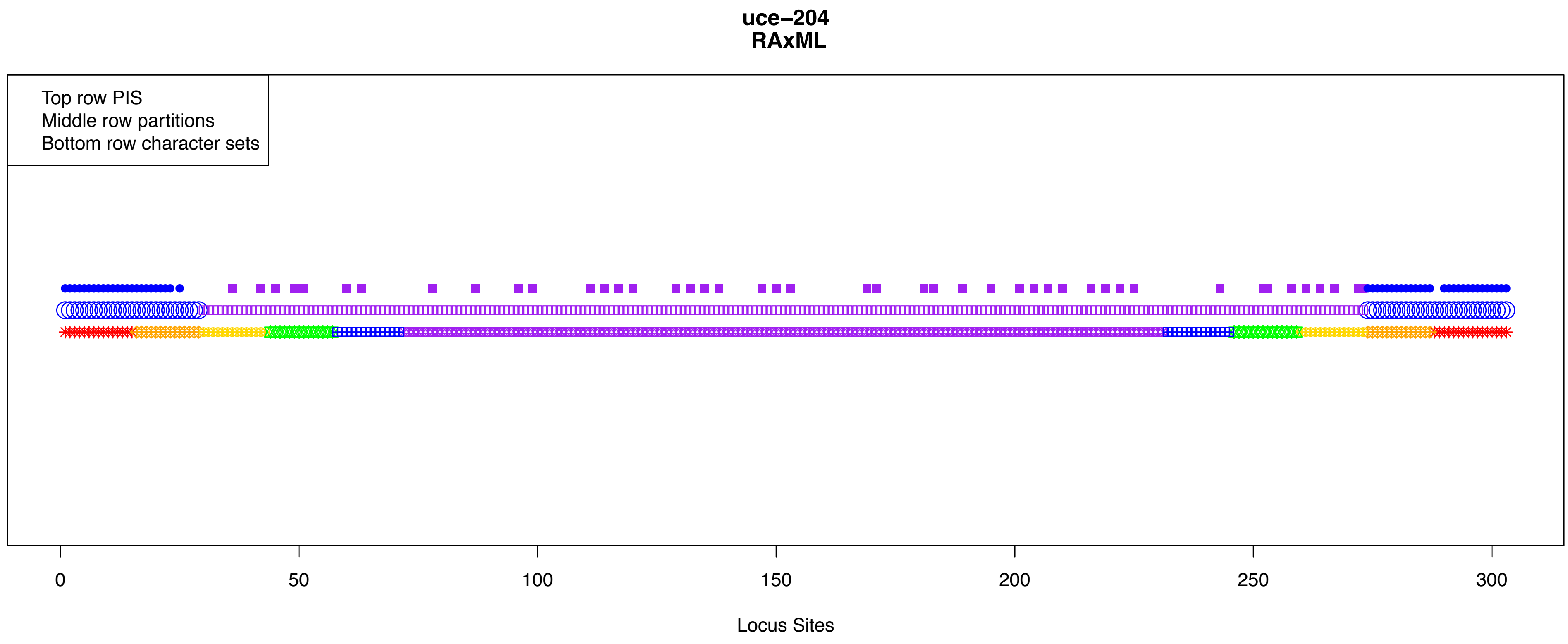
uce–204
RAxML
Top row PIS
Middle row partitions
Bottom row character sets
0
50
100
150
200
250
300
Locus Sites

uce–202
RAxML

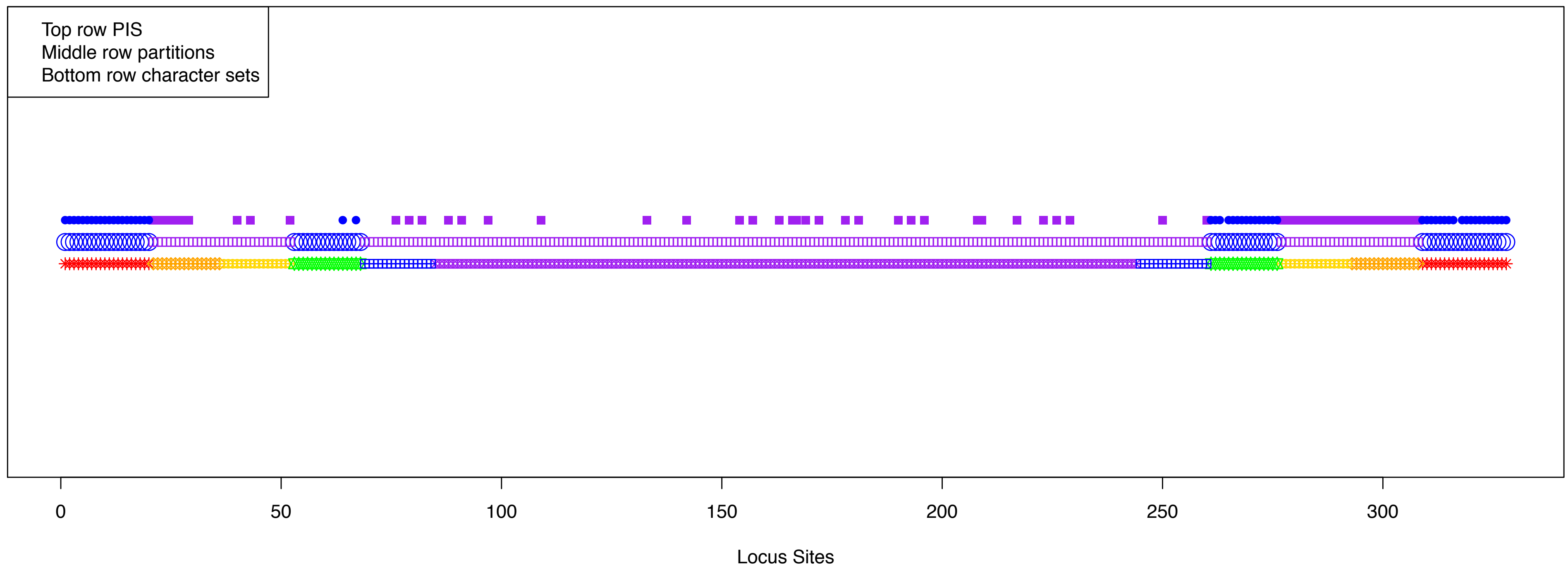

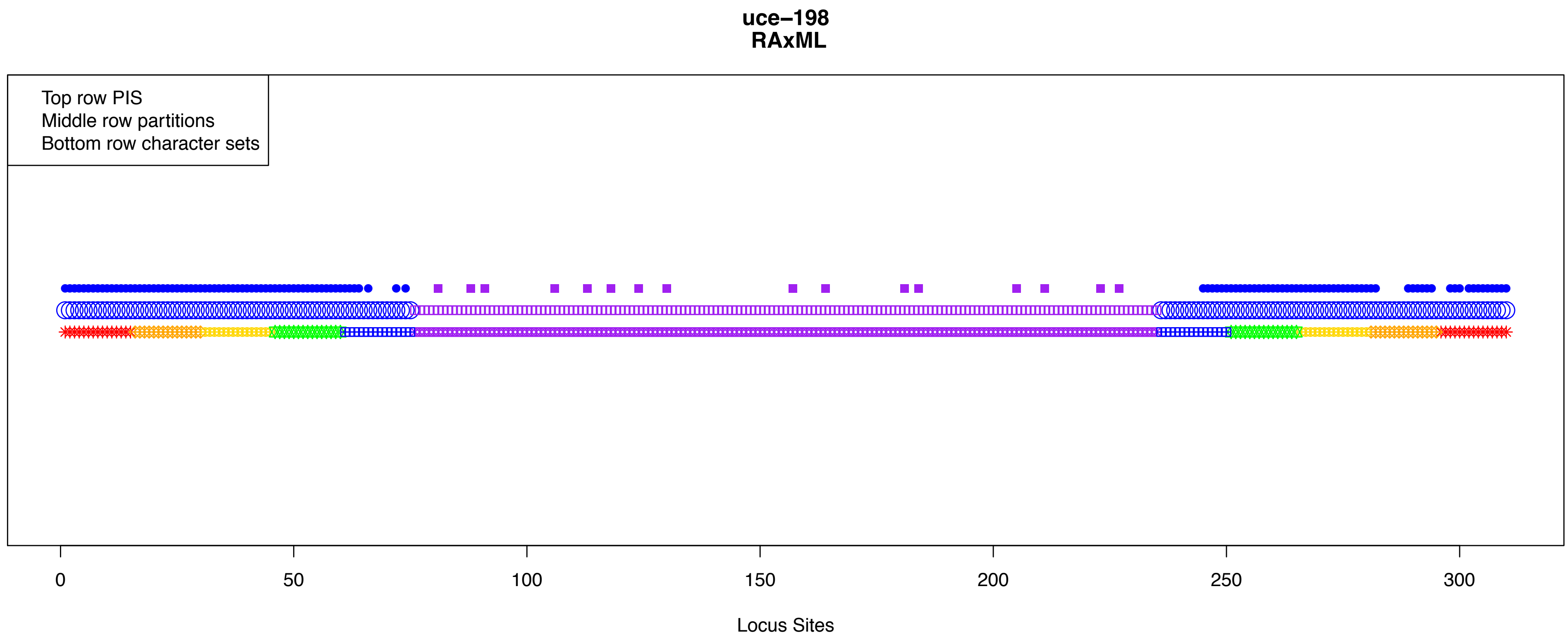
uce–198
RAxML
Top row PIS
Middle row partitions
Bottom row character sets
0
50
100
150
200
250
300
Locus Sites

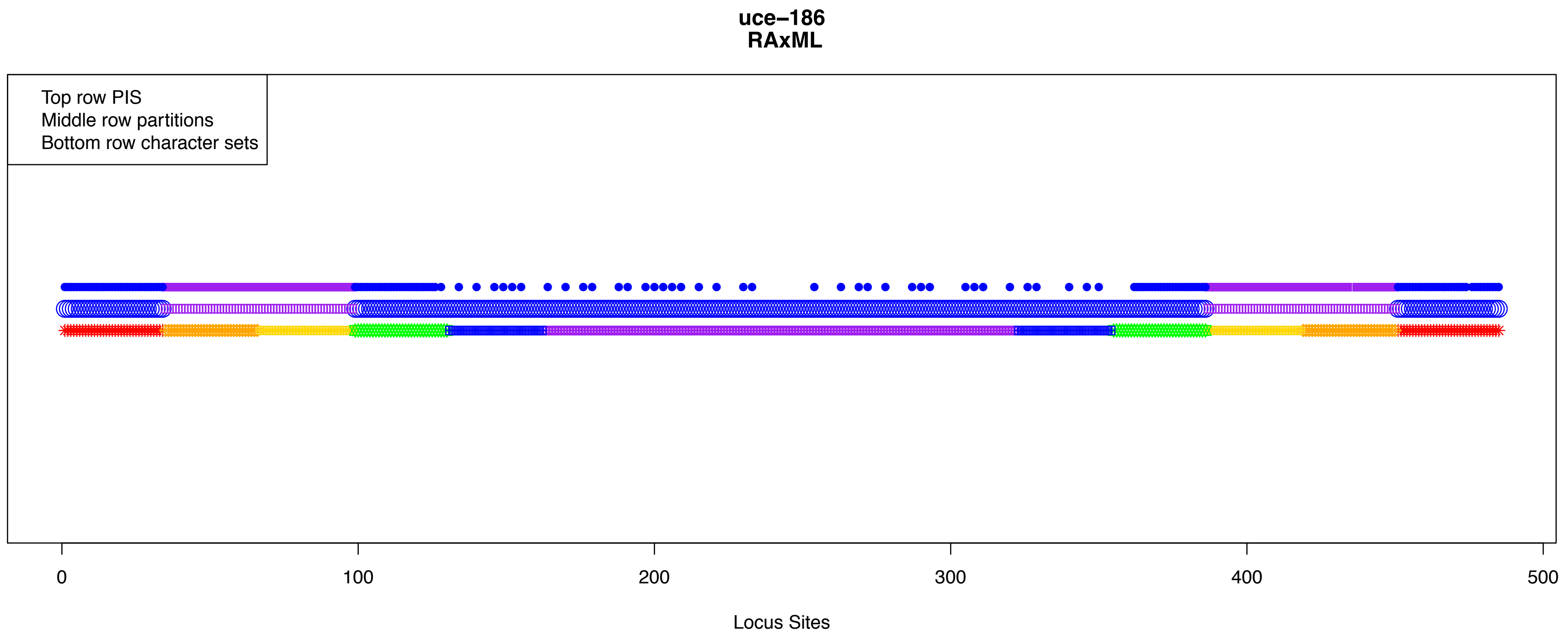
uce–186
RAxML
Top row PIS
Middle row partitions
Bottom row character sets
0
100
200
300
400
500
Locus Sites

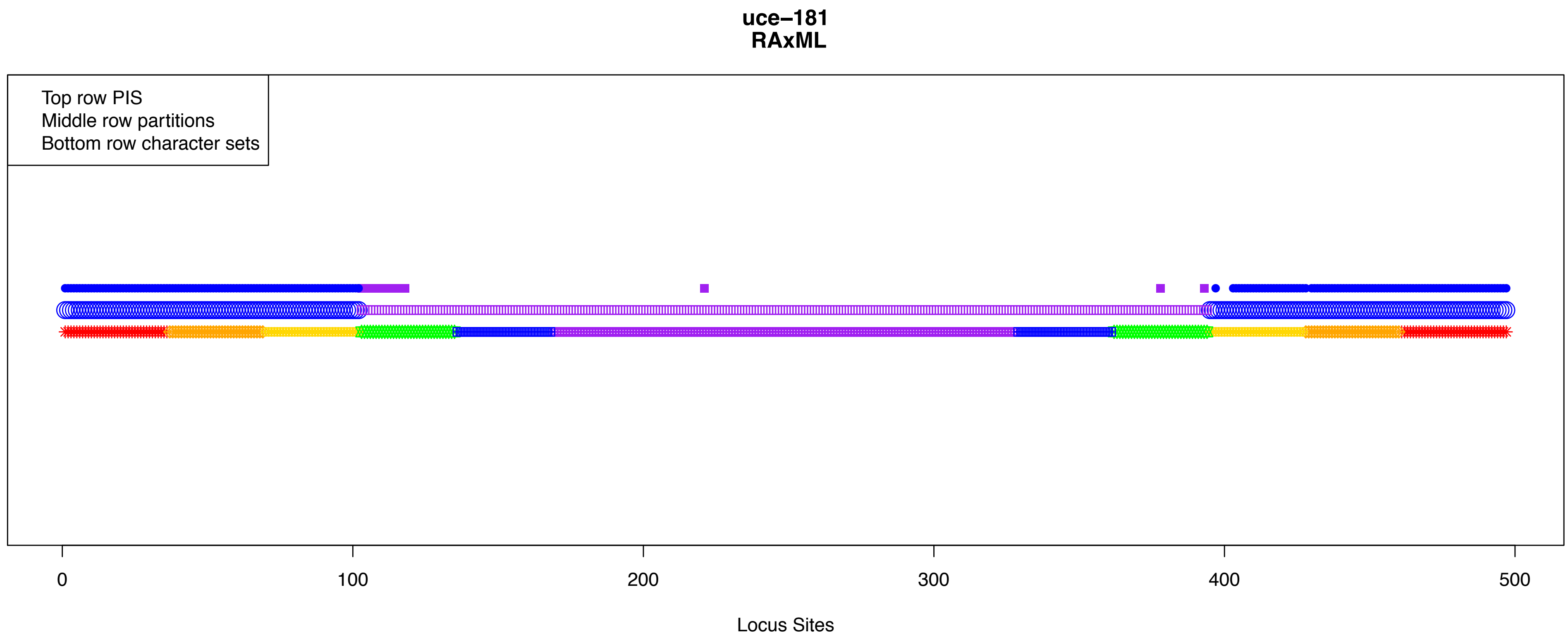

uce–181
RAxML
Top row PIS
Middle row partitions
Bottom row character sets
0
100
200
300
400
500
Locus Sites

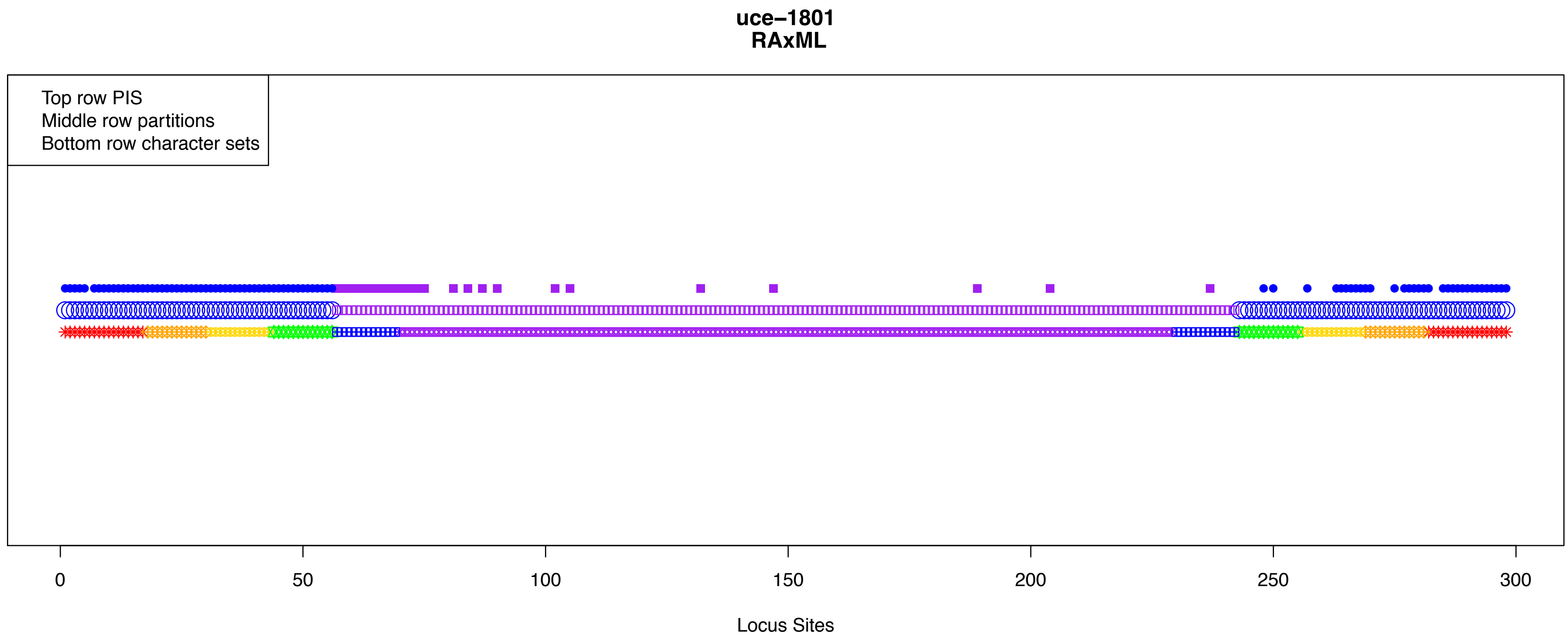
uce–1801
RAxML
Top row PIS
Middle row partitions
Bottom row character sets
0
50
100
150
200
250
300
Locus Sites

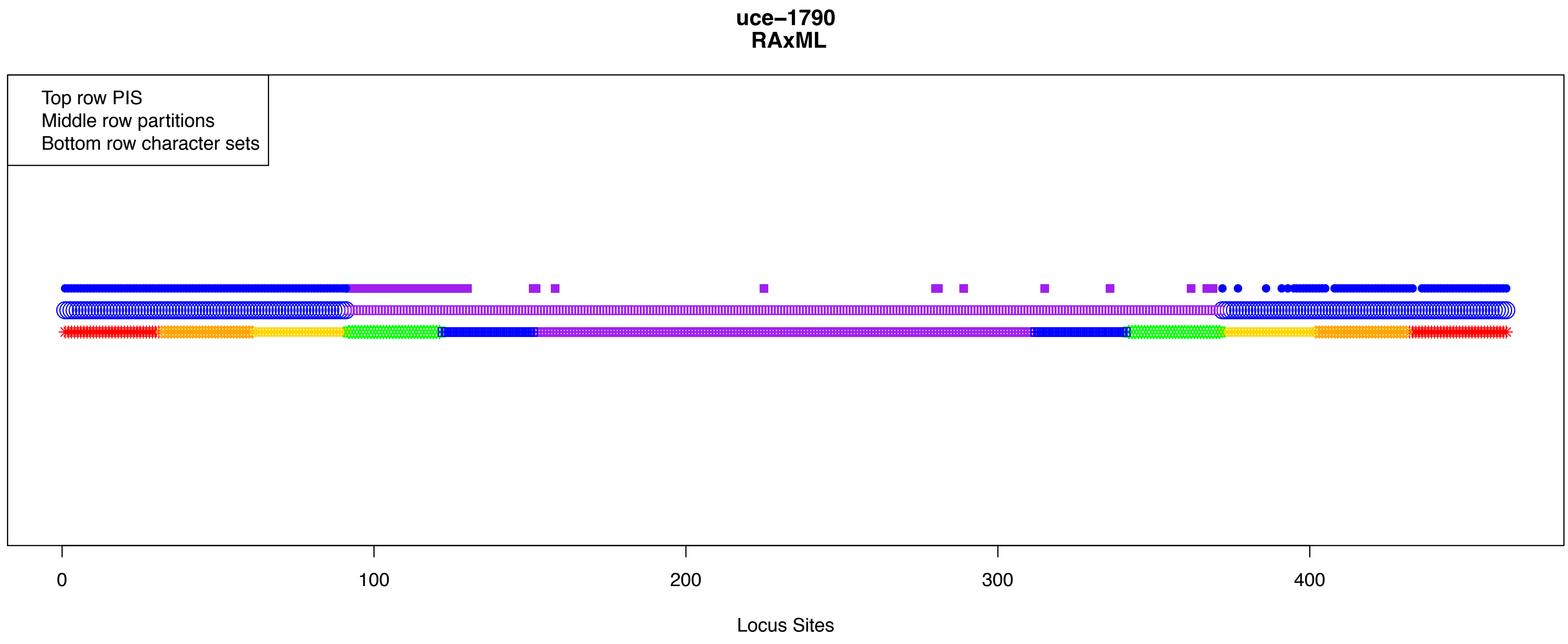
uce–1790
RAxML
Top row PIS
Middle row partitions
Bottom row character sets
0
100
200
300
400
Locus Sites

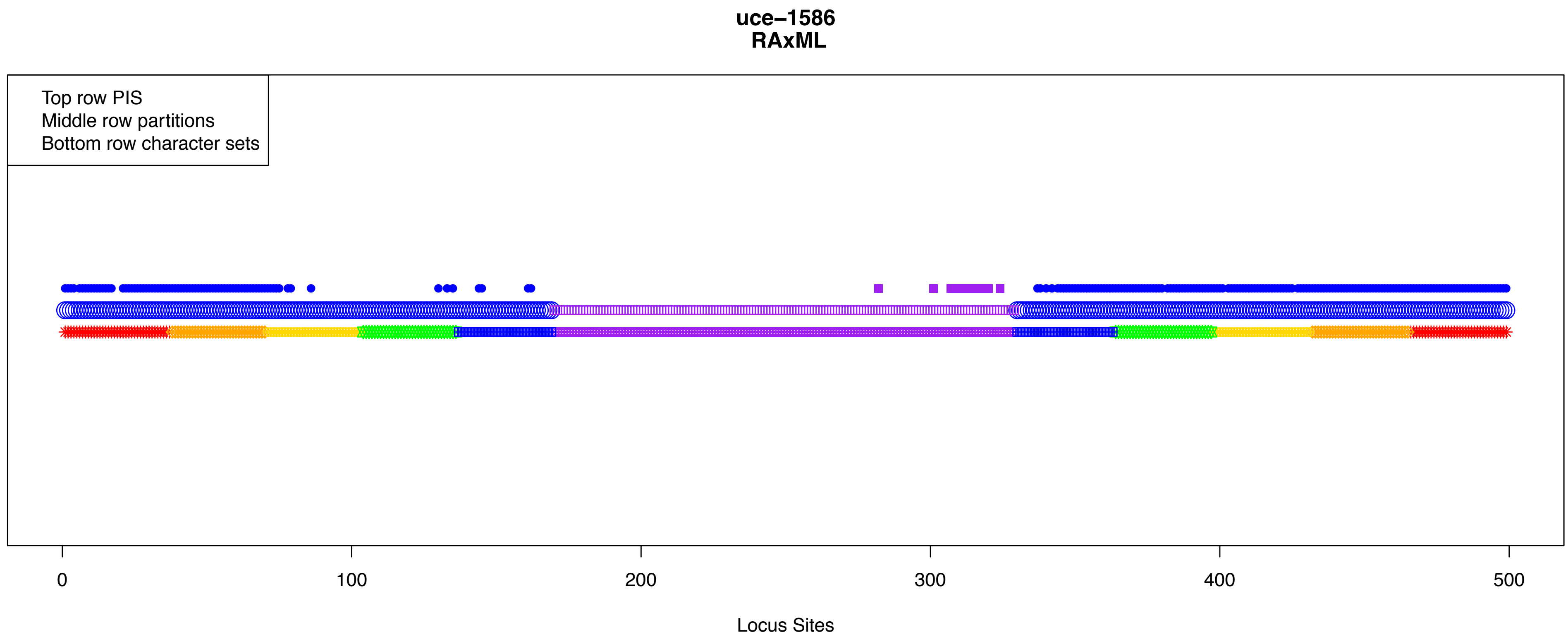
uce–1586
RAxML
Top row PIS
Middle row partitions
Bottom row character sets
0
100
200
300
400
500
Locus Sites

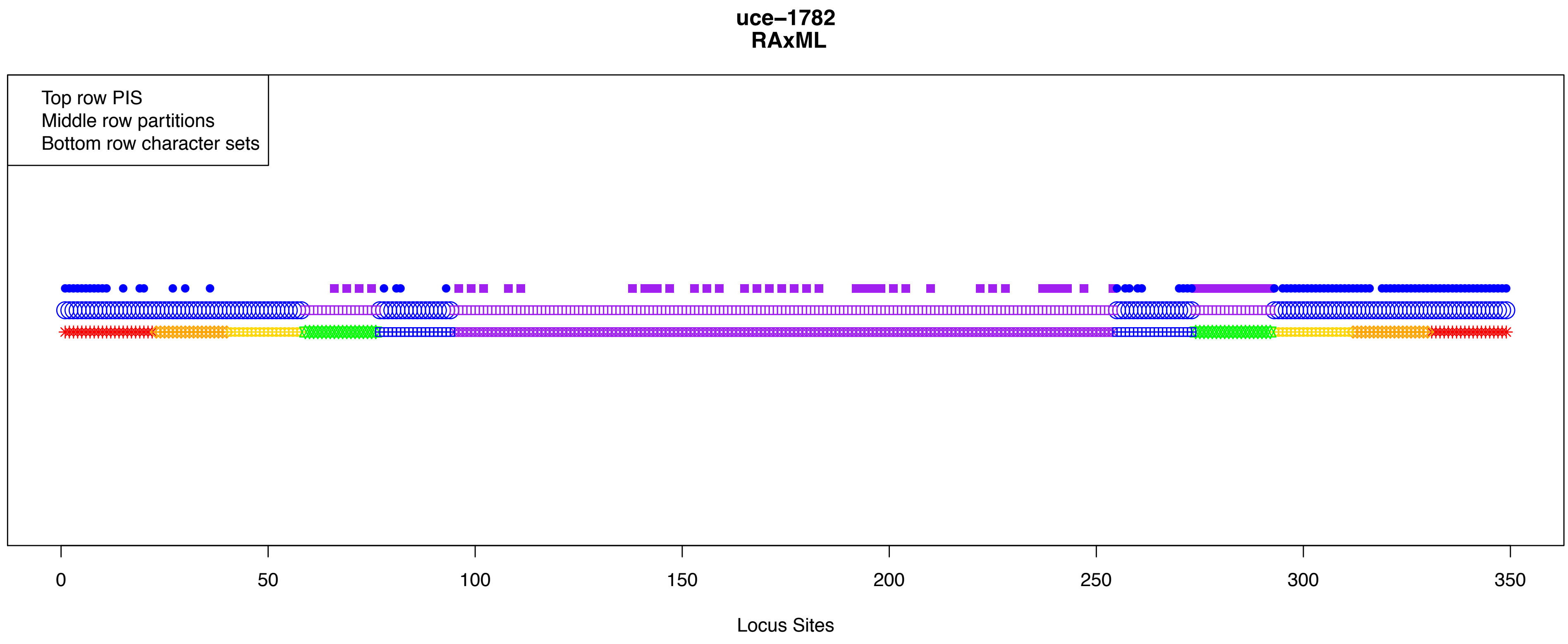
uce–1782
RAxML
Top row PIS
Middle row partitions
Bottom row character sets
0
50
100
150
200
250
300
350
Locus Sites

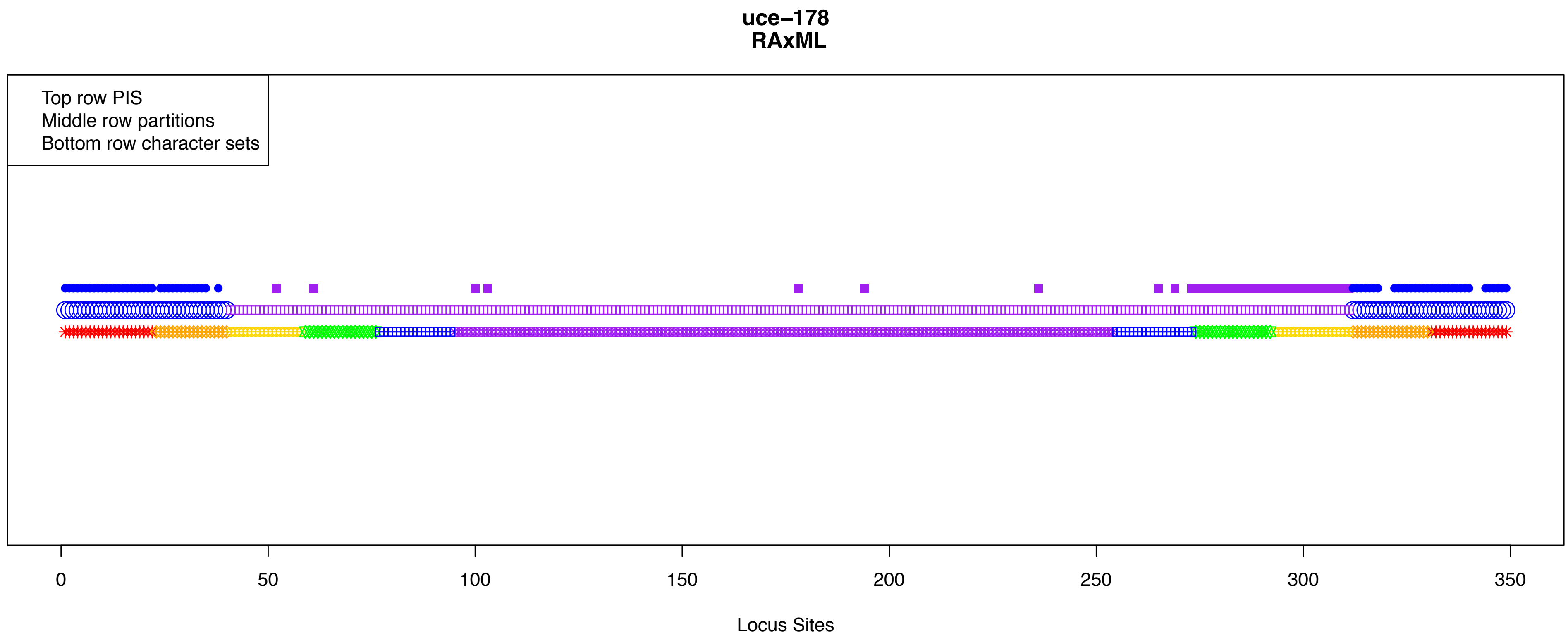
uce–178
RAxML
Top row PIS
Middle row partitions
Bottom row character sets
0
50
100
150
200
250
300
350
Locus Sites

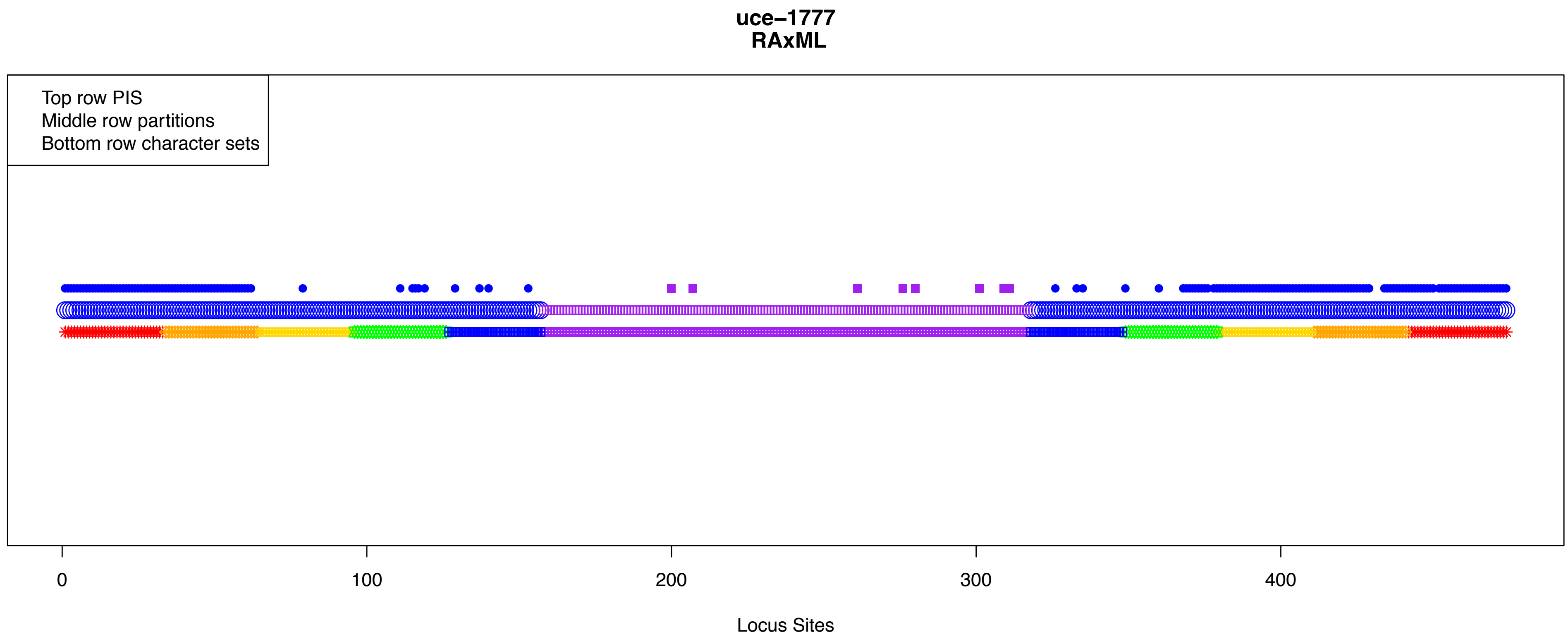

uce–1777
RAxML
Top row PIS
Middle row partitions
Bottom row character sets
0
100
200
300
400
Locus Sites

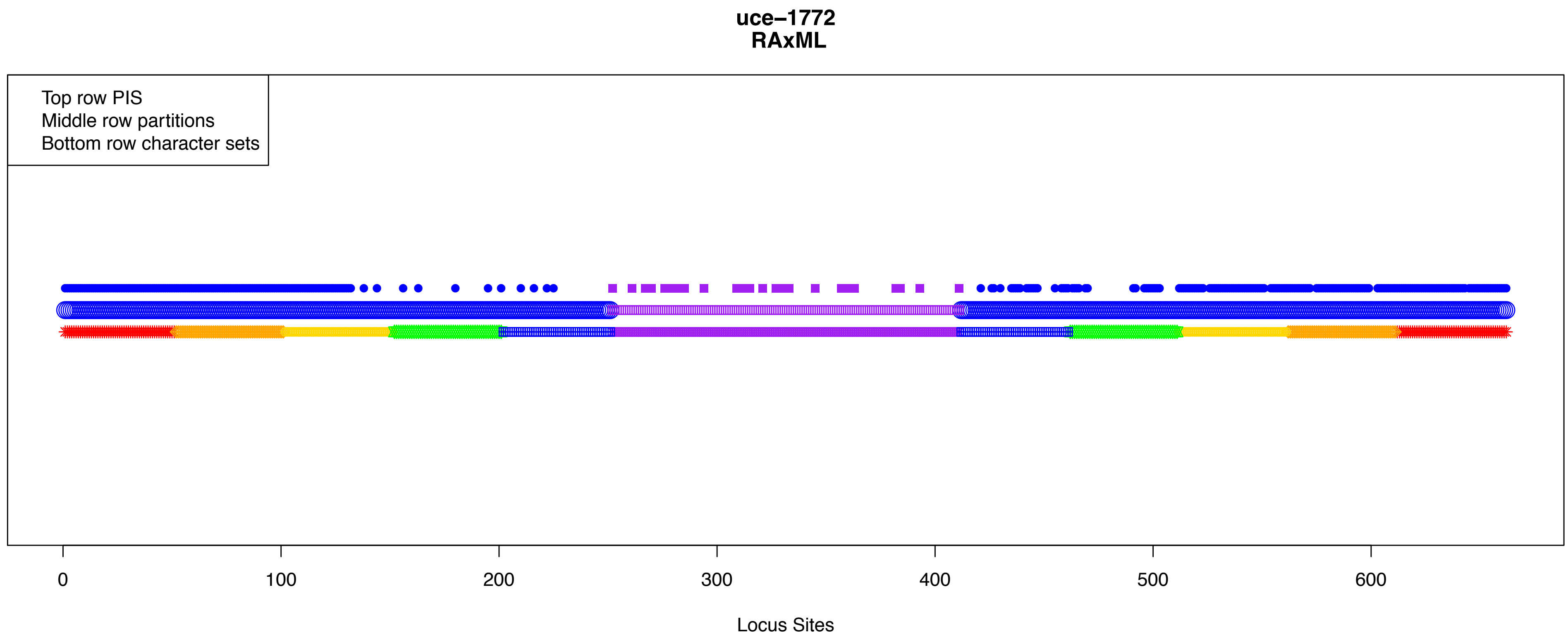
uce–1772
RAxML
Top row PIS
Middle row partitions
Bottom row character sets
0
100
200
300
400
500
600
Locus Sites

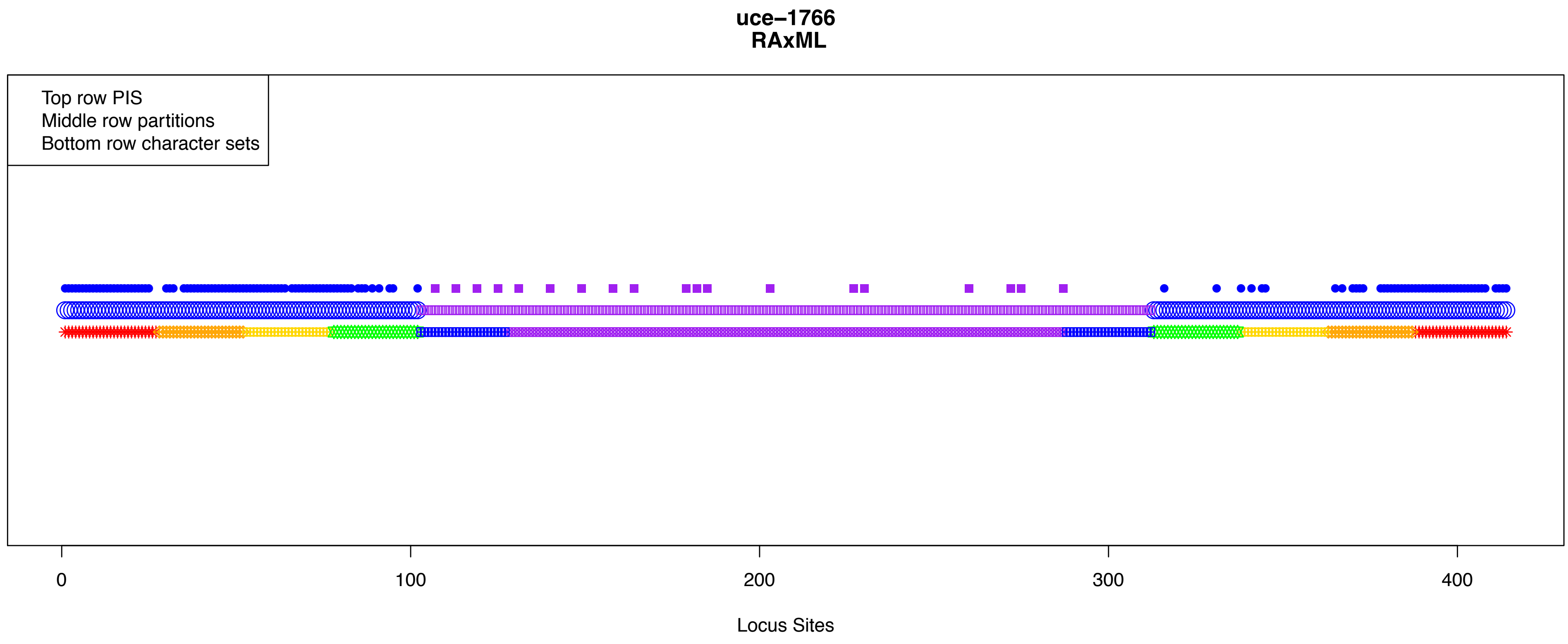
uce–1766
RAxML
Top row PIS
Middle row partitions
Bottom row character sets
0
100
200
300
400
Locus Sites

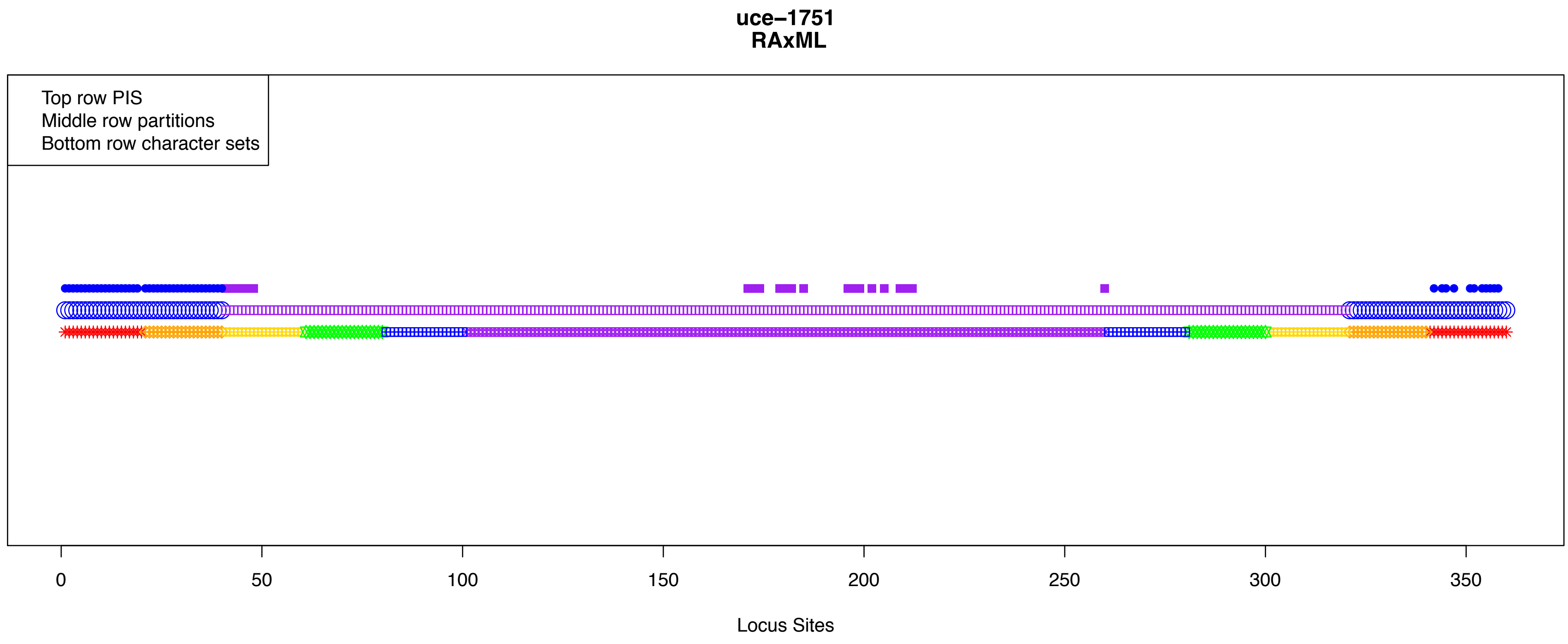
uce–1751
RAxML
Top row PIS
Middle row partitions
Bottom row character sets
0
50
100
150
200
250
300
350
Locus Sites

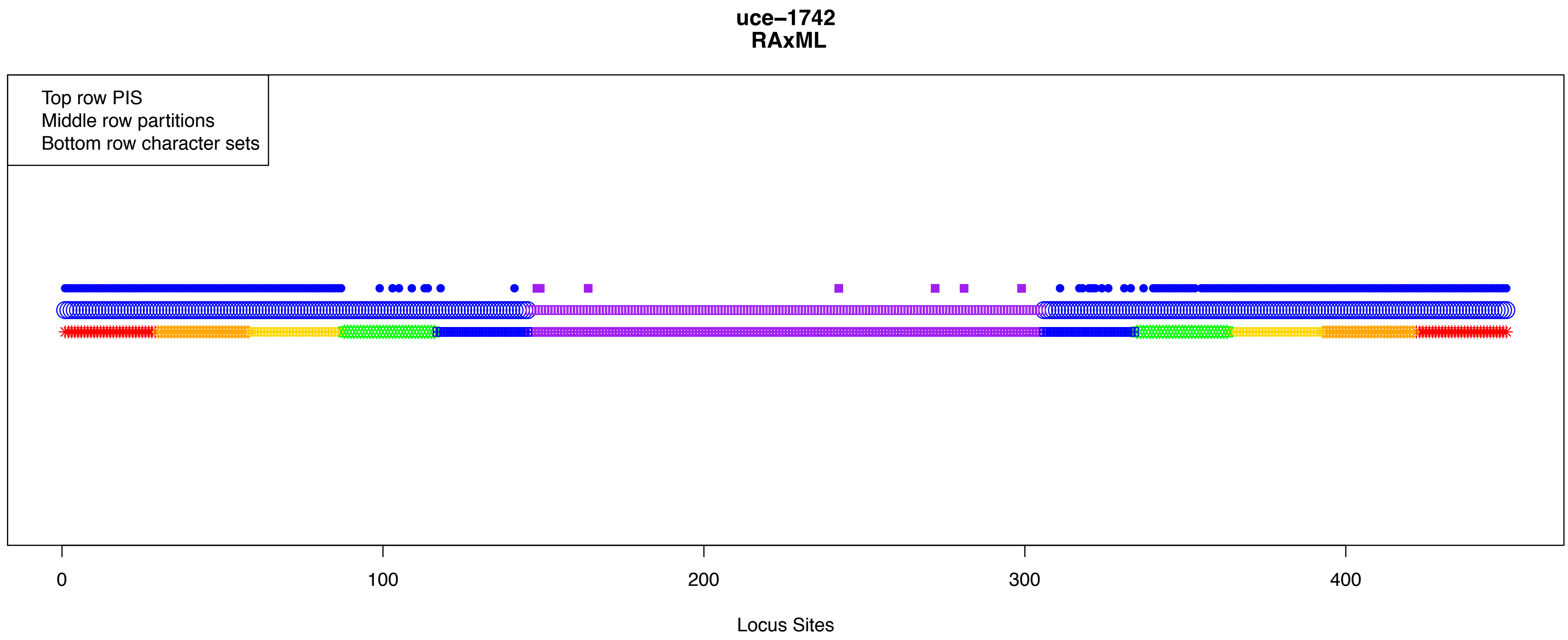
uce–1742
RAxML
Top row PIS
Middle row partitions
Bottom row character sets
0
100
200
300
400
Locus Sites

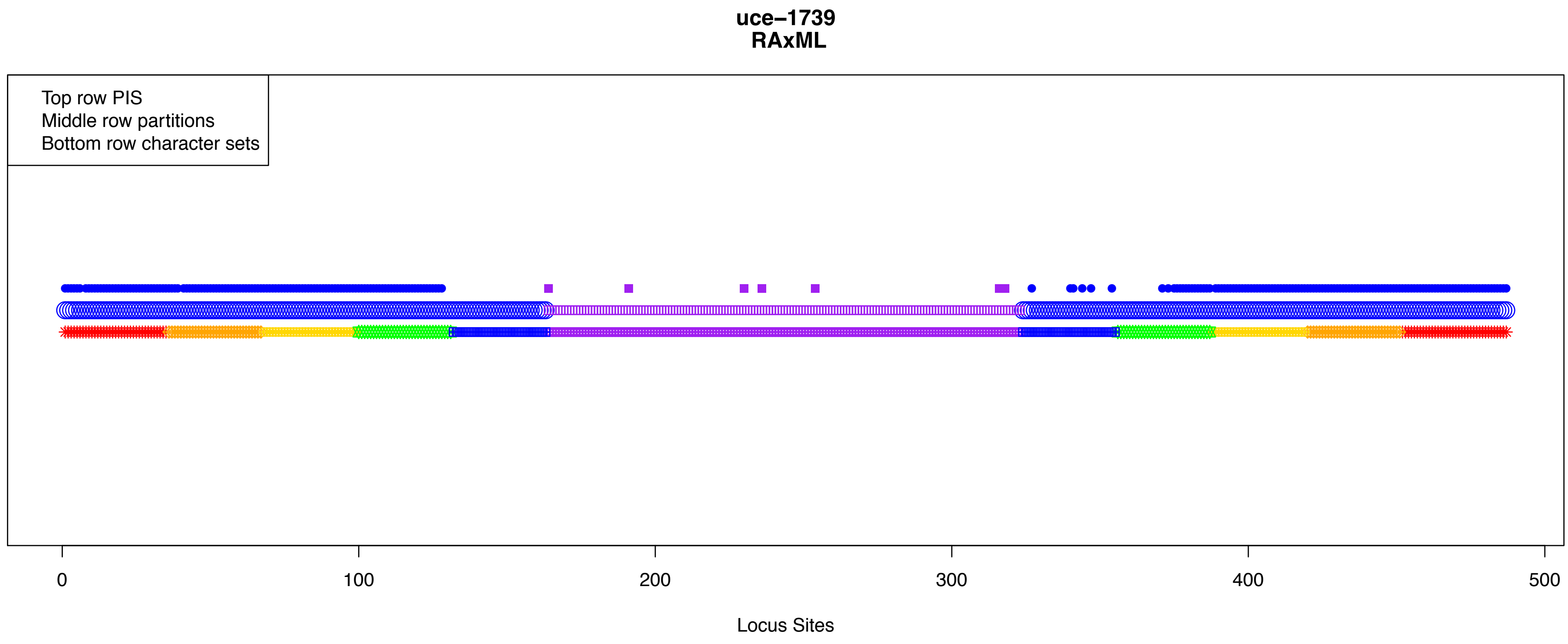
uce–1739
RAxML
Top row PIS
Middle row partitions
Bottom row character sets
0
100
200
300
400
500
Locus Sites

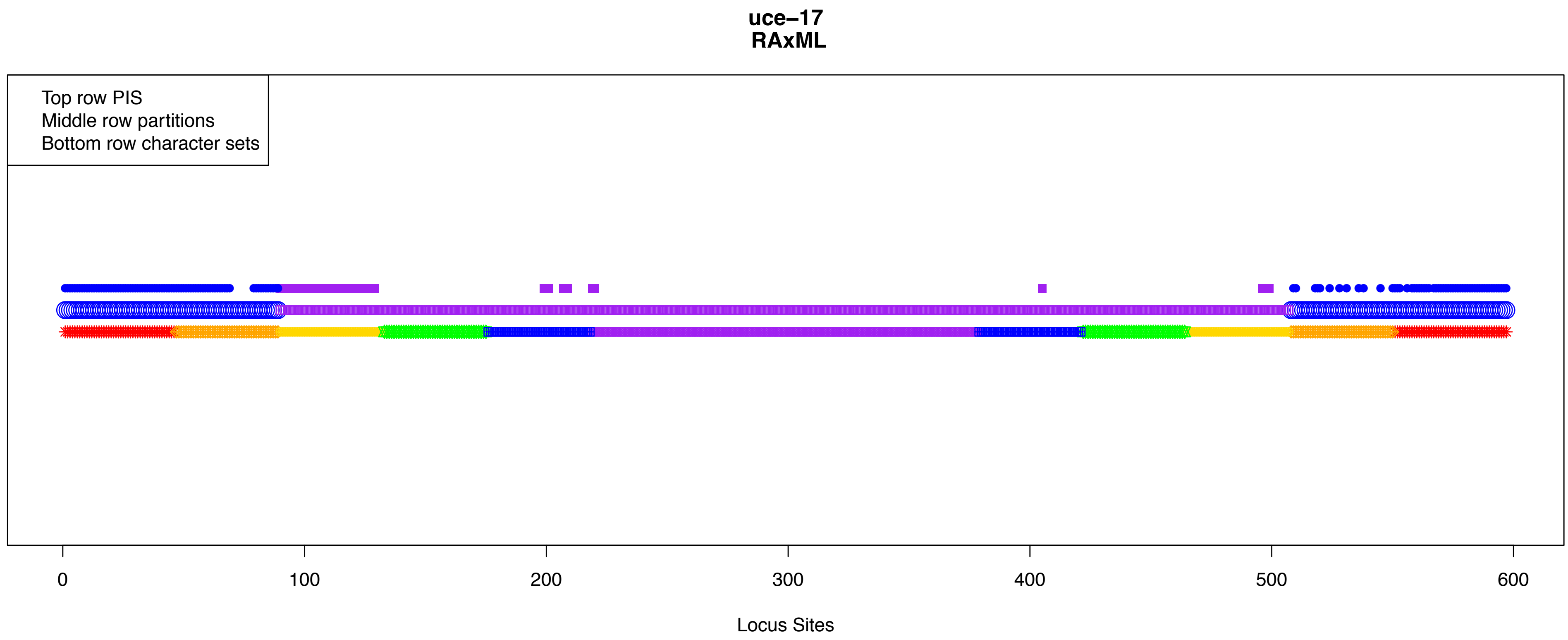

uce–17
RAxML
Top row PIS
Middle row partitions
Bottom row character sets
0
100
200
300
400
500
600
Locus Sites

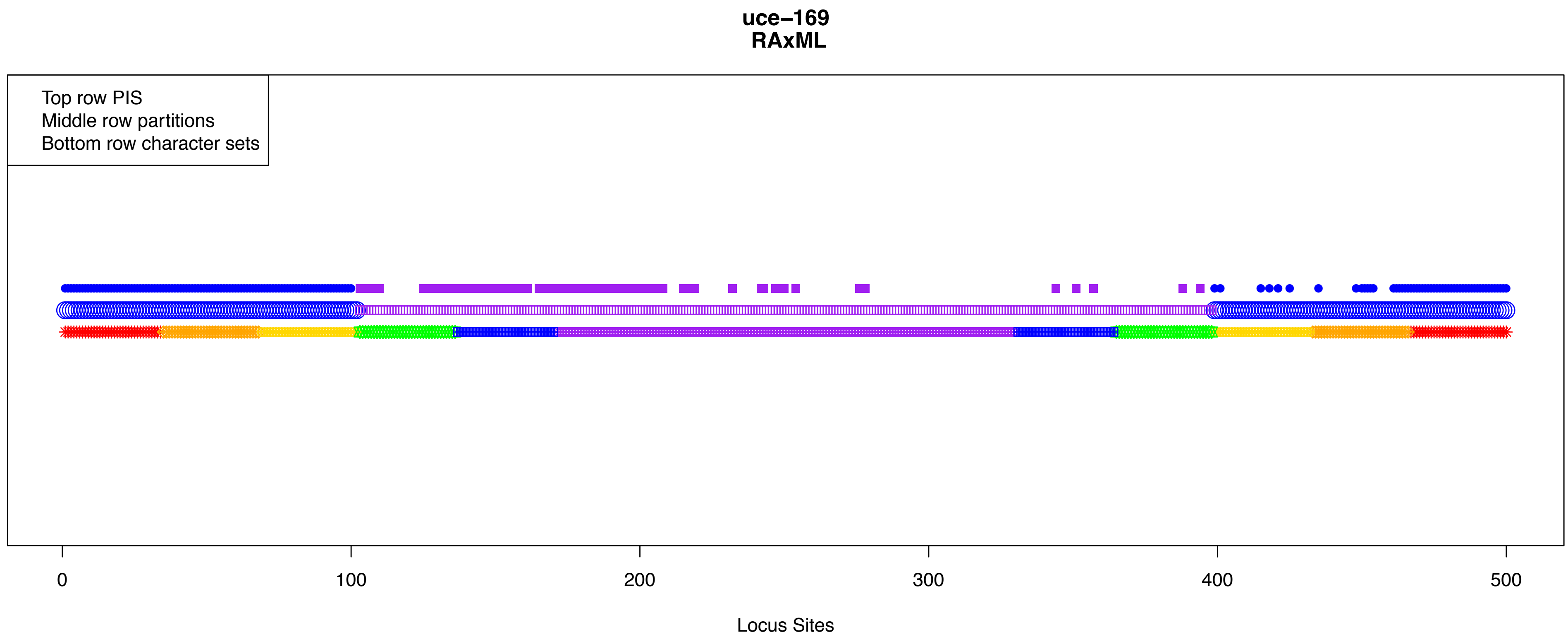
uce–169
RAxML
Top row PIS
Middle row partitions
Bottom row character sets
0
100
200
300
400
500
Locus Sites

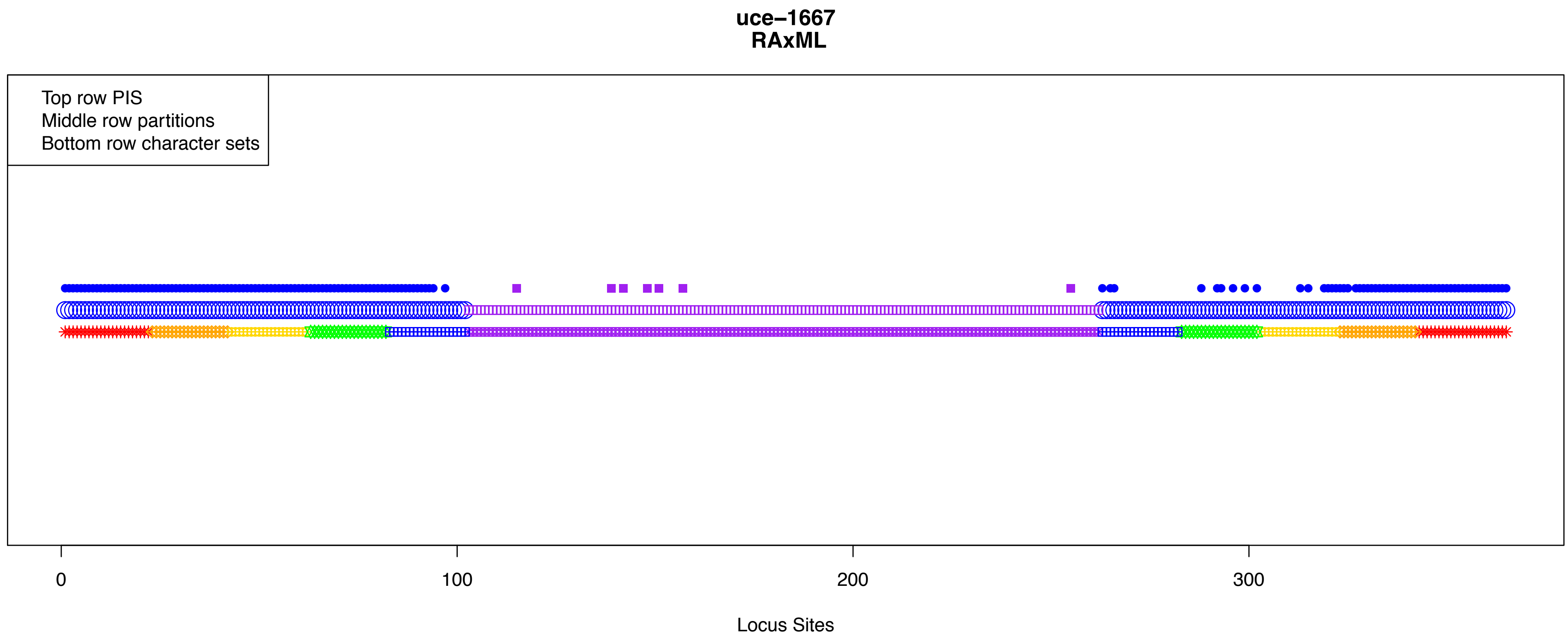
uce–1667
RAxML
Top row PIS
Middle row partitions
Bottom row character sets
0
100
200
300
Locus Sites

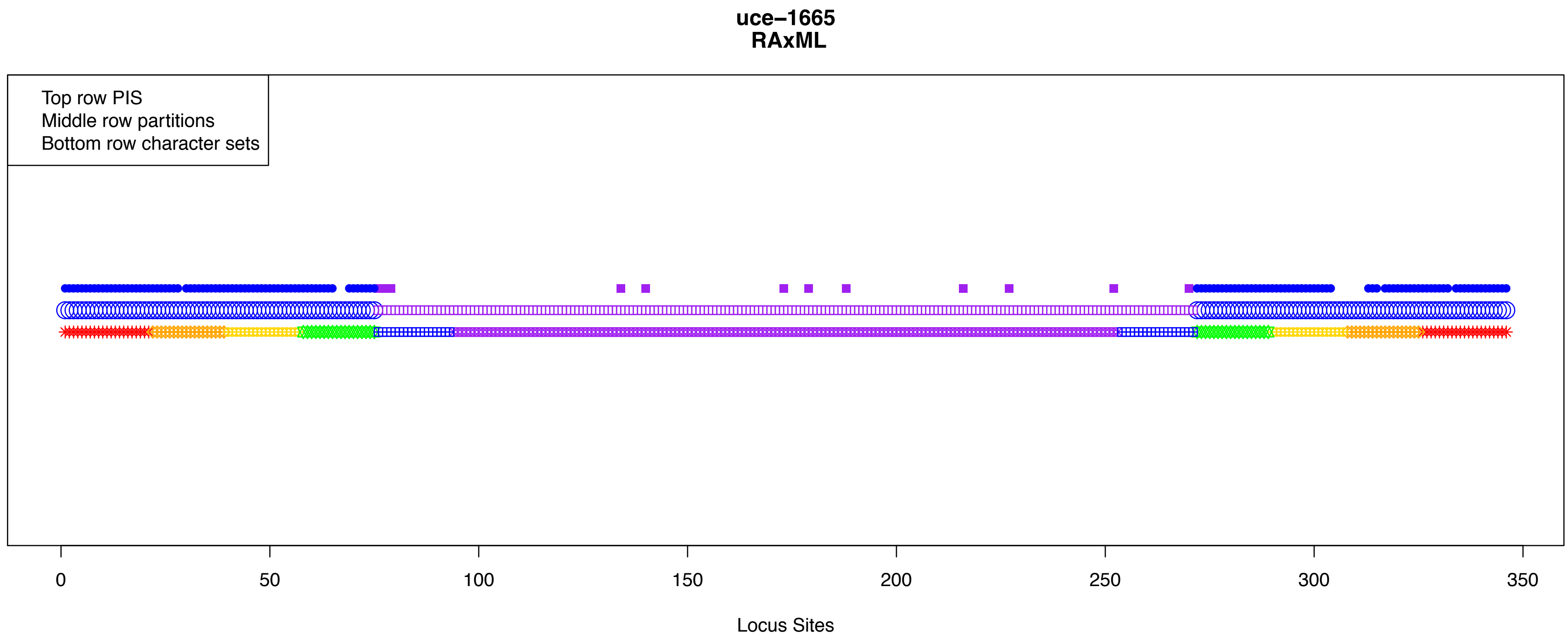
uce–1665
RAxML
Top row PIS
Middle row partitions
Bottom row character sets
0
50
100
150
200
250
300
350
Locus Sites

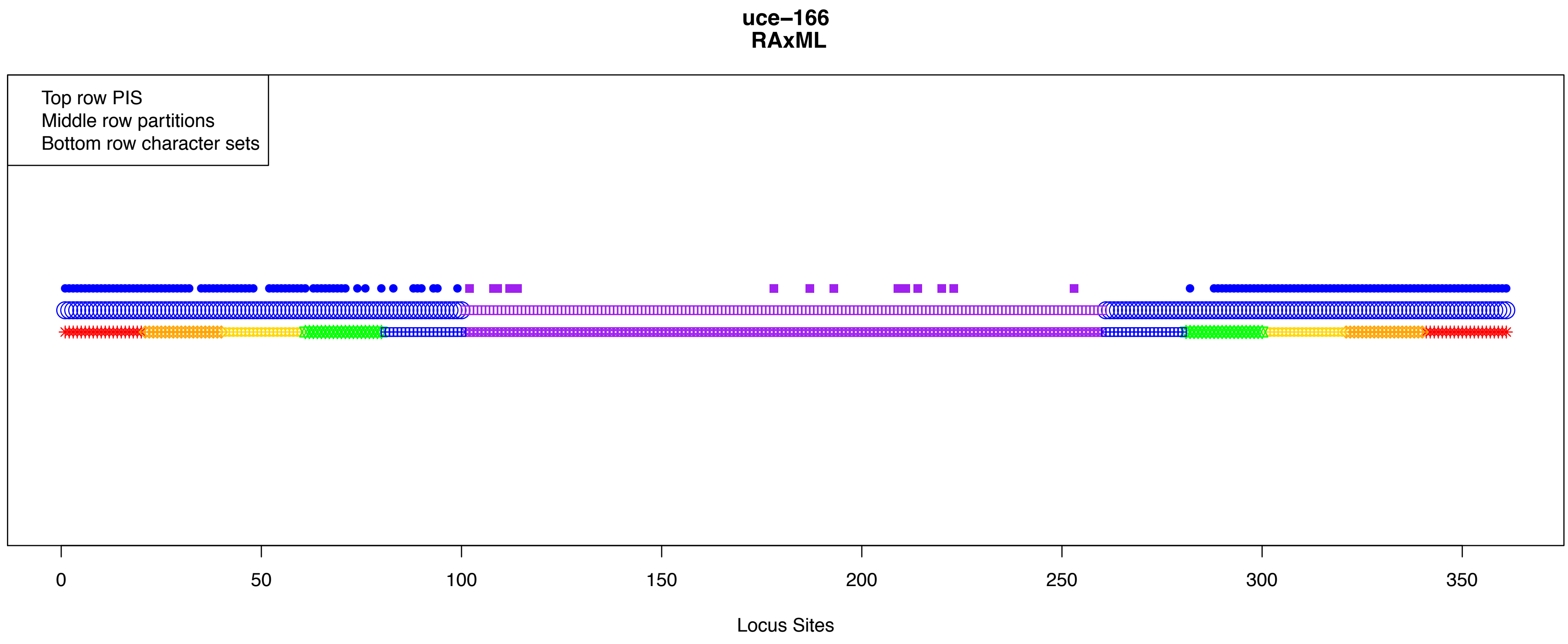
uce–166
RAxML
Top row PIS
Middle row partitions
Bottom row character sets
0
50
100
150
200
250
300
350
Locus Sites

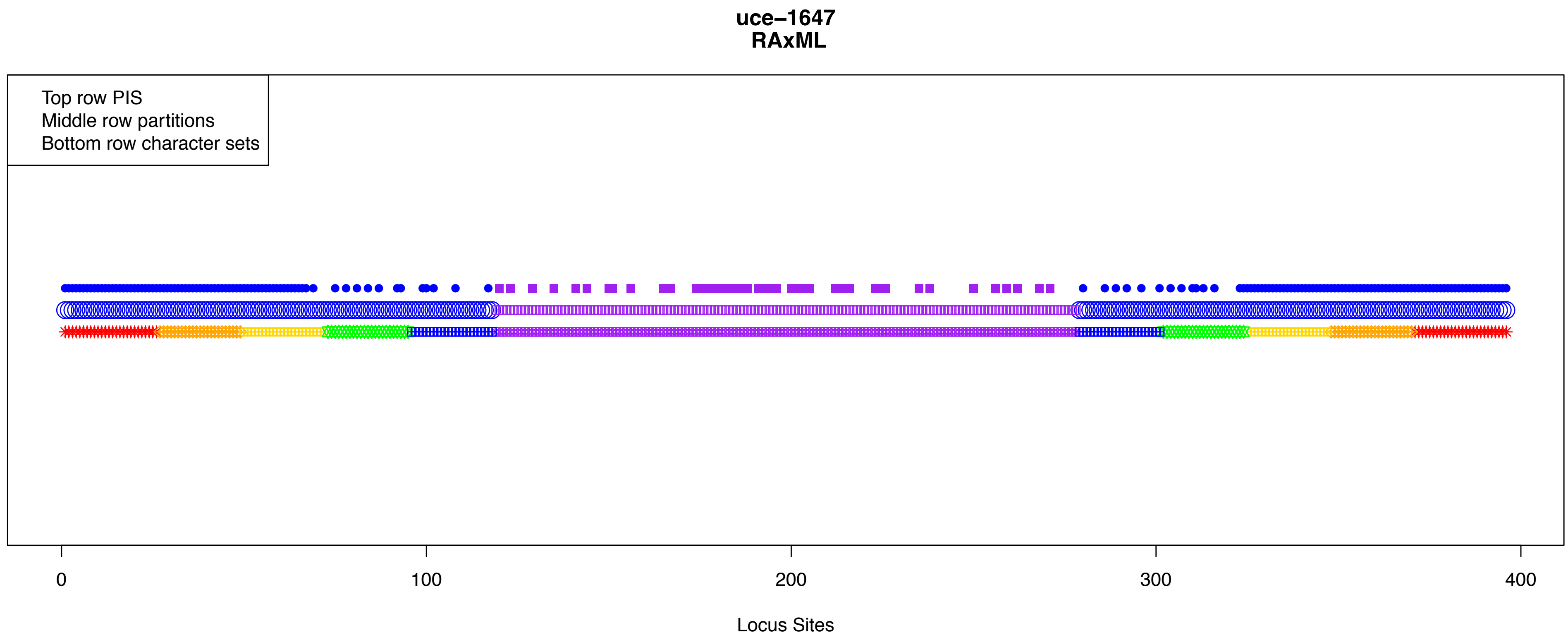
uce–1647
RAxML
Top row PIS
Middle row partitions
Bottom row character sets
0
100
200
300
400
Locus Sites

uce–1645
RAxML

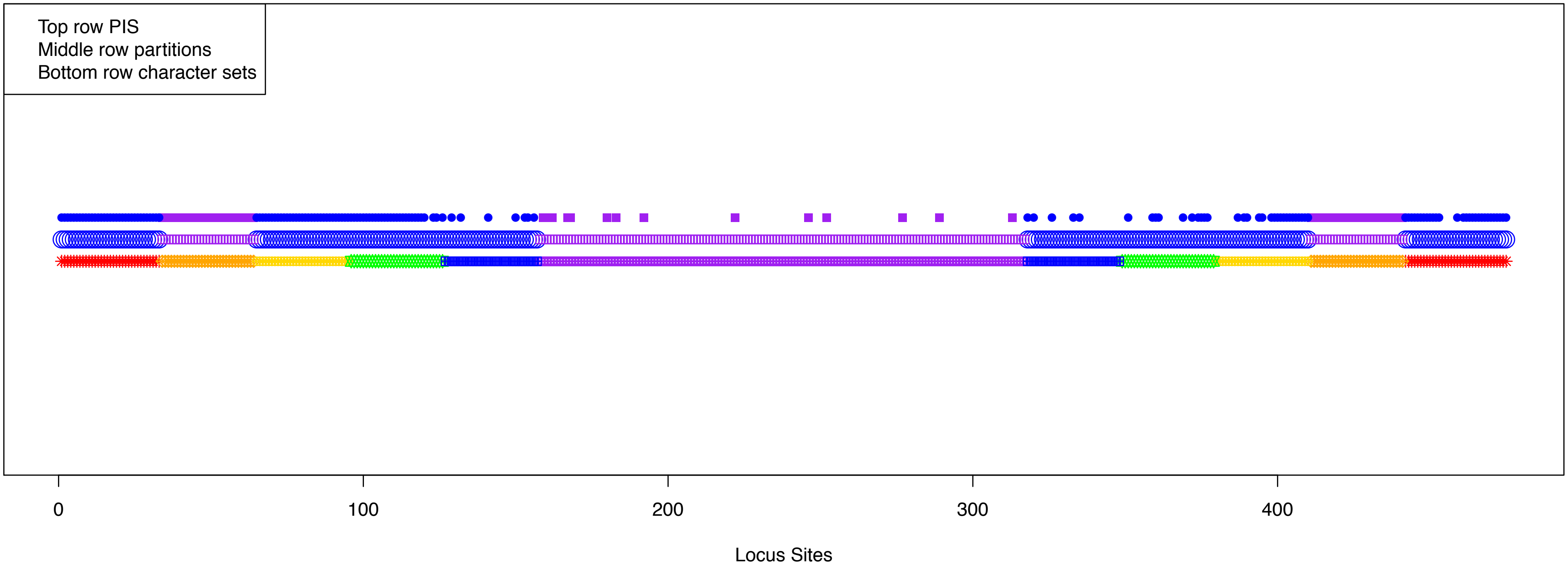

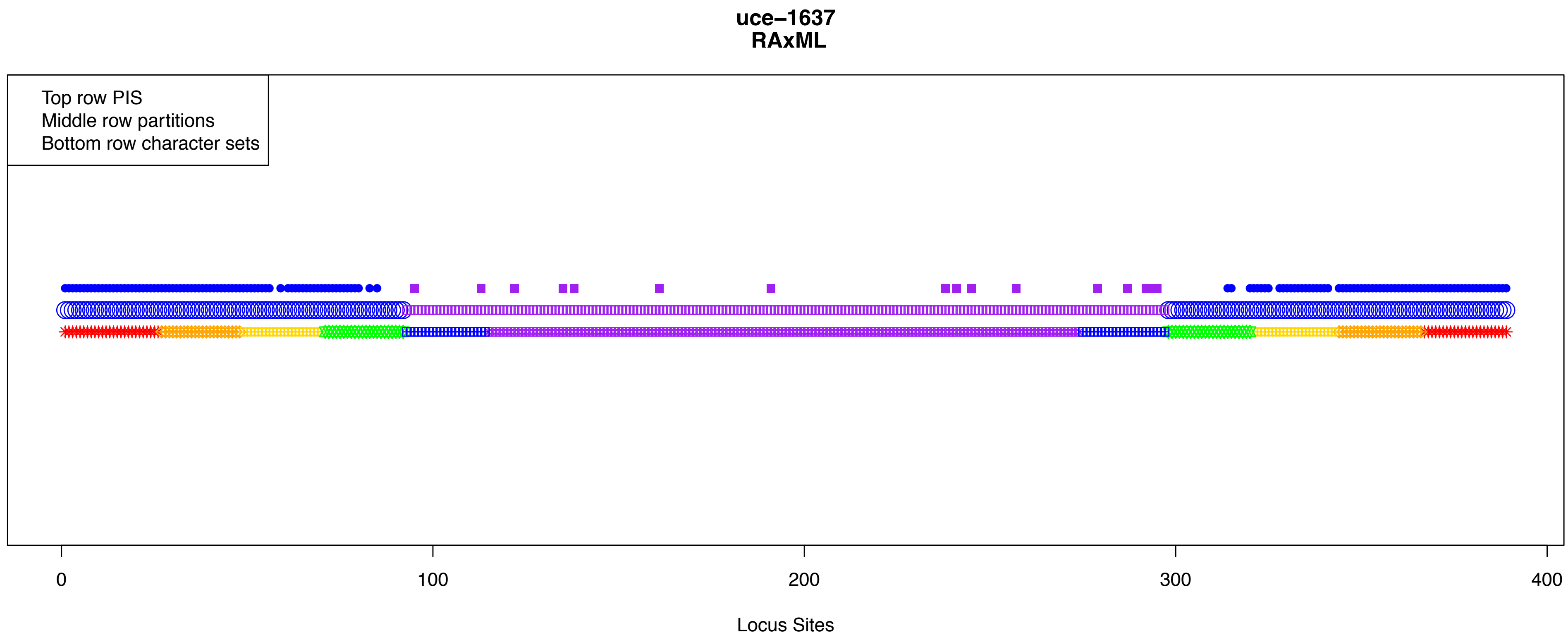
uce–1637
RAxML
Top row PIS
Middle row partitions
Bottom row character sets
0
100
200
300
400
Locus Sites

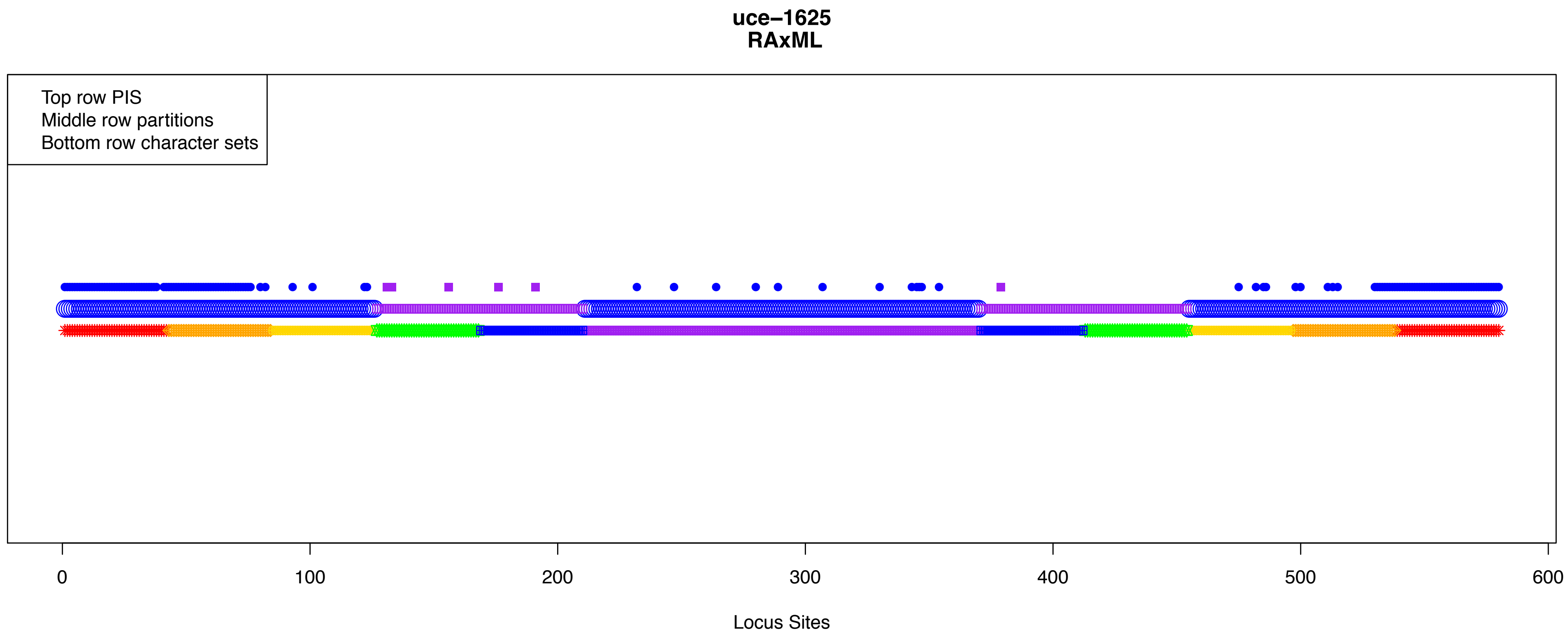

uce–1625
RAxML
Top row PIS
Middle row partitions
Bottom row character sets
0
100
200
300
400
500
600
Locus Sites

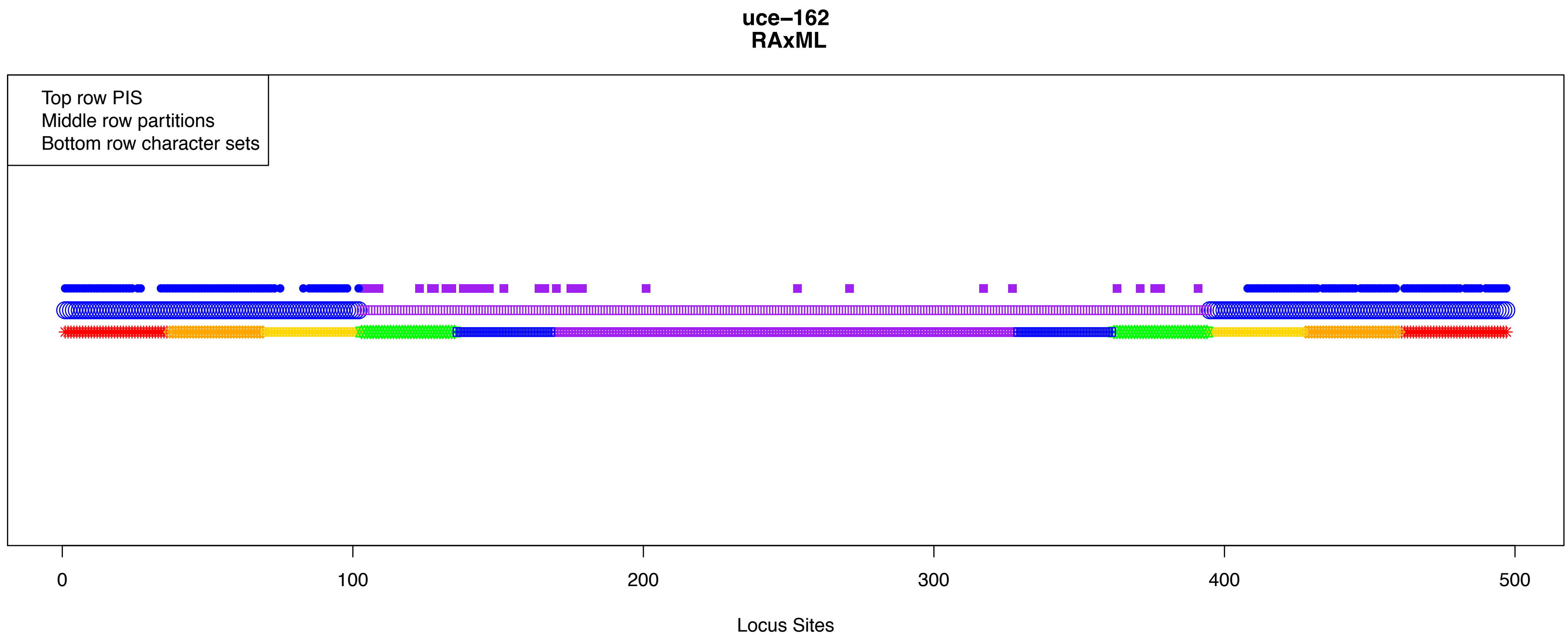
uce–162
RAxML
Top row PIS
Middle row partitions
Bottom row character sets
0
100
200
300
400
500
Locus Sites

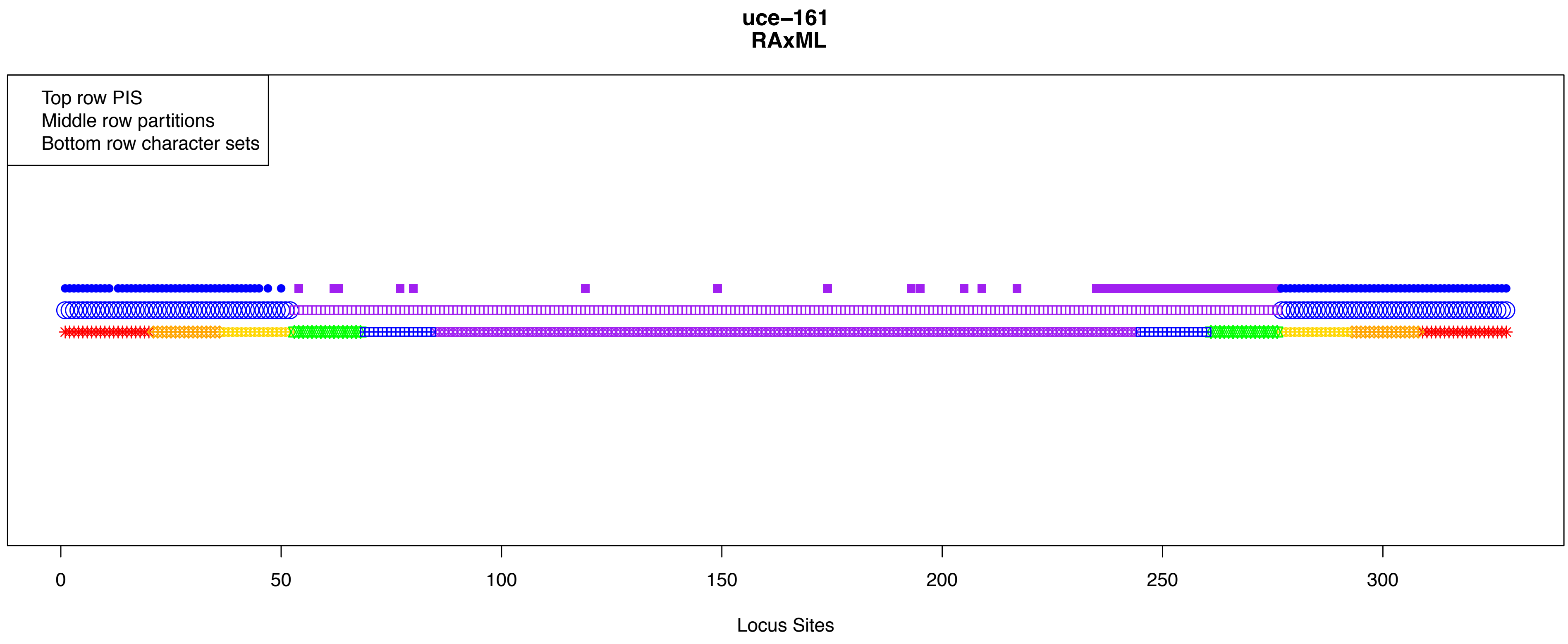
uce–161
RAxML
Top row PIS
Middle row partitions
Bottom row character sets
0
50
100
150
200
250
300
Locus Sites

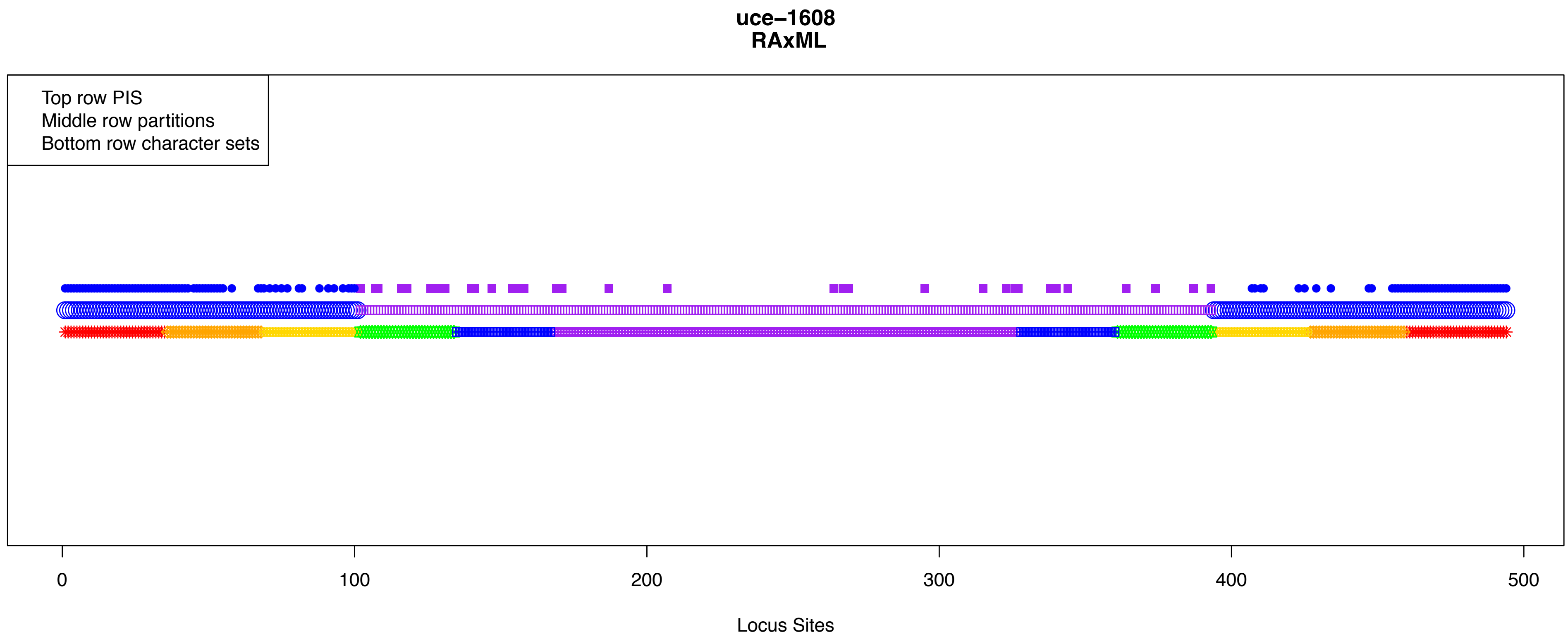
uce–1608
RAxML
Top row PIS
Middle row partitions
Bottom row character sets
0
100
200
300
400
500
Locus Sites

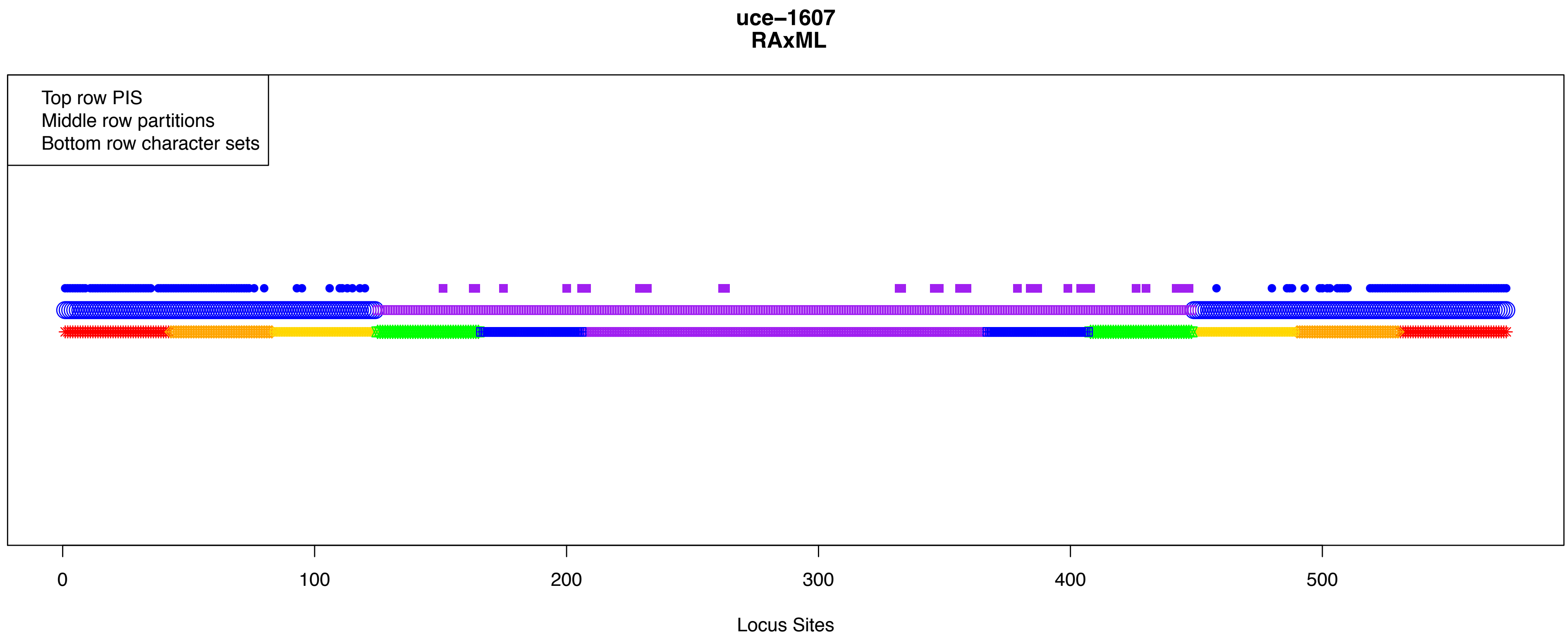
uce–1607
RAxML
Top row PIS
Middle row partitions
Bottom row character sets
0
100
200
300
400
500
Locus Sites

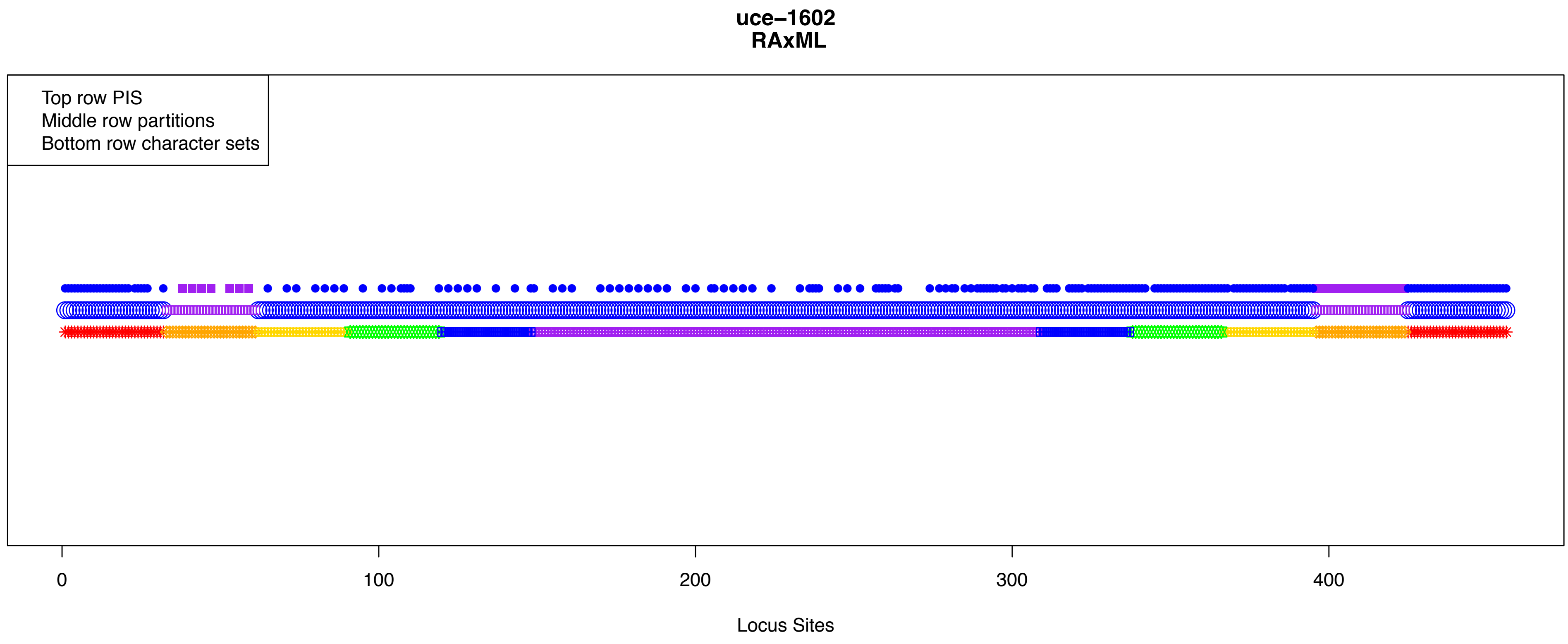
uce–1602
RAxML
Top row PIS
Middle row partitions
Bottom row character sets
0
100
200
300
400
Locus Sites

uce–1588
RAxML

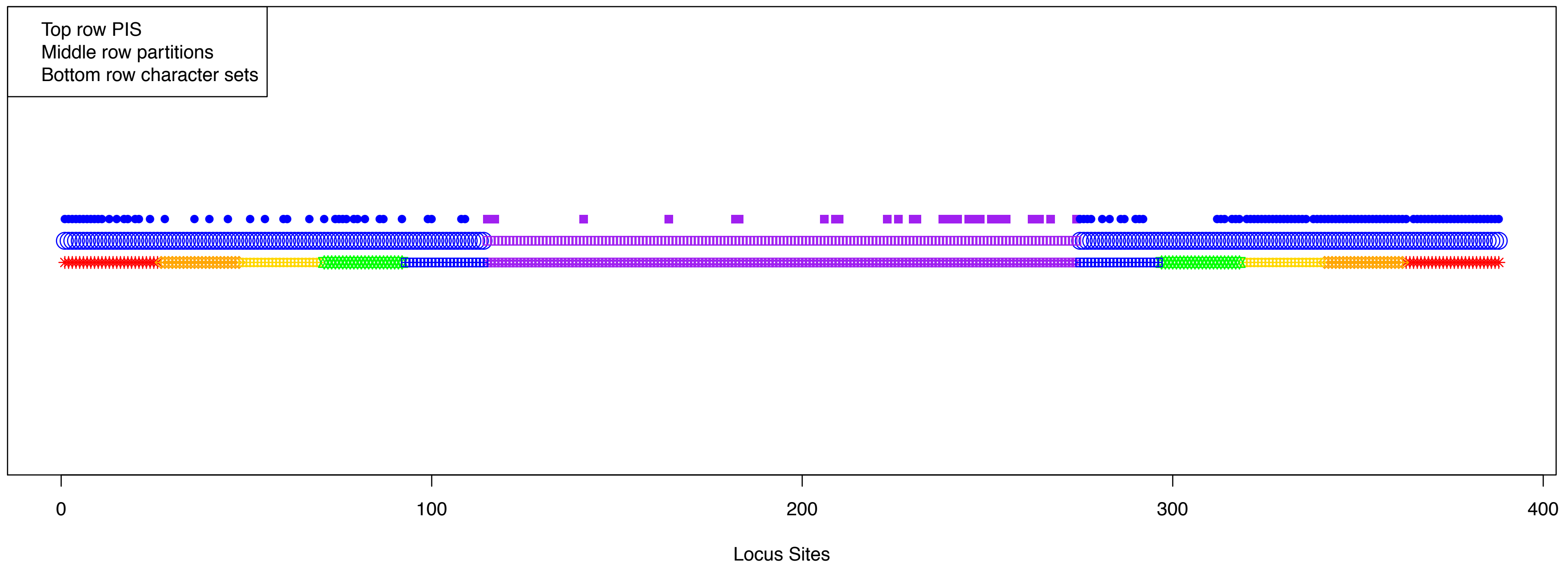

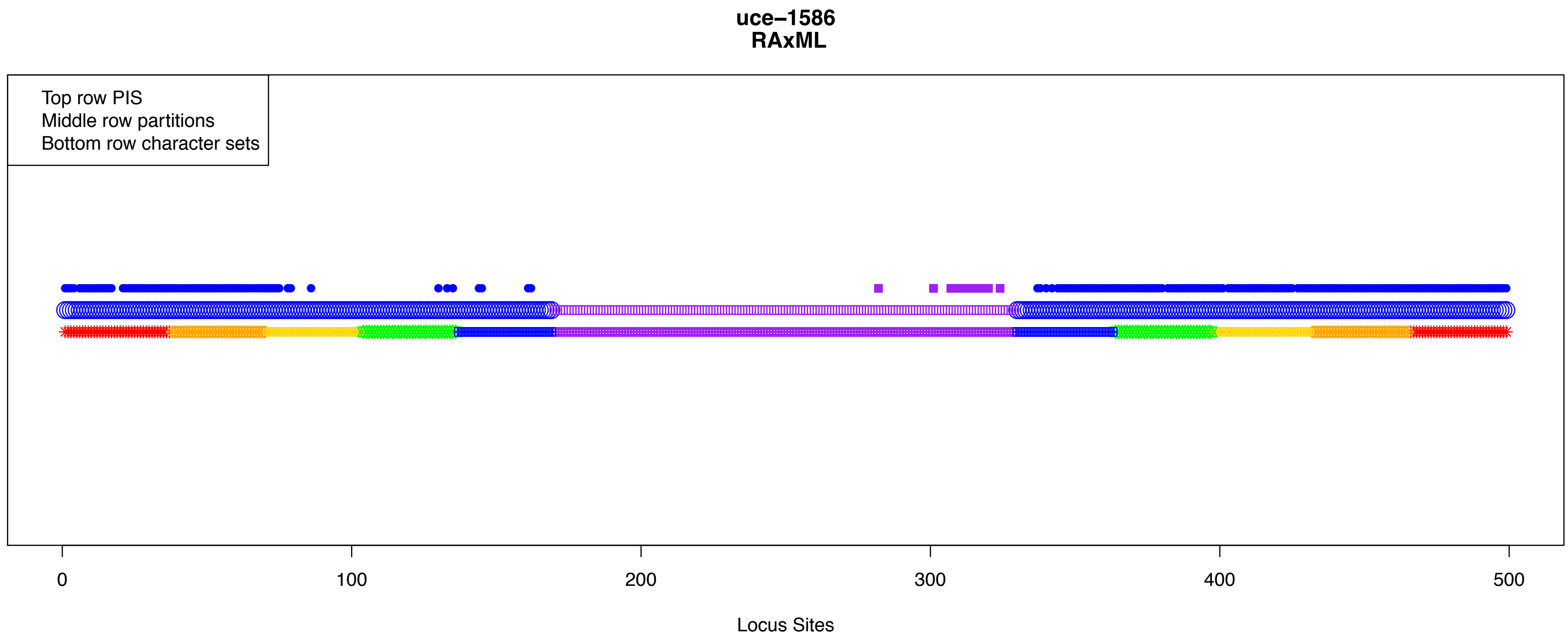
uce–1586
RAxML
Top row PIS
Middle row partitions
Bottom row character sets
0
100
200
300
400
500
Locus Sites

uce–1583
RAxML

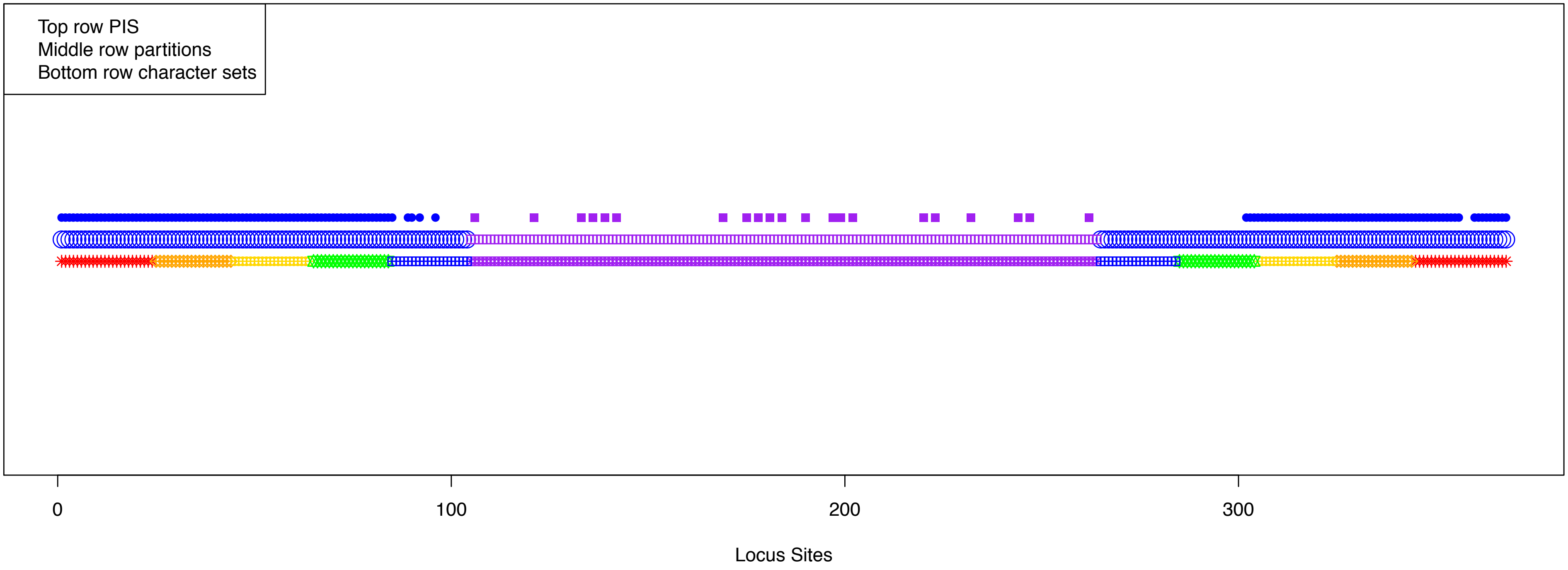

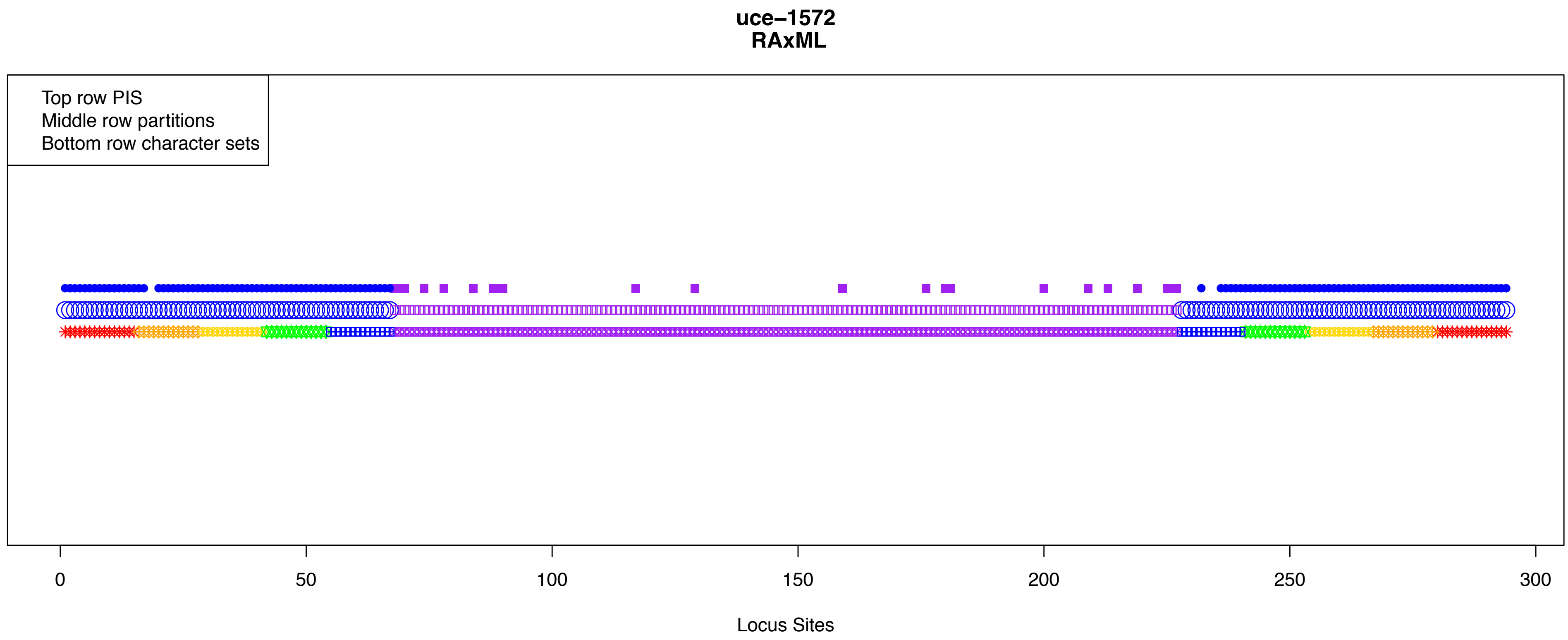
uce–1572
RAxML
Top row PIS
Middle row partitions
Bottom row character sets
0
50
100
150
200
250
300
Locus Sites

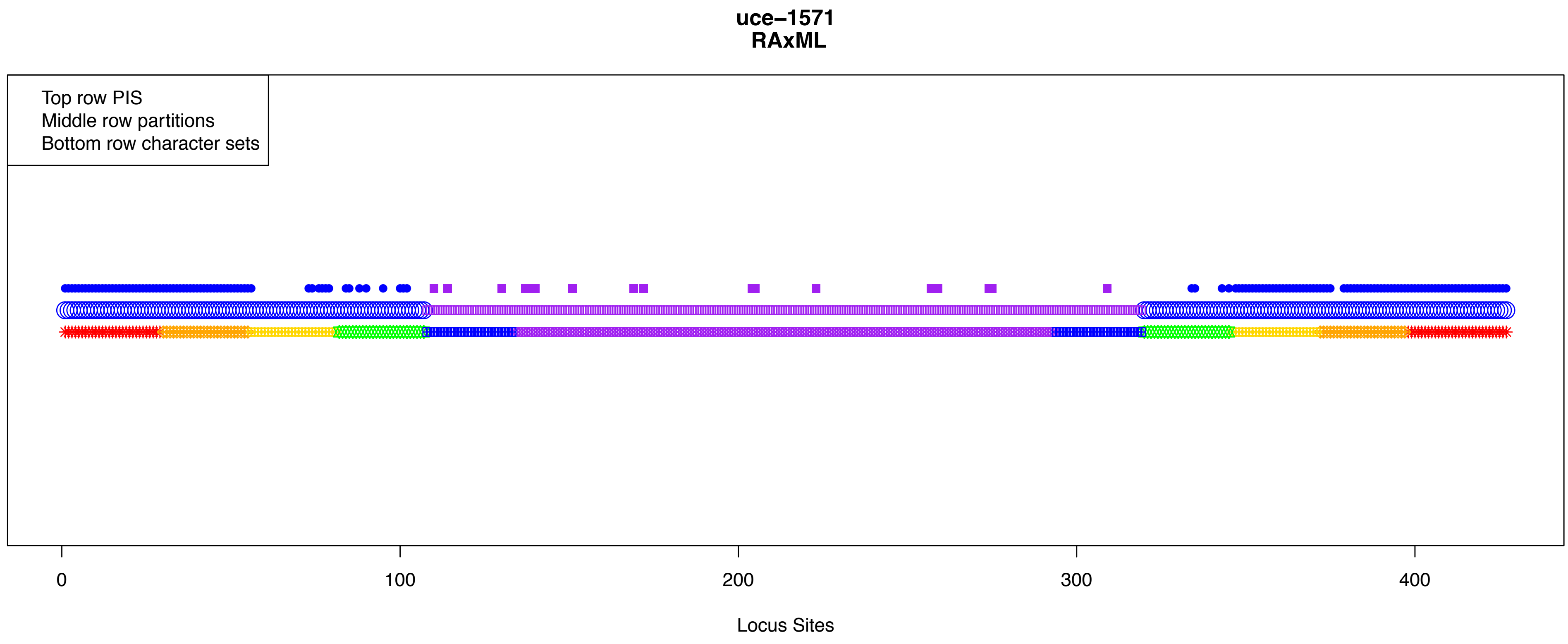
uce−1571
RAxML
Top row PIS
Middle row partitions
Bottom row character sets
0
100
200
300
400
Locus Sites

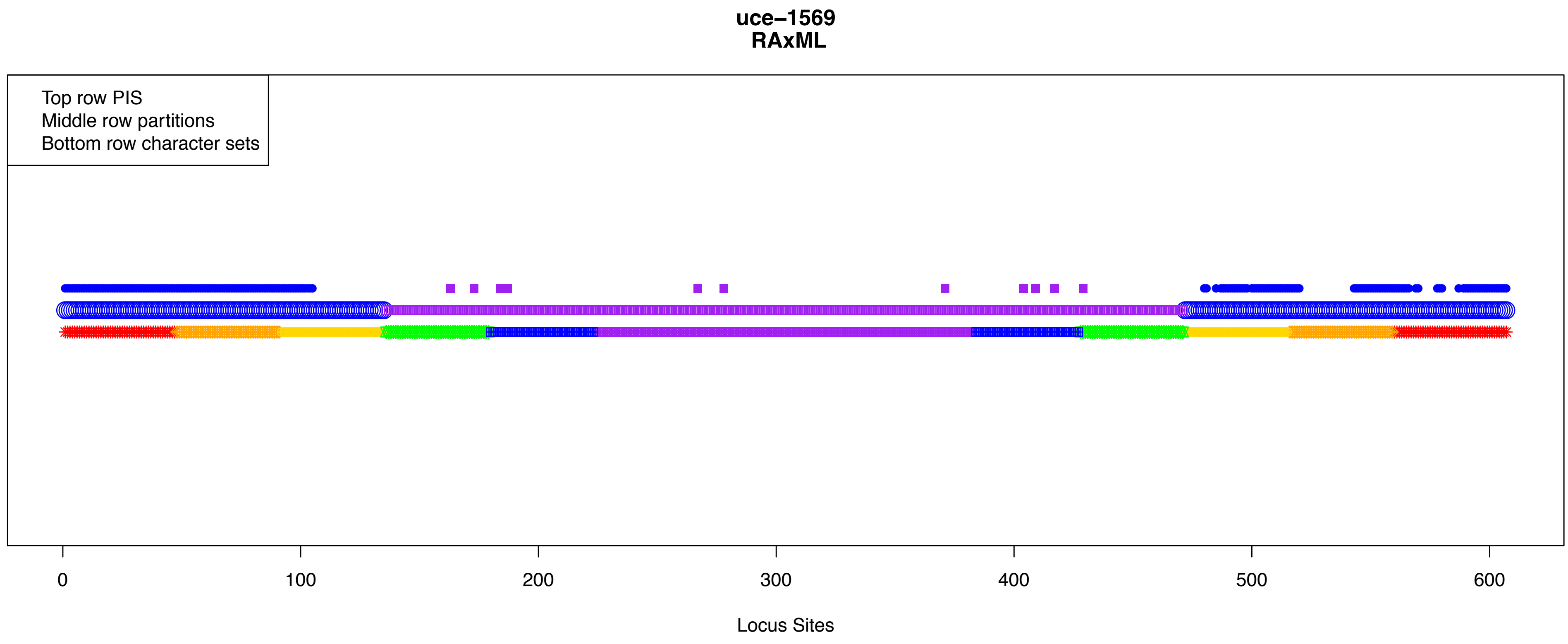
uce–1569
RAxML
Top row PIS
Middle row partitions
Bottom row character sets
0
100
200
300
400
500
600
Locus Sites

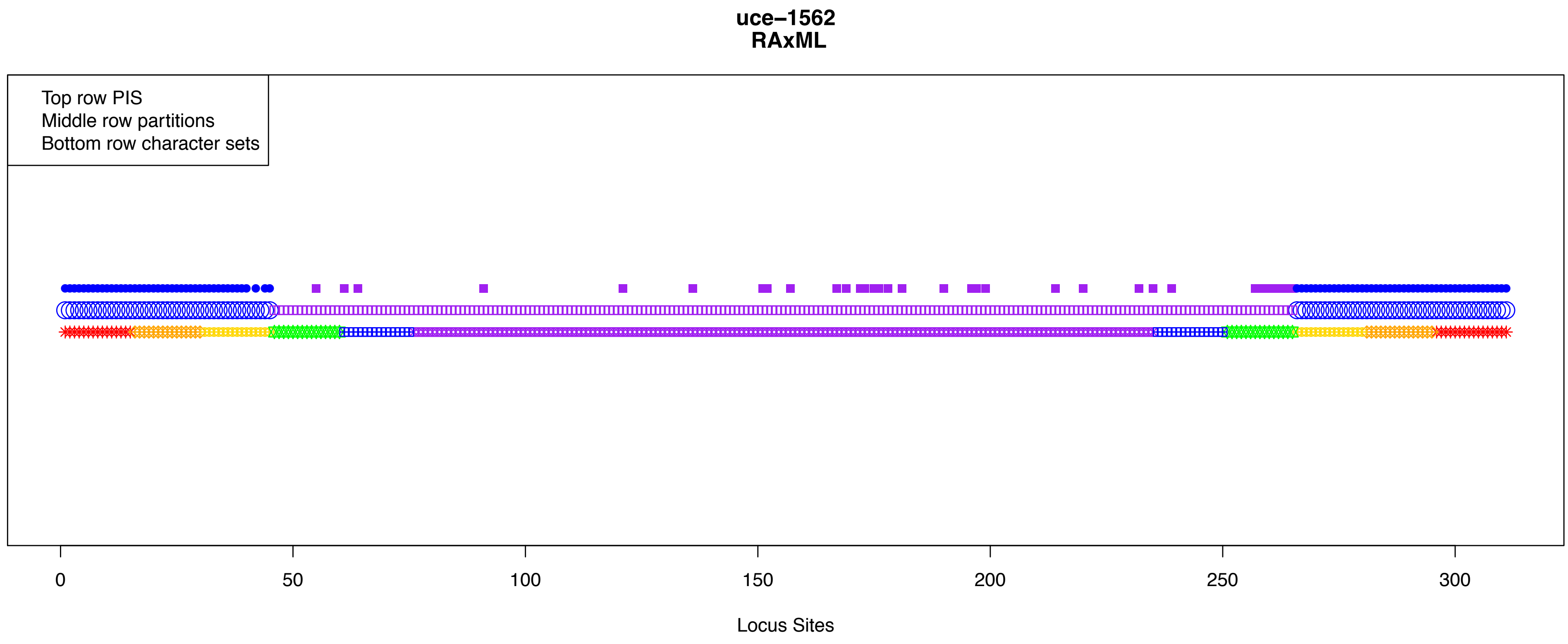
uce–1562
RAxML
Top row PIS
Middle row partitions
Bottom row character sets
0
50
100
150
200
250
300
Locus Sites

uce–1551
RAxML

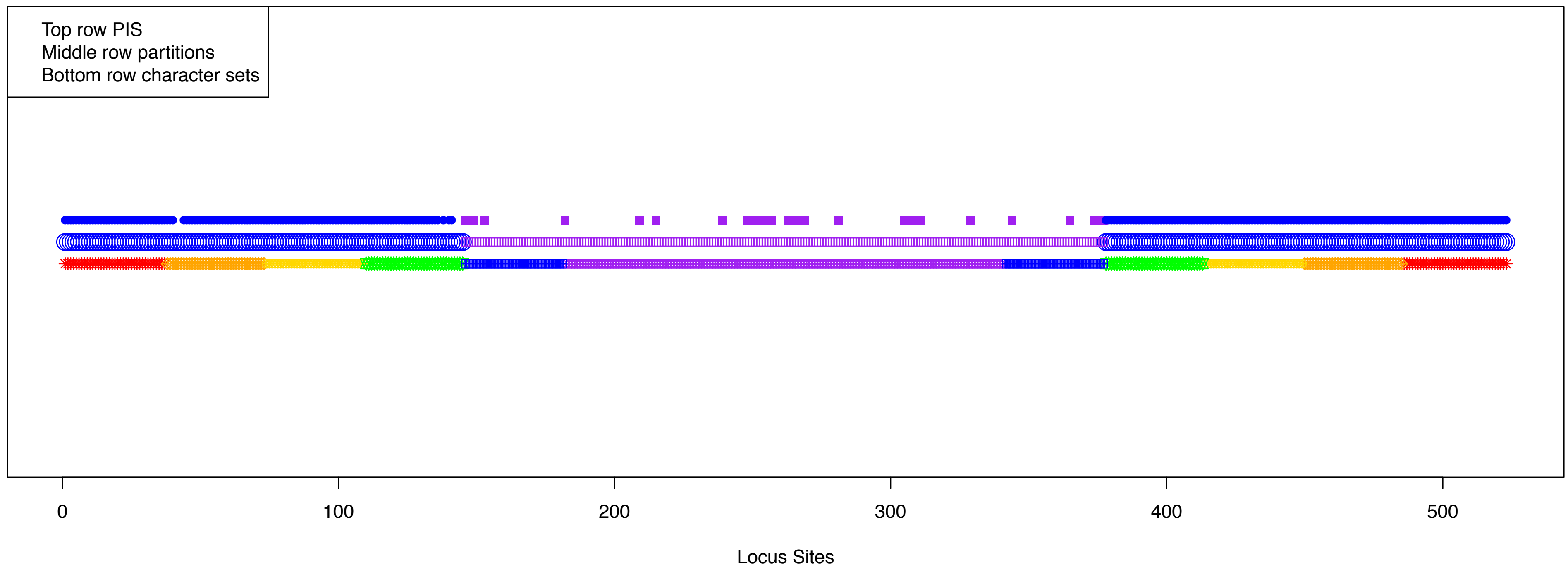

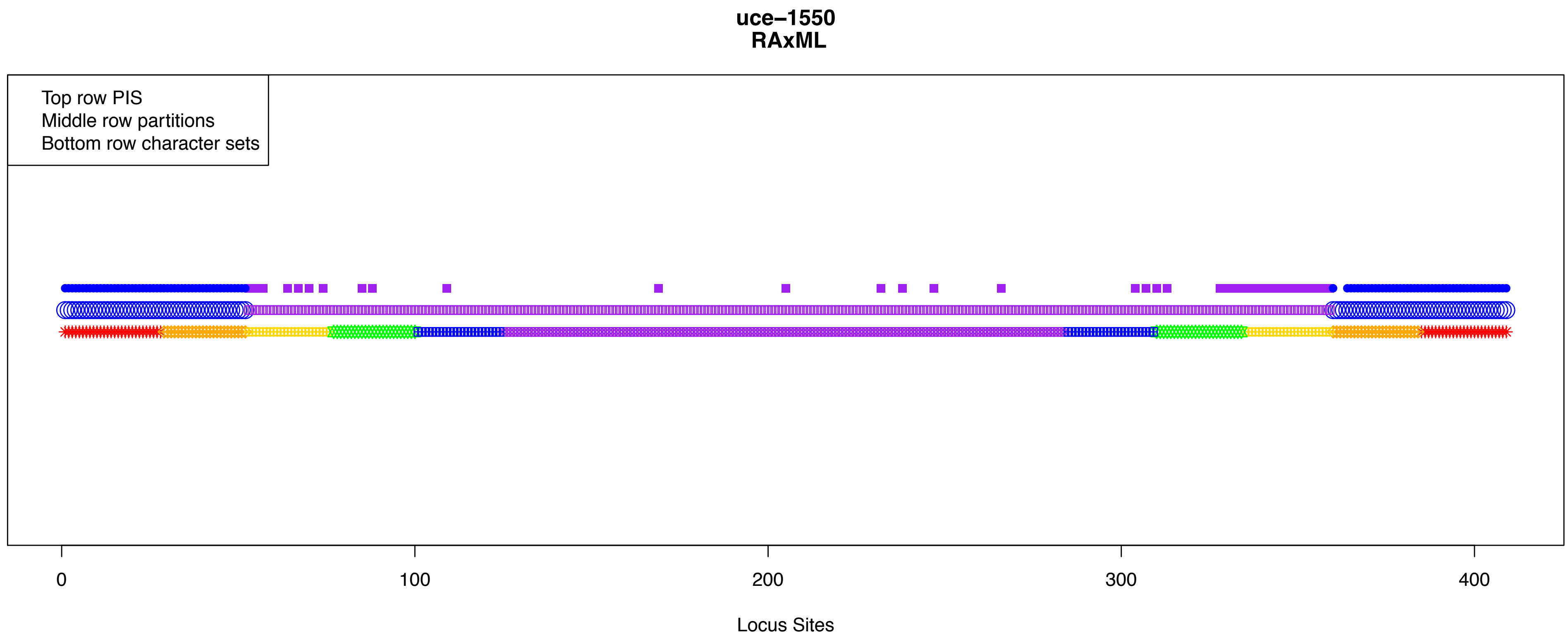
uce–1550
RAxML
Top row PIS
Middle row partitions
Bottom row character sets
0
100
200
300
400
Locus Sites

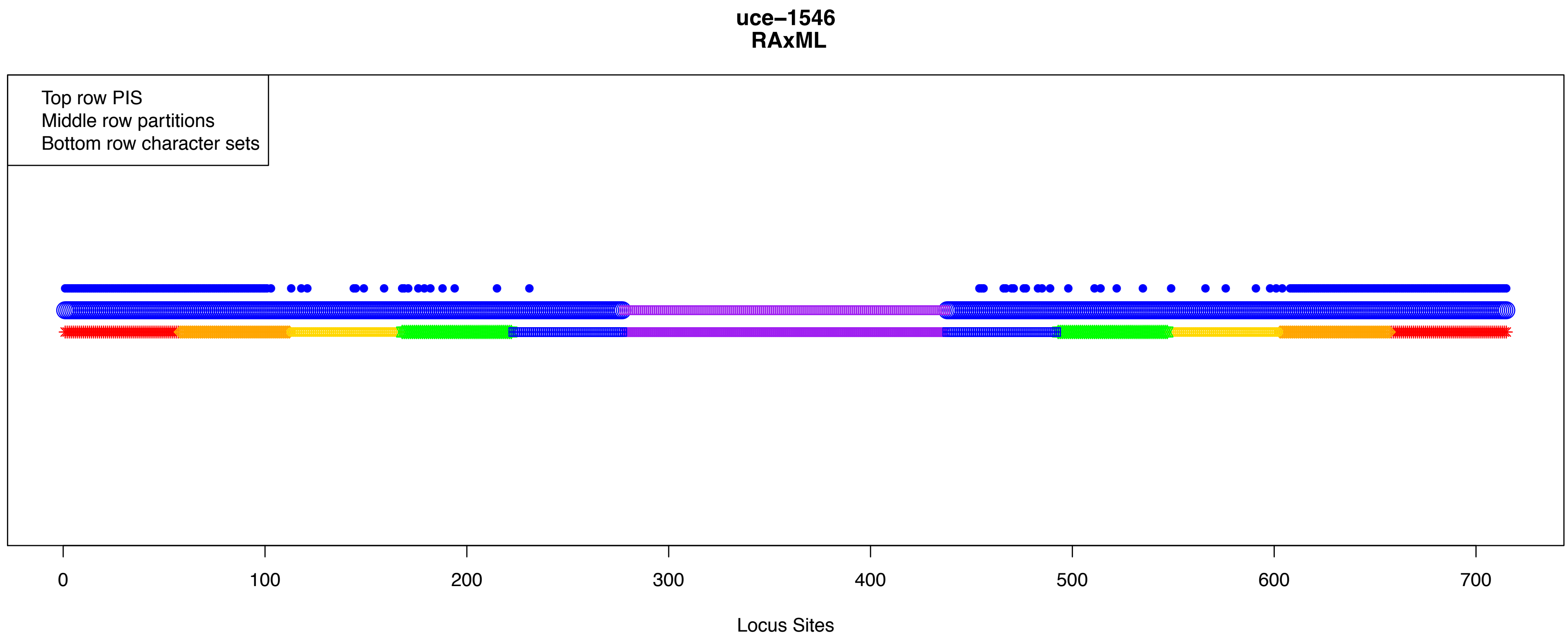
uce–1546
RAxML
Top row PIS
Middle row partitions
Bottom row character sets
0
100
200
300
400
500
600
700
Locus Sites

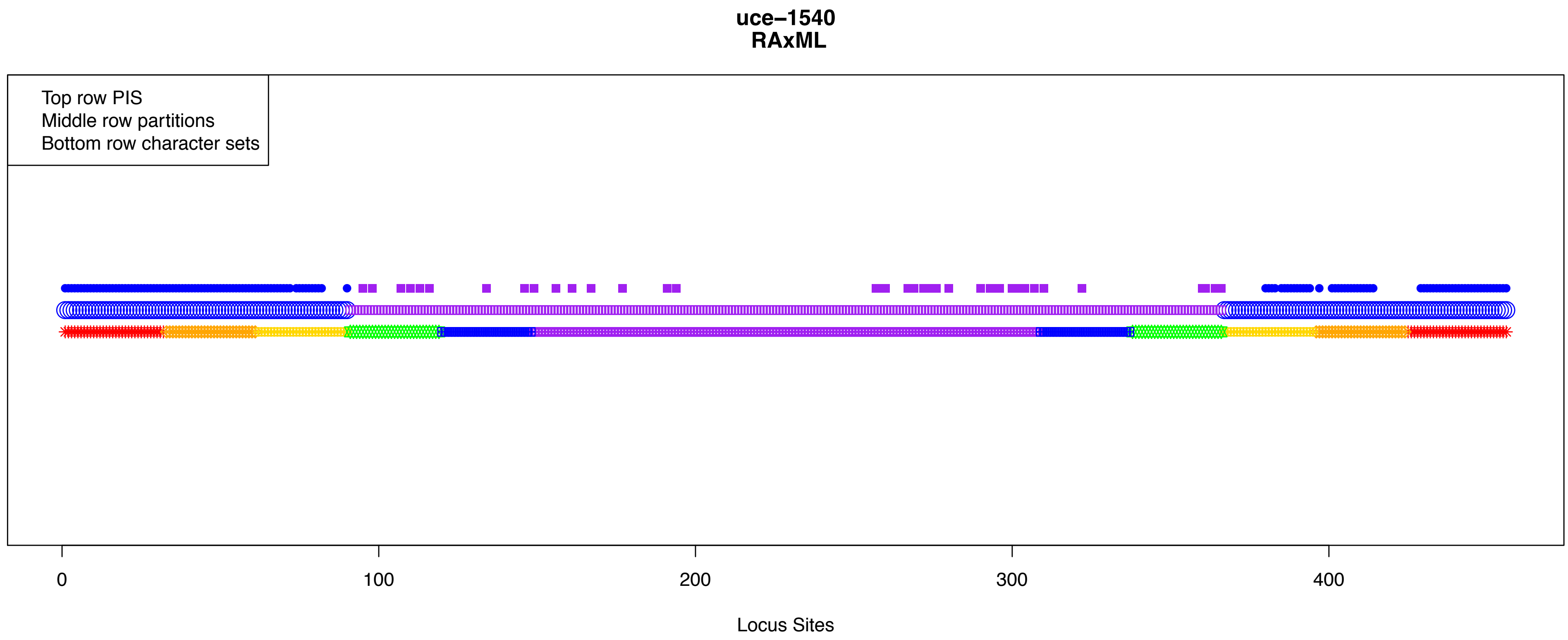

uce–1540
RAxML
Top row PIS
Middle row partitions
Bottom row character sets
0
100
200
300
400
Locus Sites

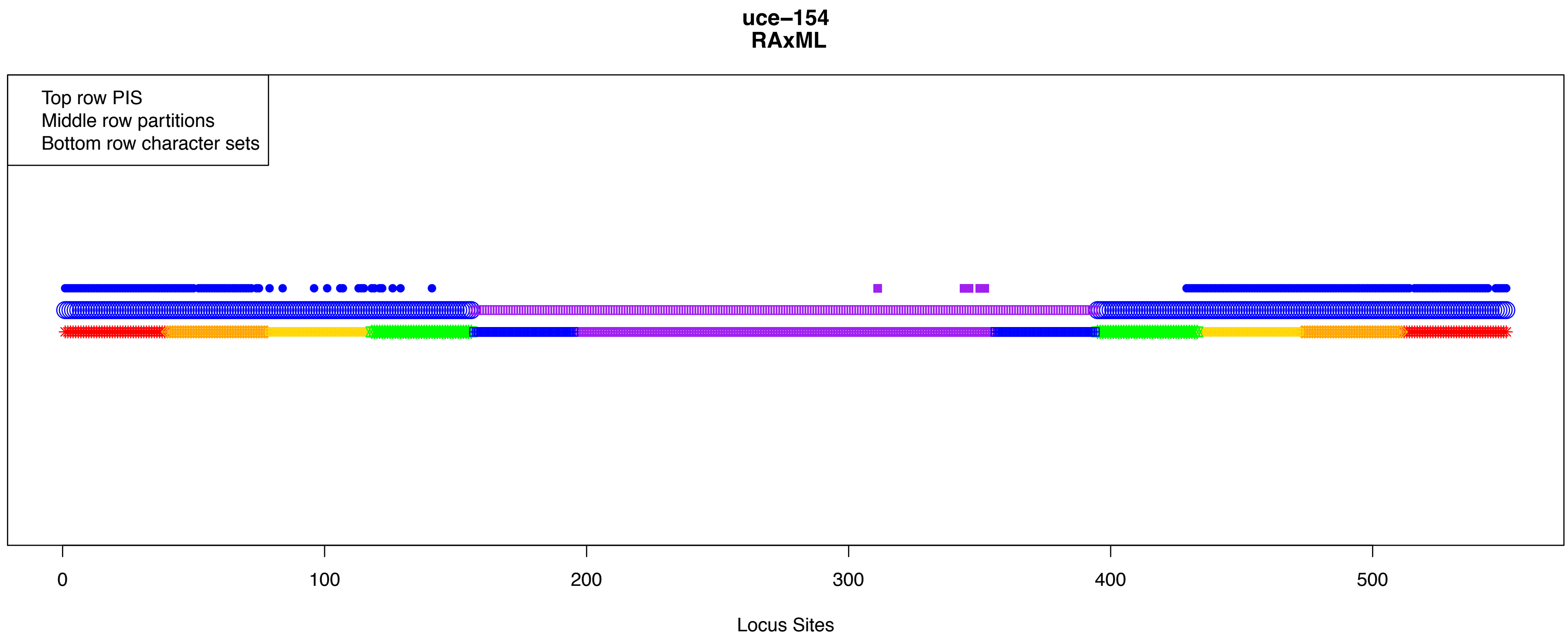

uce–154
RAxML
Top row PIS
Middle row partitions
Bottom row character sets
0
100
200
300
400
500
Locus Sites

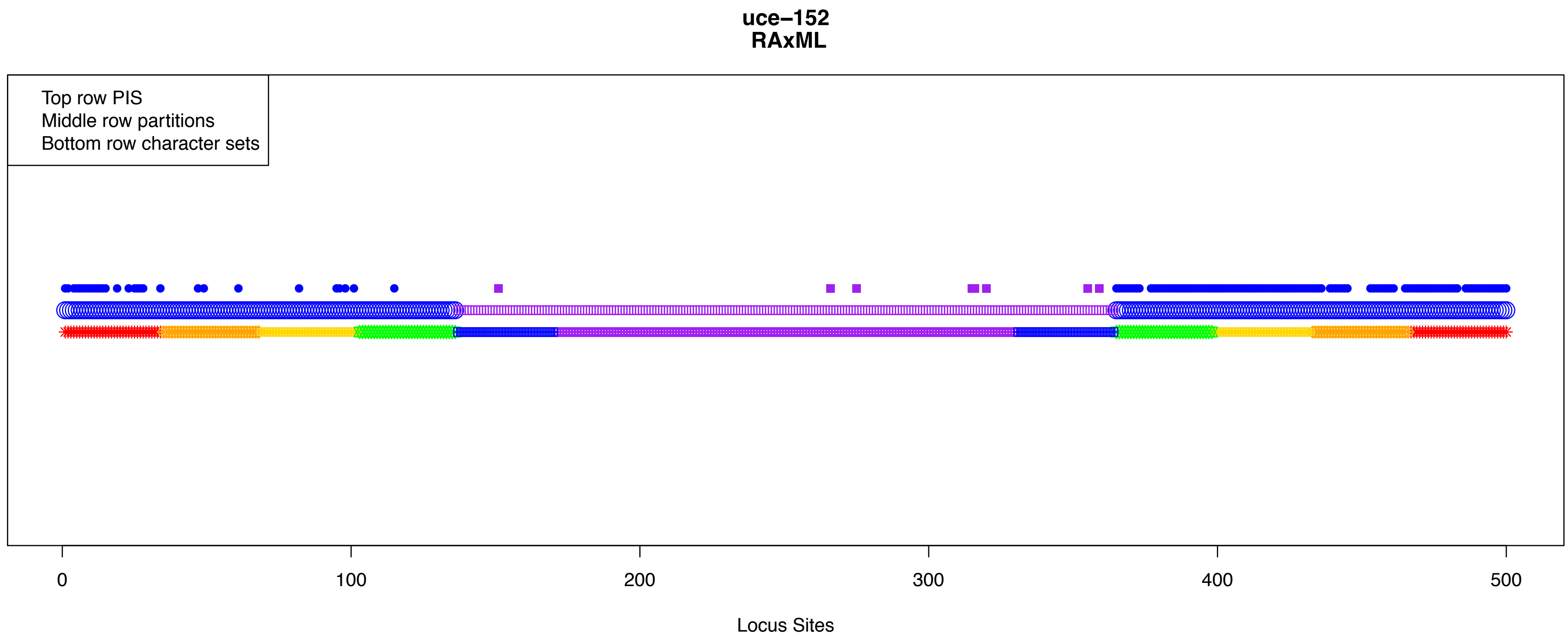
uce–152
RAxML
Top row PIS
Middle row partitions
Bottom row character sets
0
100
200
300
400
500
Locus Sites

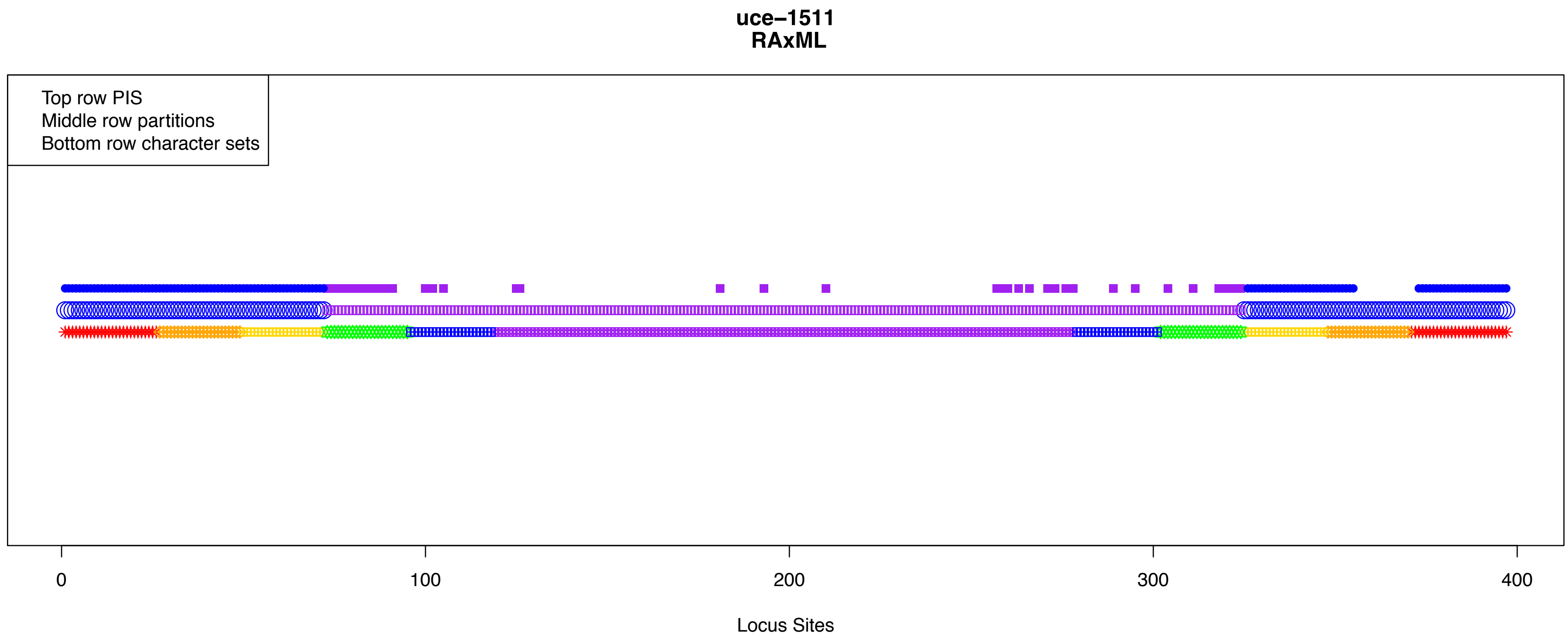
uce–1511
RAxML
Top row PIS
Middle row partitions
Bottom row character sets
0
100
200
300
400
Locus Sites

uce–1510
RAxML

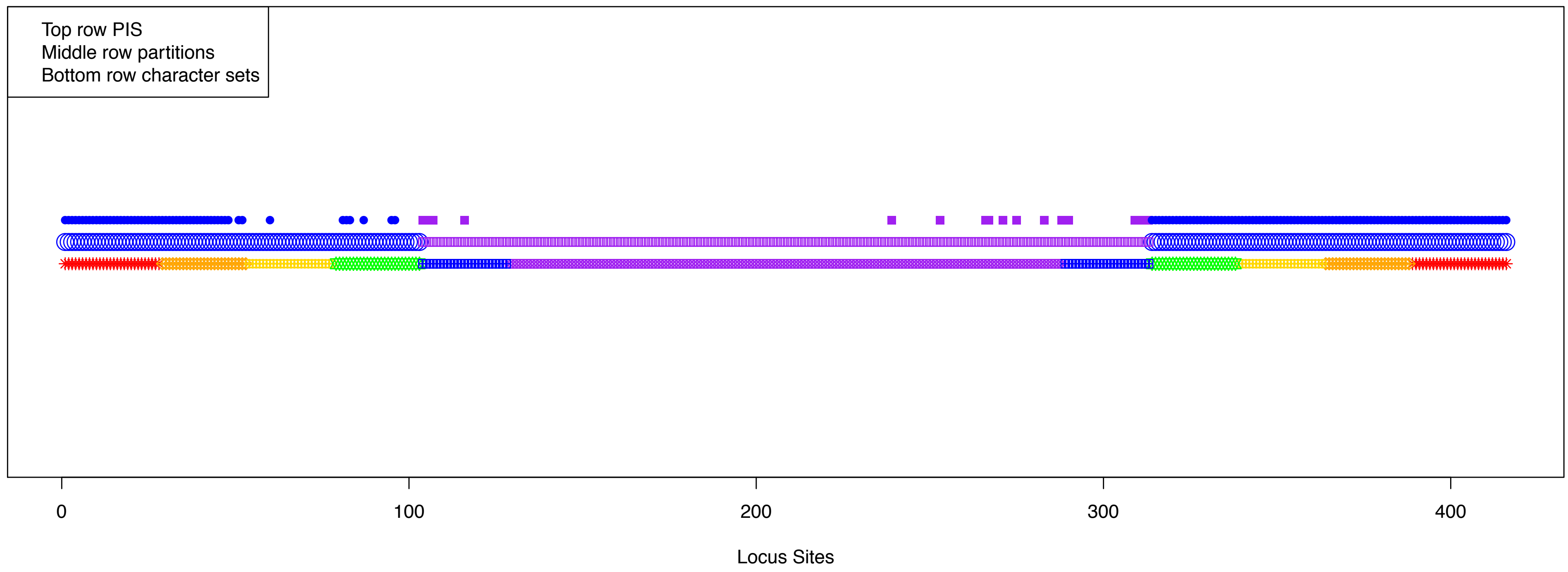

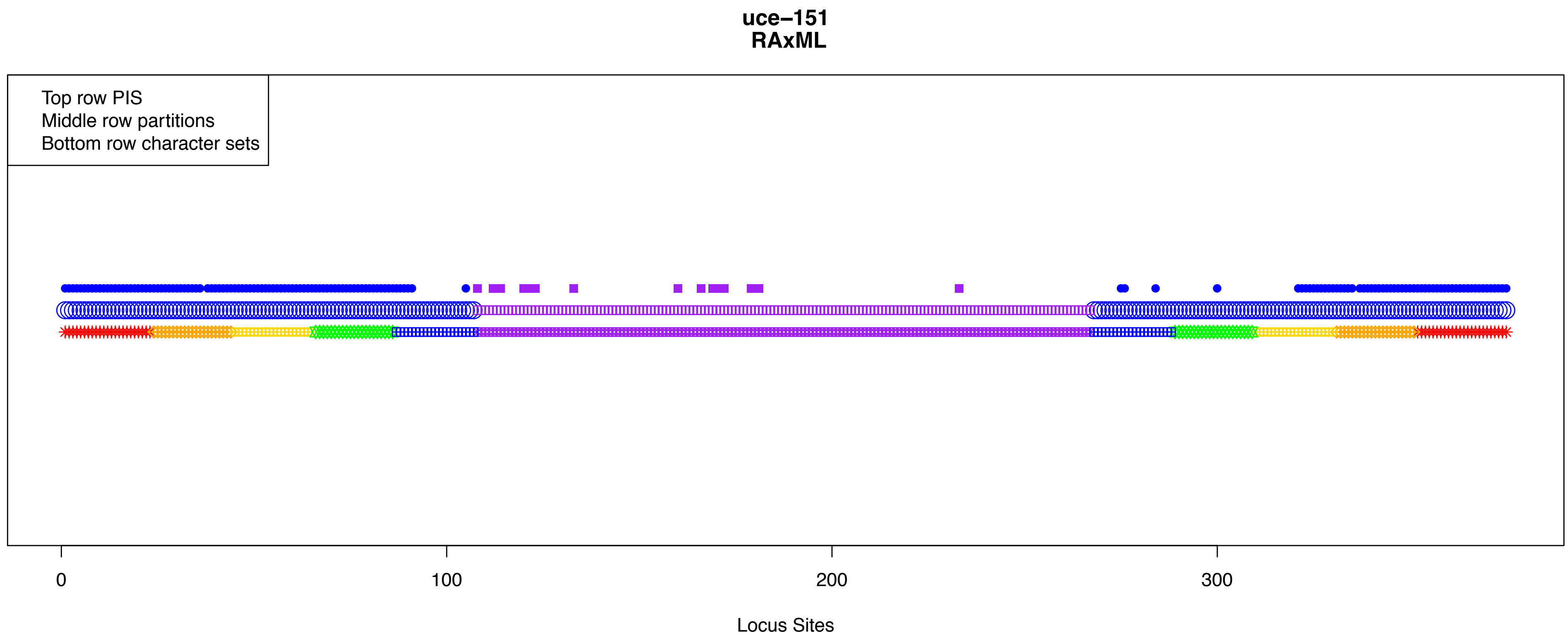

uce–151
RAxML
Top row PIS
Middle row partitions
Bottom row character sets
0
100
200
300
Locus Sites

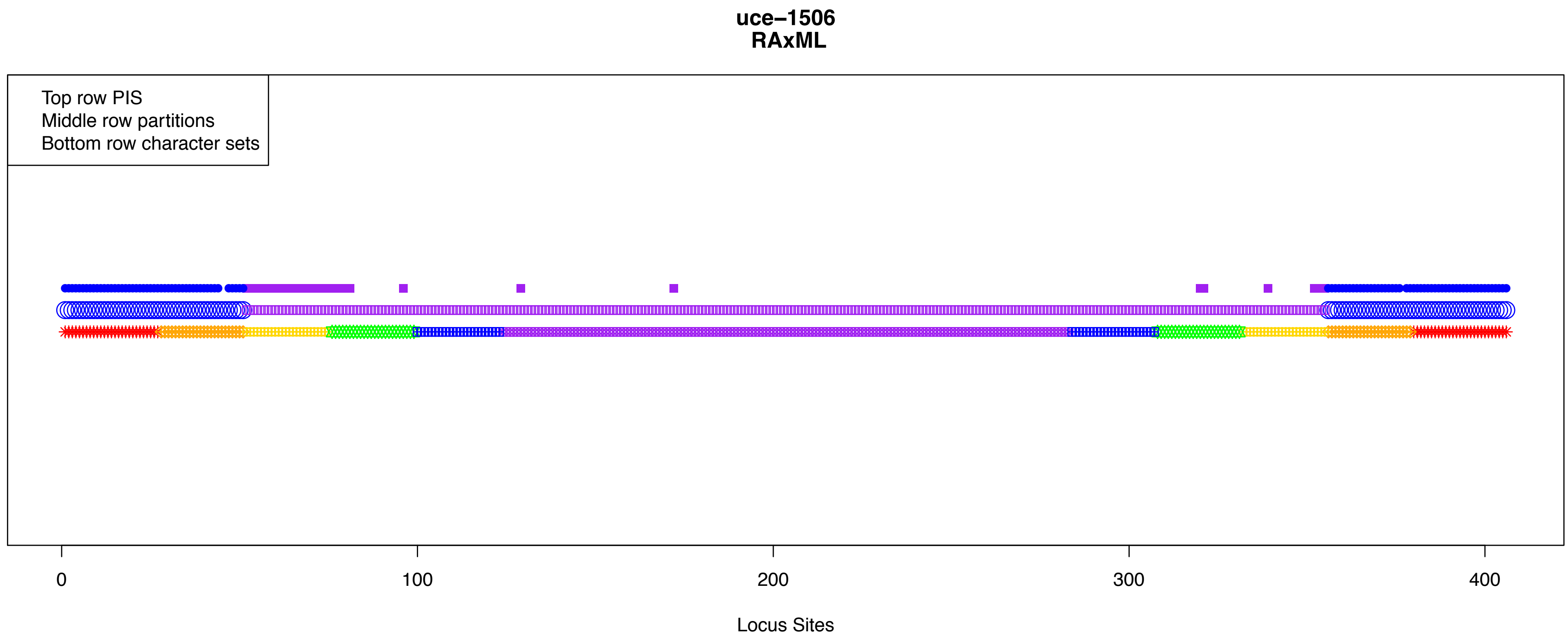

uce–1506
RAxML
Top row PIS
Middle row partitions
Bottom row character sets
0
100
200
300
400
Locus Sites

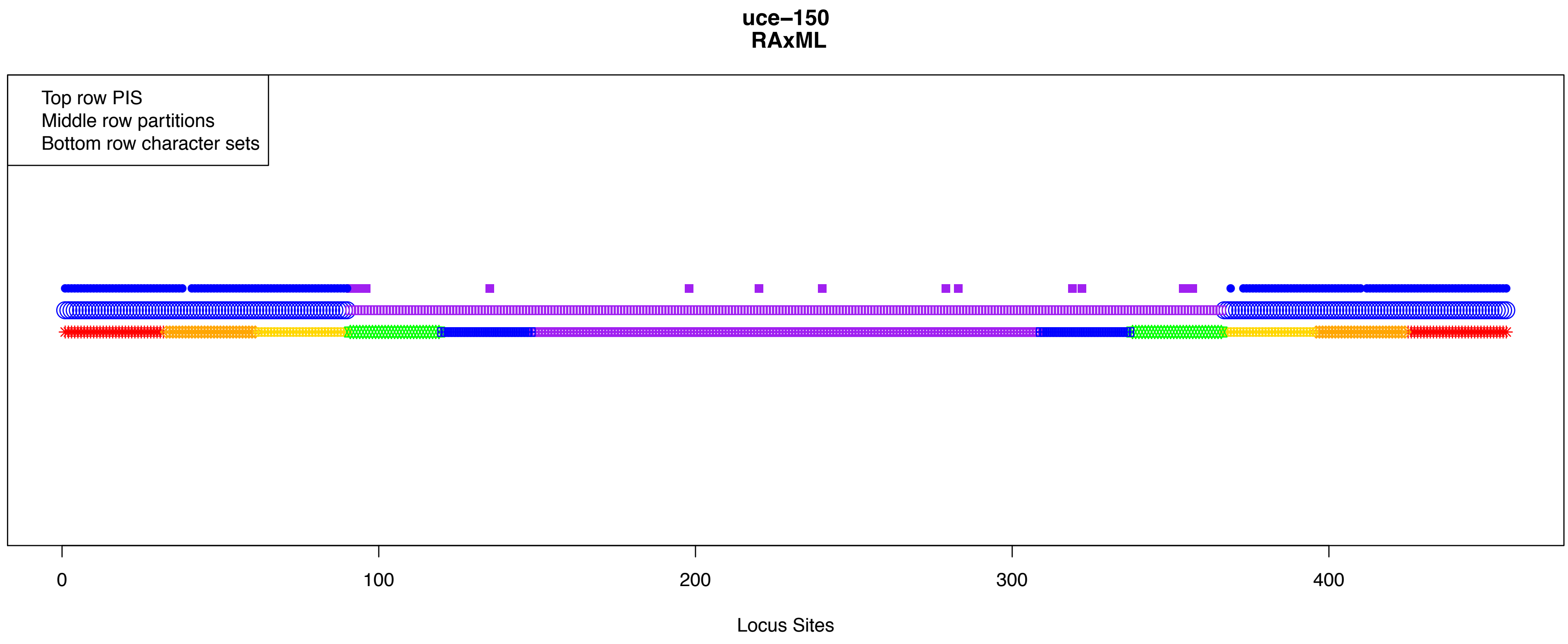
uce–150
RAxML
Top row PIS
Middle row partitions
Bottom row character sets
0
100
200
300
400
Locus Sites

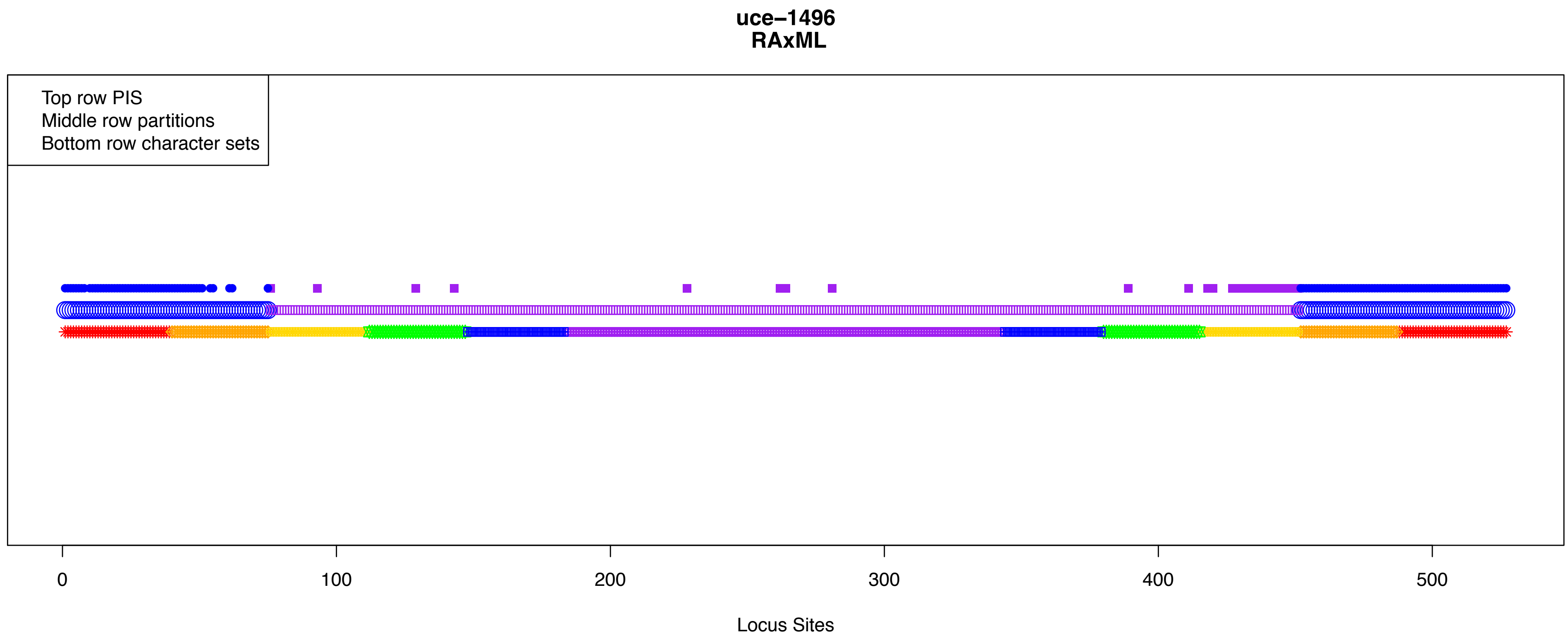
uce–1496
RAxML
Top row PIS
Middle row partitions
Bottom row character sets
0
100
200
300
400
500
Locus Sites

uce–1488
RAxML

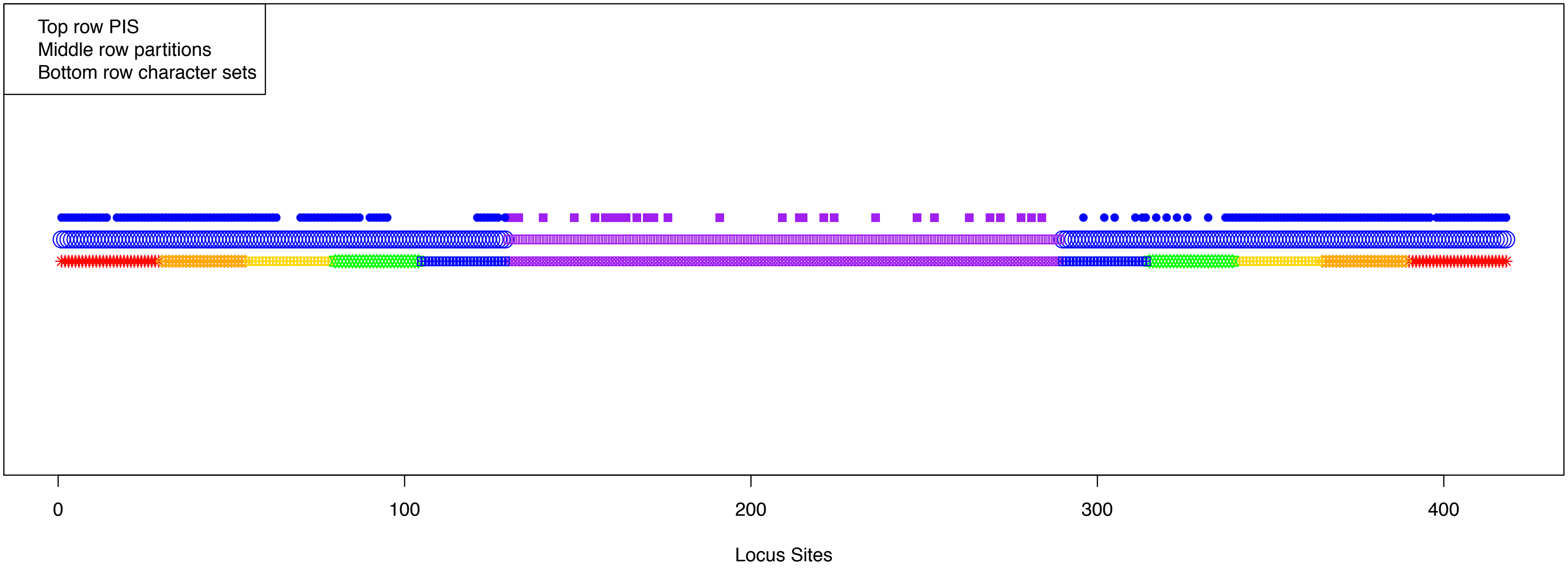

uce–1478
RAxML

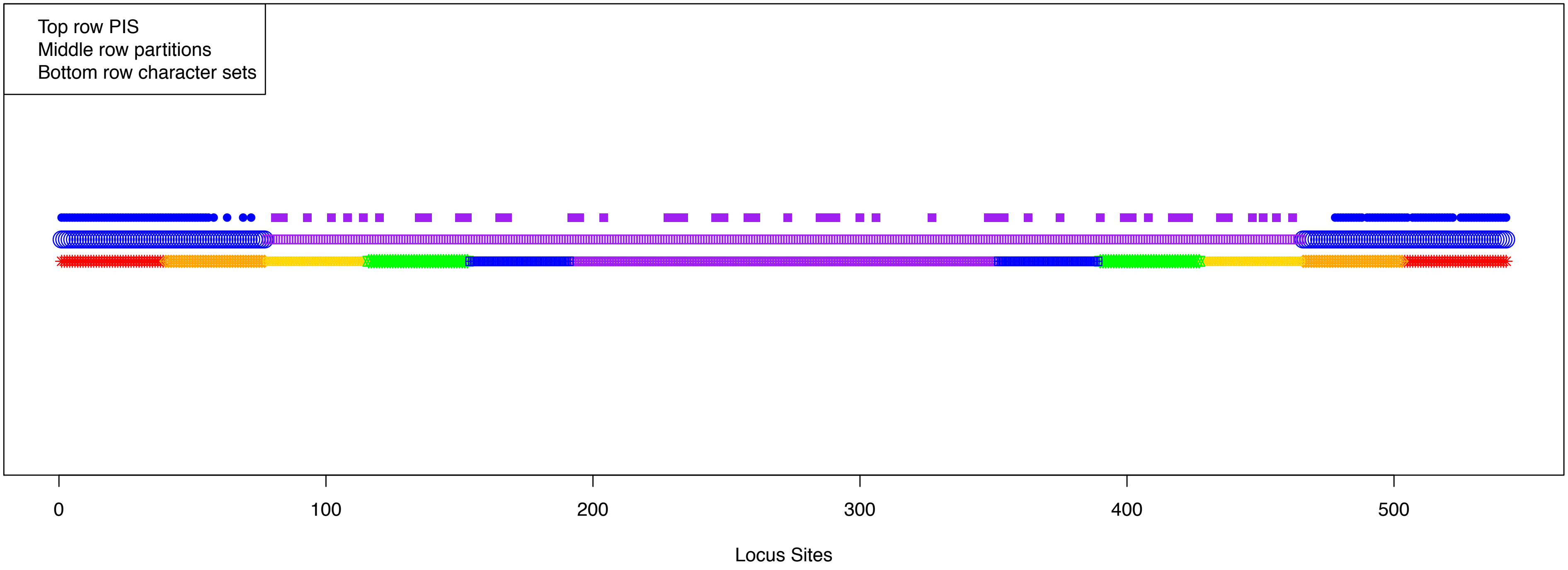

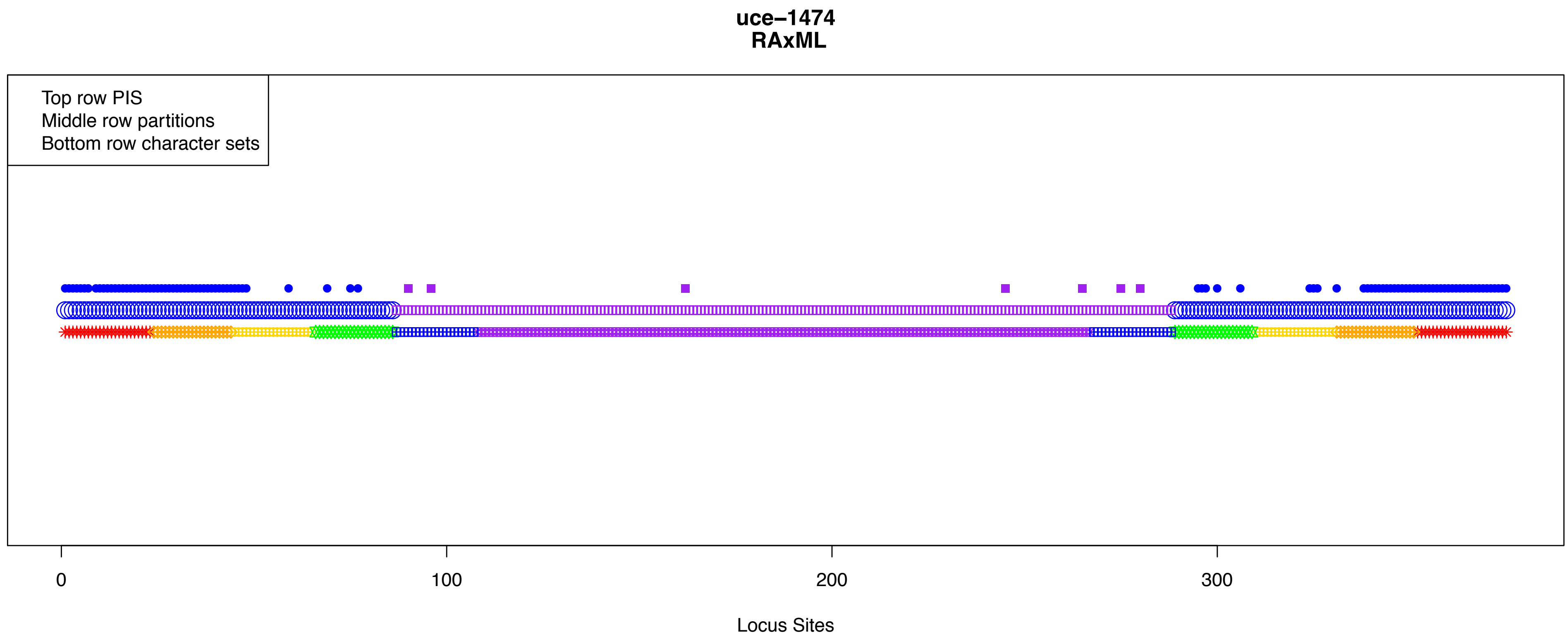
uce–1474
RAxML
Top row PIS
Middle row partitions
Bottom row character sets
0
100
200
300
Locus Sites

uce–1471
RAxML

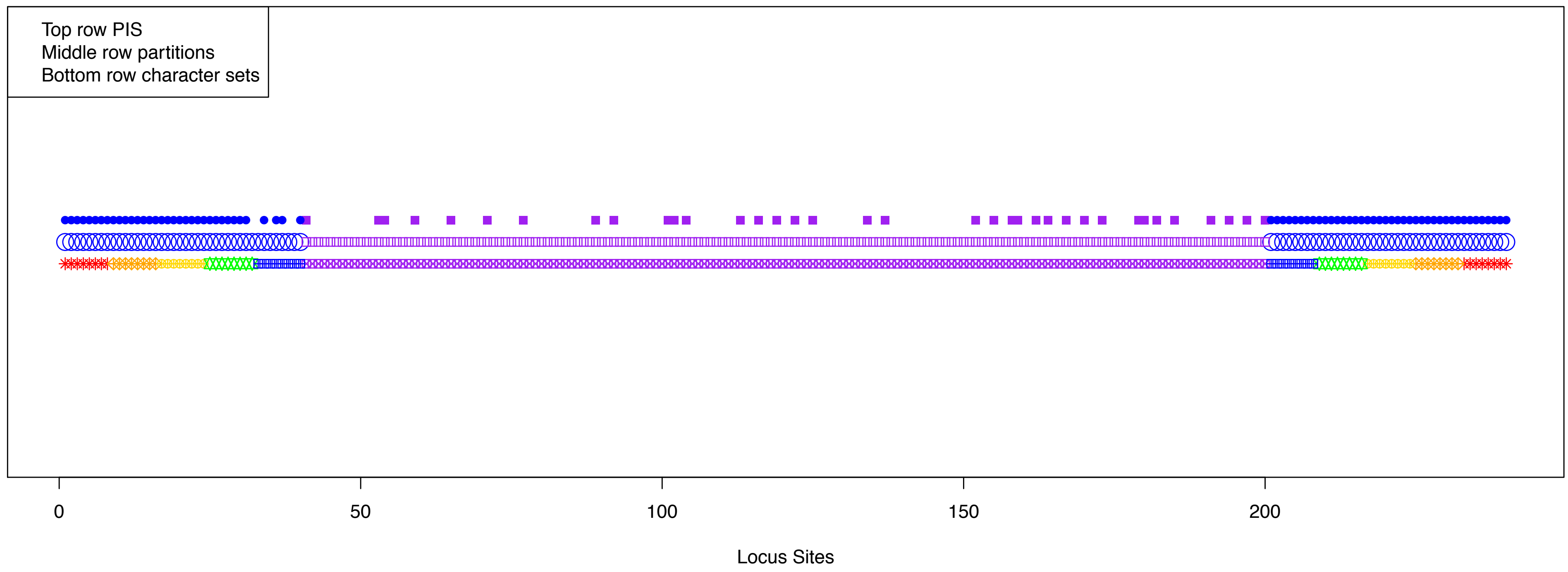

uce–1458
RAxML

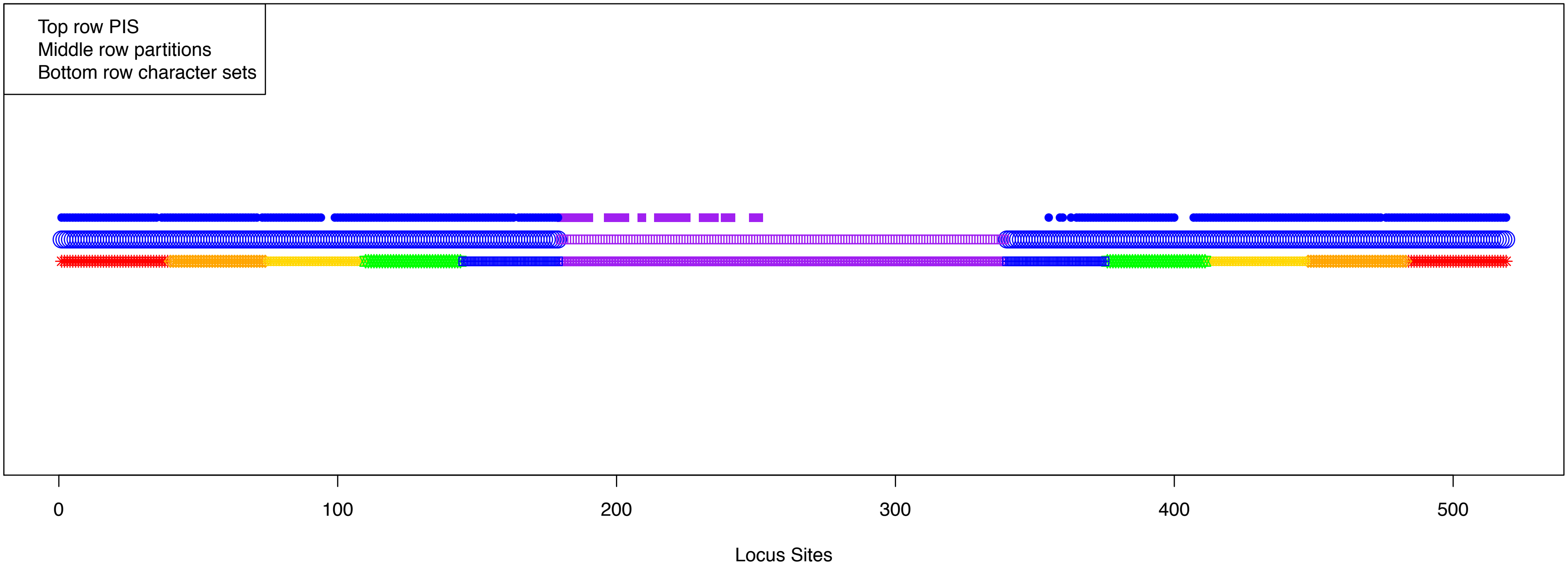

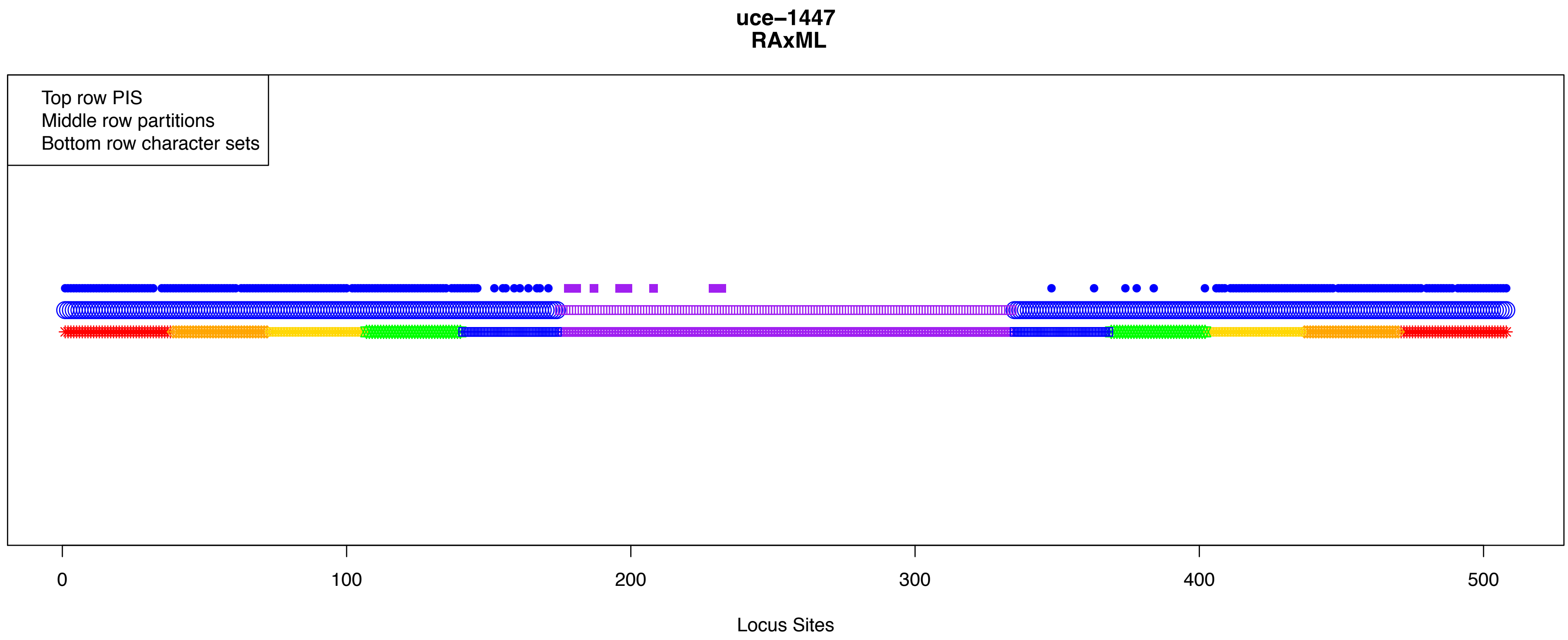
uce–1447
RAxML
Top row PIS
Middle row partitions
Bottom row character sets
0
100
200
300
400
500
Locus Sites

uce–1441
RAxML

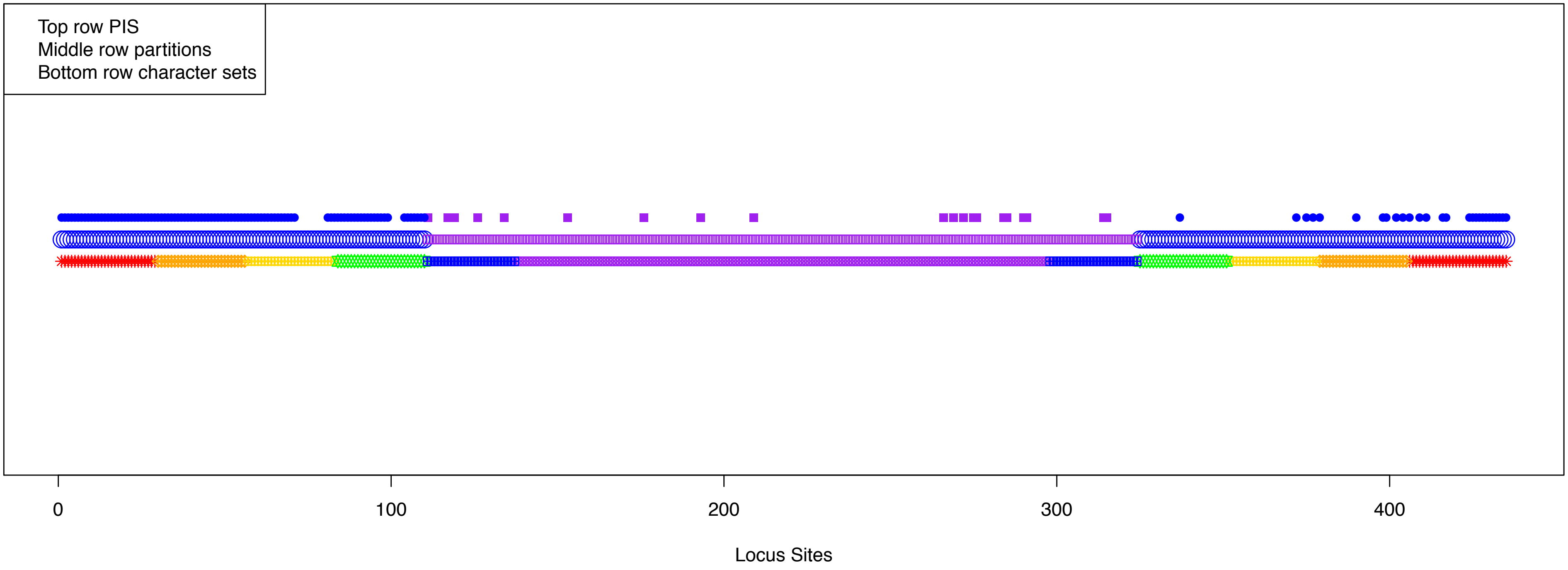

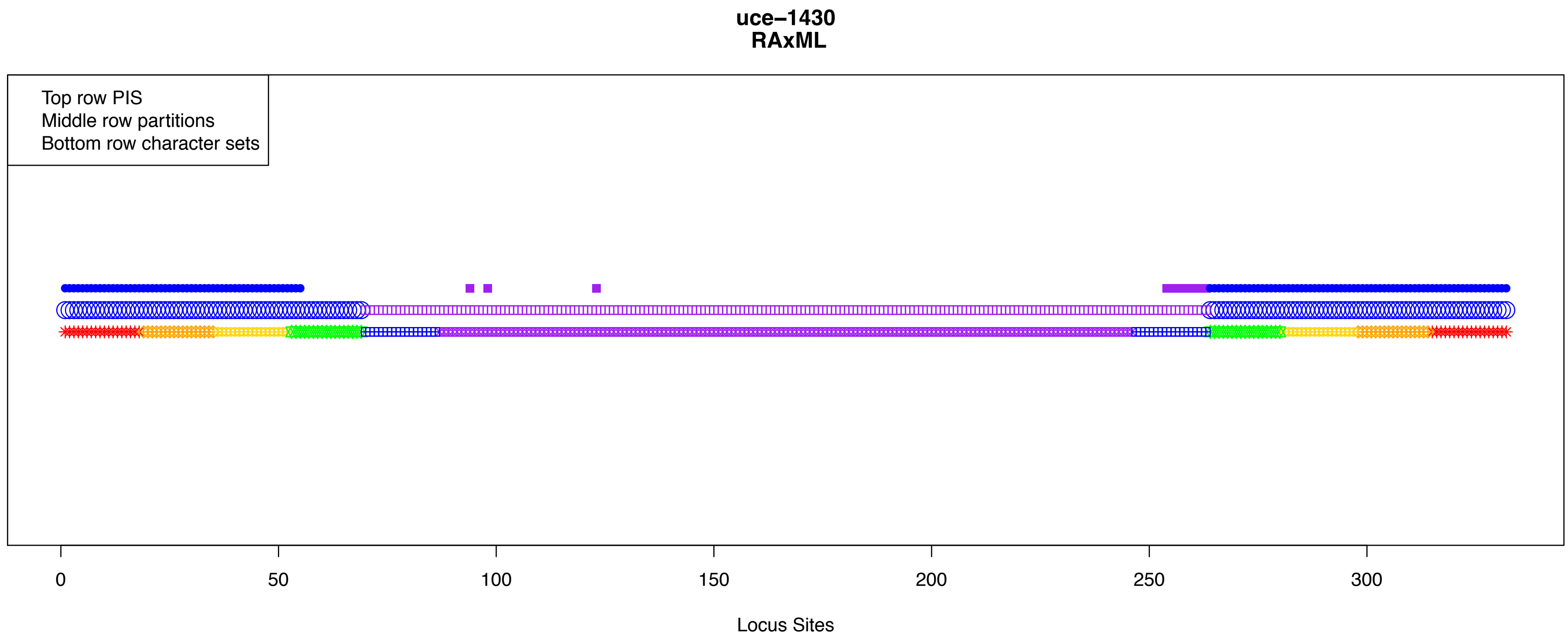
uce–1430
RAxML
Top row PIS
Middle row partitions
Bottom row character sets
0
50
100
150
200
250
300
Locus Sites

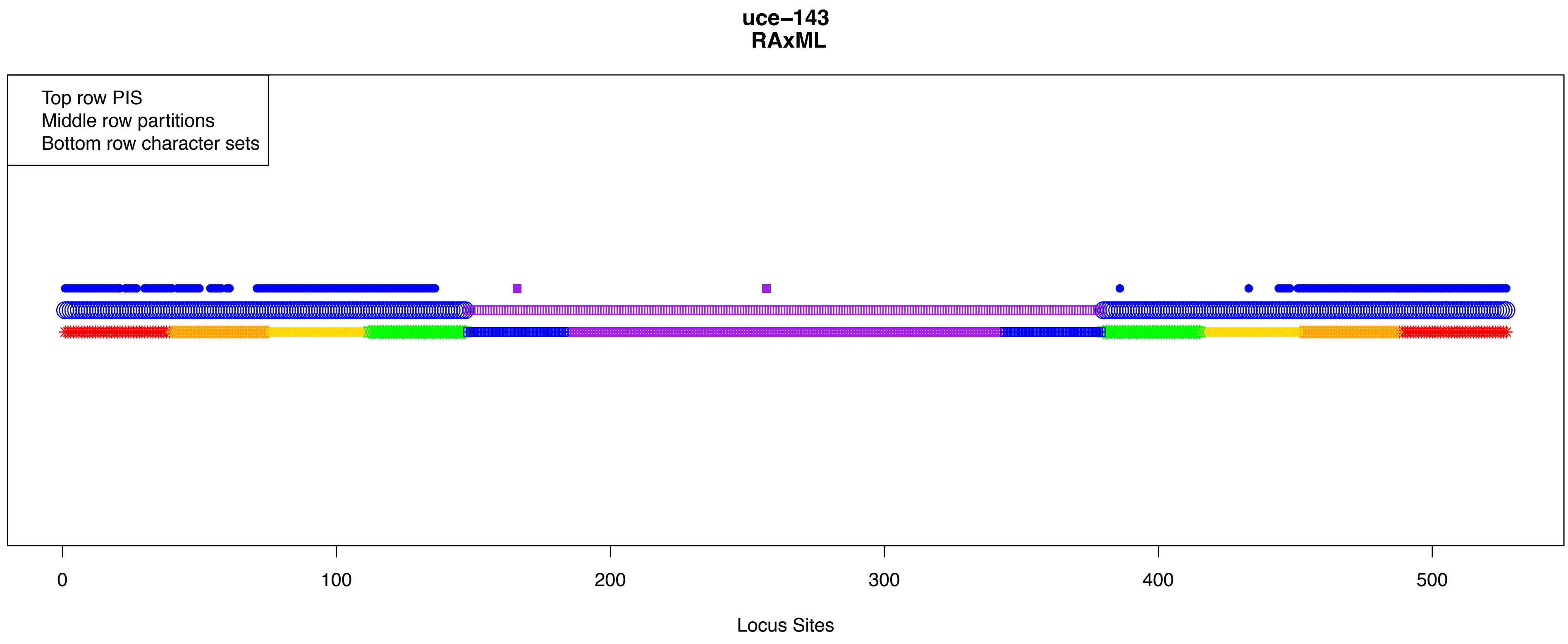

uce–143
RAxML
Top row PIS
Middle row partitions
Bottom row character sets
0
100
200
300
400
500
Locus Sites

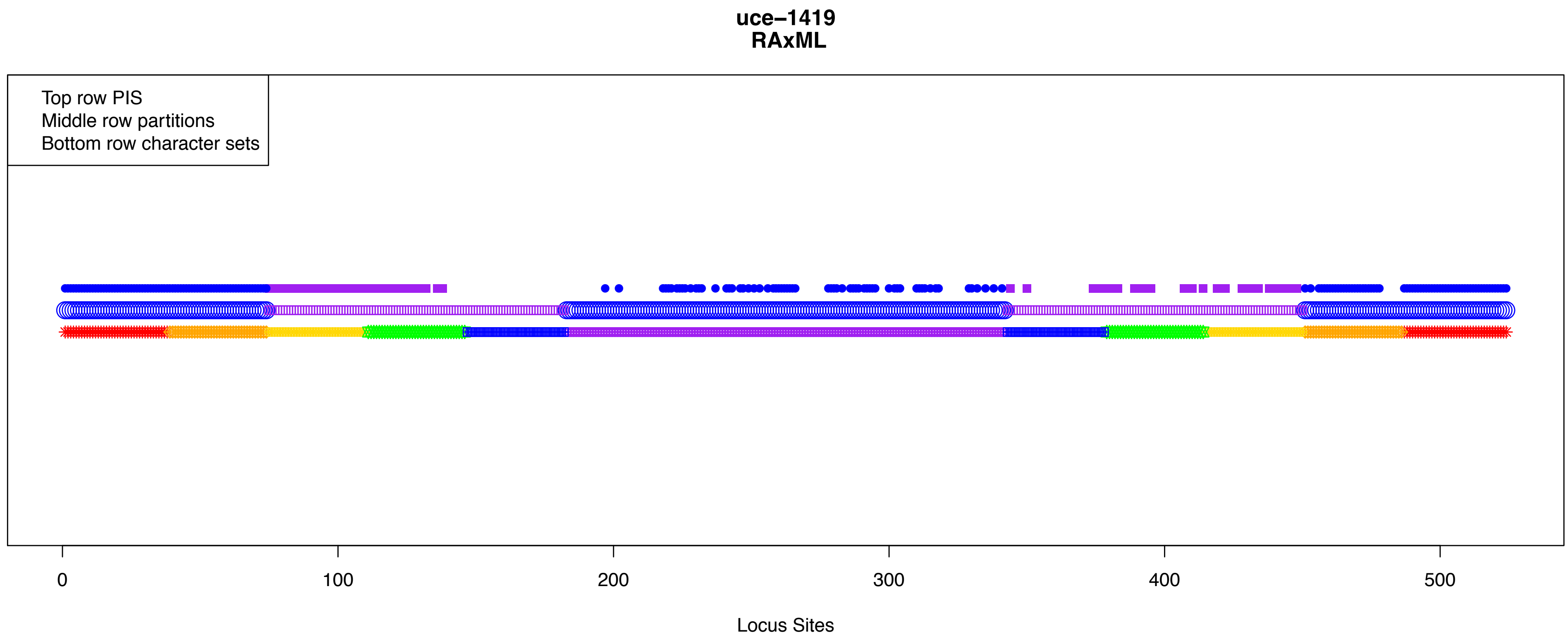
uce–1419
RAxML
Top row PIS
Middle row partitions
Bottom row character sets
0
100
200
300
400
500
Locus Sites

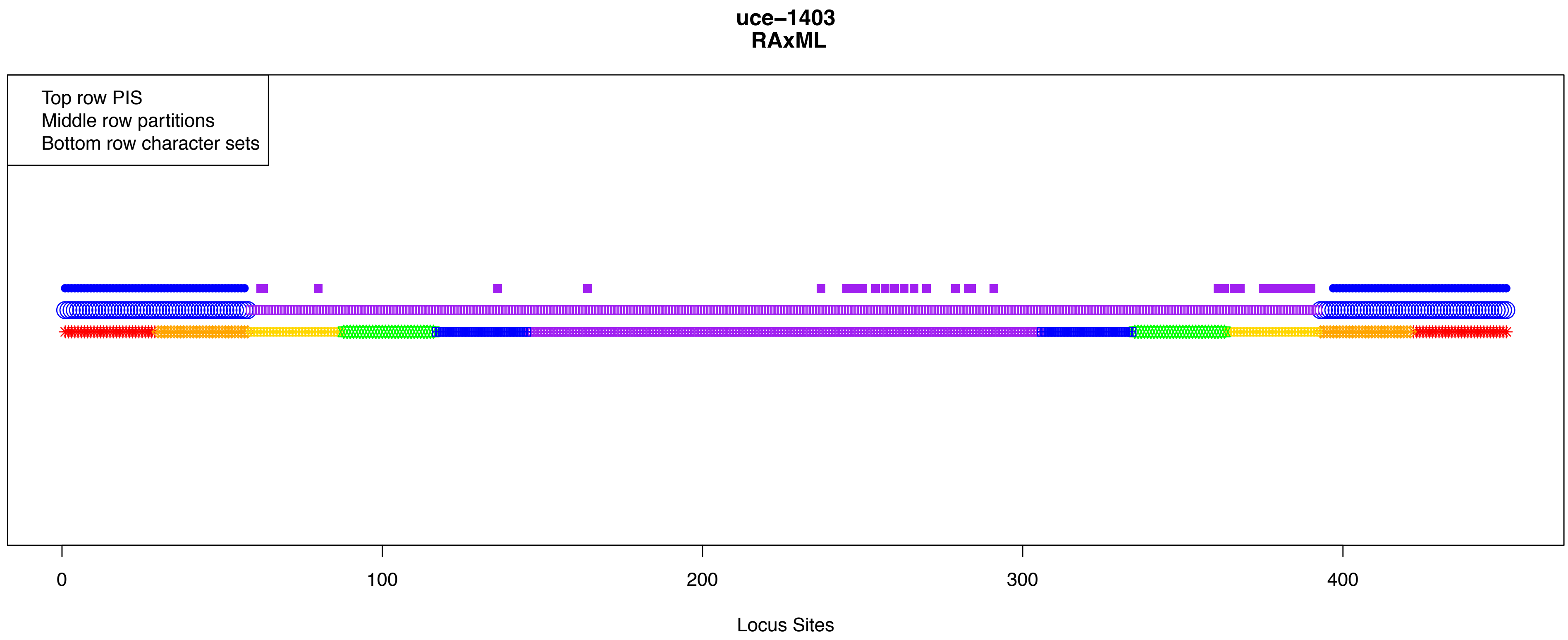
uce–1403
RAxML
Top row PIS
Middle row partitions
Bottom row character sets
0
100
200
300
400
Locus Sites

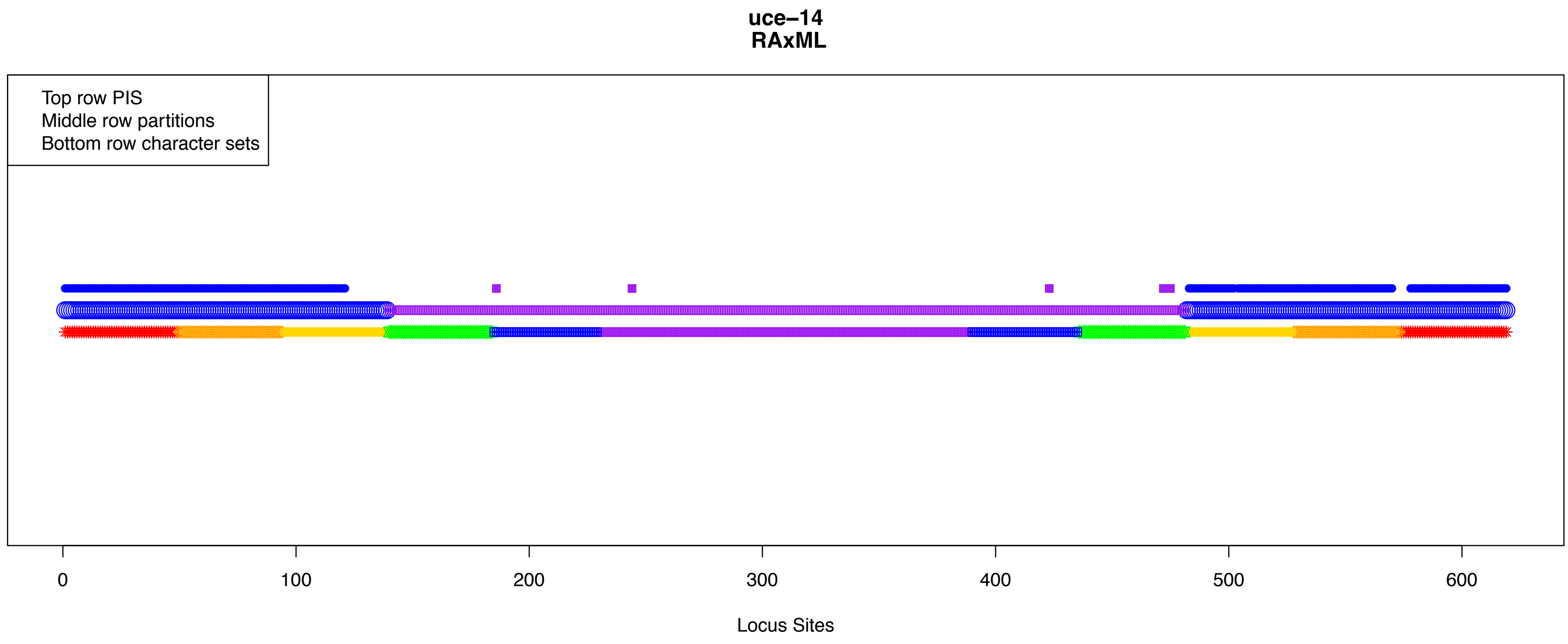
uce–14
RAxML
Top row PIS
Middle row partitions
Bottom row character sets
0
100
200
300
400
500
600
Locus Sites

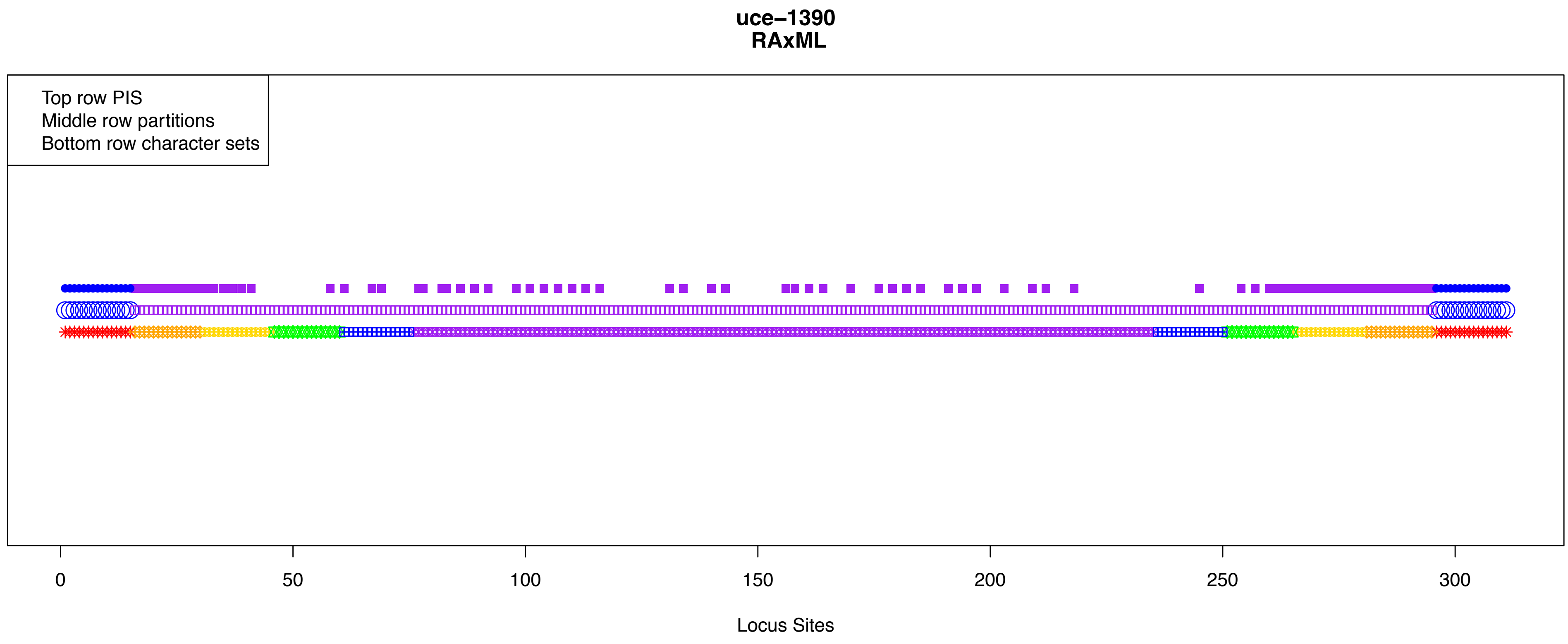
uce–1390
RAxML
Top row PIS
Middle row partitions
Bottom row character sets
0
50
100
150
200
250
300
Locus Sites

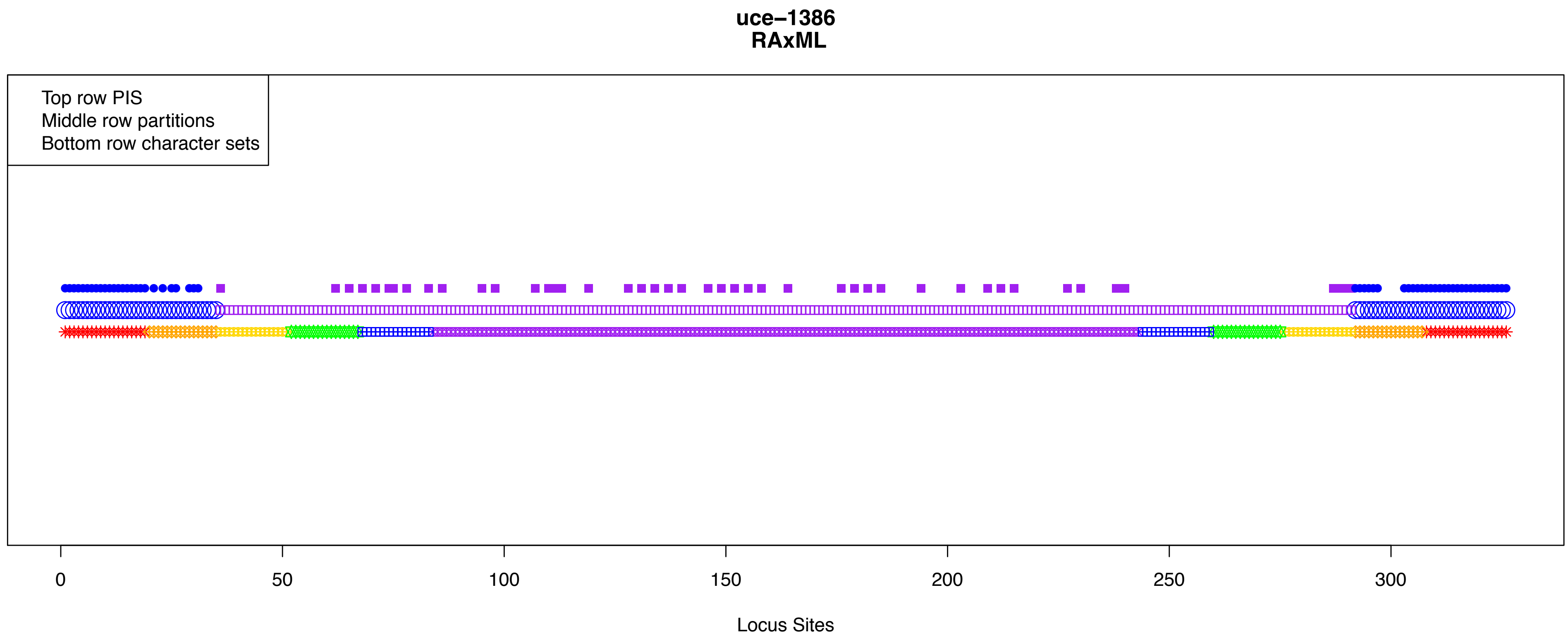
uce–1386
RAxML
Top row PIS
Middle row partitions
Bottom row character sets
0
50
100
150
200
250
300
Locus Sites

uce–1383
RAxML

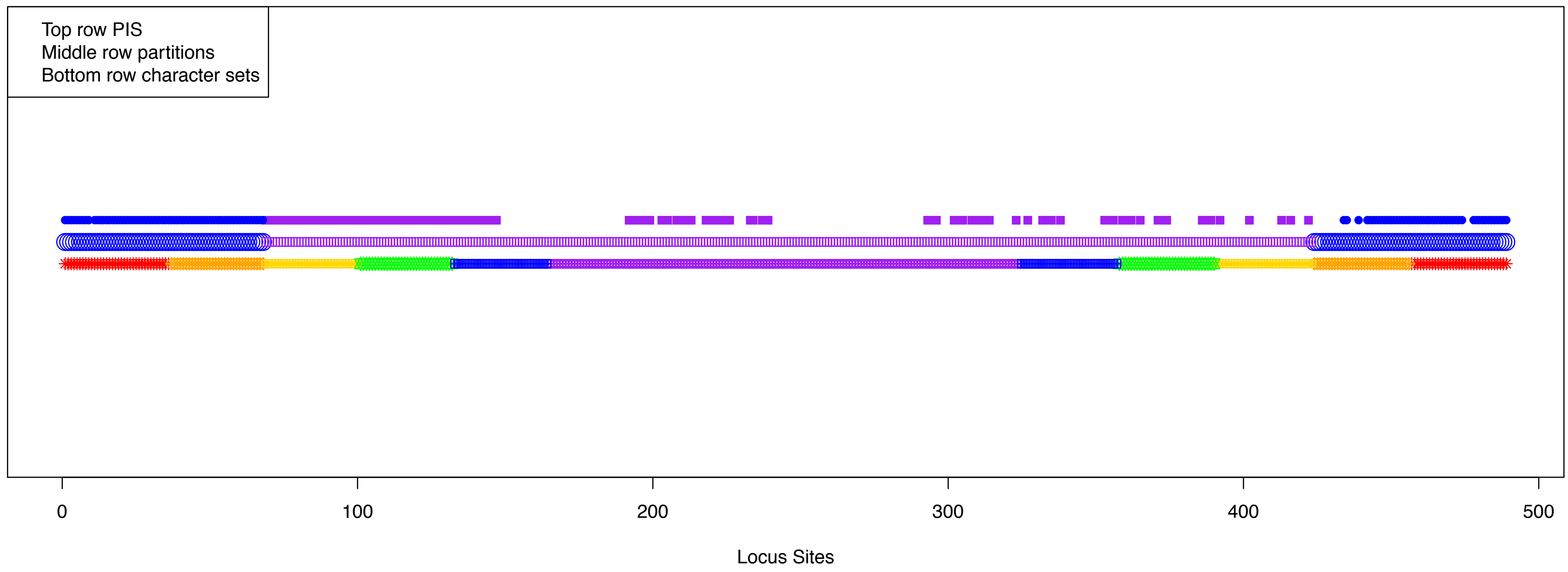

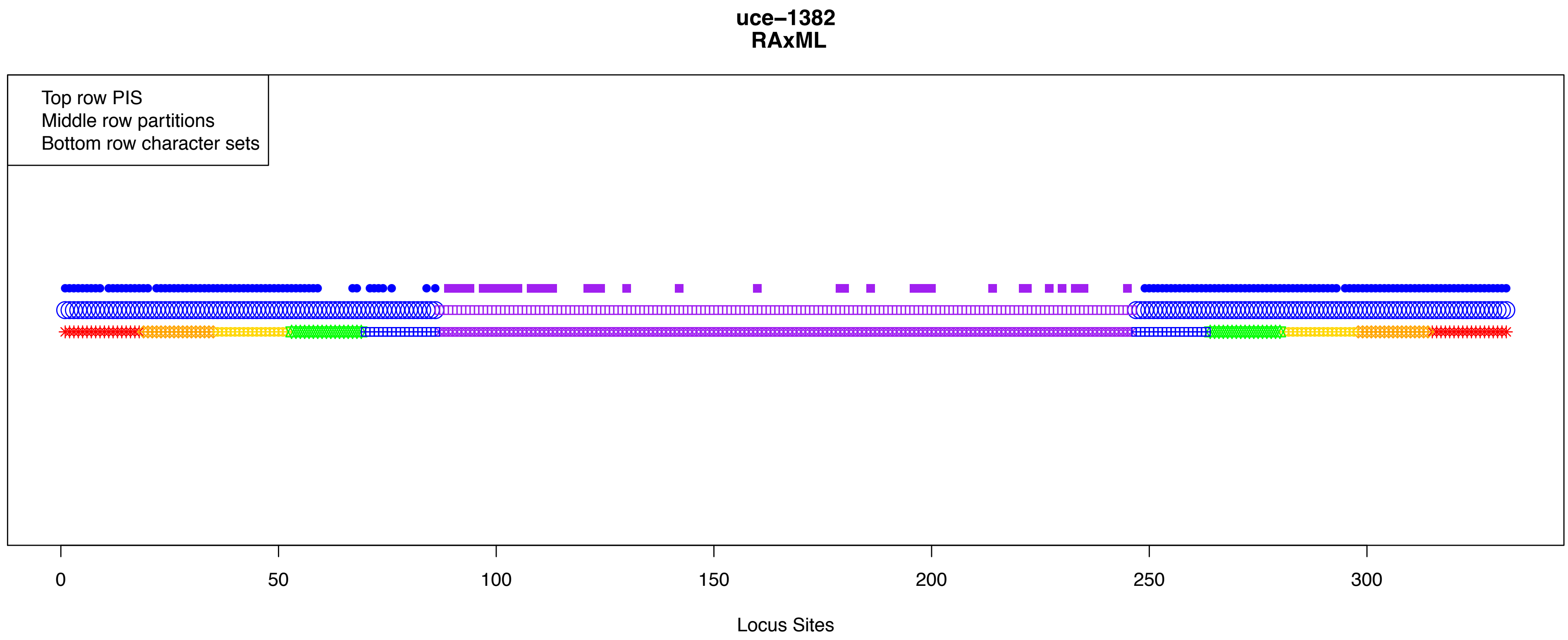
uce–1382
RAxML
Top row PIS
Middle row partitions
Bottom row character sets
0
50
100
150
200
250
300
Locus Sites

uce–1380
RAxML

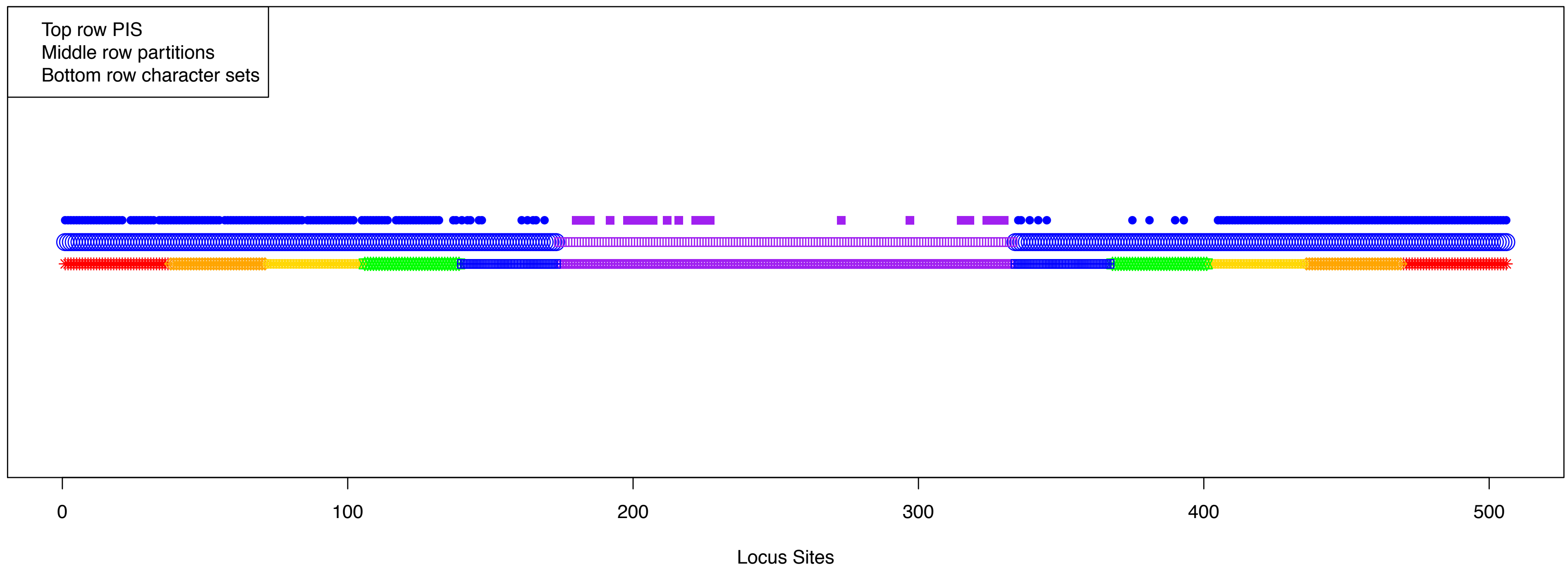

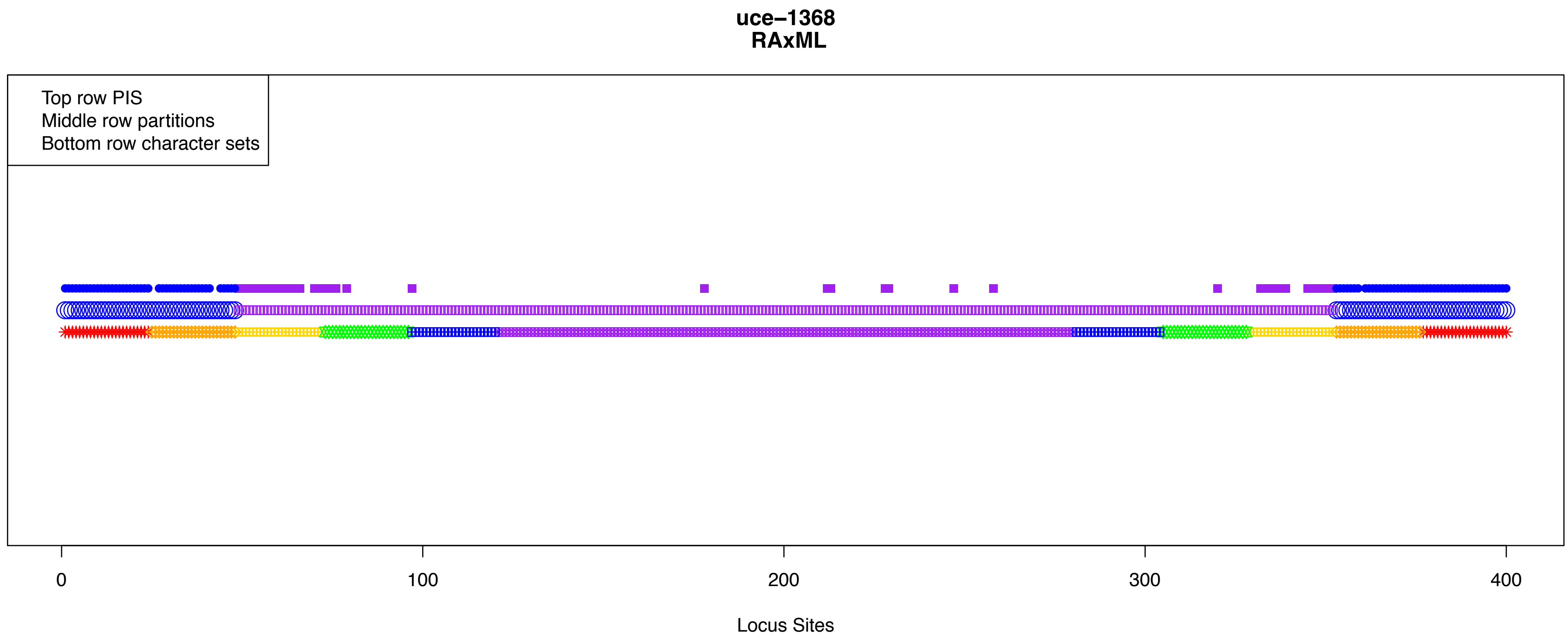

uce–1368
RAxML
Top row PIS
Middle row partitions
Bottom row character sets
0
100
200
300
400
Locus Sites

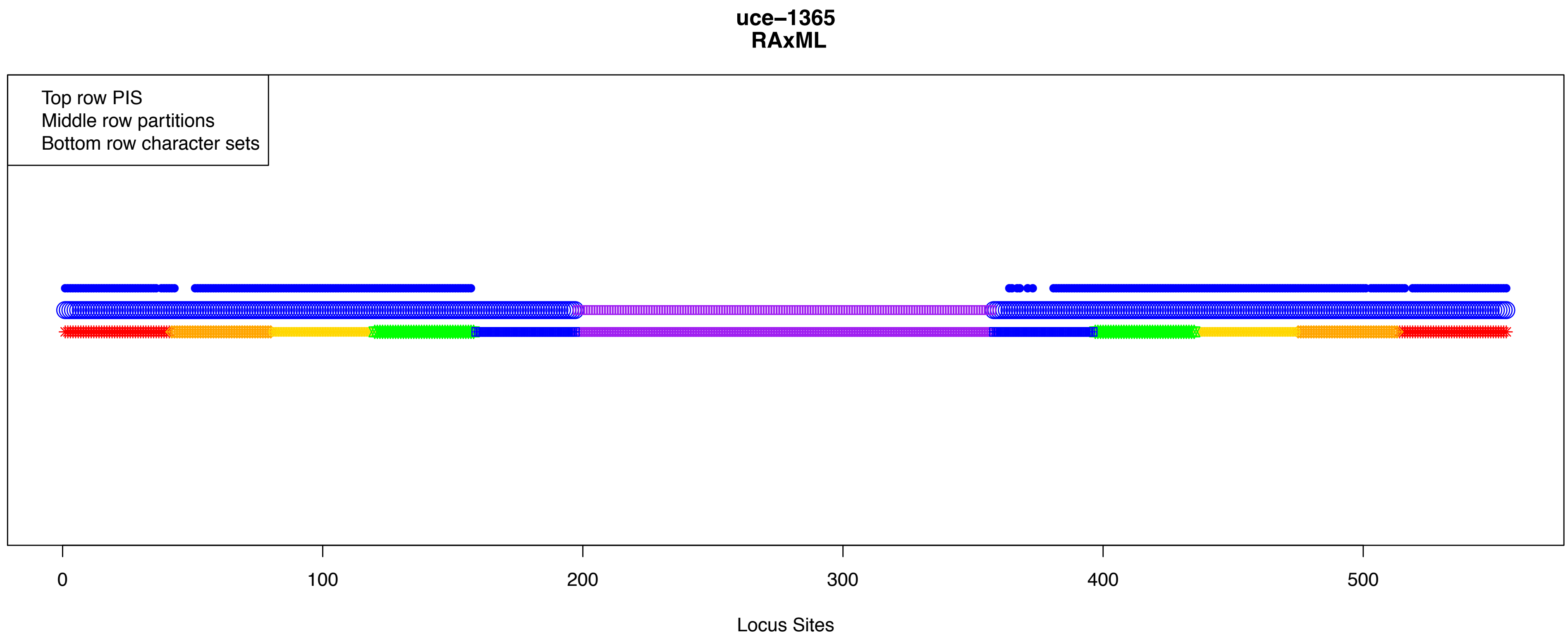
uce−1365
RAxML
Top row PIS
Middle row partitions
Bottom row character sets
0
100
200
300
400
500
Locus Sites

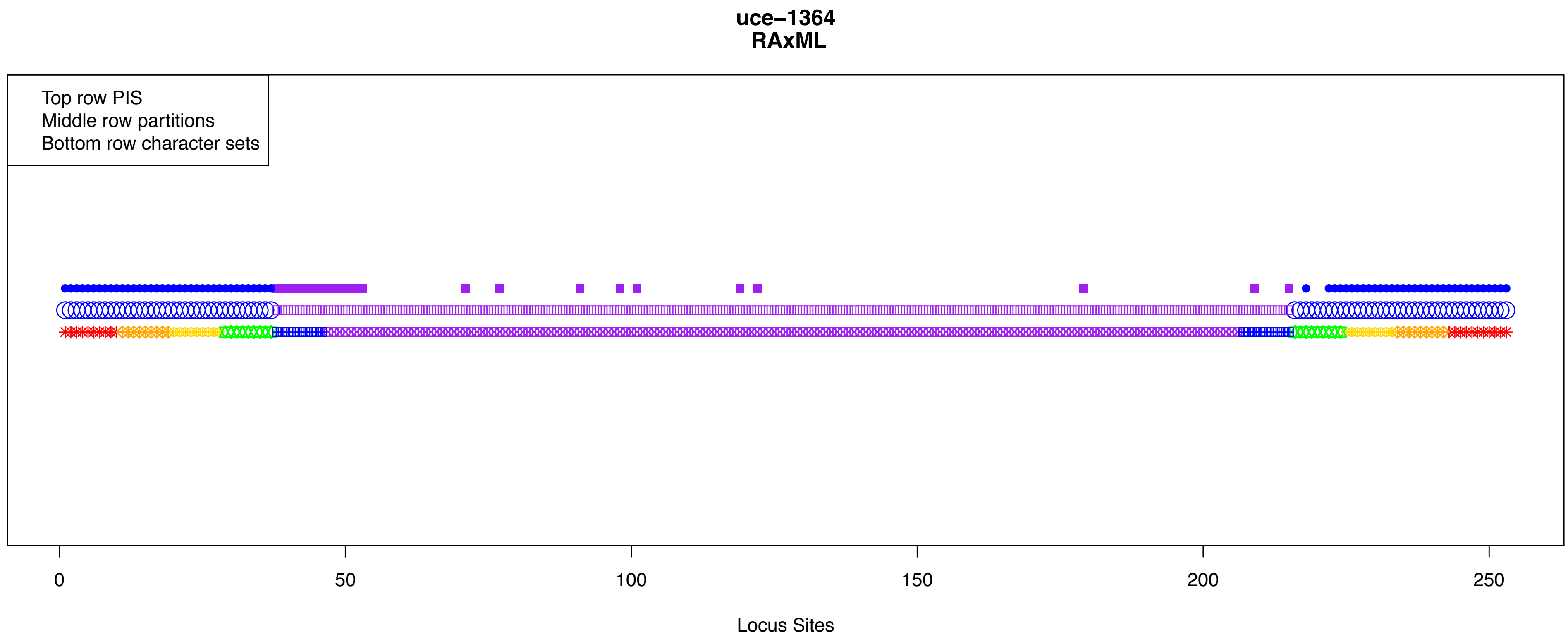
uce–1364
RAxML
Top row PIS
Middle row partitions
Bottom row character sets
0
50
100
150
200
250
Locus Sites

uce–1349
RAxML

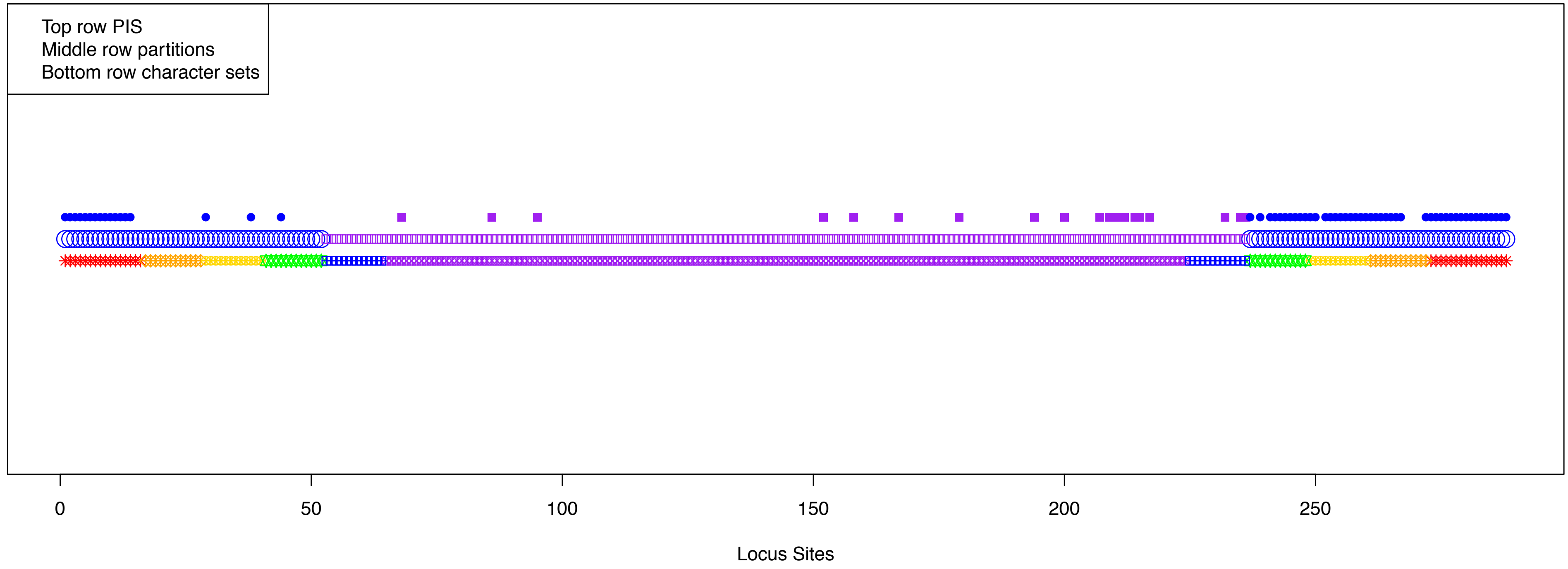

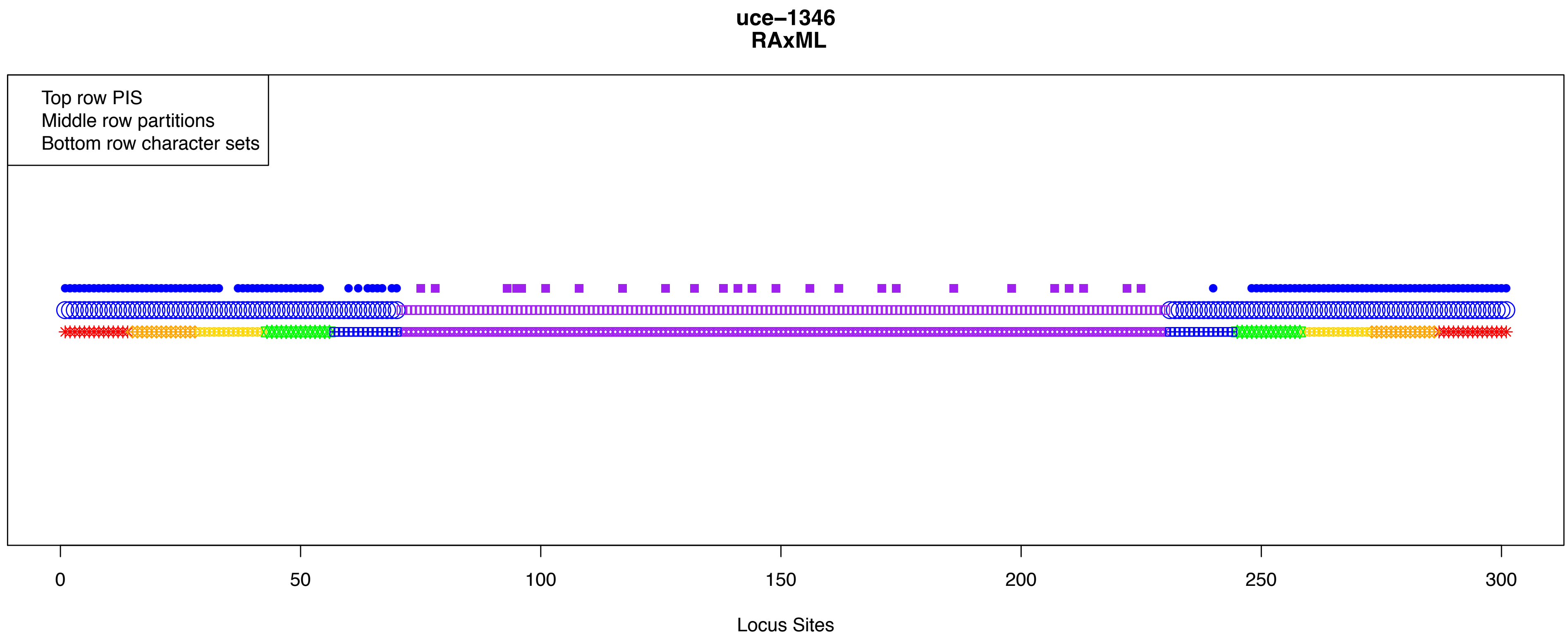
uce–1346
RAxML
Top row PIS
Middle row partitions
Bottom row character sets
0
50
100
150
200
250
300
Locus Sites

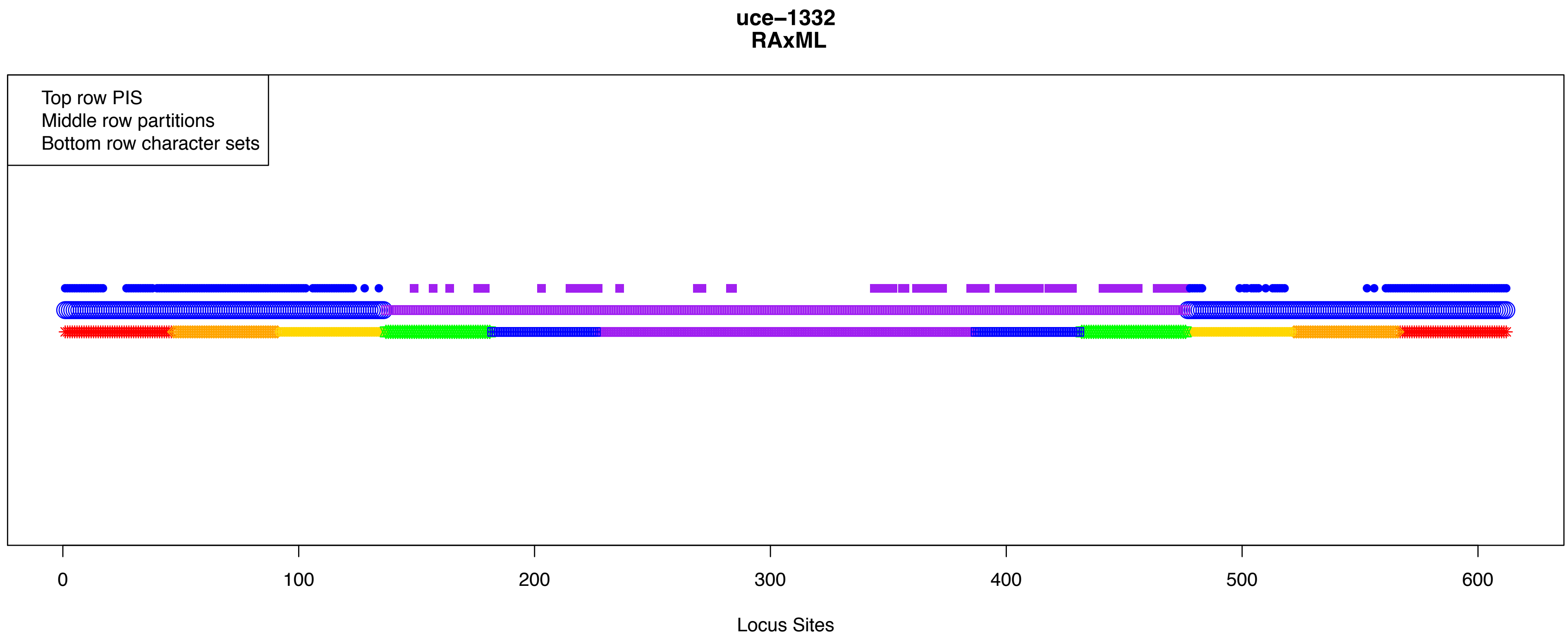
uce–1332
RAxML
Top row PIS
Middle row partitions
Bottom row character sets
0
100
200
300
400
500
600
Locus Sites

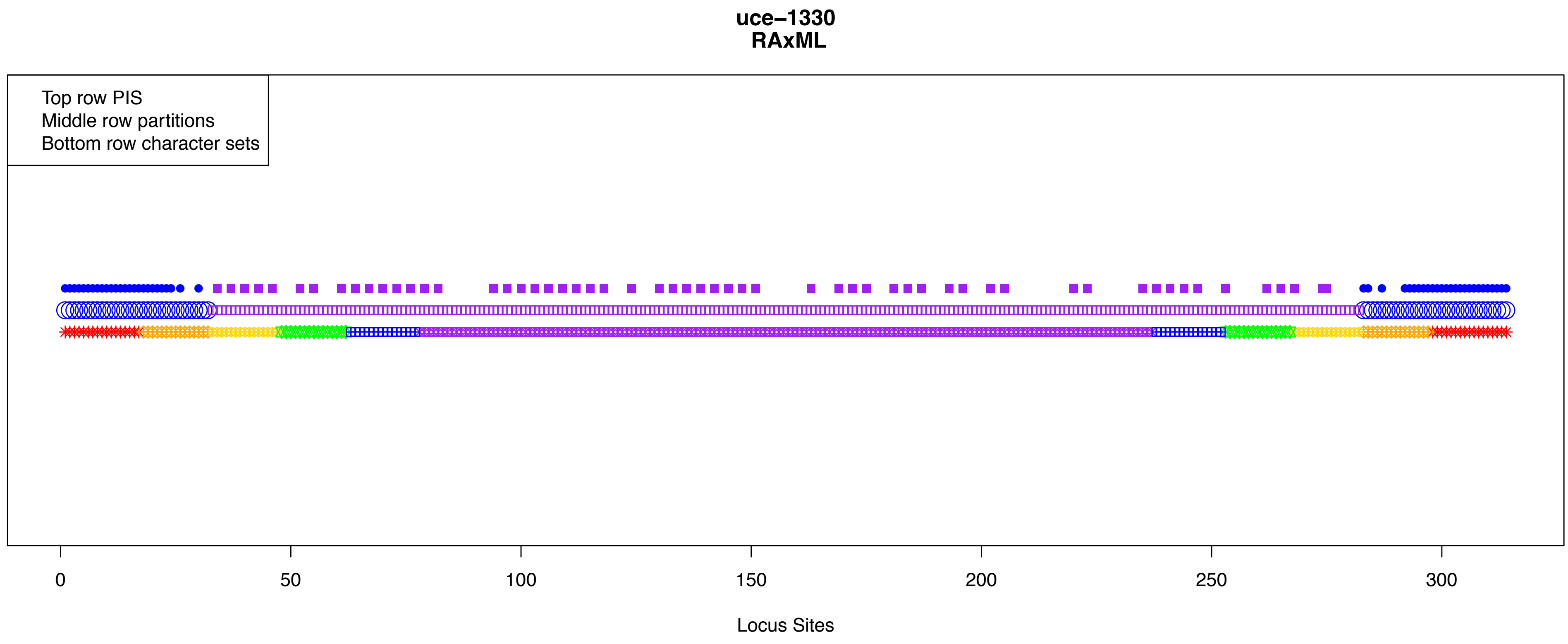
uce–1330
RAxML
Top row PIS
Middle row partitions
Bottom row character sets
0
50
100
150
200
250
300
Locus Sites

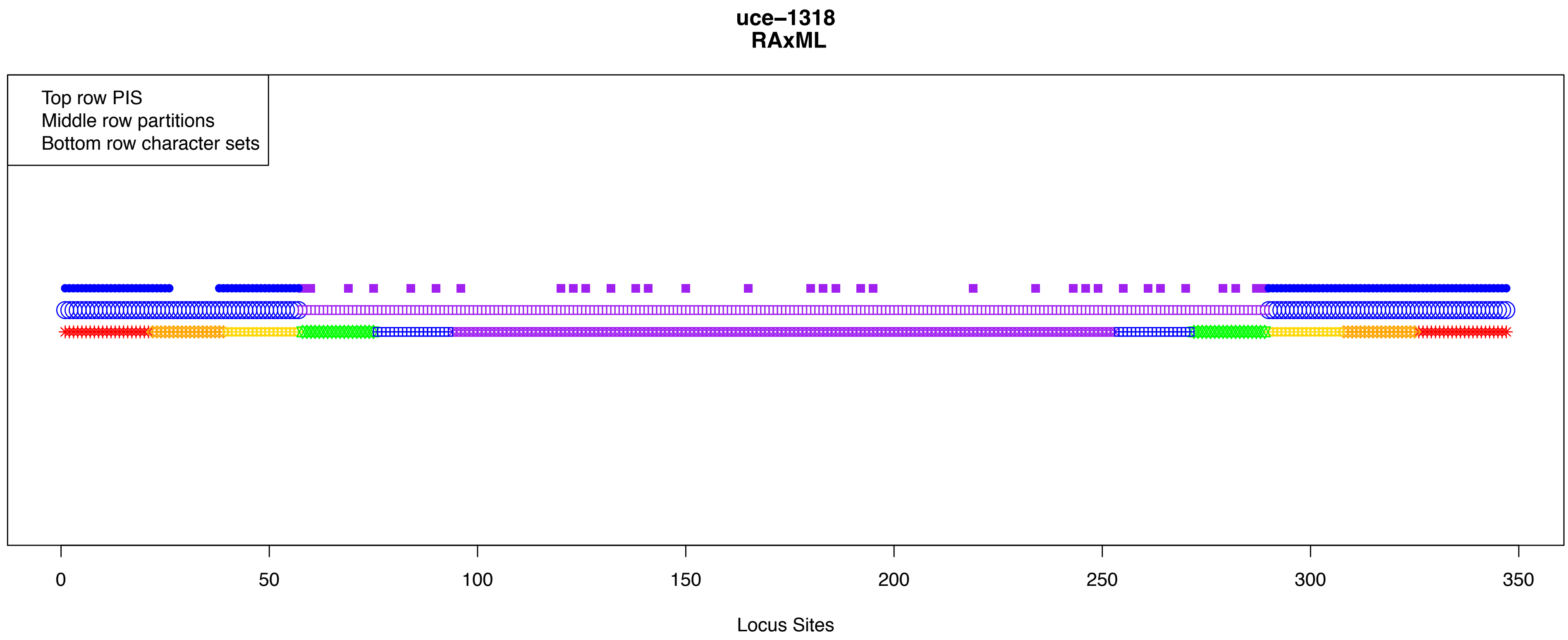
uce–1318
RAxML
Top row PIS
Middle row partitions
Bottom row character sets
0
50
100
150
200
250
300
350
Locus Sites

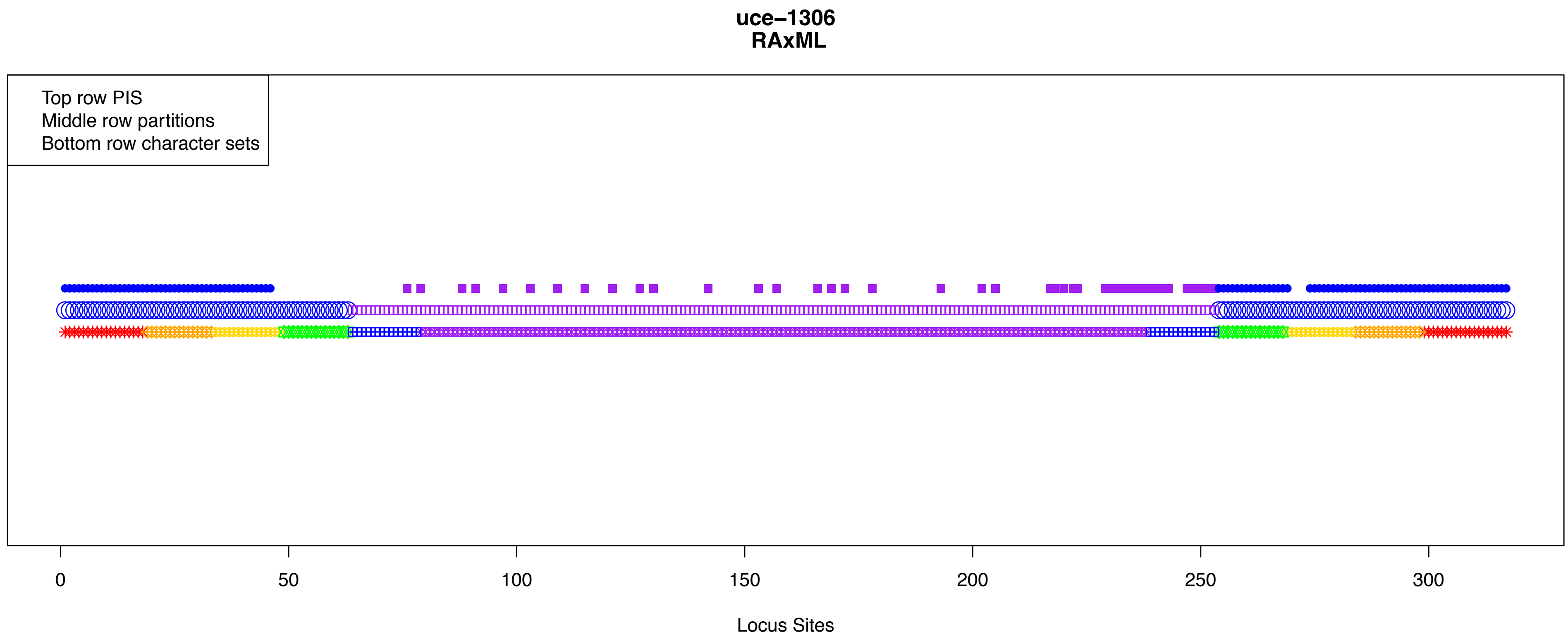

uce–1306
RAxML
Top row PIS
Middle row partitions
Bottom row character sets
0
50
100
150
200
250
300
Locus Sites

uce–130
RAxML

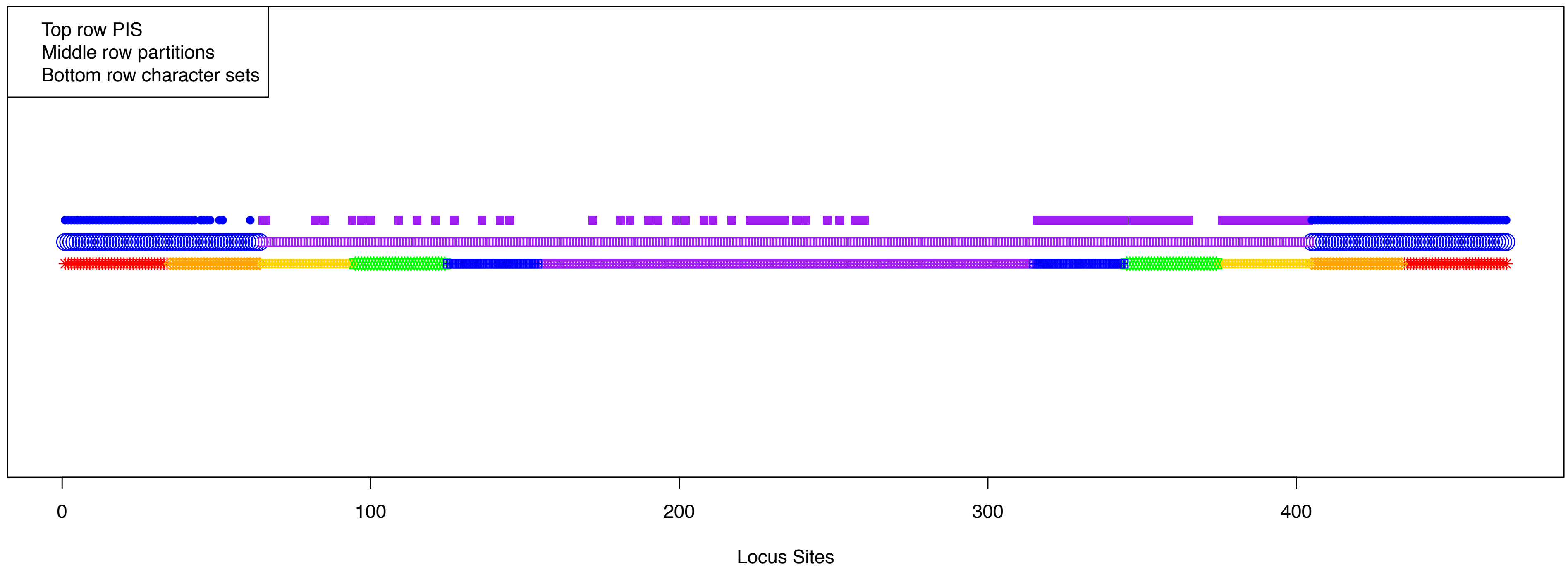

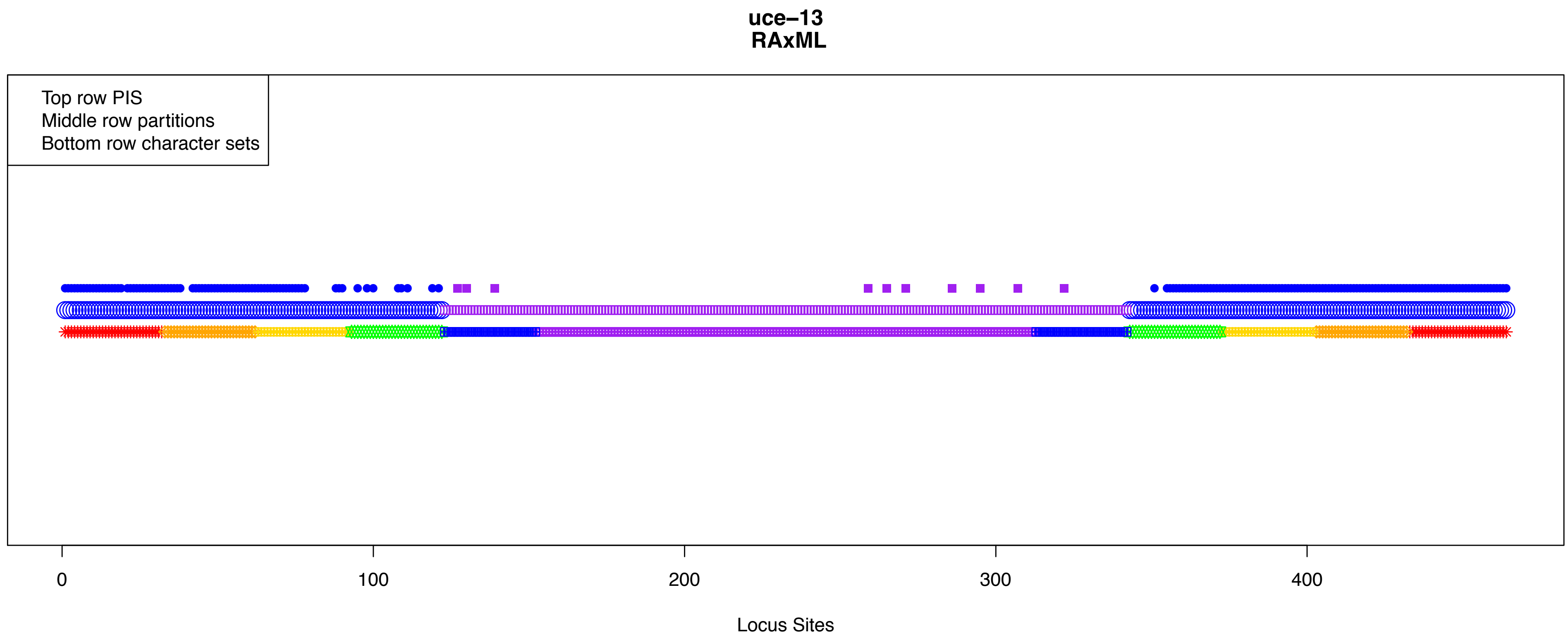
uce–13
RAxML
Top row PIS
Middle row partitions
Bottom row character sets
0
100
200
300
400
Locus Sites

uce–1294
RAxML

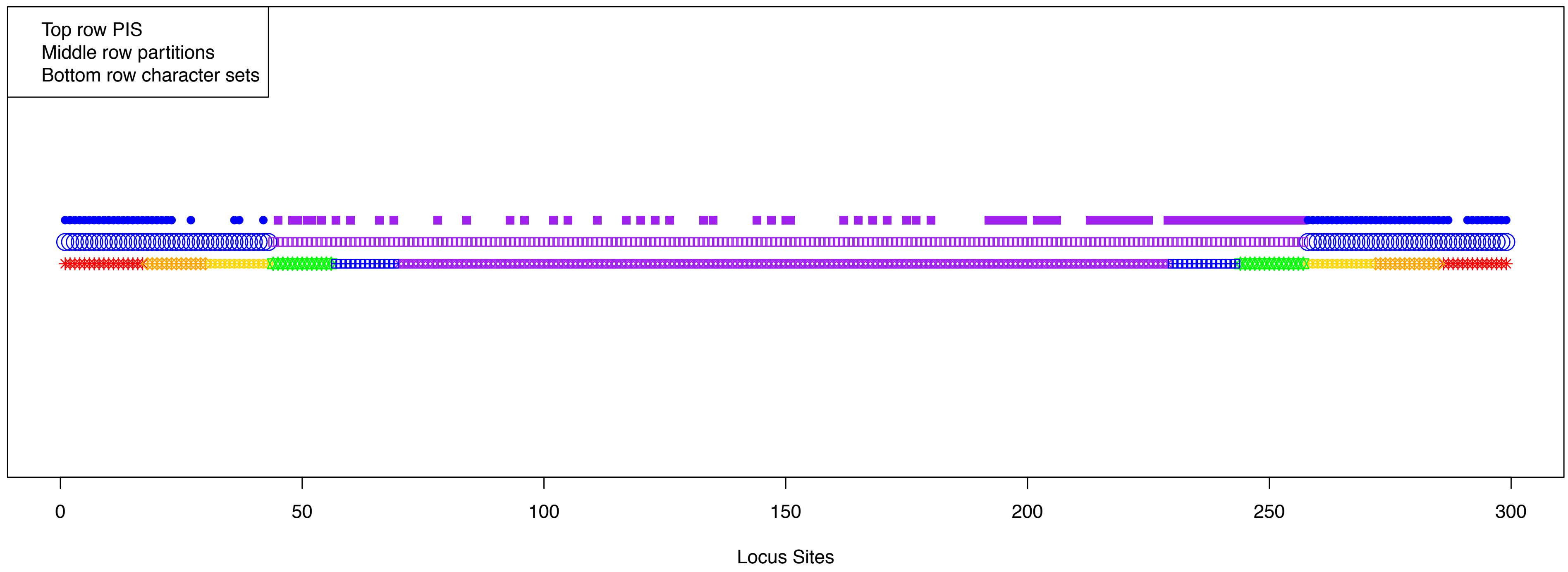

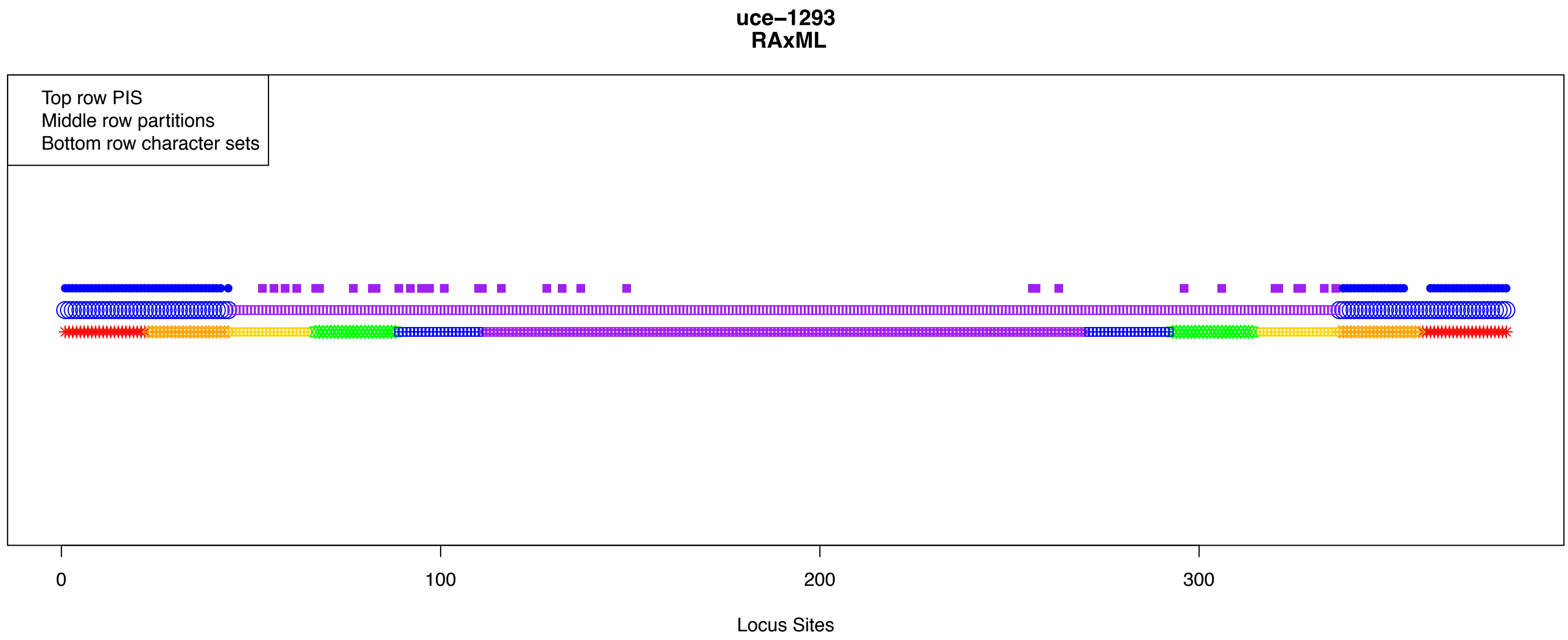
uce–1293
RAxML
Top row PIS
Middle row partitions
Bottom row character sets
0
100
200
300
Locus Sites

uce–1291
RAxML

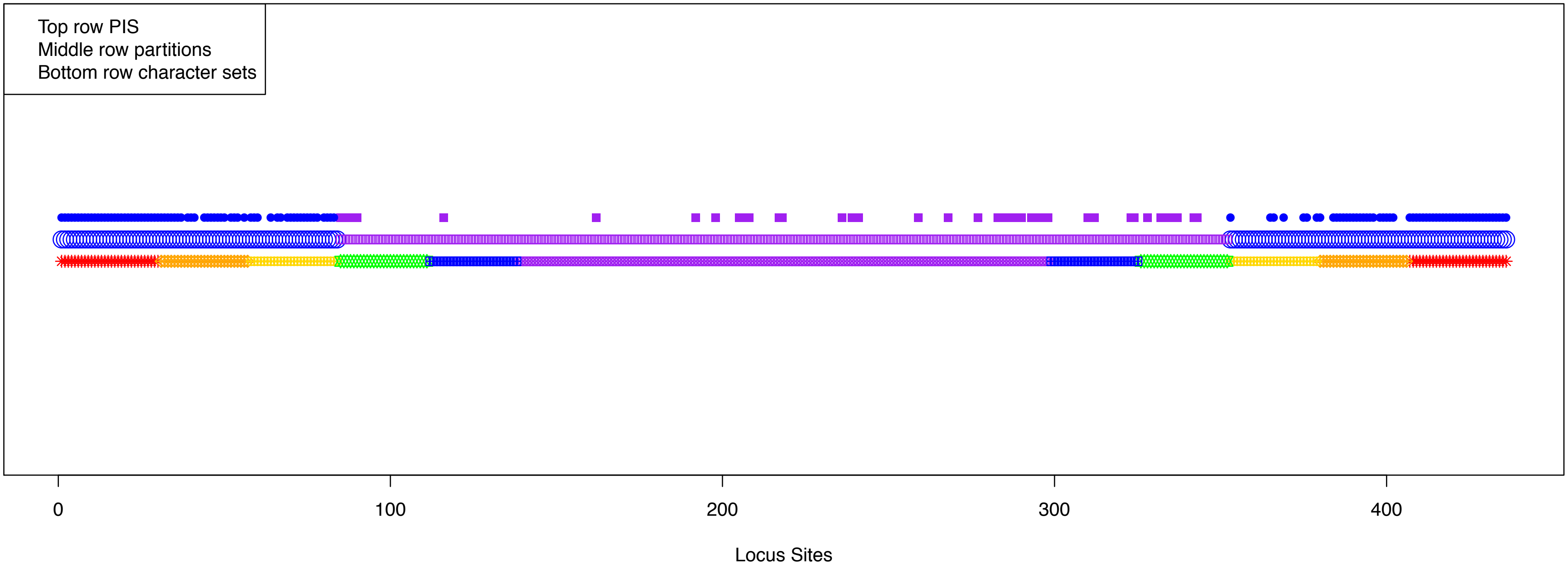

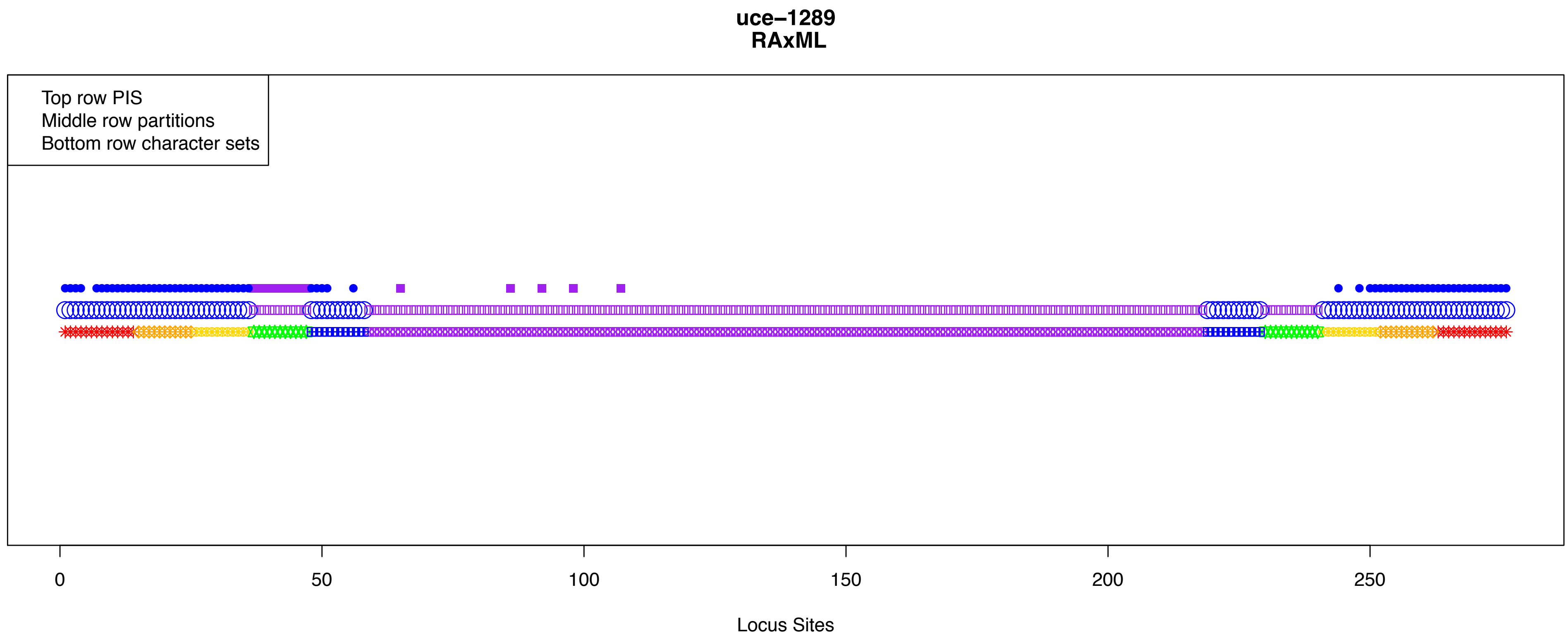
uce–1289
RAxML
Top row PIS
Middle row partitions
Bottom row character sets
0
50
100
150
200
250
Locus Sites

uce−1285
RAxML

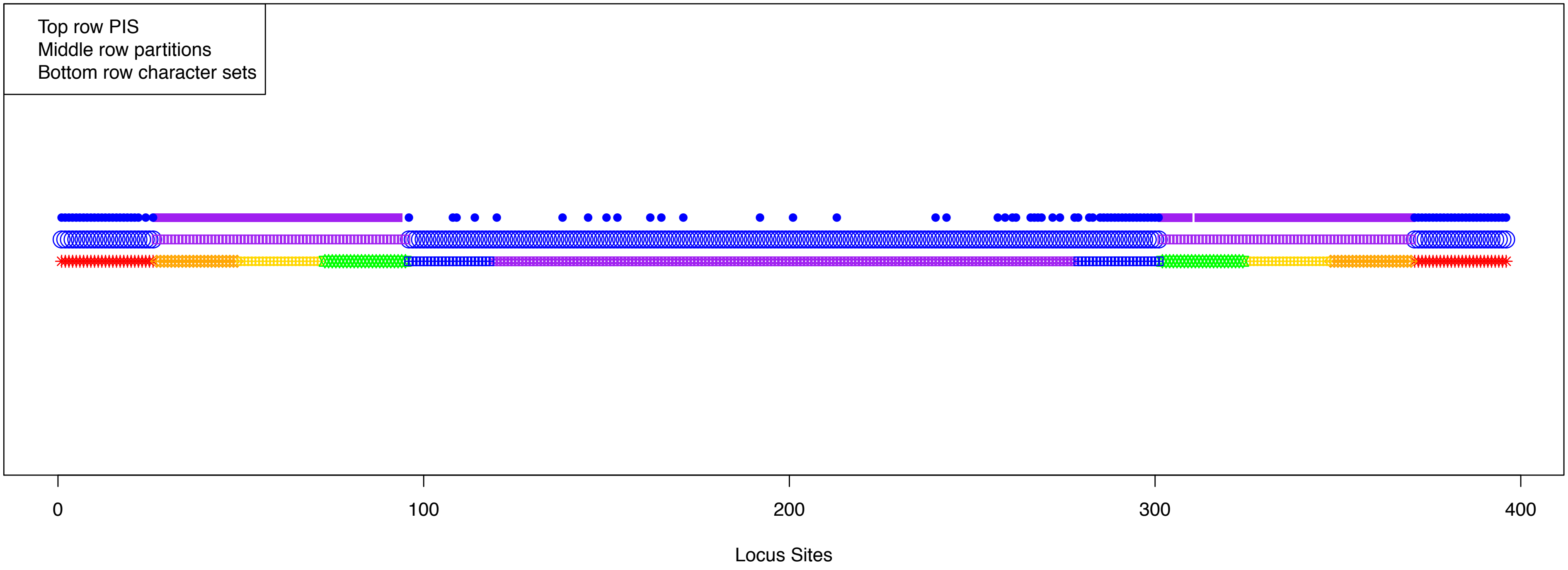

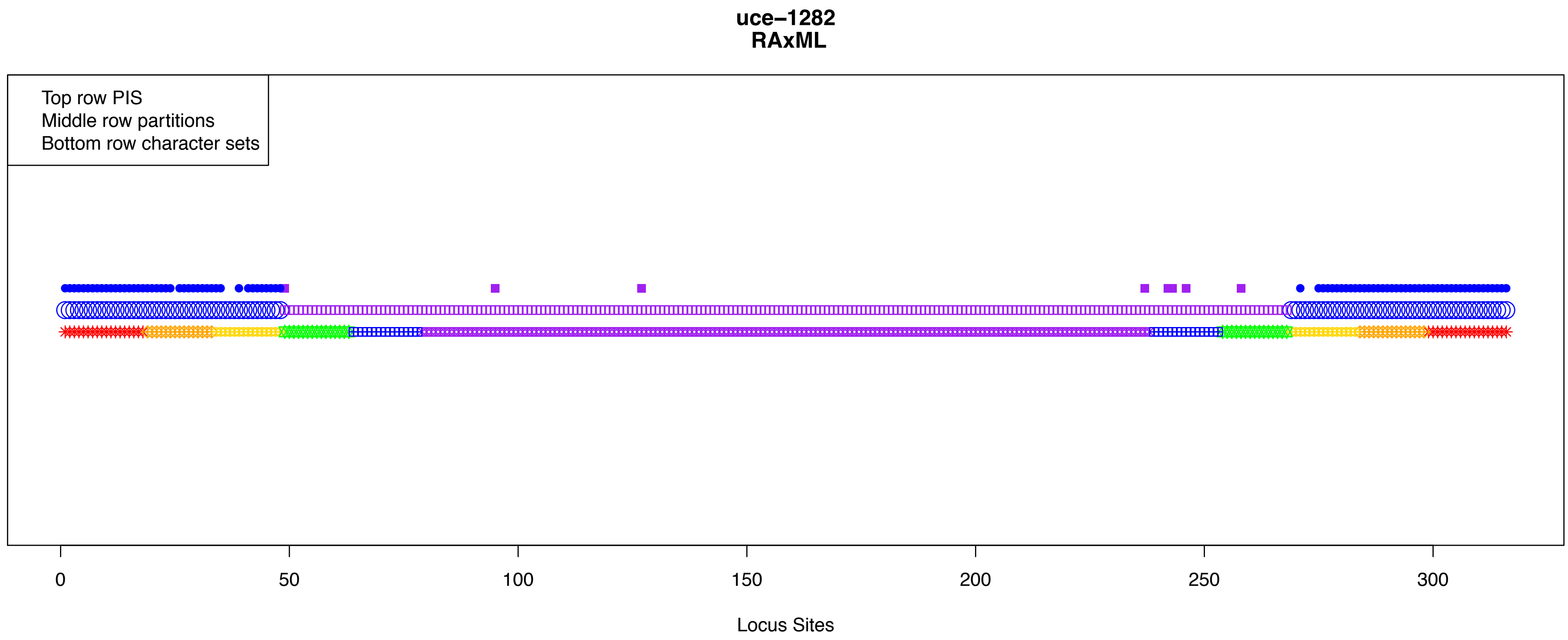

uce–1282
RAxML
Top row PIS
Middle row partitions
Bottom row character sets
0
50
100
150
200
250
300
Locus Sites

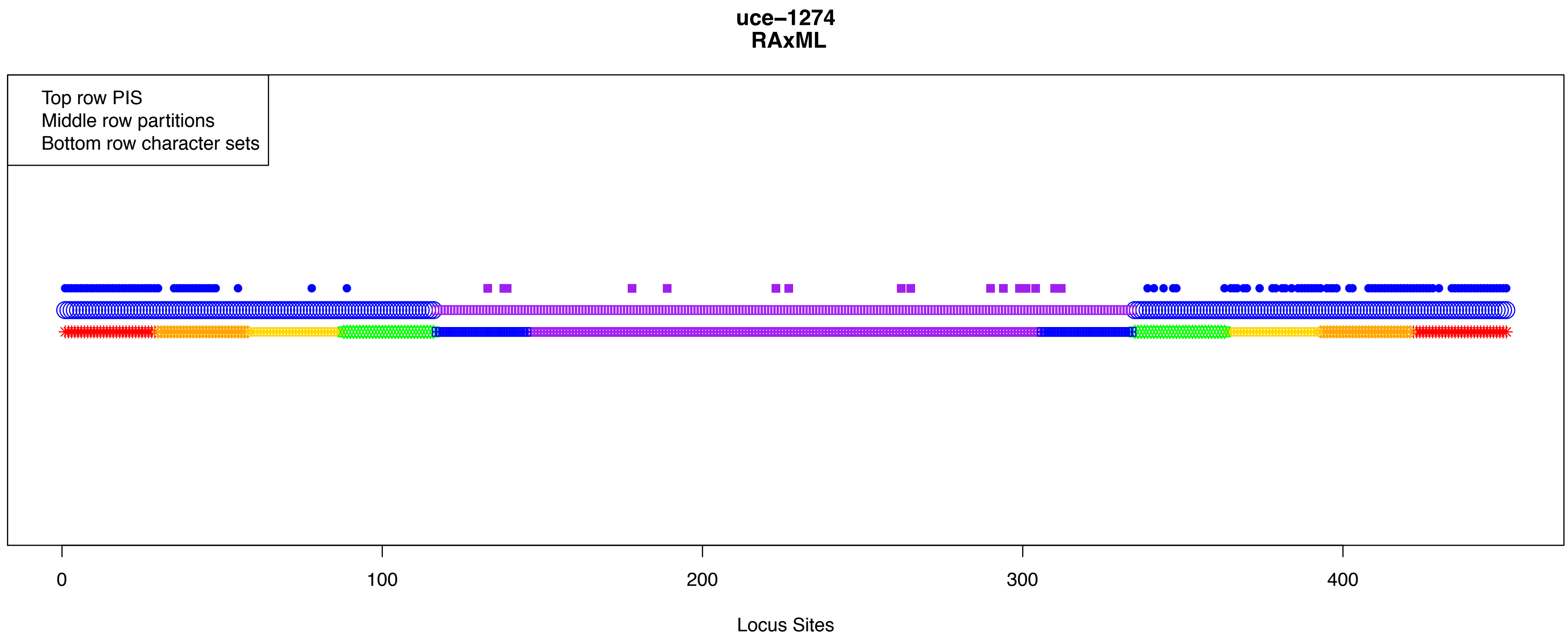
uce–1274
RAxML
Top row PIS
Middle row partitions
Bottom row character sets
0
100
200
300
400
Locus Sites

uce–1271
RAxML

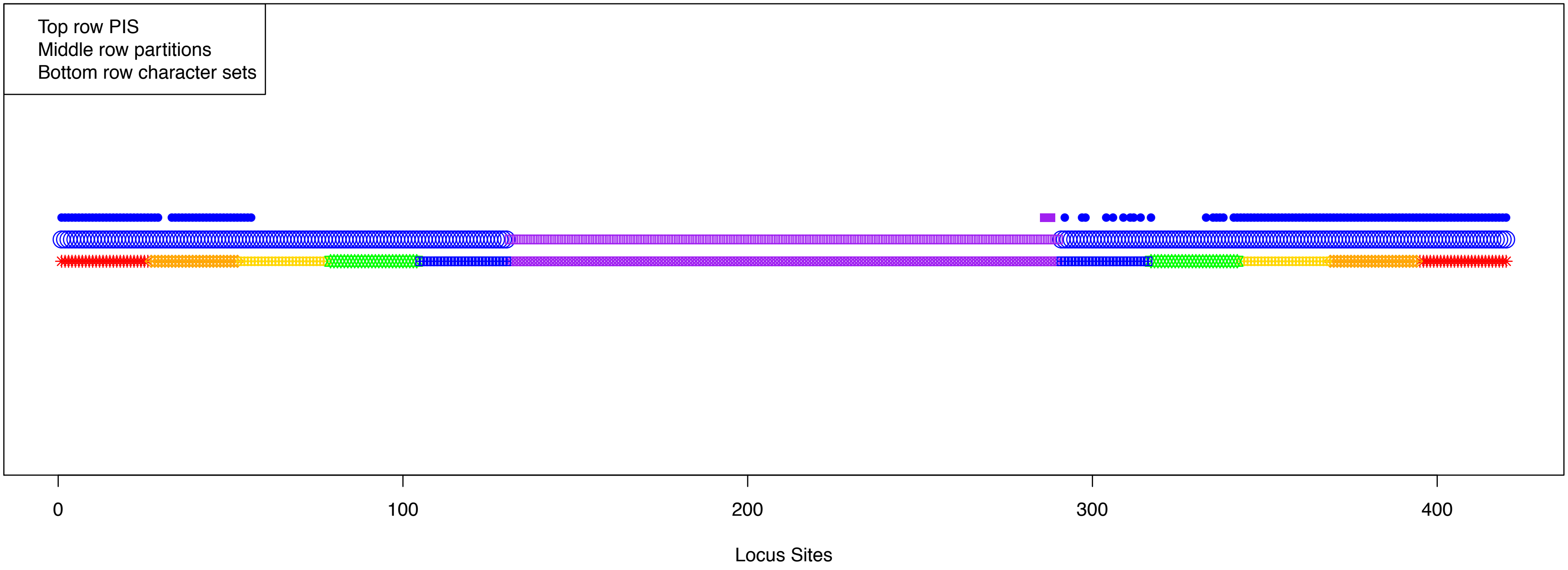

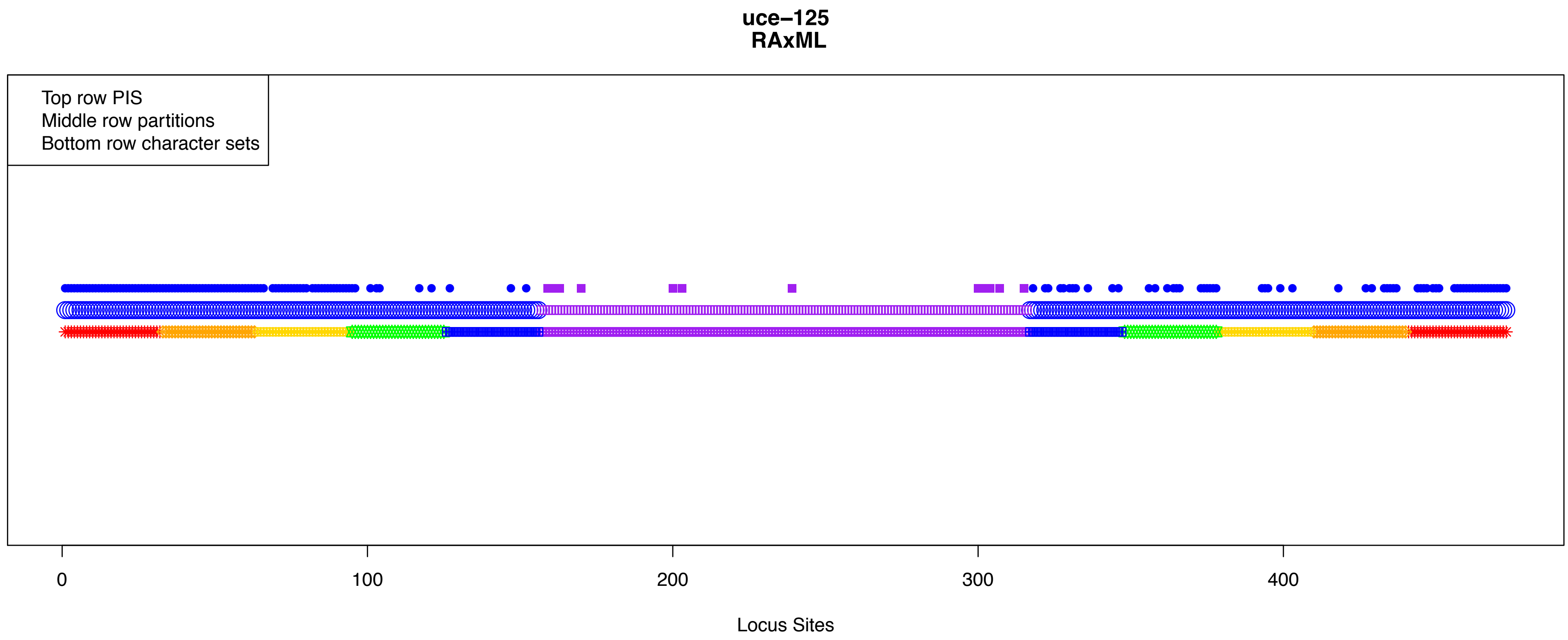
uce–125
RAxML
Top row PIS
Middle row partitions
Bottom row character sets
0
100
200
300
400
Locus Sites

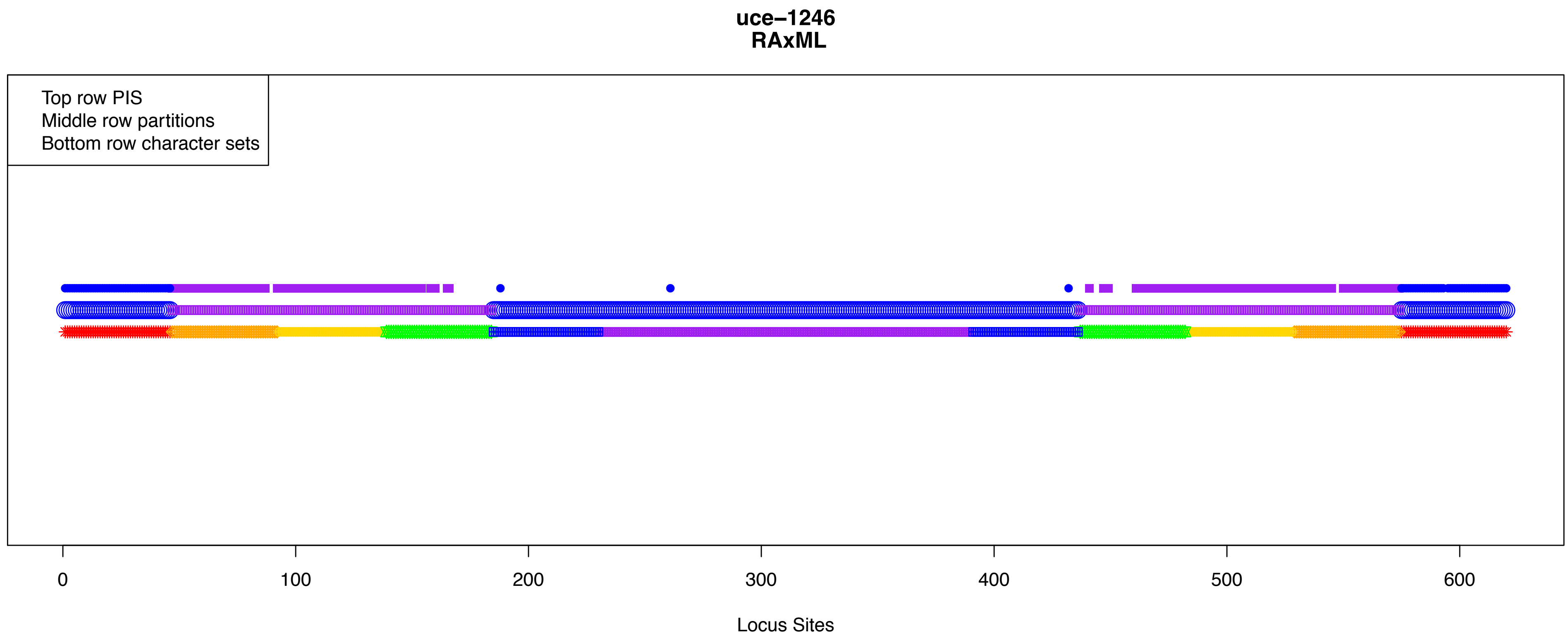

uce–1246
RAxML
Top row PIS
Middle row partitions
Bottom row character sets
0
100
200
300
400
500
600
Locus Sites

uce–1244
RAxML

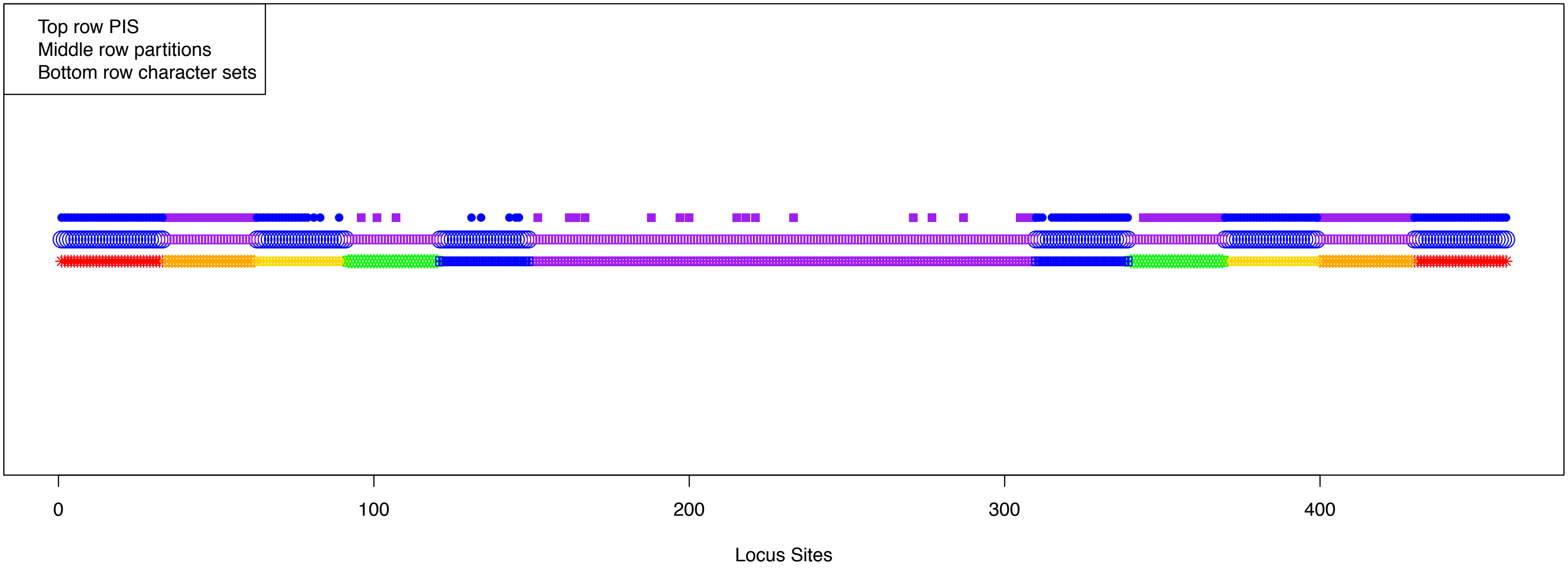

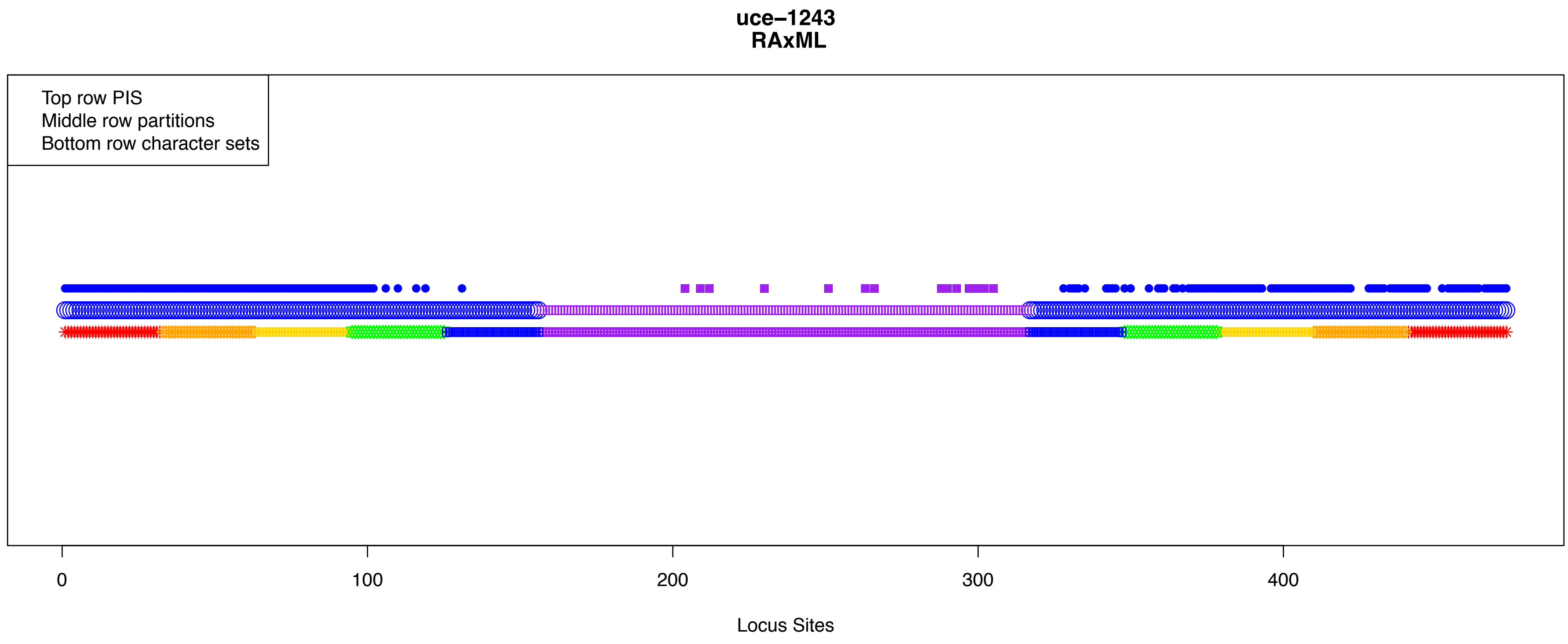
uce–1243
RAxML
Top row PIS
Middle row partitions
Bottom row character sets
0
100
200
300
400
Locus Sites

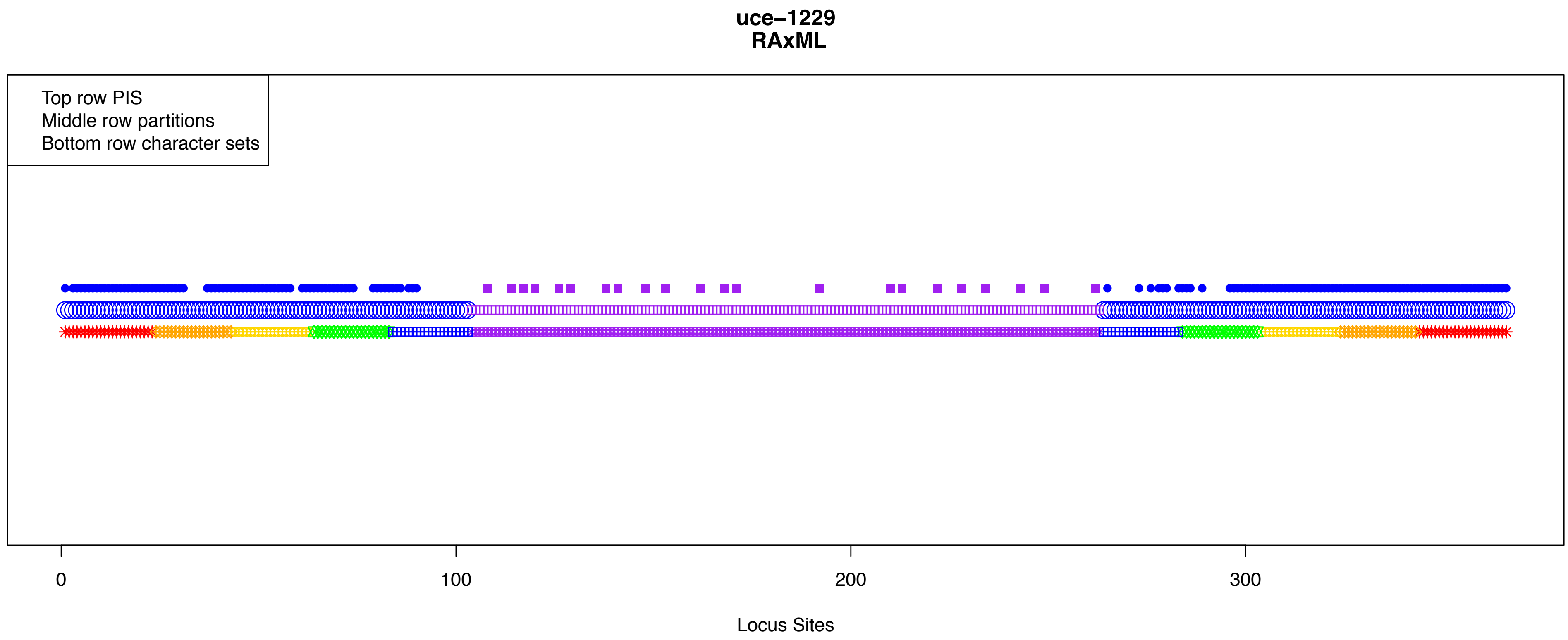

uce–1229
RAxML
Top row PIS
Middle row partitions
Bottom row character sets
0
100
200
300
Locus Sites

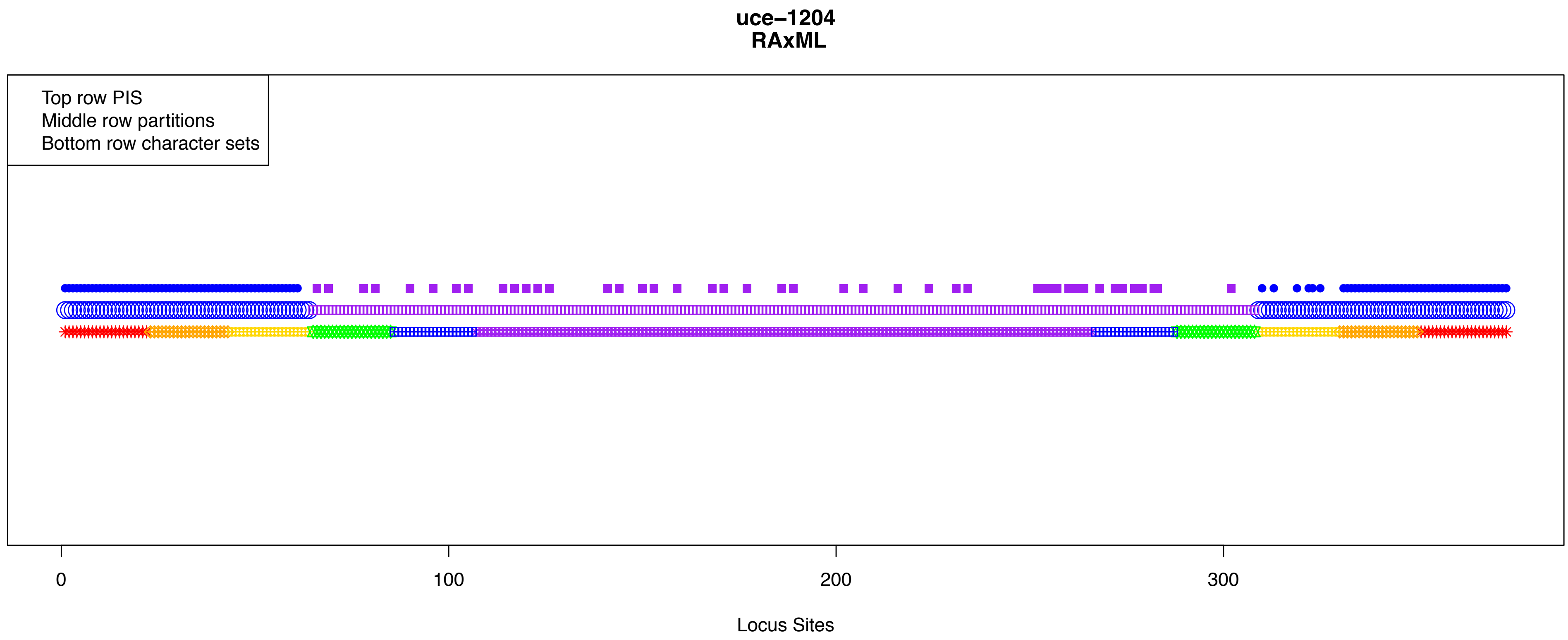

uce–1204
RAxML
Top row PIS
Middle row partitions
Bottom row character sets
0
100
200
300
Locus Sites

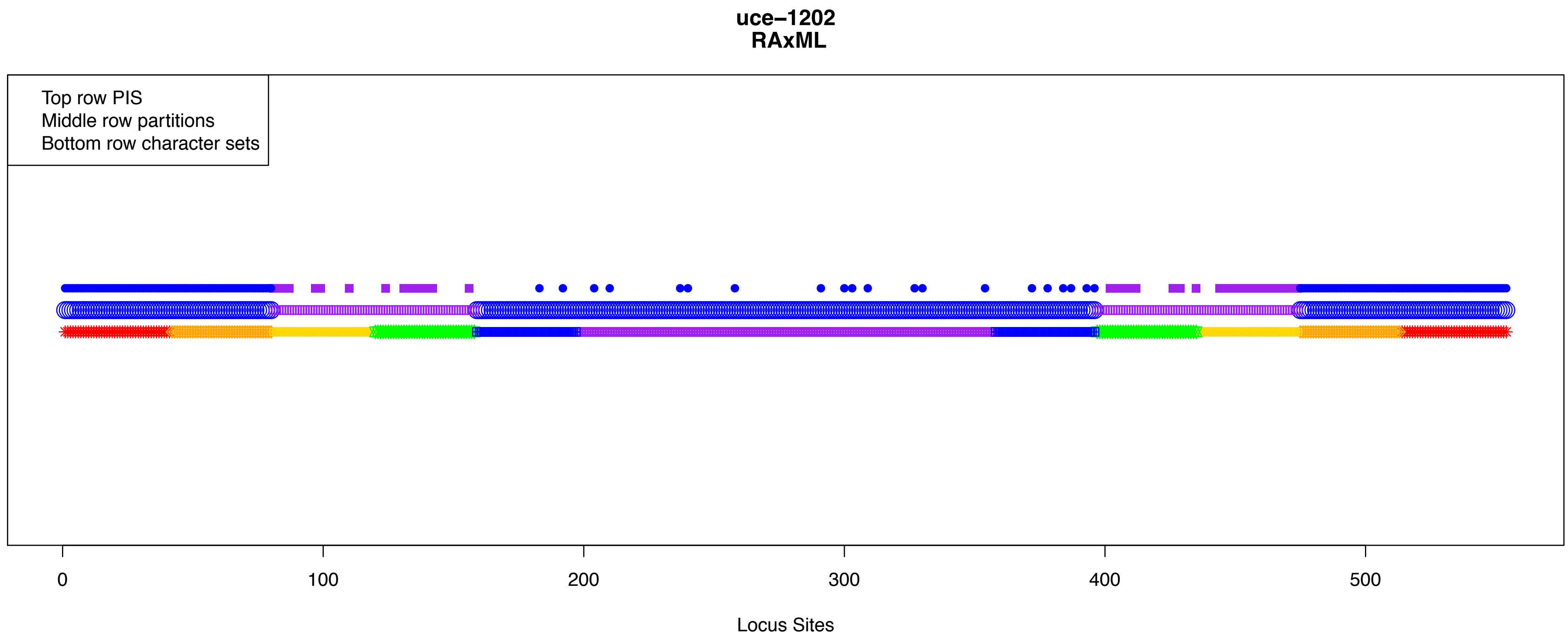
uce–1202
RAxML
Top row PIS
Middle row partitions
Bottom row character sets
0
100
200
300
400
500
Locus Sites

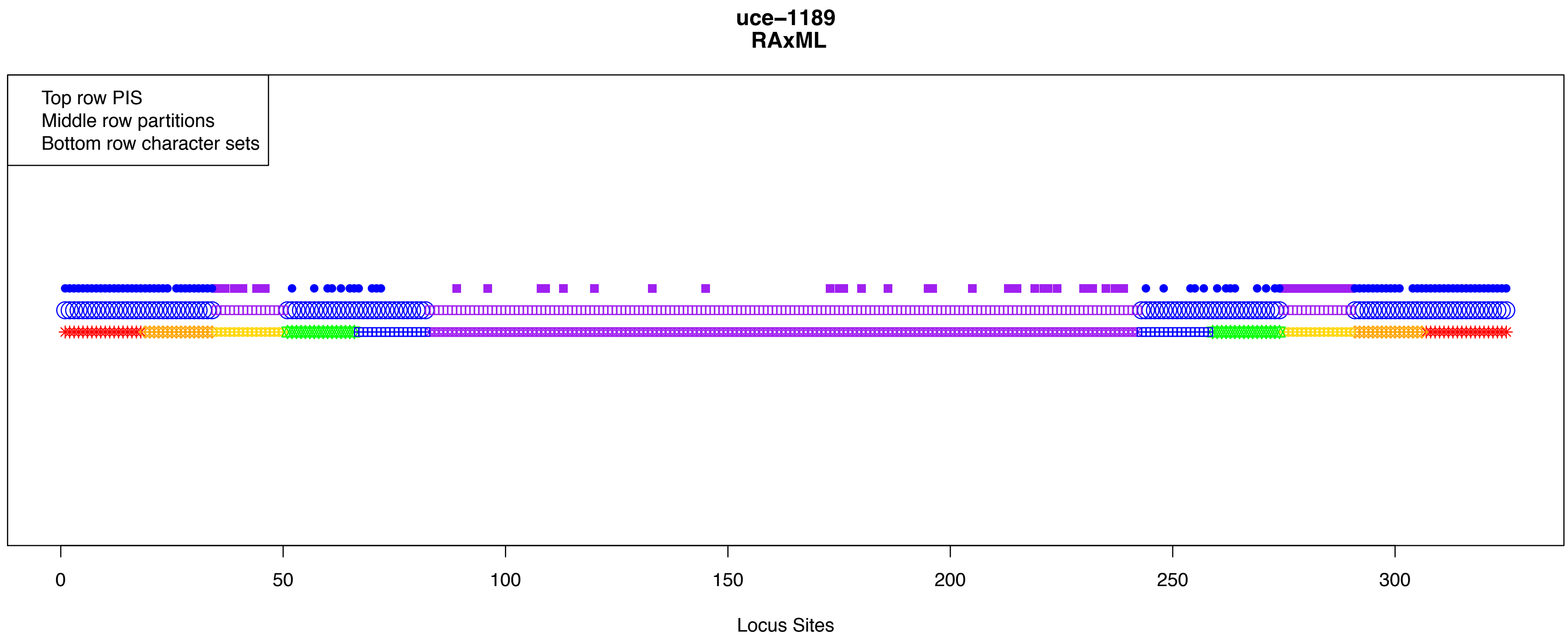
uce–1189
RAxML
Top row PIS
Middle row partitions
Bottom row character sets
0
50
100
150
200
250
300
Locus Sites

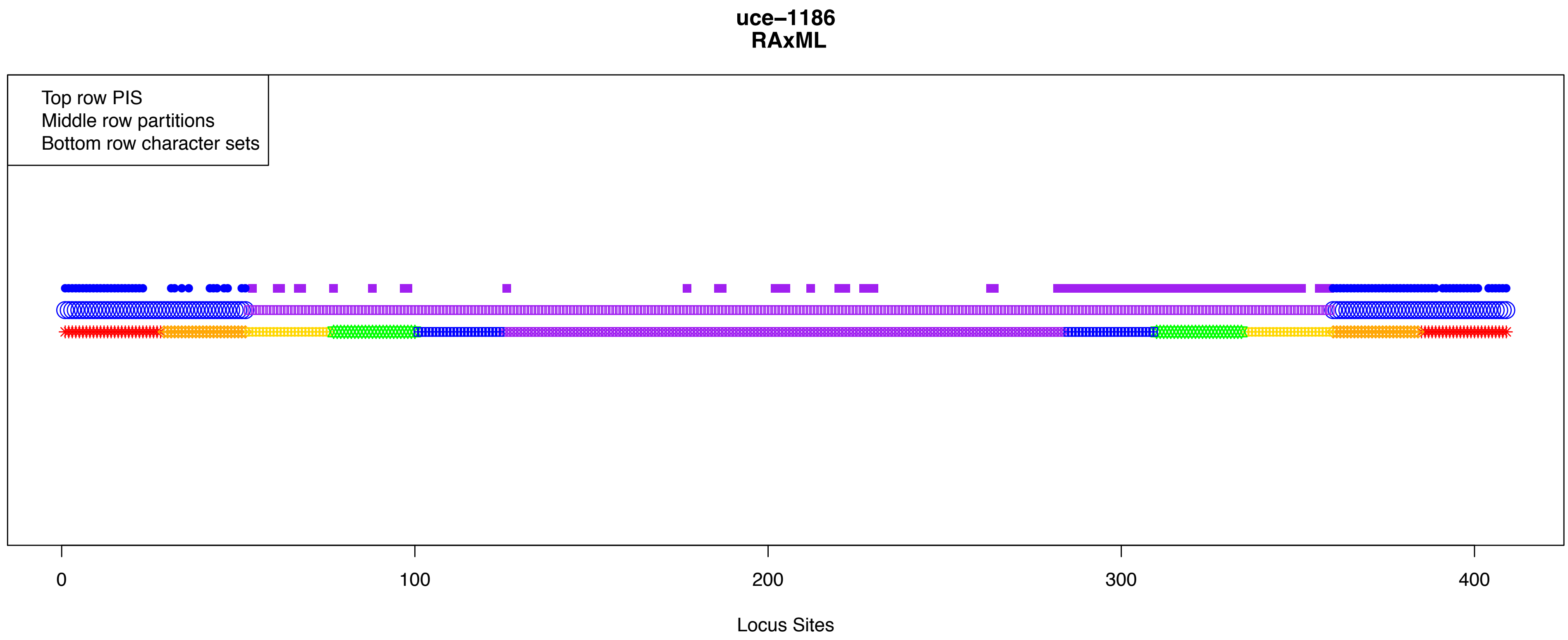

uce–1186
RAxML
Top row PIS
Middle row partitions
Bottom row character sets
0
100
200
300
400
Locus Sites

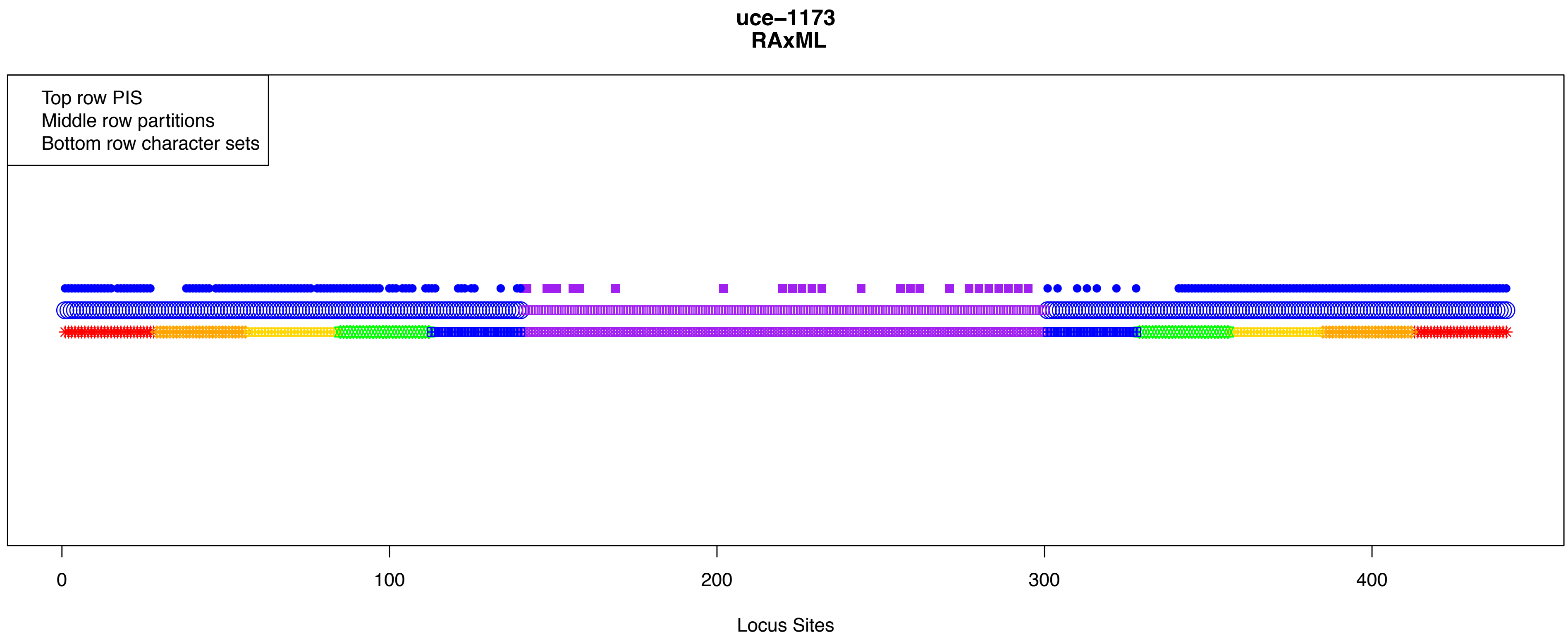

uce–1173
RAxML
Top row PIS
Middle row partitions
Bottom row character sets
0
100
200
300
400
Locus Sites

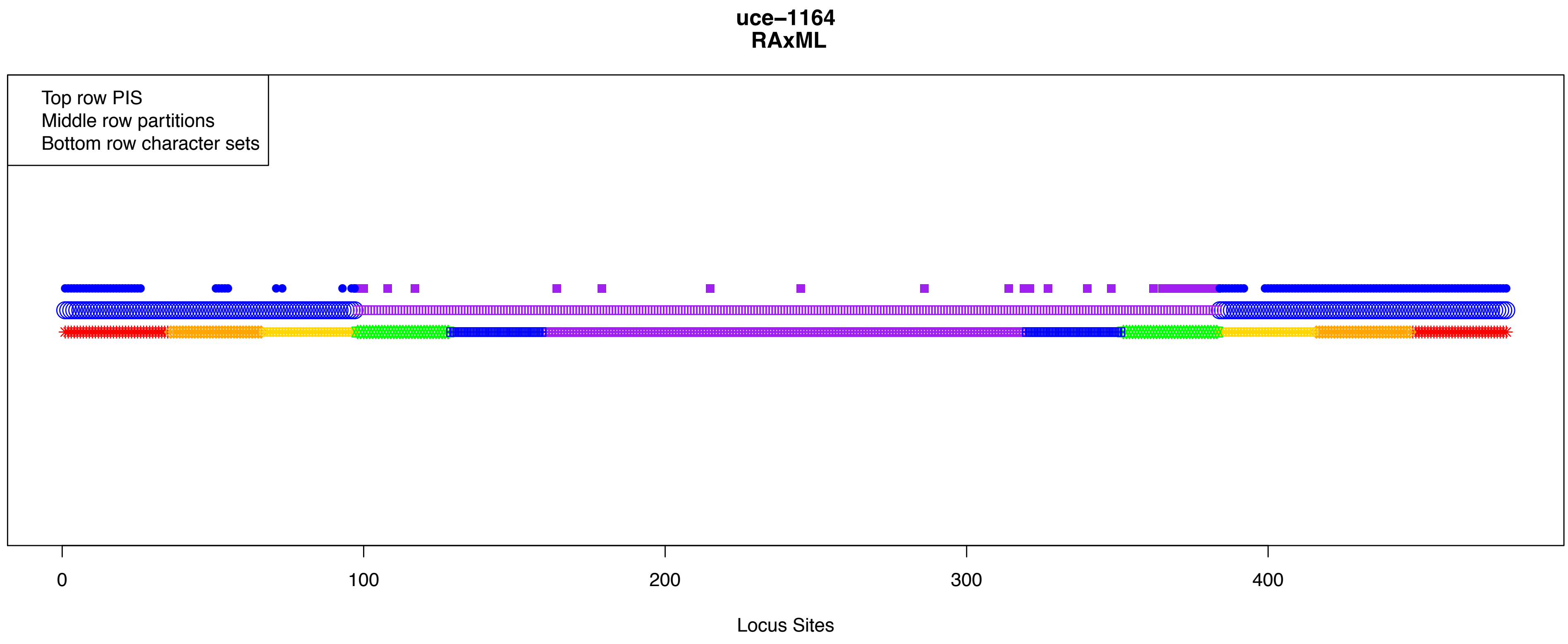
uce–1164
RAxML
Top row PIS
Middle row partitions
Bottom row character sets
0
100
200
300
400
Locus Sites

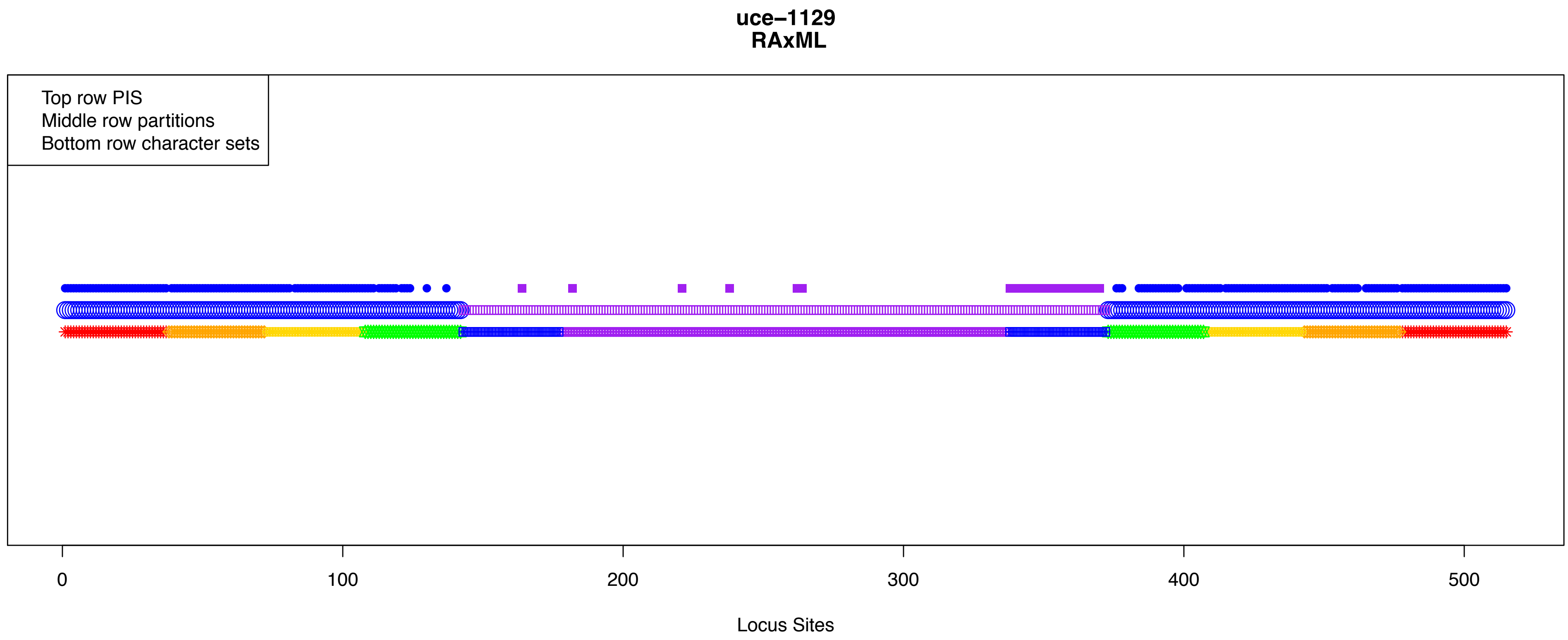
uce–1129
RAxML
Top row PIS
Middle row partitions
Bottom row character sets
0
100
200
300
400
500
Locus Sites

uce–1126
RAxML

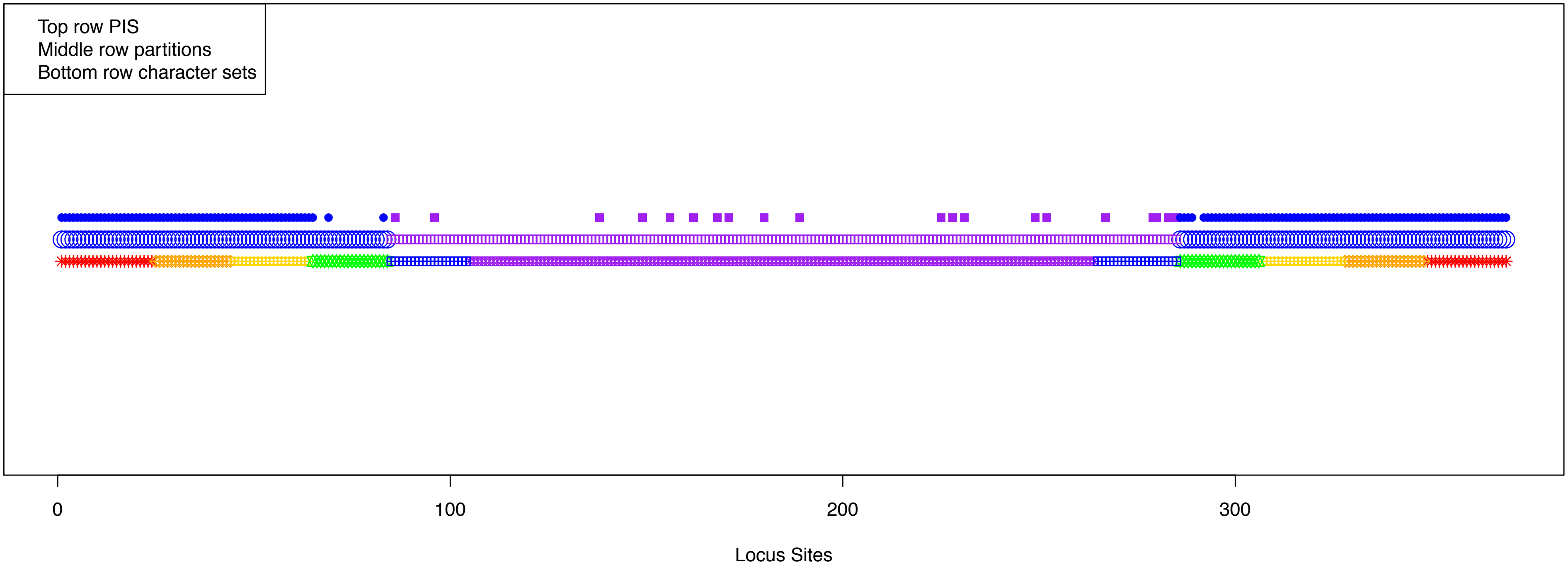

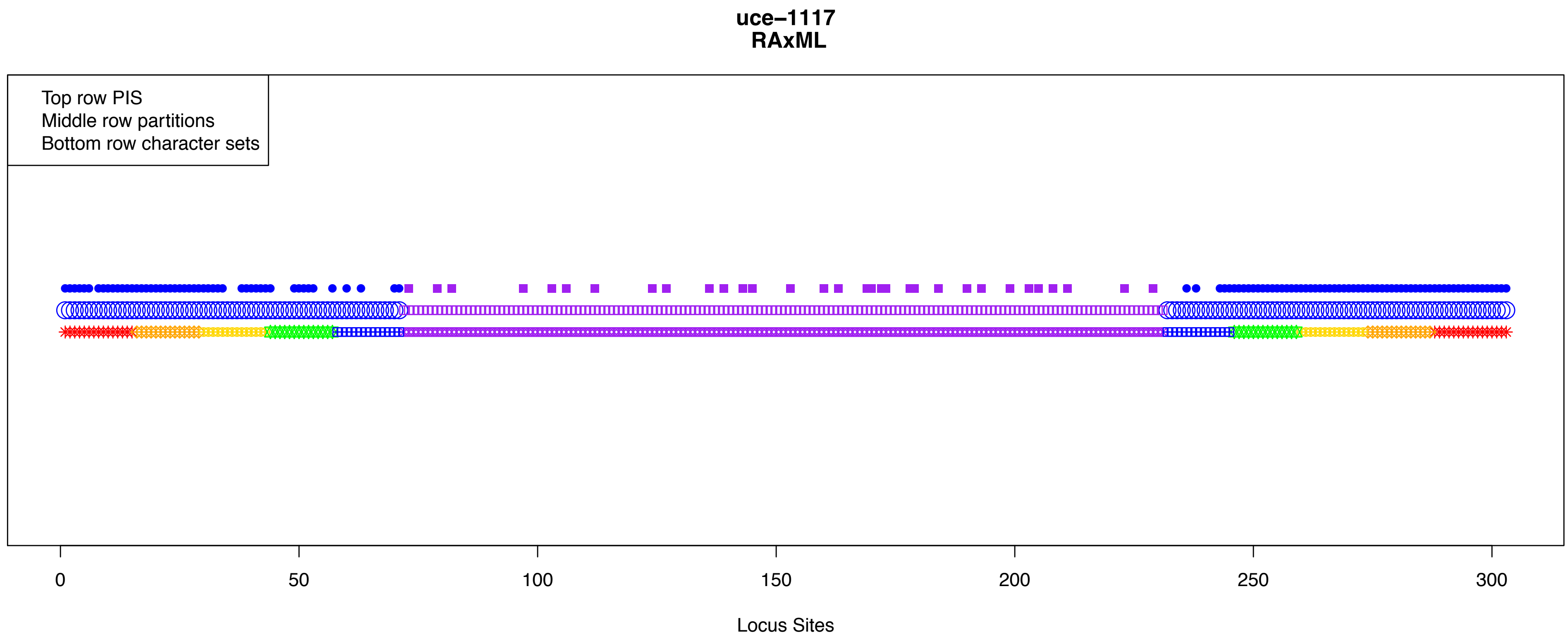
uce–1117
RAxML
Top row PIS
Middle row partitions
Bottom row character sets
0
50
100
150
200
250
300
Locus Sites

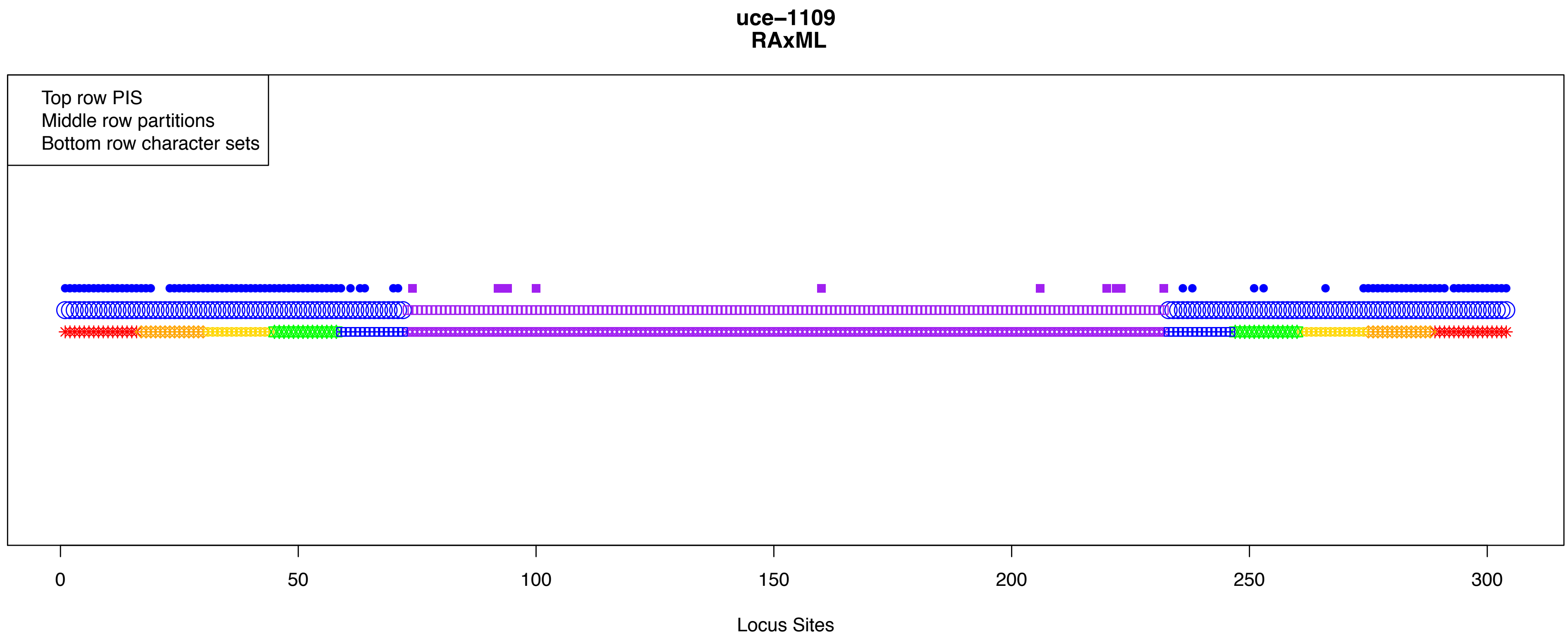
uce–1109
RAxML
Top row PIS
Middle row partitions
Bottom row character sets
0
50
100
150
200
250
300
Locus Sites

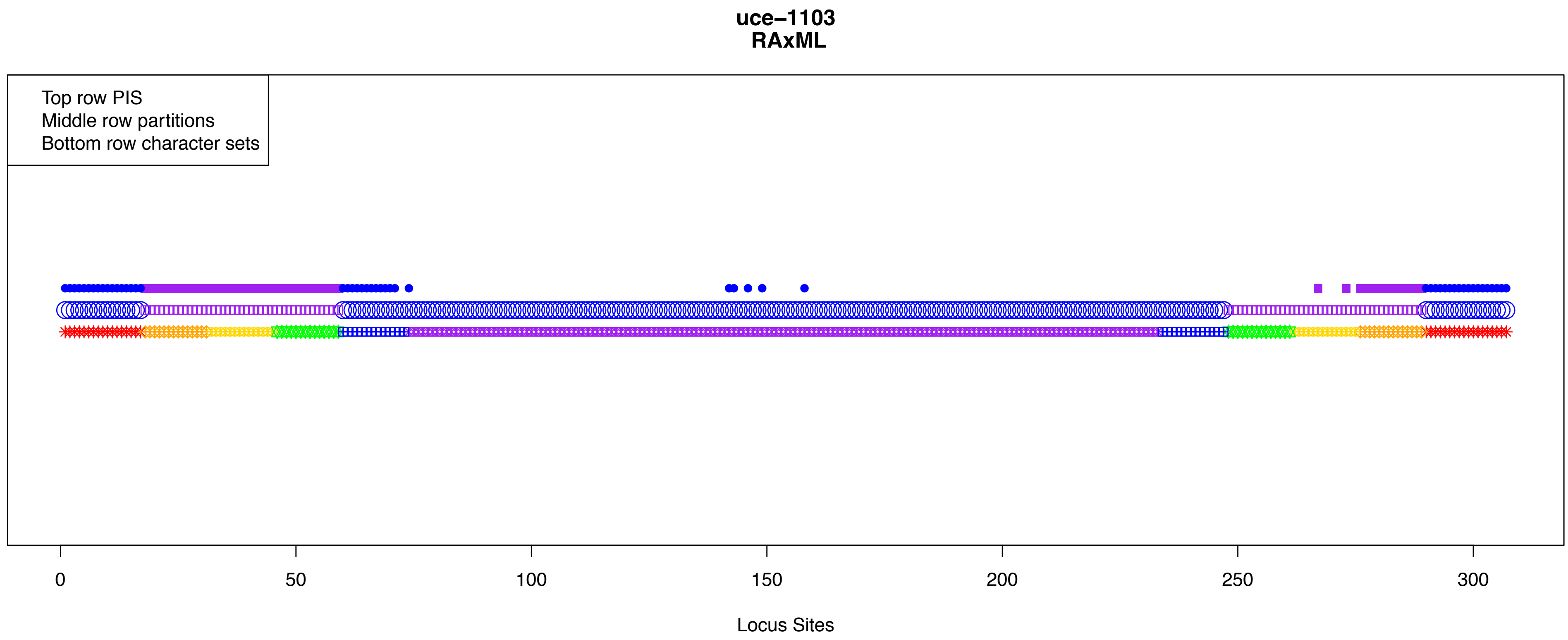

uce–1103
RAxML
Top row PIS
Middle row partitions
Bottom row character sets
0
50
100
150
200
250
300
Locus Sites

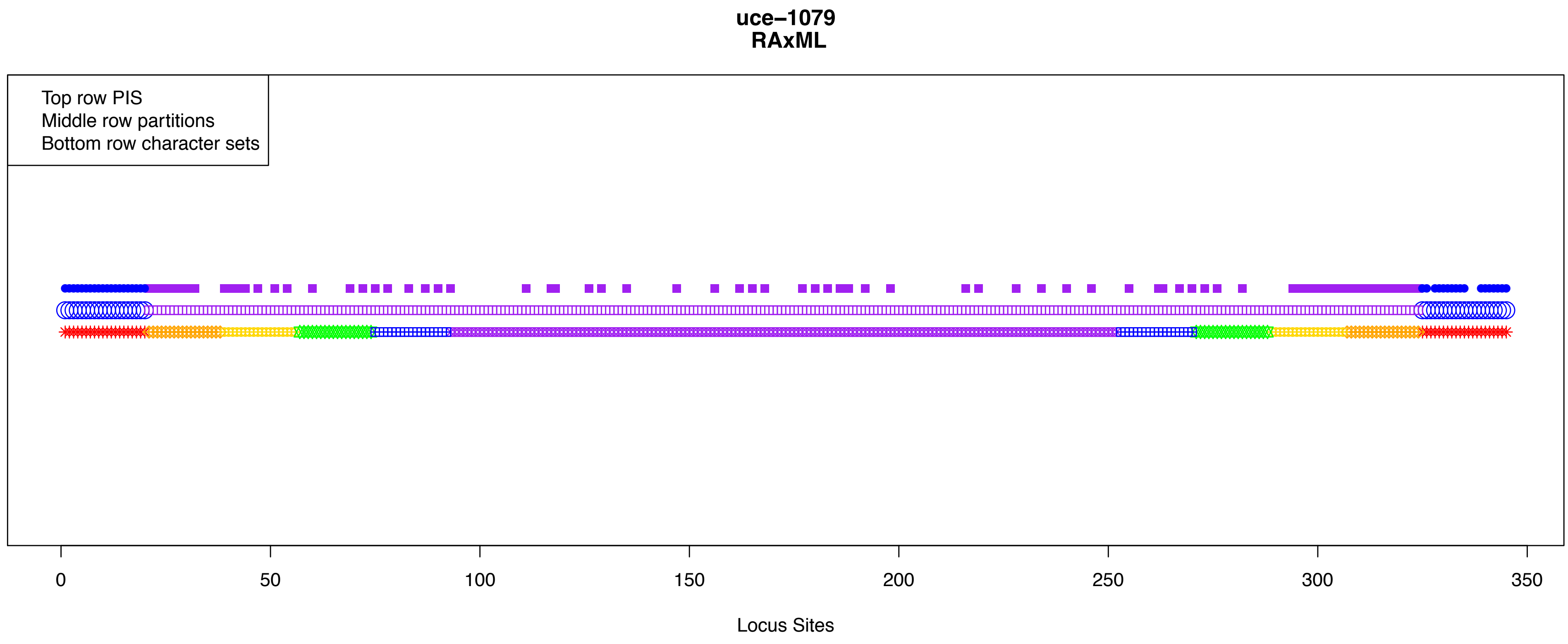
uce–1079
RAxML
Top row PIS
Middle row partitions
Bottom row character sets
0
50
100
150
200
250
300
350
Locus Sites

uce–1075
RAxML

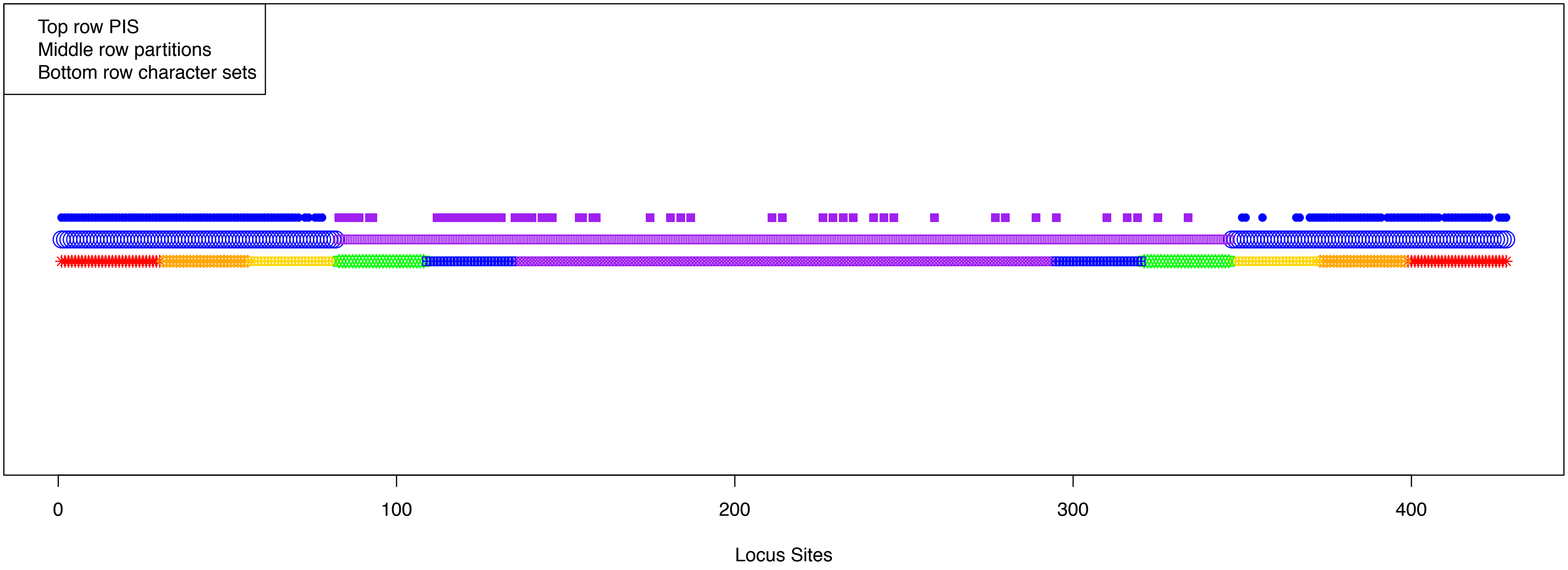

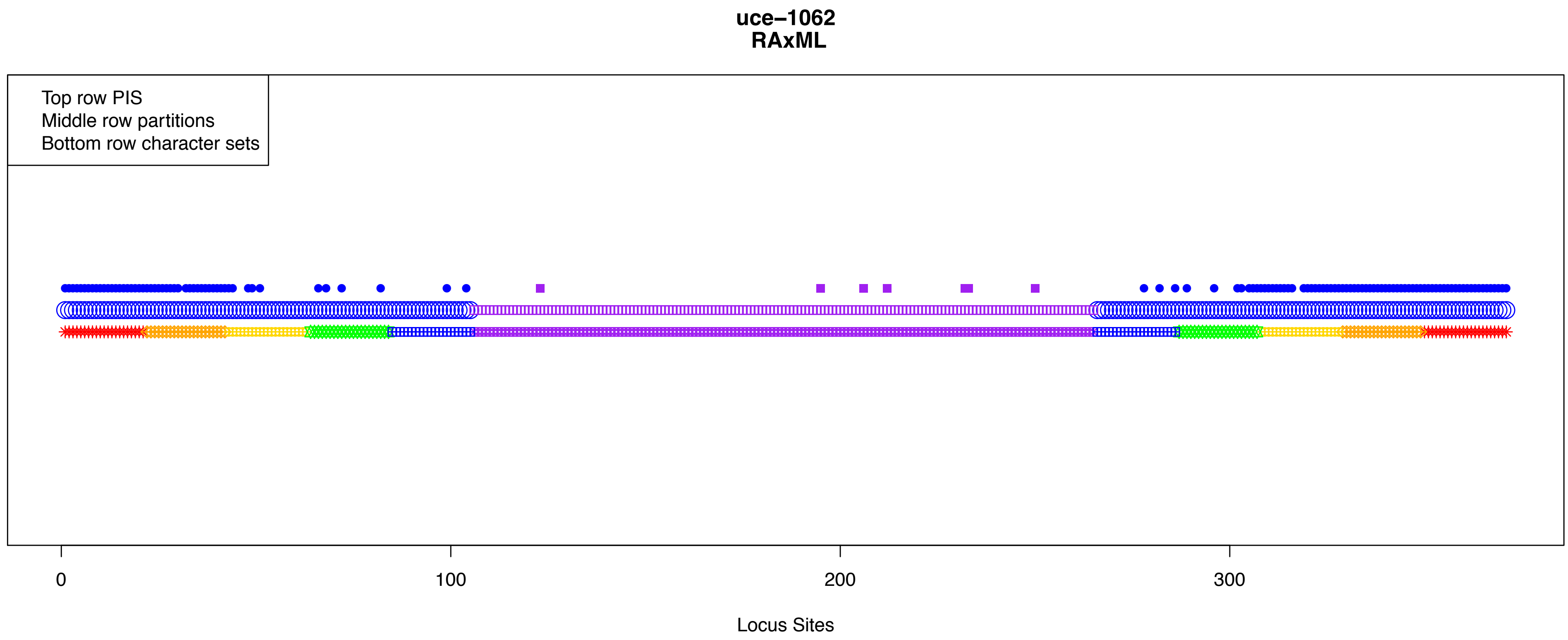
uce–1062
RAxML
Top row PIS
Middle row partitions
Bottom row character sets
0
100
200
300
Locus Sites

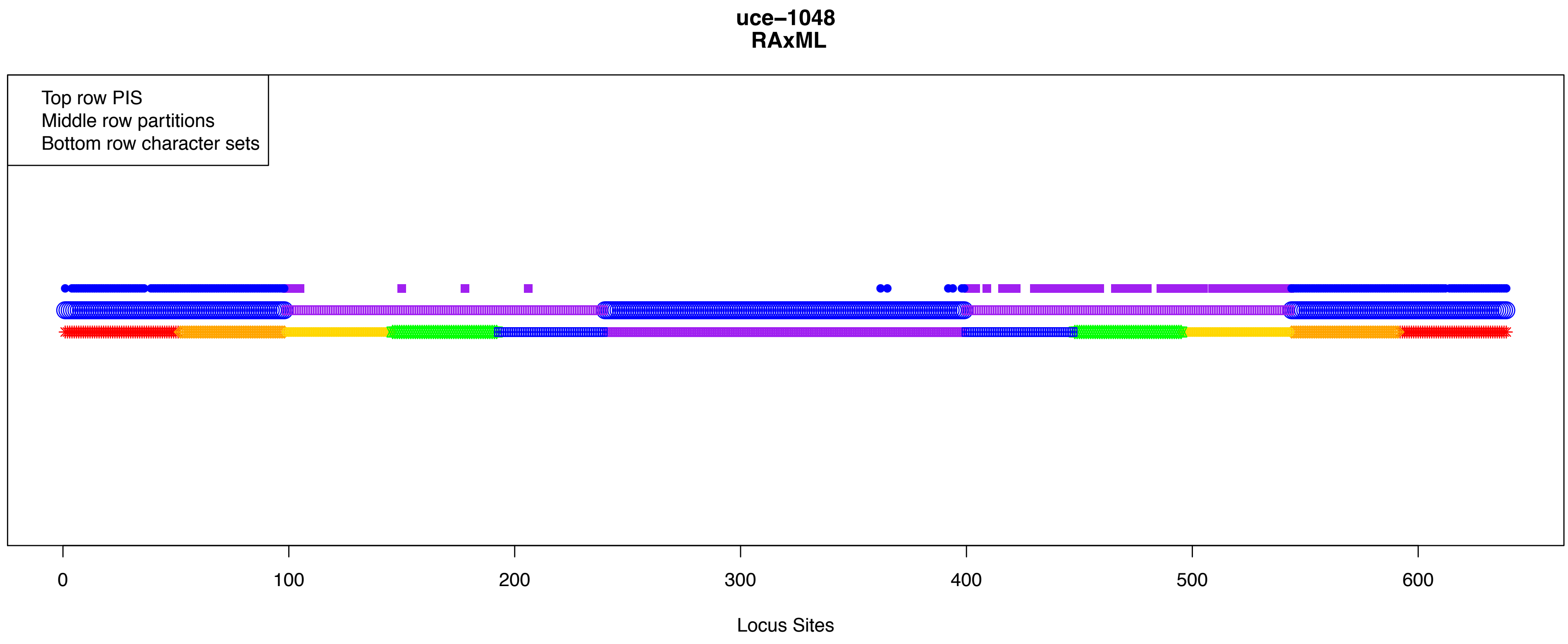
uce–1048
RAxML
Top row PIS
Middle row partitions
Bottom row character sets
0
100
200
300
400
500
600
Locus Sites

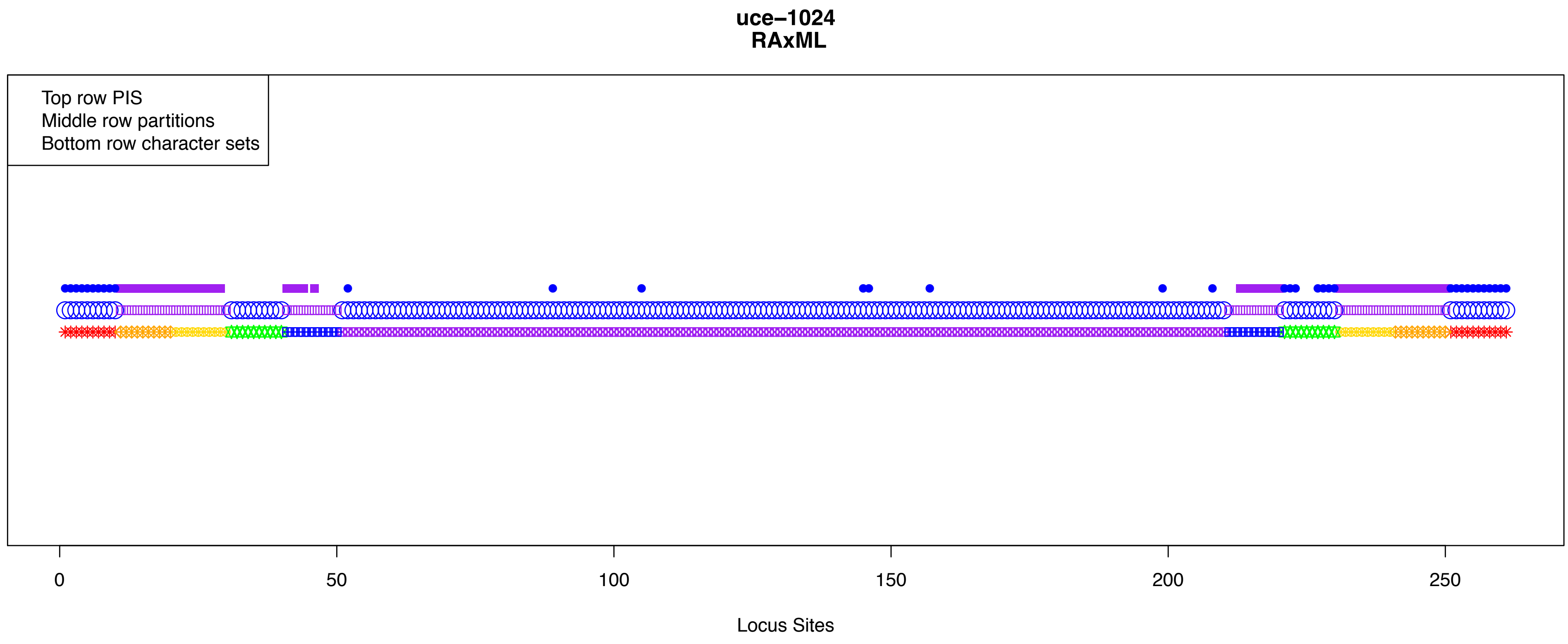
uce–1024
RAxML
Top row PIS
Middle row partitions
Bottom row character sets
0
50
100
150
200
250
Locus Sites

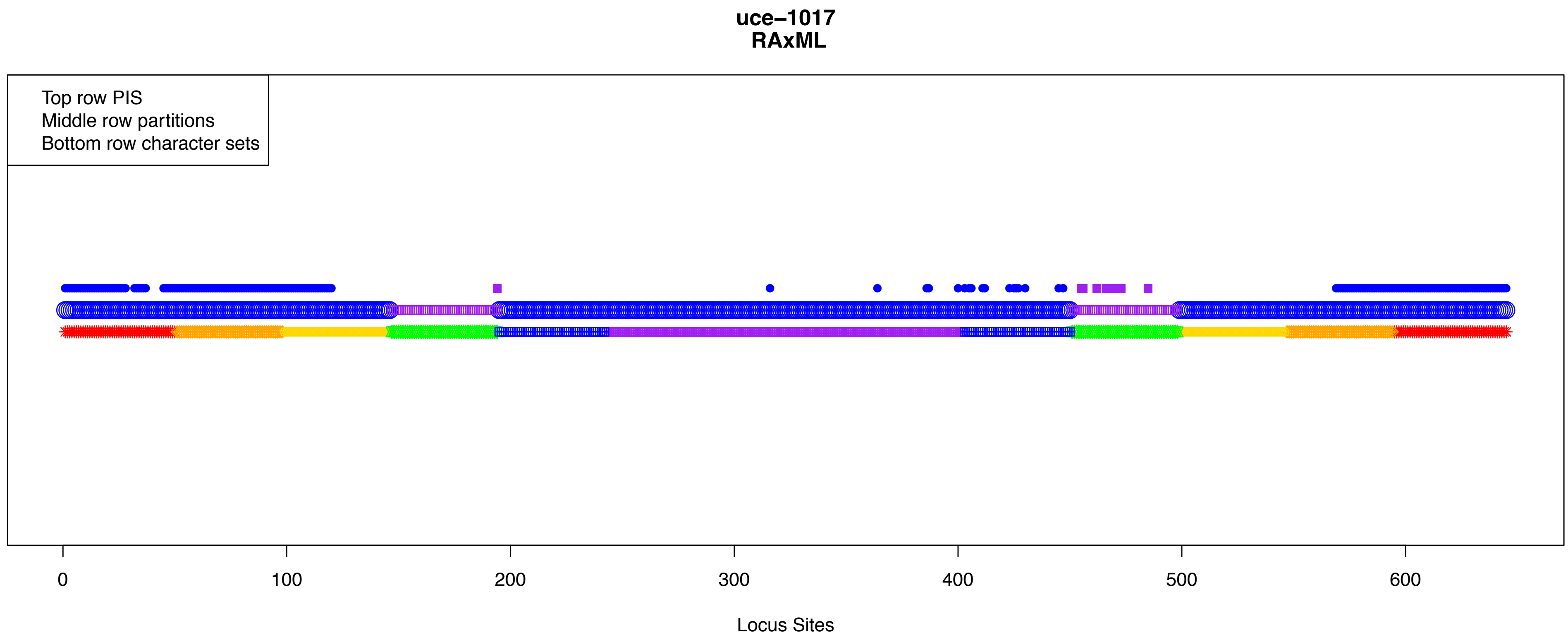
uce–1017
RAxML
Top row PIS
Middle row partitions
Bottom row character sets
0
100
200
300
400
500
600
Locus Sites

uce–1014
RAxML

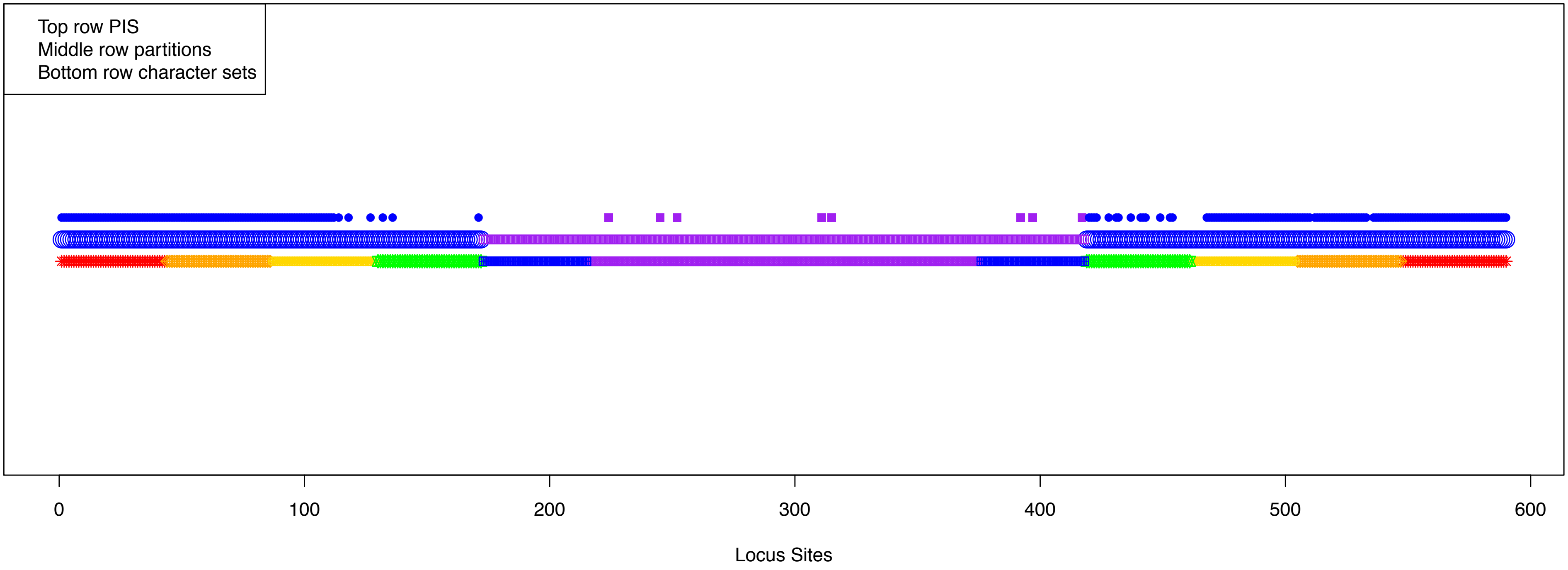

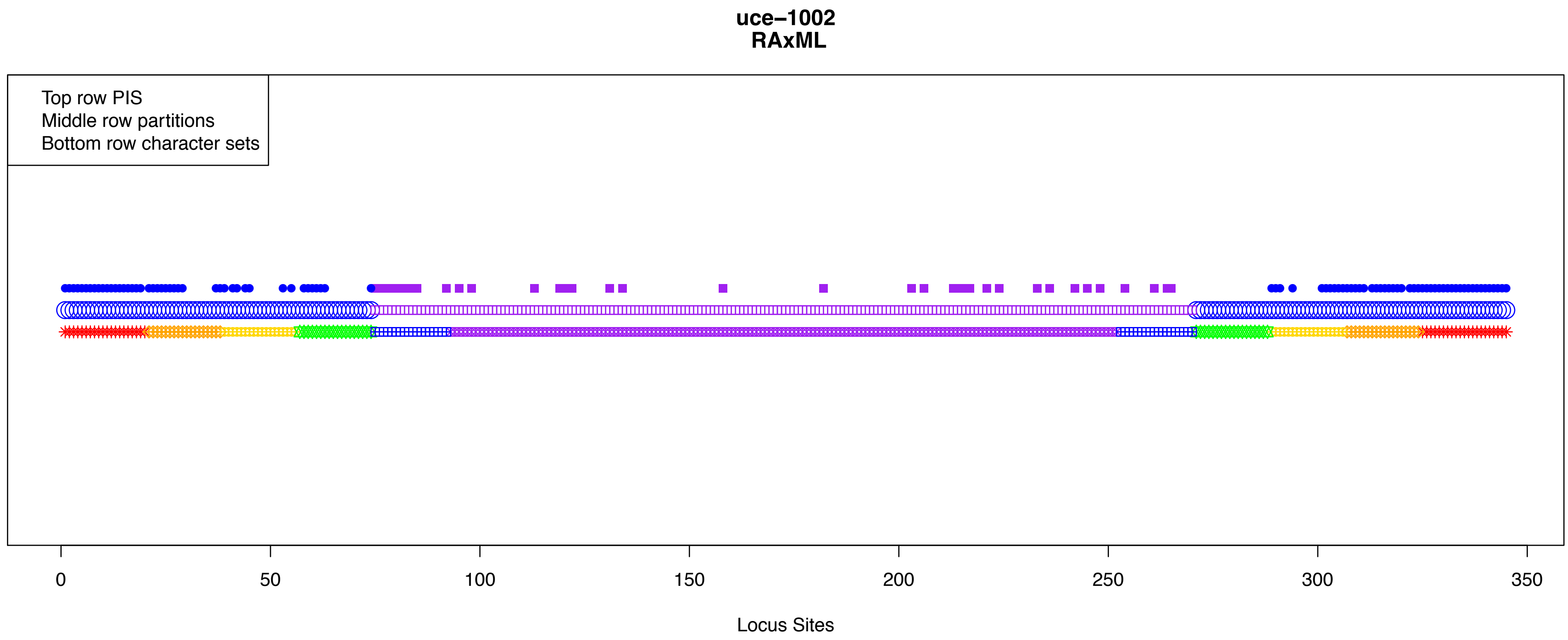

uce–1002
RAxML
Top row PIS
Middle row partitions
Bottom row character sets
0
50
100
150
200
250
300
350
Locus Sites
