# Supplementary material for: Ultraconserved elements (UCEs) resolve the phylogeny of Australasian smurf-weevils
Source: PLoS One. 2017 Nov 22;12(11):e0188044. doi: 10.1371/journal.pone.0188044 (PMC5699822; doi:10.1371/journal.pone.0188044)
Supplement: S1 File — (ZIP) [file pone.0188044.s007.zip › Supplemental_Partition_Number_of_partitions_PIS_Charsets/partitions3-RAxML.pdf]

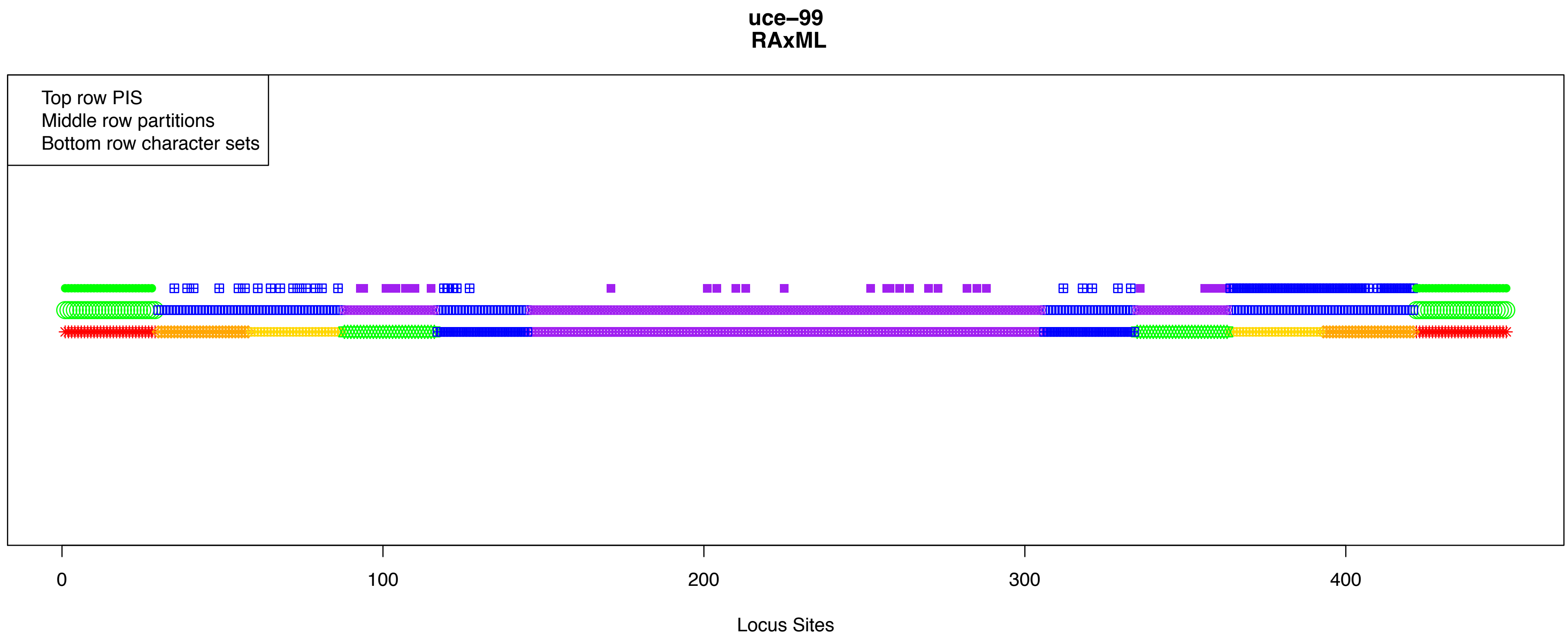
uce–99
RAxML
Top row PIS
Middle row partitions
Bottom row character sets
0
100
200
300
400
Locus Sites

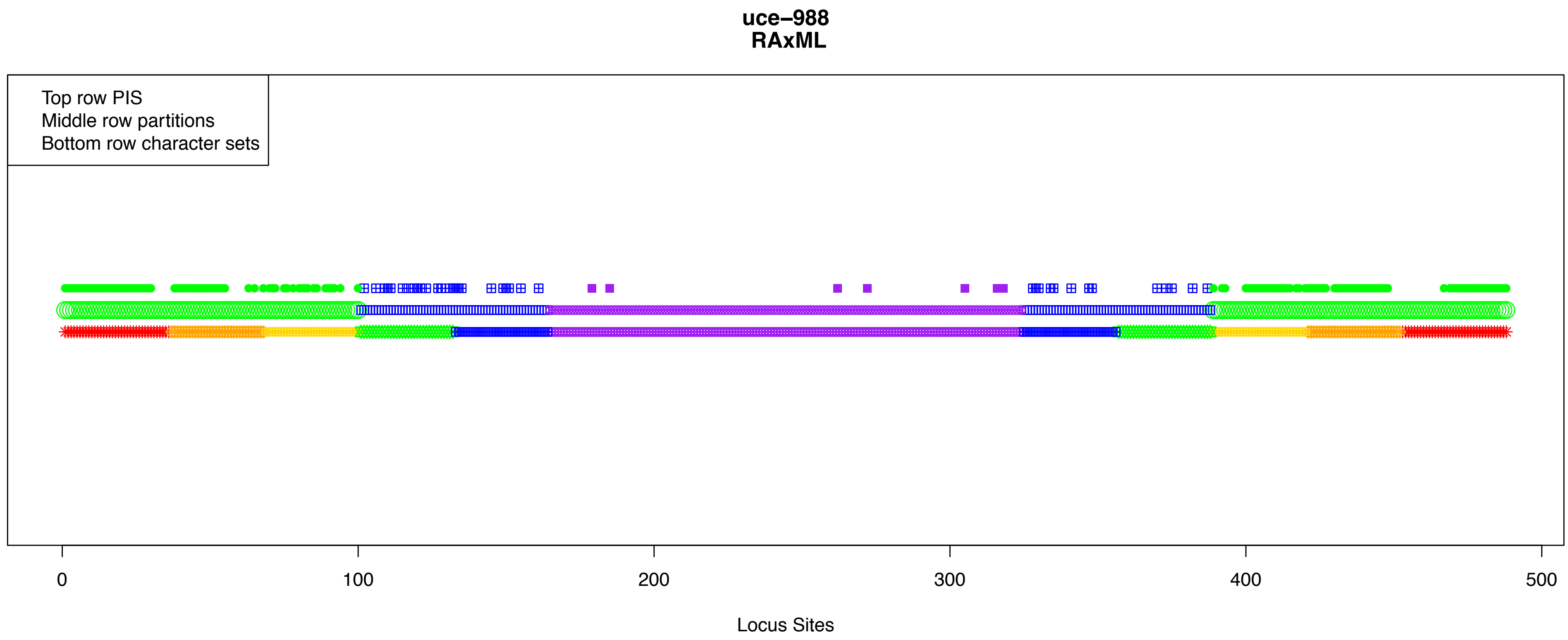

uce–988
RAxML
Top row PIS
Middle row partitions
Bottom row character sets
0
100
200
300
400
500
Locus Sites

uce–939
RAxML

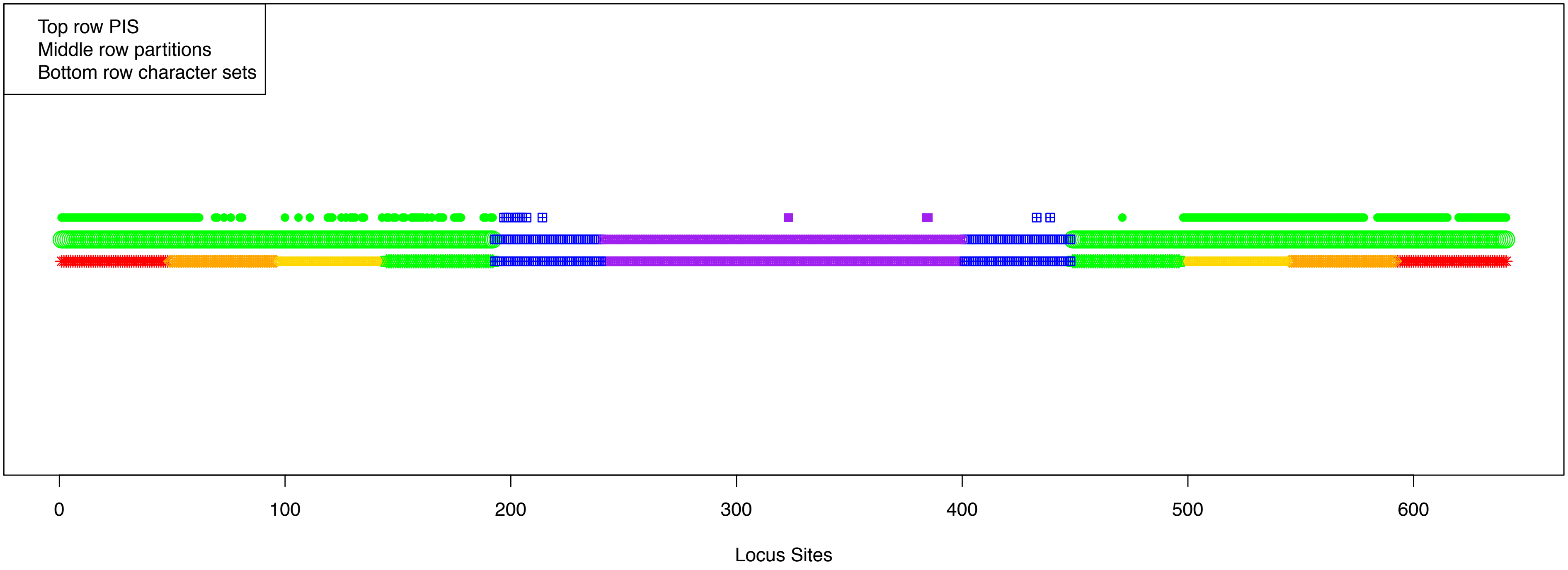

uce–926
RAxML

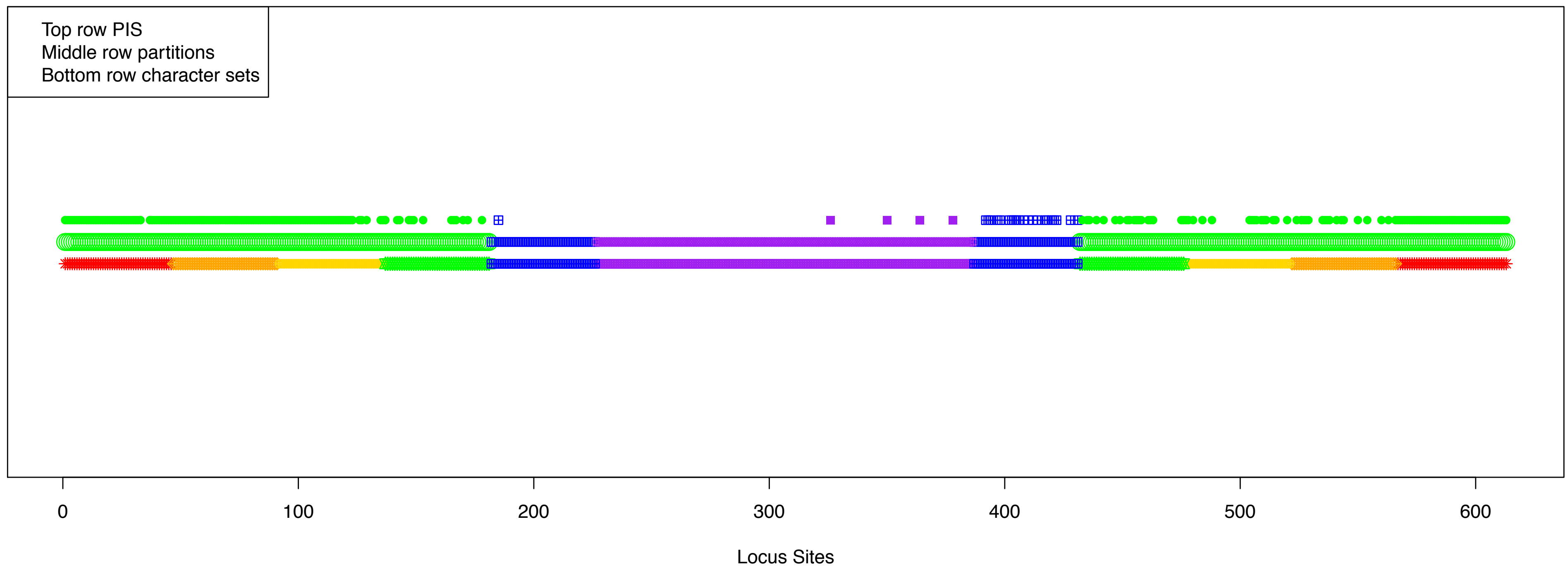

uce–924
RAxML

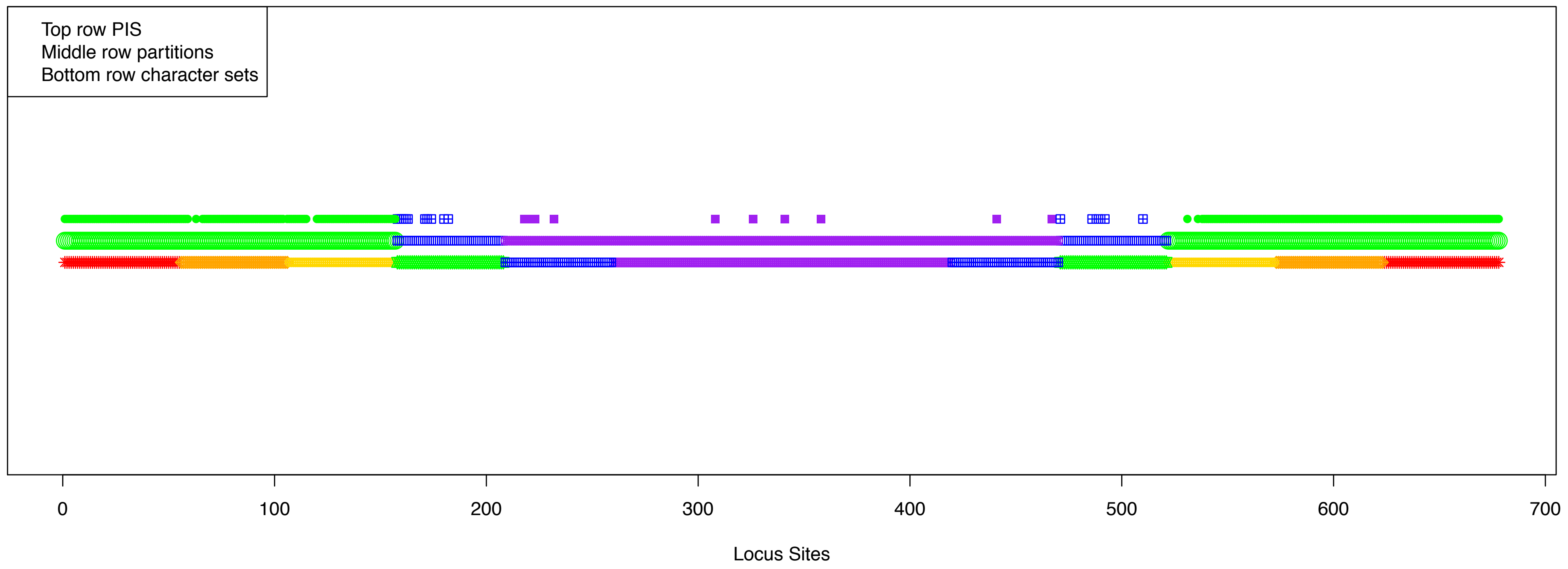

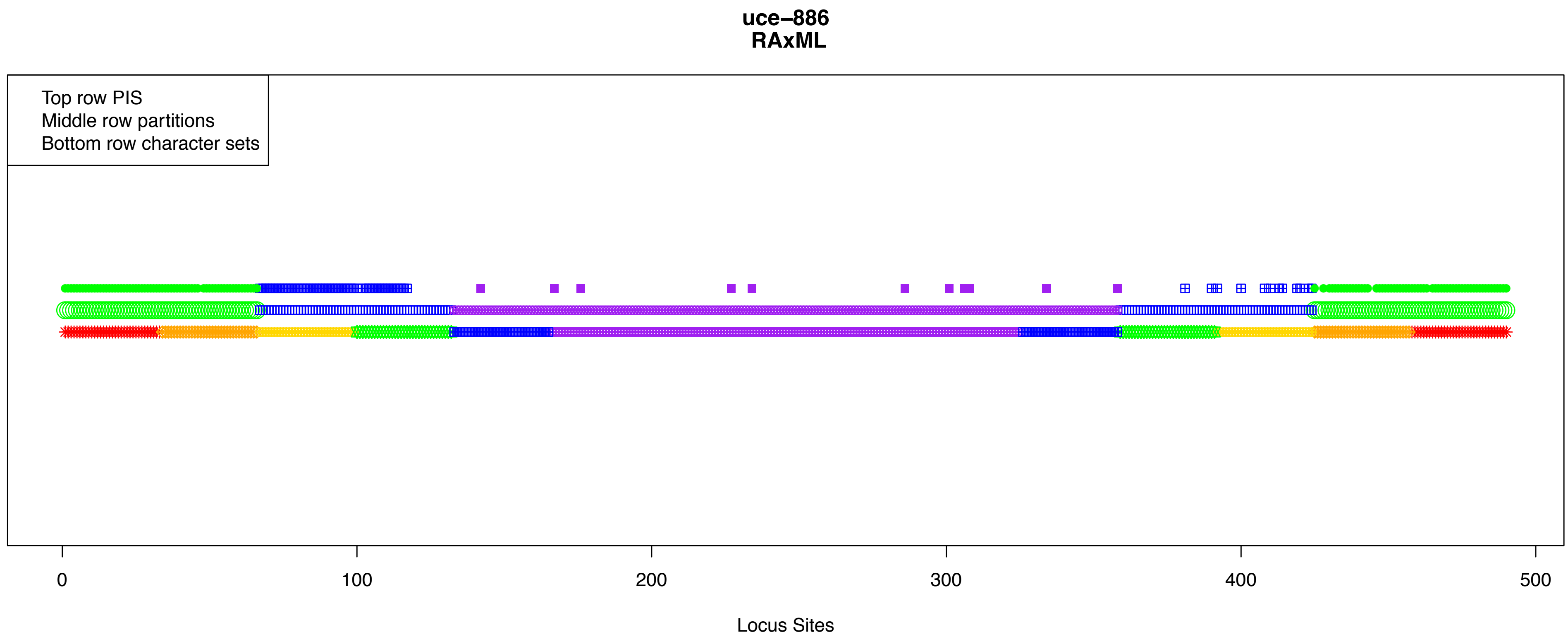

uce–886
RAxML
Top row PIS
Middle row partitions
Bottom row character sets
0
100
200
300
400
500
Locus Sites

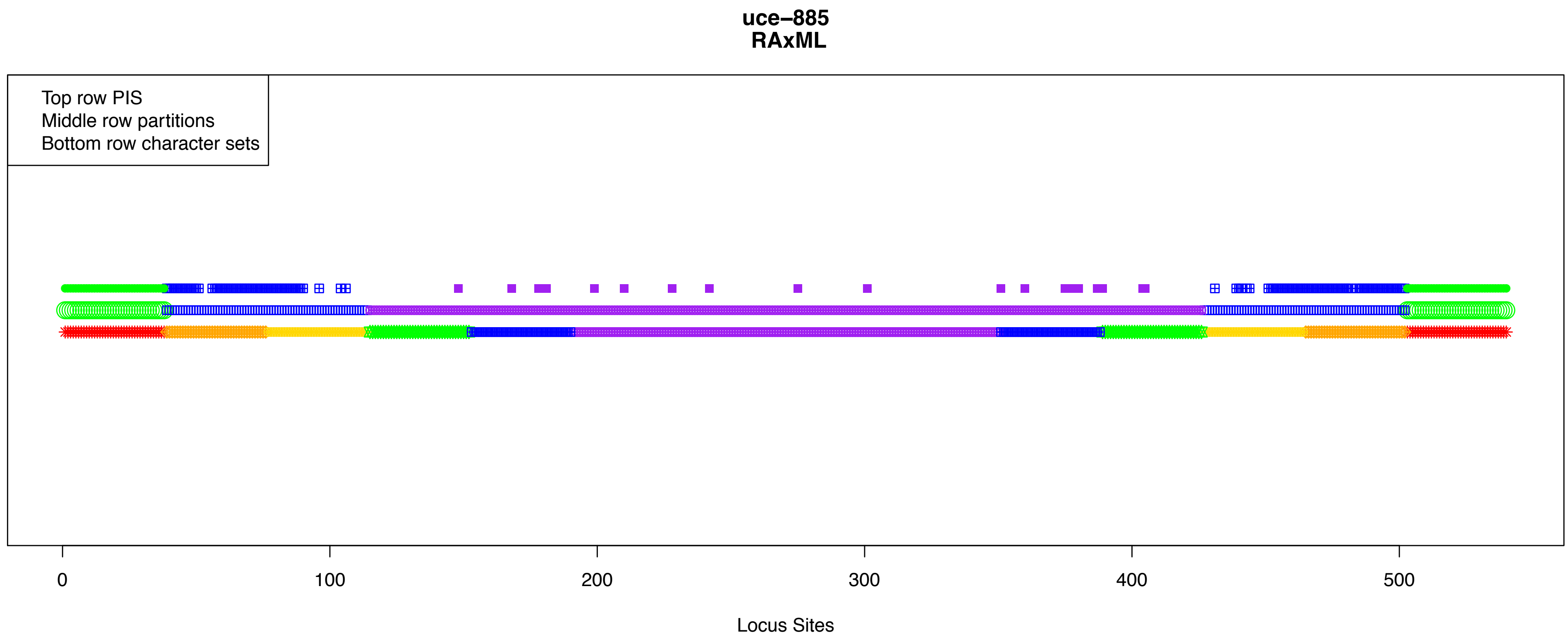

uce–885
RAxML
Top row PIS
Middle row partitions
Bottom row character sets
0
100
200
300
400
500
Locus Sites

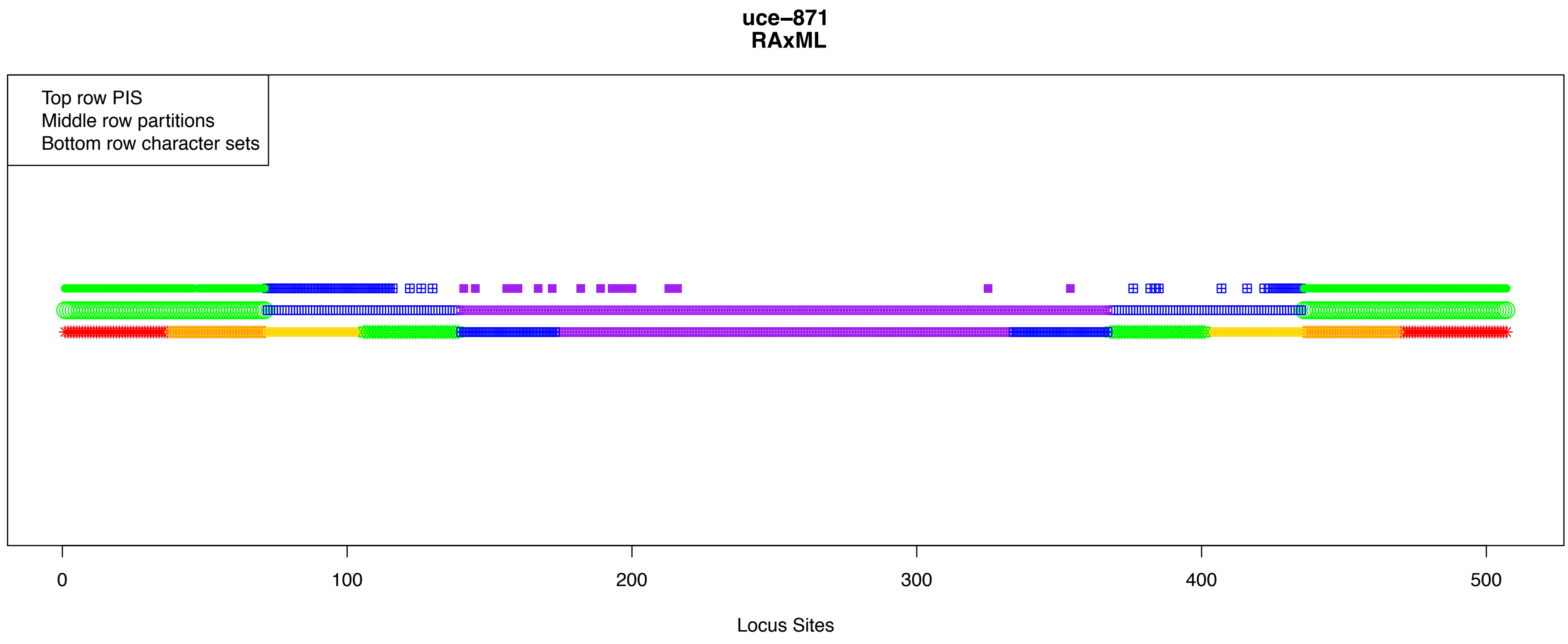

uce–871
RAxML
Top row PIS
Middle row partitions
Bottom row character sets
0
100
200
300
400
500
Locus Sites

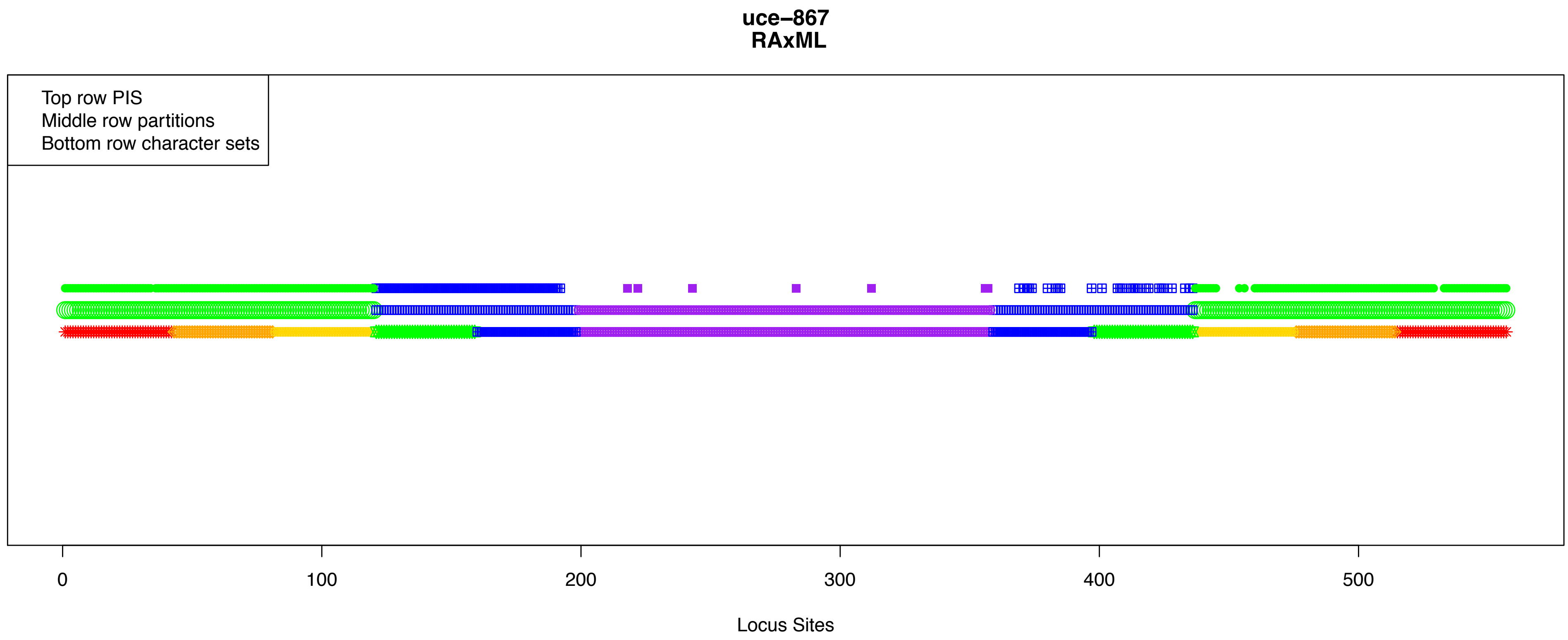
uce–867
RAxML
Top row PIS
Middle row partitions
Bottom row character sets
0
100
200
300
400
500
Locus Sites

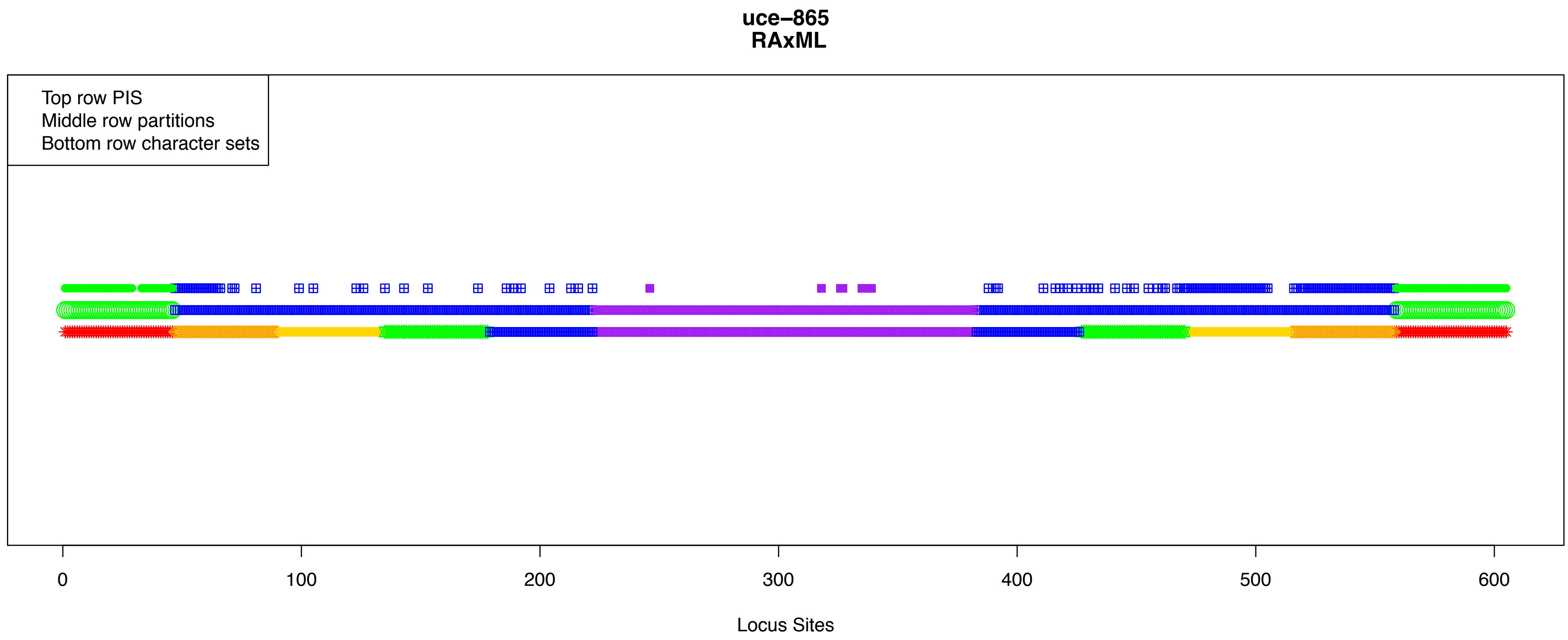

uce–865
RAxML
Top row PIS
Middle row partitions
Bottom row character sets
0
100
200
300
400
500
600
Locus Sites

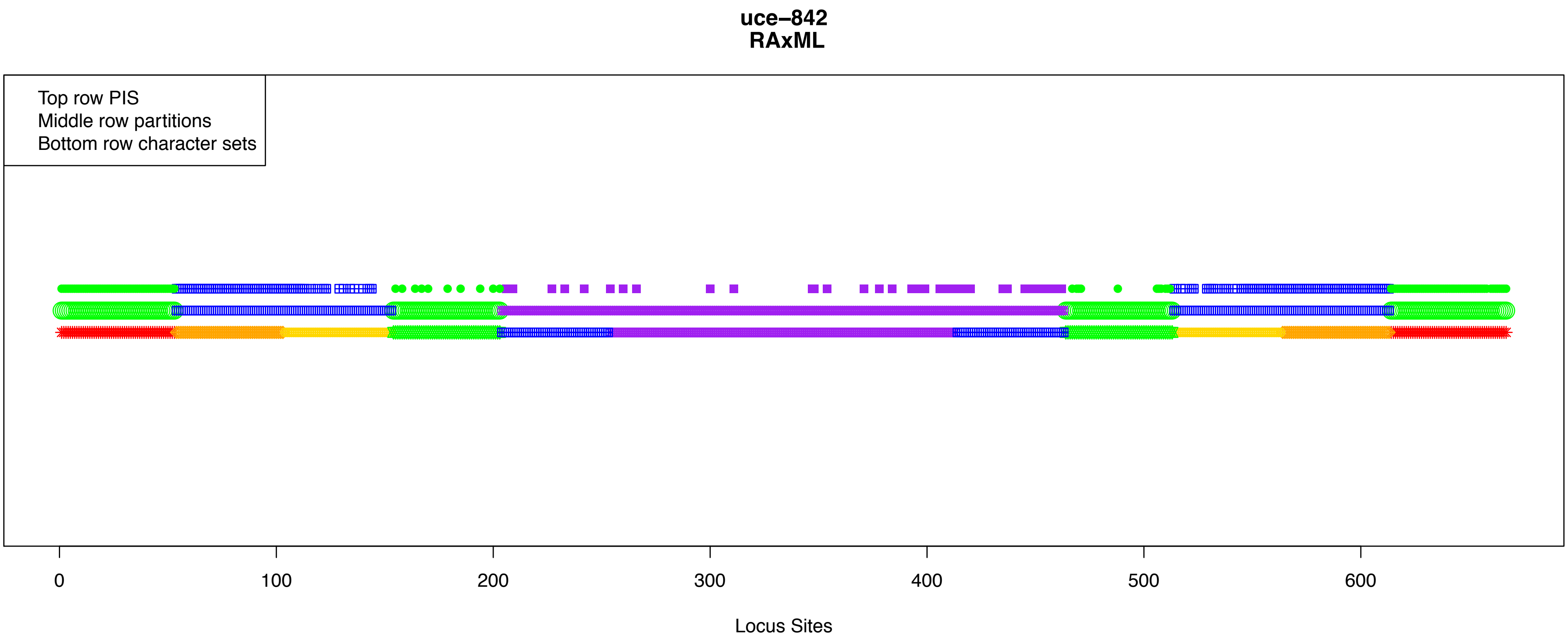
uce–842
RAxML
Top row PIS
Middle row partitions
Bottom row character sets
0
100
200
300
400
500
600
Locus Sites

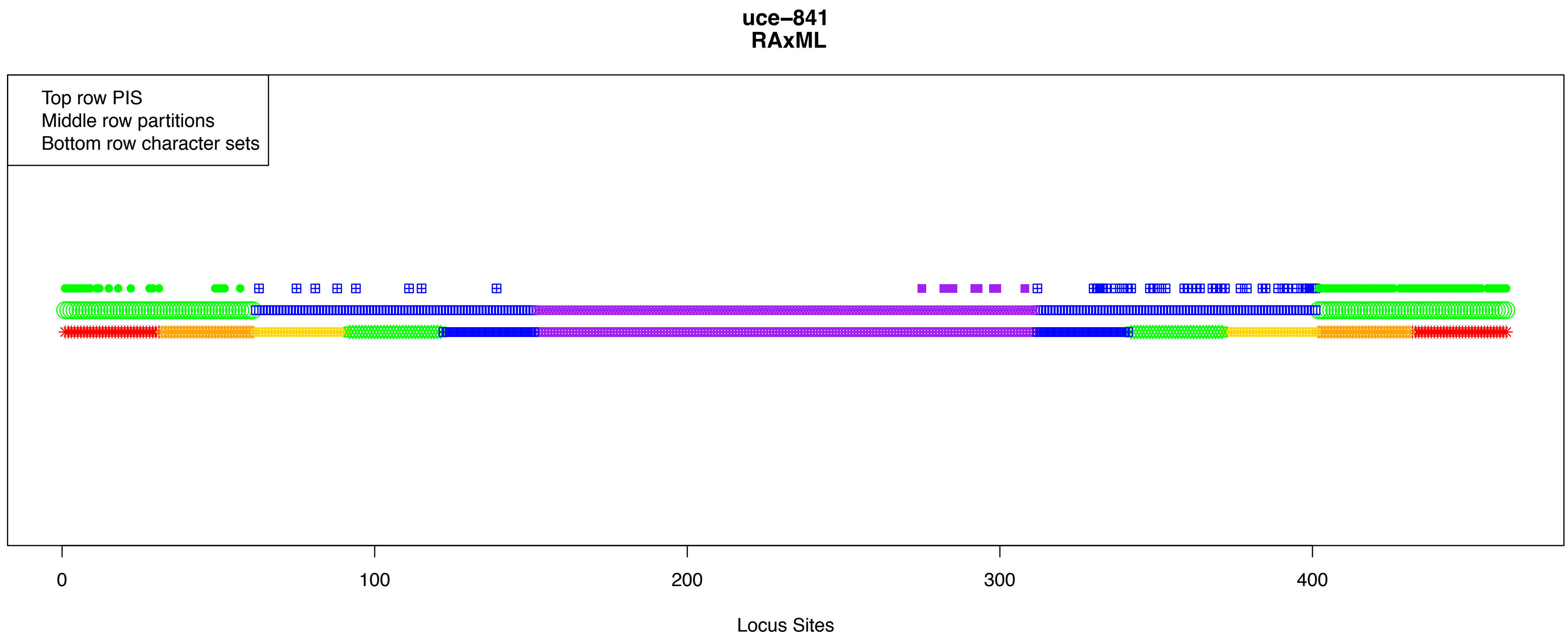
uce–841
RAxML
Top row PIS
Middle row partitions
Bottom row character sets
0
100
200
300
400
Locus Sites

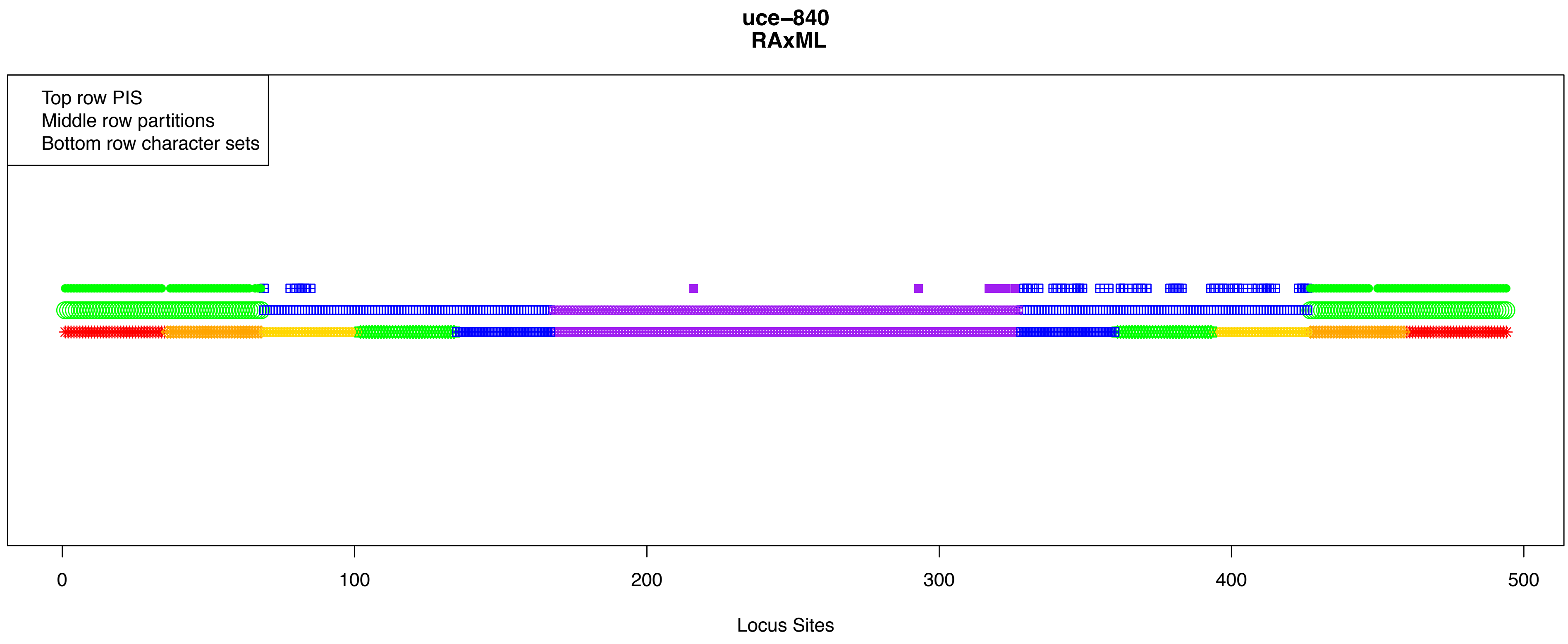
uce–840
RAxML
Top row PIS
Middle row partitions
Bottom row character sets
0
100
200
300
400
500
Locus Sites

uce–839
RAxML

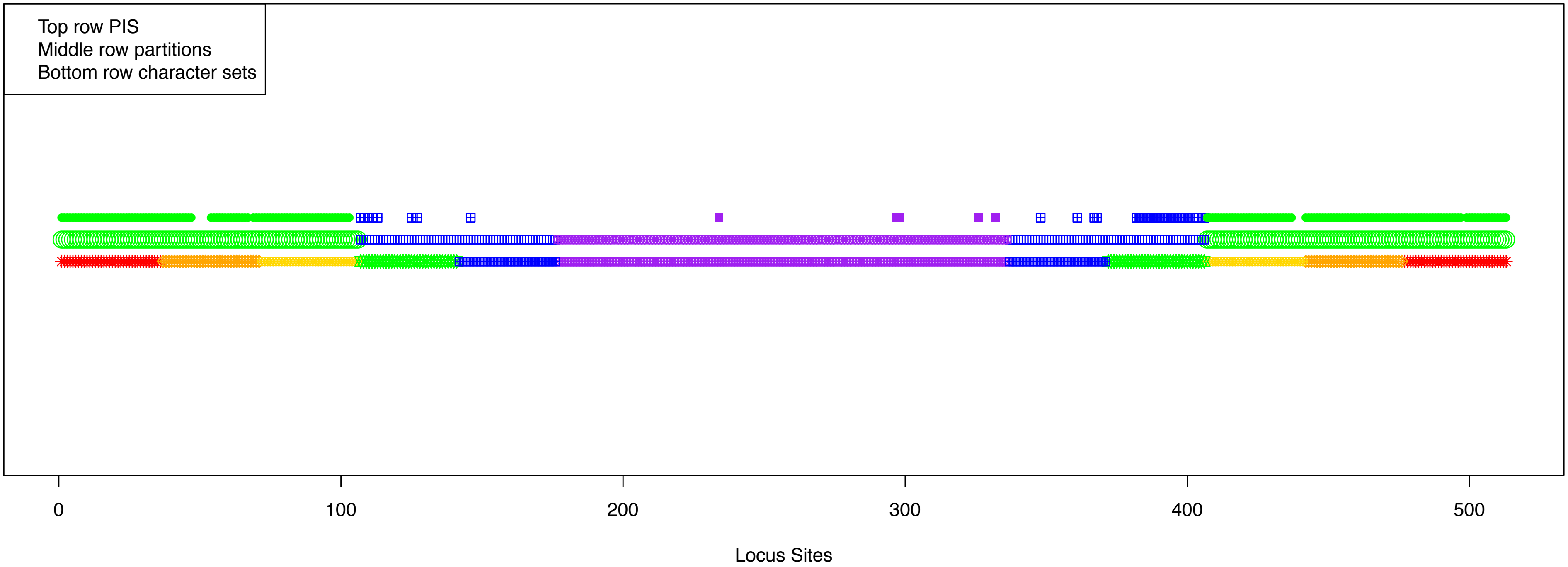

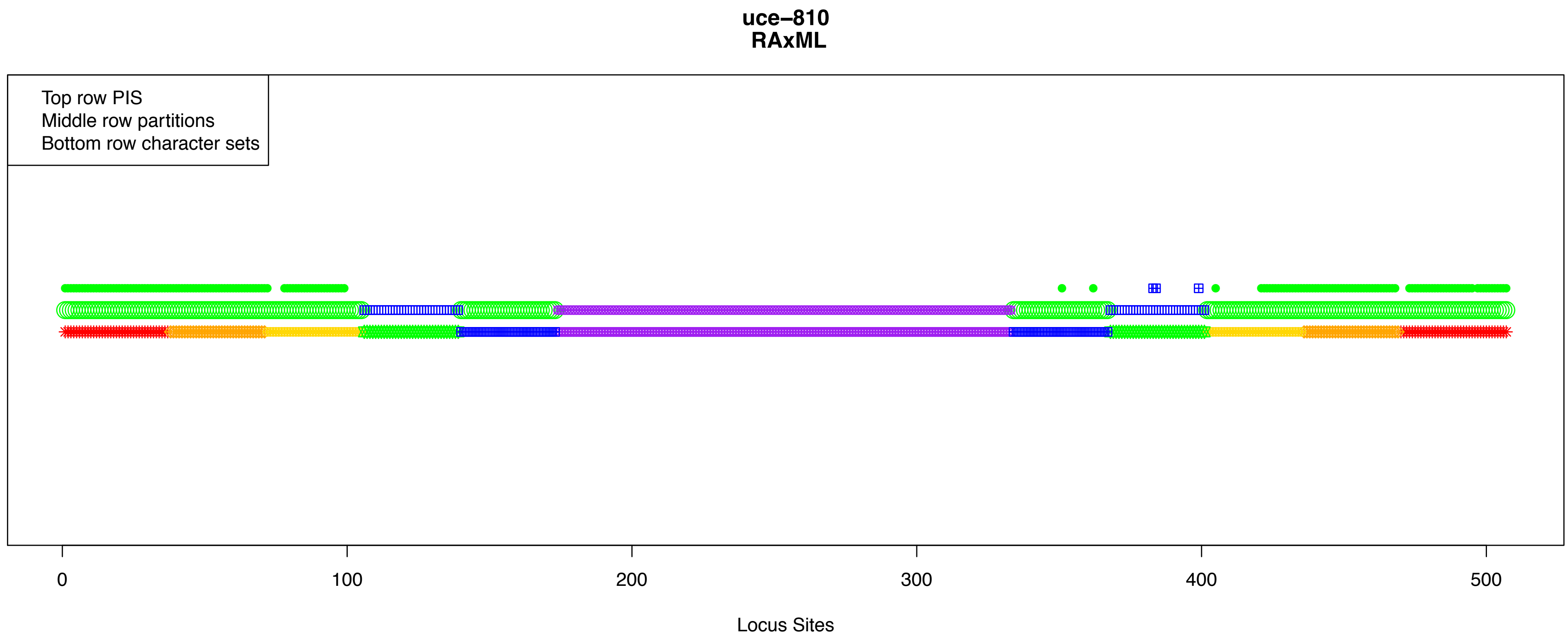
uce–810
RAxML
Top row PIS
Middle row partitions
Bottom row character sets
0
100
200
300
400
500
Locus Sites

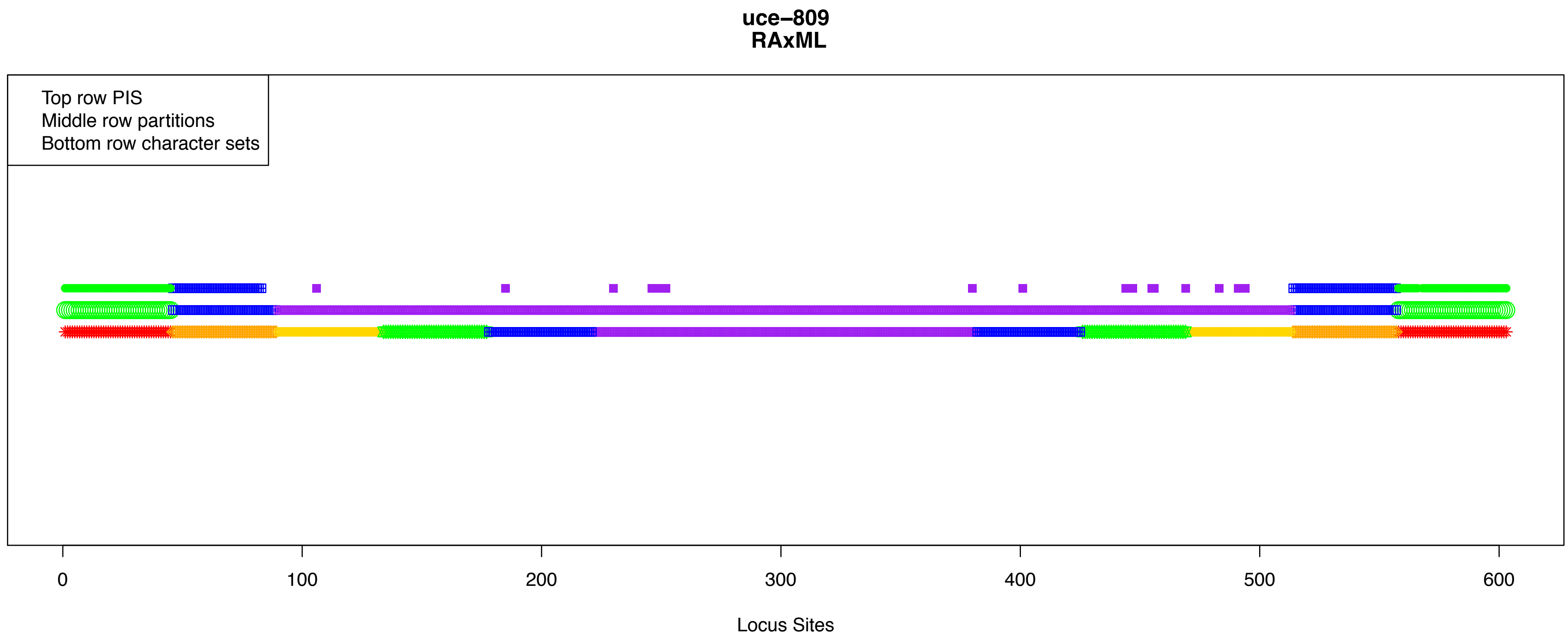
uce–809
RAxML
Top row PIS
Middle row partitions
Bottom row character sets
0
100
200
300
400
500
600
Locus Sites

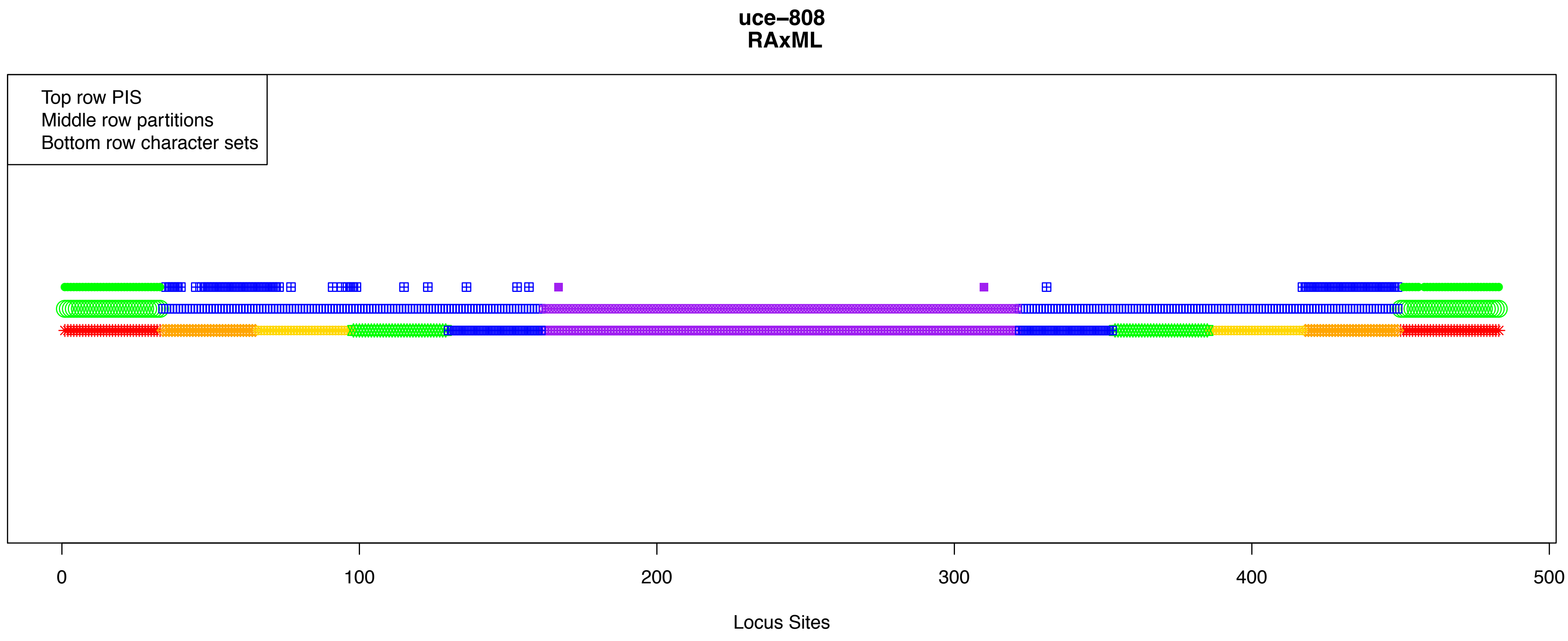

uce–808
RAxML
Top row PIS
Middle row partitions
Bottom row character sets
0
100
200
300
400
500
Locus Sites

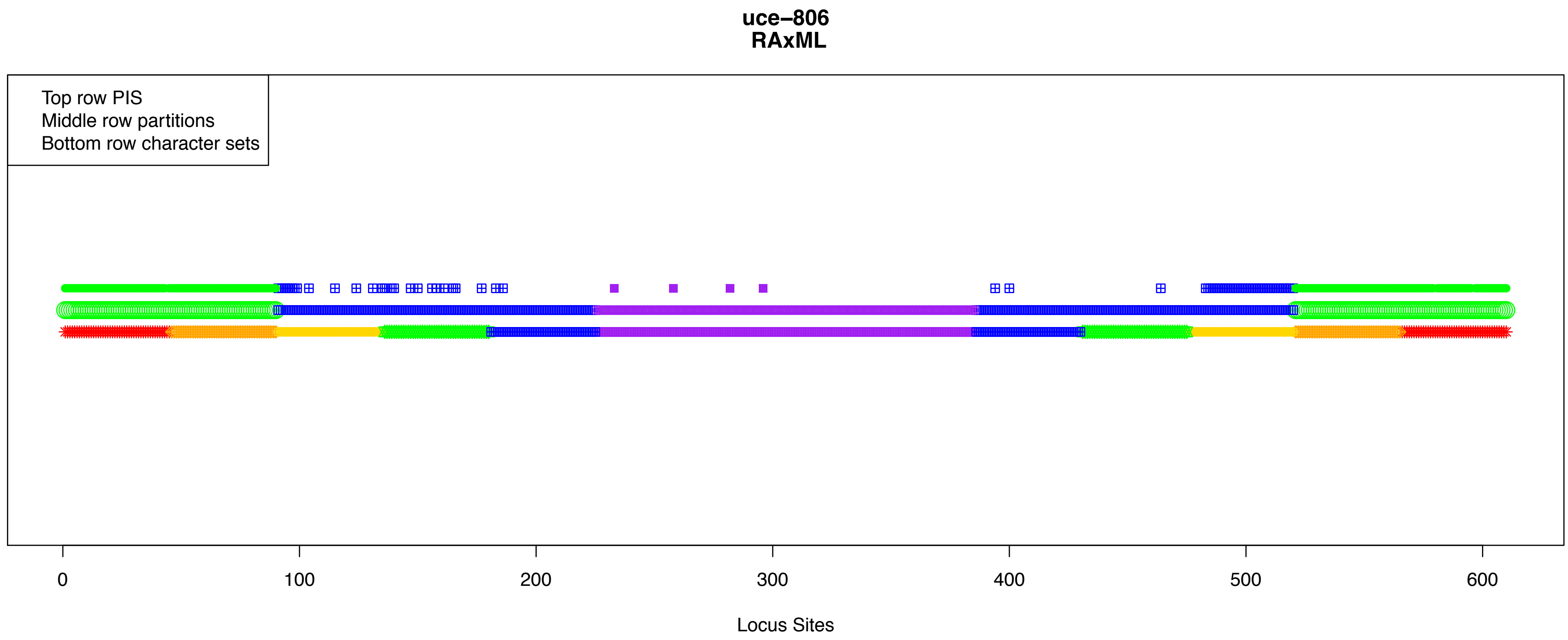
uce–806
RAxML
Top row PIS
Middle row partitions
Bottom row character sets
0
100
200
300
400
500
600
Locus Sites

uce–805
RAxML

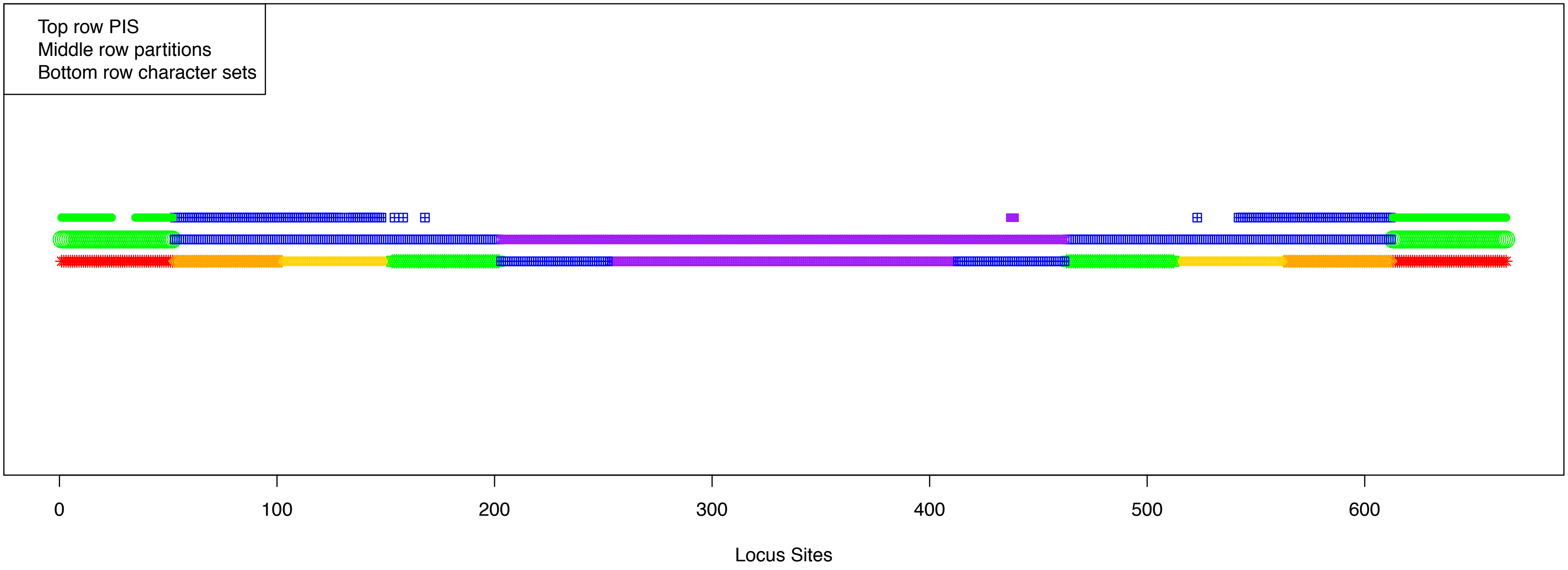

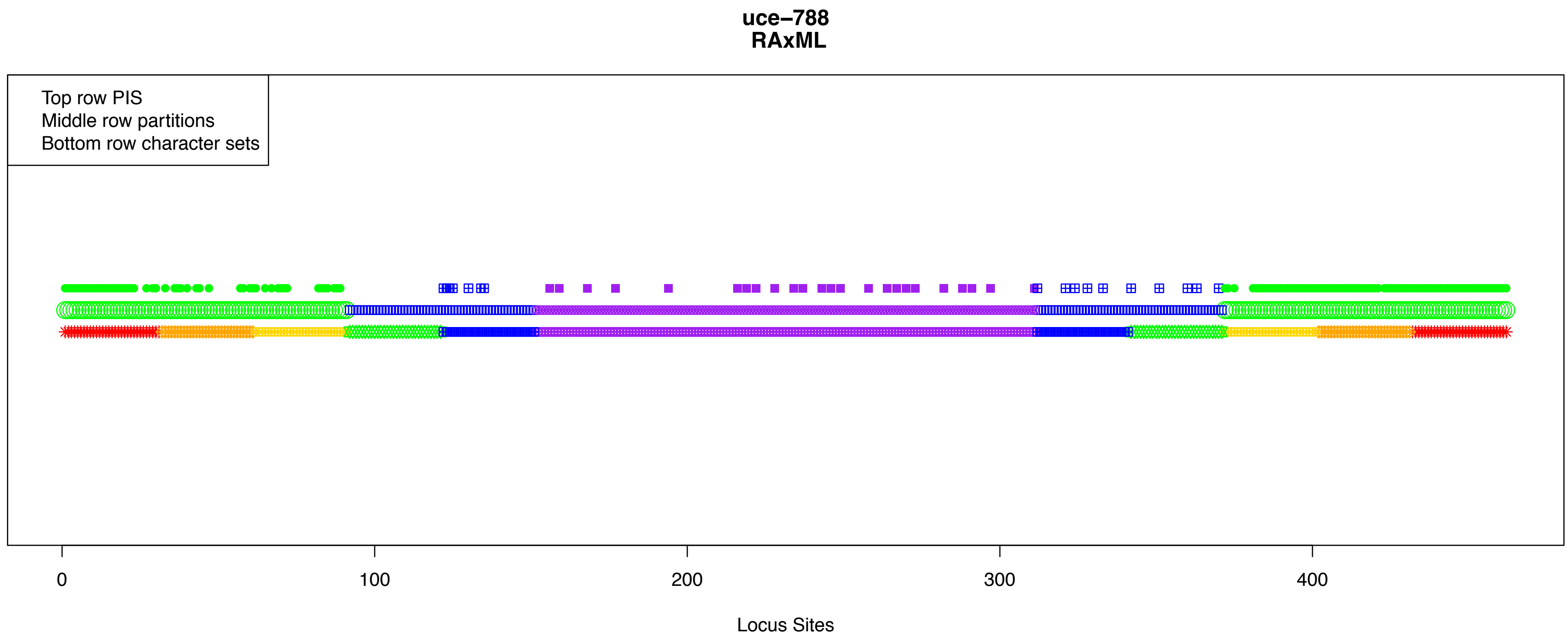

uce–788
RAxML
Top row PIS
Middle row partitions
Bottom row character sets
0
100
200
300
400
Locus Sites

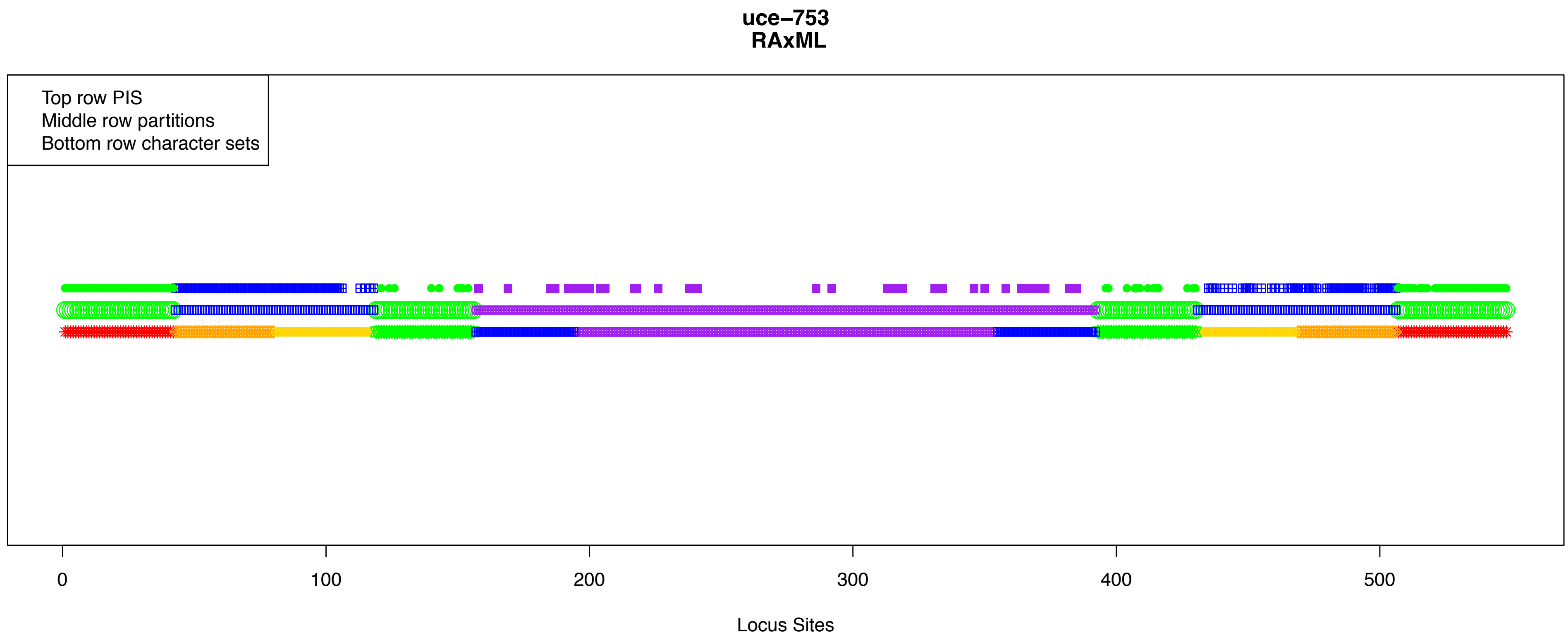
uce–753
RAxML
Top row PIS
Middle row partitions
Bottom row character sets
0
100
200
300
400
500
Locus Sites

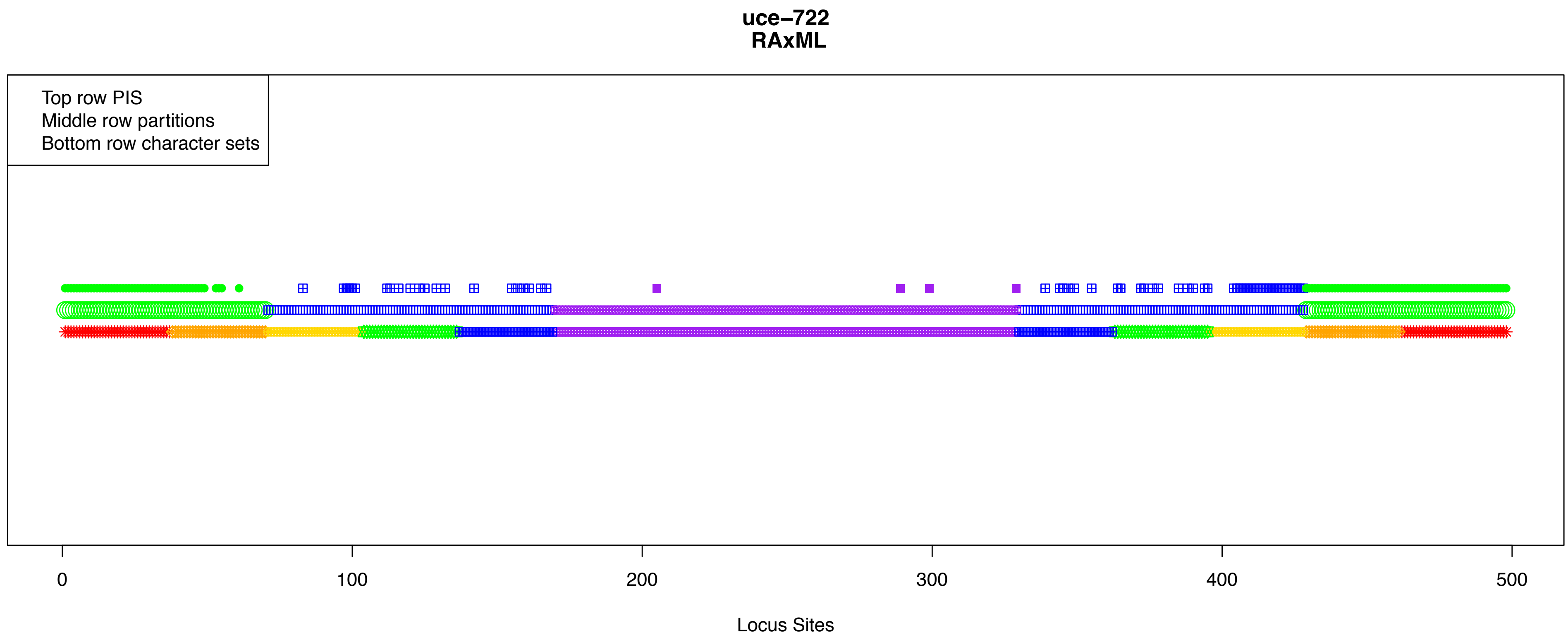
uce–722
RAxML
Top row PIS
Middle row partitions
Bottom row character sets
0
100
200
300
400
500
Locus Sites

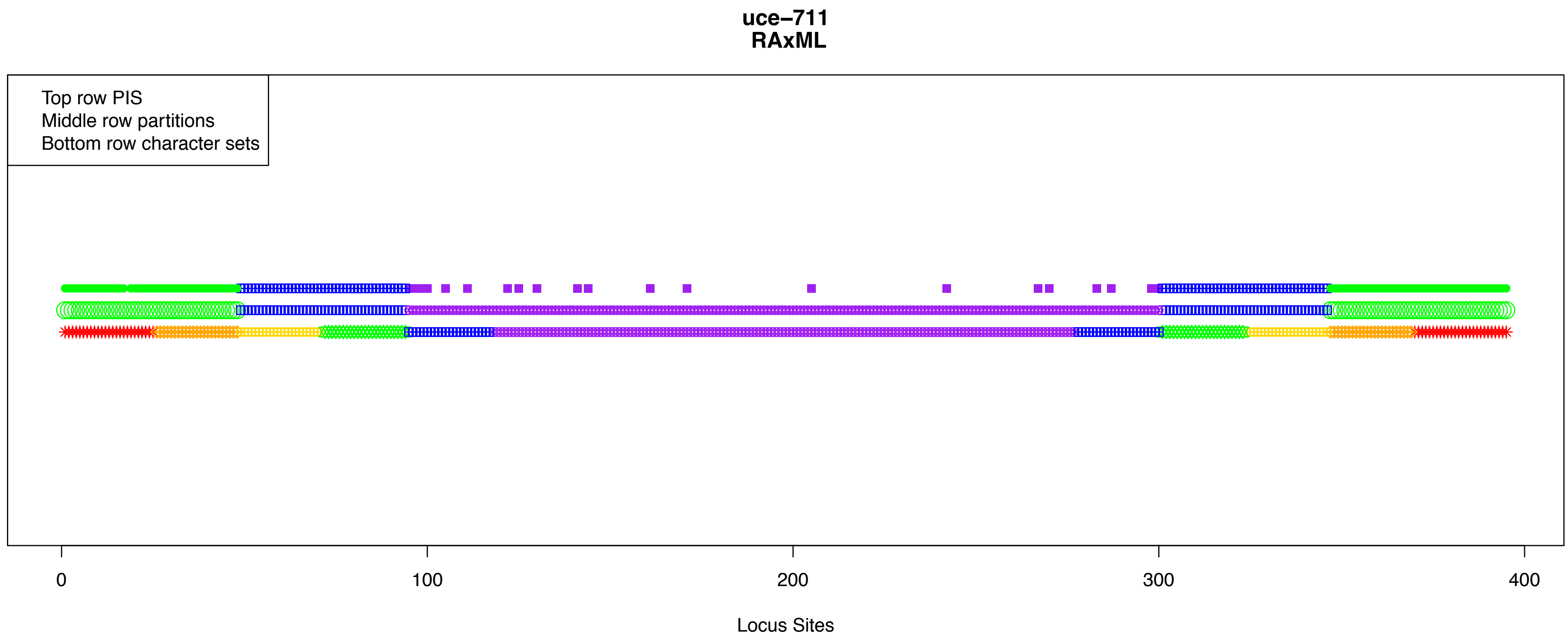
uce–711
RAxML
Top row PIS
Middle row partitions
Bottom row character sets
0
100
200
300
400
Locus Sites

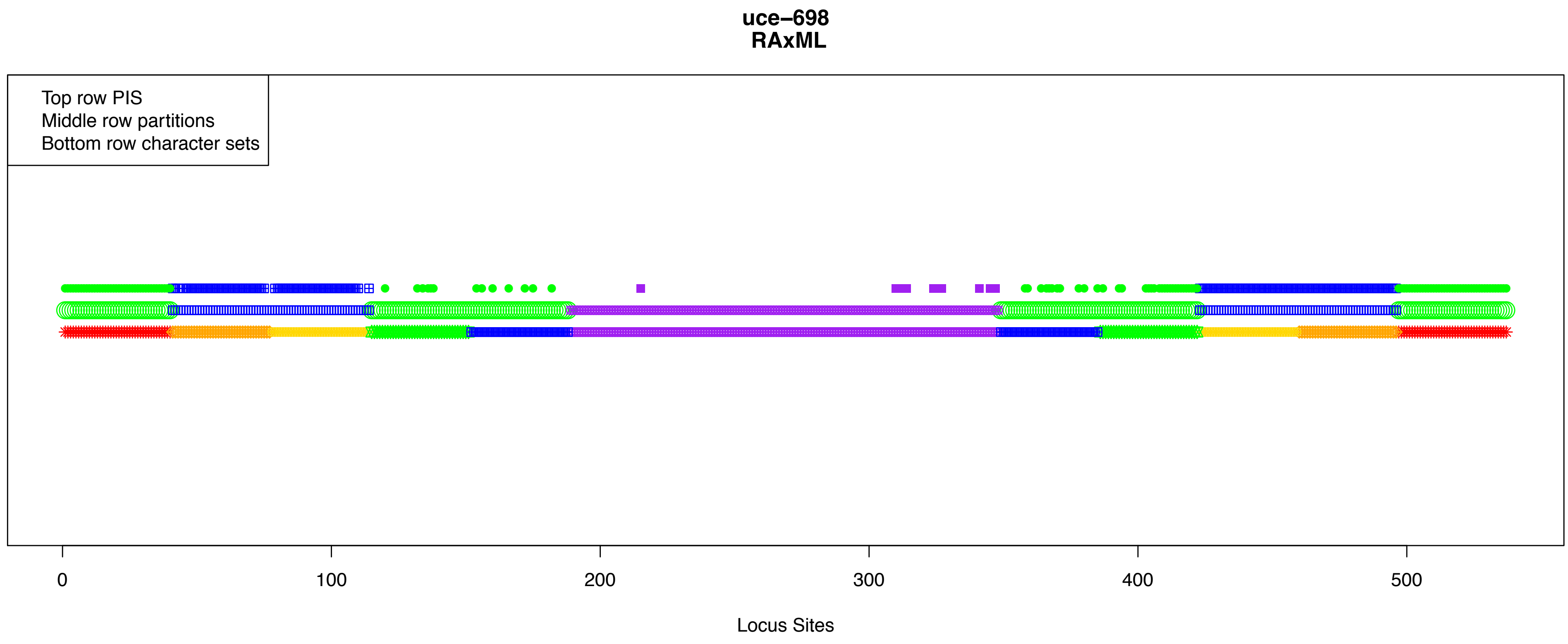

uce–698
RAxML
Top row PIS
Middle row partitions
Bottom row character sets
0
100
200
300
400
500
Locus Sites

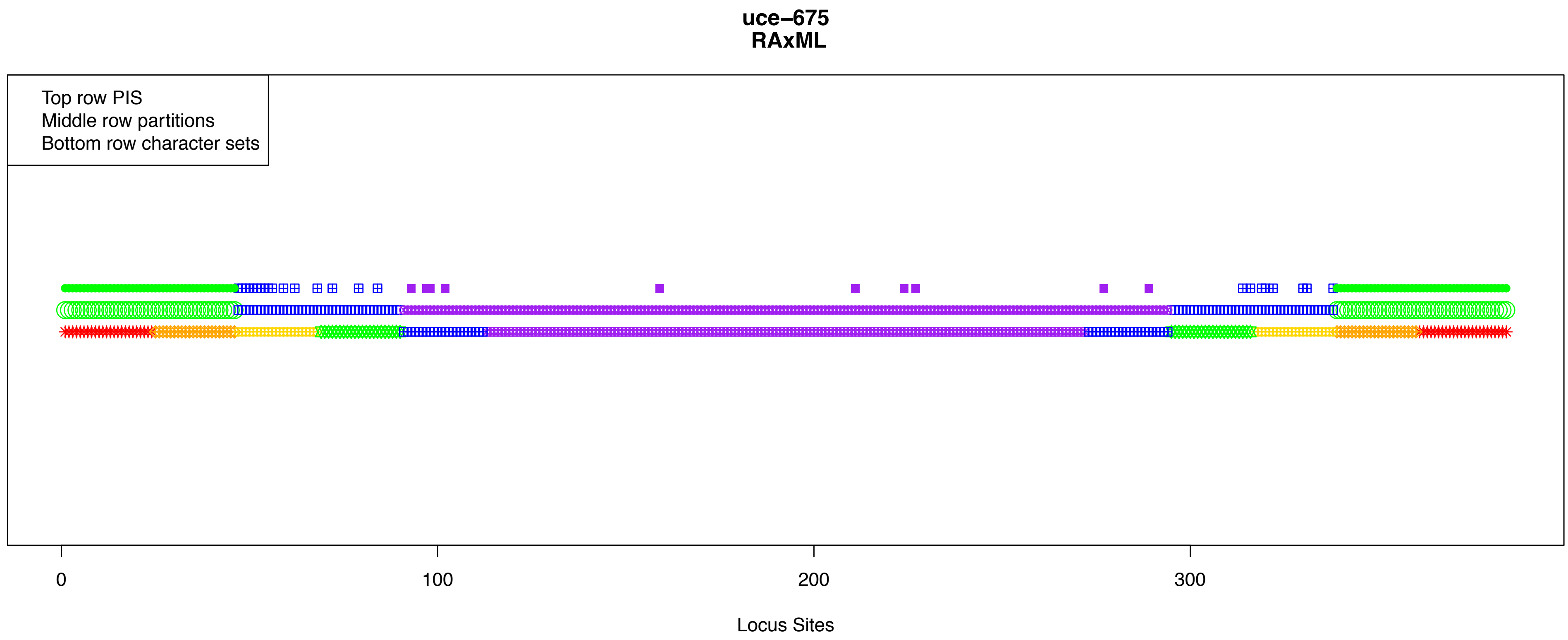

uce–675
RAxML
Top row PIS
Middle row partitions
Bottom row character sets
0
100
200
300
Locus Sites

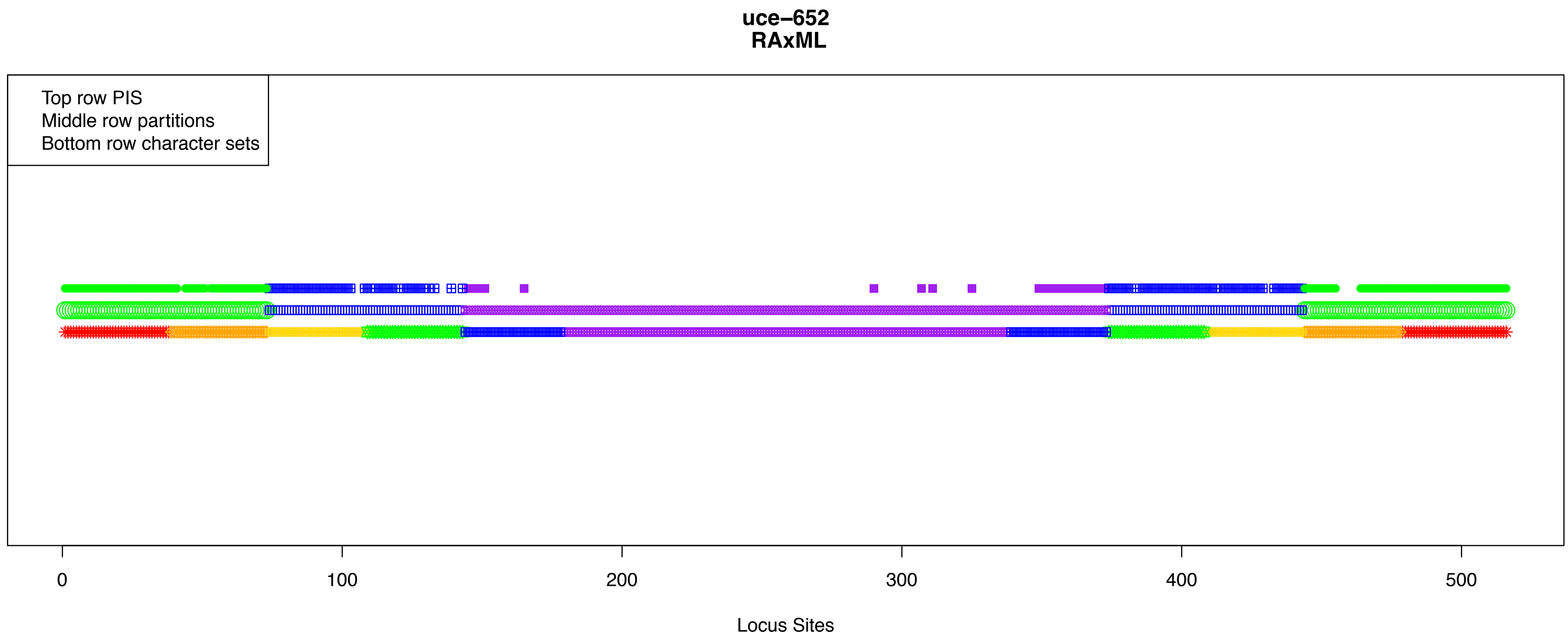
uce–652
RAxML
Top row PIS
Middle row partitions
Bottom row character sets
0
100
200
300
400
500
Locus Sites

uce–614
RAxML

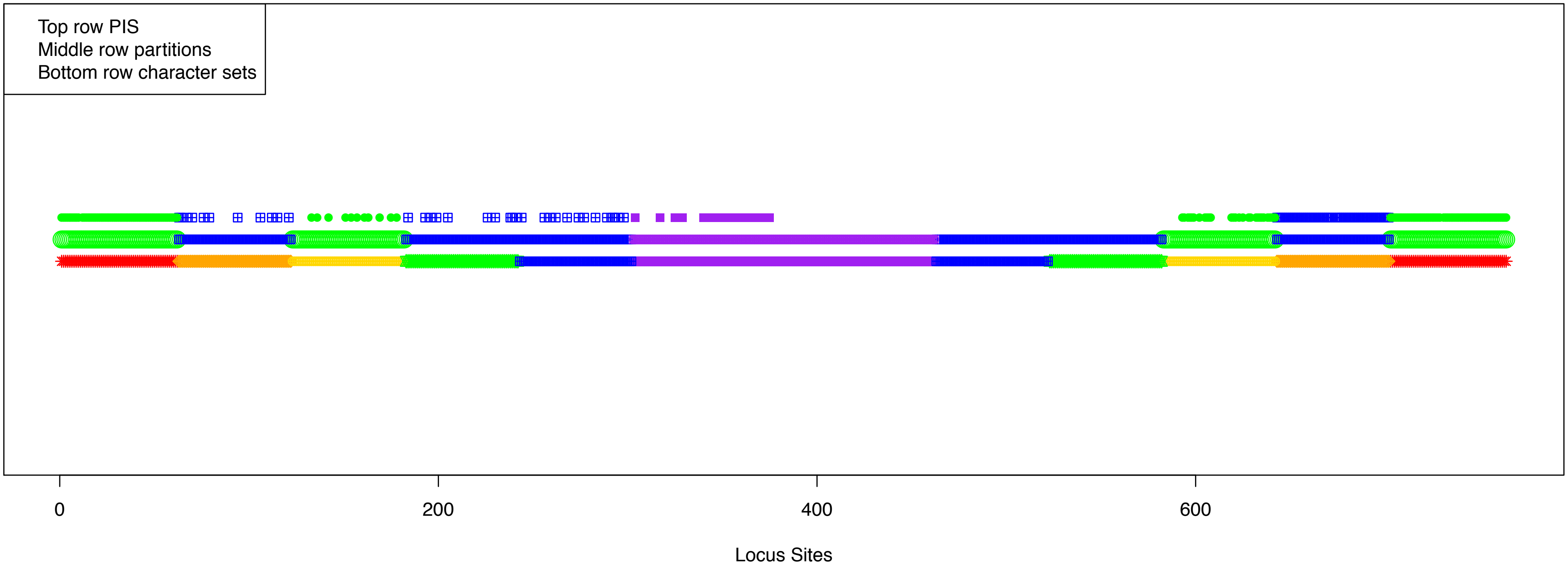

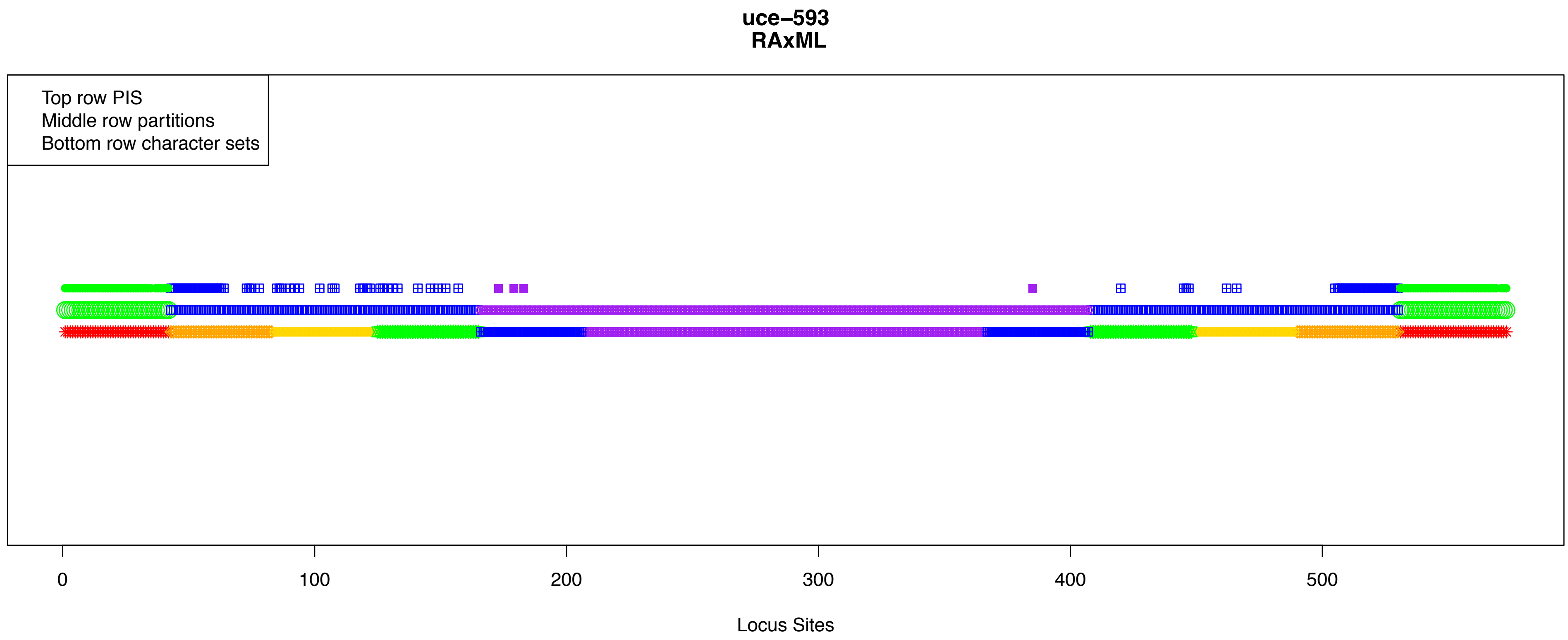

uce–593
RAxML
Top row PIS
Middle row partitions
Bottom row character sets
0
100
200
300
400
500
Locus Sites

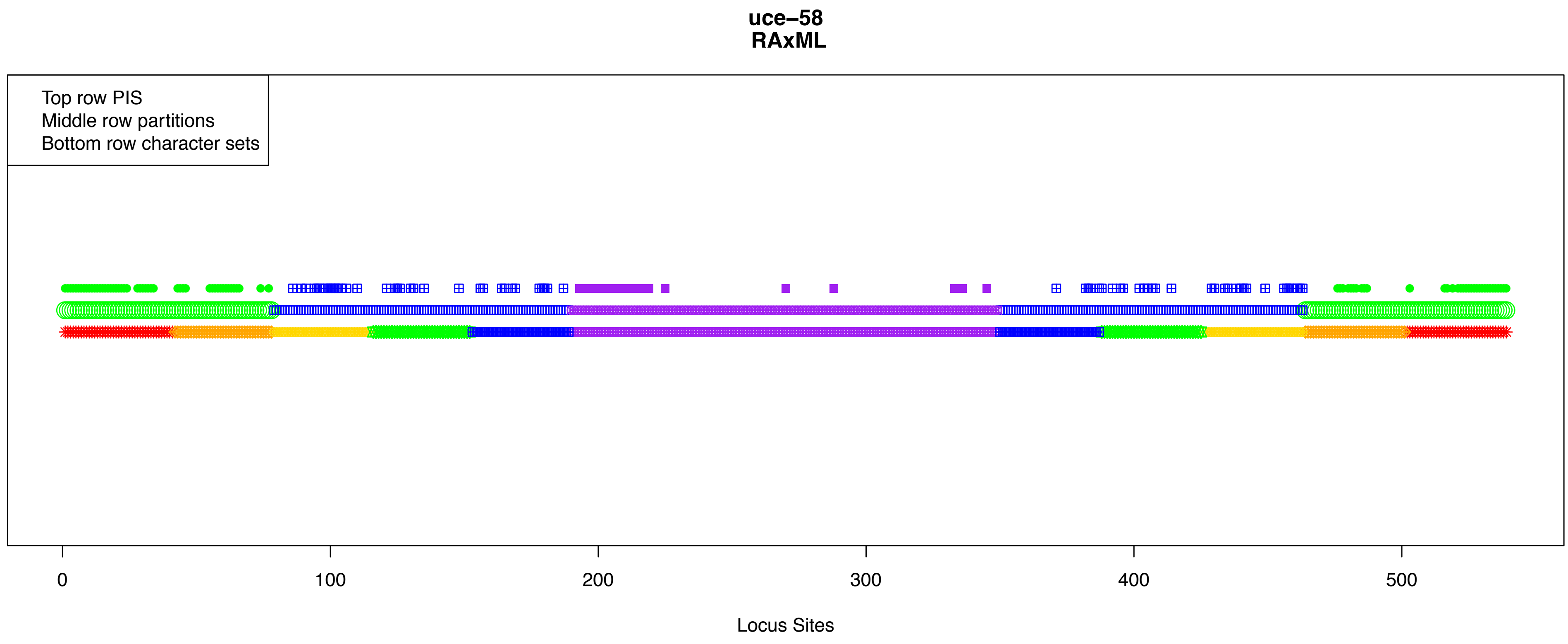
uce–58
RAxML
Top row PIS
Middle row partitions
Bottom row character sets
0
100
200
300
400
500
Locus Sites

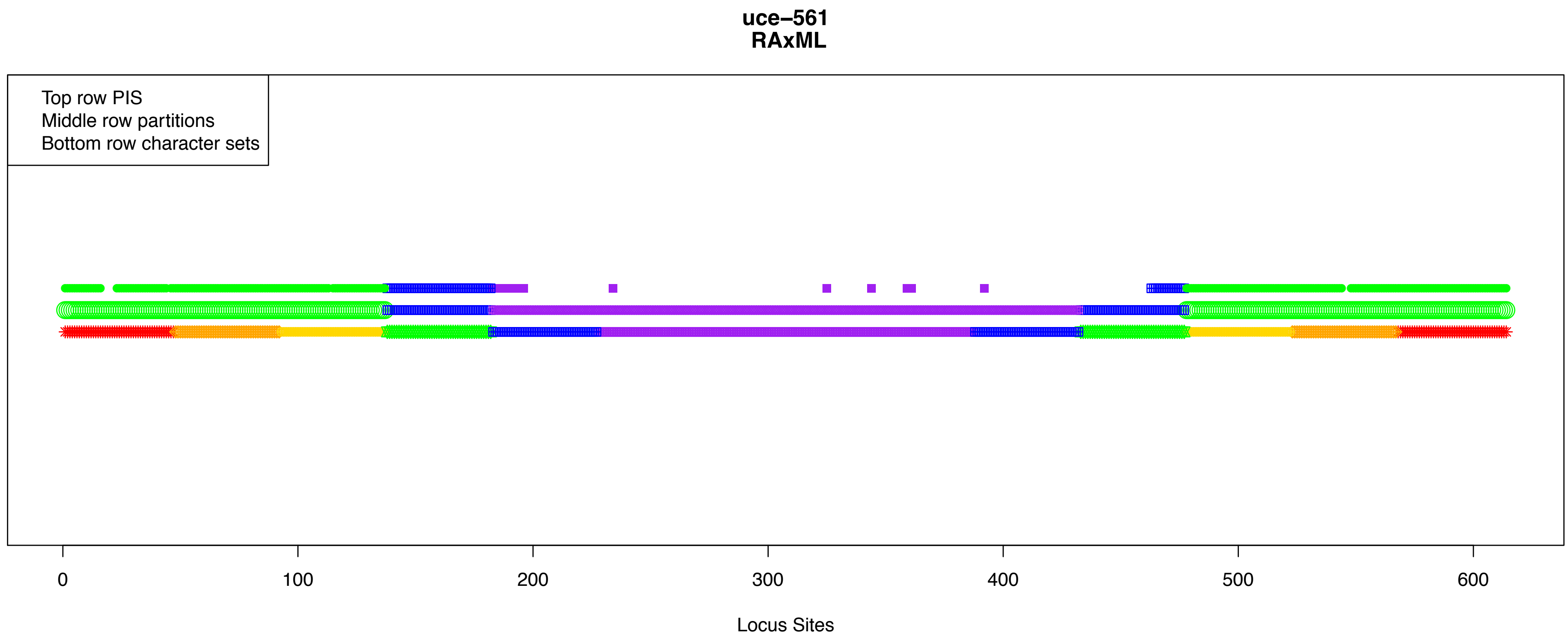

uce–561
RAxML
Top row PIS
Middle row partitions
Bottom row character sets
0
100
200
300
400
500
600
Locus Sites

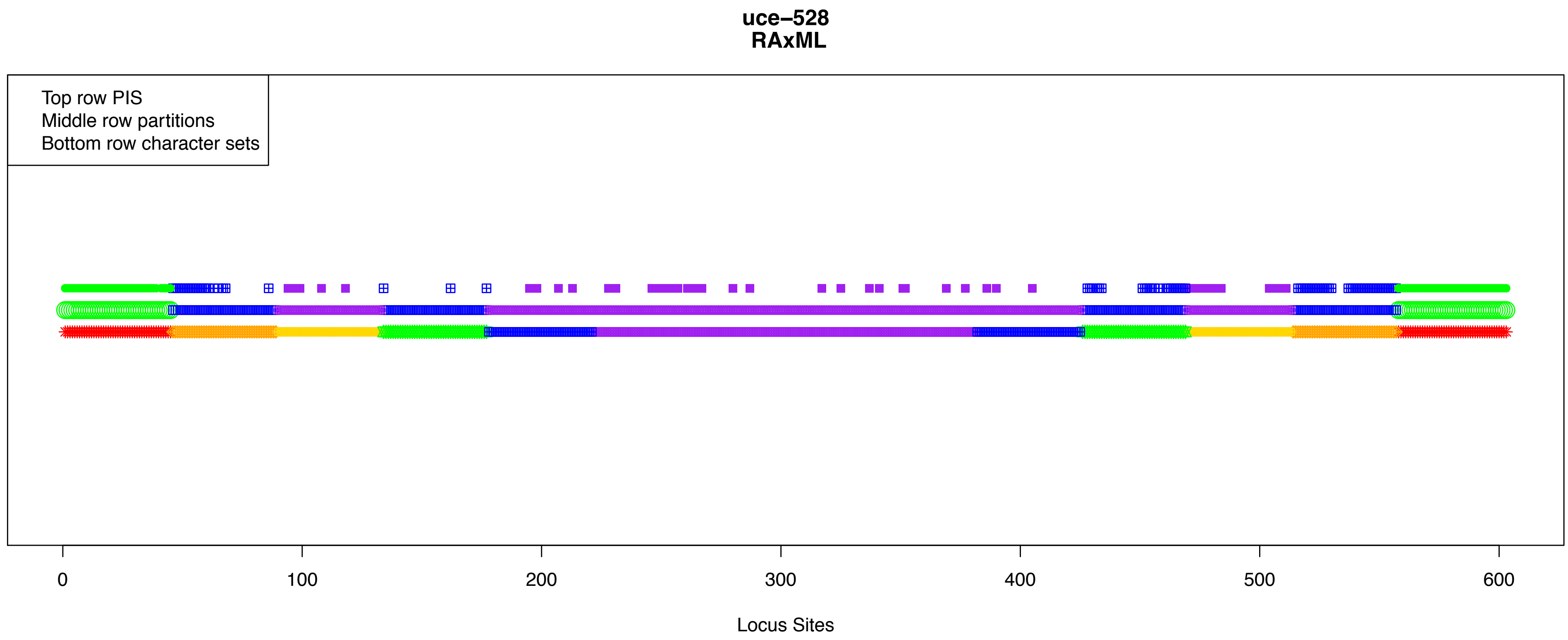

uce–528
RAxML
Top row PIS
Middle row partitions
Bottom row character sets
0
100
200
300
400
500
600
Locus Sites

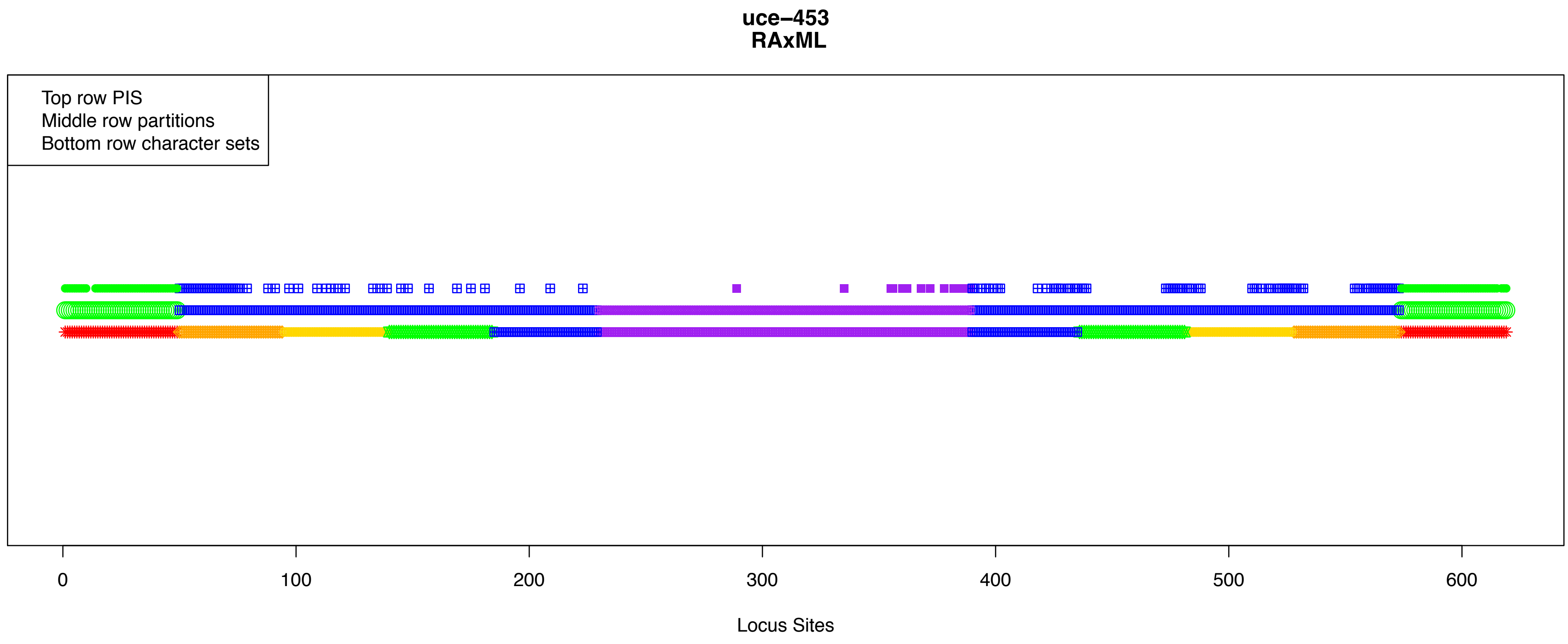
uce–453
RAxML
Top row PIS
Middle row partitions
Bottom row character sets
0
100
200
300
400
500
600
Locus Sites

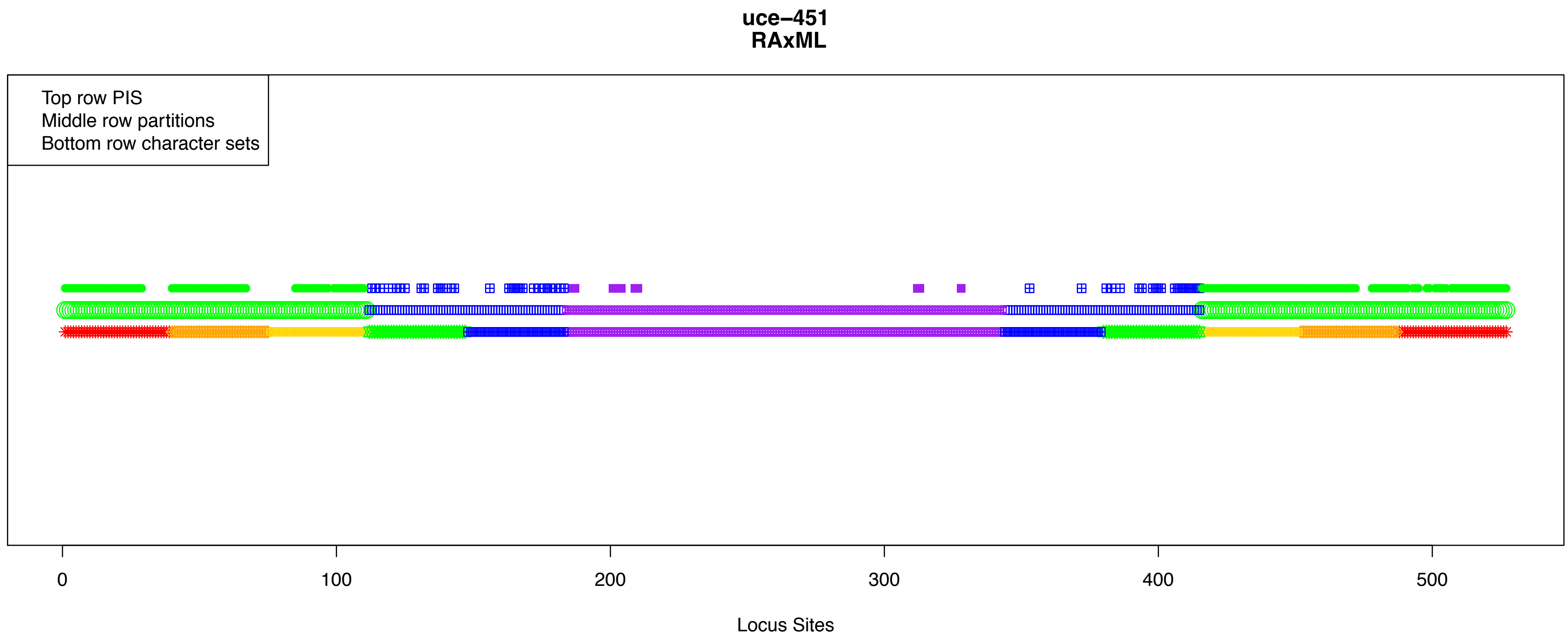

uce–451
RAxML
Top row PIS
Middle row partitions
Bottom row character sets
0
100
200
300
400
500
Locus Sites

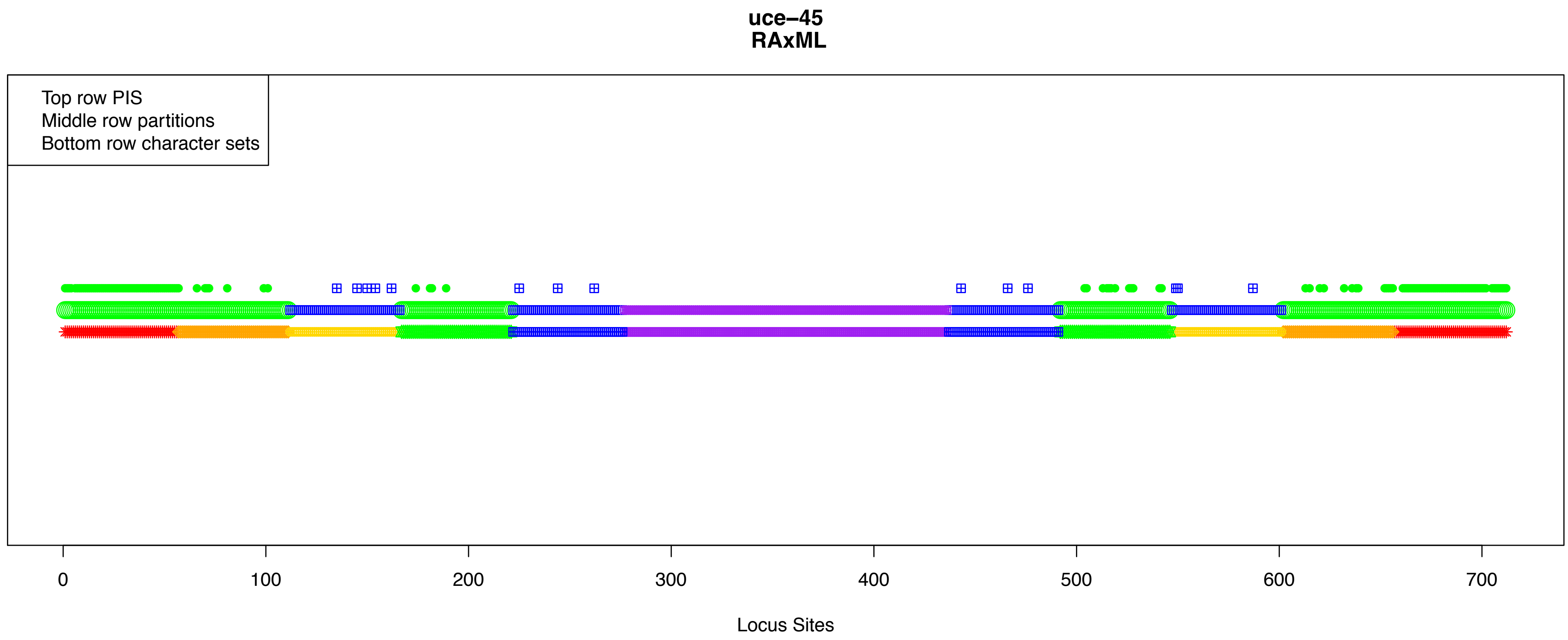

uce–45
RAxML
Top row PIS
Middle row partitions
Bottom row character sets
0
100
200
300
400
500
600
700
Locus Sites

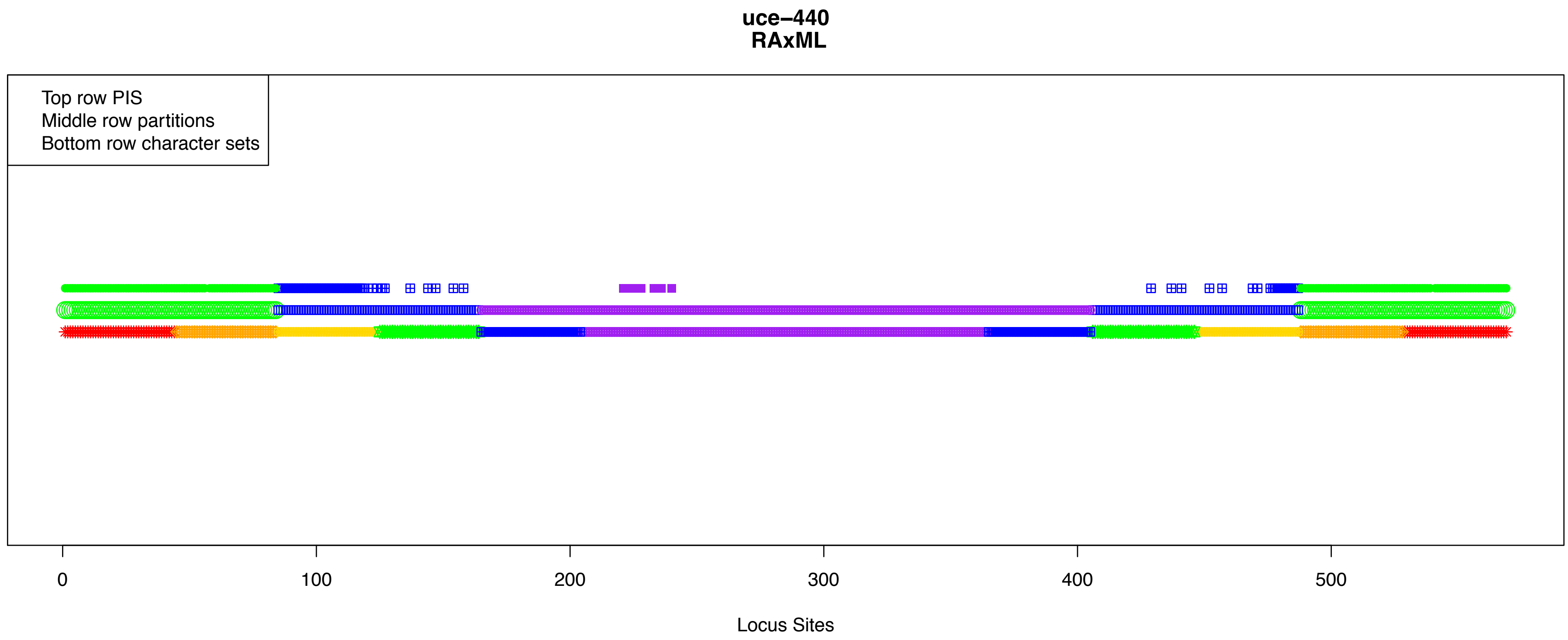

uce–440
RAxML
Top row PIS
Middle row partitions
Bottom row character sets
0
100
200
300
400
500
Locus Sites

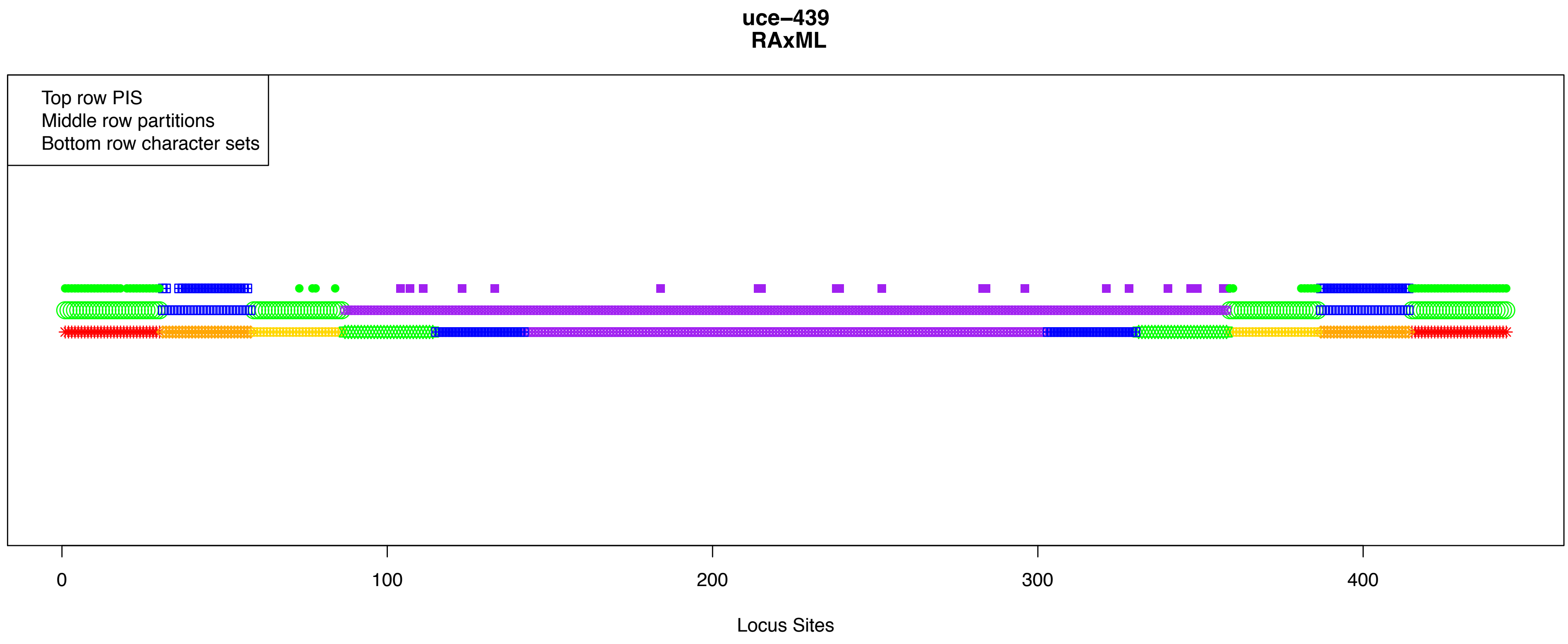
uce–439
RAxML
Top row PIS
Middle row partitions
Bottom row character sets
0
100
200
300
400
Locus Sites

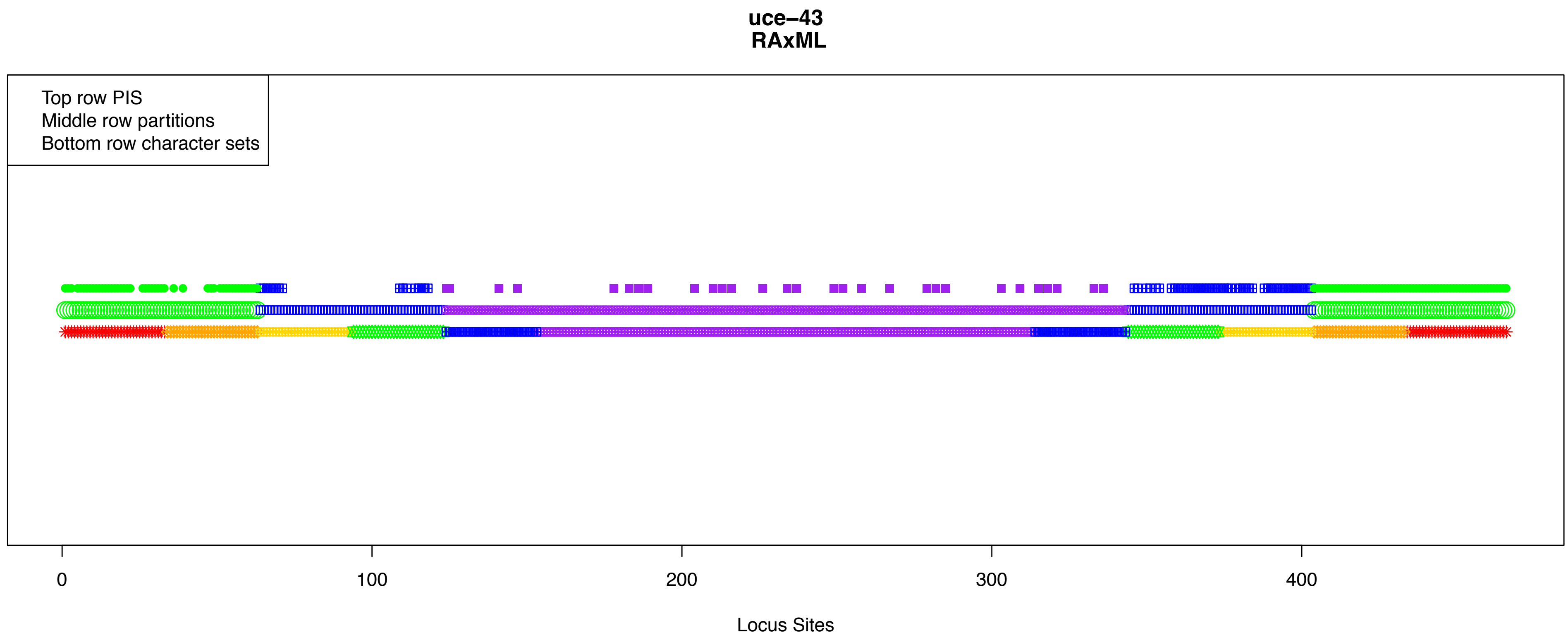

uce–43
RAxML
Top row PIS
Middle row partitions
Bottom row character sets
0
100
200
300
400
Locus Sites

uce–42
RAxML

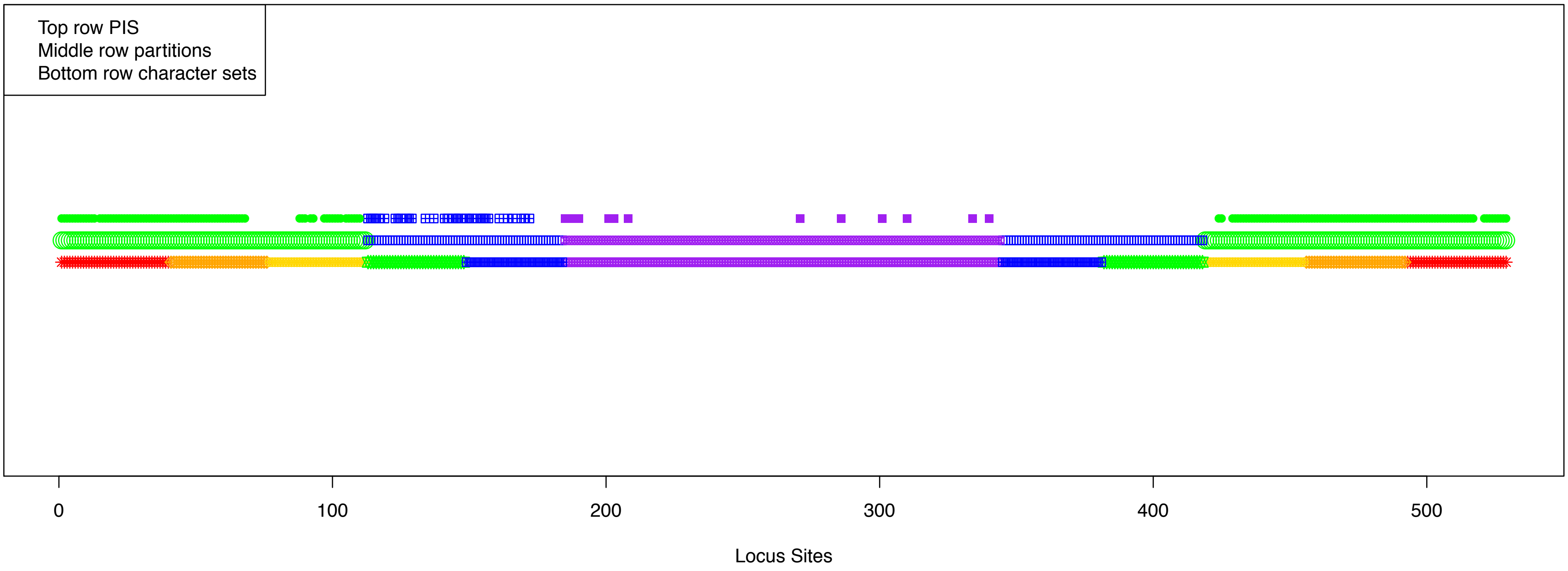

Top row PIS
Middle row partitions
Bottom row character sets
0
100
200
300
400
500
Locus Sites

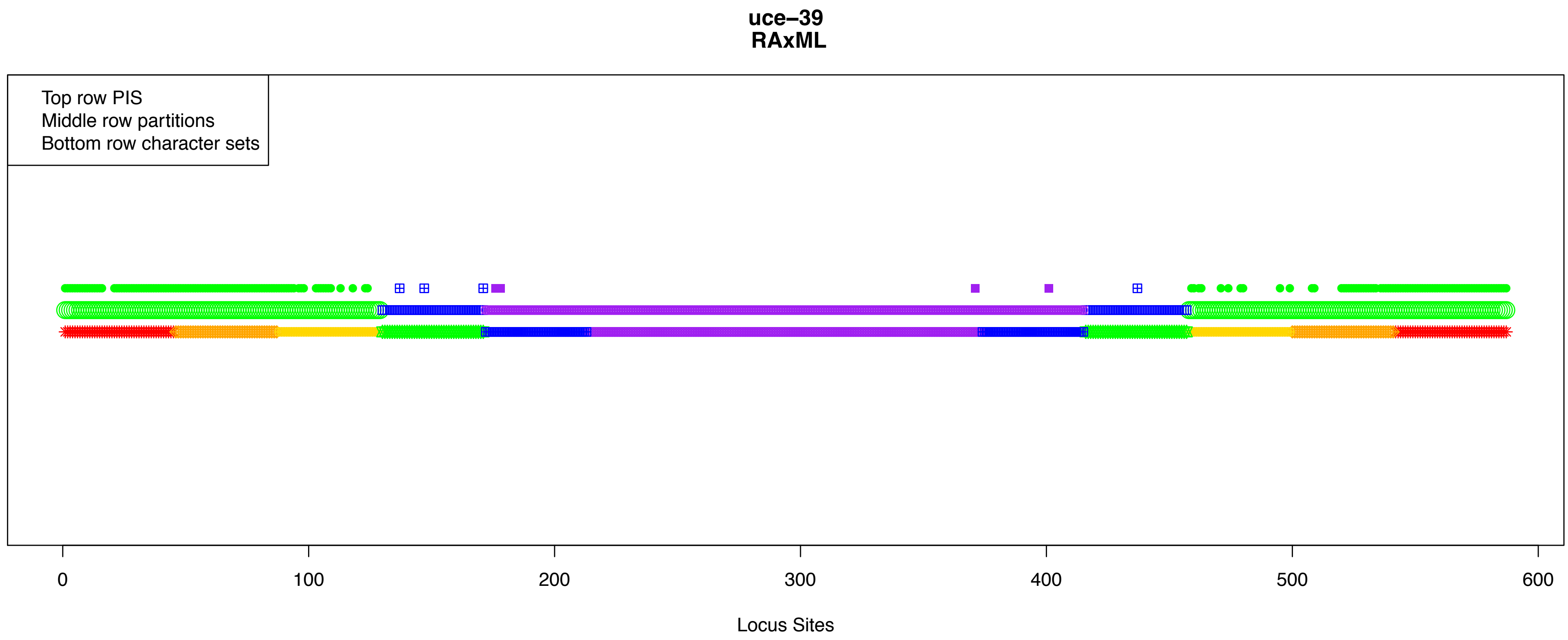
uce–39
RAxML
Top row PIS
Middle row partitions
Bottom row character sets
0
100
200
300
400
500
600
Locus Sites

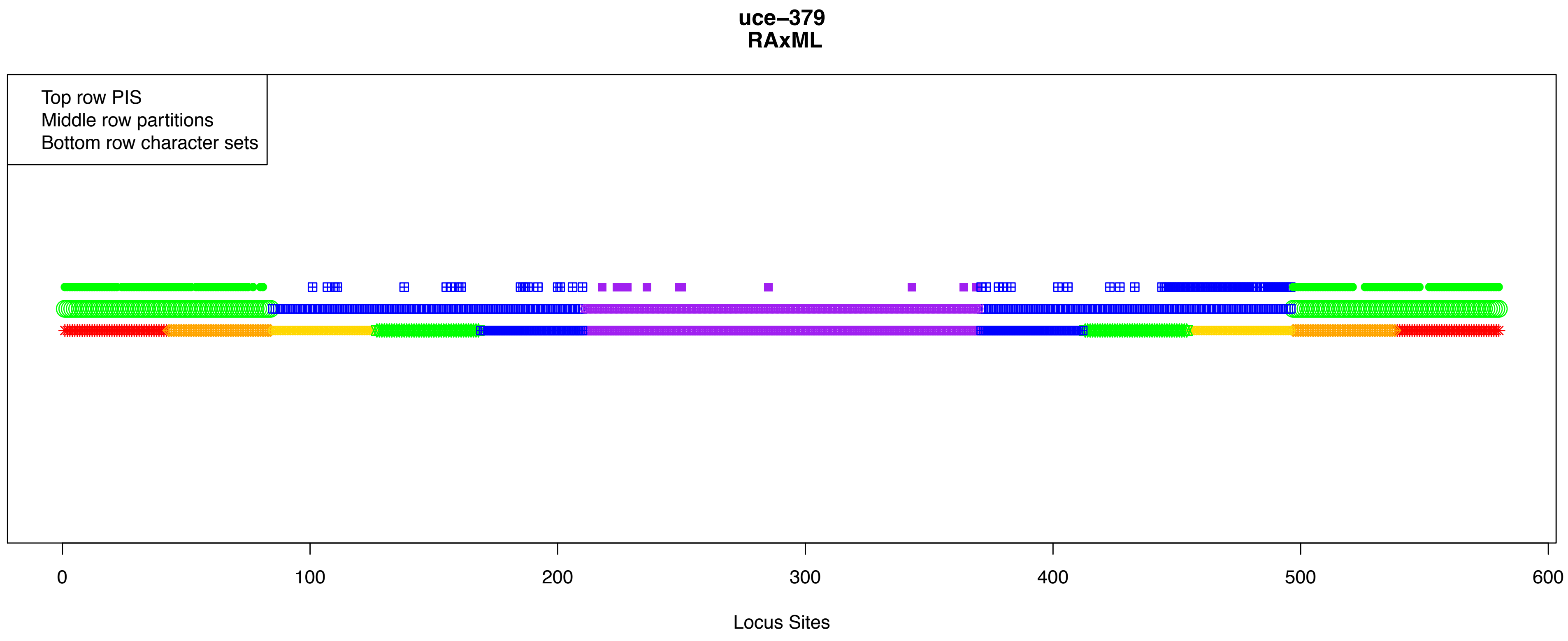
uce–379
RAxML
Top row PIS
Middle row partitions
Bottom row character sets
0
100
200
300
400
500
600
Locus Sites

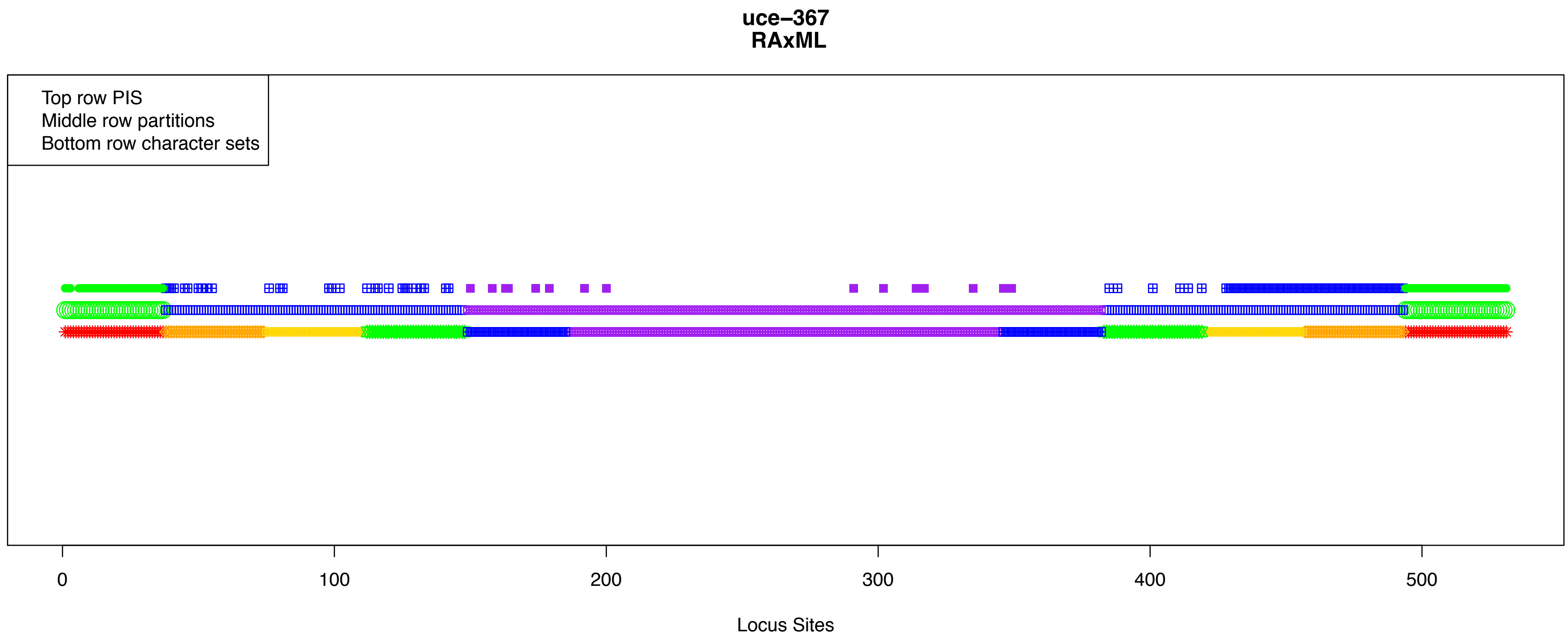
uce–367
RAxML
Top row PIS
Middle row partitions
Bottom row character sets
0
100
200
300
400
500
Locus Sites

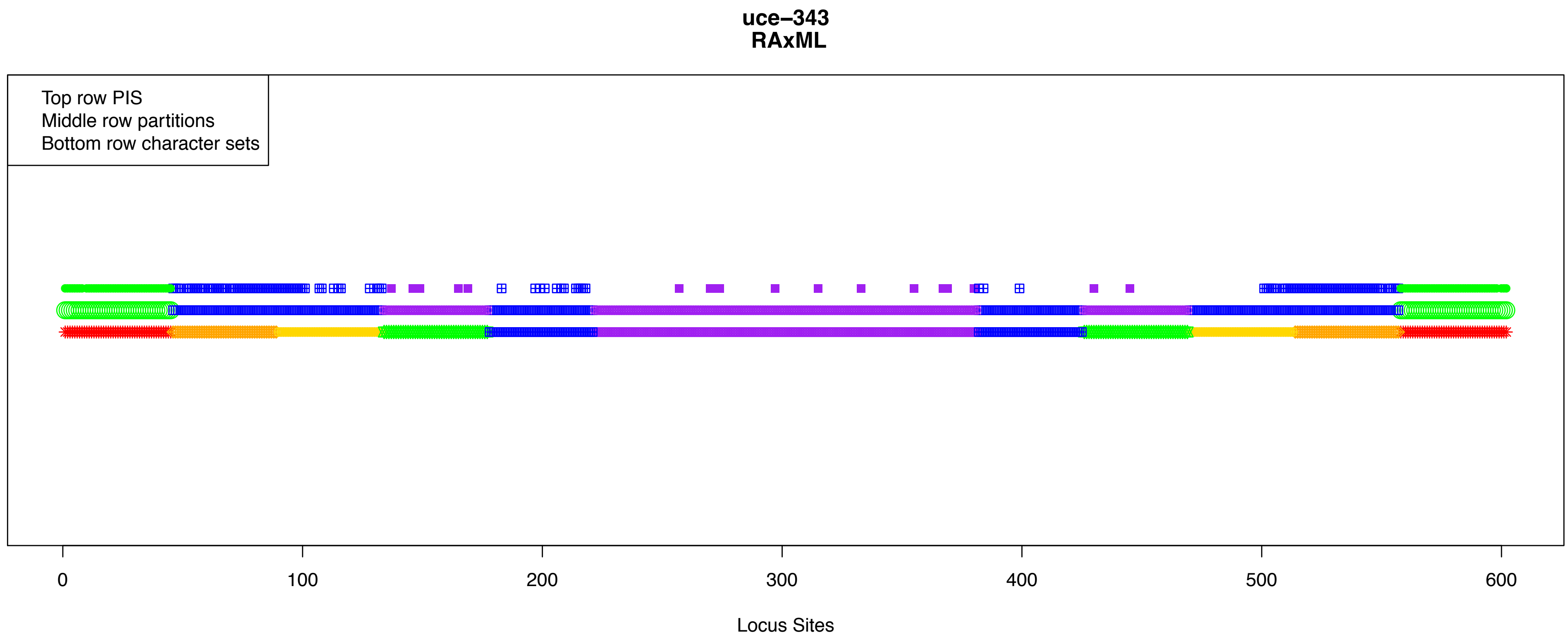
uce–343
RAxML
Top row PIS
Middle row partitions
Bottom row character sets
0
100
200
300
400
500
600
Locus Sites

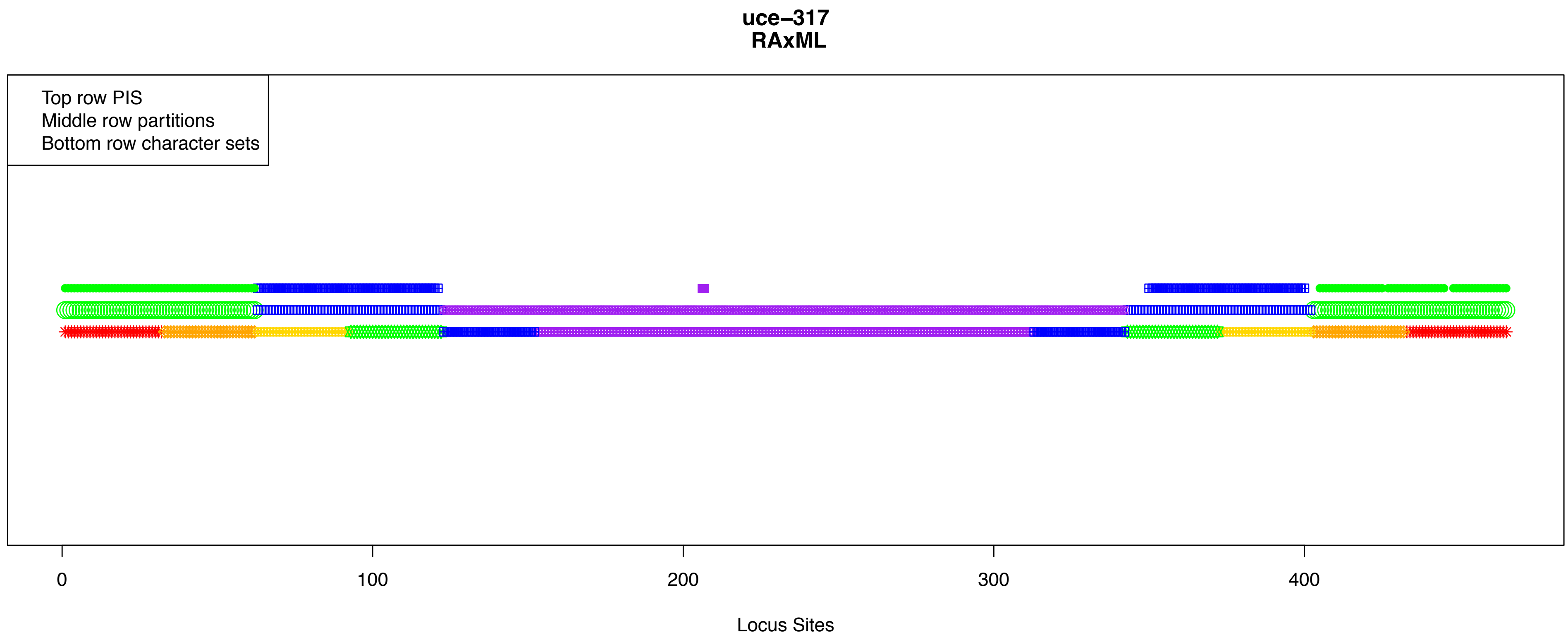
uce–317
RAxML
Top row PIS
Middle row partitions
Bottom row character sets
0
100
200
300
400
Locus Sites

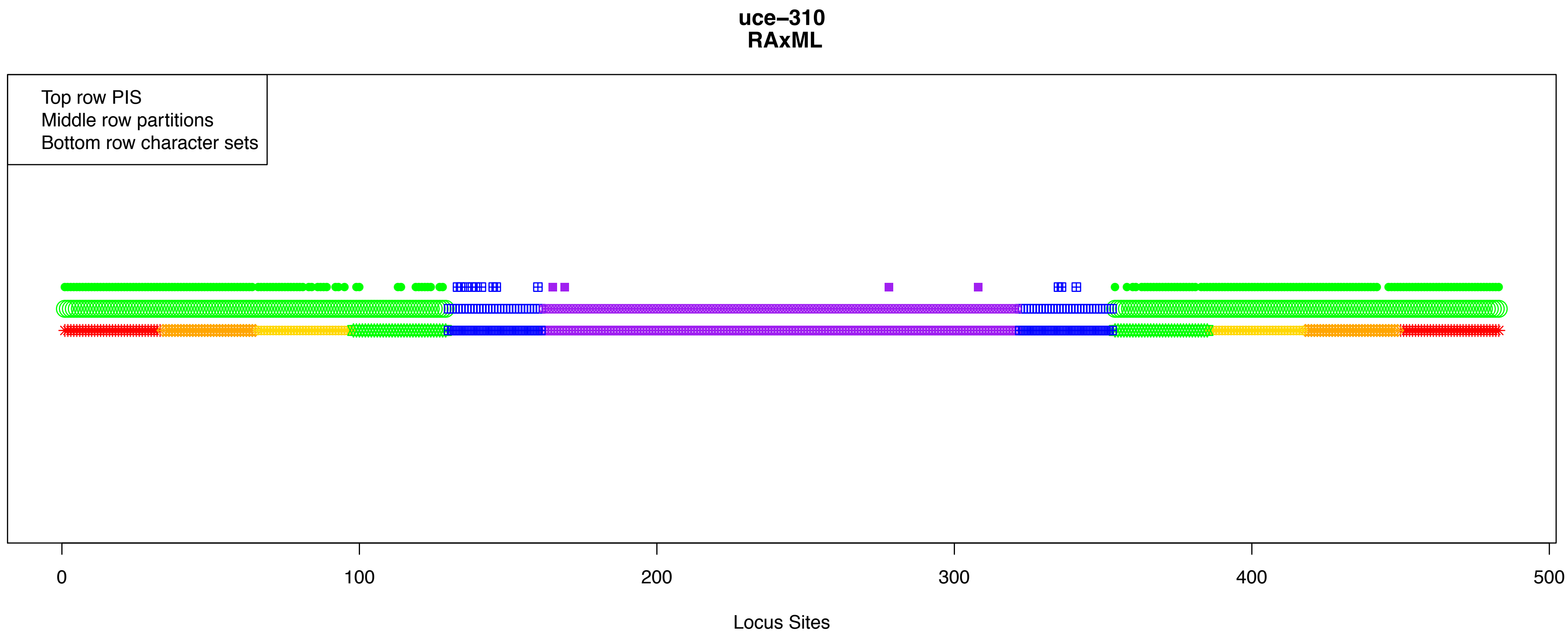
uce–310
RAxML
Top row PIS
Middle row partitions
Bottom row character sets
0
100
200
300
400
500
Locus Sites

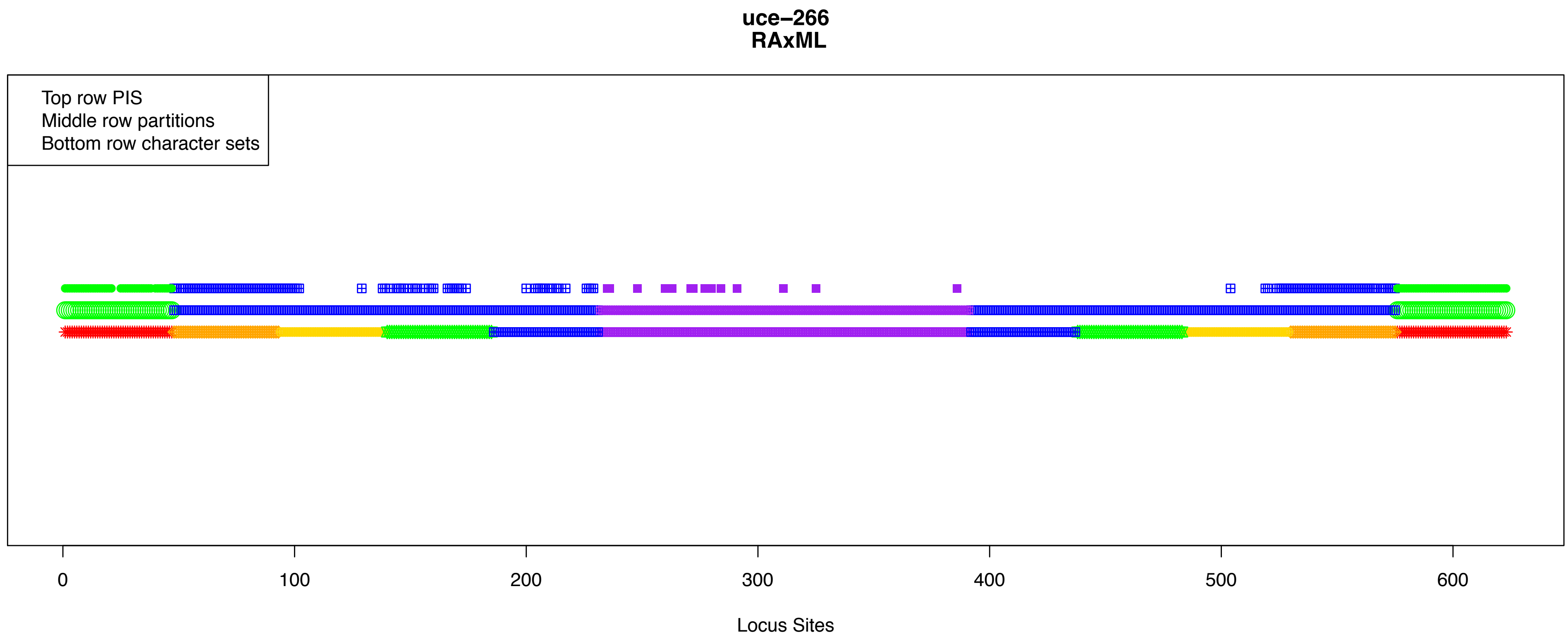
uce–266
RAxML
Top row PIS
Middle row partitions
Bottom row character sets
0
100
200
300
400
500
600
Locus Sites

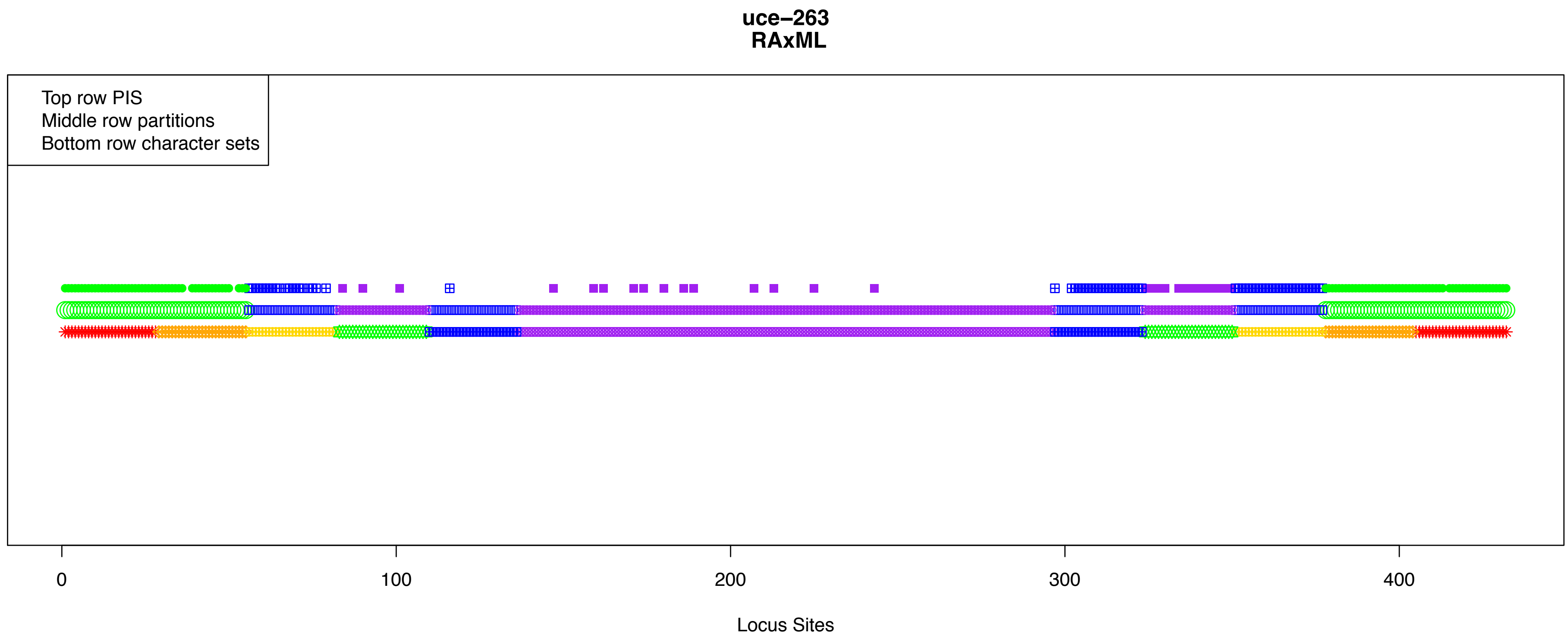

uce–263
RAxML
Top row PIS
Middle row partitions
Bottom row character sets
0
100
200
300
400
Locus Sites

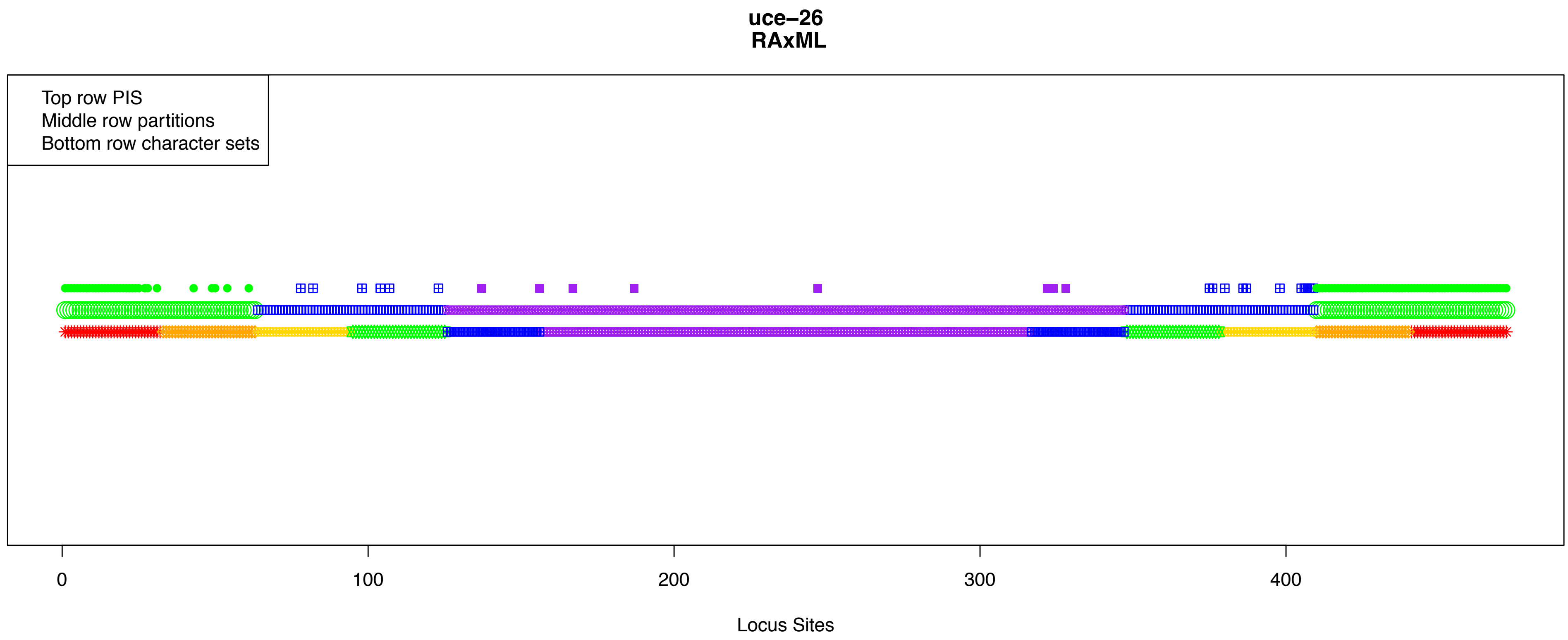

uce–26
RAxML
Top row PIS
Middle row partitions
Bottom row character sets
0
100
200
300
400
Locus Sites

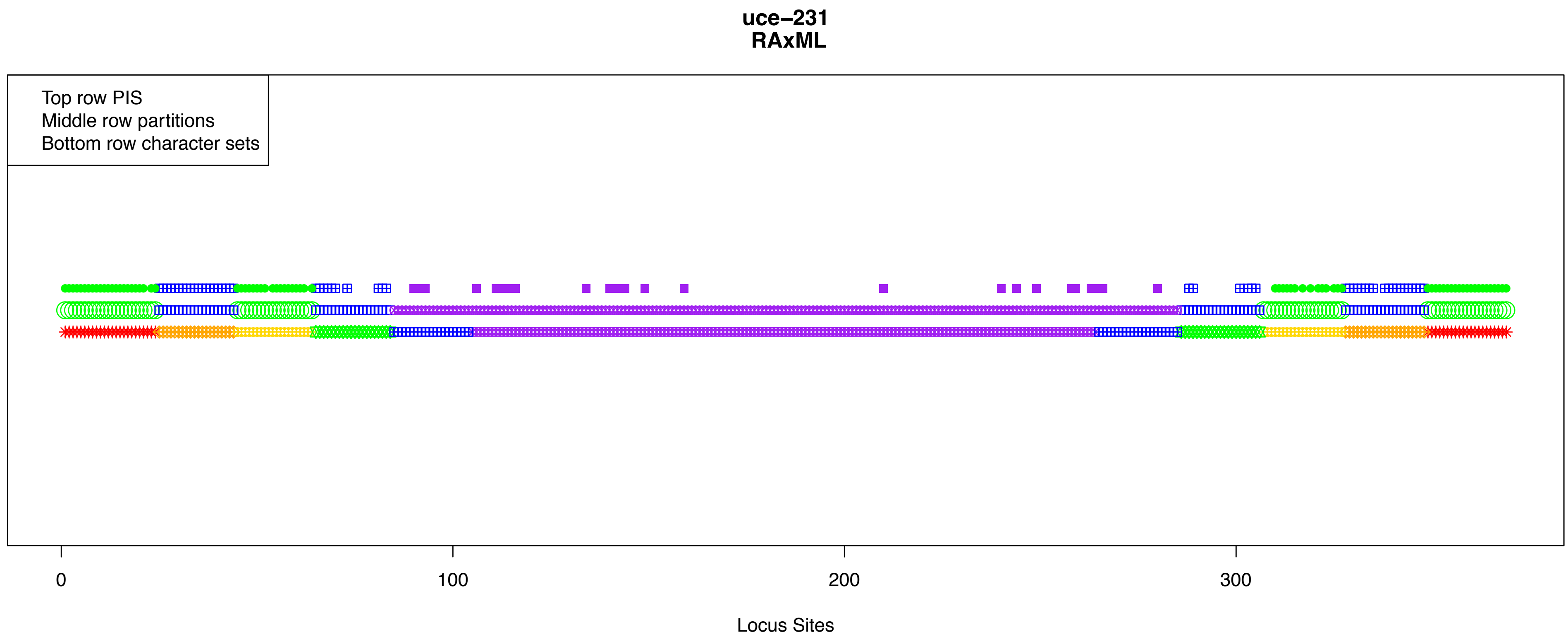

uce–231
RAxML
Top row PIS
Middle row partitions
Bottom row character sets
0
100
200
300
Locus Sites

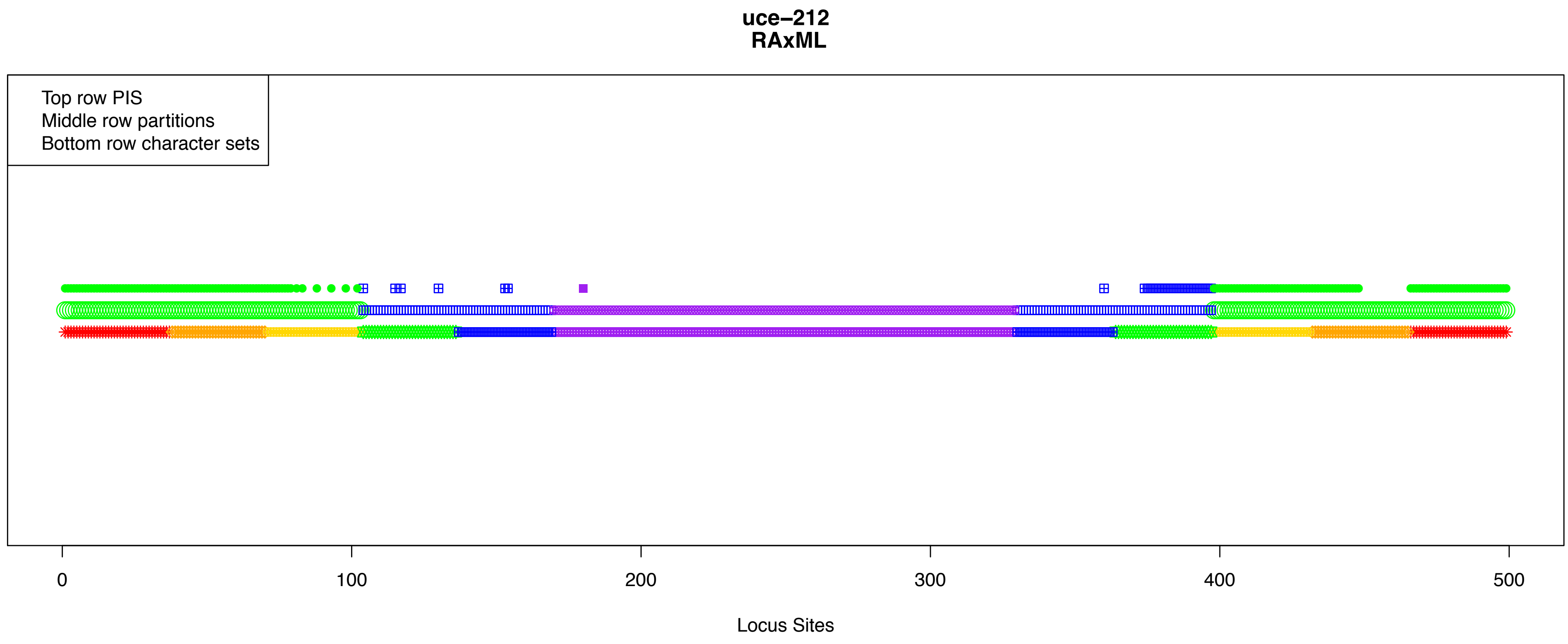
uce–212
RAxML
Top row PIS
Middle row partitions
Bottom row character sets
0
100
200
300
400
500
Locus Sites

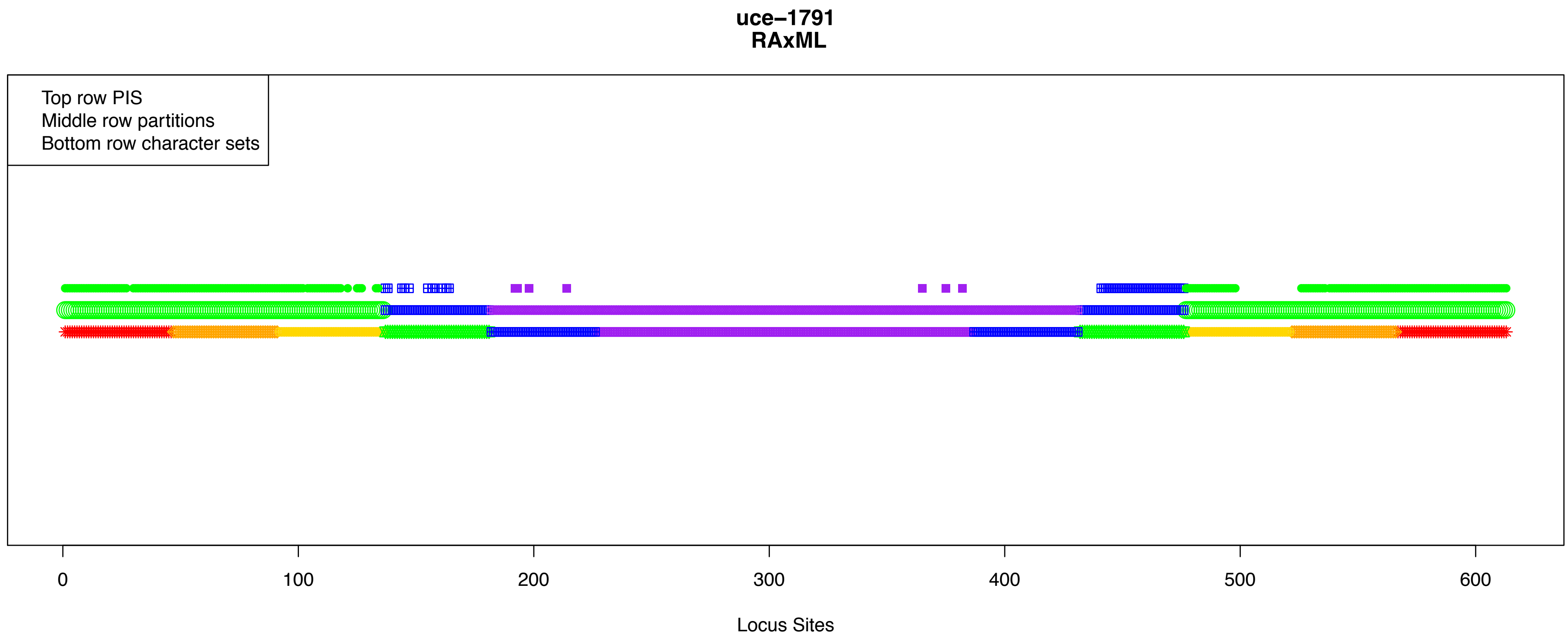

uce–1791
RAxML
Top row PIS
Middle row partitions
Bottom row character sets
0
100
200
300
400
500
600
Locus Sites

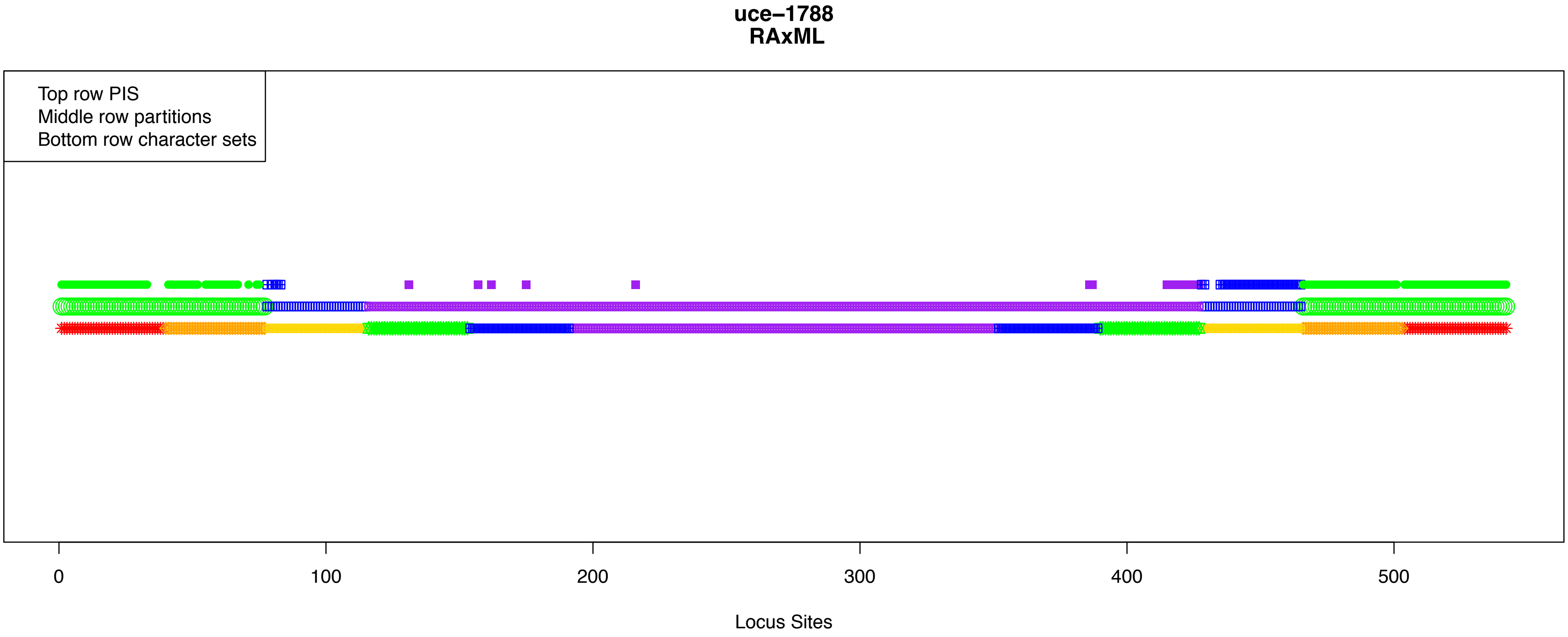
uce–1788
RAxML
Top row PIS
Middle row partitions
Bottom row character sets
0
100
200
300
400
500
Locus Sites

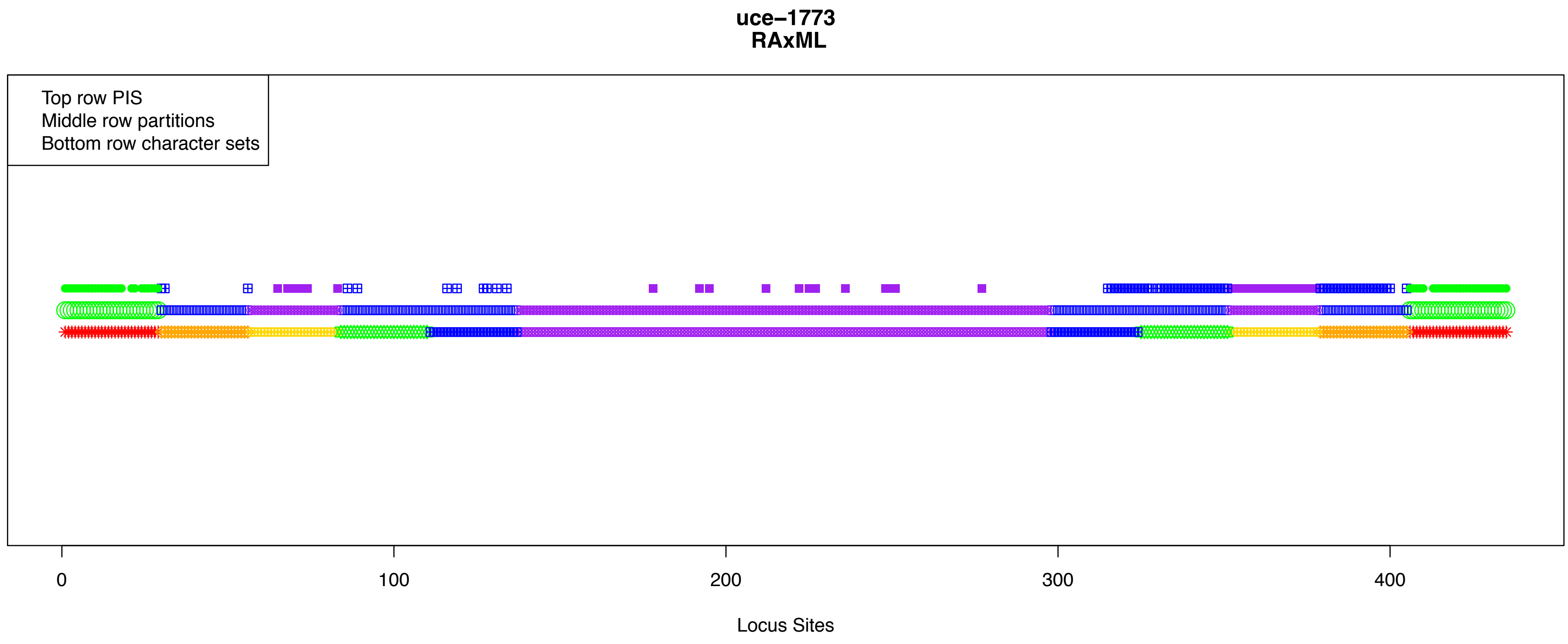

uce–1773
RAxML
Top row PIS
Middle row partitions
Bottom row character sets
0
100
200
300
400
Locus Sites

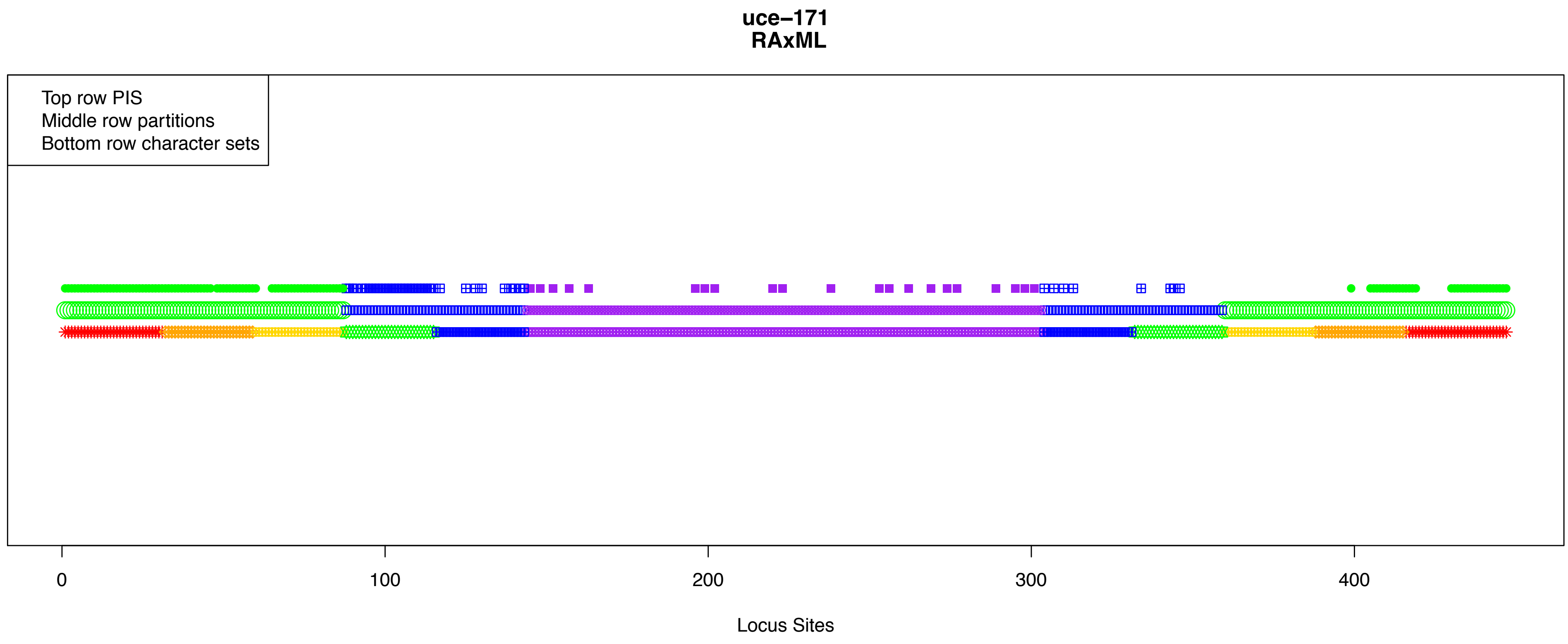

uce–171
RAxML
Top row PIS
Middle row partitions
Bottom row character sets
0
100
200
300
400
Locus Sites

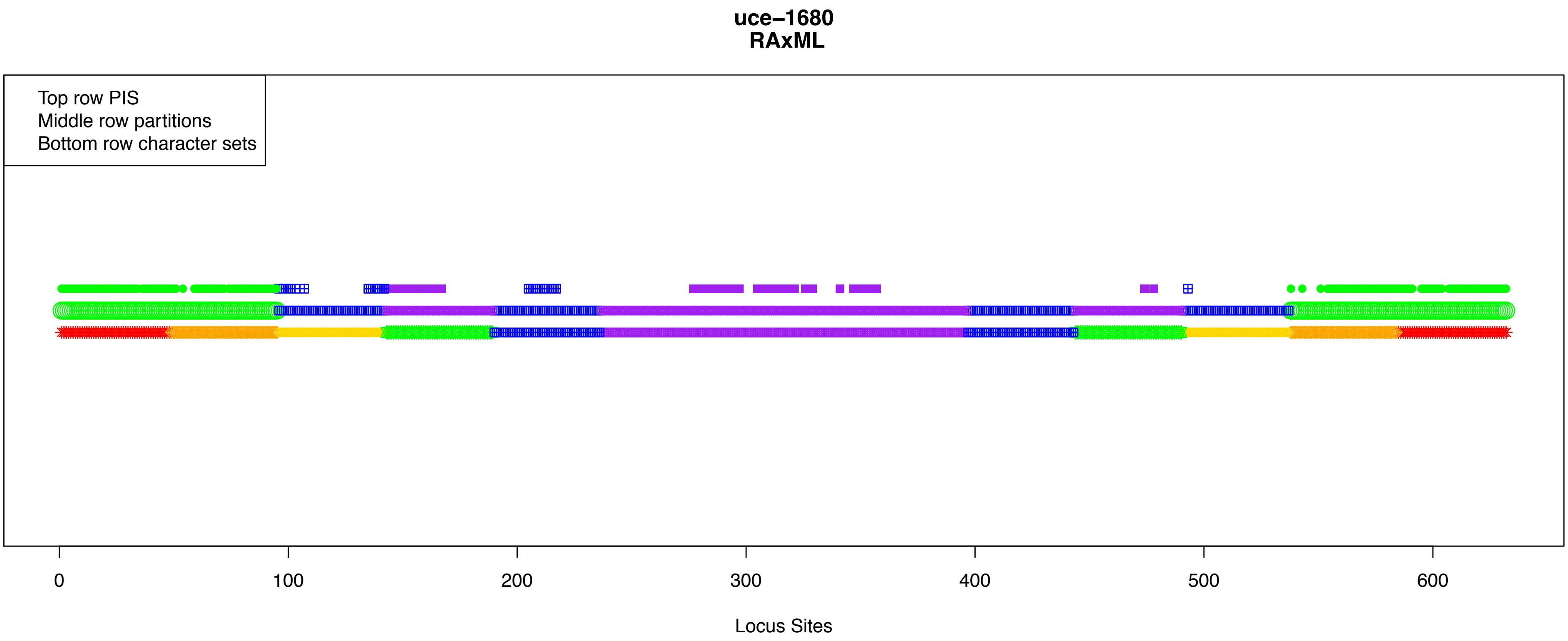
uce–1680
RAxML
Top row PIS
Middle row partitions
Bottom row character sets
0
100
200
300
400
500
600
Locus Sites

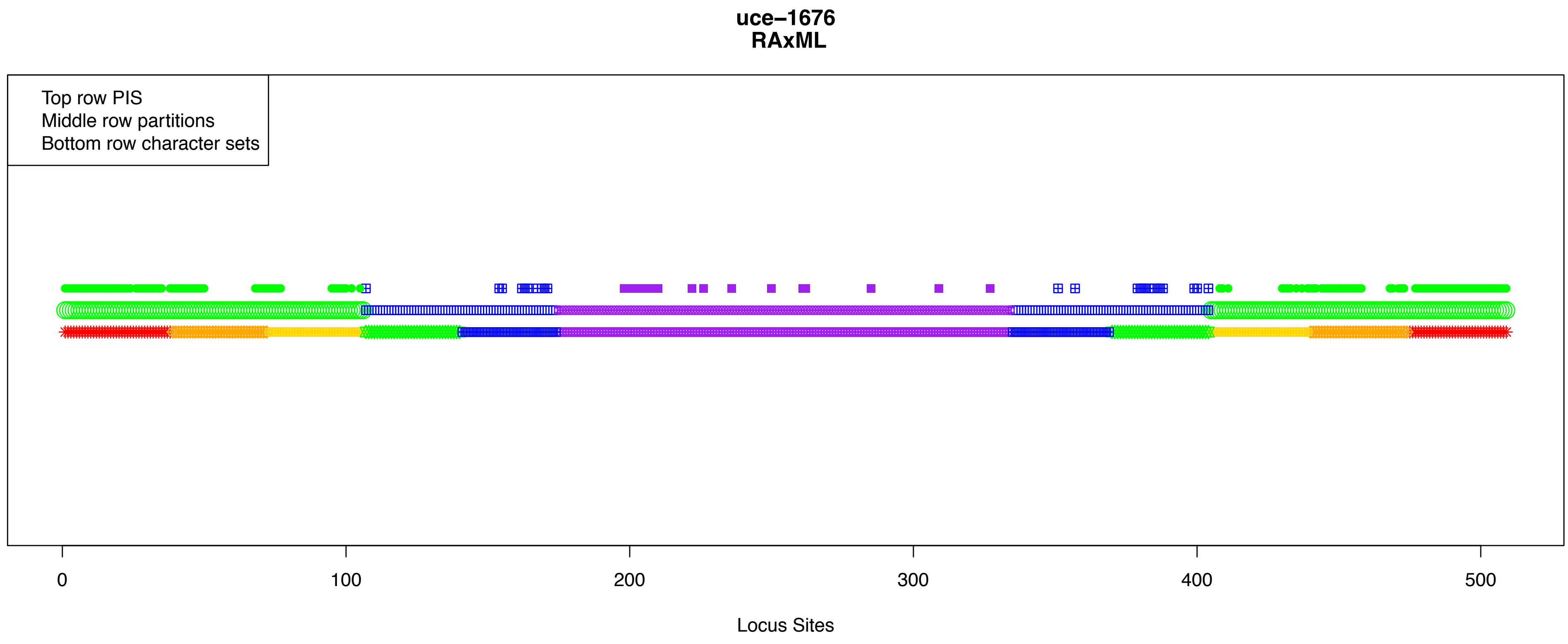
uce–1676
RAxML
Top row PIS
Middle row partitions
Bottom row character sets
0
100
200
300
400
500
Locus Sites

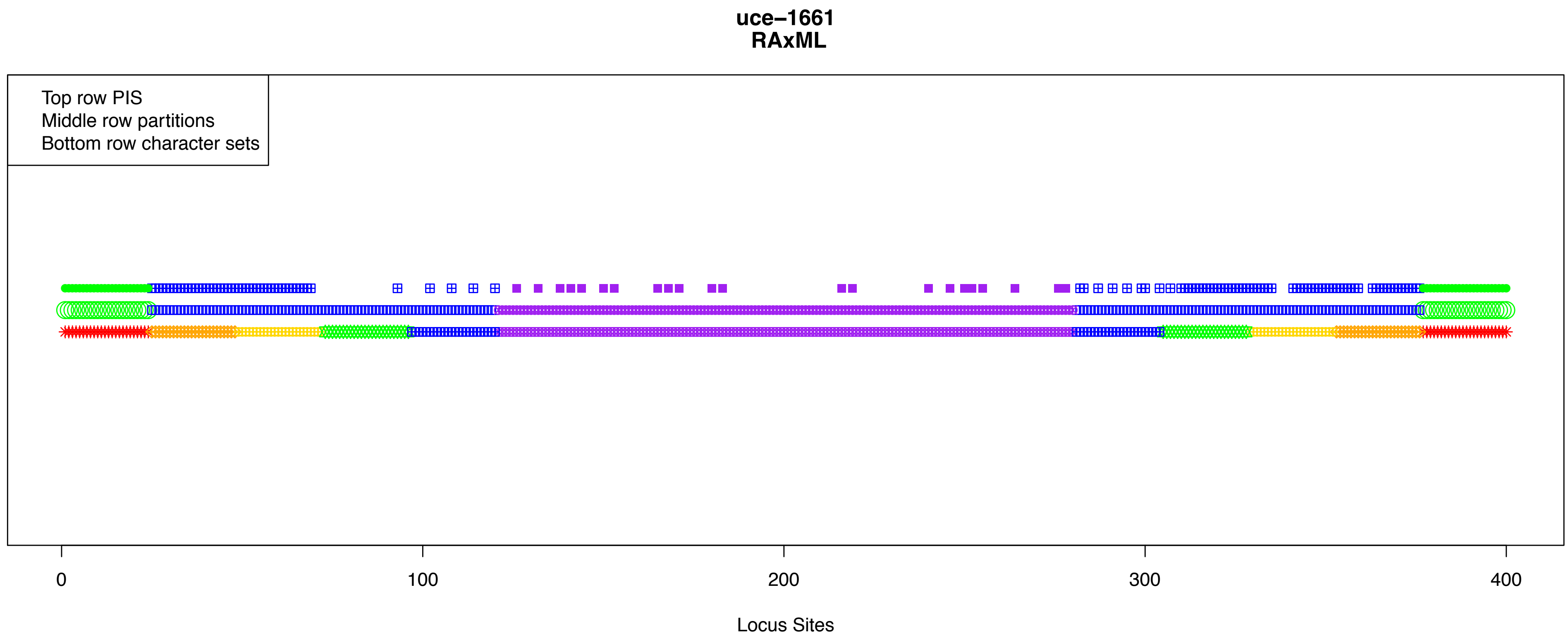
uce–1661
RAxML
Top row PIS
Middle row partitions
Bottom row character sets
0
100
200
300
400
Locus Sites

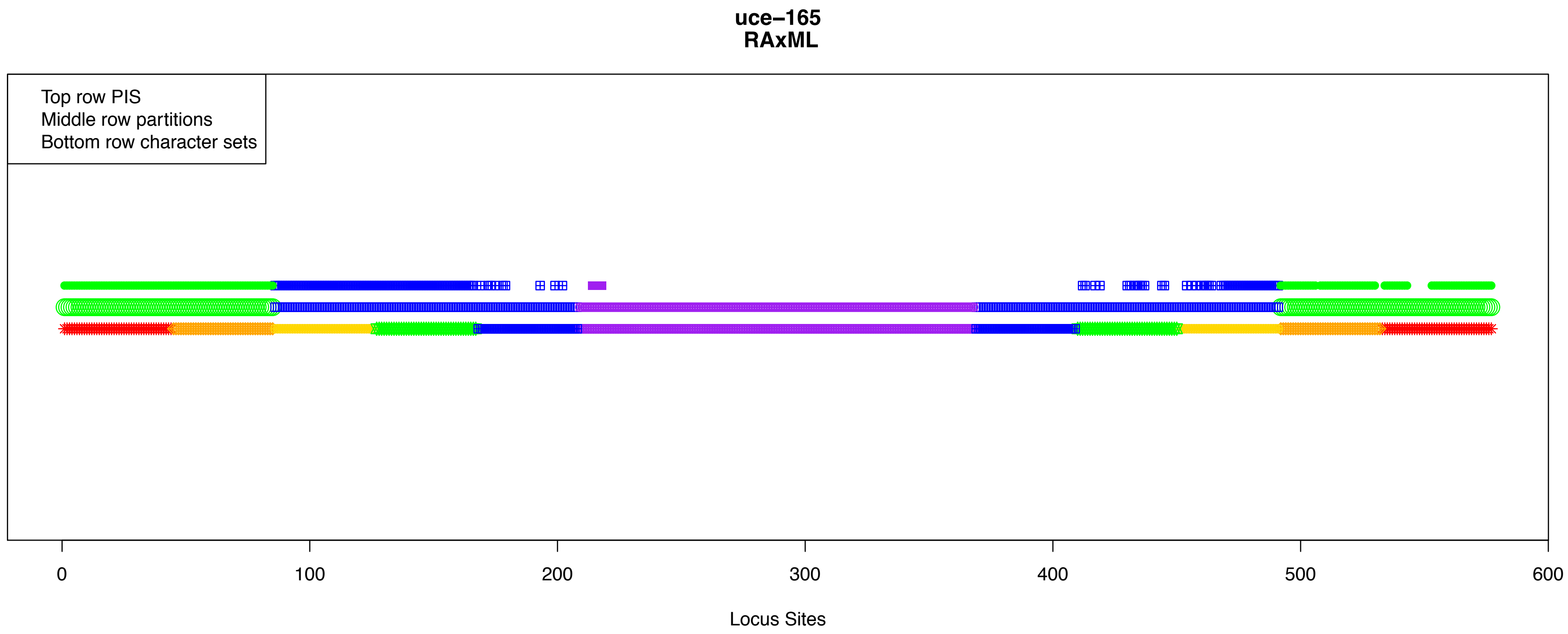

uce–165
RAxML
Top row PIS
Middle row partitions
Bottom row character sets
0
100
200
300
400
500
600
Locus Sites

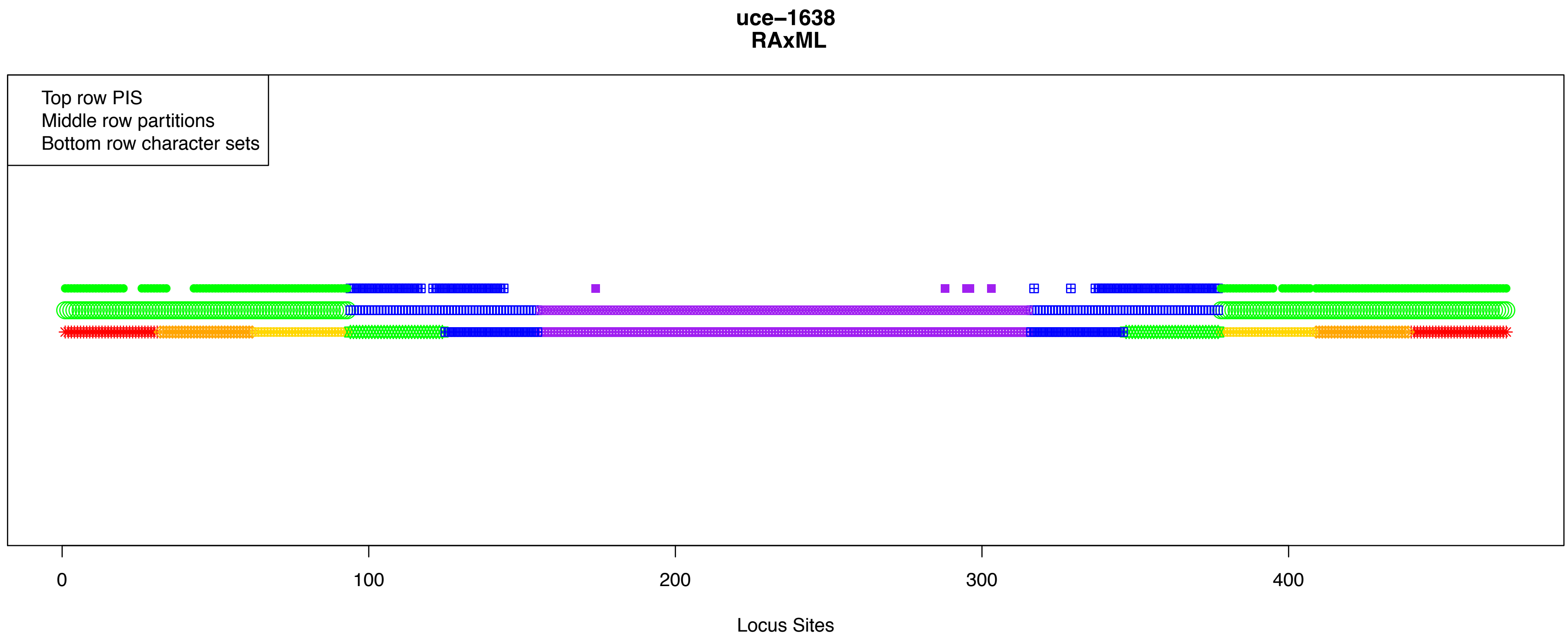
uce–1638
RAxML
Top row PIS
Middle row partitions
Bottom row character sets
0
100
200
300
400
Locus Sites

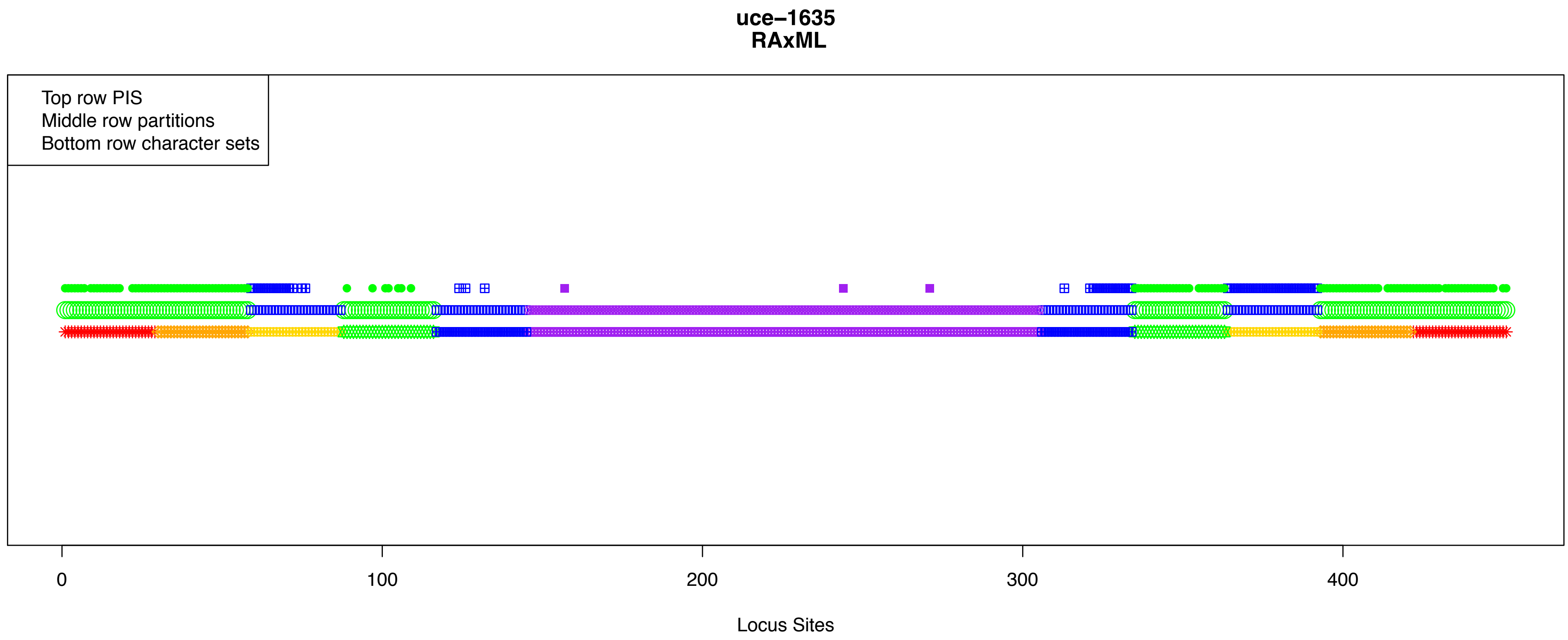
uce–1635
RAxML
Top row PIS
Middle row partitions
Bottom row character sets
0
100
200
300
400
Locus Sites

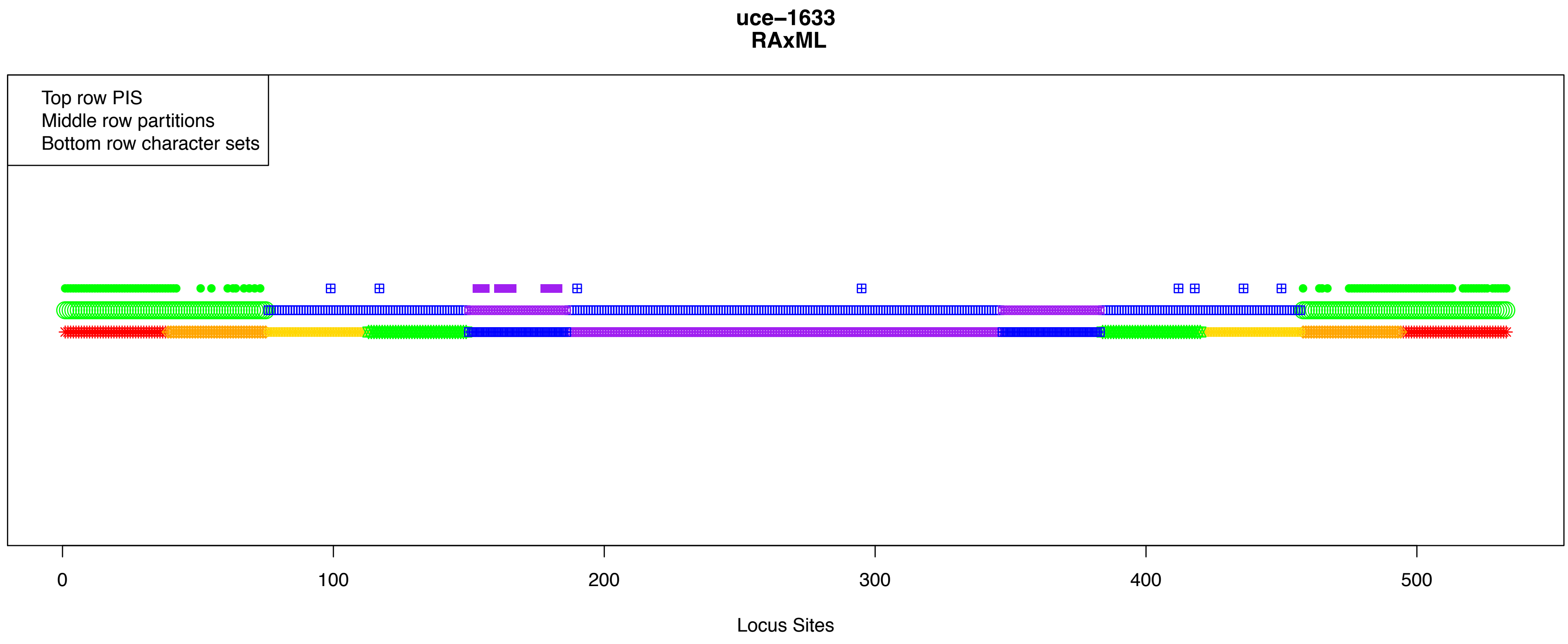
uce–1633
RAxML
Top row PIS
Middle row partitions
Bottom row character sets
0
100
200
300
400
500
Locus Sites

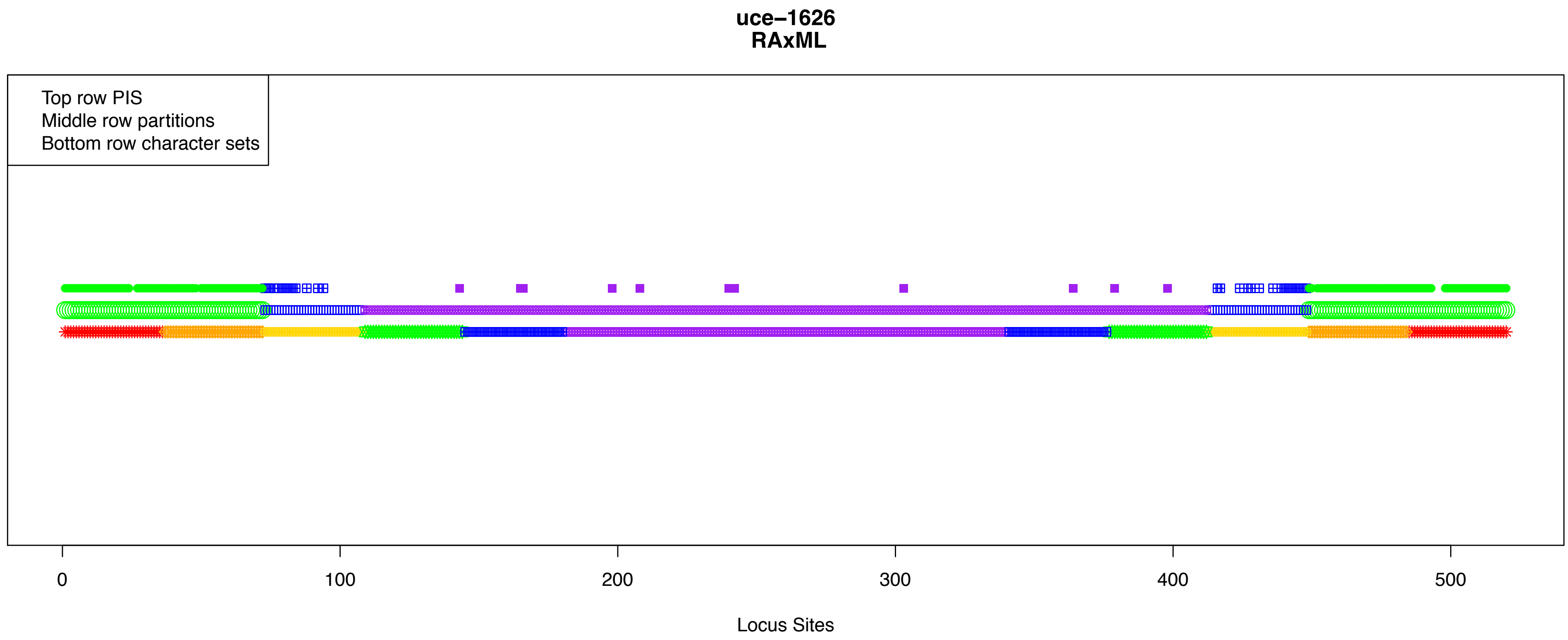

uce–1626
RAxML
Top row PIS
Middle row partitions
Bottom row character sets
0
100
200
300
400
500
Locus Sites

uce–1624
RAxML

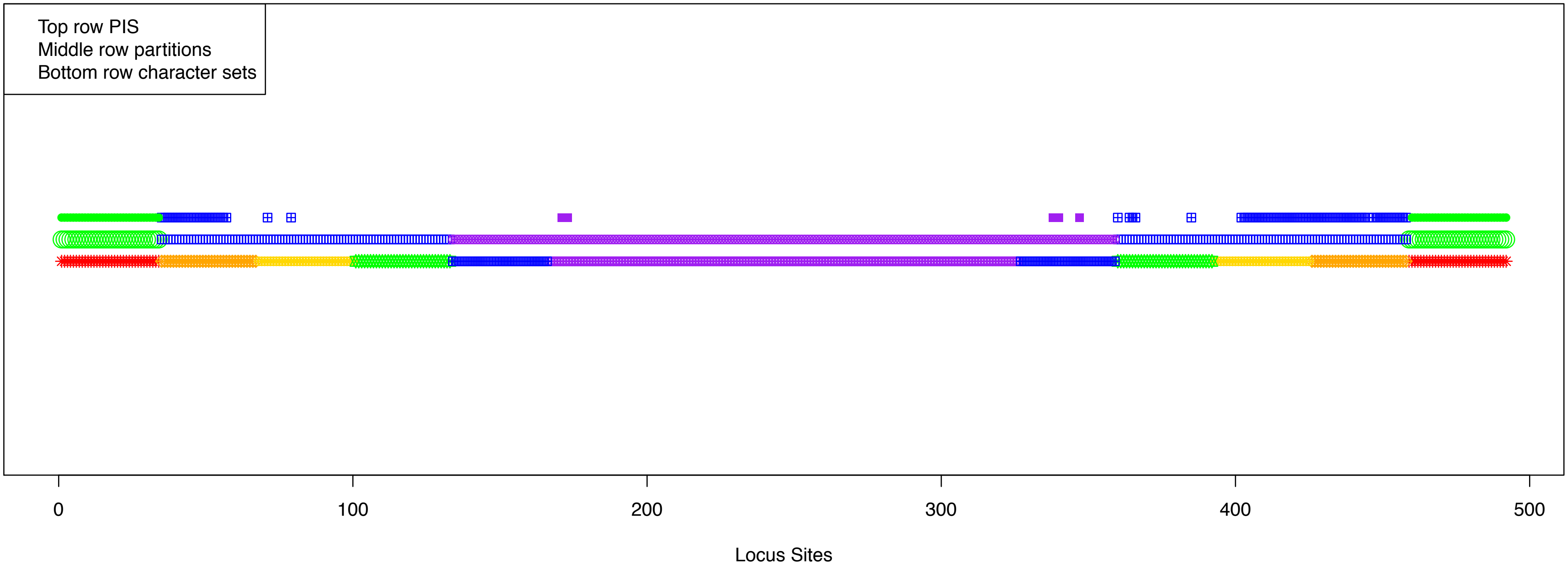

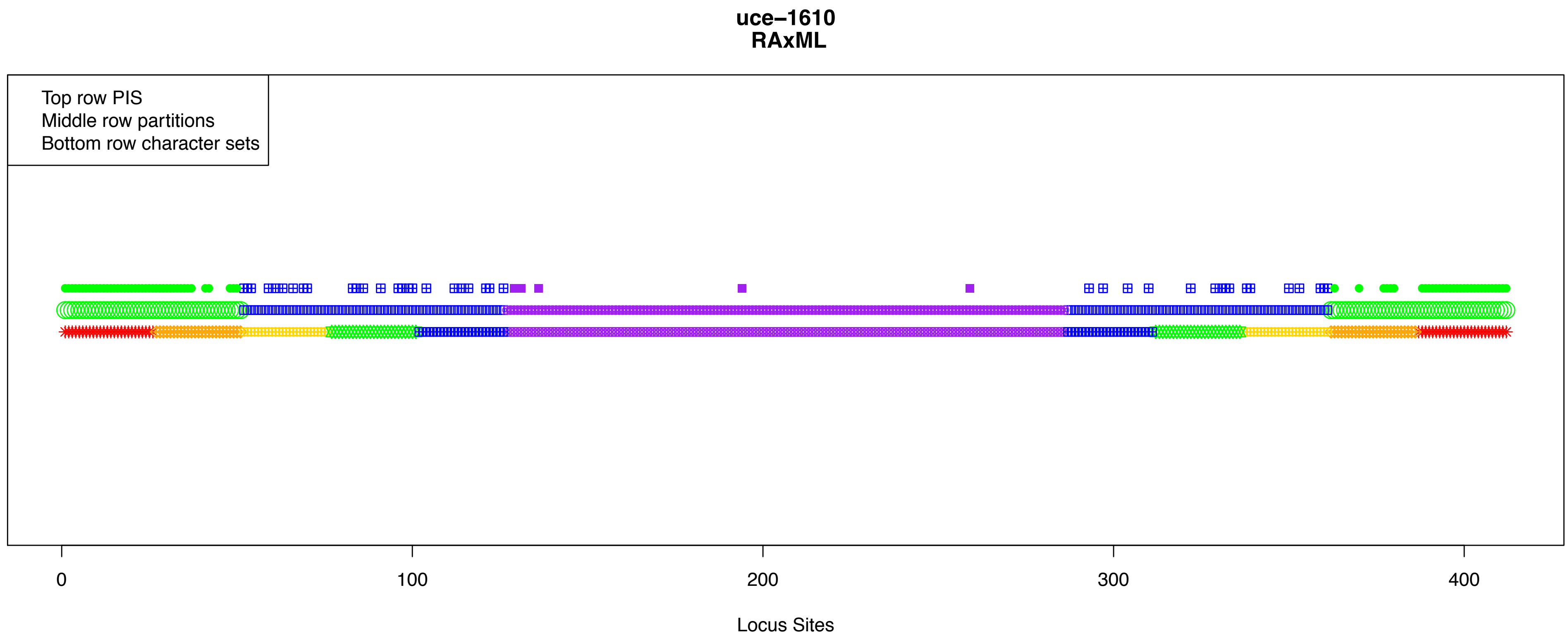

uce–1610
RAxML
Top row PIS
Middle row partitions
Bottom row character sets
0
100
200
300
400
Locus Sites

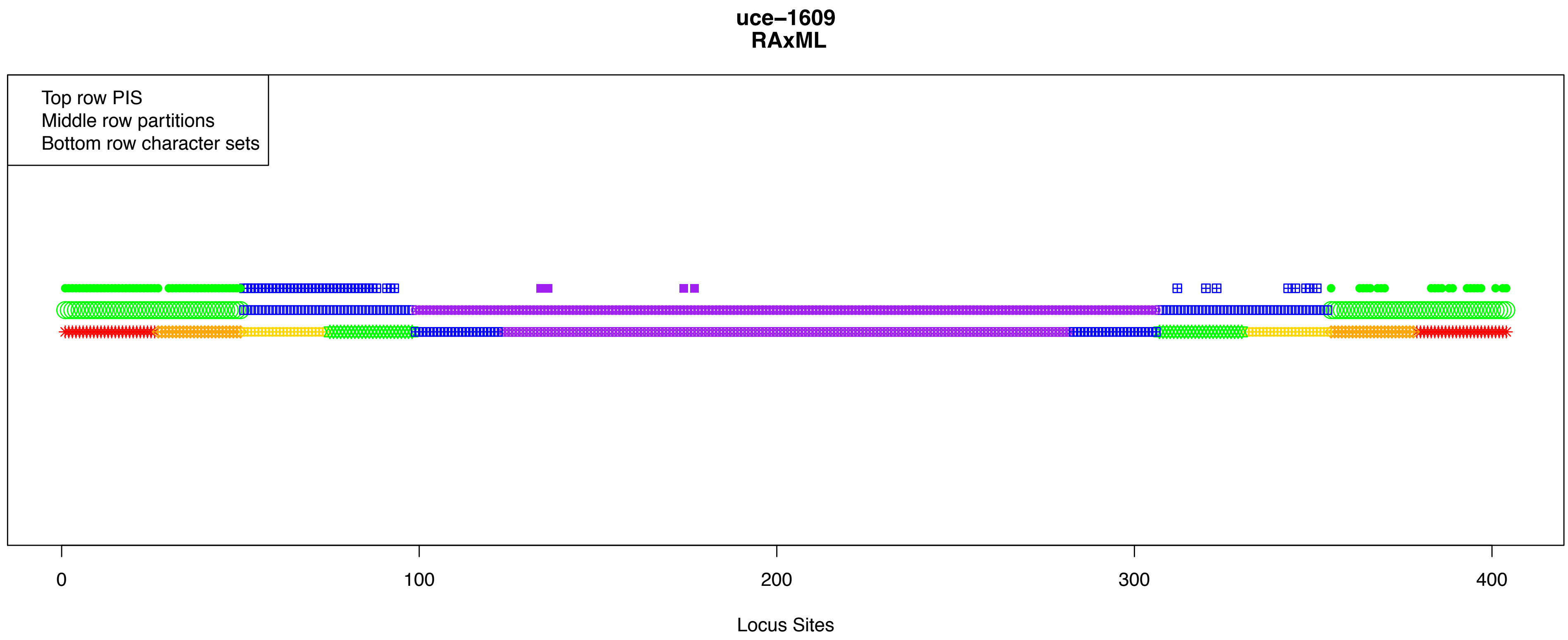
uce–1609
RAxML
Top row PIS
Middle row partitions
Bottom row character sets
0
100
200
300
400
Locus Sites

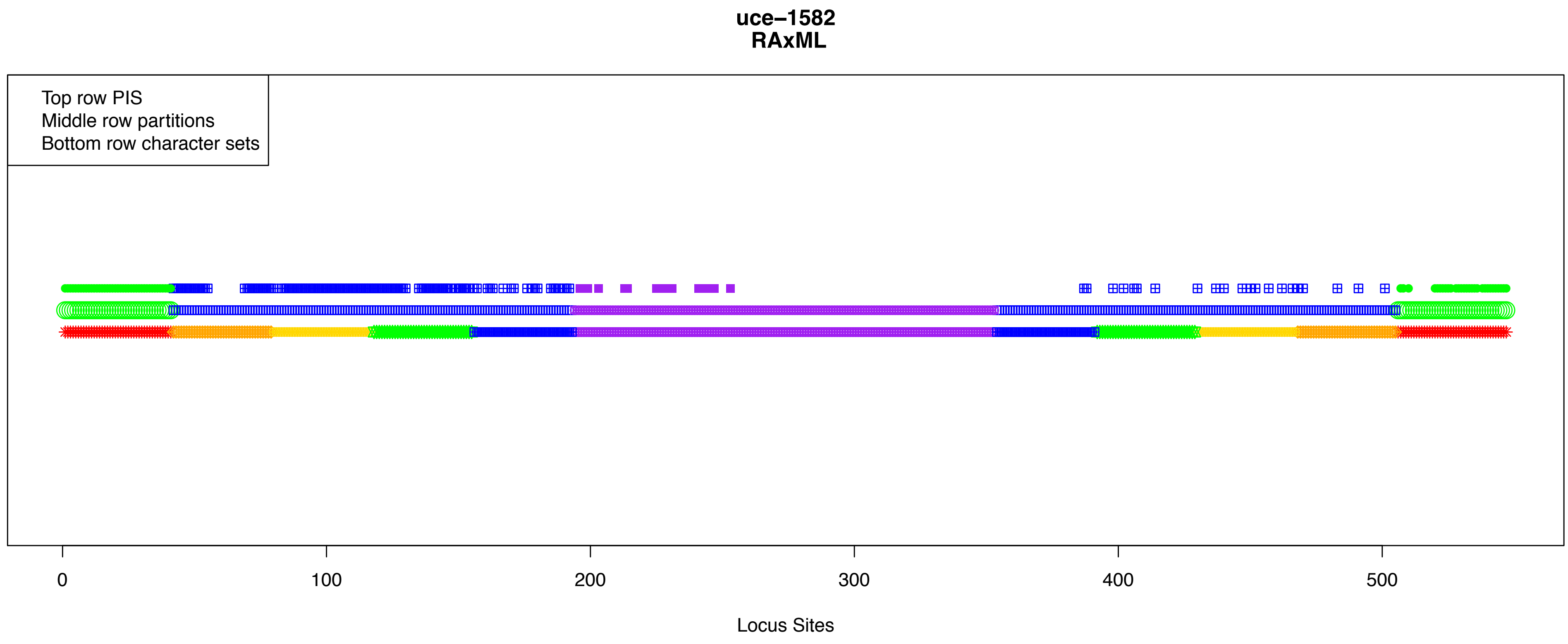

uce–1582
RAxML
Top row PIS
Middle row partitions
Bottom row character sets
0
100
200
300
400
500
Locus Sites

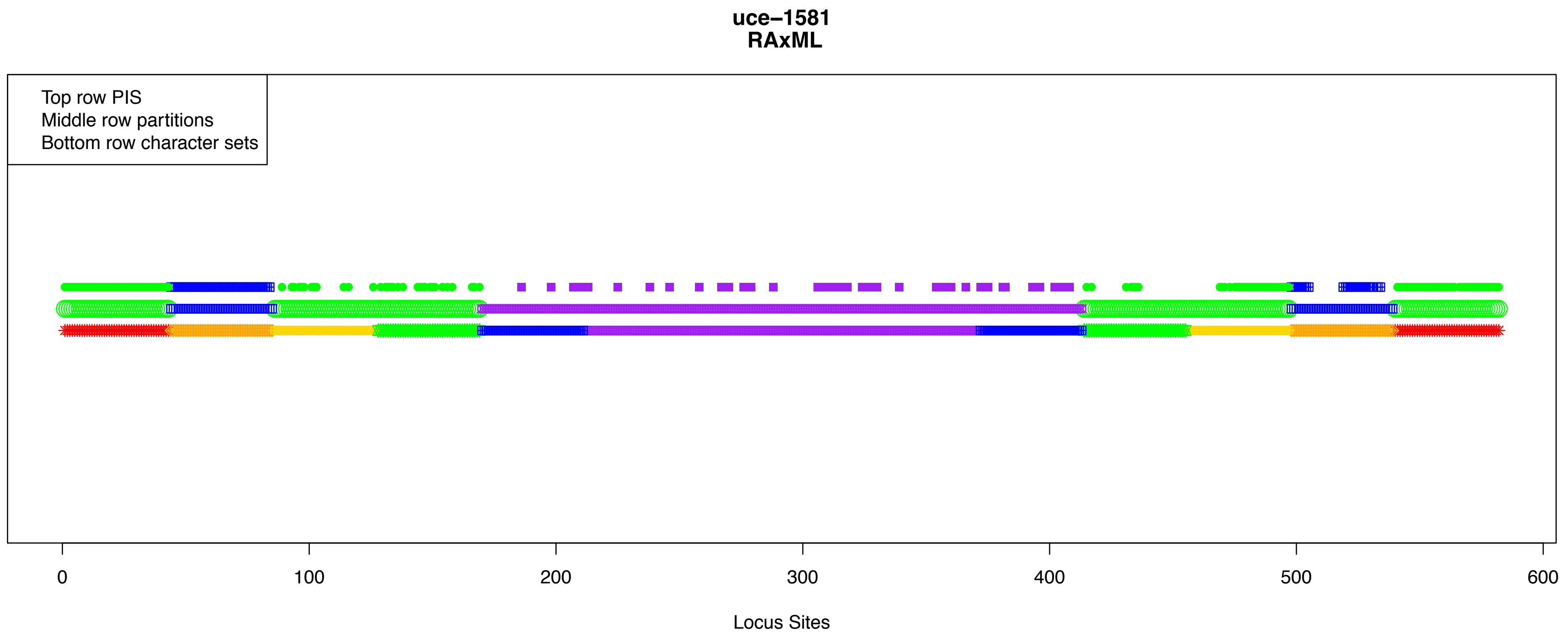
uce–1581
RAxML
Top row PIS
Middle row partitions
Bottom row character sets
0
100
200
300
400
500
600
Locus Sites

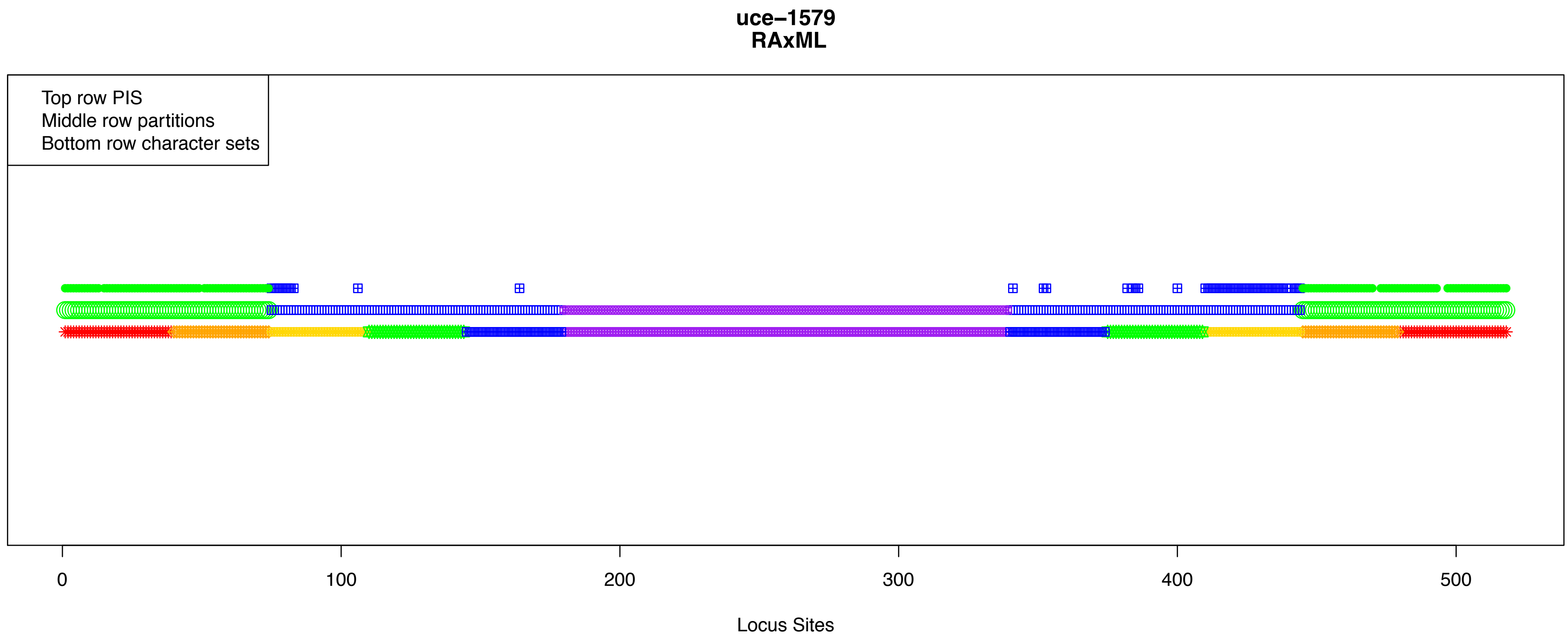
uce–1579
RAxML
Top row PIS
Middle row partitions
Bottom row character sets
0
100
200
300
400
500
Locus Sites

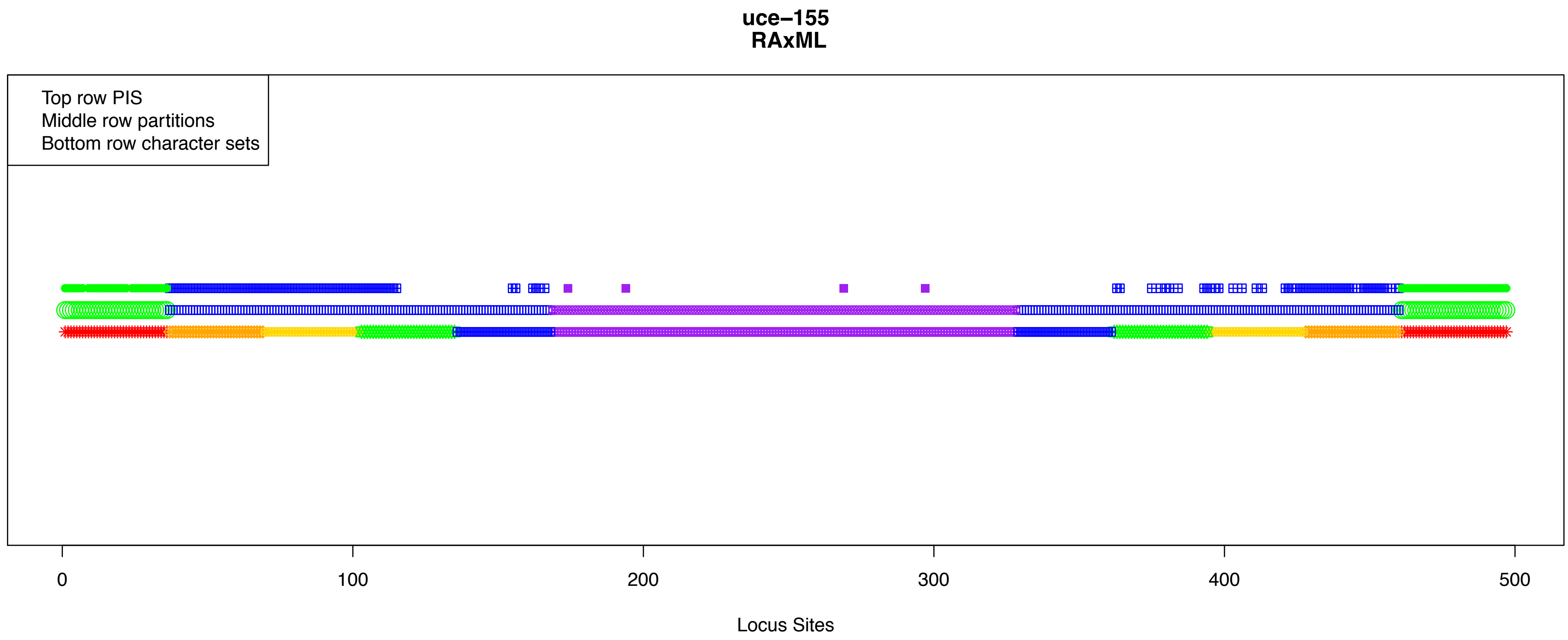
uce–155
RAxML
Top row PIS
Middle row partitions
Bottom row character sets
0
100
200
300
400
500
Locus Sites

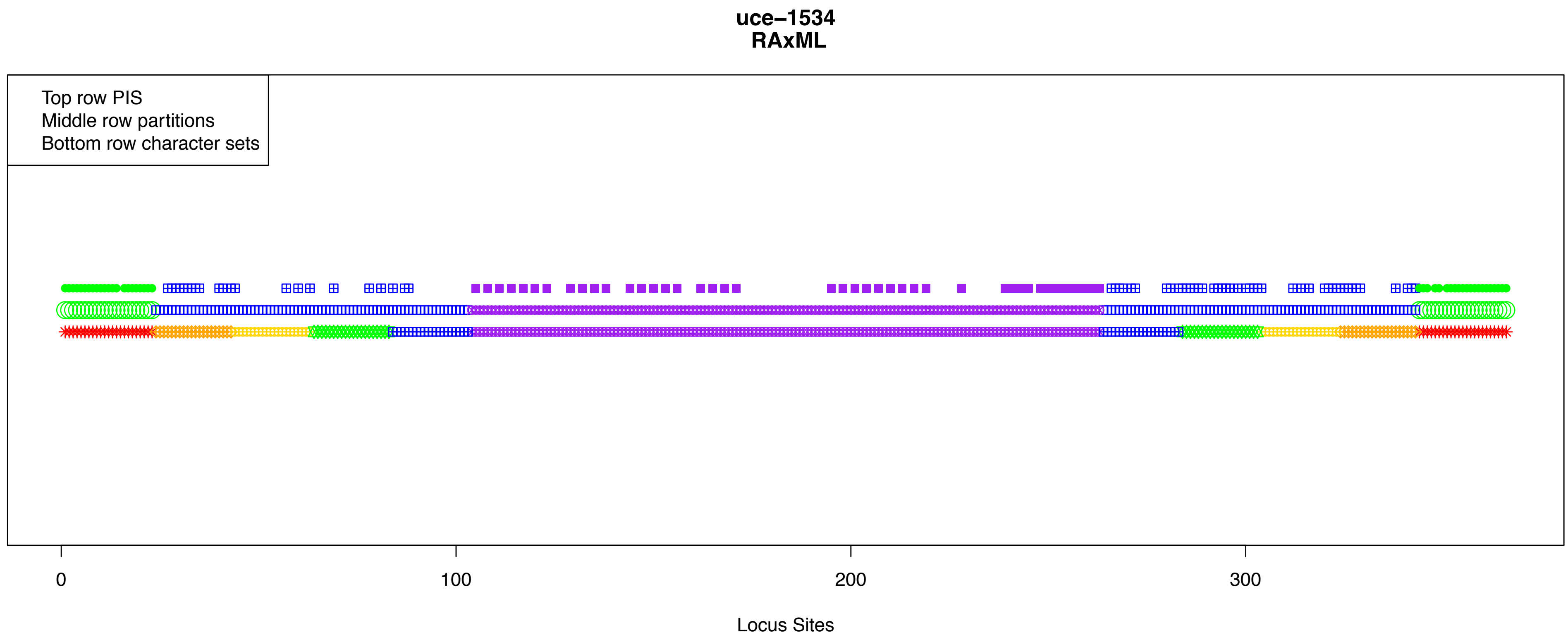

uce–1534
RAxML
Top row PIS
Middle row partitions
Bottom row character sets
0
100
200
300
Locus Sites

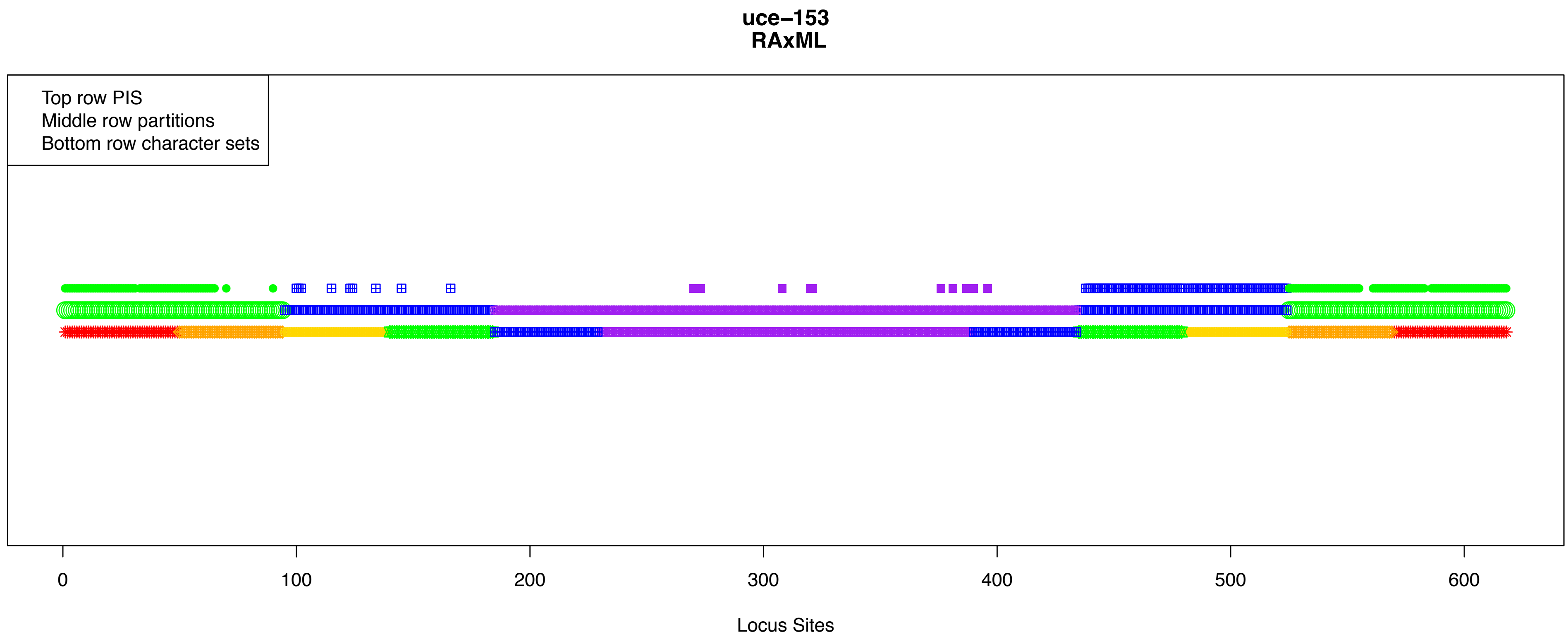
uce–153
RAxML
Top row PIS
Middle row partitions
Bottom row character sets
0
100
200
300
400
500
600
Locus Sites

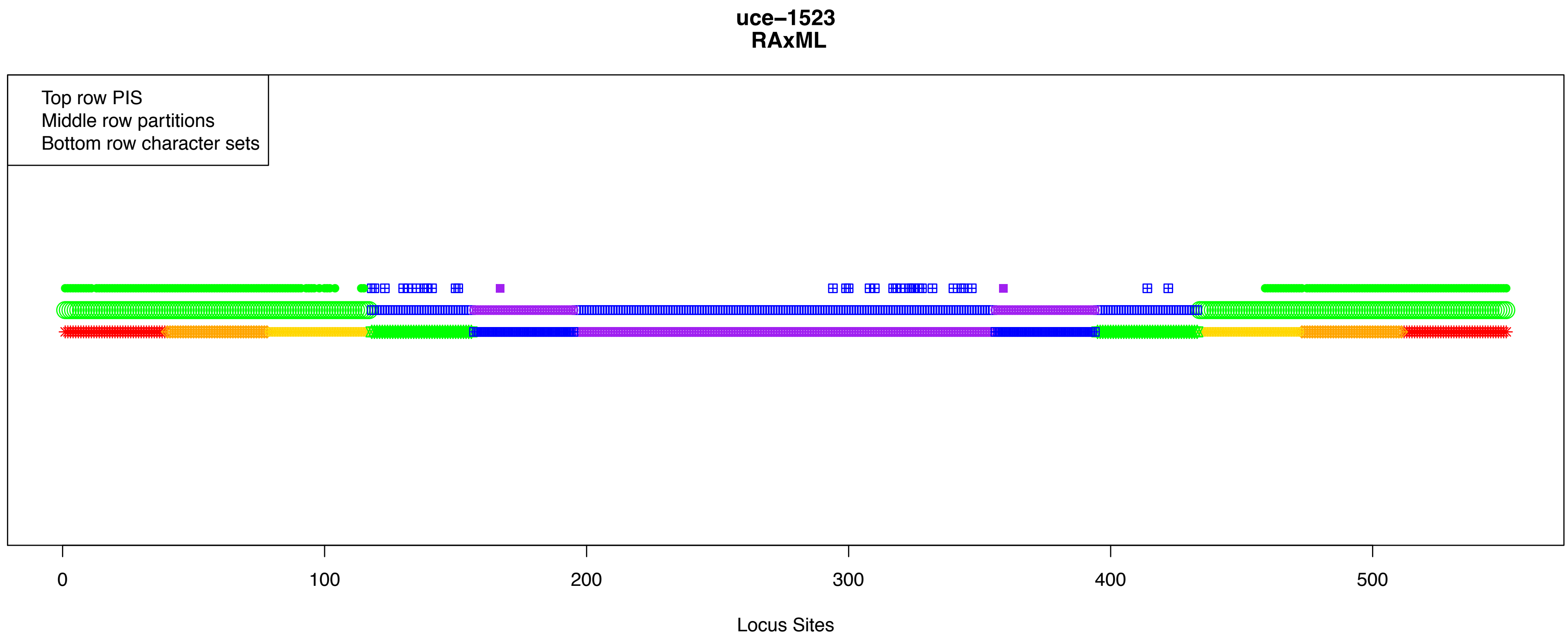
uce–1523
RAxML
Top row PIS
Middle row partitions
Bottom row character sets
0
100
200
300
400
500
Locus Sites

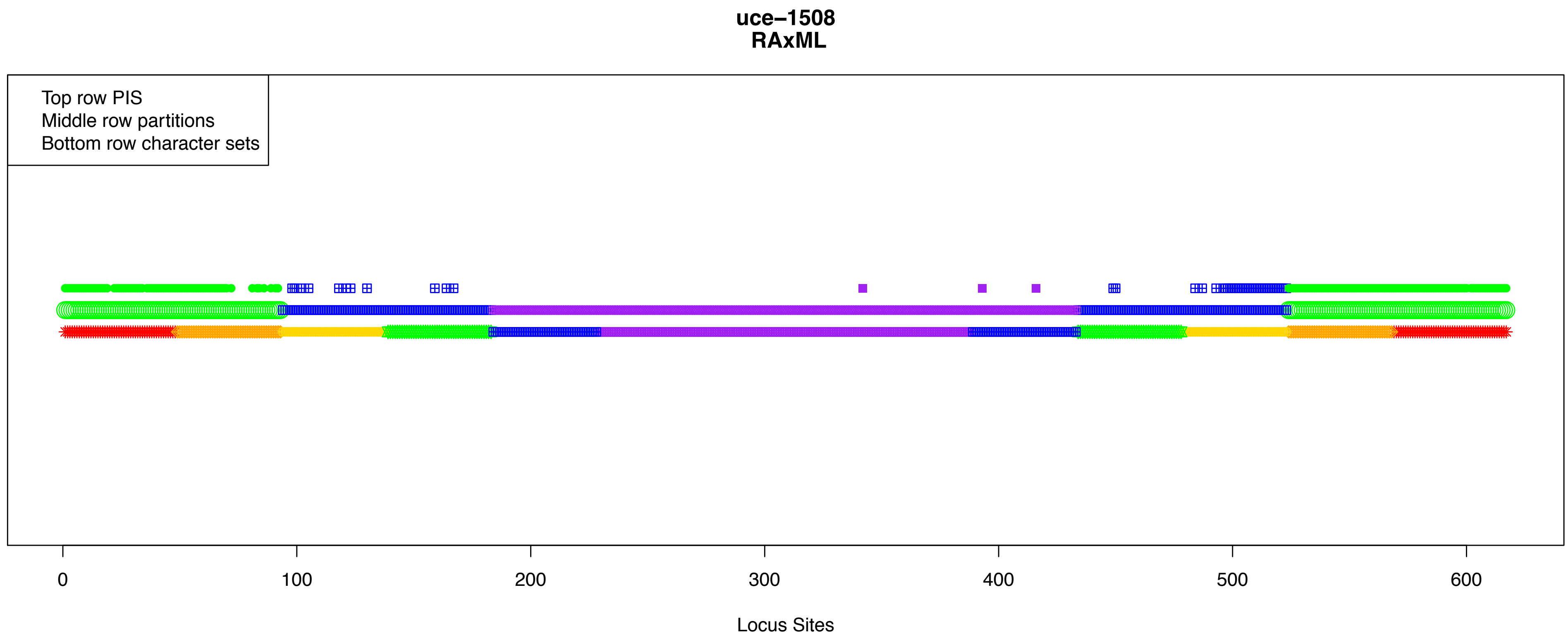
uce–1508
RAxML
Top row PIS
Middle row partitions
Bottom row character sets
0
100
200
300
400
500
600
Locus Sites

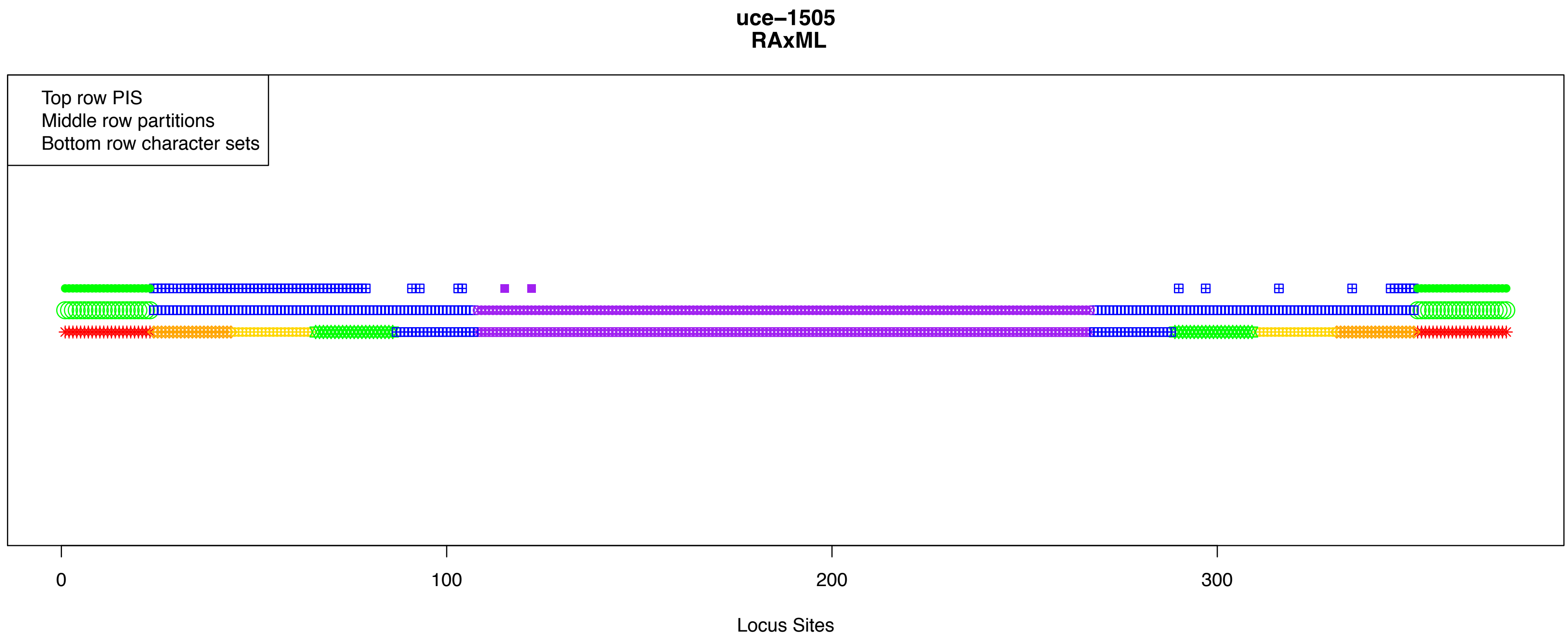

uce–1505
RAxML
Top row PIS
Middle row partitions
Bottom row character sets
0
100
200
300
Locus Sites

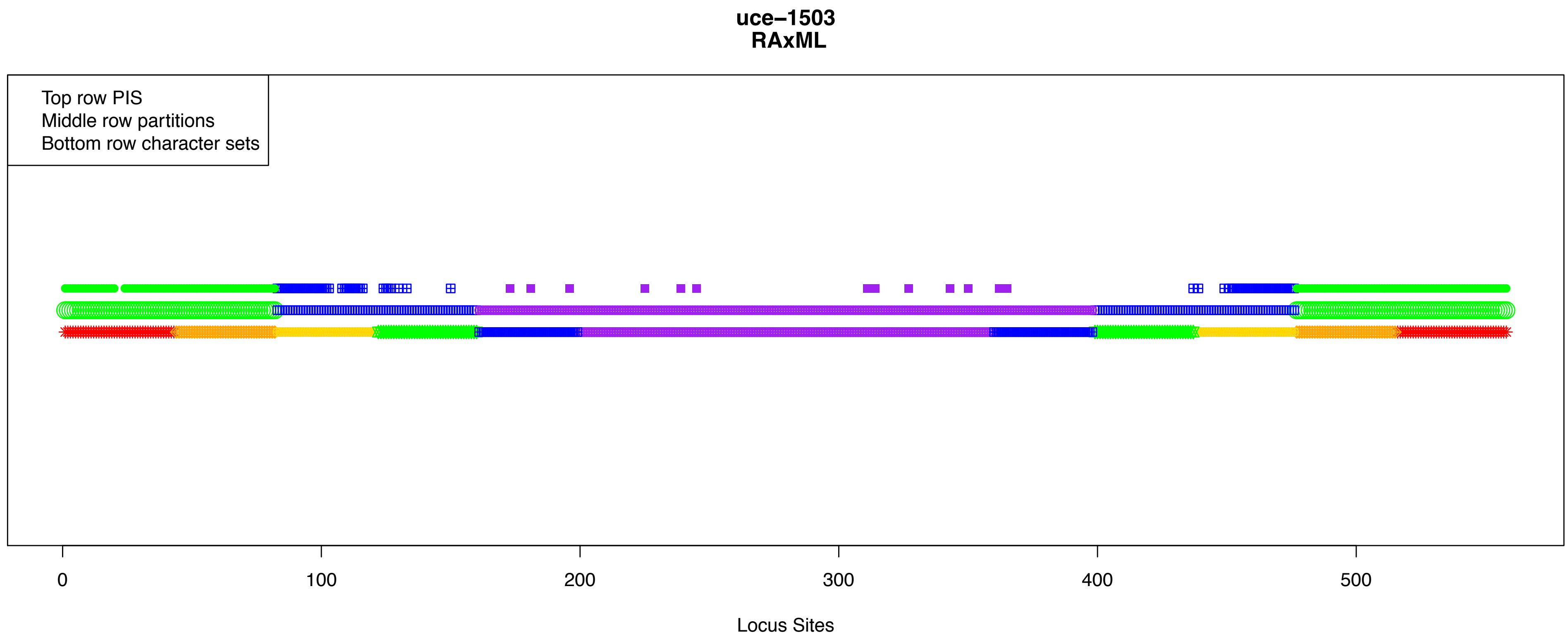
uce–1503
RAxML
Top row PIS
Middle row partitions
Bottom row character sets
0
100
200
300
400
500
Locus Sites

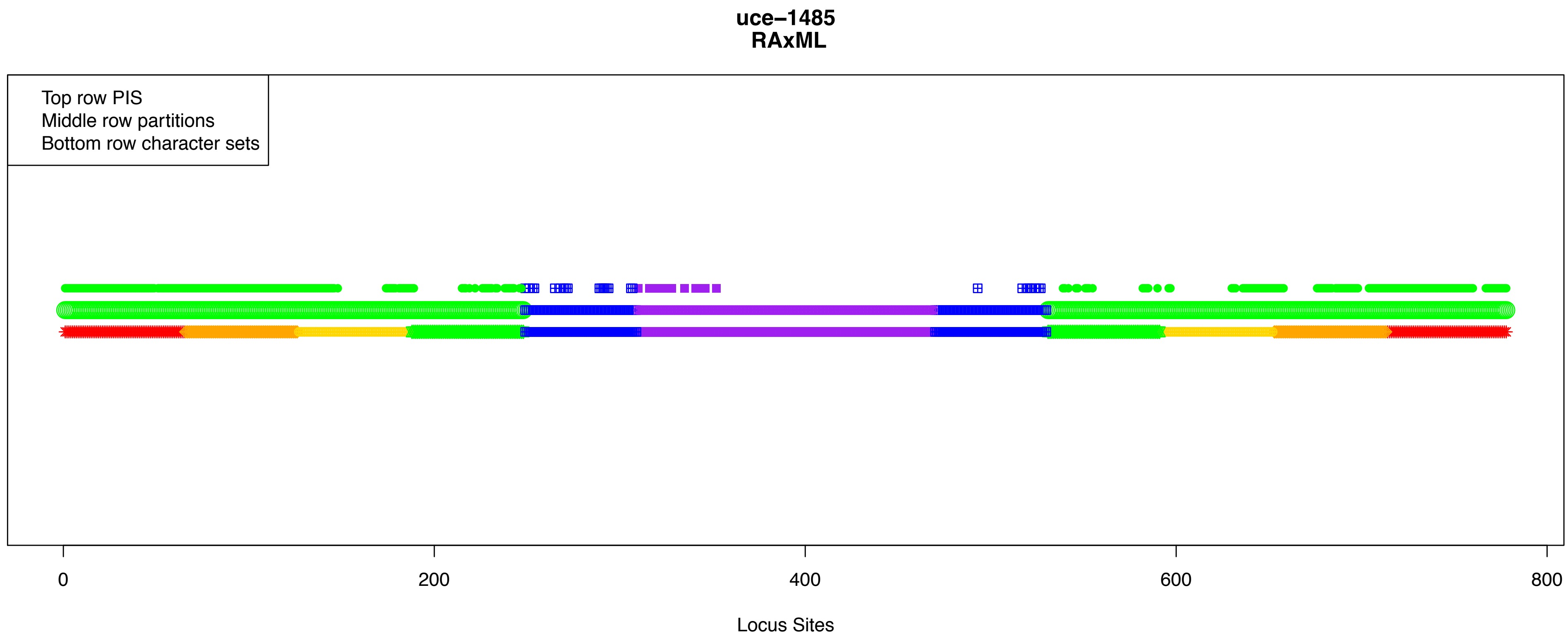
uce–1485
RAxML
Top row PIS
Middle row partitions
Bottom row character sets
0
200
400
600
800
Locus Sites

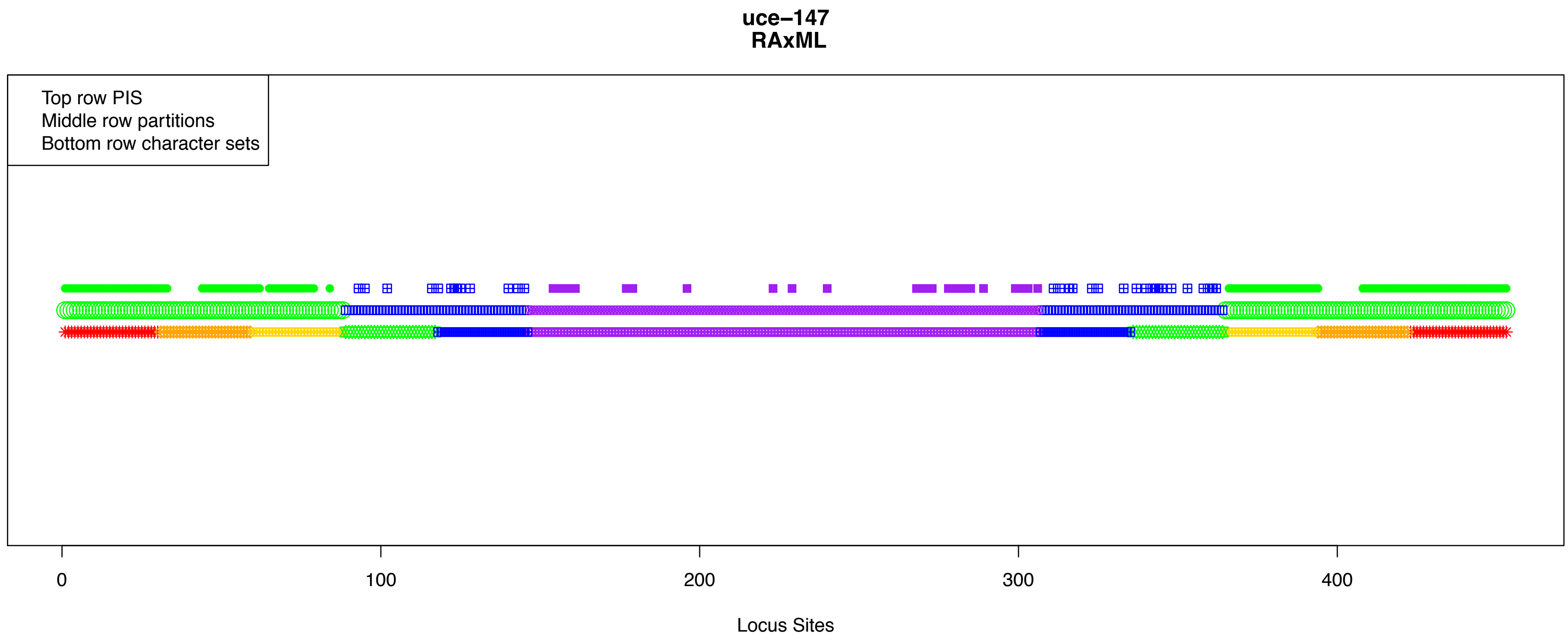

uce–147
RAxML
Top row PIS
Middle row partitions
Bottom row character sets
0
100
200
300
400
Locus Sites

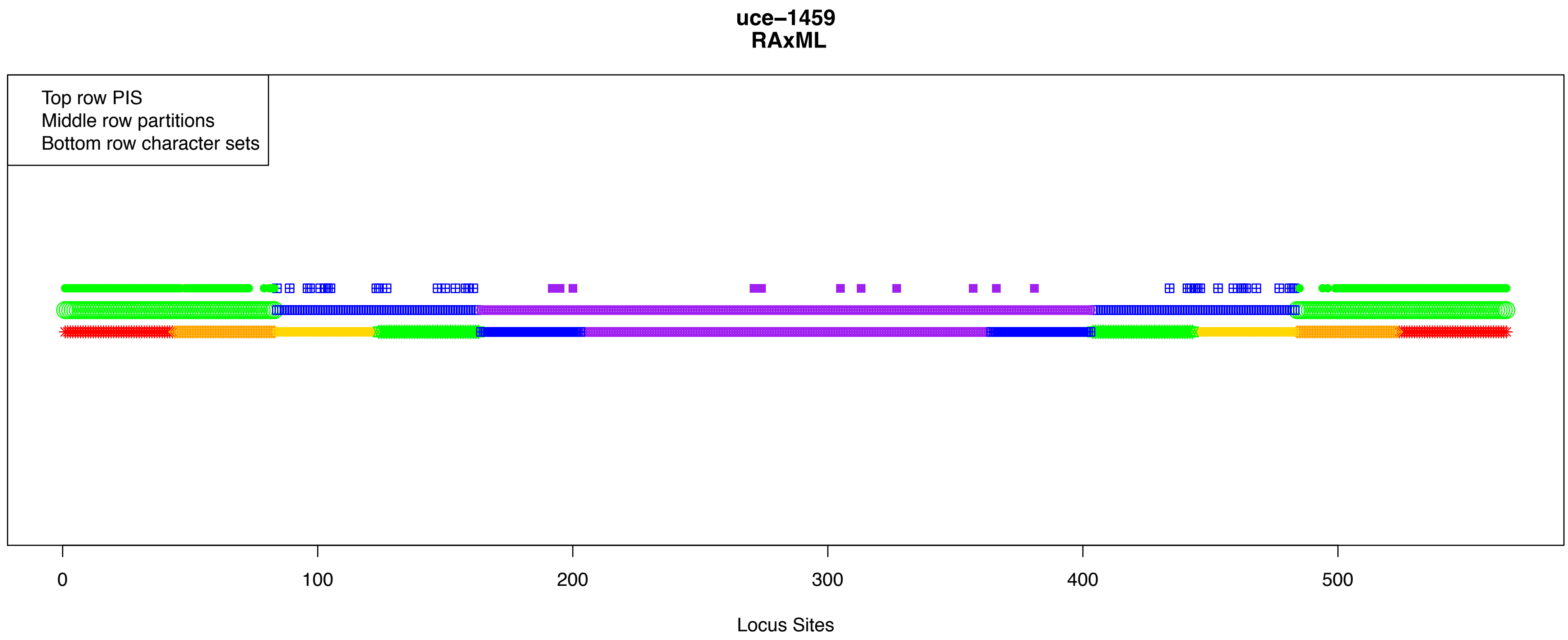

uce–1459
RAxML
Top row PIS
Middle row partitions
Bottom row character sets
0
100
200
300
400
500
Locus Sites

uce–1457
RAxML

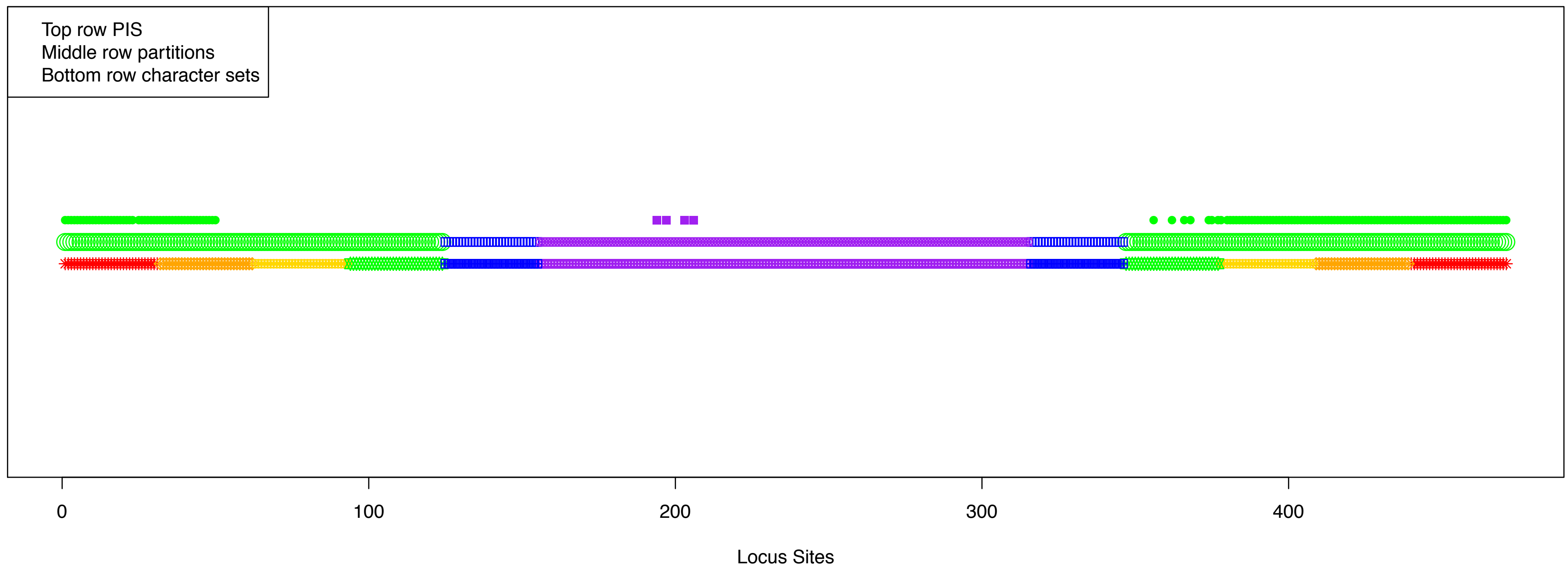

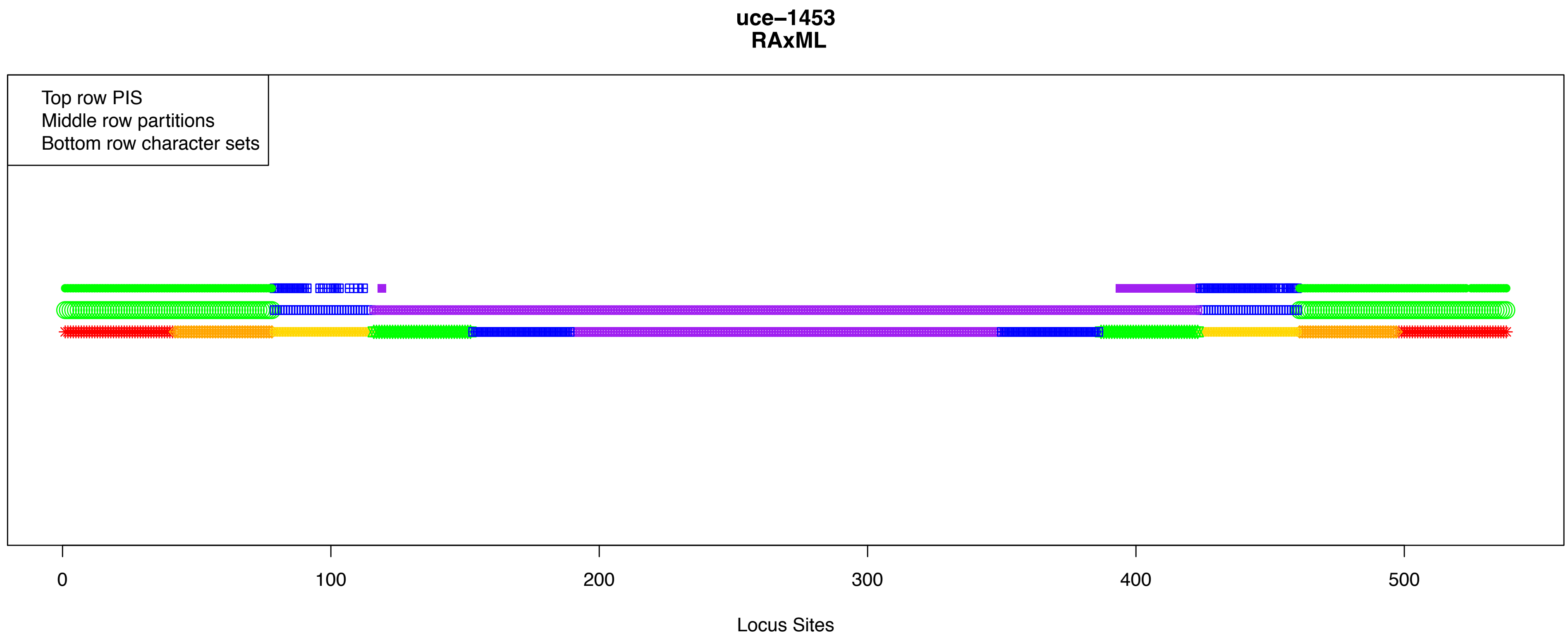

uce–1453
RAxML
Top row PIS
Middle row partitions
Bottom row character sets
0
100
200
300
400
500
Locus Sites

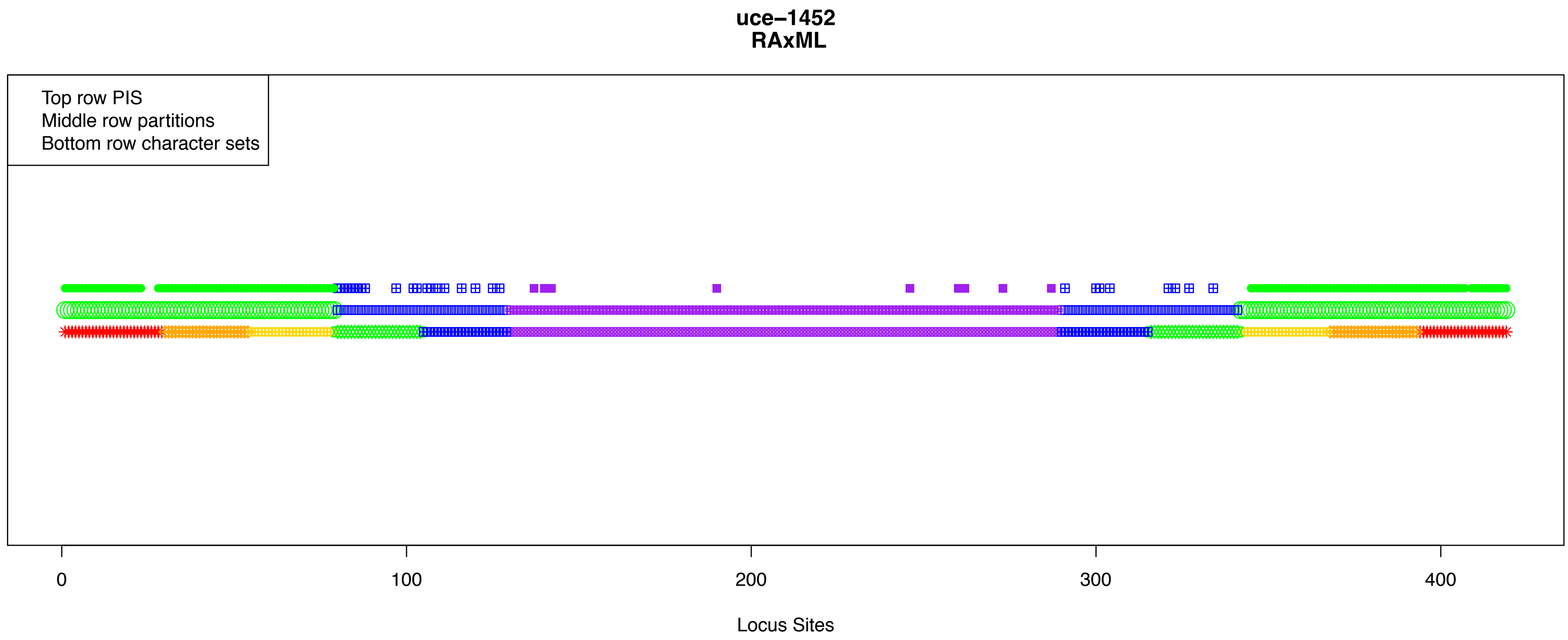

uce–1452
RAxML
Top row PIS
Middle row partitions
Bottom row character sets
0
100
200
300
400
Locus Sites

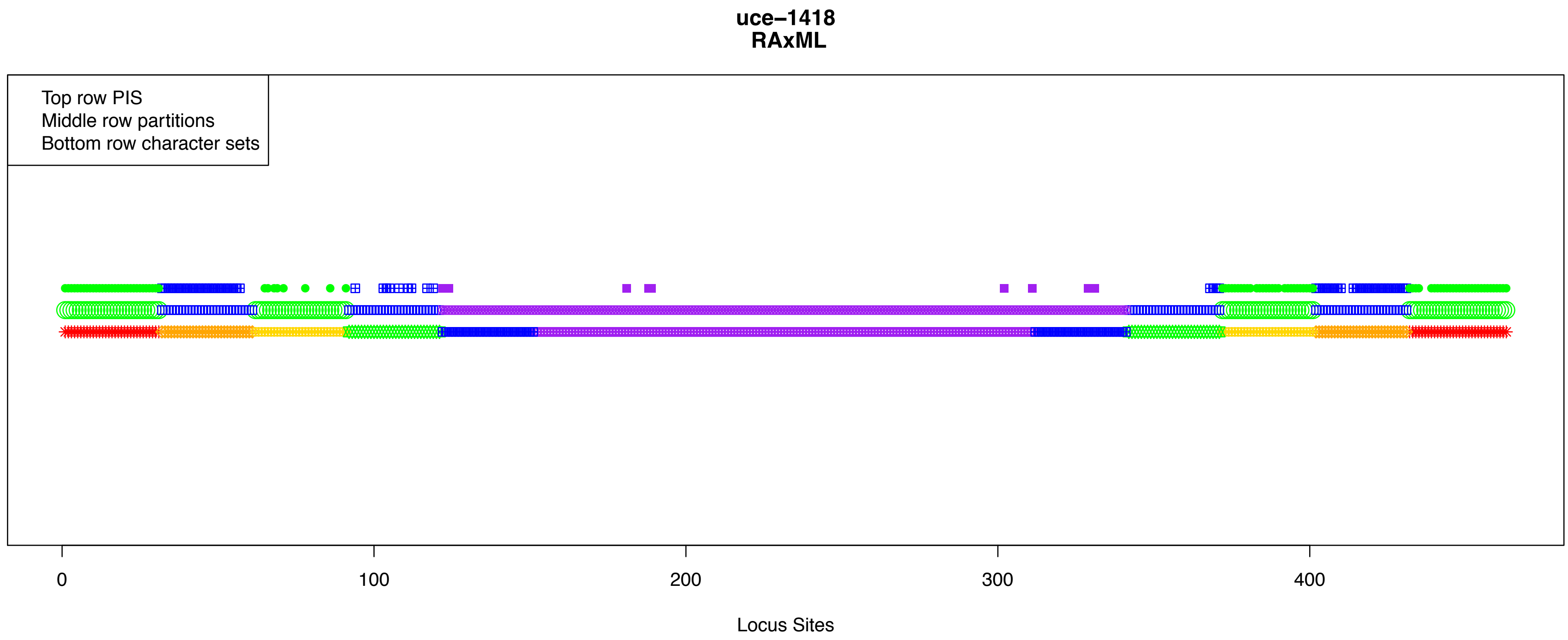
uce–1418
RAxML
Top row PIS
Middle row partitions
Bottom row character sets
0
100
200
300
400
Locus Sites

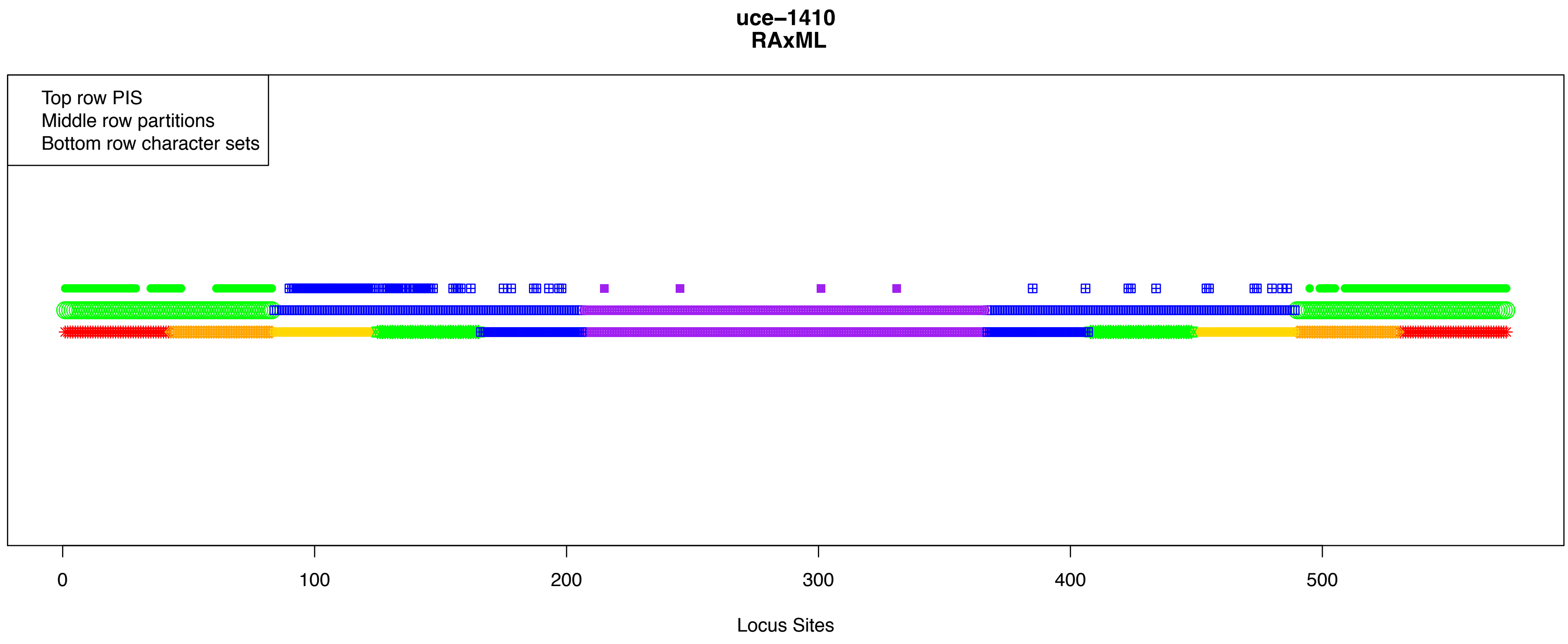

uce–1410
RAxML
Top row PIS
Middle row partitions
Bottom row character sets
0
100
200
300
400
500
Locus Sites

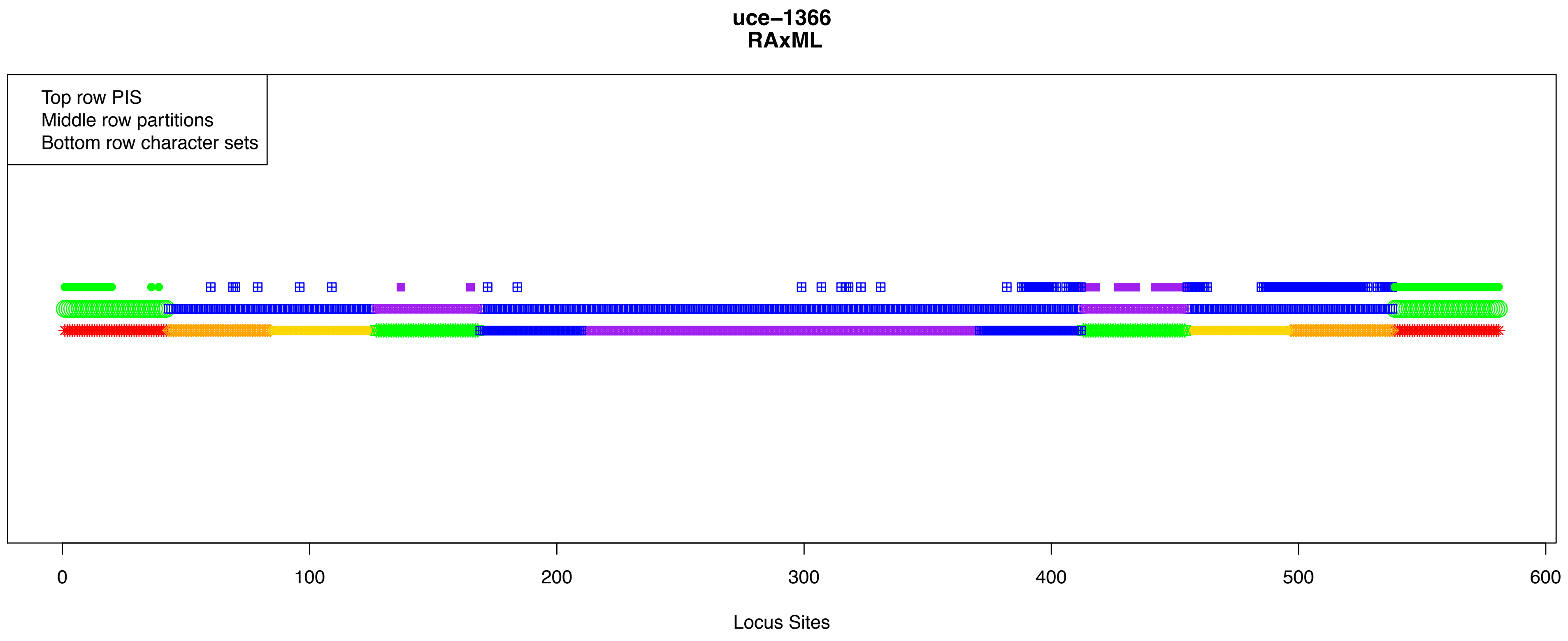

uce–1366
RAxML
Top row PIS
Middle row partitions
Bottom row character sets
0
100
200
300
400
500
600
Locus Sites

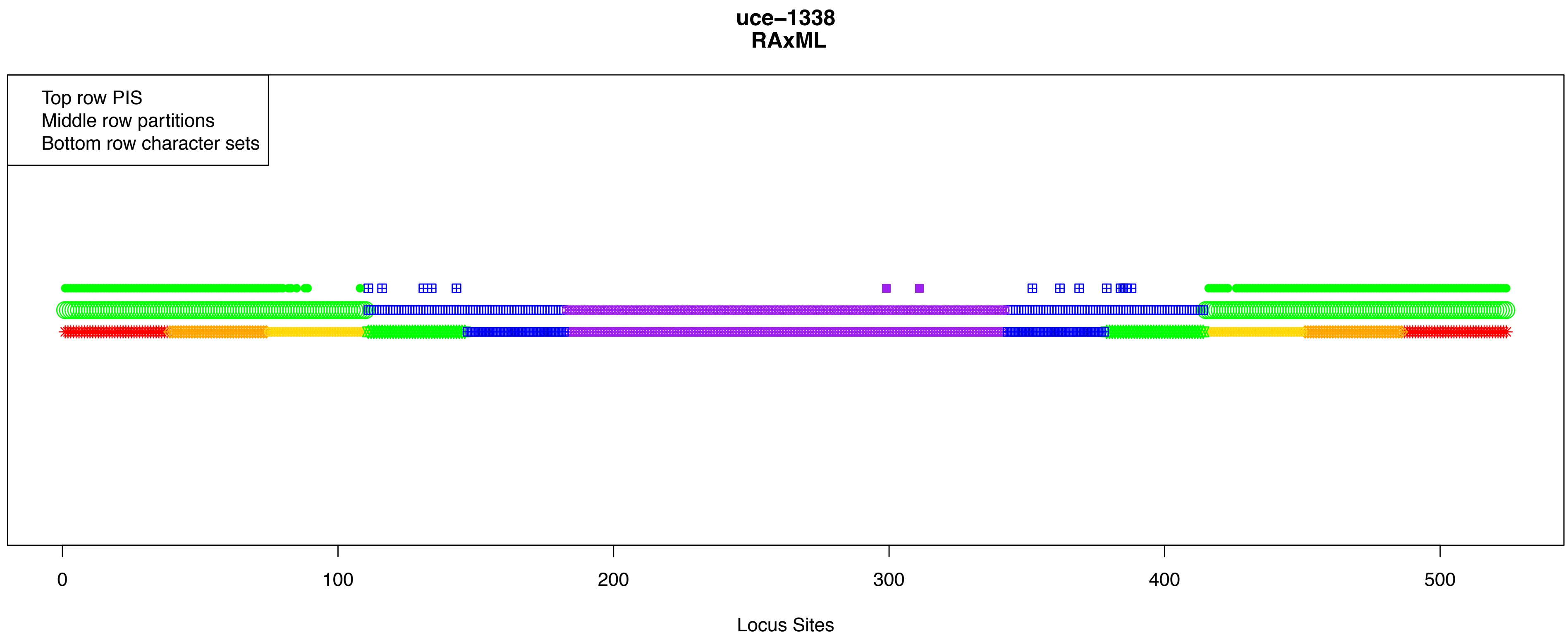
uce–1338
RAxML
Top row PIS
Middle row partitions
Bottom row character sets
0
100
200
300
400
500
Locus Sites

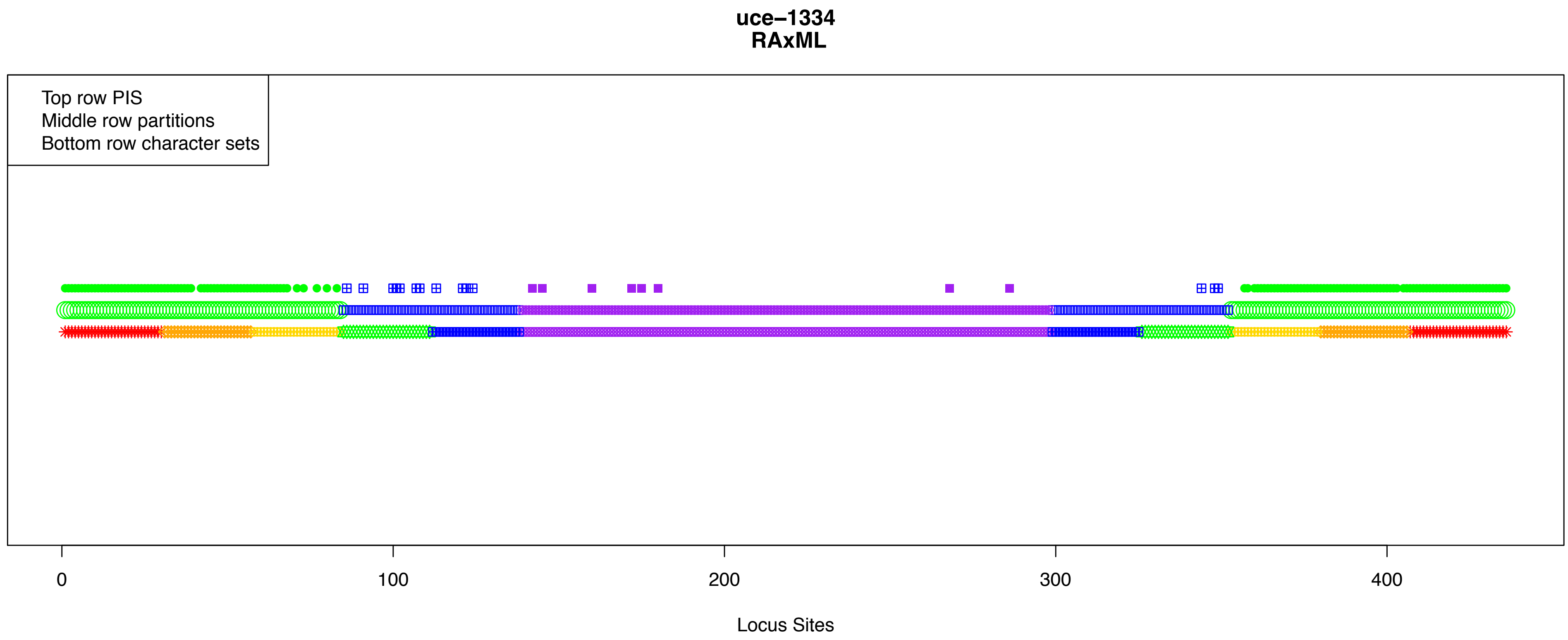
uce–1334
RAxML
Top row PIS
Middle row partitions
Bottom row character sets
0
100
200
300
400
Locus Sites

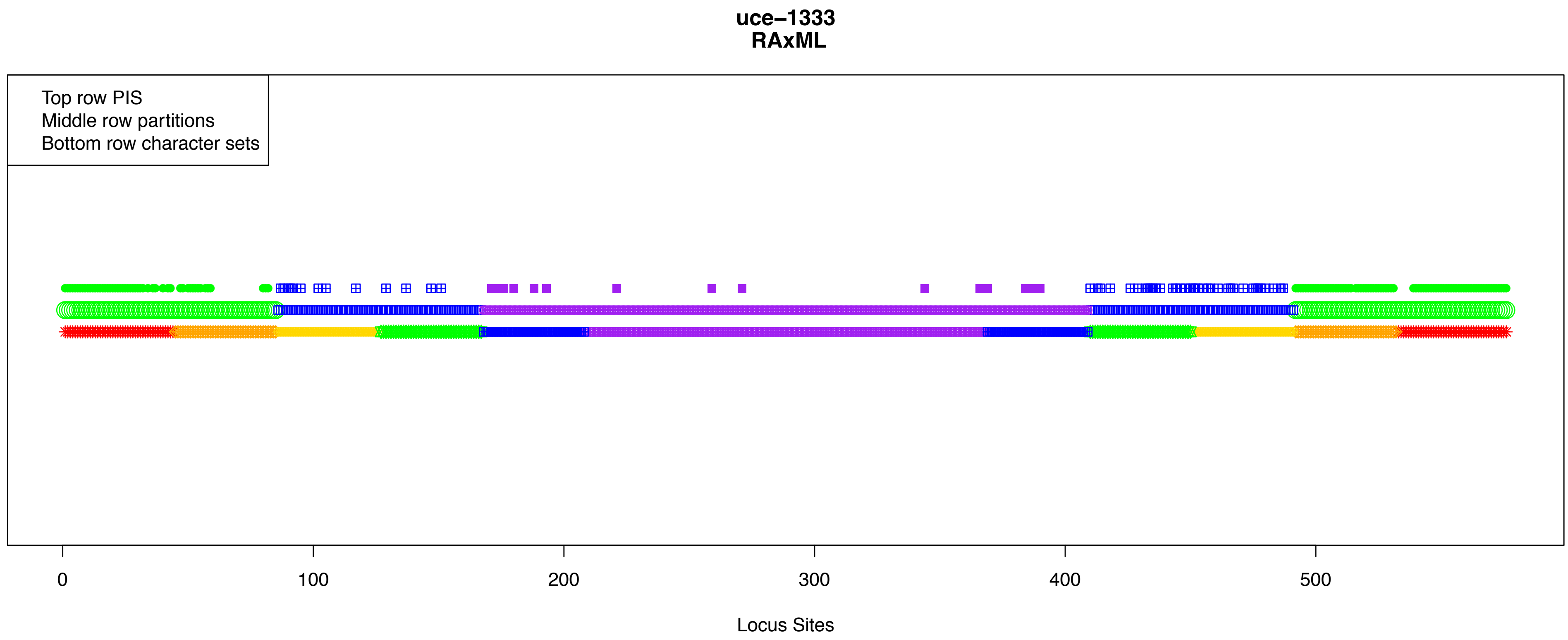

uce–1333
RAxML
Top row PIS
Middle row partitions
Bottom row character sets
0
100
200
300
400
500
Locus Sites

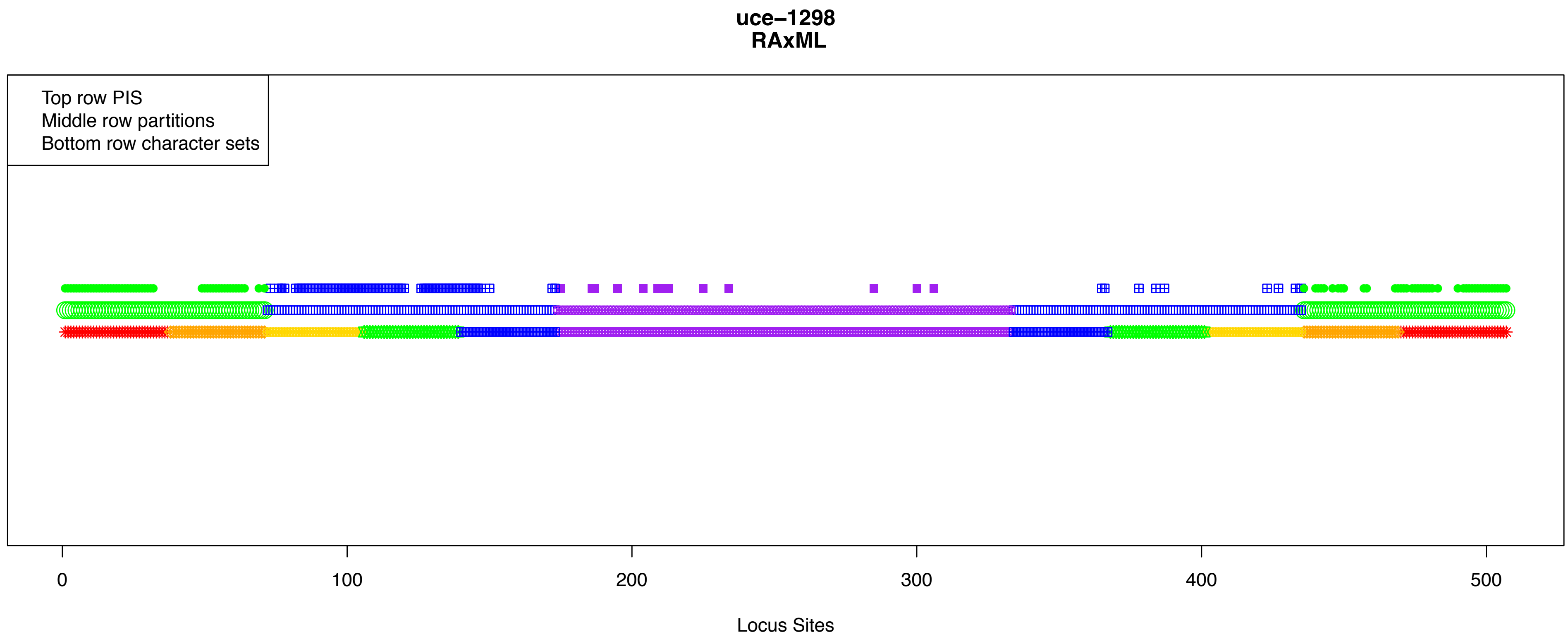

uce–1298
RAxML
Top row PIS
Middle row partitions
Bottom row character sets
0
100
200
300
400
500
Locus Sites

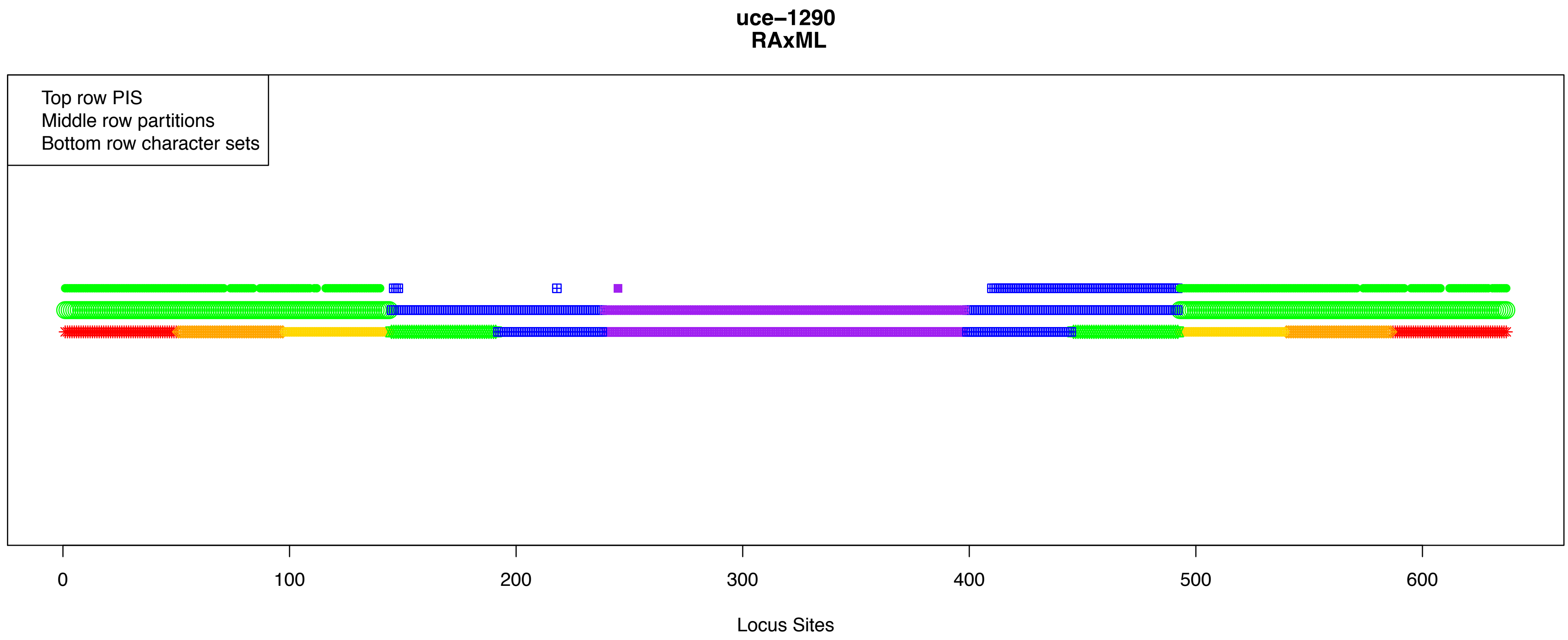

uce–1290
RAxML
Top row PIS
Middle row partitions
Bottom row character sets
0
100
200
300
400
500
600
Locus Sites

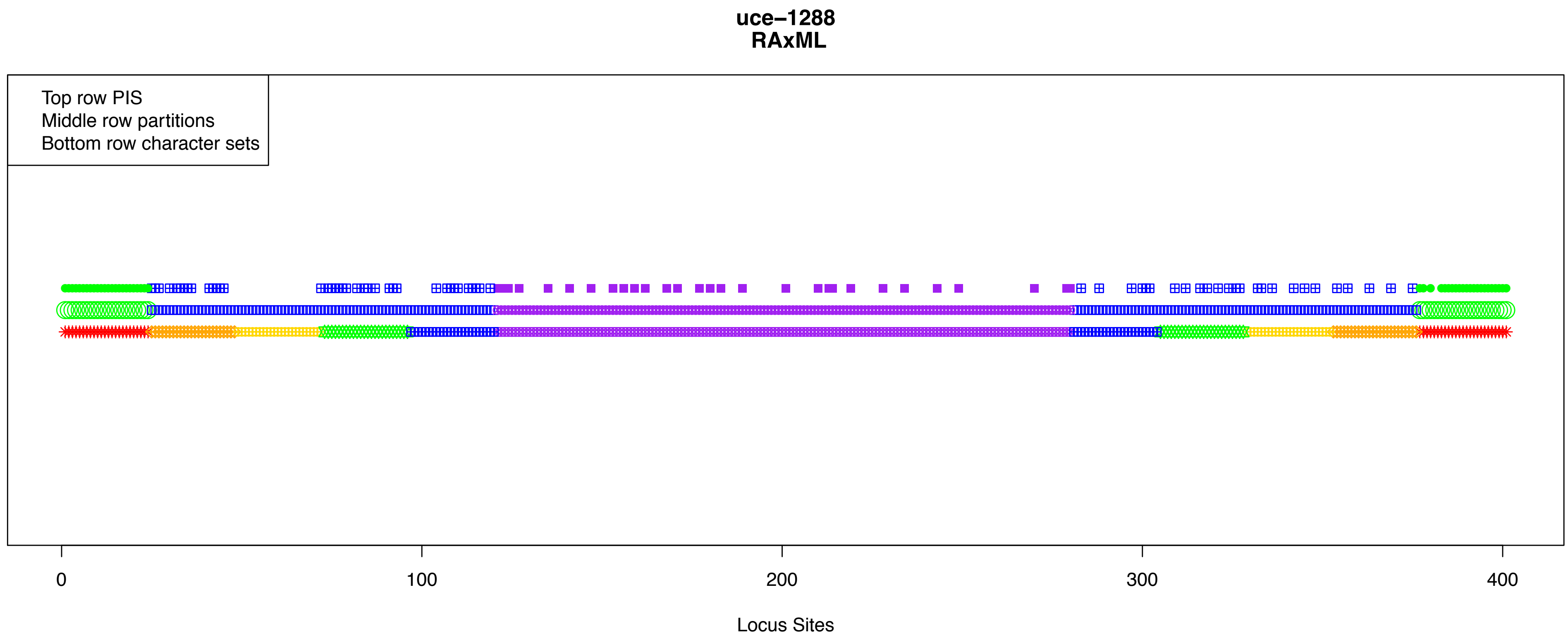
uce–1288
RAxML
Top row PIS
Middle row partitions
Bottom row character sets
0
100
200
300
400
Locus Sites

uce–1281
RAxML

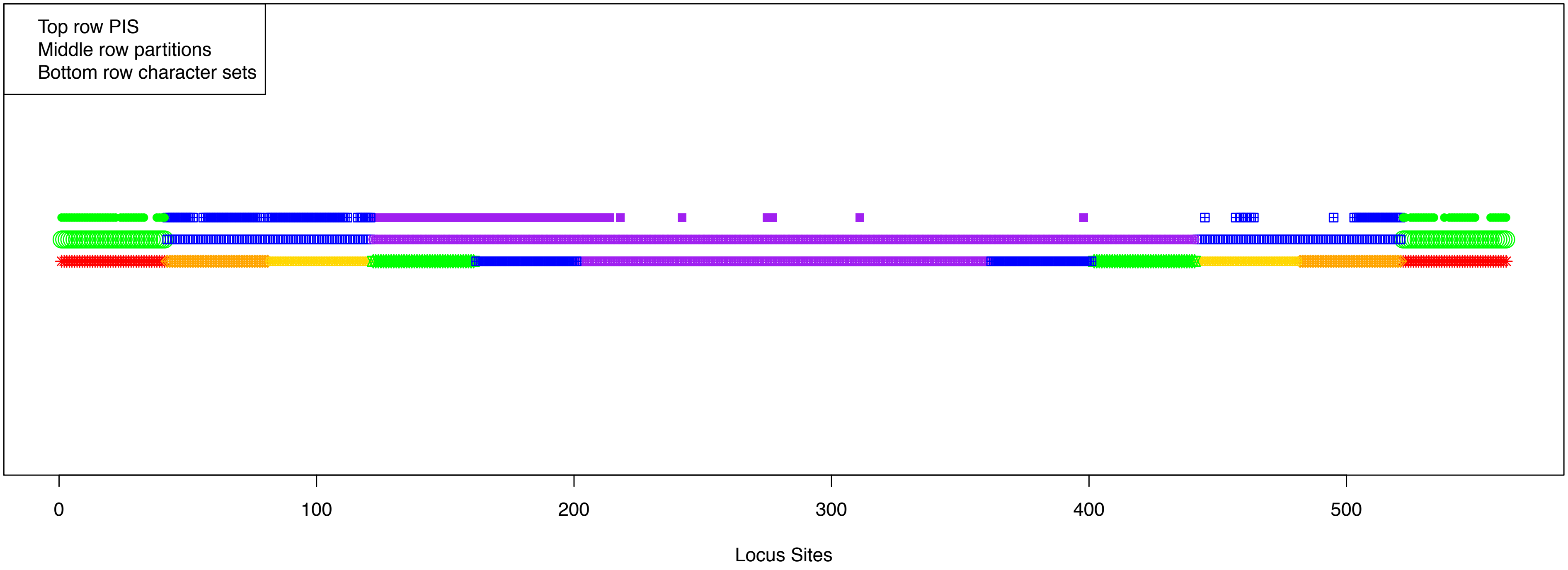

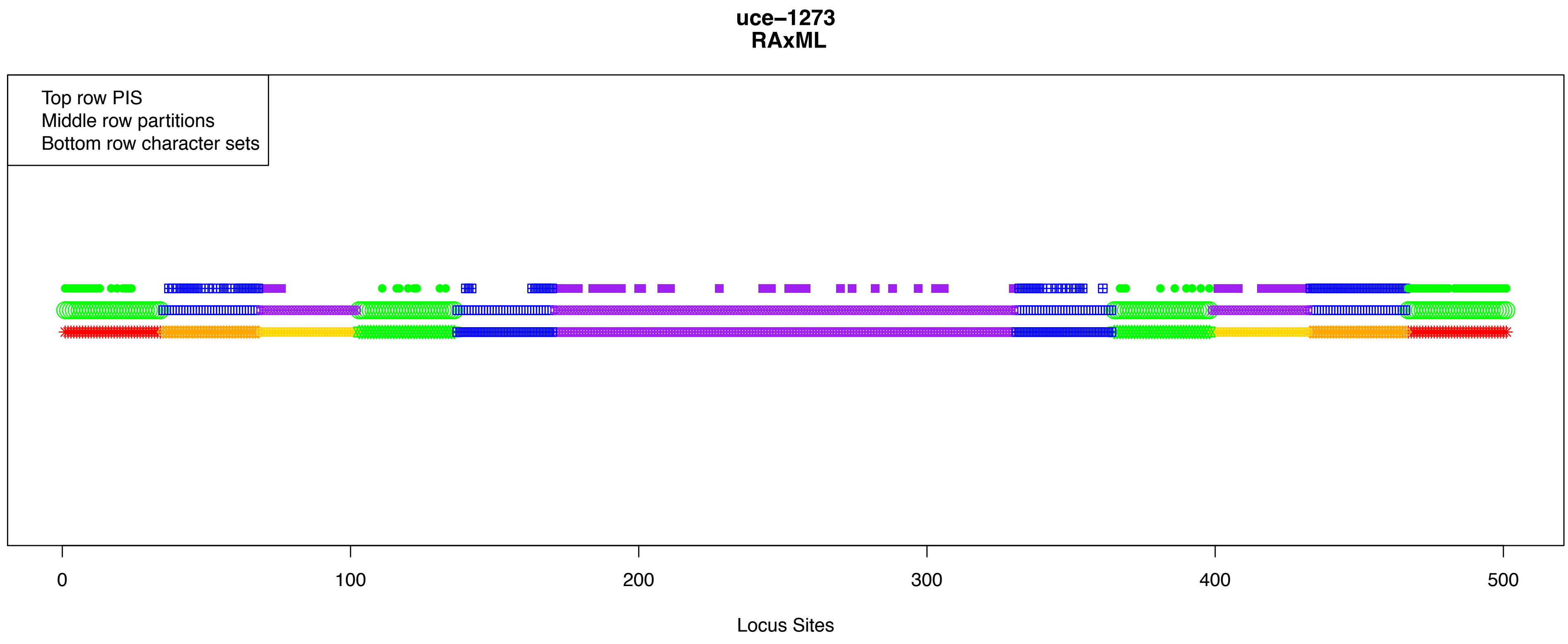

uce–1273
RAxML
Top row PIS
Middle row partitions
Bottom row character sets
0
100
200
300
400
500
Locus Sites

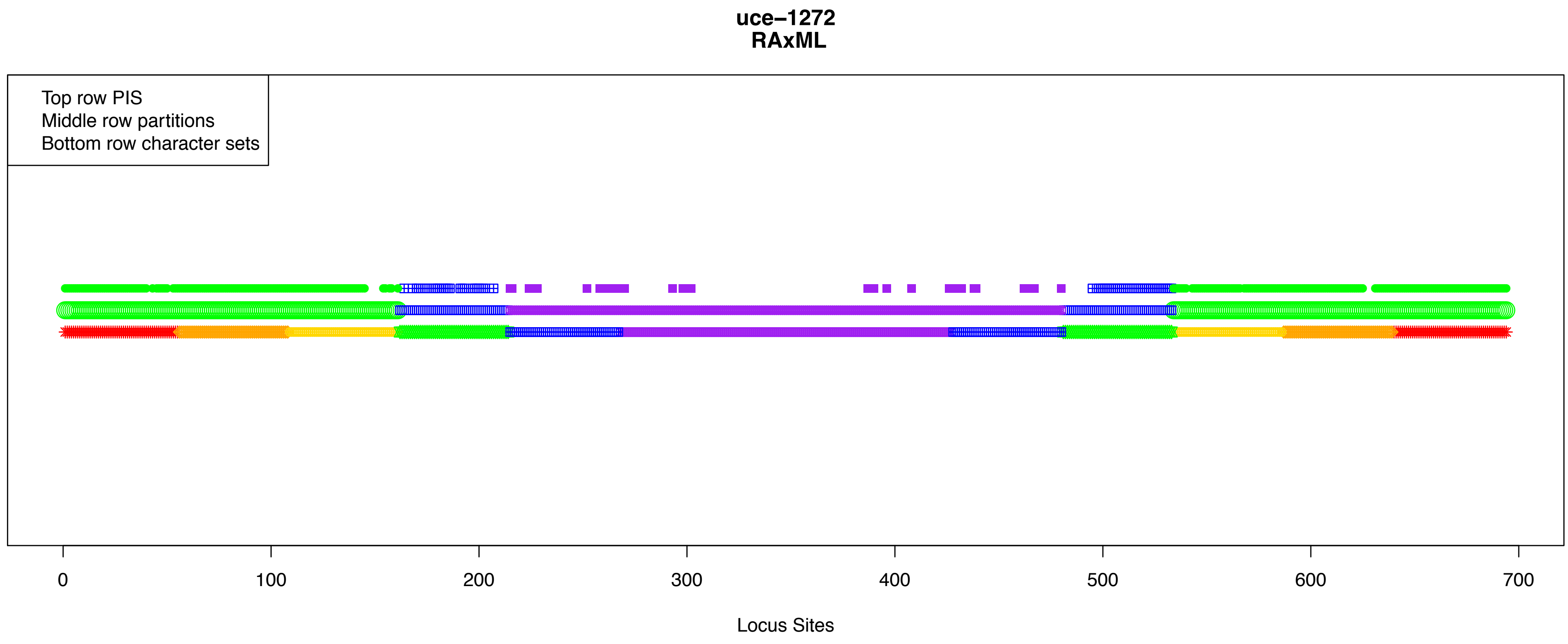
uce–1272
RAxML
Top row PIS
Middle row partitions
Bottom row character sets
0
100
200
300
400
500
600
700
Locus Sites

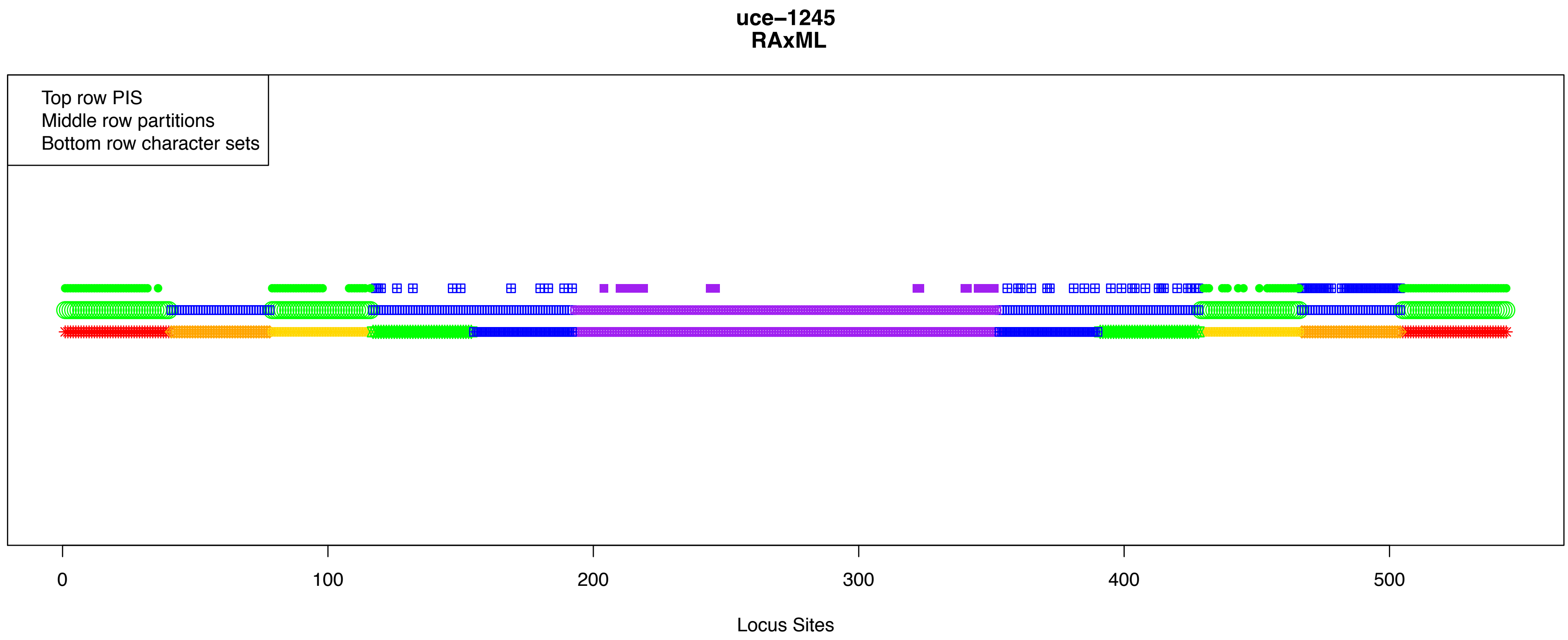

uce–1245
RAxML
Top row PIS
Middle row partitions
Bottom row character sets
0
100
200
300
400
500
Locus Sites

uce–1240
RAxML

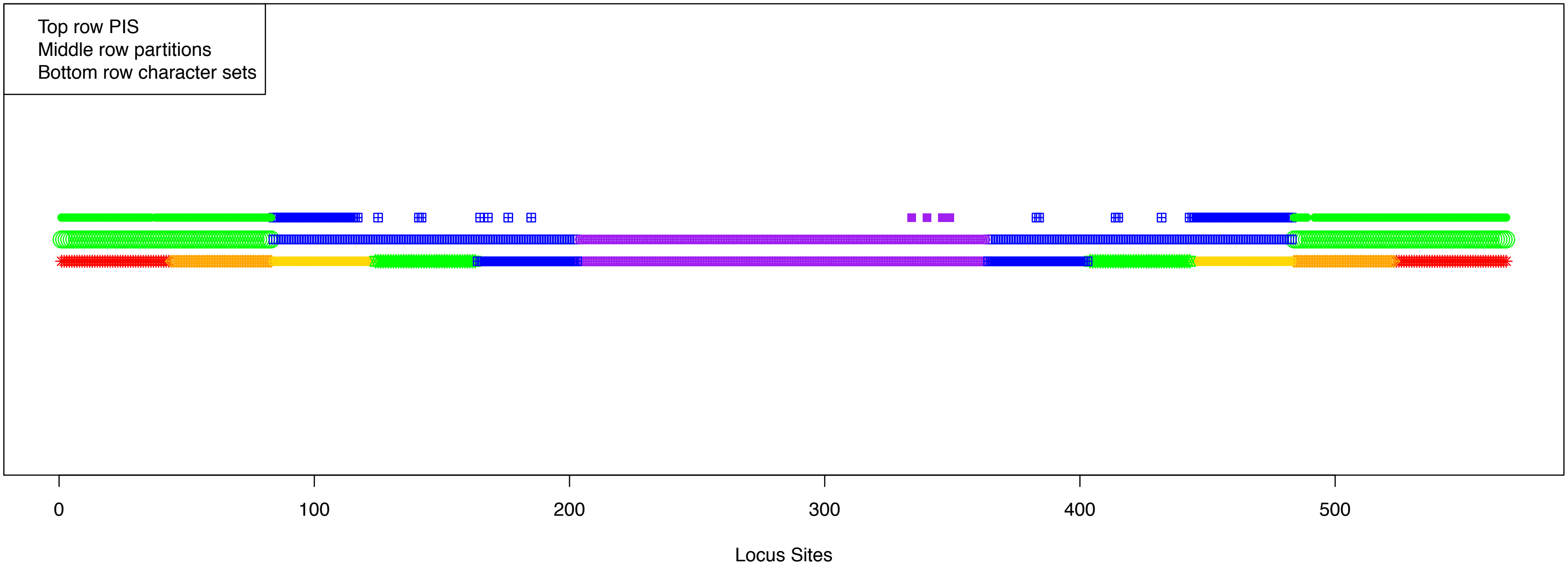

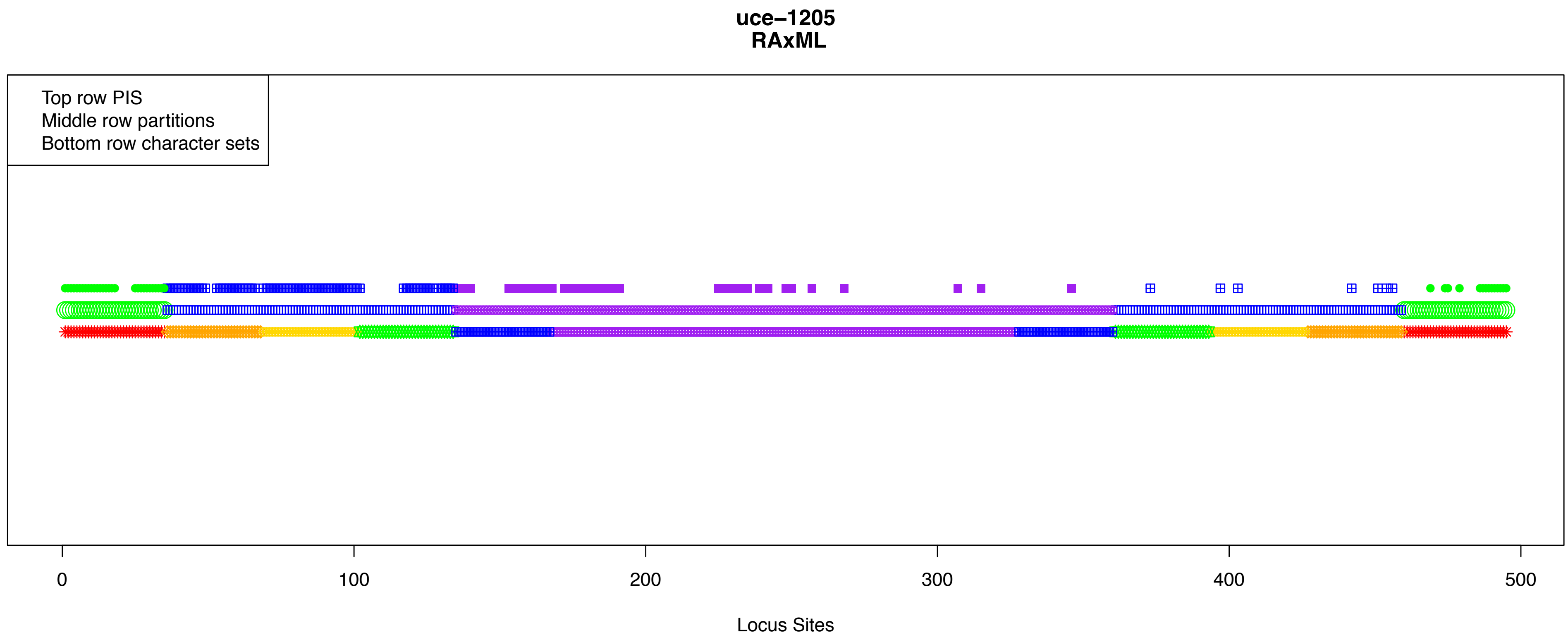
uce–1205
RAxML
Top row PIS
Middle row partitions
Bottom row character sets
0
100
200
300
400
500
Locus Sites

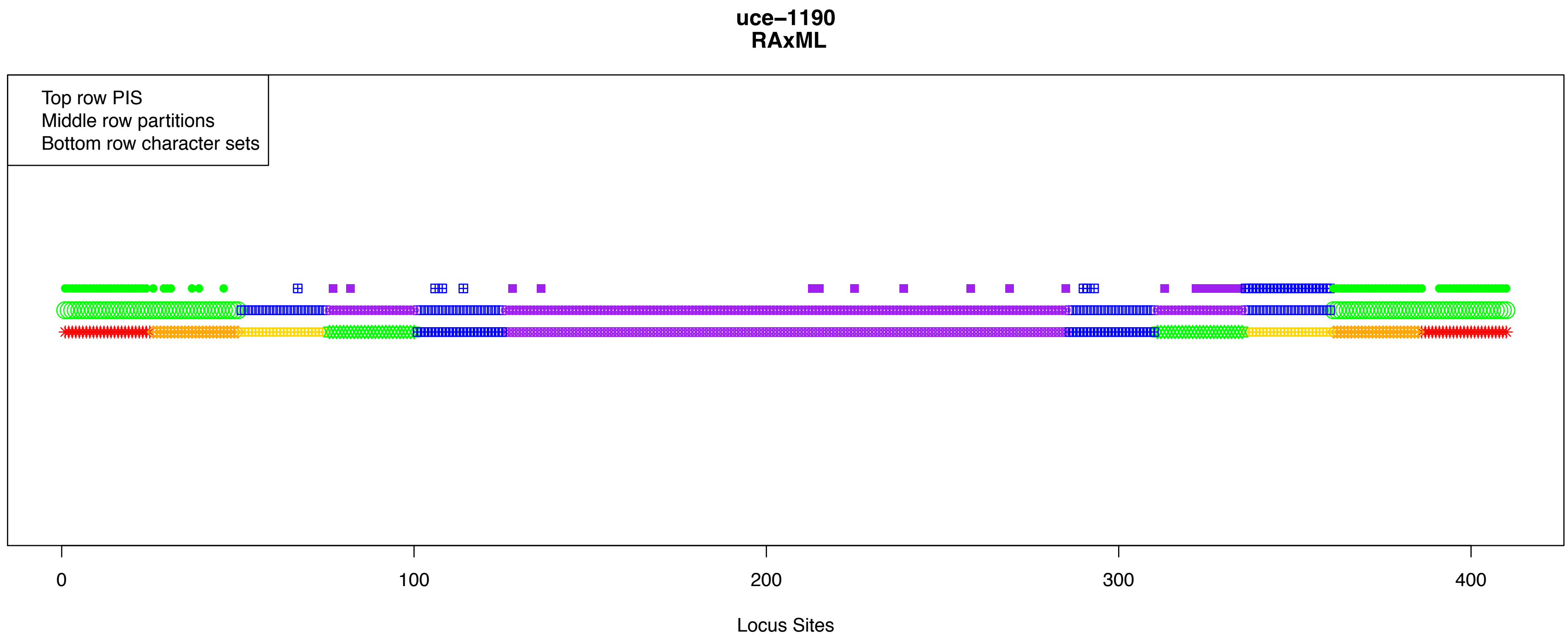
uce–1190
RAxML
Top row PIS
Middle row partitions
Bottom row character sets
0
100
200
300
400
Locus Sites

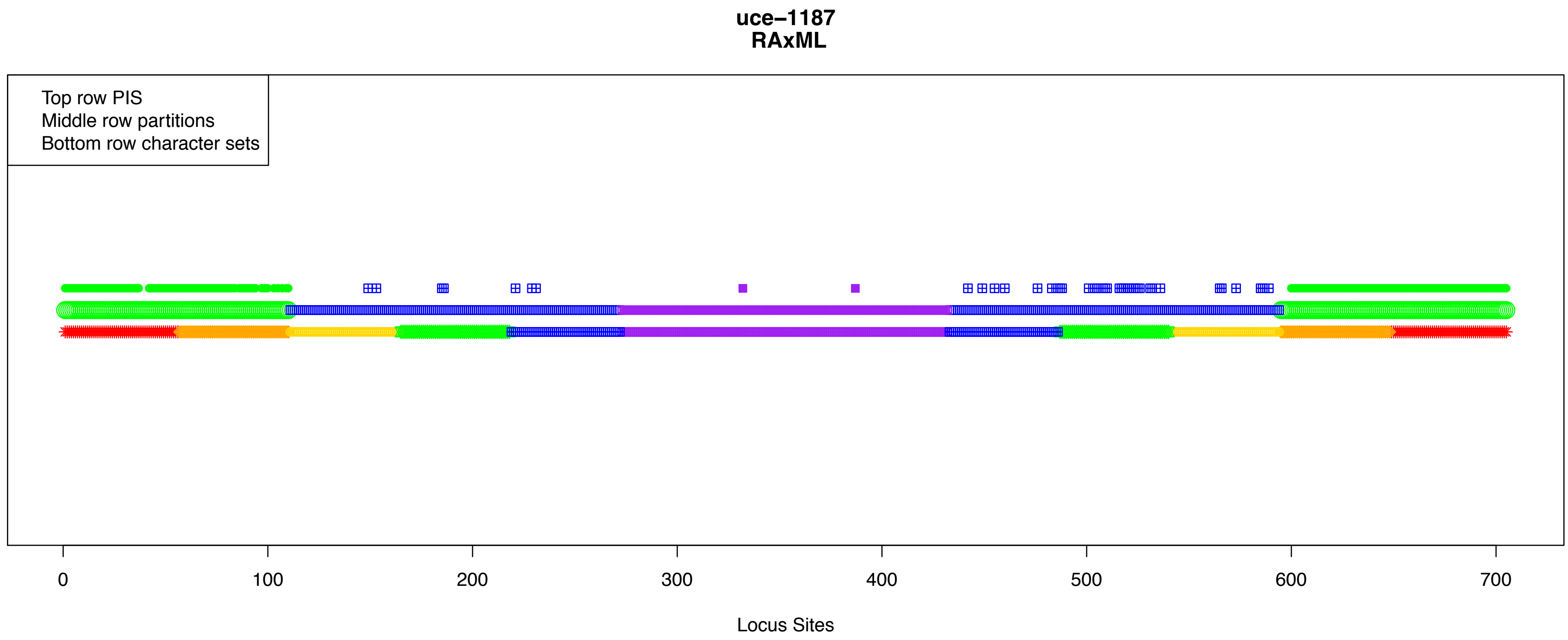
uce–1187
RAxML
Top row PIS
Middle row partitions
Bottom row character sets
0
100
200
300
400
500
600
700
Locus Sites

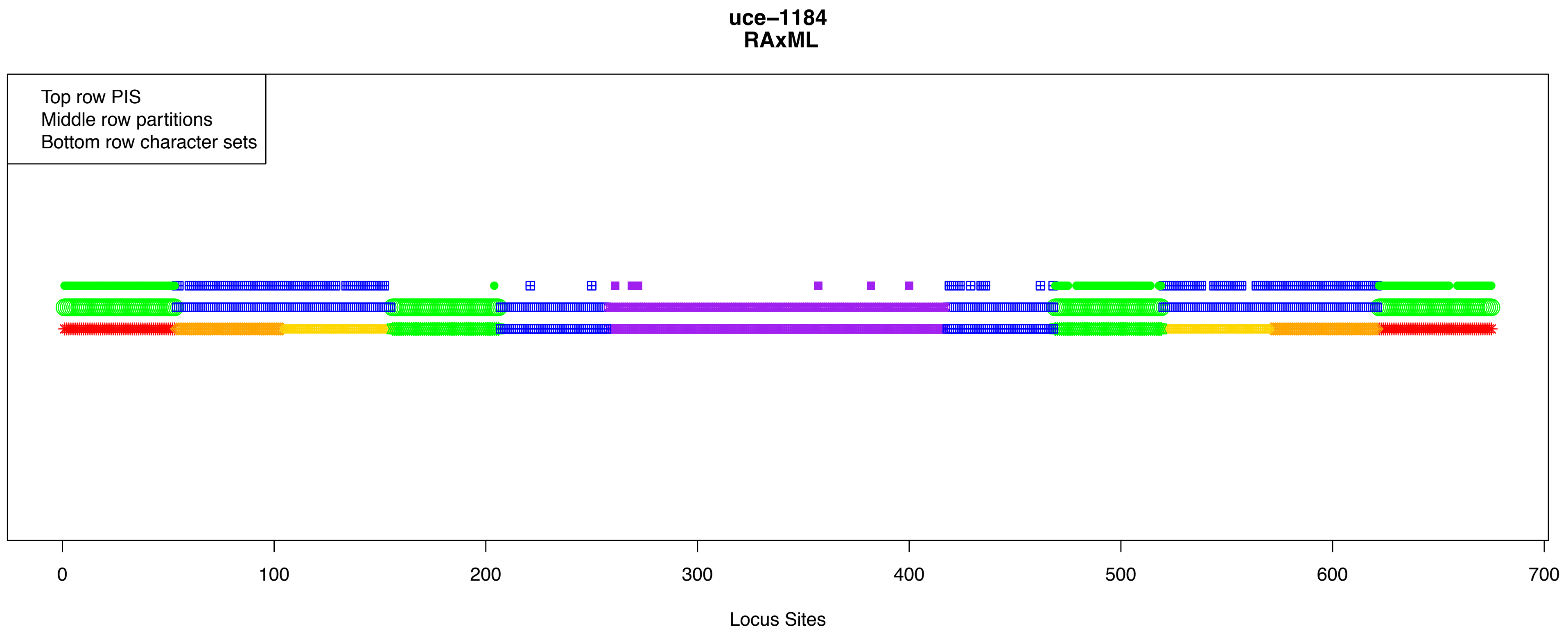

uce–1184
RAxML
Top row PIS
Middle row partitions
Bottom row character sets
0
100
200
300
400
500
600
700
Locus Sites

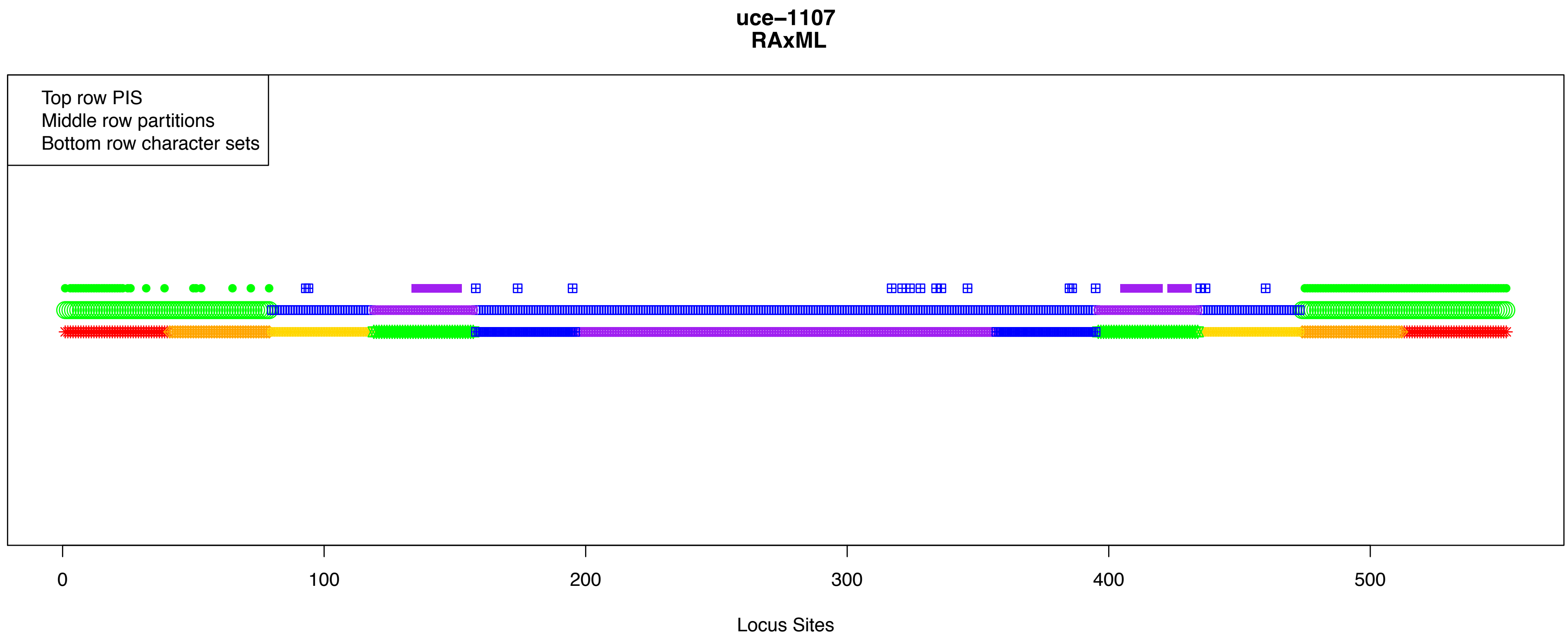
uce–1107
RAxML
Top row PIS
Middle row partitions
Bottom row character sets
0
100
200
300
400
500
Locus Sites

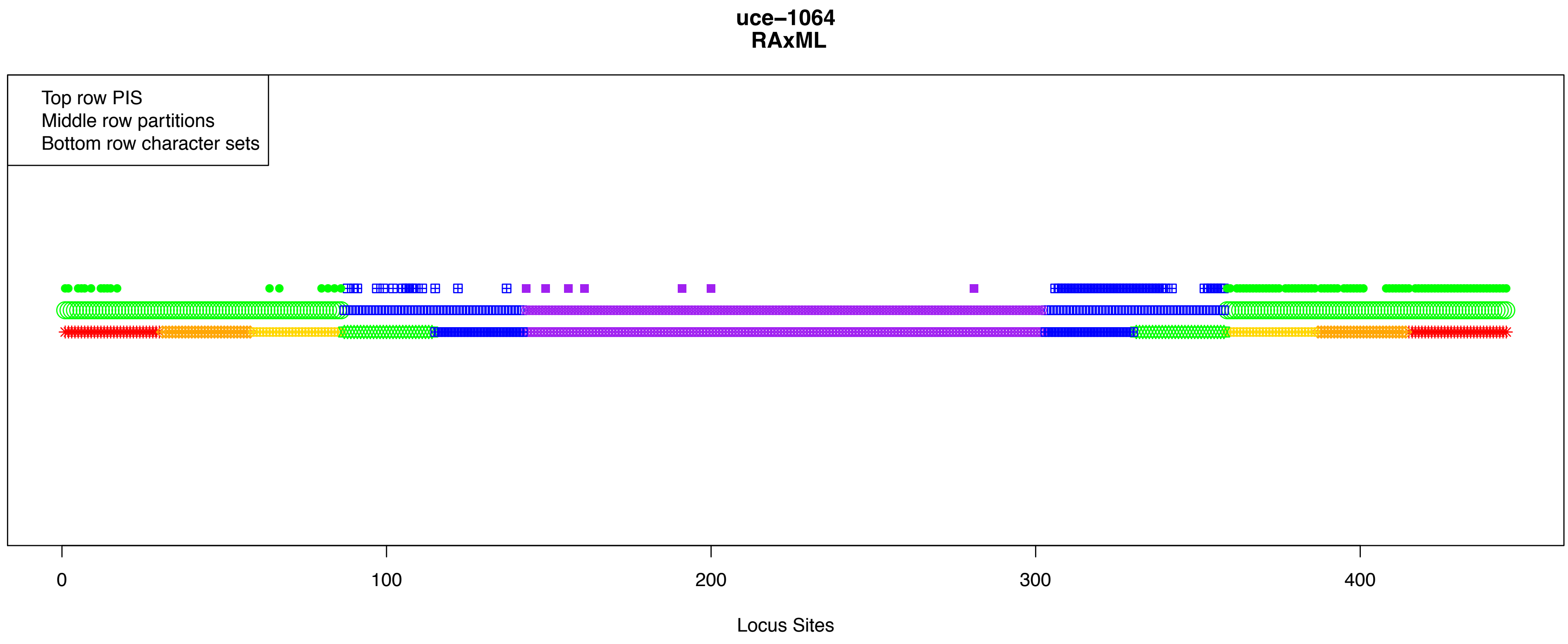
uce–1064
RAxML
Top row PIS
Middle row partitions
Bottom row character sets
0
100
200
300
400
Locus Sites

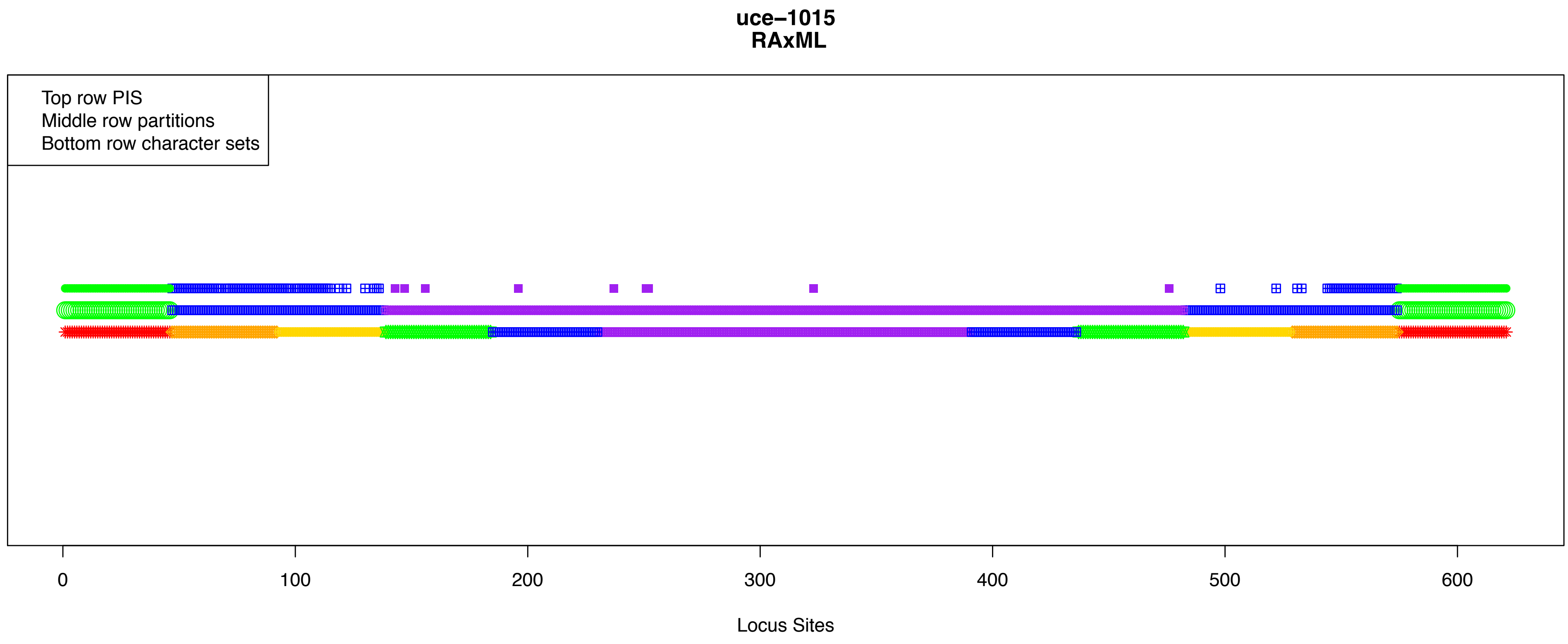
uce–1015
RAxML
Top row PIS
Middle row partitions
Bottom row character sets
0
100
200
300
400
500
600
Locus Sites

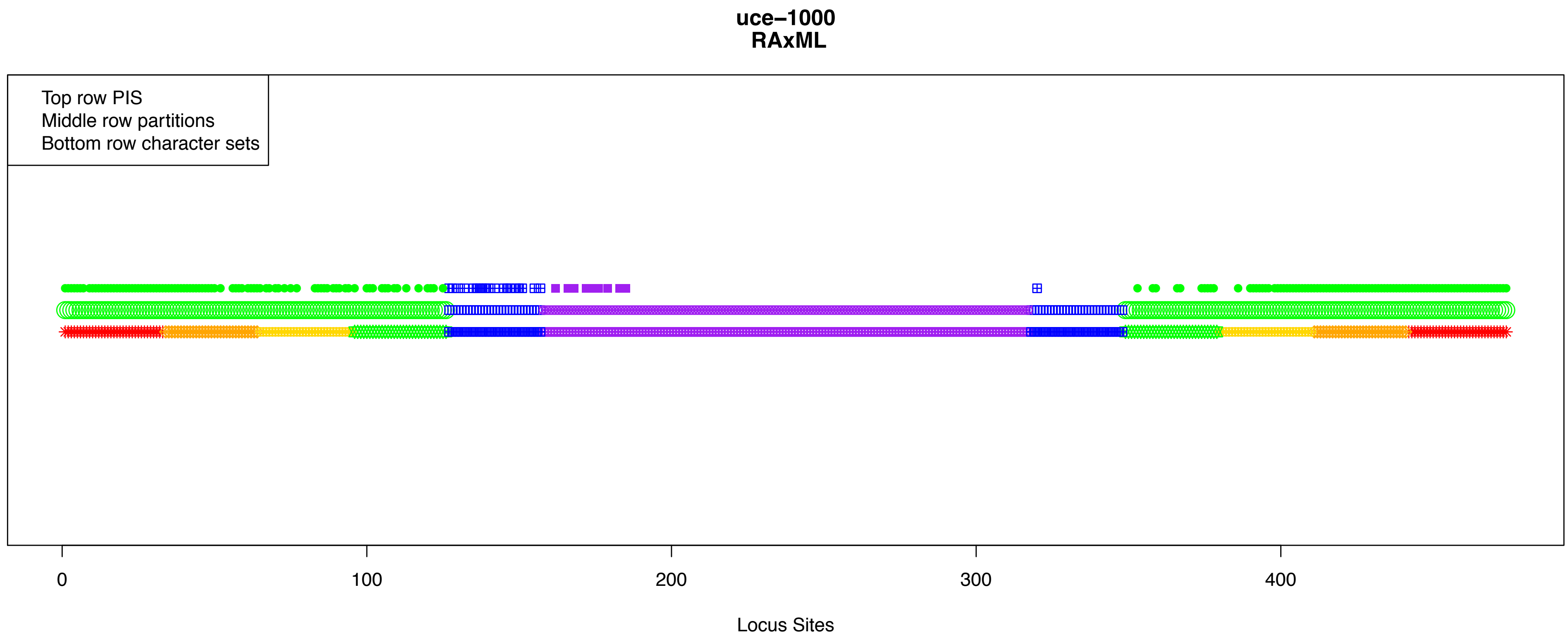
uce–1000
RAxML
Top row PIS
Middle row partitions
Bottom row character sets
0
100
200
300
400
Locus Sites
