# Supplementary material for: Ultraconserved elements (UCEs) resolve the phylogeny of Australasian smurf-weevils
Source: PLoS One. 2017 Nov 22;12(11):e0188044. doi: 10.1371/journal.pone.0188044 (PMC5699822; doi:10.1371/journal.pone.0188044)
Supplement: S1 File — (ZIP) [file pone.0188044.s007.zip › Supplemental_Partition_Number_of_partitions_PIS_Charsets/partitions4-MrBayes.pdf]

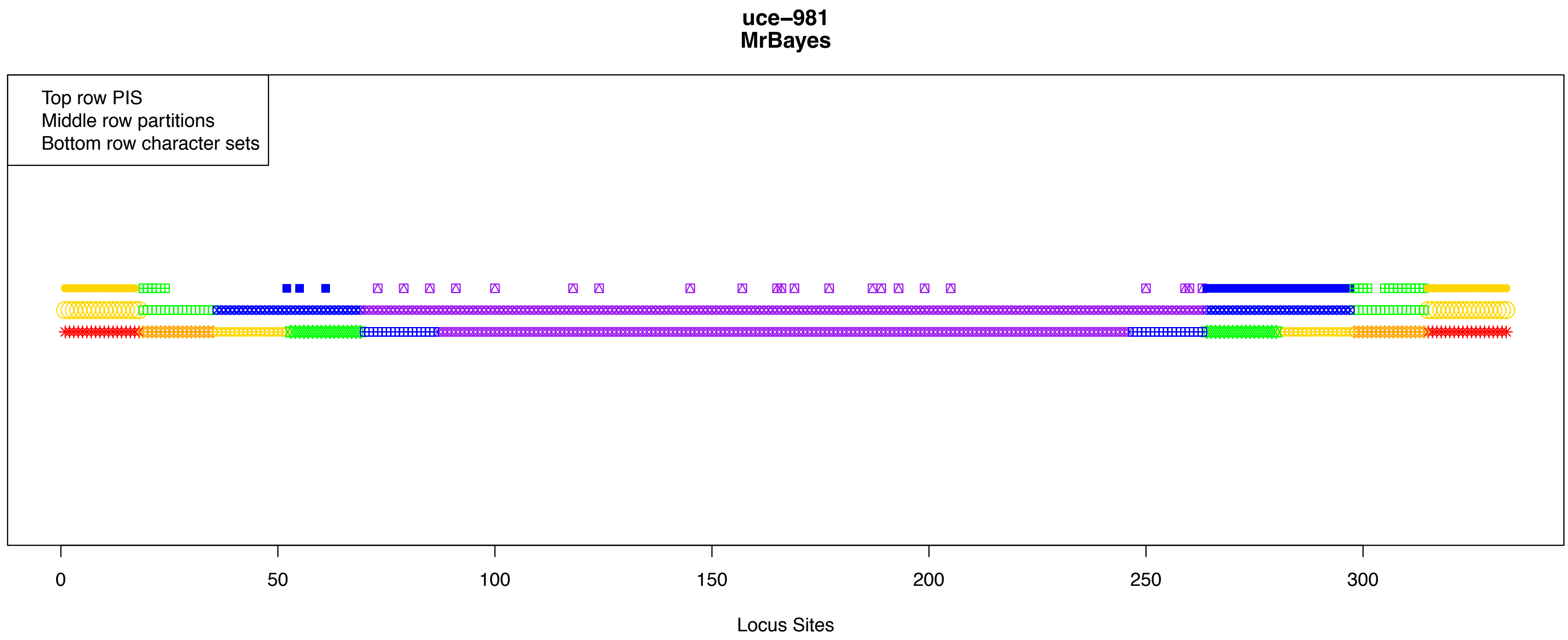

uce–981
MrBayes
Top row PIS
Middle row partitions
Bottom row character sets
0
50
100
150
200
250
300
Locus Sites

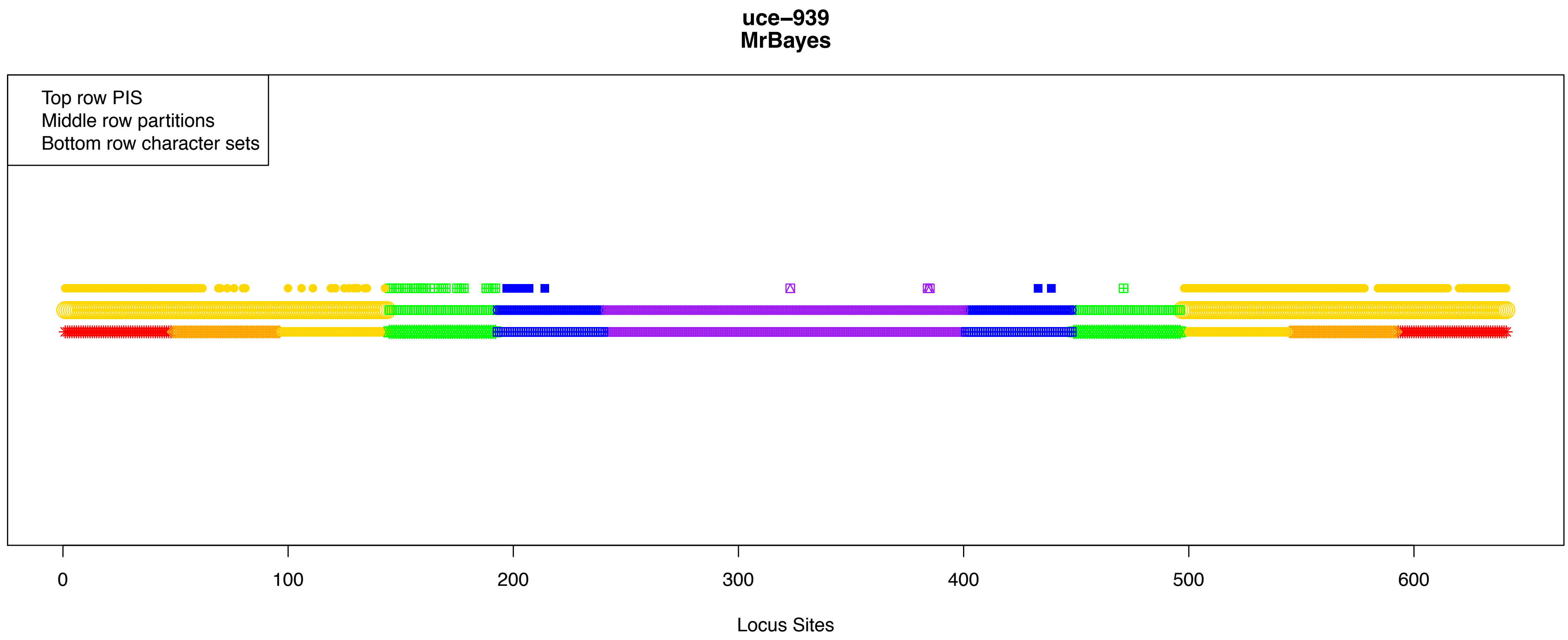

uce–939
MrBayes
Top row PIS
Middle row partitions
Bottom row character sets
0
100
200
300
400
500
600
Locus Sites

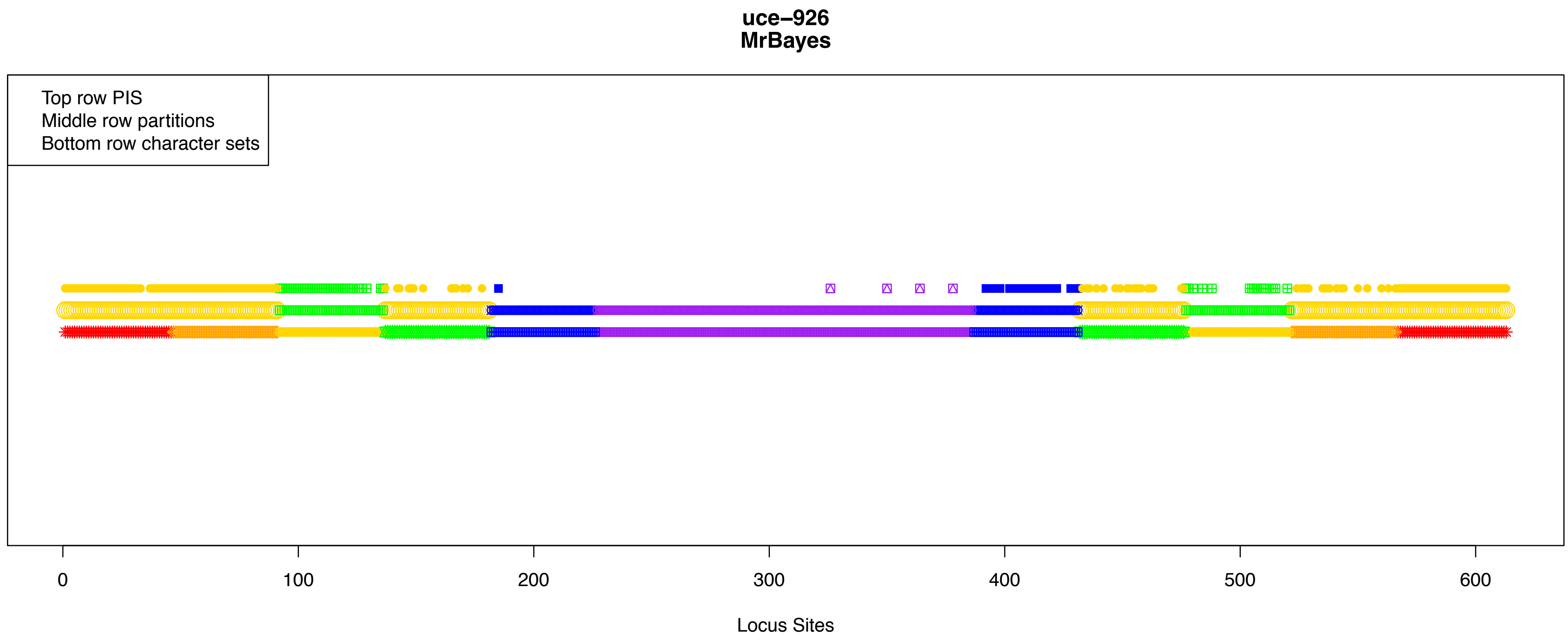
uce–926
MrBayes
Top row PIS
Middle row partitions
Bottom row character sets
0
100
200
300
400
500
600
Locus Sites

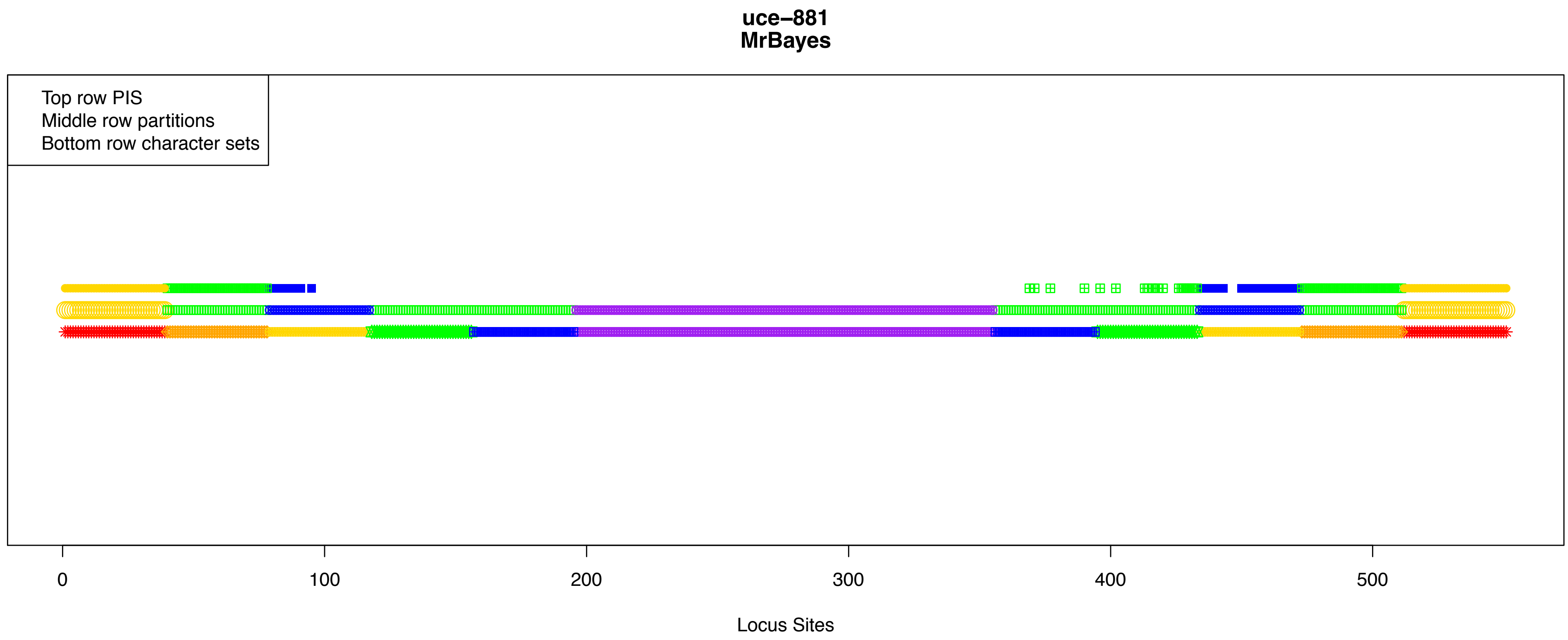
uce–881
MrBayes
Top row PIS
Middle row partitions
Bottom row character sets
0
100
200
300
400
500
Locus Sites

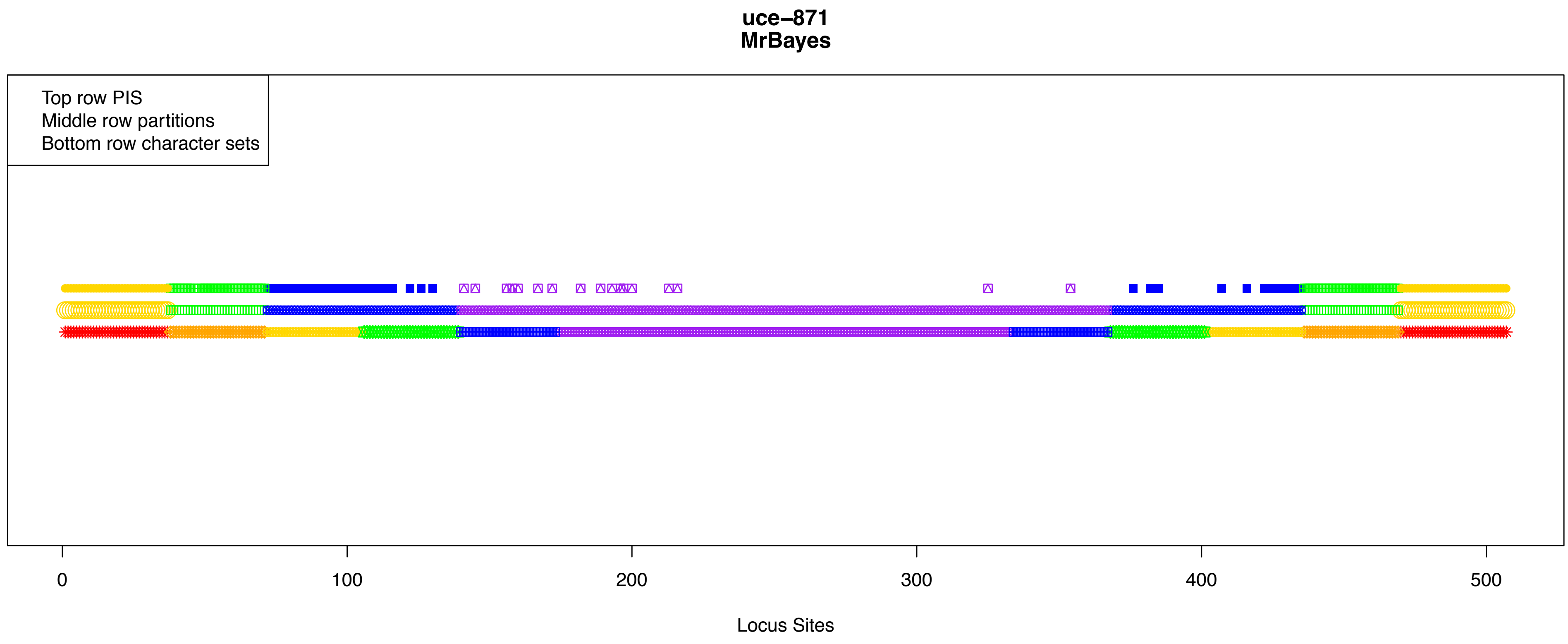
uce–871
MrBayes
Top row PIS
Middle row partitions
Bottom row character sets
0
100
200
300
400
500
Locus Sites

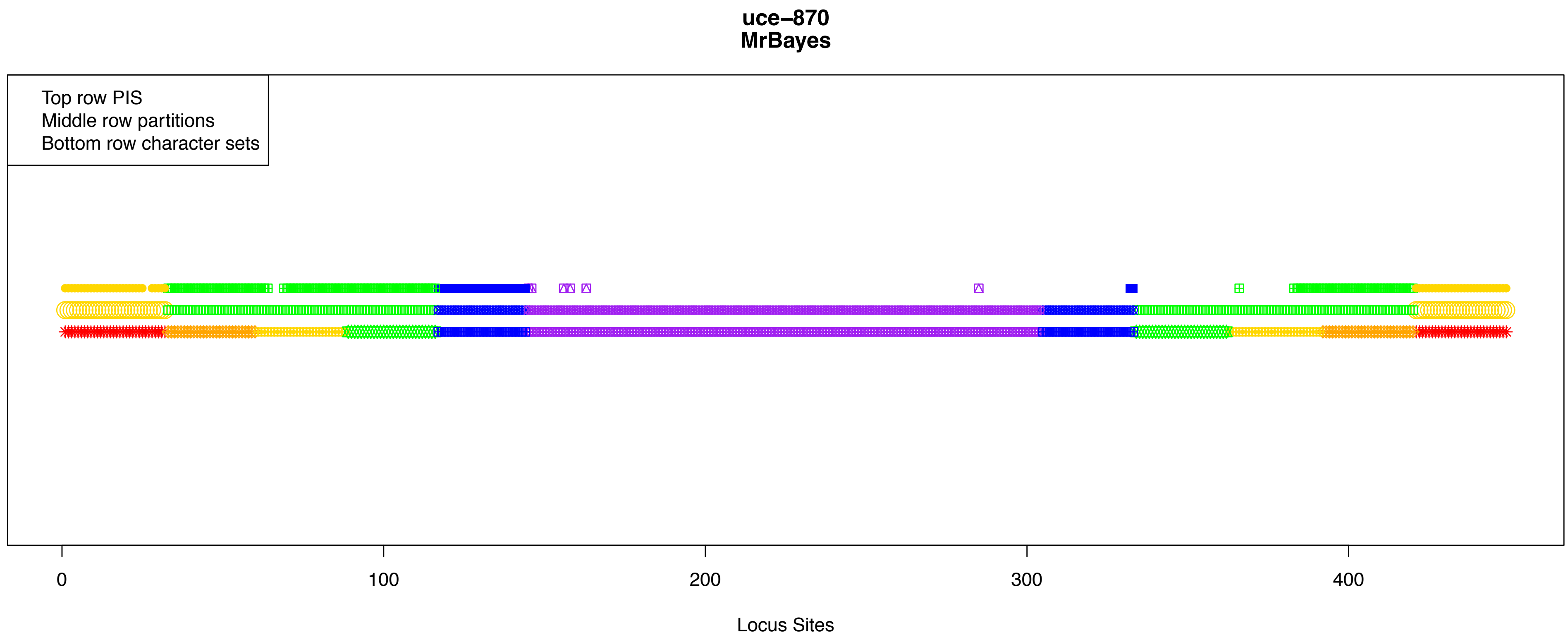

uce–870
MrBayes
Top row PIS
Middle row partitions
Bottom row character sets
0
100
200
300
400
Locus Sites

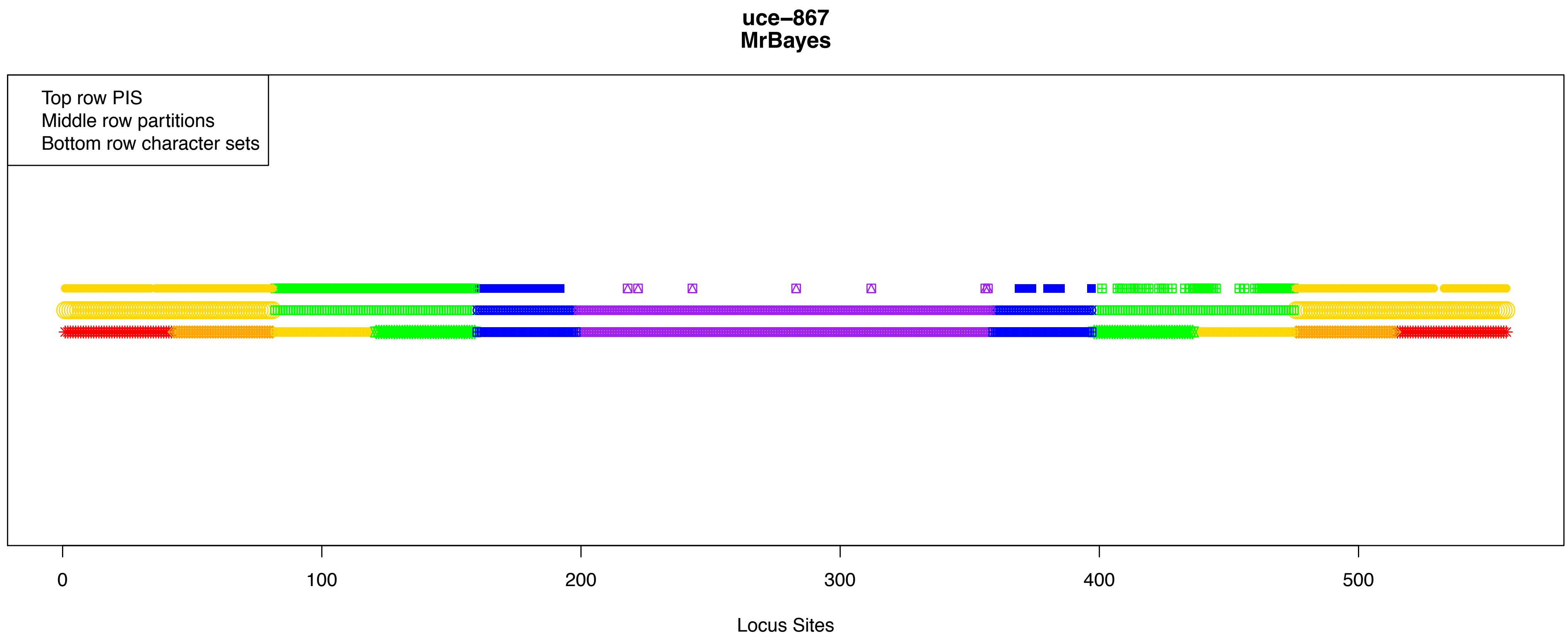

uce–867
MrBayes
Top row PIS
Middle row partitions
Bottom row character sets
0
100
200
300
400
500
Locus Sites

uce–866
MrBayes

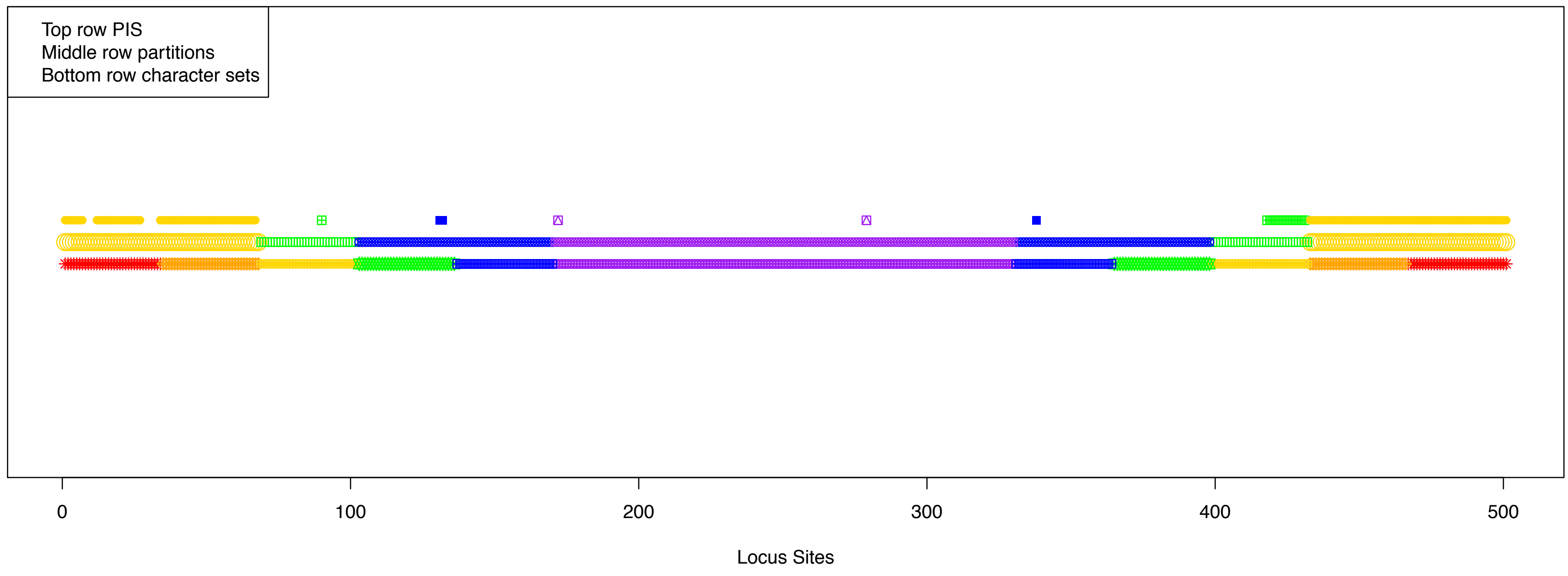

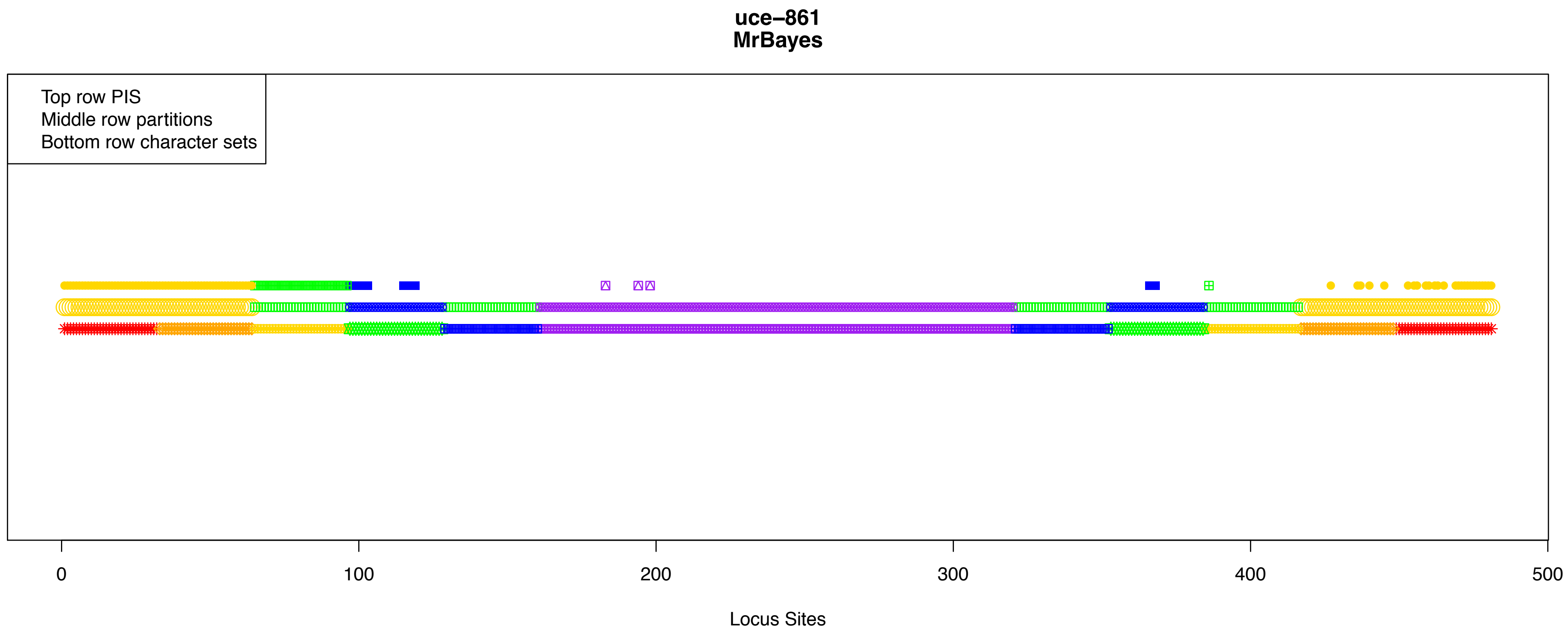
uce–861
MrBayes
Top row PIS
Middle row partitions
Bottom row character sets
0
100
200
300
400
500
Locus Sites

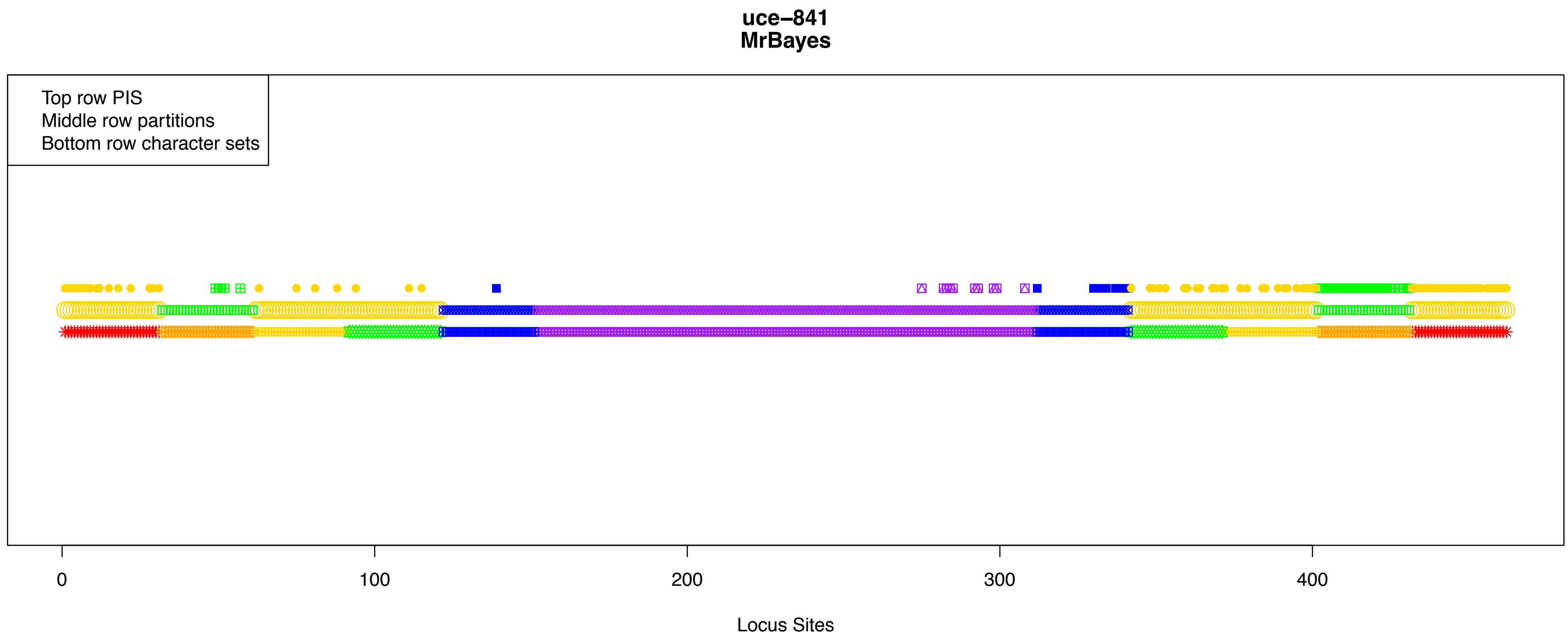
uce–841
MrBayes
Top row PIS
Middle row partitions
Bottom row character sets
0
100
200
300
400
Locus Sites

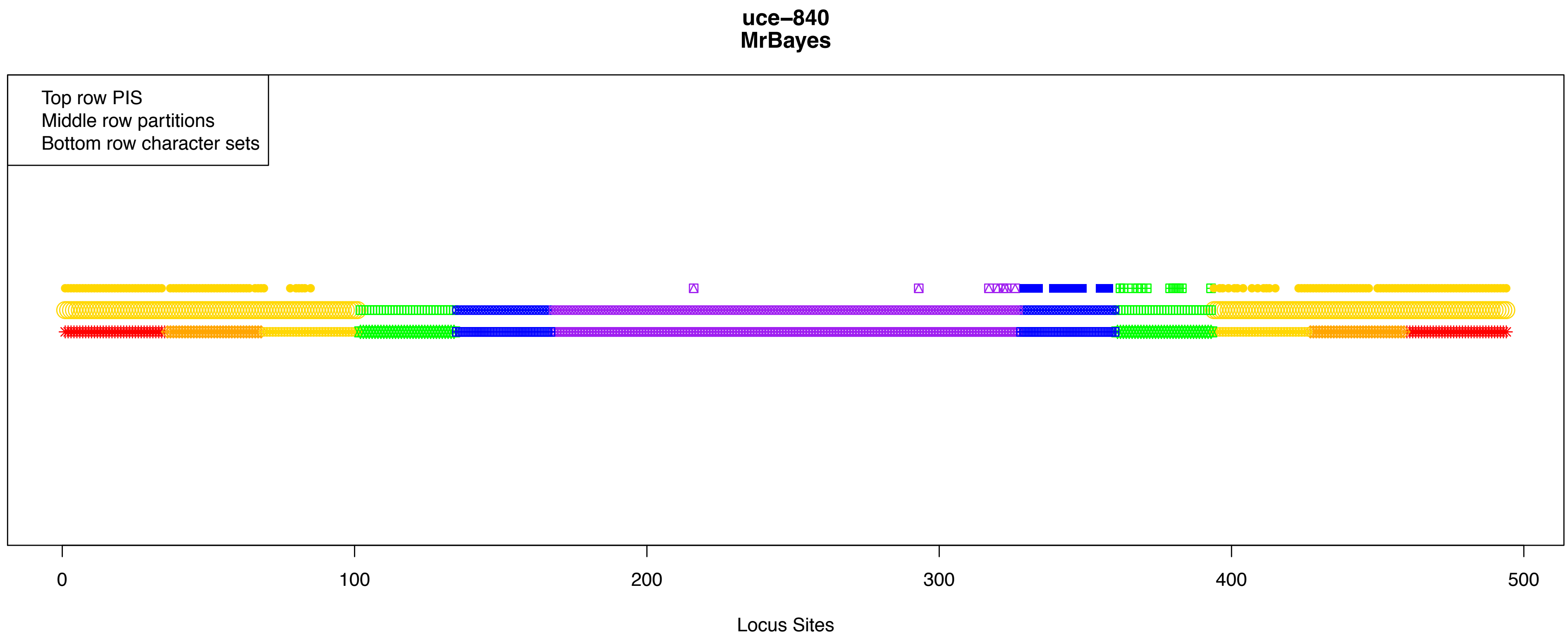

uce–840
MrBayes
Top row PIS
Middle row partitions
Bottom row character sets
0
100
200
300
400
500
Locus Sites

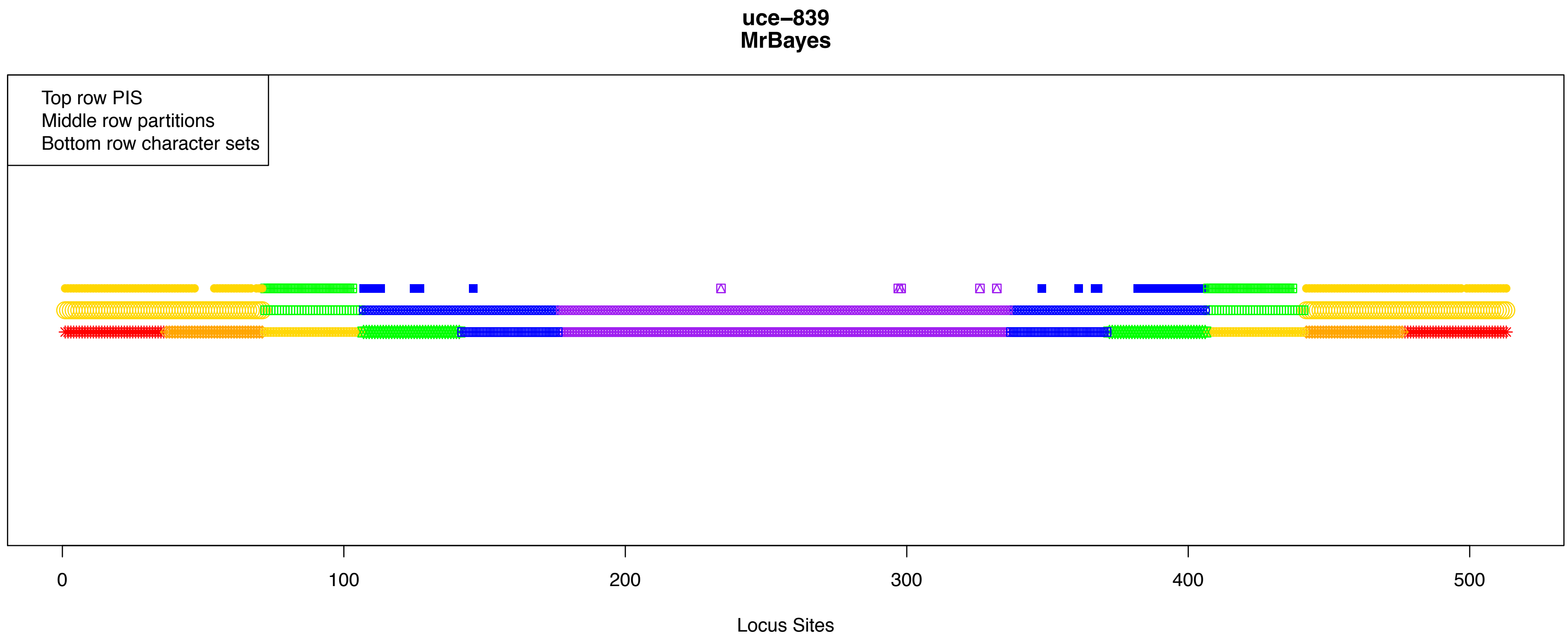
uce–839
MrBayes
Top row PIS
Middle row partitions
Bottom row character sets
0
100
200
300
400
500
Locus Sites

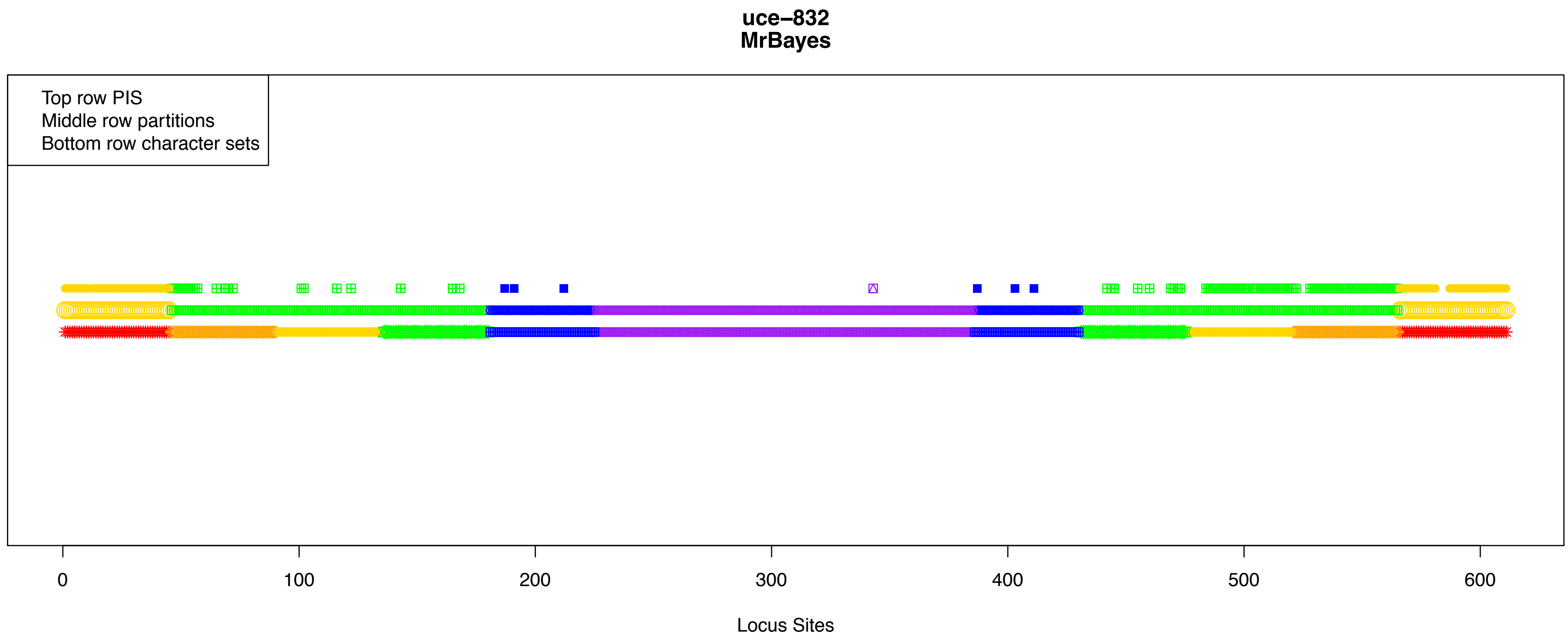

uce–832
MrBayes
Top row PIS
Middle row partitions
Bottom row character sets
0
100
200
300
400
500
600
Locus Sites

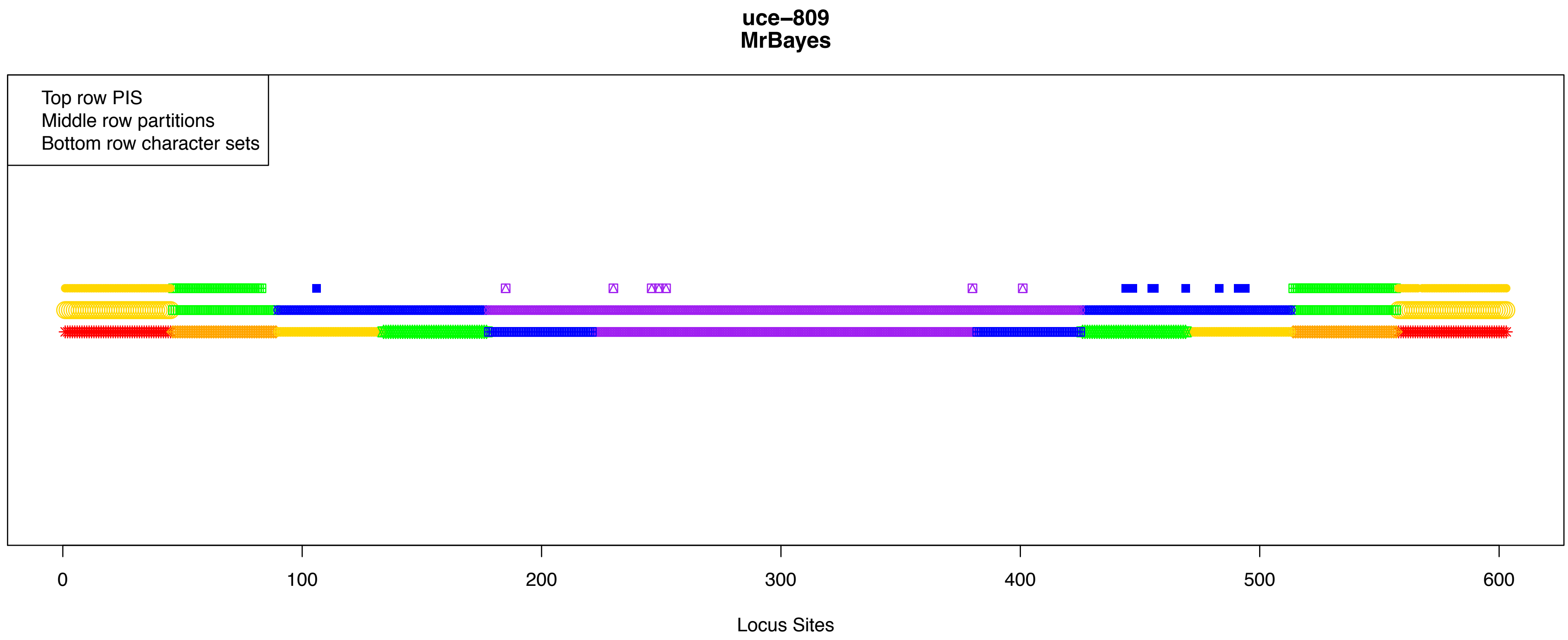

uce–809
MrBayes
Top row PIS
Middle row partitions
Bottom row character sets
0
100
200
300
400
500
600
Locus Sites

uce–808
MrBayes

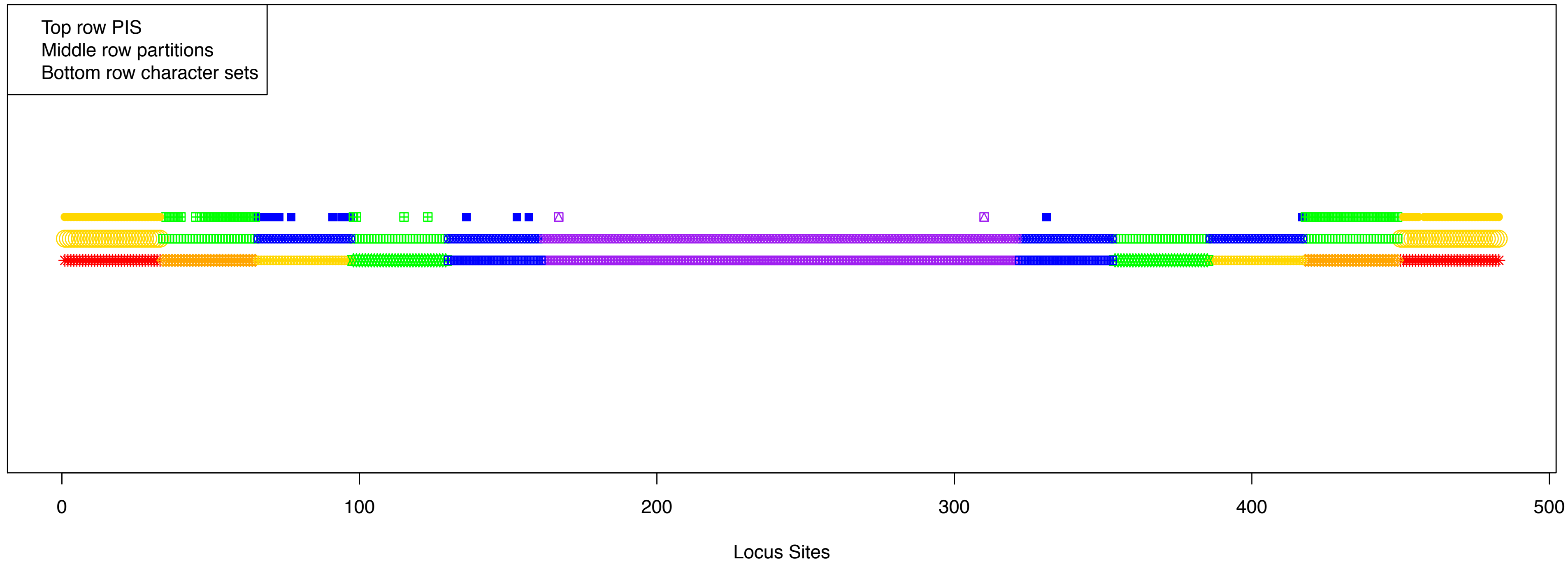

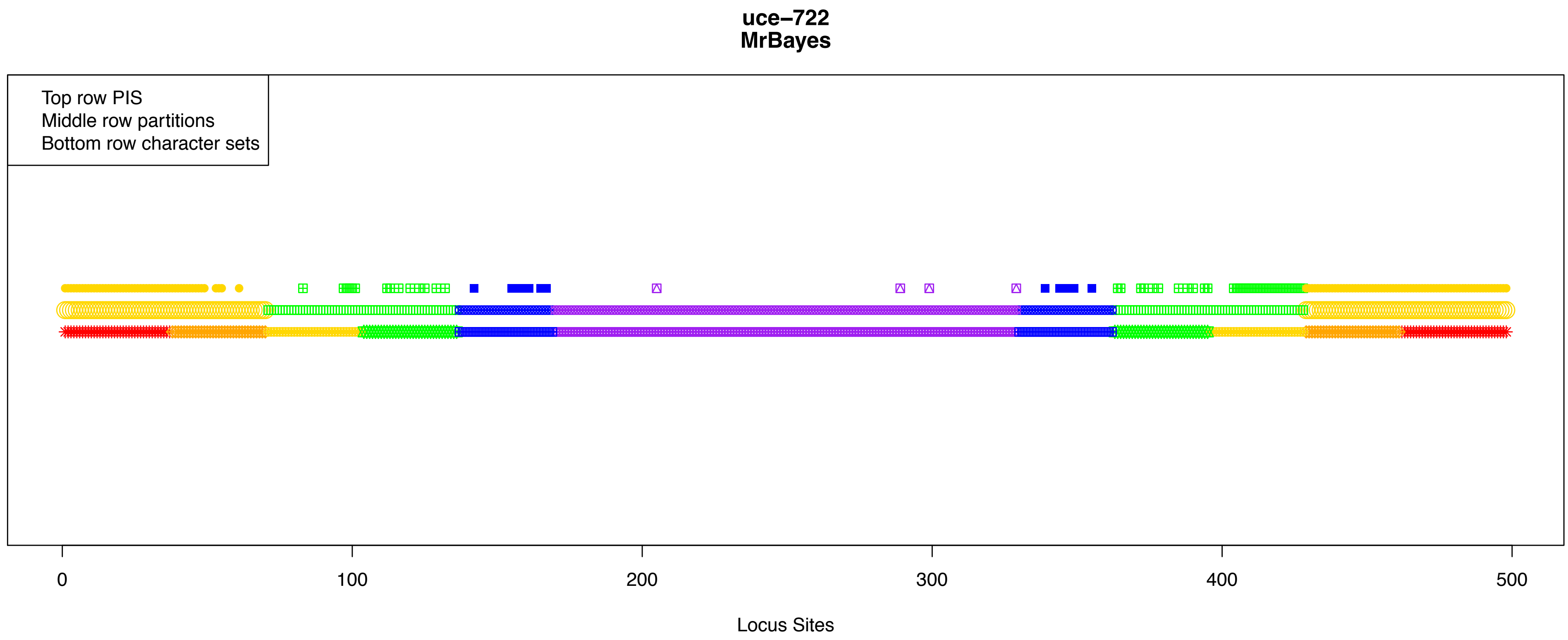

uce–722
MrBayes
Top row PIS
Middle row partitions
Bottom row character sets
0
100
200
300
400
500
Locus Sites

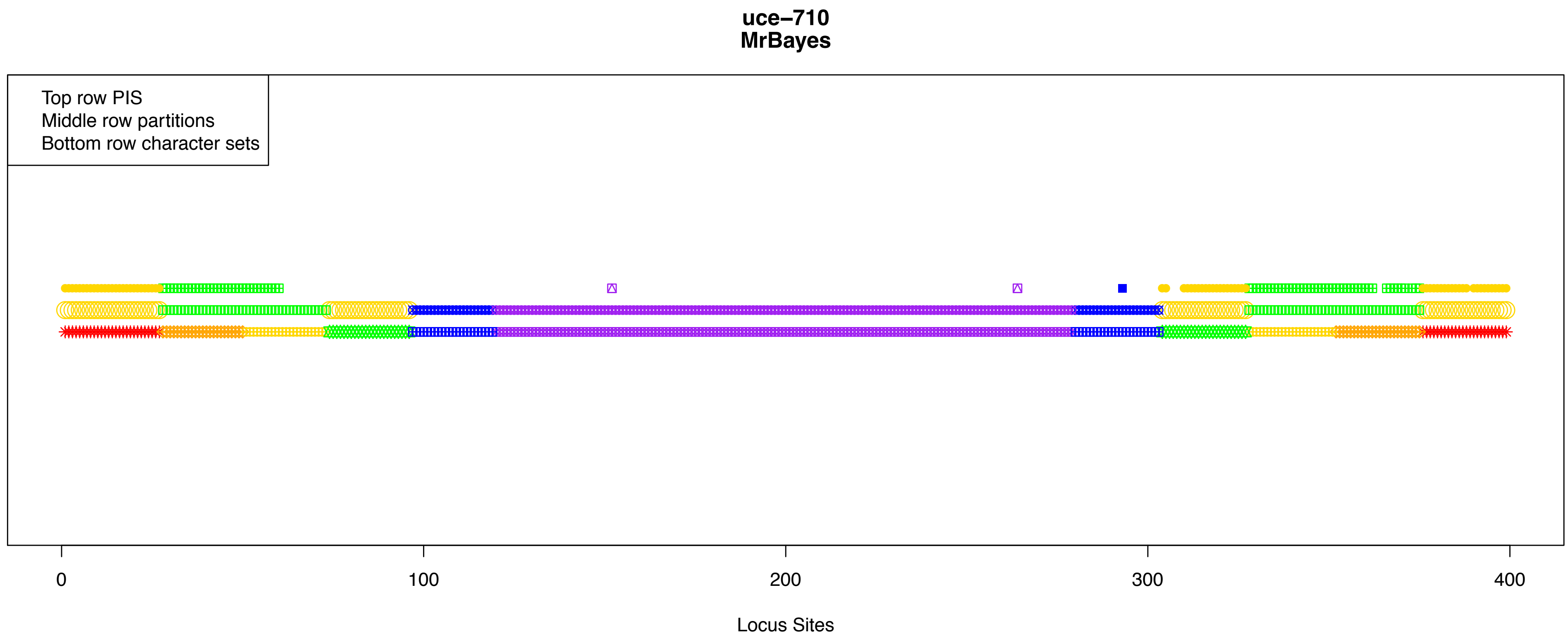
uce–710
MrBayes
Top row PIS
Middle row partitions
Bottom row character sets
0
100
200
300
400
Locus Sites

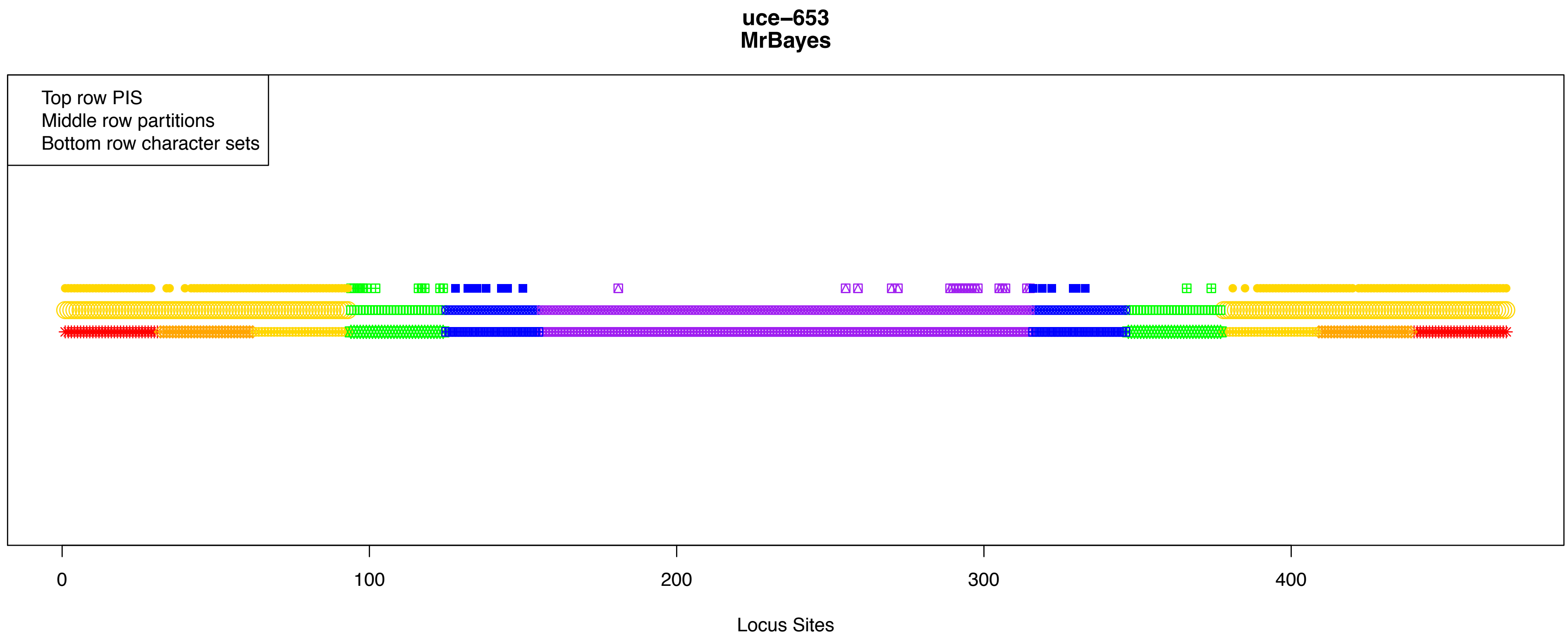

uce–653
MrBayes
Top row PIS
Middle row partitions
Bottom row character sets
0
100
200
300
400
Locus Sites

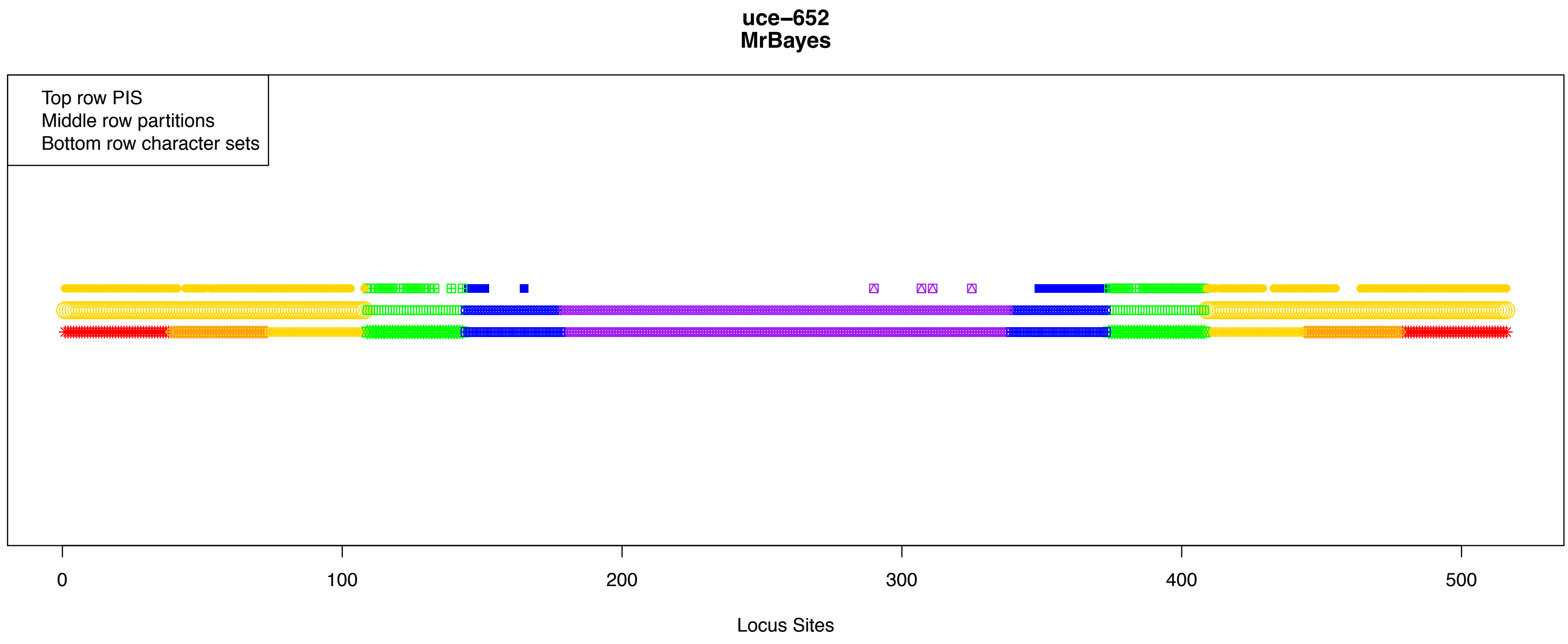
uce–652
MrBayes
Top row PIS
Middle row partitions
Bottom row character sets
0
100
200
300
400
500
Locus Sites

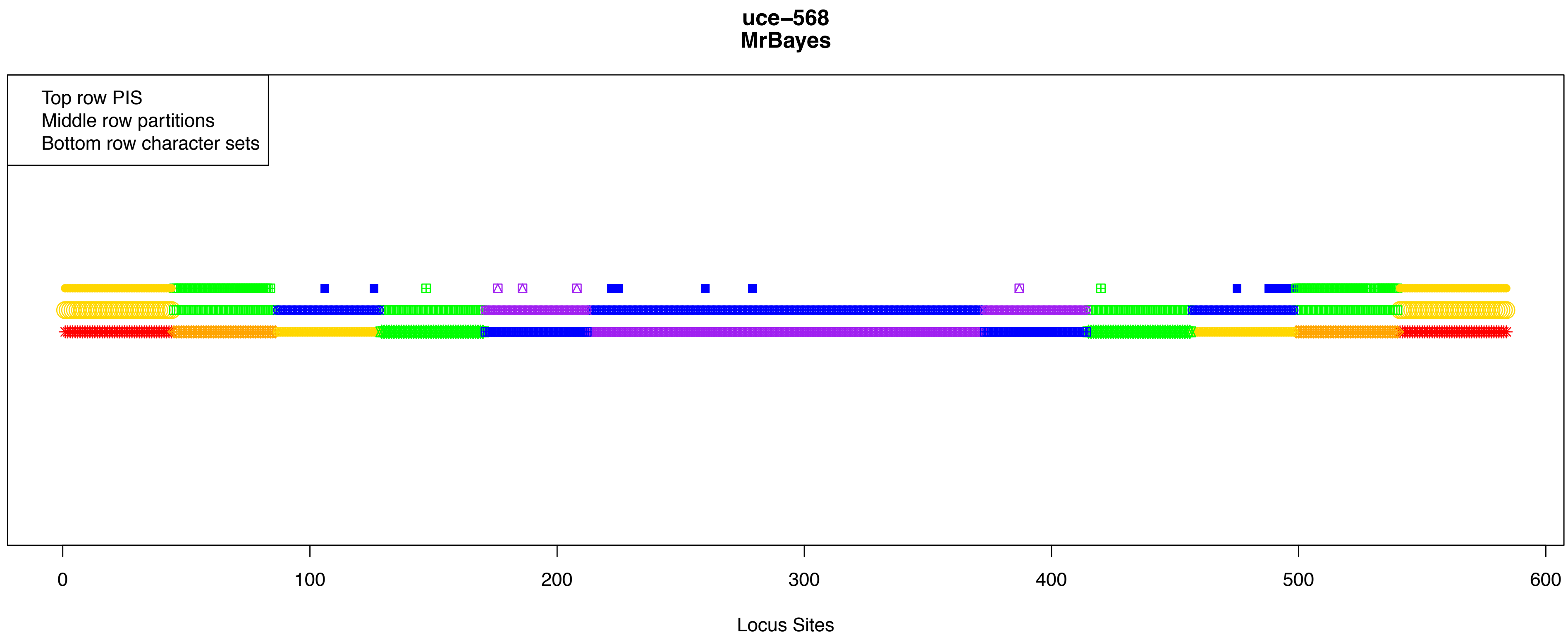
uce–568
MrBayes
Top row PIS
Middle row partitions
Bottom row character sets
0
100
200
300
400
500
600
Locus Sites

uce–561
MrBayes

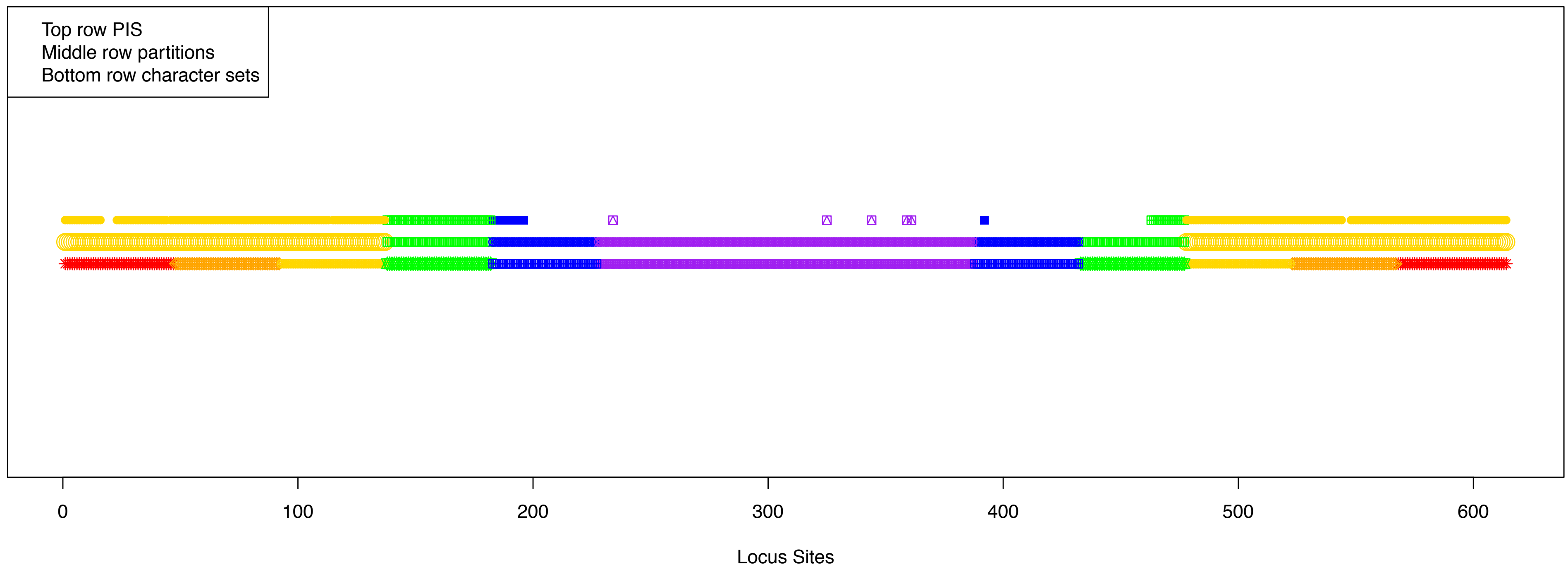

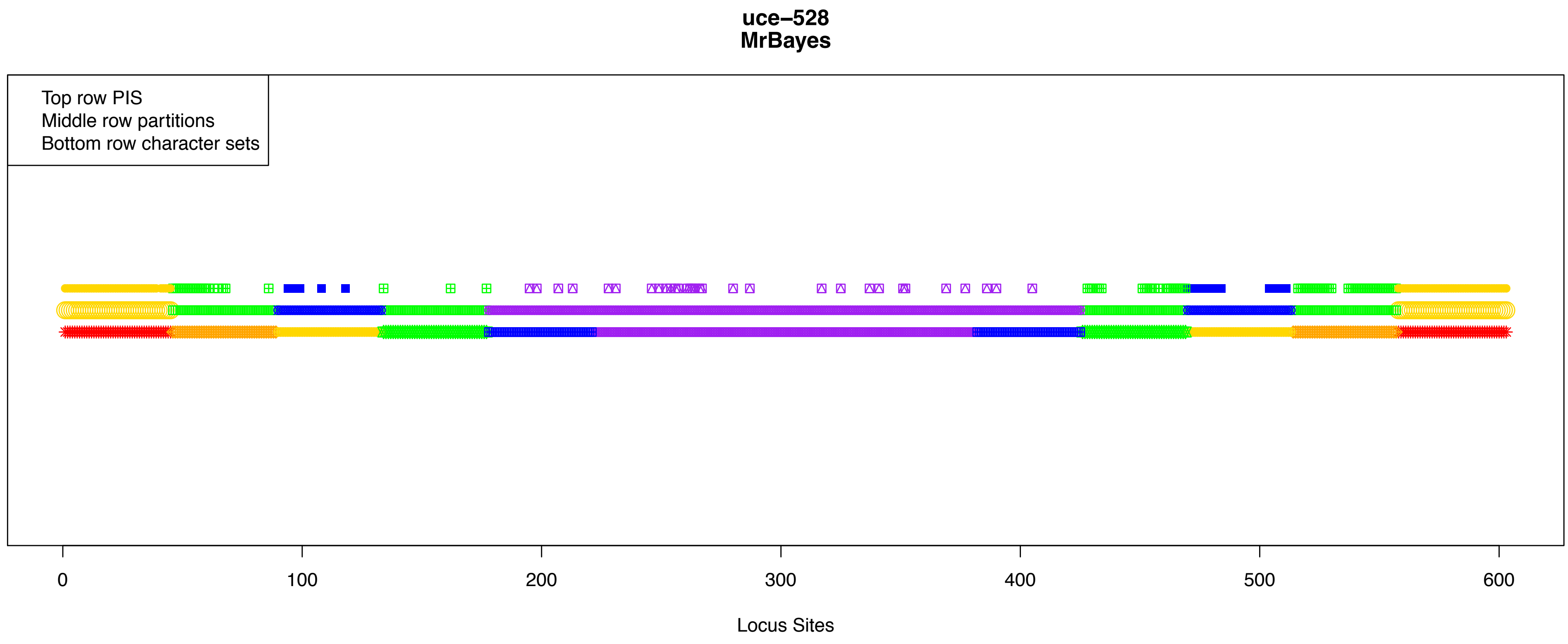
uce–528
MrBayes
Top row PIS
Middle row partitions
Bottom row character sets
0
100
200
300
400
500
600
Locus Sites

uce–495
MrBayes

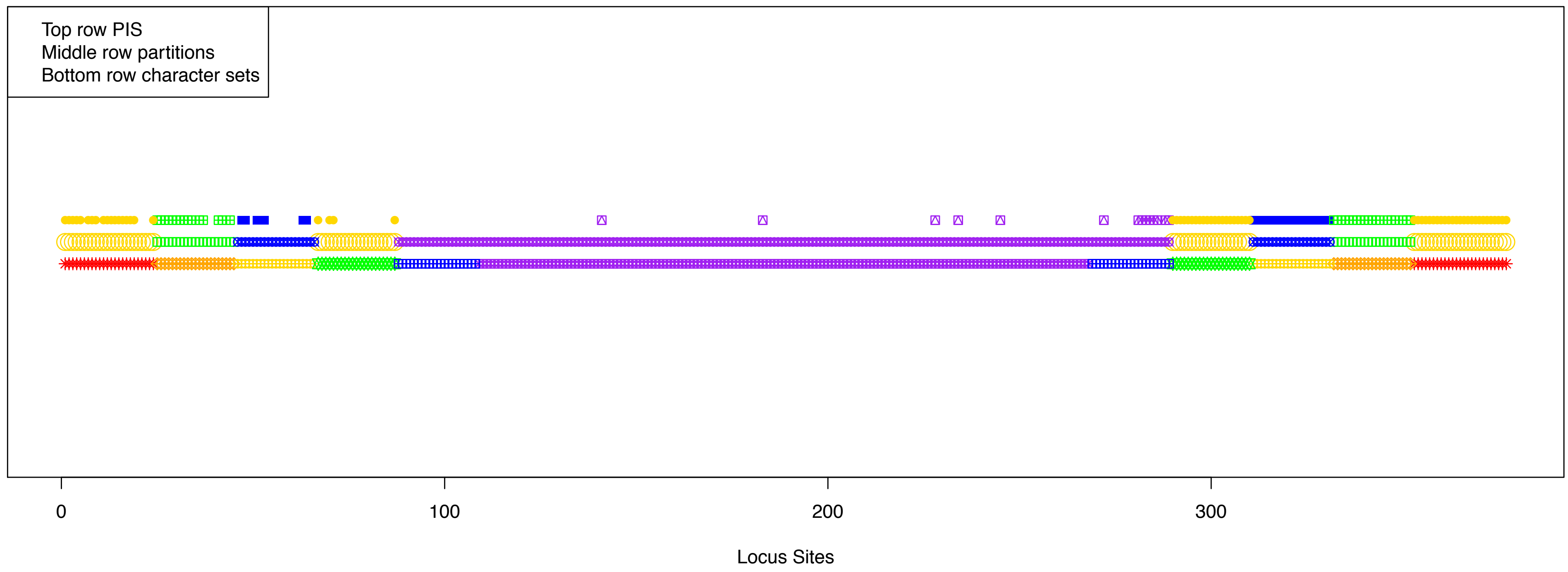

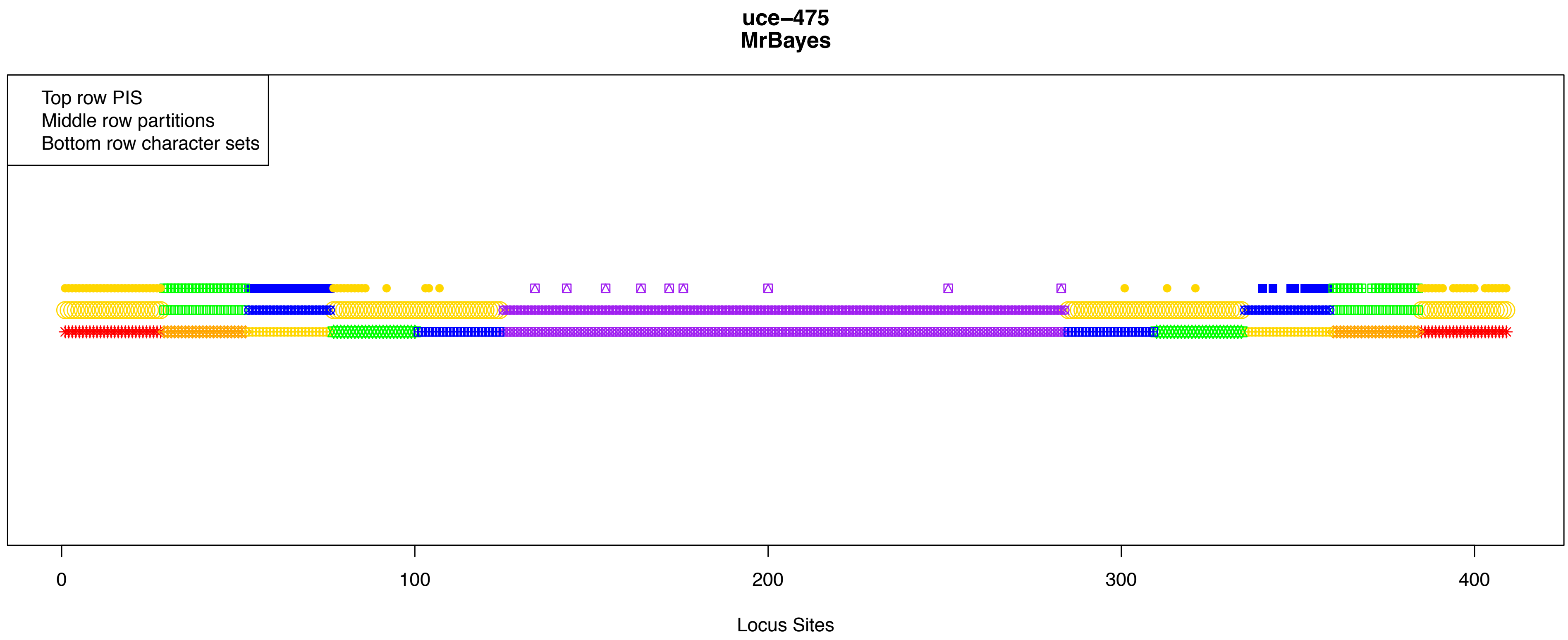

uce–475
MrBayes
Top row PIS
Middle row partitions
Bottom row character sets
0
100
200
300
400
Locus Sites

uce–453
MrBayes

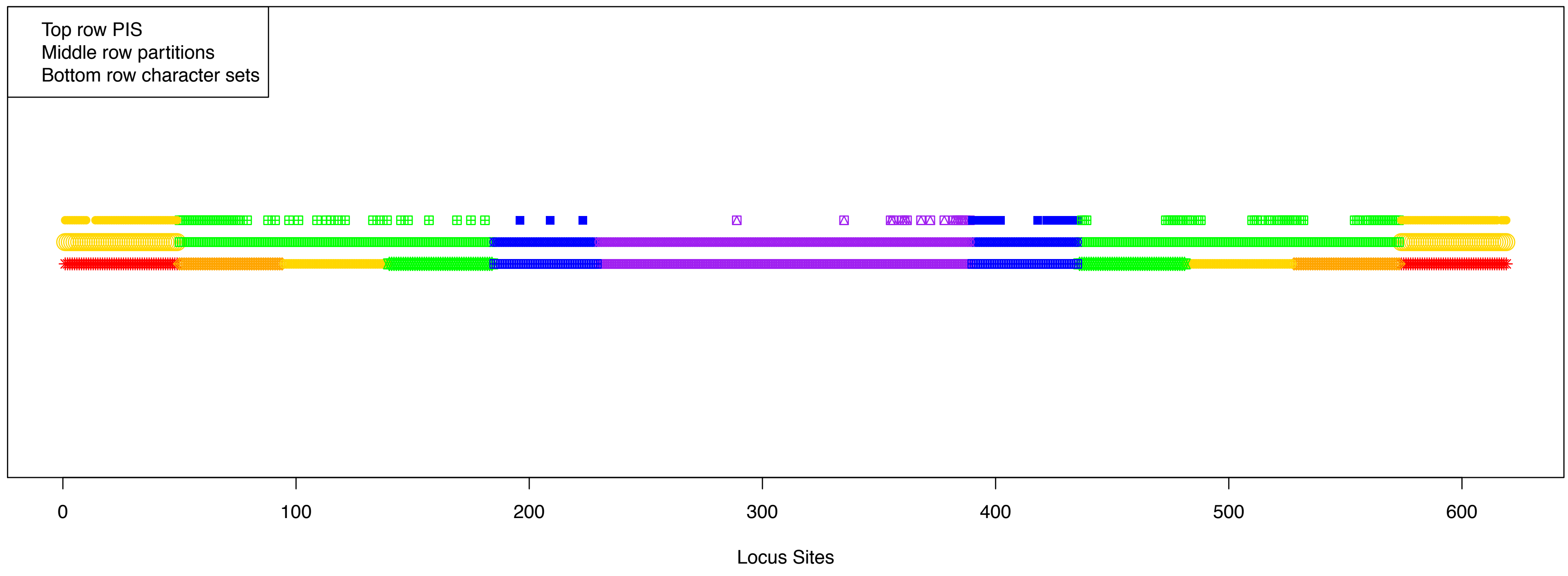

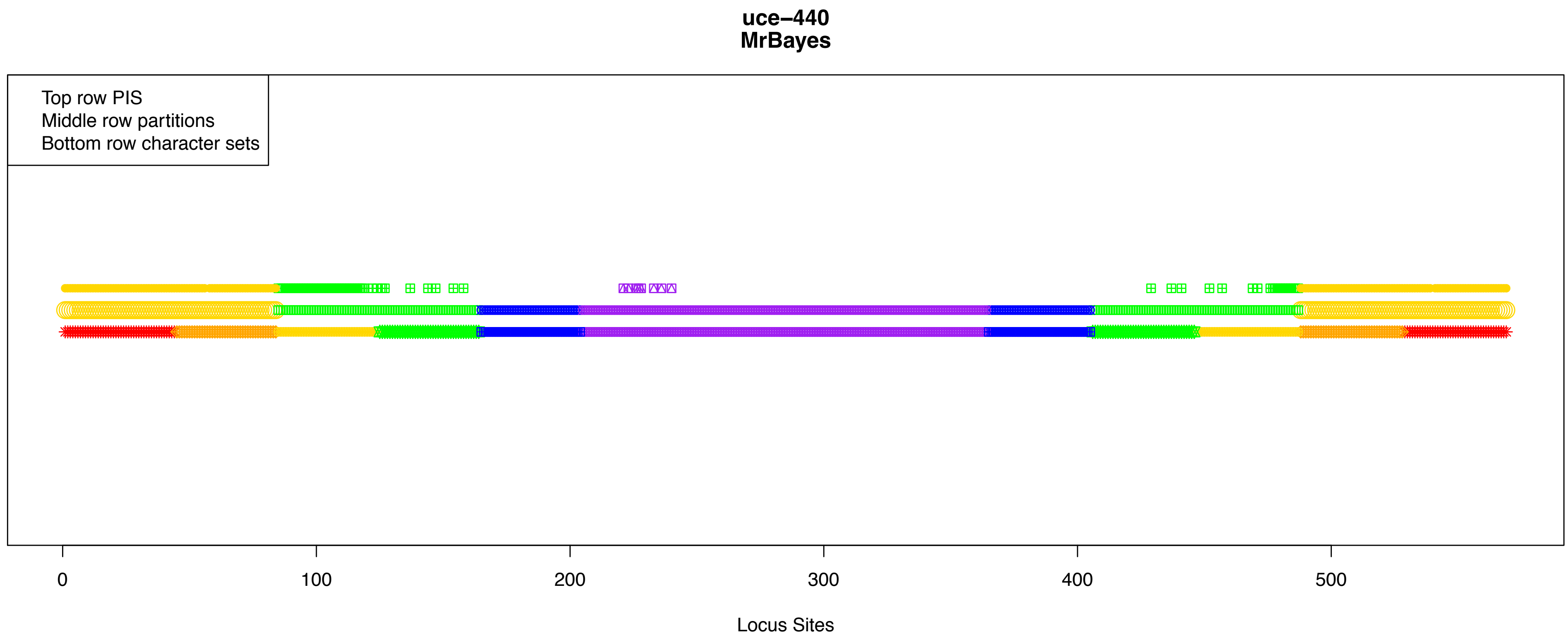

uce–440
MrBayes
Top row PIS
Middle row partitions
Bottom row character sets
0
100
200
300
400
500
Locus Sites

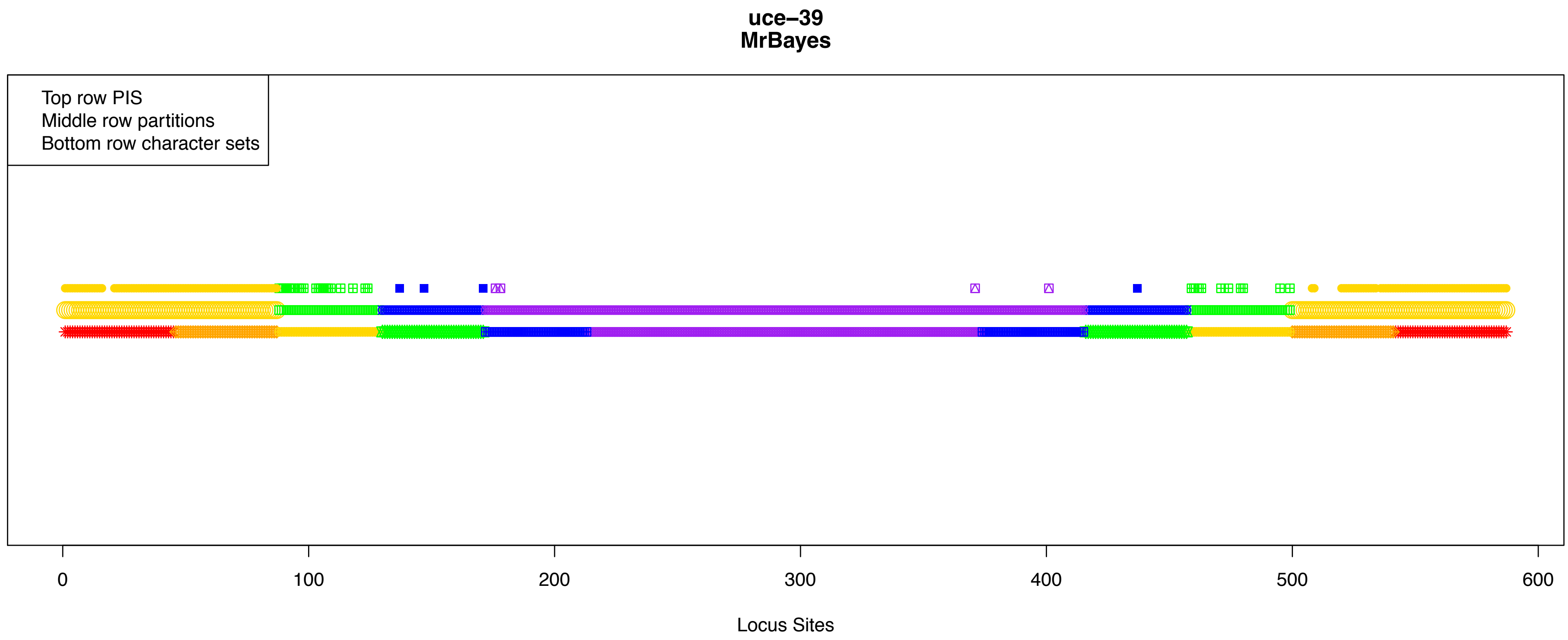

uce–39
MrBayes
Top row PIS
Middle row partitions
Bottom row character sets
0
100
200
300
400
500
600
Locus Sites

uce–378
MrBayes

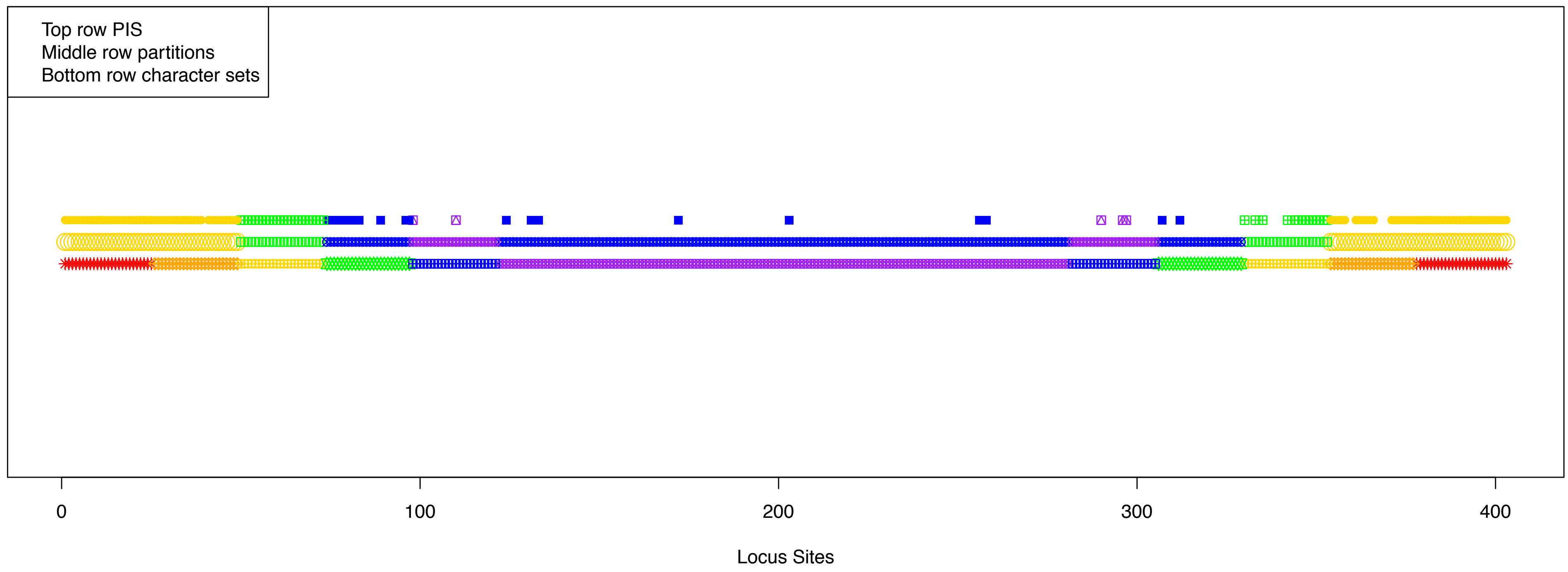

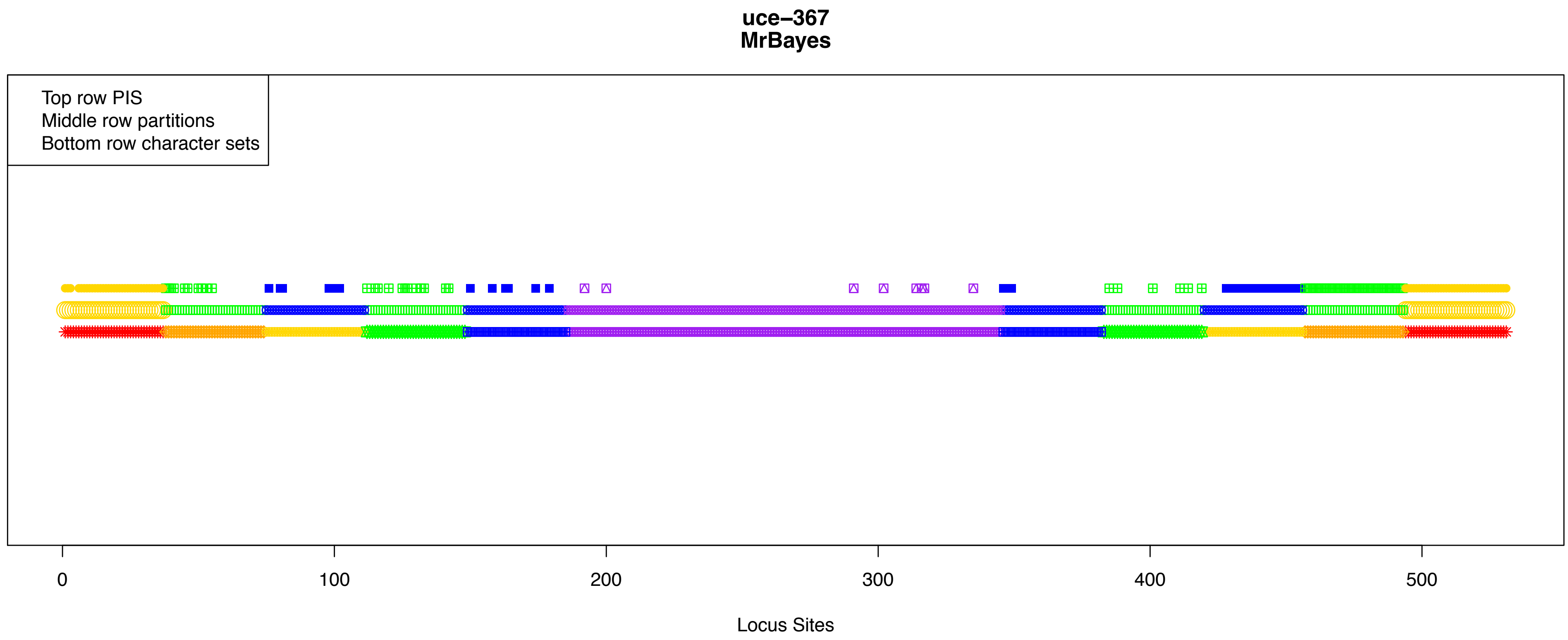
uce–367
MrBayes
Top row PIS
Middle row partitions
Bottom row character sets
0
100
200
300
400
500
Locus Sites

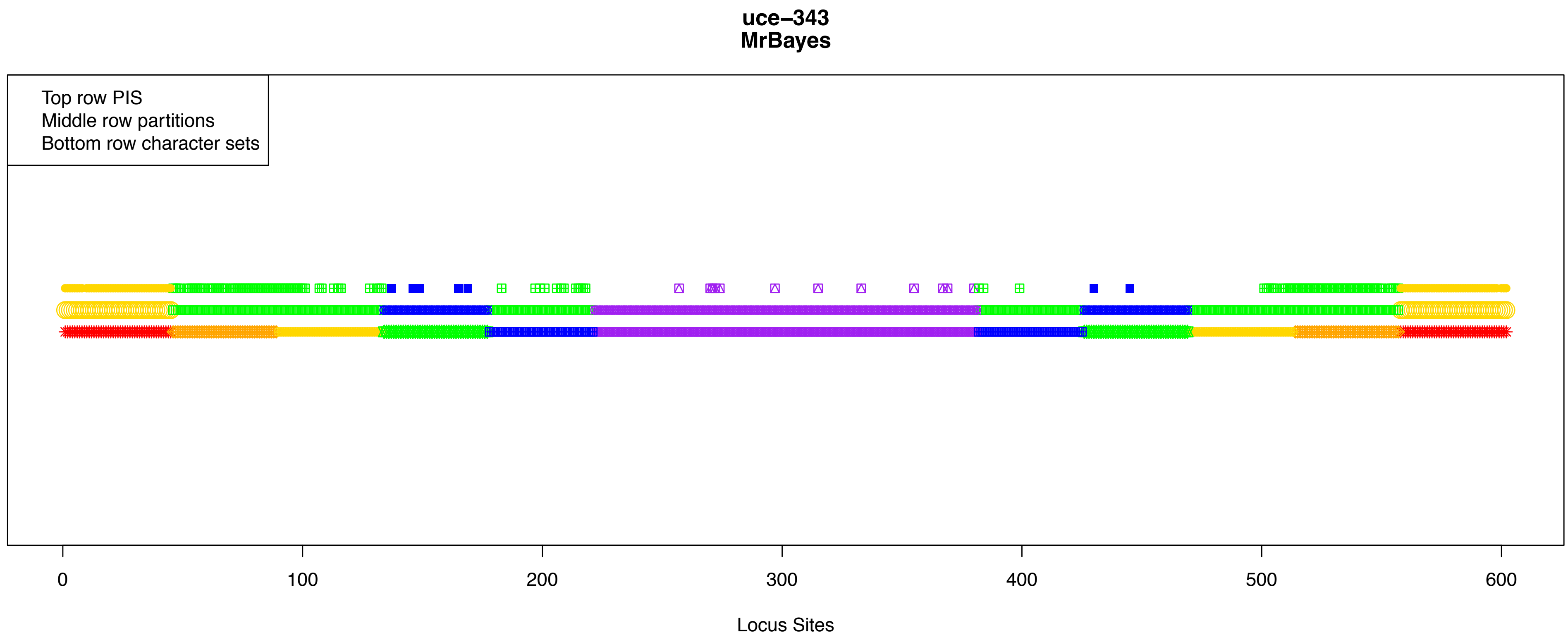

uce–343
MrBayes
Top row PIS
Middle row partitions
Bottom row character sets
0
100
200
300
400
500
600
Locus Sites

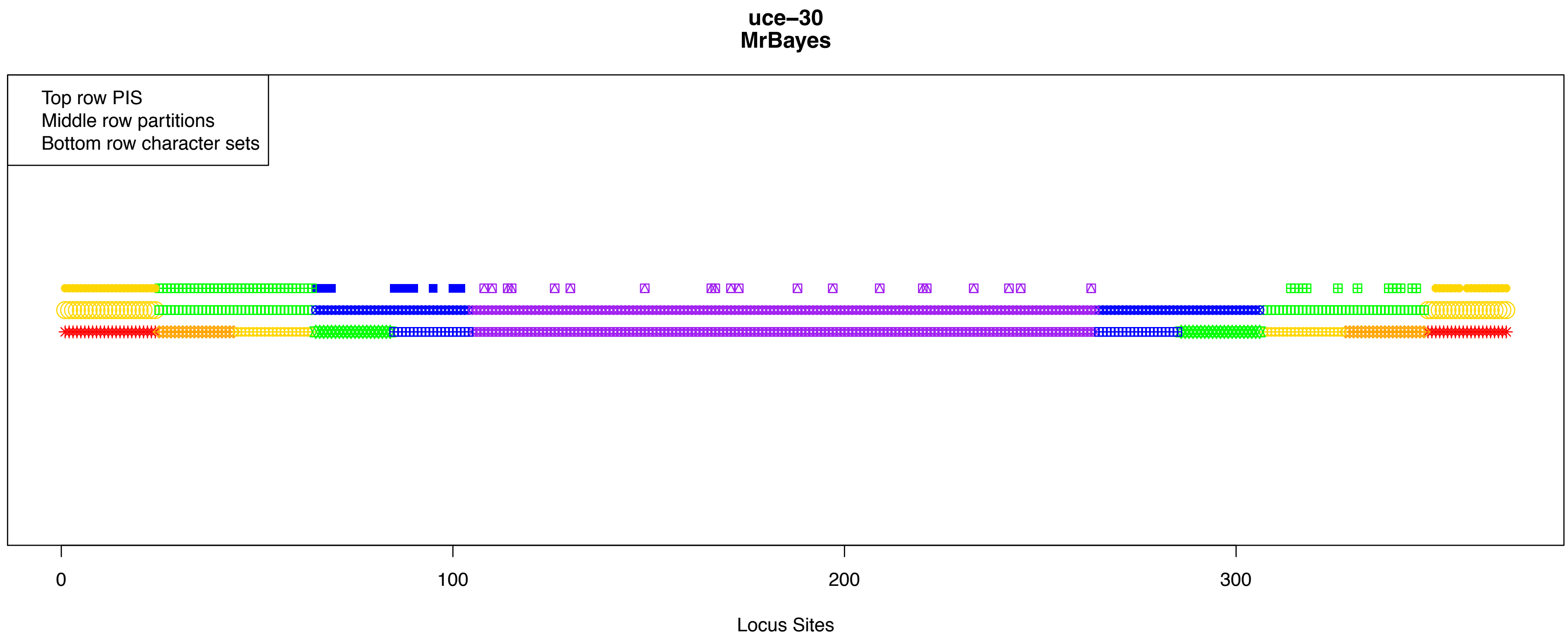
uce–30
MrBayes
Top row PIS
Middle row partitions
Bottom row character sets
0
100
200
300
Locus Sites

uce–267
MrBayes

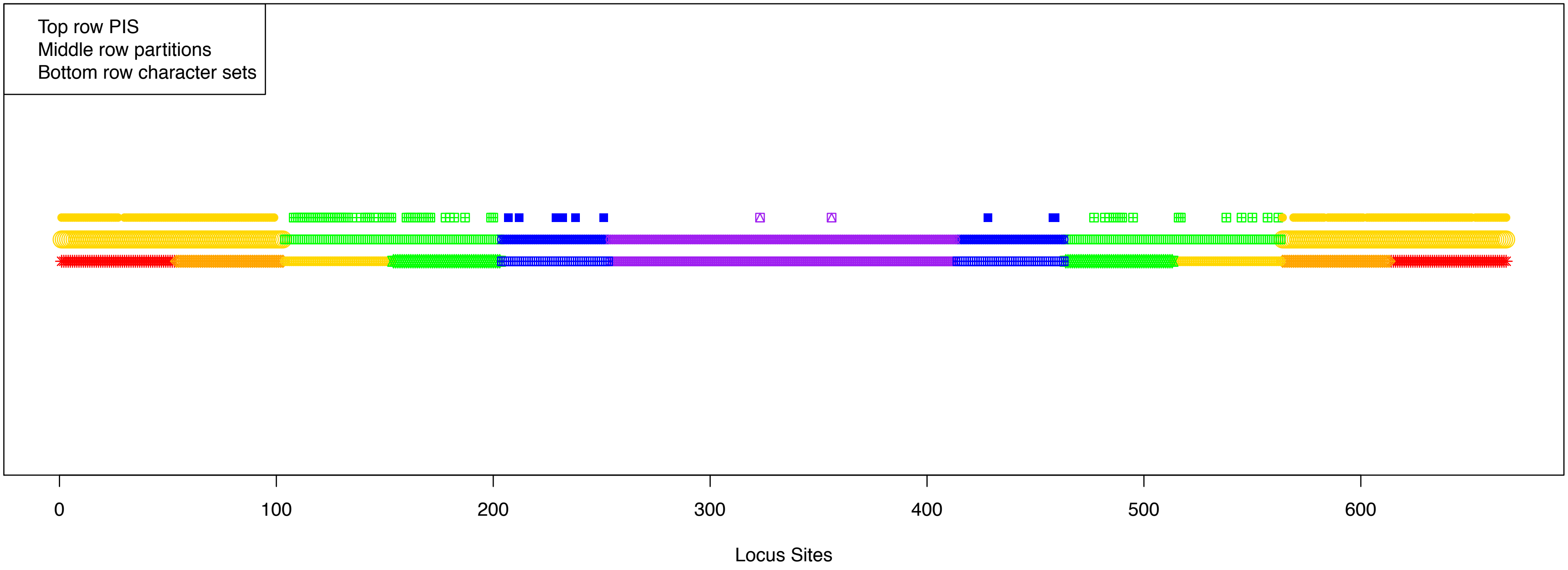

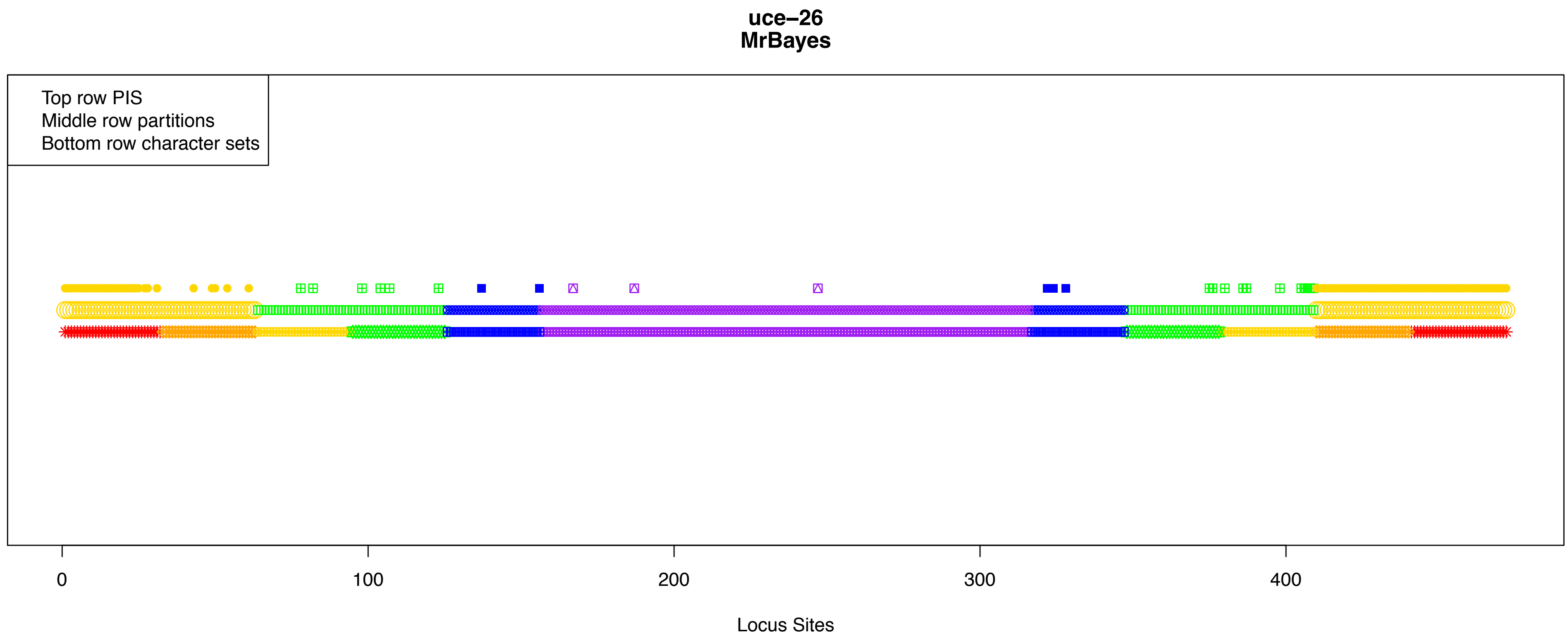
uce–26
MrBayes
Top row PIS
Middle row partitions
Bottom row character sets
0
100
200
300
400
Locus Sites

uce–243
MrBayes

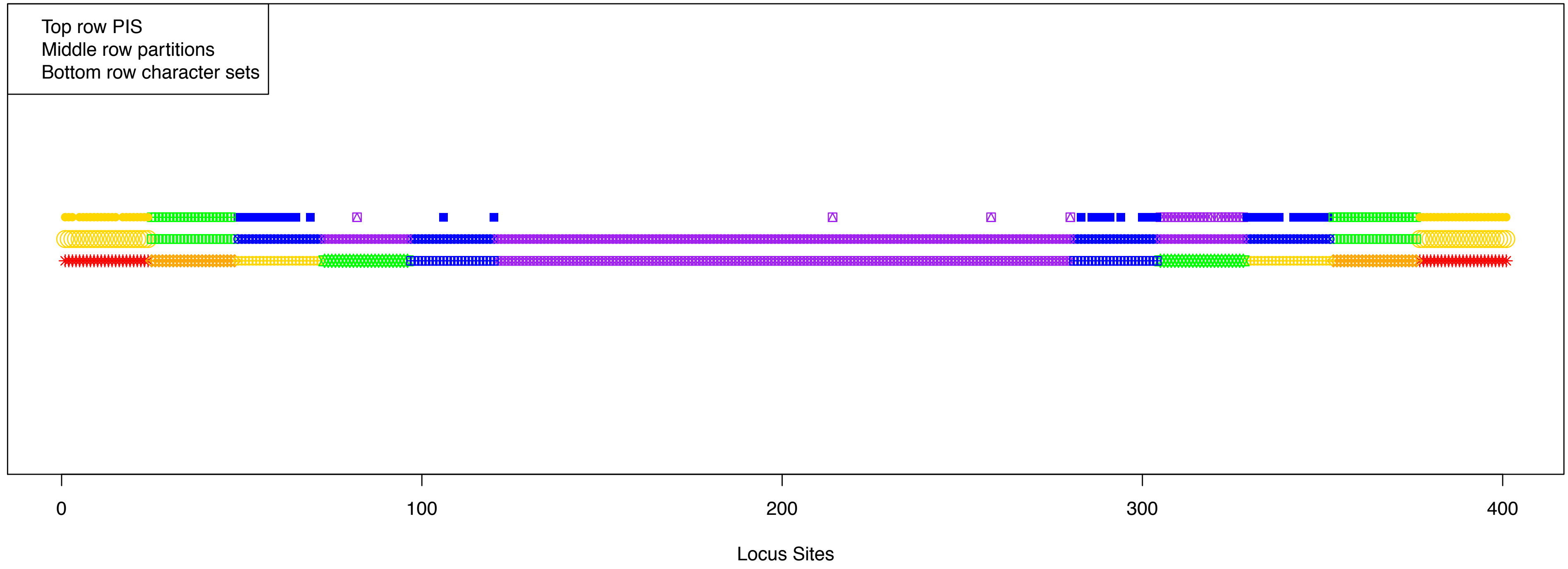

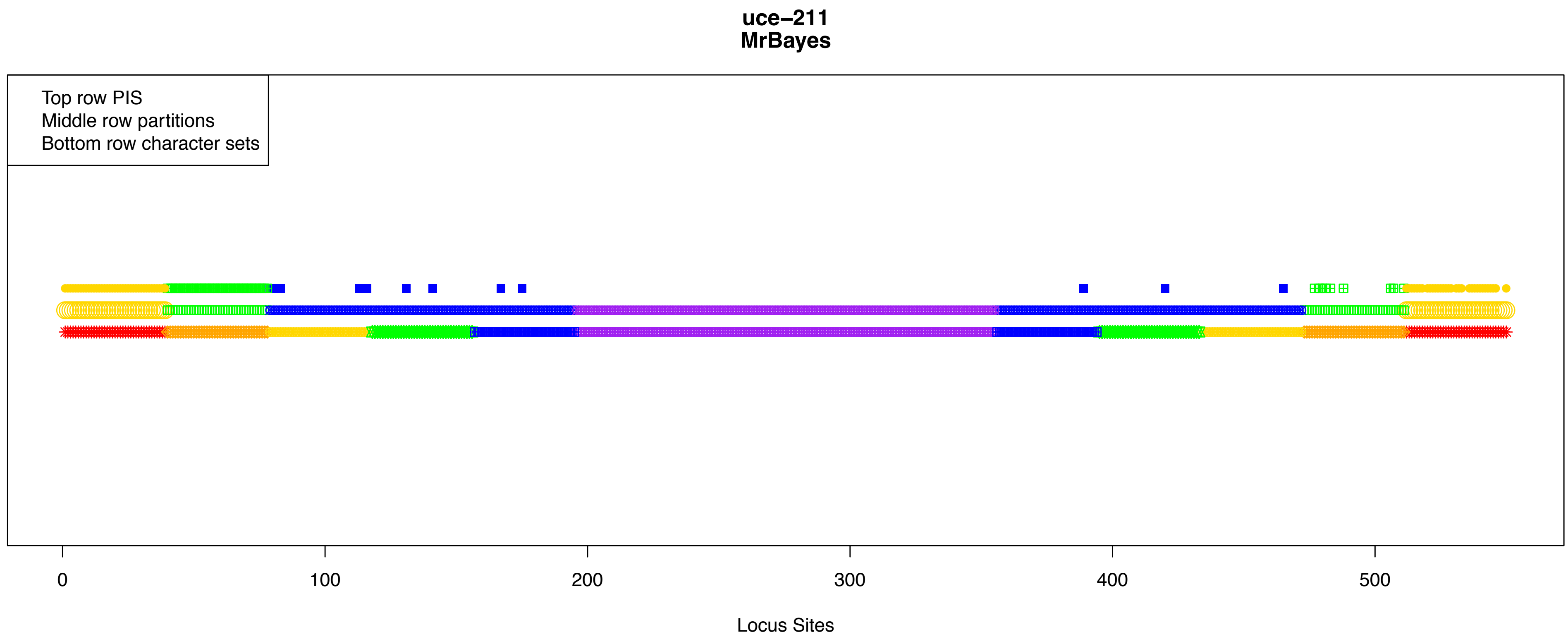
uce–211
MrBayes
Top row PIS
Middle row partitions
Bottom row character sets
0
100
200
300
400
500
Locus Sites

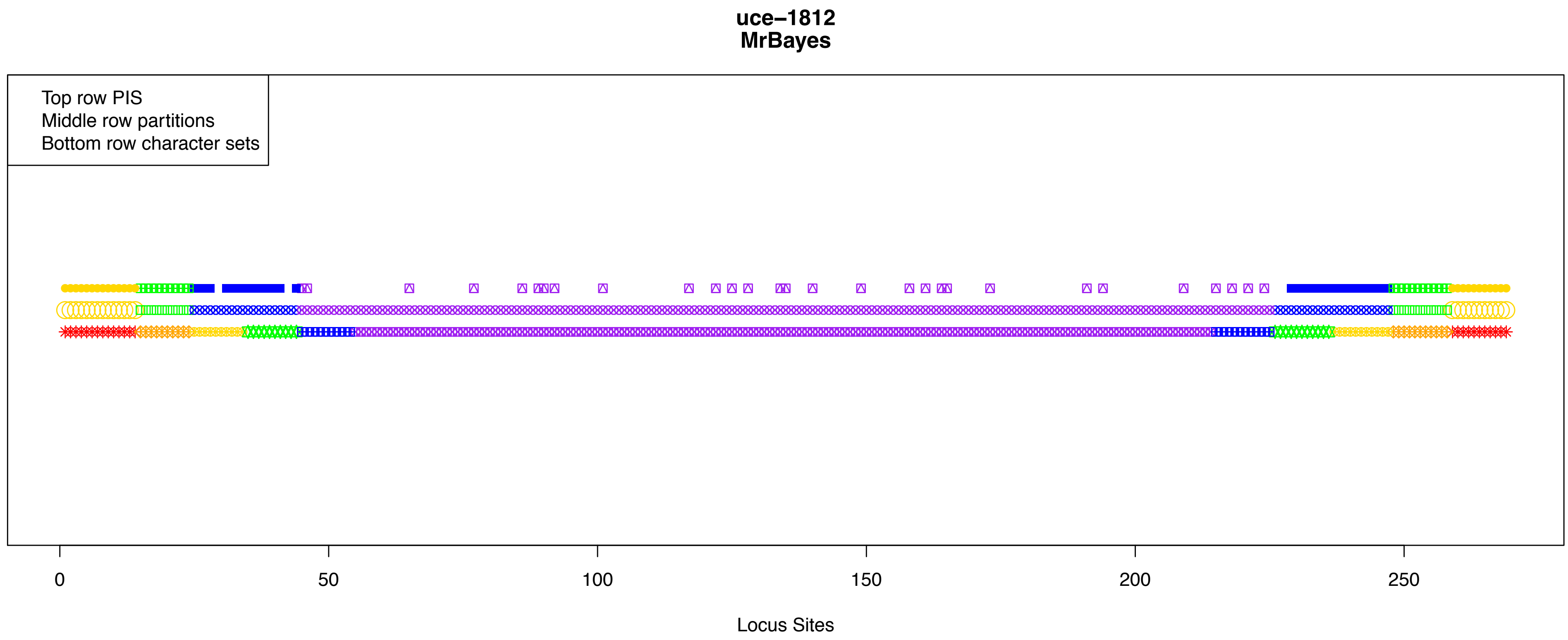

uce–1812
MrBayes
Top row PIS
Middle row partitions
Bottom row character sets
0
50
100
150
200
250
Locus Sites

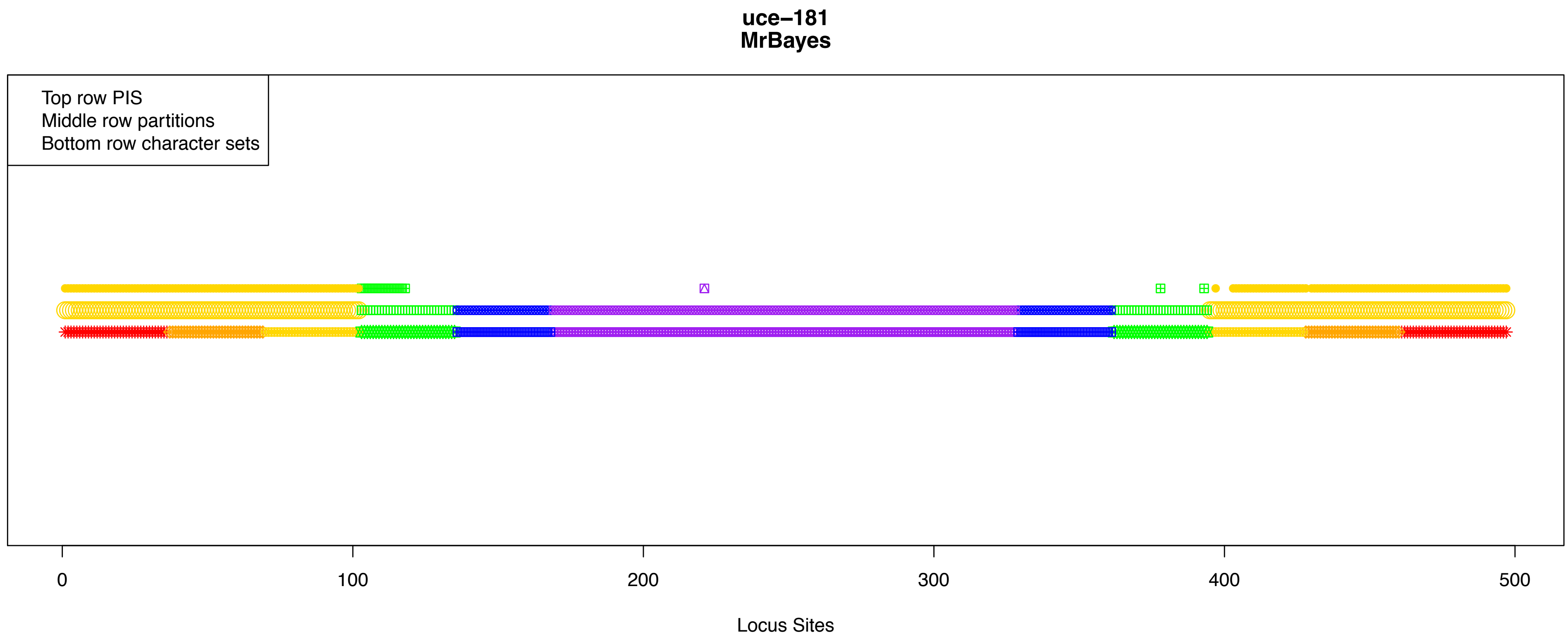
uce–181
MrBayes
Top row PIS
Middle row partitions
Bottom row character sets
0
100
200
300
400
500
Locus Sites

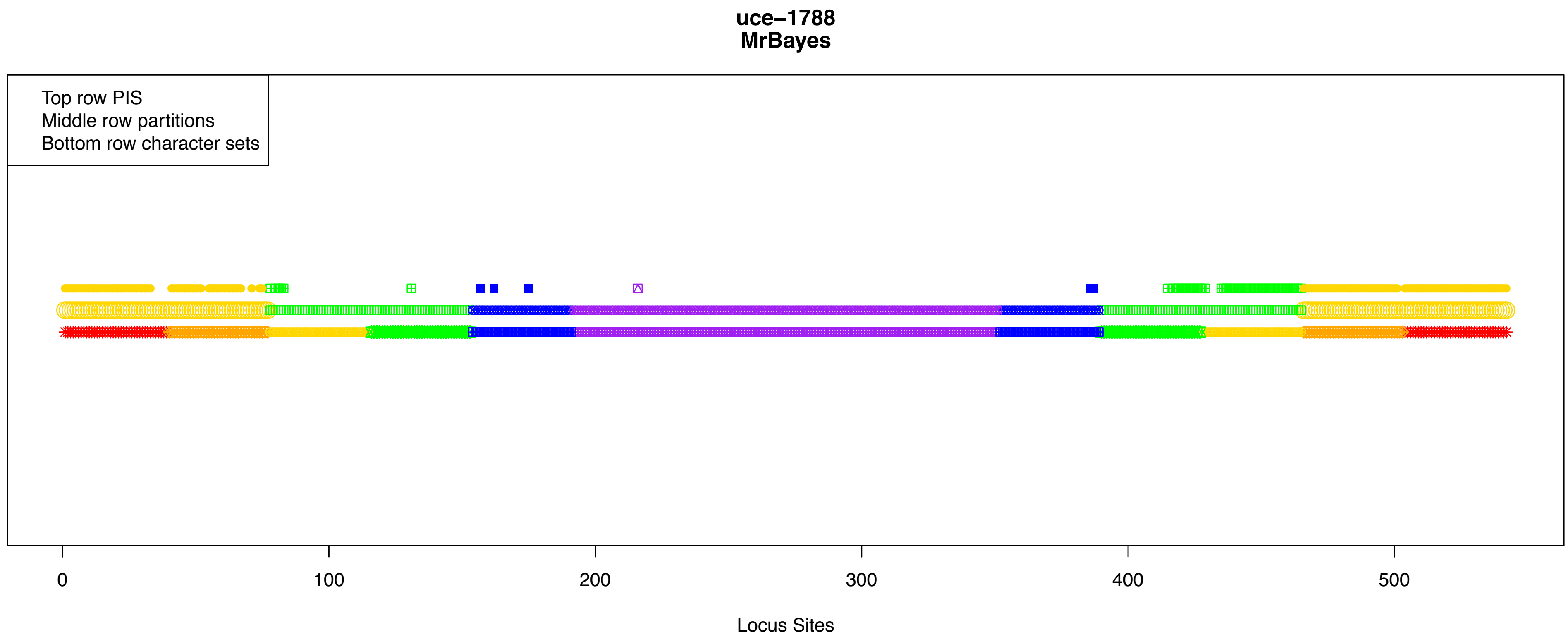
uce–1788
MrBayes
Top row PIS
Middle row partitions
Bottom row character sets
0
100
200
300
400
500
Locus Sites

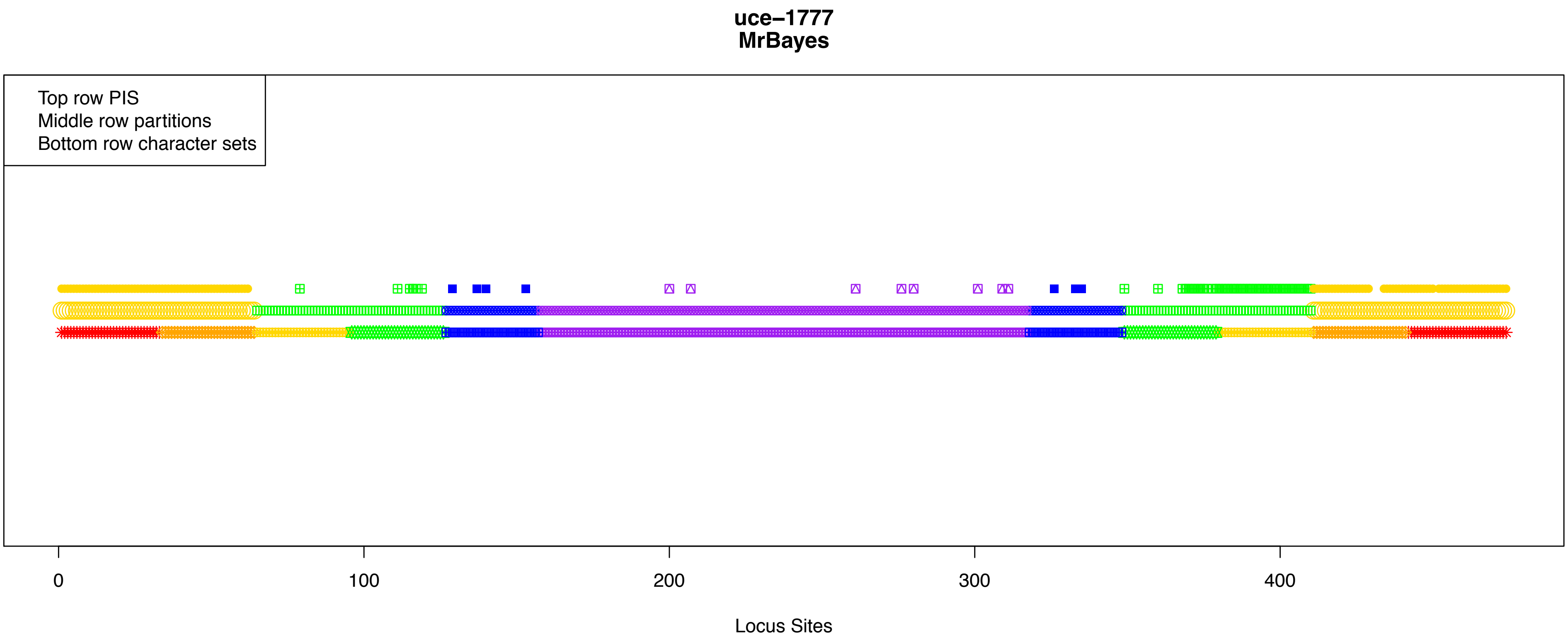
uce–1777
MrBayes
Top row PIS
Middle row partitions
Bottom row character sets
0
100
200
300
400
Locus Sites

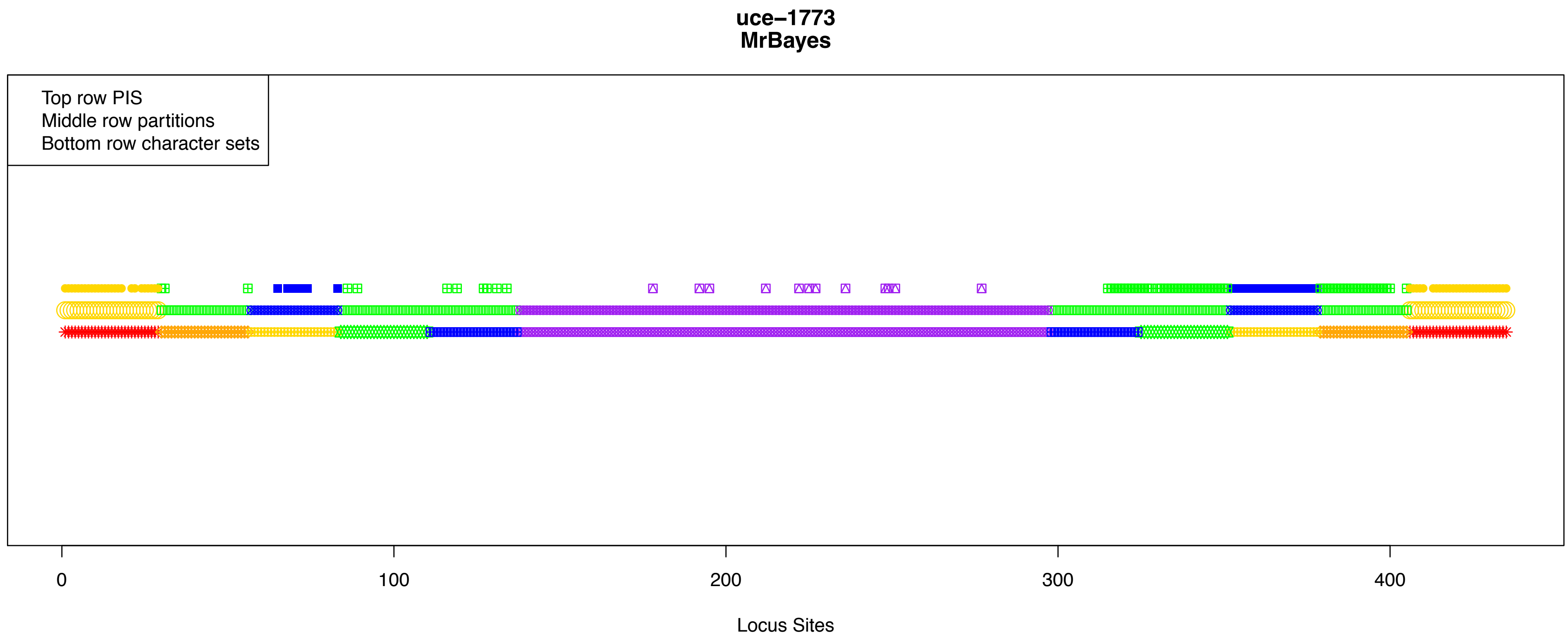
uce−1773
MrBayes
Top row PIS
Middle row partitions
Bottom row character sets
0
100
200
300
400
Locus Sites

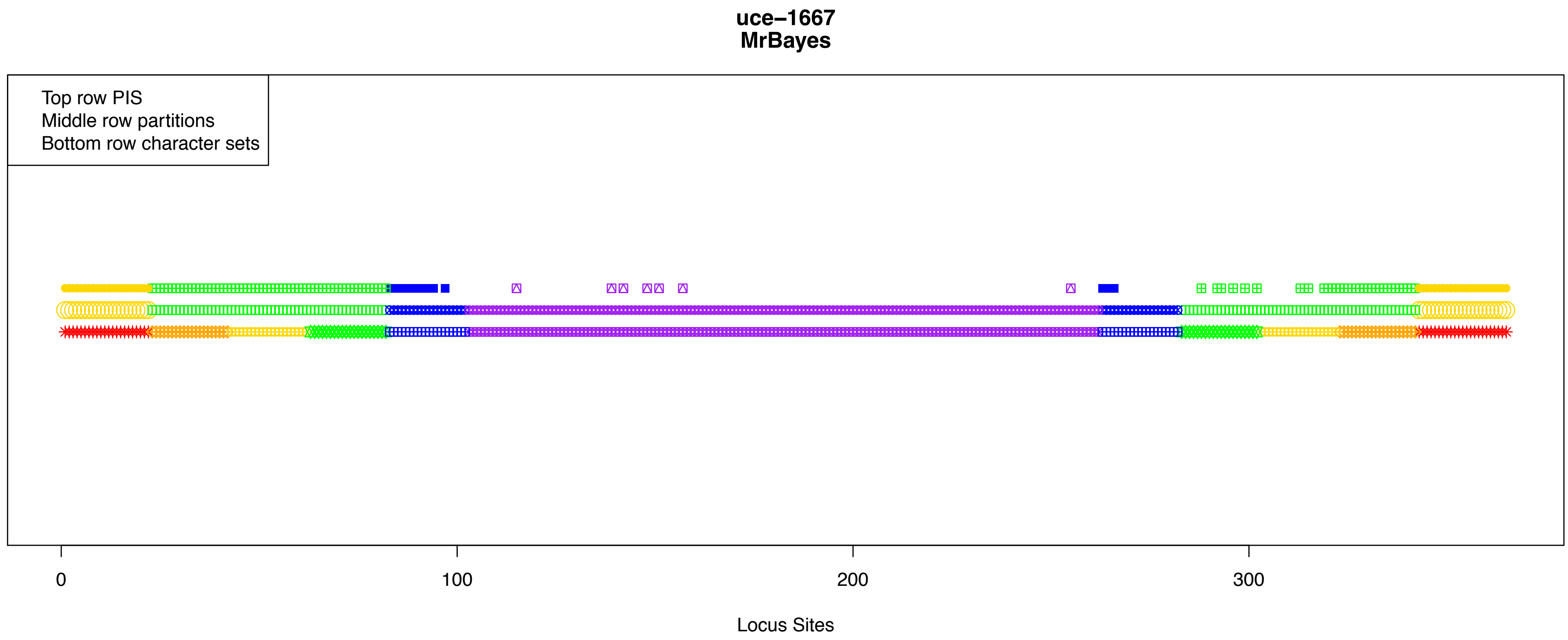
uce–1667
MrBayes
Top row PIS
Middle row partitions
Bottom row character sets
0
100
200
300
Locus Sites

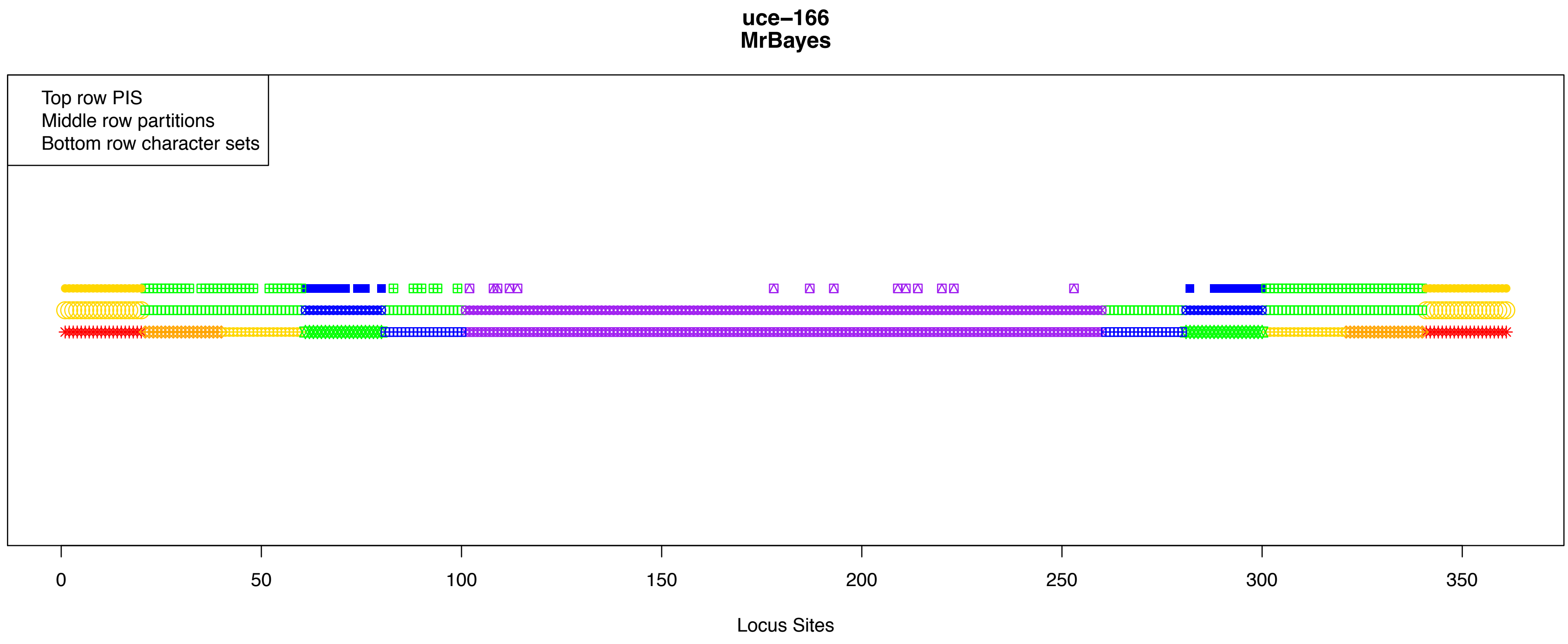
uce–166
MrBayes
Top row PIS
Middle row partitions
Bottom row character sets
0
50
100
150
200
250
300
350
Locus Sites

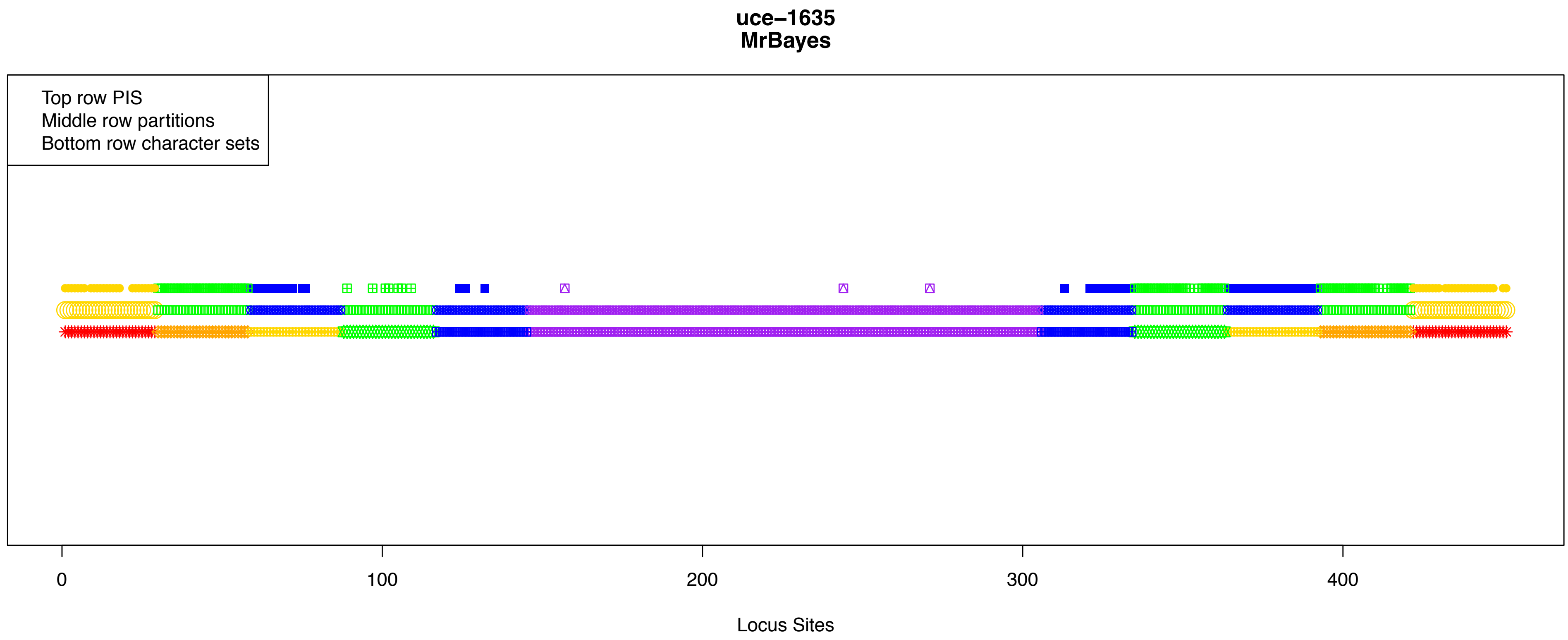

uce−1635
MrBayes
Top row PIS
Middle row partitions
Bottom row character sets
0
100
200
300
400
Locus Sites

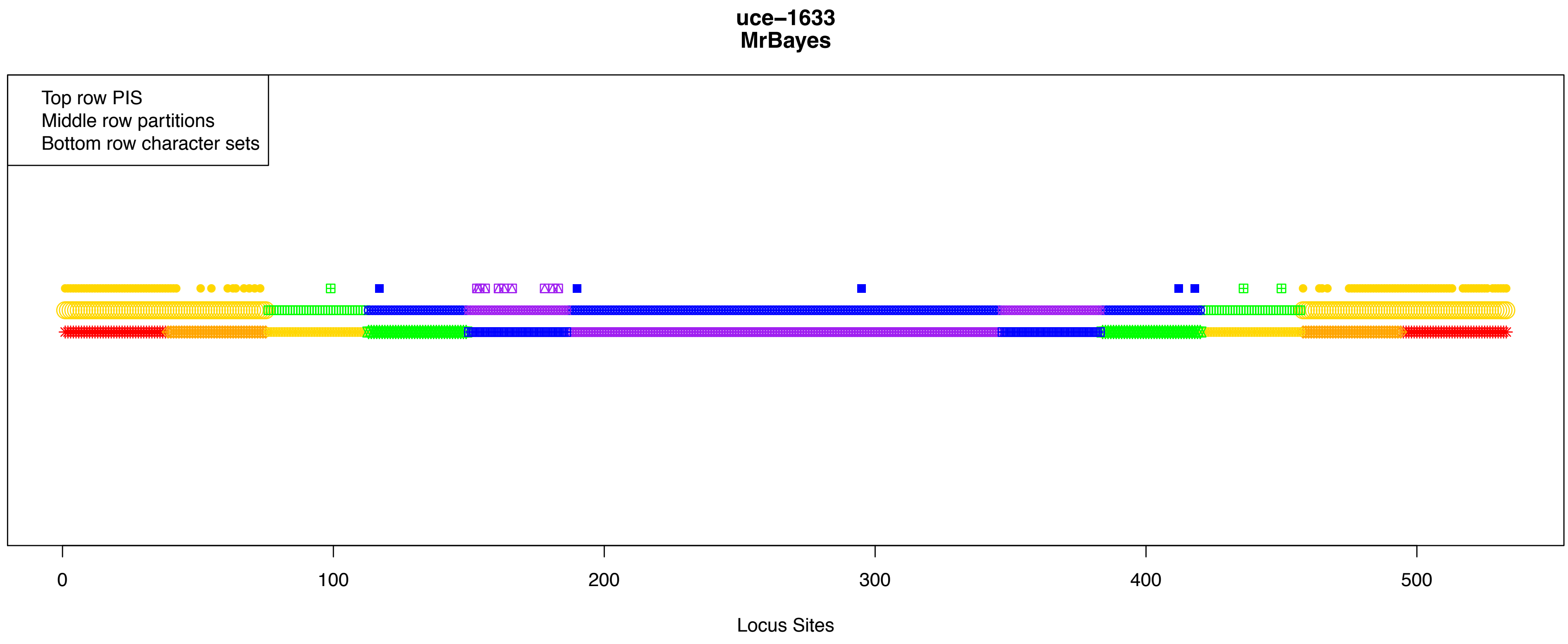

uce–1633
MrBayes
Top row PIS
Middle row partitions
Bottom row character sets
0
100
200
300
400
500
Locus Sites

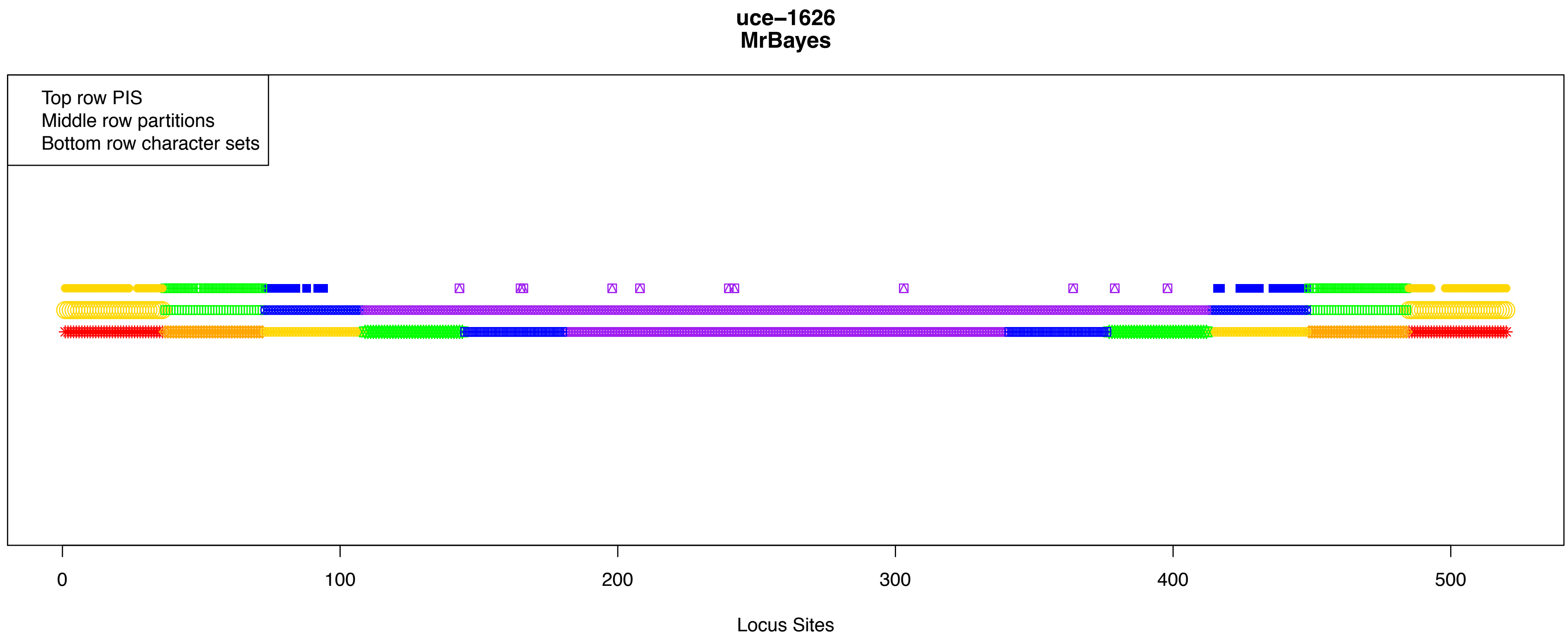
uce–1626
MrBayes
Top row PIS
Middle row partitions
Bottom row character sets
0
100
200
300
400
500
Locus Sites

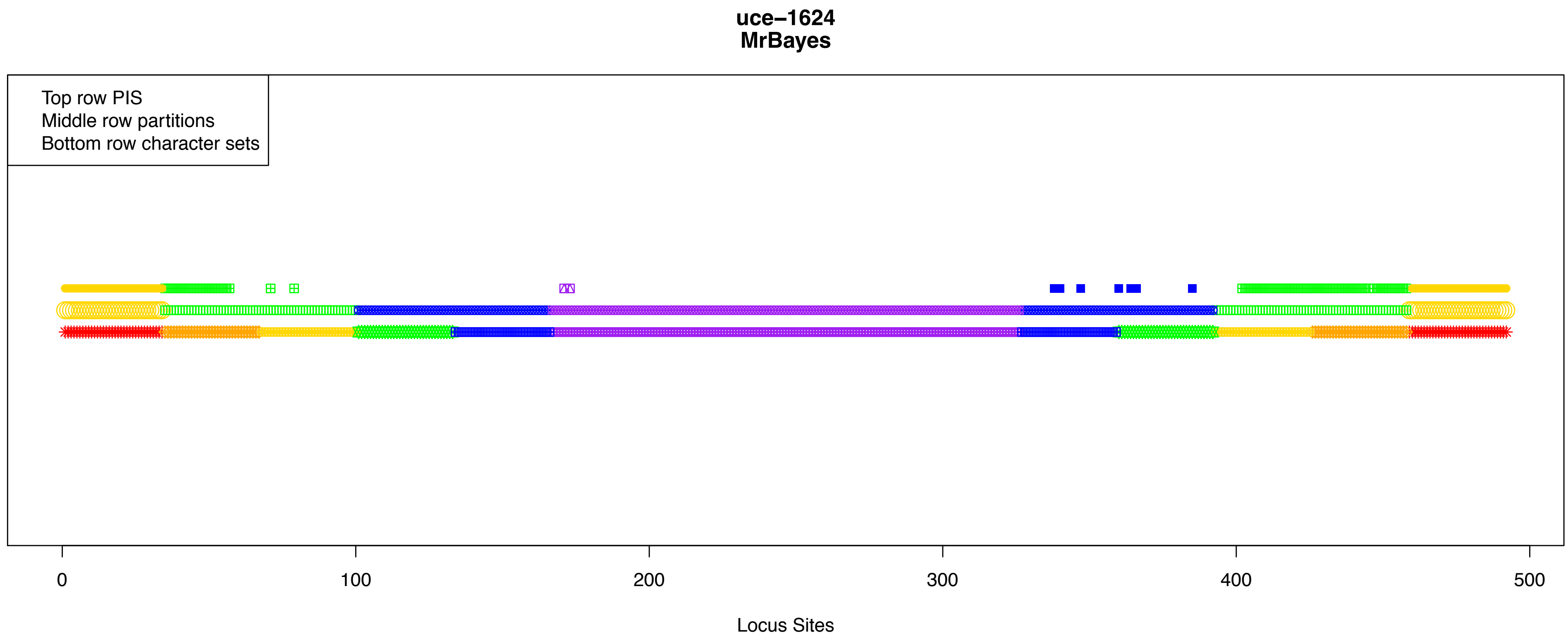
uce–1624
MrBayes
Top row PIS
Middle row partitions
Bottom row character sets
0
100
200
300
400
500
Locus Sites

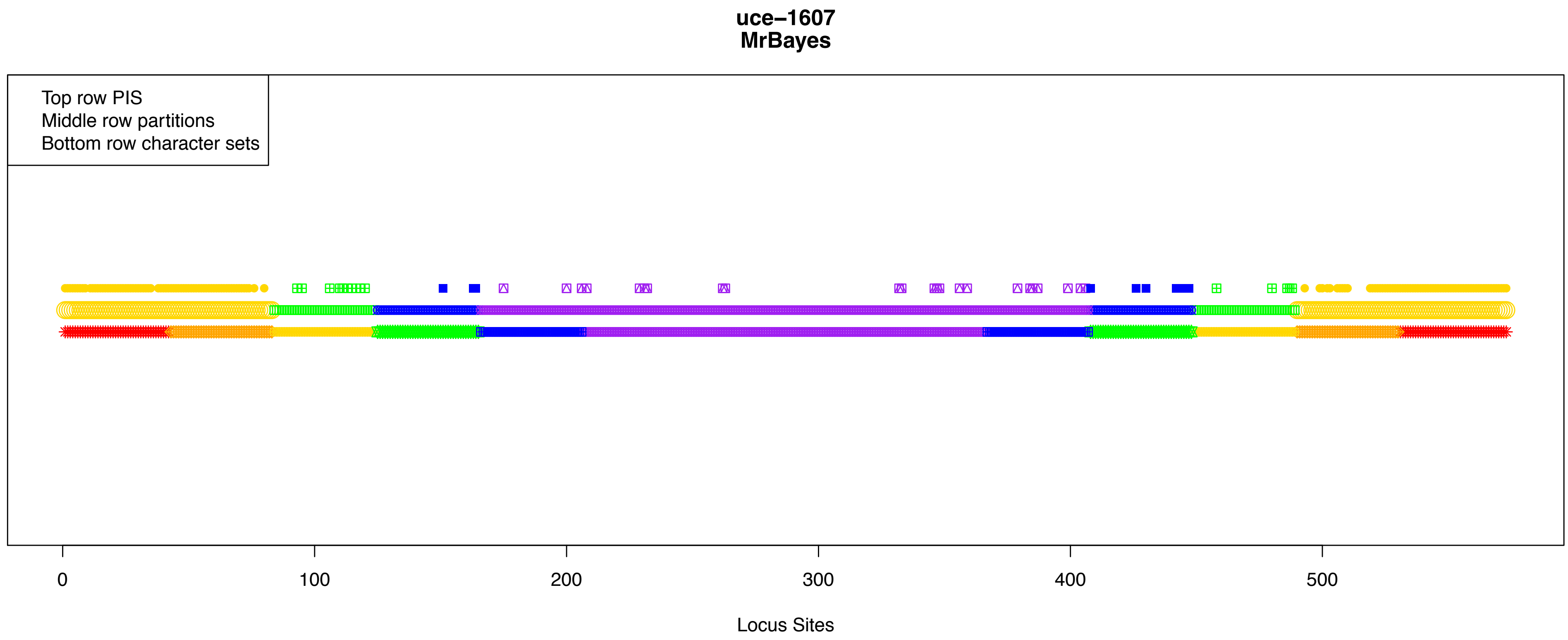

uce−1607
MrBayes
Top row PIS
Middle row partitions
Bottom row character sets
0
100
200
300
400
500
Locus Sites

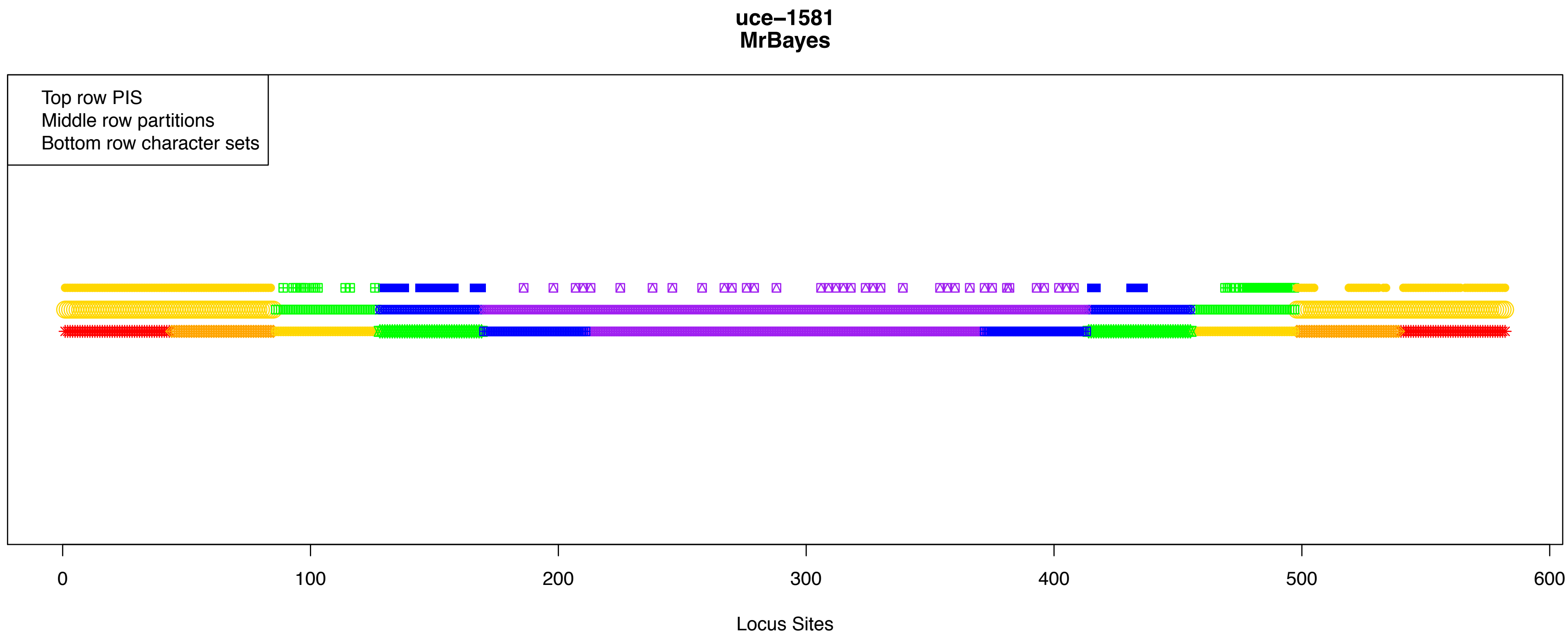
uce–1581
MrBayes
Top row PIS
Middle row partitions
Bottom row character sets
0
100
200
300
400
500
600
Locus Sites

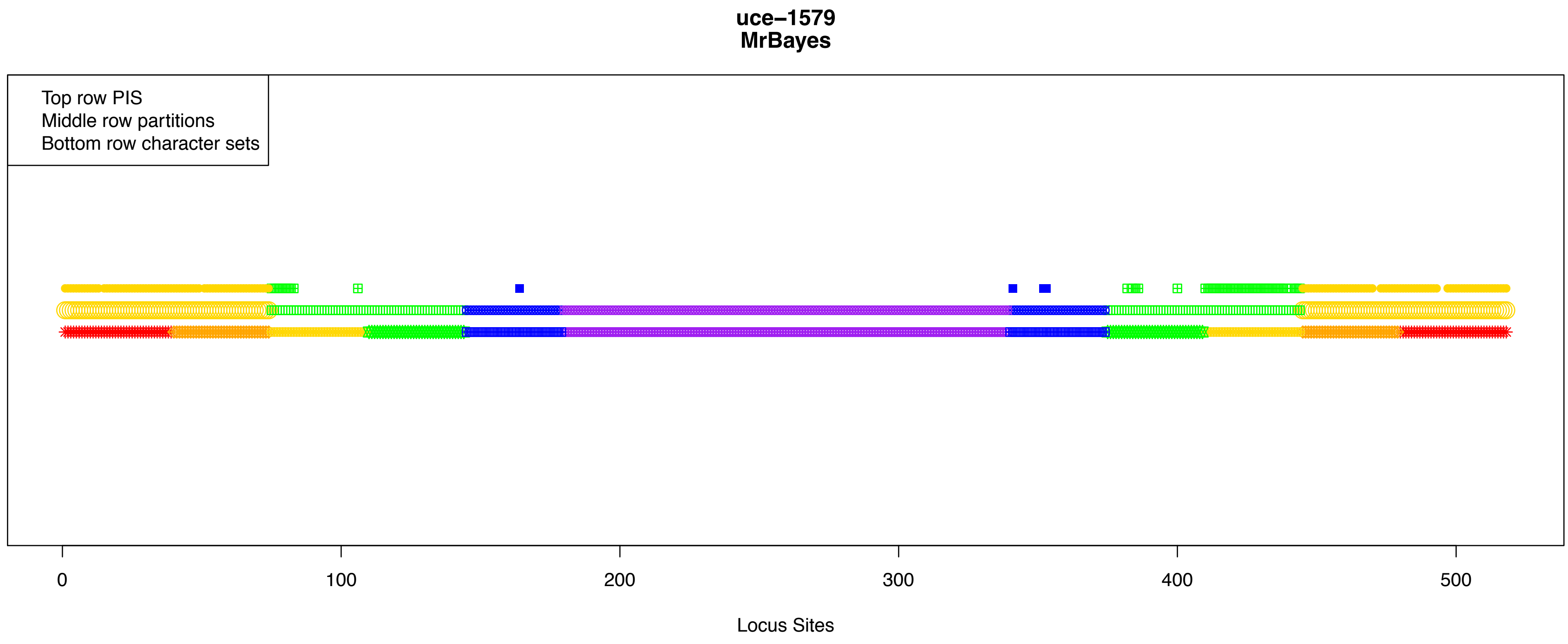

uce–1579
MrBayes
Top row PIS
Middle row partitions
Bottom row character sets
0
100
200
300
400
500
Locus Sites

uce–1569
MrBayes

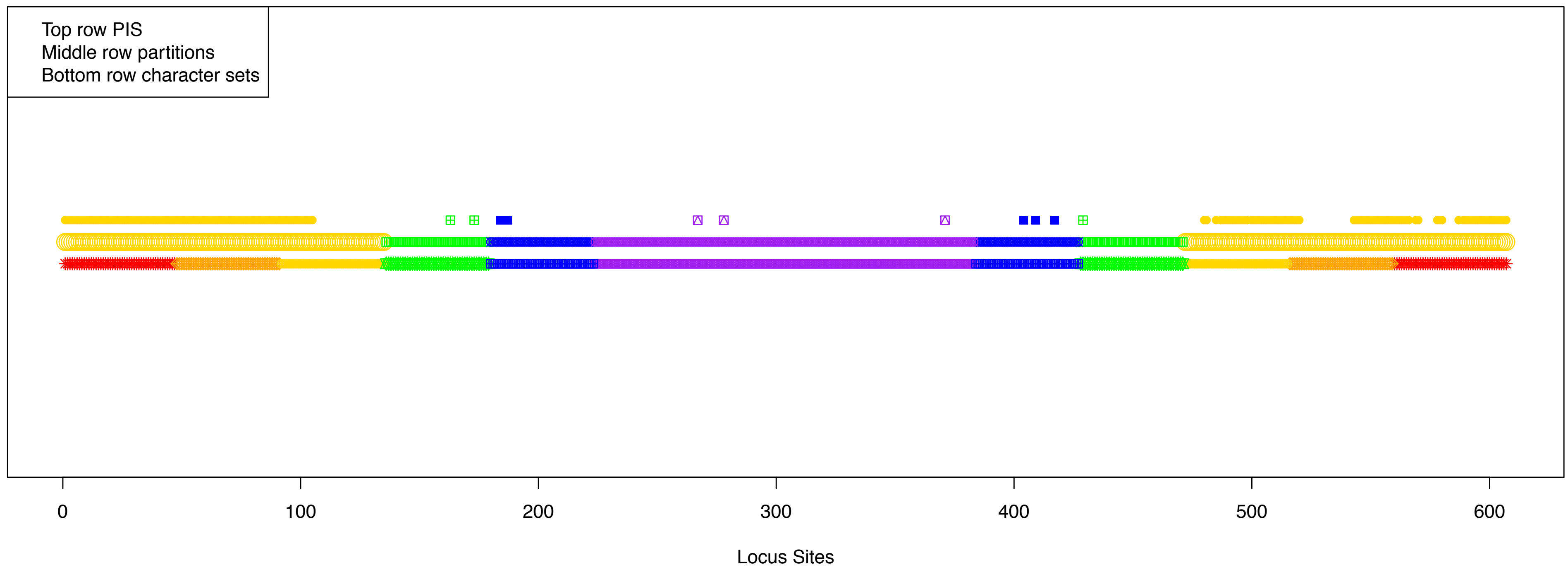

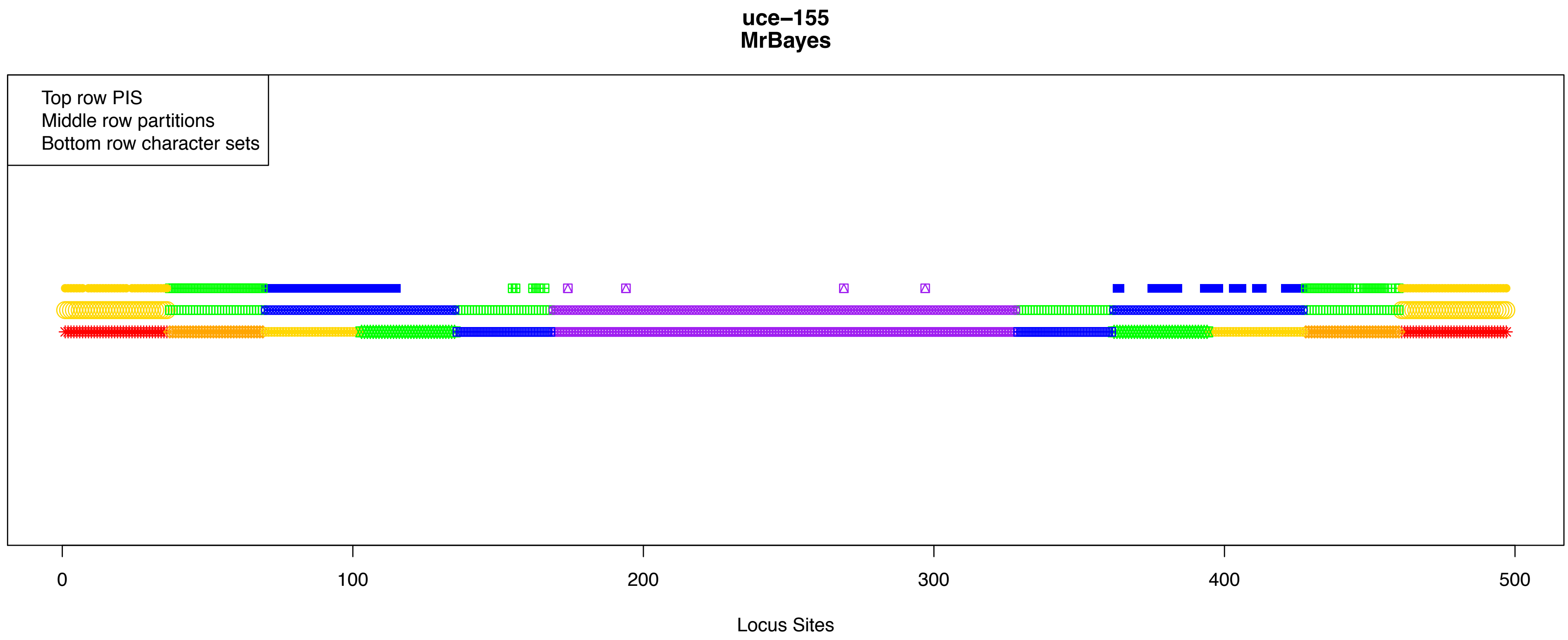

uce–155
MrBayes
Top row PIS
Middle row partitions
Bottom row character sets
0
100
200
300
400
500
Locus Sites

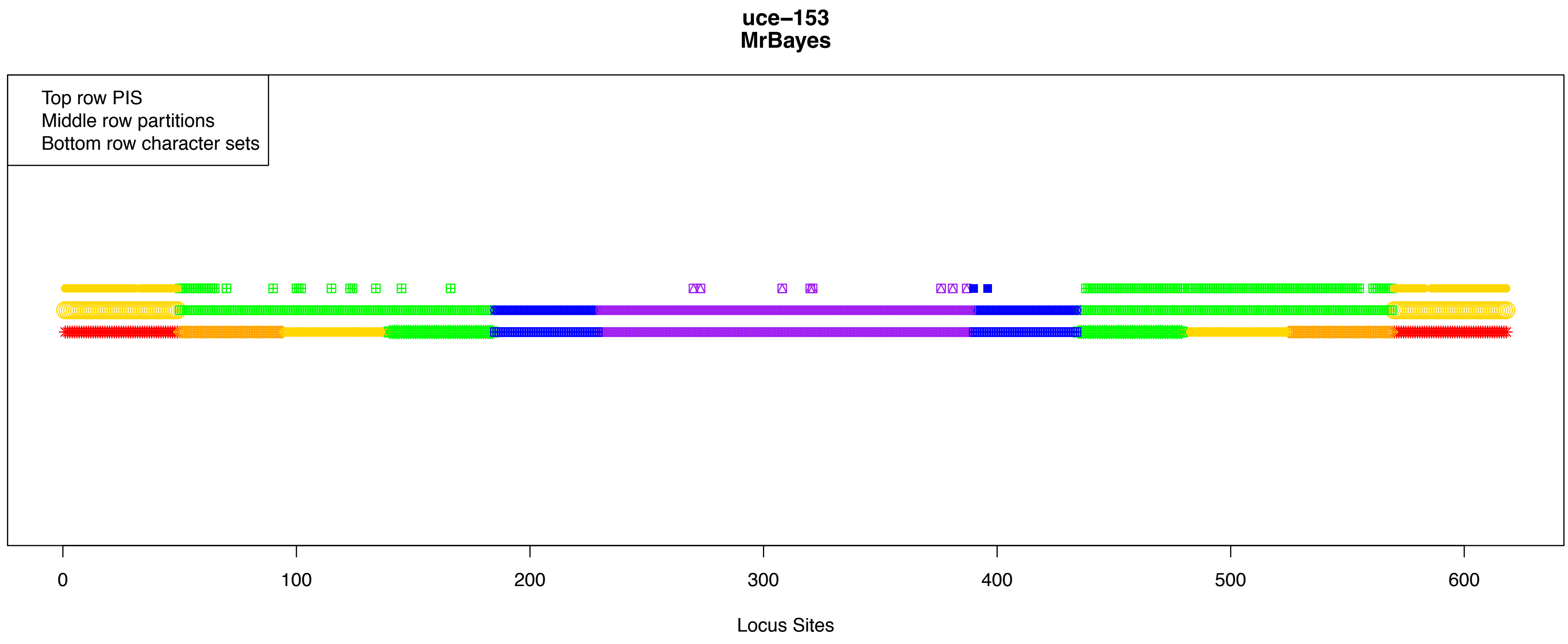
uce–153
MrBayes
Top row PIS
Middle row partitions
Bottom row character sets
0
100
200
300
400
500
600
Locus Sites

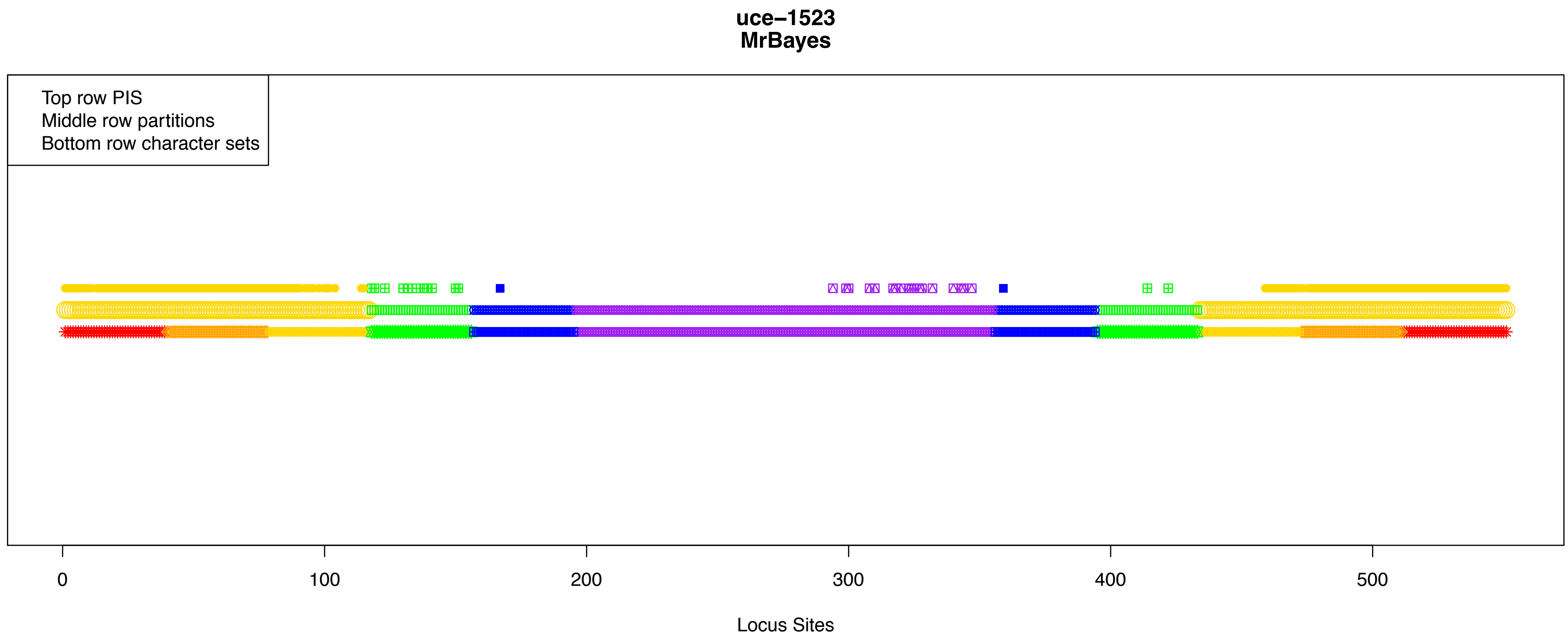
uce–1523
MrBayes
Top row PIS
Middle row partitions
Bottom row character sets
0
100
200
300
400
500
Locus Sites

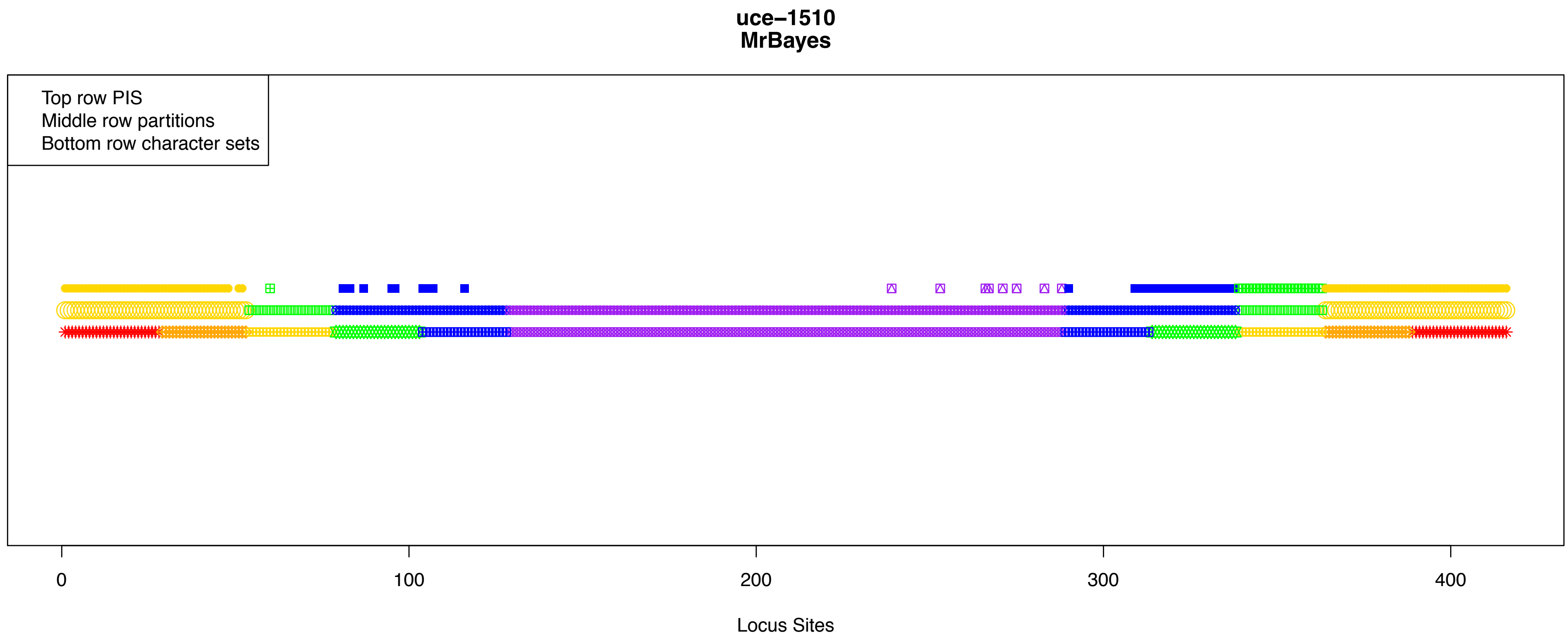

uce–1510
MrBayes
Top row PIS
Middle row partitions
Bottom row character sets
0
100
200
300
400
Locus Sites

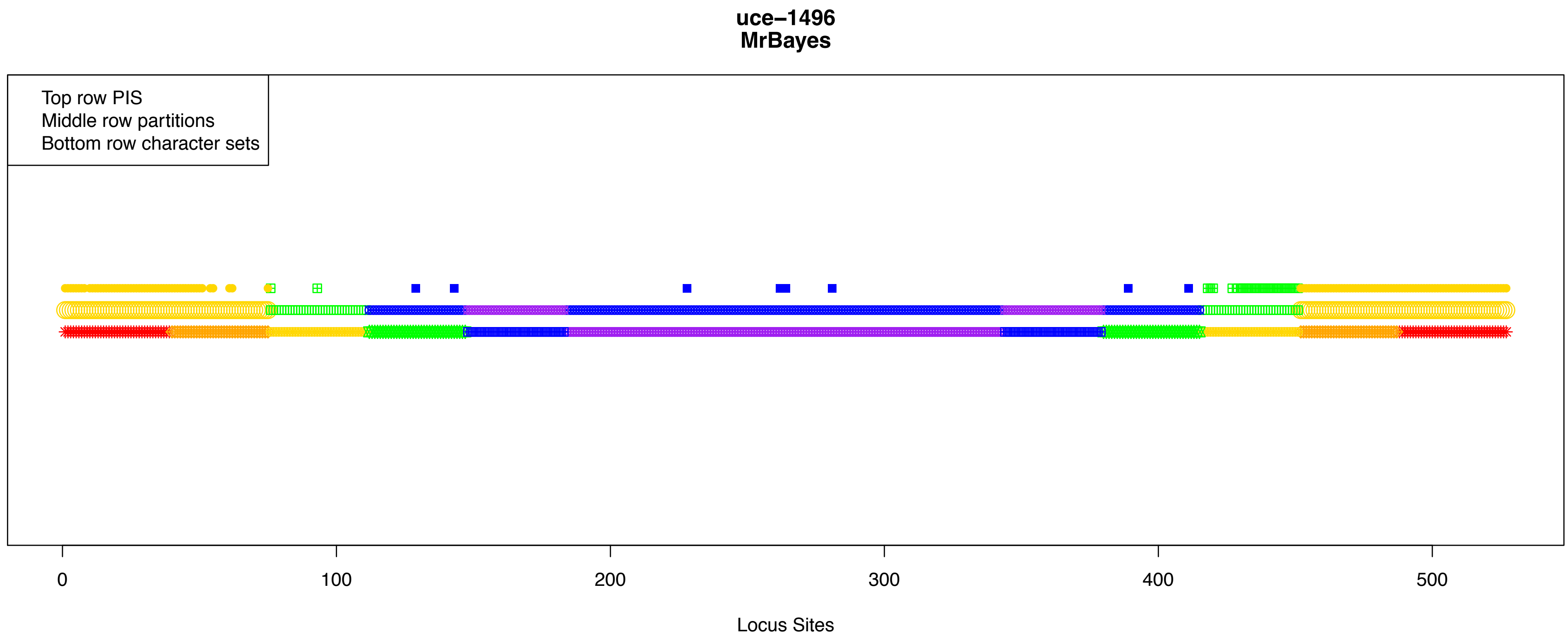
uce–1496
MrBayes
Top row PIS
Middle row partitions
Bottom row character sets
0
100
200
300
400
500
Locus Sites

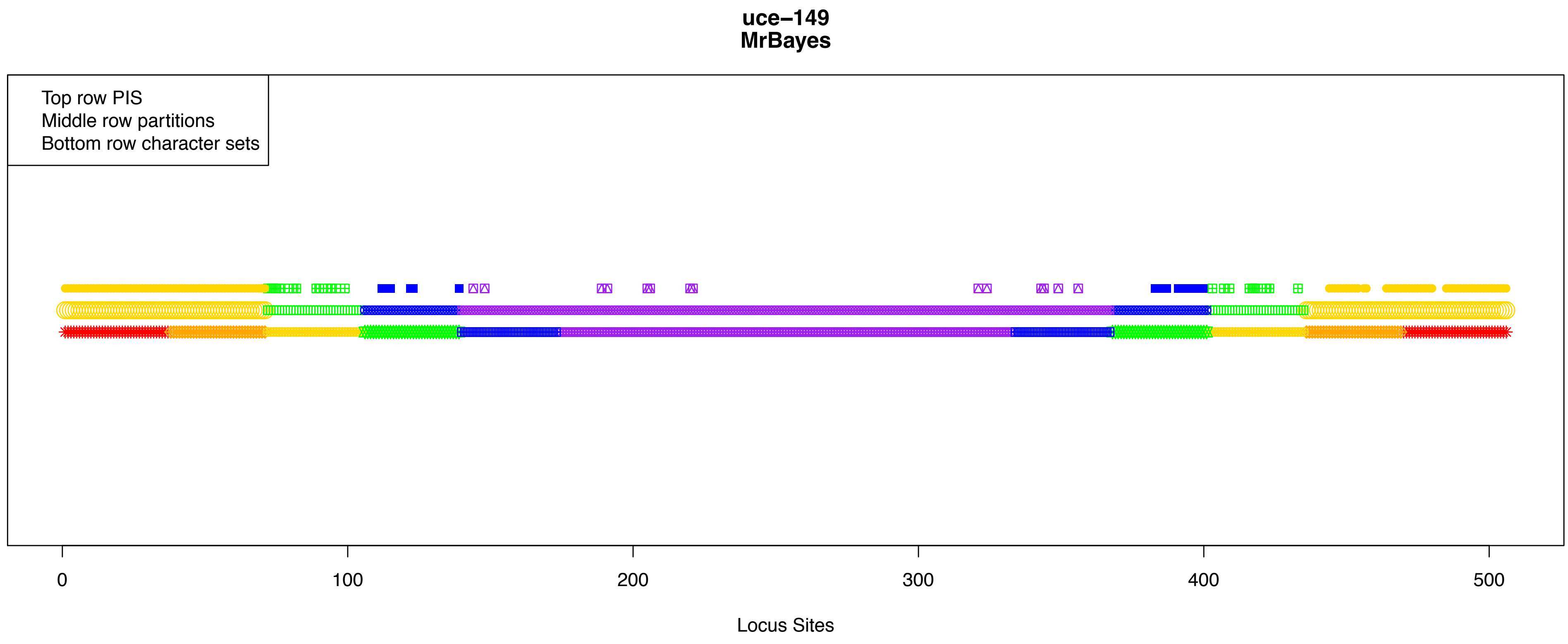

uce–149
MrBayes
Top row PIS
Middle row partitions
Bottom row character sets
0
100
200
300
400
500
Locus Sites

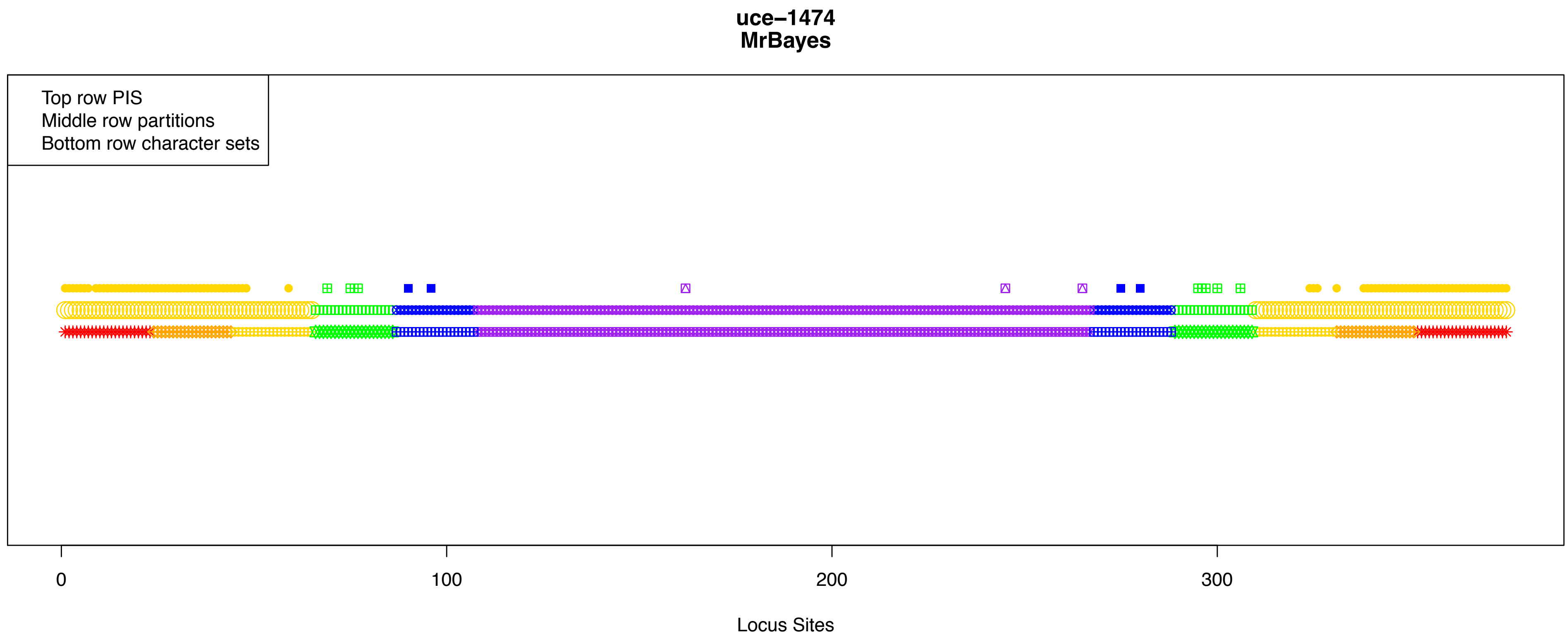
uce–1474
MrBayes
Top row PIS
Middle row partitions
Bottom row character sets
0
100
200
300
Locus Sites

uce–147
MrBayes

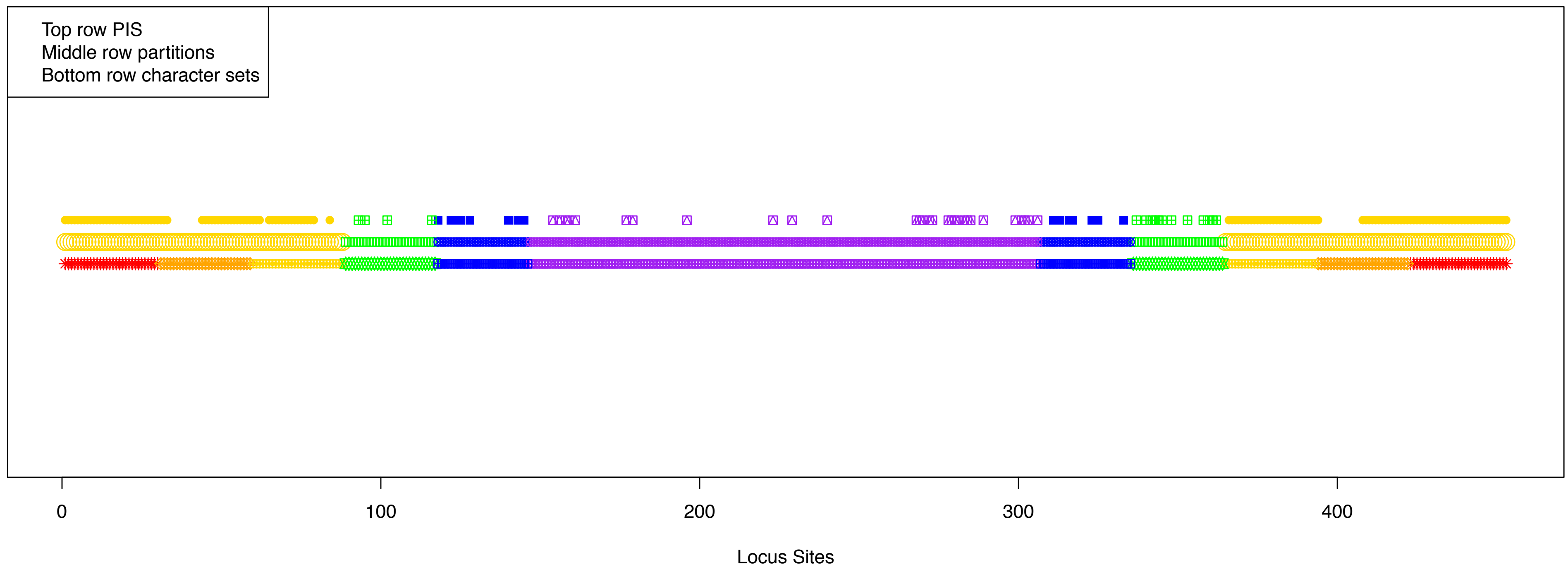

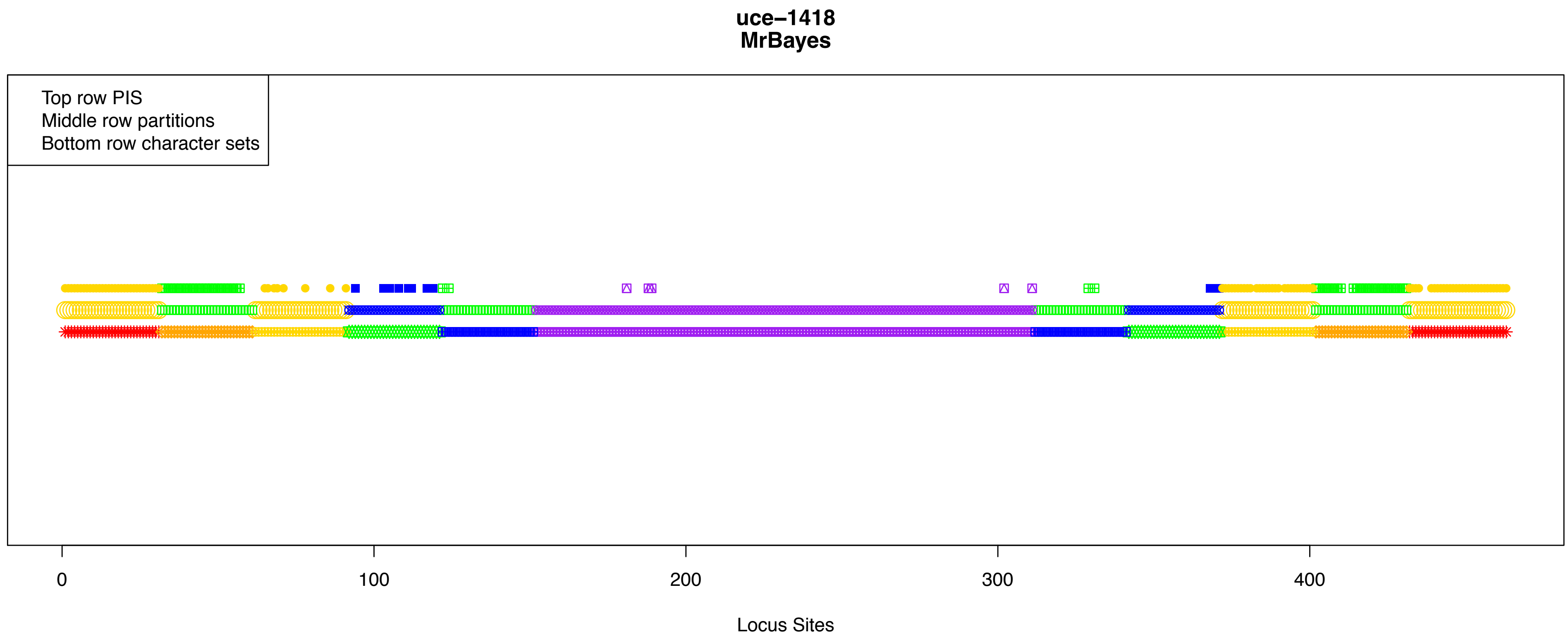
uce–1418
MrBayes
Top row PIS
Middle row partitions
Bottom row character sets
0
100
200
300
400
Locus Sites

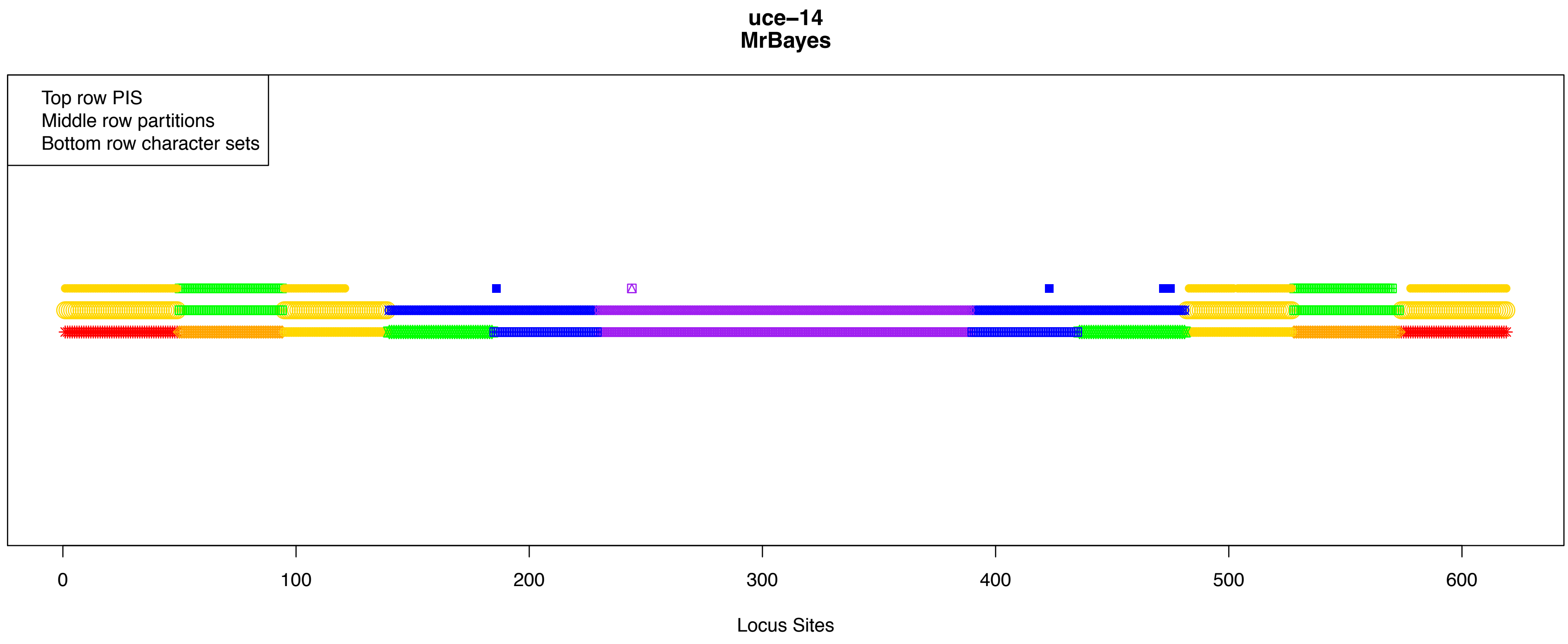

uce–14
MrBayes
Top row PIS
Middle row partitions
Bottom row character sets
0
100
200
300
400
500
600
Locus Sites

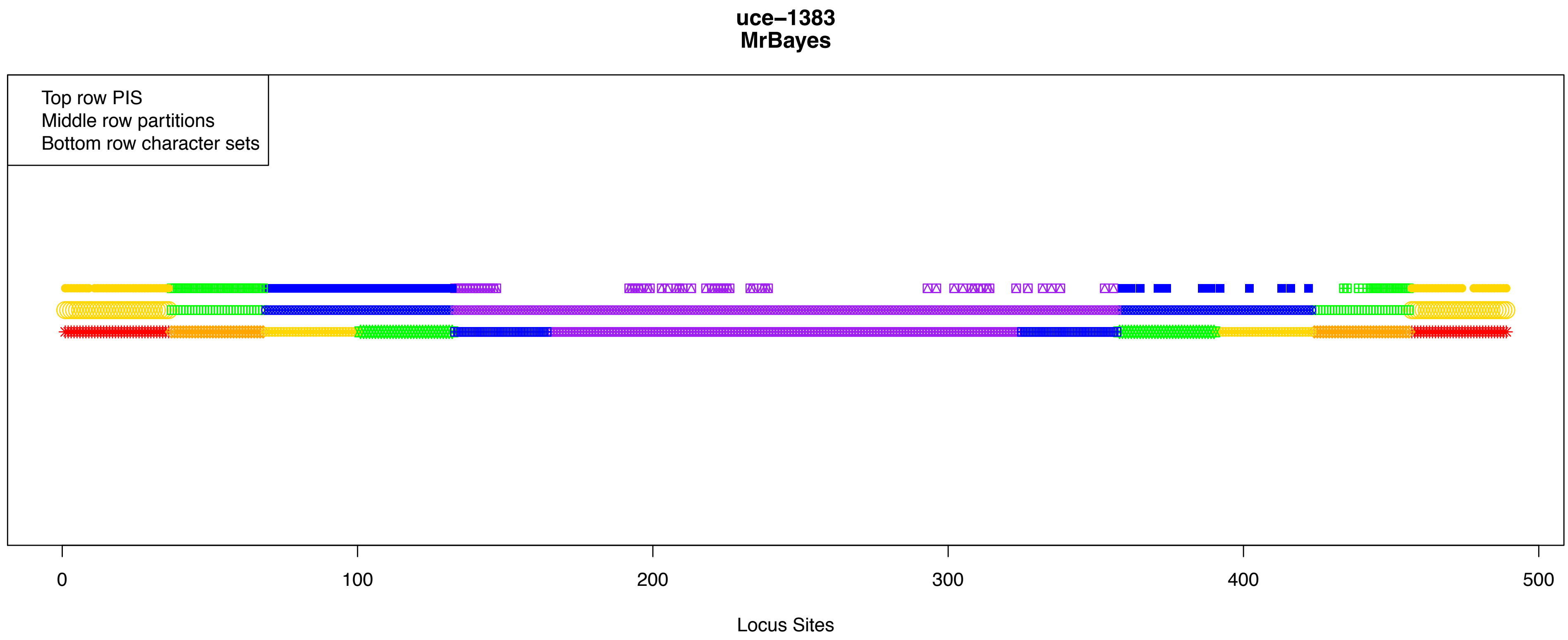

uce–1383
MrBayes
Top row PIS
Middle row partitions
Bottom row character sets
0
100
200
300
400
500
Locus Sites

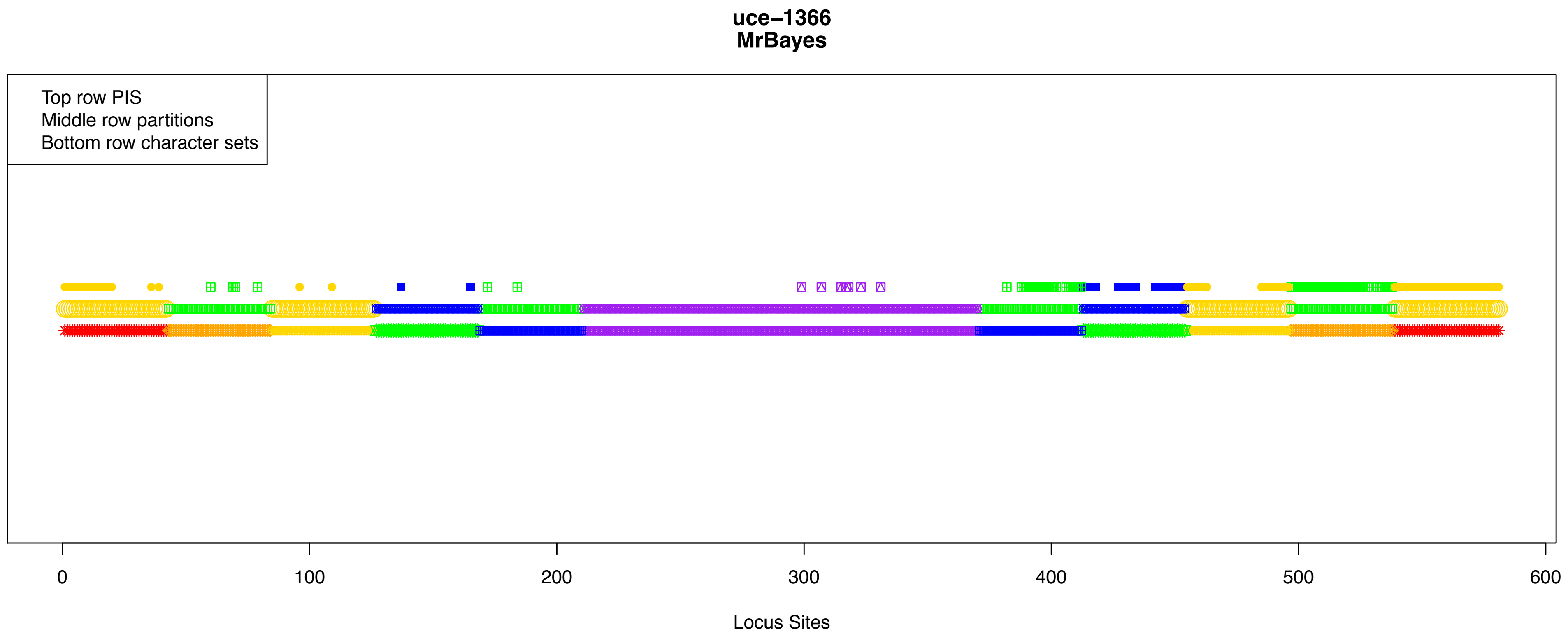
uce–1366
MrBayes
Top row PIS
Middle row partitions
Bottom row character sets
0
100
200
300
400
500
600
Locus Sites

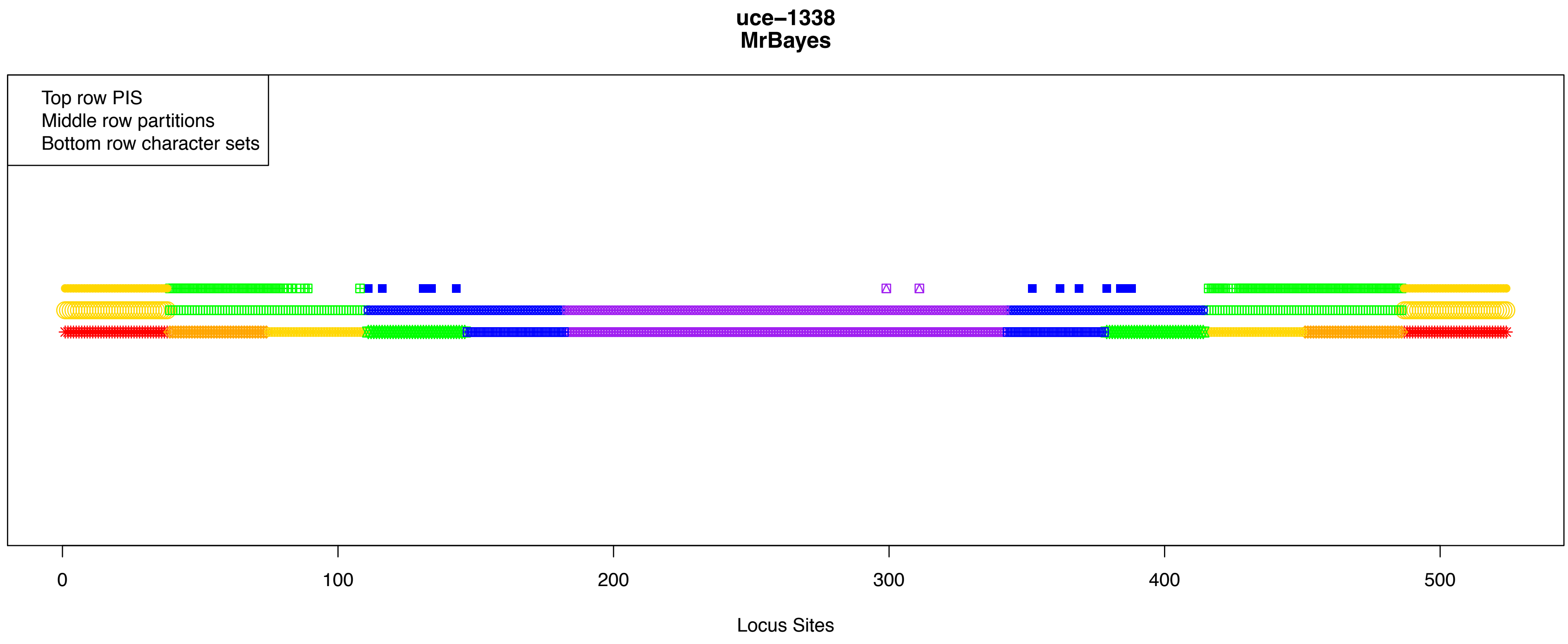
uce–1338
MrBayes
Top row PIS
Middle row partitions
Bottom row character sets
0
100
200
300
400
500
Locus Sites

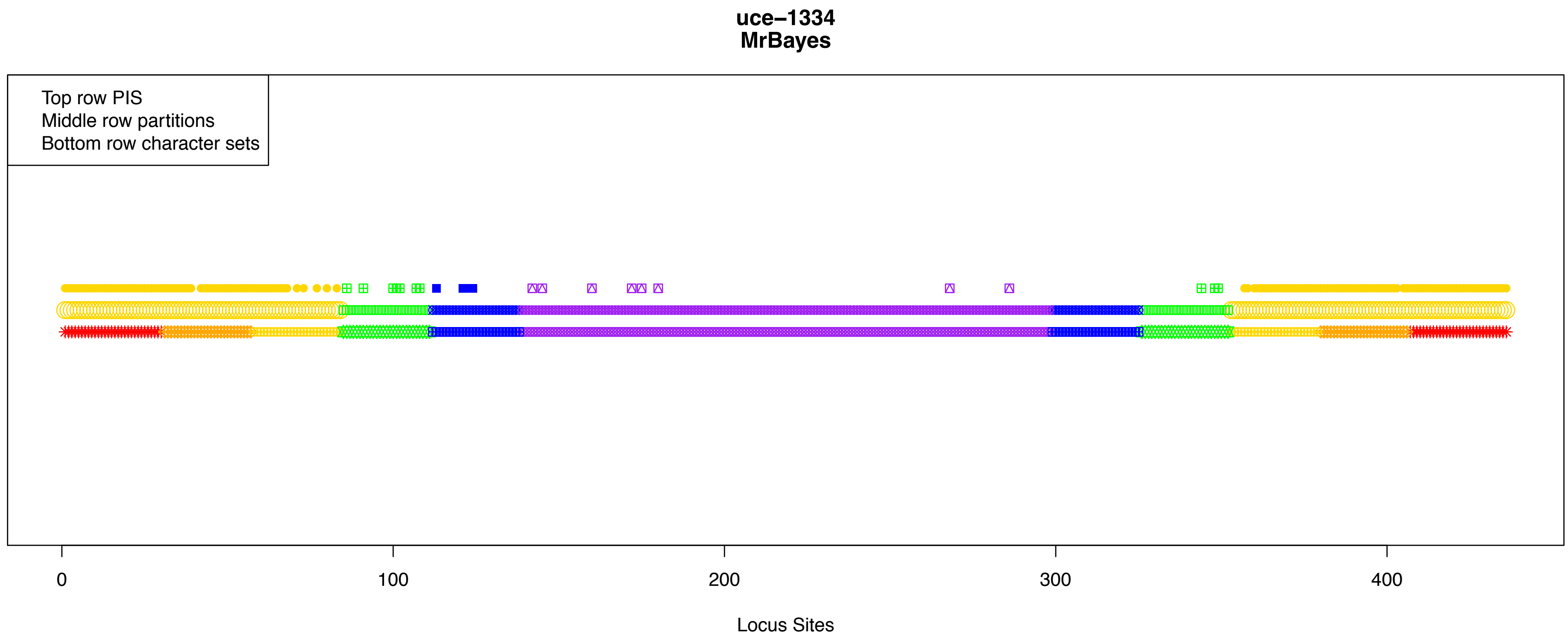
uce–1334
MrBayes
Top row PIS
Middle row partitions
Bottom row character sets
0
100
200
300
400
Locus Sites

uce–1333
MrBayes

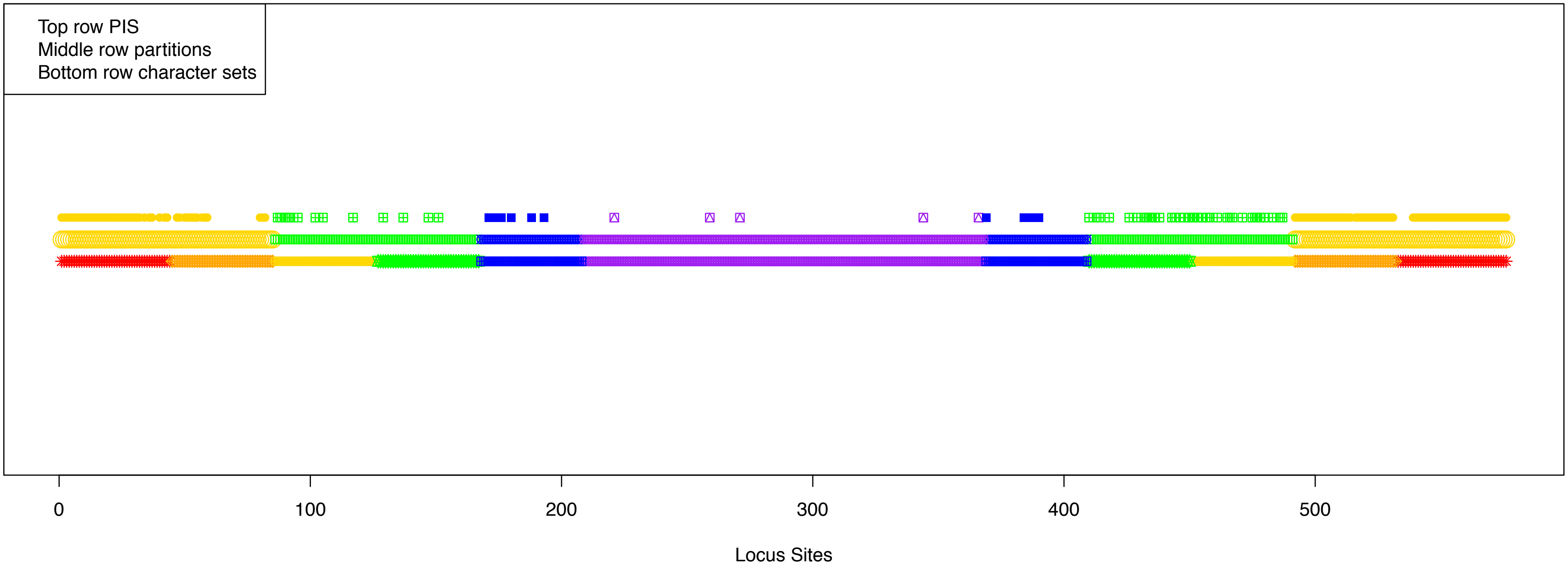

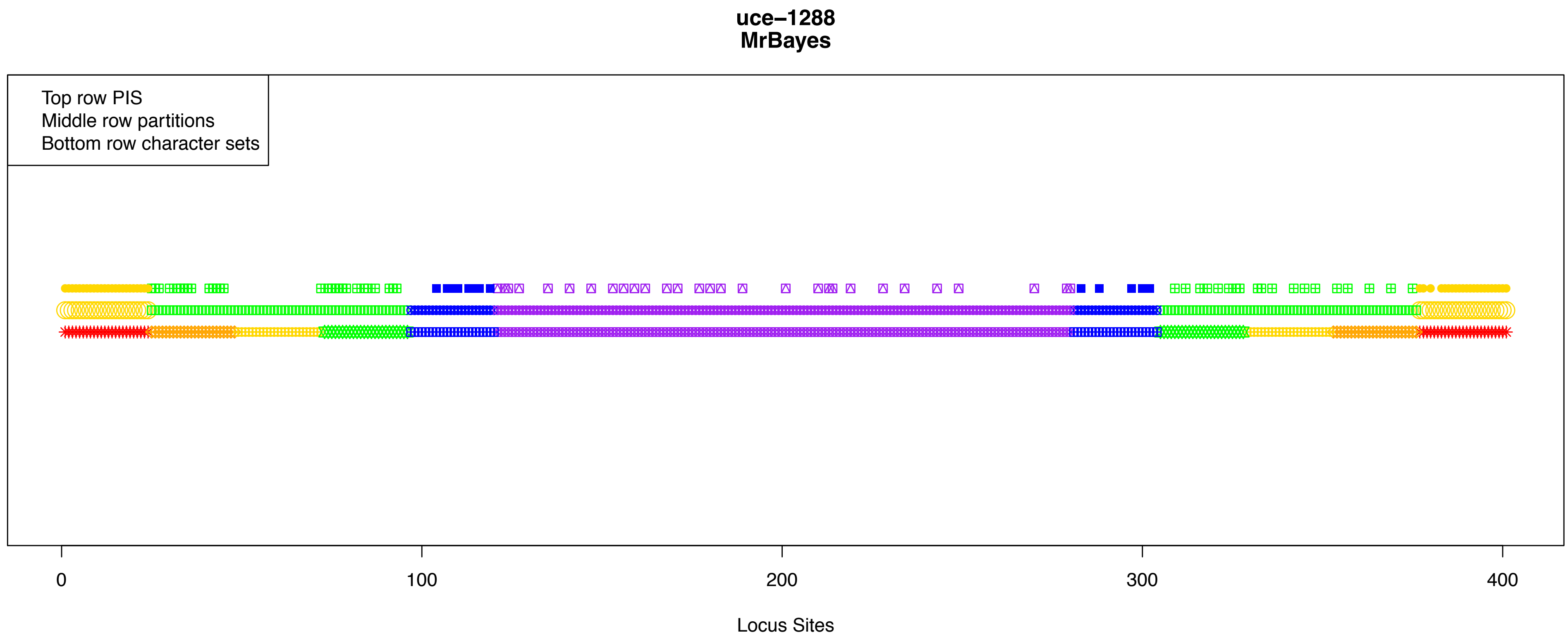
uce–1288
MrBayes
Top row PIS
Middle row partitions
Bottom row character sets
0
100
200
300
400
Locus Sites

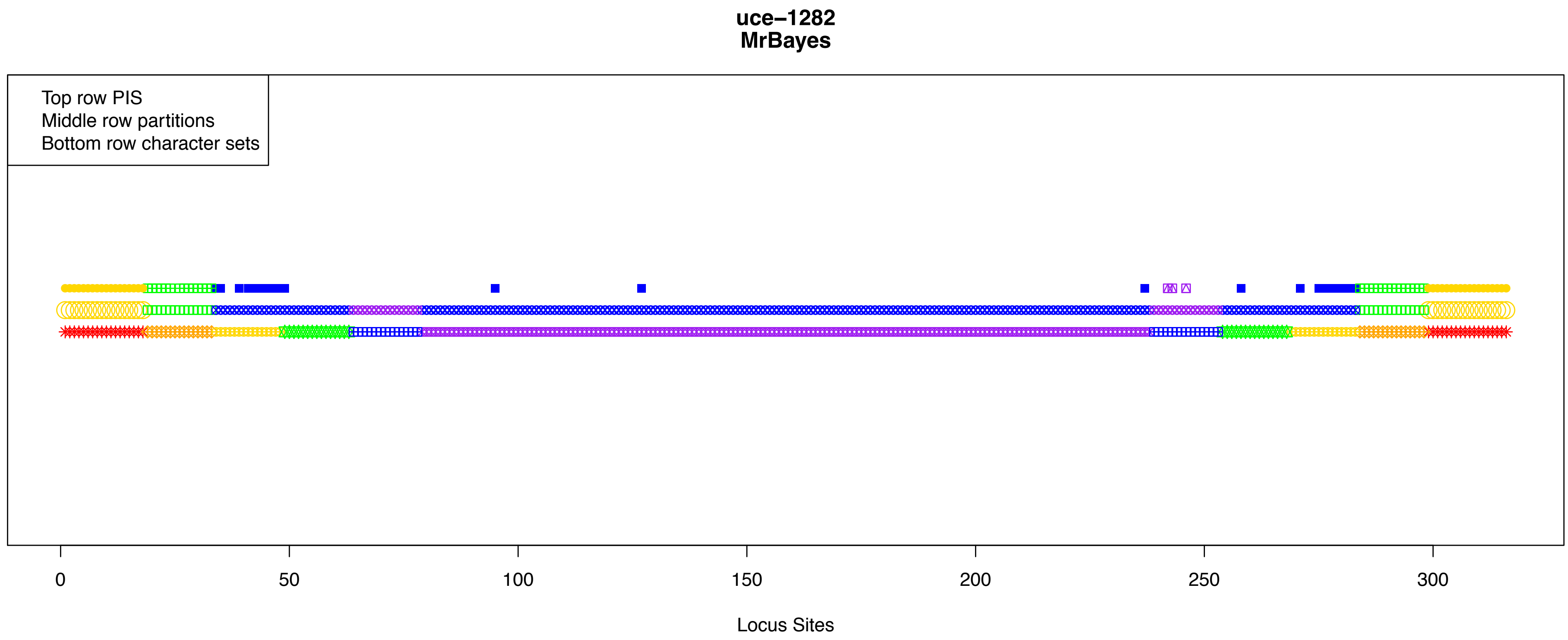
uce–1282
MrBayes
Top row PIS
Middle row partitions
Bottom row character sets
0
50
100
150
200
250
300
Locus Sites

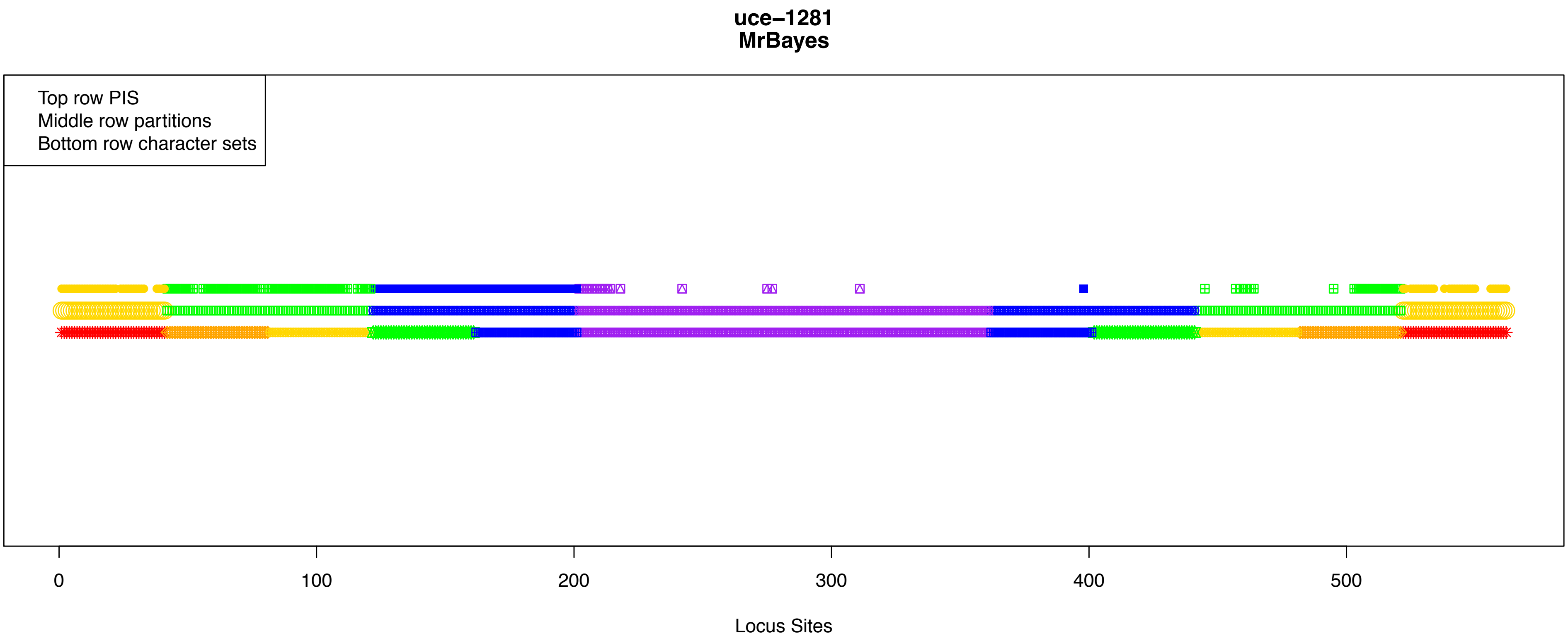
uce–1281
MrBayes
Top row PIS
Middle row partitions
Bottom row character sets
0
100
200
300
400
500
Locus Sites

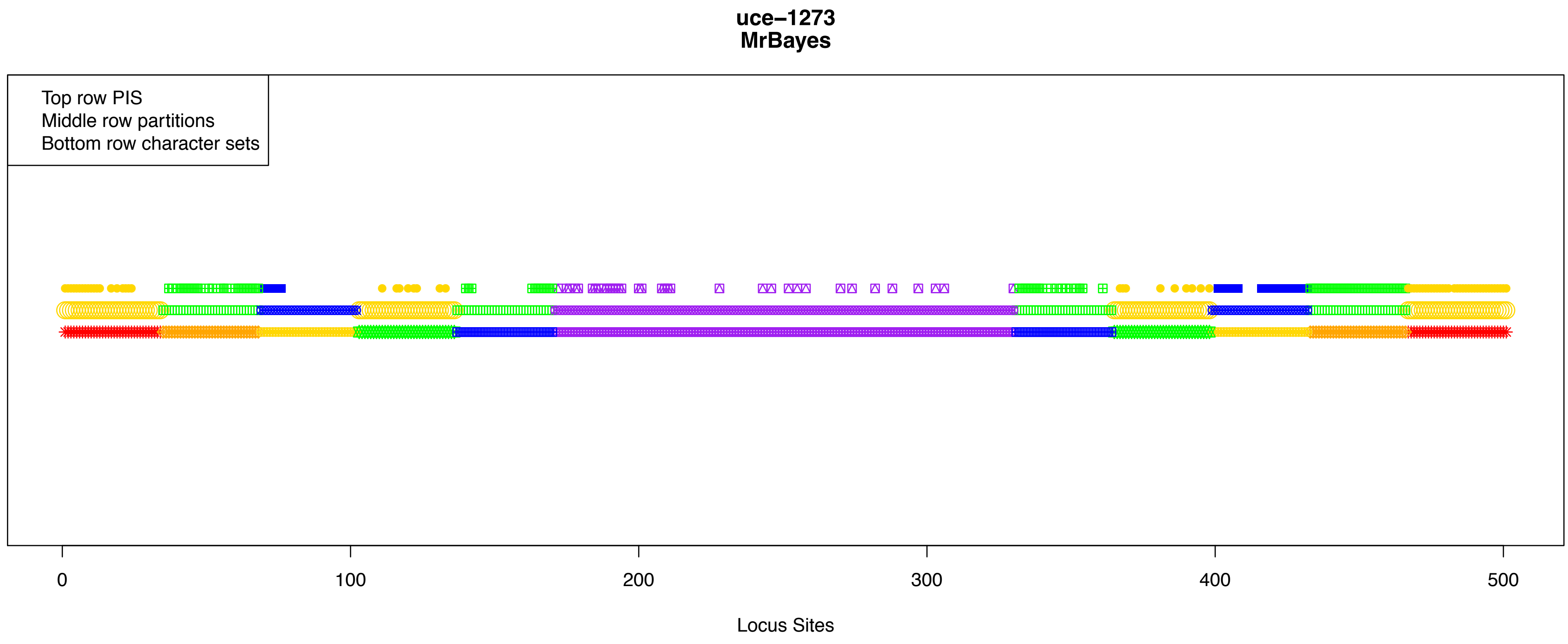
uce–1273
MrBayes
Top row PIS
Middle row partitions
Bottom row character sets
0
100
200
300
400
500
Locus Sites

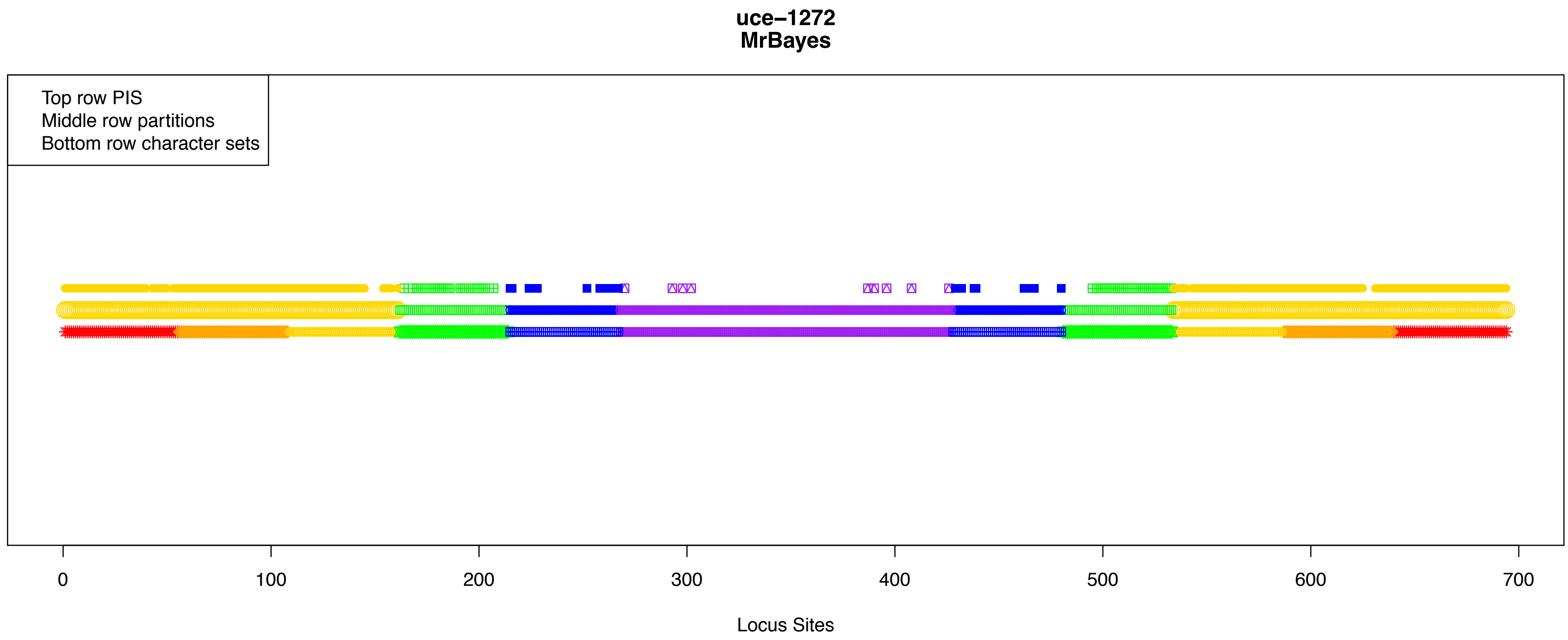

uce–1272
MrBayes
Top row PIS
Middle row partitions
Bottom row character sets
0
100
200
300
400
500
600
700
Locus Sites

uce–1271
MrBayes

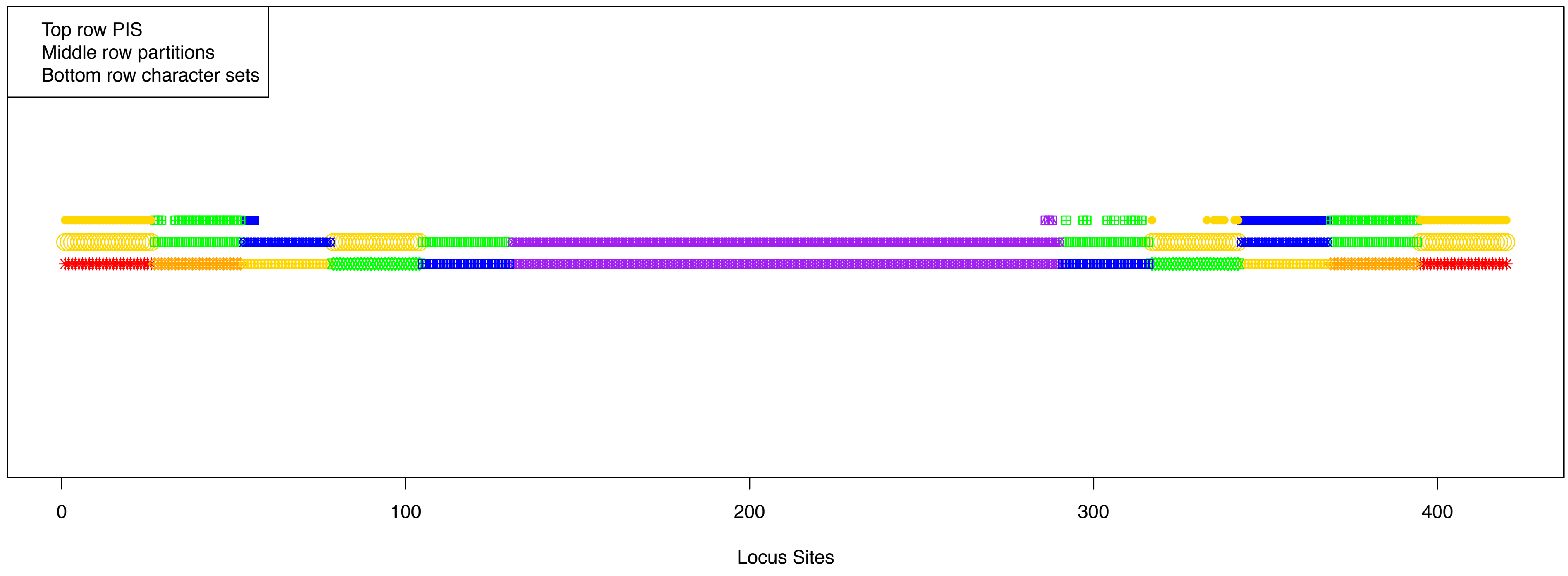

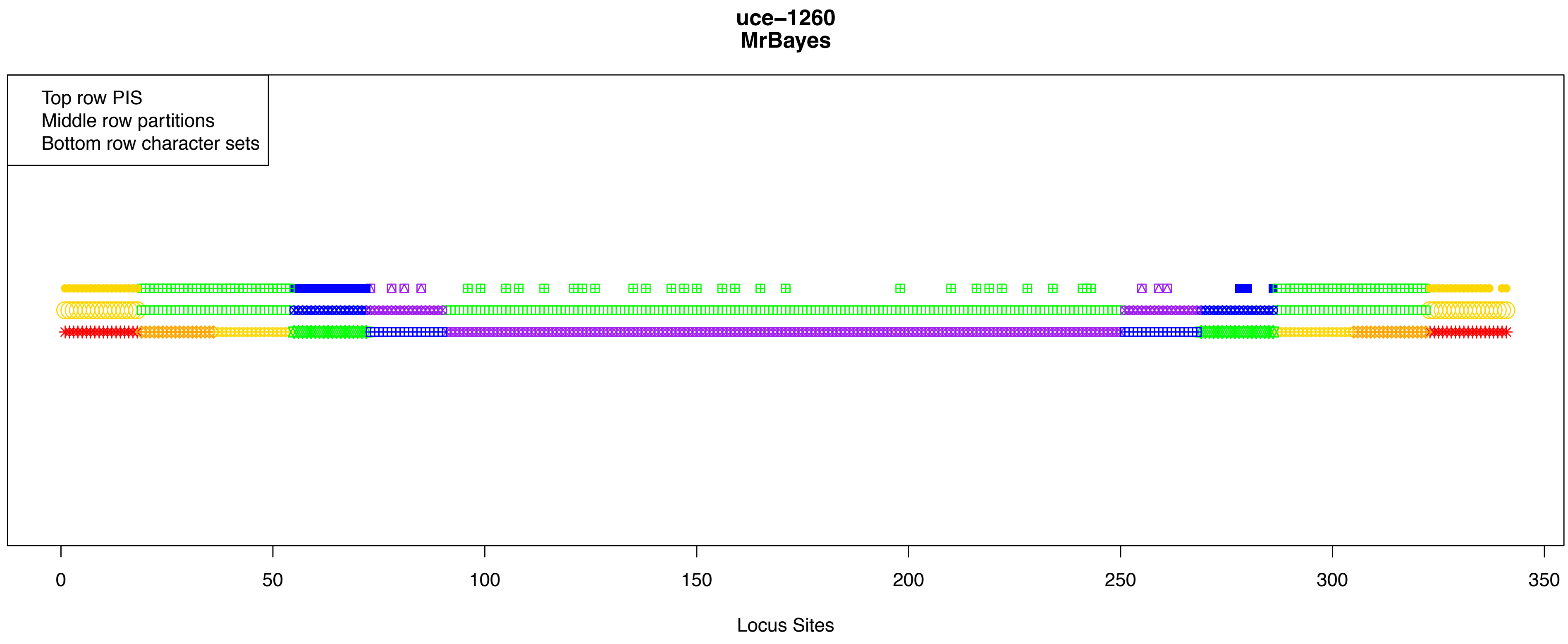
uce–1260
MrBayes
Top row PIS
Middle row partitions
Bottom row character sets
0
50
100
150
200
250
300
350
Locus Sites

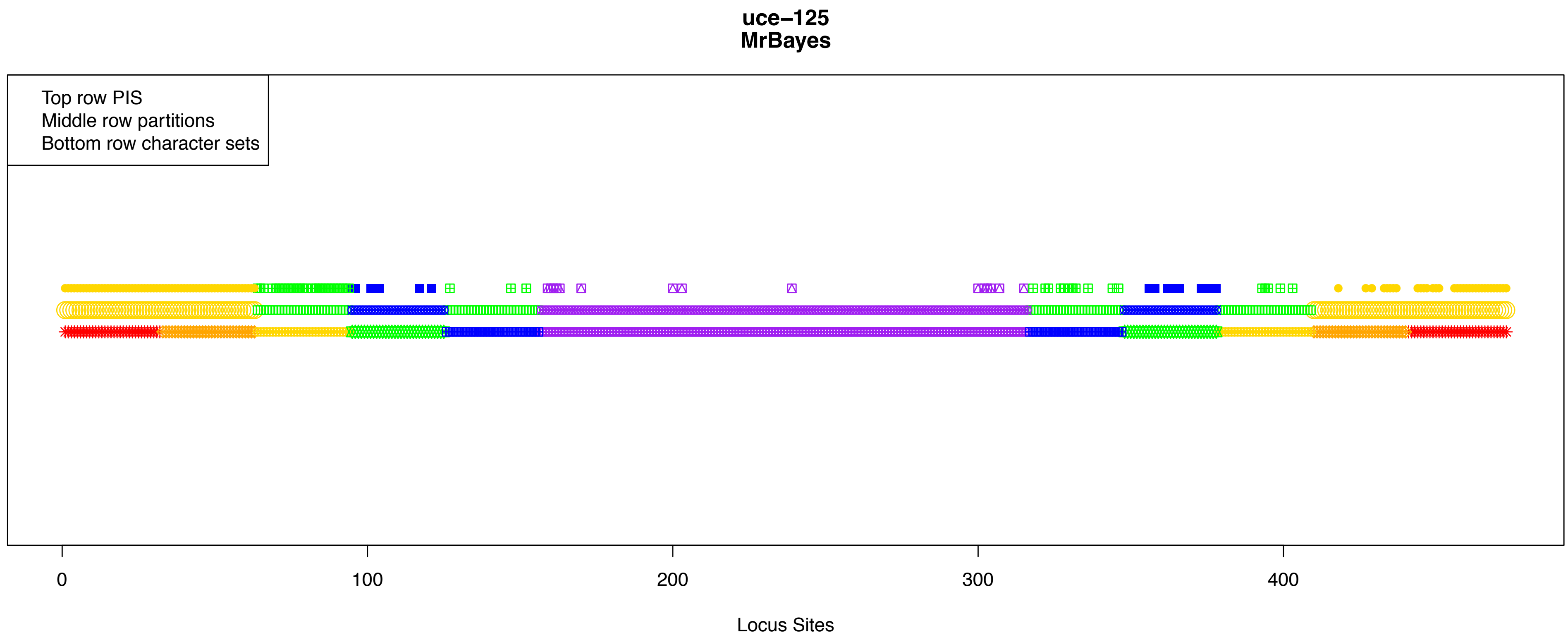

uce–125
MrBayes
Top row PIS
Middle row partitions
Bottom row character sets
0
100
200
300
400
Locus Sites

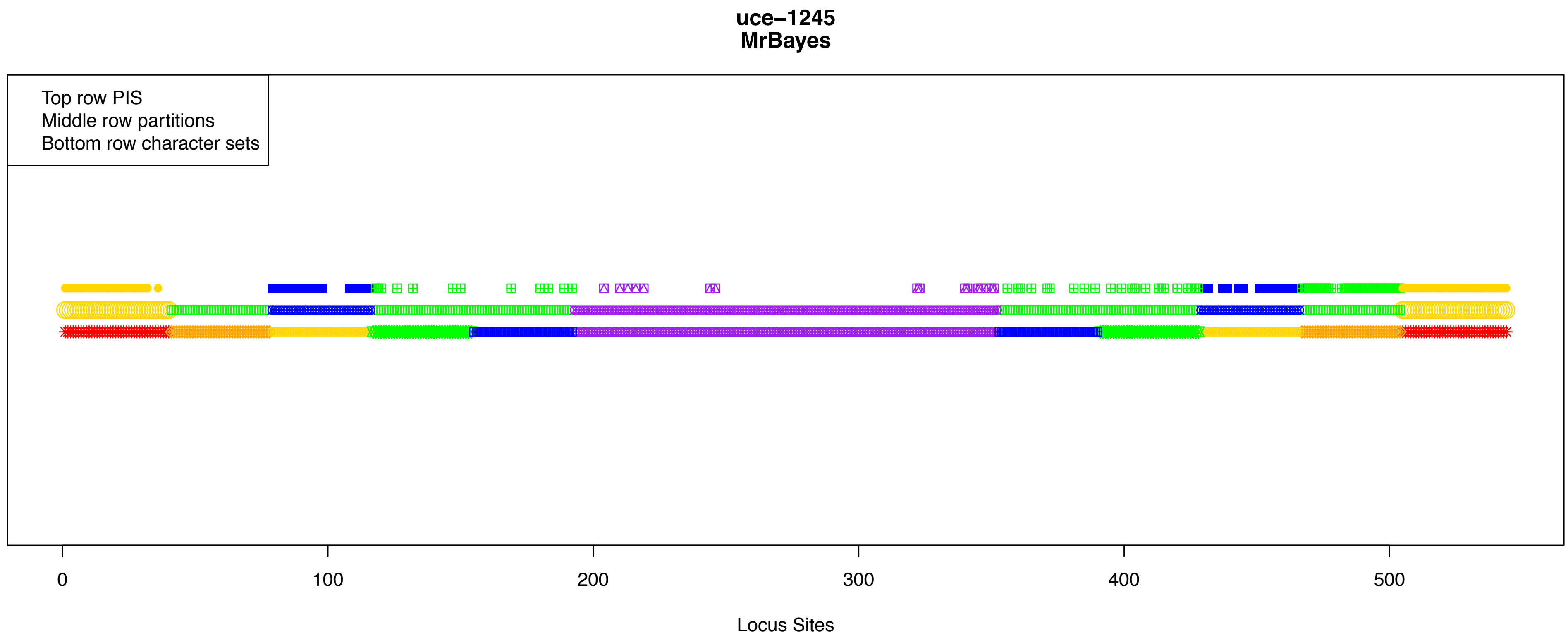

uce–1245
MrBayes
Top row PIS
Middle row partitions
Bottom row character sets
0
100
200
300
400
500
Locus Sites

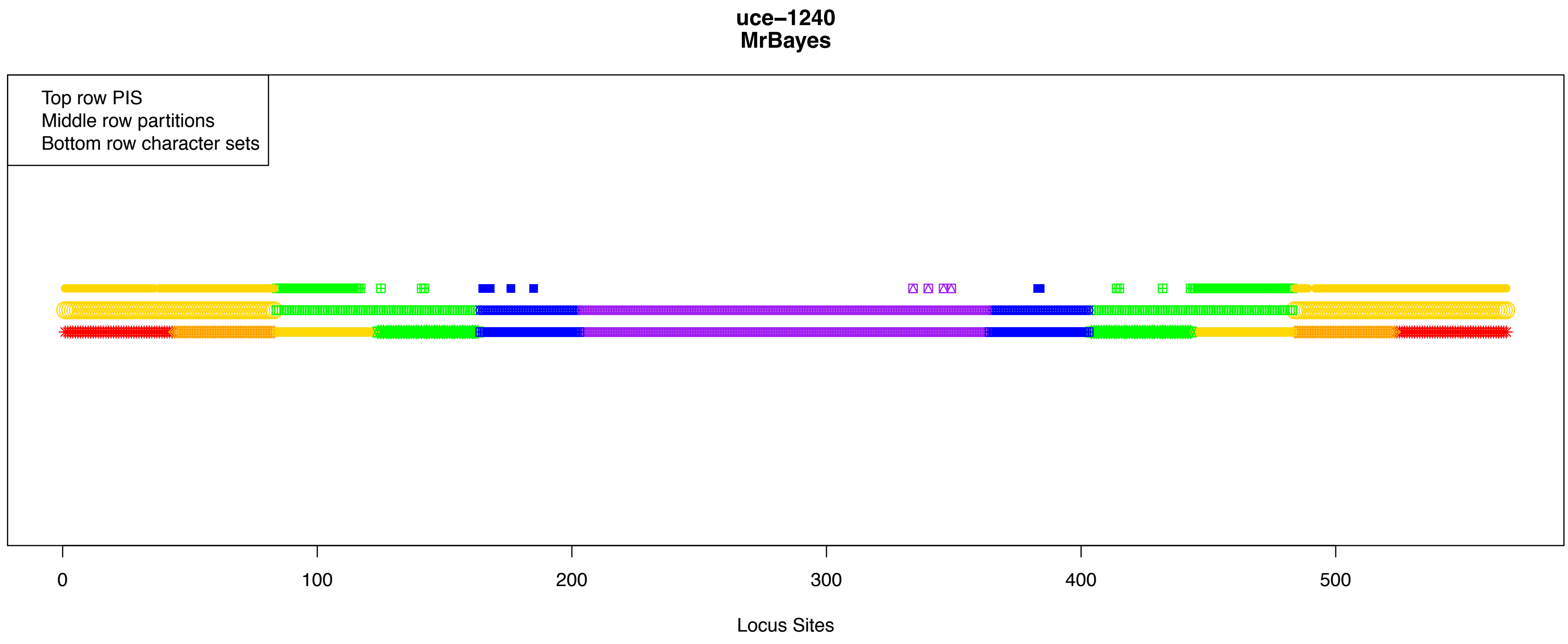
uce–1240
MrBayes
Top row PIS
Middle row partitions
Bottom row character sets
0
100
200
300
400
500
Locus Sites

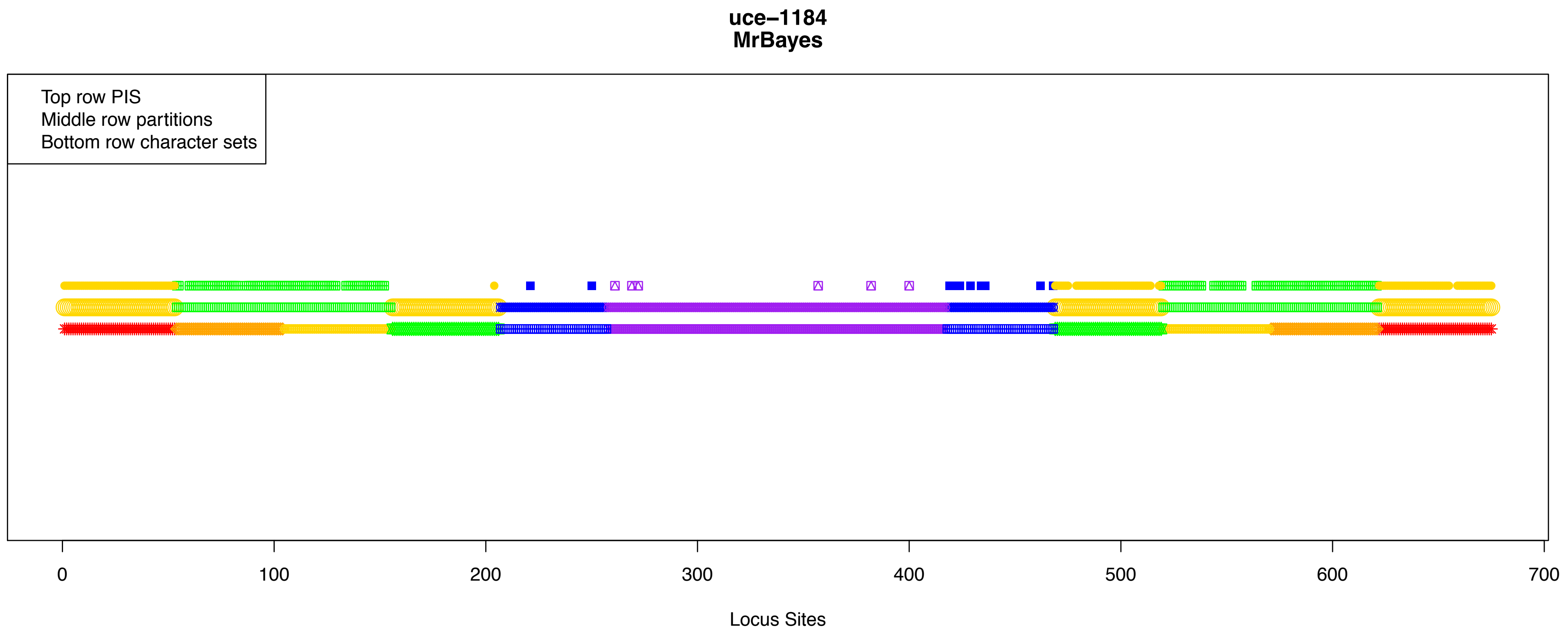

uce–1184
MrBayes
Top row PIS
Middle row partitions
Bottom row character sets
0
100
200
300
400
500
600
700
Locus Sites

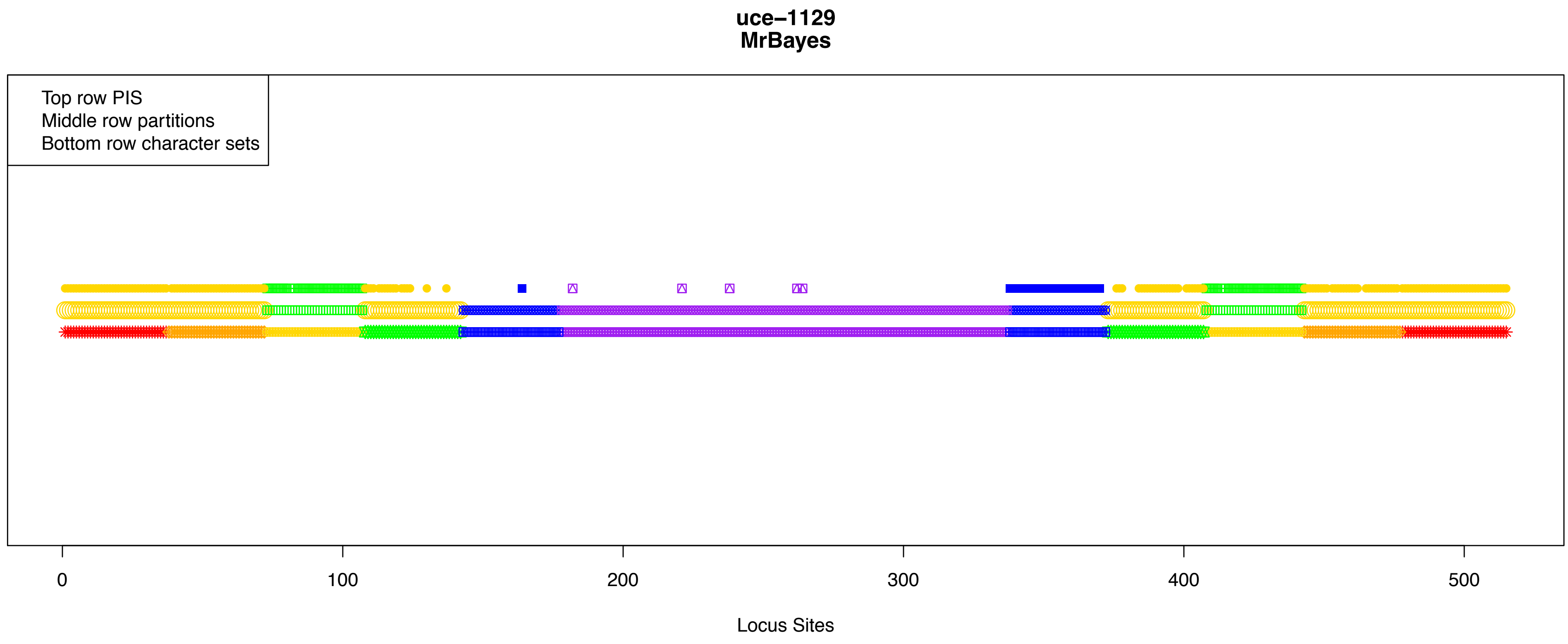
uce–1129
MrBayes
Top row PIS
Middle row partitions
Bottom row character sets
0
100
200
300
400
500
Locus Sites

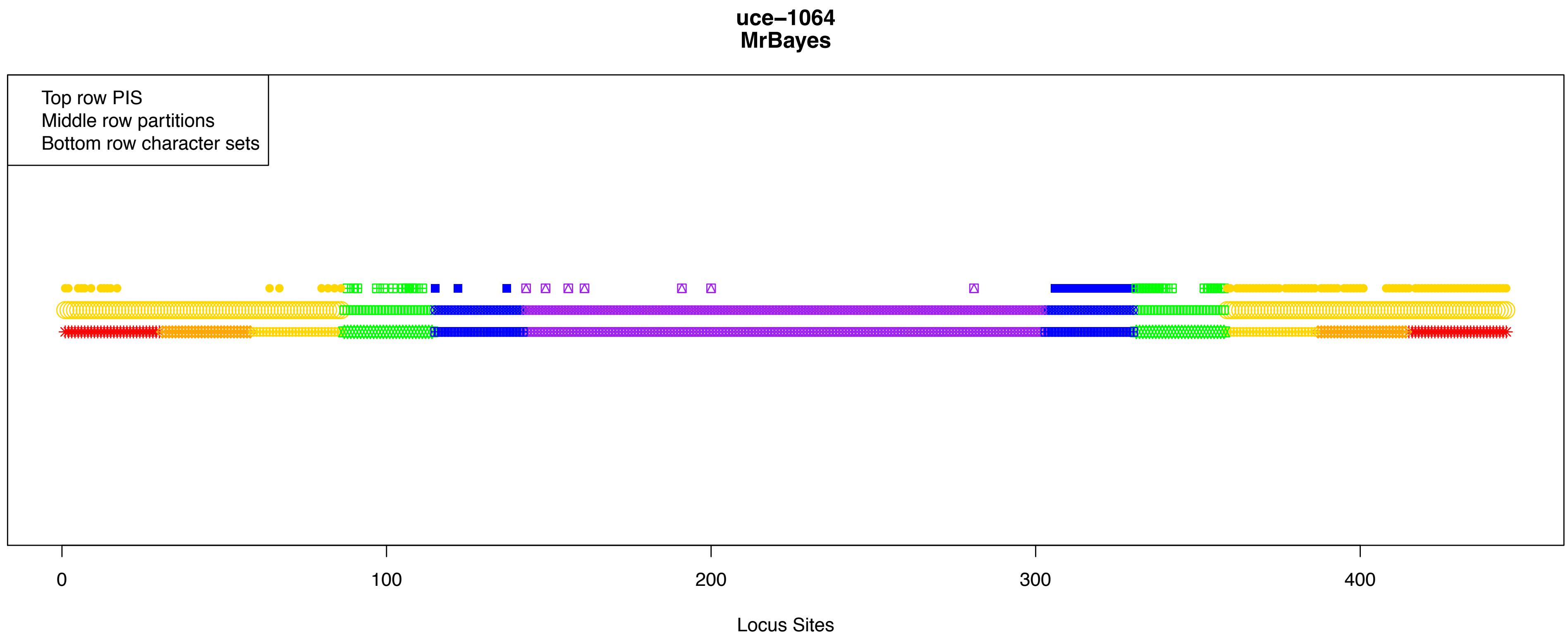

uce–1064
MrBayes
Top row PIS
Middle row partitions
Bottom row character sets
0
100
200
300
400
Locus Sites

uce–1049
MrBayes

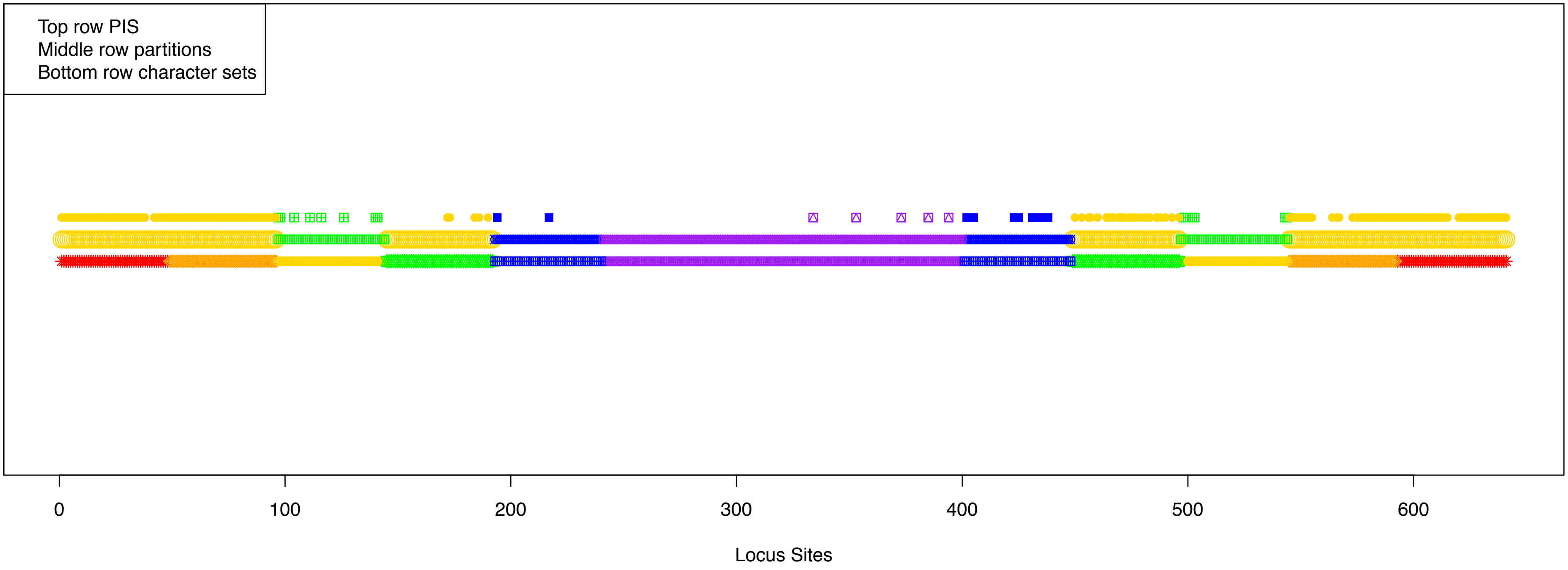

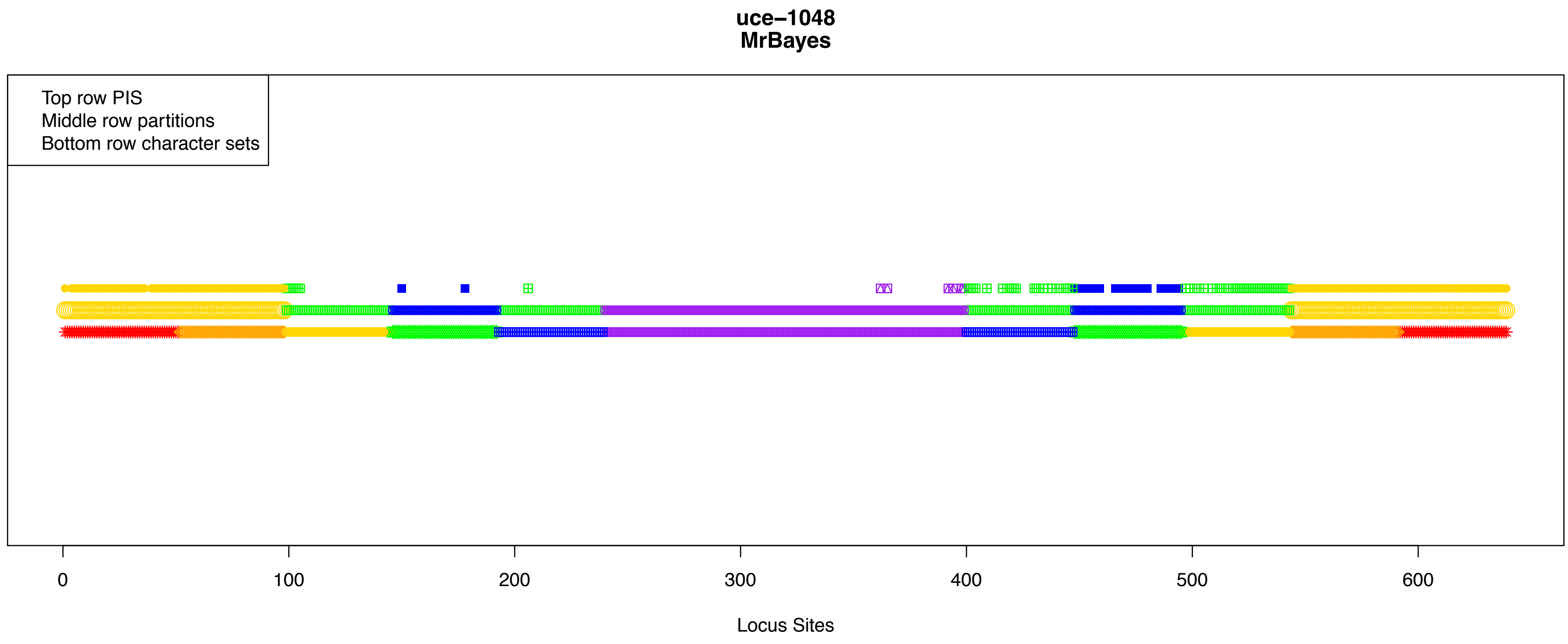
uce–1048
MrBayes
Top row PIS
Middle row partitions
Bottom row character sets
0
100
200
300
400
500
600
Locus Sites

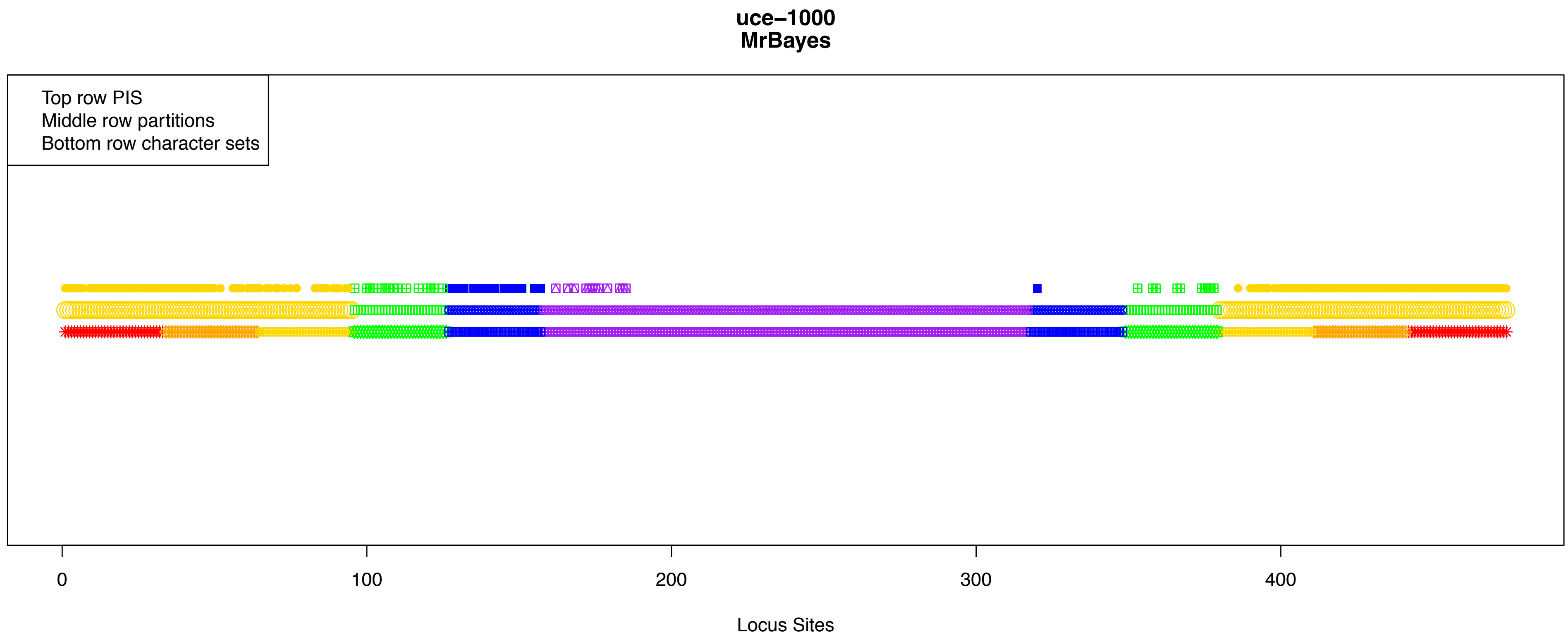
uce–1000
MrBayes
Top row PIS
Middle row partitions
Bottom row character sets
0
100
200
300
400
Locus Sites
